# Apprehending the NAD^+^–ADPr-Dependent Systems in the Virus World

**DOI:** 10.3390/v14091977

**Published:** 2022-09-07

**Authors:** Lakshminarayan M. Iyer, A. Maxwell Burroughs, Vivek Anantharaman, L. Aravind

**Affiliations:** National Center for Biotechnology Information, National Library of Medicine, National Institutes of Health, Bethesda, MD 20894, USA

**Keywords:** nicotinamide adenine dinucleotide, ADP-ribose, cyclic ADP-ribose, RNA polymerase, anti-phage systems, RNA repair, nucleotides, virus evolution, NADase, ADP-ribosyltransferase, sirtuin

## Abstract

NAD^+^ and ADP-ribose (ADPr)-containing molecules are at the interface of virus–host conflicts across life encompassing RNA processing, restriction, lysogeny/dormancy and functional hijacking. We objectively defined the central components of the NAD^+^–ADPr networks involved in these conflicts and systematically surveyed 21,191 completely sequenced viral proteomes representative of all publicly available branches of the viral world to reconstruct a comprehensive picture of the viral NAD^+^–ADPr systems. These systems have been widely and repeatedly exploited by positive-strand RNA and DNA viruses, especially those with larger genomes and more intricate life-history strategies. We present evidence that ADP-ribosyltransferases (ARTs), ADPr-targeting Macro, NADAR and Nudix proteins are frequently packaged into virions, particularly in phages with contractile tails (Myoviruses), and deployed during infection to modify host macromolecules and counter NAD^+^-derived signals involved in viral restriction. Genes encoding NAD^+^–ADPr-utilizing domains were repeatedly exchanged between distantly related viruses, hosts and endo-parasites/symbionts, suggesting selection for them across the virus world. Contextual analysis indicates that the bacteriophage versions of ADPr-targeting domains are more likely to counter soluble ADPr derivatives, while the eukaryotic RNA viral versions might prefer macromolecular ADPr adducts. Finally, we also use comparative genomics to predict host systems involved in countering viral ADP ribosylation of host molecules.

## 1. Introduction

In 1944, Max Delbrück proposed the T-bacteriophages, which infect *Escherichia coli*, as potential models for the study of host-virus interactions [1]. Over the past 80 years, the discoveries from this program have profoundly impacted our understanding of diverse areas of biology. The assembly of the phage virion provided one of the earliest models for morphogenesis and DNA packaging. Lindsay W Black, to whom this issue is dedicated, was one of the leaders in unraveling this process through his numerous studies on phage T4 morphogenesis [2]. In addition to bearing the phage genome, the head of phage T4 also carries at least eight internal proteins, some of which are injected into the host along with the viral DNA [3,4]. One of these is Alt, an enzyme that uses nicotinamide adenine dinucleotide (NAD^+^) as a substrate to transfer the ADP-ribosyl (ADPr) moiety to host proteins, such as the alpha subunit of the host RNA polymerase (RNAP), a modification that increases the preference of the RNAP for phage promoters [5,6]. This paralleled the discovery of the diphtheria toxin, which catalyzes a related reaction on the translation elongation factor EF-2 of eukaryotic host cells [7]. Notably, this ADP-ribosyltransferase toxin is encoded by the lysogenic phage β of *Corynebacterium diphtheriae* [8]. These were some of the earliest examples of ADP-ribosylation, a macromolecular modification, that over the years came to be known as a key player in numerous biological processes not just in viruses but across the tree of cellular life.

**Figure 1:**
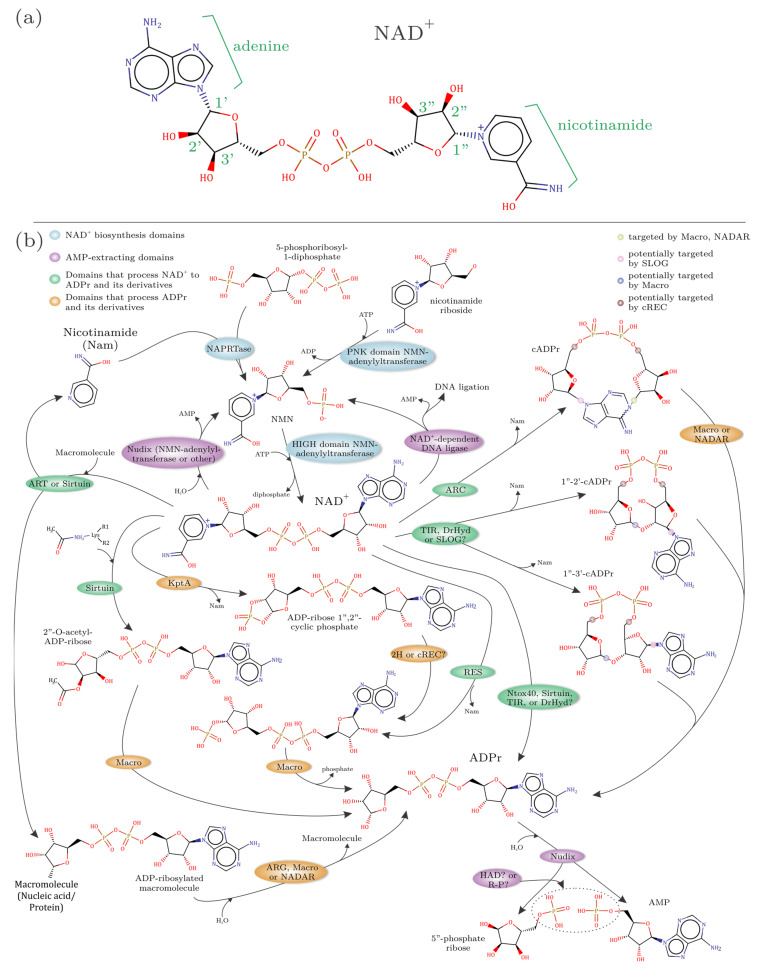
(**a**) Structure of NAD^+^ and (**b**) the substrates and products of various enzymes in the NAD^+^–ADPr network. Enzymes are color-coded based on the pathway in which they are involved. Bonds that are the target of particular enzymes are highlighted with colored circles.

The parent metabolite of ADPr, NAD^+^ and its phosphorylated derivative NADP^+^ are ubiquitous metabolites essential for cellular life (Figure 1a). Their best-understood role in cells is as an energy currency and as co-factors for enzymes catalyzing redox reactions [9]. Whereas viruses rarely encode NAD^+^-dependent redox enzymes, viral enzymes use NAD^+^ as a substrate in a variety of other contexts. Like all bacteria, numerous DNA viruses encode an NAD^+^-dependent DNA ligase, which transfers AMP from NAD^+^- to a terminal 5′-phosphate of DNA as an intermediate in the ligation reaction [10]. In recent years, it has become increasingly apparent that NAD^+^ and ADPr derivatives are at the interface of a major network of several other biochemical processes that are central to virus biology and virus–host biological conflicts [11,12,13,14,15,16,17,18,19,20]. In this article, we use the vantage point of over three decades of viral genomics to comprehensively survey the viral NAD^+^–ADPr systems, identify new components and unravel their evolutionary trajectories.

Subsequent to the discovery of the phage-encoded cholera toxin and T4 Alt, it became clear that these are only the tip of the iceberg of a vast assemblage of ADP-ribosyltransferase (ART) superfamily domains across bacteriophages and certain eukaryotic viruses [6,21,22,23,24,25,26,27,28]. T4 itself was found to deploy two further ARTs later in its infection cycle, ModA and ModB, which might also modify the RNAP subunits to facilitate its switching to the late viral promoters [6]. ModB has also been shown to transfer NAD^+^-capped RNA to protein substrates and modify their activity in a process called RNAlytion [26]. Through comparative genomic analysis, we uncovered the polyvalent effectors, a class of large multidomain, virion-embedded proteins encoded by a subset of phages, which combine a panoply of diverse domains predicted to operate early in infection [29]. These proteins are likely injected into the host along with the genetic material of the invading element. ART superfamily domains are the most common macromolecule-modifying enzymes found in these polyvalent proteins. Thus, head-packaged ARTs might be a more widespread strategy adopted by bacteriophages beyond T4 [29]. Notably, members of the ART superfamily are also at the forefront of the antiviral strategies of several bacteria and eukaryotes. For instance, the bacterial DarT-DarG toxin–antitoxin (T–A) system features the ART superfamily toxin DarT that ADP-ribosylates viral DNA incapacitating virus replication [30]. Similarly, in animals, infections induce the host ARTs such as members of the Poly ADP-ribose polymerase (PARP) and related ART enzymes (PARTs) to modify host proteins as part of the antiviral response [21,24,31,32]. The structurally unrelated sirtuin (SIR2) domain might also function comparably to the ARTs as an ADP-ribosyltransferase in these biological conflicts [33,34].

Recent studies on antiviral conflict systems have revealed a pervasive network of nucleotide-dependent signaling and immunity processes [11,12,13,14,15,16,17,18,19,20,35,36,37]. A subset of these is centered around enzymatic domains that process NAD^+^ (Figure 1a) to generate ADPr derivatives that serve as second messengers or toxins. The typical logic of these systems involves signal-generating enzymes that process NAD^+^ to generate soluble ADPr derivatives as second messengers. These signals are then sensed by sensor domains that often play a role in setting the activation threshold for the induction of a diverse array of effectors. The effectors abort the infection by either directly targeting phage macromolecules, or the conserved host machinery either to shut down functions needed by the viruses or promote cell death. The biochemically characterized signal-generating enzymes include the structurally related TIR, SLOG, DrHyd, and ADP-ribosyl cyclase (ARC) domains, RES and Frg domains from the ART superfamily, and, likely, the sirtuin domain [11,12,13,15,16,18,20,38,39]. The action of such enzymes on NAD^+^ generates signaling molecules such as the classic cyclic ADPr (cADPr), the more recently characterized “variant” cADPr molecules with 1″-2′ or 1″-3′ linkages, or other ADPr derivatives, including toxic metabolites such as ADPr-1′ phosphate that can cause cell death [18,19,40,41] (Figure 1b).

On the virus side, host-mediated macromolecular ADP-ribosylation and potentially also the soluble ADPr derivatives are either known or predicted to be degraded by a range of domains. Several eukaryotic RNA and DNA viruses, encode enzymes that counter ADPr modifications, namely the Macro and (Poly)ADP-ribosylglycohydrolase (ARG) domains [15,40,42,43]. The viral Macro, ARG, NADAR and 2H domains also likely target soluble ADPr derivative signals [18,19]. Versions of the Nudix domains from both eukaryotic and bacterial viruses cleave the nucleotide diphosphate-X linkages in NAD^+^, ADPr and their derivatives (Figure 1b) [29,40,41,44,45,46,47,48,49].

Another distinct role for NAD^+^ in virus–host conflicts is in RNA repair. During infection, host-cell-encoded endoRNases often cleave their own or virally encoded tRNAs to disrupt translation [50,51,52]. Viruses have evolved multiple enzymes as counter-strategies: they encode RNA ligases to rejoin the cleaved tRNAs. However, for ligation to proceed, they need to “clean up” the terminal cyclic 2′-3′ and 2′phosphates formed as a result of RNA-cleavage by metal-independent endoRNases [53]. This is achieved by the enzyme KptA, a member of the ART superfamily, which uses NAD^+^ to process such RNA ends in conjugation with the 2H, Macro, and possibly NADAR domains [40,54,55,56,57]. Finally, another defense strategy of the hosts is to cripple the viral NAD^+^-dependent systems by depleting cellular NAD^+^. These host systems feature NAD^+^-consuming domains, such as the NADase versions of the ART superfamily, TIR, DrHyd, and sirtuin domains [16,17,18,58]. The Frg domain, a member of the ART superfamily, which we had reported as a potential anti-viral effector in prokaryotic immunity systems, might also function as a NADase [11]. Several phages carry their own NAD^+^-synthesis enzymes as a potential counter-strategy [14,44,59].

Our previous investigations on host NAD^+^-dependent immune systems and viral NAD^+^–ADPr-related protein domains in polyvalent proteins, jumbo phages and coronaviruses indicated a vast, under-appreciated network of these systems in the viral world [14,29,60]. Several recent wet-lab studies have also uncovered a growing role for the NAD^+^–ADPr systems at the interface of the virus–host arms race [17,18,37]. Hence, we present here the results of a systematic computational investigation of these systems across all viruses (both RNA and DNA) infecting hosts from across the tree of life. Using sensitive sequence, structure, and contextual analyses, we not only probe the extent of all the above-introduced systems across the virus world but also identify several new systems and predict novel functions pointing to a greater role for NAD^+^–ADPr network in viruses than previously appreciated. Finally, we also use a reverse strategy to identify host systems potentially involved in countering ADP-ribosylation of host macromolecules catalyzed by viral enzymes.

## 2. Material and Methods

### 2.1. Dataset

To capture a comprehensive picture of the NAD^+^–ADPr systems across the viral world, we initially extracted the 40,621 viral genomes available in Genbank (6 October 2021). Of these, 38,406 were completely sequenced genomes and this set was made non-redundant based on taxid by choosing the largest proteome per NCBI taxid to arrive at a database of 21,191 proteomes and 12,49,624 protein sequences. This database was used for all our analyses and to obtain the numerical statistics reported in this article. The viruses were named as per the taxonomy division of Entrez. The complete list of viruses and their genome statistics are available in the supplement (Supplementary Materials S1). Over 50% of the genomes are of prokaryotic double-stranded DNA viruses, constituting about 86% of the protein sequences in the database. Of the remaining genomes, about 20% are single-strand RNA viruses, which contribute to about 1.5% of the total proteins. The size range of the viral proteomes varies from 1 (e.g., polyproteins of RNA viruses) to a maximum of 1430 proteins (Pandoravirus salinus).

### 2.2. Sequence Analysis

Bonafide versions of previously characterized NAD^+^–ADPr related domains (see below) were extracted from the National Center for Biotechnology Information (NCBI) Genbank database and used in sequence similarity searches with the PSI-BLAST and JackHMMER programs against the above-described virus database [61,62]. Searches for host factors were performed against the NCBI nr database or the nr database clustered down to 50% using the MMseqs program [63]. The default profile-inclusion threshold for iterative searches was set at 0.01. Profile–profile searches were performed with the HHpred program [62] against libraries of profiles based on non-redundant PDB structures, the Pfam database [64], and a custom collection of profiles of domains not detected by Pfam. Kalign with default parameters [65] and Mafft with maxiterate = 3000, globalpair, op = 1.9 and ep = 0.5 parameters [66] were used to generate multiple sequence alignments (MSA), followed by refinements using HHpred profile–profile matches [67] or HMM-align [62]. These MSAs were also finally manually adjusted, guided by structure superimpositions (See below).

### 2.3. Structure Analysis

The JPred program [68] was used to predict secondary structures using the MSAs. PDB coordinates of structures were retrieved from the Protein Data Bank (PDB). Structure similarity searches were performed using the DALIlite program [69]. DALIlite was also used to generate structural alignments. Structures were rendered, compared, and superimposed using either the Mol* [70] or PyMOL programs [71]. Structural modeling was performed using the RoseTTAFold program, which uses a “three-track” neural network, utilizing patterns of sequence conservation, distance inferred from coevolutionary changes in MSAs, and coordinate information [72]. It achieves prediction accuracy similar to another algorithmically comparable method, DeepMind’s Alphafold2 [73]. MSAs of related sequences (>30% similarity) were used to initiate HHpred searches for the initial step of correlated position and contact identification to be used by the neural networks.

### 2.4. Comparative Genomics and Phylogenetic Analysis

Clustering of protein sequences and the subsequent assignment of sequences to distinct families was performed using the BLASTCLUST program adjusting the length of aligned regions and bit-score density threshold empirically (version: 2.2.26, NCBI, Bethesda, MD, USA, ftp://ftp.ncbi.nih.gov/blast/documents/blastclust.html accessed on 6 September 2022) (RRID: SCR_016641). Divergent sequences or small clusters were merged with larger clusters if other lines of evidence, including shared sequence motifs, shared structural synapomorphies, reciprocal BLAST search results, and/or shared genome context associations, supported their inclusion. Gene neighborhoods were extracted using PERL scripts from genomes retrieved from the NCBI Genome database. These were then clustered using the BLASTCLUST program and filtered using an inter-gene distance cutoff of 100 nucleotides and phyletic patterns in order to identify conserved gene-neighborhoods. Initial phylogenetic analysis was performed using the rapid approximately maximum-likelihood method as implemented in the FastTree program [74]. This was followed by in-depth phylogenetic analyses of specific cases using the IQtree program with empirically determined best-fitting models and rates categories for each alignment [75]. The FigTree program (http://tree.bio.ed.ac.uk/software/figtree/ accessed on 6 September 2022) was used to render phylogenetic trees.

## 3. Results and Discussion

### 3.1. Defining the Building Blocks of the NAD^+^–ADPr Network

The viral NAD^+^–ADPr network can be conceived as a molecular ecosystem of domains arrayed around the use and the generation of NAD^+^ (Figure 1). We classified them into four categories based on their biochemistry (Figure 1b), listed below in the order of their prevalence in viral genomes:

(1) Domains that use NAD(P)+ as a substrate to release ADPr and its derivatives. This group includes ART, sirtuin, TIR, and DrHyd superfamily domains. The basic reaction catalyzed by these enzymes (best studied in the case of the ART superfamily) is an SN1 reaction, where an oxacarbenium ion intermediate is formed from NAD^+^ at the 1″ position of ADPr upon nicotinamide leaving [76,77] (Figure 1b). This intermediate is then available for nucleophilic attack. If the attack is by water, then free ADPr is released, i.e., a NADase reaction. Alternatively, it might be attacked by phosphate (e.g., in the case of KptA and RES domains of the ART superfamily) or acetyl (certain sirtuin domains) groups resulting in ADPr derivatives conjugated to these moieties. An attack by the adenine group from within NAD^+^ results in cyclic ADPr (cADPr; e.g., catalyzed by the ARC clade of the DrHyd superfamily) [58]. Similarly, attacks by the 2′ or 3′ ribose hydroxyls result in the variant cADPrs with 1″-2′ or 1″-3′ linkages (catalyzed by some members of the TIR and likely DrHyd superfamilies) (Figure 1b). If NADP is used as a substrate, an attack by a free nicotinate results in nicotinic acid adenine dinucleotide (NAADP; catalyzed by the ARC clade of the DrHyd superfamily) [78]. Finally, an attack by groups in macromolecules, such as bases in nucleic acids or amino acid sidechains from proteins can result in ADPr being conjugated to them (catalyzed by the ART and sirtuin superfamilies) [14,21,23,33,79,80].
(2) Domains that process ADPr derivatives. This group includes structurally unrelated but catalytically comparable domains from the Macro, ARG, 2H, cREC, NADAR and SLOG superfamilies, which are known or predicted to act on the products generated by the above group (Figure 1b). The Macro superfamily shows considerable versatility in acting on both soluble ADPr derivatives and macromolecule-conjugated versions [81,82,83]. The characterized members of the ARG superfamily act on ADP-ribosylated proteins, but contextual evidence suggests a potentially wider range of substrates such as the Macro domain [83]. The 2H family acts on 1″-2″cyclic ADPr phosphate (ADPr > P, also known as Appr > p) generated by the action of KptA [57], the ART domain involved in the clean-up of cyclic phosphate RNA-termini [53]. While the cREC, NADAR and SLOG domains are strongly predicted to process ADPr derivatives they remain experimentally uncharacterized [12,14,40].
(3) Domains that extract AMP from NAD^+^ or ADPr. This group includes two rather distinct types of domains. The first is the NAD^+^-dependent ligases, which are nucleotidyltransferases that use NAD^+^ as a substrate to adenylate DNA ends during phage DNA repair and replication [10]. The second is the Nudix domain that is involved in the breakdown of NAD^+^ to AMP and NMN and ADPr to AMP and ribose-3′-phosphate [84] (Figure 1b).
(4) Domains involved in the biosynthesis of NAD^+^. This group includes a range of enzymes involved in the synthesis of nicotinamide from nicotinic acid on one hand and the adenylation of NMN to generate NAD^+^ on the other [44,85] (Figure 1b).

### 3.2. NAD^+^–ADPr Network Is a Feature of Both Large RNA and DNA Viruses

Using sequence profiles and HMMs for the above domains as queries we systematically searched the non-redundant virus database (see Section 2.2) with profile-/HMM-based search programs; namely, PSI-BLAST, HMMscan and JACKHMMER. We further examined hits that were below the statistical threshold and conducted reverse searches with them against the NCBI nr database clustered down to 50% identity to confirm or falsify their affinities. We augmented the confirmation of distant relationships by using profile–profile searches with the HHpred program as well as neural-network-based structural modeling using the RoseTTAFold program (see Section 2.3). We also performed an extensive contextual analysis of domain fusions and conserved gene-neighborhoods to identify domains associated with our initial queries and fed them back into the above pipeline for further detection and analysis. As a result, we comprehensively detected the occurrence of domains from the NAD^+^–ADPr network across all types of viruses.

**Figure 2:**
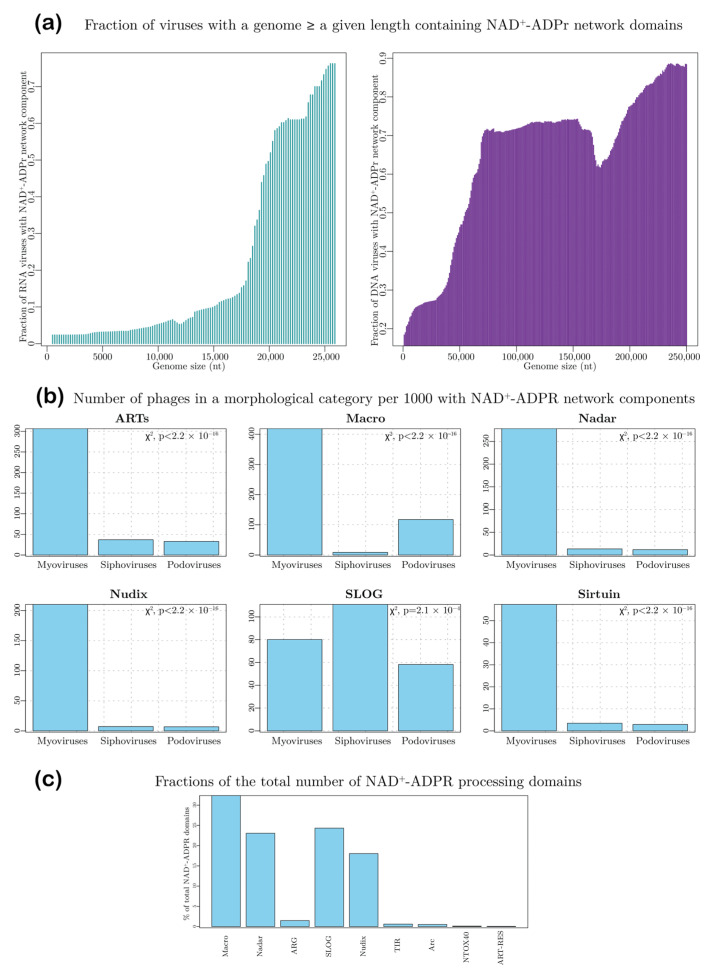
Distribution of NAD^+^–ADPr network domains in the virus world. (**a**) Fraction of RNA (left) and DNA (right) viruses with a genome ≥ a given length containing NAD^+^–ADPr network domains. (**b**) Distribution of various NAD^+^-ADPR domains in the Myoviruses, Siphoviruses and Podoviruses. The graphs depict the number of phages per 1000 containing the given domain in that morphological category. (**c**) Prevalence of various NAD^+^–ADPr processing domains depicted as a percentage of the total number.

We then plotted the fraction of viral genomes greater than or equal to a certain size threshold that code for a protein from the NAD^+^–ADPr network separately for DNA and RNA viruses. These plots showed a striking relationship between viral genome size and the coding of components of this network (Figure 2a). First, only a small minority of viruses (<0.1%) with a genome size less than 17 kb was found to have a component of the system. Among the rare exceptions is the Bat Tymo-like virus [86], the smallest virus featuring a member of this network with an RNA genome of just 6434 bp. Second, in the RNA viruses, a sharp increase in the fraction containing NAD^+^–ADPr components was observed for genome size greater than 17.5 kb. This corresponds to the largest RNA viruses, including the nidoviruses, such as coronaviruses, which feature one or more components of the NAD^+^–ADPr system (Figure 2a) [32,46,60,81,87]. Third, a sharp rise in the fraction of viruses with components from the network was observed for genome sizes beyond 60 kb in the DNA viruses. Thus, more than 70% of the medium-sized (genome sizes > 100 kb) and 85% of jumbo phages (genome size > 180 kb) contain at least one protein from the network compared to only 15% of the small phages (genome size < 25 kb) (Figure 2a) [14,59,88]. Further, among the DNA viruses, over 31% of the eukaryotic nucleocytoplasmic large DNA viruses (NCLDV) [89,90,91] contain at least one component from this network. Thus, the acquisition of components of the NAD^+^–ADPr network appear to be prevalent in viruses that deploy more of an ecological K-strategy (reliance on strong competition against host defenses) than an r-strategy (reliance on rapid replication) [92].

Among bacteriophages, we found a significant bias (χ^2^ p < 2.2 × 10^−16^) in the presence of the NAD^+^–ADPr network with respect to the virion morphology (Figure 2b): Myoviruses (phages with contractile sheaths) showed the strongest proclivity for possessing these proteins, with 60% of them having at least one representative. In contrast, only 16% of the Siphoviruses (phages with non-contractile filamentous tails) and 18% of the Podoviruses (phages with short non-contractile tails) code for components of this network (Supplementary Materials S2). This bias might relate to the fact that at least some of these proteins are packaged into phage heads and actively injected along with the DNA—a process for which the Myoviruses are probably better equipped.

Next, we investigated the viral versions of each of the superfamilies of domains from NAD^+^–ADPr system grouped into the above-outlined four biochemical classes. In the following sections, we briefly lay out their specific features, phyletic patterns, and contexts, as a platform to understand the viral functional systems they constitute.

### 3.3. Distribution of NAD^+^-Utilizing Domains of the NAD^+^–ADPr Network in Viruses

In this section, we discuss the domains belonging to the first of the above-defined groups in the network, namely those that operate on NAD^+^/NADP to generate either ADPr or its derivatives.

#### 3.3.1. The ART Superfamily

The conserved core of the ART domain is formed by a split β-sheet with two three-stranded units, with helices typically following each strand [23]. While some members of the superfamily display just this conserved core, the involvement of this superfamily across diverse biological conflict systems has selected for a range of structural elaborations in the form of additional strands and inserts [23]. Our analysis showed that the vast majority of ARTs in viruses are found among DNA viruses. Only two positive-strand *Riboviria* (RNA viruses), namely the Botrylloides leachii nidovirus that infects tunicates and the Beihai picorna-like virus 117 that infects echinoderms code for ARTs. Among DNA viruses, they are found in caudoviruses and large DNA viruses of eukaryotes, namely NCLDVs (*Varidnaviria*) and baculoviruses. Among caudoviruses, the ARTs are found in over 40% of jumbo phages (genome size > 180 KB), with *Bacillus* phage G encoding at least 10 ARTs. Notably, the distribution bias with respect to phage morphology and the presence of ARTs is rather stark—whereas 30% of the Myoviruses code for ARTs, only 3% of the Siphoviruses and Podoviruses do so (Figure 2b). This is consistent with them being potentially delivered into the host during infection as prototyped by the T4 Alt. About 20% of the baculoviruses and 10% of NCLDVs encode ARTs. Among the NCLDVs they are only found in the microbial viruses (the mimiviruses and chloroviruses such as PBCV) and the insect iridoviruses (Supplementary Materials S3). Here, again some viruses might encode a large number of ARTs—for example, the *Bodo saltans* virus codes for 12 distinct versions. We only found rare instances of ARTs in the proteomes of single-strand DNA viruses, e.g., *Vibrio* phage CTXphi which codes for the cholera toxin ART [93].

Viral ARTs encompass a diverse range of biochemical specificities. While several caudoviruses code for homologs of the cellular enzyme KptA involved in RNA repair, the majority are fast-evolving versions suggestive of a role in biological conflicts. Homologs of the phage T4 Alt, ModA and ModB, and the versions from the phage polyvalent proteins likely target host macromolecules [6,22,28]. Both bacteriophages and eukaryotic DNA viruses possess ARTs that are closely related to the cellular PARTs, suggesting that, in addition to the mono-ADP-ribosylation, certain viral versions might also catalyze poly-ADP ribosylation. We found that a minority of the phage-encoded ARTs are from Type II T–A systems or other toxin systems that are related to previously characterized bacteriocins or virulence factors of their bacterial hosts. Some of these versions might degrade the NAD^+^ either as NADases or NAD^+^ phosphorylases.

#### 3.3.2. The Sirtuin Superfamily

The sirtuin domains are NAD^+^- or NMN-binding classic Rossmann fold domains that are found in a wide range of cellular metabolic and conflict systems [12,94,95]. They catalyze ADPr-releasing reactions comparable to the ART superfamily (Figure 1b). The conserved cellular versions (e.g., CobB) uses NMN as a substrate to transfer ribose-3′phosphate to 5,6-dimethylbenzimidazole to give α-ribazole phosphate, an intermediate in cobalamin biosynthesis [96]. The remaining versions primarily act as ADP-ribosyltransferases. Among these are the prokaryotic and eukaryotic deacylases that transfer ADPr to -NH_2_ acylated proteins (e.g., acyl lysines) with the acyl group leaving as O-acyl-ADPr (OAADPr) [97]. Most remaining sirtuins are effectors in diverse conflict systems. Of these, the version from a two-gene type-II T–A-like system, where the sirtuin is coupled with a Macro domain, ADP-ribosylates target proteins [80]. Sirtuin domains are also found in numerous anti-phage systems, namely the TIR domain-coupled systems [12], the HerA-ATPase-associated mobile system [98], ABI-like systems, PIWI-coupled systems [99], and TPR-GreA/B-PIN systems [95,100]. In a characterized two-gene TIR-based anti-phage system, the TIR domain-containing protein ThsB generates variant cADPr signals that are recognized by the SLOG of the second protein ThsA resulting in its dimerization. This triggers the NADase activity of the sirtuin domain of the ThsA protein with the concomitant abortion of the viral cycle due to NAD^+^ limitation [17,18].

Surprisingly, we found that despite their catalytic convergence, the sirtuin superfamily is far less frequent across viruses than the ART superfamily. They are primarily found in caudoviruses, with single representatives in the *Bodo saltans* virus (a NCLDV) and the archaeal virus *Haloferax* virus Halfgib1. Our computational analysis indicates that in addition to the above-described activity certain phage sirtuins (those associated with the HerA-ATPase-dependent conflict system) might ADP-ribosylate DNA, whereas another version might be involved in RNA repair akin to KptA (see below).

#### 3.3.3. The TIR-DrHyd-SLOG Class of Rossmannoid Domains

Our prior studies had unified three distinct superfamilies of Rossmannoid domains, namely the TIR, DrHyd and SLOG domains into a monophyletic clade (hereinafter TDS) that might either bind or catalytically process nucleotides [12]. They possess a core three-layered α/β sandwich, with a central 5-stranded β-sheet and, like most other nucleotide-binding, Rossmannoid domains contact the substrate nucleotide via conserved polar residues in the loop after Strand-1. However, the TDS clade of Rossmannoid domains is distinguished by a unique set of active site residues in the loop and helix after Strand-3. All characterized catalytically active versions of these domains are unified by the hydrolysis/transglycosylation of N-glycosidic linkages in nucleotides/nucleosides. The inactive versions bind nucleotides or their derivatives as sensors [12]. Of these, the current biochemical studies suggest that the TIR domains are NAD^+^-specialists that either hydrolyze it to generate ADPr or its cyclic derivatives [16,18,41] (Figure 1b). TIR domains are extremely widespread as both effectors, signal-generating enzymes and sensors in a diverse array of antiviral conflict systems [11,12,16,18]. The DrHyd and SLOG superfamilies act on a broader spectrum of nucleotides but contextual information allows us to identify numerous versions from immune systems that specifically target NAD^+^ or ADPr derivatives. Different members of the DrHyd superfamily involved in biological conflicts act as NADase effectors equivalent to the TIR domain or as ADP-ribosyl cyclases (ARCs) that generate cADPr [11,12,78]. While some SLOG domains are predicted to utilize NAD^+^ to generate ADPr derivative signals, we present evidence that the viral versions act on ADPr derivatives generated by host immune mechanisms [12] (these will be discussed separately below).

**Figure 3:**
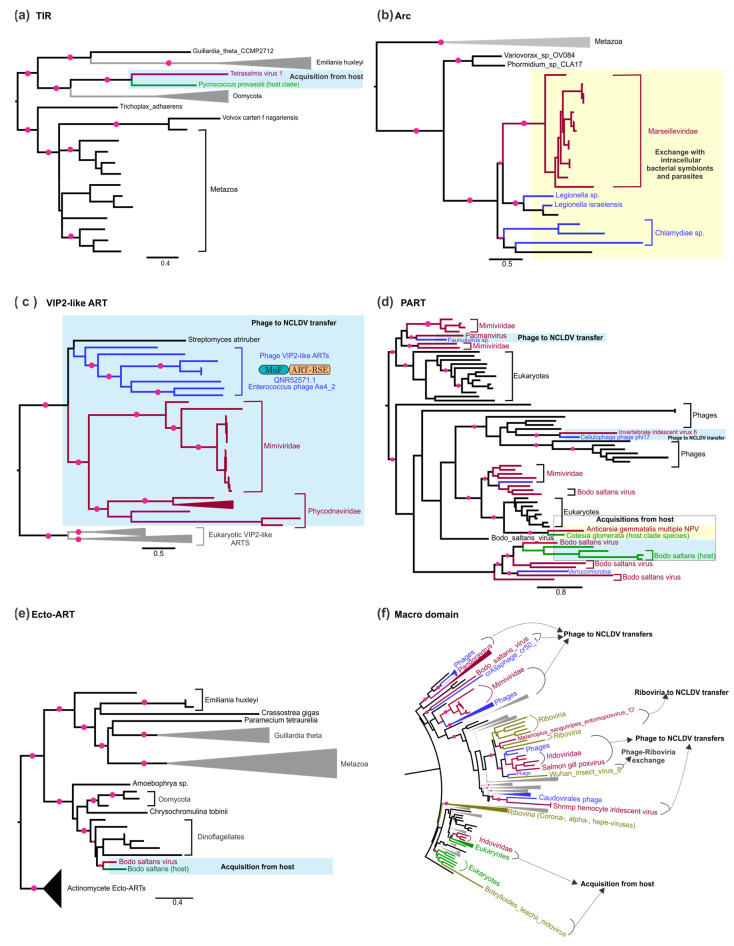
Phylogenetic trees of select viral NAD^+^–ADPr network components illustrating their origins as described in the text. (**a**) TIR, (**b**) Arc, (**c**) VIP2-like ART, (**d**) PART, (**e**) Ecto-ART, (**f**) Macro domain. Clades with a bootstrap support of > 75% are marked by colored circles. Several clades are collapsed in the trees for brevity. Relevant exchanges of genes are indicated. The raw data for the phylogenetic trees can be obtained from Supplementary S3.

Comparably to the sirtuin domains, but unlike the ART domains, TIR domains are relatively infrequently encoded by viral genomes. We found that a group of small phages that infect Firmicutes such as *Streptococcus*, *Staphylococcus*, and *Clostridium* encode a distinct version of the TIR domain. Similarly, among eukaryotic viruses, TIR domains are encoded by the NCLDVs infecting chlorophyte (*Tetraselmis* virus 1) and haptophyte (*Chrysochromulina ericina* virus) algae. Phylogenetic analysis revealed that these TIR domains are potential acquisitions from eukaryotes, which might include their hosts (Figure 3a). Within the DrHyd superfamily, we found that viruses only carry versions that are predicted to function as ARCs. These are found in a small number of caudate phages (e.g., AEO93593.1, Bacillus Phage G) and Marseilleviruses among the NCLDVs. Our phylogenetic analysis showed that Arcs of Marseilleviruses are specifically related to the effector versions deployed by multiple phylogenetically distant intracellular bacterial parasites of the genera *Legionella* and *Chlamydia*, paralleling other examples of gene transfers between NCLDVs and cohabiting endoparasites/endosymbionts (Figure 3b) [91,101,102]. The limited distribution of sirtuin, TIR and DrHyd (Arc) domains in viruses is in sharp contrast to their widespread presence in anti-viral conflict systems of both prokaryotes and eukaryotes (See Section 3.7.4). This observation suggests that, unlike ARTs, which can covalently modify macromolecular targets with ADPr, these domains, which tend to produce small, diffusible ADPr derivatives, are of lesser utility to viral host-manipulation strategies.

### 3.4. Distribution of Viral ADPr Processing Domains

In this section, we consider the second group of domains in the network that process ADPr conjugated to macromolecules or its soluble derivatives.

#### 3.4.1. Macro Domains

**Figure 4:**
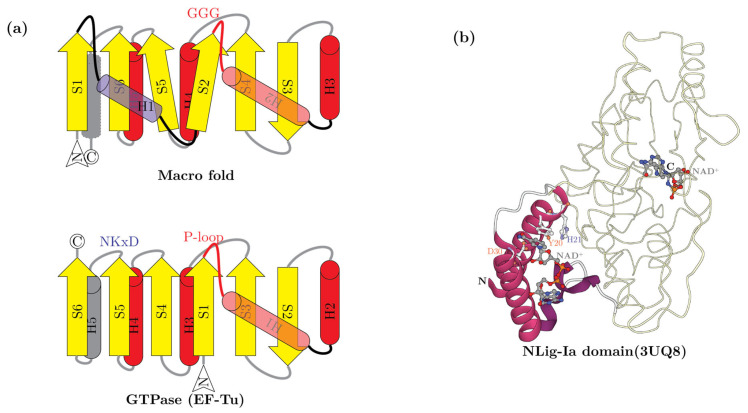
(**a**) Topology diagram of the core of the Macro domain and the EF-Tu-like GTPases illustrating their structural relationship. Strands labeled with an ‘S’ prefix followed by their order number in the core structure are shown as yellow arrows, whereas helices which are similarly labeled with a ‘H’ prefix are shown as red cylinders. (**b**) The structure of the Nlig-Ia domain (cartoon rendering) that exists as a solo domain only in viruses. The figure illustrates the residues involved in binding NAD^+^ and its relative position with respect to the C-terminal ATP-grasp and RAGNYA domain of the NAD^+^-dependent ligases (rendered as a tube).

Macro domains cleave off ADPr moieties from the entire spectrum of substrates wherein the 1″ or 2″ position of the ribose in ADPr is linked to another group (Figure 1b). These include: (1) 2″-OAADPr generated by certain sirtuins [43]; (2) ADPr-1″P (Appr1p) a byproduct of RNA repair by the KptA-2H system [51,53,54,57]; (3) ADPr conjugated to proteins or nucleic acids by either ART or sirtuin domains [23,79,80]. (4) Poly-ADPr glycohydrolases that cleave poly-ADP ribose moieties added by PARTs [42]. (5) Macro domains from anti-phage systems with TIR domains are also predicted to function in regulating them by potentially targeting cADPR [41]. (6) Certain versions might (e.g., MacroHistone 2A) act as sensors of ADPr derivatives rather than as active enzymes [42]. The structural core of the Macro domain is an α/β-fold containing a six-stranded sheet with the strands in the 1-5-6-2-4-3 order, with a well-conserved GxG motif in the loop following the second conserved strand (Figure 4a). This loop is part of the cleft that binds the ADPr substrate. DALI searches with Macro domains as queries retrieve various P-loop NTPases and show the closest alignment of secondary structure elements to the TRAFAC clade of GTPases (e.g., GTPase domain of Elongation factor G; pdb: 2dy1, DALI Z-score 5.5), albeit via a circular permutation (Figure 4a). In terms of phyletic patterns, apart from numerous viruses (see below), Macro domains are present across all superkingdoms of cellular life forms. Nevertheless, their phylogenetic affinities suggest widespread lateral transfer, in particular via conflict systems such as T–A systems. This suggests that they evolved much later as compared to the GTPases [103] and are likely to have been derived from the latter through rapid divergence for ADPr binding.

Macro domains are the most widely distributed ADPr derivative-processing domains across all viruses (Figure 2b). We observed that about 4.5% of small bacteriophages, 40% of medium-sized ones, and 56% of jumbo phages have at least one Macro domain. As with the ARTs, we also found a distinct bias in the presence of Macro domains with respect to phage morphology. Whereas 41% of the Myoviruses and 11% of the Podoviruses encode Macro domains, Siphoviruses are strikingly under-represented with only about 1% coding for them (Figure 2b). Relative to phages, Macro domains are rare in eukaryotic DNA viruses being found in only 8% of NCLDVs including Mimiviruses, poxviruses and iridoviruses. Close to 3% of all RNA viruses encode a Macro domain; however, this fraction rises to over 33% for genome sizes ≥ 19,000 nt. It is widespread in alphaviruses, coronaviruses and flaviviruses, and is also present sporadically in Hepatitis E-viruses (HEV), and the Bat Tymovirus [32,42,81,86]. The betacoronaviruses (e.g., SARS-CoV-2) are unusual in possessing three tandem Macro domains, of which two are extremely divergent [60]. In terms of their biochemistry, Macro domains have been best characterized in eukaryotic positive-strand RNA viruses. Earlier studies on the HEV Macro domain had demonstrated activity against both mono- and poly-ADPr adducts to proteins [32]. Recently, the SARS-CoV-2 Macro domains have been contradictorily claimed to either target poly-ADPr adducts conjugated by the mammalian PARP14 or alternatively against mono-ADPr adducts [81,87].

#### 3.4.2. ADP Ribosyl Glycohydrolases (ARGs)

While structurally unrelated to the Macro domain, the ARG domains catalyze equivalent reactions. The characterized members are known to release ADPr from ADP-ribosylated proteins (e.g., DraG from the DraG-DraT T–A systems) [104] or from OAADPr generated by sirtuins from acylated lysines [105]. The ARG domain is comprised of α-helical superstructure-forming repeats with an active site pocket featuring a binuclear metal-coordinating site, which along with a conserved TH motif catalyzes ADPr hydrolysis [106]. Even though ARG domains catalyze an equivalent reaction as the Macro domain, they are much rarer in viruses (Figure 1b). Unlike the Macro domains, only about 5% of the jumbo phages code for an ARG. Additionally, they are found sporadically in the medium-sized caudoviruses of actinobacteria, certain NCLDVs of microbial eukaryotes (Mimiviruses, algae-infecting PBCV, and *Only Syngen Nebraska* virus 5), and the *Catopsilia pomona* nucleopolyhedrovirus. We propose that the striking difference in the abundance of Macro and ARG domains might relate to the latter domain preferring macromolecular ADPr conjugates as opposed to low-molecular-weight derivatives that are part of the immune signaling and toxicity in prokaryotes (see Section 3.8 for a contrasting situation in host genomes).

#### 3.4.3. The NADAR Domain

NADAR domains function as ribosyl N-glycosidases acting on intermediates in riboflavin biosynthesis [107] and ADPr derivatives [40]. They are present in versions of DarT-DarG-like T–A systems in lieu of the Macro domains; hence, these NADAR domains were predicted to release ADPr from macromolecular conjugates [40]. Their association with RNA-repair systems suggests that they might additionally operate on low-molecular-weight ADPr derivatives produced during RNA repair [40]. The NADAR domains have a unique α + β fold with five core helices flanked by two small β-hairpin sheets, which is structurally unrelated to the Macro and Arg domains [40]. Hence, their comparable catalytic properties, suggest selection for multiple convergently evolving ADPr derivative-processing activities. However, we observed that several viruses possess proteins with tandem NADAR and Macro domains. This suggests that, at least in certain viruses, these domains might have evolved distinct specificities for alternative ADPr derivatives or different bonds within them, e.g., N-glycosidic bonds versus other ribosyl linkages—thus, they could preferentially target the classic cADPr signal as opposed to the variant ones which might be more suitable substrates for the Macro domain (Figure 1b).

**Figure 5:**
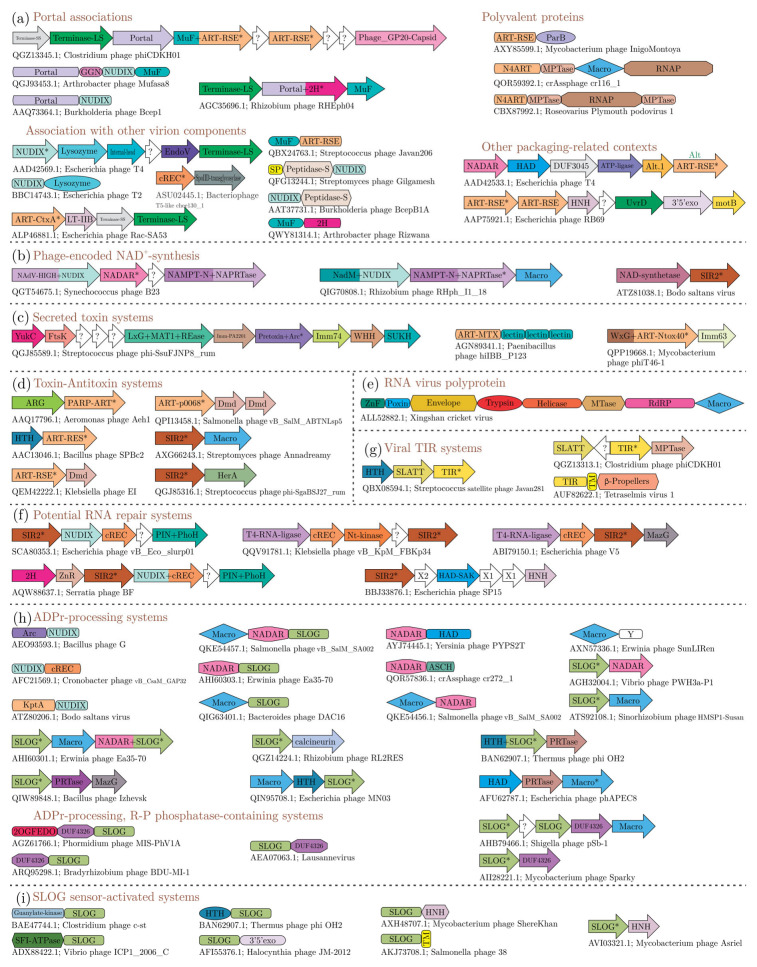
Representative contextual associations including domain architectures and gene neighborhoods of various domains of the NAD^+^–ADPr network. Gene neighborhoods are shown as box arrows with the arrowhead pointing to the 3′ gene. Domain architectures are shown by other shapes. The contextual associations are categorized based on their genomic contexts or their function including (**a**) domains associated with the Terminase-portal genes and those encoding other virion components; (**b**) domains involved in NAD^+^ synthesis; (**c**) secreted toxin domains; (**d**) domains that are components of T–A and related conflict systems; (**e**) domains in RNA virus polyproteins; (**f**) domains involved in a predicted RNA repair system; (**g**) viral TIR systems; (**h**) domains involved in ADPr-processing and; (**i**) SLOG sensor-activated systems. Gene neighborhoods are labeled with the accession number and species name of the gene marked with an asterisk.

NADAR domains are found in phages, NCLDVs, and RNA viruses. Within the bacteriophages, the presence of NADAR domains is correlated with genome size, with over 48% of medium-sized and jumbo phages containing proteins with this domain as compared to 0.3% in the small phages. Notably, while 27% of the Myoviruses display NADAR domains about 1% of the remaining phages have one. Among eukaryotic DNA viruses, the domain is found in representatives of several NCLDV clades such as phycodnaviruses, Mimiviruses, insect iridoviruses and ascoviruses. Among the RNA viruses, NADAR domains are found in nidoviruses that infect nematodes, crustaceans and insects, Hepe-like viruses of crustaceans, and annelid/bivalve picornaviruses. Their affinities suggest lateral transfer of this domain between these otherwise distantly related positive-strand RNA and DNA viruses. Contextual analysis of phage NADAR domains suggests that in several T4-like phages they are expressed as early genes along with the ARTs. In phages coding for their own NAD^+^ biosynthetic apparatus, NADAR domains are also found in gene neighborhoods with other NAD^+^ salvage pathway components (e.g., NadM adenylyltransferase and NadV phosporibosyltransferase) (Figure 5). Overall, the prevalence of NADAR domains is greater than that of ARG domains but lesser than that of Macro domains across viruses. Hence, as proposed above for the Macro domains, we posit that the viral NADAR domains might prefer soluble ADPr derivatives generated by host antiviral conflict systems or byproducts of RNA processing.

#### 3.4.4. The SLOG Domains

These domains form the third branch of the TDS clade of Rossmannoid fold domains that additionally includes the TIR and the DrHyd domains. Based on sequence- and contextual-analysis we had previously classified SLOG domains into several distinct clades. Like the DrHyd domains, different clades of SLOG domains have biochemical functions ranging from enzymes acting on diverse nucleotides/nucleosides to apparently non-catalytic sensors of nucleotide ligands (variant cADPr molecules or DNA) [12]. Our analysis of the viral SLOG domains revealed that they primarily belong to the YpsA-like clade of SLOG domains, which includes members involved in sensing cADPr and is implicated in other NAD^+^-related roles in prokaryotic immune systems [12,108]. The experimentally, best-characterized SLOG domains act on a range of AMP-containing/adenine-nucleoside-containing substrates (e.g., free AMP or tRNA) to cleave the N-glycosidic linkage between the base and ribose to release a free-base (LOG-like clade) [39,108]. Their activity is often directed towards nucleotides with modified adenines generating free modified bases that act as signaling hormones in plants (cytokinins) or bacterial signals for manipulating host behavior [39,108]. A relatively small number of phages possess SLOG domains of this LOG-like clade. Thus, the majority of viral SLOG domains are predicted to function in an NAD^+^-related pathway—a role supported by their fusions to Macro and/or NADAR domains (Figure 5).

Currently, SLOG domains are only found in Caudoviruses and NCLDVs and as with the above-discussed domains show a clear proclivity for viruses with larger genomes being present in 31% of jumbo phages as compared to 7% and 19% of the small and medium-sized phages, respectively. Among the NCLDVs, SLOG domains are found primarily in various giant amoeba-infecting viruses (i.e., Marseillevirus group). The catalytic activity of the LOG-like clade on 1′-ribosyl-base N-glycosidic linkages and the interaction of YpsA-like clade SLOG domains with variant cADPR in prokaryotic conflict systems offer precedence for predicting the potential activity of the viral SLOG domains. Accordingly, we posit that the viral SLOG domains might help counter host immune signaling by degrading cADPr or related signals by acting on the 1′-ribosyl-adenine bonds in such molecules (Figure 1b).

#### 3.4.5. The 2H and cRec Superfamilies

Terminal 2′-3′ cyclic phosphates arise in RNA from metal-independent endoRNases. Such effectors are common in both prokaryotic and eukaryotic antiviral immune mechanisms, such as the toxins from T–A systems or the eukaryotic interferon-induced RNase L [53]. RNAs with cyclic phosphates might also act as signals that regulate transcription factors (TFs) with CARF and WYL domains that act as sensors for these [109,110]. In turn, some of these TFs regulate prokaryotic conflict systems or RNA repair pathways. One mechanism for the repair of RNAs with cyclic termini is the use of the ART, KptA, which results in the formation of ADPr derivatives with cyclic phosphates (1″-2″ cADPr) [57]. Two structurally unrelated families, 2H and cRec, are known or predicted to catalyze the metal-independent resolution of these cyclic phosphates both in RNA termini and in 1″-2″ cADPr into linear phosphates [14]. Additionally, 2H domains have also been implicated in cleaving RNA, 2′-5′ oligoadenylate (2′5′A) and cyclic oligonucleotides [46,111]. The 2H superfamily is characterized by two conserved histidines coming from an internally duplicated fold (hence the name 2H) [45]. In contrast, cRec is a Rosmannoid fold domain related to the Receiver (Rec) domain found in histidine kinase-Rec phospho-relay signaling systems. While sharing a metal-independent active site containing two aspartates with the Rec domain of two-component systems, the cRec domain is only found in contexts related to RNA repair and 1″-2″ cADPr-processing, but never with a histidine kinase. Thus, the cRec domain was earlier predicted to catalyze the cleavage of cyclic phosphates comparably to the 2H domain [14].

2H domains are widely distributed in the viral world. They are found in several distant RNA-viral lineages, namely dsRNA Reoviruses (e.g., Rotaviruses), positive-strand RNA viruses, such as nidoviruses (in several toroviruses and coronaviruses), and certain retroviruses, such as the fish retrovirus, and Walleye dermal sarcoma virus [45]. In some of the nidoviruses, 2H domains are embedded in their polyproteins alongside metal-independent RNases such as the EndoU domain which generates cyclic 2′3′ ends (Figure 5) [112,113,114]. Thus, at least a subset of the RNA viral 2H domains might be involved in the processing of such ends. Others have been proposed to counter host immunity by targeting interferon-induced 2′-5′A [46]. Among DNA viruses they are found in 33–35% of medium-sized and jumbo phages and might function in conjunction with phage-encoded tRNA ligases and/or KptA and sirtuin domains (see Section 3.7.3) to repair both phage-encoded and host tRNAs. Others show fusions to MuF or Portal protein domains suggesting that they are packaged into phage heads and injected during infection to potentially counter host immune signaling via (oligo)nucleotides in the SMODS, cyclic mononucleotide-dependent and CRISPR systems [29,115]. The cRec domains are mostly found in bacteriophages (or prophages in bacterial genomes) and are present in up to 18% of jumbo phages. They often show fusions to the Nudix domain (see Section 3.5.2) (Figure 5) suggesting that they might operate synergistically with them in the degradation of 1″-2″ cADPr to mononucleotides.

### 3.5. Domains Generating AMP from NAD^+^ or ADPr-Derivatives

In this section, we consider the third category in the NAD^+^–ADPr ecosystem, which includes two domains that are unified by their capacity to generate AMP from NAD^+^ or ADPr derivatives, namely the NAD^+^-dependent DNA ligase and the Nudix domain.

#### 3.5.1. The NAD^+^-Dependent Ligases and the NAD^+^-Binding NLig-Ia Domain

DNA ligases are an essential component of all semi-conservative DNA synthesis systems. All known DNA ligases have a monophyletic origin [116] and catalyze the adenylation of 5′ phosphates at DNA termini priming it for a nucleophilic attack by the 3′ OH resulting in strand ligation [10,117]. However, they differ in terms of the substrate they use for adenylation: the universal DNA ligase of bacteria uses NAD^+^ as the source, while the universal archaeo-eukaryotic ligase uses ATP [10]. As a result, the bacterial DNA replication system is connected to the NAD^+^–ADPr network. While a subset of the DNA viruses coopts the ligase of their host for their replication, medium and larger DNA viruses tend to encode their own DNA ligase [14]. However, the ligases encoded by the DNA viruses are not necessarily of the same specificity as those of their hosts. Thus, across DNA viruses we observe mutually exclusive distribution patterns of NAD^+^- and ATP-dependent ligases, with their genomes typically coding for either one of them. NAD^+^-dependent ligases are only found in viruses with genome sizes greater than about 68kb—about 32% of jumbo phages, 12% of medium-sized phages, and 19% of NCLDVs (several iridoviruses, poxviruses and Mimiviruses) code for an NAD^+^-dependent DNA ligase.

The common structural core of both NAD^+^- and ATP-dependent ligases is an ATP-grasp domain inserted into a RAGNYA domain, which together constitute a nucleotidyltransferase module that is reconstructed as having ancestrally bound ATP [118] (Figure 4b). The NAD^+^-specificity emerged secondarily via the fusion to an N-terminal α-helical NAD^+^-binding domain, which has been termed Ia in the structure of the NAD^+^-dependent ligases (hereinafter Nlig-Ia) [119]. The Nlig-Ia domain has been proposed to either swivel the NAD^+^ close to the ligase active site lysine on the RAGNYA domain [120] or function as an allosteric NAD^+^ binding site [119]. Strikingly, we found that the Nlig-Ia domain has an existence independent of the NAD^+^-dependent ligase as a solo protein in 81 phages in our dataset (for example, ECBP2_0056 from *Escherichia* phage ECBP2). With one exception, these phages do not encode a separate NAD^+^-dependent ligase catalytic module or even the ATP-dependent ligase. This suggests that these proteins function independently of a DNA ligase. However, they conserve the key ligand-binding residues indicating that they are standalone NAD^+^-binding domains (Figure 4b). Hence, we propose that they likely function as NAD^+^ sensors which might help indicate to the phage the deployment of NADase host effectors or shield NAD^+^ from the action of such effectors.

#### 3.5.2. Nudix Domains

These domains catalyze the hydrolysis of the central phosphodiester bond in molecules of the form NDP-X to NMP and P-X, where X is any moiety (Figure 1b). Overall, Nudix enzymes display considerable substrate diversity—in addition to NAD^+^/ADPr, they act on oxidatively damaged nucleotides such as oxo-dGTP, dinucleoside polyphosphates, NDP-sugars, and RNAs with NAD^+^ and other NDP-X type caps [84]. The Nudix domain displays a rare enzymatic version of the Ubiquitin-like β-grasp fold with an active site formed by the core helix with multiple conserved metal-coordinating acidic residues and an arginine [121]. The domain is widely found across the superkingdoms of cellular life and numerous viral lineages. Among the latter, Nudix domains are common in DNA viruses, being present in numerous phages, baculoviruses and NCLDVs. Nudix domains are very rarely found in RNA viruses, being present only in the Beihai weivirus-like virus 11 and a few dsRNA mycoviruses (e.g., *Phlebiopsis gigantea* mycovirus dsRNA 1). We identified the viral versions that are likely to process ADPr or NAD^+^ based on their specific sequence relationships to previously characterized versions utilizing these substrates as well as contextual associations (gene-neighborhood linkages and fusions) to other domains with clearcut roles in the NAD^+^–ADPr system. As with several other domains in the NAD^+^–ADPr network, the presence of Nudix domains is correlated with genome size, with over 24% of medium-sized and 45% of jumbo phages containing proteins with this domain, as compared to small phages, of which only 0.25% possess Nudix domain proteins. Moreover, in a trend matching some of the above-described domains, while 21% of the Myoviruses contain Nudix domains, less than 1% of the other phages code for one (Figure 2b).

Most phage Nudix domains belong to two major clades respectively typified by the phage T4 NudE domain and that found in the NadM NAD^+^-salvage protein [49,122,123]. Biochemical studies on the T4 NudE protein indicate that in addition to ADPr it also utilizes FAD and diadenosine triphosphate as substrates [49]. In several phages, the T4-like nudE proteins are encoded by the late gene clusters along with those for the virion lysozyme or are directly fused to head proteins such as MuF, Portal and the capsid-protein-processing serine peptidase, suggesting that they might be packaged into the phage head and injected into the host during infection (Figure 5a). Outside of the phages, Nudix domains are widespread in baculoviruses and NCLDVs and can be reconstructed as being present in the respective last common ancestor of these viral clades [91]. Multiple investigations of Nudix domains from diverse NCLDV such as poxviruses and algal viruses have shown them to be involved in decapping host mRNA to limit host protein synthesis [47,124,125]. Further, experimental investigations of the ORF38 Nudix domain of the baculovirus *Autographa californica* multiple nucleopolyhedrosis virus (AcMNPV) show that it uses ADPr as a substrate and is needed for successful replication of the virus in its host [48]. Hence, it is possible that in this case, the Nudix domain degrades ADPr derivatives involved in the host antiviral response. In light of this, and the chemical similarity of mRNA caps to NAD^+^/ADPr, it cannot be ruled out that at least some of the NCLDV Nudix domains implicated in mRNA decapping also play a role in the degradation of ADPr derivatives.

### 3.6. Phage-Encoded NAD^+^-Synthesis Domains

**Figure 6:**
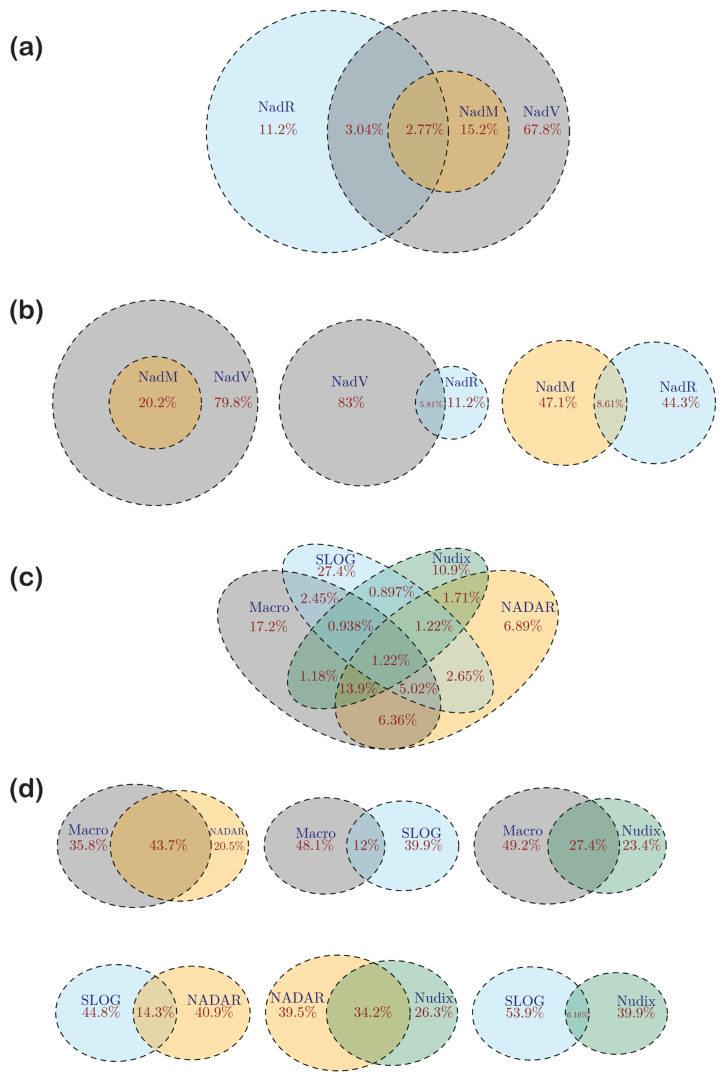
Complete and pairwise co-occurrence patterns of NAD^+^–ADPr domains depicted as Euler diagrams. (**a**,**b**) Domains involved in NAD^+^ biosynthesis/salvage in viruses. (**c**,**d**) Co-occurrence of the Macro, SLOG, Nudix and NADAR domains in DNA viruses. Co-occurrences are measured as a percentage of all the proteins that are being compared in a particular Euler diagram.

To counter cellular NAD^+^ depletion by NADase effectors from various conflict systems, certain phages encode enzymes that reconstitute NAD^+^ through a pathway that salvages nicotinamide (Figure 5b). The principal enzymes in this system include a NadV-like nicotinamide phosphoribosyltransferase (NAPRtase) and two distinct nicotinamide-nucleotide adenylyltransferases, NadM and NadR with a HIGH superfamily nucleotidyltransferase domain [122,123]. NadV-like PRTase has a sandwich-barrel hybrid motif domain at the N-terminus fused to the catalytic TIM barrel domain that synthesizes NMN from nicotinamide and 5-phosphoribose-1-diphosphate [14,44] (Figure 5b). The NMN synthesized by this protein is the substrate for the HIGH superfamily adenylyltransferases NadM or NadR. In NadM, the adenylyltransferase domain is fused to a Nudix domain [122,123]. The former domain synthesizes NAD^+^ from NMN (Figure 1b). By cleaving the phosphodiester linkage in NAD^+^, the Nudix domain in NadM regulates NAD^+^ levels. In NadR, the nucleotidyltransferase domain is fused to a P-loop kinase domain which phosphorylates nicotinamide riboside, a substrate for the nucleotidyltransferase to generate NAD^+^ [14,122]. Among the three enzymes, NadV is the most widely present and found in 22% of the medium-sized and 33% of the jumbo phages as compared to about 2% of small phages. Every NadM containing phage also has NadV suggesting that NadM strictly works with NadV in phage NAD^+^ biosynthesis. NadR is also present in medium and jumbo phages and might cooccur with NadM and NadV or both in the same genome (Figure 6a,b). Curiously, despite the prevalence of counter NAD^+^-effectors in eukaryotes, we currently find no evidence for NAD^+^ salvage in an eukaryotic virus.

### 3.7. Reconstructing Viral NAD^+^–ADPr-Based Systems

While some of the above-discussed domains are specific to particular cellular functional systems (e.g., DNA ligases or NAD^+^-biosynthesis enzymes), others participate in a disparate set of processes. Hence, to better understand their roles in the virus cycle, we systematically analyzed their domain architectures, gene-neighborhoods, and gene positions within the viral genome (contextual information) and combined it with a classification of these domains (including phylogenetic analysis) to glean additional information regarding their roles. Based on this, we present below our findings in terms of the broad functional systems in which the above domains can be situated.

#### 3.7.1. Systems Involved in Macromolecular Modifications Are the Largest Group of DNA Viral Domains in the NAD^+^–ADPr Network

Our analysis revealed that by far the largest group of domains from the NAD^+^–ADPr network in DNA viruses are those involved in covalent ADPr modifications. While these are mostly catalyzed by members of the ART and sirtuin superfamilies, their involvement in multiple processes required us to perform a more detailed investigation for teasing apart their participation in different functional systems. First, we used our previous evolutionary classification of ART domains to query the affinities of the viral ARTs. Broadly, the ARTs fall into three great clades, named H-H-h, H-Y-[EDQ] and the R-S-E clades after the profile of their conserved active site residues, each containing several distinct families [23]. Of these, the H-H-h clade is primarily involved in RNA repair, the modification of small molecules and the generation of soluble ADPr derivatives, while the remaining two feature enzymes that either modify macromolecules or act as NADases/NAD^+^-phosphorylases [19,23,126]. We found that the viral versions hail from 16 of the previously defined ART families. We combined this information with their contextual connections to arrive at a more precise functional prediction for them.

As a result, we found that the most frequently occurring viral ARTs belong to the N4-like, PART (PARP), ORF28, and the p0068-like families of the HY-[EDQ] clade, and the T4Alt/VIP2 and Mod families of the RSE clade. All the biochemically characterized members of these families catalyze protein or nucleic acid modifications through mono-ADP-ribosylation (e.g., T4 Alt and T4 ModA) [6], protein-RNAylation (e.g., T4 ModB) [26] and poly-ADP ribosylation (PARTs) [23,31]. The enigmatic ORF28 family found in evolutionarily distinct arthropod DNA viruses, namely baculoviruses, ascoviruses and iridoviruses [23], is related to the DarT-like DNA-modifying ARTs [30]; hence, a comparable action on nucleic acids remains a possibility for this family.

Further, we observed: (1) a significantly skewed enrichment (χ^2^ p < 2.2 × 10^−16^) of ARTs in Myoviruses (Figure 2b). In these viruses, they may be fused to head-associated/portal proteins (Figure 5a) [23,29]. In particular, many ARTs from the Alt/VIP2 and N4-ART families are fused to a MuF domain (Figure 5a), which is a structural component of the heads of caudoviruses utilizing the portal-terminase DNA-packaging system [115,127,128]. MuF is also fused to a wide array of other C-terminal enzymatic effectors that are packaged into the phage head. This is also in line with our earlier observation that several ARTs are part of polyvalent proteins, which combine multiple host-injected domains fused into a single polypeptide [29,115]. (2) ARTs are often encoded in the vicinity of the head-packaging and morphogenesis genes such as the terminase subunits, the portal protein, and other capsid components. Thus, they are likely synthesized along with these proteins and packaged into the phage head (Figure 5a). (3) Alternatively, in several phages, Alt/VIP2 family ARTs are encoded in genomic contexts identical to that seen in the phage T4. Overall, about 44% of phage ARTs recovered in our analysis are found in one of the above contexts suggestive of packaging into the phage head (Figure 5a). Thus, taken together, these observations suggest that the phage ARTs are often delivered into the host cell along with the viral DNA where they probably play a role in the hijacking of host functions via covalent ADPr modifications of macromolecules. As a corollary, ARTs appear to be deployed right from the earliest stages of infection, suggesting that ADP-ribosylation is one of the “first-line strategies” deployed by phages. Alternatively, the above contextual associations might also imply that the activity of these ARTs could counter immune processes that target components of the packaging apparatus (e.g., the portal or the terminase).

Our contextual analysis revealed that remaining viral ARTs are involved in a diverse array of systems that are likely to selfishly enhance host fitness to improve viral propagation. Several bacterial toxins targeting their eukaryotic hosts or their close homologs are encoded in phage genomes, such as MTX/Pierisin-type insecticidal toxins, Scabin, Cholera Toxin (CtxA), pertussis toxin, and the vegetal insecticidal toxin (VIP2) among others are encoded in phage genomes [93,129,130,131]. The emergence of such a function is sometimes accompanied by fusions to domains involved in adhesion and secretion, which have no specific role in virus–bacterium conflicts. For example, the ART domain of the mosquitocidal toxin Mtx is fused to Ricin-like lectin domains that aid in the adhesion and delivery of the toxin into the eukaryotic host of the virus [132] (Figure 5c). We observed that the HYD2 family of ARTs is shared by caudoviruses with bacterial polymorphic (and related) toxin systems [133,134]; thus, it could potentially function as a toxin or virulence factor [29]. Such systems might be of special use during (pseudo)lysogeny to facilitate improved survival of the phage’s host or compete better in their ecosystem.

In contrast to their prokaryotic hosts, we only found a small number of viral T–A systems that feature ART toxins (Figure 5d). A subset of these display ARTs that are likely to generate low-molecular-weight ADPr derivatives; these will be discussed separately below. However, some, such as an ART-ARG toxin–antitoxin pair in certain *Aeromonas* phages (comparable to the DraG-DraT system found in several cellular genomes), can be clearly predicted as macromolecular modifiers (Figure 5d). We also found another novel type-II T–A system from several *Escherichia*, *Shigella* and *Salmonella* enterophages which combines a predicted ART toxin of the p0068 family with a Dmd (discriminator of mRNA degradation) domain antitoxin [135,136,137]. Interestingly, in certain *Klebsiella* phages, the same Dmd antitoxin is paired with a distinct ART of the Mod-like family in the toxin position (Figure 5d). The prototypical phage T4 Dmd protein acts as a viral antitoxin that binds and inhibits the action of the HEPN domain endoRNase toxin RnlA from the host RnlA-RnlB T–A system that provides immunity against viruses [136]. Hence, in the systems we have uncovered here, the Dmd protein might similarly function as an antitoxin that counters the activity of more than one family of ART toxins. Given the anti-RNA action of the RnlA-RnlB system, it is tempting to speculate that these phage ARTs might function via modifying RNA substrates or protein RNAylation in the case of the Mod-like ART [135,137]. Parallel to the above association, we found a distinct type-II T–A system in actinobacterial phages typified by the *Streptomyces* phage Moab, which combine a gene for a sirtuin with one for the Macro domain (Figure 5d). This system is predicted to function similar to the previously described cellular systems with the sirtuin ADP-ribosylating target proteins and the Macro domain deconjugating those modifications [138].

Our contextual analysis suggested that the majority of viral sirtuin domains define a distinct clade that is likely to be involved in RNA repair (see Section 3.7.3). However, we found a small subset of the viral sirtuins to likely function as macromolecular ADP-ribosyltransferases in other virus–host conflicts. Notable among these are the sirtuins from phages that infect streptococci, such as *Streptococcus* phage phi-SgaBSJ27_rum, which are encoded as part of a two-gene system. The second gene in this system codes for a HerA-like P-loop ATPase that is predicted to function as a DNA translocase (Figure 5d) [98]. Comparable systems are also widespread in prokaryotic cellular genomes and include variants where the HerA-like ATPase is coupled with a gene for a DNA-binding KTSC domain protein [14] and an endoDNase in lieu of the sirtuin gene (LA, unpublished observations). Based on the mutual exclusivity of the sirtuin domain with the endoDNases we propose that it might target DNA by ADP-ribosylation. Indeed, DNA ADP-ribosylation by ARTs of the DarT, CARP and MTX/pierisin families has been shown to induce apoptosis [30,79,129]. Hence, we propose that these sirtuin-encoding gene-neighborhoods specify potential restriction systems that might trigger ADP-ribosylation on sensing invasive DNA. Systems such as this and the above-described T–A systems are predicted to be part of the phage repertoire of mechanisms to abort superinfection of their host by other viruses, especially during lysogeny.

#### 3.7.2. Systems Deconjugating ADPr Adducts to Macromolecules Are Widespread in Animal RNA Viruses

As noted above, viruses possess three unrelated domains, Macro, ARG and NADAR that can deconjugate ADPr moieties from macromolecular substrates. However, the same superfamilies of domains also release ADPr from a variety of low-molecular-weight substrates (Figure 1b). Moreover, the entire range of substrate specificities of the viral versions of these domains remains insufficiently studied. Hence, distinguishing between the two types of activities of these domains through sequence analysis alone can be challenging. The best evidence for the deconjugation of macromolecular ADP-ribosyl adducts comes from the Macro domains of animal RNA viruses, which have been proposed to remove Mono-ADP Ribosyl and Poly-ADP Ribosyl adducts of proteins [32,81,87]. NADAR domains are found in a similar group of positive-strand RNA viruses and, like the Macro domains, tend to be embedded in the polyproteins along with the viral RNA-dependent RNA polymerase [40]. The Macro and NADAR domains are largely mutually exclusive of each other in animal RNA viruses suggesting that they are likely to target similar ADP-Ribosyl moieties.

A priori there is nothing that precludes these RNA viral Macro and NADAR domains from being involved in other systems, such as RNA repair. However, we find a correlation between the occurrence of Macro domains in eukaryotic viruses and the presence of mono- or poly-ADP-ribosylation-based immunity mechanisms in their hosts. Several members of the ART superfamily from Metazoa and certain microbial eukaryotes have been implicated in anti-viral defense [23,24,40,139,140]. There is currently no evidence for land plants displaying ADP-ribosylation-based immunity. Consistent with this, we find Macro domains only in viruses infecting animals and microbial eukaryotes but not land plants. This supports the proposal that the Macro and NADAR domains of eukaryotic RNA viruses primarily target macromolecular ADP ribosylation by host enzymes. Apart from the phage-encoded T–A systems, where the coupling with ARTs or sirtuins strongly supports a role for the Macro, NADAR and ARG domains in the deconjugation of ADP-ribosyl adducts from macromolecular substrates, direct evidence for such an activity is lacking for the other phage versions of these domains. However, as noted above, despite having comparable catalytic activities, the Macro and Nadar domains outnumber ARG domains in prokaryotic viruses, suggesting that their substrates are not equivalent. By this argument, it is conceivable that the phage exemplars of Macro and Nadar domains have distinct activities from the ARGs they encode, with the latter primarily targeting macromolecular ADPr conjugates. Notably, the application of a contextual logic regarding host immunity mechanisms, similar to that presented above for the eukaryotic RNA viruses, suggests that phage Macro and NADAR domains, in contrast to their RNA viral counterparts, predominantly target low-molecular-weight ADPr derivatives (see Section 3.7.4). Nevertheless, it is conceivable that at least a few of the phage-encoded Macro and NADAR, like the ARG domains, could deconjugate macromolecular ADPr adducts to counter specific bacterial immune mechanisms involving macromolecular modifications (e.g., ADP-ribosylation of DNA by T–A systems).

#### 3.7.3. Systems Involved in RNA Repair

Previous studies have shown that a common denominator across diverse virus-restriction systems in both eukaryotes and prokaryotes is endoRNase domains that target RNAs in the translation process [13,29,52,102,134]. The cleaving of the anticodon loops of tRNAs associated with the ribosome is particularly effective as it helps “jam” the translation pipeline and prevents the synthesis of viral proteins [53,141]. Phages have evolved several counter-mechanisms in the form of RNA repair/ribosome rescue processes. These include encoding their own tRNAs that substitute for the cleaved host tRNAs [14,142,143,144], repairing cleaved tRNAs using RNA ligases, and using template-dependent or independent nucleotidyltransferases to restore missing bases in conjunction with the RNA ligases [51,53,56,145,146,147,148]. The most common endoRNases from conflict systems tend to be metal-independent enzymes that leave a 2′-3′ cyclic phosphate RNA terminus. While there are several mechanisms to “clean up” such termini, one of the most widespread mechanisms, both in cellular and viral genomes, is the use of a 2H phosphoesterase to cleave the 2′-3′ cyclic phosphate leaving behind a 2′ phosphate [45,53]. One means by which this 2′ phosphate is further processed is via the ART domain of KptA (also known as Tpt1 or the RNA 2′ phosphotransferase) family. This enzyme transfers ADPr from NAD^+^ to the 2′-phosphate resulting in it leaving as ADPr > P [46,54,57]. Thus, one wing of the RNA-repair system is intimately tied to the NAD^+^–ADPr network.

While RNA ligases and 2H domains are widespread in phages with genome sizes comparable to T4 and larger, some of the latter, especially jumbo phages, also code for KptA indicating that they facilitate the restoration of tRNAs via an NAD^+^-dependent mechanism [14]. KptA is absent or rare in archaeal and eukaryotic viruses. This might relate to the fact that KptA is a widely conserved cellular RNA-repair enzyme in the archaeo-eukaryotic lineage that might be available for cooption by their viruses [40]. Nevertheless, we found KptA domains in the nucleopolyhedrosis viruses that infect sawflies, and the *Bodo saltans* mimivirus. In the latter virus, the KptA ART domain is fused to a Nudix domain, suggesting that it likely further processes the ADPr derivatives generated by the action of the former domain (Figure 5h). Interestingly, we found a few distantly related positive-strand RNA-viruses, such as the Beihai picorna-like virus and *Botrylloides leachii* nidovirus, to also embed a KptA domain in their polyproteins. Thus, while rare, KptA joins the other animal RNA viral polyprotein-embedded domains from the NAD^+^–ADPr system, namely the Macro, NADAR and 2H. This raises the possibility that, like KptA, those latter domains might also play a role in RNA-processing in these viruses. Beyond “suicidal” attacks by host effectors on the translation apparatus, these viruses might also face other challenges related to the processing of their own genomic and sub-genomic RNAs (e.g., by viral metal-independent endoRNases such as EndoU) [149] or direct attacks on their genomes by host RNases [150,151,152,153]. Hence, it is possible that in addition to their other proposed roles, these domains also play a role in the NAD^+^-dependent processing of the 2′-3′ cyclic phosphate termini of viral genomic and sub-genomic RNAs generated by the above-stated endoRNases.

We also uncovered potential novel NAD^+^-utilizing RNA-repair systems in phages, which are centered on a distinct clade of sirtuin proteins, that parallel the above-described KptA-based systems. They are widely represented in proteobacterial phages and are encoded in the following conserved gene neighborhood configurations: (1) In several jumbo phages, such as *Serratia* phage BF, the gene encoding the sirtuin (e.g., AQW88637.1) is linked to genes coding for 2H, Nudix, cREC and a PIN domain endoRNase. Sometimes, like in the *Serratia* phage BF, the Nudix and cREC genes are fused together (Figure 5f). (2) In the second type of gene-neighborhood seen in a variety of medium-sized phages prototyped by the *Escherichia* phage V5 and the *Proteus* phage Mydo, the sirtuin gene is coupled to genes encoding a T4-like tRNA ligase, a cREC protein, a nucleotide kinase, a MazG superfamily pyrophosphatase [154] and occasionally a calcineurin-like phosphoesterase domain (Figure 5f). (3) In the third type of association seen in a group of phages typified by the *Escherichia* phage SP15, the sirtuin gene is joined in a conserved neighborhood by a “Swiss-army-knife-like” HAD superfamily phosphatase involved in processing terminal phosphates in RNA (Figure 5f) [53]. Thus, the common thread connecting all these gene-neighborhoods featuring a distinct version of the sirtuin domain is that they code for proteins involved in the clean-up of RNA ends with 2′-3′ cyclic phosphates for their subsequent ligation. Unlike the T–A systems, here the sirtuin gene is not coupled to an adjacent gene for a Macro domain. Hence, we proposed that the ADP-ribosyltransferase activity of these sirtuins is deployed in a manner analogous to that of KptA in removing the 2′-phosphates generated from 2′-3′ cyclic phosphates in RNAs cleaved by metal-independent nucleases [14,53]. Indeed, the associated 2H, cREC, HAD, calcineurin-like and Nudix enzymes in these systems (Figure 5f) could either act as the initial enzymes that resolve the cyclic phosphate to a 2′ phosphate or subsequently process the ADPr>P generated by the proposed action of the sirtuin.

#### 3.7.4. Viral Networks Generating and Processing Low-Molecular-Weight NAD^+^–ADPr-Derived Metabolites and Signaling Messengers

Our analysis also points to a wealth of virally encoded domains that are known or predicted to process and recognize low-molecular-weight NAD^+^ and ADPr derivatives. These can be inferred to participate in several disparate biological conflict systems that might be either deployed by the virus to (temporarily) enhance its host fitness or to counter the host NAD^+^–ADPr-dependent immunity mechanisms. The former systems include:

(1) Modification enzymes that confer resistance against small molecule toxins—for example, the ARTs of the rifampin ADP-ribosyltransferase family that are predicted to modify rifamycin-like antibiotics [155]. These are borne by several phages of the actinobacteria *Microbacterium* and *Gordonia*, and the *Sinorhizobium* phage PBC5. A straightforward interpretation of these proteins would be that the virus confers resistance to their host against such antibiotics produced by rival bacteria. However, one cannot rule out the possibility that these ARTs are deployed against diffusible compounds that might participate in immunity against viruses as hinted by the production of such molecules in the recently discovered apoptosis and antiviral systems of actinobacteria [100,156,157].
(2) Some T–A systems borne by phages feature a divergent ART family, RES, coupled to a helix-turn-helix transcription factor antitoxin (Figure 5d) [19]. In the RES family conserved S and E residues typical of the R-S-E clade of ARTs have been substituted by a Y and an N, respectively. These ART domains catalyze the phosphorolysis of NAD^+^ to generate ADPr-1″P, a toxic metabolite, that might either inhibit superinfecting phages or induce host dormancy to survive adverse environmental conditions [19].
(3) Some mycobacterial phages also code for polymorphic toxin systems that display a Ntox40 (TNT) family ART toxin domain coupled to a gene for its characteristic immunity protein Imm63 [134]. Recent studies have shown that the NADase activity of the Ntox40 domain from mycobacteria is also directed against the macrophages of their animal host, wherein it degrades NAD^+^ to ADPr and triggers their apoptosis [158,159](Figure 5c). Similarly, we found a Type-VII secretion system-dependent polymorphic toxin with an Arc domain and its cognate immunity protein (Imm74) toxin [134] to be encoded in the *Streptococcus* phage phi-SsuFJNP8_rum (Figure 5c). Based on the precedence of the experimentally characterized Ntox40 system, here too it is conceivable that the Arc toxin acts in targeting the immune response of the eukaryotic host of the *Streptococcus* phage. The Arc domains generate cADPr or NAADP from NAD^+^ and NADP; given that these molecules are also generated by the cell-surface receptor Arcs in animal immune systems (e.g., CD38 and CD157) and have an important role in the antibacterial response [15,58], it is possible that virally encoded Arc domain toxins provide a mechanism for the bacterium to interfere with its host’s immunity. Moreover, polymorphic toxins are a key mechanism for kin-cooperation among bacteria [134]. Thus, in both these cases, the virally encoded ART and Arc polymorphic toxins appear to be part of a multilevel biological conflict. The virus, when in a (pseudo)lysogenic state likely enhances its own survival by both fostering kin-cooperation in its bacterial host and aiding it against the immunity of the bacterium’s eukaryotic host.
(4) A relatively small set of prokaryotic and eukaryotic viruses code for TIR and Arc domains (outside of polymorphic toxins). TIR domains are carried by multiple firmicute phages (e.g., the *Streptococcus* phage Javan281 and the *Staphylococcus* phage SN10), typically in a conserved gene–neighborhood association with the 2TM SLATT domain (Figure 5g). These gene neighborhoods sometimes also code for a helix-turn-helix (HTH) domain transcription factor or a zincin-like metallopeptidase. The SLATT domain is predicted to function as an ADPr derivative-regulated effector controlling membrane permeability across diverse prokaryotic antiviral systems [12]. The TIR and Arc domains found sporadically in NCLDVs have been respectively acquired from their eukaryotic hosts and bacterial endosymbionts. Given their role in host immune systems, their presence in viruses is rather enigmatic. In the case of the TIR domain, it is conceivable that it limits superinfection via an apoptotic mechanism that relies on kin selection in a host population/multicellular ensemble that has either been lysogenized or carries the dormant virus. The cADPr or NAADP signals generated by the Arc domain could also manipulate host immunity in favor of the virus.

One of the major developments in the past decade is the realization of the centrality of the NAD^+^–ADPr network to antiviral immunity. A series of studies by us and others have shown that, both in prokaryotes and eukaryotes, the targeting of NAD^+^ plays a key role in limiting viral replication by: (1) signaling immune responses via the NAD^+^-derived small-molecule messengers, such as different varieties of cADPR or NAADP; (2) depletion of NAD^+^ through NADase activities resulting in apoptosis or dormancy; and (3) generation of toxic metabolites from NAD^+^, such as ADPr-1″P (and also probably ADPr > P) [11,12,13,15,16,18,20,38,39]. The first two processes are dominated by domains of the TIR, DrHyd, SLOG, sirtuin and ART superfamilies, while the third is currently known to be catalyzed by the RES family of ARTs [19]. Notably, our systematic survey showed that, contrary to the extensive presence of Macro, NADAR, SLOG and Nudix domains in viruses, there is a significant underrepresentation of TIR, DrHyd, and ARTs with NADase activity (χ^2^ p < 2.2×10^−16^, Figure 2c). However, prokaryotic immune systems deploy TIR, sirtuin, and ART domains of the RES and Frg clades to a greater extent than domains catalyzing ADP ribosylation of macromolecules, which are widespread in the antiviral response of a subset of eukaryotes [24]. Hence, contrary to what has been currently reported for the eukaryotic viruses, we suggest that the extensive distribution of Macro, NADAR, SLOG and Nudix domains in phages is primarily an adaptation to counter low-molecular-weight ADPr derivatives used as diffusible signals or toxins by prokaryotic immune systems.

Outside of the polyproteins of eukaryotic RNA viruses (Figure 5e), the viral exemplars of Macro, NADAR, SLOG and Nudix domains are typically standalone proteins. In phages, genes coding for Macro, NADAR and Nudix proteins might be encoded either in genomic regions that are transcribed early along with other host-manipulation and RNA repair functions or among the genes associated with virion morphogenesis and are likely packaged into the head (Figure 5a,h). Thus, in either case, they are likely deployed early in the infection cycle to head off the host NAD^+^–ADPr-dependent antiviral response. However, in sharp contrast to the animal RNA viruses, there is no tendency for mutual exclusion of NADAR and Macro domains among DNA viruses (Figure 6c). Instead, Macro, NADAR, SLOG and Nudix domains show a greater tendency to cooccur in the genomes of DNA viruses than would be expected by chance alone. Further, across diverse DNA viruses, these domains show distinctive domain architectures and/or conserved gene neighborhoods that often combine the SLOG with Macro and NADAR domains (Figure 5h). The hosts probably deploy multiple cyclic and linear ADPr derivatives as diffusible signals and toxins in their immune response (Figure 1b). In addition to the previously characterized cADPr variants, NAADP, ADPr > P and ADPr-1″P [16,19,41,78], these might include as yet uncharacterized derivatives and probably molecules processed from the widely reported NAD^+^-caps of RNAs. Hence, in light of the above, we propose that there is a division of labor among the ADPr derivative-processing domains and their coupling probably helps the virus to either tackle more than one such molecule or process the molecule by targeting more than one bond (Figure 1b).

Based on the precedence of the previously studied cytokinin-biosynthesis and TIR-SLOG-based antiviral systems [12,39], we propose that the SLOG domains probably operate both as sensors for ADPr derivatives such as cADPr and as specialists that target the 1′ ribosyl N-glycosidic linkages. In contrast, based on the known activities of the Macro domains, it is likely that different versions process various moieties linked to the nicotinamide-free ribose in ADPr/cADPr variants (Figure 1b). The NADAR domains probably process N-glycosidic linkages to either ribose of ADPr, perhaps including cADPr (Figure 1b). The Nudix domains have been characterized as processing the central diphosphate in NAD^+^ or ADPr [160]. We also found a distinctive clade of the rhodanese-phosphatase superfamily (overlaps with Pfam “Domain of Unknown Function”: DUF4326) in several DNA viruses, often fused to SLOG, Macro and NADAR domains (Figure 5h). Based on a comprehensive analysis of these domains (AMB and LA, manuscript in preparation), we propose that the DUF4326 domain likely processes the terminal phosphates linked to ribose in ADPr or its breakdown products. Notably, the presence of the DUF4326 domain both in phages and NCLDVs, especially those infecting microbial eukaryotes, points to parallel action against diffusible ADPr derivatives in eukaryotic contexts. Finally, we observed that in several phages, SLOG domains show a range of sporadic fusions to HTH, HNH, 3′-5′ exonuclease, Superfamily-1 helicase, and guanylate kinase domains (Figure 5i). These fusions to preponderantly nucleic-acid-binding/processing domains suggest that the activity of these proteins in DNA repair, anti-host restriction, or transcriptional control is potentially regulated by the SLOG domain sensing NAD^+^/ADPr derivatives generated by host immune activity.

### 3.8. Prediction of Immune Mechanisms That Counter Viral ADPr Modifications of Host Macromolecules

While there is a growing appreciation of the antiviral immune activities centered on NAD^+^-restriction and ADPr derivative-dependent signaling and toxicity, there is hardly any understanding of the host counter-measures against virus-directed ADP-ribosylation of host macromolecules. Given that we find an extensive presence of ARTs across large DNA viruses (Figure 2b), we conjectured that hosts should possess dedicated systems to counter this widespread virally induced modification of host molecules [23]. This would be apposite given the growing evidence that the modifications of host macromolecules play a role in hijacking them for viral functions since the earliest studies on the T4 Alt and ModA/ModB model [6]. Accordingly, we developed a comparative genomics and sequence/structure analysis protocol to predict potential host systems that might be involved in such a process. We started by focusing on enzyme families that are known/predicted to deconjugate macromolecular ADPr adducts and examined them for the absence of genomically coupled ARTs or sirtuins. Such a pattern would suggest that they might not function as the versions of these domains in the previously characterized ADPr transferase-coupled systems (e.g., DraG-DraT; DarT-Macro/NADAR; Macro-sirtuin), wherein they deconjugate the ADPr modifications generated by the coupled ADP-ribosylating enzyme. Instead, the absence of the modifying enzyme would imply that they act on macromolecules that have already been modified by an extrinsic ADP-ribosylating enzyme (e.g., one encoded by an invading virus).

**Figure 7:**
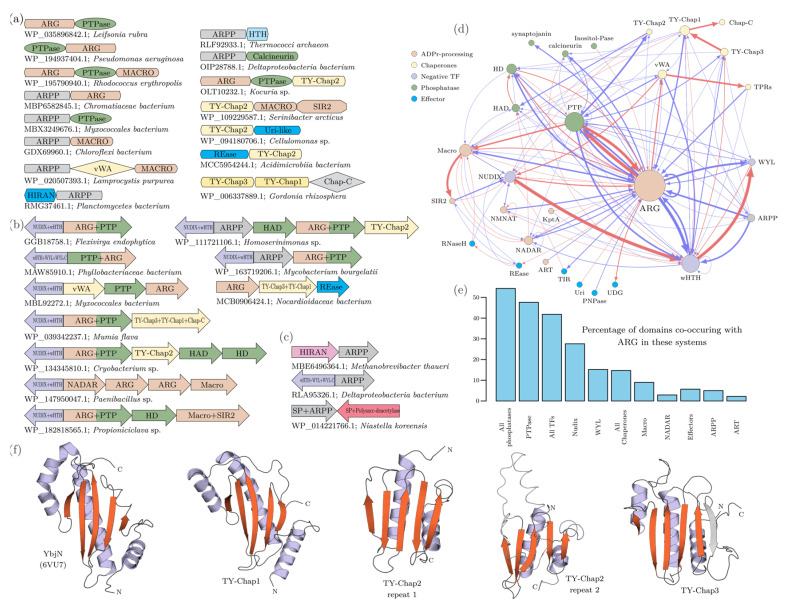
Contextual associations including (**a**) domain architectures and (**b**) gene neighborhoods of the ARG-associated systems. (**c**) More gene neighborhoods of the ARPP domain. (**d**) Contextual network diagram and (**e**) co-occurrence frequencies of domains associated with the ARG domain. (**f**) Structural comparison of the newly identified members of the TY-chaperone superfamily found in these systems.

Our search uncovered a widespread system centered on an ARG domain that is present in several bacterial lineages, including actinobacteria, planctomycetes, and diverse proteobacteria, which followed the above template (Figure 7a–e). Given that the phyletic patterns of ARG domains across viruses supported their preferred action on macromolecular ADPr adducts as opposed to soluble ADPr derivatives (see Section 3.4.2), these are good candidate systems for countering the ADP-ribosylation of host proteins by viral enzymes. Over 42% of these systems feature one-component negative regulatory transcription factors (TFs) that contain either a Nudix [109,161,162] or a WYL domain [53,109] as the sensor fused to a DNA-binding HTH domain (Figure 7a,b,f). The mutually exclusive presence of either a Nudix or WYL TF in these systems suggests they are likely to be alternative sensors of the same or similar ligands (Figure 7d). Previous studies on related TFs with a Nudix domain sensor (NrtR) have revealed that they indeed sense ADPr [162,163,164]. Likewise, the WYL domain TFs have been previously found in conflict systems where they are known or predicted to respond to cyclic oligonucleotides or RNA fragments with cyclic ends [109,110,165]. Hence, these ARG-containing systems are likely regulated by the Nudix or WYL TFs via the sensing of ADPr moieties. In a little over 55% of these neighborhoods, the ARG is coupled with a phosphatase domain either in the same polypeptide or coming from a tightly coupled adjacent gene (Figure 7a,b,f). Most commonly, this phosphatase is a member of the rhodanese-phosphatase (R-P) superfamily of metal-independent enzymes that extract terminal phosphate groups from protein tyrosines/serines/threonine or sugar/sugar alcohol substrates via phosphotransfer to an active site cysteine residue [166,167,168]. The other phosphatase domains that might occur in lieu or in addition to the R-P domains include a calcineurin-like phosphatase, a HD phosphatase related to those acting on the alarmone nucleotide and a HAD superfamily 5′ nucleotidase (Figure 7b) [169,170,171,172]. The rampant coupling of these phosphatases suggests that the ADPr cleaved off by the ARG domain is processed further by these phosphatases, likely in conjunction with the Nudix domain of the TF (Figure 7b).

These gene neighborhoods often display an additional gene encoding one of several predicted peptide-binding chaperones (Figure 7a,b). Among these is the vWA domain, a Mg^2+^-dependent peptide-binding chaperone, previously reported in several distinct systems [157] (Figure 7b). The contextual analysis of these systems also revealed a link to a SseB domain (Figure 7b), which has been previously implicated in the delivery of proteins via the Type III Secretion System (T3SS) pore [173,174]. Our sequence analysis of the SseB domain revealed a relationship to the Tic22 family of peptide-binding chaperones (HHpred probability 89%) involved in protein translocation [175], suggesting that the SseB domain is likely to function as a chaperone, too. We found evidence for a chaperone function for three further families (TY-Chap1, TY-Chap2 and TY-Chap3) of previously uncharacterized proteins that are found in these ARG-centric systems (Figure 7b,c). One of these (TY-Chap1) could be unified through sequence-profile searches (HHpred probability 85–98%) with T3SS (e.g., YopN, CesT) and the YbjN superfamily [176,177,178] of peptide-binding chaperones (TY-chaperones). Structure modeling using the RoseTTAFold algorithm indicated that TY-Chap2 is comprised of two copies of a domain with a β-sheet formed by a 5-stranded meander flanked by two helices that stack on the same side of the sheet (Figure 7f). This structural topology is identical to the TY-chaperones (recovered in DALI searches as the best hit with Z-score 5.6). The same approach indicated that TY-Chap3 likewise adopts a circularly permuted version of the TY-chaperone fold. Hence, both these domains are predicted to also function as chaperones. We propose that these predicted peptide-binding chaperone domains are likely to target proteins that are modified by ADPr and potentially misfolded as a consequence.

Finally, the conserved cores of these neighborhoods are often accompanied by an additional set of tightly coupled but variable genes that code for (Figure 7a,b): (1) other ADPr-processing domains such as Macro and NADAR. (2) A set of restriction endonuclease fold domains (REases) that have previously been found as effectors in a wide range of other biological conflict systems [11,12]. These might be further linked to genes encoding helicases. Among the REases was a previously unknown rapidly evolving REase domain—a classic hallmark of systems deployed in antiviral response (Figure 7a,b, Supplementary S6). (3) The ARG and Rhodanese-Phosphatase-superfamily-associated Protein (ARPP). While this protein is unrelated to any other previously characterized domains, it shows a distinguishing group of absolutely conserved residues, including a cysteine and a glutamine, suggestive of enzymatic activity. Structural predictions for ARPP using the RoseTTAFold algorithm revealed a core β-sheet comprised of a duplicated four-stranded β-meander with a β-barrel domain bearing the absolutely conserved residues inserted into the first repeat (Supplementary Materials S5). Outside these ARG-centered systems, we found ARPP in three other conserved two-gene neighborhoods respectively (Figure 7c) with: (1) a DNA 3′-end binding HIRAN domain [179,180]; (2) a WYL domain TF [53,109]; (3) an aminosugar deacetylase/glycosidase domain (Pfam: PF01522); in the latter association, both proteins contain signal peptides suggesting they are exported as a pair outside of the cell. The unifying biochemical theme across these diverse contextual linkages of ARPP is the presence of molecules with sugar moieties (ADPr, nucleotides and polysaccharides). Hence, we propose that in the ARG-centric systems under consideration ARPP likely acts on the ribose moieties coming from ADPr. The presence of the conserved cysteine suggests that it could function analogous to the recently characterized SRAP domain [181] by forming covalent cross-links with sugars [182].

In conclusion, these ARG-centric host systems present several features, which when taken together bear the hallmarks of conflict systems: (1) rapid evolution at the sequence level; (2) a conserved core embellished by variable modules that might be accreted or lost between systems from closely related genomes; and (3) displacement of functionally equivalent domains, chaperones or sensor domains of negative regulatory TFs implying selection for alternative versions that might sense different variants of a related signal (Figure 7b,d,e). Hence, we propose that these systems represent the first examples of host conflict systems likely to combat the ADP-ribosylation catalyzed by viral ARTs. Briefly, we advance the following plausible mechanism of their action. The presence of the negative regulatory TFs suggests that these operons tend to be off by default. However, upon phage-induced ADP-ribosylation of host macromolecules, a few ADPr or nucleoside molecules are probably released via the action of the ARG and associated phosphatase domains. These might then act as inducers that bind the Nudix or WYL domains of the TFs to release the operons from their transcriptional repression. This would then allow active transcription of these operons with the resulting increased production of the ARG and phosphatases to remove the ADPr modifications from the modified targets. The associated chaperones might specifically help bind ADP-ribosylated target proteins, whereas the effector domains when present might provide a second line of defense by either directly targeting viral nucleic acids or acting suicidally on host nucleic acids to induce dormancy [11,13]. The occasional coupling to other ADPr-acting enzymes encoded in these neighborhoods (Macro and NADAR) could supplement the core ARG function by processing different ADPr conjugates (e.g., PolyADPr or RNAylation).

### 3.9. Evolutionary Considerations

While NAD^+^ was discovered as a key metabolite over a century ago [183], the full extent of its entanglement with various cellular pathways has become clear only in the past two decades. In evolutionary terms, the study of NAD^+^-utilizing enzymes makes it clear that the two alternative modes of NAD^+^-binding by a characteristic Rossmannoid domain scaffold, respectively typified by the NAD^+^-dependent oxidoreductases and the sirtuins, were already present in the last universal common ancestor (LUCA) of all extant Life [40]. On the redox side, the ancestral NAD^+^-dependent reactions played a central role in energy- and reducing-potential-generation via the glycolytic pathway [9]. In contrast, the sirtuins were probably involved both in cobalamin synthesis from NMN and NAD^+^-dependent deacylation and ADP-ribosylation of proteins and potentially other macromolecules even in the LUCA [96]. Thus, an ecosystem of enzymes using NAD^+^, both as a cofactor and as a substrate, is inferred as being already present prior to the LUCA. Several recent studies focused on organismal longevity indicate that NAD^+^ is the linchpin for metabolism and its appropriate balance is critical for cellular health [184]. Thus, it is not surprising systems restricting NAD^+^ have come to play an important role in immunity across the tree of Life.

Against this backdrop, as part of this study, we found a relationship between the size of viral genomes and the encoding of domains from the NAD^+^–ADPr-network—they tend to be present in larger as opposed to smaller viruses with distinct size thresholds for DNA and RNA viruses (Figure 2a). As noted in Section 3.2, this appears to correlate with viruses that are more oriented towards an ecological K- as against an r-strategy [92]. To elaborate, smaller viruses, which follow more of an r-strategy, primarily rely on rapid replication as opposed to complex interactions with their host’s immunity and metabolism. Hence, any net benefit of coding for these domains might not be significantly greater than the advantages accrued from rapidly replicating a small genome. In contrast, larger viruses with more intricate and slower replication processes might have a net benefit from these domains, in terms of: (i) preventing NAD^+^-restriction by the host; (ii) countering NAD^+^-dependent host immune signaling; (iii) manipulating host systems via macromolecular modifications.

The above inference leads to the question of where the viruses acquired their NAD^+^–ADPr-network components? This is poignant since the larger viruses can be inferred to have evolved through extensive gene accretion from their smaller counterparts [90,91,185]. Our analysis reveals a combination of ancient origins in the early replicons that gave rise to viruses and a web of gene flow between viruses, their hosts and even co-resident endosymbionts. Some components such as the NAD^+^-dependent ligase are likely to have been inherited by the viruses from the ancient replicons that contributed to their origins. The separation of the NLig-Ia and the ATP-grasp domains of the ligase that we observed in viruses has no precedent in the cellular genomes (Figure 4b); thus, it could be a remnant from ancient replicons where these domains had not yet come together as a single unit. In other cases, such as the TIR domains, the polymorphic toxins and T–A systems the evidence points to more recent back-and-forth genetic exchange between viral and host genomes (Figure 3).

Several of the other domains from the NAD^+^–ADPr-network show evidence for extensive dissemination among distantly related viruses and from them to cellular genomes. Among eukaryotic viruses, namely NCLDVs and Baculoviruses, we found three principal families of ARTs. The most abundant are representatives of the Alt/VIP2 family (e.g., found in chloroviruses such as PBCV and various Mimiviruses). Our analysis indicated that these ARTs are specifically related to those from bacteriophages where they are often fused to the head-packaged MuF domain (Figure 3c). Likewise, the PART from the Invertebrate Iridescent Virus 6 can be shown as being derived from a phage PART. Similarly, we found multiple instances of gene-transfers of Macro and NADAR between eukaryotic DNA and RNA viruses and bacteriophages (Figure 3f). It is probable that the eukaryotic viruses acquired them from the phages of endo-symbiotic/parasitic bacteria that coinhabit the cells of their eukaryotic hosts. However, PARTs (e.g., ABI13815.1, *Anticarsia gemmatalis*_multiple Nucleopolyhedrovirus) and Ecto-ARTs (ATZ81114.1, *Bodo saltans* Virus) from other eukaryotic DNA viruses show evidence for being derived from their hosts or undergoing back-and-forth gene-transfers with them (Figure 3d). Such transfers point to the fungibility of these domains in virus–host conflicts across the entire range of evolutionary distances.

The viral ARTs also throw light on some aspects of the evolution of the weaponry used in host–pathogen interactions. We observed that several of the toxins of bacterial pathogens are related to ARTs from phages. Further, genome contexts of several of these ART genes indicative of packaging into the phage head are not different between phages that infect pathogenic and non-pathogenic bacteria (Figure 5a). For example, the CtxA-related heat-labile enterotoxin IIa of *Escherichia* phage Rac-SA53 is in the neighborhood of virion assembly and packaging genes encoding the terminase subunits just like ARTs from non-pathogenic bacteria (Figure 5a). Thus, several bacterial ART toxins deployed against eukaryotes, are likely to be examples of repurposing of phage ARTs that are typically deployed against their bacterial hosts. Finally, on a minimum of six independent occasions through the course of eukaryotic evolution, ARTs of phage provenance were transferred to eukaryotes either directly or via the intermediation of eukaryotic DNA viruses [23]. Interestingly, several of these appear to have been recruited as effectors in eukaryotic immunity, thus apparently reversing their original roles. Further, this use of ARTs as part of the immune response appears to be primarily directed against positive-strand RNA viruses in certain eukaryotic lineages. The resulting evolutionary arms race appears to have come to a full circle with the acquisition of Macro and NADAR domains by a diverse set of metazoan positive-strand RNA viruses to target macromolecular ADPr adducts generated by the host ARTs. Here again, the RNA viruses appear to have acquired these domains from other viruses (Figure 3) rather than directly from their eukaryotic hosts.

## 4. Conclusions

There have been several exciting, recent discoveries regarding the interplay of the NAD^+^–ADPr network with antiviral immunity and the counter-response of viruses against these host immunity mechanisms. However, a comprehensive picture of the viral adaptations pertaining to this system has so far remained unrealized. In this work, we have addressed that desideratum using a database of 21,191 complete viral proteomes that are representative of the entire virus world in currently available public sequence databases. We objectively classified the constituent domains of this network based on their biochemistry and systematically determined their presence in viruses. As a result, we were able to apprehend their evolutionary trajectories and make several predictions regarding their roles in NAD^+^-sensing, RNA repair, and multi-level biological conflicts. Among other things we show that the NAD^+^–ADPr network is pervasive across the viral world; however, the same domains might possess subtly distinct functions in different viral groups—for example, the processing of small diffusible ADPr derivatives versus macromolecular mono- or poly-ADPr adducts. We also present evidence that viruses might possess multiple sensory systems for NAD^+^ or ADPr derivatives based on domains such as SLOG and NLig-Ia that might interface with different aspects of viral biochemistry. Finally, we show that the use of ADPr modifications, likely of macromolecules, is a widespread strategy of DNA viruses that might be countered by specific host systems that are predicted for the first time in this work.

We hope that this study helps direct further experimental work to uncover as yet unexplored aspects of the biochemistry and biology of the NAD^+^–ADPr network in virus–host conflicts and helps in the development of new reagents utilizing these molecules.

## Supplementary Materials

The following supporting information can be downloaded at: https://www.mdpi.com/article/10.3390/v14091977/s1, Supplementary S1: List and features of viral genomes in the virus database. Supplementary S2: Tables with various summary statistics that are described in the text. Supplementary S3: Raw data used to build the phylogenetic trees shown in Figure 3. Supplementary S4: Contextual information and other features of viral proteins in the NAD+–ADPr network. Supplementary S5: Structure of the ARPP domain predicted using RoseTTAFold. Supplementary S6: Multiple sequence alignment of the rapidly evolving REase domain found in ARG-associated systems.
