# Supplementary material for: Apprehending the NAD+–ADPr-Dependent Systems in the Virus World
Source: Viruses. 2022 Sep 7;14(9):1977. doi: 10.3390/v14091977 (PMC9503650; doi:10.3390/v14091977)
Supplement: Supplementary file 1 [file viruses-1876971-supplementary.pdf › viruses-1876971-supplementary.pdf]

Supplementary Material

Apprehending the NAD^+-ADPr-dependent systems in the virus world

Lakshminarayan M. Iyer, A. Maxwell Burroughs, Vivek Anantharaman and L Aravind[1]*
[1]National Center for Biotechnology Information, National Library of Medicine, National Institutes of Health, Bethesda, MD 20894, USA

*Correspondence: aravind@mail.nih.gov

Abstract

NAD^+ and ADP-ribose (ADPr)-containing molecules are at the interface of virus-host conflicts across life encompassing RNA processing, restriction, lysogeny/dormancy and functional hijacking. We objectively defined the central components of the NAD^+-ADPr networks involved in these conflicts and systematically surveyed 21,191 completely sequenced viral proteomes representative of all publicly available branches of the viral world to reconstruct a comprehensive picture of the viral NAD^+-ADPr systems. These systems have been widely and repeatedly exploited by positive-strand RNA and DNA viruses, especially those with larger genomes and more intricate life-history strategies. We present evidence that ADP ribosyltransferases (ARTs), ADPr-targeting Macro, NADAR and Nudix proteins are frequently packaged into virions, particularly in phages with contractile tails (Myoviridae), and deployed during infection to modify host macromolecules and counter NAD+-derived signals involved in viral restriction. Genes encoding NAD^+-ADPr-utilizing domains were repeatedly exchanged between distant viruses, hosts and endo-parasites/symbionts, suggesting selection for them across the virus world. Contextual analysis indicates that the bacteriophage versions of ADPr-targeting domains are more likely to counter soluble ADPr derivatives, while the eukaryotic RNA viral versions might prefer macromolecular ADPr adducts. Finally, we also use comparative genomics to predict host systems involved in countering viral ADP ribosylation of host molecules.

***Note**: Please use the pdf document outline (visible in all pdf readers) to navigate the Supplementary material.

Index.

1. Supplementary S1: List and features of viral genomes in the virus database.
2. Supplementary S2: Tables with various summary statistics that are described in the text.
3. Supplementary S3: Complete phylogenetic trees shown in Figure 3.
4. Supplementary S4: Contextual information and other features of viral proteins in the NAD+-ADPR network.
5. Supplementary S5: Structure of the ARPP domain predicted using RoseTTAFold
6. Supplementary S6: Multiple sequence alignment of the rapidly evolving REase domain found in ARG-associated systems
    - Note: Use the bookmarks to navigate the pdf document

S1. Complete list of viruses analyzed in this study and their genome statistics

gca	genome.size	proteome.size	phage.name	strain	Taxonomy
GCA_000895895.1	17078	4	Rosellinia necatrix quadrivirus 1	NA	Viruses; dsRNA viruses; Quadriviridae; Quadrivirus
GCA_013086425.1	12516	4	Echarate virus	NA	Viruses; Riboviria; Orthornavirae; Negarnaviricota; Polyploviricotina; Ellioviricetes; Bunyavirales; Phenuiviridae; Phlebovirus; Echarate phlebovirus
GCA_002821845.1	2788	6	Chilli leaf curl Vellanad virus IndiaVellanad2008	NA	Viruses; Monodnaviria; Shotokuvirae; Cressdnaviricota; Repensiviricetes; Geplafuvirales; Geminiviridae; Begomovirus; Chilli leaf curl Vellanad virus
GCA_002821825.1	2754	6	Chilli leaf curl Kanpur virus IndiaKanpur2008	NA	Viruses; Monodnaviria; Shotokuvirae; Cressdnaviricota; Repensiviricetes; Geplafuvirales; Geminiviridae; Begomovirus; Chilli leaf curl Kanpur virus
GCA_002830005.1	1362	1	Tomato leaf curl Joydebpur betasatellite IndiaJaunpurChilli2007	NA	Viruses; Tolecusatellitidae; Betasatellite; Chili leaf curl Jaunpur betasatellite
GCA_000860945.1	7590	1	Ljunganvirus 1	87012	Viruses; Riboviria; Orthornavirae; Pisuviricota; Pisoniviricetes; Picornavirales; Picornaviridae; Parechovirus; Parechovirus B
GCA_002830685.1	12035	3	Rockport virus	NA	Viruses; Riboviria; Orthornavirae; Negarnaviricota; Polyploviricotina; Ellioviricetes; Bunyavirales; Hantaviridae; Mammantavirinae; Orthohantavirus; Rockport orthohantavirus
GCA_003089875.1	11492	4	Huaiyangshan virus	NA	Viruses; Riboviria; Orthornavirae; Negarnaviricota; Polyploviricotina; Ellioviricetes; Bunyavirales; Phenuiviridae; Phlebovirus; SFTS phlebovirus
GCA_008797275.1	7436	1	Ljungan virus 647855	NA	Viruses; Riboviria; Orthornavirae; Pisuviricota; Pisoniviricetes; Picornavirales; Picornaviridae; Parechovirus; Parechovirus B; Ljungan virus 4
GCA_008799995.1	7495	1	Ljungan virus M1146	NA	Viruses; Riboviria; Orthornavirae; Pisuviricota; Pisoniviricetes; Picornavirales; Picornaviridae; Parechovirus; Parechovirus B; Ljungan virus 3
GCA_004786915.1	2739	6	Vernonia yellow vein Fujian virus	NA	Viruses; Monodnaviria; Shotokuvirae; Cressdnaviricota; Repensiviricetes; Geplafuvirales; Geminiviridae; Begomovirus
GCA_000864305.1	7412	7	Bos taurus papillomavirus 7	NA	Viruses; dsDNA viruses, no RNA stage; Papillomaviridae; Dyoxipapillomavirus; Dyoxipapillomavirus 1
GCA_001448375.1	127011	100	Epizootic haematopoietic necrosis virus	NA	Viruses; dsDNA viruses, no RNA stage; Iridoviridae; Ranavirus
GCA_002826565.1	103531	100	Bohle iridovirus	NA	Viruses; Varidnaviria; Bamfordvirae; Nucleocytoviricota; Megaviricetes; Pimascovirales; Iridoviridae; Alphairidovirinae; Ranavirus; Frog virus 3
GCA_000891935.1	2383	3	Avian gyrovirus 2	NA	Viruses; ssDNA viruses; Circoviridae; Gyrovirus; unclassified Gyrovirus
GCA_002815715.1	12790	6	Drosophila ananassae sigmavirus	NA	Viruses; Riboviria; Orthornavirae; Negarnaviricota; Haploviricotina; Monjiviricetes; Mononegavirales; Rhabdoviridae; Sigmavirus
GCA_002815735.1	12486	6	Drosophila immigrans sigmavirus	SCM45623	Viruses; Riboviria; Orthornavirae; Negarnaviricota; Haploviricotina; Monjiviricetes; Mononegavirales; Rhabdoviridae; Sigmavirus
GCA_002823385.1	2633	5	Sida golden mosaic Lara virus	VenezuelaLaraM12009	Viruses; Monodnaviria; Shotokuvirae; Cressdnaviricota; Repensiviricetes; Geplafuvirales; Geminiviridae; Begomovirus
GCA_000891015.1	50661	80	Shigella virus Shfl1	NA	Viruses; dsDNA viruses, no RNA stage; Caudovirales; Siphoviridae; Tunavirinae; T1virus
GCA_000892655.1	165919	265	Shigella phage Shfl2	NA	Viruses; dsDNA viruses, no RNA stage; Caudovirales; Myoviridae; Tevenvirinae; T4virus; Shigella virus Shfl2
GCA_000856165.1	8126	1	Simian sapelovirus 1	2383	Viruses; ssRNA viruses; ssRNA positive-strand viruses, no DNA stage; Picornavirales; Picornaviridae; Sapelovirus; Sapelovirus B
GCA_000863425.1	7491	1	Porcine sapelovirus 1	V13	Viruses; ssRNA viruses; ssRNA positive-strand viruses, no DNA stage; Picornavirales; Picornaviridae; Sapelovirus; Sapelovirus A
GCA_003087695.1	11490	4	Severe fever with thrombocytopenia syndrome virus	HB156China2011	Viruses; Riboviria; Orthornavirae; Negarnaviricota; Polyploviricotina; Ellioviricetes; Bunyavirales; Phenuiviridae; Bandavirus; Dabie bandavirus
GCA_000892595.1	6942	2	Hardenbergia virus A	NA	Viruses; ssRNA viruses; ssRNA positive-strand viruses, no DNA stage; Tymovirales; Betaflexiviridae; Trivirinae; Divavirus
GCA_000896795.1	25139	10	Spissistilus festinus reovirus	Type	Viruses; dsRNA viruses; Reoviridae; unclassified Reoviridae
GCA_002664255.1	18058	12	Rotavirus A humanVictoriaCK000012004G1P8	NA	Viruses; Riboviria; Orthornavirae; Duplornaviricota; Resentoviricetes; Reovirales; Reoviridae; Sedoreovirinae; Rotavirus; Rotavirus A; Rotavirus G1
GCA_002664275.1	18017	12	Rotavirus A humanVictoriaCK000022004G1P8	NA	Viruses; Riboviria; Orthornavirae; Duplornaviricota; Resentoviricetes; Reovirales; Reoviridae; Sedoreovirinae; Rotavirus; Rotavirus A; Rotavirus G1
GCA_002664295.1	18065	12	Rotavirus A humanVictoriaCK000032004G1P8	NA	Viruses; Riboviria; Orthornavirae; Duplornaviricota; Resentoviricetes; Reovirales; Reoviridae; Sedoreovirinae; Rotavirus; Rotavirus A; Rotavirus G1
GCA_002664315.1	18065	12	Rotavirus A humanVictoriaCK000042004G1P8	NA	Viruses; Riboviria; Orthornavirae; Duplornaviricota; Resentoviricetes; Reovirales; Reoviridae; Sedoreovirinae; Rotavirus; Rotavirus A; Rotavirus G1
GCA_002664335.1	18036	12	Rotavirus A humanVictoriaCK000052004G1P8	NA	Viruses; Riboviria; Orthornavirae; Duplornaviricota; Resentoviricetes; Reovirales; Reoviridae; Sedoreovirinae; Rotavirus; Rotavirus A; Rotavirus G1
GCA_002664355.1	18033	12	Rotavirus A humanVictoriaCK000062004G1P8	NA	Viruses; Riboviria; Orthornavirae; Duplornaviricota; Resentoviricetes; Reovirales; Reoviridae; Sedoreovirinae; Rotavirus; Rotavirus A; Rotavirus G1
GCA_002664375.1	18050	12	Rotavirus A humanVictoriaCK000072004G1P8	NA	Viruses; Riboviria; Orthornavirae; Duplornaviricota; Resentoviricetes; Reovirales; Reoviridae; Sedoreovirinae; Rotavirus; Rotavirus A; Rotavirus G1

gca	genome.size	proteome.size	phage.name	strain	Taxonomy
GCA_002664395.1	18056	12	Rotavirus A humanVictoriaCK000082004G1P8	NA	Viruses; Riboviria; Orthornavirae; Duplornaviricota; Resentoviricetes; Reovirales; Reoviridae; Sedoreovirinae; Rotavirus; Rotavirus A; Rotavirus G1
GCA_002664435.1	18055	12	Rotavirus A humanVictoriaCK000092004G1P8	NA	Viruses; Riboviria; Orthornavirae; Duplornaviricota; Resentoviricetes; Reovirales; Reoviridae; Sedoreovirinae; Rotavirus; Rotavirus A; Rotavirus G1
GCA_002664455.1	18048	12	Rotavirus A humanVictoriaCK000112004G1P8	NA	Viruses; Riboviria; Orthornavirae; Duplornaviricota; Resentoviricetes; Reovirales; Reoviridae; Sedoreovirinae; Rotavirus; Rotavirus A; Rotavirus G1
GCA_002664475.1	18059	12	Rotavirus A humanVictoriaCK000122004G1P8	NA	Viruses; Riboviria; Orthornavirae; Duplornaviricota; Resentoviricetes; Reovirales; Reoviridae; Sedoreovirinae; Rotavirus; Rotavirus A; Rotavirus G1
GCA_002664495.1	18064	12	Rotavirus A humanVictoriaCK000142004G1P8	NA	Viruses; Riboviria; Orthornavirae; Duplornaviricota; Resentoviricetes; Reovirales; Reoviridae; Sedoreovirinae; Rotavirus; Rotavirus A; Rotavirus G1
GCA_002664515.1	18043	12	Rotavirus A humanVictoriaCK000152005G1P8	NA	Viruses; Riboviria; Orthornavirae; Duplornaviricota; Resentoviricetes; Reovirales; Reoviridae; Sedoreovirinae; Rotavirus; Rotavirus A; Rotavirus G1
GCA_002664535.1	18030	12	Rotavirus A humanVictoriaCK000162005G1P8	NA	Viruses; Riboviria; Orthornavirae; Duplornaviricota; Resentoviricetes; Reovirales; Reoviridae; Sedoreovirinae; Rotavirus; Rotavirus A; Rotavirus G1
GCA_002664555.1	18044	12	Rotavirus A humanVictoriaCK000172005G1P8	NA	Viruses; Riboviria; Orthornavirae; Duplornaviricota; Resentoviricetes; Reovirales; Reoviridae; Sedoreovirinae; Rotavirus; Rotavirus A; Rotavirus G1
GCA_002664575.1	18044	12	Rotavirus A humanVictoriaCK000182005G1P8	NA	Viruses; Riboviria; Orthornavirae; Duplornaviricota; Resentoviricetes; Reovirales; Reoviridae; Sedoreovirinae; Rotavirus; Rotavirus A; Rotavirus G1
GCA_002664595.1	18044	12	Rotavirus A humanVictoriaCK000192005G1P8	NA	Viruses; Riboviria; Orthornavirae; Duplornaviricota; Resentoviricetes; Reovirales; Reoviridae; Sedoreovirinae; Rotavirus; Rotavirus A; Rotavirus G1
GCA_002664615.1	18038	12	Rotavirus A humanVictoriaCK000202005G1P8	NA	Viruses; Riboviria; Orthornavirae; Duplornaviricota; Resentoviricetes; Reovirales; Reoviridae; Sedoreovirinae; Rotavirus; Rotavirus A; Rotavirus G1
GCA_002664655.1	18052	12	Rotavirus A humanVictoriaCK000212005G1P8	NA	Viruses; Riboviria; Orthornavirae; Duplornaviricota; Resentoviricetes; Reovirales; Reoviridae; Sedoreovirinae; Rotavirus; Rotavirus A; Rotavirus G1
GCA_002664675.1	18048	12	Rotavirus A humanVictoriaCK000222005G1P8	NA	Viruses; Riboviria; Orthornavirae; Duplornaviricota; Resentoviricetes; Reovirales; Reoviridae; Sedoreovirinae; Rotavirus; Rotavirus A; Rotavirus G1
GCA_002664695.1	18052	12	Rotavirus A humanVictoriaCK000232005G1P8	NA	Viruses; Riboviria; Orthornavirae; Duplornaviricota; Resentoviricetes; Reovirales; Reoviridae; Sedoreovirinae; Rotavirus; Rotavirus A; Rotavirus G1
GCA_002664715.1	18027	12	Rotavirus A humanVictoriaCK000242005G1P8	NA	Viruses; Riboviria; Orthornavirae; Duplornaviricota; Resentoviricetes; Reovirales; Reoviridae; Sedoreovirinae; Rotavirus; Rotavirus A; Rotavirus G1
GCA_002664735.1	18040	12	Rotavirus A humanVictoriaCK000262005G1P8	NA	Viruses; Riboviria; Orthornavirae; Duplornaviricota; Resentoviricetes; Reovirales; Reoviridae; Sedoreovirinae; Rotavirus; Rotavirus A; Rotavirus G1
GCA_002664755.1	17971	12	Rotavirus A humanVictoriaCK000272005G1P8	humanVictoriaCK000272054G1P8	Viruses; Riboviria; Orthornavirae; Duplornaviricota; Resentoviricetes; Reovirales; Reoviridae; Sedoreovirinae; Rotavirus; Rotavirus A; Rotavirus G1
GCA_002664775.1	18047	12	Rotavirus A humanVictoriaCK000282005G1P8	NA	Viruses; Riboviria; Orthornavirae; Duplornaviricota; Resentoviricetes; Reovirales; Reoviridae; Sedoreovirinae; Rotavirus; Rotavirus A; Rotavirus G1
GCA_002664795.1	18038	12	Rotavirus A humanVictoriaCK000292006G1P8	NA	Viruses; Riboviria; Orthornavirae; Duplornaviricota; Resentoviricetes; Reovirales; Reoviridae; Sedoreovirinae; Rotavirus; Rotavirus A; Rotavirus G1
GCA_002664815.1	18015	12	Rotavirus A humanVictoriaCK000302006G1P8	NA	Viruses; Riboviria; Orthornavirae; Duplornaviricota; Resentoviricetes; Reovirales; Reoviridae; Sedoreovirinae; Rotavirus; Rotavirus A; Rotavirus G1
GCA_002664835.1	18028	12	Rotavirus A humanVictoriaCK000322006G1P8	NA	Viruses; Riboviria; Orthornavirae; Duplornaviricota; Resentoviricetes; Reovirales; Reoviridae; Sedoreovirinae; Rotavirus; Rotavirus A; Rotavirus G1
GCA_002664875.1	17997	12	Rotavirus A humanVictoriaCK000332007G1P8	NA	Viruses; Riboviria; Orthornavirae; Duplornaviricota; Resentoviricetes; Reovirales; Reoviridae; Sedoreovirinae; Rotavirus; Rotavirus A; Rotavirus G1
GCA_002664895.1	18036	12	Rotavirus A humanVictoriaCK000342007G1P8	NA	Viruses; Riboviria; Orthornavirae; Duplornaviricota; Resentoviricetes; Reovirales; Reoviridae; Sedoreovirinae; Rotavirus; Rotavirus A; Rotavirus G1
GCA_002664915.1	18044	12	Rotavirus A humanVictoriaCK000352005G1P8	NA	Viruses; Riboviria; Orthornavirae; Duplornaviricota; Resentoviricetes; Reovirales; Reoviridae; Sedoreovirinae; Rotavirus; Rotavirus A; Rotavirus G1
GCA_002664935.1	18038	12	Rotavirus A humanVictoriaCK000362005G1P8	NA	Viruses; Riboviria; Orthornavirae; Duplornaviricota; Resentoviricetes; Reovirales; Reoviridae; Sedoreovirinae; Rotavirus; Rotavirus A; Rotavirus G1
GCA_002664955.1	18044	12	Rotavirus A humanVictoriaCK000372006G1P8	NA	Viruses; Riboviria; Orthornavirae; Duplornaviricota; Resentoviricetes; Reovirales; Reoviridae; Sedoreovirinae; Rotavirus; Rotavirus A; Rotavirus G1
GCA_002664975.1	18032	12	Rotavirus A humanVictoriaCK000392006G1P8	NA	Viruses; Riboviria; Orthornavirae; Duplornaviricota; Resentoviricetes; Reovirales; Reoviridae; Sedoreovirinae; Rotavirus; Rotavirus A; Rotavirus G1
GCA_002664995.1	18022	12	Rotavirus A humanVictoriaCK000402006G1P8	NA	Viruses; Riboviria; Orthornavirae; Duplornaviricota; Resentoviricetes; Reovirales; Reoviridae; Sedoreovirinae; Rotavirus; Rotavirus A; Rotavirus G1
GCA_002665015.1	17992	12	Rotavirus A humanVictoriaCK000412006G1P8	NA	Viruses; Riboviria; Orthornavirae; Duplornaviricota; Resentoviricetes; Reovirales; Reoviridae; Sedoreovirinae; Rotavirus; Rotavirus A; Rotavirus G1
GCA_002665035.1	18033	12	Rotavirus A humanVictoriaCK000432006G1P8	NA	Viruses; Riboviria; Orthornavirae; Duplornaviricota; Resentoviricetes; Reovirales; Reoviridae; Sedoreovirinae; Rotavirus; Rotavirus A; Rotavirus G1
GCA_002665055.1	17906	12	Rotavirus A humanVictoriaCK000452006G1P8	NA	Viruses; Riboviria; Orthornavirae; Duplornaviricota; Resentoviricetes; Reovirales; Reoviridae; Sedoreovirinae; Rotavirus; Rotavirus A; Rotavirus G1
GCA_002665095.1	18028	12	Rotavirus A humanVictoriaCK000462006G1P8	NA	Viruses; Riboviria; Orthornavirae; Duplornaviricota; Resentoviricetes; Reovirales; Reoviridae; Sedoreovirinae; Rotavirus; Rotavirus A; Rotavirus G1
GCA_002665115.1	18051	12	Rotavirus A humanVictoriaCK000472006G1P8	NA	Viruses; Riboviria; Orthornavirae; Duplornaviricota; Resentoviricetes; Reovirales; Reoviridae; Sedoreovirinae; Rotavirus; Rotavirus A; Rotavirus G1
GCA_002665135.1	18068	12	Rotavirus A humanVictoriaCK000482004G1P8	NA	Viruses; Riboviria; Orthornavirae; Duplornaviricota; Resentoviricetes; Reovirales; Reoviridae; Sedoreovirinae; Rotavirus; Rotavirus A; Rotavirus G1

gca	genome.size	proteome.size	phage.name	strain	Taxonomy
GCA_002665155.1	18035	12	Rotavirus A humanVictoriaCK000492004G1P8	NA	Viruses; Riboviria; Orthornavirae; Duplornaviricota; Resentoviricetes; Reovirales; Reoviridae; Sedoreovirinae; Rotavirus; Rotavirus A; Rotavirus G1
GCA_002665175.1	18004	12	Rotavirus A humanVictoriaCK000502005G1P8	NA	Viruses; Riboviria; Orthornavirae; Duplornaviricota; Resentoviricetes; Reovirales; Reoviridae; Sedoreovirinae; Rotavirus; Rotavirus A; Rotavirus G1
GCA_013086435.1	12121	4	Maldonado virus	FMD 0077	Viruses; Riboviria; Orthornavirae; Negarnaviricota; Polyploviricotina; Ellioviricetes; Bunyavirales; Phenuiviridae; Phlebovirus; Maldonado phlebovirus
GCA_002664155.1	18044	12	Rotavirus A humanVanderbiltVU0809162008G3P8	NA	Viruses; Riboviria; Orthornavirae; Duplornaviricota; Resentoviricetes; Reovirales; Reoviridae; Sedoreovirinae; Rotavirus; Rotavirus A; Rotavirus G3
GCA_002664175.1	18011	12	Rotavirus A humanVanderbiltVU0809202008G3P8	NA	Viruses; Riboviria; Orthornavirae; Duplornaviricota; Resentoviricetes; Reovirales; Reoviridae; Sedoreovirinae; Rotavirus; Rotavirus A; Rotavirus G3
GCA_002664215.1	18024	12	Rotavirus A humanVanderbiltVU0809272008G3P8	NA	Viruses; Riboviria; Orthornavirae; Duplornaviricota; Resentoviricetes; Reovirales; Reoviridae; Sedoreovirinae; Rotavirus; Rotavirus A; Rotavirus G3
GCA_002664115.1	18075	12	Rotavirus A humanVanderbiltVU0506722005G12P8	NA	Viruses; Riboviria; Orthornavirae; Duplornaviricota; Resentoviricetes; Reovirales; Reoviridae; Sedoreovirinae; Rotavirus; Rotavirus A; Rotavirus G12
GCA_002664135.1	18090	12	Rotavirus A humanVanderbiltVU0506742005G12P8	NA	Viruses; Riboviria; Orthornavirae; Duplornaviricota; Resentoviricetes; Reovirales; Reoviridae; Sedoreovirinae; Rotavirus; Rotavirus A; Rotavirus G12
GCA_002664235.1	18065	12	Rotavirus A humanVanderbiltVU0809392008G12P8	NA	Viruses; Riboviria; Orthornavirae; Duplornaviricota; Resentoviricetes; Reovirales; Reoviridae; Sedoreovirinae; Rotavirus; Rotavirus A; Rotavirus G12
GCA_000907975.1	59254	73	Salmonella virus iEPS5	NA	Viruses; Duplodnaviria; Heunggongvirae; Uroviricota; Caudoviricetes; Caudovirales; Siphoviridae; Chivirus
GCA_000869945.1	7711	1	Duck hepatitis A virus 1	R85952 ATCC VR191	Viruses; ssRNA viruses; ssRNA positive-strand viruses, no DNA stage; Picornavirales; Picornaviridae; Avihepatovirus; Duck hepatitis A virus
GCA_008798475.1	7790	1	Duck hepatitis A virus 3	NA	Viruses; Riboviria; Orthornavirae; Pisuviricota; Pisoniviricetes; Picornavirales; Picornaviridae; Avihepatovirus; Avihepatovirus A
GCA_000890995.1	12408	4	Aguacate virus	VP175A	Viruses; ssRNA viruses; ssRNA negative-strand viruses; Bunyaviridae; Phlebovirus; unclassified Phlebovirus
GCA_013086445.1	12399	4	Durania virus	Co Ar 171162	Viruses; Riboviria; Orthornavirae; Negarnaviricota; Polyploviricotina; Ellioviricetes; Bunyavirales; Phenuiviridae; Phlebovirus; Durania phlebovirus
GCA_013086455.1	12344	4	Ixcanal virus	CA Ar 170897	Viruses; Riboviria; Orthornavirae; Negarnaviricota; Polyploviricotina; Ellioviricetes; Bunyavirales; Phenuiviridae; Phlebovirus; Ixcanal phlebovirus
GCA_000914175.1	127188	168	Staphylococcus phage Sb1	NA	Viruses; dsDNA viruses, no RNA stage; Caudovirales; Myoviridae; Spounavirinae; Spo1virus; unclassified SPO1-like viruses
GCA_000864705.1	2828	6	Sweet potato leaf curl virus	NA	Viruses; ssDNA viruses; Geminiviridae; Begomovirus
GCA_000907535.1	142563	220	Rhodococcus phage E3	NA	Viruses; dsDNA viruses, no RNA stage; Caudovirales; Myoviridae; unclassified Myoviridae
GCA_002830765.1	4944	2	Trichomonas vaginalis virus 4	TVV4OC3	Viruses; Riboviria; Orthornavirae; Duplornaviricota; Chrymotiviricetes; Ghabrivirales; Totiviridae; Trichomonasvirus
GCA_000849285.1	10708	6	Soilborne cereal mosaic virus	NA	Viruses; ssRNA viruses; ssRNA positive-strand viruses, no DNA stage; Virgaviridae; Furovirus
GCA_017347715.1	35240	39	Human adenovirus 7d	HAdVBUSA104762019P7H7F7	Viruses; Varidnaviria; Bamfordvirae; Preplasmiviricota; Tectiliviricetes; Rowavirales; Adenoviridae; Mastadenovirus; Human mastadenovirus B; Human adenovirus B1; Human adenovirus 7
GCA_000895755.1	18168	21	Staphylococcus phage S241	NA	Viruses; dsDNA viruses, no RNA stage; Caudovirales; Podoviridae; Picovirinae; unclassified Picovirinae
GCA_002600655.1	18186	21	Staphylococcus phage S13	NA	Viruses; Duplodnaviria; Heunggongvirae; Uroviricota; Caudoviricetes; Caudovirales; Podoviridae; Rakietenvirinae; Rosenblumvirus; unclassified Rosenblumvirus
GCA_000911635.1	6277	3	Tomato blistering mosaic virus	NA	Viruses; ssRNA viruses; ssRNA positive-strand viruses, no DNA stage; Tymovirales; Tymoviridae; Tymovirus; unclassified Tymovirus
GCA_003088675.1	11490	4	Phlebovirus JS24	NA	Viruses; Riboviria; Orthornavirae; Negarnaviricota; Polyploviricotina; Ellioviricetes; Bunyavirales; Phenuiviridae; Phlebovirus; SFTS phlebovirus
GCA_003088695.1	11490	4	Phlebovirus JS26	NA	Viruses; Riboviria; Orthornavirae; Negarnaviricota; Polyploviricotina; Ellioviricetes; Bunyavirales; Phenuiviridae; Phlebovirus; SFTS phlebovirus
GCA_003088755.1	11490	4	Phlebovirus JS6	NA	Viruses; Riboviria; Orthornavirae; Negarnaviricota; Polyploviricotina; Ellioviricetes; Bunyavirales; Phenuiviridae; Phlebovirus; SFTS phlebovirus
GCA_013086465.1	11376	4	EgAN 182561 virus	NA	Viruses; Riboviria; Orthornavirae; Negarnaviricota; Polyploviricotina; Ellioviricetes; Bunyavirales; Phenuiviridae; Phlebovirus; Uukuniemi phlebovirus
GCA_013086475.1	11455	4	Murre virus	Murre H	Viruses; Riboviria; Orthornavirae; Negarnaviricota; Polyploviricotina; Ellioviricetes; Bunyavirales; Phenuiviridae; Uukuvirus; Murre uukuvirus
GCA_000858165.1	46900	79	Enterobacteria phage ES18	NA	Viruses; dsDNA viruses, no RNA stage; Caudovirales; Siphoviridae; unclassified Siphoviridae
GCA_000891095.1	7408	3	Banana streak CA virus	NA	Viruses; Riboviria; Pararnavirae; Artverviricota; Revtraviricetes; Ortervirales; Caulimoviridae; Badnavirus; unclassified Badnavirus
GCA_000892735.1	7769	3	Banana streak IM virus	NA	Viruses; Riboviria; Pararnavirae; Artverviricota; Revtraviricetes; Ortervirales; Caulimoviridae; Badnavirus
GCA_000891075.1	7519	3	Banana streak UA virus	NA	Viruses; Riboviria; Pararnavirae; Artverviricota; Revtraviricetes; Ortervirales; Caulimoviridae; Badnavirus
GCA_000892715.1	7458	3	Banana streak UI virus	NA	Viruses; Riboviria; Pararnavirae; Artverviricota; Revtraviricetes; Ortervirales; Caulimoviridae; Badnavirus

gca	genome.size	proteome.size	phage.name	strain	Taxonomy
GCA_000892055.1	7401	3	Banana streak UL virus	NA	Viruses; Riboviria; Pararnavirae; Artverviricota; Revtraviricetes; Ortervirales; Caulimoviridae; Badnavirus
GCA_000893615.1	7532	3	Banana streak UM virus	NA	Viruses; Riboviria; Pararnavirae; Artverviricota; Revtraviricetes; Ortervirales; Caulimoviridae; Badnavirus
GCA_002822385.1	2610	5	Euphorbia yellow mosaic virus Goias BrazilGOLuziania 86752009	NA	Viruses; Monodnaviria; Shotokuvirae; Cressdnaviricota; Repensiviricetes; Geplafuvirales; Geminiviridae; Begomovirus; Euphorbia yellow mosaic virus
GCA_000892075.1	7310	3	Cutthroat trout virus	NA	Viruses; ssRNA viruses; ssRNA positive-strand viruses, no DNA stage; Hepeviridae; Piscihepevirus; Piscihepevirus A
GCA_000861545.1	10196	2	Apple latent spherical virus	NA	Viruses; ssRNA viruses; ssRNA positive-strand viruses, no DNA stage; Picornavirales; Secoviridae; Cheravirus
GCA_003088515.1	11490	4	Phlebovirus JS200701	NA	Viruses; Riboviria; Orthornavirae; Negarnaviricota; Polyploviricotina; Ellioviricetes; Bunyavirales; Phenuiviridae; Phlebovirus; SFTS phlebovirus
GCA_000863345.1	8657	6	Aconitum latent virus	NA	Viruses; ssRNA viruses; ssRNA positive-strand viruses, no DNA stage; Tymovirales; Betaflexiviridae; Quinvirinae; Carlavirus
GCA_000901335.1	132978	145	Thysanoplusia orichalcea nucleopolyhedrovirus	NA	Viruses; dsDNA viruses, no RNA stage; Baculoviridae; Alphabaculovirus
GCA_900327495.1	220997	300	Cowpox virus	NA	Viruses; dsDNA viruses, no RNA stage; Poxviridae; Chordopoxvirinae; Orthopoxvirus
GCA_009738615.1	197417	213	Monkeypox virus	Israel_2018	Viruses; Varidnaviria; Bamfordvirae; Nucleocytoviricota; Pokkesviricetes; Chitovirales; Poxviridae; Chordopoxvirinae; Orthopoxvirus
GCA_003971425.1	173950	278	Vaccinia virus	NA	Viruses; dsDNA viruses, no RNA stage; Poxviridae; Chordopoxvirinae; Orthopoxvirus
GCA_006458465.1	191737	262	Vaccinia virus Copenhagen	NA	Viruses; Varidnaviria; Bamfordvirae; Nucleocytoviricota; Pokkesviricetes; Chitovirales; Poxviridae; Chordopoxvirinae; Orthopoxvirus; Vaccinia virus
GCA_006465985.1	189274	243	Vaccinia virus Tian Tan	NA	Viruses; Varidnaviria; Bamfordvirae; Nucleocytoviricota; Pokkesviricetes; Chitovirales; Poxviridae; Chordopoxvirinae; Orthopoxvirus; Vaccinia virus
GCA_000860085.1	194711	218	Vaccinia virus WR	WR Western Reserve	Viruses; Varidnaviria; Bamfordvirae; Nucleocytoviricota; Pokkesviricetes; Chitovirales; Poxviridae; Chordopoxvirinae; Orthopoxvirus; Vaccinia virus
GCA_000859885.1	185578	197	Variola virus	India1967 ssp major	Viruses; dsDNA viruses, no RNA stage; Poxviridae; Chordopoxvirinae; Orthopoxvirus
GCA_001029045.1	214699	207	Raccoonpox virus	Herman	Viruses; dsDNA viruses, no RNA stage; Poxviridae; Chordopoxvirinae; Orthopoxvirus
GCA_016811275.1	139807	132	Orf virus	NA	Viruses; dsDNA viruses, no RNA stage; Poxviridae; Chordopoxvirinae; Parapoxvirus
GCA_016906595.1	293578	282	Fowlpox virus	FWPVS	Viruses; dsDNA viruses, no RNA stage; Poxviridae; Chordopoxvirinae; Avipoxvirus
GCA_006458565.1	266145	241	Fowlpox virus isolate HP438Munich	NA	Viruses; Varidnaviria; Bamfordvirae; Nucleocytoviricota; Pokkesviricetes; Chitovirales; Poxviridae; Chordopoxvirinae; Avipoxvirus; Fowlpox virus
GCA_000922075.1	282356	224	Pigeonpox virus	NA	Viruses; dsDNA viruses, no RNA stage; Poxviridae; Chordopoxvirinae; Avipoxvirus
GCA_014918095.1	150211	152	Sheeppox virus	NA	Viruses; dsDNA viruses, no RNA stage; Poxviridae; Chordopoxvirinae; Capripoxvirus
GCA_000892775.1	178563	297	Synechococcus phage SCRM01	NA	Viruses; dsDNA viruses, no RNA stage; Caudovirales; Myoviridae; unclassified Myoviridae
GCA_000847965.1	159857	165	Rabbit fibroma virus	Kasza	Viruses; dsDNA viruses, no RNA stage; Poxviridae; Chordopoxvirinae; Leporipoxvirus
GCA_000892015.1	16791	5	Groundnut ringspot and Tomato chlorotic spot virus reassortant	NA	Viruses; ssRNA viruses; ssRNA negative-strand viruses; Bunyaviridae; Tospovirus; unclassified Tospovirus; Tospovirus reassortants
GCA_000918415.1	6317	3	Bovine astrovirus B170HK	NA	Viruses; ssRNA viruses; ssRNA positive-strand viruses, no DNA stage; Astroviridae; Mamastrovirus; unclassified Mamastrovirus
GCA_000916515.1	6300	3	Bovine astrovirus B18HK	NA	Viruses; ssRNA viruses; ssRNA positive-strand viruses, no DNA stage; Astroviridae; Mamastrovirus; unclassified Mamastrovirus
GCA_000914835.1	6301	3	Bovine astrovirus B762HK	NA	Viruses; ssRNA viruses; ssRNA positive-strand viruses, no DNA stage; Astroviridae; Mamastrovirus; unclassified Mamastrovirus
GCA_000917375.1	6253	3	Bovine astrovirus B76HK	NA	Viruses; ssRNA viruses; ssRNA positive-strand viruses, no DNA stage; Astroviridae; Mamastrovirus; unclassified Mamastrovirus
GCA_000891155.1	5819	9	Stenotrophomonas phage phiSHP2	NA	Viruses; ssDNA viruses; Inoviridae; unclassified Inoviridae
GCA_005411835.1	164579	175	Myxoma virus	NA	Viruses; dsDNA viruses, no RNA stage; Poxviridae; Chordopoxvirinae; Leporipoxvirus
GCA_002822685.1	2719	6	Jatropha curcas mosaic virusJalgaon	NA	Viruses; Monodnaviria; Shotokuvirae; Cressdnaviricota; Repensiviricetes; Geplafuvirales; Geminiviridae; Begomovirus; Indian cassava mosaic virus; Jatropha curcas mosaic virus
GCA_000926315.1	12233	5	Farmington virus	CT 114	Viruses; ssRNA viruses; ssRNA negative-strand viruses; Mononegavirales; Rhabdoviridae; unclassified Rhabdoviridae
GCA_017903395.1	146432	151	Swinepox virus	NA	Viruses; dsDNA viruses, no RNA stage; Poxviridae; Chordopoxvirinae; Suipoxvirus
GCA_018594605.1	12115	3	Oyo virus	NA	Viruses; Riboviria; Orthornavirae; Negarnaviricota; Polyploviricotina; Ellioviricetes; Bunyavirales; Peribunyaviridae; Orthobunyavirus; Oyo orthobunyavirus

gca	genome.size	proteome.size	phage.name	strain	Taxonomy
GCA_000893635.1	6423	3	Switchgrass mosaic virus	NA	Viruses; ssRNA viruses; ssRNA positive-strand viruses, no DNA stage; Tymovirales; Tymoviridae; Marafivirus; unclassified Marafivirus
GCA_000891115.1	6175	3	Asclepias asymptomatic virus	NA	Viruses; ssRNA viruses; ssRNA positive-strand viruses, no DNA stage; Tymovirales; Tymoviridae; Tymovirus; unclassified Tymovirus
GCA_016865615.1	188334	182	Molluscum contagiosum virus	NA	Viruses; Varidnaviria; Bamfordvirae; Nucleocytoviricota; Pokkesviricetes; Chitovirales; Poxviridae; Chordopoxvirinae; Molluscipoxvirus
GCA_000898595.2	10303	2	Macrobrachium rosenbergii Taihu virus	cntaihu100401	Viruses; ssRNA viruses; ssRNA positive-strand viruses, no DNA stage; Picornavirales; Dicistroviridae; unclassified Dicistroviridae
GCA_014183265.1	185655	167	Molluscum contagiosum virus subtype 1	NA	Viruses; dsDNA viruses, no RNA stage; Poxviridae; Chordopoxvirinae; Molluscipoxvirus; Molluscum contagiosum virus
GCA_006450575.1	192183	170	Molluscum contagiosum virus subtype 2	NA	Viruses; Varidnaviria; Bamfordvirae; Nucleocytoviricota; Pokkesviricetes; Chitovirales; Poxviridae; Chordopoxvirinae; Molluscipoxvirus; Molluscum contagiosum virus
GCA_002597505.1	138339	215	Staphylococcus phage ISP	NA	Viruses; Duplodnaviria; Heunggongvirae; Uroviricota; Caudoviricetes; Caudovirales; Herelleviridae; Twortvirinae; Kayvirus; Staphylococcus virus G1
GCA_008792205.1	7391	1	Porcine enterovirus 3H	swineK232008HUN	Viruses; Riboviria; Orthornavirae; Pisuviricota; Pisoniviricetes; Picornavirales; Picornaviridae; Enterovirus; Enterovirus G
GCA_000427115.1	307691	334	Choristoneura biennis entomopoxvirus	NA	Viruses; dsDNA viruses, no RNA stage; Poxviridae; Entomopoxvirinae; Betaentomopoxvirus
GCA_000859985.2	152222	77	Human alphaherpesvirus 1	17	Viruses; dsDNA viruses, no RNA stage; Herpesvirales; Herpesviridae; Alphaherpesvirinae; Simplexvirus
GCA_000902915.1	243050	262	Erwinia phage phiEaH2	NA	Viruses; dsDNA viruses, no RNA stage; Caudovirales; Siphoviridae; unclassified Siphoviridae
GCA_000893655.1	2689	4	Eragrostis minor streak virus	NA	Viruses; ssDNA viruses; Geminiviridae; Mastrevirus
GCA_003052145.1	154746	77	Human alphaherpesvirus 2	HG52	Viruses; dsDNA viruses, no RNA stage; Herpesvirales; Herpesviridae; Alphaherpesvirinae; Simplexvirus
GCA_000846965.1	150715	75	Cercopithecine alphaherpesvirus 2	NA	Viruses; dsDNA viruses, no RNA stage; Herpesvirales; Herpesviridae; Alphaherpesvirinae; Simplexvirus
GCA_000891135.1	41090	55	Clostridium phage phiCD382	NA	Viruses; dsDNA viruses, no RNA stage; Caudovirales; Siphoviridae; unclassified Siphoviridae
GCA_000892095.1	6791	4	Passion fruit mosaic virus	NA	Viruses; ssRNA viruses; ssRNA positive-strand viruses, no DNA stage; Virgaviridae; Tobamovirus
GCA_001441135.1	38049	53	Aeropyrum pernix spindleshaped virus 1	NA	Viruses; unclassified archaeal viruses
GCA_001440875.1	13769	21	Aeropyrum pernix ovoid virus 1	NA	Viruses; dsDNA viruses, no RNA stage; Guttaviridae; Betaguttavirus
GCA_002821805.1	2626	6	Chenopodium leaf curl virus	NA	Viruses; Monodnaviria; Shotokuvirae; Cressdnaviricota; Repensiviricetes; Geplafuvirales; Geminiviridae; Begomovirus
GCA_000844145.1	156789	75	Macacine alphaherpesvirus 1	E2490	Viruses; dsDNA viruses, no RNA stage; Herpesvirales; Herpesviridae; Alphaherpesvirinae; Simplexvirus
GCA_000844025.1	150224	80	Equid alphaherpesvirus 1	Ab4	Viruses; dsDNA viruses, no RNA stage; Herpesvirales; Herpesviridae; Alphaherpesvirinae; Varicellovirus
GCA_002600695.1	53171	96	Mycobacterium virus BBPiebs31	NA	Viruses; Duplodnaviria; Heunggongvirae; Uroviricota; Caudoviricetes; Caudovirales; Siphoviridae; Fromanvirus
GCA_002600715.1	50364	89	Mycobacterium virus Heldan	NA	Viruses; Duplodnaviria; Heunggongvirae; Uroviricota; Caudoviricetes; Caudovirales; Siphoviridae; Fromanvirus
GCA_002600735.1	109270	230	Mycobacterium virus Optimus	NA	Viruses; Duplodnaviria; Heunggongvirae; Uroviricota; Caudoviricetes; Caudovirales; Siphoviridae; Omegavirus
GCA_002601085.1	68839	100	Mycobacterium phage Thora	NA	Viruses; Duplodnaviria; Heunggongvirae; Uroviricota; Caudoviricetes; Caudovirales; Siphoviridae; Bclasvirinae; Pegunavirus; Mycobacterium virus Soto
GCA_002624645.1	53278	85	Mycobacterium phage Timshel	NA	Viruses; Duplodnaviria; Heunggongvirae; Uroviricota; Caudoviricetes; Caudovirales; Siphoviridae; Fromanvirus; Mycobacterium virus Timshel
GCA_000846345.1	145597	79	Equid alphaherpesvirus 4	NS80567	Viruses; dsDNA viruses, no RNA stage; Herpesvirales; Herpesviridae; Alphaherpesvirinae; Varicellovirus
GCA_000885455.1	135797	77	Felid alphaherpesvirus 1	C27	Viruses; dsDNA viruses, no RNA stage; Herpesvirales; Herpesviridae; Alphaherpesvirinae; Varicellovirus
GCA_009800365.1	156878	261	Rhizobium phage RL2RES	NA	Viruses; Duplodnaviria; Heunggongvirae; Uroviricota; Caudoviricetes; Caudovirales; Ackermannviridae; unclassified Ackermannviridae
GCA_000858285.1	124884	73	Human herpesvirus 3 strain Dumas	NA	Viruses; Duplodnaviria; Heunggongvirae; Peploviricota; Herviviricetes; Herpesvirales; Herpesviridae; Alphaherpesvirinae; Varicellovirus; Human alphaherpesvirus 3
GCA_008791775.1	7168	1	Human rhinovirus A103	WA327E09	Viruses; Riboviria; Orthornavirae; Pisuviricota; Pisoniviricetes; Picornavirales; Picornaviridae; Enterovirus; Rhinovirus A
GCA_000897275.1	16510	5	Bean necrotic mosaic virus	NA	Viruses; ssRNA viruses; ssRNA negative-strand viruses; Bunyaviridae; Tospovirus; unclassified Tospovirus
GCA_002600755.1	46450	74	Flavobacterium phage FCV1	NA	Viruses; Duplodnaviria; Heunggongvirae; Uroviricota; Caudoviricetes; Caudovirales; Myoviridae; Ficleduovirus; Flavobacterium virus FCV1
GCA_000895535.1	7140	3	Fig badnavirus 1	NA	Viruses; Riboviria; Pararnavirae; Artverviricota; Revtraviricetes; Ortervirales; Caulimoviridae; Badnavirus

gca	genome.size	proteome.size	phage.name	strain	Taxonomy
GCA_002600775.1	111688	242	Mycobacterium virus Baka	NA	Viruses; Duplodnaviria; Heunggongvirae; Uroviricota; Caudoviricetes; Caudovirales; Siphoviridae; Omegavirus
GCA_002600795.2	74997	147	Mycobacterium virus Bask21	NA	Viruses; Duplodnaviria; Heunggongvirae; Uroviricota; Caudoviricetes; Caudovirales; Siphoviridae; Kostyavirus
GCA_002600815.1	58899	105	Mycobacterium virus Dlane	DLane	Viruses; Duplodnaviria; Heunggongvirae; Uroviricota; Caudoviricetes; Caudovirales; Siphoviridae; Cheoctovirus
GCA_002600835.1	51547	97	Mycobacterium phage DaVinci	NA	Viruses; Duplodnaviria; Heunggongvirae; Uroviricota; Caudoviricetes; Caudovirales; Siphoviridae; Fromanvirus; unclassified Fromanvirus
GCA_002600855.1	51889	104	Mycobacterium virus Hammer	NA	Viruses; Duplodnaviria; Heunggongvirae; Uroviricota; Caudoviricetes; Caudovirales; Siphoviridae; Fromanvirus
GCA_002754955.1	68193	99	Mycobacterium phage Harvey	NA	Viruses; Duplodnaviria; Heunggongvirae; Uroviricota; Caudoviricetes; Caudovirales; Siphoviridae; Bclasvirinae; Pegunavirus; Mycobacterium virus Soto
GCA_002601125.1	76049	143	Mycobacterium phage Henry	NA	Viruses; Duplodnaviria; Heunggongvirae; Uroviricota; Caudoviricetes; Caudovirales; Siphoviridae; Kostyavirus; Mycobacterium virus Toto
GCA_002754975.1	68675	101	Mycobacterium phage Hertubise	NA	Viruses; Duplodnaviria; Heunggongvirae; Uroviricota; Caudoviricetes; Caudovirales; Siphoviridae; Bclasvirinae; Pegunavirus; Mycobacterium virus Pg1
GCA_002600875.1	55600	103	Mycobacterium virus Ibhubesi	NA	Viruses; Duplodnaviria; Heunggongvirae; Uroviricota; Caudoviricetes; Caudovirales; Siphoviridae; Cheoctovirus
GCA_002600905.1	52169	97	Mycobacterium virus JC27	NA	Viruses; Duplodnaviria; Heunggongvirae; Uroviricota; Caudoviricetes; Caudovirales; Siphoviridae; Fromanvirus
GCA_002600935.1	51381	92	Mycobacterium virus Kssjeb	KSSJEB	Viruses; Duplodnaviria; Heunggongvirae; Uroviricota; Caudoviricetes; Caudovirales; Siphoviridae; Fromanvirus
GCA_002600965.1	50486	89	Mycobacterium virus Lesedi	NA	Viruses; Duplodnaviria; Heunggongvirae; Uroviricota; Caudoviricetes; Caudovirales; Siphoviridae; Fromanvirus
GCA_002600985.1	109086	229	Mycobacterium virus Littlee	LittleE	Viruses; Duplodnaviria; Heunggongvirae; Uroviricota; Caudoviricetes; Caudovirales; Siphoviridae; Omegavirus
GCA_002601005.1	57278	108	Mycobacterium virus Mozy	NA	Viruses; Duplodnaviria; Heunggongvirae; Uroviricota; Caudoviricetes; Caudovirales; Siphoviridae; Cheoctovirus
GCA_002601025.1	51426	90	Mycobacterium virus Museum	NA	Viruses; Duplodnaviria; Heunggongvirae; Uroviricota; Caudoviricetes; Caudovirales; Siphoviridae; Fromanvirus
GCA_002601045.1	61147	100	Mycobacterium virus Pixie	NA	Viruses; Duplodnaviria; Heunggongvirae; Uroviricota; Caudoviricetes; Caudovirales; Siphoviridae; Timquatrovirus
GCA_002624045.1	83724	153	Mycobacterium phage Rey	NA	Viruses; Duplodnaviria; Heunggongvirae; Uroviricota; Caudoviricetes; Caudovirales; Siphoviridae; Mclasvirinae; Reyvirus; Mycobacterium virus Rey
GCA_002624745.1	75973	145	Mycobacterium phage SirDuracell	NA	Viruses; Duplodnaviria; Heunggongvirae; Uroviricota; Caudoviricetes; Caudovirales; Siphoviridae; Kostyavirus; Mycobacterium virus Sirduracell
GCA_002601065.1	64791	90	Mycobacterium phage SirHarley	NA	Viruses; Duplodnaviria; Heunggongvirae; Uroviricota; Caudoviricetes; Caudovirales; Siphoviridae; Dclasvirinae; Plotvirus; Mycobacterium virus Plot
GCA_002624705.1	52298	91	Mycobacterium phage Switzer	NA	Viruses; Duplodnaviria; Heunggongvirae; Uroviricota; Caudoviricetes; Caudovirales; Siphoviridae; Fromanvirus; Mycobacterium virus Switzer
GCA_002754995.1	68719	103	Mycobacterium phage Yoshand	NA	Viruses; Duplodnaviria; Heunggongvirae; Uroviricota; Caudoviricetes; Caudovirales; Siphoviridae; Bclasvirinae; Pegunavirus; Mycobacterium virus Soto
GCA_002597845.1	51369	86	Mycobacterium phage Shaka	NA	Viruses; Duplodnaviria; Heunggongvirae; Uroviricota; Caudoviricetes; Caudovirales; Siphoviridae; Fromanvirus; Mycobacterium virus Peaches
GCA_000892115.1	75960	129	Mycobacterium virus Faith1	NA	Viruses; Duplodnaviria; Heunggongvirae; Uroviricota; Caudoviricetes; Caudovirales; Siphoviridae; Bronvirus
GCA_002991115.1	68850	100	Mycobacterium phage ABU	NA	Viruses; Duplodnaviria; Heunggongvirae; Uroviricota; Caudoviricetes; Caudovirales; Siphoviridae; Bclasvirinae; Pegunavirus; Mycobacterium virus Pg1
GCA_002632565.1	59646	94	Mycobacterium phage Adephagia	NA	Viruses; Duplodnaviria; Heunggongvirae; Uroviricota; Caudoviricetes; Caudovirales; Siphoviridae; Timquatrovirus; unclassified Timquatrovirus
GCA_001505915.1	153401	222	Mycobacterium phage Alice	NA	Viruses; dsDNA viruses, no RNA stage; Caudovirales; Myoviridae; unclassified Myoviridae
GCA_002632605.1	51308	84	Mycobacterium virus Backyardigan	NA	Viruses; Duplodnaviria; Heunggongvirae; Uroviricota; Caudoviricetes; Caudovirales; Siphoviridae; Fromanvirus
GCA_002633495.1	70428	100	Mycobacterium phage ChrisnMich	NA	Viruses; Duplodnaviria; Heunggongvirae; Uroviricota; Caudoviricetes; Caudovirales; Siphoviridae; Bclasvirinae; Coopervirus; Mycobacterium virus Chrisnmich
GCA_002632665.1	68245	102	Mycobacterium phage Daisy	NA	Viruses; Duplodnaviria; Heunggongvirae; Uroviricota; Caudoviricetes; Caudovirales; Siphoviridae; Bclasvirinae; Pipefishvirus; unclassified Pipefishvirus
GCA_002598445.1	156281	230	Mycobacterium phage Drazdys	NA	Viruses; Duplodnaviria; Heunggongvirae; Uroviricota; Caudoviricetes; Caudovirales; Myoviridae; Bixzunavirus; Mycobacterium virus Bxz1
GCA_002632705.1	51578	82	Mycobacterium virus George	NA	Viruses; Duplodnaviria; Heunggongvirae; Uroviricota; Caudoviricetes; Caudovirales; Siphoviridae; Fromanvirus
GCA_002598425.1	155167	231	Mycobacterium phage Ghost	NA	Viruses; Duplodnaviria; Heunggongvirae; Uroviricota; Caudoviricetes; Caudovirales; Myoviridae; Bixzunavirus; Mycobacterium virus Bxz1
GCA_002632735.1	52213	96	Mycobacterium virus Gladiator	NA	Viruses; Duplodnaviria; Heunggongvirae; Uroviricota; Caudoviricetes; Caudovirales; Siphoviridae; Fromanvirus
GCA_002709675.1	50877	85	Mycobacterium virus JHC117	NA	Viruses; Duplodnaviria; Heunggongvirae; Uroviricota; Caudoviricetes; Caudovirales; Siphoviridae; Fromanvirus

gca	genome.size	proteome.size	phage.name	strain	Taxonomy
GCA_002632795.1	74914	126	Mycobacterium virus Joedirt	NA	Viruses; Duplodnaviria; Heunggongvirae; Uroviricota; Caudoviricetes; Caudovirales; Siphoviridae; Bronvirus
GCA_002600325.1	68138	100	Mycobacterium phage KLucky39	NA	Viruses; Duplodnaviria; Heunggongvirae; Uroviricota; Caudoviricetes; Caudovirales; Siphoviridae; Bclasvirinae; Pegunavirus; Mycobacterium virus Suffolk
GCA_002633455.1	65100	107	Mycobacterium phage Marvin	NA	Viruses; Duplodnaviria; Heunggongvirae; Uroviricota; Caudoviricetes; Caudovirales; Siphoviridae; Marvinvirus; Mycobacterium virus Marvin
GCA_002866965.1	50864	85	Mycobacterium virus Microwolf	NA	Viruses; Duplodnaviria; Heunggongvirae; Uroviricota; Caudoviricetes; Caudovirales; Siphoviridae; Fromanvirus
GCA_003004885.1	68735	102	Mycobacterium phage Oosterbaan	NA	Viruses; Duplodnaviria; Heunggongvirae; Uroviricota; Caudoviricetes; Caudovirales; Siphoviridae; Bclasvirinae; Pegunavirus; Mycobacterium virus Pg1
GCA_002632835.1	51339	93	Mycobacterium virus Packman	NA	Viruses; Duplodnaviria; Heunggongvirae; Uroviricota; Caudoviricetes; Caudovirales; Siphoviridae; Fromanvirus
GCA_001505255.1	68293	99	Mycobacterium phage Phipps	NA	Viruses; dsDNA viruses, no RNA stage; Caudovirales; Siphoviridae; unclassified Siphoviridae; PG1-like phages
GCA_002633545.1	47780	78	Mycobacterium phage Rockstar	NA	Viruses; Duplodnaviria; Heunggongvirae; Uroviricota; Caudoviricetes; Caudovirales; Siphoviridae; Fromanvirus; Mycobacterium virus Rockstar
GCA_002632885.1	56719	107	Mycobacterium virus Rockyhorror	NA	Viruses; Duplodnaviria; Heunggongvirae; Uroviricota; Caudoviricetes; Caudovirales; Siphoviridae; Cheoctovirus
GCA_002633475.1	71547	98	Mycobacterium phage Send513	NA	Viruses; Duplodnaviria; Heunggongvirae; Uroviricota; Caudoviricetes; Caudovirales; Siphoviridae; Papyrusvirus; Mycobacterium virus Send513
GCA_002632935.1	73784	122	Mycobacterium phage UPIE	NA	Viruses; Duplodnaviria; Heunggongvirae; Uroviricota; Caudoviricetes; Caudovirales; Siphoviridae; Bronvirus; unclassified Bronvirus
GCA_002599925.1	50963	85	Mycobacterium phage Vix	NA	Viruses; Duplodnaviria; Heunggongvirae; Uroviricota; Caudoviricetes; Caudovirales; Siphoviridae; Fromanvirus; unclassified Fromanvirus
GCA_001503635.1	68346	99	Mycobacterium phage Vortex	NA	Viruses; dsDNA viruses, no RNA stage; Caudovirales; Siphoviridae; unclassified Siphoviridae; PG1-like phages
GCA_002632965.1	58714	116	Mycobacterium virus Yoshi	NA	Viruses; Duplodnaviria; Heunggongvirae; Uroviricota; Caudoviricetes; Caudovirales; Siphoviridae; Cheoctovirus
GCA_002633525.1	71092	95	Mycobacterium phage Zemanar	NA	Viruses; Duplodnaviria; Heunggongvirae; Uroviricota; Caudoviricetes; Caudovirales; Siphoviridae; Bclasvirinae; Coopervirus; Mycobacterium virus Zemanar
GCA_002632985.1	60835	98	Mycobacterium virus Anaya	NA	Viruses; Duplodnaviria; Heunggongvirae; Uroviricota; Caudoviricetes; Caudovirales; Siphoviridae; Timquatrovirus
GCA_000923415.1	11367	5	Soybean cyst nematode socyvirus	NA	Viruses; ssRNA viruses; ssRNA negative-strand viruses; Mononegavirales; Nyamiviridae; Socyvirus
GCA_000853005.1	9010	2	Triatoma virus	NA	Viruses; ssRNA viruses; ssRNA positive-strand viruses, no DNA stage; Picornavirales; Dicistroviridae; Triatovirus
GCA_000843825.1	143461	77	Suid alphaherpesvirus 1	NA	Viruses; dsDNA viruses, no RNA stage; Herpesvirales; Herpesviridae; Alphaherpesvirinae; Varicellovirus
GCA_018577785.1	2336	2	Mosquito VEM Anellovirus SDBVL A	NA	Viruses; Anelloviridae; unclassified Anelloviridae
GCA_018577795.1	2547	3	Mosquito VEM Anellovirus SDRB A	NA	Viruses; Anelloviridae; Wawtorquevirus; Torque teno arthrovec virus 2
GCA_002825705.1	2238	4	Mosquito VEM virus SDBVL G	NA	Viruses; Monodnaviria; Shotokuvirae; Cressdnaviricota; Repensiviricetes; Geplafuvirales; Genomoviridae; Gemycircularvirus; Mosquito associated gemycircularvirus 1
GCA_003726155.1	2212	3	Mosquito VEM virus SDRBAJ	NA	Viruses; unclassified viruses
GCA_000890195.1	156742	70	Saimiriine alphaherpesvirus 1	MV 54	Viruses; dsDNA viruses, no RNA stage; Herpesvirales; Herpesviridae; Alphaherpesvirinae; Simplexvirus
GCA_001505495.1	69118	102	Mycobacterium phage OSmaximus	NA	Viruses; dsDNA viruses, no RNA stage; Caudovirales; Siphoviridae; unclassified Siphoviridae; PG1-like phages
GCA_002601105.1	75706	145	Mycobacterium phage Rakim	NA	Viruses; Duplodnaviria; Heunggongvirae; Uroviricota; Caudoviricetes; Caudovirales; Siphoviridae; Kostyavirus; unclassified Kostyavirus
GCA_002755015.1	68804	101	Mycobacterium phage Serendipity	NA	Viruses; Duplodnaviria; Heunggongvirae; Uroviricota; Caudoviricetes; Caudovirales; Siphoviridae; Bclasvirinae; Pegunavirus; Mycobacterium virus Pg1
GCA_001504435.1	75933	140	Mycobacterium phage Toto	NA	Viruses; dsDNA viruses, no RNA stage; Caudovirales; Siphoviridae; Cjw1virus
GCA_000892195.1	23931	42	Bdellovibrio phage phi1402	NA	Viruses; dsDNA viruses, no RNA stage; Caudovirales; Myoviridae; unclassified Myoviridae
GCA_000845245.1	235646	169	Human betaherpesvirus 5	Merlin	Viruses; dsDNA viruses, no RNA stage; Herpesvirales; Herpesviridae; Betaherpesvirinae; Cytomegalovirus
GCA_000849265.1	11550	6	Snakehead virus	NA	Viruses; ssRNA viruses; ssRNA negative-strand viruses; Mononegavirales; Rhabdoviridae; Novirhabdovirus
GCA_018577775.1	1375	1	Tomato leaf curl betasatellitePanipat 7 IndiaPanipatPapaya2008	NA	Viruses; Tolecusatellitidae; Betasatellite; Tomato leaf curl betasatellite
GCA_002830165.1	1333	1	Papaya leaf curl betasatellitePanipat 6 IndiaPanipatPapaya2008	NA	Viruses; Tolecusatellitidae; Betasatellite; Papaya leaf curl India betasatellite
GCA_008792765.1	230408	158	Murid betaherpesvirus 1	Smith	Viruses; dsDNA viruses, no RNA stage; Herpesvirales; Herpesviridae; Betaherpesvirinae; Muromegalovirus

gca	genome.size	proteome.size	phage.name	strain	Taxonomy
GCA_002601145.1	50988	92	Mycobacterium virus Mrgordo	NA	Viruses; Duplodnaviria; Heunggongvirae; Uroviricota; Caudoviricetes; Caudovirales; Siphoviridae; Fromanvirus
GCA_002601165.1	57701	106	Mycobacterium virus Mutaforma13	NA	Viruses; Duplodnaviria; Heunggongvirae; Uroviricota; Caudoviricetes; Caudovirales; Siphoviridae; Cheoctovirus
GCA_002624785.2	59315	107	Mycobacterium phage Shauna1	NA	Viruses; Duplodnaviria; Heunggongvirae; Uroviricota; Caudoviricetes; Caudovirales; Siphoviridae; Cheoctovirus; Mycobacterium virus Shauna1
GCA_002624765.1	59794	107	Mycobacterium phage ShiLan	NA	Viruses; Duplodnaviria; Heunggongvirae; Uroviricota; Caudoviricetes; Caudovirales; Siphoviridae; Cheoctovirus; Mycobacterium virus Shilan
GCA_000926195.1	7589	7	Apodemus sylvaticus papillomavirus 1	NA	Viruses; dsDNA viruses, no RNA stage; Papillomaviridae; Pipapillomavirus; unclassified Pipapillomavirus
GCA_003179075.1	7724	7	Rattus norvegicus papillomavirus 2	NA	Viruses; Monodnaviria; Shotokuvirae; Cossaviricota; Papovaviricetes; Zurhausenvirales; Papillomaviridae; Firstpapillomavirinae; Iotapapillomavirus; Iotapapillomavirus 1
GCA_000929435.1	182380	79	Equid gammaherpesvirus 5	214167	Viruses; dsDNA viruses, no RNA stage; Herpesvirales; Herpesviridae; Gammaherpesvirinae; Percavirus
GCA_000848125.1	153080	86	Human betaherpesvirus 7	RK	Viruses; dsDNA viruses, no RNA stage; Herpesvirales; Herpesviridae; Betaherpesvirinae; Roseolovirus
GCA_000859005.1	7564	4	Grapevine fleck virus	NA	Viruses; ssRNA viruses; ssRNA positive-strand viruses, no DNA stage; Tymovirales; Tymoviridae; Maculavirus
GCA_001698255.1	6863	2	Grapevine Red Globe virus	NA	Viruses; ssRNA viruses; ssRNA positive-strand viruses, no DNA stage; Tymovirales; Tymoviridae; Maculavirus; unclassified Maculavirus
GCA_000893695.1	3480	1	Tuber aestivum mitovirus	NA	Viruses; ssRNA viruses; ssRNA positive-strand viruses, no DNA stage; Narnaviridae; Mitovirus; unclassified Mitovirus
GCA_002402265.1	171823	95	Human gammaherpesvirus 4	B958	Viruses; Duplodnaviria; Heunggongvirae; Peploviricota; Herviviricetes; Herpesvirales; Herpesviridae; Gammaherpesvirinae; Lymphocryptovirus
GCA_000850665.1	4322	3	Pariacoto virus	NA	Viruses; ssRNA viruses; ssRNA positive-strand viruses, no DNA stage; Nodaviridae; Alphanodavirus
GCA_000864565.1	8197	2	PreXMRV1	NA	Viruses; Retro-transcribing viruses; Retroviridae; Orthoretrovirinae; Gammaretrovirus; unclassified Gammaretrovirus; Murine leukemia-related retroviruses; XMRV-related viruses
GCA_000847205.1	42575	58	Enterobacteria phage phiP27	NA	Viruses; dsDNA viruses, no RNA stage; Caudovirales; Myoviridae; unclassified Myoviridae
GCA_004788495.1	2782	6	Datura leaf curl virus	NA	Viruses; Monodnaviria; Shotokuvirae; Cressdnaviricota; Repensiviricetes; Geplafuvirales; Geminiviridae; Begomovirus
GCA_000845465.1	112930	76	Saimiriine gammaherpesvirus 2	NA	Viruses; dsDNA viruses, no RNA stage; Herpesvirales; Herpesviridae; Gammaherpesvirinae; Rhadinovirus
GCA_000839745.1	108873	79	Bovine gammaherpesvirus 4	NA	Viruses; dsDNA viruses, no RNA stage; Herpesvirales; Herpesviridae; Gammaherpesvirinae; Rhadinovirus
GCA_008787145.1	152975	79	Gallid alphaherpesvirus 1	SA2	Viruses; dsDNA viruses, no RNA stage; Herpesvirales; Herpesviridae; Alphaherpesvirinae; Iltovirus
GCA_000852745.1	17266	10	Potato yellow vein virus	NA	Viruses; ssRNA viruses; ssRNA positive-strand viruses, no DNA stage; Closteroviridae; Crinivirus
GCA_000846265.1	177874	105	Gallid alphaherpesvirus 2	NA	Viruses; dsDNA viruses, no RNA stage; Herpesvirales; Herpesviridae; Alphaherpesvirinae; Mardivirus
GCA_008800715.1	7397	1	Echovirus 1 strain Farouk ATCC VR1038	Farouk ATCC VR1038	Viruses; Riboviria; Orthornavirae; Pisuviricota; Pisoniviricetes; Picornavirales; Picornaviridae; Enterovirus; Enterovirus B; Echovirus E1
GCA_008766535.1	7420	1	Echovirus 9 strain Hill	prototype Hill	Viruses; Riboviria; Orthornavirae; Pisuviricota; Pisoniviricetes; Picornavirales; Picornaviridae; Enterovirus; Enterovirus B; Echovirus E9
GCA_000844865.1	221454	223	Rhesus cytomegalovirus strain 681	NA	Viruses; Duplodnaviria; Heunggongvirae; Peploviricota; Herviviricetes; Herpesvirales; Herpesviridae; Betaherpesvirinae; Cytomegalovirus; Macacine betaherpesvirus 3
GCA_000849685.1	195859	157	Tupaiid betaherpesvirus 1	2	Viruses; dsDNA viruses, no RNA stage; Herpesvirales; Herpesviridae; Betaherpesvirinae; unassigned Betaherpesvirinae
GCA_000866865.1	135135	73	Ovine gammaherpesvirus 2	BJ1035	Viruses; dsDNA viruses, no RNA stage; Herpesvirales; Herpesviridae; Gammaherpesvirinae; Macavirus
GCA_000839325.1	134226	90	Ictalurid herpesvirus 1	Auburn 1	Viruses; dsDNA viruses, no RNA stage; Herpesvirales; Alloherpesviridae; Ictalurivirus
GCA_000837845.1	3311	4	Ground squirrel hepatitis virus	NA	Viruses; Riboviria; Pararnavirae; Artverviricota; Revtraviricetes; Blubervirales; Hepadnaviridae; Orthohepadnavirus
GCA_003114235.1	3215	7	Hepatitis B virus	genotype F	Viruses; Riboviria; Pararnavirae; Artverviricota; Revtraviricetes; Blubervirales; Hepadnaviridae; Orthohepadnavirus
GCA_002601185.1	51702	97	Mycobacterium virus Ericb	NA	Viruses; Duplodnaviria; Heunggongvirae; Uroviricota; Caudoviricetes; Caudovirales; Siphoviridae; Fromanvirus
GCA_000891215.1	40940	49	Escherichia phage K30	NA	Viruses; Duplodnaviria; Heunggongvirae; Uroviricota; Caudoviricetes; Caudovirales; Autographiviridae; Studiervirinae; Przondovirus; Escherichia virus K30
GCA_000913135.1	139738	206	Staphylococcus phage S253	NA	Viruses; dsDNA viruses, no RNA stage; Caudovirales; Myoviridae; Spounavirinae; Kayvirus; Staphylococcus virus S253
GCA_000911475.1	132123	184	Staphylococcus phage S254	NA	Viruses; dsDNA viruses, no RNA stage; Caudovirales; Myoviridae; Spounavirinae; Twortvirus; unclassified Twortlikevirus
GCA_002270865.1	5898	3	Lake Sinai virus 1	SA	Viruses; Virus families not assigned to an order; Sinaivirus

gca	genome.size	proteome.size	phage.name	strain	Taxonomy
GCA_002601205.1	51083	90	Mycobacterium virus Benedict	NA	Viruses; Duplodnaviria; Heunggongvirae; Uroviricota; Caudoviricetes; Caudovirales; Siphoviridae; Fromanvirus
GCA_002601225.1	51241	89	Mycobacterium phage Airmid	NA	Viruses; Duplodnaviria; Heunggongvirae; Uroviricota; Caudoviricetes; Caudovirales; Siphoviridae; Fromanvirus; unclassified Fromanvirus
GCA_002271025.1	5911	3	Lake Sinai virus 2	VN3	Viruses; Virus families not assigned to an order; Sinaivirus
GCA_002219505.1	9691	2	Big Sioux River virus	Kenya P9	Viruses; ssRNA viruses; ssRNA positive-strand viruses, no DNA stage; Picornavirales; Dicistroviridae; unclassified Dicistroviridae
GCA_000891195.1	20128	7	Cavally virus	NA	Viruses; ssRNA viruses; ssRNA positive-strand viruses, no DNA stage; Nidovirales; Mesoniviridae; Alphamesonivirus; Alphamesonivirus 1
GCA_000901755.1	38329	55	Clostridium phage phiCP13O	NA	Viruses; dsDNA viruses, no RNA stage; Caudovirales; Siphoviridae; unclassified Siphoviridae
GCA_000903275.1	38309	52	Clostridium phage phiCP34O	NA	Viruses; dsDNA viruses, no RNA stage; Caudovirales; Siphoviridae; unclassified Siphoviridae
GCA_002630625.1	39594	49	Clostridium phage phiCP9O	NA	Viruses; Duplodnaviria; Heunggongvirae; Uroviricota; Caudoviricetes; Caudovirales; Siphoviridae; unclassified Siphoviridae
GCA_000863305.1	8612	6	Hop latent virus	NA	Viruses; ssRNA viruses; ssRNA positive-strand viruses, no DNA stage; Tymovirales; Betaflexiviridae; Quinvirinae; Carlavirus
GCA_000893735.1	7718	5	Blackberry virus E	NA	Viruses; ssRNA viruses; ssRNA positive-strand viruses, no DNA stage; Tymovirales; Alphaflexiviridae; unclassified Alphaflexiviridae
GCA_000885795.1	158091	77	Anatid herpesvirus 1	VAC	Viruses; dsDNA viruses, no RNA stage; Herpesvirales; Herpesviridae; Alphaherpesvirinae; Mardivirus
GCA_000846205.1	161046	164	Lymantria dispar multiple nucleopolyhedrovirus	NA	Viruses; dsDNA viruses, no RNA stage; Baculoviridae; Alphabaculovirus
GCA_000839865.1	135611	139	Spodoptera exigua multiple nucleopolyhedrovirus	NA	Viruses; dsDNA viruses, no RNA stage; Baculoviridae; Alphabaculovirus
GCA_000868825.1	131331	143	Spodoptera frugiperda multiple nucleopolyhedrovirus	NA	Viruses; dsDNA viruses, no RNA stage; Baculoviridae; Alphabaculovirus
GCA_002819145.1	137998	132	Spodoptera littoralis nucleopolyhedrovirus	NA	Viruses; Baculoviridae; Alphabaculovirus
GCA_000893755.1	4926	3	Helicoverpa armigera densovirus	SDBH20101	Viruses; ssDNA viruses; Parvoviridae; Densovirinae; Iteradensovirus; Lepidopteran iteradensovirus 5
GCA_000851685.1	20985	12	Kadipiro virus	JKT7075	Viruses; dsRNA viruses; Reoviridae; Sedoreovirinae; Seadornavirus
GCA_004786355.1	9417	2	Chaetoceros species RNA virus 02	NA	Viruses; Riboviria; Orthornavirae; Pisuviricota; Pisoniviricetes; Picornavirales; Marnaviridae; Sogarnavirus; Chaetarnavirus 2
GCA_000856145.1	18445	10	St Croix River virus	NA	Viruses; dsRNA viruses; Reoviridae; Sedoreovirinae; Orbivirus
GCA_000925275.1	11135	6	Maraba virus	NA	Viruses; ssRNA viruses; ssRNA negative-strand viruses; Mononegavirales; Rhabdoviridae; Vesiculovirus
GCA_004131725.1	119054	130	Operophtera brumata nucleopolyhedrovirus	NA	Viruses; Baculoviridae; Alphabaculovirus
GCA_003180955.1	8249	1	Cardamom mosaic virus	NA	Viruses; Riboviria; Orthornavirae; Pisuviricota; Stelpaviricetes; Patatavirales; Potyviridae; Macluravirus
GCA_002819185.1	131557	152	Agrotis segetum granulovirus	DA	Viruses; dsDNA viruses, no RNA stage; Baculoviridae; Betabaculovirus
GCA_000899815.1	8410	6	Potato virus H	NA	Viruses; ssRNA viruses; ssRNA positive-strand viruses, no DNA stage; Tymovirales; Betaflexiviridae; Quinvirinae; Carlavirus
GCA_000894875.2	5666	6	Brassica yellows virus	NA	Viruses; ssRNA viruses; ssRNA positive-strand viruses, no DNA stage; Luteoviridae; Polerovirus; unclassified Polerovirus
GCA_000895795.1	5292	7	Sida mosaic Alagoas virus	NA	Viruses; ssDNA viruses; Geminiviridae; Begomovirus; unclassified Begomovirus
GCA_000895175.1	5282	7	Abutilon mosaic Brazil virus	NA	Viruses; ssDNA viruses; Geminiviridae; Begomovirus
GCA_004787555.2	5389	9	West African Asystasia virus 1	NA	Viruses; ssDNA viruses; Geminiviridae; Begomovirus
GCA_002824745.1	2744	6	West African Asystasia virus 2	NA	Viruses; Monodnaviria; Shotokuvirae; Cressdnaviricota; Repensiviricetes; Geplafuvirales; Geminiviridae; Begomovirus
GCA_000847765.1	7560	6	Indian citrus ringspot virus	NA	Viruses; ssRNA viruses; ssRNA positive-strand viruses, no DNA stage; Tymovirales; Alphaflexiviridae; Mandarivirus
GCA_003985425.1	1832	1	Picobiliphyte sp MS5845 nanovirus	NA	Viruses; Monodnaviria; Shotokuvirae; Cressdnaviricota; Arfiviricetes; Mulpavirales; Nanoviridae; Nanovirus; unclassified Nanovirus
GCA_003344325.1	176495	235	Cyanophage STIM4	NA	Viruses; Duplodnaviria; Heunggongvirae; Uroviricota; Caudoviricetes; Caudovirales; Myoviridae; Thaumasvirus; Synechococcus virus STIM4
GCA_000914935.1	47180	74	Escherichia phage phiEB49	NA	Viruses; Duplodnaviria; Heunggongvirae; Uroviricota; Caudoviricetes; Caudovirales; Drexlerviridae; Rogunavirinae; Lindendrivevirus; Escherichia virus phiEB49
GCA_000867225.1	240596	5	Campoletis sonorensis ichnovirus	Texas AM	Viruses; dsDNA viruses, no RNA stage; Polydnaviridae; Ichnovirus

gca	genome.size	proteome.size	phage.name	strain	Taxonomy
GCA_000900415.1	9508	1	Rose yellow mosaic virus	NA	Viruses; ssRNA viruses; ssRNA positive-strand viruses, no DNA stage; Potyviridae; unassigned Potyviridae
GCA_008725575.1	36122	55	EBPR podovirus 1	NA	Viruses; Duplodnaviria; Heunggongvirae; Uroviricota; Caudoviricetes; Caudovirales; Podoviridae; unclassified Podoviridae
GCA_008725555.1	40628	67	EBPR podovirus 2	NA	Viruses; Duplodnaviria; Heunggongvirae; Uroviricota; Caudoviricetes; Caudovirales; Podoviridae; unclassified Podoviridae
GCA_008725595.1	38991	54	EBPR podovirus 3	NA	Viruses; Duplodnaviria; Heunggongvirae; Uroviricota; Caudoviricetes; Caudovirales; Podoviridae; unclassified Podoviridae
GCA_000897295.1	1376	1	Cotton leaf curl Multan alphasatellite	NA	Viruses; Satellites; Satellite Nucleic Acids; Single stranded DNA satellites; Alphasatellites; Begomovirus-associated alphasatellites
GCA_002987005.1	2792	6	Ageratum leaf curl Cameroon virus CMAGFG242009	NA	Viruses; Monodnaviria; Shotokuvirae; Cressdnaviricota; Repensiviricetes; Geplafuvirales; Geminiviridae; Begomovirus; Tomato leaf curl Ghana virus
GCA_013086495.1	12507	4	Phlebovirus CoAr 171616	NA	Viruses; Riboviria; Orthornavirae; Negarnaviricota; Polyploviricotina; Ellioviricetes; Bunyavirales; Phenuiviridae; Phlebovirus; unclassified Phlebovirus
GCA_002008555.1	12923	4	Munguba virus	NA	Viruses; Riboviria; Orthornavirae; Negarnaviricota; Polyploviricotina; Ellioviricetes; Bunyavirales; Phenuiviridae; Phlebovirus; Bujaru phlebovirus
GCA_013086505.1	12087	4	Odrenisrou virus	NA	Viruses; Riboviria; Orthornavirae; Negarnaviricota; Polyploviricotina; Ellioviricetes; Bunyavirales; Phenuiviridae; Phlebovirus; Odrenisrou phlebovirus
GCA_013086515.1	14077	4	Phlebovirus GGP2011a	NA	Viruses; Riboviria; Orthornavirae; Negarnaviricota; Polyploviricotina; Ellioviricetes; Bunyavirales; Phenuiviridae; Phlebovirus; unclassified Phlebovirus
GCA_000898295.2	166687	256	Acinetobacter phage ZZ1	NA	Viruses; dsDNA viruses, no RNA stage; Caudovirales; Myoviridae; Tevenvirinae; T4virus; unclassified T4virus
GCA_007832735.1	104968	109	Frog virus 3	NA	Viruses; dsDNA viruses, no RNA stage; Iridoviridae; Ranavirus
GCA_007998955.1	191911	246	African swine fever virus	NA	Viruses; dsDNA viruses, no RNA stage; Asfarviridae; Asfivirus
GCA_000891255.1	7753	3	Grapevine veinclearing virus	NA	Viruses; Retro-transcribing viruses; Caulimoviridae; Badnavirus
GCA_000847045.1	330611	802	Paramecium bursaria Chlorella virus 1	NA	Viruses; dsDNA viruses, no RNA stage; Phycodnaviridae; Chlorovirus
GCA_002024655.1	3134	2	Pepper cryptic virus 2	NA	Viruses; dsRNA viruses; Partitiviridae; Deltapartitivirus
GCA_000844245.1	34446	28	Bovine adenovirus 3	WBR1	Viruses; Varidnaviria; Bamfordvirae; Preplasmiviricota; Tectiliviricetes; Rowavirales; Adenoviridae; Mastadenovirus; Bovine mastadenovirus B
GCA_016759075.1	30034	30	Bovine adenovirus 7	Fukuroi	Viruses; Varidnaviria; Bamfordvirae; Preplasmiviricota; Tectiliviricetes; Rowavirales; Adenoviridae; Atadenovirus; unclassified Atadenovirus; Bovine adenovirus F
GCA_000919315.1	51421	85	Mycobacterium virus Doom	NA	Viruses; Duplodnaviria; Heunggongvirae; Uroviricota; Caudoviricetes; Caudovirales; Siphoviridae; Fromanvirus
GCA_000918655.1	53597	94	Mycobacterium virus Euphoria	NA	Viruses; Duplodnaviria; Heunggongvirae; Uroviricota; Caudoviricetes; Caudovirales; Siphoviridae; Fromanvirus
GCA_002601245.1	59749	94	Mycobacterium virus Jaws	NA	Viruses; Duplodnaviria; Heunggongvirae; Uroviricota; Caudoviricetes; Caudovirales; Siphoviridae; Timquatrovirus
GCA_000857845.1	30536	31	Canine adenovirus 1	NA	Viruses; dsDNA viruses, no RNA stage; Adenoviridae; Mastadenovirus; Canine mastadenovirus A
GCA_000856885.1	31323	31	Canine adenovirus 2	NA	Viruses; dsDNA viruses, no RNA stage; Adenoviridae; Mastadenovirus; Canine mastadenovirus A
GCA_006400915.1	35954	46	Human adenovirus 2	NA	Viruses; dsDNA viruses, no RNA stage; Adenoviridae; Mastadenovirus; Human mastadenovirus C
GCA_000849665.1	4563	8	Chlamydia phage 2	NA	Viruses; ssDNA viruses; Microviridae; Gokushovirinae; Chlamydiamicrovirus
GCA_001275515.1	40758	55	Streptococcus phage YMC2011	NA	Viruses; dsDNA viruses, no RNA stage; Caudovirales; Siphoviridae; unclassified Siphoviridae
GCA_000902475.1	39282	51	Erwinia phage vB_EamPL1	NA	Viruses; dsDNA viruses, no RNA stage; Caudovirales; Podoviridae; Autographivirinae; T7virus; unclassified T7-like viruses
GCA_002624445.1	84694	117	Erwinia phage vB_EamMM7	NA	Viruses; Duplodnaviria; Heunggongvirae; Uroviricota; Caudoviricetes; Caudovirales; Myoviridae; Ounavirinae; Kolesnikvirus; Erwinia virus M7
GCA_000901855.1	74669	115	Erwinia phage vB_EamPS6	NA	Viruses; dsDNA viruses, no RNA stage; Caudovirales; Podoviridae; N4virus; unclassified N4likevirus
GCA_000903375.1	56621	92	Erwinia phage vB_EamMY2	NA	Viruses; dsDNA viruses, no RNA stage; Caudovirales; Myoviridae; unclassified Myoviridae
GCA_000894735.2	7259	3	Grapevine Pinot gris virus	NA	Viruses; ssRNA viruses; ssRNA positive-strand viruses, no DNA stage; Tymovirales; Betaflexiviridae; Trivirinae; Trichovirus
GCA_006448615.1	35306	49	Human adenovirus 7	Gomen	Viruses; dsDNA viruses, no RNA stage; Adenoviridae; Mastadenovirus; Human mastadenovirus B; Human adenovirus B1
GCA_006443895.1	34760	40	Human adenovirus 14	NA	Viruses; Varidnaviria; Bamfordvirae; Preplasmiviricota; Tectiliviricetes; Rowavirales; Adenoviridae; Mastadenovirus; Human mastadenovirus B; Human adenovirus B2
GCA_006448335.1	34794	39	Human adenovirus 35	35p	Viruses; dsDNA viruses, no RNA stage; Adenoviridae; Mastadenovirus; Human mastadenovirus B; Human adenovirus B2

gca	genome.size	proteome.size	phage.name	strain	Taxonomy
GCA_015244825.1	34182	36	Human adenovirus 41	NA	Viruses; Varidnaviria; Bamfordvirae; Preplasmiviricota; Tectiliviricetes; Rowavirales; Adenoviridae; Mastadenovirus; Human mastadenovirus F
GCA_000919295.1	68720	101	Mycobacterium phage Oline	NA	Viruses; dsDNA viruses, no RNA stage; Caudovirales; Siphoviridae; unclassified Siphoviridae; PG1-like phages
GCA_002624925.1	155286	230	Mycobacterium phage Sebata	NA	Viruses; Duplodnaviria; Heunggongvirae; Uroviricota; Caudoviricetes; Caudovirales; Myoviridae; Bixzunavirus; Mycobacterium virus Sebata
GCA_000919335.1	106327	216	Mycobacterium phage Thibault	NA	Viruses; dsDNA viruses, no RNA stage; Caudovirales; Siphoviridae; Omegavirus
GCA_000845985.1	35083	39	Human adenovirus D9	type strain ATCC VR1086 = Hicks = NIAID V209003014	Viruses; Varidnaviria; Bamfordvirae; Preplasmiviricota; Tectiliviricetes; Rowavirales; Adenoviridae; Mastadenovirus; Human mastadenovirus D
GCA_006447935.1	34177	35	Human adenovirus 18	NA	Viruses; Varidnaviria; Bamfordvirae; Preplasmiviricota; Tectiliviricetes; Rowavirales; Adenoviridae; Mastadenovirus; Human mastadenovirus A
GCA_006448375.1	33763	34	Human adenovirus 31	NA	Viruses; Varidnaviria; Bamfordvirae; Preplasmiviricota; Tectiliviricetes; Rowavirales; Adenoviridae; Mastadenovirus; Human mastadenovirus A
GCA_000885275.1	30944	29	Murine adenovirus 1	NA	Viruses; Varidnaviria; Bamfordvirae; Preplasmiviricota; Tectiliviricetes; Rowavirales; Adenoviridae; Mastadenovirus; Murine mastadenovirus A
GCA_006449135.1	31045	27	Simian adenovirus 7	ATCC VR201 80452WN	Viruses; Varidnaviria; Bamfordvirae; Preplasmiviricota; Tectiliviricetes; Rowavirales; Adenoviridae; Mastadenovirus; unclassified Simian adenoviruses
GCA_009499455.1	35946	40	Human adenovirus 1	SH2016	Viruses; dsDNA viruses, no RNA stage; Adenoviridae; Mastadenovirus; Human mastadenovirus C
GCA_006447515.1	35758	54	Human adenovirus 6	Tonsil 99 prototype	Viruses; Varidnaviria; Bamfordvirae; Preplasmiviricota; Tectiliviricetes; Rowavirales; Adenoviridae; Mastadenovirus; Human mastadenovirus C
GCA_013373825.1	30533	31	Canine mastadenovirus A	D43	Viruses; dsDNA viruses, no RNA stage; Adenoviridae; Mastadenovirus
GCA_000857085.1	34794	38	Human adenovirus 11	Ad11p Slobitski	Viruses; Varidnaviria; Bamfordvirae; Preplasmiviricota; Tectiliviricetes; Rowavirales; Adenoviridae; Mastadenovirus; Human mastadenovirus B; Human adenovirus B2
GCA_006448115.1	34794	40	Human adenovirus 11	Slobitski	Viruses; Varidnaviria; Bamfordvirae; Preplasmiviricota; Tectiliviricetes; Rowavirales; Adenoviridae; Mastadenovirus; Human mastadenovirus B; Human adenovirus B2
GCA_000891295.1	72917	79	Escherichia phage vB_EcoP_G7C	NA	Viruses; dsDNA viruses, no RNA stage; Caudovirales; Podoviridae; Picovirinae; G7cvirus; Escherichia virus G7C
GCA_000900835.1	166452	273	Escherichia phage wV7	NA	Viruses; dsDNA viruses, no RNA stage; Caudovirales; Myoviridae; Tevenvirinae; T4virus; unclassified T4virus
GCA_001045305.1	3218	2	Beauveria bassiana RNA virus 1	NA	Viruses; Riboviria; dsRNA viruses; unclassified dsRNA viruses
GCA_013202105.1	45757	50	Fowl aviadenovirus 10	NA	Viruses; Varidnaviria; Bamfordvirae; Preplasmiviricota; Tectiliviricetes; Rowavirales; Adenoviridae; Aviadenovirus; Fowl aviadenovirus C
GCA_006448555.1	34775	41	Human adenovirus 34	Compton ATCC VR716	Viruses; Varidnaviria; Bamfordvirae; Preplasmiviricota; Tectiliviricetes; Rowavirales; Adenoviridae; Mastadenovirus; Human mastadenovirus B; Human adenovirus B2
GCA_000902495.1	166499	268	Escherichia phage ime09	NA	Viruses; dsDNA viruses, no RNA stage; Caudovirales; Myoviridae; Tevenvirinae; T4virus; Escherichia virus Ime09
GCA_000892955.1	89916	151	Salmonella phage 711	NA	Viruses; dsDNA viruses, no RNA stage; Caudovirales; Podoviridae; unclassified Podoviridae
GCA_000845105.1	43804	38	Fowl aviadenovirus 1	Phelps ATCC VR432	Viruses; Varidnaviria; Bamfordvirae; Preplasmiviricota; Tectiliviricetes; Rowavirales; Adenoviridae; Aviadenovirus; Fowl aviadenovirus A
GCA_000896435.1	7326	7	Human papillomavirus type 126	HPV126	Viruses; dsDNA viruses, no RNA stage; Papillomaviridae; Gammapapillomavirus; Gammapapillomavirus 11
GCA_002008775.1	12404	4	Uriurana virus	BeAr479776	Viruses; ssRNA viruses; ssRNA negative-strand viruses; Bunyavirales; Phenuiviridae; Phlebovirus; unclassified Phlebovirus
GCA_000863665.1	7276	8	Xipapillomavirus 1	NA	Viruses; dsDNA viruses, no RNA stage; Papillomaviridae; Xipapillomavirus
GCA_003177695.1	7296	7	Bos taurus papillomavirus 6	NA	Viruses; Monodnaviria; Shotokuvirae; Cossaviricota; Papovaviricetes; Zurhausenvirales; Papillomaviridae; Firstpapillomavirinae; Xipapillomavirus; Xipapillomavirus 1
GCA_002649955.1	17421	12	Rotavirus A KC1xUK reassortant UKg9KC1	NA	Viruses; Riboviria; Orthornavirae; Duplornaviricota; Resentoviricetes; Reovirales; Reoviridae; Sedoreovirinae; Rotavirus; Rotavirus A; Rotavirus A reassortants
GCA_002647295.1	17421	12	Rotavirus A HC91xUK reassortant UKg9KC1	HC91xUK reassortant UKg9HC91	Viruses; Riboviria; Orthornavirae; Duplornaviricota; Resentoviricetes; Reovirales; Reoviridae; Sedoreovirinae; Rotavirus; Rotavirus A; Rotavirus A reassortants
GCA_000864665.1	8095	15	Deltapapillomavirus 1	NA	Viruses; dsDNA viruses, no RNA stage; Papillomaviridae; Deltapapillomavirus
GCA_001274345.1	7320	6	Human papillomavirus	NA	Viruses; dsDNA viruses, no RNA stage; Papillomaviridae; unclassified Papillomaviridae; Human papillomavirus types
GCA_000839345.1	7687	6	Iotapapillomavirus 1	NA	Viruses; dsDNA viruses, no RNA stage; Papillomaviridae; Iotapapillomavirus
GCA_000867785.1	7393	7	Micromys minutus papillomavirus 1	NA	Viruses; dsDNA viruses, no RNA stage; Papillomaviridae; Pipapillomavirus; Pipapillomavirus 2
GCA_000920155.1	43036	68	Mycobacterium phage Charlie	NA	Viruses; dsDNA viruses, no RNA stage; Caudovirales; Siphoviridae; Charlievirus
GCA_000918635.1	62991	97	Mycobacterium virus Larva	NA	Viruses; Duplodnaviria; Heunggongvirae; Uroviricota; Caudoviricetes; Caudovirales; Siphoviridae; Timquatrovirus

gca	genome.size	proteome.size	phage.name	strain	Taxonomy
GCA_002597865.1	51368	87	Mycobacterium phage MeeZee	NA	Viruses; Duplodnaviria; Heunggongvirae; Uroviricota; Caudoviricetes; Caudovirales; Siphoviridae; Fromanvirus; Mycobacterium virus Peaches
GCA_000918615.1	51308	85	Mycobacterium phage Wile	NA	Viruses; dsDNA viruses, no RNA stage; Caudovirales; Siphoviridae; unclassified Siphoviridae
GCA_002220025.1	7480	6	Bovine papillomavirus	NA	Viruses; dsDNA viruses, no RNA stage; Papillomaviridae; unclassified Papillomaviridae; non-primate mammal papillomaviruses
GCA_003180475.1	7880	8	human papillomavirus 13	NA	Viruses; Monodnaviria; Shotokuvirae; Cossaviricota; Papovaviricetes; Zurhausenvirales; Papillomaviridae; Firstpapillomavirinae; Alphapapillomavirus; Alphapapillomavirus 10
GCA_003180495.1	7902	8	Pygmy chimpanzee papillomavirus type 1	NA	Viruses; Monodnaviria; Shotokuvirae; Cossaviricota; Papovaviricetes; Zurhausenvirales; Papillomaviridae; Firstpapillomavirinae; Alphapapillomavirus; Alphapapillomavirus 10
GCA_003179995.1	7931	9	human papillomavirus 11	NA	Viruses; Monodnaviria; Shotokuvirae; Cossaviricota; Papovaviricetes; Zurhausenvirales; Papillomaviridae; Firstpapillomavirinae; Alphapapillomavirus; Alphapapillomavirus 10
GCA_000866405.1	7815	5	Human papillomavirus type 1a	NA	Viruses; Monodnaviria; Shotokuvirae; Cossaviricota; Papovaviricetes; Zurhausenvirales; Papillomaviridae; Firstpapillomavirinae; Mupapillomavirus; Mupapillomavirus 1
GCA_003179095.1	7912	8	human papillomavirus 31	NA	Viruses; Monodnaviria; Shotokuvirae; Cossaviricota; Papovaviricetes; Zurhausenvirales; Papillomaviridae; Firstpapillomavirinae; Alphapapillomavirus; Alphapapillomavirus 9
GCA_003179955.1	7909	8	human papillomavirus 33	NA	Viruses; Monodnaviria; Shotokuvirae; Cossaviricota; Papovaviricetes; Zurhausenvirales; Papillomaviridae; Firstpapillomavirinae; Alphapapillomavirus; Alphapapillomavirus 9
GCA_003180055.1	7833	8	human papillomavirus 39	NA	Viruses; Monodnaviria; Shotokuvirae; Cossaviricota; Papovaviricetes; Zurhausenvirales; Papillomaviridae; Firstpapillomavirinae; Alphapapillomavirus; Alphapapillomavirus 7
GCA_000863845.1	7614	11	Human papillomavirus type 41	NA	Viruses; dsDNA viruses, no RNA stage; Papillomaviridae; Nupapillomavirus; Nupapillomavirus 1
GCA_003180095.1	7917	8	human papillomavirus 42	NA	Viruses; Monodnaviria; Shotokuvirae; Cossaviricota; Papovaviricetes; Zurhausenvirales; Papillomaviridae; Firstpapillomavirinae; Alphapapillomavirus; Alphapapillomavirus 1
GCA_003177675.1	7975	10	human papillomavirus 43	NA	Viruses; Monodnaviria; Shotokuvirae; Cossaviricota; Papovaviricetes; Zurhausenvirales; Papillomaviridae; Firstpapillomavirinae; Alphapapillomavirus; Alphapapillomavirus 8
GCA_003180375.1	7833	7	human papillomavirus 44	NA	Viruses; Monodnaviria; Shotokuvirae; Cossaviricota; Papovaviricetes; Zurhausenvirales; Papillomaviridae; Firstpapillomavirinae; Alphapapillomavirus; Alphapapillomavirus 10
GCA_003180735.1	7858	6	human papillomavirus 45	NA	Viruses; Monodnaviria; Shotokuvirae; Cossaviricota; Papovaviricetes; Zurhausenvirales; Papillomaviridae; Firstpapillomavirinae; Alphapapillomavirus; Alphapapillomavirus 7
GCA_003180035.1	7726	8	human papillomavirus 47	NA	Viruses; Monodnaviria; Shotokuvirae; Cossaviricota; Papovaviricetes; Zurhausenvirales; Papillomaviridae; Firstpapillomavirinae; Betapapillomavirus; Betapapillomavirus 1
GCA_003180775.1	7844	5	human papillomavirus 56	NA	Viruses; Monodnaviria; Shotokuvirae; Cossaviricota; Papovaviricetes; Zurhausenvirales; Papillomaviridae; Firstpapillomavirinae; Alphapapillomavirus; Alphapapillomavirus 6
GCA_003177895.1	7824	8	human papillomavirus 58	NA	Viruses; Monodnaviria; Shotokuvirae; Cossaviricota; Papovaviricetes; Zurhausenvirales; Papillomaviridae; Firstpapillomavirinae; Alphapapillomavirus; Alphapapillomavirus 9
GCA_000861945.1	7902	9	Human papillomavirus type 6b	type 6b HPV6b	Viruses; dsDNA viruses, no RNA stage; Papillomaviridae; Alphapapillomavirus; Alphapapillomavirus 10
GCA_003180555.1	7673	6	human papillomavirus 12	NA	Viruses; Monodnaviria; Shotokuvirae; Cossaviricota; Papovaviricetes; Zurhausenvirales; Papillomaviridae; Firstpapillomavirinae; Betapapillomavirus; Betapapillomavirus 1
GCA_003180595.1	7412	6	Human papillomavirus 15	NA	Viruses; Monodnaviria; Shotokuvirae; Cossaviricota; Papovaviricetes; Zurhausenvirales; Papillomaviridae; Firstpapillomavirinae; Betapapillomavirus; Betapapillomavirus 2
GCA_003180615.1	7427	6	Human papillomavirus 17	NA	Viruses; Monodnaviria; Shotokuvirae; Cossaviricota; Papovaviricetes; Zurhausenvirales; Papillomaviridae; Firstpapillomavirinae; Betapapillomavirus; Betapapillomavirus 2
GCA_003180635.1	7685	6	Human papillomavirus 19	NA	Viruses; Monodnaviria; Shotokuvirae; Cossaviricota; Papovaviricetes; Zurhausenvirales; Papillomaviridae; Firstpapillomavirinae; Betapapillomavirus; Betapapillomavirus 1
GCA_003180655.1	7713	6	human papillomavirus 25	NA	Viruses; Monodnaviria; Shotokuvirae; Cossaviricota; Papovaviricetes; Zurhausenvirales; Papillomaviridae; Firstpapillomavirinae; Betapapillomavirus; Betapapillomavirus 1
GCA_002987345.1	7852	6	human papillomavirus 30	NA	Viruses; Monodnaviria; Shotokuvirae; Cossaviricota; Papovaviricetes; Zurhausenvirales; Papillomaviridae; Firstpapillomavirinae; Alphapapillomavirus; Alphapapillomavirus 6
GCA_000871905.1	8404	6	Potato rough dwarf virus	NA	Viruses; Riboviria; Orthornavirae; Kitrinoviricota; Alsuviricetes; Tymovirales; Betaflexiviridae; Quinvirinae; Carlavirus; Potato virus P
GCA_003180535.1	7820	7	Human papillomavirus 3	NA	Viruses; Monodnaviria; Shotokuvirae; Cossaviricota; Papovaviricetes; Zurhausenvirales; Papillomaviridae; Firstpapillomavirinae; Alphapapillomavirus; Alphapapillomavirus 2
GCA_003180715.1	7909	6	human papillomavirus 40	NA	Viruses; Monodnaviria; Shotokuvirae; Cossaviricota; Papovaviricetes; Zurhausenvirales; Papillomaviridae; Firstpapillomavirinae; Alphapapillomavirus; Alphapapillomavirus 8
GCA_000862825.1	7560	6	Human papillomavirus type 49	NA	Viruses; dsDNA viruses, no RNA stage; Papillomaviridae; Betapapillomavirus; Betapapillomavirus 3
GCA_000864845.1	7353	7	Human papillomavirus type 4	NA	Viruses; dsDNA viruses, no RNA stage; Papillomaviridae; Gammapapillomavirus; Gammapapillomavirus 1
GCA_003180755.1	7942	6	human papillomavirus 52	NA	Viruses; Monodnaviria; Shotokuvirae; Cossaviricota; Papovaviricetes; Zurhausenvirales; Papillomaviridae; Firstpapillomavirinae; Alphapapillomavirus; Alphapapillomavirus 9
GCA_000861925.1	8027	6	Human papillomavirus type 7	NA	Viruses; dsDNA viruses, no RNA stage; Papillomaviridae; Alphapapillomavirus; Alphapapillomavirus 8
GCA_000863685.1	7434	7	Human papillomavirus type 9	NA	Viruses; dsDNA viruses, no RNA stage; Papillomaviridae; Betapapillomavirus; Betapapillomavirus 2
GCA_000837405.1	43081	66	Staphylococcus virus phiETA	NA	Viruses; Duplodnaviria; Heunggongvirae; Uroviricota; Caudoviricetes; Caudovirales; Siphoviridae; Phietavirus

gca	genome.size	proteome.size	phage.name	strain	Taxonomy
GCA_000863805.1	5130	6	JC polyomavirus	Mad1	Viruses; dsDNA viruses, no RNA stage; Polyomaviridae; Betapolyomavirus; Human polyomavirus 2
GCA_000843305.1	149696	72	Callitrichine gammaherpesvirus 3	CJ0149	Viruses; dsDNA viruses, no RNA stage; Herpesvirales; Herpesviridae; Gammaherpesvirinae; Lymphocryptovirus
GCA_000849945.1	7320	2	Porcine enteric sapovirus	Cowden	Viruses; ssRNA viruses; ssRNA positive-strand viruses, no DNA stage; Caliciviridae; Sapovirus; Sapporo virus; Sapovirus GIII
GCA_000845845.1	7932	6	Carnation etched ring virus	NA	Viruses; Riboviria; Pararnavirae; Artverviricota; Revtraviricetes; Ortervirales; Caulimoviridae; Caulimovirus
GCA_000848745.1	8024	6	Cauliflower mosaic virus	NA	Viruses; Riboviria; Pararnavirae; Artverviricota; Revtraviricetes; Ortervirales; Caulimoviridae; Caulimovirus
GCA_004790335.1	12629	6	Clerodendrum chlorotic spot virus	NA	Viruses; Riboviria; Orthornavirae; Negarnaviricota; Haploviricotina; Monjiviricetes; Mononegavirales; Rhabdoviridae; Dichorhavirus; Clerodendrum chlorotic spot dichorhavirus
GCA_000845905.1	7743	7	Figwort mosaic virus	NA	Viruses; Riboviria; Pararnavirae; Artverviricota; Revtraviricetes; Ortervirales; Caulimoviridae; Caulimovirus
GCA_000847985.1	8178	9	Soybean chlorotic mottle virus	NA	Viruses; Riboviria; Pararnavirae; Artverviricota; Revtraviricetes; Ortervirales; Caulimoviridae; Soymovirus
GCA_000849585.1	7489	3	Commelina yellow mottle virus	NA	Viruses; Riboviria; Pararnavirae; Artverviricota; Revtraviricetes; Ortervirales; Caulimoviridae; Badnavirus
GCA_000849605.1	8002	4	Rice tungro bacilliform virus	NA	Viruses; Riboviria; Pararnavirae; Artverviricota; Revtraviricetes; Ortervirales; Caulimoviridae; Tungrovirus
GCA_000837025.1	14927	31	Enterobacteria phage PRD1	NA	Viruses; dsDNA viruses, no RNA stage; Tectiviridae; Tectivirus
GCA_000840405.1	10079	22	Pseudoalteromonas virus PM2	NA	Viruses; Varidnaviria; Bamfordvirae; Preplasmiviricota; Tectiliviricetes; Vinavirales; Corticoviridae; Corticovirus
GCA_013150865.1	163826	265	Enterobacteria phage T2	NA	Viruses; Duplodnaviria; Heunggongvirae; Uroviricota; Caudoviricetes; Caudovirales; Myoviridae; Tevenvirinae; Tequatrovirus; unclassified Tequatrovirus
GCA_002599625.1	168922	202	Escherichia virus T4	wild	Viruses; Duplodnaviria; Heunggongvirae; Uroviricota; Caudoviricetes; Caudovirales; Myoviridae; Tevenvirinae; Tequatrovirus
GCA_003423385.1	168706	268	Enterobacteria phage T6	NA	Viruses; Duplodnaviria; Heunggongvirae; Uroviricota; Caudoviricetes; Caudovirales; Myoviridae; Tevenvirinae; Tequatrovirus; unclassified Tequatrovirus
GCA_000844165.1	94800	110	Escherichia virus P1	NA	Viruses; dsDNA viruses, no RNA stage; Caudovirales; Myoviridae; P1virus
GCA_000836905.1	33593	43	Escherichia virus P2	NA	Viruses; dsDNA viruses, no RNA stage; Caudovirales; Myoviridae; Peduovirinae; P2virus
GCA_000846325.1	11624	13	Enterobacteria phage P4	NA	Viruses; dsDNA viruses, no RNA stage; Caudovirales; Myoviridae; Peduovirinae; P2virus; unclassified P2likevirus
GCA_002755035.1	101660	113	Enterobacteria phage P7	NA	Viruses; Duplodnaviria; Heunggongvirae; Uroviricota; Caudoviricetes; Caudovirales; Myoviridae; Punavirus; Escherichia virus P1
GCA_002601325.1	252197	311	Bacillus virus PBS1	NA	Viruses; Duplodnaviria; Heunggongvirae; Uroviricota; Caudoviricetes; Caudovirales; Myoviridae
GCA_000881675.1	132562	204	Bacillus virus SPO1	NA	Viruses; dsDNA viruses, no RNA stage; Caudovirales; Myoviridae; Spounavirinae; Spo1virus
GCA_000903155.1	44492	70	Vibrio phage CPT1	NA	Viruses; dsDNA viruses, no RNA stage; Caudovirales; Myoviridae; unclassified Myoviridae
GCA_000837885.1	32355	41	Haemophilus virus HP1	HP1c1	Viruses; Duplodnaviria; Heunggongvirae; Uroviricota; Caudoviricetes; Caudovirales; Myoviridae; Peduovirinae; Hpunavirus
GCA_003369385.1	166677	273	Enterobacteria phage RB18	NA	Viruses; Duplodnaviria; Heunggongvirae; Uroviricota; Caudoviricetes; Caudovirales; Myoviridae; Tevenvirinae; Tequatrovirus; unclassified Tequatrovirus
GCA_000881275.1	168394	273	Enterobacteria phage RB51	NA	Viruses; dsDNA viruses, no RNA stage; Caudovirales; Myoviridae; Tevenvirinae; T4virus; unclassified T4virus
GCA_006447375.1	33776	34	Human adenovirus 61	JPN20045082P31HNEWF31	Viruses; Varidnaviria; Bamfordvirae; Preplasmiviricota; Tectiliviricetes; Rowavirales; Adenoviridae; Mastadenovirus; Human mastadenovirus A
GCA_000850125.1	9799	1	Cryphonectria hypovirus 3	NA	Viruses; dsRNA viruses; Hypoviridae; Hypovirus
GCA_008766975.1	7370	1	Enterovirus G	NA	Viruses; ssRNA viruses; ssRNA positive-strand viruses, no DNA stage; Picornavirales; Picornaviridae; Enterovirus
GCA_008800175.1	7401	1	Porcine enterovirus 10	LP 54	Viruses; Riboviria; Orthornavirae; Pisuviricota; Pisoniviricetes; Picornavirales; Picornaviridae; Enterovirus; Enterovirus G
GCA_000844125.1	49220	61	Streptomyces phage VWB	NA	Viruses; dsDNA viruses, no RNA stage; Caudovirales; Siphoviridae; Lambdavirus; unclassified Lambda-like viruses
GCA_013087105.1	139089	141	Hyposidra talaca NPV	HytaNPVIndia001	Viruses; Baculoviridae; Alphabaculovirus
GCA_001430515.1	7197	8	Bos taurus papillomavirus 12	NA	Viruses; Monodnaviria; Shotokuvirae; Cossaviricota; Papovaviricetes; Zurhausenvirales; Papillomaviridae; Firstpapillomavirinae; Xipapillomavirus; Xipapillomavirus 2
GCA_000881375.1	60195	110	Rhizobium phage 163	NA	Viruses; dsDNA viruses, no RNA stage; Caudovirales; Siphoviridae; unclassified Siphoviridae
GCA_000898235.1	7293	7	Human papillomavirus type 135	NA	Viruses; dsDNA viruses, no RNA stage; Papillomaviridae; Gammapapillomavirus; Gammapapillomavirus 15

gca	genome.size	proteome.size	phage.name	strain	Taxonomy
GCA_000899695.1	7319	7	Human papillomavirus type 136	NA	Viruses; dsDNA viruses, no RNA stage; Papillomaviridae; unclassified Papillomaviridae; Human papillomavirus types
GCA_000897255.1	7236	7	Human papillomavirus type 137	NA	Viruses; dsDNA viruses, no RNA stage; Papillomaviridae; Gammapapillomavirus; Gammapapillomavirus 16
GCA_003178915.1	7353	7	Human papillomavirus 138	NA	Viruses; Monodnaviria; Shotokuvirae; Cossaviricota; Papovaviricetes; Zurhausenvirales; Papillomaviridae; Firstpapillomavirinae; Gammapapillomavirus; Gammapapillomavirus 7
GCA_003178935.1	7360	7	Human papillomavirus 139	NA	Viruses; Monodnaviria; Shotokuvirae; Cossaviricota; Papovaviricetes; Zurhausenvirales; Papillomaviridae; Firstpapillomavirinae; Gammapapillomavirus; Gammapapillomavirus 7
GCA_000898855.1	7341	7	Human papillomavirus type 140	NA	Viruses; dsDNA viruses, no RNA stage; Papillomaviridae; unclassified Papillomaviridae; Human papillomavirus types
GCA_003178955.1	7384	7	Human papillomavirus 141	NA	Viruses; Monodnaviria; Shotokuvirae; Cossaviricota; Papovaviricetes; Zurhausenvirales; Papillomaviridae; Firstpapillomavirinae; Gammapapillomavirus; Gammapapillomavirus 11
GCA_003178975.1	7374	7	Human papillomavirus 142	NA	Viruses; Monodnaviria; Shotokuvirae; Cossaviricota; Papovaviricetes; Zurhausenvirales; Papillomaviridae; Firstpapillomavirinae; Gammapapillomavirus; Gammapapillomavirus 10
GCA_003178995.1	7715	7	Human papillomavirus 143	NA	Viruses; Monodnaviria; Shotokuvirae; Cossaviricota; Papovaviricetes; Zurhausenvirales; Papillomaviridae; Firstpapillomavirinae; Betapapillomavirus; Betapapillomavirus 1
GCA_000898255.1	7271	7	Human papillomavirus type 144	NA	Viruses; dsDNA viruses, no RNA stage; Papillomaviridae; Gammapapillomavirus; Gammapapillomavirus 17
GCA_003179015.1	7375	7	Human papillomavirus 145	NA	Viruses; Monodnaviria; Shotokuvirae; Cossaviricota; Papovaviricetes; Zurhausenvirales; Papillomaviridae; Firstpapillomavirinae; Betapapillomavirus; Betapapillomavirus 2
GCA_003179035.1	7265	7	Human papillomavirus 146	NA	Viruses; Monodnaviria; Shotokuvirae; Cossaviricota; Papovaviricetes; Zurhausenvirales; Papillomaviridae; Firstpapillomavirinae; Gammapapillomavirus; Gammapapillomavirus 15
GCA_003179055.1	7224	7	human papillomavirus 147	NA	Viruses; Monodnaviria; Shotokuvirae; Cossaviricota; Papovaviricetes; Zurhausenvirales; Papillomaviridae; Firstpapillomavirinae; Gammapapillomavirus; Gammapapillomavirus 8
GCA_000842485.1	37611	55	Pseudomonas virus D3112	NA	Viruses; Duplodnaviria; Heunggongvirae; Uroviricota; Caudoviricetes; Caudovirales; Siphoviridae; Casadabanvirus
GCA_002601385.1	74675	143	Mycobacterium phage Elph10	NA	Viruses; Duplodnaviria; Heunggongvirae; Uroviricota; Caudoviricetes; Caudovirales; Siphoviridae; Kostyavirus; unclassified Kostyavirus
GCA_000919275.1	76260	140	Mycobacterium phage Lilac	NA	Viruses; dsDNA viruses, no RNA stage; Caudovirales; Siphoviridae; unclassified Siphoviridae
GCA_002745415.1	49205	68	Escherichia virus Lambda	NA	Viruses; dsDNA viruses, no RNA stage; Caudovirales; Siphoviridae; Lambdavirus
GCA_000916615.1	35725	49	Psychrobacter phage Psymv2	NA	Viruses; dsDNA viruses, no RNA stage; Caudovirales; Siphoviridae; unclassified Siphoviridae
GCA_001015325.1	46150	63	Enterobacteria phage phi80	NA	Viruses; dsDNA viruses, no RNA stage; Caudovirales; Siphoviridae; Lambdavirus; unclassified Lambda-like viruses
GCA_008791665.1	8155	1	Kobuvirus dogAN211DUSA2009	NA	Viruses; Riboviria; Orthornavirae; Pisuviricota; Pisoniviricetes; Picornavirales; Picornaviridae; Kobuvirus; Aichivirus A
GCA_002867165.1	14304	2	Blueberry latent spherical virus	NA	Viruses; Riboviria; Orthornavirae; Pisuviricota; Pisoniviricetes; Picornavirales; Secoviridae; Comovirinae; Nepovirus
GCA_002601405.1	50965	86	Mycobacterium virus Cuco	NA	Viruses; Duplodnaviria; Heunggongvirae; Uroviricota; Caudoviricetes; Caudovirales; Siphoviridae; Fromanvirus
GCA_000917615.1	53526	92	Mycobacterium phage Trixie	NA	Viruses; dsDNA viruses, no RNA stage; Caudovirales; Siphoviridae; L5virus
GCA_000919255.1	53169	94	Mycobacterium phage Turbido	NA	Viruses; dsDNA viruses, no RNA stage; Caudovirales; Siphoviridae; L5virus
GCA_000920135.1	57387	104	Mycobacterium virus Gumbie	NA	Viruses; Duplodnaviria; Heunggongvirae; Uroviricota; Caudoviricetes; Caudovirales; Siphoviridae; Cheoctovirus
GCA_000920115.1	50844	94	Mycobacterium phage RidgeCB	NA	Viruses; dsDNA viruses, no RNA stage; Caudovirales; Siphoviridae; L5virus
GCA_002921615.1	39318	52	Bacillus phage phi105	NA	Viruses; dsDNA viruses, no RNA stage; Caudovirales; Siphoviridae; Lambdavirus; unclassified Lambda-like viruses
GCA_000848045.2	41491	53	Streptomyces virus phiC31	Norwich stock	Viruses; Duplodnaviria; Heunggongvirae; Uroviricota; Caudoviricetes; Caudovirales; Siphoviridae; Lomovskayavirus
GCA_003985465.1	2177	2	Bat circovirus ZSChina2011	NA	Viruses; Monodnaviria; Shotokuvirae; Cressdnaviricota; Arfiviricetes; Cirlivirales; Circoviridae; Cyclovirus; Dragonfly associated cyclovirus 4
GCA_000924435.1	11900	5	Bokeloh bat lyssavirus	NA	Viruses; ssRNA viruses; ssRNA negative-strand viruses; Mononegavirales; Rhabdoviridae; Lyssavirus
GCA_004369305.1	1761	1	Bat circovirus ZSYunnanChina2009	NA	Viruses; Monodnaviria; Shotokuvirae; Cressdnaviricota; Arfiviricetes; Cirlivirales; Circoviridae; Circovirus; unclassified Circovirus
GCA_002816465.1	9655	1	Lygus lineolaris virus 1	NA	Viruses; Riboviria; Orthornavirae; Pisuviricota; Pisoniviricetes; Picornavirales; Iflaviridae; Iflavirus
GCA_000847145.1	44010	97	Bacillus phage SPP1	NA	Viruses; dsDNA viruses, no RNA stage; Caudovirales; Siphoviridae; Lambdavirus; unclassified Lambda-like viruses
GCA_000902235.1	9842	1	Blue squill virus A	NA	Viruses; ssRNA viruses; ssRNA positive-strand viruses, no DNA stage; Potyviridae; Potyvirus; unclassified Potyvirus
GCA_002921405.1	121752	168	Escherichia virus T5	ATCC 11303B5	Viruses; dsDNA viruses, no RNA stage; Caudovirales; Siphoviridae; T5virus

gca	genome.size	proteome.size	phage.name	strain	Taxonomy
GCA_000899195.1	2816	4	Paspalum striate mosaic virus	NA	Viruses; ssDNA viruses; Geminiviridae; Mastrevirus
GCA_002630885.1	38552	45	Liberibacter phage FP2	NA	Viruses; Duplodnaviria; Heunggongvirae; Uroviricota; Caudoviricetes; Caudovirales; Podoviridae; unclassified Podoviridae
GCA_000899575.1	51071	86	Streptomyces phage R4	NA	Viruses; dsDNA viruses, no RNA stage; Caudovirales; Siphoviridae; unclassified Siphoviridae
GCA_000837445.1	7767	4	Tobacco vein clearing virus	NA	Viruses; Riboviria; Pararnavirae; Artverviricota; Revtraviricetes; Ortervirales; Caulimoviridae; Solendovirus
GCA_002601445.1	128375	201	Bacillus phage phi3T	NA	Viruses; Duplodnaviria; Heunggongvirae; Uroviricota; Caudoviricetes; Caudovirales; Siphoviridae; Spbetavirus; unclassified Spbetavirus
GCA_002623285.1	58619	44	Enterococcus phage SAP6	NA	Viruses; Duplodnaviria; Heunggongvirae; Uroviricota; Caudoviricetes; Caudovirales; Siphoviridae; Saphexavirus; Enterococcus virus SAP6
GCA_000901875.1	49116	45	Cronobacter virus Esp29491	NA	Viruses; Duplodnaviria; Heunggongvirae; Uroviricota; Caudoviricetes; Caudovirales; Drexlerviridae; Tunavirinae
GCA_002827325.1	4395	2	California sea lion adenoassociated virus 1	NA	Viruses; Monodnaviria; Shotokuvirae; Cossaviricota; Quintoviricetes; Piccovirales; Parvoviridae; Parvovirinae; Dependoparvovirus; Pinniped dependoparvovirus 1
GCA_002827225.1	5263	4	California sea lion bocavirus 1	NA	Viruses; Monodnaviria; Shotokuvirae; Cossaviricota; Quintoviricetes; Piccovirales; Parvoviridae; Parvovirinae; Bocaparvovirus; Pinniped bocaparvovirus 1
GCA_002827245.1	5439	4	California sea lion bocavirus 3	NA	Viruses; Monodnaviria; Shotokuvirae; Cossaviricota; Quintoviricetes; Piccovirales; Parvoviridae; Parvovirinae; Bocaparvovirus; Pinniped bocaparvovirus 2
GCA_000954235.1	4817	8	uncultured phage WWnAnB	NA	Viruses; ssDNA viruses; Inoviridae; environmental samples
GCA_000836965.1	40751	69	Escherichia virus HK022	NA	Viruses; dsDNA viruses, no RNA stage; Caudovirales; Siphoviridae; Lambdavirus
GCA_003033155.1	7704	6	Peromyscus papillomavirus 1	M14 PmPV1	Viruses; Monodnaviria; Shotokuvirae; Cossaviricota; Papovaviricetes; Zurhausenvirales; Papillomaviridae; Firstpapillomavirinae; Iotapapillomavirus; Iotapapillomavirus 2
GCA_000894795.1	6543	2	Mouse astrovirus M52USA2008	NA	Viruses; ssRNA viruses; ssRNA positive-strand viruses, no DNA stage; Astroviridae; unclassified Astroviridae
GCA_000893835.1	8198	1	Mouse kobuvirus M5USA2010	NA	Viruses; ssRNA viruses; ssRNA positive-strand viruses, no DNA stage; Picornavirales; Picornaviridae; Kobuvirus; unclassified Kobuvirus
GCA_003726295.1	2986	2	Rodent stoolassociated circular genome virus	RodSCV_V64	Viruses; unclassified viruses
GCA_000918595.1	54411	103	Mycobacterium virus Drago	NA	Viruses; Duplodnaviria; Heunggongvirae; Uroviricota; Caudoviricetes; Caudovirales; Siphoviridae; Cheoctovirus
GCA_000918575.1	41650	61	Mycobacterium virus Liefie	NA	Viruses; Duplodnaviria; Heunggongvirae; Uroviricota; Caudoviricetes; Caudovirales; Siphoviridae; Liefievirus
GCA_000917595.1	157568	238	Mycobacterium phage Dandelion	NA	Viruses; dsDNA viruses, no RNA stage; Caudovirales; Myoviridae; Bxz1virus; unclassified Bxz1virus
GCA_002601485.1	76174	145	Mycobacterium virus Eureka	NA	Viruses; Duplodnaviria; Heunggongvirae; Uroviricota; Caudoviricetes; Caudovirales; Siphoviridae; Kostyavirus
GCA_000920095.1	153882	226	Mycobacterium phage LinStu	NA	Viruses; dsDNA viruses, no RNA stage; Caudovirales; Myoviridae; Bxz1virus; unclassified Bxz1virus
GCA_000920075.1	70506	110	Mycobacterium phage Patience	NA	Viruses; dsDNA viruses, no RNA stage; Caudovirales; Siphoviridae; Barnyardvirus
GCA_000919215.1	48984	82	Mycobacterium phage BigNuz	NA	Viruses; dsDNA viruses, no RNA stage; Caudovirales; Siphoviridae; Bignuzvirus
GCA_000894815.1	10438	1	Sclerotinia sclerotiorum hypovirus 1	SZ150	Viruses; dsRNA viruses; Hypoviridae; Hypovirus; unclassified Hypovirus
GCA_006448535.1	35385	46	Human adenovirus 50	Wan ATCC VR1502	Viruses; Varidnaviria; Bamfordvirae; Preplasmiviricota; Tectiliviricetes; Rowavirales; Adenoviridae; Mastadenovirus; Human mastadenovirus B
GCA_000849625.1	19343	28	Streptococcus phage Cp1	NA	Viruses; dsDNA viruses, no RNA stage; Caudovirales; Podoviridae; Picovirinae; unassigned Picovirinae
GCA_002988105.1	19741	28	Streptococcus phage CP7	NA	Viruses; Duplodnaviria; Heunggongvirae; Uroviricota; Caudoviricetes; Caudovirales; Podoviridae; Picovirinae; Cepunavirus; Streptococcus virus Cp7
GCA_002597885.1	51367	87	Mycobacterium phage TiroTheta9	NA	Viruses; Duplodnaviria; Heunggongvirae; Uroviricota; Caudoviricetes; Caudovirales; Siphoviridae; Fromanvirus; Mycobacterium virus Peaches
GCA_000893855.1	7753	1	Bat picornavirus 1	NC16A	Viruses; ssRNA viruses; ssRNA positive-strand viruses, no DNA stage; Picornavirales; Picornaviridae; unclassified Picornaviridae
GCA_000891335.1	7693	1	Bat picornavirus 2	MH9F	Viruses; ssRNA viruses; ssRNA positive-strand viruses, no DNA stage; Picornavirales; Picornaviridae; unclassified Picornaviridae
GCA_000892935.1	7749	1	Bat picornavirus 3	TLC5F	Viruses; ssRNA viruses; ssRNA positive-strand viruses, no DNA stage; Picornavirales; Picornaviridae; unclassified Picornaviridae
GCA_003047535.1	14931	30	Bacillus phage pGIL01	NA	Viruses; Varidnaviria; Bamfordvirae; Preplasmiviricota; Tectiliviricetes; Kalamavirales; Tectiviridae; Betatectivirus; Bacillus virus Bam35
GCA_000867865.1	70153	72	Escherichia virus N4	NA	Viruses; Duplodnaviria; Heunggongvirae; Uroviricota; Caudoviricetes; Caudovirales; Podoviridae; Enquatrovirus
GCA_002755055.1	18753	27	Bacillus phage Nf	NA	Viruses; Duplodnaviria; Heunggongvirae; Uroviricota; Caudoviricetes; Caudovirales; Podoviridae; Picovirinae; unclassified Picovirinae

gca	genome.size	proteome.size	phage.name	strain	Taxonomy
GCA_000845765.1	41724	72	Salmonella virus P22	NA	Viruses; dsDNA viruses, no RNA stage; Caudovirales; Podoviridae; P22virus
GCA_000880275.1	19282	27	Bacillus virus phi29	NA	Viruses; dsDNA viruses, no RNA stage; Caudovirales; Podoviridae; Picovirinae; Phi29virus
GCA_002755075.1	19366	27	Bacillus phage PZA	NA	Viruses; Duplodnaviria; Heunggongvirae; Uroviricota; Caudoviricetes; Caudovirales; Podoviridae; Picovirinae; Salasvirus; Bacillus virus phi29
GCA_002755095.1	38202	55	Enterobacteria phage T7M	NA	Viruses; Duplodnaviria; Heunggongvirae; Uroviricota; Caudoviricetes; Caudovirales; Autographiviridae; Studiervirinae; Teetrevirus; Enterobacteria virus T7M
GCA_000841665.1	38208	55	Enterobacteria phage T3	Luria	Viruses; dsDNA viruses, no RNA stage; Caudovirales; Podoviridae; Autographivirinae; T7virus; unclassified T7-like viruses
GCA_000844825.1	39937	60	Escherichia phage T7	NA	Viruses; Duplodnaviria; Heunggongvirae; Uroviricota; Caudoviricetes; Caudovirales; Autographiviridae; Studiervirinae; Teseptimavirus; Escherichia virus T7
GCA_000845865.1	39043	66	Shigella virus Sf6	NA	Viruses; Duplodnaviria; Heunggongvirae; Uroviricota; Caudoviricetes; Caudovirales; Podoviridae; Lederbergvirus
GCA_000919235.1	53142	92	Mycobacterium virus Perseus	NA	Viruses; Duplodnaviria; Heunggongvirae; Uroviricota; Caudoviricetes; Caudovirales; Siphoviridae; Fromanvirus
GCA_000892975.1	175699	186	Yokapox virus	DakArB 4268	Viruses; Varidnaviria; Bamfordvirae; Nucleocytoviricota; Pokkesviricetes; Chitovirales; Poxviridae; Chordopoxvirinae; Centapoxvirus
GCA_000928135.1	2752	6	Catharanthus yellow mosaic virus	NA	Viruses; ssDNA viruses; Geminiviridae; Begomovirus; unclassified Begomovirus
GCA_000911935.1	53253	96	Mycobacterium phage AnnaL29	NA	Viruses; dsDNA viruses, no RNA stage; Caudovirales; Siphoviridae; unclassified Siphoviridae
GCA_002624005.1	45580	75	Mycobacterium phage Jebeks	NA	Viruses; Duplodnaviria; Heunggongvirae; Uroviricota; Caudoviricetes; Caudovirales; Siphoviridae; Pclasvirinae; Fishburnevirus; Mycobacterium virus Jebeks
GCA_001015265.1	39422	48	Vibrio phage phiVC8	NA	Viruses; dsDNA viruses, no RNA stage; Caudovirales; Podoviridae; Vp5virus; Vibrio virus VC8
GCA_008791945.1	6864	1	Human rhinovirus C35	NA	Viruses; Riboviria; Orthornavirae; Pisuviricota; Pisoniviricetes; Picornavirales; Picornaviridae; Enterovirus; Rhinovirus C
GCA_000840305.1	18630	17	Bacillus virus B103	NA	Viruses; dsDNA viruses, no RNA stage; Caudovirales; Podoviridae; Picovirinae; Phi29virus
GCA_001605815.1	2984	1	Phytophthora infestans RNA virus 4	NA	Viruses; ssRNA viruses; ssRNA positive-strand viruses, no DNA stage; Narnaviridae; unclassified Narnaviridae
GCA_000850905.1	10296	6	Sorghum chlorotic spot virus	NA	Viruses; ssRNA viruses; ssRNA positive-strand viruses, no DNA stage; Virgaviridae; Furovirus
GCA_000837625.1	5517	4	Bovine parvovirus	NA	Viruses; ssDNA viruses; Parvoviridae; Parvovirinae; Bocaparvovirus; Ungulate bocaparvovirus 1
GCA_000848925.1	5323	3	Canine parvovirus	CPVN	Viruses; ssDNA viruses; Parvoviridae; Parvovirinae; Protoparvovirus; Carnivore protoparvovirus 1; Feline panleukopenia virus
GCA_000838465.1	5149	7	Minute virus of mice	MVMp	Viruses; ssDNA viruses; Parvoviridae; Parvovirinae; Protoparvovirus; Rodent protoparvovirus 1
GCA_000839645.1	5596	6	Human parvovirus B19	NA	Viruses; ssDNA viruses; Parvoviridae; Parvovirinae; Erythroparvovirus; Primate erythroparvovirus 1
GCA_000916655.1	42594	69	Mycobacterium phage Redi	NA	Viruses; dsDNA viruses, no RNA stage; Caudovirales; Siphoviridae; Charlievirus
GCA_000917515.1	155586	236	Mycobacterium phage Pleione	NA	Viruses; dsDNA viruses, no RNA stage; Caudovirales; Myoviridae; Bxz1virus; unclassified Bxz1virus
GCA_002601585.1	67169	88	Mycobacterium phage Arbiter	NA	Viruses; Duplodnaviria; Heunggongvirae; Uroviricota; Caudoviricetes; Caudovirales; Siphoviridae; Bclasvirinae; Rosebushvirus; unclassified Rosebushvirus
GCA_000848765.1	5176	2	H1 parvovirus	NA	Viruses; Monodnaviria; Shotokuvirae; Cossaviricota; Quintoviricetes; Piccovirales; Parvoviridae; Parvovirinae; Protoparvovirus; Rodent protoparvovirus 1
GCA_000902315.1	176043	225	Synechococcus phage ACG2014c	NA	Viruses; dsDNA viruses, no RNA stage; Caudovirales; Myoviridae; Tevenvirinae; T4virus; unclassified T4virus
GCA_000901695.1	172879	234	Synechococcus phage metaGMbCM1	NA	Viruses; dsDNA viruses, no RNA stage; Caudovirales; Myoviridae; Tevenvirinae; T4virus; unclassified T4virus
GCA_000838645.1	4679	8	Adenoassociated virus 2	NA	Viruses; ssDNA viruses; Parvoviridae; Parvovirinae; Dependoparvovirus; Adeno-associated dependoparvovirus A
GCA_000893015.1	4152	4	Soybean yellow common mosaic virus	NA	Viruses; ssRNA viruses; ssRNA positive-strand viruses, no DNA stage; Sobemovirus; unclassified Sobemovirus
GCA_000895595.1	9626	1	Keunjorong mosaic virus	NA	Viruses; ssRNA viruses; ssRNA positive-strand viruses, no DNA stage; Potyviridae; unclassified Potyviridae
GCA_000893035.1	157950	201	Salmonella phage SFP10	NA	Viruses; dsDNA viruses, no RNA stage; Caudovirales; Myoviridae; Vi1virus
GCA_006444975.1	35158	45	Human mastadenovirus B	humanCHNa23820117P7H7F7	Viruses; dsDNA viruses, no RNA stage; Adenoviridae; Mastadenovirus
GCA_000892995.1	7048	7	Morelia spilota papillomavirus 1	NA	Viruses; dsDNA viruses, no RNA stage; Papillomaviridae; Dyomupapillomavirus; Dyomupapillomavirus 1
GCA_000894855.1	7784	7	Canis familiaris papillomavirus 8	NA	Viruses; dsDNA viruses, no RNA stage; Papillomaviridae; Chipapillomavirus; Chipapillomavirus 3

gca	genome.size	proteome.size	phage.name	strain	Taxonomy
GCA_000847225.1	5217	6	Abutilon mosaic virus	NA	Viruses; ssDNA viruses; Geminiviridae; Begomovirus
GCA_000891355.1	6495	5	Tamus red mosaic virus	NA	Viruses; ssRNA viruses; ssRNA positive-strand viruses, no DNA stage; Tymovirales; Alphaflexiviridae; Potexvirus
GCA_000839545.1	2750	3	Chloris striate mosaic virus	NA	Viruses; ssDNA viruses; Geminiviridae; Mastrevirus
GCA_002825065.1	2683	3	Maize streak virus	NA	Viruses; ssDNA viruses; Geminiviridae; Mastrevirus
GCA_002825265.1	2714	3	Panicum streak virus	NA	Viruses; ssDNA viruses; Geminiviridae; Mastrevirus
GCA_000842985.1	5117	7	Potato yellow mosaic virus	PYMVTT Trinidad and Tobago strain	Viruses; ssDNA viruses; Geminiviridae; Begomovirus
GCA_000837705.1	5241	6	Squash leaf curl virus	NA	Viruses; ssDNA viruses; Geminiviridae; Begomovirus
GCA_000840085.1	2580	4	Tobacco yellow dwarf virus	NA	Viruses; ssDNA viruses; Geminiviridae; Mastrevirus
GCA_002824685.1	2770	6	Tomato yellow leaf curl virus	NA	Viruses; ssDNA viruses; Geminiviridae; Begomovirus
GCA_002825495.1	2733	4	Wheat dwarf virus	NA	Viruses; ssDNA viruses; Geminiviridae; Mastrevirus
GCA_002825445.1	2745	3	Sugarcane streak virus	NA	Viruses; ssDNA viruses; Geminiviridae; Mastrevirus
GCA_000838685.1	2701	4	Digitaria streak virus	NA	Viruses; ssDNA viruses; Geminiviridae; Mastrevirus
GCA_000838545.1	5191	6	Bean dwarf mosaic virus	NA	Viruses; ssDNA viruses; Geminiviridae; Begomovirus
GCA_002821705.1	2616	5	Bean golden mosaic virus	NA	Viruses; ssDNA viruses; Geminiviridae; Begomovirus
GCA_002824825.1	2927	8	Beet curly top virus	NA	Viruses; ssDNA viruses; Geminiviridae; Curtovirus
GCA_000840785.1	5577	11	Escherichia phage G4	NA	Viruses; Monodnaviria; Sangervirae; Phixviricota; Malgrandaviricetes; Petitvirales; Microviridae; Bullavirinae; Gequatrovirus; Escherichia virus G4
GCA_002596505.1	5386	12	Enterobacteria phage S13	NA	Viruses; Monodnaviria; Sangervirae; Phixviricota; Malgrandaviricetes; Petitvirales; Microviridae; Bullavirinae; Sinsheimervirus; unclassified Sinsheimervirus; Enterobacteria phage phiX174 sensu lato
GCA_000884975.1	6094	11	Escherichia phage St1	NA	Viruses; Monodnaviria; Sangervirae; Phixviricota; Malgrandaviricetes; Petitvirales; Microviridae; Bullavirinae; Alphatrevirus; Escherichia virus St1
GCA_008605865.1	5386	12	Escherichia virus phiX174	NA	Viruses; Monodnaviria; Sangervirae; Phixviricota; Malgrandaviricetes; Petitvirales; Microviridae; Bullavirinae; Sinsheimervirus
GCA_000893895.1	5278	4	Porcine bocavirus 3	SH20F	Viruses; ssDNA viruses; Parvoviridae; Parvovirinae; Bocaparvovirus; Ungulate bocaparvovirus 5
GCA_000891375.1	5177	4	Porcine bocavirus 41	SH17N1	Viruses; ssDNA viruses; Parvoviridae; Parvovirinae; Bocaparvovirus; Ungulate bocaparvovirus 5
GCA_000917535.1	497513	675	Bacillus virus G	NA	Viruses; dsDNA viruses, no RNA stage; Caudovirales; Myoviridae; unassigned Myoviridae
GCA_002630565.1	68600	98	Mycobacterium phage Murdoc	NA	Viruses; Duplodnaviria; Heunggongvirae; Uroviricota; Caudoviricetes; Caudovirales; Siphoviridae; Bclasvirinae; Pegunavirus; Mycobacterium virus Soto
GCA_002600245.1	68307	99	Mycobacterium phage Morgushi	NA	Viruses; Duplodnaviria; Heunggongvirae; Uroviricota; Caudoviricetes; Caudovirales; Siphoviridae; Bclasvirinae; Pegunavirus; Mycobacterium virus Soto
GCA_000918555.1	59672	95	Mycobacterium phage BarrelRoll	NA	Viruses; dsDNA viruses, no RNA stage; Caudovirales; Siphoviridae; unclassified Siphoviridae
GCA_000846145.1	6087	10	Escherichia phage alpha3	NA	Viruses; Monodnaviria; Sangervirae; Phixviricota; Malgrandaviricetes; Petitvirales; Microviridae; Bullavirinae; Alphatrevirus; Escherichia virus alpha3
GCA_000847925.1	8273	12	Spiroplasma phage R8A2B	NA	Viruses; Monodnaviria; Loebvirae; Hofneiviricota; Faserviricetes; Tubulavirales; Plectroviridae; Vespertiliovirus; Spiroplasma virus R8A2B
GCA_000839985.1	4421	1	Spiroplasma virus SpV4	NA	Viruses; Monodnaviria; Sangervirae; Phixviricota; Malgrandaviricetes; Petitvirales; Microviridae; Gokushovirinae; Spiromicrovirus
GCA_002824505.1	2612	5	Tomato severe leaf curl virus Huasteca	NA	Viruses; Monodnaviria; Shotokuvirae; Cressdnaviricota; Repensiviricetes; Geplafuvirales; Geminiviridae; Begomovirus; Tomato severe leaf curl virus
GCA_000842465.1	7308	1	Xanthomonas virus Cf1c	NA	Viruses; Monodnaviria; Loebvirae; Hofneiviricota; Faserviricetes; Tubulavirales; Inoviridae; Coriovirus
GCA_000929915.1	6407	10	Enterobacteria phage f1	NA	Viruses; Monodnaviria; Loebvirae; Hofneiviricota; Faserviricetes; Tubulavirales; Inoviridae; Inovirus; Escherichia virus M13
GCA_000930555.1	6408	10	Enterobacteria phage fd	478	Viruses; ssDNA viruses; Inoviridae; Inovirus
GCA_000848985.1	6883	10	Salmonella phage IKe	NA	Viruses; Monodnaviria; Loebvirae; Hofneiviricota; Faserviricetes; Tubulavirales; Inoviridae; Lineavirus; Salmonella virus IKe
GCA_000917555.1	51083	94	Mycobacterium phage Dreamboat	NA	Viruses; dsDNA viruses, no RNA stage; Caudovirales; Siphoviridae; unclassified Siphoviridae

gca	genome.size	proteome.size	phage.name	strain	Taxonomy
GCA_000917495.1	69279	110	Mycobacterium virus Rumpelstiltskin	NA	Viruses; Duplodnaviria; Heunggongvirae; Uroviricota; Caudoviricetes; Caudovirales; Siphoviridae; Bronvirus
GCA_000836925.1	8454	10	Escherichia phage If1	NA	Viruses; Monodnaviria; Loebvirae; Hofneiviricota; Faserviricetes; Tubulavirales; Inoviridae; Infulavirus; Escherichia virus If1
GCA_000918535.1	52481	90	Mycobacterium phage Violet	NA	Viruses; dsDNA viruses, no RNA stage; Caudovirales; Siphoviridae; L5virus
GCA_000847885.1	6744	9	Escherichia virus I22	NA	Viruses; Monodnaviria; Loebvirae; Hofneiviricota; Faserviricetes; Tubulavirales; Inoviridae; Lineavirus
GCA_000905675.1	10855	4	Aspergillus foetidus dsRNA mycovirus	NA	Viruses; dsRNA viruses; unclassified dsRNA viruses
GCA_002988045.1	5194	2	Aspergillus foetidus slow virus 1	NA	Viruses; Riboviria; Orthornavirae; Duplornaviricota; Chrymotiviricetes; Ghabrivirales; Totiviridae; Victorivirus
GCA_000894895.1	7873	7	Canis familiaris papillomavirus 9	NA	Viruses; Monodnaviria; Shotokuvirae; Cossaviricota; Papovaviricetes; Zurhausenvirales; Papillomaviridae; Firstpapillomavirinae; Chipapillomavirus; Chipapillomavirus 1
GCA_000896375.1	7774	7	Canis familiaris papillomavirus 10	NA	Viruses; Monodnaviria; Shotokuvirae; Cossaviricota; Papovaviricetes; Zurhausenvirales; Papillomaviridae; Firstpapillomavirinae; Chipapillomavirus; Chipapillomavirus 3
GCA_000837805.1	5833	9	Pseudomonas phage Pf3	NA	Viruses; ssDNA viruses; Inoviridae; Inovirus
GCA_002630765.1	83331	128	Salmonella phage FO1a	NA	Viruses; Duplodnaviria; Heunggongvirae; Uroviricota; Caudoviricetes; Caudovirales; Myoviridae; Ounavirinae; Felixounavirus; Salmonella virus FelixO1
GCA_000905195.1	160221	268	Salmonella phage vB_SenMS16	NA	Viruses; dsDNA viruses, no RNA stage; Caudovirales; Myoviridae; Tevenvirinae; S16virus; Salmonella virus S16
GCA_000852125.1	13385	13	Pseudomonas virus phi6	NA	Viruses; Riboviria; Orthornavirae; Duplornaviricota; Vidaverviricetes; Mindivirales; Cystoviridae; Cystovirus
GCA_006298385.1	23570	10	Mammalian orthoreovirus 3 Dearing	T3D	Viruses; Riboviria; Orthornavirae; Duplornaviricota; Resentoviricetes; Reovirales; Reoviridae; Spinareovirinae; Orthoreovirus; Mammalian orthoreovirus; Mammalian orthoreovirus 3
GCA_002624025.3	80228	132	Mycobacterium phage Bongo	NA	Viruses; Duplodnaviria; Heunggongvirae; Uroviricota; Caudoviricetes; Caudovirales; Siphoviridae; Mclasvirinae; Bongovirus; Mycobacterium virus Bongo
GCA_000918815.1	154573	228	Mycobacterium phage MoMoMixon	NA	Viruses; dsDNA viruses, no RNA stage; Caudovirales; Myoviridae; Bxz1virus; unclassified Bxz1virus
GCA_000917815.1	156646	232	Mycobacterium phage Nappy	NA	Viruses; dsDNA viruses, no RNA stage; Caudovirales; Myoviridae; Bxz1virus; unclassified Bxz1virus
GCA_002598465.1	155299	228	Mycobacterium phage Wally	NA	Viruses; Duplodnaviria; Heunggongvirae; Uroviricota; Caudoviricetes; Caudovirales; Myoviridae; Bixzunavirus; Mycobacterium virus Bxz1
GCA_000895035.1	9565	2	Halastavi arva RNA virus	NA	Viruses; ssRNA viruses; ssRNA positive-strand viruses, no DNA stage; unclassified ssRNA positive-strand viruses
GCA_002600265.1	68133	100	Mycobacterium phage TallGrassMM	NA	Viruses; Duplodnaviria; Heunggongvirae; Uroviricota; Caudoviricetes; Caudovirales; Siphoviridae; Bclasvirinae; Pegunavirus; unclassified Pegunavirus
GCA_000919415.1	68494	101	Mycobacterium phage Vista	NA	Viruses; dsDNA viruses, no RNA stage; Caudovirales; Siphoviridae; unclassified Siphoviridae; PG1-like phages
GCA_000920295.1	68711	104	Mycobacterium phage Akoma	NA	Viruses; dsDNA viruses, no RNA stage; Caudovirales; Siphoviridae; unclassified Siphoviridae
GCA_000917775.1	53177	96	Mycobacterium virus Alma	NA	Viruses; Duplodnaviria; Heunggongvirae; Uroviricota; Caudoviricetes; Caudovirales; Siphoviridae; Fromanvirus
GCA_002623405.1	67436	92	Mycobacterium phage Ares	NA	Viruses; Duplodnaviria; Heunggongvirae; Uroviricota; Caudoviricetes; Caudovirales; Siphoviridae; Bclasvirinae; Rosebushvirus; Mycobacterium virus Godines
GCA_002623425.1	69409	105	Mycobacterium phage Athena	NA	Viruses; Duplodnaviria; Heunggongvirae; Uroviricota; Caudoviricetes; Caudovirales; Siphoviridae; Bclasvirinae; Pipefishvirus; Mycobacterium virus Athena
GCA_002601705.1	41901	62	Mycobacterium phage Avrafan	NA	Viruses; Duplodnaviria; Heunggongvirae; Uroviricota; Caudoviricetes; Caudovirales; Siphoviridae; Liefievirus; unclassified Liefievirus
GCA_000920275.1	48420	78	Mycobacterium virus Babsiella	NA	Viruses; Duplodnaviria; Heunggongvirae; Uroviricota; Caudoviricetes; Caudovirales; Siphoviridae; Chebruvirinae; Brujitavirus
GCA_000917795.1	51821	86	Mycobacterium virus Billknuckles	NA	Viruses; Duplodnaviria; Heunggongvirae; Uroviricota; Caudoviricetes; Caudovirales; Siphoviridae; Fromanvirus
GCA_000919395.1	52288	103	Mycobacterium phage Blue7	NA	Viruses; dsDNA viruses, no RNA stage; Caudovirales; Siphoviridae; unclassified Siphoviridae
GCA_000919435.1	53003	96	Mycobacterium virus Bruns	NA	Viruses; Duplodnaviria; Heunggongvirae; Uroviricota; Caudoviricetes; Caudovirales; Siphoviridae; Fromanvirus
GCA_000918795.1	110569	241	Mycobacterium virus Courthouse	NA	Viruses; Duplodnaviria; Heunggongvirae; Uroviricota; Caudoviricetes; Caudovirales; Siphoviridae; Omegavirus
GCA_000918775.1	56461	106	Mycobacterium virus Deadp	NA	Viruses; Duplodnaviria; Heunggongvirae; Uroviricota; Caudoviricetes; Caudovirales; Siphoviridae; Cheoctovirus
GCA_000920255.1	64613	94	Mycobacterium phage Dori	NA	Viruses; dsDNA viruses, no RNA stage; Caudovirales; Siphoviridae; unclassified Siphoviridae
GCA_000917755.1	67949	102	Mycobacterium phage Gadjet	NA	Viruses; dsDNA viruses, no RNA stage; Caudovirales; Siphoviridae; Pg1virus
GCA_002633745.1	67451	92	Mycobacterium phage Hedgerow	NA	Viruses; Duplodnaviria; Heunggongvirae; Uroviricota; Caudoviricetes; Caudovirales; Siphoviridae; Bclasvirinae; Rosebushvirus; Mycobacterium virus Rosebush

gca	genome.size	proteome.size	phage.name	strain	Taxonomy
GCA_002601765.1	68839	104	Mycobacterium phage IsaacEli	NA	Viruses; Duplodnaviria; Heunggongvirae; Uroviricota; Caudoviricetes; Caudovirales; Siphoviridae; Bclasvirinae; Pegunavirus; Mycobacterium virus Pg1
GCA_002601725.1	48963	92	Mycobacterium virus Jeffabunny	NA	Viruses; Duplodnaviria; Heunggongvirae; Uroviricota; Caudoviricetes; Caudovirales; Siphoviridae; Fromanvirus
GCA_002601745.1	68633	103	Mycobacterium phage Kamiyu	NA	Viruses; Duplodnaviria; Heunggongvirae; Uroviricota; Caudoviricetes; Caudovirales; Siphoviridae; Bclasvirinae; Pipefishvirus; unclassified Pipefishvirus
GCA_001505895.1	68839	104	Mycobacterium phage Kikipoo	NA	Viruses; dsDNA viruses, no RNA stage; Caudovirales; Siphoviridae; unclassified Siphoviridae; PG1-like phages; Mycobacterium phage Badfish
GCA_000918755.1	52379	95	Mycobacterium virus Kugel	NA	Viruses; Duplodnaviria; Heunggongvirae; Uroviricota; Caudoviricetes; Caudovirales; Siphoviridae; Fromanvirus
GCA_002601785.1	51813	91	Mycobacterium virus LHTSCC	NA	Viruses; Duplodnaviria; Heunggongvirae; Uroviricota; Caudoviricetes; Caudovirales; Siphoviridae; Fromanvirus
GCA_002601805.1	65108	88	Mycobacterium phage Nova	NA	Viruses; Duplodnaviria; Heunggongvirae; Uroviricota; Caudoviricetes; Caudovirales; Siphoviridae; Dclasvirinae; Plotvirus; Mycobacterium virus Plot
GCA_002598485.1	156758	238	Mycobacterium phage Pio	NA	Viruses; Duplodnaviria; Heunggongvirae; Uroviricota; Caudoviricetes; Caudovirales; Myoviridae; Bixzunavirus; Mycobacterium virus Bxz1
GCA_000917715.1	59016	105	Mycobacterium virus SG4	NA	Viruses; Duplodnaviria; Heunggongvirae; Uroviricota; Caudoviricetes; Caudovirales; Siphoviridae; Cheoctovirus
GCA_002755115.1	68992	103	Mycobacterium phage ThreeOh3D2	NA	Viruses; Duplodnaviria; Heunggongvirae; Uroviricota; Caudoviricetes; Caudovirales; Siphoviridae; Bclasvirinae; Pegunavirus; Mycobacterium virus Pg1
GCA_000918735.1	71341	128	Mycobacterium virus Firecracker	NA	Viruses; Duplodnaviria; Heunggongvirae; Uroviricota; Caudoviricetes; Caudovirales; Siphoviridae; Corndogvirus
GCA_000919375.1	68311	102	Mycobacterium phage JacAttac	NA	Viruses; dsDNA viruses, no RNA stage; Caudovirales; Siphoviridae; unclassified Siphoviridae; PG1-like phages
GCA_000918675.1	69641	95	Mycobacterium phage Stinger	NA	Viruses; dsDNA viruses, no RNA stage; Caudovirales; Siphoviridae; Pg1virus
GCA_000895635.1	8159	1	Quail picornavirus QPV1HUN2010	NA	Viruses; ssRNA viruses; ssRNA positive-strand viruses, no DNA stage; Picornavirales; Picornaviridae; unclassified Picornaviridae
GCA_002823985.1	2617	5	Tomato interveinal chlorosis virus	NA	Viruses; Monodnaviria; Shotokuvirae; Cressdnaviricota; Repensiviricetes; Geplafuvirales; Geminiviridae; Begomovirus
GCA_003085395.1	19509	11	African horse sickness virus 9	NA	Viruses; Riboviria; Orthornavirae; Duplornaviricota; Resentoviricetes; Reovirales; Reoviridae; Sedoreovirinae; Orbivirus; African horse sickness virus
GCA_000903395.1	39646	27	Enterobacteria phage IME10	NA	Viruses; dsDNA viruses, no RNA stage; Caudovirales; Podoviridae; unclassified Podoviridae
GCA_001042275.1	240413	264	Salmonella phage SPN3US	NA	Viruses; dsDNA viruses, no RNA stage; Caudovirales; Myoviridae; unclassified Myoviridae
GCA_003080935.1	19130	13	Bluetongue virus 17	17BRA201473	Viruses; Riboviria; Orthornavirae; Duplornaviricota; Resentoviricetes; Reovirales; Reoviridae; Sedoreovirinae; Orbivirus; Bluetongue virus
GCA_003078775.1	19184	11	Bluetongue virus 10	NA	Viruses; Riboviria; Orthornavirae; Duplornaviricota; Resentoviricetes; Reovirales; Reoviridae; Sedoreovirinae; Orbivirus; Bluetongue virus
GCA_000903075.1	152427	201	Dickeya virus Limestone	NA	Viruses; dsDNA viruses, no RNA stage; Caudovirales; Myoviridae; Vi1virus
GCA_003179175.1	7828	8	Canis familiaris papillomavirus 11	NA	Viruses; Monodnaviria; Shotokuvirae; Cossaviricota; Papovaviricetes; Zurhausenvirales; Papillomaviridae; Firstpapillomavirinae; Chipapillomavirus; Chipapillomavirus 1
GCA_006443855.1	35127	37	Human adenovirus 62	NA	Viruses; Varidnaviria; Bamfordvirae; Preplasmiviricota; Tectiliviricetes; Rowavirales; Adenoviridae; Mastadenovirus; Human mastadenovirus D
GCA_006443835.1	35103	37	Human adenovirus 60a	NA	Viruses; Varidnaviria; Bamfordvirae; Preplasmiviricota; Tectiliviricetes; Rowavirales; Adenoviridae; Mastadenovirus; Human mastadenovirus D
GCA_003087355.1	18298	11	Porcine rotavirus	NA	Viruses; Riboviria; Orthornavirae; Duplornaviricota; Resentoviricetes; Reovirales; Reoviridae; Sedoreovirinae; Rotavirus; Rotavirus C
GCA_001040795.1	36814	54	Pseudomonas virus LPB1	NA	Viruses; Duplodnaviria; Heunggongvirae; Uroviricota; Caudoviricetes; Caudovirales; Siphoviridae; Casadabanvirus
GCA_002650415.1	8907	11	Bovine rotavirus	KJ11	Viruses; Riboviria; Orthornavirae; Duplornaviricota; Resentoviricetes; Reovirales; Reoviridae; Sedoreovirinae; Rotavirus; Rotavirus A; Bovine rotavirus A; Bovine rotavirus A unknown G-type
GCA_000850365.1	12035	5	Equine foamy virus	NA	Viruses; Riboviria; Pararnavirae; Artverviricota; Revtraviricetes; Ortervirales; Retroviridae; Spumaretrovirinae; Equispumavirus
GCA_002601905.1	37374	55	Pseudomonas virus FHA0480	F_HA0480spPa1651	Viruses; Duplodnaviria; Heunggongvirae; Uroviricota; Caudoviricetes; Caudovirales; Siphoviridae; Casadabanvirus
GCA_002827185.1	4441	5	Gray fox amdovirus	NA	Viruses; Monodnaviria; Shotokuvirae; Cossaviricota; Quintoviricetes; Piccovirales; Parvoviridae; Parvovirinae; Amdoparvovirus; Carnivore amdoparvovirus 2
GCA_009731615.1	5975	4	Cholul virus	CHLVMex07	Viruses; Riboviria; Orthornavirae; Negarnaviricota; Polyploviricotina; Ellioviricetes; Bunyavirales; Peribunyaviridae; Orthobunyavirus; Bunyamwera orthobunyavirus
GCA_002601925.1	37840	61	Pseudomonas phage JBD26	NA	Viruses; Duplodnaviria; Heunggongvirae; Uroviricota; Caudoviricetes; Caudovirales; Siphoviridae; Casadabanvirus; unclassified Casadabanvirus
GCA_000893055.1	8315	6	Mirabilis jalapa mottle virus	NA	Viruses; ssRNA viruses; ssRNA positive-strand viruses, no DNA stage; Tymovirales; Betaflexiviridae; Quinvirinae; Carlavirus
GCA_000916535.1	6500	3	Porcine astrovirus 5	NA	Viruses; ssRNA viruses; ssRNA positive-strand viruses, no DNA stage; Astroviridae; unclassified Astroviridae

gca	genome.size	proteome.size	phage.name	strain	Taxonomy
GCA_000896395.1	5222	7	Sida yellow mottle virus	CubaSancti Spiritus15912009	Viruses; ssDNA viruses; Geminiviridae; Begomovirus
GCA_002639295.1	18484	12	Human rotavirus A	CAU12251	Viruses; Riboviria; Orthornavirae; Duplornaviricota; Resentoviricetes; Reovirales; Reoviridae; Sedoreovirinae; Rotavirus; Rotavirus A
GCA_000907835.1	17932	13	Human rotavirus B	Bang373	Viruses; dsRNA viruses; Reoviridae; Sedoreovirinae; Rotavirus; Rotavirus B
GCA_000893955.1	14153	7	Blueberry necrotic ring blotch virus	Georgia	Viruses; ssRNA viruses; ssRNA positive-strand viruses, no DNA stage; unclassified ssRNA positive-strand viruses
GCA_000864225.1	17910	11	Human rotavirus C	NA	Viruses; Riboviria; Orthornavirae; Duplornaviricota; Resentoviricetes; Reovirales; Reoviridae; Sedoreovirinae; Rotavirus; Rotavirus C
GCA_006446455.1	35172	39	Human adenovirus 65	BGD2004B172P58H10F9	Viruses; Varidnaviria; Bamfordvirae; Preplasmiviricota; Tectiliviricetes; Rowavirales; Adenoviridae; Mastadenovirus; Human mastadenovirus D
GCA_003106745.1	9352	1	Hepatitis C virus subtype 3i	NA	Viruses; Riboviria; Orthornavirae; Kitrinoviricota; Flasuviricetes; Amarillovirales; Flaviviridae; Hepacivirus; Hepacivirus C; Hepatitis C virus genotype 3
GCA_003107105.1	9317	1	Hepatitis C virus subtype 4L	NA	Viruses; Riboviria; Orthornavirae; Kitrinoviricota; Flasuviricetes; Amarillovirales; Flaviviridae; Hepacivirus; Hepacivirus C; Hepatitis C virus genotype 4
GCA_003107025.1	9314	1	Hepatitis C virus subtype 4g	NA	Viruses; Riboviria; Orthornavirae; Kitrinoviricota; Flasuviricetes; Amarillovirales; Flaviviridae; Hepacivirus; Hepacivirus C; Hepatitis C virus genotype 4
GCA_003107145.1	9305	1	Hepatitis C virus subtype 4m	NA	Viruses; Riboviria; Orthornavirae; Kitrinoviricota; Flasuviricetes; Amarillovirales; Flaviviridae; Hepacivirus; Hepacivirus C; Hepatitis C virus genotype 4
GCA_003106405.1	9383	1	Hepatitis C virus subtype 2m	NA	Viruses; Riboviria; Orthornavirae; Kitrinoviricota; Flasuviricetes; Amarillovirales; Flaviviridae; Hepacivirus; Hepacivirus C; Hepatitis C virus genotype 2
GCA_002827545.1	5249	3	Ovine hokovirus	HKS01	Viruses; Monodnaviria; Shotokuvirae; Cossaviricota; Quintoviricetes; Piccovirales; Parvoviridae; Parvovirinae; Tetraparvovirus; Ungulate tetraparvovirus 4
GCA_002660075.1	16768	12	Porcine rotavirus A	RVAPigwtKNAET8B2015G5P13	Viruses; Riboviria; Orthornavirae; Duplornaviricota; Resentoviricetes; Reovirales; Reoviridae; Sedoreovirinae; Rotavirus; Rotavirus A
GCA_006446135.1	35538	41	Human adenovirus 68	Arg 82704	Viruses; Varidnaviria; Bamfordvirae; Preplasmiviricota; Tectiliviricetes; Rowavirales; Adenoviridae; Mastadenovirus; Human mastadenovirus B; Human adenovirus B1
GCA_003087275.1	17631	11	Porcine rotavirus C	RVCPigwtIshi12015G13P4	Viruses; Riboviria; Orthornavirae; Duplornaviricota; Resentoviricetes; Reovirales; Reoviridae; Sedoreovirinae; Rotavirus; Rotavirus C
GCA_002601945.1	55363	98	Mycobacterium virus Dotproduct	NA	Viruses; Duplodnaviria; Heunggongvirae; Uroviricota; Caudoviricetes; Caudovirales; Siphoviridae; Cheoctovirus
GCA_001041915.1	58076	94	Mycobacterium virus Fionnbarth	NA	Viruses; Duplodnaviria; Heunggongvirae; Uroviricota; Caudoviricetes; Caudovirales; Siphoviridae; Timquatrovirus
GCA_002601965.1	49228	95	Mycobacterium virus Saintus	NA	Viruses; Duplodnaviria; Heunggongvirae; Uroviricota; Caudoviricetes; Caudovirales; Siphoviridae; Fromanvirus
GCA_000870625.1	25709	12	Rice gall dwarf virus	NA	Viruses; dsRNA viruses; Reoviridae; Sedoreovirinae; Phytoreovirus
GCA_001461385.2	29144	13	Maize rough dwarf virus	NA	Viruses; Riboviria; Orthornavirae; Duplornaviricota; Resentoviricetes; Reovirales; Reoviridae; Spinareovirinae; Fijivirus
GCA_000852945.1	29141	13	Rice black streaked dwarf virus	NA	Viruses; dsRNA viruses; Reoviridae; Spinareovirinae; Fijivirus; Fijivirus group 2
GCA_000850725.1	24839	12	Rice dwarf virus	NA	Viruses; dsRNA viruses; Reoviridae; Sedoreovirinae; Phytoreovirus
GCA_000855485.1	5898	3	Infectious bursal disease virus	Very virulent strain	Viruses; dsRNA viruses; Birnaviridae; Avibirnavirus
GCA_015224295.1	252940	193	Diachasmimorpha longicaudata entomopoxvirus	NA	Viruses; Varidnaviria; Bamfordvirae; Nucleocytoviricota; Pokkesviricetes; Chitovirales; Poxviridae; Entomopoxvirinae; Diachasmimorpha entomopoxvirus
GCA_000856525.1	5881	3	Infectious pancreatic necrosis virus	NA	Viruses; dsRNA viruses; Birnaviridae; Aquabirnavirus
GCA_000895915.1	40058	46	Pseudomonas phage Bf7	NA	Viruses; dsDNA viruses, no RNA stage; Caudovirales; Podoviridae; unclassified Podoviridae
GCA_000852145.1	4579	3	Saccharomyces cerevisiae virus LA	NA	Viruses; dsRNA viruses; Totiviridae; Totivirus
GCA_000894035.1	74431	103	Gordonia phage GTE7	NA	Viruses; dsDNA viruses, no RNA stage; Caudovirales; Siphoviridae; unclassified Siphoviridae
GCA_006446075.1	35075	39	Human adenovirus 67	BGD2005B109P67H9F67	Viruses; Varidnaviria; Bamfordvirae; Preplasmiviricota; Tectiliviricetes; Rowavirales; Adenoviridae; Mastadenovirus; Human mastadenovirus D
GCA_008797915.1	8193	1	Footandmouth disease virus type Asia 1	ZBCHA58att	Viruses; Riboviria; Orthornavirae; Pisuviricota; Pisoniviricetes; Picornavirales; Picornaviridae; Aphthovirus; Foot-and-mouth disease virus
GCA_000847585.1	11488	2	Barmah Forest virus	BH2193	Viruses; ssRNA viruses; ssRNA positive-strand viruses, no DNA stage; Togaviridae; Alphavirus
GCA_000862705.1	11675	3	Eastern equine encephalitis virus	ssp North American variant	Viruses; ssRNA viruses; ssRNA positive-strand viruses, no DNA stage; Togaviridae; Alphavirus; EEEV complex
GCA_000921735.1	11550	2	Middelburg virus	NA	Viruses; ssRNA viruses; ssRNA positive-strand viruses, no DNA stage; Togaviridae; Alphavirus
GCA_000881255.1	11526	2	Highlands J virus	NA	Viruses; ssRNA viruses; ssRNA positive-strand viruses, no DNA stage; Togaviridae; Alphavirus; WEEV complex

gca	genome.size	proteome.size	phage.name	strain	Taxonomy
GCA_002889255.1	11948	2	Ross River virus	QML 1	Viruses; ssRNA viruses; ssRNA positive-strand viruses, no DNA stage; Togaviridae; Alphavirus; SFV complex
GCA_000860285.1	11442	2	Semliki Forest virus	NA	Viruses; ssRNA viruses; ssRNA positive-strand viruses, no DNA stage; Togaviridae; Alphavirus; SFV complex
GCA_000860545.1	11703	3	Sindbis virus	NA	Viruses; ssRNA viruses; ssRNA positive-strand viruses, no DNA stage; Togaviridae; Alphavirus; WEEV complex
GCA_000862105.1	11444	3	Venezuelan equine encephalitis virus	P676	Viruses; ssRNA viruses; ssRNA positive-strand viruses, no DNA stage; Togaviridae; Alphavirus; VEEV complex
GCA_000850885.1	11484	2	Western equine encephalitis virus	71V1658	Viruses; ssRNA viruses; ssRNA positive-strand viruses, no DNA stage; Togaviridae; Alphavirus; WEEV complex
GCA_000863025.1	9762	2	Rubella virus	FTherien	Viruses; ssRNA viruses; ssRNA positive-strand viruses, no DNA stage; Togaviridae; Rubivirus
GCA_000847165.1	39600	59	Yersinia phage phiYeO312	NA	Viruses; dsDNA viruses, no RNA stage; Caudovirales; Podoviridae; Autographivirinae; T7virus; unclassified T7-like viruses
GCA_000860865.1	12704	9	Equine arteritis virus	Bucyrus	Viruses; ssRNA viruses; ssRNA positive-strand viruses, no DNA stage; Nidovirales; Arteriviridae; Arterivirus
GCA_000850185.1	14104	8	Lactate dehydrogenaseelevating virus	Plagemann	Viruses; ssRNA viruses; ssRNA positive-strand viruses, no DNA stage; Nidovirales; Arteriviridae; Arterivirus
GCA_003971765.1	15111	8	Lelystad virus	NA	Viruses; Riboviria; Orthornavirae; Pisuviricota; Pisoniviricetes; Nidovirales; Arnidovirineae; Arteriviridae; Variarterivirinae; Betaarterivirus; Eurpobartevirus; Betaarterivirus suid 1; Porcine reproductive and respiratory syndrome virus 1
GCA_000900055.1	8344	7	Crocuta crocuta papillomavirus 1	NA	Viruses; dsDNA viruses, no RNA stage; Papillomaviridae; Lambdapapillomavirus; Lambdapapillomavirus 5
GCA_000895315.1	47159	68	Bacteroides phage B12414	NA	Viruses; dsDNA viruses, no RNA stage; Caudovirales; Siphoviridae; unclassified Siphoviridae
GCA_000896475.1	39283	58	Rhodobacter phage RcapMu	NA	Viruses; dsDNA viruses, no RNA stage; Caudovirales; Siphoviridae; unclassified Siphoviridae
GCA_000862125.1	10735	1	Dengue virus 1	Nauru Island Western Pacific	Viruses; ssRNA viruses; ssRNA positive-strand viruses, no DNA stage; Flaviviridae; Flavivirus; Dengue virus group; Dengue virus
GCA_000894015.1	7103	11	Vibrio phage VCYphi	NA	Viruses; ssDNA viruses; Inoviridae; Inovirus; unclassified Inovirus
GCA_000914875.1	6318	3	Porcine astrovirus 2	43USA	Viruses; ssRNA viruses; ssRNA positive-strand viruses, no DNA stage; Astroviridae; Mamastrovirus; unclassified Mamastrovirus
GCA_000916575.1	6639	3	Porcine astrovirus 4	35USA	Viruses; ssRNA viruses; ssRNA positive-strand viruses, no DNA stage; Astroviridae; Mamastrovirus; unclassified Mamastrovirus
GCA_000917395.1	9840	1	Posavirus 1	NA	Viruses; ssRNA viruses; ssRNA positive-strand viruses, no DNA stage; Picornavirales; unclassified Picornavirales
GCA_000916555.1	10220	1	Posavirus 2	NA	Viruses; ssRNA viruses; ssRNA positive-strand viruses, no DNA stage; Picornavirales; unclassified Picornavirales
GCA_000927735.1	3912	5	PoCircolike virus 21	NA	Viruses; ssDNA viruses; Circoviridae; unclassified Circoviridae; Po-Circo-like virus
GCA_003985445.1	3923	4	PoCircolike virus 22	NA	Viruses; Monodnaviria; Shotokuvirae; Cressdnaviricota; Arfiviricetes; Cirlivirales; Circoviridae; unclassified Circoviridae; Po-Circo-like virus
GCA_000929675.1	2904	3	PoCircolike virus 41	NA	Viruses; ssDNA viruses; Circoviridae; unclassified Circoviridae
GCA_000928635.1	2833	3	PoCircolike virus 51	NA	Viruses; ssDNA viruses; Circoviridae; unclassified Circoviridae
GCA_000866625.1	10707	1	Dengue virus 3	NA	Viruses; ssRNA viruses; ssRNA positive-strand viruses, no DNA stage; Flaviviridae; Flavivirus; Dengue virus group; Dengue virus
GCA_000865065.1	10649	1	Dengue virus 4	NA	Viruses; ssRNA viruses; ssRNA positive-strand viruses, no DNA stage; Flaviviridae; Flavivirus; Dengue virus group; Dengue virus
GCA_000862145.1	10976	1	Japanese encephalitis virus	JaOArS982	Viruses; ssRNA viruses; ssRNA positive-strand viruses, no DNA stage; Flaviviridae; Flavivirus; Japanese encephalitis virus group
GCA_004786635.1	10664	1	Kunjin virus	MRM61C	Viruses; Riboviria; Orthornavirae; Kitrinoviricota; Flasuviricetes; Amarillovirales; Flaviviridae; Flavivirus; West Nile virus
GCA_000863565.1	11014	1	Murray Valley encephalitis virus	MVE151	Viruses; ssRNA viruses; ssRNA positive-strand viruses, no DNA stage; Flaviviridae; Flavivirus; Japanese encephalitis virus group
GCA_000866785.1	10940	1	St Louis encephalitis virus	Kern217	Viruses; ssRNA viruses; ssRNA positive-strand viruses, no DNA stage; Flaviviridae; Flavivirus; Japanese encephalitis virus group
GCA_000861085.1	10962	1	West Nile virus	NA	Viruses; ssRNA viruses; ssRNA positive-strand viruses, no DNA stage; Flaviviridae; Flavivirus; Japanese encephalitis virus group
GCA_000860485.1	10839	1	Powassan virus	LB	Viruses; ssRNA viruses; ssRNA positive-strand viruses, no DNA stage; Flaviviridae; Flavivirus; tick-borne encephalitis virus group
GCA_000863125.1	11141	1	Tickborne encephalitis virus	Neudoerfl	Viruses; ssRNA viruses; ssRNA positive-strand viruses, no DNA stage; Flaviviridae; Flavivirus; tick-borne encephalitis virus group
GCA_000860805.1	10943	1	Langat virus	TP21	Viruses; ssRNA viruses; ssRNA positive-strand viruses, no DNA stage; Flaviviridae; Flavivirus; tick-borne encephalitis virus group
GCA_000863165.1	10871	1	Louping ill virus	369T2	Viruses; ssRNA viruses; ssRNA positive-strand viruses, no DNA stage; Flaviviridae; Flavivirus; tick-borne encephalitis virus group

gca	genome.size	proteome.size	phage.name	strain	Taxonomy
GCA_000894955.1	7391	1	Feline picornavirus	073F	Viruses; ssRNA viruses; ssRNA positive-strand viruses, no DNA stage; Picornavirales; Picornaviridae; unclassified Picornaviridae
GCA_000900855.1	157785	166	Salmonella phage Sh19	NA	Viruses; Duplodnaviria; Heunggongvirae; Uroviricota; Caudoviricetes; Caudovirales; Ackermannviridae; Cvivirinae; Kuttervirus; Salmonella virus SH19
GCA_000857725.1	10862	1	Yellow fever virus	17D vaccine strain	Viruses; ssRNA viruses; ssRNA positive-strand viruses, no DNA stage; Flaviviridae; Flavivirus; Yellow fever virus group
GCA_003047495.1	3033	3	Stork hepatitis B virus	NA	Viruses; Riboviria; Pararnavirae; Artverviricota; Revtraviricetes; Blubervirales; Hepadnaviridae; Avihepadnavirus; Heron hepatitis B virus
GCA_900039495.1	12104	1	Classical swine fever virus	NA	Viruses; ssRNA viruses; ssRNA positive-strand viruses, no DNA stage; Flaviviridae; Pestivirus
GCA_000896515.1	65766	94	Gordonia phage GRU1	NA	Viruses; dsDNA viruses, no RNA stage; Caudovirales; Siphoviridae; unclassified Siphoviridae
GCA_000895775.1	46140	68	Nocardia phage NBR1	NA	Viruses; dsDNA viruses, no RNA stage; Caudovirales; Siphoviridae; unclassified Siphoviridae
GCA_000895855.1	51342	85	Rhodococcus phage REQ1	NA	Viruses; dsDNA viruses, no RNA stage; Caudovirales; Siphoviridae; unclassified Siphoviridae
GCA_000895215.1	49330	82	Rhodococcus phage REQ2	NA	Viruses; dsDNA viruses, no RNA stage; Caudovirales; Siphoviridae; unclassified Siphoviridae
GCA_000894255.1	39474	60	Rhodococcus phage REQ3	NA	Viruses; unclassified phages
GCA_000896695.1	46586	66	Rhodococcus phage RER2	NA	Viruses; dsDNA viruses, no RNA stage; Caudovirales; Siphoviridae; L5virus
GCA_000894235.1	48072	66	Rhodococcus virus RGL3	NA	Viruses; Duplodnaviria; Heunggongvirae; Uroviricota; Caudoviricetes; Caudovirales; Siphoviridae
GCA_000895835.1	14270	20	Rhodococcus phage RRH1	NA	Viruses; dsDNA viruses, no RNA stage; Caudovirales; Siphoviridae; unclassified Siphoviridae
GCA_000895135.1	44077	57	Shigella phage EP23	NA	Viruses; dsDNA viruses, no RNA stage; Caudovirales; Siphoviridae; Hk578virus; Shigella virus EP23
GCA_900167835.1	12164	1	Bovine viral diarrhea virus 1	NA	Viruses; ssRNA viruses; ssRNA positive-strand viruses, no DNA stage; Flaviviridae; Pestivirus
GCA_000861245.1	12573	1	Bovine viral diarrhea virus 1NADL	NA	Viruses; Riboviria; Orthornavirae; Kitrinoviricota; Flasuviricetes; Amarillovirales; Flaviviridae; Pestivirus; Pestivirus A
GCA_002820845.1	9413	1	Hepatitis C virus	J	Viruses; ssRNA viruses; ssRNA positive-strand viruses, no DNA stage; Flaviviridae; Hepacivirus
GCA_000895115.1	132662	149	Campylobacter virus CPX	NA	Viruses; dsDNA viruses, no RNA stage; Caudovirales; Myoviridae; Eucampyvirinae; Cp8virus
GCA_003179215.1	7240	7	human papillomavirus 153	NA	Viruses; Monodnaviria; Shotokuvirae; Cossaviricota; Papovaviricetes; Zurhausenvirales; Papillomaviridae; Firstpapillomavirinae; Gammapapillomavirus; Gammapapillomavirus 13
GCA_000904975.1	30024	30	Bovine adenovirus 6	671130	Viruses; dsDNA viruses, no RNA stage; Adenoviridae; Atadenovirus; unclassified Atadenovirus; Bovine adenovirus E
GCA_000895015.1	10347	1	Spodoptera exigua iflavirus 1	NA	Viruses; ssRNA viruses; ssRNA positive-strand viruses, no DNA stage; Picornavirales; Iflaviridae; Iflavirus
GCA_000862965.1	27608	10	Infectious bronchitis virus	Beaudette	Viruses; ssRNA viruses; ssRNA positive-strand viruses, no DNA stage; Nidovirales; Coronaviridae; Coronavirinae; Gammacoronavirus; Avian coronavirus
GCA_000915275.1	1364	1	Papaya leaf curl alphasatellite	NA	Viruses; Satellites; Satellite Nucleic Acids; Single stranded DNA satellites; Alphasatellites; Begomovirus-associated alphasatellites; unclassified Begomovirus-associated alphasatellites
GCA_002755135.1	168394	271	Enterobacteria phage RB5	NA	Viruses; Duplodnaviria; Heunggongvirae; Uroviricota; Caudoviricetes; Caudovirales; Myoviridae; Tevenvirinae; Tequatrovirus; Escherichia virus RB3
GCA_002815275.1	15378	6	Bat Paramyxovirus Epo_speAR1DRC2009	NA	Viruses; Riboviria; Orthornavirae; Negarnaviricota; Haploviricotina; Monjiviricetes; Mononegavirales; Paramyxoviridae; Rubulavirinae; Orthorubulavirus; Bat mumps orthorubulavirus
GCA_000925295.1	18530	7	Bat Paramyxovirus Eid_helGHM74aGHA2009	NA	Viruses; ssRNA viruses; ssRNA negative-strand viruses; Mononegavirales; Paramyxoviridae; unclassified Paramyxoviridae
GCA_000896495.1	7488	5	Actinidia virus B	NA	Viruses; ssRNA viruses; ssRNA positive-strand viruses, no DNA stage; Tymovirales; Betaflexiviridae; Trivirinae; Vitivirus
GCA_000862505.1	31028	12	Bovine coronavirus	NA	Viruses; ssRNA viruses; ssRNA positive-strand viruses, no DNA stage; Nidovirales; Coronaviridae; Coronavirinae; Betacoronavirus; Betacoronavirus 1
GCA_000856025.1	29355	9	Feline infectious peritonitis virus	NA	Viruses; ssRNA viruses; ssRNA positive-strand viruses, no DNA stage; Nidovirales; Coronaviridae; Coronavirinae; Alphacoronavirus; Alphacoronavirus 1; Feline coronavirus
GCA_000927575.1	52869	84	Salmonella phage 9NA	NA	Viruses; dsDNA viruses, no RNA stage; Caudovirales; Podoviridae; Nonanavirus; Salmonella virus 9NA
GCA_000853505.1	27317	8	Human coronavirus 229E	NA	Viruses; ssRNA viruses; ssRNA positive-strand viruses, no DNA stage; Nidovirales; Coronaviridae; Coronavirinae; Alphacoronavirus
GCA_003971785.1	31335	10	Murine hepatitis virus	A59	Viruses; Riboviria; Orthornavirae; Pisuviricota; Pisoniviricetes; Nidovirales; Cornidovirineae; Coronaviridae; Orthocoronavirinae; Betacoronavirus; Embecovirus; Murine coronavirus
GCA_000898175.1	46271	72	Pseudomonas phage tf	NA	Viruses; dsDNA viruses, no RNA stage; Caudovirales; Podoviridae; unclassified Podoviridae
GCA_000859845.1	6513	4	Zucchini green mottle mosaic virus	Type strain	Viruses; ssRNA viruses; ssRNA positive-strand viruses, no DNA stage; Virgaviridae; Tobamovirus

gca	genome.size	proteome.size	phage.name	strain	Taxonomy
GCA_004787155.1	127320	82	Retroperitoneal fibromatosisassociated herpesvirus	NA	Viruses; Duplodnaviria; Heunggongvirae; Peploviricota; Herviviricetes; Herpesvirales; Herpesviridae; Gammaherpesvirinae; Rhadinovirus; Macacine gammaherpesvirus 8
GCA_000856245.1	4113	2	Diaporthe ambigua RNA virus 1	NA	Viruses; ssRNA viruses; ssRNA positive-strand viruses, no DNA stage; unclassified ssRNA positive-strand viruses
GCA_002985995.1	28586	8	Transmissible gastroenteritis virus	Purdue	Viruses; Riboviria; Orthornavirae; Pisuviricota; Pisoniviricetes; Nidovirales; Cornidovirineae; Coronaviridae; Orthocoronavirinae; Alphacoronavirus; Tegacovirus; Alphacoronavirus 1
GCA_000894595.1	2459	5	Pig stool associated circular ssDNA virus GER2011	NA	Viruses; ssDNA viruses; unclassified ssDNA viruses; Pig stool associated circular ssDNA virus
GCA_000880055.1	27657	11	Turkey coronavirus	NA	Viruses; ssRNA viruses; ssRNA positive-strand viruses, no DNA stage; Nidovirales; Coronaviridae; Coronavirinae; Gammacoronavirus; Avian coronavirus
GCA_000894315.1	43221	61	Salmonella phage SE2	NA	Viruses; dsDNA viruses, no RNA stage; Caudovirales; Siphoviridae; Guernseyvirinae; Jerseyvirus
GCA_002601985.1	47343	68	Escherichia phage 1720a02	NA	Viruses; Duplodnaviria; Heunggongvirae; Uroviricota; Caudoviricetes; Caudovirales; Siphoviridae; unclassified Siphoviridae
GCA_001745435.1	4663	2	Cannabis cryptic virus	NA	Viruses; dsRNA viruses; Partitiviridae; Betapartitivirus
GCA_003090835.1	2808	3	SFTS virus Mao	mao	Viruses; Riboviria; Orthornavirae; Negarnaviricota; Polyploviricotina; Ellioviricetes; Bunyavirales; Phenuiviridae; Phlebovirus; SFTS phlebovirus
GCA_000896815.1	21592	28	Thermococcus prieurii virus 1	NA	Viruses; unclassified archaeal viruses
GCA_000905095.1	142349	242	Pectobacterium phage phiTE	NA	Viruses; dsDNA viruses, no RNA stage; Caudovirales; Myoviridae; unclassified Myoviridae
GCA_000927115.1	19152	11	Tailam virus	TL8K	Viruses; ssRNA viruses; ssRNA negative-strand viruses; Mononegavirales; Paramyxoviridae; unclassified Paramyxoviridae
GCA_000898375.1	42784	63	Staphylococcus phage SpaA1	NA	Viruses; dsDNA viruses, no RNA stage; Caudovirales; Siphoviridae; unclassified Siphoviridae
GCA_003047795.1	42932	63	Bacillus phage BceA1	NA	Viruses; Duplodnaviria; Heunggongvirae; Uroviricota; Caudoviricetes; Caudovirales; Siphoviridae; unclassified Siphoviridae
GCA_000973275.2	12917	10	Wobbly possum disease virus	NA	Viruses; Riboviria; Orthornavirae; Pisuviricota; Pisoniviricetes; Nidovirales; Arnidovirineae; Arteriviridae; Zealarterivirinae; Kappaarterivirus; Kappaarterivirus wobum
GCA_000843625.1	86462	90	Neodiprion sertifer nucleopolyhedrovirus	NA	Viruses; dsDNA viruses, no RNA stage; Baculoviridae; Gammabaculovirus
GCA_000855625.1	15384	9	Sendai virus	Ohita	Viruses; ssRNA viruses; ssRNA negative-strand viruses; Mononegavirales; Paramyxoviridae; Respirovirus
GCA_000859605.1	6514	4	Kyuri green mottle mosaic virus	KGMMVC1	Viruses; ssRNA viruses; ssRNA positive-strand viruses, no DNA stage; Virgaviridae; Tobamovirus
GCA_000839465.1	35466	45	Streptococcus virus 7201	NA	Viruses; Duplodnaviria; Heunggongvirae; Uroviricota; Caudoviricetes; Caudovirales; Siphoviridae; Moineauvirus
GCA_000895075.1	10874	2	Chocolate lily virus A	NA	Viruses; ssRNA viruses; ssRNA positive-strand viruses, no DNA stage; Picornavirales; Secoviridae; unassigned Secoviridae
GCA_003051685.1	189567	250	Ostreococcus tauri virus RT2011	NA	Viruses; Varidnaviria; Bamfordvirae; Nucleocytoviricota; Megaviricetes; Algavirales; Phycodnaviridae; Prasinovirus; unclassified Prasinovirus
GCA_000854745.1	15456	6	Bovine respirovirus 3	Kansas1562684	Viruses; Riboviria; Orthornavirae; Negarnaviricota; Haploviricotina; Monjiviricetes; Mononegavirales; Paramyxoviridae; Orthoparamyxovirinae; Respirovirus
GCA_000850205.1	15462	6	Human respirovirus 3	GP	Viruses; Riboviria; Orthornavirae; Negarnaviricota; Haploviricotina; Monjiviricetes; Mononegavirales; Paramyxoviridae; Orthoparamyxovirinae; Respirovirus
GCA_000856405.1	6614	7	Cactus virus X	NA	Viruses; ssRNA viruses; ssRNA positive-strand viruses, no DNA stage; Tymovirales; Alphaflexiviridae; Potexvirus
GCA_000858825.1	6185	6	Hydrangea ringspot virus	NA	Viruses; ssRNA viruses; ssRNA positive-strand viruses, no DNA stage; Tymovirales; Alphaflexiviridae; Potexvirus
GCA_000856965.1	6450	5	Pepino mosaic virus	NA	Viruses; ssRNA viruses; ssRNA positive-strand viruses, no DNA stage; Tymovirales; Alphaflexiviridae; Potexvirus
GCA_000910675.1	17052	7	Human parainfluenza virus 4a	M25	Viruses; ssRNA viruses; ssRNA negative-strand viruses; Mononegavirales; Paramyxoviridae; Rubulavirus; Human parainfluenza virus 4
GCA_000854065.1	15690	7	Canine morbillivirus	NA	Viruses; Riboviria; Orthornavirae; Negarnaviricota; Haploviricotina; Monjiviricetes; Mononegavirales; Paramyxoviridae; Orthoparamyxovirinae; Morbillivirus
GCA_000854845.1	15894	7	Measles virus	IchinoseB95a	Viruses; ssRNA viruses; ssRNA negative-strand viruses; Mononegavirales; Paramyxoviridae; Morbillivirus
GCA_000895695.1	6506	4	Rattail cactus necrosisassociated virus	NA	Viruses; ssRNA viruses; ssRNA positive-strand viruses, no DNA stage; Virgaviridae; Tobamovirus
GCA_003088495.1	11490	4	Phlebovirus JN1China2010	NA	Viruses; Riboviria; Orthornavirae; Negarnaviricota; Polyploviricotina; Ellioviricetes; Bunyavirales; Phenuiviridae; Phlebovirus; SFTS phlebovirus
GCA_000897015.1	36711	31	Chimpanzee adenovirus Y25	NA	Viruses; dsDNA viruses, no RNA stage; Adenoviridae; Mastadenovirus; Human mastadenovirus E
GCA_000903055.1	76107	75	Sulfolobus virus STSV2	NA	Viruses; dsDNA viruses, no RNA stage; Bicaudaviridae; unclassified Bicaudaviridae
GCA_001433625.1	15696	8	Phocine morbillivirus	PDVWadden_SeaNLD1988	Viruses; Riboviria; Orthornavirae; Negarnaviricota; Haploviricotina; Monjiviricetes; Mononegavirales; Paramyxoviridae; Orthoparamyxovirinae; Morbillivirus

gca	genome.size	proteome.size	phage.name	strain	Taxonomy
GCA_000856645.1	15882	7	Rinderpest virus strain Kabete O	Kabete O virulent	Viruses; ssRNA viruses; ssRNA negative-strand viruses; Mononegavirales; Paramyxoviridae; Morbillivirus; Rinderpest virus
GCA_000893475.1	9999	1	Celery mosaic virus	NA	Viruses; ssRNA viruses; ssRNA positive-strand viruses, no DNA stage; Potyviridae; Potyvirus
GCA_000852625.1	9346	1	Oat necrotic mottle virus	NA	Viruses; ssRNA viruses; ssRNA positive-strand viruses, no DNA stage; Potyviridae; Tritimovirus
GCA_000870725.1	5168	6	Tomato mild yellow leaf curl Aragua virus	NA	Viruses; ssDNA viruses; Geminiviridae; Begomovirus
GCA_000902395.1	44401	51	Bacillus phage phIS3501	NA	Viruses; dsDNA viruses, no RNA stage; Caudovirales; Siphoviridae; unclassified Siphoviridae
GCA_000849305.1	15140	11	Bovine respiratory syncytial virus	ATue51908	Viruses; ssRNA viruses; ssRNA negative-strand viruses; Mononegavirales; Pneumoviridae; Orthopneumovirus
GCA_000903215.1	44784	57	Escherichia phage TL2011b	NA	Viruses; dsDNA viruses, no RNA stage; Caudovirales; Podoviridae; Epsilon15virus; unclassified Epsilon15likevirus
GCA_000900675.1	60523	72	Escherichia phage TL2011c	NA	Viruses; dsDNA viruses, no RNA stage; Caudovirales; Podoviridae; unclassified Podoviridae
GCA_002815455.1	15140	11	Bovine respiratory syncytial virus ATCC51908	NA	Viruses; Riboviria; Orthornavirae; Negarnaviricota; Haploviricotina; Monjiviricetes; Mononegavirales; Pneumoviridae; Orthopneumovirus; Bovine orthopneumovirus
GCA_006446535.1	35168	37	Human adenovirus 63	humanUSABP72195963P30H30F29	Viruses; Varidnaviria; Bamfordvirae; Preplasmiviricota; Tectiliviricetes; Rowavirales; Adenoviridae; Mastadenovirus; Human mastadenovirus D
GCA_000896635.1	284757	309	Pseudomonas phage OBP	NA	Viruses; dsDNA viruses, no RNA stage; Caudovirales; Myoviridae; unclassified Myoviridae
GCA_000855545.1	15225	11	Human respiratory syncytial virus	B1	Viruses; ssRNA viruses; ssRNA negative-strand viruses; Mononegavirales; Pneumoviridae; Orthopneumovirus
GCA_000895275.1	38684	52	Salmonella phage SPN1S	NA	Viruses; dsDNA viruses, no RNA stage; Caudovirales; Podoviridae; unclassified Podoviridae
GCA_001678195.1	10158	1	Hanko virus	NA	Viruses; ssRNA viruses; ssRNA positive-strand viruses, no DNA stage; Flaviviridae; Flavivirus; unclassified Flavivirus
GCA_000895455.1	5510	8	Cassava mosaic Madagascar virus	NA	Viruses; ssDNA viruses; Geminiviridae; Begomovirus; unclassified Begomovirus
GCA_009931745.1	87722	96	Wolbachia phage WO	NA	Viruses; Duplodnaviria; Heunggongvirae; Uroviricota; Caudoviricetes; Caudovirales; Myoviridae; unclassified Myoviridae
GCA_002815495.1	14886	11	Murine orthopneumovirus	15 ATCC VR25	Viruses; Riboviria; Orthornavirae; Negarnaviricota; Haploviricotina; Monjiviricetes; Mononegavirales; Pneumoviridae; Orthopneumovirus
GCA_000930075.1	5426	2	Porcine parvovirus 2	BRGOion_09_PPV22011	Viruses; ssDNA viruses; Parvoviridae; Parvovirinae; Protoparvovirus; unclassified Protoparvovirus
GCA_000926295.1	1966	2	Silurus glanis circovirus	NA	Viruses; ssDNA viruses; Circoviridae; Circovirus; European catfish circovirus
GCA_001275535.1	41938	57	Thermoanaerobacterium phage THSA485A	NA	Viruses; dsDNA viruses, no RNA stage; Caudovirales; Siphoviridae; unclassified Siphoviridae
GCA_002602065.1	162598	272	Bacillus phage B5S	NA	Viruses; Duplodnaviria; Heunggongvirae; Uroviricota; Caudoviricetes; Caudovirales; Herelleviridae; Bastillevirinae; Bequatrovirus; Bacillus virus B4; Bacillus phage B4
GCA_000898735.1	156176	227	Bacillus phage BCP78	NA	Viruses; dsDNA viruses, no RNA stage; Caudovirales; Myoviridae; unclassified Myoviridae
GCA_002602025.1	154371	223	Bacillus phage BCU4	NA	Viruses; Duplodnaviria; Heunggongvirae; Uroviricota; Caudoviricetes; Caudovirales; Herelleviridae; Bastillevirinae; Tsarbombavirus; Bacillus virus BCP78
GCA_000906815.1	11120	5	Chandipura virus	NA	Viruses; ssRNA viruses; ssRNA negative-strand viruses; Mononegavirales; Rhabdoviridae; Vesiculovirus
GCA_000896335.1	40128	62	Salmonella phage SPN9CC	NA	Viruses; dsDNA viruses, no RNA stage; Caudovirales; Podoviridae; unclassified Podoviridae
GCA_002816055.1	11271	5	Piry virus	BeAn2423	Viruses; Riboviria; Orthornavirae; Negarnaviricota; Haploviricotina; Monjiviricetes; Mononegavirales; Rhabdoviridae; Vesiculovirus; Piry vesiculovirus
GCA_003033385.1	2600	1	Circoviridae bovine stoolBKKOR2011	NA	Viruses; Monodnaviria; Shotokuvirae; Cressdnaviricota; Arfiviricetes; Cremevirales; Smacoviridae; Bovismacovirus; Bovine associated bovismacovirus 1
GCA_000901775.1	43551	83	Aeromonas phage vB_AsaM56	NA	Viruses; dsDNA viruses, no RNA stage; Caudovirales; Myoviridae; unclassified Myoviridae
GCA_000903295.1	45354	76	Bdellovibrio phage phi1422	NA	Viruses; dsDNA viruses, no RNA stage; Caudovirales; Myoviridae; unclassified Myoviridae
GCA_000900755.1	48454	68	Pectobacterium phage ZF40	NA	Viruses; dsDNA viruses, no RNA stage; Caudovirales; Myoviridae; unclassified Myoviridae
GCA_000902415.1	44485	67	Vibrio phage vB_VchM138	NA	Viruses; dsDNA viruses, no RNA stage; Caudovirales; Myoviridae; unclassified Myoviridae
GCA_000850045.1	11161	5	Vesicular stomatitis Indiana virus	NA	Viruses; ssRNA viruses; ssRNA negative-strand viruses; Mononegavirales; Rhabdoviridae; Vesiculovirus
GCA_000895095.1	6677	3	Espirito Santo virus	NA	Viruses; dsRNA viruses; Birnaviridae; unclassified Birnaviridae
GCA_002823885.1	2632	5	Tomato Chino La Paz virus SLP 4	NA	Viruses; Monodnaviria; Shotokuvirae; Cressdnaviricota; Repensiviricetes; Geplafuvirales; Geminiviridae; Begomovirus; Tomato chino La Paz virus

gca	genome.size	proteome.size	phage.name	strain	Taxonomy
GCA_000923855.1	11123	5	Vesicular stomatitis New Jersey virus	NA	Viruses; ssRNA viruses; ssRNA negative-strand viruses; Mononegavirales; Rhabdoviridae; Vesiculovirus
GCA_000901795.1	18919	22	Clostridium phage phi24R	NA	Viruses; dsDNA viruses, no RNA stage; Caudovirales; Podoviridae; Picovirinae; unassigned Picovirinae
GCA_003985485.1	2218	2	Meles meles circoviruslike virus	NA	Viruses; Monodnaviria; Shotokuvirae; Cressdnaviricota; Arfiviricetes; Cirlivirales; Circoviridae; unclassified Circoviridae
GCA_000895735.1	3048	5	Parrot hepatitis B virus	NA	Viruses; Riboviria; Pararnavirae; Artverviricota; Revtraviricetes; Blubervirales; Hepadnaviridae; Avihepadnavirus
GCA_000896575.1	6514	4	Clitoria yellow mottle virus	NA	Viruses; ssRNA viruses; ssRNA positive-strand viruses, no DNA stage; Virgaviridae; Tobamovirus
GCA_002924555.1	1246126	1217	Megavirus courdo11	NA	Viruses; Varidnaviria; Bamfordvirae; Nucleocytoviricota; Megaviricetes; Imitervirales; Mimiviridae; Mimivirus; unclassified Mimivirus; Megavirus chiliensis
GCA_003850265.1	2198	2	Meles meles fecal virus	NA	Viruses; Monodnaviria; Shotokuvirae; Cressdnaviricota; Repensiviricetes; Geplafuvirales; Genomoviridae; Gemygorvirus; Mallard associated gemygorvirus 1
GCA_018577845.1	2618	3	Pine marten torque teno virus 1	NA	Viruses; Anelloviridae; Thetatorquevirus; Torque teno mustilid virus 1
GCA_000841925.1	231621	113	Helicoverpa zea nudivirus 2	NA	Viruses; dsDNA viruses, no RNA stage; Nudiviridae; Betanudivirus; Heliothis zea nudivirus
GCA_000850065.1	11131	6	Infectious hematopoietic necrosis virus	WRAC	Viruses; ssRNA viruses; ssRNA negative-strand viruses; Mononegavirales; Rhabdoviridae; Novirhabdovirus
GCA_000896055.1	2675	5	Centrosema yellow spot virus	NA	Viruses; ssDNA viruses; Geminiviridae; Begomovirus
GCA_000894475.1	5197	7	Macroptilium yellow net virus	NA	Viruses; ssDNA viruses; Geminiviridae; Begomovirus
GCA_000895435.1	2660	5	Macroptilium yellow spot virus	NA	Viruses; ssDNA viruses; Geminiviridae; Begomovirus
GCA_000896915.1	2656	5	Macroptilium yellow vein virus	NA	Viruses; ssDNA viruses; Geminiviridae; Begomovirus
GCA_000896755.1	49135	69	Pseudomonas phage phi297	NA	Viruses; dsDNA viruses, no RNA stage; Caudovirales; Siphoviridae; unclassified Siphoviridae
GCA_000895295.1	54024	96	Pseudomonas virus PMG1	NA	Viruses; Duplodnaviria; Heunggongvirae; Uroviricota; Caudoviricetes; Caudovirales; Siphoviridae; Detrevirus
GCA_000904995.1	174413	276	Klebsiella phage KP27	NA	Viruses; dsDNA viruses, no RNA stage; Caudovirales; Myoviridae; Tevenvirinae; T4virus; unclassified T4virus
GCA_001550825.1	49797	79	Klebsiella phage KP36	NA	Viruses; dsDNA viruses, no RNA stage; Caudovirales; Siphoviridae; Tunavirinae; Kp36virus; Klebsiella virus KP36
GCA_001041555.1	159071	220	Bacillus phage BCP82	NA	Viruses; dsDNA viruses, no RNA stage; Caudovirales; Myoviridae; Bc431virus
GCA_000896315.1	49981	81	Xanthomonas phage vB_XveM_DIBBI	NA	Viruses; dsDNA viruses, no RNA stage; Caudovirales; Myoviridae; unclassified Myoviridae
GCA_000859625.1	11932	5	Rabies lyssavirus	NA	Viruses; ssRNA viruses; ssRNA negative-strand viruses; Mononegavirales; Rhabdoviridae; Lyssavirus
GCA_000894535.1	9543	1	Valsa ceratosperma hypovirus 1	MVC86	Viruses; dsRNA viruses; Hypoviridae; Hypovirus; unclassified Hypovirus
GCA_000845545.1	14900	12	Bovine ephemeral fever virus	BB7721	Viruses; ssRNA viruses; ssRNA negative-strand viruses; Mononegavirales; Rhabdoviridae; Ephemerovirus
GCA_000848205.1	13720	6	Sonchus yellow net virus	NA	Viruses; ssRNA viruses; ssRNA negative-strand viruses; Mononegavirales; Rhabdoviridae; Nucleorhabdovirus
GCA_002823285.1	2762	6	Sauropus leaf curl virus	NA	Viruses; Monodnaviria; Shotokuvirae; Cressdnaviricota; Repensiviricetes; Geplafuvirales; Geminiviridae; Begomovirus
GCA_002602045.1	74155	137	Mycobacterium phage Fezzik	NA	Viruses; Duplodnaviria; Heunggongvirae; Uroviricota; Caudoviricetes; Caudovirales; Siphoviridae; Bronvirus; unclassified Bronvirus
GCA_000894335.1	60714	64	Erwinia phage PEp14	NA	Viruses; dsDNA viruses, no RNA stage; Caudovirales; Podoviridae; unclassified Podoviridae
GCA_000904295.1	40489	64	Rhodobacter phage RcapNL	NA	Viruses; dsDNA viruses, no RNA stage; unclassified dsDNA phages
GCA_000903315.1	136947	218	Escherichia phage FV3	NA	Viruses; dsDNA viruses, no RNA stage; Caudovirales; Myoviridae; Vequintavirinae; V5virus; Escherichia virus FV3
GCA_002867105.1	9164	2	Cucurbit mild mosaic virus	NA	Viruses; Riboviria; Orthornavirae; Pisuviricota; Pisoniviricetes; Picornavirales; Secoviridae; Comovirinae; Fabavirus
GCA_000896735.1	5065	4	Eidolon helvum bat parvovirus	NA	Viruses; ssDNA viruses; Parvoviridae; Parvovirinae; Tetraparvovirus; Chiropteran tetraparvovirus 1
GCA_000894295.1	4595	2	Artibeus jamaicensis parvovirus 1	NA	Viruses; ssDNA viruses; Parvoviridae; Parvovirinae; unclassified Parvovirinae
GCA_000895815.1	5076	4	Porcine bocavirus 5JS677	NA	Viruses; ssDNA viruses; Parvoviridae; Parvovirinae; Bocaparvovirus; unclassified Bocaparvovirus; Porcine bocavirus 5
GCA_000895195.1	13459	1	Persea americana endornavirus	NA	Viruses; dsRNA viruses; Endornaviridae; Endornavirus; unclassified Endornavirus

gca	genome.size	proteome.size	phage.name	strain	Taxonomy
GCA_000897395.1	42832	55	Burkholderia phage vB_BceS_KL1	NA	Viruses; Duplodnaviria; Heunggongvirae; Uroviricota; Caudoviricetes; Caudovirales; Siphoviridae; Kilunavirus; Burkholderia virus KL1
GCA_000898995.1	58065	78	Burkholderia phage vB_BceS_AH2	NA	Viruses; Duplodnaviria; Heunggongvirae; Uroviricota; Caudoviricetes; Caudovirales; Siphoviridae; Ahduovirus; Burkholderia virus AH2
GCA_000919615.1	18338	20	Sputnik virophage 2	NA	Viruses; Varidnaviria; Bamfordvirae; Preplasmiviricota; Maveriviricetes; Priklausovirales; Lavidaviridae; Sputnikvirus; Mimivirus-dependent virus Sputnik
GCA_000920455.1	18338	20	Sputnik virophage 3	NA	Viruses; Varidnaviria; Bamfordvirae; Preplasmiviricota; Maveriviricetes; Priklausovirales; Lavidaviridae; Sputnikvirus; Mimivirus-dependent virus Sputnik
GCA_000900615.1	41148	58	Brucella phage Tb	NA	Viruses; dsDNA viruses, no RNA stage; Caudovirales; Podoviridae; unclassified Podoviridae
GCA_000902275.1	38253	57	Brucella phage Pr	NA	Viruses; dsDNA viruses, no RNA stage; Caudovirales; Podoviridae; unclassified Podoviridae
GCA_004789575.1	12094	4	Schmallenberg virus	NA	Viruses; Riboviria; Orthornavirae; Negarnaviricota; Polyploviricotina; Ellioviricetes; Bunyavirales; Peribunyaviridae; Orthobunyavirus; Schmallenberg orthobunyavirus
GCA_003726515.1	8112	2	Phytophthora infestans RNA virus 3	NA	Viruses; Riboviria; unclassified Riboviria
GCA_000867605.1	156922	123	Spodoptera frugiperda ascovirus 1a	NA	Viruses; dsDNA viruses, no RNA stage; Ascoviridae; Ascovirus
GCA_004128795.1	2445	1	Sclerotinia sclerotiorum mitovirus 2	NA	Viruses; Riboviria; Orthornavirae; Lenarviricota; Howeltoviricetes; Cryppavirales; Mitoviridae; Mitovirus; unclassified Mitovirus
GCA_000896195.1	4138	4	Artemisia virus A	NA	Viruses; ssRNA viruses; ssRNA positive-strand viruses, no DNA stage; Sobemovirus; unclassified Sobemovirus
GCA_008787005.1	7387	1	Porcine enterovirus 15	wild boarWBD2011HUN	Viruses; Riboviria; Orthornavirae; Pisuviricota; Pisoniviricetes; Picornavirales; Picornaviridae; Enterovirus; Enterovirus G
GCA_008777615.1	7395	1	Enterovirus C116	NA	Viruses; Riboviria; Orthornavirae; Pisuviricota; Pisoniviricetes; Picornavirales; Picornaviridae; Enterovirus; Enterovirus C
GCA_001343805.1	10614	4	Lunk virus NKS1	NA	Viruses; ssRNA viruses; ssRNA negative-strand viruses; Arenaviridae; Mammarenavirus; Lunk mammarenavirus
GCA_000860785.1	6099	2	Poinsettia mosaic virus	NA	Viruses; ssRNA viruses; ssRNA positive-strand viruses, no DNA stage; Tymovirales; Tymoviridae; unassigned Tymoviridae
GCA_000895935.1	8210	1	Porcine kobuvirus SHWCHN2010China	NA	Viruses; ssRNA viruses; ssRNA positive-strand viruses, no DNA stage; Picornavirales; Picornaviridae; Kobuvirus; Aichivirus C; Porcine kobuvirus
GCA_000900775.1	93839	140	Bacillus phage BCD7	NA	Viruses; dsDNA viruses, no RNA stage; Caudovirales; Myoviridae; unclassified Myoviridae
GCA_000899095.1	61847	73	Burkholderia virus DC1	NA	Viruses; dsDNA viruses, no RNA stage; Caudovirales; Podoviridae; Bcep22likevirus
GCA_000900195.1	158305	268	Bacillus phage BPS13	NA	Viruses; dsDNA viruses, no RNA stage; Caudovirales; Myoviridae; unclassified Myoviridae
GCA_000902515.1	161440	180	Cyanophage STIM5	NA	Viruses; dsDNA viruses, no RNA stage; Caudovirales; Myoviridae; unclassified Myoviridae
GCA_000895955.1	6707	3	Astrovirus wild boarWBAstV12011HUN	NA	Viruses; ssRNA viruses; ssRNA positive-strand viruses, no DNA stage; Astroviridae; Mamastrovirus; unclassified Mamastrovirus
GCA_018577815.1	2012	3	Torque teno felis virusFcTTV2	NA	Viruses; Anelloviridae; Etatorquevirus; Torque teno felid virus 4
GCA_018577805.1	2039	3	Torque teno felis virusFcTTV1	NA	Viruses; Anelloviridae; Tautorquevirus; Torque teno felid virus 5
GCA_002029635.1	12323	3	Tucunduba virus	BeAR643361	Viruses; Riboviria; Orthornavirae; Negarnaviricota; Polyploviricotina; Ellioviricetes; Bunyavirales; Peribunyaviridae; Orthobunyavirus; Wyeomyia orthobunyavirus
GCA_004789515.1	12560	3	Cachoeira Porteira virus	BeAr328208	Viruses; Riboviria; Orthornavirae; Negarnaviricota; Polyploviricotina; Ellioviricetes; Bunyavirales; Peribunyaviridae; Orthobunyavirus; Cachoeira Porteira orthobunyavirus
GCA_000902435.1	41572	51	Aeromonas phage phiAS7	NA	Viruses; dsDNA viruses, no RNA stage; Caudovirales; Podoviridae; Autographivirinae; unassigned Autographivirinae
GCA_000897755.1	162596	277	Bacillus phage B4	NA	Viruses; dsDNA viruses, no RNA stage; Caudovirales; Myoviridae; B4virus; Bacillus virus B4
GCA_000901515.1	59203	72	Salmonella virus SPN19	NA	Viruses; Duplodnaviria; Heunggongvirae; Uroviricota; Caudoviricetes; Caudovirales; Siphoviridae; Chivirus
GCA_000900455.1	147940	269	Cronobacter phage vB_CsaM_GAP31	NA	Viruses; dsDNA viruses, no RNA stage; Caudovirales; Myoviridae; Vequintavirinae; Se1virus; Cronobacter virus GAP31
GCA_000898015.1	358663	545	Cronobacter phage vB_CsaM_GAP32	NA	Viruses; dsDNA viruses, no RNA stage; Caudovirales; Myoviridae; unclassified Myoviridae
GCA_000899595.1	76631	115	Cronobacter phage vB_CsaP_GAP52	NA	Viruses; dsDNA viruses, no RNA stage; Caudovirales; Podoviridae; Phieco32virus; unclassified Phieco32likevirus
GCA_000901535.1	178193	275	Cronobacter phage vB_CsaM_GAP161	NA	Viruses; dsDNA viruses, no RNA stage; Caudovirales; Myoviridae; Tevenvirinae; T4virus; unclassified T4virus
GCA_000900475.1	43731	56	Escherichia phage vB_EcoP_ACGC91	vB_EcoP_C91	Viruses; Duplodnaviria; Heunggongvirae; Uroviricota; Caudoviricetes; Caudovirales; Autographiviridae; Molineuxvirinae; Vectrevirus; Escherichia virus ACGC91
GCA_000900515.1	46054	78	Escherichia phage vB_Eco_ACGM12	vB_EcoS_MSHS1210	Viruses; dsDNA viruses, no RNA stage; Caudovirales; Siphoviridae; Tunavirinae; Rtpvirus; Escherichia virus ACGM12

gca	genome.size	proteome.size	phage.name	strain	Taxonomy
GCA_000899615.1	167396	282	Escherichia phage vB_EcoM_ACGC40	NA	Viruses; dsDNA viruses, no RNA stage; Caudovirales; Myoviridae; Tevenvirinae; T4virus; Escherichia virus C40
GCA_000901815.1	42608	31	Escherichia phage phiKT	NA	Viruses; dsDNA viruses, no RNA stage; Caudovirales; Podoviridae; Autographivirinae; Phikmvvirus; unclassified phiKMV-like phages
GCA_002632545.1	42965	54	Pseudomonas phage vB_PaeTbilisiM32	NA	Viruses; dsDNA viruses, no RNA stage; Caudovirales; Podoviridae; Autographivirinae; Phikmvvirus; unclassified phiKMV-like phages
GCA_000897095.1	42844	58	Pseudomonas phage vB_PaeKakheti25	NA	Viruses; dsDNA viruses, no RNA stage; Caudovirales; Siphoviridae; Septima3virus
GCA_014883545.1	8099	1	Murine kobuvirus 1	NA	Viruses; Riboviria; Orthornavirae; Pisuviricota; Pisoniviricetes; Picornavirales; Picornaviridae; Kobuvirus; Aichivirus A
GCA_000900135.1	6838	3	Murine astrovirus	STL 1	Viruses; ssRNA viruses; ssRNA positive-strand viruses, no DNA stage; Astroviridae; unclassified Astroviridae
GCA_002822985.1	2746	6	Papaya leaf curl virussoybean Lucknow	NA	Viruses; Monodnaviria; Shotokuvirae; Cressdnaviricota; Repensiviricetes; Geplafuvirales; Geminiviridae; Begomovirus; Papaya leaf curl virus
GCA_002822205.1	2735	6	Cotton leaf curl Multan virusHibiscus rosasinensis	NA	Viruses; Monodnaviria; Shotokuvirae; Cressdnaviricota; Repensiviricetes; Geplafuvirales; Geminiviridae; Begomovirus; Cotton leaf curl Multan virus
GCA_000841645.1	36798	61	Lactococcus phage ul36	NA	Viruses; dsDNA viruses, no RNA stage; Caudovirales; Siphoviridae; unclassified Siphoviridae
GCA_017654205.1	33175	32	Bovine adenovirus 2	KY191	Viruses; Varidnaviria; Bamfordvirae; Preplasmiviricota; Tectiliviricetes; Rowavirales; Adenoviridae; Mastadenovirus; Ovine mastadenovirus A
GCA_000842705.1	29576	30	Ovine adenovirus 7	OAV287	Viruses; Varidnaviria; Bamfordvirae; Preplasmiviricota; Tectiliviricetes; Rowavirales; Adenoviridae; Atadenovirus; Ovine atadenovirus D
GCA_000895335.1	7855	7	Trichechus manatus latirostris papillomavirus 2	M0920	Viruses; dsDNA viruses, no RNA stage; Papillomaviridae; unclassified Papillomaviridae; non-primate mammal papillomaviruses
GCA_003179235.1	7792	7	Tursiops truncatus papillomavirus 4	Tt08094	Viruses; Monodnaviria; Shotokuvirae; Cossaviricota; Papovaviricetes; Zurhausenvirales; Papillomaviridae; Firstpapillomavirinae; Upsilonpapillomavirus; Upsilonpapillomavirus 1
GCA_003179255.1	7853	7	Tursiops truncatus papillomavirus 5	Tt08095	Viruses; Monodnaviria; Shotokuvirae; Cossaviricota; Papovaviricetes; Zurhausenvirales; Papillomaviridae; Firstpapillomavirinae; Omikronpapillomavirus; Omikronpapillomavirus 1
GCA_003179275.1	7895	7	Tursiops truncatus papillomavirus 6	Tt08305	Viruses; Monodnaviria; Shotokuvirae; Cossaviricota; Papovaviricetes; Zurhausenvirales; Papillomaviridae; Firstpapillomavirinae; Omikronpapillomavirus; Omikronpapillomavirus 1
GCA_003179295.1	7783	7	Tursiops truncatus papillomavirus 7	Tt0943	Viruses; Monodnaviria; Shotokuvirae; Cossaviricota; Papovaviricetes; Zurhausenvirales; Papillomaviridae; Firstpapillomavirinae; Upsilonpapillomavirus; Upsilonpapillomavirus 1
GCA_006447355.1	35231	44	Human adenovirus 64	humanUSAC199364P22H19F37	Viruses; Varidnaviria; Bamfordvirae; Preplasmiviricota; Tectiliviricetes; Rowavirales; Adenoviridae; Mastadenovirus; Human mastadenovirus D
GCA_006415475.1	31750	30	Bat mastadenovirus A	Mm32	Viruses; dsDNA viruses, no RNA stage; Adenoviridae; Mastadenovirus
GCA_002647975.1	18613	11	Rotavirus A HuBELF013222009G3P6	NA	Viruses; Riboviria; Orthornavirae; Duplornaviricota; Resentoviricetes; Reovirales; Reoviridae; Sedoreovirinae; Rotavirus; Rotavirus A; Rotavirus G3
GCA_002648195.1	18613	11	Rotavirus A HuBELF014982009G3P6	NA	Viruses; Riboviria; Orthornavirae; Duplornaviricota; Resentoviricetes; Reovirales; Reoviridae; Sedoreovirinae; Rotavirus; Rotavirus A; Rotavirus G3
GCA_002648415.1	18612	11	Rotavirus A HuUSA062422006G2P6	NA	Viruses; Riboviria; Orthornavirae; Duplornaviricota; Resentoviricetes; Reovirales; Reoviridae; Sedoreovirinae; Rotavirus; Rotavirus A; Rotavirus G2
GCA_000905915.2	44714	74	Staphylococcus phage StB12	NA	Viruses; dsDNA viruses, no RNA stage; Caudovirales; Siphoviridae; unclassified Siphoviridae
GCA_000904855.1	40917	65	Staphylococcus phage StB20	NA	Viruses; dsDNA viruses, no RNA stage; Caudovirales; Siphoviridae; unclassified Siphoviridae
GCA_000903815.1	40071	53	Staphylococcus phage StB27	NA	Viruses; dsDNA viruses, no RNA stage; Caudovirales; Siphoviridae; unclassified Siphoviridae
GCA_000903335.1	345809	534	Klebsiella phage vB_KleM_RaK2	NA	Viruses; Duplodnaviria; Heunggongvirae; Uroviricota; Caudoviricetes; Caudovirales; Myoviridae; Alcyoneusvirus; Klebsiella virus RaK2
GCA_000904815.1	47595	82	Clostridium phage phi8074B1	NA	Viruses; dsDNA viruses, no RNA stage; Caudovirales; Siphoviridae; unclassified Siphoviridae
GCA_002602105.1	43804	62	Enterobacter phage phiKDA1	NA	Viruses; Duplodnaviria; Heunggongvirae; Uroviricota; Caudoviricetes; Caudovirales; Autographiviridae; Slopekvirinae; Koutsourovirus; Enterobacter virus KDA1
GCA_000900935.1	47355	71	Salmonella phage SPN3UB	NA	Viruses; dsDNA viruses, no RNA stage; Caudovirales; Siphoviridae; unclassified Siphoviridae
GCA_002624725.1	61164	110	Mycobacterium phage Spartacus	NA	Viruses; Duplodnaviria; Heunggongvirae; Uroviricota; Caudoviricetes; Caudovirales; Siphoviridae; Cheoctovirus; Mycobacterium virus Spartacus
GCA_000903555.1	43741	60	Escherichia phage HK578	NA	Viruses; dsDNA viruses, no RNA stage; Caudovirales; Siphoviridae; Hk578virus; Escherichia virus HK578
GCA_000901015.1	40710	71	Enterobacteria phage HK140	NA	Viruses; dsDNA viruses, no RNA stage; Caudovirales; Siphoviridae; Lambdavirus; unclassified Lambda-like viruses
GCA_000902675.1	45366	69	Enterobacteria phage HK225	NA	Viruses; dsDNA viruses, no RNA stage; Caudovirales; Siphoviridae; Lambdavirus; unclassified Lambda-like viruses
GCA_000902055.1	39026	60	Escherichia phage HK446	NA	Viruses; Duplodnaviria; Heunggongvirae; Uroviricota; Caudoviricetes; Caudovirales; Siphoviridae; Hendrixvirus; Escherichia virus HK446
GCA_000903575.1	47090	69	Escherichia phage HK630	NA	Viruses; Duplodnaviria; Heunggongvirae; Uroviricota; Caudoviricetes; Caudovirales; Siphoviridae; Lambdavirus; Escherichia virus HK630

gca	genome.size	proteome.size	phage.name	strain	Taxonomy
GCA_000901035.1	41528	67	Escherichia phage HK633	NA	Viruses; Duplodnaviria; Heunggongvirae; Uroviricota; Caudoviricetes; Caudovirales; Siphoviridae; Hendrixvirus; Escherichia virus HK633
GCA_000902695.1	47288	69	Escherichia phage HK629	NA	Viruses; Duplodnaviria; Heunggongvirae; Uroviricota; Caudoviricetes; Caudovirales; Siphoviridae; Lambdavirus; Escherichia virus HK629
GCA_000902075.1	42780	69	Enterobacteria phage mEp043 c1	NA	Viruses; dsDNA viruses, no RNA stage; Caudovirales; Siphoviridae; Lambdavirus; unclassified Lambda-like viruses
GCA_000903595.1	37595	61	Enterobacteria phage mEp235	NA	Viruses; dsDNA viruses, no RNA stage; Caudovirales; Siphoviridae; Lambdavirus; unclassified Lambda-like viruses
GCA_000901055.1	44375	63	Enterobacteria phage mEp237	NA	Viruses; dsDNA viruses, no RNA stage; Caudovirales; Siphoviridae; Lambdavirus; unclassified Lambda-like viruses
GCA_000902715.1	44510	59	Enterobacteria phage mEp460	NA	Viruses; dsDNA viruses, no RNA stage; Caudovirales; Siphoviridae; Lambdavirus; unclassified Lambda-like viruses
GCA_000902095.1	41567	66	Escherichia phage mEpX1	NA	Viruses; Duplodnaviria; Heunggongvirae; Uroviricota; Caudoviricetes; Caudovirales; Siphoviridae; Hendrixvirus; Escherichia virus mEpX1
GCA_000903615.1	38759	67	Escherichia phage mEpX2	NA	Viruses; Duplodnaviria; Heunggongvirae; Uroviricota; Caudoviricetes; Caudovirales; Siphoviridae; Hendrixvirus; Escherichia virus mEpX2
GCA_000901075.1	45805	74	Enterobacteria phage vB_EcoS_Rogue1	NA	Viruses; dsDNA viruses, no RNA stage; Caudovirales; Siphoviridae; Tunavirinae; Rogue1virus; Escherichia virus Rogue1
GCA_000902735.1	44120	74	Enterobacterial phage mEp213	NA	Viruses; dsDNA viruses, no RNA stage; Caudovirales; Siphoviridae; Lambdavirus; unclassified Lambda-like viruses
GCA_000902115.1	39578	61	Escherichia phage mEp234	NA	Viruses; Duplodnaviria; Heunggongvirae; Uroviricota; Caudoviricetes; Caudovirales; Siphoviridae; Hendrixvirus; Escherichia virus mEp234
GCA_000903635.1	40029	59	Enterobacterial phage mEp390	NA	Viruses; dsDNA viruses, no RNA stage; Caudovirales; Siphoviridae; Lambdavirus; unclassified Lambda-like viruses
GCA_000856505.1	11158	6	Viral hemorrhagic septicemia virus Fil3	NA	Viruses; ssRNA viruses; ssRNA negative-strand viruses; Mononegavirales; Rhabdoviridae; Novirhabdovirus; Viral hemorrhagic septicemia virus
GCA_002633045.1	57198	79	Escherichia phage JLK2012	NA	Viruses; Duplodnaviria; Heunggongvirae; Uroviricota; Caudoviricetes; Caudovirales; Siphoviridae; unclassified Siphoviridae
GCA_000841885.1	58498	98	Natrialba phage PhiCh1	NA	Viruses; dsDNA viruses, no RNA stage; Caudovirales; Myoviridae; unclassified Myoviridae
GCA_000907815.1	5329	2	Rosellinia necatrix victorivirus 1	NA	Viruses; dsRNA viruses; Totiviridae; Victorivirus
GCA_003956565.1	6310	1	Yadokari virus 1	2W1032S6	Viruses; Riboviria; dsRNA viruses; unclassified dsRNA viruses
GCA_000853105.1	3157	2	Zygosaccharomyces bailii virus Z	NA	Viruses; dsRNA viruses; Amalgaviridae; unclassified Amalgaviridae
GCA_002637075.1	18383	11	Rotavirus A HuCI812011KOR	C181	Viruses; Riboviria; Orthornavirae; Duplornaviricota; Resentoviricetes; Reovirales; Reoviridae; Sedoreovirinae; Rotavirus; Rotavirus A; Rotavirus A isolates
GCA_000854465.1	9591	1	Beet mosaic virus	NA	Viruses; ssRNA viruses; ssRNA positive-strand viruses, no DNA stage; Potyviridae; Potyvirus
GCA_000924455.1	9491	1	Carrot thin leaf virus	NA	Viruses; ssRNA viruses; ssRNA positive-strand viruses, no DNA stage; Potyviridae; Potyvirus
GCA_000906635.1	22786	1	Gentian Kobushoassociated virus	NA	Viruses; dsRNA viruses; unclassified dsRNA viruses
GCA_003423325.1	54655	82	Enterobacteria phage mEp021	NA	Viruses; Duplodnaviria; Heunggongvirae; Uroviricota; Caudoviricetes; Caudovirales; Siphoviridae; unclassified Siphoviridae
GCA_000908335.1	8785	2	Canine picodicistrovirus	209	Viruses; ssRNA viruses; ssRNA positive-strand viruses, no DNA stage; Picornavirales; Picornaviridae; Dicipivirus; Cadicivirus A
GCA_000900315.1	60894	79	Escherichia phage P13374	NA	Viruses; dsDNA viruses, no RNA stage; Caudovirales; Podoviridae; unclassified Podoviridae; Escherichia phage phi191 sensu lato
GCA_000900795.1	111506	157	Vibrio phage pVp1	NA	Viruses; dsDNA viruses, no RNA stage; Caudovirales; Siphoviridae; T5virus; unclassified T5likevirus
GCA_000901895.2	60496	80	Enterobacter phage Enc34	NA	Viruses; dsDNA viruses, no RNA stage; Caudovirales; Siphoviridae; unclassified Siphoviridae
GCA_000902455.1	219372	295	Sphingomonas phage PAU	NA	Viruses; dsDNA viruses, no RNA stage; Caudovirales; Myoviridae; unclassified Myoviridae
GCA_000902595.1	7288	12	Ralstonia phage RSS0	NA	Viruses; ssDNA viruses; Inoviridae; Inovirus; unclassified Inovirus
GCA_000928875.1	42544	46	Vibrio phage phiA318	NA	Viruses; dsDNA viruses, no RNA stage; Caudovirales; Podoviridae; unclassified Podoviridae; Escherichia phage phi191 sensu lato
GCA_000896015.1	30578	43	Haloarcula hispanica icosahedral virus 2	NA	Viruses; Virus families not assigned to an order; Sphaerolipoviridae; Alphasphaerolipovirus
GCA_000903415.1	26836	33	Helicobacter phage 1961P	NA	Viruses; dsDNA viruses, no RNA stage; Caudovirales; Podoviridae; unclassified Podoviridae
GCA_000856665.2	12906	9	Influenza C virus CAnn Arbor150	NA	Viruses; ssRNA viruses; ssRNA negative-strand viruses; Orthomyxoviridae; Influenzavirus C; Influenza C virus
GCA_000896955.1	10656	15	Halorubrum pleomorphic virus 2	NA	Viruses; ssDNA viruses; unclassified ssDNA viruses

gca	genome.size	proteome.size	phage.name	strain	Taxonomy
GCA_000894515.1	8770	12	Halorubrum pleomorphic virus 3	NA	Viruses; ssDNA viruses; unclassified ssDNA viruses
GCA_000896115.1	8549	10	Halorubrum pleomorphic virus 6	NA	Viruses; ssDNA viruses; unclassified ssDNA viruses
GCA_000895495.1	9694	15	Halogeometricum pleomorphic virus 1	NA	Viruses; ssDNA viruses; unclassified ssDNA viruses
GCA_001008455.1	8121	1	Porcine kobuvirus	NA	Viruses; Riboviria; Orthornavirae; Pisuviricota; Pisoniviricetes; Picornavirales; Picornaviridae; Kobuvirus; Aichivirus C
GCA_000854245.1	10461	7	Thogoto virus	NA	Viruses; ssRNA viruses; ssRNA negative-strand viruses; Orthomyxoviridae; Thogotovirus
GCA_002829625.1	23317	11	Piscine orthoreovirus	NA	Viruses; Riboviria; Orthornavirae; Duplornaviricota; Resentoviricetes; Reovirales; Reoviridae; Spinareovirinae; Orthoreovirus
GCA_000973215.1	6617	3	Canine astrovirus	Gillingham2012UK	Viruses; ssRNA viruses; ssRNA positive-strand viruses, no DNA stage; Astroviridae; Mamastrovirus; unclassified Mamastrovirus
GCA_002987785.1	24893	57	Aeropyrum coilshaped virus	NA	Viruses; Spiraviridae; Alphaspiravirus
GCA_009731655.1	5514	4	Germiston virus	NA	Viruses; Riboviria; Orthornavirae; Negarnaviricota; Polyploviricotina; Ellioviricetes; Bunyavirales; Peribunyaviridae; Orthobunyavirus; Bunyamwera orthobunyavirus
GCA_004790255.1	12324	5	Maguari virus	BeAr 7272	Viruses; Riboviria; Orthornavirae; Negarnaviricota; Polyploviricotina; Ellioviricetes; Bunyavirales; Peribunyaviridae; Orthobunyavirus; Maguari orthobunyavirus
GCA_002831285.1	12490	6	La Crosse virus	La Crosseoriginal	Viruses; ssRNA viruses; ssRNA negative-strand viruses; Bunyaviridae; Orthobunyavirus; California encephalitis orthobunyavirus
GCA_004789895.1	12208	4	Snowshoe hare virus	NA	Viruses; Riboviria; Orthornavirae; Negarnaviricota; Polyploviricotina; Ellioviricetes; Bunyavirales; Peribunyaviridae; Orthobunyavirus; Snowshoe hare orthobunyavirus
GCA_002145805.1	11397	8	Chaco virus	BeAn42217	Viruses; Riboviria; Orthornavirae; Negarnaviricota; Haploviricotina; Monjiviricetes; Mononegavirales; Rhabdoviridae; Sripuvirus; Chaco sripuvirus
GCA_002145745.1	13294	7	Marco virus	BeAn40290	Viruses; Riboviria; Orthornavirae; Negarnaviricota; Haploviricotina; Monjiviricetes; Mononegavirales; Rhabdoviridae; Hapavirus; Marco hapavirus
GCA_000898555.1	12135	4	Aino virus	NA	Viruses; ssRNA viruses; ssRNA negative-strand viruses; Bunyaviridae; Orthobunyavirus; Shuni virus
GCA_000901655.1	66116	94	Pseudomonas phage NH4	NA	Viruses; dsDNA viruses, no RNA stage; Caudovirales; Myoviridae; unclassified Myoviridae
GCA_001021295.1	12650	4	Punta Toro virus	PAN472868	Viruses; ssRNA viruses; ssRNA negative-strand viruses; Bunyaviridae; Phlebovirus; Punta Toro phlebovirus
GCA_002755155.1	44789	68	Pseudomonas phage MR2992	NA	Viruses; Duplodnaviria; Heunggongvirae; Uroviricota; Caudoviricetes; Caudovirales; Podoviridae; Bruynoghevirus; unclassified Bruynoghevirus
GCA_000847345.1	11979	4	Rift Valley fever virus	ZH548	Viruses; ssRNA viruses; ssRNA negative-strand viruses; Bunyaviridae; Phlebovirus; Rift Valley fever phlebovirus
GCA_000854285.1	12488	3	Toscana virus	NA	Viruses; Riboviria; Orthornavirae; Negarnaviricota; Polyploviricotina; Ellioviricetes; Bunyavirales; Phenuiviridae; Phlebovirus; Toscana phlebovirus
GCA_000851865.1	11372	4	Uukuniemi virus	S23	Viruses; ssRNA viruses; ssRNA negative-strand viruses; Bunyaviridae; Phlebovirus; Uukuniemi phlebovirus
GCA_006446415.1	35558	32	Simian adenovirus A1139	NA	Viruses; Varidnaviria; Bamfordvirae; Preplasmiviricota; Tectiliviricetes; Rowavirales; Adenoviridae; Mastadenovirus; unclassified Simian adenoviruses; Simian adenovirus B
GCA_006446395.1	35580	31	Simian adenovirus A1163	NA	Viruses; Varidnaviria; Bamfordvirae; Preplasmiviricota; Tectiliviricetes; Rowavirales; Adenoviridae; Mastadenovirus; unclassified Simian adenoviruses; Simian adenovirus B
GCA_006446375.1	35726	34	Simian adenovirus A1173	NA	Viruses; Varidnaviria; Bamfordvirae; Preplasmiviricota; Tectiliviricetes; Rowavirales; Adenoviridae; Mastadenovirus; unclassified Simian adenoviruses; Simian adenovirus B
GCA_006446355.1	35649	32	Simian adenovirus A1258	NA	Viruses; Varidnaviria; Bamfordvirae; Preplasmiviricota; Tectiliviricetes; Rowavirales; Adenoviridae; Mastadenovirus; unclassified Simian adenoviruses; Simian adenovirus B
GCA_006446335.1	35705	33	Simian adenovirus A1285	NA	Viruses; Varidnaviria; Bamfordvirae; Preplasmiviricota; Tectiliviricetes; Rowavirales; Adenoviridae; Mastadenovirus; unclassified Simian adenoviruses; Simian adenovirus B
GCA_006446295.1	35578	33	Simian adenovirus A1312	NA	Viruses; Varidnaviria; Bamfordvirae; Preplasmiviricota; Tectiliviricetes; Rowavirales; Adenoviridae; Mastadenovirus; unclassified Simian adenoviruses; Simian adenovirus B
GCA_006446275.1	35540	32	Simian adenovirus A1327	NA	Viruses; Varidnaviria; Bamfordvirae; Preplasmiviricota; Tectiliviricetes; Rowavirales; Adenoviridae; Mastadenovirus; unclassified Simian adenoviruses; Simian adenovirus B
GCA_006446255.1	35557	33	Simian adenovirus A1335	NA	Viruses; Varidnaviria; Bamfordvirae; Preplasmiviricota; Tectiliviricetes; Rowavirales; Adenoviridae; Mastadenovirus; unclassified Simian adenoviruses; Simian adenovirus B
GCA_000895355.1	11245	2	Southern elephant seal virus	NA	Viruses; ssRNA viruses; ssRNA positive-strand viruses, no DNA stage; Togaviridae; Alphavirus
GCA_002831085.1	18232	3	Hazara virus	NA	Viruses; Riboviria; Orthornavirae; Negarnaviricota; Polyploviricotina; Ellioviricetes; Bunyavirales; Nairoviridae; Orthonairovirus; Hazara orthonairovirus
GCA_000842425.1	173591	252	Aeromonas virus 44RR2	NA	Viruses; dsDNA viruses, no RNA stage; Caudovirales; Myoviridae; Biquartavirus
GCA_000896895.1	26223	9	Commonmoorhen coronavirus HKU21	HKU218295	Viruses; ssRNA viruses; ssRNA positive-strand viruses, no DNA stage; Nidovirales; Coronaviridae; Coronavirinae; Deltacoronavirus; unclassified Deltacoronavirus
GCA_000894435.1	26689	9	Magpierobin coronavirus HKU18	HKU18chu3	Viruses; ssRNA viruses; ssRNA positive-strand viruses, no DNA stage; Nidovirales; Coronaviridae; Coronavirinae; Deltacoronavirus; unclassified Deltacoronavirus

gca	genome.size	proteome.size	phage.name	strain	Taxonomy
GCA_000896035.1	26077	8	Nightheron coronavirus HKU19	HKU196918	Viruses; ssRNA viruses; ssRNA positive-strand viruses, no DNA stage; Nidovirales; Coronaviridae; Coronavirinae; Deltacoronavirus; unclassified Deltacoronavirus
GCA_000895395.2	25430	7	Porcine coronavirus HKU15	HKU1544	Viruses; ssRNA viruses; ssRNA positive-strand viruses, no DNA stage; Nidovirales; Coronaviridae; Coronavirinae; Deltacoronavirus; unclassified Deltacoronavirus
GCA_000868165.1	26083	8	Sparrow coronavirus HKU17	HKU176124	Viruses; ssRNA viruses; ssRNA positive-strand viruses, no DNA stage; Nidovirales; Coronaviridae; Coronavirinae; Deltacoronavirus; unclassified Deltacoronavirus
GCA_000896875.1	26041	8	Whiteeye coronavirus HKU16	HKU166847	Viruses; ssRNA viruses; ssRNA positive-strand viruses, no DNA stage; Nidovirales; Coronaviridae; Coronavirinae; Deltacoronavirus; unclassified Deltacoronavirus
GCA_000895415.1	26227	10	Wigeon coronavirus HKU20	HKU209243	Viruses; ssRNA viruses; ssRNA positive-strand viruses, no DNA stage; Nidovirales; Coronaviridae; Coronavirinae; Deltacoronavirus; unclassified Deltacoronavirus
GCA_000863505.1	180500	292	Escherichia virus RB43	NA	Viruses; Duplodnaviria; Heunggongvirae; Uroviricota; Caudoviricetes; Caudovirales; Myoviridae; Tevenvirinae
GCA_000910195.2	247511	405	Vibrio phage nt1	NA	Viruses; dsDNA viruses, no RNA stage; Caudovirales; Myoviridae; Tevenvirinae; Schizot4virus
GCA_000851285.1	8477	4	Pelargonium zonate spot virus	tomato	Viruses; ssRNA viruses; ssRNA positive-strand viruses, no DNA stage; Bromoviridae; Anulavirus
GCA_010478905.1	7839	3	Norovirus catGIV2CU081210EUSA2010	NA	Viruses; Riboviria; Orthornavirae; Pisuviricota; Pisoniviricetes; Picornavirales; Caliciviridae; Norovirus; Norwalk virus; Norovirus GIV; Norovirus GIV.2
GCA_000896935.1	31100	11	Rabbit coronavirus HKU14	HKU141	Viruses; ssRNA viruses; ssRNA positive-strand viruses, no DNA stage; Nidovirales; Coronaviridae; Coronavirinae; Betacoronavirus; unclassified Betacoronavirus
GCA_013087115.1	1372	1	Tomato leaf curl New Delhi alphasatellite	NA	Viruses; Alphasatellitidae; Geminialphasatellitinae; Clecrusatellite
GCA_000926475.1	7353	3	Rabbit astrovirus TN22082010	NA	Viruses; ssRNA viruses; ssRNA positive-strand viruses, no DNA stage; Astroviridae; Mamastrovirus; unclassified Mamastrovirus
GCA_000852025.1	16740	4	Impatiens necrotic spot virus	NL07	Viruses; ssRNA viruses; ssRNA negative-strand viruses; Bunyaviridae; Tospovirus; Impatiens necrotic spot tospovirus
GCA_000898195.1	41164	50	Bacillus phage PBC1	NA	Viruses; dsDNA viruses, no RNA stage; Caudovirales; Siphoviridae; unclassified Siphoviridae
GCA_000898395.1	36847	53	Pseudomonas phage MP42	NA	Viruses; dsDNA viruses, no RNA stage; Caudovirales; Siphoviridae; Lambdavirus; unclassified Lambda-like viruses
GCA_000899855.1	33594	48	Weissella phage phiYS61	NA	Viruses; dsDNA viruses, no RNA stage; Caudovirales; Podoviridae; unclassified Podoviridae
GCA_000897175.1	280538	391	Pseudomonas phage Lu11	NA	Viruses; dsDNA viruses, no RNA stage; Caudovirales; Myoviridae; unclassified Myoviridae
GCA_002617325.1	76350	102	Vibrio phage SSP002	NA	Viruses; Duplodnaviria; Heunggongvirae; Uroviricota; Caudoviricetes; Caudovirales; Siphoviridae; Mardecavirus; Vibrio virus SSP002
GCA_000897415.1	26201	40	Leuconostoc phage Lmd1	NA	Viruses; dsDNA viruses, no RNA stage; Caudovirales; Siphoviridae; unclassified Siphoviridae
GCA_000901835.1	69391	127	Agrobacterium phage 7/7/1	NA	Viruses; dsDNA viruses, no RNA stage; Caudovirales; Myoviridae; unclassified Myoviridae
GCA_002624625.1	51094	87	Mycobacterium phage Twister	NA	Viruses; Duplodnaviria; Heunggongvirae; Uroviricota; Caudoviricetes; Caudovirales; Siphoviridae; Fromanvirus; Mycobacterium virus Twister
GCA_002624665.1	50332	85	Mycobacterium phage Tiger	NA	Viruses; Duplodnaviria; Heunggongvirae; Uroviricota; Caudoviricetes; Caudovirales; Siphoviridae; Fromanvirus; Mycobacterium virus Tiger
GCA_000900975.1	49390	70	Salinivibrio phage CW02	NA	Viruses; dsDNA viruses, no RNA stage; Caudovirales; Podoviridae; unclassified Podoviridae
GCA_000929535.1	17972	25	Enterococcus phage vB_Efae230P4	NA	Viruses; dsDNA viruses, no RNA stage; Caudovirales; Podoviridae; unclassified Podoviridae
GCA_000851705.1	10681	4	Lassa mammarenavirus	NA	Viruses; ssRNA viruses; ssRNA negative-strand viruses; Arenaviridae; Mammarenavirus
GCA_000896075.1	3765	1	Clitocybe odora virus	NA	Viruses; ssRNA viruses; ssRNA positive-strand viruses, no DNA stage; Narnaviridae; Mitovirus; unclassified Mitovirus
GCA_000904155.2	5305	7	Tomato rugose yellow leaf curl virus	A	Viruses; ssDNA viruses; Geminiviridae; Begomovirus
GCA_002602165.1	38689	51	Salmonella phage SPN9TCW	NA	Viruses; Duplodnaviria; Heunggongvirae; Uroviricota; Caudoviricetes; Caudovirales; Podoviridae; Uetakevirus; unclassified Uetakevirus
GCA_000920235.1	151924	281	Cronobacter phage CR9	NA	Viruses; dsDNA viruses, no RNA stage; Caudovirales; Myoviridae; Vequintavirinae; Cr3virus; Cronobacter virus CR9
GCA_000894715.1	149273	265	Cronobacter phage CR3	NA	Viruses; dsDNA viruses, no RNA stage; Caudovirales; Myoviridae; Vequintavirinae; Cr3virus; Cronobacter virus CR3
GCA_000851025.1	10056	4	Lymphocytic choriomeningitis mammarenavirus	Armstrong 53b	Viruses; ssRNA viruses; ssRNA negative-strand viruses; Arenaviridae; Mammarenavirus
GCA_000897195.1	18397	26	Clostridium phage phiCP7R	NA	Viruses; dsDNA viruses, no RNA stage; Caudovirales; Podoviridae; Picovirinae; unclassified Picovirinae
GCA_000899755.1	17972	28	Clostridium phage phiCPV4	NA	Viruses; dsDNA viruses, no RNA stage; Caudovirales; Podoviridae; Picovirinae; unclassified Picovirinae
GCA_000897315.1	18078	27	Clostridium phage phiZP2	NA	Viruses; dsDNA viruses, no RNA stage; Caudovirales; Podoviridae; Picovirinae; unclassified Picovirinae

gca	genome.size	proteome.size	phage.name	strain	Taxonomy
GCA_004790435.1	10454	3	Raphanus sativas chrysovirus 1	NA	Viruses; Riboviria; Orthornavirae; Duplornaviricota; Chrymotiviricetes; Ghabrivirales; Chrysoviridae; Alphachrysovirus; Raphanus sativus chrysovirus
GCA_002824985.1	2603	4	Chickpea chlorosis virusC	NA	Viruses; Monodnaviria; Shotokuvirae; Cressdnaviricota; Repensiviricetes; Geplafuvirales; Geminiviridae; Mastrevirus; Chickpea chlorosis virus
GCA_002825005.1	2590	4	Chickpea chlorosis virusE	NA	Viruses; Monodnaviria; Shotokuvirae; Cressdnaviricota; Repensiviricetes; Geplafuvirales; Geminiviridae; Mastrevirus; Chickpea chlorosis virus
GCA_002825025.1	2557	4	Chickpea yellows virus	NA	Viruses; Monodnaviria; Shotokuvirae; Cressdnaviricota; Repensiviricetes; Geplafuvirales; Geminiviridae; Mastrevirus
GCA_000853725.1	10635	4	Machupo mammarenavirus	Carvallo	Viruses; ssRNA viruses; ssRNA negative-strand viruses; Arenaviridae; Mammarenavirus
GCA_000853285.1	10534	4	Tacaribe mammarenavirus	NA	Viruses; ssRNA viruses; ssRNA negative-strand viruses; Arenaviridae; Mammarenavirus
GCA_000902635.1	42391	58	Salmonella phage Ent1	NA	Viruses; dsDNA viruses, no RNA stage; Caudovirales; Siphoviridae; Guernseyvirinae; Jerseyvirus
GCA_000894495.1	7611	2	Tianjin totivirus	NA	Viruses; dsRNA viruses; Totiviridae; unclassified Totiviridae
GCA_000858025.1	8295	3	Reticuloendotheliosis virus	HA9901	Viruses; Riboviria; Pararnavirae; Artverviricota; Revtraviricetes; Ortervirales; Retroviridae; Orthoretrovirinae; Gammaretrovirus
GCA_002826965.1	7891	6	Eidolon helvum papillomavirus 1	NA	Viruses; Monodnaviria; Shotokuvirae; Cossaviricota; Papovaviricetes; Zurhausenvirales; Papillomaviridae; Firstpapillomavirinae; Dyoupsilonpapillomavirus; Dyoupsilonpapillomavirus 1
GCA_000920535.1	7467	5	Equus asinus papillomavirus 1	NA	Viruses; dsDNA viruses, no RNA stage; Papillomaviridae; Dyochipapillomavirus; Dyochipapillomavirus 1
GCA_000919755.1	7256	6	Rupicapra rupicapra papillomavirus 1	NA	Viruses; dsDNA viruses, no RNA stage; Papillomaviridae; Dyokappapapillomavirus; Dyokappapapillomavirus 2
GCA_002827145.1	7519	6	Vulpes vulpes papillomavirus 1	NA	Viruses; Monodnaviria; Shotokuvirae; Cossaviricota; Papovaviricetes; Zurhausenvirales; Papillomaviridae; Firstpapillomavirinae; Treisetapapillomavirus; Treisetapapillomavirus 1
GCA_000896975.1	2359	3	Gyrovirus GyV3	FecGy	Viruses; ssDNA viruses; Circoviridae; Gyrovirus; unclassified Gyrovirus
GCA_004788255.1	12965	5	Simian foamy virus	NA	Viruses; Riboviria; Pararnavirae; Artverviricota; Revtraviricetes; Ortervirales; Retroviridae; Spumaretrovirinae; Spumavirus
GCA_001505235.1	32414	59	Lactococcus virus ASCC191	NA	Viruses; Duplodnaviria; Heunggongvirae; Uroviricota; Caudoviricetes; Caudovirales; Siphoviridae; Skunavirus
GCA_002602185.1	32582	58	Lactococcus virus ASCC273	NA	Viruses; Duplodnaviria; Heunggongvirae; Uroviricota; Caudoviricetes; Caudovirales; Siphoviridae; Skunavirus
GCA_002602205.1	32023	57	Lactococcus virus ASCC281	NA	Viruses; Duplodnaviria; Heunggongvirae; Uroviricota; Caudoviricetes; Caudovirales; Siphoviridae; Skunavirus
GCA_002592445.1	33178	57	Lactococcus phage ASCC284	NA	Viruses; Duplodnaviria; Heunggongvirae; Uroviricota; Caudoviricetes; Caudovirales; Siphoviridae; Skunavirus; unclassified Skunavirus
GCA_002592465.1	32581	58	Lactococcus phage ASCC287	NA	Viruses; Duplodnaviria; Heunggongvirae; Uroviricota; Caudoviricetes; Caudovirales; Siphoviridae; Skunavirus; unclassified Skunavirus
GCA_002592485.1	32546	55	Lactococcus phage ASCC310	NA	Viruses; Duplodnaviria; Heunggongvirae; Uroviricota; Caudoviricetes; Caudovirales; Siphoviridae; Skunavirus; unclassified Skunavirus
GCA_002592505.1	32277	58	Lactococcus phage ASCC324	NA	Viruses; Duplodnaviria; Heunggongvirae; Uroviricota; Caudoviricetes; Caudovirales; Siphoviridae; Skunavirus; unclassified Skunavirus
GCA_002592525.1	32275	58	Lactococcus phage ASCC337	NA	Viruses; Duplodnaviria; Heunggongvirae; Uroviricota; Caudoviricetes; Caudovirales; Siphoviridae; Skunavirus; unclassified Skunavirus
GCA_002592545.1	32545	54	Lactococcus phage ASCC356	NA	Viruses; Duplodnaviria; Heunggongvirae; Uroviricota; Caudoviricetes; Caudovirales; Siphoviridae; Skunavirus; unclassified Skunavirus
GCA_002592565.1	32052	57	Lactococcus phage ASCC358	NA	Viruses; Duplodnaviria; Heunggongvirae; Uroviricota; Caudoviricetes; Caudovirales; Siphoviridae; Skunavirus; unclassified Skunavirus
GCA_002592585.1	32048	57	Lactococcus phage ASCC365	NA	Viruses; Duplodnaviria; Heunggongvirae; Uroviricota; Caudoviricetes; Caudovirales; Siphoviridae; Skunavirus; unclassified Skunavirus
GCA_002592605.1	32276	58	Lactococcus phage ASCC368	NA	Viruses; Duplodnaviria; Heunggongvirae; Uroviricota; Caudoviricetes; Caudovirales; Siphoviridae; Skunavirus; unclassified Skunavirus
GCA_002592625.1	32239	58	Lactococcus phage ASCC395	NA	Viruses; Duplodnaviria; Heunggongvirae; Uroviricota; Caudoviricetes; Caudovirales; Siphoviridae; Skunavirus; unclassified Skunavirus
GCA_002592645.1	32241	59	Lactococcus phage ASCC397	NA	Viruses; Duplodnaviria; Heunggongvirae; Uroviricota; Caudoviricetes; Caudovirales; Siphoviridae; Skunavirus; unclassified Skunavirus
GCA_002592665.1	32180	59	Lactococcus phage ASCC406	NA	Viruses; Duplodnaviria; Heunggongvirae; Uroviricota; Caudoviricetes; Caudovirales; Siphoviridae; Skunavirus; unclassified Skunavirus
GCA_002592685.1	32583	58	Lactococcus phage ASCC454	NA	Viruses; Duplodnaviria; Heunggongvirae; Uroviricota; Caudoviricetes; Caudovirales; Siphoviridae; Skunavirus; unclassified Skunavirus
GCA_002592705.1	32242	59	Lactococcus phage ASCC460	NA	Viruses; Duplodnaviria; Heunggongvirae; Uroviricota; Caudoviricetes; Caudovirales; Siphoviridae; Skunavirus; unclassified Skunavirus
GCA_002602225.1	31772	56	Lactococcus virus ASCC465	NA	Viruses; Duplodnaviria; Heunggongvirae; Uroviricota; Caudoviricetes; Caudovirales; Siphoviridae; Skunavirus
GCA_002592405.1	31772	57	Lactococcus phage ASCC473	NA	Viruses; Duplodnaviria; Heunggongvirae; Uroviricota; Caudoviricetes; Caudovirales; Siphoviridae; Skunavirus; unclassified Skunavirus

gca	genome.size	proteome.size	phage.name	strain	Taxonomy
GCA_002592725.1	32242	59	Lactococcus phage ASCC476	NA	Viruses; Duplodnaviria; Heunggongvirae; Uroviricota; Caudoviricetes; Caudovirales; Siphoviridae; Skunavirus; unclassified Skunavirus
GCA_002592425.1	30876	54	Lactococcus phage ASCC489	NA	Viruses; Duplodnaviria; Heunggongvirae; Uroviricota; Caudoviricetes; Caudovirales; Siphoviridae; Skunavirus; unclassified Skunavirus
GCA_002592745.1	31774	56	Lactococcus phage ASCC497	NA	Viruses; Duplodnaviria; Heunggongvirae; Uroviricota; Caudoviricetes; Caudovirales; Siphoviridae; Skunavirus; unclassified Skunavirus
GCA_002592765.1	32275	58	Lactococcus phage ASCC502	NA	Viruses; Duplodnaviria; Heunggongvirae; Uroviricota; Caudoviricetes; Caudovirales; Siphoviridae; Skunavirus; unclassified Skunavirus
GCA_002592785.1	32249	58	Lactococcus phage ASCC506	NA	Viruses; Duplodnaviria; Heunggongvirae; Uroviricota; Caudoviricetes; Caudovirales; Siphoviridae; Skunavirus; unclassified Skunavirus
GCA_002592805.1	32345	58	Lactococcus phage ASCC527	NA	Viruses; Duplodnaviria; Heunggongvirae; Uroviricota; Caudoviricetes; Caudovirales; Siphoviridae; Skunavirus; unclassified Skunavirus
GCA_002592825.1	31773	57	Lactococcus phage ASCC531	NA	Viruses; Duplodnaviria; Heunggongvirae; Uroviricota; Caudoviricetes; Caudovirales; Siphoviridae; Skunavirus; unclassified Skunavirus
GCA_002602245.1	32384	55	Lactococcus virus ASCC532	NA	Viruses; Duplodnaviria; Heunggongvirae; Uroviricota; Caudoviricetes; Caudovirales; Siphoviridae; Skunavirus
GCA_002592845.1	32275	58	Lactococcus phage ASCC544	NA	Viruses; Duplodnaviria; Heunggongvirae; Uroviricota; Caudoviricetes; Caudovirales; Siphoviridae; Skunavirus; unclassified Skunavirus
GCA_000848165.1	8482	5	Bovine immunodeficiency virus	NA	Viruses; Riboviria; Pararnavirae; Artverviricota; Revtraviricetes; Ortervirales; Retroviridae; Orthoretrovirinae; Lentivirus
GCA_000906595.1	47252	44	Staphylococcus phage phi7401PVL	JCSC7401	Viruses; dsDNA viruses, no RNA stage; Caudovirales; Siphoviridae; unclassified Siphoviridae
GCA_000917435.1	5275	4	Porcine bocavirus	NA	Viruses; ssDNA viruses; Parvoviridae; Parvovirinae; Bocaparvovirus; unclassified Bocaparvovirus
GCA_006446035.1	35537	34	Simian adenovirus B	NA	Viruses; dsDNA viruses, no RNA stage; Adenoviridae; Mastadenovirus; unclassified Simian adenoviruses
GCA_003179195.1	7352	7	Human papillomavirus 155	NA	Viruses; Monodnaviria; Shotokuvirae; Cossaviricota; Papovaviricetes; Zurhausenvirales; Papillomaviridae; Firstpapillomavirinae; Gammapapillomavirus; Gammapapillomavirus 7
GCA_000857525.1	9189	6	Caprine arthritis encephalitis virus	NA	Viruses; Riboviria; Pararnavirae; Artverviricota; Revtraviricetes; Ortervirales; Retroviridae; Orthoretrovirinae; Lentivirus
GCA_000849165.1	9256	6	Ovine lentivirus	NA	Viruses; Riboviria; Pararnavirae; Artverviricota; Revtraviricetes; Ortervirales; Retroviridae; Orthoretrovirinae; Lentivirus; unclassified Lentivirus
GCA_000854865.1	9474	6	Feline immunodeficiency virus	Petaluma	Viruses; Riboviria; Pararnavirae; Artverviricota; Revtraviricetes; Ortervirales; Retroviridae; Orthoretrovirinae; Lentivirus
GCA_000864765.1	9181	9	Human immunodeficiency virus 1	NA	Viruses; Riboviria; Pararnavirae; Artverviricota; Revtraviricetes; Ortervirales; Retroviridae; Orthoretrovirinae; Lentivirus
GCA_000904635.1	4655	2	Xanthophyllomyces dendrorhous virus L1A	NA	Viruses; dsRNA viruses; Totiviridae; Totivirus
GCA_002830745.1	4619	2	Xanthophyllomyces dendrorhous virus L1B	NA	Viruses; Riboviria; Orthornavirae; Duplornaviricota; Chrymotiviricetes; Ghabrivirales; Totiviridae; Totivirus
GCA_000899275.1	11902	5	Ikoma lyssavirus	NA	Viruses; ssRNA viruses; ssRNA negative-strand viruses; Mononegavirales; Rhabdoviridae; Lyssavirus
GCA_008792725.1	7588	1	Ovine hungarovirus OHUV12009HUN	NA	Viruses; Riboviria; Orthornavirae; Pisuviricota; Pisoniviricetes; Picornavirales; Picornaviridae; Hunnivirus; Hunnivirus A; Ovine hungarovirus 1
GCA_000898515.1	39974	52	Cronobacter phage phiES15	NA	Viruses; dsDNA viruses, no RNA stage; Caudovirales; Siphoviridae; unclassified Siphoviridae
GCA_000906755.1	38682	40	Vibrio phage VvAW1	NA	Viruses; dsDNA viruses, no RNA stage; Caudovirales; Podoviridae; unclassified Podoviridae
GCA_000899015.1	35700	59	Persicivirga phage P12024S	NA	Viruses; dsDNA viruses, no RNA stage; unclassified dsDNA phages
GCA_000898415.1	35652	58	Persicivirga phage P12024L	NA	Viruses; dsDNA viruses, no RNA stage; unclassified dsDNA phages
GCA_000905055.1	1670	2	Acartia tonsa copepod circovirus	NA	Viruses; ssDNA viruses; Circoviridae; Circovirus; unclassified Circovirus
GCA_000899835.1	39783	70	Salmonella phage vB_SemP_Emek	NA	Viruses; dsDNA viruses, no RNA stage; Caudovirales; Podoviridae; P22virus; unclassified P22likevirus
GCA_000899875.1	49116	79	Salmonella phage vB_SosS_Oslo	NA	Viruses; dsDNA viruses, no RNA stage; Caudovirales; Siphoviridae; unclassified Siphoviridae
GCA_013087125.1	12654	10	Maize yellow striate virus	NA	Viruses; Riboviria; Orthornavirae; Negarnaviricota; Haploviricotina; Monjiviricetes; Mononegavirales; Rhabdoviridae; Cytorhabdovirus; Maize yellow striate cytorhabdovirus
GCA_000917355.1	33525	46	Stenotrophomonas phage Smp131	NA	Viruses; dsDNA viruses, no RNA stage; Caudovirales; Myoviridae; Peduovirinae; P2virus; unclassified P2likevirus
GCA_002597905.1	51370	89	Mycobacterium phage Flux	NA	Viruses; Duplodnaviria; Heunggongvirae; Uroviricota; Caudoviricetes; Caudovirales; Siphoviridae; Fromanvirus; Mycobacterium virus Peaches
GCA_000917635.1	54470	107	Mycobacterium virus Avani	NA	Viruses; Duplodnaviria; Heunggongvirae; Uroviricota; Caudoviricetes; Caudovirales; Siphoviridae; Cheoctovirus
GCA_000919355.1	53684	99	Mycobacterium phage Aeneas	NA	Viruses; dsDNA viruses, no RNA stage; Caudovirales; Siphoviridae; unclassified Siphoviridae

gca	genome.size	proteome.size	phage.name	strain	Taxonomy
GCA_000900875.1	45552	42	Staphylococcus phage SMSAP5	NA	Viruses; dsDNA viruses, no RNA stage; Caudovirales; Siphoviridae; unclassified Siphoviridae
GCA_000896255.1	43786	43	Staphylococcus phage TEM123	NA	Viruses; dsDNA viruses, no RNA stage; Caudovirales; Siphoviridae; unclassified Siphoviridae
GCA_000905575.1	133057	178	Listeria phage vB_LmoM_AG20	NA	Viruses; dsDNA viruses, no RNA stage; Caudovirales; Myoviridae; Spounavirinae; Spo1virus; unclassified SPO1-like viruses
GCA_000849485.1	6298	4	Wasabi mottle virus	wasabi	Viruses; ssRNA viruses; ssRNA positive-strand viruses, no DNA stage; Virgaviridae; Tobamovirus
GCA_002815235.1	16050	8	Feline morbillivirus	761U	Viruses; ssRNA viruses; ssRNA negative-strand viruses; Mononegavirales; Paramyxoviridae; Morbillivirus; unclassified Morbillivirus
GCA_002024775.1	9136	3	Loreto virus	394083	Viruses; ssRNA viruses; ssRNA positive-strand viruses, no DNA stage; unclassified ssRNA positive-strand viruses; Negevirus
GCA_001661735.1	9537	3	Negev virus	PL17	Viruses; ssRNA viruses; ssRNA positive-strand viruses, no DNA stage; unclassified ssRNA positive-strand viruses; Negevirus
GCA_002024735.1	10050	3	Piura virus	CoR29	Viruses; ssRNA viruses; ssRNA positive-strand viruses, no DNA stage; unclassified ssRNA positive-strand viruses; Negevirus
GCA_013086535.1	13120	4	Saint Floris virus	Dak ANB 512	Viruses; unclassified viruses
GCA_001430335.1	2776	6	Vinca leaf curl virus	NA	Viruses; ssDNA viruses; Geminiviridae; Begomovirus; unclassified Begomovirus
GCA_000856385.1	10359	9	Human immunodeficiency virus 2	NA	Viruses; Riboviria; Pararnavirae; Artverviricota; Revtraviricetes; Ortervirales; Retroviridae; Orthoretrovirinae; Lentivirus
GCA_003092855.1	23365	12	Duck reovirus	HN5d	Viruses; Riboviria; Orthornavirae; Duplornaviricota; Resentoviricetes; Reovirales; Reoviridae; Spinareovirinae; Orthoreovirus; Avian orthoreovirus
GCA_000840505.1	5155	7	Rhynchosia golden mosaic virus	NA	Viruses; ssDNA viruses; Geminiviridae; Begomovirus
GCA_003083955.1	19520	11	African horse sickness virus 3	NA	Viruses; Riboviria; Orthornavirae; Duplornaviricota; Resentoviricetes; Reovirales; Reoviridae; Sedoreovirinae; Orbivirus; African horse sickness virus
GCA_004786995.1	7656	2	Turkey calicivirus	NA	Viruses; Riboviria; Orthornavirae; Pisuviricota; Pisoniviricetes; Picornavirales; Caliciviridae; Nacovirus; Nacovirus A
GCA_001963095.1	8176	2	Chicken calicivirus	RSBR2015	Viruses; ssRNA viruses; ssRNA positive-strand viruses, no DNA stage; Caliciviridae; unclassified Caliciviridae
GCA_000863925.1	9623	5	Simian immunodeficiency virus	NA	Viruses; Riboviria; Pararnavirae; Artverviricota; Revtraviricetes; Ortervirales; Retroviridae; Orthoretrovirinae; Lentivirus
GCA_003191605.1	10277	11	Simian immunodeficiency virus	NA	Viruses; Riboviria; Pararnavirae; Artverviricota; Revtraviricetes; Ortervirales; Retroviridae; Orthoretrovirinae; Lentivirus
GCA_003192965.1	9676	8	Simian immunodeficiency virus agm	ZMB	Viruses; Riboviria; Pararnavirae; Artverviricota; Revtraviricetes; Ortervirales; Retroviridae; Orthoretrovirinae; Lentivirus; Simian immunodeficiency virus
GCA_002037735.1	11025	5	Mogiana tick virus	NA	Viruses; ssRNA viruses; ssRNA positive-strand viruses, no DNA stage; Flaviviridae; unclassified Flaviviridae
GCA_000898895.1	53996	87	Enterococcus phage BC611	NA	Viruses; dsDNA viruses, no RNA stage; Caudovirales; Siphoviridae; Sap6virus
GCA_002867775.1	12800	8	Influenza D virus DswineOklahoma13342011	NA	Viruses; Riboviria; Orthornavirae; Negarnaviricota; Polyploviricotina; Insthoviricetes; Articulavirales; Orthomyxoviridae; Deltainfluenzavirus; Influenza D virus
GCA_000897215.1	167292	173	Halocynthia phage JM2012	NA	Viruses; dsDNA viruses, no RNA stage; Caudovirales; Myoviridae; unclassified Myoviridae
GCA_000906535.1	1764	2	Labidocera aestiva circovirus	NA	Viruses; ssDNA viruses; Circoviridae; Circovirus; unclassified Circovirus
GCA_000909375.1	38275	69	Listeria phage LP0302	NA	Viruses; dsDNA viruses, no RNA stage; Caudovirales; Siphoviridae; Psavirus; Listeria virus LP302
GCA_000921915.1	67150	114	Listeria phage LP026	NA	Viruses; dsDNA viruses, no RNA stage; Caudovirales; Siphoviridae; P70virus; Listeria virus LP26
GCA_000910455.1	64756	114	Listeria phage LP037	NA	Viruses; dsDNA viruses, no RNA stage; Caudovirales; Siphoviridae; P70virus; Listeria virus LP37
GCA_000907955.1	65132	113	Listeria phage LP110	NA	Viruses; dsDNA viruses, no RNA stage; Caudovirales; Siphoviridae; P70virus; Listeria virus LP110
GCA_000910415.1	42215	59	Salmonella phage FSL SP031	NA	Viruses; dsDNA viruses, no RNA stage; Caudovirales; Siphoviridae; Guernseyvirinae; Sp31virus; Salmonella virus SP31
GCA_000907935.1	59746	71	Salmonella virus FSLSP030	NA	Viruses; Duplodnaviria; Heunggongvirae; Uroviricota; Caudoviricetes; Caudovirales; Siphoviridae; Chivirus
GCA_002602265.1	59815	71	Salmonella phage FSL SP039	NA	Viruses; Duplodnaviria; Heunggongvirae; Uroviricota; Caudoviricetes; Caudovirales; Siphoviridae; Chivirus; Salmonella virus FSLSP030
GCA_000909515.1	59454	70	Salmonella virus FSLSP088	NA	Viruses; Duplodnaviria; Heunggongvirae; Uroviricota; Caudoviricetes; Caudovirales; Siphoviridae; Chivirus
GCA_002602285.1	59245	71	Salmonella phage FSL SP124	NA	Viruses; Duplodnaviria; Heunggongvirae; Uroviricota; Caudoviricetes; Caudovirales; Siphoviridae; Chivirus; Salmonella virus FSLSP088
GCA_000908895.1	72394	86	Salmonella phage FSL SP058	NA	Viruses; dsDNA viruses, no RNA stage; Caudovirales; Podoviridae; N4virus; unclassified N4likevirus

gca	genome.size	proteome.size	phage.name	strain	Taxonomy
GCA_000909555.1	72098	82	Salmonella phage FSL SP076	NA	Viruses; dsDNA viruses, no RNA stage; Caudovirales; Podoviridae; N4virus; unclassified N4likevirus
GCA_000921135.1	133048	177	Listeria phage LP048	NA	Viruses; dsDNA viruses, no RNA stage; Caudovirales; Myoviridae; unclassified Myoviridae
GCA_002604405.1	135764	188	Listeria phage LP124	NA	Viruses; Duplodnaviria; Heunggongvirae; Uroviricota; Caudoviricetes; Caudovirales; Herelleviridae; Jasinkavirinae; Pecentumvirus; Listeria virus LP064
GCA_000908915.1	135281	189	Listeria phage LP125	NA	Viruses; dsDNA viruses, no RNA stage; Caudovirales; Myoviridae; Spounavirinae; P100virus; Listeria virus P100
GCA_000907915.1	29742	40	Salmonella phage FSLSP004	NA	Viruses; Duplodnaviria; Heunggongvirae; Uroviricota; Caudoviricetes; Caudovirales; Myoviridae; Peduovirinae; Stockinghallvirus; Salmonella virus FSLSP004
GCA_000894555.1	2783	4	Wheat dwarf India virus	NA	Viruses; ssDNA viruses; Geminiviridae; Mastrevirus
GCA_000896155.1	5331	4	Feline bocavirus	HK797F	Viruses; ssDNA viruses; Parvoviridae; Parvovirinae; Bocaparvovirus; Carnivore bocaparvovirus 3
GCA_000850005.1	7462	5	Jaagsiekte sheep retrovirus	NA	Viruses; Riboviria; Pararnavirae; Artverviricota; Revtraviricetes; Ortervirales; Retroviridae; Orthoretrovirinae; Betaretrovirus
GCA_000898135.1	52474	94	Mycobacterium phage SWU1	NA	Viruses; dsDNA viruses, no RNA stage; Caudovirales; Siphoviridae; L5virus
GCA_000904615.1	1335	1	Ageratum leaf curl betasatellite	NA	Viruses; Tolecusatellitidae; Betasatellite; unclassified Betasatellite
GCA_002623005.1	57899	74	Pseudomonas phage PaMx25	NA	Viruses; Duplodnaviria; Heunggongvirae; Uroviricota; Caudoviricetes; Caudovirales; Siphoviridae; Nipunavirus; Pseudomonas virus PaMx25
GCA_002601365.1	36570	56	Pseudomonas phage PaMx73	NA	Viruses; Duplodnaviria; Heunggongvirae; Uroviricota; Caudoviricetes; Caudovirales; Siphoviridae; Casadabanvirus; Pseudomonas virus D3112
GCA_001503615.1	59878	81	Pseudomonas phage PaMx11	NA	Viruses; dsDNA viruses, no RNA stage; Caudovirales; Siphoviridae; unclassified Siphoviridae
GCA_001505875.1	43225	56	Pseudomonas phage PaMx42	NA	Viruses; dsDNA viruses, no RNA stage; Caudovirales; Siphoviridae; unclassified Siphoviridae
GCA_000846425.1	8805	5	Mouse mammary tumor virus	NA	Viruses; Riboviria; Pararnavirae; Artverviricota; Revtraviricetes; Ortervirales; Retroviridae; Orthoretrovirinae; Betaretrovirus
GCA_004289415.1	7357	6	Gammapapillomavirus 6	NA	Viruses; Monodnaviria; Shotokuvirae; Cossaviricota; Papovaviricetes; Zurhausenvirales; Papillomaviridae; Firstpapillomavirinae; Gammapapillomavirus
GCA_004289295.1	7410	7	Gammapapillomavirus 7	NA	Viruses; Monodnaviria; Shotokuvirae; Cossaviricota; Papovaviricetes; Zurhausenvirales; Papillomaviridae; Firstpapillomavirinae; Gammapapillomavirus
GCA_004289195.1	7273	7	Gammapapillomavirus 8	NA	Viruses; Monodnaviria; Shotokuvirae; Cossaviricota; Papovaviricetes; Zurhausenvirales; Papillomaviridae; Firstpapillomavirinae; Gammapapillomavirus
GCA_004288755.1	7257	7	Gammapapillomavirus 9	NA	Viruses; Monodnaviria; Shotokuvirae; Cossaviricota; Papovaviricetes; Zurhausenvirales; Papillomaviridae; Firstpapillomavirinae; Gammapapillomavirus
GCA_004288735.1	7367	7	Gammapapillomavirus 10	NA	Viruses; Monodnaviria; Shotokuvirae; Cossaviricota; Papovaviricetes; Zurhausenvirales; Papillomaviridae; Firstpapillomavirinae; Gammapapillomavirus
GCA_003179795.1	8029	9	Deltapapillomavirus 5	NA	Viruses; Monodnaviria; Shotokuvirae; Cossaviricota; Papovaviricetes; Zurhausenvirales; Papillomaviridae; Firstpapillomavirinae; Deltapapillomavirus
GCA_000898035.1	10057	1	Tomato necrotic stunt virus	MX9354	Viruses; ssRNA viruses; ssRNA positive-strand viruses, no DNA stage; Potyviridae; Potyvirus; unclassified Potyvirus
GCA_000901415.1	1352	1	Tomato leaf curl Yemen betasatellite	NA	Viruses; Tolecusatellitidae; Betasatellite
GCA_000912135.1	8507	3	Baboon endogenous virus strain M7	NA	Viruses; Riboviria; Pararnavirae; Artverviricota; Revtraviricetes; Ortervirales; Retroviridae; Orthoretrovirinae; Gammaretrovirus; unclassified Gammaretrovirus; Baboon endogenous virus
GCA_000897435.1	44988	68	Croceibacter phage P2559S	NA	Viruses; dsDNA viruses, no RNA stage; Caudovirales; Siphoviridae; unclassified Siphoviridae
GCA_000899035.1	31766	54	Marinomonas phage P12026	NA	Viruses; dsDNA viruses, no RNA stage; unclassified dsDNA phages
GCA_002602305.1	51440	88	Mycobacterium phage ICleared	NA	Viruses; Duplodnaviria; Heunggongvirae; Uroviricota; Caudoviricetes; Caudovirales; Siphoviridae; Fromanvirus; unclassified Fromanvirus
GCA_002598505.1	154466	232	Mycobacterium phage Ava3	NA	Viruses; Duplodnaviria; Heunggongvirae; Uroviricota; Caudoviricetes; Caudovirales; Myoviridae; Bixzunavirus; Mycobacterium virus Bxz1
GCA_002755175.1	37432	49	Yersinia phage Y	NA	Viruses; Duplodnaviria; Heunggongvirae; Uroviricota; Caudoviricetes; Caudovirales; Autographiviridae; Studiervirinae; Teseptimavirus; unclassified Teseptimavirus
GCA_000851085.1	8513	3	Canine vesivirus	NA	Viruses; Riboviria; Orthornavirae; Pisuviricota; Pisoniviricetes; Picornavirales; Caliciviridae; Vesivirus; unclassified Vesivirus
GCA_000923195.1	6677	5	Pitaya virus X	NA	Viruses; ssRNA viruses; ssRNA positive-strand viruses, no DNA stage; Tymovirales; Alphaflexiviridae; Potexvirus; unclassified Potexvirus
GCA_002633605.1	39415	44	Yersinia phage YpPG	NA	Viruses; Duplodnaviria; Heunggongvirae; Uroviricota; Caudoviricetes; Caudovirales; Autographiviridae; Studiervirinae; Berlinvirus; unclassified Berlinvirus
GCA_002991145.1	38284	52	Yersinia phage YpPR	NA	Viruses; Duplodnaviria; Heunggongvirae; Uroviricota; Caudoviricetes; Caudovirales; Autographiviridae; Studiervirinae; Teseptimavirus; unclassified Teseptimavirus
GCA_002991125.1	37714	46	Yersinia phage YpPY	NA	Viruses; Duplodnaviria; Heunggongvirae; Uroviricota; Caudoviricetes; Caudovirales; Autographiviridae; Studiervirinae; Teseptimavirus; Yersinia virus YpPY

gca	genome.size	proteome.size	phage.name	strain	Taxonomy
GCA_002991165.1	38288	52	Yersinia phage YpsPG	NA	Viruses; Duplodnaviria; Heunggongvirae; Uroviricota; Caudoviricetes; Caudovirales; Autographiviridae; Studiervirinae; Teseptimavirus; Yersinia virus YpsPG
GCA_000850105.1	8448	2	Feline leukemia virus	Rickard subgroup A	Viruses; Riboviria; Pararnavirae; Artverviricota; Revtraviricetes; Ortervirales; Retroviridae; Orthoretrovirinae; Gammaretrovirus
GCA_000898055.1	8601	6	American hop latent virus	NA	Viruses; ssRNA viruses; ssRNA positive-strand viruses, no DNA stage; Tymovirales; Betaflexiviridae; Quinvirinae; Carlavirus
GCA_000899335.1	103299	130	Salmonella phage SSU5	NA	Viruses; dsDNA viruses, no RNA stage; Caudovirales; Siphoviridae; unclassified Siphoviridae
GCA_000848265.1	5894	1	Abelson murine leukemia virus	NA	Viruses; Riboviria; Pararnavirae; Artverviricota; Revtraviricetes; Ortervirales; Retroviridae; Orthoretrovirinae; Gammaretrovirus; unclassified Gammaretrovirus
GCA_000859065.1	8323	3	Friend murine leukemia virus	FB29	Viruses; Riboviria; Pararnavirae; Artverviricota; Revtraviricetes; Ortervirales; Retroviridae; Orthoretrovirinae; Gammaretrovirus; Murine leukemia virus
GCA_002648855.1	17421	11	Rotavirus A IAL28xUK reassortant UKg9IAL28	NA	Viruses; Riboviria; Orthornavirae; Duplornaviricota; Resentoviricetes; Reovirales; Reoviridae; Sedoreovirinae; Rotavirus; Rotavirus A; Rotavirus A reassortants
GCA_002661195.1	17418	11	Rotavirus A WaxUK reassortant UKg4Wa	NA	Viruses; Riboviria; Orthornavirae; Duplornaviricota; Resentoviricetes; Reovirales; Reoviridae; Sedoreovirinae; Rotavirus; Rotavirus A; Rotavirus A reassortants
GCA_000854185.1	8332	3	Moloney murine leukemia virus	NA	Viruses; Riboviria; Pararnavirae; Artverviricota; Revtraviricetes; Ortervirales; Retroviridae; Orthoretrovirinae; Gammaretrovirus; Murine leukemia virus
GCA_000849425.1	5833	2	Moloney murine sarcoma virus	NA	Viruses; Riboviria; Pararnavirae; Artverviricota; Revtraviricetes; Ortervirales; Retroviridae; Orthoretrovirinae; Gammaretrovirus
GCA_008786875.1	6950	1	Human cosavirus A19	PK6187	Viruses; Riboviria; Orthornavirae; Pisuviricota; Pisoniviricetes; Picornavirales; Picornaviridae; Cosavirus; Cosavirus A; Human cosavirus A
GCA_008786925.1	6635	1	Human cosavirus A20	NG263	Viruses; Riboviria; Orthornavirae; Pisuviricota; Pisoniviricetes; Picornavirales; Picornaviridae; Cosavirus; Cosavirus A; Human cosavirus A
GCA_000897075.1	9124	2	Sclerotinia sclerotiorum dsRNA mycovirusL	NA	Viruses; dsRNA viruses; unclassified dsRNA viruses
GCA_008785925.1	7123	1	Teschovirus A	wild boarWB2CTV2011HUN	Viruses; Riboviria; Orthornavirae; Pisuviricota; Pisoniviricetes; Picornavirales; Picornaviridae; Teschovirus
GCA_000849885.1	6296	2	Friend spleen focusforming virus	NA	Viruses; Riboviria; Pararnavirae; Artverviricota; Revtraviricetes; Ortervirales; Retroviridae; Orthoretrovirinae; Gammaretrovirus; unclassified Gammaretrovirus; Spleen focus-forming virus
GCA_002755195.1	34719	52	Mannheimia phage vB_MhM_1152AP	NA	Viruses; Duplodnaviria; Heunggongvirae; Uroviricota; Caudoviricetes; Caudovirales; Myoviridae; Peduovirinae; Baylorvirus; Mannheimia virus PHL101
GCA_000894615.1	2882	4	Maize streak Reunion virus	NA	Viruses; ssDNA viruses; Geminiviridae; Mastrevirus
GCA_002647735.1	18801	11	Rotavirus A HuBELBE20012009G9P6	NA	Viruses; Riboviria; Orthornavirae; Duplornaviricota; Resentoviricetes; Reovirales; Reoviridae; Sedoreovirinae; Rotavirus; Rotavirus A; Rotavirus G9
GCA_000847465.1	3811	1	Murine osteosarcoma virus	NA	Viruses; Riboviria; Pararnavirae; Artverviricota; Revtraviricetes; Ortervirales; Retroviridae; Orthoretrovirinae; Gammaretrovirus; unclassified Gammaretrovirus
GCA_000897135.1	43788	57	Salisaeta icosahedral phage 1	NA	Viruses; unclassified viruses; unclassified DNA viruses; unclassified dsDNA phages
GCA_000899675.1	3068	2	Persimmon cryptic virus	NA	Viruses; dsRNA viruses; Partitiviridae; unclassified Partitiviridae
GCA_001595715.1	7527	3	Norovirus HuGIV1LakeMacquarieNSW268O2010AU	NA	Viruses; Riboviria; Orthornavirae; Pisuviricota; Pisoniviricetes; Picornavirales; Caliciviridae; Norovirus; Norwalk virus; Norovirus GIV; Norovirus GIV.1
GCA_000873665.1	8406	2	RD114 retrovirus	NA	Viruses; Riboviria; Pararnavirae; Artverviricota; Revtraviricetes; Ortervirales; Retroviridae; Orthoretrovirinae; Gammaretrovirus; unclassified Gammaretrovirus
GCA_000849965.1	8088	3	Gibbon ape leukemia virus	NA	Viruses; Riboviria; Pararnavirae; Artverviricota; Revtraviricetes; Ortervirales; Retroviridae; Orthoretrovirinae; Gammaretrovirus
GCA_000894635.1	7696	2	Bat sapovirus TLC58HK	NA	Viruses; ssRNA viruses; ssRNA positive-strand viruses, no DNA stage; Caliciviridae; Sapovirus; unclassified Sapovirus
GCA_000848405.1	8557	4	MasonPfizer monkey virus	NA	Viruses; Retro-transcribing viruses; Retroviridae; Orthoretrovirinae; Betaretrovirus
GCA_000851925.1	8785	4	Squirrel monkey retrovirusH	NA	Viruses; Riboviria; Pararnavirae; Artverviricota; Revtraviricetes; Ortervirales; Retroviridae; Orthoretrovirinae; Betaretrovirus; Squirrel monkey retrovirus
GCA_000853785.1	11985	4	Oropouche virus	BeAn19991	Viruses; ssRNA viruses; ssRNA negative-strand viruses; Bunyaviridae; Orthobunyavirus; Oropouche orthobunyavirus
GCA_000853485.1	3392	1	Avian myelocytomatosis virus 29	NA	Viruses; Riboviria; Pararnavirae; Artverviricota; Revtraviricetes; Ortervirales; Retroviridae; Orthoretrovirinae; Alpharetrovirus
GCA_000849005.1	2630	1	Avian carcinoma Mill Hill virus 2	MH2E21	Viruses; Riboviria; Pararnavirae; Artverviricota; Revtraviricetes; Ortervirales; Retroviridae; Orthoretrovirinae; Alpharetrovirus
GCA_000894675.1	46387	89	Acinetobacter phage AP22	NA	Viruses; Duplodnaviria; Heunggongvirae; Uroviricota; Caudoviricetes; Caudovirales; Myoviridae; Obolenskvirus; Acinetobacter virus AP22
GCA_002987745.1	2428	1	Avian sarcoma virus CT10	NA	Viruses; Riboviria; Pararnavirae; Artverviricota; Revtraviricetes; Ortervirales; Retroviridae; Orthoretrovirinae; Alpharetrovirus
GCA_000896275.1	33609	43	Clostridium phage PhiS63	NA	Viruses; dsDNA viruses, no RNA stage; Caudovirales; Siphoviridae; unclassified Siphoviridae
GCA_002366265.1	4035	3	Otarine picobirnavirus	PF080915	Viruses; Riboviria; Orthornavirae; Pisuviricota; Duplopiviricetes; Durnavirales; Picobirnaviridae; Picobirnavirus; unclassified Picobirnavirus

gca	genome.size	proteome.size	phage.name	strain	Taxonomy
GCA_000847725.1	4788	1	Fujinami sarcoma virus	NA	Viruses; Riboviria; Pararnavirae; Artverviricota; Revtraviricetes; Ortervirales; Retroviridae; Orthoretrovirinae; Alpharetrovirus
GCA_000855425.1	9392	4	Rous sarcoma virus	NA	Viruses; Riboviria; Pararnavirae; Artverviricota; Revtraviricetes; Ortervirales; Retroviridae; Orthoretrovirinae; Alpharetrovirus
GCA_000914895.1	13908	1	Phaseolus vulgaris endornavirus 1	NA	Viruses; dsRNA viruses; Endornaviridae; Endornavirus; Phaseolus vulgaris endornavirus
GCA_002820465.1	14817	1	Phaseolus vulgaris alphaendornavirus 2	NA	Viruses; Riboviria; Orthornavirae; Kitrinoviricota; Alsuviricetes; Martellivirales; Endornaviridae; Alphaendornavirus
GCA_000901275.1	43870	47	Xanthomonas virus CP1	NA	Viruses; Duplodnaviria; Heunggongvirae; Uroviricota; Caudoviricetes; Caudovirales; Siphoviridae; Klementvirus
GCA_000904115.1	42963	40	Xanthomonas citri phage CP2	NA	Viruses; dsDNA viruses, no RNA stage; Caudovirales; Podoviridae; unclassified Podoviridae
GCA_000901455.1	2598	2	Peristrophe mosaic virus	NA	Viruses; ssDNA viruses; Geminiviridae; unclassified Geminiviridae
GCA_000909475.1	1292	2	Cuban alphasatellite 1	NA	Viruses; Satellites; Satellite Nucleic Acids; Single stranded DNA satellites; Alphasatellites; Begomovirus-associated alphasatellites; unclassified Begomovirus-associated alphasatellites
GCA_000853665.1	8419	6	Bovine leukemia virus	NA	Viruses; Riboviria; Pararnavirae; Artverviricota; Revtraviricetes; Ortervirales; Retroviridae; Orthoretrovirinae; Deltaretrovirus
GCA_000902535.1	182833	257	Campylobacter virus CP21	NA	Viruses; dsDNA viruses, no RNA stage; Caudovirales; Myoviridae; Eucampyvirinae; Cp220virus
GCA_000863585.1	8507	6	Human Tlymphotropic virus 1	NA	Viruses; Retro-transcribing viruses; Retroviridae; Orthoretrovirinae; Deltaretrovirus; Primate T-lymphotropic virus 1
GCA_000847505.1	8952	5	Human Tlymphotropic virus 2	NA	Viruses; Retro-transcribing viruses; Retroviridae; Orthoretrovirinae; Deltaretrovirus; Primate T-lymphotropic virus 2
GCA_002602325.1	49381	72	Pseudomonas phage YMC0101P52_PAE_BP	NA	Viruses; Duplodnaviria; Heunggongvirae; Uroviricota; Caudoviricetes; Caudovirales; Siphoviridae; unclassified Siphoviridae
GCA_000852085.1	12602	1	Pestivirus giraffe1 H138	NA	Viruses; Riboviria; Orthornavirae; Kitrinoviricota; Flasuviricetes; Amarillovirales; Flaviviridae; Pestivirus; Pestivirus G
GCA_002659495.1	17439	11	Rotavirus A RVAHumanNCA18J2010G1P8	NA	Viruses; Riboviria; Orthornavirae; Duplornaviricota; Resentoviricetes; Reovirales; Reoviridae; Sedoreovirinae; Rotavirus; Rotavirus A; Rotavirus G1
GCA_002659515.1	17439	11	Rotavirus A RVAHumanNCA22J2010G1P8	NA	Viruses; Riboviria; Orthornavirae; Duplornaviricota; Resentoviricetes; Reovirales; Reoviridae; Sedoreovirinae; Rotavirus; Rotavirus A; Rotavirus G1
GCA_002659555.1	17439	11	Rotavirus A RVAHumanNCA24J2010G1P8	NA	Viruses; Riboviria; Orthornavirae; Duplornaviricota; Resentoviricetes; Reovirales; Reoviridae; Sedoreovirinae; Rotavirus; Rotavirus A; Rotavirus G1
GCA_002659575.1	17439	11	Rotavirus A RVAHumanNCA25J2010G1P8	NA	Viruses; Riboviria; Orthornavirae; Duplornaviricota; Resentoviricetes; Reovirales; Reoviridae; Sedoreovirinae; Rotavirus; Rotavirus A; Rotavirus G1
GCA_002659595.1	17439	11	Rotavirus A RVAHumanNCA26J2010G1P8	NA	Viruses; Riboviria; Orthornavirae; Duplornaviricota; Resentoviricetes; Reovirales; Reoviridae; Sedoreovirinae; Rotavirus; Rotavirus A; Rotavirus G1
GCA_002659615.1	17439	11	Rotavirus A RVAHumanNCA28J2010G1P8	NA	Viruses; Riboviria; Orthornavirae; Duplornaviricota; Resentoviricetes; Reovirales; Reoviridae; Sedoreovirinae; Rotavirus; Rotavirus A; Rotavirus G1
GCA_002659635.1	17439	11	Rotavirus A RVAHumanNCA41J2010G1P8	NA	Viruses; Riboviria; Orthornavirae; Duplornaviricota; Resentoviricetes; Reovirales; Reoviridae; Sedoreovirinae; Rotavirus; Rotavirus A; Rotavirus G1
GCA_002659655.1	17439	11	Rotavirus A RVAHumanNCA45J2010G1P8	NA	Viruses; Riboviria; Orthornavirae; Duplornaviricota; Resentoviricetes; Reovirales; Reoviridae; Sedoreovirinae; Rotavirus; Rotavirus A; Rotavirus G1
GCA_002659695.1	17439	11	Rotavirus A RVAHumanNCA72J2010G1P8	NA	Viruses; Riboviria; Orthornavirae; Duplornaviricota; Resentoviricetes; Reovirales; Reoviridae; Sedoreovirinae; Rotavirus; Rotavirus A; Rotavirus G1
GCA_002659715.1	17439	11	Rotavirus A RVAHumanNCA7J2010G1P8	NA	Viruses; Riboviria; Orthornavirae; Duplornaviricota; Resentoviricetes; Reovirales; Reoviridae; Sedoreovirinae; Rotavirus; Rotavirus A; Rotavirus G1
GCA_002659735.1	17439	11	Rotavirus A RVAHumanNCA9J2010G1P8	NA	Viruses; Riboviria; Orthornavirae; Duplornaviricota; Resentoviricetes; Reovirales; Reoviridae; Sedoreovirinae; Rotavirus; Rotavirus A; Rotavirus G1
GCA_002659475.1	17433	11	Rotavirus A RVAHumanNCA125L2010G3P8	NA	Viruses; Riboviria; Orthornavirae; Duplornaviricota; Resentoviricetes; Reovirales; Reoviridae; Sedoreovirinae; Rotavirus; Rotavirus A; Rotavirus G3
GCA_002659775.1	17421	11	Rotavirus A RVAHumanNCAOL2010G4P6	NA	Viruses; Riboviria; Orthornavirae; Duplornaviricota; Resentoviricetes; Reovirales; Reoviridae; Sedoreovirinae; Rotavirus; Rotavirus A; Rotavirus G4
GCA_002659675.1	17433	11	Rotavirus A RVAHumanNCA64J2010G3P8	NA	Viruses; Riboviria; Orthornavirae; Duplornaviricota; Resentoviricetes; Reovirales; Reoviridae; Sedoreovirinae; Rotavirus; Rotavirus A; Rotavirus G3
GCA_000897155.1	43376	34	Goose adenovirus 4	P29	Viruses; dsDNA viruses, no RNA stage; Adenoviridae; Aviadenovirus; Goose aviadenovirus A
GCA_008767675.1	7347	1	Human enterovirus	V130682	Viruses; ssRNA viruses; ssRNA positive-strand viruses, no DNA stage; Picornavirales; Picornaviridae; Enterovirus; unclassified Enterovirus
GCA_000925715.1	43871	35	Klebsiella phage NTUHK2044K11	NA	Viruses; dsDNA viruses, no RNA stage; Caudovirales; Podoviridae; Autographivirinae; Kp34virus; Klebsiella virus K244
GCA_003179315.1	7890	7	Canis familiaris papillomavirus 12	NA	Viruses; Monodnaviria; Shotokuvirae; Cossaviricota; Papovaviricetes; Zurhausenvirales; Papillomaviridae; Firstpapillomavirinae; Chipapillomavirus; Chipapillomavirus 1
GCA_008785965.1	7408	1	Ovine enterovirus	ovineTB4OEV2009HUN	Viruses; Riboviria; Orthornavirae; Pisuviricota; Pisoniviricetes; Picornavirales; Picornaviridae; Enterovirus; Enterovirus G
GCA_000891575.1	7489	3	Avian leukemia virus	NA	Viruses; Riboviria; Pararnavirae; Artverviricota; Revtraviricetes; Ortervirales; Retroviridae; Orthoretrovirinae; Alpharetrovirus; Avian leukosis virus

gca	genome.size	proteome.size	phage.name	strain	Taxonomy
GCA_000898915.1	41992	57	Bacillus phage BtCS33	NA	Viruses; dsDNA viruses, no RNA stage; Caudovirales; Siphoviridae; unclassified Siphoviridae
GCA_002602365.1	53972	100	Mycobacterium virus Nepal	NA	Viruses; Duplodnaviria; Heunggongvirae; Uroviricota; Caudoviricetes; Caudovirales; Siphoviridae; Fromanvirus
GCA_000906275.1	2063	2	Canine circovirus	NA	Viruses; ssDNA viruses; Circoviridae; Circovirus
GCA_000910015.1	45242	69	Staphylococcus phage StauST3981	NA	Viruses; dsDNA viruses, no RNA stage; Caudovirales; Siphoviridae; unclassified Siphoviridae
GCA_002602385.1	51505	92	Mycobacteriophage ElTiger69	NA	Viruses; Duplodnaviria; Heunggongvirae; Uroviricota; Caudoviricetes; Caudovirales; Siphoviridae; Fromanvirus; unclassified Fromanvirus
GCA_002602405.1	61567	99	Mycobacterium virus Macncheese	NA	Viruses; Duplodnaviria; Heunggongvirae; Uroviricota; Caudoviricetes; Caudovirales; Siphoviridae; Timquatrovirus
GCA_000908535.1	41392	69	Staphylococcus phage StauST3983	NA	Viruses; dsDNA viruses, no RNA stage; Caudovirales; Siphoviridae; unclassified Siphoviridae
GCA_000906215.1	158621	238	Bacillus phage vB_BceM_Bc431v3	NA	Viruses; Duplodnaviria; Heunggongvirae; Uroviricota; Caudoviricetes; Caudovirales; Herelleviridae; Bastillevirinae; Caeruleovirus; Bacillus virus Bc431
GCA_000903775.1	62952	75	Burkholderia virus Bcepmigl	NA	Viruses; dsDNA viruses, no RNA stage; Caudovirales; Podoviridae; Bcep22likevirus
GCA_002606865.1	52494	99	Mycobacterium virus Astro	NA	Viruses; Duplodnaviria; Heunggongvirae; Uroviricota; Caudoviricetes; Caudovirales; Siphoviridae; Fromanvirus
GCA_002597545.1	145647	232	Staphylococcus phage Staph1N	NA	Viruses; Duplodnaviria; Heunggongvirae; Uroviricota; Caudoviricetes; Caudovirales; Herelleviridae; Twortvirinae; Kayvirus; Staphylococcus virus G1
GCA_002597565.1	141018	212	Staphylococcus phage A3R	NA	Viruses; Duplodnaviria; Heunggongvirae; Uroviricota; Caudoviricetes; Caudovirales; Herelleviridae; Twortvirinae; Kayvirus; Staphylococcus virus G1
GCA_002597605.1	148481	233	Staphylococcus phage Fi200W	NA	Viruses; Duplodnaviria; Heunggongvirae; Uroviricota; Caudoviricetes; Caudovirales; Herelleviridae; Twortvirinae; Kayvirus; Staphylococcus virus G1
GCA_002597625.1	148243	232	Staphylococcus phage MSA6	NA	Viruses; Duplodnaviria; Heunggongvirae; Uroviricota; Caudoviricetes; Caudovirales; Herelleviridae; Twortvirinae; Kayvirus; Staphylococcus virus G1
GCA_002597645.1	147590	232	Staphylococcus phage P4W	NA	Viruses; Duplodnaviria; Heunggongvirae; Uroviricota; Caudoviricetes; Caudovirales; Herelleviridae; Twortvirinae; Kayvirus; Staphylococcus virus G1
GCA_002597585.1	148564	233	Staphylococcus phage 676Z	NA	Viruses; Duplodnaviria; Heunggongvirae; Uroviricota; Caudoviricetes; Caudovirales; Herelleviridae; Twortvirinae; Kayvirus; Staphylococcus virus G1
GCA_000910235.1	223989	231	Cronobacter phage CR5	NA	Viruses; dsDNA viruses, no RNA stage; Caudovirales; Myoviridae; unclassified Myoviridae
GCA_000908135.1	42652	62	Staphylococcus phage SA13	NA	Viruses; dsDNA viruses, no RNA stage; Caudovirales; Siphoviridae; unclassified Siphoviridae
GCA_000925355.1	17187	6	Sunshine Coast virus	NA	Viruses; ssRNA viruses; ssRNA negative-strand viruses; Mononegavirales; Sunviridae; Sunshinevirus; Reptile sunshinevirus 1
GCA_000898455.1	8206	4	Amazon lily mild mottle virus	Japan	Viruses; ssRNA viruses; ssRNA positive-strand viruses, no DNA stage; Bromoviridae; Anulavirus; unclassified Anulavirus
GCA_018577865.1	222870	189	Bat betaherpesvirus B7D8	NA	Viruses; Duplodnaviria; Heunggongvirae; Peploviricota; Herviviricetes; Herpesvirales; Herpesviridae; Betaherpesvirinae; unclassified Betaherpesvirinae
GCA_002826925.1	7531	7	Miniopterus schreibersii papillomavirus 1	TT20F	Viruses; Monodnaviria; Shotokuvirae; Cossaviricota; Papovaviricetes; Zurhausenvirales; Papillomaviridae; Firstpapillomavirinae; Dyotaupapillomavirus; Dyotaupapillomavirus 1
GCA_002113985.1	8468	1	Miniopterus schreibersii picornavirus 1	NA	Viruses; Riboviria; Orthornavirae; Pisuviricota; Pisoniviricetes; Picornavirales; Picornaviridae; Mischivirus; Mischivirus A
GCA_003033245.1	5038	4	Myotis myotis bocavirus 1	NA	Viruses; Monodnaviria; Shotokuvirae; Cossaviricota; Quintoviricetes; Piccovirales; Parvoviridae; Parvovirinae; Bocaparvovirus; Chiropteran bocaparvovirus 1
GCA_003033165.1	7339	5	Myotis ricketti papillomavirus 1	NA	Viruses; Monodnaviria; Shotokuvirae; Cossaviricota; Papovaviricetes; Zurhausenvirales; Papillomaviridae; Firstpapillomavirinae; Treisiotapapillomavirus; Treisiotapapillomavirus 1
GCA_002819485.1	1760	2	Rhinolophus ferrumequinum circovirus 1	NA	Viruses; Monodnaviria; Shotokuvirae; Cressdnaviricota; Arfiviricetes; Cirlivirales; Circoviridae; Circovirus; Bat associated circovirus 3
GCA_000894215.1	5034	7	Tomato dwarf leaf virus	NA	Viruses; ssDNA viruses; Geminiviridae; Begomovirus
GCA_000908695.1	7286	7	Human papillomavirus type 154	NA	Viruses; dsDNA viruses, no RNA stage; Papillomaviridae; unclassified Papillomaviridae; Human papillomavirus types
GCA_000898615.1	2806	4	Paspalum dilatatum striate mosaic virus	NA	Viruses; ssDNA viruses; Geminiviridae; Mastrevirus
GCA_000900075.1	2816	4	Digitaria ciliaris striate mosaic virus	NA	Viruses; ssDNA viruses; Geminiviridae; Mastrevirus
GCA_000895375.1	7948	1	Canine picornavirus	325F	Viruses; ssRNA viruses; ssRNA positive-strand viruses, no DNA stage; Picornavirales; unclassified Picornavirales
GCA_000870685.1	5779	4	Woolly monkey sarcoma virus	NA	Viruses; Riboviria; Pararnavirae; Artverviricota; Revtraviricetes; Ortervirales; Retroviridae; Orthoretrovirinae; Gammaretrovirus
GCA_000853345.1	7683	3	Feline calicivirus	Urbana	Viruses; ssRNA viruses; ssRNA positive-strand viruses, no DNA stage; Caliciviridae; Vesivirus
GCA_001041175.1	87603	147	Enterobacteriaphage UAB_Phi87	NA	Viruses; dsDNA viruses, no RNA stage; Caudovirales; Myoviridae; unclassified Myoviridae

gca	genome.size	proteome.size	phage.name	strain	Taxonomy
GCA_000898435.1	38889	56	Celeribacter phage P12053L	NA	Viruses; dsDNA viruses, no RNA stage; Caudovirales; unclassified Caudovirales
GCA_000899895.1	43581	66	Staphylococcus virus IPLA5	NA	Viruses; Duplodnaviria; Heunggongvirae; Uroviricota; Caudoviricetes; Caudovirales; Siphoviridae; Phietavirus
GCA_000897455.1	42123	59	Staphylococcus virus IPLA7	NA	Viruses; Duplodnaviria; Heunggongvirae; Uroviricota; Caudoviricetes; Caudovirales; Siphoviridae; Phietavirus
GCA_000901975.1	160646	230	Aeromonas phage Aes508	NA	Viruses; dsDNA viruses, no RNA stage; Caudovirales; Myoviridae; Secunda5virus; Aeromonas virus Aes508
GCA_000907075.1	161978	243	Aeromonas phage Aes012	NA	Viruses; dsDNA viruses, no RNA stage; Caudovirales; Myoviridae; Secunda5virus; Aeromonas virus Aes12
GCA_002823225.1	2753	7	Premna leaf curl virus	NA	Viruses; Monodnaviria; Shotokuvirae; Cressdnaviricota; Repensiviricetes; Geplafuvirales; Geminiviridae; Begomovirus
GCA_000907335.1	9868	1	Donkey orchid virus A	NA	Viruses; ssRNA viruses; ssRNA positive-strand viruses, no DNA stage; Potyviridae; Potyvirus
GCA_000897635.1	9847	1	Caladenia virus A	NA	Viruses; ssRNA viruses; ssRNA positive-strand viruses, no DNA stage; Potyviridae; Poacevirus; unclassified Poacevirus
GCA_002823565.1	2771	6	Sweet potato leaf curl China virus	NA	Viruses; Monodnaviria; Shotokuvirae; Cressdnaviricota; Repensiviricetes; Geplafuvirales; Geminiviridae; Begomovirus
GCA_000864005.1	7654	3	Norwalk virus	NA	Viruses; ssRNA viruses; ssRNA positive-strand viruses, no DNA stage; Caliciviridae; Norovirus
GCA_004786975.1	8970	2	Marine RNA virus SF1	NA	Viruses; Riboviria; Orthornavirae; Pisuviricota; Pisoniviricetes; Picornavirales; Marnaviridae; Locarnavirus; Sanfarnavirus 1
GCA_002867555.1	5193	7	Jacquemontia mosaic Yucatan virus	NA	Viruses; Monodnaviria; Shotokuvirae; Cressdnaviricota; Repensiviricetes; Geplafuvirales; Geminiviridae; Begomovirus
GCA_002830085.1	1262	1	Honeysuckle yellow vein mosaic betasatellite	NA	Viruses; Tolecusatellitidae; Betasatellite
GCA_000854705.1	4003	5	Carnation mottle virus	NA	Viruses; ssRNA viruses; ssRNA positive-strand viruses, no DNA stage; Tombusviridae; Alphacarmovirus
GCA_000865645.1	4266	6	Melon necrotic spot virus	NA	Viruses; ssRNA viruses; ssRNA positive-strand viruses, no DNA stage; Tombusviridae; Gammacarmovirus
GCA_000853045.1	4054	5	Turnip crinkle virus	NA	Viruses; ssRNA viruses; ssRNA positive-strand viruses, no DNA stage; Tombusviridae; Betacarmovirus
GCA_000860825.1	4212	4	Ryegrass mottle virus	NA	Viruses; ssRNA viruses; ssRNA positive-strand viruses, no DNA stage; Sobemovirus
GCA_000848645.1	14984	19	Pseudomonas phage phi8	NA	Viruses; dsRNA viruses; Cystoviridae; unclassified Cystoviridae
GCA_001737115.1	133798	177	Staphylococcus phage Stau2	NA	Viruses; dsDNA viruses, no RNA stage; Caudovirales; Myoviridae; Spounavirinae; Silviavirus; unclassified Silviavirus
GCA_000861565.1	7374	1	A2 plaque virus	NA	Viruses; Riboviria; Orthornavirae; Pisuviricota; Pisoniviricetes; Picornavirales; Picornaviridae; Enterovirus; Enterovirus H
GCA_000897335.1	1379	1	French bean leaf curl betasatelliteKanpur	NA	Viruses; Satellites; Satellite Nucleic Acids; Single stranded DNA satellites; Betasatellites
GCA_002821745.1	2741	7	Bhendi yellow vein mosaic virus	NA	Viruses; ssDNA viruses; Geminiviridae; Begomovirus
GCA_002786405.1	3575	4	Enterobacteria phage fr	NA	Viruses; Riboviria; Orthornavirae; Lenarviricota; Allassoviricetes; Levivirales; Leviviridae; Levivirus; Escherichia virus MS2
GCA_000847365.1	3466	4	Enterobacteria phage GA	NA	Viruses; Riboviria; Orthornavirae; Lenarviricota; Allassoviricetes; Levivirales; Leviviridae; Levivirus; Escherichia virus BZ13
GCA_002755235.1	3486	4	Enterobacteria phage KU1	NA	Viruses; Riboviria; Orthornavirae; Lenarviricota; Allassoviricetes; Levivirales; Leviviridae; Levivirus; Escherichia virus BZ13
GCA_000898955.1	3640	5	Citrus chlorotic dwarf associated virus	NA	Viruses; ssDNA viruses; Geminiviridae; unclassified Geminiviridae
GCA_000847485.1	3569	4	Escherichia phage MS2	NA	Viruses; Riboviria; Orthornavirae; Lenarviricota; Allassoviricetes; Levivirales; Leviviridae; Levivirus; Escherichia virus MS2
GCA_000855925.1	3588	4	Pseudomonas phage PP7	NA	Viruses; ssRNA viruses; ssRNA positive-strand viruses, no DNA stage; Leviviridae; Levivirus; unclassified Levivirus
GCA_000868365.1	3573	4	Pseudomonas phage PRR1	NA	Viruses; ssRNA viruses; ssRNA positive-strand viruses, no DNA stage; Leviviridae; unclassified Leviviridae
GCA_000862845.1	4276	4	Enterobacteria phage SP	NA	Viruses; Riboviria; Orthornavirae; Lenarviricota; Allassoviricetes; Levivirales; Leviviridae; Allolevivirus; Escherichia virus FI
GCA_000898335.1	4927	5	MW polyomavirus	MA095	Viruses; dsDNA viruses, no RNA stage; Polyomaviridae; unclassified Polyomaviridae
GCA_003177655.1	7998	7	human papillomavirus 87	NA	Viruses; Monodnaviria; Shotokuvirae; Cossaviricota; Papovaviricetes; Zurhausenvirales; Papillomaviridae; Firstpapillomavirinae; Alphapapillomavirus; Alphapapillomavirus 3
GCA_000850345.1	5964	5	Bean leafroll virus	NA	Viruses; ssRNA viruses; ssRNA positive-strand viruses, no DNA stage; Luteoviridae; Luteovirus
GCA_000902935.1	26449	32	Helicobacter phage KHP40	NA	Viruses; dsDNA viruses, no RNA stage; Caudovirales; Podoviridae; unclassified Podoviridae

gca	genome.size	proteome.size	phage.name	strain	Taxonomy
GCA_000852565.1	5666	6	Beet western yellows virus	USA	Viruses; ssRNA viruses; ssRNA positive-strand viruses, no DNA stage; Luteoviridae; Polerovirus
GCA_000856725.1	5987	6	Potato leafroll virus	1	Viruses; ssRNA viruses; ssRNA positive-strand viruses, no DNA stage; Luteoviridae; Polerovirus
GCA_002630725.1	246421	382	Vibriophage phipp2	NA	Viruses; Duplodnaviria; Heunggongvirae; Uroviricota; Caudoviricetes; Caudovirales; Myoviridae; Tevenvirinae; Schizotequatrovirus; Vibrio virus KVP40
GCA_000925915.1	58550	110	Mycobacterium phage Taj	NA	Viruses; dsDNA viruses, no RNA stage; Caudovirales; Siphoviridae; Che8virus
GCA_000903495.1	231743	427	Aeromonas phage CC2	NA	Viruses; dsDNA viruses, no RNA stage; Caudovirales; Myoviridae; Tevenvirinae; T4virus; unclassified T4virus
GCA_000899055.1	61167	77	Pseudomonas phage MP1412	NA	Viruses; dsDNA viruses, no RNA stage; Caudovirales; Siphoviridae; unclassified Siphoviridae
GCA_000925795.1	66854	94	Escherichia phage ECML117	NA	Viruses; dsDNA viruses, no RNA stage; Caudovirales; Myoviridae; unclassified Myoviridae
GCA_000925055.1	166783	270	Escherichia phage ECML134	NA	Viruses; dsDNA viruses, no RNA stage; Caudovirales; Myoviridae; Tevenvirinae; T4virus; Escherichia virus ECML134
GCA_000924955.1	157308	198	Escherichia phage ECML4	NA	Viruses; dsDNA viruses, no RNA stage; Caudovirales; Myoviridae; Vi1virus
GCA_000898755.1	50255	72	Streptomyces phage phiHau3	NA	Viruses; dsDNA viruses, no RNA stage; Caudovirales; Siphoviridae; unclassified Siphoviridae
GCA_000900215.1	37612	55	Streptomyces phage SV1	NA	Viruses; dsDNA viruses, no RNA stage; Caudovirales; Siphoviridae; unclassified Siphoviridae
GCA_000904875.1	159624	208	Salmonella phage SKML39	NA	Viruses; dsDNA viruses, no RNA stage; Caudovirales; Myoviridae; unclassified Myoviridae
GCA_001041715.1	147745	242	Salmonella phage SSE121	NA	Viruses; dsDNA viruses, no RNA stage; Caudovirales; Myoviridae; Vequintavirinae; Se1virus; Salmonella virus SSE121
GCA_001041775.1	158099	255	Salmonella phage STML198	NA	Viruses; dsDNA viruses, no RNA stage; Caudovirales; Myoviridae; Tevenvirinae; S16virus; Salmonella virus STML198
GCA_000905015.1	149844	210	Bacillus phage phiAGATE	NA	Viruses; dsDNA viruses, no RNA stage; Caudovirales; Myoviridae; Agatevirus; Bacillus virus Agate
GCA_000901555.1	48396	76	Clostridium phage phiMMP02	NA	Viruses; dsDNA viruses, no RNA stage; Caudovirales; Myoviridae; unclassified Myoviridae
GCA_000900495.1	31674	51	Clostridium phage phiMMP04	NA	Viruses; dsDNA viruses, no RNA stage; Caudovirales; Myoviridae; unclassified Myoviridae
GCA_000900535.1	215710	338	Caulobacter virus phiCbK	NA	Viruses; dsDNA viruses, no RNA stage; Caudovirales; Siphoviridae; Phicbkvirus
GCA_000906315.1	40192	47	Pseudomonas phage PhiS1	NA	Viruses; dsDNA viruses, no RNA stage; Caudovirales; Podoviridae; Autographivirinae; T7virus; unclassified T7-like viruses
GCA_000899355.1	122024	149	Pectobacterium phage My1	NA	Viruses; dsDNA viruses, no RNA stage; Caudovirales; Siphoviridae; unclassified Siphoviridae
GCA_000927555.1	40923	69	Enterococcus phage EfaCPT1	NA	Viruses; dsDNA viruses, no RNA stage; Caudovirales; Siphoviridae; unclassified Siphoviridae
GCA_002624885.1	42633	64	Salmonella phage wksl3	NA	Viruses; Duplodnaviria; Heunggongvirae; Uroviricota; Caudoviricetes; Caudovirales; Siphoviridae; Guernseyvirinae; Jerseyvirus; Salmonella virus wksl3
GCA_002617265.1	72321	88	Pseudomonas phage PA26	NA	Viruses; Duplodnaviria; Heunggongvirae; Uroviricota; Caudoviricetes; Caudovirales; Podoviridae; Litunavirus; Pseudomonas virus PA26
GCA_000901915.1	136326	186	Staphylococcus virus SA11	NA	Viruses; dsDNA viruses, no RNA stage; Caudovirales; Myoviridae; Spounavirinae; Silviavirus
GCA_000848565.1	5853	5	Soybean dwarf virus	YS	Viruses; ssRNA viruses; ssRNA positive-strand viruses, no DNA stage; Luteoviridae; Luteovirus
GCA_000927255.1	16698	8	Salem virus	NA	Viruses; ssRNA viruses; ssRNA negative-strand viruses; Mononegavirales; Paramyxoviridae; unclassified Paramyxoviridae
GCA_000857065.1	3684	5	Tobacco necrosis virus A	NA	Viruses; ssRNA viruses; ssRNA positive-strand viruses, no DNA stage; Tombusviridae; Alphanecrovirus
GCA_000848725.1	3762	6	Tobacco necrosis virus D	NA	Viruses; ssRNA viruses; ssRNA positive-strand viruses, no DNA stage; Tombusviridae; Betanecrovirus
GCA_000849705.1	40248	53	Streptococcus phage MM1	NA	Viruses; dsDNA viruses, no RNA stage; Caudovirales; Siphoviridae; unclassified Siphoviridae
GCA_003088535.1	11490	4	Phlebovirus JS2010014	NA	Viruses; Riboviria; Orthornavirae; Negarnaviricota; Polyploviricotina; Ellioviricetes; Bunyavirales; Phenuiviridae; Phlebovirus; SFTS phlebovirus
GCA_003088575.1	11490	4	Phlebovirus JS2010015	NA	Viruses; Riboviria; Orthornavirae; Negarnaviricota; Polyploviricotina; Ellioviricetes; Bunyavirales; Phenuiviridae; Phlebovirus; SFTS phlebovirus
GCA_003088595.1	11490	4	Phlebovirus JS2010018	NA	Viruses; Riboviria; Orthornavirae; Negarnaviricota; Polyploviricotina; Ellioviricetes; Bunyavirales; Phenuiviridae; Phlebovirus; SFTS phlebovirus
GCA_003088615.1	11490	4	Phlebovirus JS2010019	NA	Viruses; Riboviria; Orthornavirae; Negarnaviricota; Polyploviricotina; Ellioviricetes; Bunyavirales; Phenuiviridae; Phlebovirus; SFTS phlebovirus
GCA_000905715.1	36566	56	Lactobacillus phage phiAQ113	NA	Viruses; dsDNA viruses, no RNA stage; Caudovirales; Myoviridae; unclassified Myoviridae

gca	genome.size	proteome.size	phage.name	strain	Taxonomy
GCA_008799375.1	7418	1	Echovirus E6	DAmori	Viruses; Riboviria; Orthornavirae; Pisuviricota; Pisoniviricetes; Picornavirales; Picornaviridae; Enterovirus; Enterovirus B
GCA_002817305.1	7339	1	Human parechovirus 1	NA	Viruses; Riboviria; Orthornavirae; Pisuviricota; Pisoniviricetes; Picornavirales; Picornaviridae; Parechovirus; Parechovirus A
GCA_000903835.1	3562	4	Enterobacteria phage Hgal1	NA	Viruses; ssRNA viruses; ssRNA positive-strand viruses, no DNA stage; Leviviridae; Levivirus; unclassified Levivirus
GCA_000901295.1	3523	4	Enterobacteria phage C1 INW2012	NA	Viruses; ssRNA viruses; ssRNA positive-strand viruses, no DNA stage; Leviviridae; Levivirus; unclassified Levivirus
GCA_000863205.1	7414	1	Enterovirus E	VG527	Viruses; ssRNA viruses; ssRNA positive-strand viruses, no DNA stage; Picornavirales; Picornaviridae; Enterovirus
GCA_003047835.1	42085	50	Yersinia phage phiR801	NA	Viruses; Duplodnaviria; Heunggongvirae; Uroviricota; Caudoviricetes; Caudovirales; Autographiviridae; Melnykvirinae; Pienvirus; Yersinia virus R8-01
GCA_000903755.2	122696	168	Yersinia phage phiR201	NA	Viruses; dsDNA viruses, no RNA stage; Caudovirales; Siphoviridae; unclassified Siphoviridae
GCA_000904795.1	168809	262	Yersinia phage phiR1RT	NA	Viruses; dsDNA viruses, no RNA stage; Caudovirales; Myoviridae; Tg1virus; Yersinia virus R1RT
GCA_000901215.2	42406	57	Yersinia phage phi8018	NA	Viruses; dsDNA viruses, no RNA stage; Caudovirales; Podoviridae; Autographivirinae; unclassified Autographivirinae
GCA_001042055.1	167063	277	Yersinia phage phiD1	NA	Viruses; dsDNA viruses, no RNA stage; Caudovirales; Myoviridae; Tevenvirinae; T4virus; Yersinia virus D1
GCA_000897595.1	17798	10	Blueberry virus A	NA	Viruses; ssRNA viruses; ssRNA positive-strand viruses, no DNA stage; Closteroviridae; unclassified Closteroviridae
GCA_008798615.1	7452	1	Coxsackievirus A9	Griggs	Viruses; Riboviria; Orthornavirae; Pisuviricota; Pisoniviricetes; Picornavirales; Picornaviridae; Enterovirus; Enterovirus B
GCA_002826825.1	8017	1	human papillomavirus 71	NA	Viruses; Monodnaviria; Shotokuvirae; Cossaviricota; Papovaviricetes; Zurhausenvirales; Papillomaviridae; Firstpapillomavirinae; Alphapapillomavirus; Alphapapillomavirus 14
GCA_008799975.1	7406	1	Coxsackievirus A21	Kuykendall	Viruses; Riboviria; Orthornavirae; Pisuviricota; Pisoniviricetes; Picornavirales; Picornaviridae; Enterovirus; Enterovirus C
GCA_003108485.1	7401	1	Human coxsackievirus A21 Coe	NA	Viruses; Riboviria; Orthornavirae; Pisuviricota; Pisoniviricetes; Picornavirales; Picornaviridae; Enterovirus; Enterovirus C; Coxsackievirus A21
GCA_000861325.1	7389	1	Coxsackievirus B1	NA	Viruses; Riboviria; Orthornavirae; Pisuviricota; Pisoniviricetes; Picornavirales; Picornaviridae; Enterovirus; Enterovirus B
GCA_002816685.1	7399	1	Coxsackievirus B3	NA	Viruses; Riboviria; Orthornavirae; Pisuviricota; Pisoniviricetes; Picornavirales; Picornaviridae; Enterovirus; Enterovirus B
GCA_008766675.1	7395	1	Coxsackievirus B4	JVB Benschoten	Viruses; Riboviria; Orthornavirae; Pisuviricota; Pisoniviricetes; Picornavirales; Picornaviridae; Enterovirus; Enterovirus B
GCA_008800615.1	7400	1	Coxsackievirus B5	Faulkner	Viruses; Riboviria; Orthornavirae; Pisuviricota; Pisoniviricetes; Picornavirales; Picornaviridae; Enterovirus; Enterovirus B
GCA_008766555.1	7438	1	Echovirus E11	NA	Viruses; Riboviria; Orthornavirae; Pisuviricota; Pisoniviricetes; Picornavirales; Picornaviridae; Enterovirus; Enterovirus B
GCA_003108525.1	7441	1	Human poliovirus 1	NA	Viruses; Riboviria; Orthornavirae; Pisuviricota; Pisoniviricetes; Picornavirales; Picornaviridae; Enterovirus; Enterovirus C
GCA_003107385.1	9323	1	Hepatitis C virus subtype 4v	NA	Viruses; Riboviria; Orthornavirae; Kitrinoviricota; Flasuviricetes; Amarillovirales; Flaviviridae; Hepacivirus; Hepacivirus C; Hepatitis C virus genotype 4
GCA_000861165.1	7440	1	Human poliovirus 1 Mahoney	NA	Viruses; Riboviria; Orthornavirae; Pisuviricota; Pisoniviricetes; Picornavirales; Picornaviridae; Enterovirus; Enterovirus C; Human poliovirus 1
GCA_008766755.1	7441	1	Human poliovirus 1 strain Sabin	Sabin 1	Viruses; Riboviria; Orthornavirae; Pisuviricota; Pisoniviricetes; Picornavirales; Picornaviridae; Enterovirus; Enterovirus C; Human poliovirus 1
GCA_000902955.1	26215	30	Helicobacter phage KHP30	NA	Viruses; dsDNA viruses, no RNA stage; Caudovirales; Podoviridae; unclassified Podoviridae
GCA_003108605.1	7440	1	Human poliovirus 2	Lansing	Viruses; Riboviria; Orthornavirae; Pisuviricota; Pisoniviricetes; Picornavirales; Picornaviridae; Enterovirus; Enterovirus C
GCA_000910935.1	5403	3	Porcine partetravirus	FMV101437266	Viruses; ssDNA viruses; Parvoviridae; Parvovirinae; unclassified Parvovirinae
GCA_000910555.1	58305	104	Streptococcus phage SPQS1	NA	Viruses; dsDNA viruses, no RNA stage; Caudovirales; Siphoviridae; Sap6virus
GCA_003112265.1	7436	1	Human poliovirus 3	USOLDbac	Viruses; Riboviria; Orthornavirae; Pisuviricota; Pisoniviricetes; Picornavirales; Picornaviridae; Enterovirus; Enterovirus C
GCA_008797795.1	7459	1	Coxsackievirus A24	Joseph	Viruses; Riboviria; Orthornavirae; Pisuviricota; Pisoniviricetes; Picornavirales; Picornaviridae; Enterovirus; Enterovirus C
GCA_000861205.1	7390	1	Enterovirus D70	J67071	Viruses; Riboviria; Orthornavirae; Pisuviricota; Pisoniviricetes; Picornavirales; Picornaviridae; Enterovirus; Enterovirus D
GCA_000860505.1	7478	2	Hepatovirus A	NA	Viruses; ssRNA viruses; ssRNA positive-strand viruses, no DNA stage; Picornavirales; Picornaviridae; Hepatovirus
GCA_003178875.1	7443	7	Human papillomavirus 159	NA	Viruses; Monodnaviria; Shotokuvirae; Cossaviricota; Papovaviricetes; Zurhausenvirales; Papillomaviridae; Firstpapillomavirinae; Betapapillomavirus; Betapapillomavirus 2
GCA_000862985.1	7835	1	Encephalomyocarditis virus	Ruckert	Viruses; ssRNA viruses; ssRNA positive-strand viruses, no DNA stage; Picornavirales; Picornaviridae; Cardiovirus; Cardiovirus A

gca	genome.size	proteome.size	phage.name	strain	Taxonomy
GCA_008799075.1	8204	1	Footandmouth disease virus type A	A12119	Viruses; Riboviria; Orthornavirae; Pisuviricota; Pisoniviricetes; Picornavirales; Picornaviridae; Aphthovirus; Foot-and-mouth disease virus
GCA_002601425.1	51160	81	Streptomyces phage phiELB20	NA	Viruses; Duplodnaviria; Heunggongvirae; Uroviricota; Caudoviricetes; Caudovirales; Siphoviridae; Arquatrovirinae; Arequatrovirus; Streptomyces virus ELB20
GCA_002624865.1	41546	66	Salmonella phage vB_SenS_AG11	NA	Viruses; Duplodnaviria; Heunggongvirae; Uroviricota; Caudoviricetes; Caudovirales; Siphoviridae; Guernseyvirinae; Jerseyvirus; Salmonella virus AG11
GCA_001503215.1	162327	178	Stenotrophomonas phage IME13	NA	Viruses; dsDNA viruses, no RNA stage; Caudovirales; Myoviridae; Secunda5virus; Stenotrophomonas virus IME13
GCA_002602465.1	51500	86	Mycobacterium virus Arturo	NA	Viruses; Duplodnaviria; Heunggongvirae; Uroviricota; Caudoviricetes; Caudovirales; Siphoviridae; Fromanvirus
GCA_002602485.1	50645	86	Mycobacterium virus Goose	NA	Viruses; Duplodnaviria; Heunggongvirae; Uroviricota; Caudoviricetes; Caudovirales; Siphoviridae; Fromanvirus
GCA_002602505.1	49186	83	Mycobacterium virus Marcell	NA	Viruses; Duplodnaviria; Heunggongvirae; Uroviricota; Caudoviricetes; Caudovirales; Siphoviridae; Fromanvirus
GCA_002597925.1	51377	88	Mycobacterium phage Sabertooth	NA	Viruses; Duplodnaviria; Heunggongvirae; Uroviricota; Caudoviricetes; Caudovirales; Siphoviridae; Fromanvirus; Mycobacterium virus Peaches
GCA_002602525.1	43305	63	Staphylococcus phage SP5	NA	Viruses; Duplodnaviria; Heunggongvirae; Uroviricota; Caudoviricetes; Caudovirales; Siphoviridae; Phietavirus; unclassified Phietavirus
GCA_002602545.1	42902	61	Staphylococcus phage SP6	NA	Viruses; Duplodnaviria; Heunggongvirae; Uroviricota; Caudoviricetes; Caudovirales; Siphoviridae; Phietavirus; unclassified Phietavirus
GCA_002602565.1	43827	52	Pectobacterium phage PhiM1	NA	Viruses; Duplodnaviria; Heunggongvirae; Uroviricota; Caudoviricetes; Caudovirales; Autographiviridae; Corkvirinae; Phimunavirus; Pectobacterium virus fM1
GCA_000898715.1	9266	5	Apple green crinkle associated virus	NA	Viruses; ssRNA viruses; ssRNA positive-strand viruses, no DNA stage; Tymovirales; Betaflexiviridae; Quinvirinae; Foveavirus; unclassified Foveavirus
GCA_009734245.1	97065	88	uncultured crAssphage	NA	Viruses; Duplodnaviria; Heunggongvirae; Uroviricota; Caudoviricetes; Caudovirales; Podoviridae; unclassified Podoviridae; crAss-like viruses; environmental samples
GCA_000899915.1	13467	6	Persimmon virus A	persimmon isolate	Viruses; ssRNA viruses; ssRNA negative-strand viruses; Mononegavirales; Rhabdoviridae; Cytorhabdovirus; unclassified Cytorhabdovirus
GCA_000919775.1	7475	2	Persimmon latent virus	persimmon isolate	Viruses; dsRNA viruses; unclassified dsRNA viruses
GCA_000899635.1	279967	448	Caulobacter phage CcrColossus	NA	Viruses; dsDNA viruses, no RNA stage; Caudovirales; Siphoviridae; unclassified Siphoviridae
GCA_000901575.1	221828	353	Caulobacter virus Karma	NA	Viruses; dsDNA viruses, no RNA stage; Caudovirales; Siphoviridae; Phicbkvirus
GCA_000903095.1	218929	347	Caulobacter virus Magneto	NA	Viruses; dsDNA viruses, no RNA stage; Caudovirales; Siphoviridae; Phicbkvirus
GCA_000900555.1	223720	350	Caulobacter virus Rogue	NA	Viruses; dsDNA viruses, no RNA stage; Caudovirales; Siphoviridae; Phicbkvirus
GCA_000899655.1	219216	343	Caulobacter virus Swift	NA	Viruses; dsDNA viruses, no RNA stage; Caudovirales; Siphoviridae; Phicbkvirus
GCA_000863325.1	8134	1	Footandmouth disease virus type O	NA	Viruses; ssRNA viruses; ssRNA positive-strand viruses, no DNA stage; Picornavirales; Picornaviridae; Aphthovirus; Foot-and-mouth disease virus
GCA_008799055.1	8142	1	Footandmouth disease virus C1	C1 Oberbayern	Viruses; Riboviria; Orthornavirae; Pisuviricota; Pisoniviricetes; Picornavirales; Picornaviridae; Aphthovirus; Foot-and-mouth disease virus; Foot-and-mouth disease virus - type C
GCA_008799035.1	8179	1	Footandmouth disease virus type SAT 1	SAT13swa149	Viruses; Riboviria; Orthornavirae; Pisuviricota; Pisoniviricetes; Picornavirales; Picornaviridae; Aphthovirus; Foot-and-mouth disease virus
GCA_008798995.1	8170	1	Footandmouth disease virus type SAT 3	SAT32sa5759	Viruses; Riboviria; Orthornavirae; Pisuviricota; Pisoniviricetes; Picornavirales; Picornaviridae; Aphthovirus; Foot-and-mouth disease virus
GCA_008777655.1	8120	2	Theilers encephalomyelitis virus	DA	Viruses; Riboviria; Orthornavirae; Pisuviricota; Pisoniviricetes; Picornavirales; Picornaviridae; Cardiovirus; Cardiovirus B; Theilovirus
GCA_000861185.1	8101	1	Theilers murine encephalomyeltits virus GDVII	NA	Viruses; Riboviria; Orthornavirae; Pisuviricota; Pisoniviricetes; Picornavirales; Picornaviridae; Cardiovirus; Cardiovirus B
GCA_008766695.1	7102	1	Human rhinovirus A2	NA	Viruses; Riboviria; Orthornavirae; Pisuviricota; Pisoniviricetes; Picornavirales; Picornaviridae; Enterovirus; Rhinovirus A
GCA_000861265.1	7212	1	Rhinovirus B14	NA	Viruses; Riboviria; Orthornavirae; Pisuviricota; Pisoniviricetes; Picornavirales; Picornaviridae; Enterovirus; Rhinovirus B
GCA_000897935.1	1345	1	Tobacco leaf chlorosis betasatellite	NA	Viruses; Tolecusatellitidae; Betasatellite; unclassified Betasatellite
GCA_001885485.1	11931	5	Lleida bat lyssavirus	NA	Viruses; ssRNA viruses; ssRNA negative-strand viruses; Mononegavirales; Rhabdoviridae; Lyssavirus; unclassified Lyssavirus
GCA_000862245.1	7152	1	Human rhinovirus A89	NA	Viruses; Riboviria; Orthornavirae; Pisuviricota; Pisoniviricetes; Picornavirales; Picornaviridae; Enterovirus; Rhinovirus A
GCA_000900435.1	2781	6	Jatropha mosaic Nigerian virus	NA	Viruses; ssDNA viruses; Geminiviridae; Begomovirus; unclassified Begomovirus
GCA_002826485.1	6870	4	Ferret hepatitis E virus	SF4370	Viruses; Riboviria; Orthornavirae; Kitrinoviricota; Alsuviricetes; Hepelivirales; Hepeviridae; Orthohepevirus; Orthohepevirus C
GCA_008798215.1	7025	1	Human rhinovirus A23	NA	Viruses; Riboviria; Orthornavirae; Pisuviricota; Pisoniviricetes; Picornavirales; Picornaviridae; Enterovirus; Rhinovirus A

gca	genome.size	proteome.size	phage.name	strain	Taxonomy
GCA_000906295.1	9314	8	Rose yellow vein virus	RYVVMN1	Viruses; Riboviria; Pararnavirae; Artverviricota; Revtraviricetes; Ortervirales; Caulimoviridae; Rosadnavirus
GCA_000853145.1	9185	2	Cricket paralysis virus	NA	Viruses; ssRNA viruses; ssRNA positive-strand viruses, no DNA stage; Picornavirales; Dicistroviridae; Cripavirus
GCA_001019855.1	11511	4	Bhanja virus	ibAr2709	Viruses; ssRNA viruses; ssRNA negative-strand viruses; Bunyaviridae; Phlebovirus; unclassified Phlebovirus; Bhanja serogroup
GCA_000856925.1	4437	7	Maize chlorotic mottle virus	NA	Viruses; ssRNA viruses; ssRNA positive-strand viruses, no DNA stage; Tombusviridae; Machlomovirus
GCA_000860745.1	4132	4	Southern bean mosaic virus	NA	Viruses; ssRNA viruses; ssRNA positive-strand viruses, no DNA stage; Sobemovirus
GCA_006465505.1	105375	112	Chinese giant salamander iridovirus	NA	Viruses; Varidnaviria; Bamfordvirae; Nucleocytoviricota; Megaviricetes; Pimascovirales; Iridoviridae; Alphairidovirinae; Ranavirus; Frog virus 3
GCA_000864965.1	4789	5	Artichoke mottled crinkle virus	NA	Viruses; ssRNA viruses; ssRNA positive-strand viruses, no DNA stage; Tombusviridae; Tombusvirus
GCA_000848185.1	4701	5	Cucumber necrosis virus	NA	Viruses; ssRNA viruses; ssRNA positive-strand viruses, no DNA stage; Tombusviridae; Tombusvirus
GCA_000854345.1	4733	5	Cymbidium ringspot virus	NA	Viruses; ssRNA viruses; ssRNA positive-strand viruses, no DNA stage; Tombusviridae; Tombusvirus
GCA_000955035.1	7531	6	Citrus yellow vein clearing virus	NA	Viruses; ssRNA viruses; ssRNA positive-strand viruses, no DNA stage; Tymovirales; Alphaflexiviridae; Mandarivirus
GCA_000858805.1	4776	5	Tomato bushy stunt virus	cherry	Viruses; ssRNA viruses; ssRNA positive-strand viruses, no DNA stage; Tombusviridae; Tombusvirus
GCA_000899075.1	2034	2	Gyrovirus 4	D137	Viruses; ssDNA viruses; Circoviridae; Gyrovirus; unclassified Gyrovirus
GCA_008785305.1	7606	1	Encephalomyocarditis virus type 2	NA	Viruses; Riboviria; Orthornavirae; Pisuviricota; Pisoniviricetes; Picornavirales; Picornaviridae; Cardiovirus; unclassified Cardiovirus
GCA_000847385.1	6331	3	Eggplant mosaic virus	NA	Viruses; ssRNA viruses; ssRNA positive-strand viruses, no DNA stage; Tymovirales; Tymoviridae; Tymovirus
GCA_000847445.1	6035	3	Erysimum latent virus	NA	Viruses; ssRNA viruses; ssRNA positive-strand viruses, no DNA stage; Tymovirales; Tymoviridae; Tymovirus
GCA_000850025.1	6211	3	Ononis yellow mosaic virus	NA	Viruses; ssRNA viruses; ssRNA positive-strand viruses, no DNA stage; Tymovirales; Tymoviridae; Tymovirus
GCA_000862065.1	6318	3	Turnip yellow mosaic virus	NA	Viruses; ssRNA viruses; ssRNA positive-strand viruses, no DNA stage; Tymovirales; Tymoviridae; Tymovirus
GCA_000849065.1	6362	3	Kennedya yellow mosaic virus	NA	Viruses; ssRNA viruses; ssRNA positive-strand viruses, no DNA stage; Tymovirales; Tymoviridae; Tymovirus
GCA_000860605.1	15480	8	Beet yellows virus	NA	Viruses; ssRNA viruses; ssRNA positive-strand viruses, no DNA stage; Closteroviridae; Closterovirus
GCA_000862265.1	19296	12	Citrus tristeza virus	T36 quick decline	Viruses; ssRNA viruses; ssRNA positive-strand viruses, no DNA stage; Closteroviridae; Closterovirus
GCA_000870605.1	8855	6	Chrysanthemum virus B	NA	Viruses; ssRNA viruses; ssRNA positive-strand viruses, no DNA stage; Tymovirales; Betaflexiviridae; Quinvirinae; Carlavirus
GCA_000854985.1	8741	6	Poplar mosaic virus	NA	Viruses; ssRNA viruses; ssRNA positive-strand viruses, no DNA stage; Tymovirales; Betaflexiviridae; Quinvirinae; Carlavirus
GCA_000860585.1	8533	6	Potato virus M	Russian wild	Viruses; ssRNA viruses; ssRNA positive-strand viruses, no DNA stage; Tymovirales; Betaflexiviridae; Quinvirinae; Carlavirus
GCA_000866605.1	8478	7	Potato virus S	NA	Viruses; ssRNA viruses; ssRNA positive-strand viruses, no DNA stage; Tymovirales; Betaflexiviridae; Quinvirinae; Carlavirus
GCA_000901095.1	50552	78	Escherichia phage ADB2	NA	Viruses; dsDNA viruses, no RNA stage; Caudovirales; Siphoviridae; Tunavirinae; T1virus; Escherichia virus ADB2
GCA_000922255.1	11567	4	Heartland virus	NA	Viruses; ssRNA viruses; ssRNA negative-strand viruses; Bunyaviridae; Phlebovirus; unclassified Phlebovirus
GCA_000854125.1	8394	6	Lily symptomless virus	NA	Viruses; ssRNA viruses; ssRNA positive-strand viruses, no DNA stage; Tymovirales; Betaflexiviridae; Quinvirinae; Carlavirus
GCA_000848285.1	7555	3	Apple chlorotic leaf spot virus	P863	Viruses; ssRNA viruses; ssRNA positive-strand viruses, no DNA stage; Tymovirales; Betaflexiviridae; Trivirinae; Trichovirus
GCA_000865585.1	6227	5	Cymbidium mosaic virus	NA	Viruses; ssRNA viruses; ssRNA positive-strand viruses, no DNA stage; Tymovirales; Alphaflexiviridae; Potexvirus
GCA_000900955.1	44400	48	Pectobacterium phage PP1	NA	Viruses; dsDNA viruses, no RNA stage; Caudovirales; Podoviridae; Autographivirinae; Sp6virus; unclassified Sp6likevirus
GCA_000899935.1	5012	3	Sibine fusca densovirus	NA	Viruses; ssDNA viruses; Parvoviridae; Densovirinae; Iteradensovirus; Lepidopteran iteradensovirus 2
GCA_000849045.1	6151	5	Foxtail mosaic virus	NA	Viruses; ssRNA viruses; ssRNA positive-strand viruses, no DNA stage; Tymovirales; Alphaflexiviridae; Potexvirus
GCA_003147825.1	18252	9	Nipah virus	NA	Viruses; ssRNA viruses; ssRNA negative-strand viruses; Mononegavirales; Paramyxoviridae; Henipavirus
GCA_000849125.1	6955	6	Narcissus mosaic virus	NA	Viruses; ssRNA viruses; ssRNA positive-strand viruses, no DNA stage; Tymovirales; Alphaflexiviridae; Potexvirus

gca	genome.size	proteome.size	phage.name	strain	Taxonomy
GCA_000847685.1	6656	5	Papaya mosaic virus	NA	Viruses; ssRNA viruses; ssRNA positive-strand viruses, no DNA stage; Tymovirales; Alphaflexiviridae; Potexvirus
GCA_000852045.1	7059	6	Potato aucuba mosaic virus	NA	Viruses; ssRNA viruses; ssRNA positive-strand viruses, no DNA stage; Tymovirales; Alphaflexiviridae; Potexvirus
GCA_000866465.1	6435	5	Potato virus X	X3	Viruses; ssRNA viruses; ssRNA positive-strand viruses, no DNA stage; Tymovirales; Alphaflexiviridae; Potexvirus
GCA_000882055.1	2140	3	Torque teno zalophus virus 1	NA	Viruses; ssDNA viruses; Anelloviridae; Lambdatorquevirus
GCA_000855525.1	5966	6	Strawberry mild yellow edge virus	NA	Viruses; ssRNA viruses; ssRNA positive-strand viruses, no DNA stage; Tymovirales; Alphaflexiviridae; Potexvirus
GCA_000899975.1	2771	2	French bean severe leaf curl virus	NA	Viruses; ssDNA viruses; Geminiviridae; unclassified Geminiviridae
GCA_000855045.1	5845	5	White clover mosaic virus	NA	Viruses; ssRNA viruses; ssRNA positive-strand viruses, no DNA stage; Tymovirales; Alphaflexiviridae; Potexvirus
GCA_008797535.1	7134	1	Human rhinovirus C3	HRVQPM	Viruses; Riboviria; Orthornavirae; Pisuviricota; Pisoniviricetes; Picornavirales; Picornaviridae; Enterovirus; Rhinovirus C
GCA_000865605.1	5823	5	Lily virus X	NA	Viruses; ssRNA viruses; ssRNA positive-strand viruses, no DNA stage; Tymovirales; Alphaflexiviridae; Potexvirus
GCA_000908395.1	11530	4	Lone Star virus	NA	Viruses; ssRNA viruses; ssRNA negative-strand viruses; Bunyaviridae; unclassified Bunyaviridae
GCA_000857245.1	9992	1	Bean common mosaic virus	blackeye cowpea mosaic	Viruses; ssRNA viruses; ssRNA positive-strand viruses, no DNA stage; Potyviridae; Potyvirus
GCA_000863145.1	9532	1	Bean yellow mosaic virus	MB4	Viruses; ssRNA viruses; ssRNA positive-strand viruses, no DNA stage; Potyviridae; Potyvirus
GCA_000861485.1	9584	1	Clover yellow vein virus	No30	Viruses; ssRNA viruses; ssRNA positive-strand viruses, no DNA stage; Potyviridae; Potyvirus
GCA_000920375.1	5016	5	Raccoon polyomavirus	R45	Viruses; dsDNA viruses, no RNA stage; Polyomaviridae; unclassified Polyomaviridae
GCA_000861665.1	9465	1	Cowpea aphidborne mosaic virus	NA	Viruses; ssRNA viruses; ssRNA positive-strand viruses, no DNA stage; Potyviridae; Potyvirus
GCA_000897915.1	9512	1	Pokeweed mosaic virus	NA	Viruses; ssRNA viruses; ssRNA positive-strand viruses, no DNA stage; Potyviridae; Potyvirus
GCA_002828505.1	9455	1	Gloriosa stripe mosaic virus	NA	Viruses; Riboviria; Orthornavirae; Pisuviricota; Stelpaviricetes; Patatavirales; Potyviridae; Potyvirus
GCA_000862305.1	10080	1	Lettuce mosaic virus	E	Viruses; ssRNA viruses; ssRNA positive-strand viruses, no DNA stage; Potyviridae; Potyvirus
GCA_000863225.1	9515	1	Maize dwarf mosaic virus	Bulgaria	Viruses; ssRNA viruses; ssRNA positive-strand viruses, no DNA stage; Potyviridae; Potyvirus
GCA_000901615.1	9445	1	Ornithogalum mosaic virus	NA	Viruses; ssRNA viruses; ssRNA positive-strand viruses, no DNA stage; Potyviridae; Potyvirus
GCA_000862045.1	10326	1	Papaya ringspot virus	NA	Viruses; ssRNA viruses; ssRNA positive-strand viruses, no DNA stage; Potyviridae; Potyvirus
GCA_002602585.1	43444	56	Rhizobium phage RHEph01	NA	Viruses; Duplodnaviria; Heunggongvirae; Uroviricota; Caudoviricetes; Caudovirales; Autographiviridae; Paadamvirus; Rhizobium virus RHEph01
GCA_002755275.1	46486	60	Rhizobium phage RHEph02	NA	Viruses; Duplodnaviria; Heunggongvirae; Uroviricota; Caudoviricetes; Caudovirales; Autographiviridae; Cuernavacavirus; Rhizobium virus RHEph02
GCA_002755295.1	45912	59	Rhizobium phage RHEph03	NA	Viruses; Duplodnaviria; Heunggongvirae; Uroviricota; Caudoviricetes; Caudovirales; Autographiviridae; Cuernavacavirus; unclassified Cuernavacavirus
GCA_002617285.1	53018	81	Rhizobium phage RHEph04	NA	Viruses; Duplodnaviria; Heunggongvirae; Uroviricota; Caudoviricetes; Caudovirales; Myoviridae; Kleczkowskavirus; Rhizobium virus RHEph4
GCA_002755315.1	50426	75	Rhizobium phage RHEph05	NA	Viruses; Duplodnaviria; Heunggongvirae; Uroviricota; Caudoviricetes; Caudovirales; Myoviridae; Kleczkowskavirus; unclassified Kleczkowskavirus
GCA_002828705.1	10334	1	Papaya ringspot virus W	NA	Viruses; Riboviria; Orthornavirae; Pisuviricota; Stelpaviricetes; Patatavirales; Potyviridae; Potyvirus; Papaya ringspot virus
GCA_001040775.1	53721	82	Rhizobium phage RHEph06	NA	Viruses; Duplodnaviria; Heunggongvirae; Uroviricota; Caudoviricetes; Caudovirales; Myoviridae; Kleczkowskavirus; unclassified Kleczkowskavirus
GCA_002755335.1	43619	59	Rhizobium phage RHEph08	NA	Viruses; Duplodnaviria; Heunggongvirae; Uroviricota; Caudoviricetes; Caudovirales; Autographiviridae; Cuernavacavirus; Rhizobium virus RHEph08
GCA_002755355.1	45962	61	Rhizobium phage RHEph09	NA	Viruses; Duplodnaviria; Heunggongvirae; Uroviricota; Caudoviricetes; Caudovirales; Autographiviridae; Cuernavacavirus; Rhizobium virus RHEph09
GCA_002080335.1	115042	171	Rhizobium phage RHEph10	NA	Viruses; unclassified bacterial viruses
GCA_000860445.1	9924	1	Pea seedborne mosaic virus	DPD1	Viruses; ssRNA viruses; ssRNA positive-strand viruses, no DNA stage; Potyviridae; Potyvirus
GCA_000860525.1	9640	1	Pepper mottle virus	California	Viruses; ssRNA viruses; ssRNA positive-strand viruses, no DNA stage; Potyviridae; Potyvirus
GCA_000868525.1	9890	1	Pepper severe mosaic virus	NA	Viruses; ssRNA viruses; ssRNA positive-strand viruses, no DNA stage; Potyviridae; Potyvirus

gca	genome.size	proteome.size	phage.name	strain	Taxonomy
GCA_000862085.1	9741	1	Plum pox virus	NA	Viruses; ssRNA viruses; ssRNA positive-strand viruses, no DNA stage; Potyviridae; Potyvirus
GCA_000897535.1	5208	6	Soybean chlorotic spot virus	NA	Viruses; ssDNA viruses; Geminiviridae; Begomovirus
GCA_000899955.1	5053	3	Papilio polyxenes densovirus	NA	Viruses; ssDNA viruses; Parvoviridae; Densovirinae; Iteradensovirus; Lepidopteran iteradensovirus 4
GCA_000924595.1	18162	7	Cedar virus	NA	Viruses; ssRNA viruses; ssRNA negative-strand viruses; Mononegavirales; Paramyxoviridae; Henipavirus; unclassified Henipavirus
GCA_000899135.1	7551	5	Grapevine virus F	NA	Viruses; ssRNA viruses; ssRNA positive-strand viruses, no DNA stage; Tymovirales; Betaflexiviridae; Trivirinae; Vitivirus
GCA_000899175.1	7757	6	Soybean Putnam virus	NA	Viruses; Riboviria; Pararnavirae; Artverviricota; Revtraviricetes; Ortervirales; Caulimoviridae; Caulimovirus
GCA_000861585.1	9585	1	Potato virus A	B11	Viruses; ssRNA viruses; ssRNA positive-strand viruses, no DNA stage; Potyviridae; Potyvirus
GCA_000862905.1	9704	1	Potato virus Y	N	Viruses; ssRNA viruses; ssRNA positive-strand viruses, no DNA stage; Potyviridae; Potyvirus
GCA_004129735.1	4696	3	Bat polyomavirus	NA	Viruses; Monodnaviria; Shotokuvirae; Cossaviricota; Papovaviricetes; Sepolyvirales; Polyomaviridae; unclassified Polyomaviridae
GCA_002987635.1	9703	1	Potato virus Y strain C	NA	Viruses; Riboviria; Orthornavirae; Pisuviricota; Stelpaviricetes; Patatavirales; Potyviridae; Potyvirus; Potato virus Y
GCA_000902615.1	45364	62	Acinetobacter phage YMC0902B1251	NA	Viruses; Duplodnaviria; Heunggongvirae; Uroviricota; Caudoviricetes; Caudovirales; Siphoviridae; Vieuvirus; Acinetobacter virus B1251
GCA_000853985.1	15378	8	Reptilian ferlavirus	ATCC VR895	Viruses; Riboviria; Orthornavirae; Negarnaviricota; Haploviricotina; Monjiviricetes; Mononegavirales; Paramyxoviridae; Orthoparamyxovirinae; Ferlavirus
GCA_002987665.1	9735	1	Soybean mosaic virus	P	Viruses; ssRNA viruses; ssRNA positive-strand viruses, no DNA stage; Potyviridae; Potyvirus
GCA_000862465.1	9588	1	Soybean mosaic virus N	NA	Viruses; Riboviria; Orthornavirae; Pisuviricota; Stelpaviricetes; Patatavirales; Potyviridae; Potyvirus; Soybean mosaic virus
GCA_000926715.1	163424	277	Enterobacteria phage GEC3S	NA	Viruses; dsDNA viruses, no RNA stage; Caudovirales; Myoviridae; Tevenvirinae; T4virus; unclassified T4virus
GCA_000854025.1	9596	1	Sugarcane mosaic virus	NA	Viruses; ssRNA viruses; ssRNA positive-strand viruses, no DNA stage; Potyviridae; Potyvirus
GCA_000861345.1	9494	1	Tobacco etch virus	NA	Viruses; ssRNA viruses; ssRNA positive-strand viruses, no DNA stage; Potyviridae; Potyvirus
GCA_000860705.1	9475	1	Tobacco vein mottling virus	NA	Viruses; ssRNA viruses; ssRNA positive-strand viruses, no DNA stage; Potyviridae; Potyvirus
GCA_002828885.1	9700	1	Potato virus Y strain NTN	NA	Viruses; Riboviria; Orthornavirae; Pisuviricota; Stelpaviricetes; Patatavirales; Potyviridae; Potyvirus; Potato virus Y
GCA_000863465.1	9835	1	Turnip mosaic virus	UK1	Viruses; ssRNA viruses; ssRNA positive-strand viruses, no DNA stage; Potyviridae; Potyvirus
GCA_000861645.1	9591	1	Zucchini yellow mosaic virus	NA	Viruses; ssRNA viruses; ssRNA positive-strand viruses, no DNA stage; Potyviridae; Potyvirus
GCA_000906575.1	37095	58	Pseudomonas phage JBD24	NA	Viruses; dsDNA viruses, no RNA stage; Caudovirales; Siphoviridae; unclassified Siphoviridae
GCA_000904095.1	36947	56	Pseudomonas phage JBD30	NA	Viruses; dsDNA viruses, no RNA stage; Caudovirales; Siphoviridae; unclassified Siphoviridae
GCA_000905735.1	37740	59	Pseudomonas phage JBD5	NA	Viruses; dsDNA viruses, no RNA stage; Caudovirales; Siphoviridae; unclassified Siphoviridae
GCA_000898575.1	8050	1	Bluegill picornavirus	NA	Viruses; ssRNA viruses; ssRNA positive-strand viruses, no DNA stage; Picornavirales; Picornaviridae; Limnipivirus; Limnipivirus A
GCA_000849225.1	6424	4	Cucumber green mottle mosaic virus	SH	Viruses; ssRNA viruses; ssRNA positive-strand viruses, no DNA stage; Virgaviridae; Tobamovirus
GCA_000898535.1	10180	4	CAS virus	NA	Viruses; ssRNA viruses; ssRNA negative-strand viruses; Arenaviridae; Reptarenavirus; Alethinophid 3 reptarenavirus
GCA_000899995.1	10404	4	Golden Gate virus	NA	Viruses; ssRNA viruses; ssRNA negative-strand viruses; Arenaviridae; Reptarenavirus; Alethinophid 1 reptarenavirus
GCA_000859905.1	6618	5	Odontoglossum ringspot virus	18KDa coat protein	Viruses; ssRNA viruses; ssRNA positive-strand viruses, no DNA stage; Virgaviridae; Tobamovirus
GCA_000859645.1	6357	4	Pepper mild mottle virus	S	Viruses; ssRNA viruses; ssRNA positive-strand viruses, no DNA stage; Virgaviridae; Tobamovirus
GCA_000847545.1	6355	4	Tobacco mild green mosaic virus	NA	Viruses; ssRNA viruses; ssRNA positive-strand viruses, no DNA stage; Virgaviridae; Tobamovirus
GCA_000854365.1	6395	1	Tobacco mosaic virus	NA	Viruses; ssRNA viruses; ssRNA positive-strand viruses, no DNA stage; Virgaviridae; Tobamovirus
GCA_000917855.1	2818	3	Nepavirus	NA	Viruses; unclassified viruses; unclassified DNA viruses; unclassified ssDNA viruses
GCA_004128835.1	8966	2	Picalivirus A	NA	Viruses; Riboviria; unclassified Riboviria; unclassified ssRNA viruses; unclassified ssRNA positive-strand viruses

gca	genome.size	proteome.size	phage.name	strain	Taxonomy
GCA_008785705.1	6939	1	Kobuvirus sewage Kathmandu	NA	Viruses; Riboviria; Orthornavirae; Pisuviricota; Pisoniviricetes; Picornavirales; Picornaviridae; Kobuvirus; Aichivirus A; unclassified Aichivirus A
GCA_000918895.1	2723	7	Pouzolzia golden mosaic virus	NA	Viruses; ssDNA viruses; Geminiviridae; Begomovirus; unclassified Begomovirus
GCA_000853705.1	6383	4	Tomato mosaic virus	NA	Viruses; ssRNA viruses; ssRNA positive-strand viruses, no DNA stage; Virgaviridae; Tobamovirus
GCA_000898635.1	1316	1	Bhendi yellow vein mosaic virusassociated alphasatellite	NA	Viruses; Satellites; Satellite Nucleic Acids; Single stranded DNA satellites; Alphasatellites; Begomovirus-associated alphasatellites; unclassified Begomovirus-associated alphasatellites
GCA_001042255.1	38389	65	Enterobacteria phage SfI	NA	Viruses; dsDNA viruses, no RNA stage; Caudovirales; Myoviridae; unclassified Myoviridae
GCA_000910915.1	42874	53	Pseudomonas phage MPK7	NA	Viruses; dsDNA viruses, no RNA stage; Caudovirales; Podoviridae; Autographivirinae; Phikmvvirus
GCA_002149485.1	39552	55	Pseudomonas phage JBD25	NA	Viruses; dsDNA viruses, no RNA stage; Caudovirales; Siphoviridae; unclassified Siphoviridae
GCA_000901235.1	38002	60	Lactobacillus virus ATCC8014	NA	Viruses; Duplodnaviria; Heunggongvirae; Uroviricota; Caudoviricetes; Caudovirales; Siphoviridae; Coetzeevirus
GCA_002602605.1	80617	127	Lactobacillus phage ATCC 8014B2	NA	Viruses; Duplodnaviria; Heunggongvirae; Uroviricota; Caudoviricetes; Caudovirales; Siphoviridae; Tybeckvirinae; Douglaswolinvirus; Lactobacillus virus B2
GCA_000898775.1	67170	119	Listeria phage P70	NA	Viruses; dsDNA viruses, no RNA stage; Caudovirales; Siphoviridae; P70virus; Listeria virus P70
GCA_002602625.1	41660	66	Streptococcus phage LYGO9	NA	Viruses; Duplodnaviria; Heunggongvirae; Uroviricota; Caudoviricetes; Caudovirales; Siphoviridae; unclassified Siphoviridae
GCA_002602645.1	43028	70	Streptococcus phage JX01	NA	Viruses; Duplodnaviria; Heunggongvirae; Uroviricota; Caudoviricetes; Caudovirales; Siphoviridae; unclassified Siphoviridae
GCA_002602665.1	58866	104	Mycobacterium virus Dorothy	NA	Viruses; Duplodnaviria; Heunggongvirae; Uroviricota; Caudoviricetes; Caudovirales; Siphoviridae; Cheoctovirus
GCA_002602685.1	51235	86	Mycobacterium virus Rebeuca	NA	Viruses; Duplodnaviria; Heunggongvirae; Uroviricota; Caudoviricetes; Caudovirales; Siphoviridae; Fromanvirus
GCA_000862925.1	9657	2	Bean pod mottle virus	KY G7	Viruses; ssRNA viruses; ssRNA positive-strand viruses, no DNA stage; Picornavirales; Secoviridae; Comovirinae; Comovirus
GCA_000860425.1	9576	2	Red clover mottle virus	S	Viruses; ssRNA viruses; ssRNA positive-strand viruses, no DNA stage; Picornavirales; Secoviridae; Comovirinae; Comovirus
GCA_002602705.1	59872	84	Stx2converting phage Stx2a_F349	NA	Viruses; Duplodnaviria; Heunggongvirae; Uroviricota; Caudoviricetes; Caudovirales; Siphoviridae; unclassified Siphoviridae
GCA_002602725.1	64900	84	Stx2converting phage Stx2a_F451	NA	Viruses; Duplodnaviria; Heunggongvirae; Uroviricota; Caudoviricetes; Caudovirales; Podoviridae; Sepvirinae; Traversvirus; unclassified Traversvirus
GCA_002602745.1	62274	86	Stx2converting phage Stx2a_F723	NA	Viruses; Duplodnaviria; Heunggongvirae; Uroviricota; Caudoviricetes; Caudovirales; Podoviridae; Sepvirinae; Traversvirus; unclassified Traversvirus
GCA_002602765.1	63552	76	Stx2converting phage Stx2a_F765	NA	Viruses; Duplodnaviria; Heunggongvirae; Uroviricota; Caudoviricetes; Caudovirales; Podoviridae; Sepvirinae; Oslovirus; unclassified Oslovirus
GCA_002602785.1	56224	70	Stx2converting phage Stx2a_WGPS9	NA	Viruses; Duplodnaviria; Heunggongvirae; Uroviricota; Caudoviricetes; Caudovirales; Podoviridae; Sepvirinae; Traversvirus; unclassified Traversvirus
GCA_002602805.1	57034	65	Stx2converting phage Stx2a_1447	NA	Viruses; Duplodnaviria; Heunggongvirae; Uroviricota; Caudoviricetes; Caudovirales; Siphoviridae; unclassified Siphoviridae
GCA_002602825.1	58326	66	Stx2converting phage Stx2a_WGPS2	NA	Viruses; Duplodnaviria; Heunggongvirae; Uroviricota; Caudoviricetes; Caudovirales; Siphoviridae; unclassified Siphoviridae
GCA_002602845.1	58538	83	Stx2converting phage Stx2a_WGPS4	NA	Viruses; Duplodnaviria; Heunggongvirae; Uroviricota; Caudoviricetes; Caudovirales; Siphoviridae; unclassified Siphoviridae
GCA_002602865.1	57849	78	Stx2converting phage Stx2a_WGPS6	NA	Viruses; Duplodnaviria; Heunggongvirae; Uroviricota; Caudoviricetes; Caudovirales; Siphoviridae; unclassified Siphoviridae
GCA_002602885.1	55023	78	Stx2converting phage Stx2a_WGPS8	NA	Viruses; Duplodnaviria; Heunggongvirae; Uroviricota; Caudoviricetes; Caudovirales; Siphoviridae; unclassified Siphoviridae
GCA_000861625.1	9219	2	Squash mosaic virus	NA	Viruses; ssRNA viruses; ssRNA positive-strand viruses, no DNA stage; Picornavirales; Secoviridae; Comovirinae; Comovirus
GCA_000860385.1	9370	5	Cowpea mosaic virus	NA	Viruses; ssRNA viruses; ssRNA positive-strand viruses, no DNA stage; Picornavirales; Secoviridae; Comovirinae; Comovirus
GCA_000852165.1	5338	5	Red clover necrotic mosaic virus	NA	Viruses; ssRNA viruses; ssRNA positive-strand viruses, no DNA stage; Tombusviridae; Dianthovirus
GCA_000920395.1	8228	7	Canis familiaris papillomavirus 13	NA	Viruses; dsDNA viruses, no RNA stage; Papillomaviridae; Taupapillomavirus; Taupapillomavirus 2
GCA_000852765.1	5243	5	Carnation ringspot virus	NA	Viruses; ssRNA viruses; ssRNA positive-strand viruses, no DNA stage; Tombusviridae; Dianthovirus
GCA_000855205.1	11154	2	Arabis mosaic virus	NA	Viruses; ssRNA viruses; ssRNA positive-strand viruses, no DNA stage; Picornavirales; Secoviridae; Comovirinae; Nepovirus; Subgroup A
GCA_000862025.1	11653	2	Grapevine chrome mosaic virus	NA	Viruses; ssRNA viruses; ssRNA positive-strand viruses, no DNA stage; Picornavirales; Secoviridae; Comovirinae; Nepovirus; Subgroup B
GCA_000902035.1	5279	7	Bean chlorosis virus	NA	Viruses; ssDNA viruses; Geminiviridae; Begomovirus

gca	genome.size	proteome.size	phage.name	strain	Taxonomy
GCA_000900175.1	5214	7	Dalechampia chlorotic mosaic virus	NA	Viruses; ssDNA viruses; Geminiviridae; Begomovirus
GCA_000897735.1	5163	7	Datura leaf distortion virus	NA	Viruses; ssDNA viruses; Geminiviridae; Begomovirus
GCA_000860305.1	11116	3	Grapevine fanleaf virus	F13	Viruses; ssRNA viruses; ssRNA positive-strand viruses, no DNA stage; Picornavirales; Secoviridae; Comovirinae; Nepovirus; Subgroup A
GCA_000853325.1	11991	2	Tomato black ring virus	NA	Viruses; ssRNA viruses; ssRNA positive-strand viruses, no DNA stage; Picornavirales; Secoviridae; Comovirinae; Nepovirus; Subgroup B
GCA_000930135.1	6945	2	Mulberry badnavirus 1	NA	Viruses; Riboviria; Pararnavirae; Artverviricota; Revtraviricetes; Ortervirales; Caulimoviridae; Badnavirus
GCA_000860465.1	15485	4	Tomato ringspot virus	raspberry	Viruses; ssRNA viruses; ssRNA positive-strand viruses, no DNA stage; Picornavirales; Secoviridae; Comovirinae; Nepovirus; Subgroup C
GCA_000917415.1	9501	1	Spodoptera exigua iflavirus 2	NA	Viruses; ssRNA viruses; ssRNA positive-strand viruses, no DNA stage; Picornavirales; Iflaviridae; Iflavirus
GCA_000856105.1	11443	2	Tobacco ringspot virus	Bud Blight	Viruses; ssRNA viruses; ssRNA positive-strand viruses, no DNA stage; Picornavirales; Secoviridae; Comovirinae; Nepovirus; Subgroup A
GCA_000855345.1	4505	5	Black beetle virus	NA	Viruses; ssRNA viruses; ssRNA positive-strand viruses, no DNA stage; Nodaviridae; Alphanodavirus
GCA_000852425.1	4401	3	Boolarra virus	NA	Viruses; ssRNA viruses; ssRNA positive-strand viruses, no DNA stage; Nodaviridae; Alphanodavirus
GCA_000854385.1	4507	4	Flock house virus	NA	Viruses; ssRNA viruses; ssRNA positive-strand viruses, no DNA stage; Nodaviridae; Alphanodavirus
GCA_000847805.1	4540	3	Nodamura virus	NA	Viruses; ssRNA viruses; ssRNA positive-strand viruses, no DNA stage; Nodaviridae; Alphanodavirus
GCA_000926615.1	15444	10	Malakal virus	NA	Viruses; ssRNA viruses; ssRNA negative-strand viruses; Mononegavirales; Rhabdoviridae; Ephemerovirus; unclassified Ephemerovirus
GCA_008703965.1	7677	3	Norovirus GI	HuJP1998GIPNAdGI6No20Saitama9817	Viruses; Riboviria; Orthornavirae; Pisuviricota; Pisoniviricetes; Picornavirales; Caliciviridae; Norovirus; Norwalk virus
GCA_003638685.1	7567	3	Norovirus GII	NA	Viruses; Riboviria; Orthornavirae; Pisuviricota; Pisoniviricetes; Picornavirales; Caliciviridae; Norovirus; Norwalk virus
GCA_000917875.1	2806	3	Baminivirus	NA	Viruses; ssDNA viruses; Geminiviridae; unclassified Geminiviridae
GCA_000919535.1	2383	4	Niminivirus	NA	Viruses; ssDNA viruses; Geminiviridae; unclassified Geminiviridae
GCA_000855885.1	10447	7	Pea earlybrowning virus	NA	Viruses; ssRNA viruses; ssRNA positive-strand viruses, no DNA stage; Virgaviridae; Tobravirus
GCA_000851445.1	10646	7	Tobacco rattle virus	NA	Viruses; ssRNA viruses; ssRNA positive-strand viruses, no DNA stage; Virgaviridae; Tobravirus
GCA_003047875.1	45469	64	Pseudomonas phage vB_PaeP_C114_Or	NA	Viruses; Duplodnaviria; Heunggongvirae; Uroviricota; Caudoviricetes; Caudovirales; Podoviridae; Bruynoghevirus; unclassified Bruynoghevirus
GCA_000910615.1	30910	55	Lactococcus phage BM13	NA	Viruses; dsDNA viruses, no RNA stage; Caudovirales; Siphoviridae; unclassified Siphoviridae
GCA_000899455.1	133572	162	Campylobacter phage CP30A	NA	Viruses; dsDNA viruses, no RNA stage; Caudovirales; Myoviridae; unclassified Myoviridae
GCA_000906475.1	147737	269	Escherichia phage phAPEC8	NA	Viruses; dsDNA viruses, no RNA stage; Caudovirales; Myoviridae; unclassified Myoviridae
GCA_000902755.1	41351	62	Vibrio phage vB_VpaM_MAR	NA	Viruses; dsDNA viruses, no RNA stage; Caudovirales; Myoviridae; Vhmlvirus; Vibrio virus MAR
GCA_000902135.1	78751	107	Vibrio phage vB_VpaS_MAR10	NA	Viruses; dsDNA viruses, no RNA stage; Caudovirales; Siphoviridae; Ssp2virus; Vibrio virus MAR10
GCA_001041415.1	86231	136	Escherichia phage EC6	NA	Viruses; dsDNA viruses, no RNA stage; Caudovirales; Myoviridae; unclassified Myoviridae
GCA_001503595.1	43216	82	Acinetobacter phage phiAC1	NA	Viruses; dsDNA viruses, no RNA stage; Caudovirales; Myoviridae; unclassified Myoviridae
GCA_002602905.1	59363	71	Acidithiobacillus phage AcaML1	NA	Viruses; Duplodnaviria; Heunggongvirae; Uroviricota; Caudoviricetes; Caudovirales; Myoviridae; unclassified Myoviridae
GCA_000899375.1	29214	45	Propionibacterium phage P91	NA	Viruses; dsDNA viruses, no RNA stage; Caudovirales; Siphoviridae; unclassified Siphoviridae
GCA_000898795.1	29729	47	Propionibacterium phage P144	NA	Viruses; dsDNA viruses, no RNA stage; Caudovirales; Siphoviridae; unclassified Siphoviridae
GCA_000900255.1	29574	47	Propionibacterium phage P101A	NA	Viruses; dsDNA viruses, no RNA stage; Caudovirales; Siphoviridae; unclassified Siphoviridae
GCA_000897815.1	29371	45	Propionibacterium phage P104A	NA	Viruses; dsDNA viruses, no RNA stage; Caudovirales; Siphoviridae; unclassified Siphoviridae
GCA_000899395.1	29202	45	Propionibacterium phage P105	NA	Viruses; dsDNA viruses, no RNA stage; Caudovirales; Siphoviridae; unclassified Siphoviridae
GCA_000898815.1	29506	47	Propionibacterium phage P100D	NA	Viruses; dsDNA viruses, no RNA stage; Caudovirales; Siphoviridae; unclassified Siphoviridae

gca	genome.size	proteome.size	phage.name	strain	Taxonomy
GCA_000900275.1	29505	45	Propionibacterium phage P100_A	NA	Viruses; dsDNA viruses, no RNA stage; Caudovirales; Siphoviridae; unclassified Siphoviridae
GCA_000897835.1	29612	47	Propionibacterium phage P100_1	NA	Viruses; dsDNA viruses, no RNA stage; Caudovirales; Siphoviridae; unclassified Siphoviridae
GCA_000899415.1	29348	45	Propionibacterium phage P11	NA	Viruses; dsDNA viruses, no RNA stage; Caudovirales; Siphoviridae; unclassified Siphoviridae
GCA_000898835.1	29516	46	Propionibacterium phage ATCC29399B_T	NA	Viruses; dsDNA viruses, no RNA stage; Caudovirales; Siphoviridae; unclassified Siphoviridae
GCA_000900295.1	29516	46	Propionibacterium phage ATCC29399B_C	NA	Viruses; dsDNA viruses, no RNA stage; Caudovirales; Siphoviridae; unclassified Siphoviridae
GCA_000851565.1	8250	4	Broad bean mottle virus	NA	Viruses; ssRNA viruses; ssRNA positive-strand viruses, no DNA stage; Bromoviridae; Bromovirus
GCA_000854965.1	8210	4	Brome mosaic virus	Russian	Viruses; ssRNA viruses; ssRNA positive-strand viruses, no DNA stage; Bromoviridae; Bromovirus
GCA_900004605.1	13133	11	Influenza A virus AEngland050000882010H1N1	NA	Viruses; Riboviria; Orthornavirae; Negarnaviricota; Polyploviricotina; Insthoviricetes; Articulavirales; Orthomyxoviridae; Alphainfluenzavirus; Influenza A virus; H1N1 subtype
GCA_002831165.1	11202	3	Herbert virus strain F23CI2004	NA	Viruses; Riboviria; Orthornavirae; Negarnaviricota; Polyploviricotina; Ellioviricetes; Bunyavirales; Peribunyaviridae; Herbevirus; Herbert herbevirus
GCA_000851205.1	8118	4	Cowpea chlorotic mottle virus	NA	Viruses; ssRNA viruses; ssRNA positive-strand viruses, no DNA stage; Bromoviridae; Bromovirus
GCA_002602925.1	37159	51	Nitratiruptor phage NrS1	NA	Viruses; Duplodnaviria; Heunggongvirae; Uroviricota; Caudoviricetes; Caudovirales; Siphoviridae; unclassified Siphoviridae
GCA_003110345.1	8634	5	Cucumber mosaic virus	ALS	Viruses; ssRNA viruses; ssRNA positive-strand viruses, no DNA stage; Bromoviridae; Cucumovirus
GCA_000902875.1	92777	158	Pseudomonas phage vB_PaeM_C210_Ab1	NA	Viruses; dsDNA viruses, no RNA stage; Caudovirales; Myoviridae; Pakpunavirus; Pseudomonas virus CAb1
GCA_000898675.1	1478	1	Cassava mosaic Madagascar alphasatellite	NA	Viruses; Satellites; Satellite Nucleic Acids; Single stranded DNA satellites; Alphasatellites; Begomovirus-associated alphasatellites; unclassified Begomovirus-associated alphasatellites
GCA_000863785.1	8492	4	Peanut stunt virus	ER	Viruses; ssRNA viruses; ssRNA positive-strand viruses, no DNA stage; Bromoviridae; Cucumovirus
GCA_000899255.1	1362	1	Tomato leaf curl Hajipur betasatellite	NA	Viruses; Tolecusatellitidae; Betasatellite; unclassified Betasatellite
GCA_000853065.1	8870	4	Tomato aspermy virus	V	Viruses; ssRNA viruses; ssRNA positive-strand viruses, no DNA stage; Bromoviridae; Cucumovirus
GCA_000865505.1	8622	5	Tobacco streak virus	NA	Viruses; ssRNA viruses; ssRNA positive-strand viruses, no DNA stage; Bromoviridae; Ilarvirus; Ilarvirus subgroup 1
GCA_000849545.1	8511	4	Apple mosaic virus	NA	Viruses; ssRNA viruses; ssRNA positive-strand viruses, no DNA stage; Bromoviridae; Ilarvirus; Ilarvirus subgroup 3
GCA_000898655.1	11634	3	Eilat virus	NA	Viruses; ssRNA viruses; ssRNA positive-strand viruses, no DNA stage; Togaviridae; Alphavirus
GCA_000847525.1	8274	4	Alfalfa mosaic virus	NA	Viruses; ssRNA viruses; ssRNA positive-strand viruses, no DNA stage; Bromoviridae; Alfamovirus
GCA_000855725.1	10221	7	Barley stripe mosaic virus	type strain ATCCPV43	Viruses; ssRNA viruses; ssRNA positive-strand viruses, no DNA stage; Virgaviridae; Hordeivirus
GCA_000851385.1	17145	7	Rice stripe virus	NA	Viruses; ssRNA viruses; ssRNA negative-strand viruses; unassigned ssRNA negative-strand viruses; Tenuivirus
GCA_002890455.1	16916	7	Rice hoja blanca tenuivirus	NA	Viruses; Riboviria; Orthornavirae; Negarnaviricota; Polyploviricotina; Ellioviricetes; Bunyavirales; Phenuiviridae; Tenuivirus
GCA_000920655.1	7802	1	Human cosavirus	NA	Viruses; ssRNA viruses; ssRNA positive-strand viruses, no DNA stage; Picornavirales; Picornaviridae; Cosavirus; unclassified Cosavirus
GCA_000910375.1	42313	53	Vibrio phage VPMS1	NA	Viruses; dsDNA viruses, no RNA stage; Caudovirales; Podoviridae; unclassified Podoviridae
GCA_000865225.1	185683	198	Clostridium phage cst	NA	Viruses; dsDNA viruses, no RNA stage; Caudovirales; Myoviridae; unclassified Myoviridae
GCA_000870405.1	2762	6	Honeysuckle yellow vein Kagoshima virus	NA	Viruses; ssDNA viruses; Geminiviridae; Begomovirus
GCA_000902175.1	44030	58	Pseudomonas phage vB_PaeP_p210_Or1	NA	Viruses; dsDNA viruses, no RNA stage; Caudovirales; Podoviridae; Luz24virus; unclassified Luz24likevirus; Pseudomonas phage MR299-2; Pseudomonas phage vB_PaeP_C1-14_Or
GCA_000873305.1	34659	51	Lactobacillus phage LLH	NA	Viruses; dsDNA viruses, no RNA stage; Caudovirales; Siphoviridae; unclassified Siphoviridae
GCA_000917895.1	2067	2	Dragonfly circularisvirus	NA	Viruses; unclassified viruses; unclassified DNA viruses; unclassified ssDNA viruses; Circularisvirus
GCA_000918915.1	1915	2	Dragonfly orbiculatusvirus	NA	Viruses; unclassified viruses; unclassified DNA viruses; unclassified ssDNA viruses
GCA_000919555.1	1831	2	Dragonfly cyclicusvirus	NA	Viruses; unclassified viruses; unclassified DNA viruses; unclassified ssDNA viruses
GCA_000920575.1	1741	2	Dragonfly cyclovirus 1	NA	Viruses; ssDNA viruses; Circoviridae; unclassified Circoviridae; suggested genus Cyclovirus

gca	genome.size	proteome.size	phage.name	strain	Taxonomy
GCA_000919015.1	1896	2	Dragonfly cyclovirus 2	NA	Viruses; ssDNA viruses; Circoviridae; unclassified Circoviridae; suggested genus Cyclovirus
GCA_000917995.1	1711	3	Dragonfly cyclovirus 3	NA	Viruses; ssDNA viruses; Circoviridae; unclassified Circoviridae; suggested genus Cyclovirus
GCA_000919655.1	1740	2	Dragonfly cyclovirus 4	NA	Viruses; ssDNA viruses; Circoviridae; unclassified Circoviridae; suggested genus Cyclovirus
GCA_000920495.1	1784	2	Dragonfly cyclovirus 5	NA	Viruses; ssDNA viruses; Circoviridae; unclassified Circoviridae; suggested genus Cyclovirus
GCA_000918995.1	2208	4	Dragonflyassociated circular virus 3	NA	Viruses; ssDNA viruses; unclassified ssDNA viruses
GCA_000919635.1	2236	4	Dragonflyassociated circular virus 2	NA	Viruses; ssDNA viruses; unclassified ssDNA viruses
GCA_000917975.1	2225	4	Dragonflyassociated circular virus 1	NA	Viruses; ssDNA viruses; unclassified ssDNA viruses
GCA_000901395.1	4472	5	Dragonflyassociated microphage 1	NA	Viruses; ssDNA viruses; unclassified ssDNA viruses
GCA_000858005.1	167560	273	Escherichia phage RB69	NA	Viruses; Duplodnaviria; Heunggongvirae; Uroviricota; Caudoviricetes; Caudovirales; Myoviridae; Tevenvirinae; Mosigvirus; Escherichia virus RB69
GCA_000906615.1	1230522	1176	Megavirus lba	NA	Viruses; Varidnaviria; Bamfordvirae; Nucleocytoviricota; Megaviricetes; Imitervirales; Mimiviridae; Mimivirus; unclassified Mimivirus; Megavirus chiliensis
GCA_000906795.1	7551	7	Equus caballus papillomavirus 6	NA	Viruses; Monodnaviria; Shotokuvirae; Cossaviricota; Papovaviricetes; Zurhausenvirales; Papillomaviridae; Firstpapillomavirinae; Dyorhopapillomavirus; Dyorhopapillomavirus 1
GCA_000906495.1	7554	7	Equus ferus caballus papillomavirus type 4	NA	Viruses; dsDNA viruses, no RNA stage; Papillomaviridae; unclassified Papillomaviridae; non-primate mammal papillomaviruses
GCA_000904015.1	7519	7	Equus ferus caballus papillomavirus type 5	NA	Viruses; dsDNA viruses, no RNA stage; Papillomaviridae; unclassified Papillomaviridae; non-primate mammal papillomaviruses
GCA_000904315.1	7619	7	Equus ferus caballus papillomavirus type 7	NA	Viruses; dsDNA viruses, no RNA stage; Papillomaviridae; unclassified Papillomaviridae; non-primate mammal papillomaviruses
GCA_000897855.1	58104	41	Providencia phage Redjac	NA	Viruses; dsDNA viruses, no RNA stage; Caudovirales; unclassified Caudovirales
GCA_000902155.1	3405	4	Enterobacteria phage M	NA	Viruses; ssRNA viruses; ssRNA positive-strand viruses, no DNA stage; Leviviridae; unclassified Leviviridae
GCA_000911055.1	29511	45	Propionibacterium phage PHL010M04	NA	Viruses; dsDNA viruses, no RNA stage; Caudovirales; Siphoviridae; unclassified Siphoviridae
GCA_002623045.1	29443	45	Propionibacterium phage PHL037M02	NA	Viruses; Duplodnaviria; Heunggongvirae; Uroviricota; Caudoviricetes; Caudovirales; Siphoviridae; Pahexavirus; Propionibacterium virus PHL037M02
GCA_000912735.1	29514	46	Propionibacterium phage PHL060L00	NA	Viruses; dsDNA viruses, no RNA stage; Caudovirales; Siphoviridae; unclassified Siphoviridae
GCA_002755375.1	29512	45	Propionibacterium phage PHL066M04	NA	Viruses; Duplodnaviria; Heunggongvirae; Uroviricota; Caudoviricetes; Caudovirales; Siphoviridae; Pahexavirus; unclassified Pahexavirus
GCA_000912075.1	29377	45	Propionibacterium phage PHL067M10	NA	Viruses; dsDNA viruses, no RNA stage; Caudovirales; Siphoviridae; unclassified Siphoviridae
GCA_000911015.1	29467	45	Propionibacterium phage PHL071N05	NA	Viruses; dsDNA viruses, no RNA stage; Caudovirales; Siphoviridae; unclassified Siphoviridae
GCA_002755395.1	29503	45	Propionibacterium phage PHL073M02	NA	Viruses; Duplodnaviria; Heunggongvirae; Uroviricota; Caudoviricetes; Caudovirales; Siphoviridae; Pahexavirus; unclassified Pahexavirus
GCA_002755415.1	29451	45	Propionibacterium phage PHL085M01	NA	Viruses; Duplodnaviria; Heunggongvirae; Uroviricota; Caudoviricetes; Caudovirales; Siphoviridae; Pahexavirus; unclassified Pahexavirus
GCA_000912055.1	29140	45	Propionibacterium phage PHL111M01	NA	Viruses; dsDNA viruses, no RNA stage; Caudovirales; Siphoviridae; unclassified Siphoviridae
GCA_000912675.1	29266	46	Propionibacterium phage PHL112N00	NA	Viruses; dsDNA viruses, no RNA stage; Caudovirales; Siphoviridae; unclassified Siphoviridae
GCA_000912035.1	29200	44	Propionibacterium phage PHL113M01	NA	Viruses; dsDNA viruses, no RNA stage; Caudovirales; Siphoviridae; unclassified Siphoviridae
GCA_000913515.1	29464	46	Propionibacterium phage PHL114L00	NA	Viruses; dsDNA viruses, no RNA stage; Caudovirales; Siphoviridae; unclassified Siphoviridae
GCA_002755435.1	29453	45	Propionibacterium phage PHL115M02	NA	Viruses; Duplodnaviria; Heunggongvirae; Uroviricota; Caudoviricetes; Caudovirales; Siphoviridae; Pahexavirus; unclassified Pahexavirus
GCA_000910895.1	134643	174	Staphylococcus phage vB_SauM_Remus	NA	Viruses; dsDNA viruses, no RNA stage; Caudovirales; Myoviridae; Spounavirinae; Twortvirus; unclassified Twortlikevirus
GCA_000907055.1	131332	164	Staphylococcus phage vB_SauM_Romulus	NA	Viruses; dsDNA viruses, no RNA stage; Caudovirales; Myoviridae; Spounavirinae; Twortvirus; unclassified Twortlikevirus
GCA_000900335.1	45517	75	Pseudomonas phage UFVP2	NA	Viruses; dsDNA viruses, no RNA stage; Caudovirales; Podoviridae; unclassified Podoviridae
GCA_000902895.1	42689	65	Pseudomonas phage AF	Pseudomonas putida GB1	Viruses; dsDNA viruses, no RNA stage; Caudovirales; Podoviridae; unclassified Podoviridae
GCA_002600425.1	68910	105	Mycobacterium phage Alex	NA	Viruses; Duplodnaviria; Heunggongvirae; Uroviricota; Caudoviricetes; Caudovirales; Siphoviridae; Bclasvirinae; Pegunavirus; Mycobacterium virus Pg1

gca	genome.size	proteome.size	phage.name	strain	Taxonomy
GCA_002600405.1	68004	99	Mycobacterium phage Gyarad	NA	Viruses; Duplodnaviria; Heunggongvirae; Uroviricota; Caudoviricetes; Caudovirales; Siphoviridae; Bclasvirinae; Pegunavirus; Mycobacterium virus Soto
GCA_002600385.1	69321	104	Mycobacterium phage Nacho	NA	Viruses; Duplodnaviria; Heunggongvirae; Uroviricota; Caudoviricetes; Caudovirales; Siphoviridae; Bclasvirinae; Pegunavirus; Mycobacterium virus Suffolk
GCA_002600365.2	68992	101	Mycobacterium phage Piglet	NA	Viruses; Duplodnaviria; Heunggongvirae; Uroviricota; Caudoviricetes; Caudovirales; Siphoviridae; Bclasvirinae; Pegunavirus; Mycobacterium virus Pg1
GCA_002600345.1	68884	104	Mycobacterium phage Serpentine	NA	Viruses; Duplodnaviria; Heunggongvirae; Uroviricota; Caudoviricetes; Caudovirales; Siphoviridae; Bclasvirinae; Pegunavirus; Mycobacterium virus Pg1
GCA_002755455.1	37637	47	Burkholderia phage phiX216	NA	Viruses; Duplodnaviria; Heunggongvirae; Uroviricota; Caudoviricetes; Caudovirales; Myoviridae; Peduovirinae; Tigrvirus; Burkholderia virus phi52237
GCA_000907515.1	42185	54	Acinetobacter phage Abp1	NA	Viruses; dsDNA viruses, no RNA stage; Caudovirales; Podoviridae; unclassified Podoviridae
GCA_000901155.1	30119	11	Human betacoronavirus 2c EMC2012	HCoVEMC	Viruses; Riboviria; Orthornavirae; Pisuviricota; Pisoniviricetes; Nidovirales; Cornidovirineae; Coronaviridae; Orthocoronavirinae; Betacoronavirus; Merbecovirus; Middle East respiratory syndrome-related coronavirus
GCA_000905755.1	48814	68	Klebsiella phage JD001	NA	Viruses; dsDNA viruses, no RNA stage; Caudovirales; Myoviridae; unclassified Myoviridae
GCA_000898695.1	6471	2	Culex originated Tymoviridaelike virus	NA	Viruses; ssRNA viruses; ssRNA positive-strand viruses, no DNA stage; Tymovirales; Tymoviridae; Maculavirus; unclassified Maculavirus
GCA_004787355.1	129492	95	Macaca nemestrina rhadinovirus 2	J97167	Viruses; Duplodnaviria; Heunggongvirae; Peploviricota; Herviviricetes; Herpesvirales; Herpesviridae; Gammaherpesvirinae; Rhadinovirus; Macacine gammaherpesvirus 12
GCA_000902815.1	5273	4	Ateles paniscus polyomavirus 1	NA	Viruses; dsDNA viruses, no RNA stage; Polyomaviridae; Alphapolyomavirus
GCA_000902195.1	5013	4	Cebus albifrons polyomavirus 1	NA	Viruses; dsDNA viruses, no RNA stage; Polyomaviridae; Betapolyomavirus
GCA_000929995.1	5189	5	Cercopithecus erythrotis polyomavirus 1	NA	Viruses; dsDNA viruses, no RNA stage; Polyomaviridae; Betapolyomavirus
GCA_000903715.1	5087	4	Macaca fascicularis polyomavirus 1	NA	Viruses; dsDNA viruses, no RNA stage; Polyomaviridae; Alphapolyomavirus
GCA_001184945.1	5159	6	Pan troglodytes troglodytes polyomavirus 1	NA	Viruses; dsDNA viruses, no RNA stage; Polyomaviridae; unclassified Polyomaviridae
GCA_003033345.1	5148	5	Piliocolobus badius polyomavirus 2	NA	Viruses; Monodnaviria; Shotokuvirae; Cossaviricota; Papovaviricetes; Sepolyvirales; Polyomaviridae; Alphapolyomavirus; Piliocolobus badius polyomavirus 1
GCA_000901195.1	5140	4	Piliocolobus rufomitratus polyomavirus 1	NA	Viruses; dsDNA viruses, no RNA stage; Polyomaviridae; Alphapolyomavirus
GCA_002827925.1	5067	5	Saimiri sciureus polyomavirus 1	NA	Viruses; Monodnaviria; Shotokuvirae; Cossaviricota; Papovaviricetes; Sepolyvirales; Polyomaviridae; Betapolyomavirus
GCA_000904835.1	36932	53	Bacillus virus BMBtp2	NA	Viruses; dsDNA viruses, no RNA stage; Caudovirales; Siphoviridae; Tp21virus
GCA_000902855.1	7826	7	Canis familiaris papillomavirus 14	NA	Viruses; Monodnaviria; Shotokuvirae; Cossaviricota; Papovaviricetes; Zurhausenvirales; Papillomaviridae; Firstpapillomavirinae; Chipapillomavirus; Chipapillomavirus 3
GCA_013086835.1	6638	2	Mosquitoe x virus	NA	Viruses; Riboviria; Orthornavirae; Birnaviridae; unclassified Birnaviridae
GCA_000899435.1	77448	109	Escherichia phage NJ01	NA	Viruses; Duplodnaviria; Heunggongvirae; Uroviricota; Caudoviricetes; Caudovirales; Podoviridae; Kuravirus; Escherichia virus NJ01
GCA_000844945.1	2773	6	Tomato yellow leaf curl Sardinia virus	NA	Viruses; ssDNA viruses; Geminiviridae; Begomovirus
GCA_000901375.1	169158	62	Escherichia phage HX01	NA	Viruses; dsDNA viruses, no RNA stage; Caudovirales; Myoviridae; Tevenvirinae; Rb69virus; Escherichia virus HX01
GCA_000900355.1	1360	1	Mungbean yellow mosaic India virus associated betasatellite India Faizabad Cow Pea2012	NA	Viruses; Tolecusatellitidae; Betasatellite; Mungbean yellow mosaic betasatellite
GCA_002816415.1	20307	6	Dak Nong virus	HL30	Viruses; Riboviria; Orthornavirae; Pisuviricota; Pisoniviricetes; Nidovirales; Mesnidovirineae; Mesoniviridae; Hexponivirinae; Alphamesonivirus; Karsalivirus; Alphamesonivirus 3
GCA_000843485.1	32172	51	Lactococcus phage phiLC3	IMNC3	Viruses; dsDNA viruses, no RNA stage; Caudovirales; Siphoviridae; unclassified Siphoviridae
GCA_000901595.1	37282	55	Streptococcus phage phiNJ2	NA	Viruses; dsDNA viruses, no RNA stage; Caudovirales; Siphoviridae; unclassified Siphoviridae
GCA_000903135.1	38513	45	Stenotrophomonas phage IME15	NA	Viruses; dsDNA viruses, no RNA stage; Caudovirales; Podoviridae; Autographivirinae; T7virus; unclassified T7-like viruses
GCA_000903675.1	141836	217	Staphylococcus phage JD007	NA	Viruses; dsDNA viruses, no RNA stage; Caudovirales; Myoviridae; Spounavirinae; Kayvirus; Staphylococcus virus JD7
GCA_000903115.1	72570	91	Escherichia phage IME11	NA	Viruses; dsDNA viruses, no RNA stage; Caudovirales; Podoviridae; Picovirinae; G7cvirus; Escherichia virus IME11
GCA_002597525.1	137031	198	Staphylococcus phage SA5	NA	Viruses; Duplodnaviria; Heunggongvirae; Uroviricota; Caudoviricetes; Caudovirales; Herelleviridae; Twortvirinae; Kayvirus; Staphylococcus virus G1
GCA_002602965.1	50348	72	Streptomyces phage phiCAM	NA	Viruses; Duplodnaviria; Heunggongvirae; Uroviricota; Caudoviricetes; Caudovirales; Siphoviridae; Arquatrovirinae; Camvirus; Streptomyces virus phiCAM
GCA_000857825.1	7003	3	Avastrovirus 1	NA	Viruses; Riboviria; Orthornavirae; Pisuviricota; Stelpaviricetes; Stellavirales; Astroviridae; Avastrovirus

gca	genome.size	proteome.size	phage.name	strain	Taxonomy
GCA_000856205.1	7355	3	Avastrovirus 3	NA	Viruses; Riboviria; Orthornavirae; Pisuviricota; Stelpaviricetes; Stellavirales; Astroviridae; Avastrovirus
GCA_000906515.1	9796	2	Brugmansia mosaic virus	SK	Viruses; ssRNA viruses; ssRNA positive-strand viruses, no DNA stage; Potyviridae; unclassified Potyviridae
GCA_001736695.1	6673	2	Mamastrovirus 1	NA	Viruses; ssRNA viruses; ssRNA positive-strand viruses, no DNA stage; Astroviridae; Mamastrovirus
GCA_002194425.1	6347	3	Mamastrovirus 2	NA	Viruses; ssRNA viruses; ssRNA positive-strand viruses, no DNA stage; Astroviridae; Mamastrovirus
GCA_000927095.2	6705	3	Mamastrovirus 3	NA	Viruses; ssRNA viruses; ssRNA positive-strand viruses, no DNA stage; Astroviridae; Mamastrovirus
GCA_000884395.1	6534	3	Mamastrovirus 8	NA	Viruses; Riboviria; Orthornavirae; Pisuviricota; Stelpaviricetes; Stellavirales; Astroviridae; Mamastrovirus
GCA_000853425.1	6610	3	Mamastrovirus 10	NA	Viruses; ssRNA viruses; ssRNA positive-strand viruses, no DNA stage; Astroviridae; Mamastrovirus
GCA_000855165.1	6440	3	Mamastrovirus 13	NA	Viruses; ssRNA viruses; ssRNA positive-strand viruses, no DNA stage; Astroviridae; Mamastrovirus
GCA_002818805.1	2899	4	Torque teno sus virus k2b	NA	Viruses; Anelloviridae; Kappatorquevirus
GCA_008797295.1	7108	1	Human rhinovirus C11	CL170085	Viruses; Riboviria; Orthornavirae; Pisuviricota; Pisoniviricetes; Picornavirales; Picornaviridae; Enterovirus; Rhinovirus C
GCA_000848885.1	42935	73	Streptococcus phage EJ1	NA	Viruses; dsDNA viruses, no RNA stage; Caudovirales; Myoviridae; unclassified Myoviridae
GCA_000843405.1	30636	42	Salmonella virus PsP3	NA	Viruses; dsDNA viruses, no RNA stage; Caudovirales; Myoviridae; Peduovirinae; P2virus
GCA_001504015.1	2788	6	Okra leaf curl Oman virus	NA	Viruses; ssDNA viruses; Geminiviridae; Begomovirus; unclassified Begomovirus
GCA_000900375.1	211518	118	Abalone herpesvirus VictoriaAUS2009	NA	Viruses; dsDNA viruses, no RNA stage; Herpesvirales; unclassified Herpesvirales
GCA_000848805.1	43785	63	Lactobacillus phage phiadh	NA	Viruses; dsDNA viruses, no RNA stage; Caudovirales; Siphoviridae; unclassified Siphoviridae
GCA_001429915.1	6410	3	Le Blanc nodavirus	JU1498	Viruses; ssRNA viruses; ssRNA positive-strand viruses, no DNA stage; Nodaviridae; unclassified Nodaviridae
GCA_000899495.1	28494	9	Rousettus bat coronavirus HKU10	NA	Viruses; ssRNA viruses; ssRNA positive-strand viruses, no DNA stage; Nidovirales; Coronaviridae; Coronavirinae; Alphacoronavirus; Bat coronavirus HKU10
GCA_000914195.1	5805	2	Porcine parvovirus 5	NA	Viruses; ssDNA viruses; Parvoviridae; Parvovirinae; Protoparvovirus; unclassified Protoparvovirus
GCA_013087155.1	129528	139	Spodoptera exempta nucleopolyhedrovirus	2441	Viruses; Baculoviridae; Alphabaculovirus
GCA_000915015.1	8424	6	Garlic virus D	NA	Viruses; ssRNA viruses; ssRNA positive-strand viruses, no DNA stage; Tymovirales; Alphaflexiviridae; Allexivirus
GCA_000847865.1	8405	6	Garlic virus C	NA	Viruses; ssRNA viruses; ssRNA positive-strand viruses, no DNA stage; Tymovirales; Alphaflexiviridae; Allexivirus
GCA_000900395.1	8328	5	Rubus canadensis virus 1	NA	Viruses; ssRNA viruses; ssRNA positive-strand viruses, no DNA stage; Tymovirales; Betaflexiviridae; Quinvirinae; Foveavirus
GCA_002602985.1	43665	82	Acinetobacter phage IMEAB2	NA	Viruses; Duplodnaviria; Heunggongvirae; Uroviricota; Caudoviricetes; Caudovirales; Myoviridae; Obolenskvirus; Acinetobacter virus AB2
GCA_000928855.1	8336	6	Garlic virus B	NA	Viruses; ssRNA viruses; ssRNA positive-strand viruses, no DNA stage; Tymovirales; Alphaflexiviridae; Allexivirus
GCA_000848665.1	8660	6	Garlic virus A	NA	Viruses; ssRNA viruses; ssRNA positive-strand viruses, no DNA stage; Tymovirales; Alphaflexiviridae; Allexivirus
GCA_004130945.1	10280	1	Culex theileri flavivirus	JKT8650	Viruses; Riboviria; Orthornavirae; Kitrinoviricota; Flasuviricetes; Amarillovirales; Flaviviridae; Flavivirus; unclassified Flavivirus
GCA_002116075.1	10370	1	Ochlerotatus caspius flavivirus	NA	Viruses; ssRNA viruses; ssRNA positive-strand viruses, no DNA stage; Flaviviridae; Flavivirus; unclassified Flavivirus
GCA_000900595.1	41549	48	Edwardsiella phage KF1	NA	Viruses; dsDNA viruses, no RNA stage; Caudovirales; Podoviridae; unclassified Podoviridae
GCA_002603005.1	41684	48	Edwardsiella phage IW1	NA	Viruses; Duplodnaviria; Heunggongvirae; Uroviricota; Caudoviricetes; Caudovirales; Podoviridae; Kafunavirus; Edwardsiella virus KF1; Edwardsiella phage KF-1
GCA_000848385.1	1801	1	Saccharomyces cerevisiae killer virus M1	TF325	Viruses; dsRNA viruses; unclassified dsRNA viruses
GCA_000851365.1	7680	3	Raspberry bushy dwarf virus	NA	Viruses; ssRNA viruses; ssRNA positive-strand viruses, no DNA stage; Idaeovirus
GCA_003033305.1	4909	2	Wuharv parvovirus 1	NA	Viruses; Monodnaviria; Shotokuvirae; Cossaviricota; Quintoviricetes; Piccovirales; Parvoviridae; Parvovirinae; Protoparvovirus; Primate protoparvovirus 2
GCA_008777515.1	8059	1	WUHARV Sapelovirus 1	NA	Viruses; Riboviria; Orthornavirae; Pisuviricota; Pisoniviricetes; Picornavirales; Picornaviridae; Sapelovirus; unclassified Sapelovirus
GCA_000861865.1	6813	2	Human astrovirus 1	NA	Viruses; Riboviria; Orthornavirae; Pisuviricota; Stelpaviricetes; Stellavirales; Astroviridae; Mamastrovirus; Mamastrovirus 1

gca	genome.size	proteome.size	phage.name	strain	Taxonomy
GCA_000861065.1	8363	6	Garlic latent virus	NA	Viruses; ssRNA viruses; ssRNA positive-strand viruses, no DNA stage; Tymovirales; Betaflexiviridae; Quinvirinae; Carlavirus; Shallot latent virus
GCA_000904575.1	53028	95	Mycobacterium phage First	NA	Viruses; dsDNA viruses, no RNA stage; Caudovirales; Siphoviridae; unclassified Siphoviridae
GCA_000902255.1	27431	37	Sulfolobales Mexican rudivirus 1	NA	Viruses; dsDNA viruses, no RNA stage; Ligamenvirales; Rudiviridae; Rudivirus; unclassified Rudivirus
GCA_000860765.1	11219	2	Barley yellow mosaic virus	NA	Viruses; ssRNA viruses; ssRNA positive-strand viruses, no DNA stage; Potyviridae; Bymovirus
GCA_000862285.1	10786	2	Barley mild mosaic virus	NA	Viruses; ssRNA viruses; ssRNA positive-strand viruses, no DNA stage; Potyviridae; Bymovirus
GCA_000897995.1	5285	7	Blechum interveinal chlorosis virus	NA	Viruses; ssDNA viruses; Geminiviridae; Begomovirus
GCA_002818885.1	199721	194	Heliothis virescens ascovirus 3g	NA	Viruses; Varidnaviria; Bamfordvirae; Nucleocytoviricota; Megaviricetes; Pimascovirales; Ascoviridae; Ascovirus; Heliothis virescens ascovirus 3a
GCA_001505475.1	1317	1	Malvastrum yellow vein Chitwan betasatellite	NA	Viruses; Tolecusatellitidae; Betasatellite; unclassified Betasatellite
GCA_002830865.1	4275	5	Lucerne transient streak virus	New Zealand isolate	Viruses; ssRNA viruses; ssRNA positive-strand viruses, no DNA stage; Sobemovirus
GCA_002004135.1	4297	4	Solanum nodiflorum mottle virus	NA	Viruses; ssRNA viruses; ssRNA positive-strand viruses, no DNA stage; Sobemovirus
GCA_000897955.1	6518	3	Astrovirus VA4	NA	Viruses; ssRNA viruses; ssRNA positive-strand viruses, no DNA stage; Astroviridae; unclassified Astroviridae
GCA_000899535.1	6124	3	Astrovirus MLB3	NA	Viruses; ssRNA viruses; ssRNA positive-strand viruses, no DNA stage; Astroviridae; unclassified Astroviridae
GCA_000899555.1	6956	2	Diuris virus A	NA	Viruses; ssRNA viruses; ssRNA positive-strand viruses, no DNA stage; Tymovirales; Betaflexiviridae; Trivirinae; Divavirus
GCA_000901495.1	7001	2	Diuris virus B	NA	Viruses; ssRNA viruses; ssRNA positive-strand viruses, no DNA stage; Tymovirales; Betaflexiviridae; Trivirinae; Divavirus
GCA_000862585.1	4258	4	Subterranean clover mottle virus	p23	Viruses; ssRNA viruses; ssRNA positive-strand viruses, no DNA stage; Sobemovirus
GCA_000889275.1	4247	4	Velvet tobacco mottle virus	K1	Viruses; ssRNA viruses; ssRNA positive-strand viruses, no DNA stage; Sobemovirus
GCA_002966435.1	1016844	970	Moumouvirus goulette	NA	Viruses; Varidnaviria; Bamfordvirae; Nucleocytoviricota; Megaviricetes; Imitervirales; Mimiviridae; unclassified Mimiviridae; Moumouvirus
GCA_000837045.1	1291	6	Coconut foliar decay virus	NA	Viruses; Monodnaviria; Shotokuvirae; Cressdnaviricota; Arfiviricetes; Mulpavirales; Nanoviridae
GCA_003033635.1	1677	2	Hepatitis delta virus	genotype III	Viruses; Deltavirus
GCA_000860565.1	5270	5	African green monkey polyomavirus	NA	Viruses; dsDNA viruses, no RNA stage; Polyomaviridae; unclassified Polyomaviridae
GCA_008777345.1	7318	1	Human enterovirus 113	NA	Viruses; Riboviria; Orthornavirae; Pisuviricota; Pisoniviricetes; Picornavirales; Picornaviridae; Enterovirus; unclassified Enterovirus
GCA_008776735.1	7420	1	Enterovirus B106	NA	Viruses; Riboviria; Orthornavirae; Pisuviricota; Pisoniviricetes; Picornavirales; Picornaviridae; Enterovirus; Enterovirus B
GCA_002006915.1	7329	7	Human papillomavirus type 156	NA	Viruses; Monodnaviria; Shotokuvirae; Cossaviricota; Papovaviricetes; Zurhausenvirales; Papillomaviridae; Firstpapillomavirinae; Gammapapillomavirus; Gammapapillomavirus 18
GCA_000902995.1	1350	1	Okra enation leaf curl alphasatellite	NA	Viruses; Satellites; Satellite Nucleic Acids; Single stranded DNA satellites; Alphasatellites; Begomovirus-associated alphasatellites; unclassified Begomovirus-associated alphasatellites
GCA_003986445.1	2460	5	Pig stool associated circular ssDNA virus	NA	Viruses; Monodnaviria; Shotokuvirae; Cressdnaviricota; Arfiviricetes; Cremevirales; Smacoviridae; Drosmacovirus; unclassified Drosmacovirus
GCA_000903435.1	2650	4	Dragonflyassociated mastrevirus	NA	Viruses; ssDNA viruses; Geminiviridae; Mastrevirus; unclassified Mastrevirus
GCA_000902555.1	12154	1	Grapevine endophyte endornavirus	NA	Viruses; dsRNA viruses; Endornaviridae; Endornavirus; unclassified Endornavirus
GCA_002868435.1	4516	2	Heterobasidion partitivirus 8	HetPV8ir1	Viruses; Riboviria; Orthornavirae; Pisuviricota; Duplopiviricetes; Durnavirales; Partitiviridae; Betapartitivirus
GCA_000901935.1	1350	1	Sunflower leaf curl Karnataka alphasatellite	NA	Viruses; Satellites; Satellite Nucleic Acids; Single stranded DNA satellites; Alphasatellites; Begomovirus-associated alphasatellites; unclassified Begomovirus-associated alphasatellites
GCA_000913975.1	1118	1	Olive viral satellite RNA	NA	Viruses; Satellites; Satellite Nucleic Acids; RNA satellites
GCA_000861805.1	5908	4	Junonia coenia densovirus	NA	Viruses; ssDNA viruses; Parvoviridae; Densovirinae; Ambidensovirus; Lepidopteran ambidensovirus 1
GCA_000852805.1	5283	2	Leishmania RNA virus 1 4	NA	Viruses; Riboviria; Orthornavirae; Duplornaviricota; Chrymotiviricetes; Ghabrivirales; Totiviridae; Leishmaniavirus; Leishmania RNA virus 1
GCA_000855505.1	10787	1	Omsk hemorrhagic fever virus	Bogoluvovska	Viruses; ssRNA viruses; ssRNA positive-strand viruses, no DNA stage; Flaviviridae; Flavivirus; tick-borne encephalitis virus group
GCA_004130905.1	10804	1	Nanay virus	PRD316PER09	Viruses; Riboviria; Orthornavirae; Kitrinoviricota; Flasuviricetes; Amarillovirales; Flaviviridae; Flavivirus; unclassified Flavivirus

gca	genome.size	proteome.size	phage.name	strain	Taxonomy
GCA_000903455.1	6460	3	Porcine astrovirus 3	NA	Viruses; ssRNA viruses; ssRNA positive-strand viruses, no DNA stage; Astroviridae; Mamastrovirus; unclassified Mamastrovirus
GCA_000855645.1	11950	3	Seoul virus 8039	NA	Viruses; Riboviria; Orthornavirae; Negarnaviricota; Polyploviricotina; Ellioviricetes; Bunyavirales; Hantaviridae; Mammantavirinae; Orthohantavirus; Seoul orthohantavirus
GCA_000900915.1	1335	1	Tomato yellow leaf curl China betasatellite	NA	Viruses; Tolecusatellitidae; Betasatellite
GCA_000862445.1	4148	4	Sesbania mosaic virus	NA	Viruses; ssRNA viruses; ssRNA positive-strand viruses, no DNA stage; Sobemovirus
GCA_000904335.1	11487	5	Perch rhabdovirus	NA	Viruses; ssRNA viruses; ssRNA negative-strand viruses; Mononegavirales; Rhabdoviridae; Perhabdovirus
GCA_000861145.1	5078	4	Bombyx mori densovirus 5	NA	Viruses; ssDNA viruses; Parvoviridae; Densovirinae; Iteradensovirus; Lepidopteran iteradensovirus 1; Bombyx mori densovirus
GCA_003124705.1	6194	6	Banana bunchy top virus	NA	Viruses; ssDNA viruses; Nanoviridae; Babuvirus
GCA_002617245.1	70912	80	Escherichia phage EC1UPM	NA	Viruses; Duplodnaviria; Heunggongvirae; Uroviricota; Caudoviricetes; Caudovirales; Podoviridae; Gamaleyavirus; Escherichia virus EC1UPM
GCA_018855775.1	14852	32	Bacillus phage Sato	NA	Viruses; Varidnaviria; Bamfordvirae; Preplasmiviricota; Tectiliviricetes; Kalamavirales; Tectiviridae; unclassified Tectiviridae
GCA_018855805.1	14444	30	Bacillus phage Sole	NA	Viruses; Varidnaviria; Bamfordvirae; Preplasmiviricota; Tectiliviricetes; Kalamavirales; Tectiviridae; unclassified Tectiviridae
GCA_004035695.1	2036	6	Columbid circovirus	NA	Viruses; ssDNA viruses; Circoviridae; Circovirus; unclassified Circovirus
GCA_000902015.1	1378	1	Ageratum enation alphasatellite	NA	Viruses; Satellites; Satellite Nucleic Acids; Single stranded DNA satellites; Alphasatellites; Begomovirus-associated alphasatellites; unclassified Begomovirus-associated alphasatellites
GCA_000925575.1	15624	8	Achimota virus 1	NA	Viruses; ssRNA viruses; ssRNA negative-strand viruses; Mononegavirales; Paramyxoviridae; Rubulavirus; unclassified Rubulavirus
GCA_000924735.1	15504	8	Achimota virus 2	NA	Viruses; ssRNA viruses; ssRNA negative-strand viruses; Mononegavirales; Paramyxoviridae; Rubulavirus; unclassified Rubulavirus
GCA_000893515.1	14278	2	Cherry leaf roll virus	NA	Viruses; ssRNA viruses; ssRNA positive-strand viruses, no DNA stage; Picornavirales; Secoviridae; Comovirinae; Nepovirus; Subgroup C
GCA_000902655.2	202949	137	Murid betaherpesvirus 8	NA	Viruses; dsDNA viruses, no RNA stage; Herpesvirales; Herpesviridae; Betaherpesvirinae; Muromegalovirus
GCA_000864805.1	2319	3	Chicken anemia virus	NA	Viruses; ssDNA viruses; Circoviridae; Gyrovirus
GCA_000924875.1	140903	217	Staphylococcus phage Team1	NA	Viruses; dsDNA viruses, no RNA stage; Caudovirales; Myoviridae; Spounavirinae; Kayvirus; Staphylococcus virus G1
GCA_000905035.1	157834	198	Serratia phage phiMAM1	NA	Viruses; dsDNA viruses, no RNA stage; Caudovirales; Myoviridae; unclassified Myoviridae
GCA_000911255.1	42957	50	Pseudomonas phage MPK6	NA	Viruses; dsDNA viruses, no RNA stage; Caudovirales; Podoviridae; Autographivirinae; Phikmvvirus; unclassified phiKMV-like phages
GCA_000923735.1	26752	39	Leuconostoc phage phiLN03	NA	Viruses; dsDNA viruses, no RNA stage; Caudovirales; Siphoviridae; unclassified Siphoviridae
GCA_000906195.1	25865	39	Leuconostoc phage phiLN04	NA	Viruses; dsDNA viruses, no RNA stage; Caudovirales; Siphoviridae; unclassified Siphoviridae
GCA_000921195.1	28193	41	Leuconostoc phage phiLN12	NA	Viruses; dsDNA viruses, no RNA stage; Caudovirales; Siphoviridae; unclassified Siphoviridae
GCA_000922055.1	28427	41	Leuconostoc phage phiLN25	NA	Viruses; dsDNA viruses, no RNA stage; Caudovirales; Siphoviridae; unclassified Siphoviridae
GCA_000921175.1	28022	41	Leuconostoc phage phiLN34	NA	Viruses; dsDNA viruses, no RNA stage; Caudovirales; Siphoviridae; unclassified Siphoviridae
GCA_000923055.1	25740	39	Leuconostoc phage phiLN6B	NA	Viruses; dsDNA viruses, no RNA stage; Caudovirales; Siphoviridae; unclassified Siphoviridae
GCA_000922015.1	28015	41	Leuconostoc phage phiLNTR3	NA	Viruses; dsDNA viruses, no RNA stage; Caudovirales; Siphoviridae; unclassified Siphoviridae
GCA_000905595.1	26769	38	Leuconostoc phage P793	NA	Viruses; dsDNA viruses, no RNA stage; Caudovirales; Siphoviridae; unclassified Siphoviridae
GCA_000922035.1	28336	42	Leuconostoc phage phiLNTR2	NA	Viruses; dsDNA viruses, no RNA stage; Caudovirales; Siphoviridae; unclassified Siphoviridae
GCA_015147065.1	40218	58	Escherichia phage CLB_P1	NA	Viruses; Duplodnaviria; Heunggongvirae; Uroviricota; Caudoviricetes; Caudovirales; Autographiviridae; Studiervirinae; Kayfunavirus; unclassified Kayfunavirus
GCA_000905695.1	68527	103	Halovirus HSTV2	NA	Viruses; dsDNA viruses, no RNA stage; unclassified dsDNA viruses; unclassified archaeal dsDNA viruses; Haloviruses
GCA_000905075.1	102319	173	Halovirus HVTV1	NA	Viruses; dsDNA viruses, no RNA stage; unclassified dsDNA viruses; unclassified archaeal dsDNA viruses; Haloviruses
GCA_000907675.1	32189	53	Halovirus HSTV1	NA	Viruses; dsDNA viruses, no RNA stage; unclassified dsDNA viruses; unclassified archaeal dsDNA viruses; Haloviruses
GCA_000907755.1	45942	73	Mycobacterium phage BTCU1	NA	Viruses; dsDNA viruses, no RNA stage; Caudovirales; Siphoviridae; unclassified Siphoviridae

gca	genome.size	proteome.size	phage.name	strain	Taxonomy
GCA_000910635.1	32337	58	Lactococcus phage 340	NA	Viruses; dsDNA viruses, no RNA stage; Caudovirales; Siphoviridae; unclassified Siphoviridae
GCA_000909735.1	31090	59	Lactococcus phage jm2	NA	Viruses; dsDNA viruses, no RNA stage; Caudovirales; Siphoviridae; unclassified Siphoviridae
GCA_000911695.1	28674	52	Lactococcus phage jm3	NA	Viruses; dsDNA viruses, no RNA stage; Caudovirales; Siphoviridae; unclassified Siphoviridae
GCA_000911715.1	29631	49	Lactococcus phage P680	NA	Viruses; dsDNA viruses, no RNA stage; Caudovirales; Siphoviridae; unclassified Siphoviridae
GCA_000908115.1	32382	57	Lactococcus phage phi7	NA	Viruses; dsDNA viruses, no RNA stage; Caudovirales; Siphoviridae; unclassified Siphoviridae
GCA_000914495.1	246964	327	Vibrio phage VH7D	NA	Viruses; dsDNA viruses, no RNA stage; Caudovirales; Myoviridae; Tevenvirinae; Schizot4virus; unclassified Schizot4virus
GCA_000851965.1	4029	6	Cowpea mottle virus	NA	Viruses; ssRNA viruses; ssRNA positive-strand viruses, no DNA stage; Tombusviridae; Gammacarmovirus
GCA_002816195.1	30111	10	Betacoronavirus England 1	NA	Viruses; Riboviria; Orthornavirae; Pisuviricota; Pisoniviricetes; Nidovirales; Cornidovirineae; Coronaviridae; Orthocoronavirinae; Betacoronavirus; Merbecovirus; Middle East respiratory syndrome-related coronavirus
GCA_000847905.1	3027	7	Duck hepatitis B virus	NA	Viruses; Riboviria; Pararnavirae; Artverviricota; Revtraviricetes; Blubervirales; Hepadnaviridae; Avihepadnavirus
GCA_000841905.1	209771	173	Ectromelia virus	Moscow	Viruses; dsDNA viruses, no RNA stage; Poxviridae; Chordopoxvirinae; Orthopoxvirus
GCA_000905655.1	42746	66	Edwardsiella phage MSW3	NA	Viruses; dsDNA viruses, no RNA stage; Caudovirales; Myoviridae; unclassified Myoviridae
GCA_000903995.1	41796	49	Cronobacter phage vB_CskP_GAP227	NA	Viruses; dsDNA viruses, no RNA stage; Caudovirales; Podoviridae; Autographivirinae; Phikmvvirus; unclassified phiKMV-like phages
GCA_002823305.2	5245	7	Sida ciliaris golden mosaic virus	VenezuelaLaraM32009	Viruses; Monodnaviria; Shotokuvirae; Cressdnaviricota; Repensiviricetes; Geplafuvirales; Geminiviridae; Begomovirus
GCA_001661775.1	28434	9	Ferret coronavirus	NA	Viruses; ssRNA viruses; ssRNA positive-strand viruses, no DNA stage; Nidovirales; Coronaviridae; Coronavirinae; Alphacoronavirus; unclassified Alphacoronavirus
GCA_018595535.1	4190	2	Rhizoctonia solani dsRNA virus 1	NA	Viruses; Riboviria; Orthornavirae; Pisuviricota; Duplopiviricetes; Durnavirales; Curvulaviridae; Orthocurvulavirus; Rhizoctonia solani orthocurvulavirus 1
GCA_000843985.2	184439	78	Equid gammaherpesvirus 2	8667	Viruses; dsDNA viruses, no RNA stage; Herpesvirales; Herpesviridae; Gammaherpesvirinae; Percavirus
GCA_000954755.2	16930	5	Mulberry vein banding virus	NA	Viruses; ssRNA viruses; ssRNA negative-strand viruses; Bunyaviridae; unclassified Bunyaviridae
GCA_000926355.1	5124	8	Tomato mottle wrinkle virus	NA	Viruses; ssDNA viruses; Geminiviridae; Begomovirus; unclassified Begomovirus
GCA_000905775.1	2676	5	Sida yellow net virus	NA	Viruses; ssDNA viruses; Geminiviridae; Begomovirus
GCA_000905175.1	2664	5	Sida yellow blotch virus	NA	Viruses; ssDNA viruses; Geminiviridae; Begomovirus
GCA_000906655.1	2690	5	Sida yellow mosaic Alagoas virus	NA	Viruses; ssDNA viruses; Geminiviridae; Begomovirus
GCA_000904175.1	2649	5	Sida mottle Alagoas virus	NA	Viruses; ssDNA viruses; Geminiviridae; Begomovirus
GCA_000911375.1	37375	49	Streptococcus phage phiBHN167	NA	Viruses; unclassified phages
GCA_003972785.1	12804	3	Groundnut ringspot virus	NA	Viruses; Riboviria; Orthornavirae; Negarnaviricota; Polyploviricotina; Ellioviricetes; Bunyavirales; Tospoviridae; Orthotospovirus; Groundnut ringspot tospovirus
GCA_002816495.1	10967	1	Nilaparvata lugens honeydew virus 1	NA	Viruses; Riboviria; Orthornavirae; Pisuviricota; Pisoniviricetes; Picornavirales; Iflaviridae; Iflavirus
GCA_006457925.1	192353	229	Vaccinia virus Ankara	chorioallantois vaccinia virus Ankara CVA	Viruses; Varidnaviria; Bamfordvirae; Nucleocytoviricota; Pokkesviricetes; Chitovirales; Poxviridae; Chordopoxvirinae; Orthopoxvirus; Vaccinia virus
GCA_004787695.1	6532	1	Tigray virus	NA	Viruses; Riboviria; Orthornavirae; Negarnaviricota; Polyploviricotina; Ellioviricetes; Bunyavirales; Hantaviridae; Mammantavirinae; Orthohantavirus; Tigray orthohantavirus
GCA_006449695.1	190519	185	Heliothis virescens ascovirus 3h	NA	Viruses; Varidnaviria; Bamfordvirae; Nucleocytoviricota; Megaviricetes; Pimascovirales; Ascoviridae; Ascovirus; Heliothis virescens ascovirus 3a
GCA_002817335.1	8749	1	Rosavirus M7	RosaM7	Viruses; Riboviria; Orthornavirae; Pisuviricota; Pisoniviricetes; Picornavirales; Picornaviridae; Rosavirus; Rosavirus A; Rosavirus A1
GCA_000904035.1	1021348	894	Acanthamoeba polyphaga moumouvirus	M10A	Viruses; dsDNA viruses, no RNA stage; Mimiviridae; unclassified Mimiviridae; Moumouvirus
GCA_003308835.1	6062	10	Xanthomonas phage phiLF	ATCC 33913	Viruses; Monodnaviria; Loebvirae; Hofneiviricota; Faserviricetes; Tubulavirales; Inoviridae; unclassified Inoviridae
GCA_003102595.1	8396	9	Human immunodeficiency virus	URF5	Viruses; Riboviria; Pararnavirae; Artverviricota; Revtraviricetes; Ortervirales; Retroviridae; Orthoretrovirinae; Lentivirus; Primate lentivirus group; unclassified Primate lentivirus group
GCA_003179475.1	7776	6	Canis familiaris papillomavirus 15	NA	Viruses; Monodnaviria; Shotokuvirae; Cossaviricota; Papovaviricetes; Zurhausenvirales; Papillomaviridae; Firstpapillomavirinae; Chipapillomavirus; Chipapillomavirus 3
GCA_002145965.1	12403	9	Gray Lodge virus	BFN3187	Viruses; Riboviria; Orthornavirae; Negarnaviricota; Haploviricotina; Monjiviricetes; Mononegavirales; Rhabdoviridae; Hapavirus; Gray Lodge hapavirus

gca	genome.size	proteome.size	phage.name	strain	Taxonomy
GCA_002145765.1	13155	9	Joinjakaka virus	AusMK7937	Viruses; Riboviria; Orthornavirae; Negarnaviricota; Haploviricotina; Monjiviricetes; Mononegavirales; Rhabdoviridae; Hapavirus; Joinjakaka hapavirus
GCA_013087165.1	11211	7	Kwatta virus	A57	Viruses; Riboviria; Orthornavirae; Negarnaviricota; Haploviricotina; Monjiviricetes; Mononegavirales; Rhabdoviridae; Sunrhavirus; Kwatta sunrhavirus
GCA_002145565.1	15721	14	La Joya virus	J134	Viruses; Riboviria; Orthornavirae; Negarnaviricota; Haploviricotina; Monjiviricetes; Mononegavirales; Rhabdoviridae; Hapavirus; La Joya hapavirus
GCA_002146145.1	13695	11	Landjia virus	DakAnB769d	Viruses; Riboviria; Orthornavirae; Negarnaviricota; Haploviricotina; Monjiviricetes; Mononegavirales; Rhabdoviridae; Hapavirus; Landjia hapavirus
GCA_002145945.1	13784	11	Manitoba virus	Mn93677	Viruses; Riboviria; Orthornavirae; Negarnaviricota; Haploviricotina; Monjiviricetes; Mononegavirales; Rhabdoviridae; Hapavirus; Manitoba hapavirus
GCA_013087175.1	11156	5	New Minto virus	579	Viruses; Riboviria; Orthornavirae; Negarnaviricota; Haploviricotina; Monjiviricetes; Mononegavirales; Rhabdoviridae; Sawgrhavirus; Minto sawgrhavirus
GCA_002145845.1	11355	5	Oita virus	2961972	Viruses; Riboviria; Orthornavirae; Negarnaviricota; Haploviricotina; Monjiviricetes; Mononegavirales; Rhabdoviridae; Ledantevirus; Oita ledantevirus
GCA_018583895.1	14932	10	Puchong virus	NA	Viruses; Riboviria; Orthornavirae; Negarnaviricota; Haploviricotina; Monjiviricetes; Mononegavirales; Rhabdoviridae; Alpharhabdovirinae; Ephemerovirus; Puchong ephemerovirus
GCA_002145605.1	11422	7	Sena Madureira virus	BeAn303197	Viruses; Riboviria; Orthornavirae; Negarnaviricota; Haploviricotina; Monjiviricetes; Mononegavirales; Rhabdoviridae; Sripuvirus; Sena Madureira sripuvirus
GCA_002145685.1	13141	8	Sweetwater Branch virus	UF11	Viruses; Riboviria; Orthornavirae; Negarnaviricota; Haploviricotina; Monjiviricetes; Mononegavirales; Rhabdoviridae; Tibrovirus; Sweetwater Branch tibrovirus
GCA_000926455.1	12240	7	Xiburema virus	NA	Viruses; ssRNA viruses; ssRNA negative-strand viruses; Mononegavirales; Rhabdoviridae; unclassified Rhabdoviridae
GCA_001431995.1	14478	11	Yata virus	NA	Viruses; ssRNA viruses; ssRNA negative-strand viruses; Mononegavirales; Rhabdoviridae; Ephemerovirus; unclassified Ephemerovirus
GCA_013087185.1	11906	7	Aruac virus	TRVL9223	Viruses; Riboviria; Orthornavirae; Negarnaviricota; Haploviricotina; Monjiviricetes; Mononegavirales; Rhabdoviridae; unclassified Rhabdoviridae
GCA_013087195.1	11169	7	Connecticut virus	Ar115278	Viruses; Riboviria; Orthornavirae; Negarnaviricota; Haploviricotina; Monjiviricetes; Mononegavirales; Rhabdoviridae; unclassified Rhabdoviridae
GCA_001020035.1	1346	1	Kenaf leaf curl betasatellite	NA	Viruses; Tolecusatellitidae; Betasatellite
GCA_002603025.1	43017	65	Enterobacteria phage phiJLA23	NA	Viruses; Duplodnaviria; Heunggongvirae; Uroviricota; Caudoviricetes; Caudovirales; Drexlerviridae; Rogunavirinae; Rogunavirus; Escherichia virus phiJLA23
GCA_002603045.1	65270	71	Pseudomonas virus H66	NA	Viruses; Duplodnaviria; Heunggongvirae; Uroviricota; Caudoviricetes; Caudovirales; Podoviridae; Hollowayvirus
GCA_002831005.1	43895	70	Pseudomonas phage PaP4	NA	Viruses; Duplodnaviria; Heunggongvirae; Uroviricota; Caudoviricetes; Caudovirales; Podoviridae; Bruynoghevirus; Pseudomonas virus PaP4
GCA_000929515.1	46547	50	Synechococcus phage SCBP1	NA	Viruses; dsDNA viruses, no RNA stage; Caudovirales; Podoviridae; Autographivirinae; unclassified Autographivirinae
GCA_000907895.1	43458	66	Staphylococcus phage JS01	NA	Viruses; dsDNA viruses, no RNA stage; Caudovirales; Siphoviridae; unclassified Siphoviridae
GCA_002755475.1	5475	11	Enterobacteria phage ID204 MoscowID	NA	Viruses; Monodnaviria; Sangervirae; Phixviricota; Malgrandaviricetes; Petitvirales; Microviridae; Bullavirinae; Gequatrovirus; Escherichia virus Talmos
GCA_001041035.1	348113	551	Escherichia phage PBECO4	NA	Viruses; Duplodnaviria; Heunggongvirae; Uroviricota; Caudoviricetes; Caudovirales; Myoviridae; Asteriusvirus; Escherichia virus PBECO4
GCA_000904275.1	49259	79	Bacillus virus Andromeda	NA	Viruses; dsDNA viruses, no RNA stage; Caudovirales; Siphoviridae; Andromedavirus
GCA_000905895.1	49425	77	Bacillus virus Curly	NA	Viruses; dsDNA viruses, no RNA stage; Caudovirales; Siphoviridae; Andromedavirus
GCA_000905295.1	49458	75	Bacillus virus Eoghan	NA	Viruses; dsDNA viruses, no RNA stage; Caudovirales; Siphoviridae; Andromedavirus
GCA_000906775.1	50161	77	Bacillus virus Finn	NA	Viruses; dsDNA viruses, no RNA stage; Caudovirales; Siphoviridae; Andromedavirus
GCA_002603065.1	49362	79	Bacillus phage Gemini	NA	Viruses; Duplodnaviria; Heunggongvirae; Uroviricota; Caudoviricetes; Caudovirales; Siphoviridae; Andromedavirus; Bacillus virus Andromeda
GCA_002603085.1	49492	75	Bacillus virus Taylor	NA	Viruses; Duplodnaviria; Heunggongvirae; Uroviricota; Caudoviricetes; Caudovirales; Siphoviridae; Andromedavirus
GCA_000909275.1	103257	160	Halovirus HCTV1	NA	Viruses; dsDNA viruses, no RNA stage; unclassified dsDNA viruses; unclassified archaeal dsDNA viruses; Haloviruses
GCA_000907655.1	54291	86	Halovirus HCTV2	NA	Viruses; dsDNA viruses, no RNA stage; unclassified dsDNA viruses; unclassified archaeal dsDNA viruses; Haloviruses
GCA_000910135.1	102105	166	Halovirus HCTV5	NA	Viruses; dsDNA viruses, no RNA stage; unclassified dsDNA viruses; unclassified archaeal dsDNA viruses; Haloviruses
GCA_000908655.1	143855	281	Halovirus HGTV1	NA	Viruses; dsDNA viruses, no RNA stage; unclassified dsDNA viruses; unclassified archaeal dsDNA viruses; Haloviruses
GCA_000909255.1	49107	74	Halovirus HHTV1	NA	Viruses; dsDNA viruses, no RNA stage; unclassified dsDNA viruses; unclassified archaeal dsDNA viruses; Haloviruses
GCA_000907635.1	52643	88	Halovirus HHTV2	NA	Viruses; dsDNA viruses, no RNA stage; unclassified dsDNA viruses; unclassified archaeal dsDNA viruses; Haloviruses

gca	genome.size	proteome.size	phage.name	strain	Taxonomy
GCA_000910115.1	35722	73	Halovirus HRTV4	NA	Viruses; dsDNA viruses, no RNA stage; unclassified dsDNA viruses; unclassified archaeal dsDNA viruses; Haloviruses
GCA_000908635.1	76134	118	Halovirus HRTV5	NA	Viruses; dsDNA viruses, no RNA stage; unclassified dsDNA viruses; unclassified archaeal dsDNA viruses; Haloviruses
GCA_000909235.1	69048	105	Halovirus HRTV7	NA	Viruses; dsDNA viruses, no RNA stage; unclassified dsDNA viruses; unclassified archaeal dsDNA viruses; Haloviruses
GCA_000907615.1	74519	124	Halovirus HRTV8	NA	Viruses; dsDNA viruses, no RNA stage; unclassified dsDNA viruses; unclassified archaeal dsDNA viruses; Haloviruses
GCA_000852185.1	9650	1	Infectious flacherie virus	NA	Viruses; ssRNA viruses; ssRNA positive-strand viruses, no DNA stage; Picornavirales; Iflaviridae; Iflavirus
GCA_000837165.1	8784	10	Vibrio phage VfO3K6	NA	Viruses; ssDNA viruses; Inoviridae; Inovirus; unclassified Inovirus
GCA_000839665.1	6891	8	Vibrio phage VfO4K68	NA	Viruses; ssDNA viruses; Inoviridae; Inovirus; unclassified Inovirus
GCA_000845325.1	7965	7	Vibrio virus Vf33	NA	Viruses; Monodnaviria; Loebvirae; Hofneiviricota; Faserviricetes; Tubulavirales; Inoviridae; Villovirus
GCA_002603105.1	7965	7	Vibrio phage Vf12	NA	Viruses; Monodnaviria; Loebvirae; Hofneiviricota; Faserviricetes; Tubulavirales; Inoviridae; Villovirus; Vibrio virus Vf33
GCA_006465545.1	139793	98	Grouper iridovirus	NA	Viruses; Varidnaviria; Bamfordvirae; Nucleocytoviricota; Megaviricetes; Pimascovirales; Iridoviridae; Alphairidovirinae; Ranavirus; Singapore grouper iridovirus
GCA_000904955.1	4899	5	Chaerephon polyomavirus 1	NA	Viruses; dsDNA viruses, no RNA stage; Polyomaviridae; unclassified Polyomaviridae
GCA_000904055.1	4776	6	STL polyomavirus	MA138	Viruses; dsDNA viruses, no RNA stage; Polyomaviridae; unclassified Polyomaviridae
GCA_000910095.1	45572	62	Staphylococcus phage StauST3982	NA	Viruses; dsDNA viruses, no RNA stage; Caudovirales; Siphoviridae; unclassified Siphoviridae
GCA_000862945.1	9871	1	Parsnip yellow fleck virus	NA	Viruses; ssRNA viruses; ssRNA positive-strand viruses, no DNA stage; Picornavirales; Secoviridae; Sequivirus
GCA_000915475.1	159590	271	Bacillus phage BPS10C	NA	Viruses; dsDNA viruses, no RNA stage; Caudovirales; Myoviridae; unclassified Myoviridae
GCA_002603125.1	35598	56	Streptococcus phage phiSS12	NA	Viruses; Duplodnaviria; Heunggongvirae; Uroviricota; Caudoviricetes; Caudovirales; Siphoviridae; unclassified Siphoviridae
GCA_002603145.1	38940	56	Streptococcus phage phiST1	NA	Viruses; Duplodnaviria; Heunggongvirae; Uroviricota; Caudoviricetes; Caudovirales; Siphoviridae; unclassified Siphoviridae
GCA_002595125.1	38253	58	Brucella phage Wb	NA	Viruses; Duplodnaviria; Heunggongvirae; Uroviricota; Caudoviricetes; Caudovirales; Podoviridae; Perisivirus; Brucella virus Tb
GCA_003146825.1	38208	59	Brucella phage RC	NA	Viruses; Duplodnaviria; Heunggongvirae; Uroviricota; Caudoviricetes; Caudovirales; Podoviridae; Perisivirus; Brucella virus Pr
GCA_002633075.1	38253	58	Brucella phage Bk2	NA	Viruses; Duplodnaviria; Heunggongvirae; Uroviricota; Caudoviricetes; Caudovirales; Podoviridae; Perisivirus; unclassified Perisivirus
GCA_002596205.1	174351	216	Cyanophage SRIM12	NA	Viruses; Duplodnaviria; Heunggongvirae; Uroviricota; Caudoviricetes; Caudovirales; Myoviridae; Brizovirus; unclassified Brizovirus
GCA_002599065.1	180780	220	Cyanophage SRIM14	NA	Viruses; Duplodnaviria; Heunggongvirae; Uroviricota; Caudoviricetes; Caudovirales; Myoviridae; Ahtivirus; unclassified Ahtivirus
GCA_001754165.1	194437	230	Cyanophage SRIM32	NA	Viruses; dsDNA viruses, no RNA stage; Caudovirales; Myoviridae; unclassified Myoviridae
GCA_002599205.1	197868	236	Cyanophage SRIM44	NA	Viruses; Duplodnaviria; Heunggongvirae; Uroviricota; Caudoviricetes; Caudovirales; Myoviridae; Vellamovirus; Synechococcus virus SRIM44
GCA_002633125.1	41142	58	Brucella phage Fi	NA	Viruses; Duplodnaviria; Heunggongvirae; Uroviricota; Caudoviricetes; Caudovirales; Podoviridae; Perisivirus; unclassified Perisivirus
GCA_000915175.1	43564	66	Pseudomonas phage PPpW3	NA	Viruses; dsDNA viruses, no RNA stage; Caudovirales; Myoviridae; unclassified Myoviridae
GCA_000916695.1	41386	50	Pseudomonas phage PPpW4	NA	Viruses; dsDNA viruses, no RNA stage; Caudovirales; Podoviridae; unclassified Podoviridae
GCA_001461325.1	2617	1	Sclerotinia sclerotiorum mitovirus 3	NA	Viruses; ssRNA viruses; ssRNA positive-strand viruses, no DNA stage; Narnaviridae; Mitovirus; unclassified Mitovirus
GCA_000854425.1	11849	2	Raspberry ringspot virus	cherry	Viruses; ssRNA viruses; ssRNA positive-strand viruses, no DNA stage; Picornavirales; Secoviridae; Comovirinae; Nepovirus; Subgroup A
GCA_001008475.1	7521	2	Sapovirus HuNagoyaNGY12012JPN	HuSaVNagoyaNGY12012JPN	Viruses; Riboviria; Orthornavirae; Pisuviricota; Pisoniviricetes; Picornavirales; Caliciviridae; Sapovirus; Sapporo virus; Sapovirus isolates
GCA_000856445.1	15191	10	Respiratory syncytial virus	S2 ts1C	Viruses; ssRNA viruses; ssRNA negative-strand viruses; Mononegavirales; Pneumoviridae; Pneumovirus; unclassified Pneumovirus
GCA_004787815.1	9656	1	Rodent hepacivirus	NA	Viruses; ssRNA viruses; ssRNA positive-strand viruses, no DNA stage; Flaviviridae; Hepacivirus; unclassified Hepacivirus
GCA_013090075.1	6624	1	Bat picornavirus	NA	Viruses; Riboviria; Orthornavirae; Pisuviricota; Pisoniviricetes; Picornavirales; Picornaviridae; Shanbavirus; Shanbavirus A
GCA_000919075.1	2308	2	Cyanoramphus nest associated circular X DNA virus	NA	Viruses; unclassified viruses; unclassified DNA viruses; unclassified ssDNA viruses

gca	genome.size	proteome.size	phage.name	strain	Taxonomy
GCA_000918055.1	2087	2	Cyanoramphus nest associated circular K DNA virus	NA	Viruses; unclassified viruses; unclassified DNA viruses; unclassified ssDNA viruses
GCA_000908235.1	28072	49	Haloarcula hispanica virus PH1	NA	Viruses; Virus families not assigned to an order; Sphaerolipoviridae; Alphasphaerolipovirus
GCA_000908615.1	35430	49	Burkholderia phage ST79	NA	Viruses; dsDNA viruses, no RNA stage; Caudovirales; Myoviridae; unclassified Myoviridae
GCA_000909435.1	42724	69	Serratia phage Eta	NA	Viruses; dsDNA viruses, no RNA stage; Caudovirales; Siphoviridae; unclassified Siphoviridae
GCA_000908755.1	69278	79	Vibrio phage JA1	NA	Viruses; dsDNA viruses, no RNA stage; Caudovirales; Podoviridae; N4virus; unclassified N4likevirus
GCA_002603165.1	68964	79	Vibrio phage VCO139	NA	Viruses; Duplodnaviria; Heunggongvirae; Uroviricota; Caudoviricetes; Caudovirales; Podoviridae; unclassified Podoviridae
GCA_000905255.1	147284	198	Pelagibacter phage HTVC008M	NA	Viruses; dsDNA viruses, no RNA stage; Caudovirales; Myoviridae; unclassified Myoviridae
GCA_000906735.1	34892	64	Pelagibacter phage HTVC010P	NA	Viruses; dsDNA viruses, no RNA stage; Caudovirales; Podoviridae; unclassified Podoviridae
GCA_000904255.1	39921	45	Pelagibacter phage HTVC011P	NA	Viruses; dsDNA viruses, no RNA stage; Caudovirales; Podoviridae; unclassified Podoviridae
GCA_000905875.1	42084	59	Pelagibacter phage HTVC019P	NA	Viruses; dsDNA viruses, no RNA stage; Caudovirales; Podoviridae; unclassified Podoviridae
GCA_000917655.1	13023	2	Fusarium graminearum hypovirus 1	NA	Viruses; dsRNA viruses; Hypoviridae; Hypovirus; unclassified Hypovirus
GCA_000863185.1	10820	1	Sweet potato feathery mottle virus	NA	Viruses; ssRNA viruses; ssRNA positive-strand viruses, no DNA stage; Potyviridae; Potyvirus
GCA_002270845.1	17031	5	Tomato chlorotic spot virus	NA	Viruses; Riboviria; Orthornavirae; Negarnaviricota; Polyploviricotina; Ellioviricetes; Bunyavirales; Tospoviridae; Orthotospovirus; Tomato chlorotic spot tospovirus
GCA_000869065.1	190054	173	Nile crocodilepox virus	NA	Viruses; dsDNA viruses, no RNA stage; Poxviridae; Chordopoxvirinae; Crocodylidpoxvirus
GCA_000923535.2	10878	1	Nienokoue virus	NA	Viruses; ssRNA viruses; ssRNA positive-strand viruses, no DNA stage; Flaviviridae; unclassified Flaviviridae
GCA_000930015.1	5064	5	Yellow baboon polyomavirus 1	NA	Viruses; dsDNA viruses, no RNA stage; Polyomaviridae; unclassified Polyomaviridae
GCA_000928975.1	5181	6	Yellow baboon polyomavirus 2	NA	Viruses; Monodnaviria; Shotokuvirae; Cossaviricota; Papovaviricetes; Sepolyvirales; Polyomaviridae; Betapolyomavirus; Papio cynocephalus polyomavirus 2
GCA_006465665.1	186103	189	Variola major virus	Bangladesh1975	Viruses; Varidnaviria; Bamfordvirae; Nucleocytoviricota; Pokkesviricetes; Chitovirales; Poxviridae; Chordopoxvirinae; Orthopoxvirus; Variola virus
GCA_000868945.1	14462	35	His 1 virus	NA	Viruses; dsDNA viruses, no RNA stage; Salterprovirus
GCA_000864585.1	16067	35	His2 virus	NA	Viruses; dsDNA viruses, no RNA stage; Salterprovirus; unclassified Salterprovirus
GCA_001430295.1	9553	2	Bean rugose mosaic virus	NA	Viruses; ssRNA viruses; ssRNA positive-strand viruses, no DNA stage; Picornavirales; Secoviridae; Comovirinae; Comovirus
GCA_000849185.1	1239	1	Satellite tobacco mosaic virus	NA	Viruses; unassigned viruses; Virtovirus; Tobacco virtovirus 1
GCA_003179515.1	7830	7	Human papillomavirus type XS2	NA	Viruses; Monodnaviria; Shotokuvirae; Cossaviricota; Papovaviricetes; Zurhausenvirales; Papillomaviridae; Firstpapillomavirinae; Alphapapillomavirus; Alphapapillomavirus 2
GCA_000897555.1	8224	1	Chinese yam necrotic mosaic virus	NA	Viruses; ssRNA viruses; ssRNA positive-strand viruses, no DNA stage; Potyviridae; Macluravirus
GCA_003106905.1	9273	1	Hepatitis C virus subtype 4d	NA	Viruses; Riboviria; Orthornavirae; Kitrinoviricota; Flasuviricetes; Amarillovirales; Flaviviridae; Hepacivirus; Hepacivirus C; Hepatitis C virus genotype 4
GCA_000925415.1	11082	5	Tench rhabdovirus S64	NA	Viruses; ssRNA viruses; ssRNA negative-strand viruses; Mononegavirales; Rhabdoviridae; unclassified Rhabdoviridae
GCA_000924575.1	11096	5	Grass carp virus	NA	Viruses; Riboviria; Orthornavirae; Negarnaviricota; Haploviricotina; Monjiviricetes; Mononegavirales; Rhabdoviridae; Sprivivirus; Pike fry sprivivirus
GCA_002592865.1	30692	51	Lactococcus phage CaseusJM1	NA	Viruses; Duplodnaviria; Heunggongvirae; Uroviricota; Caudoviricetes; Caudovirales; Siphoviridae; Skunavirus; unclassified Skunavirus
GCA_000848505.1	18959	9	Ebola virus Mayinga Zaire 1976	NA	Viruses; Riboviria; Orthornavirae; Negarnaviricota; Haploviricotina; Monjiviricetes; Mononegavirales; Filoviridae; Ebolavirus; Zaire ebolavirus; Ebola virus
GCA_002603185.1	33516	54	Streptococcus phage phi20c	NA	Viruses; Duplodnaviria; Heunggongvirae; Uroviricota; Caudoviricetes; Caudovirales; Siphoviridae; unclassified Siphoviridae
GCA_002603205.1	42703	67	Streptococcus phage phi30c	NA	Viruses; Duplodnaviria; Heunggongvirae; Uroviricota; Caudoviricetes; Caudovirales; Siphoviridae; unclassified Siphoviridae
GCA_002603225.1	38539	64	Streptococcus phage phi5218	NA	Viruses; Duplodnaviria; Heunggongvirae; Uroviricota; Caudoviricetes; Caudovirales; Siphoviridae; unclassified Siphoviridae
GCA_002603245.1	36128	62	Streptococcus phage phi7917	NA	Viruses; Duplodnaviria; Heunggongvirae; Uroviricota; Caudoviricetes; Caudovirales; Siphoviridae; unclassified Siphoviridae
GCA_002603265.1	34367	57	Streptococcus phage phi891591	NA	Viruses; Duplodnaviria; Heunggongvirae; Uroviricota; Caudoviricetes; Caudovirales; Siphoviridae; unclassified Siphoviridae

gca	genome.size	proteome.size	phage.name	strain	Taxonomy
GCA_002745655.1	50470	60	Streptococcus phage phiD12	NA	Viruses; Duplodnaviria; Heunggongvirae; Uroviricota; Caudoviricetes; Caudovirales; Siphoviridae; unclassified Siphoviridae
GCA_002603285.1	35373	54	Streptococcus phage phiS10	NA	Viruses; Duplodnaviria; Heunggongvirae; Uroviricota; Caudoviricetes; Caudovirales; Siphoviridae; unclassified Siphoviridae
GCA_000839005.1	45636	63	Staphylococcus prophage phiPV83	P83 = ATCC 31890	Viruses; dsDNA viruses, no RNA stage; Caudovirales; Siphoviridae; unclassified Siphoviridae
GCA_000852305.1	8747	3	Citrus leaf blotch virus	NA	Viruses; ssRNA viruses; ssRNA positive-strand viruses, no DNA stage; Tymovirales; Betaflexiviridae; Trivirinae; Citrivirus
GCA_000849465.1	8432	7	Cherry necrotic rusty mottle virus	NA	Viruses; ssRNA viruses; ssRNA positive-strand viruses, no DNA stage; Tymovirales; Betaflexiviridae; unassigned Betaflexiviridae
GCA_000908295.1	2517	4	Acheta domesticus volvovirus	NA	Viruses; unclassified viruses; unclassified DNA viruses; unclassified ssDNA viruses; Volvovirus
GCA_002603305.1	46318	49	Vibrio phage JSF7	NA	Viruses; Duplodnaviria; Heunggongvirae; Uroviricota; Caudoviricetes; Caudovirales; Autographiviridae; Tawavirus; Vibrio virus JSF7
GCA_000427175.1	282895	296	Choristoneura rosaceana entomopoxvirus L	NA	Viruses; dsDNA viruses, no RNA stage; Poxviridae; Entomopoxvirinae; Betaentomopoxvirus; unclassified Betaentomopoxvirus
GCA_000427135.1	228750	247	Adoxophyes honmai entomopoxvirus L	NA	Viruses; dsDNA viruses, no RNA stage; Poxviridae; Entomopoxvirinae; Betaentomopoxvirus; unclassified Betaentomopoxvirus
GCA_000427155.1	281182	306	Mythimna separata entomopoxvirus L	NA	Viruses; dsDNA viruses, no RNA stage; Poxviridae; Entomopoxvirinae; Betaentomopoxvirus; unclassified Betaentomopoxvirus
GCA_000848525.1	6827	2	Botrytis virus F	NA	Viruses; ssRNA viruses; ssRNA positive-strand viruses, no DNA stage; Tymovirales; Gammaflexiviridae; Mycoflexivirus
GCA_000914395.1	1359	1	Sida yellow vein China alphasatellite	NA	Viruses; Satellites; Satellite Nucleic Acids; Single stranded DNA satellites; Alphasatellites; Begomovirus-associated alphasatellites; unclassified Begomovirus-associated alphasatellites
GCA_000909315.1	7537	1	Sebokele virus 1	NA	Viruses; ssRNA viruses; ssRNA positive-strand viruses, no DNA stage; Picornavirales; Picornaviridae; Parechovirus; unclassified Parechovirus
GCA_008797515.1	7447	1	Enterovirus C99	USAGA8410636	Viruses; Riboviria; Orthornavirae; Pisuviricota; Pisoniviricetes; Picornavirales; Picornaviridae; Enterovirus; Enterovirus C
GCA_000905815.1	6226	3	Andean potato mild mosaic virus	NA	Viruses; ssRNA viruses; ssRNA positive-strand viruses, no DNA stage; Tymovirales; Tymoviridae; Tymovirus
GCA_000905215.1	2351	2	Gastropod associated circular ssDNA virus	chc	Viruses; unclassified viruses; unclassified DNA viruses; unclassified ssDNA viruses
GCA_003443355.1	128298	227	Vibrio phage ICP1_2006_E	NA	Viruses; Duplodnaviria; Heunggongvirae; Uroviricota; Caudoviricetes; Caudovirales; Myoviridae; unclassified Myoviridae
GCA_003575525.1	126861	222	Vibrio phage ICP1_2011_A	NA	Viruses; Duplodnaviria; Heunggongvirae; Uroviricota; Caudoviricetes; Caudovirales; Myoviridae; unclassified Myoviridae
GCA_002603325.1	46048	57	Staphylococcus phage LH1	NA	Viruses; Duplodnaviria; Heunggongvirae; Uroviricota; Caudoviricetes; Caudovirales; Siphoviridae; Triavirus; unclassified Triavirus
GCA_000904695.1	41491	66	Mycobacterium phage Butters	NA	Viruses; dsDNA viruses, no RNA stage; Caudovirales; Siphoviridae; unclassified Siphoviridae
GCA_001470295.1	68355	101	Mycobacterium phage ShiVal	NA	Viruses; dsDNA viruses, no RNA stage; Caudovirales; Siphoviridae; unclassified Siphoviridae; PG1-like phages
GCA_002603345.1	82008	138	Bacillus phage Basilisk	NA	Viruses; Duplodnaviria; Heunggongvirae; Uroviricota; Caudoviricetes; Caudovirales; Siphoviridae; unclassified Siphoviridae
GCA_001504395.1	137918	218	Bacillus phage JL	NA	Viruses; dsDNA viruses, no RNA stage; Caudovirales; Myoviridae; Cp51virus; Bacillus virus JL
GCA_001505855.1	138877	220	Bacillus phage Shanette	NA	Viruses; dsDNA viruses, no RNA stage; Caudovirales; Myoviridae; Cp51virus; Bacillus virus Shanette
GCA_002624405.1	54312	103	Brevibacillus phage Jimmer2	NA	Viruses; Duplodnaviria; Heunggongvirae; Uroviricota; Caudoviricetes; Caudovirales; Myoviridae; Jimmervirus; Brevibacillus virus Jimmer
GCA_001551445.1	54312	103	Brevibacillus phage Jimmer1	NA	Viruses; dsDNA viruses, no RNA stage; Caudovirales; Myoviridae; unclassified Myoviridae
GCA_002603365.1	58573	102	Brevibacillus phage Emery	NA	Viruses; Duplodnaviria; Heunggongvirae; Uroviricota; Caudoviricetes; Caudovirales; Myoviridae; unclassified Myoviridae
GCA_001505195.1	45552	94	Brevibacillus phage Abouo	NA	Viruses; dsDNA viruses, no RNA stage; Caudovirales; Myoviridae; unclassified Myoviridae
GCA_000914135.1	45798	94	Brevibacillus phage Davies	NA	Viruses; dsDNA viruses, no RNA stage; Caudovirales; Myoviridae; unclassified Myoviridae
GCA_003177395.1	7871	8	human papillomavirus 82	NA	Viruses; Monodnaviria; Shotokuvirae; Cossaviricota; Papovaviricetes; Zurhausenvirales; Papillomaviridae; Firstpapillomavirinae; Alphapapillomavirus; Alphapapillomavirus 5
GCA_000886295.1	145289	134	Pseudocowpox virus	VR634	Viruses; dsDNA viruses, no RNA stage; Poxviridae; Chordopoxvirinae; Parapoxvirus
GCA_000844045.1	134431	131	Bovine papular stomatitis virus	BVAR02	Viruses; dsDNA viruses, no RNA stage; Poxviridae; Chordopoxvirinae; Parapoxvirus
GCA_000886335.1	9965	1	Sunflower chlorotic mottle virus	Common C	Viruses; ssRNA viruses; ssRNA positive-strand viruses, no DNA stage; Potyviridae; Potyvirus
GCA_000906695.1	2743	7	Corchorus yellow vein mosaic virus	NA	Viruses; ssDNA viruses; Geminiviridae; Begomovirus; unclassified Begomovirus

gca	genome.size	proteome.size	phage.name	strain	Taxonomy
GCA_000904215.1	1390	1	Corchorus yellow vein mosaic betasatellite	NA	Viruses; Tolecusatellitidae; Betasatellite
GCA_003726535.1	2692	2	Citrus yellow veinassociated virus	NA	Viruses; unclassified viruses
GCA_004146425.1	83142	98	Thermus phage TSP4	NA	Viruses; Duplodnaviria; Heunggongvirae; Uroviricota; Caudoviricetes; Caudovirales; Siphoviridae; Oshimavirus; unclassified Oshimavirus
GCA_000904595.1	14847	24	Sulfolobales Mexican fusellovirus 1	NA	Viruses; dsDNA viruses, no RNA stage; Fuselloviridae; unclassified Fuselloviridae
GCA_006445915.1	33802	36	Human mastadenovirus A	humanUSAUFL__Adv31200531P31H31F31	Viruses; dsDNA viruses, no RNA stage; Adenoviridae; Mastadenovirus
GCA_006433735.1	35818	53	Human mastadenovirus C	humanRUS16700200157P1H57F6	Viruses; dsDNA viruses, no RNA stage; Adenoviridae; Mastadenovirus
GCA_000847265.1	34185	29	Bovine mastadenovirus A	NA	Viruses; dsDNA viruses, no RNA stage; Adenoviridae; Mastadenovirus
GCA_000907735.1	103910	120	Alteromonas phage vB__AmaP__AD45P1	NA	Viruses; dsDNA viruses, no RNA stage; Caudovirales; Podoviridae; unclassified Podoviridae; Alteromonas phage vB__AmaP__AD45-P
GCA_002755495.1	104036	122	Alteromonas phage vB__AmaP__AD45P2	NA	Viruses; Duplodnaviria; Heunggongvirae; Uroviricota; Caudoviricetes; Caudovirales; Podoviridae; unclassified Podoviridae
GCA_002755515.1	101724	117	Alteromonas phage vB__AmaP__AD45P3	NA	Viruses; Duplodnaviria; Heunggongvirae; Uroviricota; Caudoviricetes; Caudovirales; Podoviridae; unclassified Podoviridae
GCA_002755535.1	100619	116	Alteromonas phage vB__AmaP__AD45P4	NA	Viruses; Duplodnaviria; Heunggongvirae; Uroviricota; Caudoviricetes; Caudovirales; Podoviridae; unclassified Podoviridae
GCA_002830705.1	4590	3	Scheffersomyces segobiensis virus L	NA	Viruses; Riboviria; Orthornavirae; Duplornaviricota; Chrymotiviricetes; Ghabrivirales; Totiviridae; Totivirus
GCA_013087205.1	17771	8	Bat paramyxovirus	NA	Viruses; Riboviria; Orthornavirae; Negarnaviricota; Haplovirícotina; Monjiviricetes; Mononegavirales; Paramyxoviridae; unclassified Paramyxoviridae
GCA_000910315.1	5983	5	Citrus vein enation virus	NA	Viruses; ssRNA viruses; ssRNA positive-strand viruses, no DNA stage; Luteoviridae; unclassified Luteoviridae
GCA_019446095.1	25386	25	Psittacine adenovirus 2	WVL1906501E	Viruses; Varidnaviria; Bamfordvirae; Preplasmiviricota; Tectiliviricetes; Rowavirales; Adenoviridae; unclassified Adenoviridae
GCA_000909775.1	197693	167	Invertebrate iridovirus 22	NA	Viruses; dsDNA viruses, no RNA stage; Iridoviridae; Iridovirus; unclassified Iridovirus
GCA_000914535.1	204815	177	Invertebrate iridovirus 25	NA	Viruses; dsDNA viruses, no RNA stage; Iridoviridae; Iridovirus; unclassified Iridovirus
GCA_002003815.1	10095	1	Palm Creek virus	NA	Viruses; ssRNA viruses; ssRNA positive-strand viruses, no DNA stage; Flaviviridae; Flavivirus; unclassified Flavivirus
GCA_000911975.1	2572	4	Chickpea chlorosis Australia virus	NA	Viruses; ssDNA viruses; Geminiviridae; Mastrevirus
GCA_000897615.1	2789	4	Sporobolus striate mosaic virus 1	NA	Viruses; ssDNA viruses; Geminiviridae; Mastrevirus
GCA_000899215.1	2716	4	Sporobolus striate mosaic virus 2	NA	Viruses; ssDNA viruses; Geminiviridae; Mastrevirus
GCA_000918835.1	10283	4	ROUT virus	NA	Viruses; ssRNA viruses; ssRNA negative-strand viruses; Arenaviridae; Reptarenavirus; Alethinophid 2 reptarenavirus
GCA_006426055.1	35968	47	Human mastadenovirus E	HAdVEUSA40542015P4H4F4	Viruses; dsDNA viruses, no RNA stage; Adenoviridae; Mastadenovirus
GCA_000846685.1	34214	34	Human mastadenovirus F	NA	Viruses; dsDNA viruses, no RNA stage; Adenoviridae; Mastadenovirus
GCA_006444035.1	35169	47	Human mastadenovirus D	humanDEUHEIM__00089198896P23H32F62	Viruses; dsDNA viruses, no RNA stage; Adenoviridae; Mastadenovirus
GCA_015221715.1	33215	28	Duck atadenovirus A	NA	Viruses; Varidnaviria; Bamfordvirae; Preplasmiviricota; Tectiliviricetes; Rowavirales; Adenoviridae; Atadenovirus
GCA_000886775.1	33213	31	Duck adenovirus 1	NA	Viruses; Varidnaviria; Bamfordvirae; Preplasmiviricota; Tectiliviricetes; Rowavirales; Adenoviridae; Atadenovirus; Duck atadenovirus A
GCA_000906235.1	5033	5	Human polyomavirus 12	hu1403	Viruses; dsDNA viruses, no RNA stage; Polyomaviridae; Alphapolyomavirus
GCA_000915295.1	2761	6	Tomato leaf curl Liwa virus	NA	Viruses; ssDNA viruses; Geminiviridae; Begomovirus; unclassified Begomovirus
GCA_014621645.1	32051	35	Porcine mastadenovirus B	Kasza__vL	Viruses; Varidnaviria; Bamfordvirae; Preplasmiviricota; Tectiliviricetes; Rowavirales; Adenoviridae; Mastadenovirus
GCA_018594905.1	11848	3	Xuan son virus	NA	Viruses; Riboviria; Orthornavirae; Negarnaviricota; Polyploviricotina; Ellioviricetes; Bunyavirales; Hantaviridae; Mammantavirinae; Mobatvirus; Xuan son mobatvirus
GCA_000837385.1	42942	60	Staphylococcus virus phiSLT	NA	Viruses; Duplodnaviria; Heunggongvirae; Uroviricota; Caudoviricetes; Caudovirales; Siphoviridae; Triavirus
GCA_000838125.1	108252	109	Culex nigripalpus nucleopolyhedrovirus	NA	Viruses; dsDNA viruses, no RNA stage; Baculoviridae; Deltabaculovirus
GCA_000909215.1	39108	50	Clostridium phage vB__CpeSCP51	NA	Viruses; dsDNA viruses, no RNA stage; Caudovirales; Siphoviridae; unclassified Siphoviridae

gca	genome.size	proteome.size	phage.name	strain	Taxonomy
GCA_000907175.1	47724	73	Mycobacterium phage vB_MapS_FF47	NA	Viruses; dsDNA viruses, no RNA stage; Caudovirales; Siphoviridae; Ff47virus; Mycobacterium virus Ff47
GCA_001271015.1	2745	7	Alfalfa leaf curl virus	NA	Viruses; ssDNA viruses; Geminiviridae; unclassified Geminiviridae; Capulaviruses
GCA_006437475.1	45622	55	Fowl aviadenovirus 4	NA	Viruses; Varidnaviria; Bamfordvirae; Preplasmiviricota; Tectiliviricetes; Rowavirales; Adenoviridae; Aviadenovirus; Fowl aviadenovirus C
GCA_002821325.1	10479	1	Theilers diseaseassociated virus	NA	Viruses; Riboviria; Orthornavirae; Kitrinoviricota; Flasuviricetes; Amarillovirales; Flaviviridae; Pegivirus; Pegivirus D
GCA_002640235.1	18511	11	Rotavirus A RVAHumanwtITAASTI232007G9P8	NA	Viruses; Riboviria; Orthornavirae; Duplornaviricota; Resentoviricetes; Reovirales; Reoviridae; Sedoreovirinae; Rotavirus; Rotavirus A; Rotavirus G9
GCA_002640255.1	18490	11	Rotavirus A RVAHumanwtITAAV212010G9P8	NA	Viruses; Riboviria; Orthornavirae; Duplornaviricota; Resentoviricetes; Reovirales; Reoviridae; Sedoreovirinae; Rotavirus; Rotavirus A; Rotavirus G9
GCA_002640275.1	18518	11	Rotavirus A RVAHumanwtITAAV282010G9P8	NA	Viruses; Riboviria; Orthornavirae; Duplornaviricota; Resentoviricetes; Reovirales; Reoviridae; Sedoreovirinae; Rotavirus; Rotavirus A; Rotavirus G9
GCA_002640295.1	18510	11	Rotavirus A RVAHumanwtITAJES112010G9P8	NA	Viruses; Riboviria; Orthornavirae; Duplornaviricota; Resentoviricetes; Reovirales; Reoviridae; Sedoreovirinae; Rotavirus; Rotavirus A; Rotavirus G9
GCA_002659935.1	17928	11	Rotavirus A RVAPigwtITA2CR2009G9P23	NA	Viruses; Riboviria; Orthornavirae; Duplornaviricota; Resentoviricetes; Reovirales; Reoviridae; Sedoreovirinae; Rotavirus; Rotavirus A; Rotavirus G9
GCA_002659955.1	17696	11	Rotavirus A RVAPigwtITA3BS2009G9P23	NA	Viruses; Riboviria; Orthornavirae; Duplornaviricota; Resentoviricetes; Reovirales; Reoviridae; Sedoreovirinae; Rotavirus; Rotavirus A; Rotavirus G9
GCA_002659995.1	17855	11	Rotavirus A RVAPigwtITA7RE2009G9P23	NA	Viruses; Riboviria; Orthornavirae; Duplornaviricota; Resentoviricetes; Reovirales; Reoviridae; Sedoreovirinae; Rotavirus; Rotavirus A; Rotavirus G9
GCA_000851145.1	13498	10	Influenza A virus AHong Kong107399H9N2	NA	Viruses; ssRNA viruses; ssRNA negative-strand viruses; Orthomyxoviridae; Influenzavirus A; Influenza A virus; H9N2 subtype
GCA_000847425.1	9550	1	Pegivirus A	Alab	Viruses; ssRNA viruses; ssRNA positive-strand viruses, no DNA stage; Flaviviridae; Pegivirus
GCA_000904655.1	11197	1	Equine pegivirus 1	NA	Viruses; ssRNA viruses; ssRNA positive-strand viruses, no DNA stage; Flaviviridae; Pegivirus; unclassified Pegivirus; Equine pegivirus
GCA_000907595.1	176227	273	Escherichia phage Lw1	NA	Viruses; dsDNA viruses, no RNA stage; Caudovirales; Myoviridae; Tevenvirinae; T4virus; unclassified T4virus
GCA_000870385.1	111953	126	Maruca vitrata nucleopolyhedrovirus	NA	Viruses; dsDNA viruses, no RNA stage; Baculoviridae; Alphabaculovirus
GCA_000870065.1	168041	169	Leucania separata nucleopolyhedrovirus	AH1	Viruses; dsDNA viruses, no RNA stage; Baculoviridae; Alphabaculovirus
GCA_000869305.1	135454	129	Clanis bilineata nucleopolyhedrovirus	NA	Viruses; dsDNA viruses, no RNA stage; Baculoviridae; Alphabaculovirus
GCA_000898495.1	8857	5	Sweet potato C6 virus	NA	Viruses; ssRNA viruses; ssRNA positive-strand viruses, no DNA stage; Tymovirales; Betaflexiviridae; Quinvirinae; Carlavirus
GCA_002815695.1	14409	6	Drosophila affinis sigmavirus	NA	Viruses; Riboviria; Orthornavirae; Negarnaviricota; Haploviricotina; Monjiviricetes; Mononegavirales; Rhabdoviridae; Sigmavirus
GCA_000915835.1	168876	272	Bacillus phage vB_BanSTsamsa	NA	Viruses; dsDNA viruses, no RNA stage; Caudovirales; Siphoviridae; unclassified Siphoviridae
GCA_002755555.1	38253	56	Brucella phage Bk	NA	Viruses; Duplodnaviria; Heunggongvirae; Uroviricota; Caudoviricetes; Caudovirales; Podoviridae; Perisivirus; Brucella virus Pr
GCA_002755575.1	41142	58	Brucella phage Fz	NA	Viruses; Duplodnaviria; Heunggongvirae; Uroviricota; Caudoviricetes; Caudovirales; Podoviridae; Perisivirus; Brucella virus Tb
GCA_002755595.1	38253	56	Brucella phage S708	NA	Viruses; Duplodnaviria; Heunggongvirae; Uroviricota; Caudoviricetes; Caudovirales; Podoviridae; Perisivirus; Brucella virus Pr
GCA_000914455.1	42906	65	Staphylococcus phage StauST3984	NA	Viruses; dsDNA viruses, no RNA stage; Caudovirales; Siphoviridae; unclassified Siphoviridae
GCA_000916135.1	43301	65	Staphylococcus phage StauST3985	NA	Viruses; dsDNA viruses, no RNA stage; Caudovirales; Siphoviridae; unclassified Siphoviridae
GCA_002622505.1	61818	89	Pseudomonas phage LKO4	NA	Viruses; Duplodnaviria; Heunggongvirae; Uroviricota; Caudoviricetes; Caudovirales; Siphoviridae; Yuavirus; Pseudomonas virus LKO4
GCA_000910075.1	27190	42	Bacillus phage MGB1	NA	Viruses; dsDNA viruses, no RNA stage; Caudovirales; Podoviridae; unclassified Podoviridae
GCA_001292935.1	3341	2	Verticillium dahliae partitivirus 1	NA	Viruses; dsRNA viruses; Partitiviridae; unclassified Partitiviridae
GCA_000847745.1	5776	5	Beet chlorosis virus	NA	Viruses; ssRNA viruses; ssRNA positive-strand viruses, no DNA stage; Luteoviridae; Polerovirus
GCA_000855905.1	5641	6	Turnip yellows virus	NA	Viruses; ssRNA viruses; ssRNA positive-strand viruses, no DNA stage; Luteoviridae; Polerovirus
GCA_000911775.1	5208	7	Capraria yellow spot Yucatan virus	NA	Viruses; ssDNA viruses; Geminiviridae; Begomovirus; unclassified Begomovirus
GCA_000848465.1	8018	4	Cherry mottle leaf virus	SA116221	Viruses; ssRNA viruses; ssRNA positive-strand viruses, no DNA stage; Tymovirales; Betaflexiviridae; Trivirinae; Trichovirus
GCA_000904675.1	5142	2	Ustilaginoidea virens RNA virus 1	NA	Viruses; dsRNA viruses; Totiviridae; Victorivirus; unclassified Victorivirus

gca	genome.size	proteome.size	phage.name	strain	Taxonomy
GCA_002821485.1	2629	5	Abutilon golden mosaic Yucatan virus	NA	Viruses; Monodnaviria; Shotokuvirae; Cressdnaviricota; Repensiviricetes; Geplafuvirales; Geminiviridae; Begomovirus; Abutilon golden mosaic virus
GCA_002824725.1	2602	5	Vigna yellow mosaic virus	NA	Viruses; Monodnaviria; Shotokuvirae; Cressdnaviricota; Repensiviricetes; Geplafuvirales; Geminiviridae; Begomovirus
GCA_000907155.1	8397	7	Cherry rusty mottle associated virus	NA	Viruses; ssRNA viruses; ssRNA positive-strand viruses, no DNA stage; Tymovirales; Betaflexiviridae; Quinvirinae; Robigovirus
GCA_000861885.1	8251	1	Aichi virus 1	NA	Viruses; ssRNA viruses; ssRNA positive-strand viruses, no DNA stage; Picornavirales; Picornaviridae; Kobuvirus; Aichivirus A
GCA_002822425.1	2763	6	Honeysuckle yellow vein virusAustraliaAyr1983	NA	Viruses; Monodnaviria; Shotokuvirae; Cressdnaviricota; Repensiviricetes; Geplafuvirales; Geminiviridae; Begomovirus; Honeysuckle yellow vein virus
GCA_000899515.1	7212	6	human papillomavirus 166	NA	Viruses; Monodnaviria; Shotokuvirae; Cossaviricota; Papovaviricetes; Zurhausenvirales; Papillomaviridae; Firstpapillomavirinae; Gammapapillomavirus; Gammapapillomavirus 19
GCA_003179375.1	7252	7	Human papillomavirus 169	NA	Viruses; Monodnaviria; Shotokuvirae; Cossaviricota; Papovaviricetes; Zurhausenvirales; Papillomaviridae; Firstpapillomavirinae; Gammapapillomavirus; Gammapapillomavirus 11
GCA_003179395.1	7233	7	human papillomavirus 164	NA	Viruses; Monodnaviria; Shotokuvirae; Cossaviricota; Papovaviricetes; Zurhausenvirales; Papillomaviridae; Firstpapillomavirinae; Gammapapillomavirus; Gammapapillomavirus 8
GCA_001430115.1	7233	6	human papillomavirus 163	NA	Viruses; Monodnaviria; Shotokuvirae; Cossaviricota; Papovaviricetes; Zurhausenvirales; Papillomaviridae; Firstpapillomavirinae; Gammapapillomavirus; Gammapapillomavirus 20
GCA_003179415.1	7214	6	human papillomavirus 162	NA	Viruses; Monodnaviria; Shotokuvirae; Cossaviricota; Papovaviricetes; Zurhausenvirales; Papillomaviridae; Firstpapillomavirinae; Gammapapillomavirus; Gammapapillomavirus 19
GCA_002826985.1	7238	6	Human papillomavirus type 161	NA	Viruses; Monodnaviria; Shotokuvirae; Cossaviricota; Papovaviricetes; Zurhausenvirales; Papillomaviridae; Firstpapillomavirinae; Gammapapillomavirus; Gammapapillomavirus 19
GCA_003179435.1	7417	6	Human papillomavirus 170	NA	Viruses; Monodnaviria; Shotokuvirae; Cossaviricota; Papovaviricetes; Zurhausenvirales; Papillomaviridae; Firstpapillomavirinae; Gammapapillomavirus; Gammapapillomavirus 7
GCA_003179455.1	7129	6	Human papillomavirus 165	NA	Viruses; Monodnaviria; Shotokuvirae; Cossaviricota; Papovaviricetes; Zurhausenvirales; Papillomaviridae; Firstpapillomavirinae; Gammapapillomavirus; Gammapapillomavirus 12
GCA_003087875.1	11490	4	SFTS virus HNXY_115	NA	Viruses; Riboviria; Orthornavirae; Negarnaviricota; Polyploviricotina; Ellioviricetes; Bunyavirales; Phenuiviridae; Phlebovirus; SFTS phlebovirus
GCA_003087915.1	11490	4	SFTS virus HNXY_130	NA	Viruses; Riboviria; Orthornavirae; Negarnaviricota; Polyploviricotina; Ellioviricetes; Bunyavirales; Phenuiviridae; Phlebovirus; SFTS phlebovirus
GCA_003087935.1	11490	4	SFTS virus HNXY_144	NA	Viruses; Riboviria; Orthornavirae; Negarnaviricota; Polyploviricotina; Ellioviricetes; Bunyavirales; Phenuiviridae; Phlebovirus; SFTS phlebovirus
GCA_003087955.1	11490	4	SFTS virus HNXY_164	NA	Viruses; Riboviria; Orthornavirae; Negarnaviricota; Polyploviricotina; Ellioviricetes; Bunyavirales; Phenuiviridae; Phlebovirus; SFTS phlebovirus
GCA_003087975.1	11490	4	SFTS virus HNXY_170	NA	Viruses; Riboviria; Orthornavirae; Negarnaviricota; Polyploviricotina; Ellioviricetes; Bunyavirales; Phenuiviridae; Phlebovirus; SFTS phlebovirus
GCA_003087995.1	11490	4	SFTS virus HNXY_174	NA	Viruses; Riboviria; Orthornavirae; Negarnaviricota; Polyploviricotina; Ellioviricetes; Bunyavirales; Phenuiviridae; Phlebovirus; SFTS phlebovirus
GCA_003088015.1	11490	4	SFTS virus HNXY_182	NA	Viruses; Riboviria; Orthornavirae; Negarnaviricota; Polyploviricotina; Ellioviricetes; Bunyavirales; Phenuiviridae; Phlebovirus; SFTS phlebovirus
GCA_003088035.1	11490	4	SFTS virus HNXY_186	NA	Viruses; Riboviria; Orthornavirae; Negarnaviricota; Polyploviricotina; Ellioviricetes; Bunyavirales; Phenuiviridae; Phlebovirus; SFTS phlebovirus
GCA_003088055.1	11490	4	SFTS virus HNXY_188	NA	Viruses; Riboviria; Orthornavirae; Negarnaviricota; Polyploviricotina; Ellioviricetes; Bunyavirales; Phenuiviridae; Phlebovirus; SFTS phlebovirus
GCA_003088075.1	11490	4	SFTS virus HNXY_191	NA	Viruses; Riboviria; Orthornavirae; Negarnaviricota; Polyploviricotina; Ellioviricetes; Bunyavirales; Phenuiviridae; Phlebovirus; SFTS phlebovirus
GCA_003088095.1	11490	4	SFTS virus HNXY_195	NA	Viruses; Riboviria; Orthornavirae; Negarnaviricota; Polyploviricotina; Ellioviricetes; Bunyavirales; Phenuiviridae; Phlebovirus; SFTS phlebovirus
GCA_003088135.1	11490	4	SFTS virus HNXY_2	NA	Viruses; Riboviria; Orthornavirae; Negarnaviricota; Polyploviricotina; Ellioviricetes; Bunyavirales; Phenuiviridae; Phlebovirus; SFTS phlebovirus
GCA_003088155.1	11490	4	SFTS virus HNXY_202	NA	Viruses; Riboviria; Orthornavirae; Negarnaviricota; Polyploviricotina; Ellioviricetes; Bunyavirales; Phenuiviridae; Phlebovirus; SFTS phlebovirus
GCA_003088175.1	11490	4	SFTS virus HNXY_206	NA	Viruses; Riboviria; Orthornavirae; Negarnaviricota; Polyploviricotina; Ellioviricetes; Bunyavirales; Phenuiviridae; Phlebovirus; SFTS phlebovirus
GCA_003088195.1	11490	4	SFTS virus HNXY_207	NA	Viruses; Riboviria; Orthornavirae; Negarnaviricota; Polyploviricotina; Ellioviricetes; Bunyavirales; Phenuiviridae; Phlebovirus; SFTS phlebovirus
GCA_003088215.1	11490	4	SFTS virus HNXY_212	NA	Viruses; Riboviria; Orthornavirae; Negarnaviricota; Polyploviricotina; Ellioviricetes; Bunyavirales; Phenuiviridae; Phlebovirus; SFTS phlebovirus
GCA_003088235.1	11490	4	SFTS virus HNXY_224	NA	Viruses; Riboviria; Orthornavirae; Negarnaviricota; Polyploviricotina; Ellioviricetes; Bunyavirales; Phenuiviridae; Phlebovirus; SFTS phlebovirus
GCA_003088255.1	11490	4	SFTS virus HNXY_231	NA	Viruses; Riboviria; Orthornavirae; Negarnaviricota; Polyploviricotina; Ellioviricetes; Bunyavirales; Phenuiviridae; Phlebovirus; SFTS phlebovirus
GCA_003088275.1	11490	4	SFTS virus HNXY_245	NA	Viruses; Riboviria; Orthornavirae; Negarnaviricota; Polyploviricotina; Ellioviricetes; Bunyavirales; Phenuiviridae; Phlebovirus; SFTS phlebovirus
GCA_003088295.1	11490	4	SFTS virus HNXY_262	NA	Viruses; Riboviria; Orthornavirae; Negarnaviricota; Polyploviricotina; Ellioviricetes; Bunyavirales; Phenuiviridae; Phlebovirus; SFTS phlebovirus
GCA_003088315.1	11490	4	SFTS virus HNXY_278	NA	Viruses; Riboviria; Orthornavirae; Negarnaviricota; Polyploviricotina; Ellioviricetes; Bunyavirales; Phenuiviridae; Phlebovirus; SFTS phlebovirus

gca	genome.size	proteome.size	phage.name	strain	Taxonomy
GCA_003088355.1	11490	4	SFTS virus HNXY_293	NA	Viruses; Riboviria; Orthornavirae; Negarnaviricota; Polyploviricotina; Ellioviricetes; Bunyavirales; Phenuiviridae; Phlebovirus; SFTS phlebovirus
GCA_003088375.1	11490	4	SFTS virus HNXY_31	NA	Viruses; Riboviria; Orthornavirae; Negarnaviricota; Polyploviricotina; Ellioviricetes; Bunyavirales; Phenuiviridae; Phlebovirus; SFTS phlebovirus
GCA_003088395.1	11490	4	SFTS virus HNXY_319	NA	Viruses; Riboviria; Orthornavirae; Negarnaviricota; Polyploviricotina; Ellioviricetes; Bunyavirales; Phenuiviridae; Phlebovirus; SFTS phlebovirus
GCA_003088415.1	11490	4	SFTS virus HNXY_327	NA	Viruses; Riboviria; Orthornavirae; Negarnaviricota; Polyploviricotina; Ellioviricetes; Bunyavirales; Phenuiviridae; Phlebovirus; SFTS phlebovirus
GCA_003088435.1	11490	4	SFTS virus HNXY_93	NA	Viruses; Riboviria; Orthornavirae; Negarnaviricota; Polyploviricotina; Ellioviricetes; Bunyavirales; Phenuiviridae; Phlebovirus; SFTS phlebovirus
GCA_000907495.1	43882	56	Pseudoalteromonas phage RIO1	NA	Viruses; dsDNA viruses, no RNA stage; Caudovirales; Podoviridae; unclassified Podoviridae
GCA_000919815.2	5462	8	Allamanda leaf mottle distortion virus	NA	Viruses; ssDNA viruses; Geminiviridae; Begomovirus; unclassified Begomovirus
GCA_002633585.1	17602	20	Staphylococcus phage PSa3	NA	Viruses; Duplodnaviria; Heunggongvirae; Uroviricota; Caudoviricetes; Caudovirales; Podoviridae; Rakietenvirinae; Rosenblumvirus; Staphylococcus virus PSa3
GCA_002821505.1	2760	6	Ageratum enation virusPapaver somniferum	NA	Viruses; Monodnaviria; Shotokuvirae; Cressdnaviricota; Repensiviricetes; Geplafuvirales; Geminiviridae; Begomovirus; Ageratum enation virus
GCA_000906375.1	4780	2	Hop trefoil cryptic virus 2	NA	Viruses; dsRNA viruses; Partitiviridae; Betapartitivirus
GCA_000904755.1	4783	2	Red clover cryptic virus 2	NA	Viruses; dsRNA viruses; Partitiviridae; Betapartitivirus
GCA_000907235.1	4783	2	White clover cryptic virus 2	NA	Viruses; dsRNA viruses; Partitiviridae; Betapartitivirus
GCA_002868195.1	4798	2	Crimson clover cryptic virus 2	NA	Viruses; Riboviria; Orthornavirae; Pisuviricota; Duplopiviricetes; Durnavirales; Partitiviridae; Betapartitivirus
GCA_000908315.1	4784	2	Dill cryptic virus 2	NA	Viruses; dsRNA viruses; Partitiviridae; Betapartitivirus
GCA_000908975.1	9499	2	Habenaria mosaic virus	NA	Viruses; ssRNA viruses; ssRNA positive-strand viruses, no DNA stage; Potyviridae; Potyvirus; unclassified Potyvirus
GCA_000847185.1	144575	151	Yabalike disease virus	NA	Viruses; dsDNA viruses, no RNA stage; Poxviridae; Chordopoxvirinae; Yatapoxvirus; Tanapox virus
GCA_000886555.1	8517	7	Kalanchoe latent virus	NA	Viruses; ssRNA viruses; ssRNA positive-strand viruses, no DNA stage; Tymovirales; Betaflexiviridae; Quinvirinae; Carlavirus
GCA_000908875.1	11353	7	Burdock mottle virus	NA	Viruses; ssRNA viruses; ssRNA positive-strand viruses, no DNA stage; Benyviridae; Benyvirus
GCA_000915155.1	43378	71	Edwardsiella phage PEi21	NA	Viruses; dsDNA viruses, no RNA stage; Caudovirales; Myoviridae; unclassified Myoviridae; Edwardsiella phage PEi2
GCA_001882015.1	9224	1	Bat hepacivirus	NA	Viruses; ssRNA viruses; ssRNA positive-strand viruses, no DNA stage; Flaviviridae; Hepacivirus; unclassified Hepacivirus
GCA_000909695.1	46978	63	Flavobacterium phage 6H	NA	Viruses; dsDNA viruses, no RNA stage; unclassified dsDNA phages
GCA_000909975.1	80955	138	Mycobacterium phage PegLeg	NA	Viruses; dsDNA viruses, no RNA stage; Caudovirales; Siphoviridae; unclassified Siphoviridae
GCA_000909195.1	53369	97	Mycobacterium phage CASbig	NA	Viruses; dsDNA viruses, no RNA stage; Caudovirales; Siphoviridae; unclassified Siphoviridae
GCA_000907575.1	53359	83	Mycobacterium phage WIVsmall	NA	Viruses; dsDNA viruses, no RNA stage; Caudovirales; Siphoviridae; unclassified Siphoviridae
GCA_000915675.1	43153	51	Croceibacter phage P2559Y	NA	Viruses; dsDNA viruses, no RNA stage; Caudovirales; Siphoviridae; unclassified Siphoviridae
GCA_000908495.1	51252	75	Streptomyces phage Lika	NA	Viruses; dsDNA viruses, no RNA stage; Caudovirales; Siphoviridae; unclassified Siphoviridae
GCA_000909095.1	51552	77	Streptomyces phage Sujidade	NA	Viruses; dsDNA viruses, no RNA stage; Caudovirales; Siphoviridae; unclassified Siphoviridae
GCA_000910055.1	51077	76	Streptomyces phage Zemlya	NA	Viruses; dsDNA viruses, no RNA stage; Caudovirales; Siphoviridae; unclassified Siphoviridae
GCA_000908575.1	154872	232	Mycobacterium phage Astraea	NA	Viruses; dsDNA viruses, no RNA stage; Caudovirales; Myoviridae; Bxz1virus; unclassified Bxz1virus
GCA_002603465.1	76652	132	Mycobacterium phage Breezona	NA	Viruses; Duplodnaviria; Heunggongvirae; Uroviricota; Caudoviricetes; Caudovirales; Siphoviridae; Bronvirus; Mycobacterium virus Faith1
GCA_000909955.2	75736	147	Mycobacterium phage Dumbo	NA	Viruses; dsDNA viruses, no RNA stage; Caudovirales; Siphoviridae; unclassified Siphoviridae
GCA_000908475.1	47109	77	Mycobacterium phage Fishburne	NA	Viruses; dsDNA viruses, no RNA stage; Caudovirales; Siphoviridae; unclassified Siphoviridae
GCA_000909075.1	68598	97	Mycobacterium phage Newman	NA	Viruses; dsDNA viruses, no RNA stage; Caudovirales; Siphoviridae; unclassified Siphoviridae; PG1-like phages
GCA_002597945.1	51373	88	Mycobacterium phage Dhanush	NA	Viruses; Duplodnaviria; Heunggongvirae; Uroviricota; Caudoviricetes; Caudovirales; Siphoviridae; Fromanvirus; Mycobacterium virus Peaches

gca	genome.size	proteome.size	phage.name	strain	Taxonomy
GCA_000907455.1	52617	84	Mycobacterium phage HINdeR	NA	Viruses; dsDNA viruses, no RNA stage; Caudovirales; Siphoviridae; unclassified Siphoviridae
GCA_000910215.1	59626	106	Mycobacterium phage Job42	NA	Viruses; dsDNA viruses, no RNA stage; Caudovirales; Siphoviridae; unclassified Siphoviridae
GCA_000909355.1	50679	85	Mycobacterium phage Jobu08	NA	Viruses; dsDNA viruses, no RNA stage; Caudovirales; Siphoviridae; unclassified Siphoviridae
GCA_000909935.1	52057	92	Mycobacterium phage PattyP	NA	Viruses; dsDNA viruses, no RNA stage; Caudovirales; Siphoviridae; unclassified Siphoviridae
GCA_000908455.1	74711	141	Mycobacterium phage Phrux	NA	Viruses; dsDNA viruses, no RNA stage; Caudovirales; Siphoviridae; unclassified Siphoviridae
GCA_002755615.1	67702	100	Mycobacterium phage SDcharge11	NA	Viruses; Duplodnaviria; Heunggongvirae; Uroviricota; Caudoviricetes; Caudovirales; Siphoviridae; Bclasvirinae; Pegunavirus; Mycobacterium virus Soto
GCA_000907435.1	49894	80	Mycobacterium phage Severus	NA	Viruses; dsDNA viruses, no RNA stage; Caudovirales; Siphoviridae; unclassified Siphoviridae
GCA_000909915.1	56279	99	Mycobacterium phage SiSi	NA	Viruses; dsDNA viruses, no RNA stage; Caudovirales; Siphoviridae; unclassified Siphoviridae
GCA_002603445.1	76653	132	Mycobacterium phage Winky	NA	Viruses; Duplodnaviria; Heunggongvirae; Uroviricota; Caudoviricetes; Caudovirales; Siphoviridae; Bronvirus; Mycobacterium virus Faith1
GCA_002755635.1	50891	83	Mycobacterium phage Methuselah	NA	Viruses; Duplodnaviria; Heunggongvirae; Uroviricota; Caudoviricetes; Caudovirales; Siphoviridae; Fromanvirus; Mycobacterium virus Bxz2
GCA_000923635.1	149162	269	Cronobacter phage CR8	NA	Viruses; dsDNA viruses, no RNA stage; Caudovirales; Myoviridae; Vequintavirinae; Cr3virus; Cronobacter virus CR8
GCA_001503575.1	182145	268	Cronobacter phage S13	NA	Viruses; dsDNA viruses, no RNA stage; Caudovirales; Myoviridae; unclassified Myoviridae
GCA_000909175.1	157482	241	Mycobacterium phage Gizmo	NA	Viruses; dsDNA viruses, no RNA stage; Caudovirales; Myoviridae; Bxz1virus; unclassified Bxz1virus
GCA_000908435.1	76479	145	Mycobacterium phage Phaux	NA	Viruses; dsDNA viruses, no RNA stage; Caudovirales; Siphoviridae; unclassified Siphoviridae
GCA_000907555.1	156558	237	Mycobacterium phage ArcherS7	NA	Viruses; dsDNA viruses, no RNA stage; Caudovirales; Myoviridae; Bxz1virus; unclassified Bxz1virus
GCA_000909035.1	76179	146	Mycobacterium phage Murphy	NA	Viruses; dsDNA viruses, no RNA stage; Caudovirales; Siphoviridae; unclassified Siphoviridae
GCA_002603525.1	64746	66	Pseudomonas phage LKA5	NA	Viruses; Duplodnaviria; Heunggongvirae; Uroviricota; Caudoviricetes; Caudovirales; Podoviridae; Hollowayvirus; unclassified Hollowayvirus
GCA_002755655.1	38689	51	Salmonella phage SPC32H	NA	Viruses; Duplodnaviria; Heunggongvirae; Uroviricota; Caudoviricetes; Caudovirales; Podoviridae; Uetakevirus; Salmonella virus SPN1S
GCA_002755675.1	38689	51	Salmonella phage SPC32N	NA	Viruses; Duplodnaviria; Heunggongvirae; Uroviricota; Caudoviricetes; Caudovirales; Podoviridae; Uetakevirus; Salmonella virus SPN1S
GCA_000910035.1	46639	73	Mycobacterium phage Chy4	NA	Viruses; dsDNA viruses, no RNA stage; Caudovirales; Siphoviridae; unclassified Siphoviridae
GCA_000908555.1	51214	88	Mycobacterium phage Chy5	NA	Viruses; dsDNA viruses, no RNA stage; Caudovirales; Siphoviridae; unclassified Siphoviridae
GCA_002755695.1	39318	58	Mycobacterium phage Bo4	NA	Viruses; Duplodnaviria; Heunggongvirae; Uroviricota; Caudoviricetes; Caudovirales; Siphoviridae; Liefievirus; unclassified Liefievirus
GCA_002755715.1	39520	60	Mycobacterium phage DNAIII	NA	Viruses; Duplodnaviria; Heunggongvirae; Uroviricota; Caudoviricetes; Caudovirales; Siphoviridae; Liefievirus; unclassified Liefievirus
GCA_000910295.1	39981	59	Mycobacterium phage Leo	NA	Viruses; dsDNA viruses, no RNA stage; Caudovirales; Siphoviridae; unclassified Siphoviridae
GCA_000913575.1	136910	220	Escherichia phage JES2013	NA	Viruses; dsDNA viruses, no RNA stage; Caudovirales; Myoviridae; Vequintavirinae; V5virus; Escherichia virus JES2013
GCA_000915135.1	88135	164	Pseudomonas phage PAK_P5	NA	Viruses; dsDNA viruses, no RNA stage; Caudovirales; Myoviridae; Kpp10virus; Pseudomonas virus PAKP3
GCA_000915815.1	88255	166	Pseudomonas phage CHA_P1	NA	Viruses; dsDNA viruses, no RNA stage; Caudovirales; Myoviridae; Kpp10virus; Pseudomonas virus PAKP3
GCA_000914095.1	93147	172	Pseudomonas phage PAK_P4	NA	Viruses; dsDNA viruses, no RNA stage; Caudovirales; Myoviridae; Pakpunavirus; Pseudomonas virus PAKP4
GCA_000908955.1	11703	29	Cellulophaga phage phi482	NA	Viruses; unclassified viruses; unclassified DNA viruses; unclassified ssDNA bacterial viruses
GCA_000910435.1	6453	13	Cellulophaga phage phi122	NA	Viruses; Monodnaviria; Sangervirae; Phixviricota; Malgrandaviricetes; Petitvirales; Microviridae; unclassified Microviridae
GCA_000908935.1	57447	114	Cellulophaga phage phi191	NA	Viruses; Duplodnaviria; Heunggongvirae; Uroviricota; Caudoviricetes; Caudovirales; Siphoviridae; unclassified Siphoviridae
GCA_000909575.1	75994	129	Cellulophaga phage phi193	NA	Viruses; Duplodnaviria; Heunggongvirae; Uroviricota; Caudoviricetes; Caudovirales; Podoviridae; unclassified Podoviridae; crAss-like viruses
GCA_000908055.1	145343	198	Cellulophaga phage phi172	NA	Viruses; Duplodnaviria; Heunggongvirae; Uroviricota; Caudoviricetes; Caudovirales; Podoviridae; Lightbulbvirus; Cellulophaga virus Cba172
GCA_002600445.1	54015	80	Cellulophaga phage phi3ST2	NA	Viruses; Duplodnaviria; Heunggongvirae; Uroviricota; Caudoviricetes; Caudovirales; Myoviridae; unclassified Myoviridae

gca	genome.size	proteome.size	phage.name	strain	Taxonomy
GCA_000908035.1	34844	54	Cellulophaga phage phi461	NA	Viruses; Duplodnaviria; Heunggongvirae; Uroviricota; Caudoviricetes; Caudovirales; Siphoviridae; unclassified Siphoviridae
GCA_002603605.1	72529	101	Cellulophaga phage phi401	NA	Viruses; Duplodnaviria; Heunggongvirae; Uroviricota; Caudoviricetes; Caudovirales; Podoviridae; unclassified Podoviridae; crAss-like viruses
GCA_000909675.1	39148	64	Cellulophaga phage phi121	NA	Viruses; Duplodnaviria; Heunggongvirae; Uroviricota; Caudoviricetes; Caudovirales; Siphoviridae; Helsingorvirus; Cellulophaga virus Cba121
GCA_000909635.1	72534	101	Cellulophaga phage phi381	NA	Viruses; Duplodnaviria; Heunggongvirae; Uroviricota; Caudoviricetes; Caudovirales; Podoviridae; unclassified Podoviridae; crAss-like viruses
GCA_002603585.1	39151	64	Cellulophaga phage phi123	NA	Viruses; Duplodnaviria; Heunggongvirae; Uroviricota; Caudoviricetes; Caudovirales; Siphoviridae; Helsingorvirus; Cellulophaga virus Cba121; Cellulophaga phage phi12:1
GCA_000909655.1	38776	65	Cellulophaga phage phi171	NA	Viruses; Duplodnaviria; Heunggongvirae; Uroviricota; Caudoviricetes; Caudovirales; Siphoviridae; Helsingorvirus; Cellulophaga virus Cba171
GCA_000910535.1	53664	106	Cellulophaga phage phi101	NA	Viruses; Duplodnaviria; Heunggongvirae; Uroviricota; Caudoviricetes; Caudovirales; Siphoviridae; unclassified Siphoviridae
GCA_000911595.1	39189	65	Cellulophaga phage phi181	NA	Viruses; Duplodnaviria; Heunggongvirae; Uroviricota; Caudoviricetes; Caudovirales; Siphoviridae; Helsingorvirus; Cellulophaga virus Cba181
GCA_000910515.1	71443	123	Cellulophaga phage phi183	NA	Viruses; Duplodnaviria; Heunggongvirae; Uroviricota; Caudoviricetes; Caudovirales; Podoviridae; unclassified Podoviridae; crAss-like viruses
GCA_002755735.1	78275	109	Cellulophaga phage phi192	NA	Viruses; Duplodnaviria; Heunggongvirae; Uroviricota; Caudoviricetes; Caudovirales; Siphoviridae; Cbastvirus; Cellulophaga virus ST; Cellulophaga phage phiST
GCA_000911615.1	72961	121	Cellulophaga phage phi463	NA	Viruses; Duplodnaviria; Heunggongvirae; Uroviricota; Caudoviricetes; Caudovirales; Podoviridae; unclassified Podoviridae; crAss-like viruses
GCA_000910495.1	6478	13	Cellulophaga phage phi12a1	NA	Viruses; Monodnaviria; Sangervirae; Phixviricota; Malgrandaviricetes; Petitvirales; Microviridae; unclassified Microviridae
GCA_000909615.1	100418	133	Cellulophaga phage phi142	NA	Viruses; Duplodnaviria; Heunggongvirae; Uroviricota; Caudoviricetes; Caudovirales; Podoviridae; unclassified Podoviridae; crAss-like viruses
GCA_002755755.1	76666	104	Cellulophaga phage phi131	NA	Viruses; Duplodnaviria; Heunggongvirae; Uroviricota; Caudoviricetes; Caudovirales; Siphoviridae; Cbastvirus; Cellulophaga virus ST; Cellulophaga phage phiST
GCA_000908015.1	28760	48	Cellulophaga phage phi391	NA	Viruses; Duplodnaviria; Heunggongvirae; Uroviricota; Caudoviricetes; Caudovirales; Siphoviridae; unclassified Siphoviridae
GCA_002603625.1	38476	63	Cellulophaga phage phi182	NA	Viruses; Duplodnaviria; Heunggongvirae; Uroviricota; Caudoviricetes; Caudovirales; Siphoviridae; Helsingorvirus; Cellulophaga virus Cba181; Cellulophaga phage phi18:1
GCA_002755775.1	6478	13	Cellulophaga phage phi184	NA	Viruses; Monodnaviria; Sangervirae; Phixviricota; Malgrandaviricetes; Petitvirales; Microviridae; unclassified Microviridae; Cellulophaga phage phi12a:1
GCA_002600485.1	54012	80	Cellulophaga phage phi382	NA	Viruses; Duplodnaviria; Heunggongvirae; Uroviricota; Caudoviricetes; Caudovirales; Myoviridae; unclassified Myoviridae
GCA_002755795.1	6478	13	Cellulophaga phage phi481	NA	Viruses; Monodnaviria; Sangervirae; Phixviricota; Malgrandaviricetes; Petitvirales; Microviridae; unclassified Microviridae; Cellulophaga phage phi12a:1
GCA_000910475.1	145865	197	Cellulophaga phage phi41	NA	Viruses; Duplodnaviria; Heunggongvirae; Uroviricota; Caudoviricetes; Caudovirales; Podoviridae; Lightbulbvirus; Cellulophaga virus Cba41
GCA_000907995.1	72369	127	Cellulophaga phage phi132	NA	Viruses; Duplodnaviria; Heunggongvirae; Uroviricota; Caudoviricetes; Caudovirales; Podoviridae; unclassified Podoviridae; crAss-like viruses
GCA_000908375.1	2539	2	Porcine stoolassociated circular virus 2	NA	Viruses; ssDNA viruses; unclassified ssDNA viruses
GCA_003963695.1	2502	2	Porcine stoolassociated circular virus 3	NA	Viruses; ssDNA viruses; unclassified ssDNA viruses
GCA_018580185.1	4619	3	Fox parvovirus	NA	Viruses; Monodnaviria; Shotokuvirae; Cossaviricota; Quintoviricetes; Piccovirales; Parvoviridae; Parvovirinae; Protoparvovirus; Carnivore protoparvovirus 4
GCA_000907355.1	2739	6	Ageratum Yellow vein China virus OX1	NA	Viruses; Monodnaviria; Shotokuvirae; Cressdnaviricota; Repensiviricetes; Geplafuvirales; Geminiviridae; Begomovirus; unclassified Begomovirus
GCA_000907295.1	2744	6	Tomato leaf curl China virus OX2	NA	Viruses; Monodnaviria; Shotokuvirae; Cressdnaviricota; Repensiviricetes; Geplafuvirales; Geminiviridae; Begomovirus; unclassified Begomovirus
GCA_002354985.1	176688	191	Eptesipox virus	Washington	Viruses; Varidnaviria; Bamfordvirae; Nucleocytoviricota; Pokkesviricetes; Chitovirales; Poxviridae; Chordopoxvirinae; Vespertilionpoxvirus
GCA_001564365.1	5203	4	Bat bocavirus	NA	Viruses; ssDNA viruses; Parvoviridae; Parvovirinae; Bocaparvovirus; unclassified Bocaparvovirus
GCA_002355085.1	2113	2	Bat circovirus	NA	Viruses; ssDNA viruses; Circoviridae; Circovirus; unclassified Circovirus
GCA_000906455.1	3788	3	Cryphonectria parasitica bipartite mycovirus 1	09269	Viruses; dsRNA viruses; unclassified dsRNA viruses
GCA_000917935.1	9739	1	Melegrivirus A	turkeyB407THV2011HUN	Viruses; Riboviria; Orthornavirae; Pisuviricota; Pisoniviricetes; Picornavirales; Picornaviridae; Megrivirus
GCA_000885595.1	7632	1	Cosavirus A	HCoSVA1	Viruses; ssRNA viruses; ssRNA positive-strand viruses, no DNA stage; Picornavirales; Picornaviridae; Cosavirus
GCA_000907315.1	7397	1	Enterovirus F	BEV261 M2 RM2	Viruses; ssRNA viruses; ssRNA positive-strand viruses, no DNA stage; Picornavirales; Picornaviridae; Enterovirus
GCA_000875085.1	7351	1	Enterovirus J	1631	Viruses; ssRNA viruses; ssRNA positive-strand viruses, no DNA stage; Picornavirales; Picornaviridae; Enterovirus

gca	genome.size	proteome.size	phage.name	strain	Taxonomy
GCA_000885035.1	7989	1	Salivirus A	NA	Viruses; ssRNA viruses; ssRNA positive-strand viruses, no DNA stage; Picornavirales; Picornaviridae; Salivirus
GCA_001465485.1	6888	6	Actinidia virus X	RV3124	Viruses; ssRNA viruses; ssRNA positive-strand viruses, no DNA stage; Tymovirales; Alphaflexiviridae; Potexvirus; unclassified Potexvirus
GCA_003369305.1	45477	56	Synechococcus T7like phage STIP37	NA	Viruses; Duplodnaviria; Heunggongvirae; Uroviricota; Caudoviricetes; Caudovirales; Autographiviridae; Igirivirus; Synechococcus virus STIP37
GCA_000928555.1	13191	12	Influenza A virus AShanghai022013H7N9	NA	Viruses; ssRNA viruses; ssRNA negative-strand viruses; Orthomyxoviridae; Influenzavirus A; Influenza A virus; H7N9 subtype
GCA_002816295.1	20795	3	Karang Sari virus	JKT10701	Viruses; Riboviria; Orthornavirae; Pisuviricota; Pisoniviricetes; Nidovirales; Mesnidovirineae; Mesoniviridae; Hexponivirinae; Alphamesonivirus; Karsalivirus; Alphamesonivirus 2
GCA_000909895.1	2742	7	Malvastrum leaf curl Philippines virus	NA	Viruses; ssDNA viruses; Geminiviridae; Begomovirus; unclassified Begomovirus
GCA_002603645.1	86537	96	Leptospira phage vB__LalZ__80412LE1	NA	Viruses; Duplodnaviria; Heunggongvirae; Uroviricota; Caudoviricetes; Caudovirales; Myoviridae; unclassified Myoviridae
GCA_002603665.1	87887	97	Leptospira phage vB__LnoZ__CZ214LE1	NA	Viruses; Duplodnaviria; Heunggongvirae; Uroviricota; Caudoviricetes; Caudovirales; Myoviridae; unclassified Myoviridae
GCA_002603685.1	47417	34	Leptospira phage vb__LkmZ__Bejolso9LE1	NA	Viruses; unclassified viruses; unclassified DNA viruses; unclassified dsDNA phages
GCA_002603705.1	87308	103	Leptospira phage vB__LbrZ__5399LE1	NA	Viruses; Duplodnaviria; Heunggongvirae; Uroviricota; Caudoviricetes; Caudovirales; Myoviridae; unclassified Myoviridae
GCA_002603725.1	89607	103	Leptospira phage vB__LinZ__10LE1	NA	Viruses; Duplodnaviria; Heunggongvirae; Uroviricota; Caudoviricetes; Caudovirales; Myoviridae; unclassified Myoviridae
GCA_000912555.1	258139	317	Pseudomonas phage PaBG	NA	Viruses; dsDNA viruses, no RNA stage; Caudovirales; Myoviridae; unclassified Myoviridae
GCA_002004435.1	7958	6	Eidolon helvum papillomavirus 2	NA	Viruses; dsDNA viruses, no RNA stage; Papillomaviridae; unclassified Papillomaviridae; non-primate mammal papillomaviruses
GCA_002004275.1	7991	6	Eidolon helvum papillomavirus 3	NA	Viruses; dsDNA viruses, no RNA stage; Papillomaviridae; unclassified Papillomaviridae; non-primate mammal papillomaviruses
GCA_000909155.1	19527	16	Phaeocystis globosa virus virophage	NA	Viruses; unclassified virophages
GCA_000908515.1	1224	1	Chili leaf curl Bhatinda betasatellite	NA	Viruses; Tolecusatellitidae; Betasatellite; unclassified Betasatellite
GCA_000909115.1	2754	6	Tomato yellow leaf curl Yunnan virus	NA	Viruses; ssDNA viruses; Geminiviridae; Begomovirus; unclassified Begomovirus
GCA_000910155.1	7583	8	Felis catus papillomavirus 3	NA	Viruses; dsDNA viruses, no RNA stage; Papillomaviridae; Taupapillomavirus; Taupapillomavirus 3
GCA_004089515.1	1759	6	Porcine circovirus 1	NA	Viruses; ssDNA viruses; Circoviridae; Circovirus
GCA_006445975.1	35080	41	Human adenovirus 66	87922	Viruses; Varidnaviria; Bamfordvirae; Preplasmiviricota; Tectiliviricetes; Rowavirales; Adenoviridae; Mastadenovirus; Human mastadenovirus B; unclassified Human adenovirus B
GCA_000910595.1	41294	68	Paenibacillus phage phiIBB__Pl23	NA	Viruses; dsDNA viruses, no RNA stage; Caudovirales; unclassified Caudovirales
GCA_000842245.1	4981	9	Budgerigar fledgling disease virus 1	NA	Viruses; Monodnaviria; Shotokuvirae; Cossaviricota; Papovaviricetes; Sepolyvirales; Polyomaviridae; Gammapolyomavirus; Aves polyomavirus 1
GCA_002826885.1	7533	7	Talpa europaea papillomavirus 1	NA	Viruses; Monodnaviria; Shotokuvirae; Cossaviricota; Papovaviricetes; Zurhausenvirales; Papillomaviridae; Firstpapillomavirinae; Dyophipapillomavirus; Dyophipapillomavirus 1
GCA_000910395.1	43447	69	Salmonella phage Jersey	NA	Viruses; dsDNA viruses, no RNA stage; Caudovirales; Siphoviridae; Guernseyvirinae; Jerseyvirus
GCA_000912515.1	59179	107	Mycobacterium phage Bobi	NA	Viruses; dsDNA viruses, no RNA stage; Caudovirales; Siphoviridae; unclassified Siphoviridae
GCA_000910855.1	108297	231	Mycobacterium phage Redno2	NA	Viruses; dsDNA viruses, no RNA stage; Caudovirales; Siphoviridae; unclassified Siphoviridae
GCA_002603745.1	52807	94	Mycobacterium phage Odin	NA	Viruses; Duplodnaviria; Heunggongvirae; Uroviricota; Caudoviricetes; Caudovirales; Siphoviridae; Fromanvirus; unclassified Fromanvirus
GCA_001516995.1	154434	236	Mycobacterium phage Breeniome	NA	Viruses; dsDNA viruses, no RNA stage; Caudovirales; Myoviridae; unclassified Myoviridae
GCA_000912495.1	109960	240	Mycobacterium phage Wanda	NA	Viruses; dsDNA viruses, no RNA stage; Caudovirales; Siphoviridae; unclassified Siphoviridae
GCA_000907795.1	8555	12	Vibrio phage VFJ	NA	Viruses; ssDNA viruses; Inoviridae; Inovirus; unclassified Inovirus
GCA_002823845.1	2619	4	Tomato bright yellow mosaic virus	NA	Viruses; Monodnaviria; Shotokuvirae; Cressdnaviricota; Repensiviricetes; Geplafuvirales; Geminiviridae; Begomovirus
GCA_001504375.1	87712	131	Escherichia phage JH2	NA	Viruses; dsDNA viruses, no RNA stage; Caudovirales; Myoviridae; unclassified Myoviridae
GCA_002622385.1	139928	200	Staphylococcus phage phiIBBSEP1	NA	Viruses; Duplodnaviria; Heunggongvirae; Uroviricota; Caudoviricetes; Caudovirales; Herelleviridae; Twortvirinae; Sepunavirus; Staphylococcus virus SEP1
GCA_004117615.1	17637	11	Bat rotavirus	NA	Viruses; Riboviria; Orthornavirae; Duplornaviricota; Resentoviricetes; Reovirales; Reoviridae; Sedoreovirinae; Rotavirus; unclassified Rotavirus

gca	genome.size	proteome.size	phage.name	strain	Taxonomy
GCA_002755815.1	132667	147	Campylobacter phage CP8	NA	Viruses; Duplodnaviria; Heunggongvirae; Uroviricota; Caudoviricetes; Caudovirales; Myoviridae; Eucampyvirinae; Fletchervirus; Campylobacter virus CP81
GCA_000918375.1	189311	221	Synechococcus phage ACG2014h	NA	Viruses; dsDNA viruses, no RNA stage; Caudovirales; Myoviridae; Tevenvirinae; T4virus; unclassified T4virus
GCA_002755835.1	176044	215	Synechococcus phage SMbCM25	NA	Viruses; Duplodnaviria; Heunggongvirae; Uroviricota; Caudoviricetes; Caudovirales; Myoviridae; Acionnavirus; unclassified Acionnavirus
GCA_000917315.1	170438	210	Synechococcus phage SMbCM100	NA	Viruses; dsDNA viruses, no RNA stage; Caudovirales; Myoviridae; Tevenvirinae; T4virus; unclassified T4virus
GCA_002755855.1	5486	11	Enterobacteria phage FL68 TallahasseeFL2012	NA	Viruses; Monodnaviria; Sangervirae; Phixviricota; Malgrandaviricetes; Petitvirales; Microviridae; Bullavirinae; Gequatrovirus; Escherichia virus Talmos
GCA_002755875.1	5486	11	Enterobacteria phage FL76 TallahasseeFL2012	NA	Viruses; Monodnaviria; Sangervirae; Phixviricota; Malgrandaviricetes; Petitvirales; Microviridae; Bullavirinae; Gequatrovirus; Escherichia virus Talmos
GCA_000912575.1	50690	87	Mycobacterium phage LittleCherry	NA	Viruses; dsDNA viruses, no RNA stage; Caudovirales; Siphoviridae; unclassified Siphoviridae
GCA_000913335.1	72108	128	Mycobacterium phage Catdawg	NA	Viruses; dsDNA viruses, no RNA stage; Caudovirales; Siphoviridae; unclassified Siphoviridae
GCA_000911835.1	55213	106	Mycobacterium phage Jabbawokkie	NA	Viruses; dsDNA viruses, no RNA stage; Caudovirales; Siphoviridae; unclassified Siphoviridae
GCA_000911875.1	54314	100	Mycobacterium phage Velveteen	NA	Viruses; dsDNA viruses, no RNA stage; Caudovirales; Siphoviridae; unclassified Siphoviridae
GCA_000910835.1	58043	102	Mycobacterium phage Daenerys	NA	Viruses; dsDNA viruses, no RNA stage; Caudovirales; Siphoviridae; unclassified Siphoviridae
GCA_000913295.1	57155	105	Mycobacterium phage Hamulus	NA	Viruses; dsDNA viruses, no RNA stage; Caudovirales; Siphoviridae; unclassified Siphoviridae
GCA_000911855.1	52102	94	Mycobacterium phage Trouble	NA	Viruses; dsDNA viruses, no RNA stage; Caudovirales; Siphoviridae; unclassified Siphoviridae
GCA_000910815.1	76050	128	Mycobacterium phage Whirlwind	NA	Viruses; dsDNA viruses, no RNA stage; Caudovirales; Siphoviridae; unclassified Siphoviridae
GCA_000912115.1	53701	99	Mycobacterium phage Sarfire	NA	Viruses; dsDNA viruses, no RNA stage; Caudovirales; Siphoviridae; unclassified Siphoviridae
GCA_002604045.1	70120	97	Mycobacterium phage Reprobate	NA	Viruses; Duplodnaviria; Heunggongvirae; Uroviricota; Caudoviricetes; Caudovirales; Siphoviridae; Bclasvirinae; Acadianvirus; Mycobacterium virus Reprobate
GCA_000913315.1	48228	71	Mycobacterium phage Muddy	NA	Viruses; dsDNA viruses, no RNA stage; Caudovirales; Siphoviridae; Ff47virus; Mycobacterium virus Muddy
GCA_000911815.1	70838	95	Mycobacterium phage KayaCho	NA	Viruses; dsDNA viruses, no RNA stage; Caudovirales; Siphoviridae; unclassified Siphoviridae
GCA_000913555.1	69815	121	Mycobacterium phage Dylan	NA	Viruses; dsDNA viruses, no RNA stage; Caudovirales; Siphoviridae; unclassified Siphoviridae
GCA_000912455.1	74533	140	Mycobacterium phage Contagion	NA	Viruses; dsDNA viruses, no RNA stage; Caudovirales; Siphoviridae; unclassified Siphoviridae
GCA_002597965.1	51384	87	Mycobacterium phage Medusa	NA	Viruses; Duplodnaviria; Heunggongvirae; Uroviricota; Caudoviricetes; Caudovirales; Siphoviridae; Fromanvirus; unclassified Fromanvirus
GCA_002598525.1	155714	235	Mycobacterium phage Shrimp	NA	Viruses; Duplodnaviria; Heunggongvirae; Uroviricota; Caudoviricetes; Caudovirales; Myoviridae; Bixzunavirus; Mycobacterium virus Bxz1
GCA_000910795.1	76129	133	Mycobacterium phage Crossroads	NA	Viruses; dsDNA viruses, no RNA stage; Caudovirales; Siphoviridae; unclassified Siphoviridae
GCA_000910875.1	3780	3	Parvovirus NIHCQV	NA	Viruses; ssDNA viruses; Parvoviridae; Parvovirinae; unclassified Parvovirinae
GCA_000912935.1	34145	43	Vibrio phage VPUSM 8	NA	Viruses; dsDNA viruses, no RNA stage; Caudovirales; Myoviridae; unclassified Myoviridae
GCA_000910175.1	1060	2	Grapevine satellite virus	NA	Viruses; Satellites; Satellite Viruses; Tobacco necrosis satellite virus-like
GCA_000908815.1	7069	10	Stenotrophomonas phage phiSMA7	NA	Viruses; ssDNA viruses; Inoviridae; unclassified Inoviridae
GCA_000845565.1	1351	1	Cotton leaf curl betasatellite	NA	Viruses; Tolecusatellitidae; Betasatellite
GCA_000921855.1	149459	70	Fruit bat alphaherpesvirus 1	NA	Viruses; dsDNA viruses, no RNA stage; Herpesvirales; Herpesviridae; Alphaherpesvirinae; Simplexvirus; unclassified Simplexvirus
GCA_000916795.1	7315	4	Apricot vein clearing associated virus	NA	Viruses; ssRNA viruses; ssRNA positive-strand viruses, no DNA stage; Tymovirales; Betaflexiviridae; Trivirinae; Prunevirus
GCA_004787415.1	120023	134	Lonomia obliqua multiple nucleopolyhedrovirus	NA	Viruses; Baculoviridae; Alphabaculovirus; Lonomia obliqua nucleopolyhedrovirus
GCA_000912275.1	10692	5	American bat vesiculovirus TFFN2013	NA	Viruses; ssRNA viruses; ssRNA negative-strand viruses; Mononegavirales; Rhabdoviridae; Vesiculovirus; unclassified Vesiculovirus
GCA_000852445.1	13652	13	Pseudomonas phage phi13	NA	Viruses; dsRNA viruses; Cystoviridae; unclassified Cystoviridae
GCA_000908835.1	1832	2	Human cyclovirus VS5700009	NA	Viruses; ssDNA viruses; Circoviridae; unclassified Circoviridae; suggested genus Cyclovirus

gca	genome.size	proteome.size	phage.name	strain	Taxonomy
GCA_000909415.1	10985	1	Nilaparvata lugens honeydew virus2	NA	Viruses; ssRNA viruses; ssRNA positive-strand viruses, no DNA stage; Picornavirales; Iflaviridae; unclassified Iflaviridae
GCA_000910275.1	10600	1	Nilaparvata lugens honeydew virus3	NA	Viruses; ssRNA viruses; ssRNA positive-strand viruses, no DNA stage; Picornavirales; Iflaviridae; unclassified Iflaviridae
GCA_000839705.1	25120	1	Trichoplusia ni cypovirus 15	NA	Viruses; dsRNA viruses; Reoviridae; Spinareovirinae; Cypovirus; Cypovirus 15
GCA_000850385.1	7830	4	American plum line pattern virus	NA	Viruses; ssRNA viruses; ssRNA positive-strand viruses, no DNA stage; Bromoviridae; Ilarvirus; Ilarvirus subgroup 5
GCA_000837305.1	5232	6	Cucurbit leaf crumple virus	NA	Viruses; ssDNA viruses; Geminiviridae; Begomovirus
GCA_000919735.1	2526	4	Circolike virusBrazil hs1	NA	Viruses; ssDNA viruses; unclassified ssDNA viruses; Circo-like virus-Brazil
GCA_003985525.1	2533	4	Circolike virusBrazil hs2	NA	Viruses; unclassified viruses; unclassified DNA viruses; unclassified ssDNA viruses; Circo-like virus-Brazil
GCA_000923335.1	151109	139	Peridroma alphabaculovirus	NA	Viruses; dsDNA viruses, no RNA stage; Baculoviridae; Alphabaculovirus; unclassified Alphabaculovirus
GCA_002625225.1	7648	10	Stenotrophomonas phage SMA6	NA	Viruses; Monodnaviria; Loebvirae; Hofneiviricota; Faserviricetes; Tubulavirales; Inoviridae; Scuticavirus; Stenotrophomonas virus SMA6
GCA_000908995.1	42902	58	Staphylococcus phage SA12	NA	Viruses; dsDNA viruses, no RNA stage; Caudovirales; Siphoviridae; unclassified Siphoviridae
GCA_003178895.1	7359	7	Human papillomavirus 174	NA	Viruses; Monodnaviria; Shotokuvirae; Cossaviricota; Papovaviricetes; Zurhausenvirales; Papillomaviridae; Firstpapillomavirinae; Betapapillomavirus; Betapapillomavirus 2
GCA_002630305.1	166007	274	Enterobacteria phage RB33	NA	Viruses; Duplodnaviria; Heunggongvirae; Uroviricota; Caudoviricetes; Caudovirales; Myoviridae; Tevenvirinae; Tequatrovirus; unclassified Tequatrovirus
GCA_000926595.1	11124	5	Niakha virus	NA	Viruses; ssRNA viruses; ssRNA negative-strand viruses; Mononegavirales; Rhabdoviridae; unclassified Rhabdoviridae
GCA_000923835.1	1855	2	Cyclovirus VN	NA	Viruses; ssDNA viruses; Circoviridae; unclassified Circoviridae; suggested genus Cyclovirus
GCA_000913255.1	92495	176	Pseudomonas phage PAK_P2	NA	Viruses; dsDNA viruses, no RNA stage; Caudovirales; Myoviridae; unclassified Myoviridae
GCA_002662535.1	18613	11	Rotavirus A humanwtUSALB15622010G9P4	NA	Viruses; Riboviria; Orthornavirae; Duplornaviricota; Resentoviricetes; Reovirales; Reoviridae; Sedoreovirinae; Rotavirus; Rotavirus A; Rotavirus G9
GCA_000911655.1	1908524	1070	Pandoravirus dulcis	NA	Viruses; unclassified viruses; unclassified DNA viruses; unclassified dsDNA viruses; Pandoravirus
GCA_000911955.1	2473870	1430	Pandoravirus salinus	NA	Viruses; unclassified viruses; unclassified DNA viruses; unclassified dsDNA viruses; Pandoravirus
GCA_002146085.1	7299	3	Goose astrovirus	NA	Viruses; ssRNA viruses; ssRNA positive-strand viruses, no DNA stage; Astroviridae; Avastrovirus; unclassified Avastrovirus
GCA_000917075.1	48775	51	Sulfolobus monocaudavirus SMV1	NA	Viruses; dsDNA viruses, no RNA stage; unclassified dsDNA viruses
GCA_002623265.1	57081	98	Enterococcus phage IMEEF1	NA	Viruses; Duplodnaviria; Heunggongvirae; Uroviricota; Caudoviricetes; Caudovirales; Siphoviridae; Saphexavirus; Enterococcus virus IMEEF1
GCA_001042115.1	167785	271	Yersinia phage PST	NA	Viruses; dsDNA viruses, no RNA stage; Caudovirales; Myoviridae; Tevenvirinae; T4virus; Yersinia virus PST
GCA_000908095.1	8576	3	Ralstonia phage RSS30	NA	Viruses; ssDNA viruses; Inoviridae; Inovirus; unclassified Inovirus
GCA_000909595.1	38099	60	Thermus phage phi OH2	NA	Viruses; dsDNA viruses, no RNA stage; Caudovirales; unclassified Caudovirales
GCA_000914255.1	7435	5	Castor canadensis papillomavirus 1	NA	Viruses; dsDNA viruses, no RNA stage; Papillomaviridae; Dyosigmapapillomavirus; Dyosigmapapillomavirus 1
GCA_000910355.1	4195	6	Thin paspalum asymptomatic virus	2005	Viruses; ssRNA viruses; ssRNA positive-strand viruses, no DNA stage; Tombusviridae; Panicovirus
GCA_000907855.1	10770	1	Sclerotinia sclerotiorum endornavirus1	JZJL2	Viruses; dsRNA viruses; Endornaviridae; Endornavirus; unclassified Endornavirus
GCA_000919715.1	2925	2	Fur seal faeces associated circular DNA virus	NA	Viruses; ssDNA viruses; Circoviridae; unclassified Circoviridae
GCA_000926255.2	8021	2	Leptopilina boulardi Totilike virus	NSref	Viruses; dsRNA viruses; Totiviridae; unclassified Totiviridae
GCA_001885465.1	10471	1	Guereza hepacivirus	NA	Viruses; ssRNA viruses; ssRNA positive-strand viruses, no DNA stage; Flaviviridae; Hepacivirus; unclassified Hepacivirus
GCA_000911075.1	70236	98	Mycobacterium phage Adawi	NA	Viruses; dsDNA viruses, no RNA stage; Caudovirales; Siphoviridae; unclassified Siphoviridae
GCA_000912715.2	69309	94	Mycobacterium phage Bane1	NA	Viruses; dsDNA viruses, no RNA stage; Caudovirales; Siphoviridae; unclassified Siphoviridae
GCA_002603825.1	69306	94	Mycobacterium phage Bane2	NA	Viruses; Duplodnaviria; Heunggongvirae; Uroviricota; Caudoviricetes; Caudovirales; Siphoviridae; Bclasvirinae; Coopervirus; Mycobacterium virus Bane1
GCA_000913775.1	52282	103	Mycobacterium phage Fredward	NA	Viruses; dsDNA viruses, no RNA stage; Caudovirales; Siphoviridae; unclassified Siphoviridae

gca	genome.size	proteome.size	phage.name	strain	Taxonomy
GCA_001505835.2	50854	93	Mycobacterium phage Anubis	NA	Viruses; dsDNA viruses, no RNA stage; Caudovirales; Siphoviridae; L5virus; unclassified L5likevirus
GCA_000912695.1	53636	97	Mycobacterium phage PhrostyMug	NA	Viruses; dsDNA viruses, no RNA stage; Caudovirales; Siphoviridae; unclassified Siphoviridae
GCA_002603845.1	53693	100	Mycobacterium phage SargentShorty9	NA	Viruses; Duplodnaviria; Heunggongvirae; Uroviricota; Caudoviricetes; Caudovirales; Siphoviridae; Fromanvirus; unclassified Fromanvirus
GCA_000911035.1	76586	151	Mycobacterium phage Quink	NA	Viruses; dsDNA viruses, no RNA stage; Caudovirales; Siphoviridae; unclassified Siphoviridae
GCA_011604345.1	43840	37	Fowl adenovirus	NA	Viruses; Varidnaviria; Bamfordvirae; Preplasmiviricota; Tectiliviricetes; Rowavirales; Adenoviridae; Aviadenovirus; unclassified Aviadenovirus
GCA_000925555.1	15396	9	Porcine respirovirus 1	S206N	Viruses; Riboviria; Orthornavirae; Negarnaviricota; Haploviricotina; Monjiviricetes; Mononegavirales; Paramyxoviridae; Orthoparamyxovirinae; Respirovirus
GCA_001023565.1	194701	377	Sinorhizobium phage phiM12	NA	Viruses; Duplodnaviria; Heunggongvirae; Uroviricota; Caudoviricetes; Caudovirales; Myoviridae; Emdodecavirus; Sinorhizobium virus M12
GCA_000910715.1	77367	144	Mycobacterium phage DrDrey	NA	Viruses; dsDNA viruses, no RNA stage; Caudovirales; Siphoviridae; unclassified Siphoviridae
GCA_001041895.1	41268	46	Proteus phage PM16	NA	Viruses; dsDNA viruses, no RNA stage; Caudovirales; Podoviridae; unclassified Podoviridae
GCA_002603985.1	43278	66	Bacillus phage proCM3	NA	Viruses; Duplodnaviria; Heunggongvirae; Uroviricota; Caudoviricetes; Caudovirales; Siphoviridae; Lwoffvirus; unclassified Lwoffvirus
GCA_000916355.1	38772	56	Bacillus phage phiCM3	NA	Viruses; dsDNA viruses, no RNA stage; Caudovirales; Siphoviridae; unclassified Siphoviridae
GCA_000927355.1	155997	286	Caulobacter phage Cr30	NA	Viruses; dsDNA viruses, no RNA stage; Caudovirales; Myoviridae; unclassified Myoviridae
GCA_001505175.1	169223	170	Microcystis phage MaMVDC	NA	Viruses; dsDNA viruses, no RNA stage; Caudovirales; Myoviridae; unclassified Myoviridae
GCA_000923615.1	41750	38	Anabaena phage A4L	NA	Viruses; dsDNA viruses, no RNA stage; Caudovirales; Podoviridae; unclassified Podoviridae
GCA_000912255.1	33628	40	Enterobacteria phage fiAA91ss	NA	Viruses; dsDNA viruses, no RNA stage; Caudovirales; Myoviridae; Peduovirinae; P2virus; unclassified P2likevirus
GCA_002755895.1	76131	143	Mycobacterium phage ABCat	NA	Viruses; Duplodnaviria; Heunggongvirae; Uroviricota; Caudoviricetes; Caudovirales; Siphoviridae; Kostyavirus; unclassified Kostyavirus
GCA_002219685.1	12886	3	Lettuce necrotic leaf curl virus	NA	Viruses; ssRNA viruses; ssRNA positive-strand viruses, no DNA stage; Picornavirales; Secoviridae; Torradovirus
GCA_000843225.1	39898	32	Roseobacter virus SIO1	NA	Viruses; Duplodnaviria; Heunggongvirae; Uroviricota; Caudoviricetes; Caudovirales; Podoviridae
GCA_002604005.1	39940	62	Pseudoalteromonas phage TW1	NA	Viruses; Duplodnaviria; Heunggongvirae; Uroviricota; Caudoviricetes; Caudovirales; Siphoviridae; unclassified Siphoviridae
GCA_002823205.1	2739	6	Pouzolzia mosaic Guangdong virus	NA	Viruses; Monodnaviria; Shotokuvirae; Cressdnaviricota; Repensiviricetes; Geplafuvirales; Geminiviridae; Begomovirus
GCA_000909795.1	140633	137	Hemileuca sp nucleopolyhedrovirus	NA	Viruses; Baculoviridae; Alphabaculovirus; Hemileuca species nucleopolyhedrovirus
GCA_002825665.1	2200	5	Hypericum japonicum associated circular DNA virus	NA	Viruses; Monodnaviria; Shotokuvirae; Cressdnaviricota; Repensiviricetes; Geplafuvirales; Genomoviridae; Gemycircularvirus; Hypericum associated gemycircularvirus 1
GCA_000909835.1	13242	4	Brazoran virus	NA	Viruses; ssRNA viruses; ssRNA negative-strand viruses; Bunyaviridae; Orthobunyavirus; unclassified Orthobunyavirus
GCA_000911755.1	7267	7	Rangifer tarandus papillomavirus 2	NA	Viruses; dsDNA viruses, no RNA stage; Papillomaviridae; Xipapillomavirus; Xipapillomavirus 3
GCA_001561005.1	140122	76	Macropodid alphaherpesvirus 1	NA	Viruses; dsDNA viruses, no RNA stage; Herpesvirales; Herpesviridae; Alphaherpesvirinae; Simplexvirus
GCA_000858065.1	9751	1	Cucumber vein yellowing virus	ALM32	Viruses; ssRNA viruses; ssRNA positive-strand viruses, no DNA stage; Potyviridae; Ipomovirus
GCA_013363035.1	73623	81	Leptospira phage LE1	NA	Viruses; Duplodnaviria; Heunggongvirae; Uroviricota; Caudoviricetes; Caudovirales; Myoviridae; unclassified Myoviridae
GCA_000865385.1	4094	5	Maize necrotic streak virus	NA	Viruses; ssRNA viruses; ssRNA positive-strand viruses, no DNA stage; Tombusviridae; Zeavirus
GCA_000884835.1	8995	1	Cassava brown streak virus	NA	Viruses; ssRNA viruses; ssRNA positive-strand viruses, no DNA stage; Potyviridae; Ipomovirus
GCA_008777255.1	9192	1	Pigeon mesivirus 2	pigeonGALII5PiMeV2011HUN	Viruses; Riboviria; Orthornavirae; Pisuviricota; Pisoniviricetes; Picornavirales; Picornaviridae; unclassified Picornaviridae
GCA_008776855.1	8219	1	Feline kobuvirus	FK13	Viruses; Riboviria; Orthornavirae; Pisuviricota; Pisoniviricetes; Picornavirales; Picornaviridae; Kobuvirus; Aichivirus A
GCA_000927715.1	4023	4	Circoviridae 1 LDMD2013	NA	Viruses; ssDNA viruses; Circoviridae; environmental samples
GCA_000928715.1	5749	4	Bacillariodnavirus LDMD2013	NA	Viruses; unassigned viruses; Bacilladnavirus; environmental samples
GCA_000930395.1	3584	2	Circoviridae 2 LDMD2013	NA	Viruses; ssDNA viruses; Circoviridae; environmental samples

gca	genome.size	proteome.size	phage.name	strain	Taxonomy
GCA_000929755.1	2389	2	Circoviridae 3 LDMD2013	NA	Viruses; ssDNA viruses; Circoviridae; environmental samples
GCA_000927835.1	3393	3	Circoviridae 4 LDMD2013	NA	Viruses; ssDNA viruses; Circoviridae; environmental samples; Circoviridae 17 LDMD-2013
GCA_000927755.1	2603	3	Circoviridae 5 LDMD2013	NA	Viruses; ssDNA viruses; Circoviridae; environmental samples; Circoviridae LDMD-2013a
GCA_000928735.1	2711	3	Circoviridae 6 LDMD2013	NA	Viruses; ssDNA viruses; Circoviridae; environmental samples
GCA_000927815.1	4715	5	Circoviridae 7 LDMD2013	NA	Viruses; ssDNA viruses; Circoviridae; environmental samples
GCA_000930375.1	3999	5	Circoviridae 8 LDMD2013	NA	Viruses; ssDNA viruses; Circoviridae; environmental samples
GCA_000929735.1	3175	3	Circoviridae 9 LDMD2013	NA	Viruses; ssDNA viruses; Circoviridae; environmental samples
GCA_000928695.1	3333	3	Circoviridae 10 LDMD2013	NA	Viruses; ssDNA viruses; Circoviridae; environmental samples
GCA_000927795.1	2917	5	Circoviridae 11 LDMD2013	NA	Viruses; ssDNA viruses; Circoviridae; environmental samples
GCA_003986385.1	2827	3	Circoviridae 12 LDMD2013	NA	Viruses; Monodnaviria; Shotokuvirae; Cressdnaviricota; Arfiviricetes; Cirlivirales; Circoviridae; environmental samples; Circoviridae LDMD-2013b
GCA_000930355.1	2658	2	Circoviridae 13 LDMD2013	NA	Viruses; ssDNA viruses; Circoviridae; environmental samples
GCA_000929715.1	3161	3	Circoviridae 14 LDMD2013	NA	Viruses; ssDNA viruses; Circoviridae; environmental samples
GCA_000928675.1	2676	3	Circoviridae 15 LDMD2013	NA	Viruses; ssDNA viruses; Circoviridae; environmental samples
GCA_000927775.1	2262	4	Circoviridae 16 LDMD2013	NA	Viruses; ssDNA viruses; Circoviridae; environmental samples
GCA_000930335.1	3393	3	Circoviridae 17 LDMD2013	NA	Viruses; Monodnaviria; Shotokuvirae; Cressdnaviricota; Arfiviricetes; Cirlivirales; Circoviridae; environmental samples
GCA_000929775.1	2834	2	Circoviridae 18 LDMD2013	NA	Viruses; ssDNA viruses; Circoviridae; environmental samples; Circoviridae LDMD-2013b
GCA_000929695.1	1064	1	Circoviridae 19 LDMD2013	NA	Viruses; ssDNA viruses; Circoviridae; environmental samples
GCA_003986405.1	2604	4	Circoviridae 20 LDMD2013	NA	Viruses; Monodnaviria; Shotokuvirae; Cressdnaviricota; Arfiviricetes; Cirlivirales; Circoviridae; environmental samples; Circoviridae LDMD-2013a
GCA_000928655.1	2296	3	Circoviridae 21 LDMD2013	NA	Viruses; ssDNA viruses; Circoviridae; environmental samples
GCA_000924715.1	4089	3	Boiling Springs Lake RNADNA hybrid virus	NA	Viruses; environmental samples
GCA_006449655.1	106890	109	Rana catesbeiana virus	NA	Viruses; Varidnaviria; Bamfordvirae; Nucleocytoviricota; Megaviricetes; Pimascovirales; Iridoviridae; Alphairidovirinae; Ranavirus; unclassified Ranavirus
GCA_003986365.1	1955	2	Farfantepenaeus duorarum circovirus	NA	Viruses; Monodnaviria; Shotokuvirae; Cressdnaviricota; Arfiviricetes; Cirlivirales; Circoviridae; Circovirus; unclassified Circovirus
GCA_000909815.1	3206	6	Grapevine redblotch associated virus	NA	Viruses; ssDNA viruses; Geminiviridae; unclassified Geminiviridae
GCA_000927995.1	18368	3	Leopards Hill virus	11SB17	Viruses; ssRNA viruses; ssRNA negative-strand viruses; Bunyaviridae; Nairovirus; unclassified Nairovirus
GCA_000913355.1	18643	11	Blackberry vein bandingassociated virus	NA	Viruses; Riboviria; Orthornavirae; Kitrinoviricota; Alsuviricetes; Martellivirales; Closteroviridae; Ampelovirus
GCA_000923715.1	137090	70	Alcelaphine gammaherpesvirus 2	NA	Viruses; dsDNA viruses, no RNA stage; Herpesvirales; Herpesviridae; Gammaherpesvirinae; Macavirus
GCA_000918855.1	10334	4	University of Helsinki virus	UHV1	Viruses; ssRNA viruses; ssRNA negative-strand viruses; Arenaviridae; Reptarenavirus; Alethinophid 2 reptarenavirus
GCA_000915435.1	24041	10	Inachis io cypovirus 2	NA	Viruses; dsRNA viruses; Reoviridae; Spinareovirinae; Cypovirus; Cypovirus 2
GCA_000913375.1	163019	289	Bacillus phage Troll	NA	Viruses; dsDNA viruses, no RNA stage; Caudovirales; Myoviridae; B4virus; Bacillus virus Troll
GCA_000916915.1	5552	3	Chaetoceros sp DNA virus 7	NA	Viruses; Monodnaviria; Shotokuvirae; Cressdnaviricota; Arfiviricetes; Baphyvirales; Bacilladnaviridae; Protobacilladnavirus; Chaetoceros protobacilladnavirus 2
GCA_000911895.1	2825	7	Hemidesmus yellow mosaic virus	NA	Viruses; ssDNA viruses; Geminiviridae; Begomovirus; unclassified Begomovirus
GCA_000909855.1	53588	96	Mycobacterium phage Wheeler	NA	Viruses; dsDNA viruses, no RNA stage; Caudovirales; Siphoviridae; unclassified Siphoviridae
GCA_000913275.1	70115	95	Mycobacterium phage Phelemich	NA	Viruses; dsDNA viruses, no RNA stage; Caudovirales; Siphoviridae; unclassified Siphoviridae
GCA_000911795.1	70657	100	Mycobacterium phage Papyrus	NA	Viruses; dsDNA viruses, no RNA stage; Caudovirales; Siphoviridae; unclassified Siphoviridae

gca	genome.size	proteome.size	phage.name	strain	Taxonomy
GCA_000913395.1	76483	143	Mycobacterium phage Goku	NA	Viruses; dsDNA viruses, no RNA stage; Caudovirales; Siphoviridae; unclassified Siphoviridae
GCA_000910775.1	52519	96	Mycobacterium phage Adzzy	NA	Viruses; dsDNA viruses, no RNA stage; Caudovirales; Siphoviridae; unclassified Siphoviridae
GCA_018594955.1	12532	4	Sedlec virus	Av 172	Viruses; Riboviria; Orthornavirae; Negarnaviricota; Polyploviricotina; Ellioviricetes; Bunyavirales; Peribunyaviridae; Orthobunyavirus; Sedlec orthobunyavirus
GCA_000913415.1	28035	7	Bat coronavirus CDPHE15USA2006	batUSACDPHE152006	Viruses; ssRNA viruses; ssRNA positive-strand viruses, no DNA stage; Nidovirales; Coronaviridae; Coronavirinae; Alphacoronavirus; Bat coronavirus CDPHE15
GCA_000914215.1	2760	6	Tomato leaf curl Gandhinagar virus	NA	Viruses; ssDNA viruses; Geminiviridae; Begomovirus; unclassified Begomovirus
GCA_000915235.1	1365	1	Tomato leaf curl Gandhinagar betasatellite	NA	Viruses; Tolecusatellitidae; Betasatellite
GCA_000911295.1	41757	52	Streptococcus phage TP778L	NA	Viruses; dsDNA viruses, no RNA stage; Caudovirales; Siphoviridae; unclassified Siphoviridae
GCA_000912235.1	30175	12	Betacoronavirus ErinaceusVMCDEU2012	NA	Viruses; ssRNA viruses; ssRNA positive-strand viruses, no DNA stage; Nidovirales; Coronaviridae; Coronavirinae; Betacoronavirus; unclassified Betacoronavirus
GCA_002921725.1	4200	11	Marine gokushovirus	NA	Viruses; ssDNA viruses; Microviridae; Gokushovirinae; unclassified Gokushovirinae
GCA_000912595.1	8577	5	Ageratum latent virus	NA	Viruses; ssRNA viruses; ssRNA positive-strand viruses, no DNA stage; Bromoviridae; Ilarvirus; unclassified Ilarvirus
GCA_008800495.1	7017	1	Porcine teschovirus 2	T80	Viruses; Riboviria; Orthornavirae; Pisuviricota; Pisoniviricetes; Picornavirales; Picornaviridae; Teschovirus; Teschovirus A
GCA_008800475.1	7012	1	Porcine teschovirus 3	O 2b	Viruses; Riboviria; Orthornavirae; Pisuviricota; Pisoniviricetes; Picornavirales; Picornaviridae; Teschovirus; Teschovirus A
GCA_008800455.1	7014	1	Porcine teschovirus 4	PS 36	Viruses; Riboviria; Orthornavirae; Pisuviricota; Pisoniviricetes; Picornavirales; Picornaviridae; Teschovirus; Teschovirus A
GCA_008800435.1	7008	1	Porcine teschovirus 5	F 26	Viruses; Riboviria; Orthornavirae; Pisuviricota; Pisoniviricetes; Picornavirales; Picornaviridae; Teschovirus; Teschovirus A
GCA_008800415.1	7018	1	Porcine teschovirus 6	PS 37	Viruses; Riboviria; Orthornavirae; Pisuviricota; Pisoniviricetes; Picornavirales; Picornaviridae; Teschovirus; Teschovirus A
GCA_008800395.1	7014	1	Porcine teschovirus 7	F 43	Viruses; Riboviria; Orthornavirae; Pisuviricota; Pisoniviricetes; Picornavirales; Picornaviridae; Teschovirus; Teschovirus A
GCA_004787035.2	5084	7	Pepper leafroll virus	NA	Viruses; Monodnaviria; Shotokuvirae; Cressdnaviricota; Repensiviricetes; Geplafuvirales; Geminiviridae; Begomovirus
GCA_002378515.1	4528	2	Heterobasidion partitivirus 7	NA	Viruses; Riboviria; Orthornavirae; Pisuviricota; Duplopiviricetes; Durnavirales; Partitiviridae; Betapartitivirus
GCA_009731695.1	12184	3	Iquitos virus	NA	Viruses; Riboviria; Orthornavirae; Negarnaviricota; Polyploviricotina; Ellioviricetes; Bunyavirales; Peribunyaviridae; Orthobunyavirus; Oropouche orthobunyavirus
GCA_000929895.1	23325	10	Cangyuan orthoreovirus	NA	Viruses; dsRNA viruses; Reoviridae; Spinareovirinae; Orthoreovirus; Pteropine orthoreovirus
GCA_000910975.1	6795	3	Feline astrovirus 2	1637F	Viruses; ssRNA viruses; ssRNA positive-strand viruses, no DNA stage; Astroviridae; Mamastrovirus; unclassified Mamastrovirus
GCA_000883255.1	7890	6	Potato latent virus	NA	Viruses; ssRNA viruses; ssRNA positive-strand viruses, no DNA stage; Tymovirales; Betaflexiviridae; Quinvirinae; Carlavirus
GCA_000930475.1	2229	2	Faeces associated gemycircularvirus 10	NA	Viruses; unclassified viruses; Genomoviridae; Gemycircularvirus; unclassified Gemycircularvirus
GCA_000929835.1	2159	2	Faeces associated gemycircularvirus 11	NA	Viruses; unclassified viruses; Genomoviridae; Gemycircularvirus; unclassified Gemycircularvirus
GCA_000928795.1	2122	2	Faeces associated gemycircularvirus 12	NA	Viruses; unclassified viruses; Genomoviridae; Gemycircularvirus; unclassified Gemycircularvirus
GCA_000930455.1	2160	2	Faecalassociated gemycircularvirus 1b	NA	Viruses; Monodnaviria; Shotokuvirae; Cressdnaviricota; Repensiviricetes; Geplafuvirales; Genomoviridae; Gemycircularvirus; unclassified Gemycircularvirus; Faeces associated gemycircularvirus 1
GCA_000929815.1	2185	2	Faecalassociated gemycircularvirus 1c	NA	Viruses; Monodnaviria; Shotokuvirae; Cressdnaviricota; Repensiviricetes; Geplafuvirales; Genomoviridae; Gemycircularvirus; Blackbird associated gemycircularvirus 1
GCA_000928775.1	2175	2	Faeces associated gemycircularvirus 2	NA	Viruses; unclassified viruses; Genomoviridae; Gemycircularvirus; unclassified Gemycircularvirus
GCA_000927875.1	2199	2	Faeces associated gemycircularvirus 3	NA	Viruses; unclassified viruses; Genomoviridae; Gemycircularvirus; unclassified Gemycircularvirus
GCA_003849225.1	2224	3	Faeces associated gemycircularvirus 4	NA	Viruses; unclassified viruses; Genomoviridae; Gemycircularvirus; unclassified Gemycircularvirus
GCA_000929795.1	2187	2	Faeces associated gemycircularvirus 5	NA	Viruses; unclassified viruses; Genomoviridae; Gemycircularvirus; unclassified Gemycircularvirus
GCA_000928755.1	2288	2	Faeces associated gemycircularvirus 6	NA	Viruses; unclassified viruses; Genomoviridae; Gemycircularvirus; unclassified Gemycircularvirus
GCA_000927855.1	2290	2	Faeces associated gemycircularvirus 7	NA	Viruses; unclassified viruses; Genomoviridae; Gemycircularvirus; unclassified Gemycircularvirus
GCA_000928815.1	2174	2	Faecesassociated gemycircularvirus 8	NA	Viruses; unclassified viruses; Genomoviridae; Gemycircularvirus; unclassified Gemycircularvirus

gca	genome.size	proteome.size	phage.name	strain	Taxonomy
GCA_000927915.1	2130	2	Faeces associated gemycircularvirus 9	NA	Viruses; unclassified viruses; Genomoviridae; Gemycircularvirus; unclassified Gemycircularvirus
GCA_000911575.1	74210	141	Mycobacterium phage Bruin	NA	Viruses; dsDNA viruses, no RNA stage; Caudovirales; Siphoviridae; unclassified Siphoviridae
GCA_000914075.1	75894	147	Mycobacterium phage Nala	NA	Viruses; dsDNA viruses, no RNA stage; Caudovirales; Siphoviridae; unclassified Siphoviridae
GCA_000913235.1	76217	148	Mycobacterium phage PhatBacter	NA	Viruses; dsDNA viruses, no RNA stage; Caudovirales; Siphoviridae; unclassified Siphoviridae
GCA_000926735.1	138658	221	Bacillus phage CP51	NA	Viruses; dsDNA viruses, no RNA stage; Caudovirales; Myoviridae; Cp51virus; Bacillus virus CP51
GCA_000922475.1	36838	50	Pseudomonas phage MP48	NA	Viruses; dsDNA viruses, no RNA stage; Caudovirales; Siphoviridae; Lambdavirus; unclassified Lambda-like viruses
GCA_000924215.1	71778	83	Escherichia phage vB_EcoP_PhAPEC7	NA	Viruses; dsDNA viruses, no RNA stage; Caudovirales; Podoviridae; Picovirinae; G7cvirus; Escherichia virus APEC7
GCA_000926115.1	167318	254	Escherichia phage vB_EcoM_PhAPEC2	NA	Viruses; dsDNA viruses, no RNA stage; Caudovirales; Myoviridae; Tevenvirinae; T4virus; unclassified T4virus
GCA_000915095.1	148567	271	Escherichia phage 4MG	NA	Viruses; dsDNA viruses, no RNA stage; Caudovirales; Myoviridae; Vequintavirinae; Se1virus; Escherichia virus 4MG
GCA_000915115.1	52344	102	Mycobacterium phage Artemis2UCLA	NA	Viruses; dsDNA viruses, no RNA stage; Caudovirales; Siphoviridae; unclassified Siphoviridae; Mycobacterium phage Zaka
GCA_000915075.1	52873	103	Mycobacterium phage CloudWang3	NA	Viruses; dsDNA viruses, no RNA stage; Caudovirales; Siphoviridae; unclassified Siphoviridae; Mycobacterium phage Zaka
GCA_000913215.1	50755	86	Mycobacterium phage Conspiracy	NA	Viruses; dsDNA viruses, no RNA stage; Caudovirales; Siphoviridae; unclassified Siphoviridae
GCA_000914115.1	52823	97	Mycobacterium phage Graduation	NA	Viruses; dsDNA viruses, no RNA stage; Caudovirales; Siphoviridae; unclassified Siphoviridae
GCA_000914035.1	52122	101	Mycobacterium phage Zaka	NA	Viruses; dsDNA viruses, no RNA stage; Caudovirales; Siphoviridae; unclassified Siphoviridae
GCA_002149665.1	45532	62	Phormidium phage MISPhV1A	NA	Viruses; dsDNA viruses, no RNA stage; unclassified dsDNA phages
GCA_002149645.1	41291	57	Phormidium phage MISPhV1B	NA	Viruses; dsDNA viruses, no RNA stage; unclassified dsDNA phages
GCA_000914675.1	43017	54	Edwardsiella phage eiAU183	NA	Viruses; dsDNA viruses, no RNA stage; Caudovirales; Myoviridae; unclassified Myoviridae; Edwardsiella phage eiAU
GCA_000924255.1	4733	4	Bufavirus3	BTN63	Viruses; ssDNA viruses; Parvoviridae; Parvovirinae; unclassified Parvovirinae
GCA_000911995.1	6398	4	Tomato mottle mosaic virus	NA	Viruses; ssRNA viruses; ssRNA positive-strand viruses, no DNA stage; Virgaviridae; Tobamovirus; unclassified Tobamovirus
GCA_000910955.1	2775	7	Tomato yellow leaf curl Saudi virus	NA	Viruses; ssDNA viruses; Geminiviridae; Begomovirus; unclassified Begomovirus
GCA_000916975.1	48800	68	Streptococcus phage 20617	NA	Viruses; unclassified phages
GCA_000913435.1	2834	6	Sweet potato leaf curl Shanghai virus	NA	Viruses; ssDNA viruses; Geminiviridae; Begomovirus; unclassified Begomovirus
GCA_000862725.1	15186	6	Newcastle disease virus B1	NA	Viruses; ssRNA viruses; ssRNA negative-strand viruses; Mononegavirales; Paramyxoviridae; Avulavirus; Newcastle disease virus
GCA_000914155.1	5084	6	Butcherbird polyomavirus	NA	Viruses; dsDNA viruses, no RNA stage; Polyomaviridae; unclassified Polyomaviridae
GCA_008777065.1	8052	1	Ferret kobuvirus	NA	Viruses; Riboviria; Orthornavirae; Pisuviricota; Pisoniviricetes; Picornavirales; Picornaviridae; Kobuvirus; unclassified Kobuvirus
GCA_002118445.1	7066	1	Ferret parechovirus	NA	Viruses; ssRNA viruses; ssRNA positive-strand viruses, no DNA stage; Picornavirales; Picornaviridae; Parechovirus; Parechovirus D
GCA_006465445.1	374041	483	Cannes 8 virus	NA	Viruses; Varidnaviria; Bamfordvirae; Nucleocytoviricota; Megaviricetes; Pimascovirales; Marseilleviridae; Marseillevirus; unclassified Marseillevirus
GCA_000922515.1	71248	83	Escherichia phage vB_EcoP_PhAPEC5	NA	Viruses; dsDNA viruses, no RNA stage; Caudovirales; Podoviridae; Picovirinae; G7cvirus; Escherichia virus APEC5
GCA_002867455.1	5320	7	Cotton chlorotic spot virus	NA	Viruses; Monodnaviria; Shotokuvirae; Cressdnaviricota; Repensiviricetes; Geplafuvirales; Geminiviridae; Begomovirus
GCA_000869485.1	1358	1	Tomato leaf curl Karnataka betasatellite	NA	Viruses; Tolecusatellitidae; Betasatellite; unclassified Betasatellite
GCA_000910995.1	7539	1	Eel picornavirus 1	F1505	Viruses; ssRNA viruses; ssRNA positive-strand viruses, no DNA stage; Picornavirales; Picornaviridae; Potamipivirus; Potamipivirus A
GCA_006465465.1	106734	101	Andrias davidianus ranavirus	NA	Viruses; Varidnaviria; Bamfordvirae; Nucleocytoviricota; Megaviricetes; Pimascovirales; Iridoviridae; Alphairidovirinae; Ranavirus; Common midwife toad virus
GCA_000912755.1	7616	7	Felis catus papillomavirus 4	NA	Viruses; dsDNA viruses, no RNA stage; Papillomaviridae; Taupapillomavirus; unclassified Taupapillomavirus
GCA_000842565.1	40190	46	Lactobacillus prophage Lj965	NA	Viruses; dsDNA viruses, no RNA stage; Caudovirales; Siphoviridae; unclassified Siphoviridae

gca	genome.size	proteome.size	phage.name	strain	Taxonomy
GCA_000871405.1	40881	56	Lactobacillus prophage Lj771	NA	Viruses; unclassified phages
GCA_000843425.1	38384	50	Lactobacillus prophage Lj928	NA	Viruses; dsDNA viruses, no RNA stage; Caudovirales; Siphoviridae; unclassified Siphoviridae
GCA_000916595.1	5557	5	Anophelineassociated C virus	NA	Viruses; unclassified viruses
GCA_000911115.1	5722	5	African elephant polyomavirus 1	NA	Viruses; dsDNA viruses, no RNA stage; Polyomaviridae; unclassified Polyomaviridae
GCA_000920475.1	55098	63	Pectobacterium phage PM1	NA	Viruses; dsDNA viruses, no RNA stage; Caudovirales; Myoviridae; unclassified Myoviridae
GCA_000913855.1	36969	46	Lactobacillus phage phiJB	NA	Viruses; dsDNA viruses, no RNA stage; Caudovirales; Siphoviridae; unclassified Siphoviridae
GCA_000912975.1	33996	59	Lactobacillus phage phiLdb	NA	Viruses; dsDNA viruses, no RNA stage; Caudovirales; Siphoviridae; unclassified Siphoviridae
GCA_000912915.1	44459	62	Staphylococcus phage YMC0904R1988	NA	Viruses; dsDNA viruses, no RNA stage; Caudovirales; Siphoviridae; unclassified Siphoviridae
GCA_003028975.1	9101	1	pigeon mesivirus 1	HK21	Viruses; Riboviria; Orthornavirae; Pisuviricota; Pisoniviricetes; Picornavirales; Picornaviridae; Megrivirus; Megrivirus B
GCA_018580195.1	5492	4	Pigeon parvovirus A	HK8	Viruses; Monodnaviria; Shotokuvirae; Cossaviricota; Quintoviricetes; Piccovirales; Parvoviridae; unclassified Parvoviridae
GCA_002118085.1	11879	3	Bowe virus	VN1512	Viruses; Riboviria; Orthornavirae; Negarnaviricota; Polyploviricotina; Ellioviricetes; Bunyavirales; Hantaviridae; Mammantavirinae; Orthohantavirus; Bowe orthohantavirus
GCA_000914975.1	7838	6	Donkey orchid symptomless virus	NA	Viruses; ssRNA viruses; ssRNA positive-strand viruses, no DNA stage; Tymovirales; Alphaflexiviridae; Platypuvirus
GCA_000916055.1	7934	1	Fathead minnow picornavirus	09283	Viruses; ssRNA viruses; ssRNA positive-strand viruses, no DNA stage; Picornavirales; Picornaviridae; Limnipivirus; Limnipivirus C
GCA_000914995.1	41604	49	Burkholderia phage JG068	NA	Viruses; dsDNA viruses, no RNA stage; Caudovirales; Podoviridae; Autographivirinae; Phikmvvirus; unclassified phiKMV-like phages
GCA_001654405.1	16146	6	Avian paramyxovirus gooseShimane672000	NA	Viruses; Riboviria; Orthornavirae; Negarnaviricota; Haploviricotina; Monjiviricetes; Mononegavirales; Paramyxoviridae; Avulavirinae; Orthoavulavirus; Avian orthoavulavirus 13
GCA_002595145.1	41143	58	Brucella phage F1	NA	Viruses; Duplodnaviria; Heunggongvirae; Uroviricota; Caudoviricetes; Caudovirales; Podoviridae; Perisivirus; Brucella virus Tb
GCA_000914555.1	218339	241	Erwinia phage PhiEaH1	NA	Viruses; dsDNA viruses, no RNA stage; Caudovirales; Siphoviridae; unclassified Siphoviridae
GCA_000911455.1	44578	50	Ralstonia phage RSB3	NA	Viruses; dsDNA viruses, no RNA stage; Caudovirales; Podoviridae; Autographivirinae; Phikmvvirus; unclassified phiKMV-like phages
GCA_000913115.1	43394	62	Staphylococcus phage phiRS7	NA	Viruses; dsDNA viruses, no RNA stage; Caudovirales; Siphoviridae; unclassified Siphoviridae
GCA_004134065.1	9952	1	Pleione flower breaking virus	NA	Viruses; Riboviria; Orthornavirae; Pisuviricota; Stelpaviricetes; Patatavirales; Potyviridae; unclassified Potyviridae
GCA_000912155.1	4945	4	Acheta domesticus mini ambidensovirus	NA	Viruses; ssDNA viruses; Parvoviridae; Densovirinae; unclassified Densovirinae
GCA_002116115.1	11501	4	Botryosphaeria dothidea chrysovirus 1	G1	Viruses; dsRNA viruses; Chrysoviridae; Chrysovirus; unclassified Chrysovirus
GCA_000913675.1	11470	4	Razdan virus	LEIVArm2741	Viruses; ssRNA viruses; ssRNA negative-strand viruses; Bunyaviridae; Phlebovirus; unclassified Phlebovirus; Bhanja serogroup
GCA_000913635.1	12058	3	Murrumbidgee virus	NA	Viruses; ssRNA viruses; ssRNA negative-strand viruses; Bunyaviridae; Orthobunyavirus; unclassified Orthobunyavirus
GCA_002604085.1	81291	111	Thermus phage G20c	NA	Viruses; Duplodnaviria; Heunggongvirae; Uroviricota; Caudoviricetes; Caudovirales; Siphoviridae; Oshimavirus; unclassified Oshimavirus
GCA_002118925.1	11728	3	Tai virus	F47CI2004	Viruses; Riboviria; Orthornavirae; Negarnaviricota; Polyploviricotina; Ellioviricetes; Bunyavirales; Peribunyaviridae; Herbevirus; Tai herbevirus
GCA_002119005.1	11322	3	Kibale virus	P05UG2008	Viruses; Riboviria; Orthornavirae; Negarnaviricota; Polyploviricotina; Ellioviricetes; Bunyavirales; Peribunyaviridae; Herbevirus; Kibale herbevirus
GCA_000916495.1	40739	45	Acinetobacter phage Petty	NA	Viruses; dsDNA viruses, no RNA stage; Caudovirales; Podoviridae; Autographivirinae; Phikmvvirus; unclassified phiKMV-like phages
GCA_000917335.1	77792	95	Acinetobacter phage Presley	NA	Viruses; dsDNA viruses, no RNA stage; Caudovirales; Podoviridae; N4virus; unclassified N4likevirus
GCA_000912355.1	165238	291	Bacillus phage BigBertha	NA	Viruses; dsDNA viruses, no RNA stage; Caudovirales; Myoviridae; B4virus; Bacillus virus Bigbertha
GCA_000912395.1	50354	79	Bacillus virus Blastoid	NA	Viruses; dsDNA viruses, no RNA stage; Caudovirales; Siphoviridae; Andromedavirus
GCA_000911315.1	146193	231	Bacillus phage CampHawk	NA	Viruses; dsDNA viruses, no RNA stage; Caudovirales; Myoviridae; unclassified Myoviridae
GCA_000912995.1	49246	78	Bacillus virus Glittering	NA	Viruses; dsDNA viruses, no RNA stage; Caudovirales; Siphoviridae; Andromedavirus
GCA_000913015.1	156648	252	Bacillus phage Grass	NA	Viruses; dsDNA viruses, no RNA stage; Caudovirales; Myoviridae; Nit1virus; Bacillus virus Grass

gca	genome.size	proteome.size	phage.name	strain	Taxonomy
GCA_000912335.1	39874	50	Bacillus phage Page	NA	Viruses; dsDNA viruses, no RNA stage; Caudovirales; Podoviridae; Pagevirus; Bacillus virus Page
GCA_002604105.1	39874	50	Bacillus phage poppyseed	NA	Viruses; Duplodnaviria; Heunggongvirae; Uroviricota; Caudoviricetes; Caudovirales; Podoviridae; Pagevirus; Bacillus virus Page; Bacillus phage Page
GCA_000912375.1	49836	79	Bacillus virus Riggi	NA	Viruses; dsDNA viruses, no RNA stage; Caudovirales; Siphoviridae; Andromedavirus
GCA_000911355.1	39844	48	Bacillus phage Pony	NA	Viruses; dsDNA viruses, no RNA stage; Caudovirales; Podoviridae; Pagevirus; Bacillus virus Pony
GCA_000913795.1	80382	111	Bacillus phage Slash	NA	Viruses; dsDNA viruses, no RNA stage; Caudovirales; Siphoviridae; Slashvirus; Bacillus virus Slash
GCA_000913815.1	164297	283	Bacillus phage Spock	NA	Viruses; dsDNA viruses, no RNA stage; Caudovirales; Myoviridae; B4virus; Bacillus virus Spock
GCA_000913835.1	81656	113	Bacillus phage Staley	NA	Viruses; dsDNA viruses, no RNA stage; Caudovirales; Siphoviridae; Slashvirus; Bacillus virus Staley
GCA_000914735.1	56789	78	Clavibacter phage CN1A	NA	Viruses; dsDNA viruses, no RNA stage; Caudovirales; Siphoviridae; unclassified Siphoviridae
GCA_000912955.1	156338	205	Salmonella phage Marshall	NA	Viruses; dsDNA viruses, no RNA stage; Caudovirales; Myoviridae; unclassified Myoviridae
GCA_000911335.1	154701	200	Salmonella phage Maynard	NA	Viruses; dsDNA viruses, no RNA stage; Caudovirales; Myoviridae; unclassified Myoviridae
GCA_000915255.1	10346	1	Zucchini tigre mosaic virus	NA	Viruses; ssRNA viruses; ssRNA positive-strand viruses, no DNA stage; Potyviridae; Potyvirus; unclassified Potyvirus
GCA_000914475.1	45696	65	Pseudomonas phage TL	NA	Viruses; dsDNA viruses, no RNA stage; Caudovirales; Podoviridae; unclassified Podoviridae
GCA_001961415.1	1369	1	Tomato leaf curl alphasatellite	NA	Viruses; Satellites; Satellite Nucleic Acids; Single stranded DNA satellites; Alphasatellites; Begomovirus-associated alphasatellites
GCA_000903535.1	1389	1	Ageratum yellow vein India alphasatellite	NA	Viruses; Alphasatellitidae; Geminialphasatellitinae; Colecusatellite
GCA_000913735.1	39758	54	Shigella phage SfIV	NA	Viruses; dsDNA viruses, no RNA stage; Caudovirales; Myoviridae; unclassified Myoviridae
GCA_000912295.1	39860	51	Erwinia phage FE44	NA	Viruses; dsDNA viruses, no RNA stage; Caudovirales; Podoviridae; Autographivirinae; T7virus; unclassified T7-like viruses
GCA_002584285.1	39860	80	uncultured Mediterranean phage uvMED	NA	Viruses; Duplodnaviria; Heunggongvirae; Uroviricota; Caudoviricetes; Caudovirales; Siphoviridae; environmental samples
GCA_000913695.1	7647	7	Mesocricetus auratus papillomavirus 1	NA	Viruses; dsDNA viruses, no RNA stage; Papillomaviridae; unclassified Papillomaviridae; non-primate mammal papillomaviruses
GCA_000912835.1	17486	1	Rhizoctonia cerealis endornavirus 1	NA	Viruses; dsRNA viruses; Endornaviridae; Endornavirus; unclassified Endornavirus
GCA_000912215.1	19944	10	Mobuck virus	122	Viruses; dsRNA viruses; Reoviridae; Sedoreovirinae; Orbivirus; unclassified Orbivirus
GCA_000927015.1	11139	5	Fikirini rhabdovirus	NA	Viruses; Riboviria; Orthornavirae; Negarnaviricota; Haploviricotina; Monjiviricetes; Mononegavirales; Rhabdoviridae; Ledantevirus; Fikirini ledantevirus
GCA_000912195.1	42940	33	Turkey aviadenovirus 4	NA	Viruses; dsDNA viruses, no RNA stage; Adenoviridae; Aviadenovirus; Turkey aviadenovirus C
GCA_000913655.1	43686	37	Turkey aviadenovirus 5	NA	Viruses; dsDNA viruses, no RNA stage; Adenoviridae; Aviadenovirus; Turkey aviadenovirus D
GCA_000911175.1	3646	2	Red clover cryptic virus 1	NA	Viruses; dsRNA viruses; Partitiviridae; unclassified Partitiviridae
GCA_000912815.1	3850	2	Dill cryptic virus 1	NA	Viruses; dsRNA viruses; Partitiviridae; unclassified Partitiviridae
GCA_000915495.1	38966	45	Cronobacter phage Dev2	NA	Viruses; dsDNA viruses, no RNA stage; Caudovirales; Podoviridae; Autographivirinae; unclassified Autographivirinae
GCA_000911155.1	9271	1	Halyomorpha halys virus	NA	Viruses; ssRNA viruses; ssRNA positive-strand viruses, no DNA stage; Picornavirales; Iflaviridae; unclassified Iflaviridae
GCA_000922715.1	54279	84	Clostridium phage CDMH1	NA	Viruses; dsDNA viruses, no RNA stage; Caudovirales; Myoviridae; unclassified Myoviridae
GCA_000918495.1	3830	2	Rhizoctonia solani dsRNA virus 2	NA	Viruses; dsRNA viruses; Partitiviridae; unclassified Partitiviridae
GCA_000915035.1	17276	20	Zamilon virus	strain	Viruses; Virus families not assigned to an order; Lavidaviridae; Sputnikvirus; Mimivirus-dependent virus Zamilon
GCA_000859245.1	7801	3	Banana streak VN virus	Acuminata Vietnam	Viruses; Riboviria; Pararnavirae; Artverviricota; Revtraviricetes; Ortervirales; Caulimoviridae; Badnavirus
GCA_000916455.1	128546	189	Lactococcus phage phiL47	NA	Viruses; dsDNA viruses, no RNA stage; Caudovirales; Siphoviridae; unclassified Siphoviridae
GCA_004033275.1	2041	3	Pigeon circovirus	PiCVJapan22010	Viruses; Monodnaviria; Shotokuvirae; Cressdnaviricota; Arfiviricetes; Cirlivirales; Circoviridae; Circovirus
GCA_000911235.1	9359	1	Tall oatgrass mosaic virus	NA	Viruses; ssRNA viruses; ssRNA positive-strand viruses, no DNA stage; Potyviridae; Tritimovirus; unclassified Tritimovirus

gca	genome.size	proteome.size	phage.name	strain	Taxonomy
GCA_002003955.1	16934	5	Pepper chlorotic spot virus	NA	Viruses; ssRNA viruses; ssRNA negative-strand viruses; Bunyaviridae; Tospovirus; unclassified Tospovirus
GCA_000912895.1	5280	5	Solenopsis invicta densovirus	NA	Viruses; ssDNA viruses; Parvoviridae; Densovirinae; Ambidensovirus; unclassified Densovirus
GCA_000911275.1	40931	63	Lactobacillus phage J1	NA	Viruses; dsDNA viruses, no RNA stage; Caudovirales; Siphoviridae; unclassified Siphoviridae
GCA_000916995.1	109558	181	Rhizobium phage vB_RleS_L338C	NA	Viruses; dsDNA viruses, no RNA stage; Caudovirales; Siphoviridae; unclassified Siphoviridae
GCA_000915775.1	71629	103	Shigella phage pSb1	NA	Viruses; dsDNA viruses, no RNA stage; Caudovirales; Podoviridae; Picovirinae; G7cvirus; Shigella virus Sb1
GCA_000915795.1	77837	96	Vibrio phage SHOU24	NA	Viruses; dsDNA viruses, no RNA stage; Caudovirales; Siphoviridae; unclassified Siphoviridae
GCA_002593005.1	28452	53	Lactococcus phage SK1833	NA	Viruses; Duplodnaviria; Heunggongvirae; Uroviricota; Caudoviricetes; Caudovirales; Siphoviridae; Skunavirus; Lactococcus virus sk1
GCA_000911555.1	37372	68	Arthrobacter phage vB_ArSArV2	NA	Viruses; dsDNA viruses, no RNA stage; Caudovirales; Siphoviridae; unclassified Siphoviridae
GCA_000914435.1	62466	105	Mycobacterium phage Validus	NA	Viruses; dsDNA viruses, no RNA stage; Caudovirales; Siphoviridae; unclassified Siphoviridae
GCA_000914795.1	68262	97	Mycobacterium phage Suffolk	NA	Viruses; dsDNA viruses, no RNA stage; Caudovirales; Siphoviridae; unclassified Siphoviridae; PG1-like phages; Mycobacterium phage KLucky39
GCA_002604125.1	41510	60	Enterobacteria phage IME_EC2	NA	Viruses; Duplodnaviria; Heunggongvirae; Uroviricota; Caudoviricetes; Caudovirales; Podoviridae; Murrayvirus; Escherichia virus EC2
GCA_000914055.1	43869	51	Xylella phage Paz	NA	Viruses; dsDNA viruses, no RNA stage; Caudovirales; Podoviridae; Autographivirinae; Phikmvvirus; unclassified phiKMV-like phages
GCA_000913195.1	43940	52	Xylella phage Prado	NA	Viruses; dsDNA viruses, no RNA stage; Caudovirales; Podoviridae; Autographivirinae; Phikmvvirus; unclassified phiKMV-like phages
GCA_002604145.1	55601	72	Xylella phage Salvo	NA	Viruses; Duplodnaviria; Heunggongvirae; Uroviricota; Caudoviricetes; Caudovirales; Siphoviridae; Sanovirus; Xylella virus Salvo
GCA_002604165.1	56147	77	Xylella phage Sano	NA	Viruses; Duplodnaviria; Heunggongvirae; Uroviricota; Caudoviricetes; Caudovirales; Siphoviridae; Sanovirus; Xylella virus Sano
GCA_000913875.1	2020	3	Gyrovirus Tu243	NA	Viruses; ssDNA viruses; Circoviridae; Gyrovirus; unclassified Gyrovirus
GCA_000912415.1	2282	3	Gyrovirus Tu789	NA	Viruses; ssDNA viruses; Circoviridae; Gyrovirus; unclassified Gyrovirus
GCA_000908155.1	3064	2	Ustilaginoidea virens partitivirus 2	NA	Viruses; dsRNA viruses; Partitiviridae; unclassified Partitiviridae
GCA_002604185.1	73659	89	Achromobacter phage JWDelta	NA	Viruses; Duplodnaviria; Heunggongvirae; Uroviricota; Caudoviricetes; Caudovirales; Podoviridae; Jwalphavirus; unclassified Jwalphavirus
GCA_000914775.1	72329	91	Achromobacter phage JWAlpha	NA	Viruses; dsDNA viruses, no RNA stage; Caudovirales; Podoviridae; N4virus; unclassified N4likevirus
GCA_000916415.1	43766	51	Klebsiella phage F19	Klebsiella pneumoniae	Viruses; dsDNA viruses, no RNA stage; Caudovirales; Podoviridae; Autographivirinae; Kp34virus; Klebsiella virus F19
GCA_000917055.1	41687	69	Enterococcus phage IME_EF3	NA	Viruses; dsDNA viruses, no RNA stage; Caudovirales; Siphoviridae; unclassified Siphoviridae
GCA_000913895.1	7807	1	Feline sakobuvirus A	NA	Viruses; ssRNA viruses; ssRNA positive-strand viruses, no DNA stage; Picornavirales; Picornaviridae; Sakobuvirus
GCA_000912435.1	6379	4	Yellow tailflower mild mottle virus	NA	Viruses; ssRNA viruses; ssRNA positive-strand viruses, no DNA stage; Virgaviridae; Tobamovirus; unclassified Tobamovirus
GCA_002604205.1	46719	78	Escherichia phage bV_EcoS_AHP24	NA	Viruses; Duplodnaviria; Heunggongvirae; Uroviricota; Caudoviricetes; Caudovirales; Drexlerviridae; Rogunavirinae; Rogunavirus; Escherichia virus AHS24; Escherichia phage vB_EcoS_AHS24
GCA_000921675.1	46847	76	Escherichia phage vB_EcoS_AHP42	NA	Viruses; dsDNA viruses, no RNA stage; Caudovirales; Siphoviridae; Tunavirinae; Rogue1virus; Escherichia virus AHP42
GCA_000921635.1	46440	81	Escherichia phage vB_EcoS_AHS24	NA	Viruses; dsDNA viruses, no RNA stage; Caudovirales; Siphoviridae; Tunavirinae; Rogue1virus; Escherichia virus AHS24
GCA_000924195.1	45746	74	Escherichia phage vB_EcoS_AKS96	NA	Viruses; dsDNA viruses, no RNA stage; Caudovirales; Siphoviridae; Tunavirinae; Rogue1virus; Escherichia virus AKS96
GCA_001551625.1	60875	76	Shigella phage 7502 Stx	NA	Viruses; dsDNA viruses, no RNA stage; Caudovirales; Podoviridae; unclassified Podoviridae
GCA_001507495.1	38269	54	Lactobacillus phage phi jlb1	NA	Viruses; dsDNA viruses, no RNA stage; Caudovirales; Myoviridae; unclassified Myoviridae
GCA_000922575.1	9355	1	Equine hepacivirus JPN3JAPAN2013	NA	Viruses; ssRNA viruses; ssRNA positive-strand viruses, no DNA stage; Flaviviridae; Hepacivirus; unclassified Hepacivirus; Equine hepacivirus
GCA_004787055.1	9321	2	Marine RNA virus SF2	NA	Viruses; Riboviria; Orthornavirae; Pisuviricota; Pisoniviricetes; Picornavirales; Marnaviridae; Locarnavirus; Sanfarnavirus 2
GCA_006465485.1	150194	151	Goatpox virus FZ	NA	Viruses; Varidnaviria; Bamfordvirae; Nucleocytoviricota; Pokkesviricetes; Chitovirales; Poxviridae; Chordopoxvirinae; Capripoxvirus; Goatpox virus
GCA_003260795.1	127879	132	Sea otter poxvirus	ELK	Viruses; Varidnaviria; Bamfordvirae; Nucleocytoviricota; Pokkesviricetes; Chitovirales; Poxviridae; Chordopoxvirinae; Mustelpoxvirus; Sea otterpox virus

gca	genome.size	proteome.size	phage.name	strain	Taxonomy
GCA_013086555.1	12720	4	Salanga virus	AnB 904a	Viruses; Riboviria; Orthornavirae; Negarnaviricota; Polyploviricotina; Ellioviricetes; Bunyavirales; Phenuiviridae; Phlebovirus; Salanga phlebovirus
GCA_018580205.1	7408	6	Ambrosia asymptomatic virus 1	NA	Viruses; Riboviria; Orthornavirae; Kitrinoviricota; Alsuviricetes; Tymovirales; Alphaflexiviridae; Potexvirus
GCA_000913935.1	40471	54	Ralstonia phage RSK1	NA	Viruses; dsDNA viruses, no RNA stage; Caudovirales; Podoviridae; unclassified Podoviridae
GCA_000911415.1	5479	4	Feline bocavirus 2	NA	Viruses; ssDNA viruses; Parvoviridae; Parvovirinae; Bocaparvovirus; unclassified Bocaparvovirus
GCA_002830225.1	1359	1	Siegesbeckia yellow vein betasatellite	NA	Viruses; Tolecusatellitidae; Betasatellite
GCA_000855465.1	1114	1	Grapevine fanleaf virus satellite RNA	NA	Viruses; Satellites; Satellite Nucleic Acids; Single stranded RNA satellites; Large single stranded RNA satellites
GCA_003069445.1	17070	23	Vibrio virus CTXphi	NA	Viruses; Monodnaviria; Loebvirae; Hofneiviricota; Faserviricetes; Tubulavirales; Inoviridae; Affertcholeramvirus
GCA_004787095.1	8648	1	Marine RNA virus SF3	NA	Viruses; Riboviria; Orthornavirae; Pisuviricota; Pisoniviricetes; Picornavirales; Marnaviridae; Locarnavirus; Sanfarnavirus 3
GCA_006457465.1	103266	95	Zoo ranavirus	NA	Viruses; Varidnaviria; Bamfordvirae; Nucleocytoviricota; Megaviricetes; Pimascovirales; Iridoviridae; Alphairidovirinae; Ranavirus; Frog virus 3
GCA_000913915.1	1777	3	Penaeus monodon circovirus VN11	NA	Viruses; ssDNA viruses; Circoviridae; Circovirus; unclassified Circovirus
GCA_003179555.1	7204	7	human papillomavirus 168	NA	Viruses; Monodnaviria; Shotokuvirae; Cossaviricota; Papovaviricetes; Zurhausenvirales; Papillomaviridae; Firstpapillomavirinae; Gammapapillomavirus; Gammapapillomavirus 8
GCA_000913075.1	7228	6	Human papillomavirus 167	NA	Viruses; dsDNA viruses, no RNA stage; Papillomaviridae; Gammapapillomavirus; Gammapapillomavirus 21
GCA_004030235.1	1975	3	Anguilla anguilla circovirus	NA	Viruses; ssDNA viruses; Circoviridae; Circovirus; unclassified Circovirus
GCA_000849385.1	10205	2	Taura syndrome virus	NA	Viruses; ssRNA viruses; ssRNA positive-strand viruses, no DNA stage; Picornavirales; Dicistroviridae; Aparavirus
GCA_000913095.1	4464	5	Yam spherical virus	NA	Viruses; ssRNA viruses; ssRNA positive-strand viruses, no DNA stage; Tombusviridae; Aureusvirus; unclassified Aureusvirus
GCA_002826725.1	380011	484	Tunisvirus fontaine2	U484	Viruses; Varidnaviria; Bamfordvirae; Nucleocytoviricota; Megaviricetes; Pimascovirales; Marseilleviridae; Tunisvirus
GCA_000911435.1	14581	1	Sclerotinia sclerotiorum hypovirus 2	NA	Viruses; dsRNA viruses; Hypoviridae; Hypovirus; unclassified Hypovirus
GCA_000921255.1	8431	7	Cherry twisted leaf associated virus	NA	Viruses; ssRNA viruses; ssRNA positive-strand viruses, no DNA stage; Tymovirales; Betaflexiviridae; Quinvirinae; Robigovirus
GCA_000921215.1	11840	6	Sierra Nevada virus	NA	Viruses; ssRNA viruses; ssRNA negative-strand viruses; Mononegavirales; Nyamiviridae; unclassified Nyamiviridae
GCA_002145785.1	11910	3	Anjozorobe virus	AnjozorobeEmMDG2009ATD49	Viruses; Riboviria; Orthornavirae; Negarnaviricota; Polyploviricotina; Ellioviricetes; Bunyavirales; Hantaviridae; Mammantavirinae; Orthohantavirus; Thailand orthohantavirus
GCA_000875265.1	7961	1	Saffold virus 2	NA	Viruses; Riboviria; Orthornavirae; Pisuviricota; Pisoniviricetes; Picornavirales; Picornaviridae; Cardiovirus; Cardiovirus B
GCA_000925095.1	8047	3	Carrot Ch virus 2	NA	Viruses; ssRNA viruses; ssRNA positive-strand viruses, no DNA stage; Tymovirales; Betaflexiviridae; Trivirinae; Chordovirus
GCA_000925115.1	8155	3	Carrot Ch virus 1	NA	Viruses; ssRNA viruses; ssRNA positive-strand viruses, no DNA stage; Tymovirales; Betaflexiviridae; Trivirinae; Chordovirus
GCA_000927615.2	11939	3	Carrot torradovirus 1	NA	Viruses; ssRNA viruses; ssRNA positive-strand viruses, no DNA stage; Picornavirales; Secoviridae; Torradovirus
GCA_019181185.1	15923	9	Carrot closterovirus	NA	Viruses; Riboviria; Orthornavirae; Kitrinoviricota; Alsuviricetes; Martellivirales; Closteroviridae; unclassified Closteroviridae
GCA_003071245.1	2158	3	Beet western yellows virus associated RNA	NA	Viruses; Riboviria; Virus-associated RNAs
GCA_000846785.1	1952	2	Canary circovirus	NA	Viruses; ssDNA viruses; Circoviridae; Circovirus
GCA_018580175.1	5419	4	Cassava alphaflexivirus	NA	Viruses; Riboviria; Orthornavirae; Kitrinoviricota; Alsuviricetes; Tymovirales; Alphaflexiviridae; unclassified Alphaflexiviridae
GCA_000921755.1	2169	3	Seal anellovirus 3	NA	Viruses; ssDNA viruses; Anelloviridae; unclassified Anelloviridae
GCA_000924275.1	2149	3	Seal anellovirus 2	NA	Viruses; ssDNA viruses; Anelloviridae; unclassified Anelloviridae
GCA_013087225.1	4913	3	Seal parvovirus	NA	Viruses; Monodnaviria; Shotokuvirae; Cossaviricota; Quintoviricetes; Piccovirales; Parvoviridae; Parvovirinae; Erythroparvovirus; Pinniped erythroparvovirus 1
GCA_001343745.1	24835	12	Cimodo virus	C74CI2004	Viruses; dsRNA viruses; Reoviridae; unclassified Reoviridae
GCA_000915855.1	1348	2	Hedyotis yellow mosaic betasatellite	NA	Viruses; Tolecusatellitidae; Betasatellite
GCA_002822405.1	2749	9	Hedyotis uncinella yellow mosaic virus	NA	Viruses; Monodnaviria; Shotokuvirae; Cressdnaviricota; Repensiviricetes; Geplafuvirales; Geminiviridae; Begomovirus

gca	genome.size	proteome.size	phage.name	strain	Taxonomy
GCA_000922955.1	1392	1	Chilli leaf curl alphasatellite	NA	Viruses; Satellites; Satellite Nucleic Acids; Single stranded DNA satellites; Alphasatellites; Begomovirus-associated alphasatellites
GCA_000875045.1	8550	6	Hop mosaic virus	NA	Viruses; ssRNA viruses; ssRNA positive-strand viruses, no DNA stage; Tymovirales; Betaflexiviridae; Quinvirinae; Carlavirus
GCA_000925335.1	11120	5	Kolente virus	NA	Viruses; ssRNA viruses; ssRNA negative-strand viruses; Mononegavirales; Rhabdoviridae; unclassified Rhabdoviridae
GCA_003146845.1	60941	79	Escherichia phage P13803	NA	Viruses; Duplodnaviria; Heunggongvirae; Uroviricota; Caudoviricetes; Caudovirales; Podoviridae; Sepvirinae; Oslovirus; Escherichia virus 191
GCA_003146865.1	61343	79	Escherichia phage P8983	NA	Viruses; Duplodnaviria; Heunggongvirae; Uroviricota; Caudoviricetes; Caudovirales; Podoviridae; Sepvirinae; Oslovirus; Escherichia virus 191
GCA_003146885.1	61566	79	Escherichia phage P13771	NA	Viruses; Duplodnaviria; Heunggongvirae; Uroviricota; Caudoviricetes; Caudovirales; Podoviridae; Sepvirinae; Oslovirus; Escherichia virus 191
GCA_003146905.1	61465	79	Escherichia phage P14437	NA	Viruses; Duplodnaviria; Heunggongvirae; Uroviricota; Caudoviricetes; Caudovirales; Podoviridae; Sepvirinae; Oslovirus; Escherichia virus 191
GCA_000911495.1	45344	69	Pseudomonas phage phiIBBPAA2	NA	Viruses; dsDNA viruses, no RNA stage; Caudovirales; Podoviridae; Luz24virus; unclassified Luz24likevirus
GCA_000914755.1	52974	95	Mycobacterium phage EagleEye	NA	Viruses; dsDNA viruses, no RNA stage; Caudovirales; Siphoviridae; unclassified Siphoviridae
GCA_000917295.1	75568	94	Erwinia phage Ea92	NA	Viruses; dsDNA viruses, no RNA stage; Caudovirales; Podoviridae; N4virus; unclassified N4likevirus
GCA_000914635.1	271084	318	Erwinia phage Ea3570	NA	Viruses; dsDNA viruses, no RNA stage; Caudovirales; Myoviridae; unclassified Myoviridae
GCA_000915695.1	51236	89	Mycobacterium phage BellusTerra	NA	Viruses; dsDNA viruses, no RNA stage; Caudovirales; Siphoviridae; unclassified Siphoviridae; Mycobacterium phage Eagle
GCA_002755915.1	51369	91	Mycobacterium phage Melvin	NA	Viruses; Duplodnaviria; Heunggongvirae; Uroviricota; Caudoviricetes; Caudovirales; Siphoviridae; Fromanvirus; unclassified Fromanvirus
GCA_000917195.1	47162	78	Mycobacterium phage Donovan	NA	Viruses; dsDNA viruses, no RNA stage; Caudovirales; Siphoviridae; unclassified Siphoviridae
GCA_001503535.1	170286	291	Pectobacterium bacteriophage PM2	NA	Viruses; dsDNA viruses, no RNA stage; Caudovirales; Myoviridae; unclassified Myoviridae
GCA_000911535.1	68196	100	Mycobacterium phage Bernardo	NA	Viruses; dsDNA viruses, no RNA stage; Caudovirales; Siphoviridae; unclassified Siphoviridae
GCA_000914015.1	52390	91	Mycobacterium phage HanShotFirst	NA	Viruses; dsDNA viruses, no RNA stage; Caudovirales; Siphoviridae; unclassified Siphoviridae
GCA_000916435.1	70841	98	Mycobacterium phage JAMaL	NA	Viruses; dsDNA viruses, no RNA stage; Caudovirales; Siphoviridae; unclassified Siphoviridae
GCA_000915055.1	51319	84	Mycobacterium phage Jovo	NA	Viruses; dsDNA viruses, no RNA stage; Caudovirales; Siphoviridae; unclassified Siphoviridae
GCA_002603805.1	74533	141	Mycobacterium phage Mosby	NA	Viruses; Duplodnaviria; Heunggongvirae; Uroviricota; Caudoviricetes; Caudovirales; Siphoviridae; Kostyavirus; unclassified Kostyavirus
GCA_000911515.1	76323	146	Mycobacterium phage HufflyPuff	NA	Viruses; dsDNA viruses, no RNA stage; Caudovirales; Siphoviridae; unclassified Siphoviridae
GCA_003571885.1	37129	39	Methanobacterium virus Drs3	NA	Viruses; Duplodnaviria; Heunggongvirae; Uroviricota; Caudoviricetes; Caudovirales; Siphoviridae; unclassified Siphoviridae
GCA_000919915.2	169148	273	Escherichia phage vB_EcoM_JS09	NA	Viruses; dsDNA viruses, no RNA stage; Caudovirales; Myoviridae; Tevenvirinae; Rb69virus; Escherichia virus JS09
GCA_000923935.1	2778	6	Mirabilis leaf curl virus	NA	Viruses; Monodnaviria; Shotokuvirae; Cressdnaviricota; Repensiviricetes; Geplafuvirales; Geminiviridae; Begomovirus
GCA_000897695.1	7583	1	Bovine hungarovirus 1	BHUV12008HUN	Viruses; ssRNA viruses; ssRNA positive-strand viruses, no DNA stage; Picornavirales; Picornaviridae; Hunnivirus; Hunnivirus A
GCA_000916715.1	10145	2	Lamium mild mosaic virus	NA	Viruses; ssRNA viruses; ssRNA positive-strand viruses, no DNA stage; Picornavirales; Secoviridae; Comovirinae; Fabavirus
GCA_002604225.1	42409	42	Burkholderia phage BpAMP1	NA	Viruses; Duplodnaviria; Heunggongvirae; Uroviricota; Caudoviricetes; Caudovirales; Autographiviridae; Okabevirinae; Ampunavirus; Burkholderia virus BpAMP1
GCA_001021255.1	4194	5	Rottboellia yellow mottle virus	NA	Viruses; ssRNA viruses; ssRNA positive-strand viruses, no DNA stage; Sobemovirus; unclassified Sobemovirus
GCA_000915875.1	2753	6	Spinach yellow vein Sikar virus	NA	Viruses; ssDNA viruses; Geminiviridae; Begomovirus; unclassified Begomovirus
GCA_000916395.1	40692	60	Enterococcus phage IMEEF4	NA	Viruses; dsDNA viruses, no RNA stage; Caudovirales; Siphoviridae; unclassified Siphoviridae
GCA_000916195.1	46243	59	Oenococcus phage phiS11	NA	Viruses; dsDNA viruses, no RNA stage; unclassified dsDNA phages
GCA_000916215.1	43454	54	Oenococcus phage phiS13	NA	Viruses; dsDNA viruses, no RNA stage; unclassified dsDNA phages
GCA_000915215.1	9554	2	Formica exsecta virus 1	NA	Viruses; ssRNA viruses; ssRNA positive-strand viruses, no DNA stage; Picornavirales; Dicistroviridae; unclassified Dicistroviridae
GCA_000916735.1	9160	1	Formica exsecta virus 2	NA	Viruses; ssRNA viruses; ssRNA positive-strand viruses, no DNA stage; Picornavirales; Iflaviridae; Iflavirus; unclassified Iflavirus

gca	genome.size	proteome.size	phage.name	strain	Taxonomy
GCA_001646555.1	8082	6	Cervus papillomavirus 2	NA	Viruses; dsDNA viruses, no RNA stage; Papillomaviridae; Epsilonpapillomavirus; unclassified Epsilonpapillomavirus
GCA_002602145.1	42542	34	Vibrio phage AS51	NA	Viruses; Duplodnaviria; Heunggongvirae; Uroviricota; Caudoviricetes; Caudovirales; Autographiviridae; Colwellvirinae; Kaohsiungvirus; Vibrio virus AS51
GCA_000915755.1	92417	129	Staphylococcus phage vB_SepS_SEP9	NA	Viruses; dsDNA viruses, no RNA stage; Caudovirales; Siphoviridae; Sextaecvirus; Staphylococcus virus SEP9
GCA_001041535.1	166977	255	Escherichia phage HY01	NA	Viruses; dsDNA viruses, no RNA stage; Caudovirales; Myoviridae; unclassified Myoviridae
GCA_004788635.1	144144	86	Beluga whale alphaherpesvirus 1	LN31311	Viruses; Duplodnaviria; Heunggongvirae; Peploviricota; Herviviricetes; Herpesvirales; Herpesviridae; Alphaherpesvirinae; Varicellovirus; Monodontid alphaherpesvirus 1
GCA_002827025.1	7226	7	Human papillomavirus 175	NA	Viruses; Monodnaviria; Shotokuvirae; Cossaviricota; Papovaviricetes; Zurhausenvirales; Papillomaviridae; Firstpapillomavirinae; Gammapapillomavirus; Gammapapillomavirus 23
GCA_003179575.1	7261	7	Human papillomavirus 171	NA	Viruses; Monodnaviria; Shotokuvirae; Cossaviricota; Papovaviricetes; Zurhausenvirales; Papillomaviridae; Firstpapillomavirinae; Gammapapillomavirus; Gammapapillomavirus 11
GCA_002827005.1	7203	7	Human papillomavirus 172	NA	Viruses; Monodnaviria; Shotokuvirae; Cossaviricota; Papovaviricetes; Zurhausenvirales; Papillomaviridae; Firstpapillomavirinae; Gammapapillomavirus; Gammapapillomavirus 22
GCA_003179595.1	7297	6	Human papillomavirus 173	NA	Viruses; Monodnaviria; Shotokuvirae; Cossaviricota; Papovaviricetes; Zurhausenvirales; Papillomaviridae; Firstpapillomavirinae; Gammapapillomavirus; Gammapapillomavirus 1
GCA_000916755.1	6996	2	Citrus endogenous pararetrovirus	NA	Viruses; Riboviria; Pararnavirae; Artverviricota; Revtraviricetes; Ortervirales; Retroviridae; unclassified Retroviridae
GCA_003159115.1	19274	10	Koyama Hill virus	S7	Viruses; Riboviria; Orthornavirae; Duplornaviricota; Resentoviricetes; Reovirales; Reoviridae; Sedoreovirinae; Orbivirus; Umatilla virus
GCA_000916175.1	46145	55	Oenococcus phage phi9805	NA	Viruses; dsDNA viruses, no RNA stage; unclassified dsDNA phages
GCA_000915595.1	2563	1	Sclerotinia sclerotiorum mitovirus 6	NA	Viruses; ssRNA viruses; ssRNA positive-strand viruses, no DNA stage; Narnaviridae; Mitovirus; unclassified Mitovirus
GCA_001651105.1	4483	2	Sclerotinia sclerotiorum umbralike virus 1	NA	Viruses; ssRNA viruses; ssRNA positive-strand viruses, no DNA stage; Tombusviridae; Umbravirus; unclassified Umbravirus
GCA_000960965.1	10009	6	Sclerotinia sclerotiorum negativestranded RNA virus 3	NA	Viruses; ssRNA viruses; ssRNA negative-strand viruses; Mononegavirales; unassigned Mononegavirales
GCA_000922875.1	6233	3	Bovine astrovirus	BAstVGX7CHN2014	Viruses; ssRNA viruses; ssRNA positive-strand viruses, no DNA stage; Astroviridae; Mamastrovirus; unclassified Mamastrovirus
GCA_000917255.1	76184	126	Escherichia phage KBNP1711	NA	Viruses; dsDNA viruses, no RNA stage; Caudovirales; Podoviridae; unclassified Podoviridae
GCA_000915955.1	7825	8	Pea yellow stunt virus	NA	Viruses; ssDNA viruses; Nanoviridae; unclassified Nanoviridae
GCA_003087675.1	11490	4	FTLS virus	NA	Viruses; Riboviria; Orthornavirae; Negarnaviricota; Polyploviricotina; Ellioviricetes; Bunyavirales; Phenuiviridae; Phlebovirus; SFTS phlebovirus
GCA_002818825.1	10528	4	Loei River mammarenavirus	NA	Viruses; Riboviria; Orthornavirae; Negarnaviricota; Polyploviricotina; Ellioviricetes; Bunyavirales; Arenaviridae; Mammarenavirus
GCA_002604265.1	42492	41	Burkholderia phage BpAMP2	NA	Viruses; Duplodnaviria; Heunggongvirae; Uroviricota; Caudoviricetes; Caudovirales; Autographiviridae; Okabevirinae; Ampunavirus; unclassified Ampunavirus
GCA_002604285.1	42112	43	Burkholderia phage BpAMP4	NA	Viruses; Duplodnaviria; Heunggongvirae; Uroviricota; Caudoviricetes; Caudovirales; Autographiviridae; Okabevirinae; Ampunavirus; unclassified Ampunavirus
GCA_001550945.1	33596	50	Clostridium phage phiCDHM13	NA	Viruses; dsDNA viruses, no RNA stage; Caudovirales; Myoviridae; unclassified Myoviridae
GCA_000914695.1	53159	100	Mycobacterium phage Echild	NA	Viruses; dsDNA viruses, no RNA stage; Caudovirales; Siphoviridae; unclassified Siphoviridae
GCA_000851645.1	4536	3	Epinephelus tauvina nervous necrosis virus	Singapore	Viruses; Riboviria; Orthornavirae; Kitrinoviricota; Magsaviricetes; Nodamuvirales; Nodaviridae; Betanodavirus; Redspotted grouper nervous necrosis virus
GCA_000915915.1	7697	1	Carp picornavirus 1	NA	Viruses; ssRNA viruses; ssRNA positive-strand viruses, no DNA stage; Picornavirales; Picornaviridae; Limnipivirus; Limnipivirus B
GCA_001041755.1	346602	64	Klebsiella phage K641	NA	Viruses; dsDNA viruses, no RNA stage; Caudovirales; Myoviridae; unclassified Myoviridae
GCA_000917835.1	11624	2	Madariaga virus	MADVCebus apellaBRABEAN51221956	Viruses; ssRNA viruses; ssRNA positive-strand viruses, no DNA stage; Togaviridae; Alphavirus
GCA_000919695.1	995	1	Faba bean necrotic stunt alphasatellite	NA	Viruses; Alphasatellitidae; Nanoalphasatellitinae; Mivedwarsatellite
GCA_000920555.1	1030	1	Black medic leafroll alphasatellite 1	NA	Viruses; Satellites; Satellite Nucleic Acids; Single stranded DNA satellites; Alphasatellites; Babu- and nanovirus-associated alphasatellites; unclassified Babu- and nanovirus-associated alphasatellites
GCA_003028995.1	1021	1	Pea necrotic yellow dwarf alphasatellite 1	NA	Viruses; Alphasatellitidae; Nanoalphasatellitinae; Mivedwarsatellite
GCA_003029005.1	1000	1	Pea necrotic yellow dwarf alphasatellite 3	NA	Viruses; Alphasatellitidae; Nanoalphasatellitinae; unclassified Nanoalphasatellitinae
GCA_000919055.1	1001	1	Faba bean necrotic stunt alphasatellite 1	NA	Viruses; Satellites; Satellite Nucleic Acids; Single stranded DNA satellites; Alphasatellites; Babu- and nanovirus-associated alphasatellites; unclassified Babu- and nanovirus-associated alphasatellites
GCA_008776955.1	7428	1	Enterovirus B111	Q0011XZCHN2000	Viruses; Riboviria; Orthornavirae; Pisuviricota; Pisoniviricetes; Picornavirales; Picornaviridae; Enterovirus; Enterovirus B; unclassified Enterovirus B

gca	genome.size	proteome.size	phage.name	strain	Taxonomy
GCA_000914515.1	8176	8	Haloarcula hispanica pleomorphic virus 2	NA	Viruses; dsDNA viruses, no RNA stage; unclassified dsDNA phages
GCA_013087235.1	3539	2	Turkey parvovirus TP12012HUN	NA	Viruses; Monodnaviria; Shotokuvirae; Cossaviricota; Quintoviricetes; Piccovirales; Parvoviridae; Parvovirinae; unclassified Parvovirinae; Chapparvovirus
GCA_000922535.1	10365	1	Ilomantsi virus	NA	Viruses; ssRNA viruses; ssRNA positive-strand viruses, no DNA stage; Flaviviridae; unclassified Flaviviridae
GCA_000916375.1	51250	87	Mycobacterium phage Nyxis	NA	Viruses; dsDNA viruses, no RNA stage; Caudovirales; Siphoviridae; unclassified Siphoviridae
GCA_000916475.1	51797	89	Mycobacterium phage Obama12	NA	Viruses; dsDNA viruses, no RNA stage; Caudovirales; Siphoviridae; L5virus; Mycobacterium phage LHTSCC
GCA_000917275.1	42240	70	Mycobacterium phage MichelleMyBell	NA	Viruses; dsDNA viruses, no RNA stage; Caudovirales; Siphoviridae; unclassified Siphoviridae
GCA_000917235.1	69099	92	Mycobacterium phage Oaker	NA	Viruses; dsDNA viruses, no RNA stage; Caudovirales; Siphoviridae; unclassified Siphoviridae
GCA_000915735.1	57775	101	Mycobacterium phage Saal	NA	Viruses; dsDNA viruses, no RNA stage; Caudovirales; Siphoviridae; unclassified Siphoviridae
GCA_000915655.1	50781	86	Mycobacterium phage 32HC	NA	Viruses; dsDNA viruses, no RNA stage; Caudovirales; Siphoviridae; unclassified Siphoviridae
GCA_001501795.1	51366	76	Bacillus phage vB_BtS_BMBtp3	NA	Viruses; dsDNA viruses, no RNA stage; Caudovirales; Siphoviridae; unclassified Siphoviridae
GCA_001551765.1	151417	211	Bacillus phage Bp8pC	NA	Viruses; Duplodnaviria; Heunggongvirae; Uroviricota; Caudoviricetes; Caudovirales; Herelleviridae; Bastillevirinae; Agatevirus; Bacillus virus Bp8pC
GCA_002604305.1	151419	211	Bacillus phage Bp8pT	NA	Viruses; dsDNA viruses, no RNA stage; Caudovirales; Myoviridae; unclassified Myoviridae
GCA_000921115.1	42597	70	Enterococcus phage IMEEFm1	NA	Viruses; dsDNA viruses, no RNA stage; Caudovirales; Siphoviridae; unclassified Siphoviridae
GCA_000917215.2	54791	67	Shewanella phage Spp001	NA	Viruses; dsDNA viruses, no RNA stage; Caudovirales; Myoviridae; unclassified Myoviridae
GCA_000954975.2	159914	259	Salmonella phage STP4a	NA	Viruses; dsDNA viruses, no RNA stage; Caudovirales; Myoviridae; Tevenvirinae; T4virus; unclassified T4virus
GCA_000920795.1	108483	156	Escherichia phage vB_EcoS_FFH_1	NA	Viruses; Duplodnaviria; Heunggongvirae; Uroviricota; Caudoviricetes; Caudovirales; Demerecviridae; Markadamsvirinae; Tequintavirus; Escherichia virus FFH1
GCA_000919935.1	139020	218	Escherichia phage FFH2	NA	Viruses; dsDNA viruses, no RNA stage; Caudovirales; Myoviridae; Vequintavirinae; V5virus; Escherichia virus FFH2
GCA_000922695.1	162910	210	Salmonella phage vB_SalM_SJ3	NA	Viruses; dsDNA viruses, no RNA stage; Caudovirales; Myoviridae; unclassified Myoviridae
GCA_000872565.1	10396	4	Allpahuayo mammarenavirus	CLHP2472	Viruses; ssRNA viruses; ssRNA negative-strand viruses; Arenaviridae; Mammarenavirus
GCA_000921875.1	38392	56	Streptococcus phage DCC1738	NA	Viruses; unclassified phages
GCA_000921895.1	39360	53	Streptococcus phage K13	NA	Viruses; unclassified phages
GCA_000922935.1	39965	49	Streptococcus phage IC1	NA	Viruses; unclassified phages
GCA_000915555.1	45550	69	Pseudomonas phage vB_PaeP_Tr60_Ab31	NA	Viruses; dsDNA viruses, no RNA stage; unclassified dsDNA phages
GCA_003179495.1	7356	7	Human papillomavirus 180	NA	Viruses; Monodnaviria; Shotokuvirae; Cossaviricota; Papovaviricetes; Zurhausenvirales; Papillomaviridae; Firstpapillomavirinae; Gammapapillomavirus; Gammapapillomavirus 10
GCA_000955375.1	5077	8	Uncultured phage WWnAnB strain 2	NA	Viruses; ssDNA viruses; Inoviridae; environmental samples
GCA_000954655.1	5210	8	Uncultured phage WWnAnB strain 3	NA	Viruses; ssDNA viruses; Inoviridae; environmental samples
GCA_000917015.1	142094	207	Staphylococcus phage phiSA12	phiSA012	Viruses; dsDNA viruses, no RNA stage; Caudovirales; Myoviridae; Spounavirinae; Kayvirus; Staphylococcus virus SA12
GCA_001736915.1	41871	52	Pseudomonas phage phi2	NA	Viruses; dsDNA viruses, no RNA stage; Caudovirales; Siphoviridae; unclassified Siphoviridae
GCA_000916835.1	610033	467	Pithovirus sibericum	NA	Viruses; unclassified viruses; unclassified DNA viruses; unclassified dsDNA viruses; Pithoviridae; Pithovirus
GCA_000924495.1	15480	7	Sosuga virus	NA	Viruses; ssRNA viruses; ssRNA negative-strand viruses; Mononegavirales; Paramyxoviridae; Rubulavirus; unclassified Rubulavirus
GCA_000915315.1	8305	1	Caprine kobuvirus	NA	Viruses; ssRNA viruses; ssRNA positive-strand viruses, no DNA stage; Picornavirales; Picornaviridae; Kobuvirus; unclassified Kobuvirus
GCA_001430095.1	122522	87	Felis catus gammaherpesvirus 1	NA	Viruses; dsDNA viruses, no RNA stage; Herpesvirales; Herpesviridae; Gammaherpesvirinae; unclassified Gammaherpesvirinae
GCA_006458505.1	206527	183	Ectromelia virus ERPV	NA	Viruses; Varidnaviria; Bamfordvirae; Nucleocytoviricota; Pokkesviricetes; Chitovirales; Poxviridae; Chordopoxvirinae; Orthopoxvirus; Ectromelia virus
GCA_000926155.1	1002	3	Faba bean necrotic yellows C1 alphasatellite	NA	Viruses; Satellites; Satellite Nucleic Acids; Single stranded DNA satellites; Alphasatellites; Babu- and nanovirus-associated alphasatellites

gca	genome.size	proteome.size	phage.name	strain	Taxonomy
GCA_000922135.1	1004	1	Faba bean necrotic yellows C11 alphasatellite	NA	Viruses; Satellites; Satellite Nucleic Acids; Single stranded DNA satellites; Alphasatellites; Babu- and nanovirus-associated alphasatellites
GCA_000921295.1	1015	1	Faba bean necrotic yellows C7 alphasatellite	NA	Viruses; Satellites; Satellite Nucleic Acids; Single stranded DNA satellites; Alphasatellites; Babu- and nanovirus-associated alphasatellites
GCA_000923815.1	1007	1	Faba bean necrotic yellows C9 alphasatellite	NA	Viruses; Satellites; Satellite Nucleic Acids; Single stranded DNA satellites; Alphasatellites; Babu- and nanovirus-associated alphasatellites
GCA_002630005.1	65729	92	Pseudomonas phage SPM1	NA	Viruses; Duplodnaviria; Heunggongvirae; Uroviricota; Caudoviricetes; Caudovirales; Myoviridae; Pbunavirus; Pseudomonas virus F8
GCA_000914715.1	17869	21	Staphylococcus phage GRCS	NA	Viruses; dsDNA viruses, no RNA stage; Caudovirales; Podoviridae; Picovirinae; P68virus; unclassified P68virus
GCA_003032555.1	17427	3	Burana virus	760	Viruses; Riboviria; Orthornavirae; Negarnaviricota; Polyploviricotina; Ellioviricetes; Bunyavirales; Nairoviridae; Orthonairovirus; Tamdy orthonairovirus
GCA_014883195.1	17523	3	Caspiy virus	LEIV63Az	Viruses; Riboviria; Orthornavirae; Negarnaviricota; Polyploviricotina; Ellioviricetes; Bunyavirales; Nairoviridae; Orthonairovirus; unclassified Nairovirus
GCA_014883205.1	18045	3	Geran virus	LEIV10899Az	Viruses; Riboviria; Orthornavirae; Negarnaviricota; Polyploviricotina; Ellioviricetes; Bunyavirales; Nairoviridae; Orthonairovirus; unclassified Nairovirus
GCA_014883185.1	19289	3	IssykKul virus	LEIV315K	Viruses; Riboviria; Orthornavirae; Negarnaviricota; Polyploviricotina; Ellioviricetes; Bunyavirales; Nairoviridae; Orthonairovirus; unclassified Nairovirus
GCA_014883335.1	18785	3	Tamdy virus	XJ01	Viruses; Riboviria; Orthornavirae; Negarnaviricota; Polyploviricotina; Ellioviricetes; Bunyavirales; Nairoviridae; Orthonairovirus; unclassified Nairovirus
GCA_000916895.1	2668	2	Dragonfly larvae associated circular virus1	NA	Viruses; ssDNA viruses; unclassified ssDNA viruses
GCA_000914355.1	2259	2	Dragonfly larvae associated circular virus10	NA	Viruses; ssDNA viruses; unclassified ssDNA viruses
GCA_000916035.1	2398	2	Dragonfly larvae associated circular virus2	NA	Viruses; ssDNA viruses; unclassified ssDNA viruses
GCA_000914335.1	2249	2	Dragonfly larvae associated circular virus3	NA	Viruses; ssDNA viruses; unclassified ssDNA viruses
GCA_000915375.1	2121	2	Dragonfly larvae associated circular virus4	NA	Viruses; ssDNA viruses; unclassified ssDNA viruses
GCA_000916875.1	1960	2	Dragonfly larvae associated circular virus5	NA	Viruses; ssDNA viruses; unclassified ssDNA viruses
GCA_000915355.1	1700	2	Dragonfly larvae associated circular virus6	NA	Viruses; ssDNA viruses; unclassified ssDNA viruses
GCA_000916015.1	2253	2	Dragonfly larvae associated circular virus7	NA	Viruses; ssDNA viruses; unclassified ssDNA viruses
GCA_000914315.1	1628	2	Dragonfly larvae associated circular virus8	NA	Viruses; ssDNA viruses; unclassified ssDNA viruses
GCA_000915335.1	1722	2	Dragonfly larvae associated circular virus9	NA	Viruses; ssDNA viruses; unclassified ssDNA viruses
GCA_000921975.1	9525	1	Simian pegivirus	SPgVkrc	Viruses; ssRNA viruses; ssRNA positive-strand viruses, no DNA stage; Flaviviridae; Pegivirus; Pegivirus A
GCA_000915415.1	1365	1	Ageratum yellow vein China alphasatellite	NA	Viruses; Satellites; Satellite Nucleic Acids; Single stranded DNA satellites; Alphasatellites; Begomovirus-associated alphasatellites; unclassified Begomovirus-associated alphasatellites
GCA_000916315.1	173276	294	Enterobacter phage PG7	NA	Viruses; dsDNA viruses, no RNA stage; Caudovirales; Myoviridae; Tevenvirinae; Cc31virus; Enterobacter virus PG7
GCA_000915535.1	39207	57	Citrobacter phage CR44b	NA	Viruses; dsDNA viruses, no RNA stage; Caudovirales; Podoviridae; Autographivirinae; unclassified Autographivirinae
GCA_000917035.1	39651	57	Citrobacter phage CR8	NA	Viruses; dsDNA viruses, no RNA stage; Caudovirales; Podoviridae; Autographivirinae; unclassified Autographivirinae
GCA_000915455.1	1007	1	Milk vetch dwarf C1 alphasatellite	NA	Viruses; Satellites; Satellite Nucleic Acids; Single stranded DNA satellites; Alphasatellites; Babu- and nanovirus-associated alphasatellites
GCA_000916635.1	1022	1	Milk vetch dwarf C10 alphasatellite	NA	Viruses; Satellites; Satellite Nucleic Acids; Single stranded DNA satellites; Alphasatellites; Babu- and nanovirus-associated alphasatellites
GCA_000916115.1	1009	1	Milk vetch dwarf C2 alphasatellite	NA	Viruses; Satellites; Satellite Nucleic Acids; Single stranded DNA satellites; Alphasatellites; Babu- and nanovirus-associated alphasatellites
GCA_000914415.1	1000	1	Milk vetch dwarf C3 alphasatellite	NA	Viruses; Satellites; Satellite Nucleic Acids; Single stranded DNA satellites; Alphasatellites; Babu- and nanovirus-associated alphasatellites
GCA_000838865.1	4594	11	Bdellovibrio phage phiMH2K	NA	Viruses; ssDNA viruses; Microviridae; Gokushovirinae; Bdellomicrovirus
GCA_000915395.1	10688	1	Kama virus	LEIV20776Tat	Viruses; ssRNA viruses; ssRNA positive-strand viruses, no DNA stage; Flaviviridae; Flavivirus; unclassified Flavivirus
GCA_000916075.1	9023	3	Wallerfield virus	TR7904	Viruses; ssRNA viruses; ssRNA positive-strand viruses, no DNA stage; unclassified ssRNA positive-strand viruses; Negevirus
GCA_002814615.1	10812	3	Cumuto virus	TR7904	Viruses; Riboviria; Orthornavirae; Negarnaviricota; Polyploviricotina; Ellioviricetes; Bunyavirales; Phenuiviridae; Goukovirus; Cumuto goukovirus
GCA_001550565.2	12429	4	Arrabida virus	PoSFPhlebV1262008	Viruses; ssRNA viruses; ssRNA negative-strand viruses; Bunyaviridae; Phlebovirus; unclassified Phlebovirus

gca	genome.size	proteome.size	phage.name	strain	Taxonomy
GCA_001271075.2	11367	4	Hunter Island virus	NA	Viruses; ssRNA viruses; ssRNA negative-strand viruses; Bunyaviridae; unclassified Bunyaviridae
GCA_002149165.1	1022	1	Subterranean clover stunt C2 alphasatellite	NA	Viruses; Satellites; Satellite Nucleic Acids; Single stranded DNA satellites; Alphasatellites; Babu- and nanovirus-associated alphasatellites; unclassified Babu- and nanovirus-associated alphasatellites
GCA_002149265.1	1017	1	Subterranean clover stunt C6 alphasatellite	NA	Viruses; Satellites; Satellite Nucleic Acids; Single stranded DNA satellites; Alphasatellites; Babu- and nanovirus-associated alphasatellites; unclassified Babu- and nanovirus-associated alphasatellites
GCA_000859285.1	4270	3	Human picobirnavirus	Hy005102	Viruses; dsRNA viruses; Picobirnaviridae; Picobirnavirus
GCA_000915715.1	43050	57	Acinetobacter phage IME_AB3	NA	Viruses; dsDNA viruses, no RNA stage; Caudovirales; Siphoviridae; unclassified Siphoviridae
GCA_000917955.1	59254	120	Microbacterium phage vB_MoxSISF9	NA	Viruses; dsDNA viruses, no RNA stage; Caudovirales; Siphoviridae; unclassified Siphoviridae
GCA_000917175.1	56024	95	Rhizobium phage vB_RglS_P106B	NA	Viruses; dsDNA viruses, no RNA stage; Caudovirales; Siphoviridae; unclassified Siphoviridae
GCA_001470555.1	61035	87	Escherichia phage phi191	NA	Viruses; dsDNA viruses, no RNA stage; Caudovirales; Podoviridae; unclassified Podoviridae; Escherichia phage phi191 sensu lato
GCA_002755935.1	68662	103	Mycobacterium phage Audrey	NA	Viruses; Duplodnaviria; Heunggongvirae; Uroviricota; Caudoviricetes; Caudovirales; Siphoviridae; Bclasvirinae; Pipefishvirus; unclassified Pipefishvirus
GCA_001470175.1	69030	102	Mycobacterium phage Badfish	NA	Viruses; dsDNA viruses, no RNA stage; Caudovirales; Siphoviridae; unclassified Siphoviridae; PG1-like phages
GCA_000920975.1	67383	104	Mycobacterium phage Hawkeye	NA	Viruses; dsDNA viruses, no RNA stage; Caudovirales; Siphoviridae; unclassified Siphoviridae
GCA_002755955.1	68628	103	Mycobacterium phage Heathcliff	NA	Viruses; Duplodnaviria; Heunggongvirae; Uroviricota; Caudoviricetes; Caudovirales; Siphoviridae; Bclasvirinae; Pipefishvirus; unclassified Pipefishvirus
GCA_002755975.1	68607	102	Mycobacterium phage Numberten	NA	Viruses; Duplodnaviria; Heunggongvirae; Uroviricota; Caudoviricetes; Caudovirales; Siphoviridae; Bclasvirinae; Pegunavirus; Mycobacterium virus Suffolk
GCA_000918335.1	51781	94	Mycobacterium phage Seabiscuit	NA	Viruses; dsDNA viruses, no RNA stage; Caudovirales; Siphoviridae; unclassified Siphoviridae
GCA_001470715.1	68735	103	Mycobacterium phage Swish	NA	Viruses; dsDNA viruses, no RNA stage; Caudovirales; Siphoviridae; unclassified Siphoviridae; PG1-like phages
GCA_000918975.1	66316	91	Vibrio phage CHOED	NA	Viruses; dsDNA viruses, no RNA stage; Caudovirales; Podoviridae; unclassified Podoviridae
GCA_000917135.1	53396	90	Mycobacterium phage 40AC	NA	Viruses; dsDNA viruses, no RNA stage; Caudovirales; Siphoviridae; unclassified Siphoviridae
GCA_000919595.1	52136	98	Mycobacterium phage Alsfro	NA	Viruses; dsDNA viruses, no RNA stage; Caudovirales; Siphoviridae; unclassified Siphoviridae
GCA_000925895.1	67744	98	Mycobacterium phage Soto	NA	Viruses; dsDNA viruses, no RNA stage; Caudovirales; Siphoviridae; unclassified Siphoviridae; PG1-like phages; Mycobacterium phage Oosterbaan
GCA_000916335.1	53124	96	Mycobacterium phage 20ES	NA	Viruses; dsDNA viruses, no RNA stage; Caudovirales; Siphoviridae; unclassified Siphoviridae
GCA_000917155.1	44306	62	Mycobacterium phage Jolie2	NA	Viruses; dsDNA viruses, no RNA stage; Caudovirales; Siphoviridae; unclassified Siphoviridae
GCA_000914655.1	52963	94	Mycobacterium phage CRB1	NA	Viruses; dsDNA viruses, no RNA stage; Caudovirales; Siphoviridae; unclassified Siphoviridae
GCA_000914595.1	39078	62	Geobacillus phage GBK2	NA	Viruses; dsDNA viruses, no RNA stage; Caudovirales; Siphoviridae; unclassified Siphoviridae
GCA_000916255.2	98287	170	Pseudomonas phage phiPsa374	NA	Viruses; dsDNA viruses, no RNA stage; Caudovirales; Myoviridae; unclassified Myoviridae
GCA_000914615.1	46739	73	Mycobacterium phage RhynO	NA	Viruses; dsDNA viruses, no RNA stage; Caudovirales; Siphoviridae; unclassified Siphoviridae
GCA_000922735.2	72583	94	Escherichia phage Bp4	NA	Viruses; dsDNA viruses, no RNA stage; Caudovirales; Podoviridae; Picovirinae; G7cvirus; Escherichia virus Bp4
GCA_000917915.1	152460	197	Salmonella phage vB_SalM_SJ2	NA	Viruses; dsDNA viruses, no RNA stage; Caudovirales; Myoviridae; unclassified Myoviridae
GCA_000920875.1	55726	88	Enterococcus phage VD13	NA	Viruses; dsDNA viruses, no RNA stage; Caudovirales; Siphoviridae; Sap6virus
GCA_000921995.1	41156	73	Listeria phage LP0303	NA	Viruses; dsDNA viruses, no RNA stage; Caudovirales; Siphoviridae; unclassified Siphoviridae
GCA_002604385.1	135279	188	Listeria phage LP064	NA	Viruses; Duplodnaviria; Heunggongvirae; Uroviricota; Caudoviricetes; Caudovirales; Herelleviridae; Jasinkavirinae; Pecentumvirus; Listeria virus LP064
GCA_000923695.1	135831	189	Listeria phage LP0832	NA	Viruses; dsDNA viruses, no RNA stage; Caudovirales; Myoviridae; Spounavirinae; P100virus; Listeria virus P100
GCA_000923075.1	43767	70	Listeria phage LP101	NA	Viruses; dsDNA viruses, no RNA stage; Caudovirales; Siphoviridae; unclassified Siphoviridae
GCA_000921155.1	66676	116	Listeria phage LP114	NA	Viruses; dsDNA viruses, no RNA stage; Caudovirales; Siphoviridae; P70virus; Listeria virus LP114
GCA_002604425.1	42252	63	Flavobacterium sp phage 132	NA	Viruses; Duplodnaviria; Heunggongvirae; Uroviricota; Caudoviricetes; Caudovirales; Siphoviridae; unclassified Siphoviridae

gca	genome.size	proteome.size	phage.name	strain	Taxonomy
GCA_000925035.1	133824	235	Shewanella sp phage 14	NA	Viruses; Duplodnaviria; Heunggongvirae; Uroviricota; Caudoviricetes; Caudovirales; Myoviridae; unclassified Myoviridae
GCA_000927595.1	139004	236	Shewanella sp phage 140	NA	Viruses; Duplodnaviria; Heunggongvirae; Uroviricota; Caudoviricetes; Caudovirales; Myoviridae; unclassified Myoviridae
GCA_000927535.1	43510	69	Shewanella sp phage 141	NA	Viruses; Duplodnaviria; Heunggongvirae; Uroviricota; Caudoviricetes; Caudovirales; Myoviridae; unclassified Myoviridae
GCA_000929495.1	49640	75	Shewanella sp phage 144	NA	Viruses; Duplodnaviria; Heunggongvirae; Uroviricota; Caudoviricetes; Caudovirales; Siphoviridae; unclassified Siphoviridae
GCA_000925875.1	40161	70	Shewanella sp phage 349	NA	Viruses; Duplodnaviria; Heunggongvirae; Uroviricota; Caudoviricetes; Caudovirales; Siphoviridae; unclassified Siphoviridae
GCA_000926995.1	11176	5	Long Island tick rhabdovirus	LS1	Viruses; ssRNA viruses; ssRNA negative-strand viruses; Mononegavirales; Rhabdoviridae; unclassified Rhabdoviridae
GCA_000916095.1	7435	4	Hibiscus bacilliform virus GD1	NA	Viruses; Riboviria; Pararnavirae; Artverviricota; Revtraviricetes; Ortervirales; Caulimoviridae; Badnavirus; unclassified Badnavirus
GCA_002676835.1	18487	11	Rotavirus RVAHumanwtUSA12US11342012G3P9	NA	Viruses; Riboviria; Orthornavirae; Duplornaviricota; Resentoviricetes; Reovirales; Reoviridae; Sedoreovirinae; Rotavirus; Rotavirus A; Rotavirus G3
GCA_000915515.1	8460	16	Ralstonia phage 1 NP2014	RIP1	Viruses; ssDNA viruses; Inoviridae; unclassified Inoviridae
GCA_000916935.1	10176	1	Antheraea pernyi iflavirus	NA	Viruses; ssRNA viruses; ssRNA positive-strand viruses, no DNA stage; Picornavirales; Iflaviridae; Iflavirus
GCA_000905835.1	180421	113	Elephantid betaherpesvirus 1	Raman	Viruses; dsDNA viruses, no RNA stage; Herpesvirales; Herpesviridae; Betaherpesvirinae; Proboscivirus
GCA_000837325.1	1821	4	Goose circovirus	NA	Viruses; ssDNA viruses; Circoviridae; Circovirus
GCA_014514555.1	100180	176	Pseudomonas phage phiPsa267	NA	Viruses; Duplodnaviria; Heunggongvirae; Uroviricota; Caudoviricetes; Caudovirales; Myoviridae; Otagovirus; unclassified Otagovirus
GCA_014514585.1	99272	171	Pseudomonas phage phiPsa300	NA	Viruses; Duplodnaviria; Heunggongvirae; Uroviricota; Caudoviricetes; Caudovirales; Myoviridae; Otagovirus; unclassified Otagovirus
GCA_014514595.1	98739	172	Pseudomonas phage phiPsa315	NA	Viruses; Duplodnaviria; Heunggongvirae; Uroviricota; Caudoviricetes; Caudovirales; Myoviridae; Otagovirus; unclassified Otagovirus
GCA_014514655.1	99689	174	Pseudomonas phage phiPsa347	NA	Viruses; Duplodnaviria; Heunggongvirae; Uroviricota; Caudoviricetes; Caudovirales; Myoviridae; Otagovirus; unclassified Otagovirus
GCA_014514695.1	98800	173	Pseudomonas phage phiPsa381	NA	Viruses; Duplodnaviria; Heunggongvirae; Uroviricota; Caudoviricetes; Caudovirales; Myoviridae; Otagovirus; unclassified Otagovirus
GCA_014514725.1	98951	173	Pseudomonas phage phiPsa397	NA	Viruses; Duplodnaviria; Heunggongvirae; Uroviricota; Caudoviricetes; Caudovirales; Myoviridae; Otagovirus; unclassified Otagovirus
GCA_000916155.1	5075	2	Ustilaginoidea virens RNA virus 3	NA	Viruses; dsRNA viruses; Totiviridae; Victorivirus; unclassified Victorivirus
GCA_013086575.1	11758	4	Gordil virus	NA	Viruses; Riboviria; Orthornavirae; Negarnaviricota; Polyploviricotina; Ellioviricetes; Bunyavirales; Phenuiviridae; Phlebovirus; Gordil phlebovirus
GCA_000852525.1	8289	3	Walrus calicivirus	NA	Viruses; ssRNA viruses; ssRNA positive-strand viruses, no DNA stage; Caliciviridae; Vesivirus; Vesicular exanthema of swine virus
GCA_002966455.1	1181380	971	Samba virus	NA	Viruses; Varidnaviria; Bamfordvirae; Nucleocytoviricota; Megaviricetes; Imitervirales; Mimiviridae; Mimivirus; Acanthamoeba polyphaga mimivirus
GCA_002604445.1	41323	63	Streptococcus phage Spn1	NA	Viruses; Duplodnaviria; Heunggongvirae; Uroviricota; Caudoviricetes; Caudovirales; Siphoviridae; unclassified Siphoviridae
GCA_006443875.1	34780	40	Human adenovirus 1134	IC763	Viruses; Varidnaviria; Bamfordvirae; Preplasmiviricota; Tectiliviricetes; Rowavirales; Adenoviridae; Mastadenovirus; Human mastadenovirus B; Human adenovirus B2
GCA_000915635.1	41529	21	Vibrio phage PVA1	NA	Viruses; dsDNA viruses, no RNA stage; Caudovirales; Podoviridae; unclassified Podoviridae
GCA_000918115.1	1344	1	Malvastrum leaf curl Guangdong betasatellite	NA	Viruses; Tolecusatellitidae; Betasatellite
GCA_002633165.1	62774	95	Pseudomonas phage KPP23	NA	Viruses; Duplodnaviria; Heunggongvirae; Uroviricota; Caudoviricetes; Caudovirales; Siphoviridae; unclassified Siphoviridae
GCA_000918235.1	64113	88	Pseudomonas phage KPP25	NA	Viruses; dsDNA viruses, no RNA stage; Caudovirales; Podoviridae; unclassified Podoviridae
GCA_001654125.1	5813	7	Pepo aphidborne yellows virus	NA	Viruses; ssRNA viruses; ssRNA positive-strand viruses, no DNA stage; Luteoviridae; Polerovirus; unclassified Polerovirus
GCA_001271315.1	5961	6	Luffa aphidborne yellows virus	NA	Viruses; ssRNA viruses; ssRNA positive-strand viruses, no DNA stage; Luteoviridae; Polerovirus; unclassified Polerovirus
GCA_000866745.1	36466	51	Lactobacillus phage LcNu	NA	Viruses; dsDNA viruses, no RNA stage; Caudovirales; Siphoviridae; unclassified Siphoviridae
GCA_002601625.1	7325	13	Xanthomonas phage XacF1	NA	Viruses; Monodnaviria; Loebvirae; Hofneiviricota; Faserviricetes; Tubulavirales; Inoviridae; Coriovirus; Xanthomonas virus Cf1c
GCA_000915615.1	71565	100	Mycobacterium phage 39HC	NA	Viruses; dsDNA viruses, no RNA stage; Caudovirales; Siphoviridae; unclassified Siphoviridae
GCA_002604465.1	71565	100	Mycobacterium phage 40BC	NA	Viruses; Duplodnaviria; Heunggongvirae; Uroviricota; Caudoviricetes; Caudovirales; Siphoviridae; Bclasvirinae; unclassified Bclasvirinae

gca	genome.size	proteome.size	phage.name	strain	Taxonomy
GCA_000922775.1	70667	97	Mycobacterium phage Hosp	NA	Viruses; dsDNA viruses, no RNA stage; Caudovirales; Siphoviridae; unclassified Siphoviridae
GCA_000916275.1	71058	98	Mycobacterium phage Jolie1	NA	Viruses; dsDNA viruses, no RNA stage; Caudovirales; Siphoviridae; unclassified Siphoviridae
GCA_000922895.1	8132	12	Fulmarus glacialis papillomavirus 1	NA	Viruses; dsDNA viruses, no RNA stage; Papillomaviridae; Treiszetapapillomavirus; Treiszetapapillomavirus 1
GCA_002826905.1	7668	6	Eptesicus serotinus papillomavirus 1	NA	Viruses; Monodnaviria; Shotokuvirae; Cossaviricota; Papovaviricetes; Zurhausenvirales; Papillomaviridae; Firstpapillomavirinae; Dyopsipapillomavirus; Dyopsipapillomavirus 1
GCA_002826865.1	7574	6	Eptesicus serotinus papillomavirus 2	NA	Viruses; Monodnaviria; Shotokuvirae; Cossaviricota; Papovaviricetes; Zurhausenvirales; Papillomaviridae; Firstpapillomavirinae; Dyoomegapapillomavirus; Dyoomegapapillomavirus 1
GCA_003179535.1	7711	6	Eptesicus serotinus papillomavirus 3	NA	Viruses; Monodnaviria; Shotokuvirae; Cossaviricota; Papovaviricetes; Zurhausenvirales; Papillomaviridae; Firstpapillomavirinae; Dyopsipapillomavirus; Dyopsipapillomavirus 1
GCA_002827105.1	8249	6	Rhinolophus ferrumequinum papillomavirus 1	NA	Viruses; Monodnaviria; Shotokuvirae; Cossaviricota; Papovaviricetes; Zurhausenvirales; Papillomaviridae; Firstpapillomavirinae; Treisdeltapapillomavirus; Treisdeltapapillomavirus 1
GCA_000923675.1	41941	62	Staphylococcus phage DW2	NA	Viruses; dsDNA viruses, no RNA stage; Caudovirales; Siphoviridae; unclassified Siphoviridae
GCA_000929575.1	8247	4	Cassava Ivorian bacilliform virus	SCRICV1	Viruses; ssRNA viruses; ssRNA positive-strand viruses, no DNA stage; Bromoviridae; Anulavirus; unclassified Anulavirus
GCA_000914575.1	10929	1	Laodelphax striatella honeydew virus 1	NA	Viruses; ssRNA viruses; ssRNA positive-strand viruses, no DNA stage; Picornavirales; Iflaviridae; unclassified Iflaviridae
GCA_000849365.1	6562	3	Cucumber fruit mottle mosaic virus	NA	Viruses; ssRNA viruses; ssRNA positive-strand viruses, no DNA stage; Virgaviridae; Tobamovirus
GCA_000856625.1	10035	1	Watermelon mosaic virus	WMVFr	Viruses; ssRNA viruses; ssRNA positive-strand viruses, no DNA stage; Potyviridae; Potyvirus
GCA_000920035.1	42764	60	Salmonella phage vB_SenSEnt3	NA	Viruses; dsDNA viruses, no RNA stage; Caudovirales; Siphoviridae; unclassified Siphoviridae
GCA_000917095.1	42093	56	Salmonella phage vB_SenSEnt2	NA	Viruses; dsDNA viruses, no RNA stage; Caudovirales; Siphoviridae; unclassified Siphoviridae
GCA_002921815.1	38756	51	Podovirus Lau218	TahiMoana	Viruses; dsDNA viruses, no RNA stage; Caudovirales; Podoviridae; unclassified Podoviridae
GCA_000918955.1	163023	131	Anopheles minimus irodovirus	NA	Viruses; dsDNA viruses, no RNA stage; Iridoviridae; unclassified Iridoviridae
GCA_000856785.1	4114	4	Oat chlorotic stunt virus	NA	Viruses; ssRNA viruses; ssRNA positive-strand viruses, no DNA stage; Tombusviridae; Avenavirus
GCA_000918295.1	53255	96	Mycobacterium phage Lamina13	NA	Viruses; dsDNA viruses, no RNA stage; Caudovirales; Siphoviridae; unclassified Siphoviridae
GCA_000919115.1	8659	6	Gaillardia latent virus	NA	Viruses; ssRNA viruses; ssRNA positive-strand viruses, no DNA stage; Tymovirales; Betaflexiviridae; Quinvirinae; Carlavirus
GCA_002604485.1	79178	129	Synechococcus phage SEIVl	NA	Viruses; unclassified bacterial viruses
GCA_000920815.1	56702	71	Enterobacteria phage 9g	NA	Viruses; dsDNA viruses, no RNA stage; Caudovirales; Siphoviridae; Nonagvirus; Escherichia virus 9g
GCA_000918455.1	15088	1	Lagenaria siceraria endornavirusCalifornia	NA	Viruses; dsRNA viruses; Endornaviridae; Endornavirus; unclassified Endornavirus
GCA_001962155.1	1370	1	Ageratum yellow vein Pakistan alphasatellite	NA	Viruses; Satellites; Satellite Nucleic Acids; Single stranded DNA satellites; Alphasatellites; Begomovirus-associated alphasatellites; unclassified Begomovirus-associated alphasatellites
GCA_001963675.1	1375	1	Hibiscus leaf curl alphasatellite	NA	Viruses; Satellites; Satellite Nucleic Acids; Single stranded DNA satellites; Alphasatellites; Begomovirus-associated alphasatellites; unclassified Begomovirus-associated alphasatellites
GCA_002867895.1	3690	2	Heterobasidion partitivirus 12	HetPV12an1	Viruses; Riboviria; Orthornavirae; Pisuviricota; Duplopiviricetes; Durnavirales; Partitiviridae; Alphapartitivirus
GCA_002867915.1	3649	2	Heterobasidion partitivirus 13	HetPV13an1	Viruses; Riboviria; Orthornavirae; Pisuviricota; Duplopiviricetes; Durnavirales; Partitiviridae; Alphapartitivirus
GCA_002867995.1	3673	2	Heterobasidion partitivirus 15	HetPV15pa1	Viruses; Riboviria; Orthornavirae; Pisuviricota; Duplopiviricetes; Durnavirales; Partitiviridae; Alphapartitivirus
GCA_000918875.1	5006	3	Danaus plexippus plexippus iteravirus	NA	Viruses; ssDNA viruses; Parvoviridae; Densovirinae; Iteradensovirus; unclassified Iteravirus
GCA_002755995.1	37940	60	Campylobacter phage CJIE41	NA	Viruses; Duplodnaviria; Heunggongvirae; Uroviricota; Caudoviricetes; Caudovirales; Siphoviridae; unclassified Siphoviridae
GCA_002756015.1	36734	58	Campylobacter phage CJIE42	NA	Viruses; Duplodnaviria; Heunggongvirae; Uroviricota; Caudoviricetes; Caudovirales; Siphoviridae; unclassified Siphoviridae
GCA_002756035.1	35417	58	Campylobacter phage CJIE43	NA	Viruses; Duplodnaviria; Heunggongvirae; Uroviricota; Caudoviricetes; Caudovirales; Siphoviridae; unclassified Siphoviridae
GCA_002756055.1	35459	58	Campylobacter phage CJIE44	NA	Viruses; Duplodnaviria; Heunggongvirae; Uroviricota; Caudoviricetes; Caudovirales; Siphoviridae; unclassified Siphoviridae
GCA_002756075.1	36991	59	Campylobacter phage CJIE45	NA	Viruses; Duplodnaviria; Heunggongvirae; Uroviricota; Caudoviricetes; Caudovirales; Siphoviridae; unclassified Siphoviridae
GCA_001019755.1	6988	4	Grapevine roditis leaf discolorationassociated virus	NA	Viruses; Retro-transcribing viruses; Caulimoviridae; Badnavirus

gca	genome.size	proteome.size	phage.name	strain	Taxonomy
GCA_000919975.1	65243	111	Mycobacterium phage MosMoris	NA	Viruses; dsDNA viruses, no RNA stage; Caudovirales; Siphoviridae; unclassified Siphoviridae
GCA_002756095.1	53746	84	Mycobacterium phage HH92	NA	Viruses; Duplodnaviria; Heunggongvirae; Uroviricota; Caudoviricetes; Caudovirales; Siphoviridae; Gilesvirus; Mycobacterium virus Giles
GCA_002756115.1	68043	100	Mycobacterium phage KingVeVeVe	NA	Viruses; Duplodnaviria; Heunggongvirae; Uroviricota; Caudoviricetes; Caudovirales; Siphoviridae; Bclasvirinae; Pegunavirus; Mycobacterium virus Soto
GCA_000912535.1	7228	6	Human papillomavirus type 179	NA	Viruses; dsDNA viruses, no RNA stage; Papillomaviridae; unclassified Papillomaviridae; Human papillomavirus types
GCA_002987365.1	7324	6	Human papillomavirus 184	NA	Viruses; Monodnaviria; Shotokuvirae; Cossaviricota; Papovaviricetes; Zurhausenvirales; Papillomaviridae; Firstpapillomavirinae; Gammapapillomavirus; Gammapapillomavirus 25
GCA_000922315.1	6835	3	Prunus virus T	NA	Viruses; ssRNA viruses; ssRNA positive-strand viruses, no DNA stage; Tymovirales; Betaflexiviridae; Trivirinae; Tepovirus
GCA_000919095.1	8194	7	Enhydra lutris papillomavirus 1	NA	Viruses; dsDNA viruses, no RNA stage; Papillomaviridae; Lambdapapillomavirus; unclassified Lambdapapillomavirus
GCA_000920435.1	6148	2	Porcine parvovirus 6	NA	Viruses; ssDNA viruses; Parvoviridae; Parvovirinae; Protoparvovirus; unclassified Protoparvovirus
GCA_000921795.1	82438	144	Nitrincola phage 1M316	NA	Viruses; dsDNA viruses, no RNA stage; Caudovirales; unclassified Caudovirales
GCA_000926555.1	18406	9	Mojiang virus	NA	Viruses; ssRNA viruses; ssRNA negative-strand viruses; Mononegavirales; Paramyxoviridae; Henipavirus; unclassified Henipavirus
GCA_000922335.1	370920	376	Aureococcus anophagefferens virus	NA	Viruses; dsDNA viruses, no RNA stage; Phycodnaviridae; unclassified Phycodnaviridae
GCA_000919035.1	2980	2	Porcine stoolassociated circular virus 4	NA	Viruses; ssDNA viruses; Circoviridae; unclassified Circoviridae
GCA_000919675.1	3062	2	Porcine stoolassociated circular virus 5	NA	Viruses; ssDNA viruses; Circoviridae; unclassified Circoviridae
GCA_000918015.1	1753	2	Mink circovirus	MiCVDL13	Viruses; ssDNA viruses; Circoviridae; Circovirus
GCA_000922215.1	6286	2	Rosellinia necatrix fusarivirus 1	NW10	Viruses; ssRNA viruses; unclassified ssRNA viruses; Fusariviridae
GCA_000923035.1	43056	52	Pseudomonas phage vB_PaeS_SCH_Ab26	NA	Viruses; dsDNA viruses, no RNA stage; Caudovirales; Siphoviridae; Septima3virus
GCA_000918275.1	72028	83	Pseudomonas phage vB_PaeP_C210_Ab09	NA	Viruses; dsDNA viruses, no RNA stage; Caudovirales; Podoviridae; Lit1virus; Pseudomonas virus Ab09
GCA_000920515.2	1379	1	Andrographis yellow vein leaf curl betasatellite	NA	Viruses; Tolecusatellitidae; Betasatellite
GCA_000920415.1	2858	4	Axonopus compressus streak virus	NA	Viruses; ssDNA viruses; Geminiviridae; Mastrevirus; unclassified Mastrevirus
GCA_001503195.2	9895	2	Currant latent virus	NA	Viruses; ssRNA viruses; ssRNA positive-strand viruses, no DNA stage; Picornavirales; Secoviridae; Cheravirus; unclassified Cheravirus
GCA_008798015.1	7148	1	Human rhinovirus A28	NA	Viruses; Riboviria; Orthornavirae; Pisuviricota; Pisoniviricetes; Picornavirales; Picornaviridae; Enterovirus; Rhinovirus A
GCA_008798075.1	7141	1	Human rhinovirus A36	NA	Viruses; Riboviria; Orthornavirae; Pisuviricota; Pisoniviricetes; Picornavirales; Picornaviridae; Enterovirus; Rhinovirus A
GCA_008797095.1	7119	1	Human rhinovirus A18	ATCC VR1128	Viruses; Riboviria; Orthornavirae; Pisuviricota; Pisoniviricetes; Picornavirales; Picornaviridae; Enterovirus; Rhinovirus A
GCA_008797055.1	7163	1	Human rhinovirus A20	ATCC VR1130	Viruses; Riboviria; Orthornavirae; Pisuviricota; Pisoniviricetes; Picornavirales; Picornaviridae; Enterovirus; Rhinovirus A
GCA_008796785.1	7114	1	Human rhinovirus A45	ATCC VR1155	Viruses; Riboviria; Orthornavirae; Pisuviricota; Pisoniviricetes; Picornavirales; Picornaviridae; Enterovirus; Rhinovirus A
GCA_008796045.1	7216	1	Human rhinovirus B52	ATCC VR1162	Viruses; Riboviria; Orthornavirae; Pisuviricota; Pisoniviricetes; Picornavirales; Picornaviridae; Enterovirus; Rhinovirus B
GCA_008796445.1	7211	1	Rhinovirus B69	ATCC VR1179	Viruses; Riboviria; Orthornavirae; Pisuviricota; Pisoniviricetes; Picornavirales; Picornaviridae; Enterovirus; Rhinovirus B
GCA_008798395.1	7214	1	Rhinovirus B48	NA	Viruses; Riboviria; Orthornavirae; Pisuviricota; Pisoniviricetes; Picornavirales; Picornaviridae; Enterovirus; Rhinovirus B
GCA_008797575.1	7216	1	Human rhinovirus B37	NA	Viruses; Riboviria; Orthornavirae; Pisuviricota; Pisoniviricetes; Picornavirales; Picornaviridae; Enterovirus; Rhinovirus B
GCA_008798435.1	7216	1	Human rhinovirus B6	NA	Viruses; Riboviria; Orthornavirae; Pisuviricota; Pisoniviricetes; Picornavirales; Picornaviridae; Enterovirus; Rhinovirus B
GCA_008796825.1	7223	1	Human rhinovirus B42	ATCC VR338	Viruses; Riboviria; Orthornavirae; Pisuviricota; Pisoniviricetes; Picornavirales; Picornaviridae; Enterovirus; Rhinovirus B
GCA_008797695.1	7124	1	Human rhinovirus A12	NA	Viruses; Riboviria; Orthornavirae; Pisuviricota; Pisoniviricetes; Picornavirales; Picornaviridae; Enterovirus; Rhinovirus A
GCA_008797635.1	7145	1	Human rhinovirus A78	NA	Viruses; Riboviria; Orthornavirae; Pisuviricota; Pisoniviricetes; Picornavirales; Picornaviridae; Enterovirus; Rhinovirus A
GCA_000920955.1	54452	78	Lactococcus phage P078	NA	Viruses; dsDNA viruses, no RNA stage; Caudovirales; Siphoviridae; unclassified Siphoviridae

gca	genome.size	proteome.size	phage.name	strain	Taxonomy
GCA_000920915.1	53513	76	Lactococcus phage P092	NA	Viruses; dsDNA viruses, no RNA stage; Caudovirales; Siphoviridae; unclassified Siphoviridae
GCA_000922855.1	54173	80	Lactococcus phage P118	NA	Viruses; dsDNA viruses, no RNA stage; Caudovirales; Siphoviridae; unclassified Siphoviridae
GCA_000923495.1	55584	82	Lactococcus phage P162	NA	Viruses; dsDNA viruses, no RNA stage; Caudovirales; Siphoviridae; unclassified Siphoviridae
GCA_002604685.1	41476	67	Vibrio phage phi 2	NA	Viruses; Duplodnaviria; Heunggongvirae; Uroviricota; Caudoviricetes; Caudovirales; Myoviridae; unclassified Myoviridae
GCA_000858465.1	7650	3	Banana streak MY virus	NA	Viruses; Riboviria; Pararnavirae; Artverviricota; Revtraviricetes; Ortervirales; Caulimoviridae; Badnavirus
GCA_008787185.1	6843	1	Rhinovirus A	HRVA24_p1025_sR2625_2009	Viruses; ssRNA viruses; ssRNA positive-strand viruses, no DNA stage; Picornavirales; Picornaviridae; Enterovirus
GCA_008797155.1	7212	1	Human rhinovirus B5	ATCC VR485	Viruses; Riboviria; Orthornavirae; Pisuviricota; Pisoniviricetes; Picornavirales; Picornaviridae; Enterovirus; Rhinovirus B
GCA_000923015.1	75028	86	Dinoroseobacter phage DFL12phi1	NA	Viruses; dsDNA viruses, no RNA stage; Caudovirales; Podoviridae; N4virus; unclassified N4likevirus
GCA_013187205.1	477407	514	Faustovirus	LCD7	Viruses; unclassified viruses
GCA_000924915.1	155346	196	Dickeya phage RC2014	NA	Viruses; dsDNA viruses, no RNA stage; Caudovirales; Myoviridae; unclassified Myoviridae
GCA_000918935.1	9566	1	Chicken megrivirus	chickenCHKIVCHV2013HUN	Viruses; ssRNA viruses; ssRNA positive-strand viruses, no DNA stage; Picornavirales; Picornaviridae; Megrivirus; unclassified Megrivirus
GCA_000918095.1	7314	7	Human papillomavirus 178	NA	Viruses; dsDNA viruses, no RNA stage; Papillomaviridae; Gammapapillomavirus; Gammapapillomavirus 24
GCA_000926695.1	11876	6	Puerto Almendras virus	NA	Viruses; ssRNA viruses; ssRNA negative-strand viruses; Mononegavirales; Rhabdoviridae; unclassified Rhabdoviridae
GCA_000920595.1	7637	7	Pygoscelis adeliae papillomavirus 1	NA	Viruses; dsDNA viruses, no RNA stage; Papillomaviridae; Treisepsilonpapillomavirus; Treisepsilonpapillomavirus 1
GCA_002604545.1	44541	44	Vibrio phage Vc1	NA	Viruses; Duplodnaviria; Heunggongvirae; Uroviricota; Caudoviricetes; Caudovirales; Autographiviridae; Colwellvirinae; Gutovirus; Vibrio virus Vc1
GCA_000920775.1	167266	275	Serratia phage PS2	NA	Viruses; dsDNA viruses, no RNA stage; Caudovirales; Myoviridae; Tevenvirinae; T4virus; unclassified T4virus
GCA_000927195.1	13534	6	Spodoptera frugiperda rhabdovirus	NA	Viruses; ssRNA viruses; ssRNA negative-strand viruses; Mononegavirales; Rhabdoviridae; unclassified Rhabdoviridae
GCA_000925015.1	99730	156	Acinetobacter phage vB_AbaM_Acibel004	NA	Viruses; dsDNA viruses, no RNA stage; Caudovirales; Myoviridae; unclassified Myoviridae
GCA_000927515.1	42654	53	Acinetobacter phage vB_AbaP_Acibel007	NA	Viruses; dsDNA viruses, no RNA stage; Caudovirales; Podoviridae; unclassified Podoviridae
GCA_000922915.1	37380	58	Pseudomonas phage JD024	NA	Viruses; dsDNA viruses, no RNA stage; Caudovirales; Siphoviridae; unclassified Siphoviridae
GCA_000918135.1	8398	1	Mosavirus A2	SZAL6MoV2011HUN	Viruses; ssRNA viruses; ssRNA positive-strand viruses, no DNA stage; Picornavirales; Picornaviridae; Mosavirus; Mosavirus A
GCA_001308355.1	12148	3	Tomato necrotic dwarf virus	NA	Viruses; ssRNA viruses; ssRNA positive-strand viruses, no DNA stage; Picornavirales; Secoviridae; Torradovirus; unclassified Torradovirus
GCA_001503995.1	44360	43	Ralstonia phage RSJ2	NA	Viruses; dsDNA viruses, no RNA stage; Caudovirales; Podoviridae; unclassified Podoviridae
GCA_000919795.1	19917	6	Casuarina virus	NA	Viruses; ssRNA viruses; ssRNA positive-strand viruses, no DNA stage; Nidovirales; Mesoniviridae; Alphamesonivirus; Alphamesonivirus 4
GCA_000919155.1	8976	1	Duck picornavirus GL12	NA	Viruses; ssRNA viruses; ssRNA positive-strand viruses, no DNA stage; Picornavirales; Picornaviridae; unclassified Picornaviridae
GCA_001551265.1	41941	56	Ralstonia phage RS138	NA	Viruses; unclassified phages
GCA_000838025.1	131405	135	Helicoverpa armigera nucleopolyhedrovirus G4	NA	Viruses; Baculoviridae; Alphabaculovirus; Helicoverpa armigera nucleopolyhedrovirus
GCA_000920635.1	7204	1	Tortoise rafivirus A	NA	Viruses; ssRNA viruses; ssRNA positive-strand viruses, no DNA stage; Picornavirales; Picornaviridae; unclassified Picornaviridae
GCA_000925535.1	10002	6	Sclerotinia sclerotiorum negativestranded RNA virus 1	AH98	Viruses; ssRNA viruses; ssRNA negative-strand viruses; Mononegavirales; Mymonaviridae; Sclerotimonavirus; Sclerotinia sclerotimonavirus
GCA_000881575.1	2757	6	Jatropha yellow mosaic virus	NA	Viruses; Monodnaviria; Shotokuvirae; Cressdnaviricota; Repensiviricetes; Geplafuvirales; Geminiviridae; Begomovirus
GCA_000920675.1	10891	1	Nhumirim virus	BrMSMQ10	Viruses; ssRNA viruses; ssRNA positive-strand viruses, no DNA stage; Flaviviridae; unclassified Flaviviridae
GCA_000922795.1	5327	2	Beauveria bassiana victorivirus NZL1980	NA	Viruses; dsRNA viruses; Totiviridae; Victorivirus; unclassified Victorivirus
GCA_000837245.1	50550	86	Mycobacterium virus Bxb1	NA	Viruses; Duplodnaviria; Heunggongvirae; Uroviricota; Caudoviricetes; Caudovirales; Siphoviridae; Fromanvirus
GCA_000922975.1	68807	101	Mycobacterium phage Manad	NA	Viruses; dsDNA viruses, no RNA stage; Caudovirales; Siphoviridae; unclassified Siphoviridae; PG1-like phages

gca	genome.size	proteome.size	phage.name	strain	Taxonomy
GCA_002598545.1	155476	233	Mycobacterium phage Willis	NA	Viruses; Duplodnaviria; Heunggongvirae; Uroviricota; Caudoviricetes; Caudovirales; Myoviridae; Bixzunavirus; Mycobacterium virus Bxz1
GCA_000924175.1	132541	177	Listeria phage LMTA148	NA	Viruses; dsDNA viruses, no RNA stage; Caudovirales; Myoviridae; unclassified Myoviridae
GCA_002756155.1	136589	196	Listeria phage LMTA57	NA	Viruses; Duplodnaviria; Heunggongvirae; Uroviricota; Caudoviricetes; Caudovirales; Herelleviridae; Jasinkavirinae; Pecentumvirus; Listeria virus LMSP25
GCA_002756175.1	136468	189	Listeria phage LMTA94	NA	Viruses; Duplodnaviria; Heunggongvirae; Uroviricota; Caudoviricetes; Caudovirales; Herelleviridae; Jasinkavirinae; Pecentumvirus; Listeria virus LMSP25
GCA_000923595.1	138036	187	Listeria phage LMSP25	NA	Viruses; dsDNA viruses, no RNA stage; Caudovirales; Myoviridae; unclassified Myoviridae
GCA_000923575.1	131952	174	Listeria phage List36	NA	Viruses; dsDNA viruses, no RNA stage; Caudovirales; Myoviridae; unclassified Myoviridae
GCA_000919955.1	18899	24	Lactococcus phage WP2	NA	Viruses; Duplodnaviria; Heunggongvirae; Uroviricota; Caudoviricetes; Caudovirales; Podoviridae; Picovirinae; Negarvirus; Lactococcus virus WP2
GCA_000919995.1	42392	60	Mycobacterium phage Bernal13	NA	Viruses; dsDNA viruses, no RNA stage; Caudovirales; Siphoviridae; unclassified Siphoviridae
GCA_000922755.1	51378	88	Mycobacterium phage Kampy	NA	Viruses; dsDNA viruses, no RNA stage; Caudovirales; Siphoviridae; unclassified Siphoviridae
GCA_000920855.1	50101	91	Mycobacterium phage Phantastic	NA	Viruses; dsDNA viruses, no RNA stage; Caudovirales; Siphoviridae; unclassified Siphoviridae
GCA_000920835.1	57315	92	Mycobacterium phage ZoeJ	NA	Viruses; dsDNA viruses, no RNA stage; Caudovirales; Siphoviridae; unclassified Siphoviridae
GCA_000927435.1	40286	59	Enterococcus phage EFC1	NA	Viruses; dsDNA viruses, no RNA stage; Caudovirales; Siphoviridae; unclassified Siphoviridae
GCA_000921955.1	68386	93	Mycobacterium phage Damien	NA	Viruses; dsDNA viruses, no RNA stage; Caudovirales; Siphoviridae; unclassified Siphoviridae
GCA_002604585.1	60310	100	Mycobacterium phage Emerson	NA	Viruses; Duplodnaviria; Heunggongvirae; Uroviricota; Caudoviricetes; Caudovirales; Siphoviridae; Timquatrovirus; unclassified Timquatrovirus
GCA_002756195.1	68428	101	Mycobacterium phage EmpTee	NA	Viruses; Duplodnaviria; Heunggongvirae; Uroviricota; Caudoviricetes; Caudovirales; Siphoviridae; Bclasvirinae; Pegunavirus; Mycobacterium virus Suffolk
GCA_000955315.1	90460	179	Mycobacterium phage Gaia	NA	Viruses; dsDNA viruses, no RNA stage; Caudovirales; Siphoviridae; unclassified Siphoviridae
GCA_000923655.1	62661	96	Mycobacterium phage OkiRoe	NA	Viruses; dsDNA viruses, no RNA stage; Caudovirales; Siphoviridae; unclassified Siphoviridae
GCA_002604605.1	55307	108	Mycobacterium phage Zapner	NA	Viruses; Duplodnaviria; Heunggongvirae; Uroviricota; Caudoviricetes; Caudovirales; Siphoviridae; Cheoctovirus; unclassified Cheoctovirus
GCA_000920935.1	160541	287	Bacillus phage CAM003	NA	Viruses; dsDNA viruses, no RNA stage; Caudovirales; Myoviridae; Bastillevirus; Bacillus virus CAM003
GCA_000922835.1	159656	294	Bacillus phage Evoli	NA	Viruses; dsDNA viruses, no RNA stage; Caudovirales; Myoviridae; unclassified Myoviridae
GCA_000922815.1	158100	294	Bacillus phage Hakuna	NA	Viruses; dsDNA viruses, no RNA stage; Caudovirales; Myoviridae; unclassified Myoviridae
GCA_000920895.1	159837	270	Bacillus phage Hoody T	NA	Viruses; dsDNA viruses, no RNA stage; Caudovirales; Myoviridae; unclassified Myoviridae
GCA_000920055.1	158750	290	Bacillus phage Megatron	NA	Viruses; dsDNA viruses, no RNA stage; Caudovirales; Myoviridae; unclassified Myoviridae
GCA_000926075.1	162816	290	Bacillus phage Riley	NA	Viruses; dsDNA viruses, no RNA stage; Caudovirales; Myoviridae; B4virus; Bacillus virus Riley
GCA_000918155.1	9910	1	Heliconius erato iflavirus	HeratoCRSEC	Viruses; ssRNA viruses; ssRNA positive-strand viruses, no DNA stage; Picornavirales; Iflaviridae; Iflavirus; unclassified Iflavirus
GCA_003182635.1	7630	6	Phodopus sungorus papillomavirus 1	NA	Viruses; Monodnaviria; Shotokuvirae; Cossaviricota; Papovaviricetes; Zurhausenvirales; Papillomaviridae; Firstpapillomavirinae; Pipapillomavirus; Pipapillomavirus 1
GCA_000919895.1	2589	2	PoSCV Kor J481	NA	Viruses; ssDNA viruses; unclassified ssDNA viruses
GCA_000973295.1	2235	3	Human genitalassociated circular DNA virus1	NA	Viruses; unclassified viruses
GCA_000848545.1	7352	5	Banana mild mosaic virus	NA	Viruses; ssRNA viruses; ssRNA positive-strand viruses, no DNA stage; Tymovirales; Betaflexiviridae; unassigned Betaflexiviridae
GCA_000837285.2	3372	2	Discula destructiva virus 1	NA	Viruses; dsRNA viruses; Partitiviridae; Gammapartitivirus
GCA_000907875.1	2766	6	Sweet potato leaf curl Henan virus	NA	Viruses; ssDNA viruses; Geminiviridae; Begomovirus; unclassified Begomovirus
GCA_000921495.1	2831	6	Sweet potato leaf curl Guangxi virus	NA	Viruses; ssDNA viruses; Geminiviridae; Begomovirus; unclassified Begomovirus
GCA_000920695.1	9578	3	Tanay virus	NA	Viruses; unclassified viruses
GCA_000919195.1	7443	2	Atlantic salmon calicivirus	NA	Viruses; ssRNA viruses; ssRNA positive-strand viruses, no DNA stage; Caliciviridae; unclassified Caliciviridae

gca	genome.size	proteome.size	phage.name	strain	Taxonomy
GCA_003029025.2	5189	7	Jacquemontia yellow mosaic virus	NA	Viruses; Monodnaviria; Shotokuvirae; Cressdnaviricota; Repensiviricetes; Geplafuvirales; Geminiviridae; Begomovirus
GCA_000919875.1	11579	5	Jingmen tick virus	NA	Viruses; ssRNA viruses; ssRNA positive-strand viruses, no DNA stage; unclassified ssRNA positive-strand viruses
GCA_000925655.1	11056	7	Sunguru virus	NA	Viruses; ssRNA viruses; ssRNA negative-strand viruses; Mononegavirales; Rhabdoviridae; unclassified Rhabdoviridae
GCA_000919135.1	2830	4	Sugarcane white streak virus	NA	Viruses; ssDNA viruses; Geminiviridae; Mastrevirus; unclassified Mastrevirus
GCA_002595785.1	172372	212	Synechococcus phage ACG2014a	NA	Viruses; Duplodnaviria; Heunggongvirae; Uroviricota; Caudoviricetes; Caudovirales; Myoviridae; Acionnavirus; unclassified Acionnavirus
GCA_002597145.1	172874	218	Synechococcus phage ACG2014b	NA	Viruses; dsDNA viruses, no RNA stage; Caudovirales; Myoviridae; Tevenvirinae; T4virus; unclassified T4virus
GCA_002548905.1	179233	221	Synechococcus phage ACG2014d	NA	Viruses; dsDNA viruses, no RNA stage; Caudovirales; Myoviridae; Tevenvirinae; T4virus; unclassified T4virus
GCA_002921755.1	189418	217	Synechococcus phage ACG2014e	NA	Viruses; dsDNA viruses, no RNA stage; Caudovirales; Myoviridae; Tevenvirinae; T4virus; unclassified T4virus
GCA_002594065.1	221746	294	Synechococcus phage ACG2014f	NA	Viruses; dsDNA viruses, no RNA stage; Caudovirales; Myoviridae; Tevenvirinae; T4virus; unclassified T4virus
GCA_000982345.1	174885	216	Synechococcus phage ACG2014g	NA	Viruses; dsDNA viruses, no RNA stage; Caudovirales; Myoviridae; Tevenvirinae; T4virus; unclassified T4virus
GCA_001019995.1	190768	212	Synechococcus phage ACG2014i	NA	Viruses; dsDNA viruses, no RNA stage; Caudovirales; Myoviridae; Tevenvirinae; T4virus; unclassified T4virus
GCA_000982305.1	192108	230	Synechococcus phage ACG2014j	NA	Viruses; dsDNA viruses, no RNA stage; Caudovirales; Myoviridae; Tevenvirinae; T4virus; unclassified T4virus
GCA_000928155.1	18569	12	Persimmon virus B	NA	Viruses; ssRNA viruses; ssRNA positive-strand viruses, no DNA stage; Closteroviridae; unclassified Closteroviridae
GCA_000920715.1	8013	2	Goose calicivirus	N	Viruses; ssRNA viruses; ssRNA positive-strand viruses, no DNA stage; Caliciviridae; unclassified Caliciviridae
GCA_000943685.1	2530	1	Sclerotinia sclerotiorum mitovirus 1 HC025	NA	Viruses; ssRNA viruses; ssRNA positive-strand viruses, no DNA stage; Narnaviridae; Mitovirus; unclassified Mitovirus; Sclerotinia sclerotiorum mitovirus 1
GCA_009731715.1	12192	3	Madre de Dios virus	NA	Viruses; Riboviria; Orthornavirae; Negarnaviricota; Polyploviricotina; Ellioviricetes; Bunyavirales; Peribunyaviridae; Orthobunyavirus; Oropouche orthobunyavirus
GCA_009731735.1	12254	3	Utive virus	NA	Viruses; Riboviria; Orthornavirae; Negarnaviricota; Polyploviricotina; Ellioviricetes; Bunyavirales; Peribunyaviridae; Orthobunyavirus; Utinga orthobunyavirus
GCA_000918255.1	168470	279	Escherichia phage vB_EcoM_112	NA	Viruses; dsDNA viruses, no RNA stage; Caudovirales; Myoviridae; Tevenvirinae; T4virus; Escherichia virus E112
GCA_000918355.1	47112	72	Escherichia phage e41c	NA	Viruses; dsDNA viruses, no RNA stage; Caudovirales; Siphoviridae; Tunavirinae; Rogue1virus; Escherichia virus E41c
GCA_000918195.1	19199	1	Soybean cyst nematode virus 5	ScV5	Viruses; ssRNA viruses; ssRNA positive-strand viruses, no DNA stage; Flaviviridae; unclassified Flaviviridae
GCA_000922635.1	12390	3	Cat Que virus	VN042108	Viruses; ssRNA viruses; ssRNA negative-strand viruses; Bunyaviridae; Orthobunyavirus; Manzanilla orthobunyavirus
GCA_000864685.1	12301	1	Classical swine fever virus strain Eystrup	NA	Viruses; Riboviria; Orthornavirae; Kitrinoviricota; Flasuviricetes; Amarillovirales; Flaviviridae; Pestivirus; Pestivirus C; Classical swine fever virus isolates
GCA_000918215.1	5193	5	Leishmania aethiopica RNA virus	NA	Viruses; dsRNA viruses; Totiviridae; Leishmaniavirus; unclassified Leishmaniavirus
GCA_000922675.1	5108	6	New Jersey polyomavirus2013	NA	Viruses; dsDNA viruses, no RNA stage; Polyomaviridae; unclassified Polyomaviridae
GCA_001443785.1	8768	3	Papaya meleira virus	RN Brazil	Viruses; dsRNA viruses; unclassified dsRNA viruses
GCA_002602125.1	6386	11	Ralstonia phage RS611	NA	Viruses; Monodnaviria; Loebvirae; Hofneiviricota; Faserviricetes; Tubulavirales; Inoviridae; unclassified Inoviridae
GCA_002604665.1	42205	57	Cronobacter phage vB_CsaP_Ss1	NA	Viruses; Duplodnaviria; Heunggongvirae; Uroviricota; Caudoviricetes; Caudovirales; Podoviridae; unclassified Podoviridae
GCA_001041015.1	145518	220	Enterococcus phage ECP3	NA	Viruses; dsDNA viruses, no RNA stage; Caudovirales; Myoviridae; unclassified Myoviridae
GCA_002604525.1	74806	85	Dinoroseobacter phage vBDshPR2C	NA	Viruses; Duplodnaviria; Heunggongvirae; Uroviricota; Caudoviricetes; Caudovirales; Podoviridae; Baltimorevirus; unclassified Baltimorevirus
GCA_000923095.1	39093	48	Escherichia phage PE31	NA	Viruses; dsDNA viruses, no RNA stage; Caudovirales; Podoviridae; Autographivirinae; unclassified Autographivirinae
GCA_001504335.1	40592	54	Staphylococcus phage SA97	NA	Viruses; dsDNA viruses, no RNA stage; Caudovirales; Siphoviridae; unclassified Siphoviridae
GCA_001040975.1	45403	62	Escherichia phage ECBP5	NA	Viruses; dsDNA viruses, no RNA stage; Caudovirales; Podoviridae; unclassified Podoviridae
GCA_001042095.1	29741	45	Propionibacterium phage Pacnes 201215	NA	Viruses; dsDNA viruses, no RNA stage; Caudovirales; Siphoviridae; unclassified Siphoviridae
GCA_002756215.1	40086	59	Mycobacterium phage Guo1	NA	Viruses; Duplodnaviria; Heunggongvirae; Uroviricota; Caudoviricetes; Caudovirales; Siphoviridae; Liefievirus; unclassified Liefievirus

gca	genome.size	proteome.size	phage.name	strain	Taxonomy
GCA_000921935.1	50610	87	Mycobacterium phage Pinto	NA	Viruses; dsDNA viruses, no RNA stage; Caudovirales; Siphoviridae; unclassified Siphoviridae
GCA_001041155.1	159492	201	Bacillus phage JBP901	NA	Viruses; dsDNA viruses, no RNA stage; Caudovirales; Myoviridae; Bc431virus
GCA_002601525.1	19347	27	Streptococcus phage SOCP	NA	Viruses; Duplodnaviria; Heunggongvirae; Uroviricota; Caudoviricetes; Caudovirales; Podoviridae; Picovirinae; Cepunavirus; Streptococcus virus Cp1; Streptococcus phage Cp1
GCA_000926815.1	42600	68	Staphylococcus phage phiSa119	NA	Viruses; dsDNA viruses, no RNA stage; Caudovirales; Siphoviridae; unclassified Siphoviridae
GCA_000925775.1	62699	79	Shigella phage POCJ13	NA	Viruses; dsDNA viruses, no RNA stage; Caudovirales; Podoviridae; unclassified Podoviridae; Escherichia phage phi191 sensu lato
GCA_000927395.1	54506	94	Rhizobium phage vB_RleM_PPF1	NA	Viruses; dsDNA viruses, no RNA stage; Caudovirales; Myoviridae; unclassified Myoviridae
GCA_000922655.1	2753	6	Tomato leaf curl Barka virus	NA	Viruses; ssDNA viruses; Geminiviridae; Begomovirus; unclassified Begomovirus
GCA_000921075.1	93794	142	Staphylococcus phage 6ec	NA	Viruses; dsDNA viruses, no RNA stage; Caudovirales; Siphoviridae; Sextaecvirus; Staphylococcus virus Sextaec
GCA_000924375.1	10021	7	Halovirus VNH1	NA	Viruses; unclassified viruses
GCA_004789535.1	12191	4	Jatobal virus	NA	Viruses; Riboviria; Orthornavirae; Negarnaviricota; Polyploviricotina; Ellioviricetes; Bunyavirales; Peribunyaviridae; Orthobunyavirus; Jatobal orthobunyavirus
GCA_002601345.1	44395	71	Vibrio phage 24	NA	Viruses; Duplodnaviria; Heunggongvirae; Uroviricota; Caudoviricetes; Caudovirales; Myoviridae; unclassified Myoviridae
GCA_000922995.1	41569	67	Vibrio phage X29	NA	Viruses; dsDNA viruses, no RNA stage; Caudovirales; Myoviridae; unclassified Myoviridae
GCA_000925735.1	32975	50	Lactobacillus phage Ld17	NA	Viruses; dsDNA viruses, no RNA stage; Caudovirales; Siphoviridae; unclassified Siphoviridae
GCA_000924895.1	32799	51	Lactobacillus phage Ld25A	NA	Viruses; dsDNA viruses, no RNA stage; Caudovirales; Siphoviridae; unclassified Siphoviridae
GCA_000927375.1	29616	49	Lactobacillus phage Ld3	NA	Viruses; dsDNA viruses, no RNA stage; Caudovirales; Siphoviridae; unclassified Siphoviridae
GCA_000921095.1	40472	47	Pseudomonas phage phiPSA2	NA	Viruses; dsDNA viruses, no RNA stage; Caudovirales; Podoviridae; Autographivirinae; T7virus; unclassified T7-like viruses
GCA_000921055.1	51090	52	Pseudomonas phage phiPSA1	NA	Viruses; dsDNA viruses, no RNA stage; Caudovirales; Siphoviridae; unclassified Siphoviridae
GCA_001041315.1	29503	45	Propionibacterium phage PHL009M11	NA	Viruses; Duplodnaviria; Heunggongvirae; Uroviricota; Caudoviricetes; Caudovirales; Siphoviridae; Pahexavirus; Propionibacterium virus PHL009M11
GCA_001042395.1	29496	43	Propionibacterium phage PHL025M00	NA	Viruses; Duplodnaviria; Heunggongvirae; Uroviricota; Caudoviricetes; Caudovirales; Siphoviridae; Pahexavirus; Propionibacterium virus PHL025M00
GCA_001042035.1	29423	45	Propionibacterium phage PHL030N00	NA	Viruses; Duplodnaviria; Heunggongvirae; Uroviricota; Caudoviricetes; Caudovirales; Siphoviridae; Pahexavirus; unclassified Pahexavirus
GCA_001041675.1	29412	45	Propionibacterium phage PHL041M10	NA	Viruses; Duplodnaviria; Heunggongvirae; Uroviricota; Caudoviricetes; Caudovirales; Siphoviridae; Pahexavirus; Propionibacterium virus PHL041M10
GCA_001041295.1	29264	45	Propionibacterium phage PHL055N00	NA	Viruses; Duplodnaviria; Heunggongvirae; Uroviricota; Caudoviricetes; Caudovirales; Siphoviridae; Pahexavirus; unclassified Pahexavirus
GCA_002756235.1	29424	46	Propionibacterium phage PHL064M01	NA	Viruses; Duplodnaviria; Heunggongvirae; Uroviricota; Caudoviricetes; Caudovirales; Siphoviridae; Pahexavirus; unclassified Pahexavirus
GCA_002756255.1	29407	46	Propionibacterium phage PHL064M02	NA	Viruses; Duplodnaviria; Heunggongvirae; Uroviricota; Caudoviricetes; Caudovirales; Siphoviridae; Pahexavirus; unclassified Pahexavirus
GCA_002623065.1	29377	46	Propionibacterium phage PHL067M01	NA	Viruses; Duplodnaviria; Heunggongvirae; Uroviricota; Caudoviricetes; Caudovirales; Siphoviridae; Pahexavirus; Propionibacterium virus PHL067M01
GCA_002605885.1	29386	46	Propionibacterium phage PHL067M09	NA	Viruses; Duplodnaviria; Heunggongvirae; Uroviricota; Caudoviricetes; Caudovirales; Siphoviridae; Pahexavirus; unclassified Pahexavirus
GCA_001041655.1	29421	44	Propionibacterium phage PHL070N00	NA	Viruses; Duplodnaviria; Heunggongvirae; Uroviricota; Caudoviricetes; Caudovirales; Siphoviridae; Pahexavirus; Propionibacterium virus PHL070N00
GCA_001041275.1	29491	47	Propionibacterium phage PHL082M00	NA	Viruses; Duplodnaviria; Heunggongvirae; Uroviricota; Caudoviricetes; Caudovirales; Siphoviridae; Pahexavirus; unclassified Pahexavirus
GCA_002756275.1	29491	46	Propionibacterium phage PHL082M02	NA	Viruses; Duplodnaviria; Heunggongvirae; Uroviricota; Caudoviricetes; Caudovirales; Siphoviridae; Pahexavirus; unclassified Pahexavirus
GCA_002623085.1	29491	46	Propionibacterium phage PHL082M03	NA	Viruses; Duplodnaviria; Heunggongvirae; Uroviricota; Caudoviricetes; Caudovirales; Siphoviridae; Pahexavirus; Propionibacterium virus PHL082M03
GCA_002756295.1	29491	46	Propionibacterium phage PHL082M04	NA	Viruses; Duplodnaviria; Heunggongvirae; Uroviricota; Caudoviricetes; Caudovirales; Siphoviridae; Pahexavirus; unclassified Pahexavirus
GCA_001042355.1	29454	44	Propionibacterium phage PHL085N00	NA	Viruses; Duplodnaviria; Heunggongvirae; Uroviricota; Caudoviricetes; Caudovirales; Siphoviridae; Pahexavirus; unclassified Pahexavirus
GCA_001041995.1	29261	45	Propionibacterium phage PHL092M00	NA	Viruses; Duplodnaviria; Heunggongvirae; Uroviricota; Caudoviricetes; Caudovirales; Siphoviridae; Pahexavirus; Propionibacterium virus PHL092M00
GCA_001041635.1	29751	45	Propionibacterium phage PHL095N00	NA	Viruses; Duplodnaviria; Heunggongvirae; Uroviricota; Caudoviricetes; Caudovirales; Siphoviridae; Pahexavirus; Propionibacterium virus PHL095N00

gca	genome.size	proteome.size	phage.name	strain	Taxonomy
GCA_002602945.1	29464	45	Propionibacterium phage PHL114N00	NA	Viruses; Duplodnaviria; Heunggongvirae; Uroviricota; Caudoviricetes; Caudovirales; Siphoviridae; Pahexavirus; Propionibacterium virus PHL114L00
GCA_001042335.1	29394	46	Propionibacterium phage PHL116M00	NA	Viruses; Duplodnaviria; Heunggongvirae; Uroviricota; Caudoviricetes; Caudovirales; Siphoviridae; Pahexavirus; Propionibacterium virus PHL116M00
GCA_002605905.1	29396	46	Propionibacterium phage PHL116M10	NA	Viruses; Duplodnaviria; Heunggongvirae; Uroviricota; Caudoviricetes; Caudovirales; Siphoviridae; Pahexavirus; unclassified Pahexavirus
GCA_002623105.1	29255	45	Propionibacterium phage PHL117M00	NA	Viruses; Duplodnaviria; Heunggongvirae; Uroviricota; Caudoviricetes; Caudovirales; Siphoviridae; Pahexavirus; Propionibacterium virus PHL117M00
GCA_002623125.1	29422	46	Propionibacterium phage PHL117M01	NA	Viruses; Duplodnaviria; Heunggongvirae; Uroviricota; Caudoviricetes; Caudovirales; Siphoviridae; Pahexavirus; Propionibacterium virus PHL117M01
GCA_001041615.1	29003	42	Propionibacterium phage PHL132N00	NA	Viruses; Duplodnaviria; Heunggongvirae; Uroviricota; Caudoviricetes; Caudovirales; Siphoviridae; Pahexavirus; Propionibacterium virus PHL132N00
GCA_001041235.1	29494	45	Propionibacterium phage PHL141N00	NA	Viruses; Duplodnaviria; Heunggongvirae; Uroviricota; Caudoviricetes; Caudovirales; Siphoviridae; Pahexavirus; Propionibacterium virus PHL141N00
GCA_001038615.1	29438	45	Propionibacterium phage PHL150M00	NA	Viruses; Duplodnaviria; Heunggongvirae; Uroviricota; Caudoviricetes; Caudovirales; Siphoviridae; Pahexavirus; unclassified Pahexavirus
GCA_002623145.1	29511	46	Propionibacterium phage PHL151M00	NA	Viruses; Duplodnaviria; Heunggongvirae; Uroviricota; Caudoviricetes; Caudovirales; Siphoviridae; Pahexavirus; Propionibacterium virus PHL151M00
GCA_002623165.1	29511	46	Propionibacterium phage PHL151N00	NA	Viruses; Duplodnaviria; Heunggongvirae; Uroviricota; Caudoviricetes; Caudovirales; Siphoviridae; Pahexavirus; Propionibacterium virus PHL151N00
GCA_001041955.1	29247	45	Propionibacterium phage PHL152M00	NA	Viruses; Duplodnaviria; Heunggongvirae; Uroviricota; Caudoviricetes; Caudovirales; Siphoviridae; Pahexavirus; Propionibacterium virus PHL152M00
GCA_002623185.1	29264	46	Propionibacterium phage PHL163M00	NA	Viruses; Duplodnaviria; Heunggongvirae; Uroviricota; Caudoviricetes; Caudovirales; Siphoviridae; Pahexavirus; Propionibacterium virus PHL163M00
GCA_001041215.1	29327	45	Propionibacterium phage PHL171M01	NA	Viruses; Duplodnaviria; Heunggongvirae; Uroviricota; Caudoviricetes; Caudovirales; Siphoviridae; Pahexavirus; Propionibacterium virus PHL171M01
GCA_001042295.1	29428	45	Propionibacterium phage PHL179M00	NA	Viruses; Duplodnaviria; Heunggongvirae; Uroviricota; Caudoviricetes; Caudovirales; Siphoviridae; Pahexavirus; Propionibacterium virus PHL179M00
GCA_002623205.1	29264	45	Propionibacterium phage PHL194M00	NA	Viruses; Duplodnaviria; Heunggongvirae; Uroviricota; Caudoviricetes; Caudovirales; Siphoviridae; Pahexavirus; Propionibacterium virus PHL194M00
GCA_001040755.1	29806	45	Propionibacterium phage PHL199M00	NA	Viruses; Duplodnaviria; Heunggongvirae; Uroviricota; Caudoviricetes; Caudovirales; Siphoviridae; Pahexavirus; Propionibacterium virus PHL199M00
GCA_001041575.1	29323	45	Propionibacterium phage PHL301M00	NA	Viruses; Duplodnaviria; Heunggongvirae; Uroviricota; Caudoviricetes; Caudovirales; Siphoviridae; Pahexavirus; Propionibacterium virus PHL301M00
GCA_002623225.1	29442	45	Propionibacterium phage PHL308M00	NA	Viruses; Duplodnaviria; Heunggongvirae; Uroviricota; Caudoviricetes; Caudovirales; Siphoviridae; Pahexavirus; Propionibacterium virus PHL308M00
GCA_000922235.1	12276	4	Drosophila subobscura Nora virus	NA	Viruses; ssRNA viruses; ssRNA positive-strand viruses, no DNA stage; unclassified ssRNA positive-strand viruses
GCA_000921395.1	12265	4	Drosophila immigrans Nora virus	NA	Viruses; ssRNA viruses; ssRNA positive-strand viruses, no DNA stage; unclassified ssRNA positive-strand viruses
GCA_000973375.1	6688	4	Plumeria mosaic virus	NA	Viruses; ssRNA viruses; ssRNA positive-strand viruses, no DNA stage; Virgaviridae; Tobamovirus; unclassified Tobamovirus
GCA_000920995.1	5534	1	Sclerotinia sclerotiorum debilitationassociated RNA virus 2	NA	Viruses; ssRNA viruses; ssRNA positive-strand viruses, no DNA stage; Tymovirales; unclassified Flexiviridae
GCA_000856365.1	8106	6	Garlic virus X	NA	Viruses; ssRNA viruses; ssRNA positive-strand viruses, no DNA stage; Tymovirales; Alphaflexiviridae; Allexivirus
GCA_000852345.1	8451	8	Garlic virus E	NA	Viruses; ssRNA viruses; ssRNA positive-strand viruses, no DNA stage; Tymovirales; Alphaflexiviridae; Allexivirus
GCA_000886195.1	248526	134	Anguillid herpesvirus 1	500138	Viruses; dsDNA viruses, no RNA stage; Herpesvirales; Alloherpesviridae; Cyprinivirus
GCA_001503155.1	27935	6	BtMrAlphaCoVSAX2011	NA	Viruses; ssRNA viruses; ssRNA positive-strand viruses, no DNA stage; Nidovirales; Coronaviridae; Coronavirinae; Alphacoronavirus; unclassified Alphacoronavirus
GCA_001505415.1	27783	6	BtNvAlphaCoVSC2013	NA	Viruses; ssRNA viruses; ssRNA positive-strand viruses, no DNA stage; Nidovirales; Coronaviridae; Coronavirinae; Alphacoronavirus; unclassified Alphacoronavirus
GCA_001504755.1	27608	7	BtRfAlphaCoVHuB2013	NA	Viruses; ssRNA viruses; ssRNA positive-strand viruses, no DNA stage; Nidovirales; Coronaviridae; Coronavirinae; Alphacoronavirus; unclassified Alphacoronavirus
GCA_001501755.1	26975	6	BtRfAlphaCoVYN2012	NA	Viruses; ssRNA viruses; ssRNA positive-strand viruses, no DNA stage; Nidovirales; Coronaviridae; Coronavirinae; Alphacoronavirus; unclassified Alphacoronavirus
GCA_000926955.1	6158	5	Yam virus X	NA	Viruses; ssRNA viruses; ssRNA positive-strand viruses, no DNA stage; Tymovirales; Alphaflexiviridae; Potexvirus; unclassified Potexvirus
GCA_001041735.1	47800	67	Aurantimonas phage AmM1	NA	Viruses; dsDNA viruses, no RNA stage; Caudovirales; unclassified Caudovirales
GCA_000921015.1	144898	79	Bovine gammaherpesvirus 6	NA	Viruses; dsDNA viruses, no RNA stage; Herpesvirales; Herpesviridae; Gammaherpesvirinae; Macavirus
GCA_008776875.1	6488	2	Genet fecal theilovirus	NA	Viruses; Riboviria; Orthornavirae; Pisuviricota; Pisoniviricetes; Picornavirales; Picornaviridae; Cardiovirus; Cardiovirus B
GCA_001503895.1	15541	8	Fox fecal rhabdovirus	NA	Viruses; ssRNA viruses; ssRNA negative-strand viruses; Mononegavirales; Rhabdoviridae; unclassified Rhabdoviridae

gca	genome.size	proteome.size	phage.name	strain	Taxonomy
GCA_001019895.1	2741	6	Hollyhock yellow vein mosaic Islamabad virus	NA	Viruses; ssDNA viruses; Geminiviridae; Begomovirus; unclassified Begomovirus
GCA_001963075.1	2750	6	Papaya leaf curl Faisalabad virus	NA	Viruses; unclassified viruses
GCA_001503095.1	41931	66	Staphylococcus phage 3MRA	NA	Viruses; dsDNA viruses, no RNA stage; Caudovirales; Siphoviridae; unclassified Siphoviridae
GCA_001504695.1	43098	66	Staphylococcus phage 23MRA	NA	Viruses; dsDNA viruses, no RNA stage; Caudovirales; Siphoviridae; unclassified Siphoviridae
GCA_000926095.1	44844	84	Acinetobacter phage YMC1301C62	NA	Viruses; dsDNA viruses, no RNA stage; Caudovirales; Myoviridae; Ap22virus; Acinetobacter virus AbC62
GCA_002604725.1	70214	124	Mycobacterium phage YungJamal	NA	Viruses; Duplodnaviria; Heunggongvirae; Uroviricota; Caudoviricetes; Caudovirales; Siphoviridae; Corndogvirus; unclassified Corndogvirus
GCA_000929475.1	53744	86	Aeromonas phage pAh6C	NA	Viruses; dsDNA viruses, no RNA stage; Caudovirales; Myoviridae; unclassified Myoviridae
GCA_003177535.1	7948	7	human papillomavirus 84	NA	Viruses; Monodnaviria; Shotokuvirae; Cossaviricota; Papovaviricetes; Zurhausenvirales; Papillomaviridae; Firstpapillomavirinae; Alphapapillomavirus; Alphapapillomavirus 3
GCA_000924935.1	7679	13	Ralstonia phage RS603	NA	Viruses; ssDNA viruses; Inoviridae; Inovirus; unclassified Inovirus
GCA_000923515.1	7424	5	Pagoda yellow mosaic associated virus	pymav01	Viruses; Riboviria; Pararnavirae; Artverviricota; Revtraviricetes; Ortervirales; Caulimoviridae; Badnavirus
GCA_000921035.1	1353	1	Luffa puckering and leaf distortionassociated betasatellite IndiaGurdaspurOkra2013	NA	Viruses; Satellites; Satellite Nucleic Acids; Single stranded DNA satellites; Betasatellites; unclassified Begomovirus-associated DNA beta-like sequences
GCA_000923555.1	1374	1	Sida yellow vein alphasatellite	NA	Viruses; Satellites; Satellite Nucleic Acids; Single stranded DNA satellites; Alphasatellites; Begomovirus-associated alphasatellites; unclassified Begomovirus-associated alphasatellites
GCA_004058655.1	648	2	Porcine circoviruslike virus P1	NA	Viruses; Monodnaviria; Shotokuvirae; Cressdnaviricota; Arfiviricetes; Cirlivirales; Circoviridae; Circovirus; unclassified Circovirus
GCA_000925995.1	1865	2	Cyclovirus ZM36a	NA	Viruses; ssDNA viruses; Circoviridae; unclassified Circoviridae; suggested genus Cyclovirus
GCA_001505155.1	57253	92	Mycobacterium phage Cheetobro	NA	Viruses; dsDNA viruses, no RNA stage; Caudovirales; Siphoviridae; unclassified Siphoviridae
GCA_000923295.1	1406	1	Mirabilis leaf curl India virus associated betasatellite	NA	Viruses; Tolecusatellitidae; Betasatellite; unclassified Betasatellite
GCA_001045325.1	1375	1	Chilli leaf curl India alphasatellite	NA	Viruses; Satellites; Satellite Nucleic Acids; Single stranded DNA satellites; Alphasatellites; Begomovirus-associated alphasatellites; unclassified Begomovirus-associated alphasatellites
GCA_002830145.1	1350	1	Papaya leaf curl China betasatellite ChinaHainan2014	NA	Viruses; Tolecusatellitidae; Betasatellite; Papaya leaf curl China betasatellite
GCA_002118885.1	29642	10	Bat coronavirus	NA	Viruses; ssRNA viruses; ssRNA positive-strand viruses, no DNA stage; Nidovirales; Coronaviridae; Coronavirinae; unclassified coronaviruses
GCA_001962315.1	28763	5	Lucheng Rn rat coronavirus	NA	Viruses; ssRNA viruses; ssRNA positive-strand viruses, no DNA stage; Nidovirales; Coronaviridae; Coronavirinae; Alphacoronavirus; unclassified Alphacoronavirus
GCA_002219865.1	25995	5	Wencheng Sm shrew coronavirus	NA	Viruses; ssRNA viruses; ssRNA positive-strand viruses, no DNA stage; Nidovirales; Coronaviridae; Coronavirinae; Alphacoronavirus; unclassified Alphacoronavirus
GCA_002596525.1	5386	11	Enterobacteria phage MED1	NA	Viruses; Monodnaviria; Sangervirae; Phixviricota; Malgrandaviricetes; Petitvirales; Microviridae; Bullavirinae; Sinsheimervirus; unclassified Sinsheimervirus; Enterobacteria phage phiX174 sensu lato
GCA_002624465.1	88531	132	Escherichia typing phage 1	NA	Viruses; Duplodnaviria; Heunggongvirae; Uroviricota; Caudoviricetes; Caudovirales; Myoviridae; Ounavirinae; Felixounavirus; Escherichia virus TP1
GCA_002604765.1	39234	50	Escherichia coli O157 typing phage 10	NA	Viruses; Duplodnaviria; Heunggongvirae; Uroviricota; Caudoviricetes; Caudovirales; Podoviridae; Uetakevirus; unclassified Uetakevirus
GCA_002604785.1	88771	132	Escherichia coli O157 typing phage 11	NA	Viruses; Duplodnaviria; Heunggongvirae; Uroviricota; Caudoviricetes; Caudovirales; Myoviridae; Ounavirinae; Felixounavirus; unclassified Felixounavirus
GCA_002604805.1	88632	132	Escherichia coli O157 typing phage 12	NA	Viruses; Duplodnaviria; Heunggongvirae; Uroviricota; Caudoviricetes; Caudovirales; Myoviridae; Ounavirinae; Felixounavirus; unclassified Felixounavirus
GCA_002604825.1	168733	270	Escherichia coli O157 typing phage 3	NA	Viruses; Duplodnaviria; Heunggongvirae; Uroviricota; Caudoviricetes; Caudovirales; Myoviridae; Tevenvirinae; Mosigvirus; Escherichia virus O157tp3
GCA_002604845.1	137973	214	Escherichia coli O157 typing phage 5	NA	Viruses; Duplodnaviria; Heunggongvirae; Uroviricota; Caudoviricetes; Caudovirales; Myoviridae; Vequintavirinae; Vequintavirus; unclassified Vequintavirus
GCA_002604865.1	160570	251	Escherichia coli O157 typing phage 6	NA	Viruses; Duplodnaviria; Heunggongvirae; Uroviricota; Caudoviricetes; Caudovirales; Myoviridae; Tevenvirinae; Mosigvirus; Escherichia virus O157tp6
GCA_000921235.1	3368	4	Roundleaf bat hepatitis B virus	NA	Viruses; Riboviria; Pararnavirae; Artverviricota; Revtraviricetes; Blubervirales; Hepadnaviridae; Orthohepadnavirus
GCA_000923115.1	3377	4	Horseshoe bat hepatitis B virus	NA	Viruses; Riboviria; Pararnavirae; Artverviricota; Revtraviricetes; Blubervirales; Hepadnaviridae; Orthohepadnavirus; Roundleaf bat hepatitis B virus
GCA_000923755.1	3149	4	Tentmaking bat hepatitis B virus	NA	Viruses; Retro-transcribing viruses; Hepadnaviridae; Orthohepadnavirus
GCA_008797595.1	7219	1	Human rhinovirus B17	NA	Viruses; Riboviria; Orthornavirae; Pisuviricota; Pisoniviricetes; Picornavirales; Picornaviridae; Enterovirus; Rhinovirus B
GCA_000922095.1	204054	130	Falconid herpesvirus 1	S18	Viruses; dsDNA viruses, no RNA stage; Herpesvirales; Herpesviridae; Alphaherpesvirinae; Mardivirus; unclassified Mardivirus

gca	genome.size	proteome.size	phage.name	strain	Taxonomy
GCA_000850805.1	8632	5	Tulare apple mosaic virus	NA	Viruses; ssRNA viruses; ssRNA positive-strand viruses, no DNA stage; Bromoviridae; Ilarvirus; Ilarvirus subgroup 2
GCA_000925695.1	45648	72	Bacillus phage Waukesha92	NA	Viruses; dsDNA viruses, no RNA stage; Caudovirales; Siphoviridae; unclassified Siphoviridae
GCA_000924815.1	11141	5	Dolphin rhabdovirus	NA	Viruses; ssRNA viruses; ssRNA negative-strand viruses; Mononegavirales; Rhabdoviridae; unclassified Rhabdoviridae
GCA_900197175.1	6905	1	Pasivirus A	NA	Viruses; Riboviria; Orthornavirae; Pisuviricota; Pisoniviricetes; Picornavirales; Picornaviridae; Pasivirus
GCA_000898975.1	6916	1	Pasivirus A1	NA	Viruses; ssRNA viruses; ssRNA positive-strand viruses, no DNA stage; Picornavirales; Picornaviridae; Pasivirus; Pasivirus A
GCA_008777635.1	6858	1	pasivirus A2	NA	Viruses; Riboviria; Orthornavirae; Pisuviricota; Pisoniviricetes; Picornavirales; Picornaviridae; Pasivirus; Pasivirus A
GCA_000918175.1	8931	1	Rosavirus A2	GA7403	Viruses; ssRNA viruses; ssRNA positive-strand viruses, no DNA stage; Picornavirales; Picornaviridae; Rosavirus; Rosavirus A
GCA_000851545.1	3090	2	Fusarium solani virus 1	NA	Viruses; dsRNA viruses; Partitiviridae; Gammapartitivirus
GCA_003033285.1	5543	7	Primate bocaparvovirus 1	NA	Viruses; Monodnaviria; Shotokuvirae; Cossaviricota; Quintoviricetes; Piccovirales; Parvoviridae; Parvovirinae; Bocaparvovirus
GCA_000886375.1	5104	5	Human bocavirus 4 NI	HBoV4NI385	Viruses; ssDNA viruses; Parvoviridae; Parvovirinae; Bocaparvovirus; Primate bocaparvovirus 2
GCA_000905315.1	5413	4	Canine bocavirus 1	NA	Viruses; ssDNA viruses; Parvoviridae; Parvovirinae; Bocaparvovirus; Carnivore bocaparvovirus 2
GCA_002827465.1	4822	4	Primate protoparvovirus 1	BF86	Viruses; Monodnaviria; Shotokuvirae; Cossaviricota; Quintoviricetes; Piccovirales; Parvoviridae; Parvovirinae; Protoparvovirus
GCA_001430475.1	5111	3	Ungulate tetraparvovirus 1	NA	Viruses; ssDNA viruses; Parvoviridae; Parvovirinae; Tetraparvovirus
GCA_002219485.1	5533	2	Ungulate tetraparvovirus 3	NA	Viruses; ssDNA viruses; Parvoviridae; Parvovirinae; Tetraparvovirus
GCA_000861005.1	5268	2	Human parvovirus 4 G1	NA	Viruses; ssDNA viruses; Parvoviridae; Parvovirinae; Tetraparvovirus; Primate tetraparvovirus 1
GCA_009731755.1	7178	3	Aglaonema bacilliform virus	NA	Viruses; Riboviria; Pararnavirae; Artverviricota; Revtraviricetes; Ortervirales; Caulimoviridae; Badnavirus
GCA_000882875.1	3776	3	Aedes aegypti densovirus 2	0814616	Viruses; ssDNA viruses; Parvoviridae; Densovirinae; Brevidensovirus; Dipteran brevidensovirus 1; Aedes albopictus densovirus
GCA_000847125.1	4176	3	Aedes albopictus densovirus 2	NA	Viruses; ssDNA viruses; Parvoviridae; Densovirinae; Brevidensovirus; Dipteran brevidensovirus 2
GCA_000866565.1	6321	3	Penaeus monodon hepandensovirus 1	NA	Viruses; ssDNA viruses; Parvoviridae; Densovirinae; Hepandensovirus; Decapod hepandensovirus 1
GCA_000882415.1	6310	3	Penaeus monodon hepandensovirus 4	NA	Viruses; ssDNA viruses; Parvoviridae; Densovirinae; Hepandensovirus; Decapod hepandensovirus 1
GCA_000844705.1	3909	3	Decapod penstyldensovirus 1	NA	Viruses; ssDNA viruses; Parvoviridae; Densovirinae; Penstyldensovirus
GCA_003033215.1	3873	3	Penaeus stylirostris penstyldensovirus 1	NA	Viruses; Monodnaviria; Shotokuvirae; Cossaviricota; Quintoviricetes; Piccovirales; Parvoviridae; Hamaparvovirinae; Penstylhamaparvovirus; Decapod penstylhamaparvovirus 1
GCA_000896415.1	18927	9	Lloviu cuevavirus	MSLiver862003	Viruses; ssRNA viruses; ssRNA negative-strand viruses; Mononegavirales; Filoviridae; Cuevavirus
GCA_004288695.1	7252	7	Gammapapillomavirus 11	NA	Viruses; Monodnaviria; Shotokuvirae; Cossaviricota; Papovaviricetes; Zurhausenvirales; Papillomaviridae; Firstpapillomavirinae; Gammapapillomavirus
GCA_004288615.1	7110	7	Gammapapillomavirus 12	NA	Viruses; Monodnaviria; Shotokuvirae; Cossaviricota; Papovaviricetes; Zurhausenvirales; Papillomaviridae; Firstpapillomavirinae; Gammapapillomavirus
GCA_004288555.1	7243	7	Gammapapillomavirus 13	NA	Viruses; Monodnaviria; Shotokuvirae; Cossaviricota; Papovaviricetes; Zurhausenvirales; Papillomaviridae; Firstpapillomavirinae; Gammapapillomavirus
GCA_004288535.1	7159	7	Gammapapillomavirus 14	NA	Viruses; Monodnaviria; Shotokuvirae; Cossaviricota; Papovaviricetes; Zurhausenvirales; Papillomaviridae; Firstpapillomavirinae; Gammapapillomavirus
GCA_004288455.1	7221	7	Gammapapillomavirus 15	NA	Viruses; Monodnaviria; Shotokuvirae; Cossaviricota; Papovaviricetes; Zurhausenvirales; Papillomaviridae; Firstpapillomavirinae; Gammapapillomavirus
GCA_004288435.1	7209	7	Gammapapillomavirus 16	NA	Viruses; Monodnaviria; Shotokuvirae; Cossaviricota; Papovaviricetes; Zurhausenvirales; Papillomaviridae; Firstpapillomavirinae; Gammapapillomavirus
GCA_004288395.1	7292	7	Gammapapillomavirus 18	NA	Viruses; dsDNA viruses, no RNA stage; Papillomaviridae; Gammapapillomavirus
GCA_004288335.1	7282	7	Gammapapillomavirus 19	NA	Viruses; Monodnaviria; Shotokuvirae; Cossaviricota; Papovaviricetes; Zurhausenvirales; Papillomaviridae; Firstpapillomavirinae; Gammapapillomavirus
GCA_000884335.1	8242	7	Canis familiaris papillomavirus 6	NA	Viruses; dsDNA viruses, no RNA stage; Papillomaviridae; Lambdapapillomavirus; Lambdapapillomavirus 3
GCA_000894935.1	14702	8	Hibiscus green spot virus 2	NA	Viruses; Riboviria; Orthornavirae; Kitrinoviricota; Alsuviricetes; Martellivirales; Kitaviridae; Higrevirus
GCA_000929935.1	4928	5	Raccoon dog amdovirus	NA	Viruses; ssDNA viruses; Parvoviridae; Parvovirinae; Amdoparvovirus; unclassified Amdoparvovirus

gca	genome.size	proteome.size	phage.name	strain	Taxonomy
GCA_000926175.1	5453	9	Bitter gourd yellow vein virus	NA	Viruses; ssDNA viruses; Geminiviridae; Begomovirus
GCA_002867615.1	5103	7	Rhynchosia golden mosaic Sinaloa virus	NA	Viruses; Monodnaviria; Shotokuvirae; Cressdnaviricota; Repensiviricetes; Geplafuvirales; Geminiviridae; Begomovirus
GCA_001967215.1	2781	5	Sweet potato mosaic virus	SPMaV	Viruses; ssDNA viruses; Geminiviridae; Begomovirus
GCA_002823825.1	2803	6	Sweet potato mosaic virus BrazilBrasilia12007	NA	Viruses; Monodnaviria; Shotokuvirae; Cressdnaviricota; Repensiviricetes; Geplafuvirales; Geminiviridae; Begomovirus; Sweet potato mosaic virus
GCA_018580265.1	2235	4	Rodent Torque teno virus 1	NA	Viruses; Anelloviridae; Rhotorquevirus; Torque teno rodent virus 1
GCA_000930715.1	2572	3	Rodent Torque teno virus 2	NA	Viruses; ssDNA viruses; Anelloviridae; unclassified Anelloviridae
GCA_003147425.1	7368	3	Bat norovirus	NA	Viruses; Riboviria; Orthornavirae; Pisuviricota; Pisoniviricetes; Picornavirales; Caliciviridae; Norovirus; Norwalk virus
GCA_000923175.1	13954	1	Yerba mate endornavirus	INTA	Viruses; dsRNA viruses; Endornaviridae; Endornavirus; unclassified Endornavirus
GCA_002756315.1	37631	53	Burkholderia phage BEK	NA	Viruses; Duplodnaviria; Heunggongvirae; Uroviricota; Caudoviricetes; Caudovirales; Myoviridae; Peduovirinae; Tigrvirus; Burkholderia virus phi52237
GCA_000849825.2	46675	55	Synechococcus virus P60	NA	Viruses; Duplodnaviria; Heunggongvirae; Uroviricota; Caudoviricetes; Caudovirales; Autographiviridae; Tiilvirus
GCA_000839025.1	14510	21	Lactococcus phage bIL311	NA	Viruses; dsDNA viruses, no RNA stage; Caudovirales; Siphoviridae; unclassified Siphoviridae
GCA_000838885.1	35538	62	Lactococcus phage bIL285	NA	Viruses; dsDNA viruses, no RNA stage; Caudovirales; Siphoviridae; unclassified Siphoviridae
GCA_000845385.1	41834	61	Lactococcus phage bIL286	NA	Viruses; dsDNA viruses, no RNA stage; Caudovirales; Siphoviridae; unclassified Siphoviridae
GCA_000842505.1	36949	56	Lactococcus phage bIL309	NA	Viruses; dsDNA viruses, no RNA stage; Caudovirales; Siphoviridae; unclassified Siphoviridae
GCA_000838045.1	14957	28	Lactococcus phage bIL310	NA	Viruses; dsDNA viruses, no RNA stage; Caudovirales; Siphoviridae; unclassified Siphoviridae
GCA_000837265.1	15179	27	Lactococcus phage bIL312	NA	Viruses; dsDNA viruses, no RNA stage; Caudovirales; Siphoviridae; unclassified Siphoviridae
GCA_000844345.1	38439	59	Pseudomonas phage B3	ATCC15692B1	Viruses; dsDNA viruses, no RNA stage; Caudovirales; Siphoviridae; unclassified Siphoviridae
GCA_001974015.1	8739	5	Botryosphaeria dothidea virus 1	YZN115	Viruses; dsRNA viruses; Partitiviridae; unclassified Partitiviridae
GCA_000921475.1	2152	3	HCBI8215 virus	NA	Viruses; ssDNA viruses; unclassified ssDNA viruses
GCA_000923995.1	2121	3	HCBI9212 virus	NA	Viruses; ssDNA viruses; unclassified ssDNA viruses
GCA_000923355.1	2259	3	MSSI2225 virus	NA	Viruses; ssDNA viruses; unclassified ssDNA viruses
GCA_003114135.1	23379	25	Qinghai Lake virophage	NA	Viruses; Varidnaviria; Bamfordvirae; Preplasmiviricota; Maveriviricetes; Priklausovirales; Lavidaviridae; unclassified Lavidaviridae
GCA_000930655.1	5770	3	Chaetoceros tenuissimus DNA virus typeII	NA	Viruses; ssDNA viruses; unclassified ssDNA viruses
GCA_000930635.1	9562	2	Chaetoceros tenuissimus RNA virus typeII	NA	Viruses; ssRNA viruses; unclassified ssRNA viruses
GCA_013086855.1	8415	2	American dog tick phlebovirus	NA	Viruses; Riboviria; Orthornavirae; Negarnaviricota; Polyploviricotina; Ellioviricetes; Bunyavirales; Phenuiviridae; Phlebovirus; unclassified Phlebovirus
GCA_000923215.1	6577	3	Human astrovirus BF34	NA	Viruses; ssRNA viruses; ssRNA positive-strand viruses, no DNA stage; Astroviridae; Mamastrovirus; unclassified Mamastrovirus; Burkina Faso astrovirus
GCA_002818845.1	10559	2	Arenaviridae sp 13ZR68	NA	Viruses; Riboviria; Orthornavirae; Negarnaviricota; Polyploviricotina; Ellioviricetes; Bunyavirales; Arenaviridae; Mammarenavirus; Solwezi mammarenavirus
GCA_000928375.1	5049	2	Sesavirus CSL10538	NA	Viruses; ssDNA viruses; Parvoviridae; unclassified Parvoviridae
GCA_003726635.1	2777	2	Sewageassociated circular DNA virus1	NA	Viruses; unclassified viruses
GCA_003726655.1	2246	2	Sewageassociated circular DNA virus10	NA	Viruses; unclassified viruses
GCA_003726675.1	2336	2	Sewageassociated circular DNA virus11	NA	Viruses; unclassified viruses
GCA_003726695.1	1805	2	Sewageassociated circular DNA virus12	NA	Viruses; unclassified viruses
GCA_003726715.1	3533	2	Sewageassociated circular DNA virus13	NA	Viruses; unclassified viruses
GCA_003726735.1	2335	2	Sewageassociated circular DNA virus14	NA	Viruses; unclassified viruses

gca	genome.size	proteome.size	phage.name	strain	Taxonomy
GCA_001441035.1	2531	2	Sewageassociated circular DNA virus2	NA	Viruses; unclassified viruses
GCA_003726755.1	2112	2	Sewageassociated circular DNA virus3	NA	Viruses; unclassified viruses
GCA_003726775.1	1899	2	Sewageassociated circular DNA virus4	NA	Viruses; unclassified viruses
GCA_003726795.1	1969	2	Sewageassociated circular DNA virus5	NA	Viruses; unclassified viruses
GCA_003726815.1	2540	2	Sewageassociated circular DNA virus6	NA	Viruses; unclassified viruses
GCA_003726835.1	2311	3	Sewageassociated circular DNA virus7	NA	Viruses; unclassified viruses
GCA_003726855.1	2577	3	Sewageassociated circular DNA virus8	NA	Viruses; unclassified viruses
GCA_003726875.1	2423	2	Sewageassociated circular DNA virus9	NA	Viruses; Monodnaviria; Shotokuvirae; Cressdnaviricota; Arfiviricetes; Cremevirales; Smacoviridae; unclassified Smacoviridae
GCA_000928235.1	2115	3	Sewageassociated gemycircularvirus 4	NA	Viruses; unclassified viruses; Genomoviridae; Gemycircularvirus; unclassified Gemycircularvirus
GCA_000928215.1	2243	3	Sewageassociated gemycircularvirus 5	NA	Viruses; unclassified viruses; Genomoviridae; Gemycircularvirus; unclassified Gemycircularvirus
GCA_000930835.1	2147	2	Sewageassociated gemycircularvirus 6	NA	Viruses; unclassified viruses; Genomoviridae; Gemycircularvirus; unclassified Gemycircularvirus
GCA_000930195.1	2277	3	Sewageassociated gemycircularvirus7a	NA	Viruses; unclassified viruses; Genomoviridae; Gemycircularvirus; unclassified Gemycircularvirus; Sewage-associated gemycircularvirus 7
GCA_000929155.1	2273	3	Sewageassociated gemycircularvirus 9	NA	Viruses; unclassified viruses; Genomoviridae; Gemycircularvirus; unclassified Gemycircularvirus
GCA_002825965.1	2237	3	Sewageassociated gemycircularvirus7b	NA	Viruses; Monodnaviria; Shotokuvirae; Cressdnaviricota; Repensiviricetes; Geplafuvirales; Genomoviridae; Gemycircularvirus; Sewage derived gemycircularvirus 4
GCA_000929175.1	2193	3	Sewageassociated gemycircularvirus 2	NA	Viruses; unclassified viruses; Genomoviridae; Gemycircularvirus; unclassified Gemycircularvirus
GCA_000930855.1	2249	3	Sewageassociated gemycircularvirus10a	NA	Viruses; unclassified viruses; Genomoviridae; Gemycircularvirus; unclassified Gemycircularvirus; Sewage-associated gemycircularvirus 10
GCA_003849505.1	2254	3	Sewageassociated gemycircularvirus10b	NA	Viruses; Monodnaviria; Shotokuvirae; Cressdnaviricota; Repensiviricetes; Geplafuvirales; Genomoviridae; Gemykibivirus; Human associated gemykibivirus 1
GCA_001042235.1	77327	125	Phage vB_EcoP_SU10	NA	Viruses; dsDNA viruses, no RNA stage; Caudovirales; Podoviridae; unclassified Podoviridae
GCA_000930755.1	43748	46	Staphylococcus phage phiBU01	NA	Viruses; dsDNA viruses, no RNA stage; Caudovirales; Siphoviridae; unclassified Siphoviridae
GCA_006437935.1	43842	37	Duck adenovirus 2	CHGD122014	Viruses; dsDNA viruses, no RNA stage; Adenoviridae; Aviadenovirus; Duck aviadenovirus B
GCA_000921335.2	11467	4	Blueberry mosaic associated virus	NA	Viruses; ssRNA viruses; ssRNA negative-strand viruses; Ophioviridae; Ophiovirus
GCA_002820225.1	1710	2	Cyclovirus SL108277	NA	Viruses; Monodnaviria; Shotokuvirae; Cressdnaviricota; Arfiviricetes; Cirlivirales; Circoviridae; Cyclovirus; Human associated cyclovirus 11
GCA_006426535.1	35379	48	Human adenovirus 21a	NA	Viruses; Varidnaviria; Bamfordvirae; Preplasmiviricota; Tectiliviricetes; Rowavirales; Adenoviridae; Mastadenovirus; Human mastadenovirus B; Human adenovirus B1; Human adenovirus 21
GCA_000923955.1	10044	1	Lymantria dispar iflavirus 1	NA	Viruses; ssRNA viruses; ssRNA positive-strand viruses, no DNA stage; Picornavirales; Iflaviridae; Iflavirus
GCA_000921375.1	2089	3	McMurdo Ice Shelf pondassociated circular DNA virus1	NA	Viruses; ssDNA viruses; unclassified ssDNA viruses
GCA_000923895.1	2614	4	McMurdo Ice Shelf pondassociated circular DNA virus2	NA	Viruses; ssDNA viruses; unclassified ssDNA viruses
GCA_000923255.1	3120	2	McMurdo Ice Shelf pondassociated circular DNA virus3	NA	Viruses; ssDNA viruses; unclassified ssDNA viruses
GCA_000922195.1	2698	3	McMurdo Ice Shelf pondassociated circular DNA virus4	NA	Viruses; ssDNA viruses; unclassified ssDNA viruses
GCA_000921355.1	2026	2	McMurdo Ice Shelf pondassociated circular DNA virus5	NA	Viruses; ssDNA viruses; unclassified ssDNA viruses
GCA_000923875.1	2549	3	McMurdo Ice Shelf pondassociated circular DNA virus6	NA	Viruses; ssDNA viruses; unclassified ssDNA viruses
GCA_000923235.1	1901	2	McMurdo Ice Shelf pondassociated circular DNA virus7	NA	Viruses; ssDNA viruses; unclassified ssDNA viruses
GCA_000922175.1	2911	3	McMurdo Ice Shelf pondassociated circular DNA virus8	NA	Viruses; ssDNA viruses; unclassified ssDNA viruses
GCA_000870945.1	12414	6	Orchid fleck dichorhavirus	NA	Viruses; ssRNA viruses; ssRNA negative-strand viruses; Mononegavirales; Rhabdoviridae; Dichorhavirus
GCA_000922275.1	6530	3	Bovine astrovirus CH13	NA	Viruses; ssRNA viruses; ssRNA positive-strand viruses, no DNA stage; Astroviridae; unclassified Astroviridae

gca	genome.size	proteome.size	phage.name	strain	Taxonomy
GCA_000851525.1	5133	2	Gremmeniella abietina RNA virus L1	NA	Viruses; dsRNA viruses; Totiviridae; Victorivirus
GCA_000923275.1	8842	2	Asterionellopsis glacialis RNA virus	NA	Viruses; ssRNA viruses; ssRNA positive-strand viruses, no DNA stage; Picornavirales; unassigned Picornavirales; Bacillarnavirus; unclassified Bacillarnavirus
GCA_000866025.1	15516	7	Menangle virus	NA	Viruses; ssRNA viruses; ssRNA negative-strand viruses; Mononegavirales; Paramyxoviridae; Rubulavirus; unclassified Rubulavirus
GCA_000922295.1	9760	1	Phomopsis longicolla hypovirus	NA	Viruses; dsRNA viruses; Hypoviridae; Hypovirus; unclassified Hypovirus
GCA_002604885.1	50431	67	Vibrio phage VPp1	NA	Viruses; Duplodnaviria; Heunggongvirae; Uroviricota; Caudoviricetes; Caudovirales; Podoviridae; unclassified Podoviridae
GCA_001041875.1	38742	66	Enterobacteria phage Sf101	NA	Viruses; dsDNA viruses, no RNA stage; Caudovirales; Podoviridae; unclassified Podoviridae
GCA_001534635.1	50827	72	Sinorhizobium phage phiLM21	NA	Viruses; dsDNA viruses, no RNA stage; Caudovirales; Siphoviridae; unclassified Siphoviridae
GCA_000924855.1	141907	204	Staphylococcus phage MCE2014	NA	Viruses; dsDNA viruses, no RNA stage; Caudovirales; Myoviridae; Spounavirinae; Kayvirus; Staphylococcus virus MCE2014
GCA_000924015.1	2836	4	Human circovirus VS6600022	NA	Viruses; ssDNA viruses; Circoviridae; Circovirus; unclassified Circovirus
GCA_008800535.1	7411	1	Enterovirus CA551988	NA	Viruses; Riboviria; Orthornavirae; Pisuviricota; Pisoniviricetes; Picornavirales; Picornaviridae; Enterovirus; Enterovirus B; Enterovirus B73
GCA_000926755.1	140807	226	Staphylococcus phage P108	NA	Viruses; dsDNA viruses, no RNA stage; Caudovirales; Myoviridae; Spounavirinae; Kayvirus; Staphylococcus virus P108
GCA_018594685.1	19428	2	South Bay virus	NA	Viruses; Riboviria; Orthornavirae; Negarnaviricota; Polyploviricotina; Ellioviricetes; Bunyavirales; Nairoviridae; Sabavirus; South Bay sabavirus
GCA_013086865.1	9201	2	Blacklegged tick phlebovirus 1	NA	Viruses; Riboviria; Orthornavirae; Negarnaviricota; Polyploviricotina; Ellioviricetes; Bunyavirales; Phenuiviridae; Phlebovirus; unclassified Phlebovirus
GCA_001040815.1	42092	71	Sulfitobacter phage NYA2014a	NA	Viruses; dsDNA viruses, no RNA stage; unclassified dsDNA phages
GCA_002867205.1	10925	2	Mulberry mosaic leaf roll associated virus	NA	Viruses; Riboviria; Orthornavirae; Pisuviricota; Pisoniviricetes; Picornavirales; Secoviridae; Comovirinae; Nepovirus
GCA_000954595.1	53636	102	Mycobacterium phage Cerasum	NA	Viruses; dsDNA viruses, no RNA stage; Caudovirales; Siphoviridae; unclassified Siphoviridae
GCA_000921655.1	160281	247	Bacillus phage Bobb	NA	Viruses; dsDNA viruses, no RNA stage; Caudovirales; Myoviridae; Agatevirus; Bacillus virus Bobb
GCA_000955455.1	63334	91	Mycobacterium phage Sparky	NA	Viruses; dsDNA viruses, no RNA stage; Caudovirales; Siphoviridae; unclassified Siphoviridae
GCA_001470415.1	68472	99	Mycobacterium phage Eremos	NA	Viruses; dsDNA viruses, no RNA stage; Caudovirales; Siphoviridae; unclassified Siphoviridae; PG1-like phages
GCA_001041375.1	38810	49	Escherichia phage CICC 80001	NA	Viruses; dsDNA viruses, no RNA stage; Caudovirales; Podoviridae; Autographivirinae; T7virus
GCA_000954735.1	54986	102	Mycobacterium phage Hades	NA	Viruses; dsDNA viruses, no RNA stage; Caudovirales; Siphoviridae; unclassified Siphoviridae
GCA_000954375.1	60285	102	Mycobacterium phage Squirty	NA	Viruses; dsDNA viruses, no RNA stage; Caudovirales; Siphoviridae; unclassified Siphoviridae
GCA_000955095.1	109871	238	Mycobacterium phage Minerva	NA	Viruses; dsDNA viruses, no RNA stage; Caudovirales; Siphoviridae; unclassified Siphoviridae
GCA_000922495.1	44332	62	Escherichia phage EK99P1	NA	Viruses; Duplodnaviria; Heunggongvirae; Uroviricota; Caudoviricetes; Caudovirales; Siphoviridae; Dhillonvirus; Escherichia virus EK99P1
GCA_000929595.1	36844	65	Idiomarinaceae phage Phi1M22	NA	Viruses; dsDNA viruses, no RNA stage; Caudovirales; Siphoviridae; unclassified Siphoviridae
GCA_001505795.1	50882	90	Mycobacterium phage MarQuardt	NA	Viruses; dsDNA viruses, no RNA stage; Caudovirales; Siphoviridae; L5virus; unclassified L5likevirus
GCA_002604905.1	49190	84	Mycobacterium phage Farber	NA	Viruses; Duplodnaviria; Heunggongvirae; Uroviricota; Caudoviricetes; Caudovirales; Siphoviridae; Fromanvirus; unclassified Fromanvirus
GCA_000928035.1	120938	209	Escherichia phage Av05	NA	Viruses; dsDNA viruses, no RNA stage; Caudovirales; Myoviridae; unclassified Myoviridae
GCA_001505815.1	52647	94	Mycobacterium phage Piro94	NA	Viruses; dsDNA viruses, no RNA stage; Caudovirales; Siphoviridae; unclassified Siphoviridae
GCA_000929655.1	9524	4	Macrophomina phaseolina tobamolike virus	IL01 2013	Viruses; ssRNA viruses; ssRNA positive-strand viruses, no DNA stage; Virgaviridae; unclassified Virgaviridae
GCA_000987735.1	178171	277	Citrobacter phage Miller	NA	Viruses; dsDNA viruses, no RNA stage; Caudovirales; Myoviridae; Tevenvirinae; T4virus; unclassified T4virus
GCA_001041515.1	43227	54	Pseudomonas phage vB_PaeP_PPAABTNL	NA	Viruses; dsDNA viruses, no RNA stage; Caudovirales; Podoviridae; Autographivirinae; Phikmvvirus; unclassified phiKMV-like phages
GCA_001504355.1	86252	125	Escherichia phage HY02	NA	Viruses; dsDNA viruses, no RNA stage; Caudovirales; Myoviridae; unclassified Myoviridae
GCA_002756335.1	67496	92	Mycobacterium phage LizLemon	NA	Viruses; Duplodnaviria; Heunggongvirae; Uroviricota; Caudoviricetes; Caudovirales; Siphoviridae; Bclasvirinae; Rosebushvirus; Mycobacterium virus Rosebush

gca	genome.size	proteome.size	phage.name	strain	Taxonomy
GCA_001503555.1	49717	87	Mycobacterium phage Swirley	NA	Viruses; dsDNA viruses, no RNA stage; Caudovirales; Siphoviridae; unclassified Siphoviridae
GCA_001501075.1	50768	89	Mycobacterium phage Tiffany	NA	Viruses; dsDNA viruses, no RNA stage; Caudovirales; Siphoviridae; L5virus; unclassified L5likevirus
GCA_000955435.1	44716	65	Mycobacterium phage Trike	NA	Viruses; dsDNA viruses, no RNA stage; Caudovirales; Siphoviridae; unclassified Siphoviridae
GCA_000954715.1	57052	102	Mycobacterium phage Inventum	NA	Viruses; dsDNA viruses, no RNA stage; Caudovirales; Siphoviridae; unclassified Siphoviridae
GCA_002593065.1	30862	55	Lactococcus phage phi145	NA	Viruses; Duplodnaviria; Heunggongvirae; Uroviricota; Caudoviricetes; Caudovirales; Siphoviridae; Skunavirus; unclassified Skunavirus
GCA_002593025.1	31945	55	Lactococcus phage phi15	NA	Viruses; Duplodnaviria; Heunggongvirae; Uroviricota; Caudoviricetes; Caudovirales; Siphoviridae; Skunavirus; unclassified Skunavirus
GCA_002593045.1	31841	55	Lactococcus phage phi93	NA	Viruses; Duplodnaviria; Heunggongvirae; Uroviricota; Caudoviricetes; Caudovirales; Siphoviridae; Skunavirus; unclassified Skunavirus
GCA_000925855.1	156446	257	Rhizobium phage vB_RleM_P10VF	NA	Viruses; dsDNA viruses, no RNA stage; Caudovirales; Myoviridae; unclassified Myoviridae
GCA_000924995.1	45718	64	Mesorhizobium phage vB_MloP_Lo5R7ANS	NA	Viruses; dsDNA viruses, no RNA stage; Caudovirales; Podoviridae; unclassified Podoviridae
GCA_000922375.1	119638	115	Penaeus monodon nudivirus	NA	Viruses; dsDNA viruses, no RNA stage; Nudiviridae; unclassified Nudiviridae
GCA_002756355.1	50250	70	Vibrio phage ICP2_2011_A	NA	Viruses; Duplodnaviria; Heunggongvirae; Uroviricota; Caudoviricetes; Caudovirales; Podoviridae; unclassified Podoviridae
GCA_000921695.1	50440	57	Vibrio phage ICP2_2013_A_Haiti	NA	Viruses; dsDNA viruses, no RNA stage; Caudovirales; Podoviridae; unclassified Podoviridae
GCA_001443845.1	15618	6	Caprine parainfluenza virus 3	JS2013	Viruses; Riboviria; Orthornavirae; Negarnaviricota; Haplovirictina; Monjiviricetes; Mononegavirales; Paramyxoviridae; Orthoparamyxovirinae; Respirovirus; Bovine respirovirus 3
GCA_000921535.1	8886	5	Cladosporium cladosporioides virus 1	DF15	Viruses; unclassified viruses
GCA_003813225.1	7745	3	Norovirus GIHuJP2007GIP3_GI3ShimizuKK2866	NA	Viruses; Riboviria; Orthornavirae; Pisuviricota; Pisoniviricetes; Picornavirales; Caliciviridae; Norovirus; Norwalk virus; Norovirus GI; Norovirus genogroup 1 isolates
GCA_007609035.1	7543	3	Norovirus GIIHuJP2011GIIYuzawaGira2HS	NA	Viruses; Riboviria; Orthornavirae; Pisuviricota; Pisoniviricetes; Picornavirales; Caliciviridae; Norovirus; Norwalk virus; Norovirus GII; Norovirus GII isolates
GCA_000924355.1	40002	49	Ralstonia phage RSY1	NA	Viruses; dsDNA viruses, no RNA stage; Caudovirales; Myoviridae; Peduovirinae; P2virus; unclassified P2likevirus
GCA_002630405.1	8956	15	Ralstonia phage RSMSuper	NA	Viruses; Monodnaviria; Loebvirae; Hofneiviricota; Faserviricetes; Tubulavirales; Inoviridae; Habenivirus; Ralstonia virus RSM3; Ralstonia phage RSM3
GCA_003028955.1	2744	6	Jatropha leaf yellow mosaic Katarniaghat virus	NA	Viruses; Monodnaviria; Shotokuvirae; Cressdnaviricota; Repensiviricetes; Geplafuvirales; Geminiviridae; Begomovirus; Jatropha leaf yellow mosaic virus
GCA_003389975.1	7443	1	Picornaviridae sp	NA	Viruses; Riboviria; Orthornavirae; Pisuviricota; Pisoniviricetes; Picornavirales; Picornaviridae; unclassified Picornaviridae
GCA_001040995.1	73008	95	Pseudomonas phage Pa2	NA	Viruses; dsDNA viruses, no RNA stage; Caudovirales; Podoviridae; N4virus; unclassified N4likevirus
GCA_000923395.1	1773	2	Feline cyclovirus	NA	Viruses; ssDNA viruses; Circoviridae; unclassified Circoviridae; suggested genus Cyclovirus
GCA_013086585.1	11305	4	Rukutama virus	LEIV6269C	Viruses; Riboviria; Orthornavirae; Negarnaviricota; Polyploviricotina; Ellioviricetes; Bunyavirales; Phenuiviridae; Uukuvirus; Rukutama uukuvirus
GCA_000924055.1	3415	2	Alternaria longipes dsRNA virus 1	HN28	Viruses; dsRNA viruses; unclassified dsRNA viruses
GCA_000924035.1	6002	2	Penicillium roqueforti ssRNA mycovirus 1	PRG427	Viruses; ssRNA viruses; unclassified ssRNA viruses; Fusariviridae
GCA_003093015.1	23420	10	Duck reovirus SD12	NA	Viruses; Riboviria; Orthornavirae; Duplornaviricota; Resentoviricetes; Reovirales; Reoviridae; Spinareovirinae; Orthoreovirus; Avian orthoreovirus
GCA_000922555.1	5440	8	Coccinia mosaic Tamil Nadu virus	NA	Viruses; ssDNA viruses; Geminiviridae; Begomovirus; unclassified Begomovirus
GCA_000923455.1	8287	1	Chicken picornavirus 1	NA	Viruses; ssRNA viruses; ssRNA positive-strand viruses, no DNA stage; Picornavirales; Picornaviridae; unclassified Picornaviridae
GCA_000924115.1	7310	1	Chicken picornavirus 2	NA	Viruses; ssRNA viruses; ssRNA positive-strand viruses, no DNA stage; Picornavirales; Picornaviridae; unclassified Picornaviridae
GCA_000923475.1	7167	1	Chicken picornavirus 3	NA	Viruses; ssRNA viruses; ssRNA positive-strand viruses, no DNA stage; Picornavirales; Picornaviridae; unclassified Picornaviridae
GCA_000922415.1	9564	1	Chicken picornavirus 4	NA	Viruses; ssRNA viruses; ssRNA positive-strand viruses, no DNA stage; Picornavirales; Picornaviridae; unclassified Picornaviridae
GCA_000921575.1	9591	1	Chicken picornavirus 5	NA	Viruses; ssRNA viruses; ssRNA positive-strand viruses, no DNA stage; Picornavirales; Picornaviridae; unclassified Picornaviridae
GCA_013377415.1	43842	33	Duck aviadenovirus B	NA	Viruses; Varidnaviria; Bamfordvirae; Preplasmiviricota; Tectiliviricetes; Rowavirales; Adenoviridae; Aviadenovirus
GCA_000894115.1	196691	141	Saimiriine betaherpesvirus 4	SqSHV	Viruses; dsDNA viruses, no RNA stage; Herpesvirales; Herpesviridae; Betaherpesvirinae; Cytomegalovirus

gca	genome.size	proteome.size	phage.name	strain	Taxonomy
GCA_001430315.1	1387	1	Sida yellow vein mosaic alphasatellite	NA	Viruses; Alphasatellitidae; Geminialphasatellitinae; unclassified Begomovirus-associated alphasatellites; Mesta yellow vein mosaic virus alphasatellite
GCA_000930495.1	2914	3	Torque teno mini virus ALA22	NA	Viruses; ssDNA viruses; Anelloviridae; Betatorquevirus; unclassified Betatorquevirus
GCA_000929855.1	2855	2	Torque teno mini virus ALH8	NA	Viruses; ssDNA viruses; Anelloviridae; Betatorquevirus; unclassified Betatorquevirus
GCA_000930915.1	10609	4	Gairo mammarenavirus	TZ27421	Viruses; ssRNA viruses; ssRNA negative-strand viruses; Arenaviridae; Mammarenavirus
GCA_000927495.1	34773	56	Idiomarinaceae phage 1N22	NA	Viruses; dsDNA viruses, no RNA stage; unclassified dsDNA phages
GCA_001041135.1	37359	58	Pseudomonas phage H70	NA	Viruses; dsDNA viruses, no RNA stage; Caudovirales; Siphoviridae; unclassified Siphoviridae
GCA_000927415.1	40981	47	Escherichia phage J865	NA	Viruses; Duplodnaviria; Heunggongvirae; Uroviricota; Caudoviricetes; Caudovirales; Autographiviridae; Bonnellvirus; Escherichia virus J8-65
GCA_000954355.1	59419	110	Mycobacterium phage BuzzLyseyear	NA	Viruses; dsDNA viruses, no RNA stage; Caudovirales; Siphoviridae; unclassified Siphoviridae
GCA_002756375.1	68873	102	Mycobacterium phage Vivaldi	NA	Viruses; Duplodnaviria; Heunggongvirae; Uroviricota; Caudoviricetes; Caudovirales; Siphoviridae; Bclasvirinae; Pegunavirus; Mycobacterium virus Soto
GCA_000955075.1	60727	112	Mycobacterium phage Estave1	NA	Viruses; dsDNA viruses, no RNA stage; Caudovirales; Siphoviridae; unclassified Siphoviridae
GCA_001042215.1	28921	45	Leuconostoc phage Ln8	NA	Viruses; dsDNA viruses, no RNA stage; Caudovirales; Siphoviridae; unclassified Siphoviridae
GCA_001041855.1	28527	48	Leuconostoc phage Ln9	NA	Viruses; dsDNA viruses, no RNA stage; Caudovirales; Siphoviridae; unclassified Siphoviridae
GCA_000926875.1	38646	45	Yersinia phage vB_YenP_AP5	NA	Viruses; dsDNA viruses, no RNA stage; Caudovirales; Podoviridae; Autographivirinae; T7virus; unclassified T7-like viruses
GCA_000954295.1	43129	82	Edwardsiella phage GF2	NA	Viruses; dsDNA viruses, no RNA stage; Caudovirales; Myoviridae; unclassified Myoviridae
GCA_002634935.1	17454	11	Rotavirus ARatwtGERKS115732011G3P3	NA	Viruses; Riboviria; Orthornavirae; Duplornaviricota; Resentoviricetes; Reovirales; Reoviridae; Sedoreovirinae; Rotavirus; Rotavirus A; Rotavirus G3
GCA_003967895.1	2601	2	Porcine stoolassociated circular virus 7	NA	Viruses; ssDNA viruses; unclassified ssDNA viruses
GCA_008776395.1	8251	1	Kobuvirus SZAL6KoV2011HUN	NA	Viruses; Riboviria; Orthornavirae; Pisuviricota; Pisoniviricetes; Picornavirales; Picornaviridae; Kobuvirus; Aichivirus A
GCA_001431915.1	11072	7	Kumasi rhabdovirus	NA	Viruses; Riboviria; Orthornavirae; Negarnaviricota; Haploviricotina; Monjiviricetes; Mononegavirales; Rhabdoviridae; Ledantevirus; Kumasi ledantevirus
GCA_001503875.1	43745	41	Ralstonia phage RSJ5	NA	Viruses; dsDNA viruses, no RNA stage; Caudovirales; Podoviridae; unclassified Podoviridae
GCA_000921515.1	6598	3	Feline astrovirus D1	NA	Viruses; ssRNA viruses; ssRNA positive-strand viruses, no DNA stage; Astroviridae; Mamastrovirus; unclassified Mamastrovirus
GCA_004789735.1	12467	3	Pacui virus	BEAN27326	Viruses; Riboviria; Orthornavirae; Negarnaviricota; Polyploviricotina; Ellioviricetes; Bunyavirales; Peribunyaviridae; Pacuvirus; Pacui pacuvirus
GCA_004789755.1	12329	3	Rio Preto da Eva virus	BEAR540870	Viruses; Riboviria; Orthornavirae; Negarnaviricota; Polyploviricotina; Ellioviricetes; Bunyavirales; Peribunyaviridae; Pacuvirus; Rio Preto da Eva pacuvirus
GCA_004789775.1	12234	3	Tapirape virus	BEAN767592	Viruses; Riboviria; Orthornavirae; Negarnaviricota; Polyploviricotina; Ellioviricetes; Bunyavirales; Peribunyaviridae; Pacuvirus; Tapirape pacuvirus
GCA_000921715.1	4236	4	Ethiopian tobacco bushy top virus	NA	Viruses; ssRNA viruses; ssRNA positive-strand viruses, no DNA stage; Tombusviridae; Umbravirus; unclassified Umbravirus
GCA_000955015.2	56637	80	Vibrio phage VpKK5	NA	Viruses; dsDNA viruses, no RNA stage; Caudovirales; Siphoviridae; unclassified Siphoviridae
GCA_002149325.1	164302	241	Bacillus phage Mater	NA	Viruses; dsDNA viruses, no RNA stage; Caudovirales; Myoviridae; unclassified Myoviridae
GCA_002149605.1	161239	237	Bacillus phage Moonbeam	NA	Viruses; dsDNA viruses, no RNA stage; Caudovirales; Myoviridae; unclassified Myoviridae
GCA_002149225.1	39639	50	Bacillus phage Pascal	NA	Viruses; Duplodnaviria; Heunggongvirae; Uroviricota; Caudoviricetes; Caudovirales; Podoviridae; Pagevirus; Bacillus virus Pascal
GCA_002149425.1	40214	52	Bacillus phage Pookie	NA	Viruses; Duplodnaviria; Heunggongvirae; Uroviricota; Caudoviricetes; Caudovirales; Podoviridae; Pagevirus; Bacillus virus Pookie
GCA_002149585.1	87999	126	Citrobacter phage Moogle	NA	Viruses; Duplodnaviria; Heunggongvirae; Uroviricota; Caudoviricetes; Caudovirales; Myoviridae; Ounavirinae; Mooglevirus; Citrobacter virus Moogle
GCA_001041495.1	170341	298	Citrobacter phage Moon	NA	Viruses; dsDNA viruses, no RNA stage; Caudovirales; Myoviridae; unclassified Myoviridae
GCA_001041115.1	49816	90	Citrobacter virus Stevie	NA	Viruses; Duplodnaviria; Heunggongvirae; Uroviricota; Caudoviricetes; Caudovirales; Drexlerviridae; Tempevirinae; Tlsvirus
GCA_001042195.1	68365	85	Escherichia phage Pollock	NA	Viruses; dsDNA viruses, no RNA stage; Caudovirales; Podoviridae; unclassified Podoviridae
GCA_002149205.1	56776	88	Escherichia phage Seurat	NA	Viruses; Duplodnaviria; Heunggongvirae; Uroviricota; Caudoviricetes; Caudovirales; Siphoviridae; Seuratvirus; Escherichia virus Seurat

gca	genome.size	proteome.size	phage.name	strain	Taxonomy
GCA_002149405.1	123475	179	Salmonella virus Stitch	NA	Viruses; dsDNA viruses, no RNA stage; Caudovirales; Siphoviridae; T5virus
GCA_000929095.1	41880	58	Salmonella phage LSPA1	NA	Viruses; dsDNA viruses, no RNA stage; Caudovirales; Siphoviridae; unclassified Siphoviridae
GCA_000929955.1	10192	1	Dinocampus coccinellae paralysis virus	Quebec2013	Viruses; ssRNA viruses; ssRNA positive-strand viruses, no DNA stage; Picornavirales; Iflaviridae; Iflavirus
GCA_000926915.1	31491	9	Bat HpbetacoronavirusZhejiang2013	NA	Viruses; ssRNA viruses; ssRNA positive-strand viruses, no DNA stage; Nidovirales; Coronaviridae; Coronavirinae; Betacoronavirus; unclassified Betacoronavirus
GCA_000901135.1	8427	3	Mink calicivirus	MCVDL2007CN	Viruses; ssRNA viruses; ssRNA positive-strand viruses, no DNA stage; Caliciviridae; Vesivirus; unclassified Vesivirus
GCA_001884615.1	134927	171	Campylobacter phage PC14	NA	Viruses; dsDNA viruses, no RNA stage; Caudovirales; Myoviridae; Eucampyvirinae; Cp8virus; unclassified Cp8unalikevirus
GCA_002604945.1	131095	172	Campylobacter phage PC5	NA	Viruses; Duplodnaviria; Heunggongvirae; Uroviricota; Caudoviricetes; Caudovirales; Myoviridae; Eucampyvirinae; Fletchervirus; unclassified Fletchervirus
GCA_001500415.1	61511	95	Mycobacterium phage Omnicron	NA	Viruses; dsDNA viruses, no RNA stage; Caudovirales; Siphoviridae; unclassified Siphoviridae
GCA_002601285.1	69271	102	Mycobacterium phage Zonia	NA	Viruses; Duplodnaviria; Heunggongvirae; Uroviricota; Caudoviricetes; Caudovirales; Siphoviridae; Bclasvirinae; Pegunavirus; Mycobacterium virus Oline
GCA_001502455.1	58472	111	Mycobacterium phage Llama	NA	Viruses; dsDNA viruses, no RNA stage; Caudovirales; Siphoviridae; unclassified Siphoviridae
GCA_001501835.2	109801	245	Mycobacterium phage Ariel	NA	Viruses; dsDNA viruses, no RNA stage; Caudovirales; Siphoviridae; unclassified Siphoviridae
GCA_000925755.1	169062	267	Shigella phage Shf125875	NA	Viruses; dsDNA viruses, no RNA stage; Caudovirales; Myoviridae; Tevenvirinae; Rb69virus; Shigella virus UTAM
GCA_000925835.1	42351	51	Escherichia phage vB_EcoMep3	NA	Viruses; dsDNA viruses, no RNA stage; Caudovirales; Myoviridae; Cvm10virus; Escherichia virus ep3
GCA_002600285.1	68576	101	Mycobacterium phage Lasso	NA	Viruses; Duplodnaviria; Heunggongvirae; Uroviricota; Caudoviricetes; Caudovirales; Siphoviridae; Bclasvirinae; Pegunavirus; Mycobacterium virus Soto
GCA_000924975.1	59578	75	Salmonella virus Chi	NA	Viruses; Duplodnaviria; Heunggongvirae; Uroviricota; Caudoviricetes; Caudovirales; Siphoviridae; Chivirus
GCA_002756395.1	72646	94	Pseudomonas phage RWG	NA	Viruses; Duplodnaviria; Heunggongvirae; Uroviricota; Caudoviricetes; Caudovirales; Podoviridae; Litunavirus; Pseudomonas virus Ab09
GCA_002756415.1	73048	92	Pseudomonas phage phi176	NA	Viruses; Duplodnaviria; Heunggongvirae; Uroviricota; Caudoviricetes; Caudovirales; Podoviridae; Litunavirus; Pseudomonas virus Ab09
GCA_001501055.1	68871	97	Pseudomonas phage vB_Pae_PS44	NA	Viruses; dsDNA viruses, no RNA stage; Caudovirales; Myoviridae; unclassified Myoviridae
GCA_002602085.1	43047	54	Pseudomonas phage vB_Pae_PS9N	NA	Viruses; Duplodnaviria; Heunggongvirae; Uroviricota; Caudoviricetes; Caudovirales; Siphoviridae; Septimatrevirus; Pseudomonas virus Kakheti25
GCA_001500395.1	84583	153	Pseudomonas phage vB_PaeM_PS24	NA	Viruses; dsDNA viruses, no RNA stage; Caudovirales; Myoviridae; unclassified Myoviridae
GCA_002604985.1	39841	65	Pseudomonas phage JBD68	NA	Viruses; Duplodnaviria; Heunggongvirae; Uroviricota; Caudoviricetes; Caudovirales; Siphoviridae; unclassified Siphoviridae
GCA_002601885.1	152308	190	Dickeya phage phiD3	NA	Viruses; Duplodnaviria; Heunggongvirae; Uroviricota; Caudoviricetes; Caudovirales; Ackermannviridae; Aglimvirinae; Limestonevirus; Dickeya virus Limestone
GCA_003179635.1	7278	7	Human papillomavirus 197	NA	Viruses; Monodnaviria; Shotokuvirae; Cossaviricota; Papovaviricetes; Zurhausenvirales; Papillomaviridae; Firstpapillomavirinae; Gammapapillomavirus; Gammapapillomavirus 24
GCA_001503075.1	197361	246	Prochlorococcus phage PTIM68	NA	Viruses; dsDNA viruses, no RNA stage; Caudovirales; Myoviridae; unclassified Myoviridae
GCA_000927475.1	44366	49	Lelliottia phage phD2B	NA	Viruses; dsDNA viruses, no RNA stage; Caudovirales; Podoviridae; Autographivirinae; Sp6virus; unclassified Sp6likevirus
GCA_000926215.1	2232	2	Caribou fecesassociated gemycircularvirus	NA	Viruses; unclassified viruses; Genomoviridae; Gemycircularvirus; unclassified Gemycircularvirus
GCA_000922615.1	2231	2	Ancient caribou feces associated virus	NA	Viruses; unclassified viruses
GCA_001551105.1	52155	100	Mycobacterium phage VohminGhazi	NA	Viruses; dsDNA viruses, no RNA stage; Caudovirales; Siphoviridae; unclassified Siphoviridae
GCA_000989135.1	12292	7	Influenza D virus DbovineFrance29862012	NA	Viruses; ssRNA viruses; ssRNA negative-strand viruses; Orthomyxoviridae; unclassified Orthomyxoviridae; Influenzavirus D; Influenza D virus
GCA_001755025.1	39696	47	Salmonella phage BP12A	NA	Viruses; dsDNA viruses, no RNA stage; Caudovirales; Podoviridae; Autographivirinae; T7virus; unclassified T7-like viruses
GCA_001754725.1	43602	50	Salmonella phage BP12B	NA	Viruses; dsDNA viruses, no RNA stage; Caudovirales; Podoviridae; Autographivirinae; Sp6virus; unclassified Sp6likevirus
GCA_001754285.1	60606	76	Salmonella phage BP12C	NA	Viruses; dsDNA viruses, no RNA stage; Caudovirales; Siphoviridae; unclassified Siphoviridae
GCA_001755145.1	52437	76	Salmonella phage BP63	NA	Viruses; dsDNA viruses, no RNA stage; Caudovirales; unclassified Caudovirales
GCA_001745035.1	17641	20	Staphylococcus phage BP39	NA	Viruses; dsDNA viruses, no RNA stage; Caudovirales; Podoviridae; Picovirinae; P68virus; unclassified P68virus

gca	genome.size	proteome.size	phage.name	strain	Taxonomy
GCA_000924295.1	17656	13	Rose leaf rosetteassociated virus	NA	Viruses; ssRNA viruses; ssRNA positive-strand viruses, no DNA stage; Closteroviridae; Closterovirus; unclassified Closterovirus
GCA_009731775.1	12450	4	Mojui dos Campos virus	BeAn276121	Viruses; Riboviria; Orthornavirae; Negarnaviricota; Polyploviricotina; Ellioviricetes; Bunyavirales; Peribunyaviridae; Orthobunyavirus; unclassified Orthobunyavirus
GCA_000855865.1	15045	11	Little cherry virus 2	NA	Viruses; ssRNA viruses; ssRNA positive-strand viruses, no DNA stage; Closteroviridae; Ampelovirus
GCA_000921775.1	2367	3	Torque teno Tadarida brasiliensis virus	NA	Viruses; ssDNA viruses; Anelloviridae; unclassified Anelloviridae
GCA_000928195.1	5755	7	Microviridae IME16	NA	Viruses; ssDNA viruses; Microviridae; unclassified Microviridae
GCA_000925235.1	1359	1	Synedrella leaf curl alphasatellite	NA	Viruses; Satellites; Satellite Nucleic Acids; Single stranded DNA satellites; Alphasatellites; Babu- and nanovirus-associated alphasatellites; unclassified Babu- and nanovirus-associated alphasatellites
GCA_001712785.1	9443	1	Hepatitis C virus QC69	NA	Viruses; Riboviria; Orthornavirae; Kitrinoviricota; Flasuviricetes; Amarillovirales; Flaviviridae; Hepacivirus; Hepacivirus C; Hepatitis C virus genotype 7
GCA_003179615.1	7184	6	Human papillomavirus 199	NA	Viruses; Monodnaviria; Shotokuvirae; Cossaviricota; Papovaviricetes; Zurhausenvirales; Papillomaviridae; Firstpapillomavirinae; Gammapapillomavirus; Gammapapillomavirus 12
GCA_000925195.1	14927	13	Mikumi yellow baboon virus 1	NA	Viruses; ssRNA viruses; ssRNA positive-strand viruses, no DNA stage; Nidovirales; Arteriviridae; Arterivirus
GCA_000924335.1	14851	14	Southwest baboon virus 1	NA	Viruses; ssRNA viruses; ssRNA positive-strand viruses, no DNA stage; Nidovirales; Arteriviridae; unclassified Arteriviridae
GCA_000930875.1	145704	131	Tipula oleracea nudivirus	NA	Viruses; dsDNA viruses, no RNA stage; Nudiviridae; unclassified Nudiviridae
GCA_001925995.1	5991	4	Lake Sinai virus	WHCC111282	Viruses; Virus families not assigned to an order; Sinaivirus; unclassified Sinaivirus
GCA_000926235.1	7998	1	Salivirus FHB	NA	Viruses; ssRNA viruses; ssRNA positive-strand viruses, no DNA stage; Picornavirales; Picornaviridae; Salivirus; unclassified Salivirus
GCA_000930415.1	2735	7	Jatropha leaf crumple virus	NA	Viruses; ssDNA viruses; Geminiviridae; Begomovirus; unclassified Begomovirus
GCA_000924415.1	5322	2	Botryosphaeria dothidea victorivirus 1	NA	Viruses; dsRNA viruses; Totiviridae; Victorivirus; unclassified Victorivirus
GCA_000850225.1	4268	4	Acinetobacter phage AP205	NA	Viruses; ssRNA viruses; ssRNA positive-strand viruses, no DNA stage; Leviviridae; Levivirus; unclassified Levivirus
GCA_000851485.1	826	2	Panicum mosaic satellite virus	NA	Viruses; Virus families not assigned to an order; Papanivirus; Panicum papanivirus 1
GCA_000924395.1	2439	3	Gyrovirus GyV7SF	NA	Viruses; ssDNA viruses; Circoviridae; Gyrovirus; unclassified Gyrovirus
GCA_000926895.1	859	1	Circoviridae SFBeef	NA	Viruses; ssDNA viruses; Circoviridae; unclassified Circoviridae
GCA_002366025.1	8870	6	Parrot bornavirus 1	NA	Viruses; Riboviria; Orthornavirae; Negarnaviricota; Haploviricotina; Monjiviricetes; Mononegavirales; Bornaviridae; Orthobornavirus; Psittaciform 1 orthobornavirus
GCA_002366345.1	8914	6	Parrot bornavirus 2	NA	Viruses; Riboviria; Orthornavirae; Negarnaviricota; Haploviricotina; Monjiviricetes; Mononegavirales; Bornaviridae; Orthobornavirus; Psittaciform 1 orthobornavirus
GCA_001430055.1	8915	6	Parrot bornavirus 4	NA	Viruses; Riboviria; Orthornavirae; Negarnaviricota; Haploviricotina; Monjiviricetes; Mononegavirales; Bornaviridae; Orthobornavirus; Psittaciform 1 orthobornavirus
GCA_002366005.1	8901	6	Parrot bornavirus 7	NA	Viruses; Riboviria; Orthornavirae; Negarnaviricota; Haploviricotina; Monjiviricetes; Mononegavirales; Bornaviridae; Orthobornavirus; Psittaciform 1 orthobornavirus
GCA_002987395.1	93848	175	Pseudomonas phage vB_PaeM_C210_Ab02	NA	Viruses; Duplodnaviria; Heunggongvirae; Uroviricota; Caudoviricetes; Caudovirales; Myoviridae; Pakpunavirus; Pseudomonas virus CAb02
GCA_000955355.1	86246	156	Pseudomonas phage vB_PaeM_PAO1_Ab03	NA	Viruses; dsDNA viruses, no RNA stage; Caudovirales; Myoviridae; Kpp10virus; Pseudomonas virus Ab03
GCA_000954635.1	43639	54	Pseudomonas phage vB_PaeP_PAO1_Ab05	NA	Viruses; dsDNA viruses, no RNA stage; Caudovirales; Podoviridae; unclassified Podoviridae
GCA_003147005.1	93503	175	Pseudomonas phage vB_PaeM_C210_Ab08	NA	Viruses; Duplodnaviria; Heunggongvirae; Uroviricota; Caudoviricetes; Caudovirales; Myoviridae; Pakpunavirus; Pseudomonas virus CAb1
GCA_003147025.1	83598	149	Pseudomonas phage vB_PaeM_PAO1_Ab17	NA	Viruses; Duplodnaviria; Heunggongvirae; Uroviricota; Caudoviricetes; Caudovirales; Myoviridae; Nankokuvirus; Pseudomonas virus Ab03
GCA_000954275.1	56537	76	Pseudomonas phage vB_PaeS_PAO1_Ab18	NA	Viruses; dsDNA viruses, no RNA stage; Caudovirales; Siphoviridae; unclassified Siphoviridae
GCA_000954995.1	45808	71	Pseudomonas phage vB_PaeP_C210_Ab22	NA	Viruses; dsDNA viruses, no RNA stage; Caudovirales; Podoviridae; unclassified Podoviridae
GCA_000954955.1	66299	93	Pseudomonas phage vB_PaeM_PAO1_Ab27	NA	Viruses; dsDNA viruses, no RNA stage; Caudovirales; Myoviridae; unclassified Myoviridae
GCA_003147045.1	42750	49	Pseudomonas phage vB_PaeP_PAO1_115pyo	NA	Viruses; Duplodnaviria; Heunggongvirae; Uroviricota; Caudoviricetes; Caudovirales; Autographiviridae; Krylovirinae; Phikmvvirus; Pseudomonas virus 15pyo
GCA_003147065.1	86668	36	Pseudomonas phage vB_PaeM_PAO1_Ab04	NA	Viruses; Duplodnaviria; Heunggongvirae; Uroviricota; Caudoviricetes; Caudovirales; Myoviridae; Nankokuvirus; Pseudomonas virus Ab03
GCA_003147085.1	84759	36	Pseudomonas phage vB_PaeM_PAO1_Ab06	NA	Viruses; Duplodnaviria; Heunggongvirae; Uroviricota; Caudoviricetes; Caudovirales; Myoviridae; Nankokuvirus; Pseudomonas virus Ab03

gca	genome.size	proteome.size	phage.name	strain	Taxonomy
GCA_000954615.1	37238	59	Pseudomonas phage vB_PaeS_PAO1_Ab30	NA	Viruses; dsDNA viruses, no RNA stage; Caudovirales; Siphoviridae; unclassified Siphoviridae
GCA_001630085.1	10323	10	Tilapia lake virus	NA	Viruses; ssRNA viruses; ssRNA negative-strand viruses; Orthomyxoviridae; unclassified Orthomyxoviridae
GCA_001432135.1	16133	11	Koolpinyah virus	NA	Viruses; ssRNA viruses; ssRNA negative-strand viruses; Mononegavirales; Rhabdoviridae; Ephemerovirus; unclassified Ephemerovirus
GCA_000838905.1	38297	58	Salmonella phage HK620	NA	Viruses; dsDNA viruses, no RNA stage; Caudovirales; Podoviridae; P22virus
GCA_000930595.1	164999	266	Shigella phage pSs1	NA	Viruses; dsDNA viruses, no RNA stage; Caudovirales; Myoviridae; Tevenvirinae; T4virus; Shigella virus Pss1
GCA_002605005.1	50066	87	Mycobacterium phage QuinnKiro	NA	Viruses; Duplodnaviria; Heunggongvirae; Uroviricota; Caudoviricetes; Caudovirales; Siphoviridae; Fromanvirus; unclassified Fromanvirus
GCA_001502435.1	52530	91	Mycobacterium phage Abrogate	NA	Viruses; dsDNA viruses, no RNA stage; Caudovirales; Siphoviridae; unclassified Siphoviridae
GCA_001501815.1	58985	106	Mycobacterium phage Bipolar	NA	Viruses; dsDNA viruses, no RNA stage; Caudovirales; Siphoviridae; unclassified Siphoviridae
GCA_000926975.1	5145	6	Sea otter polyomavirus 1	NA	Viruses; dsDNA viruses, no RNA stage; Polyomaviridae; unclassified Polyomaviridae
GCA_001432055.1	12162	7	Santa barbara virus	AR775619	Viruses; ssRNA viruses; ssRNA negative-strand viruses; Mononegavirales; Rhabdoviridae; unclassified Rhabdoviridae
GCA_001707005.1	9863	3	Brejeira virus	AR800208B	Viruses; ssRNA viruses; ssRNA positive-strand viruses, no DNA stage; unclassified ssRNA positive-strand viruses; Negevirus
GCA_002605025.1	53395	95	Mycobacterium phage Power	NA	Viruses; Duplodnaviria; Heunggongvirae; Uroviricota; Caudoviricetes; Caudovirales; Siphoviridae; Fromanvirus; unclassified Fromanvirus
GCA_000955415.1	74806	79	Lactobacillus phage Ldl1	NA	Viruses; dsDNA viruses, no RNA stage; Caudovirales; Siphoviridae; unclassified Siphoviridae
GCA_009731795.1	12848	4	Calchaqui virus	AG 831347	Viruses; Riboviria; Orthornavirae; Negarnaviricota; Polyploviricotina; Ellioviricetes; Bunyavirales; Peribunyaviridae; Orthobunyavirus; unclassified Orthobunyavirus
GCA_002831185.1	13166	4	Alajuela virus	MARU 11079	Viruses; Riboviria; Orthornavirae; Negarnaviricota; Polyploviricotina; Ellioviricetes; Bunyavirales; Peribunyaviridae; Orthobunyavirus; Alajuela orthobunyavirus
GCA_000849445.1	5358	2	Eimeria brunetti RNA virus 1	NA	Viruses; dsRNA viruses; Totiviridae; unclassified Totiviridae
GCA_001431975.1	15522	7	Teviot virus	NA	Viruses; ssRNA viruses; ssRNA negative-strand viruses; Mononegavirales; Paramyxoviridae; unclassified Paramyxoviridae
GCA_001501035.1	53042	96	Mycobacterium phage Equemioh13	NA	Viruses; dsDNA viruses, no RNA stage; Caudovirales; Siphoviridae; unclassified Siphoviridae
GCA_000926855.1	348532	611	Escherichia phage 121Q	NA	Viruses; dsDNA viruses, no RNA stage; Caudovirales; Myoviridae; unclassified Myoviridae
GCA_002605045.1	43865	59	Klebsiella phage phiBO1E	NA	Viruses; Duplodnaviria; Heunggongvirae; Uroviricota; Caudoviricetes; Caudovirales; Autographiviridae; Slopekvirinae; Drulisvirus; Klebsiella virus BO1E
GCA_000925815.1	59601	82	Ruegeria phage DSS3P1	NA	Viruses; dsDNA viruses, no RNA stage; Caudovirales; Siphoviridae; unclassified Siphoviridae
GCA_001505995.1	43680	71	Mycobacterium phage Carcharodon	NA	Viruses; dsDNA viruses, no RNA stage; Caudovirales; Siphoviridae; unclassified Siphoviridae
GCA_002605065.1	42596	61	Mycobacterium phage RonRayGun	NA	Viruses; Duplodnaviria; Heunggongvirae; Uroviricota; Caudoviricetes; Caudovirales; Siphoviridae; Bernalvirus; unclassified Bernalvirus
GCA_001517015.2	53184	95	Mycobacterium phage SweetiePie	NA	Viruses; dsDNA viruses, no RNA stage; Caudovirales; Siphoviridae; unclassified Siphoviridae
GCA_001041835.1	88403	132	Escherichia phage vB_EcoMVpaE1	NA	Viruses; dsDNA viruses, no RNA stage; Caudovirales; Myoviridae; unclassified Myoviridae
GCA_001505335.1	57328	107	Mycobacterium phage CaptainTrips	NA	Viruses; dsDNA viruses, no RNA stage; Caudovirales; Siphoviridae; unclassified Siphoviridae
GCA_001550725.1	133531	235	Streptomyces phage Jay2Jay	NA	Viruses; dsDNA viruses, no RNA stage; Caudovirales; Siphoviridae; unclassified Siphoviridae
GCA_001440895.1	29767	32	Yellowstone Lake virophage 5	NA	Viruses; unclassified virophages
GCA_001440975.1	24837	29	Yellowstone Lake virophage 6	NA	Viruses; unclassified virophages
GCA_001441055.1	23193	26	Yellowstone Lake virophage 7	NA	Viruses; unclassified virophages
GCA_002599745.1	39542	48	Vibrio phage CJY	NA	Viruses; Duplodnaviria; Heunggongvirae; Uroviricota; Caudoviricetes; Caudovirales; Podoviridae; Enhodamvirus; unclassified Enhodamvirus
GCA_002599765.1	39530	48	Vibrio phage H1	NA	Viruses; Duplodnaviria; Heunggongvirae; Uroviricota; Caudoviricetes; Caudovirales; Podoviridae; Enhodamvirus; unclassified Enhodamvirus
GCA_002599805.1	39530	48	Vibrio phage H3	NA	Viruses; Duplodnaviria; Heunggongvirae; Uroviricota; Caudoviricetes; Caudovirales; Podoviridae; Enhodamvirus; unclassified Enhodamvirus
GCA_001041475.1	39530	48	Vibrio phage J2	NA	Viruses; dsDNA viruses, no RNA stage; Caudovirales; Podoviridae; unclassified Podoviridae

gca	genome.size	proteome.size	phage.name	strain	Taxonomy
GCA_002599825.1	39782	49	Vibrio phage J3	NA	Viruses; Duplodnaviria; Heunggongvirae; Uroviricota; Caudoviricetes; Caudovirales; Podoviridae; Enhodamvirus; unclassified Enhodamvirus
GCA_001041095.1	39725	48	Vibrio phage QH	NA	Viruses; dsDNA viruses, no RNA stage; Caudovirales; Podoviridae; unclassified Podoviridae
GCA_001042175.1	41480	25	Proteus phage PM 75	NA	Viruses; dsDNA viruses, no RNA stage; Caudovirales; Podoviridae; unclassified Podoviridae
GCA_001041815.1	43642	23	Proteus phage PM 85	NA	Viruses; dsDNA viruses, no RNA stage; Caudovirales; Podoviridae; unclassified Podoviridae
GCA_001041455.1	45169	47	Proteus phage PM 93	NA	Viruses; dsDNA viruses, no RNA stage; Caudovirales; Podoviridae; unclassified Podoviridae
GCA_001503715.1	52967	92	Mycobacterium phage Larenn	NA	Viruses; dsDNA viruses, no RNA stage; Caudovirales; Siphoviridae; unclassified Siphoviridae
GCA_001504515.1	60609	95	Mycobacterium phage Murucutumbu	NA	Viruses; dsDNA viruses, no RNA stage; Caudovirales; Siphoviridae; unclassified Siphoviridae
GCA_001041075.1	130008	178	Lactococcus phage WRP3	NA	Viruses; dsDNA viruses, no RNA stage; Caudovirales; Siphoviridae; unclassified Siphoviridae
GCA_013087275.1	4424	3	Tusavirus 1	Tu491	Viruses; Monodnaviria; Shotokuvirae; Cossaviricota; Quintoviricetes; Piccovirales; Parvoviridae; Parvovirinae; Protoparvovirus; unclassified Protoparvovirus
GCA_001042155.1	98170	162	Acinetobacter phage YMC1303R2096	NA	Viruses; dsDNA viruses, no RNA stage; Caudovirales; Myoviridae; unclassified Myoviridae
GCA_000928615.1	32661	34	Simian adenovirus DM2014	NA	Viruses; dsDNA viruses, no RNA stage; Adenoviridae; Mastadenovirus; unclassified Simian adenoviruses
GCA_000926435.1	9165	3	Goutanap virus	F33CI2004	Viruses; ssRNA viruses; ssRNA positive-strand viruses, no DNA stage; unclassified ssRNA positive-strand viruses
GCA_000924835.1	369360	448	Melbournevirus	NA	Viruses; dsDNA viruses, no RNA stage; Marseilleviridae; unclassified Marseilleviridae
GCA_006451795.1	191718	189	Heliothis virescens ascovirus 3j	NA	Viruses; Varidnaviria; Bamfordvirae; Nucleocytoviricota; Megaviricetes; Pimascovirales; Ascoviridae; Ascovirus; Heliothis virescens ascovirus 3a
GCA_000928595.1	7661	4	Yacon necrotic mottle virus	NA	Viruses; Riboviria; Pararnavirae; Artverviricota; Revtraviricetes; Ortervirales; Caulimoviridae; Badnavirus
GCA_002220005.1	12031	3	Motherwort yellow mottle virus	NA	Viruses; ssRNA viruses; ssRNA positive-strand viruses, no DNA stage; Picornavirales; Secoviridae; Torradovirus
GCA_000925395.1	5654	2	Ustilaginoidea virens RNA virus L	NA	Viruses; dsRNA viruses; Totiviridae; Victorivirus; unclassified Victorivirus
GCA_000924555.1	2714	1	Ustilaginoidea virens RNA virus M	NA	Viruses; dsRNA viruses; Totiviridae; Victorivirus; unclassified Victorivirus
GCA_000930535.1	4861	5	Bovine polyomavirus 3	NA	Viruses; dsDNA viruses, no RNA stage; Polyomaviridae; unclassified Polyomaviridae
GCA_000927675.1	7496	1	Norway rat hunnivirus	NA	Viruses; ssRNA viruses; ssRNA positive-strand viruses, no DNA stage; Picornavirales; Picornaviridae; Hunnivirus; unclassified Hunnivirus
GCA_000929635.1	10023	1	Norway rat pegivirus	NA	Viruses; ssRNA viruses; ssRNA positive-strand viruses, no DNA stage; Flaviviridae; Pegivirus; unclassified Pegivirus
GCA_014883375.1	7433	1	Rat picornavirus	NA	Viruses; Riboviria; Orthornavirae; Pisuviricota; Pisoniviricetes; Picornavirales; Picornaviridae; unclassified Picornaviridae
GCA_001038595.1	39167	46	Pseudomonas phage Pf10	NA	Viruses; dsDNA viruses, no RNA stage; Caudovirales; Podoviridae; Autographivirinae; T7virus; unclassified T7-like viruses
GCA_000955475.1	2414	1	Fusarium globosum mitovirus 1	NA	Viruses; ssRNA viruses; ssRNA positive-strand viruses, no DNA stage; Narnaviridae; Mitovirus; unclassified Mitovirus
GCA_000955595.1	2423	1	Fusarium coeruleum mitovirus 1	NA	Viruses; ssRNA viruses; ssRNA positive-strand viruses, no DNA stage; Narnaviridae; Mitovirus; unclassified Mitovirus
GCA_001465085.1	135952	131	Sucra jujuba nucleopolyhedrovirus	NA	Viruses; dsDNA viruses, no RNA stage; Baculoviridae; Alphabaculovirus; unclassified Alphabaculovirus
GCA_002037815.1	73882	88	Delftia phage RG2014	NA	Viruses; dsDNA viruses, no RNA stage; Caudovirales; Podoviridae; unclassified Podoviridae
GCA_001041355.1	162160	145	Erwinia phage phiEa2809	NA	Viruses; dsDNA viruses, no RNA stage; Caudovirales; Myoviridae; unclassified Myoviridae
GCA_013086595.1	11548	4	Kismayo virus	NA	Viruses; Riboviria; Orthornavirae; Negarnaviricota; Polyploviricotina; Ellioviricetes; Bunyavirales; Phenuiviridae; Phlebovirus; unclassified Phlebovirus; Bhanja serogroup
GCA_013086605.1	11353	4	Silverwater virus	NA	Viruses; Riboviria; Orthornavirae; Negarnaviricota; Polyploviricotina; Ellioviricetes; Bunyavirales; Phenuiviridae; Uukuvirus; Silverwater uukuvirus
GCA_013090055.1	7570	1	Bovine picornavirus	Bo11392009JPN	Viruses; ssRNA viruses; ssRNA positive-strand viruses, no DNA stage; Picornavirales; Picornaviridae; unclassified Picornaviridae
GCA_004117475.1	11464	4	Kaisodi virus	NA	Viruses; Riboviria; Orthornavirae; Negarnaviricota; Polyploviricotina; Ellioviricetes; Bunyavirales; Phenuiviridae; Uukuvirus; Kaisodi uukuvirus
GCA_000925155.1	8489	3	San Miguel sea lion virus 8	NA	Viruses; ssRNA viruses; ssRNA positive-strand viruses, no DNA stage; Caliciviridae; Vesivirus; unclassified Vesivirus
GCA_000927455.1	5986	6	Strawberry polerovirus 1	NA	Viruses; ssRNA viruses; ssRNA positive-strand viruses, no DNA stage; Luteoviridae; Polerovirus; unclassified Polerovirus

gca	genome.size	proteome.size	phage.name	strain	Taxonomy
GCA_001431895.1	2923	2	Colletotrichum higginsianum nonsegmented dsRNA virus 1	NA	Viruses; Riboviria; dsRNA viruses; unclassified dsRNA viruses
GCA_000930795.1	2223	1	Seal anellovirus 4	NA	Viruses; ssDNA viruses; Anelloviridae; unclassified Anelloviridae
GCA_001310195.1	10761	1	Paraiso Escondido virus	NA	Viruses; ssRNA viruses; ssRNA positive-strand viruses, no DNA stage; Flaviviridae; Flavivirus; unclassified Flavivirus
GCA_006450895.1	170141	165	Western grey kangaroopox virus	Western Australia	Viruses; Varidnaviria; Bamfordvirae; Nucleocytoviricota; Pokkesviricetes; Chitovirales; Poxviridae; Chordopoxvirinae; Macropopoxvirus; Western kangaroopox virus
GCA_000927955.1	11958	7	Wellfleet Bay virus	NA	Viruses; ssRNA viruses; ssRNA negative-strand viruses; Orthomyxoviridae; Quaranjavirus; unclassified Quaranjavirus
GCA_013087285.1	3661	1	Protoparvovirus HK2014	NA	Viruses; Monodnaviria; Shotokuvirae; Cossaviricota; Quintoviricetes; Piccovirales; Parvoviridae; Parvovirinae; Protoparvovirus; unclassified Protoparvovirus
GCA_000929115.1	6006	2	Eimeria tenella RNA virus 1	JL610V	Viruses; dsRNA viruses; Totiviridae; Victorivirus; unclassified Victorivirus
GCA_000927935.1	10889	1	Laodelphax striatellus picornalike virus 2	NA	Viruses; ssRNA viruses; ssRNA positive-strand viruses, no DNA stage; Picornavirales; Iflaviridae; unclassified Iflaviridae
GCA_000857745.1	5722	4	Beet mild yellowing virus	NA	Viruses; ssRNA viruses; ssRNA positive-strand viruses, no DNA stage; Luteoviridae; Polerovirus
GCA_001041795.1	33756	50	Streptococcus phage SpSL1	NA	Viruses; dsDNA viruses, no RNA stage; Caudovirales; Siphoviridae; unclassified Siphoviridae
GCA_000954695.1	71200	101	Arthrobacter phage vB_ArtMArV1	NA	Viruses; dsDNA viruses, no RNA stage; Caudovirales; Myoviridae; unclassified Myoviridae
GCA_002598565.1	157204	231	Mycobacterium phage ZygoTaiga	NA	Viruses; Duplodnaviria; Heunggongvirae; Uroviricota; Caudoviricetes; Caudovirales; Myoviridae; Bixzunavirus; Mycobacterium virus Bxz1
GCA_001041435.1	73050	90	Pseudomonas phage YH6	NA	Viruses; dsDNA viruses, no RNA stage; Caudovirales; Podoviridae; N4virus; unclassified N4likevirus
GCA_001041055.1	44613	62	Escherichia phage YD2008s	NA	Viruses; dsDNA viruses, no RNA stage; Caudovirales; Siphoviridae; unclassified Siphoviridae
GCA_001505975.1	110764	250	Mycobacterium phage MiaZeal	NA	Viruses; dsDNA viruses, no RNA stage; Caudovirales; Siphoviridae; unclassified Siphoviridae
GCA_002605085.1	108418	133	Enterobacteria phage DT5712	NA	Viruses; Duplodnaviria; Heunggongvirae; Uroviricota; Caudoviricetes; Caudovirales; Demerecviridae; Markadamsvirinae; Tequintavirus; unclassified Tequintavirus
GCA_002149505.1	47020	67	Clostridium phage phiCT19406A	NA	Viruses; dsDNA viruses, no RNA stage; unclassified dsDNA phages
GCA_002149685.1	38243	66	Clostridium phage phiCT19406B	NA	Viruses; dsDNA viruses, no RNA stage; unclassified dsDNA phages
GCA_001505355.1	35703	63	Clostridium phage phiCT19406C	NA	Viruses; dsDNA viruses, no RNA stage; unclassified dsDNA phages
GCA_002149745.1	42158	62	Clostridium phage phiCT453A	NA	Viruses; dsDNA viruses, no RNA stage; unclassified dsDNA phages
GCA_002149525.1	36712	61	Clostridium phage phiCT453B	NA	Viruses; dsDNA viruses, no RNA stage; Caudovirales; Siphoviridae; unclassified Siphoviridae
GCA_001503735.1	46895	77	Clostridium phage phiCT9441A	NA	Viruses; dsDNA viruses, no RNA stage; unclassified dsDNA phages
GCA_002149705.1	47020	67	Clostridium phage phiCTC2A	NA	Viruses; dsDNA viruses, no RNA stage; unclassified dsDNA phages
GCA_002149825.1	38243	66	Clostridium phage phiCTC2B	NA	Viruses; dsDNA viruses, no RNA stage; unclassified dsDNA phages
GCA_001041695.1	37146	55	Shigella phage SfMu	NA	Viruses; Duplodnaviria; Heunggongvirae; Uroviricota; Caudoviricetes; Caudovirales; Myoviridae; Muvirus; Shigella virus SfMu
GCA_001041335.1	87510	126	Salmonella phage vB_SPuM_SP116	NA	Viruses; Duplodnaviria; Heunggongvirae; Uroviricota; Caudoviricetes; Caudovirales; Myoviridae; Ounavirinae; Felixounavirus; Salmonella virus SP116
GCA_001505315.1	170473	271	Escherichia phage vb_EcoMVR5	NA	Viruses; Duplodnaviria; Heunggongvirae; Uroviricota; Caudoviricetes; Caudovirales; Myoviridae; Tevenvirinae; Dhakavirus; Escherichia virus VR5
GCA_001503695.1	170336	286	Escherichia phage vB_EcoM_VR20	NA	Viruses; dsDNA viruses, no RNA stage; Caudovirales; Myoviridae; Tevenvirinae; Sp18virus; Escherichia virus VR20
GCA_001504495.1	170822	293	Escherichia phage vB_EcoM_VR25	NA	Viruses; dsDNA viruses, no RNA stage; Caudovirales; Myoviridae; Tevenvirinae; Sp18virus; Escherichia virus VR25
GCA_001505955.1	171541	291	Escherichia phage vB_EcoM_VR26	NA	Viruses; dsDNA viruses, no RNA stage; Caudovirales; Myoviridae; Tevenvirinae; Sp18virus; Escherichia virus VR26
GCA_000925135.1	23170	9	Fako virus	CSW77	Viruses; dsRNA viruses; Reoviridae; Spinareovirinae; Dinovernavirus; unclassified Dinovernavirus
GCA_002605105.1	49267	85	Mycobacterium phage Phoxy	NA	Viruses; Duplodnaviria; Heunggongvirae; Uroviricota; Caudoviricetes; Caudovirales; Siphoviridae; Fromanvirus; unclassified Fromanvirus
GCA_002605125.1	50989	91	Mycobacterium phage Spike509	NA	Viruses; Duplodnaviria; Heunggongvirae; Uroviricota; Caudoviricetes; Caudovirales; Siphoviridae; Fromanvirus; unclassified Fromanvirus
GCA_001551425.1	31703	50	Lactobacillus phage LfeSau	NA	Viruses; dsDNA viruses, no RNA stage; Caudovirales; Siphoviridae; unclassified Siphoviridae

gca	genome.size	proteome.size	phage.name	strain	Taxonomy
GCA_002605145.2	69451	103	Mycobacterium phage Chandler	NA	Viruses; Duplodnaviria; Heunggongvirae; Uroviricota; Caudoviricetes; Caudovirales; Siphoviridae; Bclasvirinae; Pipefishvirus; unclassified Pipefishvirus
GCA_000954335.1	49577	86	Mycobacterium phage Alvin	NA	Viruses; dsDNA viruses, no RNA stage; Caudovirales; Siphoviridae; unclassified Siphoviridae
GCA_002605165.1	78229	148	Mycobacterium phage Cosmo	NA	Viruses; Duplodnaviria; Heunggongvirae; Uroviricota; Caudoviricetes; Caudovirales; Siphoviridae; Wildcatvirus; unclassified Wildcatvirus
GCA_001551745.1	51492	91	Mycobacterium phage Edtherson	NA	Viruses; dsDNA viruses, no RNA stage; Caudovirales; Siphoviridae; unclassified Siphoviridae
GCA_002756455.1	68716	103	Mycobacterium phage FluffyNinja	NA	Viruses; Duplodnaviria; Heunggongvirae; Uroviricota; Caudoviricetes; Caudovirales; Siphoviridae; Bclasvirinae; Pegunavirus; Mycobacterium virus Soto
GCA_002605185.1	51376	88	Mycobacterium phage Gadost	NA	Viruses; Duplodnaviria; Heunggongvirae; Uroviricota; Caudoviricetes; Caudovirales; Siphoviridae; Fromanvirus; unclassified Fromanvirus
GCA_000955055.1	61251	101	Mycobacterium phage Keshu	NA	Viruses; dsDNA viruses, no RNA stage; Caudovirales; Siphoviridae; unclassified Siphoviridae
GCA_000955395.1	46869	79	Mycobacterium phage Malithi	NA	Viruses; dsDNA viruses, no RNA stage; Caudovirales; Siphoviridae; unclassified Siphoviridae
GCA_000954675.1	58211	94	Mycobacterium phage Milly	NA	Viruses; dsDNA viruses, no RNA stage; Caudovirales; Siphoviridae; unclassified Siphoviridae
GCA_002601665.1	68328	99	Mycobacterium phage Pipsqueak	NA	Viruses; Duplodnaviria; Heunggongvirae; Uroviricota; Caudoviricetes; Caudovirales; Siphoviridae; Bclasvirinae; Pegunavirus; Mycobacterium virus Soto
GCA_000954315.1	55832	89	Mycobacterium phage Sbash	NA	Viruses; dsDNA viruses, no RNA stage; Caudovirales; Siphoviridae; unclassified Siphoviridae
GCA_002756475.1	68311	101	Mycobacterium phage Sigman	NA	Viruses; Duplodnaviria; Heunggongvirae; Uroviricota; Caudoviricetes; Caudovirales; Siphoviridae; Bclasvirinae; Pegunavirus; Mycobacterium virus Pg1
GCA_002599945.1	50877	91	Mycobacterium phage Taurus	NA	Viruses; Duplodnaviria; Heunggongvirae; Uroviricota; Caudoviricetes; Caudovirales; Siphoviridae; Fromanvirus; unclassified Fromanvirus
GCA_002605205.1	53058	98	Mycobacterium phage Thor	NA	Viruses; Duplodnaviria; Heunggongvirae; Uroviricota; Caudoviricetes; Caudovirales; Siphoviridae; Fromanvirus; unclassified Fromanvirus
GCA_002605225.1	53008	94	Mycobacterium phage Treddle	NA	Viruses; Duplodnaviria; Heunggongvirae; Uroviricota; Caudoviricetes; Caudovirales; Siphoviridae; Fromanvirus; unclassified Fromanvirus
GCA_002605245.1	51370	84	Mycobacterium phage HamSlice	NA	Viruses; Duplodnaviria; Heunggongvirae; Uroviricota; Caudoviricetes; Caudovirales; Siphoviridae; Fromanvirus; unclassified Fromanvirus
GCA_001551085.1	106071	124	Lactobacillus phage LfeInf	NA	Viruses; dsDNA viruses, no RNA stage; Caudovirales; Myoviridae; unclassified Myoviridae
GCA_001505295.1	58546	70	Proteus phage pPM_01	NA	Viruses; dsDNA viruses, no RNA stage; Caudovirales; Siphoviridae; unclassified Siphoviridae
GCA_000930115.1	35814	53	Enterobacteria phage P88	NA	Viruses; dsDNA viruses, no RNA stage; Caudovirales; Myoviridae; unclassified Myoviridae
GCA_001736435.1	39873	56	Bacillus phage BalMu1	NA	Viruses; dsDNA viruses, no RNA stage; unclassified dsDNA phages
GCA_000929135.1	8952	2	Botrytis cinerea RNA virus 1	BerBc1	Viruses; dsRNA viruses; unclassified dsRNA viruses
GCA_000927635.1	2547	4	Chickpea yellow dwarf virus	NA	Viruses; ssDNA viruses; Geminiviridae; Mastrevirus; unclassified Mastrevirus
GCA_001551605.1	8628	6	Elderberry carlavirus A	NA	Viruses; ssRNA viruses; ssRNA positive-strand viruses, no DNA stage; Tymovirales; Betaflexiviridae; Quinvirinae; Carlavirus; unclassified Carlavirus
GCA_001550925.1	8607	6	Elderberry carlavirus B	NA	Viruses; ssRNA viruses; ssRNA positive-strand viruses, no DNA stage; Tymovirales; Betaflexiviridae; Quinvirinae; Carlavirus; unclassified Carlavirus
GCA_001550545.1	8613	6	Elderberry carlavirus C	NA	Viruses; ssRNA viruses; ssRNA positive-strand viruses, no DNA stage; Tymovirales; Betaflexiviridae; Quinvirinae; Carlavirus; unclassified Carlavirus
GCA_001551245.1	8612	6	Elderberry carlavirus D	NA	Viruses; ssRNA viruses; ssRNA positive-strand viruses, no DNA stage; Tymovirales; Betaflexiviridae; Quinvirinae; Carlavirus; unclassified Carlavirus
GCA_001551585.1	8540	6	Elderberry carlavirus E	NA	Viruses; ssRNA viruses; ssRNA positive-strand viruses, no DNA stage; Tymovirales; Betaflexiviridae; Quinvirinae; Carlavirus; unclassified Carlavirus
GCA_004130965.1	7106	5	Ribes americanum virus A	NA	Viruses; Riboviria; Orthornavirae; Kitrinoviricota; Alsuviricetes; Tymovirales; Betaflexiviridae; Trivirinae; Ravavirus
GCA_001432115.1	11214	8	Walkabout Creek virus	NA	Viruses; ssRNA viruses; ssRNA negative-strand viruses; Mononegavirales; Rhabdoviridae; unclassified Rhabdoviridae
GCA_013087295.1	11284	9	Harrison Dam virus	NA	Viruses; Riboviria; Orthornavirae; Negarnaviricota; Haploviricotina; Monjiviricetes; Mononegavirales; Rhabdoviridae; Sunrhavirus; Harrison sunrhavirus
GCA_004114875.1	11677	4	Mukawa virus	NA	Viruses; Riboviria; Orthornavirae; Negarnaviricota; Polyploviricotina; Ellioviricetes; Bunyavirales; Phenuiviridae; Phlebovirus; Mukawa phlebovirus
GCA_000858245.1	9324	1	Scallion mosaic virus	NA	Viruses; ssRNA viruses; ssRNA positive-strand viruses, no DNA stage; Potyviridae; Potyvirus
GCA_900094155.1	18959	9	Ebola virus	NA	Viruses; Riboviria; Orthornavirae; Negarnaviricota; Haploviricotina; Monjiviricetes; Mononegavirales; Filoviridae; Ebolavirus; Zaire ebolavirus
GCA_000928935.1	2739	4	Switchgrass mosaicassociated virus 1	NA	Viruses; ssDNA viruses; Geminiviridae; Mastrevirus; unclassified Mastrevirus

gca	genome.size	proteome.size	phage.name	strain	Taxonomy
GCA_004066075.1	1608	2	Bat circovirus POA2012I	NA	Viruses; Monodnaviria; Shotokuvirae; Cressdnaviricota; Arfiviricetes; Cirlivirales; Circoviridae; Circovirus; unclassified Circovirus
GCA_000930515.1	1755	2	Bat circovirus POA2012II	NA	Viruses; ssDNA viruses; Circoviridae; Circovirus; unclassified Circovirus
GCA_004066055.1	1728	2	Bat circovirus POA2012V	NA	Viruses; Monodnaviria; Shotokuvirae; Cressdnaviricota; Arfiviricetes; Cirlivirales; Circoviridae; Circovirus; unclassified Circovirus
GCA_000929875.1	1790	2	Bat circovirus POA2012VI	NA	Viruses; ssDNA viruses; Circoviridae; Circovirus; unclassified Circovirus
GCA_000928835.1	7369	5	Black sea bass polyomavirus 1	NA	Viruses; dsDNA viruses, no RNA stage; Polyomaviridae; unclassified Polyomaviridae
GCA_000842865.1	31508	36	Haemophilus virus HP2	NA	Viruses; Duplodnaviria; Heunggongvirae; Uroviricota; Caudoviricetes; Caudovirales; Myoviridae; Peduovirinae; Hpunavirus
GCA_000899795.1	7659	3	Gooseberry vein banding associated virus	NA	Viruses; Riboviria; Pararnavirae; Artverviricota; Revtraviricetes; Ortervirales; Caulimoviridae; Badnavirus
GCA_000929055.1	50109	83	Shigella phage pSf2	NA	Viruses; dsDNA viruses, no RNA stage; Caudovirales; Siphoviridae; Tunavirinae; T1virus; Shigella virus PSf2
GCA_001504675.1	142348	213	Staphylococcus phage phiIPLARODI	NA	Viruses; dsDNA viruses, no RNA stage; Caudovirales; Myoviridae; unclassified Myoviridae
GCA_001501675.1	140961	203	Staphylococcus phage phiIPLAC1C	NA	Viruses; dsDNA viruses, no RNA stage; Caudovirales; Myoviridae; unclassified Myoviridae
GCA_001550705.1	141298	201	Geobacillus virus E3	NA	Viruses; dsDNA viruses, no RNA stage; Caudovirales; Siphoviridae; unclassified Siphoviridae
GCA_001500355.1	120098	171	Salmonella phage Shivani	NA	Viruses; dsDNA viruses, no RNA stage; Caudovirales; Siphoviridae; T5virus; Salmonella virus Shivani
GCA_002620065.1	87709	128	Salmonella phage Mushroom	NA	Viruses; Duplodnaviria; Heunggongvirae; Uroviricota; Caudoviricetes; Caudovirales; Myoviridae; Ounavirinae; Felixounavirus; Salmonella virus Mushroom
GCA_002756495.1	34565	51	Mannheimia phage vB_MhM_535AP1	NA	Viruses; Duplodnaviria; Heunggongvirae; Uroviricota; Caudoviricetes; Caudovirales; Myoviridae; Peduovirinae; Baylorvirus; Mannheimia virus PHL101
GCA_001503675.1	50078	79	Mannheimia phage vB_MhS_535AP2	NA	Viruses; dsDNA viruses, no RNA stage; Caudovirales; Siphoviridae; Lambdavirus; unclassified Lambda-like viruses
GCA_001504475.1	35764	51	Mannheimia phage vB_MhM_587AP1	NA	Viruses; dsDNA viruses, no RNA stage; Caudovirales; Myoviridae; Peduovirinae; P2virus; unclassified P2likevirus
GCA_001482975.1	48594	77	Mannheimia phage vB_MhS_587AP2	NA	Viruses; dsDNA viruses, no RNA stage; Caudovirales; Siphoviridae; Lambdavirus; unclassified Lambda-like viruses
GCA_002605285.1	36745	52	Mannheimia phage vB_MhM_1127AP1	NA	Viruses; Duplodnaviria; Heunggongvirae; Uroviricota; Caudoviricetes; Caudovirales; Myoviridae; Peduovirinae; Baylorvirus; Mannheimia virus 1127AP1
GCA_001505935.1	52139	79	Mannheimia phage vB_MhS_1152AP2	NA	Viruses; dsDNA viruses, no RNA stage; Caudovirales; Siphoviridae; Lambdavirus; unclassified Lambda-like viruses
GCA_002756515.1	34926	52	Mannheimia phage vB_MhM_2256AP1	NA	Viruses; Duplodnaviria; Heunggongvirae; Uroviricota; Caudoviricetes; Caudovirales; Myoviridae; Peduovirinae; Baylorvirus; Mannheimia virus PHL101
GCA_002605305.1	52049	83	Mannheimia phage vB_MhS_3927AP1	NA	Viruses; Duplodnaviria; Heunggongvirae; Uroviricota; Caudoviricetes; Caudovirales; Siphoviridae; unclassified Siphoviridae
GCA_001505275.1	33755	50	Mannheimia phage vB_MhM_3927AP2	NA	Viruses; dsDNA viruses, no RNA stage; Caudovirales; Myoviridae; Muvirus; unclassified Mu-like viruses
GCA_001503655.1	40829	45	Escherichia phage P483	NA	Viruses; dsDNA viruses, no RNA stage; Caudovirales; Podoviridae; unclassified Podoviridae
GCA_001504455.1	40477	50	Escherichia phage P694	NA	Viruses; dsDNA viruses, no RNA stage; Caudovirales; Podoviridae; unclassified Podoviridae
GCA_001550905.1	48666	79	Pseudomonas phage PS1	NA	Viruses; dsDNA viruses, no RNA stage; Caudovirales; Siphoviridae; unclassified Siphoviridae
GCA_001433605.1	4961	2	Rosellinia necatrix partitivirus 6	W113	Viruses; dsRNA viruses; Partitiviridae; Betapartitivirus; unclassified Betapartitivirus
GCA_004787235.1	11681	5	Lepeophtheirus salmonis rhabdovirus 9	NA	Viruses; Riboviria; Orthornavirae; Negarnaviricota; Haploviricotina; Monjiviricetes; Mononegavirales; Rhabdoviridae; Caligrhavirus; Salmonlouse caligrhavirus
GCA_004787255.1	11519	5	Lepeophtheirus salmonis rhabdovirus 127	NA	Viruses; Riboviria; Orthornavirae; Negarnaviricota; Haploviricotina; Monjiviricetes; Mononegavirales; Rhabdoviridae; Caligrhavirus; Lepeophtheirus caligrhavirus
GCA_000928055.1	8712	1	Fisavirus 1	HAL1	Viruses; ssRNA viruses; unclassified ssRNA viruses
GCA_002819985.1	1761	2	Dragonfly associated cyclovirus 6	NA	Viruses; Monodnaviria; Shotokuvirae; Cressdnaviricota; Arfiviricetes; Cirlivirales; Circoviridae; Cyclovirus
GCA_002820005.1	1757	2	Dragonfly associated cyclovirus 7	NA	Viruses; Monodnaviria; Shotokuvirae; Cressdnaviricota; Arfiviricetes; Cirlivirales; Circoviridae; Cyclovirus
GCA_002820025.1	1764	2	Dragonfly associated cyclovirus 8	NA	Viruses; Monodnaviria; Shotokuvirae; Cressdnaviricota; Arfiviricetes; Cirlivirales; Circoviridae; Cyclovirus
GCA_000929015.1	2948	3	Dromedary stoolassociated circular ssDNA virus	NA	Viruses; ssDNA viruses; Circoviridae; unclassified Circoviridae
GCA_006441475.1	35079	15	Rhesus adenovirus 51	NA	Viruses; Varidnaviria; Bamfordvirae; Preplasmiviricota; Tectiliviricetes; Rowavirales; Adenoviridae; Mastadenovirus; Human mastadenovirus G

gca	genome.size	proteome.size	phage.name	strain	Taxonomy
GCA_006441455.1	34391	15	Rhesus adenovirus 52	NA	Viruses; Varidnaviria; Bamfordvirae; Preplasmiviricota; Tectiliviricetes; Rowavirales; Adenoviridae; Mastadenovirus; Human mastadenovirus G
GCA_006441435.1	34475	15	Rhesus adenovirus 53	NA	Viruses; Varidnaviria; Bamfordvirae; Preplasmiviricota; Tectiliviricetes; Rowavirales; Adenoviridae; Mastadenovirus; Human mastadenovirus G
GCA_001432035.1	10795	4	Suffolk virus	NA	Viruses; ssRNA viruses; ssRNA negative-strand viruses; Mononegavirales; unassigned Mononegavirales
GCA_001184965.1	8041	6	Pea streak virus	NA	Viruses; ssRNA viruses; ssRNA positive-strand viruses, no DNA stage; Tymovirales; Betaflexiviridae; Quinvirinae; Carlavirus
GCA_000930615.1	32753	16	Dendrolimus punctatus cypovirus 22	NA	Viruses; dsRNA viruses; Reoviridae; Spinareovirinae; Cypovirus; unclassified Cypovirus
GCA_000929975.1	5820	6	Sauropus yellowing virus	NA	Viruses; ssRNA viruses; ssRNA positive-strand viruses, no DNA stage; Luteoviridae; Polerovirus; unclassified Polerovirus
GCA_000927975.1	5085	6	Bovine polyomavirus 2	BPyV2SF	Viruses; dsDNA viruses, no RNA stage; Polyomaviridae; unclassified Polyomaviridae
GCA_000862525.1	9834	2	Oat mosaic virus	NA	Viruses; ssRNA viruses; ssRNA positive-strand viruses, no DNA stage; Potyviridae; Bymovirus
GCA_003029205.1	2731	6	Euphorbia yellow leaf curl virus	NA	Viruses; Monodnaviria; Shotokuvirae; Cressdnaviricota; Repensiviricetes; Geplafuvirales; Geminiviridae; Begomovirus
GCA_000930675.1	7093	1	Tortoise picornavirus	1404	Viruses; ssRNA viruses; ssRNA positive-strand viruses, no DNA stage; Picornavirales; Picornaviridae; unclassified Picornaviridae
GCA_000845065.1	32308	45	Sulfolobus islandicus rodshaped virus 1	NA	Viruses; dsDNA viruses, no RNA stage; Ligamenvirales; Rudiviridae; Rudivirus
GCA_000857285.1	35450	54	Sulfolobus islandicus rodshaped virus 2	HVE104	Viruses; dsDNA viruses, no RNA stage; Ligamenvirales; Rudiviridae; Rudivirus
GCA_000843165.1	34692	56	Streptococcus phage SM1	NA	Viruses; dsDNA viruses, no RNA stage; Caudovirales; Siphoviridae; unclassified Siphoviridae
GCA_000930695.1	139981	130	Parapoxvirus red deerHL953	NA	Viruses; dsDNA viruses, no RNA stage; Poxviridae; Chordopoxvirinae; Parapoxvirus; unclassified Parapoxvirus
GCA_000929035.1	31735	29	Psittacine adenovirus 3	NA	Viruses; dsDNA viruses, no RNA stage; Adenoviridae; Atadenovirus; unclassified Atadenovirus
GCA_000928115.1	148981	150	Agrotis segetum nucleopolyhedrovirus B	NA	Viruses; dsDNA viruses, no RNA stage; Baculoviridae; Alphabaculovirus
GCA_001503855.1	50918	65	Sulfolobus monocaudavirus SMV2	NA	Viruses; dsDNA viruses, no RNA stage; unclassified dsDNA viruses
GCA_000930775.1	5076	2	Phomopsis vexans RNA virus	NA	Viruses; dsRNA viruses; Totiviridae; Victorivirus; unclassified Victorivirus
GCA_000928255.1	10290	1	Alternaria brassicicola endornavirus	NA	Viruses; dsRNA viruses; Endornaviridae; Endornavirus; unclassified Endornavirus
GCA_000930815.1	3246	2	Magnaporthe oryzae RNA virus	NA	Viruses; unclassified viruses
GCA_000930035.1	4844	2	Slow loris parvovirus 1	Buddha_08	Viruses; ssDNA viruses; Parvoviridae; unclassified Parvoviridae
GCA_000930055.1	7213	1	Cosavirus JMY2014	NA	Viruses; ssRNA viruses; ssRNA positive-strand viruses, no DNA stage; Picornavirales; Picornaviridae; Cosavirus; unclassified Cosavirus
GCA_003147205.1	44129	63	Clostridium phage phiCD241	NA	Viruses; Duplodnaviria; Heunggongvirae; Uroviricota; Caudoviricetes; Caudovirales; Siphoviridae; unclassified Siphoviridae
GCA_001504535.1	41560	55	Clostridium phage phiCD111	NA	Viruses; dsDNA viruses, no RNA stage; Caudovirales; Siphoviridae; unclassified Siphoviridae
GCA_001503755.1	41507	52	Clostridium phage phiCD146	NA	Viruses; dsDNA viruses, no RNA stage; Caudovirales; Siphoviridae; unclassified Siphoviridae
GCA_002277885.1	131704	190	Clostridium phage phiCD211	NA	Viruses; dsDNA viruses, no RNA stage; Caudovirales; Myoviridae; unclassified Myoviridae
GCA_001505375.1	32846	54	Clostridium phage phiCD4811	NA	Viruses; dsDNA viruses, no RNA stage; Caudovirales; Myoviridae; unclassified Myoviridae
GCA_001506015.1	49316	76	Clostridium phage phiCD505	NA	Viruses; dsDNA viruses, no RNA stage; Caudovirales; Myoviridae; unclassified Myoviridae
GCA_001504555.1	33274	53	Clostridium phage phiCD506	NA	Viruses; dsDNA viruses, no RNA stage; Caudovirales; Myoviridae; unclassified Myoviridae
GCA_001503775.1	44461	81	Clostridium phage phiMMP01	NA	Viruses; dsDNA viruses, no RNA stage; Caudovirales; Myoviridae; unclassified Myoviridae
GCA_001505395.1	52261	93	Clostridium phage phiMMP03	NA	Viruses; dsDNA viruses, no RNA stage; Caudovirales; Myoviridae; unclassified Myoviridae
GCA_002605365.1	52491	58	Streptococcus phage phi12073	NA	Viruses; Duplodnaviria; Heunggongvirae; Uroviricota; Caudoviricetes; Caudovirales; Siphoviridae; unclassified Siphoviridae
GCA_000865945.1	12640	4	Penicillium chrysogenum virus	NA	Viruses; dsRNA viruses; Chrysoviridae; Chrysovirus
GCA_000874065.1	5164	7	Tomato severe rugose virus	Petrolina de Goias	Viruses; ssDNA viruses; Geminiviridae; Begomovirus

gca	genome.size	proteome.size	phage.name	strain	Taxonomy
GCA_008799855.1	7556	3	Human calicivirus HuNLVGIIMD145121987US	NA	Viruses; Riboviria; Orthornavirae; Pisuviricota; Pisoniviricetes; Picornavirales; Caliciviridae; Norovirus; Norwalk virus; Norovirus GII; Norovirus GII isolates
GCA_001684605.1	13434	6	Diachasmimorpha longicaudata rhabdovirus	NA	Viruses; ssRNA viruses; ssRNA negative-strand viruses; Mononegavirales; Rhabdoviridae; unclassified Rhabdoviridae
GCA_006471845.1	103876	76	Tortoise ranavirus	NA	Viruses; Varidnaviria; Bamfordvirae; Nucleocytoviricota; Megaviricetes; Pimascovirales; Iridoviridae; Alphairidovirinae; Ranavirus; Common midwife toad virus
GCA_001503055.1	223932	237	Ralstonia phage RSL2	NA	Viruses; dsDNA viruses, no RNA stage; Caudovirales; Myoviridae; unclassified Myoviridae
GCA_002289195.1	4638	3	Arhar cryptic virusI	Hyderabad	Viruses; unclassified viruses
GCA_001430655.1	178262	248	Yellowstone lake phycodnavirus 1	NA	Viruses; dsDNA viruses, no RNA stage; Phycodnaviridae; unclassified Phycodnaviridae
GCA_001430455.1	171045	225	Yellowstone lake phycodnavirus 2	NA	Viruses; dsDNA viruses, no RNA stage; Phycodnaviridae; unclassified Phycodnaviridae
GCA_001430035.1	171454	236	Yellowstone lake phycodnavirus 3	NA	Viruses; dsDNA viruses, no RNA stage; Phycodnaviridae; unclassified Phycodnaviridae
GCA_000881595.1	119343	119	Diadromus pulchellus ascovirus 4a	NA	Viruses; dsDNA viruses, no RNA stage; Ascoviridae; Toursvirus
GCA_004114775.1	9613	3	Persea americana chrysovirus	NA	Viruses; Riboviria; Orthornavirae; Duplornaviricota; Chrymotiviricetes; Ghabrivirales; Chrysoviridae; Alphachrysovirus
GCA_000970565.1	10558	2	Black grass varicosaviruslike virus	NA	Viruses; unclassified viruses
GCA_000970585.1	3993	2	Black grass cryptic virus 2	NA	Viruses; unclassified viruses
GCA_001503795.1	39133	42	Enterobacter phage phiEap1	NA	Viruses; dsDNA viruses, no RNA stage; Caudovirales; Podoviridae; Autographivirinae; T7virus; unclassified T7-like viruses
GCA_001504655.1	30664	40	Streptococcus phage M102AD	NA	Viruses; dsDNA viruses, no RNA stage; Caudovirales; Siphoviridae; unclassified Siphoviridae
GCA_000929075.1	4882	5	Tadarida brasiliensis polyomavirus 1	NA	Viruses; dsDNA viruses, no RNA stage; Polyomaviridae; unclassified Polyomaviridae
GCA_000928175.1	4893	5	Tadarida brasiliensis polyomavirus 2	NA	Viruses; dsDNA viruses, no RNA stage; Polyomaviridae; unclassified Polyomaviridae
GCA_000930735.1	10483	4	Wenzhou mammarenavirus	NA	Viruses; ssRNA viruses; ssRNA negative-strand viruses; Arenaviridae; Mammarenavirus
GCA_003849445.1	2199	2	Gemycircularvirus BZ1	NA	Viruses; Monodnaviria; Shotokuvirae; Cressdnaviricota; Repensiviricetes; Geplafuvirales; Genomoviridae; Gemykibivirus; Human associated gemykibivirus 2
GCA_003849425.1	2199	2	Gemycircularvirus BZ2	NA	Viruses; Monodnaviria; Shotokuvirae; Cressdnaviricota; Repensiviricetes; Geplafuvirales; Genomoviridae; Gemykibivirus; Human associated gemykibivirus 2
GCA_003849405.1	2199	2	Gemycircularvirus NP	NA	Viruses; Monodnaviria; Shotokuvirae; Cressdnaviricota; Repensiviricetes; Geplafuvirales; Genomoviridae; Gemykibivirus; Human associated gemykibivirus 2
GCA_000973195.1	2199	2	Gemycircularvirus SL1	NA	Viruses; unclassified viruses; Genomoviridae; Gemycircularvirus; unclassified Gemycircularvirus
GCA_003849485.1	2199	2	Gemycircularvirus SL2	NA	Viruses; Monodnaviria; Shotokuvirae; Cressdnaviricota; Repensiviricetes; Geplafuvirales; Genomoviridae; Gemykibivirus; Human associated gemykibivirus 2
GCA_003849465.1	2199	2	Gemycircularvirus SL3	NA	Viruses; Monodnaviria; Shotokuvirae; Cressdnaviricota; Repensiviricetes; Geplafuvirales; Genomoviridae; Gemykibivirus; Human associated gemykibivirus 2
GCA_001505135.1	162101	262	Yersinia phage vB_YenM_TG1	NA	Viruses; dsDNA viruses, no RNA stage; Caudovirales; Myoviridae; Tg1virus; Yersinia virus TG1
GCA_001470775.1	36595	57	Streptococcus phage StrPAP1	NA	Viruses; dsDNA viruses, no RNA stage; Caudovirales; Podoviridae; unclassified Podoviridae; Escherichia phage phi191 sensu lato
GCA_001503515.1	45626	73	Pseudomonas phage PhiCHU	NA	Viruses; dsDNA viruses, no RNA stage; Caudovirales; Podoviridae; unclassified Podoviridae
GCA_001504315.1	52927	84	Mycobacterium phage Sheen	NA	Viruses; dsDNA viruses, no RNA stage; Caudovirales; Siphoviridae; unclassified Siphoviridae
GCA_001505775.1	66708	110	Vibrio phage phi 1	NA	Viruses; dsDNA viruses, no RNA stage; Caudovirales; Podoviridae; unclassified Podoviridae
GCA_001505115.1	116138	156	Vibrio phage phi 3	NA	Viruses; dsDNA viruses, no RNA stage; Caudovirales; Siphoviridae; T5virus; unclassified T5likevirus
GCA_001470935.1	188632	235	Cyanophage PTIM40	NA	Viruses; dsDNA viruses, no RNA stage; Caudovirales; Myoviridae; unclassified Myoviridae
GCA_001503495.1	49714	67	Achromobacter phage JWX	NA	Viruses; dsDNA viruses, no RNA stage; Caudovirales; Siphoviridae; unclassified Siphoviridae
GCA_001504295.1	48216	61	Achromobacter phage 8324	NA	Viruses; dsDNA viruses, no RNA stage; Caudovirales; Siphoviridae; unclassified Siphoviridae
GCA_001551405.1	81541	118	Achromobacter phage JWF	NA	Viruses; dsDNA viruses, no RNA stage; Caudovirales; Siphoviridae; unclassified Siphoviridae
GCA_001505755.1	35644	56	Paenibacillus phage HB10c2	NA	Viruses; dsDNA viruses, no RNA stage; Caudovirales; Siphoviridae; Sitaravirus; Paenibacillus virus Hb10c2

gca	genome.size	proteome.size	phage.name	strain	Taxonomy
GCA_001505095.1	37246	60	Paenibacillus phage Diva	NA	Viruses; dsDNA viruses, no RNA stage; Caudovirales; Siphoviridae; Sitaravirus; Paenibacillus virus Diva
GCA_001503475.1	44952	74	Bacteriophage Lily	NA	Viruses; dsDNA viruses, no RNA stage; Caudovirales; Siphoviridae; unclassified Siphoviridae
GCA_001551725.1	37990	61	Paenibacillus phage Rani	NA	Viruses; dsDNA viruses, no RNA stage; Caudovirales; Siphoviridae; Sitaravirus; Paenibacillus virus Rani
GCA_002605385.1	37971	61	Bacteriophage Redbud	NA	Viruses; Duplodnaviria; Heunggongvirae; Uroviricota; Caudoviricetes; Caudovirales; Siphoviridae; Sitaravirus; unclassified Sitaravirus
GCA_002617305.1	41152	68	Paenibacillus phage Shelly	NA	Viruses; Duplodnaviria; Heunggongvirae; Uroviricota; Caudoviricetes; Caudovirales; Siphoviridae; Sitaravirus; Paenibacillus virus Shelly
GCA_001504275.1	43724	74	Paenibacillus phage Sitara	NA	Viruses; dsDNA viruses, no RNA stage; Caudovirales; Siphoviridae; Sitaravirus; Paenibacillus virus Sitara
GCA_000930895.1	2776	4	Bromusassociated circular DNA virus 1	NA	Viruses; ssDNA viruses; unclassified ssDNA viruses
GCA_000930255.1	2542	4	Bromusassociated circular DNA virus 2	NA	Viruses; ssDNA viruses; unclassified ssDNA viruses
GCA_000931075.1	2238	2	Bromusassociated circular DNA virus 3	NA	Viruses; ssDNA viruses; unclassified ssDNA viruses
GCA_000929195.1	2806	3	Bromusassociated circular DNA virus 4	NA	Viruses; ssDNA viruses; unclassified ssDNA viruses
GCA_000931275.1	2751	3	Trifoliumassociated circular DNA virus 1	NA	Viruses; ssDNA viruses; unclassified ssDNA viruses
GCA_000930095.1	31249	10	Betacoronavirus HKU24	HKU24R05005I	Viruses; ssRNA viruses; ssRNA positive-strand viruses, no DNA stage; Nidovirales; Coronaviridae; Coronavirinae; Betacoronavirus; unclassified Betacoronavirus
GCA_003146805.1	115154	124	Escherichia phage RCS47	NA	Viruses; Duplodnaviria; Heunggongvirae; Uroviricota; Caudoviricetes; Caudovirales; Myoviridae; Punavirus; Escherichia virus RCS47
GCA_000930155.1	4988	5	Adelie penguin polyomavirus	NA	Viruses; dsDNA viruses, no RNA stage; Polyomaviridae; Avipolyomavirus group
GCA_018594675.1	12186	3	Buffalo Creek virus	NA	Viruses; Riboviria; Orthornavirae; Negarnaviricota; Polyploviricotina; Ellioviricetes; Bunyavirales; Peribunyaviridae; Orthobunyavirus; Buffalo Creek orthobunyavirus
GCA_018594665.1	12110	3	Mapputta virus	NA	Viruses; Riboviria; Orthornavirae; Negarnaviricota; Polyploviricotina; Ellioviricetes; Bunyavirales; Peribunyaviridae; Orthobunyavirus; Mapputta orthobunyavirus
GCA_000929375.1	12113	3	Maprik virus	NA	Viruses; ssRNA viruses; ssRNA negative-strand viruses; Bunyaviridae; unclassified Bunyaviridae
GCA_001505735.1	38392	60	Listeria phage vB_LmoS_188	NA	Viruses; dsDNA viruses, no RNA stage; Caudovirales; Siphoviridae; unclassified Siphoviridae
GCA_001505075.1	40759	72	Listeria phage vB_LmoS_293	NA	Viruses; dsDNA viruses, no RNA stage; Caudovirales; Siphoviridae; unclassified Siphoviridae
GCA_000851745.1	9518	8	Simian immunodeficiency virus SIVmnd 2	NA	Viruses; Retro-transcribing viruses; Retroviridae; Orthoretrovirinae; Lentivirus; Primate lentivirus group; Simian immunodeficiency virus; Simian immunodeficiency virus - mnd
GCA_009731815.1	12018	4	Yaba7 virus	Yaba 7	Viruses; Riboviria; Orthornavirae; Negarnaviricota; Polyploviricotina; Ellioviricetes; Bunyavirales; Peribunyaviridae; Orthobunyavirus; Akabane orthobunyavirus
GCA_004789315.1	12058	4	Sabo virus	NA	Viruses; Riboviria; Orthornavirae; Negarnaviricota; Polyploviricotina; Ellioviricetes; Bunyavirales; Peribunyaviridae; Orthobunyavirus; Sabo orthobunyavirus
GCA_006298165.1	12414	3	Manzanilla virus	NA	Viruses; Riboviria; Orthornavirae; Negarnaviricota; Polyploviricotina; Ellioviricetes; Bunyavirales; Peribunyaviridae; Orthobunyavirus; Manzanilla orthobunyavirus
GCA_004789715.1	12310	3	Buttonwillow virus	NA	Viruses; Riboviria; Orthornavirae; Negarnaviricota; Polyploviricotina; Ellioviricetes; Bunyavirales; Peribunyaviridae; Orthobunyavirus; Buttonwillow orthobunyavirus
GCA_000899155.1	12034	4	Sathuperi virus	NA	Viruses; ssRNA viruses; ssRNA negative-strand viruses; Bunyaviridae; Orthobunyavirus
GCA_009731835.1	12035	4	Douglas virus	NA	Viruses; Riboviria; Orthornavirae; Negarnaviricota; Polyploviricotina; Ellioviricetes; Bunyavirales; Peribunyaviridae; Orthobunyavirus; Schmallenberg orthobunyavirus
GCA_004789615.1	12289	3	Faceys Paddock virus	NA	Viruses; Riboviria; Orthornavirae; Negarnaviricota; Polyploviricotina; Ellioviricetes; Bunyavirales; Peribunyaviridae; Orthobunyavirus; Faceys paddock orthobunyavirus
GCA_004789675.1	12253	3	Utinga virus	NA	Viruses; Riboviria; Orthornavirae; Negarnaviricota; Polyploviricotina; Ellioviricetes; Bunyavirales; Peribunyaviridae; Orthobunyavirus; Utinga orthobunyavirus
GCA_000930295.1	9816	1	Thaumetopoea pityocampa iflavirus 1	NA	Viruses; ssRNA viruses; ssRNA positive-strand viruses, no DNA stage; Picornavirales; Iflaviridae; unclassified Iflaviridae
GCA_004789635.1	12388	3	Ingwavuma virus	NA	Viruses; Riboviria; Orthornavirae; Negarnaviricota; Polyploviricotina; Ellioviricetes; Bunyavirales; Peribunyaviridae; Orthobunyavirus; Ingwavuma orthobunyavirus
GCA_004789655.1	12332	3	Mermet virus	NA	Viruses; Riboviria; Orthornavirae; Negarnaviricota; Polyploviricotina; Ellioviricetes; Bunyavirales; Peribunyaviridae; Orthobunyavirus; Mermet orthobunyavirus
GCA_006298405.1	12112	4	Shuni orthobunyavirus	SAE1809	Viruses; Riboviria; Orthornavirae; Negarnaviricota; Polyploviricotina; Ellioviricetes; Bunyavirales; Peribunyaviridae; Orthobunyavirus
GCA_009731855.1	12070	4	Kaikalur virus	VRC 7134232	Viruses; Riboviria; Orthornavirae; Negarnaviricota; Polyploviricotina; Ellioviricetes; Bunyavirales; Peribunyaviridae; Orthobunyavirus; Shuni orthobunyavirus
GCA_000900015.1	12104	4	Shamonda virus	NA	Viruses; ssRNA viruses; ssRNA negative-strand viruses; Bunyaviridae; Orthobunyavirus

gca	genome.size	proteome.size	phage.name	strain	Taxonomy
GCA_004789295.1	12004	4	Peaton virus	NA	Viruses; Riboviria; Orthornavirae; Negarnaviricota; Polyploviricotina; Ellioviricetes; Bunyavirales; Peribunyaviridae; Orthobunyavirus; Peaton orthobunyavirus
GCA_004789335.1	11980	4	Sango virus	NA	Viruses; Riboviria; Orthornavirae; Negarnaviricota; Polyploviricotina; Ellioviricetes; Bunyavirales; Peribunyaviridae; Orthobunyavirus; Sango orthobunyavirus
GCA_000842825.1	5380	4	Planococcus citri densovirus	NA	Viruses; ssDNA viruses; Parvoviridae; Densovirinae; Ambidensovirus; Hemipteran ambidensovirus 1
GCA_011559005.1	186627	140	Lymphocystis disease virus 2	LCDVJP_Oita_2018	Viruses; Varidnaviria; Bamfordvirae; Nucleocytoviricota; Megaviricetes; Pimascovirales; Iridoviridae; Alphairidovirinae; Lymphocystivirus
GCA_000928495.1	2234	2	Sewageassociated gemycircularvirus 1	NA	Viruses; unclassified viruses; Genomoviridae; Gemycircularvirus; unclassified Gemycircularvirus
GCA_000931055.1	1605	2	Sewageassociated circular DNA virus15	NA	Viruses; ssDNA viruses; unclassified ssDNA viruses
GCA_000931255.1	2042	2	Sewageassociated circular DNA virus16	NA	Viruses; ssDNA viruses; unclassified ssDNA viruses
GCA_000929355.1	2077	2	Sewageassociated circular DNA virus17	NA	Viruses; ssDNA viruses; unclassified ssDNA viruses
GCA_000928475.1	2290	2	Sewageassociated circular DNA virus18	NA	Viruses; ssDNA viruses; unclassified ssDNA viruses
GCA_000931035.1	2478	2	Sewageassociated circular DNA virus19	NA	Viruses; ssDNA viruses; unclassified ssDNA viruses
GCA_000931235.1	2089	2	Sewageassociated circular DNA virus20	NA	Viruses; ssDNA viruses; unclassified ssDNA viruses
GCA_000929335.1	2906	2	Sewageassociated circular DNA virus21	NA	Viruses; ssDNA viruses; unclassified ssDNA viruses
GCA_000928455.1	2861	2	Sewageassociated circular DNA virus22	NA	Viruses; ssDNA viruses; unclassified ssDNA viruses
GCA_000931015.1	2309	2	Sewageassociated circular DNA virus23	NA	Viruses; ssDNA viruses; unclassified ssDNA viruses
GCA_000931215.1	2605	2	Sewageassociated circular DNA virus24	NA	Viruses; ssDNA viruses; unclassified ssDNA viruses
GCA_000929315.1	3588	3	Sewageassociated circular DNA virus25	NA	Viruses; ssDNA viruses; unclassified ssDNA viruses
GCA_000928435.1	3348	2	Sewageassociated circular DNA virus26	NA	Viruses; ssDNA viruses; unclassified ssDNA viruses
GCA_000930995.1	2638	2	Sewageassociated circular DNA virus27	NA	Viruses; ssDNA viruses; unclassified ssDNA viruses
GCA_000931195.1	2496	2	Sewageassociated circular DNA virus28	NA	Viruses; ssDNA viruses; unclassified ssDNA viruses
GCA_000929295.1	4037	3	Sewageassociated circular DNA virus29	NA	Viruses; ssDNA viruses; unclassified ssDNA viruses
GCA_000928415.1	2741	2	Sewageassociated circular DNA virus30	NA	Viruses; ssDNA viruses; unclassified ssDNA viruses
GCA_000930975.1	4202	3	Sewageassociated circular DNA virus31	NA	Viruses; ssDNA viruses; unclassified ssDNA viruses
GCA_000931175.1	2711	2	Sewageassociated circular DNA virus32	NA	Viruses; ssDNA viruses; unclassified ssDNA viruses
GCA_000929275.1	2846	2	Sewageassociated circular DNA virus33	NA	Viruses; ssDNA viruses; unclassified ssDNA viruses
GCA_000928395.1	2220	2	Sewageassociated circular DNA virus34	NA	Viruses; ssDNA viruses; unclassified ssDNA viruses
GCA_000930955.1	2472	2	Sewageassociated circular DNA virus35	NA	Viruses; ssDNA viruses; unclassified ssDNA viruses
GCA_000930315.1	2254	2	Sewageassociated circular DNA virus36	NA	Viruses; ssDNA viruses; unclassified ssDNA viruses
GCA_000929255.1	2155	2	Sewageassociated circular DNA virus37	NA	Viruses; ssDNA viruses; unclassified ssDNA viruses
GCA_004128935.1	1989	2	Odonataassociated circular virus1	NA	Viruses; unclassified viruses; unclassified DNA viruses; unclassified ssDNA viruses
GCA_004129195.1	1914	2	Odonataassociated circular virus10	NA	Viruses; unclassified viruses; unclassified DNA viruses; unclassified ssDNA viruses
GCA_004128955.1	1712	2	Odonataassociated circular virus11	NA	Viruses; unclassified viruses; unclassified DNA viruses; unclassified ssDNA viruses
GCA_004128975.1	1991	2	Odonataassociated circular virus13	NA	Viruses; unclassified viruses; unclassified DNA viruses; unclassified ssDNA viruses
GCA_004128995.1	1760	2	Odonataassociated circular virus14	NA	Viruses; unclassified viruses; unclassified DNA viruses; unclassified ssDNA viruses
GCA_004129015.1	2203	2	Odonataassociated circular virus15	NA	Viruses; unclassified viruses; unclassified DNA viruses; unclassified ssDNA viruses

gca	genome.size	proteome.size	phage.name	strain	Taxonomy
GCA_004129175.1	2016	2	Odonataassociated circular virus16	NA	Viruses; unclassified viruses; unclassified DNA viruses; unclassified ssDNA viruses
GCA_004129055.1	2029	2	Odonataassociated circular virus17	NA	Viruses; unclassified viruses; unclassified DNA viruses; unclassified ssDNA viruses
GCA_004129075.1	1913	2	Odonataassociated circular virus18	NA	Viruses; unclassified viruses; unclassified DNA viruses; unclassified ssDNA viruses
GCA_004129115.1	1890	2	Odonataassociated circular virus19	NA	Viruses; unclassified viruses; unclassified DNA viruses; unclassified ssDNA viruses
GCA_004129035.1	1740	2	Odonataassociated circular virus2	NA	Viruses; unclassified viruses; unclassified DNA viruses; unclassified ssDNA viruses
GCA_003033395.1	2609	2	Odonataassociated circular virus 21	NA	Viruses; Monodnaviria; Shotokuvirae; Cressdnaviricota; Arfiviricetes; Cremevirales; Smacoviridae; Bovismacovirus; Dragonfly associated bovismacovirus 1
GCA_004129135.1	1639	2	Odonataassociated circular virus3	NA	Viruses; unclassified viruses; unclassified DNA viruses; unclassified ssDNA viruses
GCA_004129155.1	2046	2	Odonataassociated circular virus4	NA	Viruses; unclassified viruses; unclassified DNA viruses; unclassified ssDNA viruses
GCA_003033405.1	2609	2	Odonataassociated circular virus 5	NA	Viruses; Monodnaviria; Shotokuvirae; Cressdnaviricota; Arfiviricetes; Cremevirales; Smacoviridae; Dragsmacovirus; Dragonfly associated dragsmacovirus 1
GCA_004128875.1	2942	3	Odonataassociated circular virus7	NA	Viruses; unclassified viruses; unclassified DNA viruses; unclassified ssDNA viruses
GCA_004128895.1	2445	2	Odonataassociated circular virus8	NA	Viruses; unclassified viruses; unclassified DNA viruses; unclassified ssDNA viruses
GCA_004128915.1	1931	2	Odonataassociated circular virus9	NA	Viruses; unclassified viruses; unclassified DNA viruses; unclassified ssDNA viruses
GCA_000930235.1	1040	1	Sewageassociated circular DNA molecule	NA	Viruses; Satellites; Satellite Nucleic Acids; unclassified Satellite Nucleic Acids
GCA_002149465.1	967	1	Sewageassociated circular DNA molecule1	NA	Viruses; Satellites; Satellite Nucleic Acids; unclassified Satellite Nucleic Acids
GCA_000930175.1	1132	1	Sewageassociated circular DNA molecule3	NA	Viruses; Satellites; Satellite Nucleic Acids; unclassified Satellite Nucleic Acids
GCA_000928515.1	139132	141	Pseudoplusia includens SNPV IE	NA	Viruses; dsDNA viruses, no RNA stage; Baculoviridae; Alphabaculovirus; Chrysodeixis includens nucleopolyhedrovirus
GCA_000838185.1	7610	6	Planaria asexual strainspecific viruslike element type 1	NA	Viruses; unclassified viruses
GCA_000931335.1	125767	138	Condylorrhiza vestigialis MNPV	NA	Viruses; dsDNA viruses, no RNA stage; Baculoviridae; Alphabaculovirus; unclassified Alphabaculovirus
GCA_001184985.1	1789	2	Cyclovirus TsCyV1_JPNUBS2014	NA	Viruses; ssDNA viruses; Circoviridae; unclassified Circoviridae; suggested genus Cyclovirus
GCA_000989075.1	4001	2	Rhizoctonia fumigata mycovirus	NA	Viruses; unclassified viruses
GCA_000988995.1	8531	6	Yam latent virus	NA	Viruses; ssRNA viruses; ssRNA positive-strand viruses, no DNA stage; Tymovirales; Betaflexiviridae; Quinvirinae; Carlavirus; unclassified Carlavirus
GCA_002149805.1	1169	1	Sewageassociated circular DNA molecule2	NA	Viruses; Satellites; Satellite Nucleic Acids; unclassified Satellite Nucleic Acids
GCA_000929235.1	2460	2	Human smacovirus 1	NA	Viruses; unclassified viruses; Smacovirusgroup
GCA_001503455.1	40000	50	Bacillus phage Palmer	NA	Viruses; dsDNA viruses, no RNA stage; Caudovirales; Podoviridae; Pagevirus; Bacillus virus Palmer
GCA_002605405.1	38731	54	Ralstonia phage phiITL1	NA	Viruses; Duplodnaviria; Heunggongvirae; Uroviricota; Caudoviricetes; Caudovirales; Autographiviridae; Serkorvirus; Ralstonia virus ITL1
GCA_002605425.1	66381	94	Pseudomonas phage phiKT28	NA	Viruses; Duplodnaviria; Heunggongvirae; Uroviricota; Caudoviricetes; Caudovirales; Myoviridae; Pbunavirus; unclassified Pbunavirus
GCA_002605445.1	65994	91	Pseudomonas phage phiKTN6	NA	Viruses; Duplodnaviria; Heunggongvirae; Uroviricota; Caudoviricetes; Caudovirales; Myoviridae; Pbunavirus; Pseudomonas virus KTN6
GCA_001504255.1	147589	192	Enterococcus phage EFDG1	NA	Viruses; dsDNA viruses, no RNA stage; Caudovirales; Myoviridae; unclassified Myoviridae
GCA_001505715.1	77266	130	Escherichia phage 1721	NA	Viruses; dsDNA viruses, no RNA stage; Caudovirales; Podoviridae; unclassified Podoviridae
GCA_001020015.1	4083	4	Cymbidium chlorotic mosaic virus	NA	Viruses; ssRNA viruses; ssRNA positive-strand viruses, no DNA stage; Sobemovirus; unclassified Sobemovirus
GCA_000928355.1	2661	6	Oxalis yellow vein virus	USALA01	Viruses; ssDNA viruses; Geminiviridae; Begomovirus; unclassified Begomovirus
GCA_002925585.1	13262	13	Pseudomonas phage phiNN	NA	Viruses; Riboviria; Orthornavirae; Duplornaviricota; Vidaverviricetes; Mindivirales; Cystoviridae; Cystovirus; Pseudomonas virus phiNN
GCA_003963855.1	2529	2	Gorilla smacovirus	NA	Viruses; unclassified viruses; Smacovirusgroup
GCA_000889995.1	9745	1	Pepper yellow mosaic virus	DF	Viruses; ssRNA viruses; ssRNA positive-strand viruses, no DNA stage; Potyviridae; Potyvirus

gca	genome.size	proteome.size	phage.name	strain	Taxonomy
GCA_014883555.1	7278	1	Rabovirus A	RuianRn932	Viruses; ssRNA viruses; ssRNA positive-strand viruses, no DNA stage; Picornavirales; Picornaviridae; unclassified Picornaviridae
GCA_000931095.1	7687	1	Lesavirus 1	NA	Viruses; ssRNA viruses; ssRNA positive-strand viruses, no DNA stage; Picornavirales; Picornaviridae; unclassified Picornaviridae
GCA_000931295.1	7593	1	Lesavirus 2	NA	Viruses; ssRNA viruses; ssRNA positive-strand viruses, no DNA stage; Picornavirales; Picornaviridae; unclassified Picornaviridae
GCA_006441615.1	35124	40	Human adenovirus 69	NA	Viruses; Varidnaviria; Bamfordvirae; Preplasmiviricota; Tectiliviricetes; Rowavirales; Adenoviridae; Mastadenovirus; Human mastadenovirus D
GCA_000897795.1	77315	120	Escherichia phage ECBP2	NA	Viruses; dsDNA viruses, no RNA stage; Caudovirales; Podoviridae; unclassified Podoviridae
GCA_000900235.1	69855	82	Escherichia phage ECBP1	NA	Viruses; dsDNA viruses, no RNA stage; Caudovirales; Podoviridae; Picovirinae; G7cvirus; Escherichia virus ECBP1
GCA_008776215.1	7054	1	Human rhinovirus C54	NA	Viruses; Riboviria; Orthornavirae; Pisuviricota; Pisoniviricetes; Picornavirales; Picornaviridae; Enterovirus; Rhinovirus C
GCA_000851345.1	3392	2	Discula destructiva virus 2	NA	Viruses; dsRNA viruses; Partitiviridae; Gammapartitivirus
GCA_001618995.1	7382	1	Yak enterovirus	SWUNAB001	Viruses; ssRNA viruses; ssRNA positive-strand viruses, no DNA stage; Picornavirales; Picornaviridae; Enterovirus; unclassified Enterovirus
GCA_000885375.1	5168	5	Bornean orangutan polyomavirus	NA	Viruses; dsDNA viruses, no RNA stage; Polyomaviridae; unclassified Polyomaviridae
GCA_001430235.1	5358	5	Sumatran orangutan polyomavirus	NA	Viruses; dsDNA viruses, no RNA stage; Polyomaviridae; unclassified Polyomaviridae
GCA_003004805.1	38465	69	ANMV1 virus	NA	Viruses; unclassified archaeal viruses
GCA_001755565.1	104906	165	Acinetobacter phage vB_AbaM_phiAbaA1	NA	Viruses; dsDNA viruses, no RNA stage; Caudovirales; Myoviridae; unclassified Myoviridae
GCA_002820765.1	9531	1	Nonprimate hepacivirus NZP1	NA	Viruses; Riboviria; Orthornavirae; Kitrinoviricota; Flasuviricetes; Amarillovirales; Flaviviridae; Hepacivirus; Hepacivirus A
GCA_000892215.1	124335	82	Cricetid gammaherpesvirus 2	NA	Viruses; dsDNA viruses, no RNA stage; Herpesvirales; Herpesviridae; Gammaherpesvirinae; Rhadinovirus
GCA_002827825.1	5187	5	Artibeus planirostris polyomavirus 3	NA	Viruses; Monodnaviria; Shotokuvirae; Cossaviricota; Papovaviricetes; Sepolyvirales; Polyomaviridae; Alphapolyomavirus
GCA_002827865.1	5058	4	Sturnira lilium polyomavirus 1	NA	Viruses; Monodnaviria; Shotokuvirae; Cossaviricota; Papovaviricetes; Sepolyvirales; Polyomaviridae; Alphapolyomavirus
GCA_001430435.1	5352	4	Carollia perspicillata polyomavirus 1	NA	Viruses; dsDNA viruses, no RNA stage; Polyomaviridae; Alphapolyomavirus
GCA_001430215.1	4903	4	Molossus molossus polyomavirus 1	NA	Viruses; dsDNA viruses, no RNA stage; Polyomaviridae; Alphapolyomavirus
GCA_002605465.1	41903	62	Mycobacterium phage Gomashi	NA	Viruses; Duplodnaviria; Heunggongvirae; Uroviricota; Caudoviricetes; Caudovirales; Siphoviridae; Liefievirus; unclassified Liefievirus
GCA_001505055.1	62738	99	Mycobacterium phage Kratio	NA	Viruses; dsDNA viruses, no RNA stage; Caudovirales; Siphoviridae; unclassified Siphoviridae
GCA_002080315.1	7253	1	Bovine rhinovirus 1	SD1	Viruses; ssRNA viruses; ssRNA positive-strand viruses, no DNA stage; Picornavirales; Picornaviridae; Aphthovirus; unclassified Aphthovirus
GCA_006458085.1	105057	105	Tiger frog virus	NA	Viruses; Varidnaviria; Bamfordvirae; Nucleocytoviricota; Megaviricetes; Pimascovirales; Iridoviridae; Alphairidovirinae; Ranavirus; Frog virus 3
GCA_003191565.1	10279	9	Simian immunodeficiency virus 17ECl	NA	Viruses; Riboviria; Pararnavirae; Artverviricota; Revtraviricetes; Ortervirales; Retroviridae; Orthoretrovirinae; Lentivirus; Simian immunodeficiency virus; Simian immunodeficiency virus - mac
GCA_001745695.1	222832	211	Skunkpox virus	WA	Viruses; dsDNA viruses, no RNA stage; Poxviridae; Chordopoxvirinae; Orthopoxvirus
GCA_001746095.1	11843	5	Bole Tick Virus 2	BL076	Viruses; ssRNA viruses; ssRNA negative-strand viruses; Mononegavirales; Rhabdoviridae; unclassified Rhabdoviridae
GCA_001441115.1	11183	3	Bole Tick Virus 3	BL199	Viruses; ssRNA viruses; ssRNA negative-strand viruses; unclassified ssRNA negative-strand viruses
GCA_001440855.1	10877	3	Changping Tick Virus 2	CP14	Viruses; ssRNA viruses; ssRNA negative-strand viruses; unclassified ssRNA negative-strand viruses
GCA_001440935.1	9527	2	Changping Tick Virus 3	CP13	Viruses; ssRNA viruses; ssRNA negative-strand viruses; unclassified ssRNA negative-strand viruses
GCA_013086875.1	8338	3	Dabieshan Tick Virus	D3	Viruses; Riboviria; Orthornavirae; Negarnaviricota; Polyploviricotina; Ellioviricetes; Bunyavirales; Phenuiviridae; Phlebovirus; unclassified Phlebovirus
GCA_001744775.1	18467	3	Huangpi Tick Virus 1	H1241	Viruses; ssRNA viruses; ssRNA negative-strand viruses; Bunyaviridae; unclassified Bunyaviridae
GCA_001744095.1	11718	4	Huangpi Tick Virus 2	H11417	Viruses; ssRNA viruses; ssRNA negative-strand viruses; Bunyaviridae; Phlebovirus; unclassified Phlebovirus
GCA_001745415.1	13169	5	Huangpi Tick Virus 3	H1242	Viruses; ssRNA viruses; ssRNA negative-strand viruses; Mononegavirales; Rhabdoviridae; unclassified Rhabdoviridae
GCA_013086885.1	8279	2	Lihan tick virus	LH1	Viruses; Riboviria; Orthornavirae; Negarnaviricota; Polyploviricotina; Ellioviricetes; Bunyavirales; Phenuiviridae; Uukuvirus; Lihan uukuvirus

gca	genome.size	proteome.size	phage.name	strain	Taxonomy
GCA_003673665.1	11477	3	Lishi spider virus 1	LSZZ11	Viruses; Riboviria; Orthornavirae; Negarnaviricota; Haploviricotina; Monjiviricetes; Jingchuvirales; Chuviridae; Mivirus; Lishi mivirus
GCA_001755725.1	9924	3	Lishi Spider Virus 2	LSZZ4	Viruses; Riboviria; Orthornavirae; Negarnaviricota; Haploviricotina; Monjiviricetes; Mononegavirales; Lispiviridae; Arlivirus; Lishi arlivirus
GCA_001746075.1	18593	3	Sanxia Water Strider Virus 1	SXSSP08	Viruses; ssRNA viruses; ssRNA negative-strand viruses; Bunyaviridae; unclassified Bunyaviridae
GCA_004789795.1	12992	3	Sanxia Water Strider Virus 2	SXSSP02	Viruses; Riboviria; Orthornavirae; Negarnaviricota; Polyploviricotina; Ellioviricetes; Bunyavirales; Phasmaviridae; Sawastrivirus; Sanxia sawastrivirus
GCA_001744755.1	12930	4	Sanxia water strider virus 4	SXSSP12	Viruses; Riboviria; Orthornavirae; Negarnaviricota; Haploviricotina; Monjiviricetes; Mononegavirales; Lispiviridae; Arlivirus; Gerrid arlivirus
GCA_001744075.1	12978	5	Sanxia Water Strider Virus 5	SXSSP11	Viruses; ssRNA viruses; ssRNA negative-strand viruses; Mononegavirales; Rhabdoviridae; unclassified Rhabdoviridae
GCA_001755425.1	13732	3	Shayang Fly Virus 1	SYY31	Viruses; ssRNA viruses; ssRNA negative-strand viruses; unclassified ssRNA negative-strand viruses
GCA_001754565.1	12291	6	Shayang Fly Virus 2	SYY18	Viruses; ssRNA viruses; ssRNA negative-strand viruses; Mononegavirales; Rhabdoviridae; unclassified Rhabdoviridae
GCA_001754125.1	15462	5	Shayang Fly Virus 3	SYY11	Viruses; ssRNA viruses; ssRNA negative-strand viruses; Mononegavirales; Rhabdoviridae; unclassified Rhabdoviridae
GCA_001754985.1	20231	3	Shayang Spider Virus 1	SYZZ4	Viruses; ssRNA viruses; ssRNA negative-strand viruses; Bunyaviridae; unclassified Bunyaviridae
GCA_001755405.1	10925	3	Shuangao Bedbug Virus 2	SACC1	Viruses; ssRNA viruses; ssRNA negative-strand viruses; unclassified ssRNA negative-strand viruses
GCA_003673765.1	6275	1	Shuangao Fly Virus 2	QSA05	Viruses; Riboviria; Orthornavirae; Negarnaviricota; Haploviricotina; Monjiviricetes; Mononegavirales; Xinmoviridae; Anphevirus; Shuangao anphevirus
GCA_001754545.1	13309	3	Shuangao Insect Virus 1	QSA02	Viruses; ssRNA viruses; ssRNA negative-strand viruses; Bunyaviridae; unclassified Bunyaviridae
GCA_002814495.1	10261	4	Shuangao Insect Virus 2	QSA03	Viruses; Riboviria; Orthornavirae; Negarnaviricota; Polyploviricotina; Ellioviricetes; Bunyavirales; Phasmaviridae; Inshuvirus; Insect inshuvirus
GCA_003673585.1	6714	1	Shuangao lacewing virus	QSA01	Viruses; Riboviria; Orthornavirae; Negarnaviricota; Haploviricotina; Monjiviricetes; Jingchuvirales; Chuviridae; Mivirus; Lacewing mivirus
GCA_001755105.1	19171	3	Tacheng Tick Virus 1	TC253	Viruses; ssRNA viruses; ssRNA negative-strand viruses; Bunyaviridae; unclassified Bunyaviridae
GCA_013086895.1	8844	2	Tacheng Tick Virus 2	TC252	Viruses; Riboviria; Orthornavirae; Negarnaviricota; Polyploviricotina; Ellioviricetes; Bunyavirales; Phenuiviridae; Uukuvirus; Tacheng uukuvirus
GCA_001755525.1	8683	2	Tacheng Tick Virus 3	TC255	Viruses; ssRNA viruses; ssRNA negative-strand viruses; Mononegavirales; Rhabdoviridae; unclassified Rhabdoviridae
GCA_001441015.1	10815	3	Tacheng Tick Virus 4	TCRP1	Viruses; ssRNA viruses; ssRNA negative-strand viruses; unclassified ssRNA negative-strand viruses
GCA_001441095.1	10017	2	Tacheng Tick Virus 5	TC254	Viruses; ssRNA viruses; ssRNA negative-strand viruses; unclassified ssRNA negative-strand viruses
GCA_001754665.1	10787	4	Tacheng Tick Virus 6	TCRP2	Viruses; ssRNA viruses; ssRNA negative-strand viruses; unclassified ssRNA negative-strand viruses
GCA_001754225.1	13408	6	Tacheng Tick Virus 7	TCRP3	Viruses; ssRNA viruses; ssRNA negative-strand viruses; Mononegavirales; Rhabdoviridae; unclassified Rhabdoviridae
GCA_001755085.1	11280	3	Taishun Tick Virus	BL198	Viruses; ssRNA viruses; ssRNA negative-strand viruses; Mononegavirales; Rhabdoviridae; unclassified Rhabdoviridae
GCA_001755505.1	9591	3	Wenzhou Crab Virus 1	RBX2	Viruses; ssRNA viruses; ssRNA negative-strand viruses; Mononegavirales; unassigned Mononegavirales; Crustavirus
GCA_003673645.1	11657	3	Wenzhou crab virus 2	ZCX13	Viruses; Riboviria; Orthornavirae; Negarnaviricota; Haploviricotina; Monjiviricetes; Jingchuvirales; Chuviridae; Mivirus; Crab mivirus
GCA_001754645.1	12860	4	Wenzhou Crab Virus 3	RBX9	Viruses; ssRNA viruses; ssRNA negative-strand viruses; unclassified ssRNA negative-strand viruses
GCA_001754205.1	18291	3	Wenzhou Tick Virus	TS12	Viruses; ssRNA viruses; ssRNA negative-strand viruses; Bunyaviridae; unclassified Bunyaviridae
GCA_001755065.1	10151	3	Wenzhou Shrimp Virus 1	BJDX5	Viruses; ssRNA viruses; ssRNA negative-strand viruses; Bunyaviridae; unclassified Bunyaviridae
GCA_001755485.1	10937	3	Wuchang Cockroach Virus 1	WCZL5	Viruses; ssRNA viruses; ssRNA negative-strand viruses; unclassified ssRNA negative-strand viruses
GCA_003673625.1	11940	3	Wuchang Cockroach Virus 3	WCZL1	Viruses; Riboviria; Orthornavirae; Negarnaviricota; Haploviricotina; Monjiviricetes; Jingchuvirales; Chuviridae; Mivirus; Cockroach mivirus
GCA_001754625.1	7202	1	Wuhan Ant Virus	WHMY02	Viruses; ssRNA viruses; ssRNA negative-strand viruses; Mononegavirales; Rhabdoviridae; unclassified Rhabdoviridae
GCA_001754185.1	12349	3	Wuhan Fly Virus 1	SYY19	Viruses; ssRNA viruses; ssRNA negative-strand viruses; Bunyaviridae; unclassified Bunyaviridae
GCA_001755045.1	12247	6	Wuhan Fly Virus 2	SYY13	Viruses; ssRNA viruses; ssRNA negative-strand viruses; Mononegavirales; Rhabdoviridae; unclassified Rhabdoviridae
GCA_001755465.1	12651	6	Wuhan House Fly Virus 1	SYY24	Viruses; ssRNA viruses; ssRNA negative-strand viruses; Mononegavirales; Rhabdoviridae; unclassified Rhabdoviridae

gca	genome.size	proteome.size	phage.name	strain	Taxonomy
GCA_001754605.1	14731	5	Wuhan House Fly Virus 2	SYY45	Viruses; ssRNA viruses; ssRNA negative-strand viruses; Mononegavirales; Rhabdoviridae; unclassified Rhabdoviridae
GCA_001706905.1	15458	3	Wuhan Insect virus 2	WHDL02	Viruses; ssRNA viruses; ssRNA negative-strand viruses; unclassified ssRNA negative-strand viruses
GCA_001755445.1	13490	6	Wuhan Insect virus 4	YCYC03	Viruses; ssRNA viruses; ssRNA negative-strand viruses; Mononegavirales; Rhabdoviridae; Cytorhabdovirus; unclassified Cytorhabdovirus
GCA_001755325.1	12734	6	Wuhan Insect virus 5	YCYC02	Viruses; ssRNA viruses; ssRNA negative-strand viruses; Mononegavirales; Rhabdoviridae; Cytorhabdovirus; unclassified Cytorhabdovirus
GCA_001755745.1	14191	6	Wuhan Insect virus 6	SXCC011	Viruses; ssRNA viruses; ssRNA negative-strand viruses; Mononegavirales; Rhabdoviridae; Cytorhabdovirus; unclassified Cytorhabdovirus
GCA_001754885.1	11165	5	Wuhan Insect virus 7	WHYC02	Viruses; ssRNA viruses; ssRNA negative-strand viruses; Mononegavirales; Rhabdoviridae; unclassified Rhabdoviridae
GCA_001754445.1	11541	5	Wuhan Louse Fly Virus 10	BFJSC8	Viruses; ssRNA viruses; ssRNA negative-strand viruses; Mononegavirales; Rhabdoviridae; unclassified Rhabdoviridae
GCA_001754465.1	11103	5	Wuhan Louse Fly Virus 5	BFJSC5	Viruses; ssRNA viruses; ssRNA negative-strand viruses; Mononegavirales; Rhabdoviridae; unclassified Rhabdoviridae
GCA_003673705.1	9068	2	Wuhan louse fly virus 6	BFJSC2	Viruses; Riboviria; Orthornavirae; Negarnaviricota; Haploviricotina; Monjiviricetes; Jingchuvirales; Chuviridae; Mivirus; Louse fly mivirus
GCA_003673605.1	9151	2	Wuhan Louse Fly Virus 7	BFJSC3	Viruses; Riboviria; Orthornavirae; Negarnaviricota; Haploviricotina; Monjiviricetes; Jingchuvirales; Chuviridae; Mivirus; Hippoboscid mivirus
GCA_001755785.1	11370	5	Wuhan Louse Fly Virus 9	BFJSC7	Viruses; ssRNA viruses; ssRNA negative-strand viruses; unclassified ssRNA negative-strand viruses
GCA_003972765.1	20457	3	Wuhan Millipede Virus 2	WHWG03	Viruses; Riboviria; Orthornavirae; Negarnaviricota; Polyploviricotina; Ellioviricetes; Bunyavirales; Wupedeviridae; Wumivirus; Millipede wumivirus
GCA_001754925.1	10802	3	Wuhan mosquito virus 1	WT315	Viruses; Riboviria; Orthornavirae; Negarnaviricota; Polyploviricotina; Ellioviricetes; Bunyavirales; Phasmaviridae; Orthophasmavirus; Wuhan mosquito orthophasmavirus 1
GCA_001754485.1	10855	3	Wuhan Mosquito Virus 2	QN27	Viruses; ssRNA viruses; ssRNA negative-strand viruses; unclassified ssRNA negative-strand viruses
GCA_001440835.1	12004	3	Wuhan Mosquito Virus 8	XC27	Viruses; ssRNA viruses; ssRNA negative-strand viruses; unclassified ssRNA negative-strand viruses
GCA_001755345.1	13706	6	Wuhan Mosquito Virus 9	JX113	Viruses; ssRNA viruses; ssRNA negative-strand viruses; Mononegavirales; Rhabdoviridae; unclassified Rhabdoviridae
GCA_001755765.1	10306	4	Wuhan Tick Virus 1	X782	Viruses; ssRNA viruses; ssRNA negative-strand viruses; unclassified ssRNA negative-strand viruses
GCA_001440995.1	11395	3	Wuhan tick virus 2	X781	Viruses; Riboviria; Orthornavirae; Negarnaviricota; Haploviricotina; Monjiviricetes; Mononegavirales; Rhabdoviridae; unclassified Rhabdoviridae
GCA_001754905.1	15940	4	Wuhan horsefly Virus	JJ21	Viruses; ssRNA viruses; ssRNA negative-strand viruses; unclassified ssRNA negative-strand viruses
GCA_001755005.1	12774	4	Xincheng Mosquito Virus	XC16	Viruses; Riboviria; Orthornavirae; Negarnaviricota; Haploviricotina; Monjiviricetes; Mononegavirales; Xinmoviridae; Anphevirus; Xincheng anphevirus
GCA_018594725.1	17683	3	Xinzhou Spider Virus	XZZZ2	Viruses; Riboviria; Orthornavirae; Negarnaviricota; Polyploviricotina; Ellioviricetes; Bunyavirales; Nairoviridae; Xinspivirus; Xinzhou xinspivirus
GCA_001754945.1	10729	3	Yichang Insect virus	YCYC01	Viruses; ssRNA viruses; ssRNA negative-strand viruses; Bunyavirales; unclassified Bunyaviridae
GCA_013086905.1	8357	3	Yongjia Tick Virus 1	YJ11	Viruses; Riboviria; Orthornavirae; Negarnaviricota; Polyploviricotina; Ellioviricetes; Bunyavirales; Phenuiviridae; Uukuvirus; Yongjia uukuvirus
GCA_001754505.1	10833	5	Yongjia Tick Virus 2	YJ12	Viruses; ssRNA viruses; ssRNA negative-strand viruses; Mononegavirales; Rhabdoviridae; unclassified Rhabdoviridae
GCA_003029065.1	2746	6	Tomato latent virus	NA	Viruses; Monodnaviria; Shotokuvirae; Cressdnaviricota; Repensiviricetes; Geplafuvirales; Geminiviridae; Begomovirus
GCA_001470475.1	177643	301	Edwardsiella phage PEi20	NA	Viruses; dsDNA viruses, no RNA stage; Caudovirales; Myoviridae; unclassified Myoviridae
GCA_002633215.1	177215	300	Edwardsiella phage PEi26	NA	Viruses; Duplodnaviria; Heunggongvirae; Uroviricota; Caudoviricetes; Caudovirales; Myoviridae; Tevenvirinae; unclassified Tevenvirinae
GCA_000931155.1	5187	5	Meles meles polyomavirus 1	NA	Viruses; dsDNA viruses, no RNA stage; Polyomaviridae; Betapolyomavirus
GCA_000917115.1	9687	1	Narcissus late season yellows virus	NA	Viruses; ssRNA viruses; ssRNA positive-strand viruses, no DNA stage; Potyviridae; Potyvirus
GCA_000882395.1	9650	1	Narcissus yellow stripe virus	NA	Viruses; ssRNA viruses; ssRNA positive-strand viruses, no DNA stage; Potyviridae; Potyvirus
GCA_000869965.1	8539	6	Narcissus common latent virus	NA	Viruses; ssRNA viruses; ssRNA positive-strand viruses, no DNA stage; Tymovirales; Betaflexiviridae; Quinvirinae; Carlavirus
GCA_001021315.1	3141	2	Chimeric virus 14	NA	Viruses; unclassified viruses
GCA_001021275.1	3753	3	Phytophthora parasitica virus	NA	Viruses; unclassified viruses
GCA_001028985.1	5704	2	Camponotus yamaokai virus	TH2013a	Viruses; dsRNA viruses; Totiviridae; unclassified Totiviridae

gca	genome.size	proteome.size	phage.name	strain	Taxonomy
GCA_002080175.1	1374	1	Tomato leaf curl Pakistan betasatellite	NA	Viruses; Tolecusatellitidae; Betasatellite; unclassified Betasatellite
GCA_000959675.1	6083	9	Eel River basin pequenovirus	NA	Viruses; ssDNA viruses; Microviridae; unclassified Microviridae; Pequenovirus
GCA_001506035.1	38625	43	Citrobacter phage phiCFP1	NA	Viruses; dsDNA viruses, no RNA stage; Caudovirales; Podoviridae; unclassified Podoviridae
GCA_001504575.1	45045	64	Achromobacter phage phiAxp1	NA	Viruses; dsDNA viruses, no RNA stage; Caudovirales; Siphoviridae; unclassified Siphoviridae
GCA_001501655.1	59670	91	Escherichia phage CAjan	NA	Viruses; dsDNA viruses, no RNA stage; Caudovirales; Siphoviridae; Seuratvirus; Escherichia virus Cajan
GCA_001503435.1	49462	72	Klebsiella phage 1513	NA	Viruses; dsDNA viruses, no RNA stage; Caudovirales; Siphoviridae; Tunavirinae; Kp36virus; Klebsiella virus 1513
GCA_002597685.1	140218	209	Staphylococcus phage IMESA1	NA	Viruses; Duplodnaviria; Heunggongvirae; Uroviricota; Caudoviricetes; Caudovirales; Herelleviridae; Twortvirinae; Kayvirus; Staphylococcus virus G1
GCA_002597705.1	140906	212	Staphylococcus phage IMESA2	NA	Viruses; Duplodnaviria; Heunggongvirae; Uroviricota; Caudoviricetes; Caudovirales; Herelleviridae; Twortvirinae; Kayvirus; Staphylococcus virus G1
GCA_002149345.1	80148	110	Bacillus phage Stahl	NA	Viruses; Duplodnaviria; Heunggongvirae; Uroviricota; Caudoviricetes; Caudovirales; Siphoviridae; Slashvirus; Bacillus virus Stahl
GCA_002149365.1	80798	110	Bacillus phage Stills	NA	Viruses; Duplodnaviria; Heunggongvirae; Uroviricota; Caudoviricetes; Caudovirales; Siphoviridae; Slashvirus; Bacillus virus Stills
GCA_001501315.1	43809	54	Klebsiella phage vB_KpnP_SU503	NA	Viruses; dsDNA viruses, no RNA stage; Caudovirales; Podoviridae; Autographivirinae; Kp34virus; Klebsiella virus SU503
GCA_001500655.1	43595	54	Klebsiella phage vB_KpnP_SU552A	NA	Viruses; dsDNA viruses, no RNA stage; Caudovirales; Podoviridae; Autographivirinae; Kp34virus; Klebsiella virus SU552A
GCA_001503835.1	60747	86	Enterobacteria phage JenK1	NA	Viruses; dsDNA viruses, no RNA stage; Caudovirales; Siphoviridae; Nonagvirus; Escherichia virus JenK1
GCA_001503035.1	60754	87	Enterobacteria phage JenP1	NA	Viruses; dsDNA viruses, no RNA stage; Caudovirales; Siphoviridae; Nonagvirus; Escherichia virus JenP1
GCA_001504635.1	59802	87	Enterobacteria phage JenP2	NA	Viruses; dsDNA viruses, no RNA stage; Caudovirales; Siphoviridae; Nonagvirus; Escherichia virus JenP2
GCA_001501635.1	248088	390	Vibrio phage ValKK3	NA	Viruses; dsDNA viruses, no RNA stage; Caudovirales; Myoviridae; Tevenvirinae; Schizot4virus; Vibrio virus ValKK3
GCA_001502695.1	41843	62	Staphylococcus phage IMESA4	NA	Viruses; dsDNA viruses, no RNA stage; Caudovirales; Siphoviridae; unclassified Siphoviridae
GCA_000943745.1	1759	2	Mosquito circovirus	B19	Viruses; ssDNA viruses; Circoviridae; unclassified Circoviridae
GCA_000943705.1	5404	8	Asystasia mosaic Madagascar virus	NA	Viruses; ssDNA viruses; Geminiviridae; Begomovirus; unclassified Begomovirus
GCA_001470275.1	41868	53	Salmonella phage f18SE	NA	Viruses; dsDNA viruses, no RNA stage; Caudovirales; Siphoviridae; unclassified Siphoviridae
GCA_000973255.1	27004	5	Chinook salmon bafinivirus	NA	Viruses; ssRNA viruses; ssRNA positive-strand viruses, no DNA stage; Nidovirales; Coronaviridae; Torovirinae; Bafinivirus; unclassified Bafinivirus
GCA_000943725.1	2934	2	Cucurbit aphid borne yellows virus associated RNA	NA	Viruses; Virus-associated RNAs
GCA_001550965.1	12389	4	Adana virus	195	Viruses; Riboviria; Orthornavirae; Negarnaviricota; Polyploviricotina; Ellioviricetes; Bunyavirales; Phenuiviridae; Phlebovirus; Adana phlebovirus
GCA_009828255.1	11449	15	Adoxophyes honmai entomopoxvirus L virophage 1	NA	Viruses; unclassified virophages
GCA_009828275.1	12058	15	Adoxophyes honmai entomopoxvirus L virophage 2	NA	Viruses; unclassified virophages
GCA_009828295.1	12737	16	Choristoneura biennis entomopoxvirus L virophage	NA	Viruses; unclassified virophages
GCA_000867205.1	126629	147	Antheraea pernyi nucleopolyhedrovirus	Liaoning	Viruses; dsDNA viruses, no RNA stage; Baculoviridae; Alphabaculovirus
GCA_001019935.1	8376	3	Vesivirus ferret badgerJX12China2012	FBCVJX12	Viruses; ssRNA viruses; ssRNA positive-strand viruses, no DNA stage; Caliciviridae; Vesivirus; unclassified Vesivirus
GCA_001550785.1	18467	3	Tofla virus	Toku_Hfla_2013	Viruses; ssRNA viruses; ssRNA negative-strand viruses; Bunyaviridae; Nairovirus; unclassified Nairovirus
GCA_000861685.1	10053	1	Tamana bat virus	NA	Viruses; ssRNA viruses; ssRNA positive-strand viruses, no DNA stage; Flaviviridae; Flavivirus; unclassified Flavivirus
GCA_000851005.1	13173	15	Pseudomonas phage phi12	NA	Viruses; dsRNA viruses; Cystoviridae; unclassified Cystoviridae
GCA_000954875.1	1763	2	AvonHeathcote Estuary associated circular virus 1	NA	Viruses; ssDNA viruses; Circoviridae; unclassified Circoviridae
GCA_000954515.1	1981	2	AvonHeathcote Estuary associated circular virus 10	NA	Viruses; ssDNA viruses; Circoviridae; unclassified Circoviridae
GCA_000955235.1	2307	2	AvonHeathcote Estuary associated circular virus 11	NA	Viruses; ssDNA viruses; Circoviridae; unclassified Circoviridae

gca	genome.size	proteome.size	phage.name	strain	Taxonomy
GCA_000955575.1	2161	2	AvonHeathcote Estuary associated circular virus 12	NA	Viruses; ssDNA viruses; Circoviridae; unclassified Circoviridae
GCA_000954855.1	2212	2	AvonHeathcote Estuary associated circular virus 13	NA	Viruses; ssDNA viruses; Circoviridae; unclassified Circoviridae
GCA_000954495.1	2255	2	AvonHeathcote Estuary associated circular virus 14	NA	Viruses; ssDNA viruses; Circoviridae; unclassified Circoviridae
GCA_000955215.1	2817	2	AvonHeathcote Estuary associated circular virus 15	NA	Viruses; ssDNA viruses; Circoviridae; unclassified Circoviridae
GCA_000955555.1	1990	2	AvonHeathcote Estuary associated circular virus 16	NA	Viruses; ssDNA viruses; Circoviridae; unclassified Circoviridae
GCA_000954835.1	1741	2	AvonHeathcote Estuary associated circular virus 17	NA	Viruses; ssDNA viruses; Circoviridae; unclassified Circoviridae
GCA_000955195.1	2784	2	AvonHeathcote Estuary associated circular virus 19	NA	Viruses; ssDNA viruses; Circoviridae; unclassified Circoviridae
GCA_000955535.1	1865	2	AvonHeathcote Estuary associated circular virus 2	NA	Viruses; ssDNA viruses; Circoviridae; unclassified Circoviridae
GCA_000954815.1	2537	2	AvonHeathcote Estuary associated circular virus 20	NA	Viruses; ssDNA viruses; Circoviridae; unclassified Circoviridae
GCA_000954455.1	1769	2	AvonHeathcote Estuary associated circular virus 21	NA	Viruses; ssDNA viruses; Circoviridae; unclassified Circoviridae
GCA_000955175.1	2109	2	AvonHeathcote Estuary associated circular virus 22	NA	Viruses; ssDNA viruses; Circoviridae; unclassified Circoviridae
GCA_000955515.1	1942	2	AvonHeathcote Estuary associated circular virus 23	NA	Viruses; ssDNA viruses; Circoviridae; unclassified Circoviridae
GCA_000954795.1	2007	2	AvonHeathcote Estuary associated circular virus 24	NA	Viruses; ssDNA viruses; Circoviridae; unclassified Circoviridae
GCA_000954435.1	2142	2	AvonHeathcote Estuary associated circular virus 25	NA	Viruses; ssDNA viruses; Circoviridae; unclassified Circoviridae
GCA_000955155.1	2456	2	AvonHeathcote Estuary associated circular virus 26	NA	Viruses; ssDNA viruses; Circoviridae; unclassified Circoviridae
GCA_000955495.1	2632	2	AvonHeathcote Estuary associated circular virus 27	NA	Viruses; ssDNA viruses; Circoviridae; unclassified Circoviridae
GCA_000954775.1	2422	2	AvonHeathcote Estuary associated circular virus 28	NA	Viruses; ssDNA viruses; Circoviridae; unclassified Circoviridae
GCA_000954415.1	1825	2	AvonHeathcote Estuary associated circular virus 29	NA	Viruses; ssDNA viruses; Circoviridae; unclassified Circoviridae
GCA_000954575.1	2656	2	AvonHeathcote Estuary associated circular virus 3	NA	Viruses; ssDNA viruses; Circoviridae; unclassified Circoviridae
GCA_000955295.1	1835	2	AvonHeathcote Estuary associated circular virus 4	NA	Viruses; ssDNA viruses; Circoviridae; unclassified Circoviridae
GCA_000955635.1	2434	2	AvonHeathcote Estuary associated circular virus 5	NA	Viruses; ssDNA viruses; Circoviridae; unclassified Circoviridae
GCA_000954915.1	2096	2	AvonHeathcote Estuary associated circular virus 6	NA	Viruses; ssDNA viruses; Circoviridae; unclassified Circoviridae
GCA_000954555.1	1976	2	AvonHeathcote Estuary associated circular virus 7	NA	Viruses; ssDNA viruses; Circoviridae; unclassified Circoviridae
GCA_000955275.1	2184	2	AvonHeathcote Estuary associated circular virus 8	NA	Viruses; ssDNA viruses; Circoviridae; unclassified Circoviridae
GCA_000955615.1	1806	2	AvonHeathcote Estuary associated circular virus 9	NA	Viruses; ssDNA viruses; Circoviridae; unclassified Circoviridae
GCA_000959655.1	3612	3	Simian torque teno virus 30	NA	Viruses; ssDNA viruses; Anelloviridae; Alphatorquevirus; unclassified Alphatorquevirus
GCA_000954935.1	3907	4	Simian torque teno virus 31	NA	Viruses; ssDNA viruses; Anelloviridae; Alphatorquevirus; unclassified Alphatorquevirus
GCA_000959695.1	3812	4	Simian torque teno virus 32	NA	Viruses; ssDNA viruses; Anelloviridae; Alphatorquevirus; unclassified Alphatorquevirus
GCA_000954895.1	7796	8	Canis familiaris papillomavirus 16	NA	Viruses; Monodnaviria; Shotokuvirae; Cossaviricota; Papovaviricetes; Zurhausenvirales; Papillomaviridae; Firstpapillomavirinae; Chipapillomavirus; Chipapillomavirus 2
GCA_002819385.1	7609	3	Dioscorea bacilliform AL virus	NA	Viruses; Riboviria; Pararnavirae; Artverviricota; Revtraviricetes; Ortervirales; Caulimoviridae; Badnavirus
GCA_000852405.1	15522	8	Tioman virus	NA	Viruses; ssRNA viruses; ssRNA negative-strand viruses; Mononegavirales; Paramyxoviridae; Rubulavirus; unclassified Rubulavirus
GCA_004128855.1	11230	5	Zahedan rhabdovirus	NA	Viruses; Riboviria; Orthornavirae; Negarnaviricota; Haploviricotina; Monjiviricetes; Mononegavirales; Rhabdoviridae; Zarhavirus; Zahedan zarhavirus
GCA_006471825.1	103681	73	German gecko ranavirus	NA	Viruses; Varidnaviria; Bamfordvirae; Nucleocytoviricota; Megaviricetes; Pimascovirales; Iridoviridae; Alphairidovirinae; Ranavirus; Frog virus 3
GCA_001431955.1	12020	6	Inhangapi virus	BEAR177325	Viruses; ssRNA viruses; ssRNA negative-strand viruses; Mononegavirales; Rhabdoviridae; unclassified Rhabdoviridae

gca	genome.size	proteome.size	phage.name	strain	Taxonomy
GCA_002145625.1	13070	10	Iriri virus	BeAr408005	Viruses; Riboviria; Orthornavirae; Negarnaviricota; Haploviricotina; Monjiviricetes; Mononegavirales; Rhabdoviridae; Curiovirus; Iriri curiovirus
GCA_002146265.1	13189	10	Ord River virus	OR1023	Viruses; Riboviria; Orthornavirae; Negarnaviricota; Haploviricotina; Monjiviricetes; Mononegavirales; Rhabdoviridae; Hapavirus; Ord River hapavirus
GCA_013087315.1	11216	5	Sawgrass virus	64A1247	Viruses; Riboviria; Orthornavirae; Negarnaviricota; Haploviricotina; Monjiviricetes; Mononegavirales; Rhabdoviridae; Sawgrhavirus; Sawgrass sawgrhavirus
GCA_002146185.1	11290	9	Sripur virus	733646	Viruses; Riboviria; Orthornavirae; Negarnaviricota; Haploviricotina; Monjiviricetes; Mononegavirales; Rhabdoviridae; Sripuvirus; Sripur sripuvirus
GCA_000865625.1	13335	9	Human metapneumovirus	NA	Viruses; ssRNA viruses; ssRNA negative-strand viruses; Mononegavirales; Pneumoviridae; Metapneumovirus
GCA_001012905.1	10485	2	Desmodus rotundus endogenous retrovirus	NA	Viruses; Riboviria; Pararnavirae; Artverviricota; Revtraviricetes; Ortervirales; Retroviridae; Orthoretrovirinae; Betaretrovirus; unclassified Betaretrovirus
GCA_002605485.1	57061	77	Pseudomonas phage ZC01	NA	Viruses; Duplodnaviria; Heunggongvirae; Uroviricota; Caudoviricetes; Caudovirales; Siphoviridae; Abidjanvirus; unclassified Abidjanvirus
GCA_002605505.1	69844	85	Pseudomonas phage ZC03	NA	Viruses; Duplodnaviria; Heunggongvirae; Uroviricota; Caudoviricetes; Caudovirales; Podoviridae; unclassified Podoviridae
GCA_002605525.1	70774	83	Pseudomonas phage ZC08	NA	Viruses; Duplodnaviria; Heunggongvirae; Uroviricota; Caudoviricetes; Caudovirales; Podoviridae; unclassified Podoviridae
GCA_001502075.1	75167	90	Gordonia phage Gmala1	NA	Viruses; dsDNA viruses, no RNA stage; Caudovirales; Siphoviridae; unclassified Siphoviridae
GCA_001551065.1	76276	97	Gordonia phage GordDuk1	NA	Viruses; dsDNA viruses, no RNA stage; Caudovirales; Siphoviridae; unclassified Siphoviridae
GCA_001550685.1	75987	98	Gordonia phage GordTnk2	NA	Viruses; dsDNA viruses, no RNA stage; Caudovirales; Siphoviridae; unclassified Siphoviridae
GCA_001736655.1	43505	71	Gordonia phage Gsput1	NA	Viruses; dsDNA viruses, no RNA stage; Caudovirales; Siphoviridae; unclassified Siphoviridae
GCA_001308795.2	47636	83	Citrobacter phage CVT22	NA	Viruses; dsDNA viruses, no RNA stage; Caudovirales; Podoviridae; unclassified Podoviridae
GCA_002118625.2	12229	3	Bahig virus	EgB 90	Viruses; Riboviria; Orthornavirae; Negarnaviricota; Polyploviricotina; Ellioviricetes; Bunyavirales; Peribunyaviridae; Orthobunyavirus; Tete orthobunyavirus
GCA_004789815.1	12052	3	Tataguine virus	H9963	Viruses; Riboviria; Orthornavirae; Negarnaviricota; Polyploviricotina; Ellioviricetes; Bunyavirales; Peribunyaviridae; Orthobunyavirus; Tataguine orthobunyavirus
GCA_002827785.1	5040	4	Bat polyomavirus 5b	NA	Viruses; dsDNA viruses, no RNA stage; Polyomaviridae; unclassified Polyomaviridae
GCA_000970605.1	5019	4	Bat polyomavirus 6a	NA	Viruses; dsDNA viruses, no RNA stage; Polyomaviridae; unclassified Polyomaviridae
GCA_000969155.1	4956	4	Bat polyomavirus 6d	NA	Viruses; dsDNA viruses, no RNA stage; Polyomaviridae; unclassified Polyomaviridae
GCA_000969095.1	5075	4	Bat polyomavirus 5a	NA	Viruses; dsDNA viruses, no RNA stage; Polyomaviridae; unclassified Polyomaviridae
GCA_000969035.1	5046	4	Bat polyomavirus 6c	NA	Viruses; dsDNA viruses, no RNA stage; Polyomaviridae; unclassified Polyomaviridae
GCA_000969215.1	5039	4	Bat polyomavirus 6b	NA	Viruses; dsDNA viruses, no RNA stage; Polyomaviridae; unclassified Polyomaviridae
GCA_001501295.1	44846	85	Acinetobacter phage YMC1112R2315	NA	Viruses; dsDNA viruses, no RNA stage; Caudovirales; Myoviridae; unclassified Myoviridae
GCA_002605545.1	47575	80	Acinetobacter phage YMC1111R3177	NA	Viruses; Duplodnaviria; Heunggongvirae; Uroviricota; Caudoviricetes; Caudovirales; Siphoviridae; Vieuvirus; Acinetobacter virus R3177
GCA_002605565.1	44866	85	Acinetobacter phage YMC1112R1215	NA	Viruses; Duplodnaviria; Heunggongvirae; Uroviricota; Caudoviricetes; Caudovirales; Myoviridae; Obolenskvirus; unclassified Obolenskvirus
GCA_009731875.1	12217	4	Perdoes virus	NA	Viruses; Riboviria; Orthornavirae; Negarnaviricota; Polyploviricotina; Ellioviricetes; Bunyavirales; Peribunyaviridae; Orthobunyavirus; Oropouche orthobunyavirus
GCA_001190495.1	3442	5	Temperate fruit decayassociated virus	NA	Viruses; ssDNA viruses; unclassified ssDNA viruses
GCA_000960945.1	9617	1	Graminella nigrifrons virus 1	NA	Viruses; ssRNA viruses; ssRNA positive-strand viruses, no DNA stage; Picornavirales; Iflaviridae; unclassified Iflaviridae
GCA_013086915.1	13653	3	Groundnut chlorotic fanspot virus	NA	Viruses; Riboviria; Orthornavirae; Negarnaviricota; Polyploviricotina; Ellioviricetes; Bunyavirales; Tospoviridae; Orthotospovirus; Groundnut chlorotic fan spot orthotospovirus
GCA_002604705.1	40525	49	Pseudomonas phage phiPsa17	NA	Viruses; Duplodnaviria; Heunggongvirae; Uroviricota; Caudoviricetes; Caudovirales; Autographiviridae; Studiervirinae; Ghunavirus; Pseudomonas virus PhiPsa17
GCA_000969135.1	3774	4	Simian torque teno virus 33	NA	Viruses; ssDNA viruses; Anelloviridae; Alphatorquevirus; unclassified Alphatorquevirus
GCA_000969075.1	3621	4	Simian torque teno virus 34	NA	Viruses; ssDNA viruses; Anelloviridae; Alphatorquevirus; unclassified Alphatorquevirus
GCA_001461625.1	2497	1	Botrytis cinerea mitovirus 2	NA	Viruses; ssRNA viruses; ssRNA positive-strand viruses, no DNA stage; Narnaviridae; Mitovirus; unclassified Mitovirus
GCA_001461465.1	2922	1	Botrytis cinerea mitovirus 3	NA	Viruses; ssRNA viruses; ssRNA positive-strand viruses, no DNA stage; Narnaviridae; Mitovirus; unclassified Mitovirus

gca	genome.size	proteome.size	phage.name	strain	Taxonomy
GCA_001461185.1	2768	1	Botrytis cinerea mitovirus 4	NA	Viruses; ssRNA viruses; ssRNA positive-strand viruses, no DNA stage; Narnaviridae; Mitovirus; unclassified Mitovirus
GCA_001461065.1	8543	1	Botrytis cinerea negativestranded RNA virus 1	NA	Viruses; ssRNA viruses; ssRNA negative-strand viruses; unclassified ssRNA negative-strand viruses
GCA_002989855.1	50307	70	Marinitoga camini virus 2	NA	Viruses; Duplodnaviria; Heunggongvirae; Uroviricota; Caudoviricetes; Caudovirales; Siphoviridae; unclassified Siphoviridae
GCA_002989835.1	53412	77	Marinitoga camini virus 1	NA	Viruses; Duplodnaviria; Heunggongvirae; Uroviricota; Caudoviricetes; Caudovirales; Siphoviridae; unclassified Siphoviridae
GCA_000969015.1	2952	5	Mulberry mosaic dwarf associated virus	NA	Viruses; ssDNA viruses; Geminiviridae; unclassified Geminiviridae
GCA_001021215.1	20261	7	Bovine nidovirus TCH5	NA	Viruses; ssRNA viruses; ssRNA positive-strand viruses, no DNA stage; Nidovirales; unclassified Nidovirales
GCA_000969195.1	2756	6	Tomato enation leaf curl virus	NA	Viruses; ssDNA viruses; Geminiviridae; Begomovirus; unclassified Begomovirus
GCA_000969115.1	2737	6	Malvastrum yellow vein Cambodia virus	NA	Viruses; ssDNA viruses; Geminiviridae; Begomovirus; unclassified Begomovirus
GCA_002826765.1	12374	3	Laibin virus	BT20	Viruses; Riboviria; Orthornavirae; Negarnaviricota; Polyploviricotina; Ellioviricetes; Bunyavirales; Hantaviridae; Mammantavirinae; Mobatvirus; Laibin mobatvirus
GCA_000868405.1	17212	5	Capsicum chlorosis virus	NA	Viruses; ssRNA viruses; ssRNA negative-strand viruses; Bunyaviridae; Tospovirus; unclassified Tospovirus
GCA_009731895.1	12634	4	Itaya virus	IQT9646	Viruses; Riboviria; Orthornavirae; Negarnaviricota; Polyploviricotina; Ellioviricetes; Bunyavirales; Peribunyaviridae; Orthobunyavirus; Caraparu orthobunyavirus
GCA_000969055.1	2932	6	Apple geminivirus PL2015	NA	Viruses; ssDNA viruses; Geminiviridae; unclassified Geminiviridae
GCA_002605585.1	41913	56	Listeria phage LWP01	NA	Viruses; Duplodnaviria; Heunggongvirae; Uroviricota; Caudoviricetes; Caudovirales; Siphoviridae; unclassified Siphoviridae
GCA_000972615.1	8850	1	Bovine hepacivirus	NA	Viruses; ssRNA viruses; ssRNA positive-strand viruses, no DNA stage; Flaviviridae; Hepacivirus; unclassified Hepacivirus
GCA_000989015.1	5132	7	Sheep polyomavirus 1	NA	Viruses; dsDNA viruses, no RNA stage; Polyomaviridae; unclassified Polyomaviridae
GCA_004129215.1	2085	2	Apteryx rowi circoviruslike virus	NA	Viruses; Monodnaviria; Shotokuvirae; Cressdnaviricota; Arfiviricetes; Cirlivirales; Circoviridae; unclassified Circoviridae
GCA_000973355.1	2112	2	Badger fecesassociated gemycircularvirus	588t	Viruses; unclassified viruses; Genomoviridae; Gemycircularvirus; unclassified Gemycircularvirus
GCA_000973335.1	2089	2	Mongoose fecesassociated gemycircularvirus a	181a	Viruses; unclassified viruses; Genomoviridae; Gemycircularvirus; unclassified Gemycircularvirus
GCA_000973415.1	2189	2	Mongoose fecesassociated gemycircularvirus b	160b	Viruses; unclassified viruses; Genomoviridae; Gemycircularvirus; unclassified Gemycircularvirus
GCA_000973475.1	2251	2	Mongoose fecesassociated gemycircularvirus d	478d	Viruses; unclassified viruses; Genomoviridae; Gemycircularvirus; unclassified Gemycircularvirus
GCA_000973235.1	2123	2	Mongoose fecesassociated gemycircularvirus c	541c	Viruses; unclassified viruses; Genomoviridae; Gemycircularvirus; unclassified Gemycircularvirus
GCA_000973315.1	7641	6	Taro bacilliform CH virus	NA	Viruses; Riboviria; Pararnavirae; Artverviricota; Revtraviricetes; Ortervirales; Caulimoviridae; Badnavirus
GCA_001470155.1	149218	271	Sinorhizobium phage phiM9	NA	Viruses; dsDNA viruses, no RNA stage; Caudovirales; Myoviridae; unclassified Myoviridae
GCA_001500635.1	56528	70	Streptomyces phage YDN12	NA	Viruses; dsDNA viruses, no RNA stage; Caudovirales; Siphoviridae; unclassified Siphoviridae
GCA_001502675.1	57168	71	Streptomyces phage TP1604	NA	Viruses; dsDNA viruses, no RNA stage; Caudovirales; Siphoviridae; unclassified Siphoviridae
GCA_001551385.1	72192	86	Pseudomonas phage YH30	NA	Viruses; dsDNA viruses, no RNA stage; Caudovirales; Podoviridae; N4virus; unclassified N4likevirus
GCA_001503815.1	43190	75	Pseudoalteromonas phage H103	NA	Viruses; dsDNA viruses, no RNA stage; Caudovirales; unclassified Caudovirales
GCA_002605605.1	36499	51	Burkholderia phage AP3	NA	Viruses; Duplodnaviria; Heunggongvirae; Uroviricota; Caudoviricetes; Caudovirales; Myoviridae; Peduovirinae; Kisquinquevirus; Burkholderia virus AP3
GCA_001502055.1	87372	131	Escherichia phage vB_EcoM_AYO145A	NA	Viruses; dsDNA viruses, no RNA stage; Caudovirales; Myoviridae; Felixo1virus; unclassified FelixO1likevirus
GCA_001503015.1	42881	64	Staphylococcus phage B166	NA	Viruses; dsDNA viruses, no RNA stage; Caudovirales; Siphoviridae; Phietavirus; unclassified Phietalikevirus
GCA_001504615.1	43228	67	Staphylococcus phage B236	NA	Viruses; dsDNA viruses, no RNA stage; Caudovirales; Siphoviridae; Phietavirus; unclassified Phietalikevirus
GCA_001501275.1	44573	53	Proteus phage vB_PmiP_Pm5460	NA	Viruses; dsDNA viruses, no RNA stage; Caudovirales; Podoviridae; Autographivirinae; Sp6virus; unclassified Sp6likevirus
GCA_001500615.1	161989	256	Proteus phage vB_PmiM_Pm5461	NA	Viruses; dsDNA viruses, no RNA stage; Caudovirales; Myoviridae; Tevenvirinae; T4virus; unclassified T4virus
GCA_002756535.1	45653	86	Paenibacillus phage Diane	NA	Viruses; Duplodnaviria; Heunggongvirae; Uroviricota; Caudoviricetes; Caudovirales; Siphoviridae; Vegasvirus; Paenibacillus virus Vegas

gca	genome.size	proteome.size	phage.name	strain	Taxonomy
GCA_001502655.1	37995	68	Paenibacillus phage Fern	NA	Viruses; dsDNA viruses, no RNA stage; Caudovirales; Siphoviridae; unclassified Siphoviridae
GCA_003369225.1	55560	90	Paenibacillus phage Halcyone	NA	Viruses; Duplodnaviria; Heunggongvirae; Uroviricota; Caudoviricetes; Caudovirales; Siphoviridae; Trippvirus; unclassified Trippvirus
GCA_001501775.1	44249	84	Paenibacillus phage Harrison	NA	Viruses; dsDNA viruses, no RNA stage; Caudovirales; Siphoviridae; unclassified Siphoviridae
GCA_003369205.1	55560	90	Paenibacillus phage Heath	NA	Viruses; Duplodnaviria; Heunggongvirae; Uroviricota; Caudoviricetes; Caudovirales; Siphoviridae; Trippvirus; unclassified Trippvirus
GCA_003369185.1	55990	91	Paenibacillus phage Scottie	NA	Viruses; Duplodnaviria; Heunggongvirae; Uroviricota; Caudoviricetes; Caudovirales; Siphoviridae; Trippvirus; unclassified Trippvirus
GCA_002756555.1	45653	86	Paenibacillus phage Vadim	NA	Viruses; Duplodnaviria; Heunggongvirae; Uroviricota; Caudoviricetes; Caudovirales; Siphoviridae; Vegasvirus; Paenibacillus virus Vegas
GCA_001502035.1	45653	86	Paenibacillus phage Vegas	NA	Viruses; dsDNA viruses, no RNA stage; Caudovirales; Siphoviridae; unclassified Siphoviridae
GCA_002605625.1	37994	68	Paenibacillus phage Willow	NA	Viruses; Duplodnaviria; Heunggongvirae; Uroviricota; Caudoviricetes; Caudovirales; Siphoviridae; Sitaravirus; Paenibacillus virus Willow
GCA_001501255.1	41149	77	Paenibacillus phage Xenia	NA	Viruses; dsDNA viruses, no RNA stage; Caudovirales; Siphoviridae; unclassified Siphoviridae
GCA_001551705.1	36315	44	Rhodoferax phage P26218	NA	Viruses; dsDNA viruses, no RNA stage; Caudovirales; Podoviridae; unclassified Podoviridae
GCA_001534595.1	32894	52	Verrucomicrobia phage P8625	NA	Viruses; dsDNA viruses, no RNA stage; Caudovirales; Siphoviridae; unclassified Siphoviridae
GCA_001550525.1	36051	41	Enterobacter phage E2	NA	Viruses; dsDNA viruses, no RNA stage; Caudovirales; Podoviridae; unclassified Podoviridae
GCA_001501615.1	31522	36	Enterobacter phage E3	NA	Viruses; dsDNA viruses, no RNA stage; Caudovirales; Podoviridae; unclassified Podoviridae
GCA_002605665.1	39142	52	Enterobacter phage E4	NA	Viruses; Duplodnaviria; Heunggongvirae; Uroviricota; Caudoviricetes; Caudovirales; Autographiviridae; Studiervirinae; Teetrevirus; unclassified Teetrevirus
GCA_002605685.1	55748	67	Skermania phage SPI1	NA	Viruses; Duplodnaviria; Heunggongvirae; Uroviricota; Caudoviricetes; Caudovirales; Siphoviridae; unclassified Siphoviridae
GCA_001500595.1	76964	107	Tsukamurella phage TIN2	NA	Viruses; dsDNA viruses, no RNA stage; Caudovirales; Siphoviridae; unclassified Siphoviridae
GCA_001502635.1	76269	109	Tsukamurella phage TIN3	NA	Viruses; dsDNA viruses, no RNA stage; Caudovirales; Siphoviridae; unclassified Siphoviridae
GCA_002623325.1	76268	109	Tsukamurella phage TIN4	NA	Viruses; Duplodnaviria; Heunggongvirae; Uroviricota; Caudoviricetes; Caudovirales; Siphoviridae; Tinduovirus; Tsukamurella virus TIN4
GCA_002986605.1	2748	6	Hollyhock leaf curl virus	NA	Viruses; Monodnaviria; Shotokuvirae; Cressdnaviricota; Repensiviricetes; Geplafuvirales; Geminiviridae; Begomovirus
GCA_002593725.1	31561	57	Lactococcus phage 936 group phage Phi16	NA	Viruses; Duplodnaviria; Heunggongvirae; Uroviricota; Caudoviricetes; Caudovirales; Siphoviridae; Skunavirus; unclassified Skunavirus
GCA_002593765.1	31805	63	Lactococcus phage 936 group phage Phi105	NA	Viruses; Duplodnaviria; Heunggongvirae; Uroviricota; Caudoviricetes; Caudovirales; Siphoviridae; Skunavirus; unclassified Skunavirus
GCA_002593445.1	31033	54	Lactococcus phage 936 group phage Phi109	NA	Viruses; Duplodnaviria; Heunggongvirae; Uroviricota; Caudoviricetes; Caudovirales; Siphoviridae; Skunavirus; unclassified Skunavirus
GCA_002593345.1	30532	53	Lactococcus phage 936 group phage Phi114	NA	Viruses; Duplodnaviria; Heunggongvirae; Uroviricota; Caudoviricetes; Caudovirales; Siphoviridae; Skunavirus; unclassified Skunavirus
GCA_002593285.1	30178	54	Lactococcus phage 936 group phage Phi129	NA	Viruses; Duplodnaviria; Heunggongvirae; Uroviricota; Caudoviricetes; Caudovirales; Siphoviridae; Skunavirus; unclassified Skunavirus
GCA_002593365.1	30577	54	Lactococcus phage 936 group phage Phi1316	NA	Viruses; Duplodnaviria; Heunggongvirae; Uroviricota; Caudoviricetes; Caudovirales; Siphoviridae; Skunavirus; unclassified Skunavirus
GCA_002593625.1	32150	55	Lactococcus phage 936 group phage Phi155	NA	Viruses; Duplodnaviria; Heunggongvirae; Uroviricota; Caudoviricetes; Caudovirales; Siphoviridae; Skunavirus; unclassified Skunavirus
GCA_002593325.1	30463	56	Lactococcus phage 936 group phage Phi17	NA	Viruses; Duplodnaviria; Heunggongvirae; Uroviricota; Caudoviricetes; Caudovirales; Siphoviridae; Skunavirus; unclassified Skunavirus
GCA_002593125.1	28437	50	Lactococcus phage 936 group phage Phi19	NA	Viruses; Duplodnaviria; Heunggongvirae; Uroviricota; Caudoviricetes; Caudovirales; Siphoviridae; Skunavirus; unclassified Skunavirus
GCA_002593745.1	29984	55	Lactococcus phage 936 group phage Phi192	NA	Viruses; Duplodnaviria; Heunggongvirae; Uroviricota; Caudoviricetes; Caudovirales; Siphoviridae; Skunavirus; unclassified Skunavirus
GCA_002593165.1	28811	52	Lactococcus phage 936 group phage Phi193	NA	Viruses; Duplodnaviria; Heunggongvirae; Uroviricota; Caudoviricetes; Caudovirales; Siphoviridae; Skunavirus; unclassified Skunavirus
GCA_002593085.1	28764	51	Lactococcus phage 936 group phage Phi4	NA	Viruses; Duplodnaviria; Heunggongvirae; Uroviricota; Caudoviricetes; Caudovirales; Siphoviridae; Skunavirus; unclassified Skunavirus
GCA_002593485.1	31375	59	Lactococcus phage 936 group phage Phi42	NA	Viruses; Duplodnaviria; Heunggongvirae; Uroviricota; Caudoviricetes; Caudovirales; Siphoviridae; Skunavirus; unclassified Skunavirus
GCA_002593565.1	31631	57	Lactococcus phage 936 group phage Phi40	NA	Viruses; Duplodnaviria; Heunggongvirae; Uroviricota; Caudoviricetes; Caudovirales; Siphoviridae; Skunavirus; unclassified Skunavirus
GCA_002593265.1	29416	54	Lactococcus phage 936 group phage Phi43	NA	Viruses; Duplodnaviria; Heunggongvirae; Uroviricota; Caudoviricetes; Caudovirales; Siphoviridae; Skunavirus; unclassified Skunavirus

gca	genome.size	proteome.size	phage.name	strain	Taxonomy
GCA_002593505.1	31379	52	Lactococcus phage 936 group phage Phi44	NA	Viruses; Duplodnaviria; Heunggongvirae; Uroviricota; Caudoviricetes; Caudovirales; Siphoviridae; Skunavirus; unclassified Skunavirus
GCA_002593225.1	29026	53	Lactococcus phage 936 group phage Phi512	NA	Viruses; Duplodnaviria; Heunggongvirae; Uroviricota; Caudoviricetes; Caudovirales; Siphoviridae; Skunavirus; unclassified Skunavirus
GCA_002593525.1	31495	58	Lactococcus phage 936 group phage Phi91127	NA	Viruses; Duplodnaviria; Heunggongvirae; Uroviricota; Caudoviricetes; Caudovirales; Siphoviridae; Skunavirus; unclassified Skunavirus
GCA_002593185.1	28873	52	Lactococcus phage 936 group phage PhiA1127	NA	Viruses; Duplodnaviria; Heunggongvirae; Uroviricota; Caudoviricetes; Caudovirales; Siphoviridae; Skunavirus; unclassified Skunavirus
GCA_002593145.1	28785	53	Lactococcus phage 936 group phage PhiB1127	NA	Viruses; Duplodnaviria; Heunggongvirae; Uroviricota; Caudoviricetes; Caudovirales; Siphoviridae; Skunavirus; unclassified Skunavirus
GCA_002593245.1	29032	53	Lactococcus phage 936 group phage PhiC0139	NA	Viruses; Duplodnaviria; Heunggongvirae; Uroviricota; Caudoviricetes; Caudovirales; Siphoviridae; Skunavirus; unclassified Skunavirus
GCA_002593205.1	28833	53	Lactococcus phage 936 group phage PhiD18	NA	Viruses; Duplodnaviria; Heunggongvirae; Uroviricota; Caudoviricetes; Caudovirales; Siphoviridae; Skunavirus; unclassified Skunavirus
GCA_002593645.1	32270	60	Lactococcus phage 936 group phage PhiE1127	NA	Viruses; Duplodnaviria; Heunggongvirae; Uroviricota; Caudoviricetes; Caudovirales; Siphoviridae; Skunavirus; unclassified Skunavirus
GCA_002593305.1	30389	56	Lactococcus phage 936 group phage PhiF17	NA	Viruses; Duplodnaviria; Heunggongvirae; Uroviricota; Caudoviricetes; Caudovirales; Siphoviridae; Skunavirus; unclassified Skunavirus
GCA_002593405.1	30811	57	Lactococcus phage 936 group phage PhiF0139	NA	Viruses; Duplodnaviria; Heunggongvirae; Uroviricota; Caudoviricetes; Caudovirales; Siphoviridae; Skunavirus; unclassified Skunavirus
GCA_002593385.1	30594	58	Lactococcus phage 936 group phage PhiG	NA	Viruses; Duplodnaviria; Heunggongvirae; Uroviricota; Caudoviricetes; Caudovirales; Siphoviridae; Skunavirus; unclassified Skunavirus
GCA_002593605.1	31901	58	Lactococcus phage 936 group phage PhiJF1	NA	Viruses; Duplodnaviria; Heunggongvirae; Uroviricota; Caudoviricetes; Caudovirales; Siphoviridae; Skunavirus; unclassified Skunavirus
GCA_002593425.1	30882	55	Lactococcus phage 936 group phage PhiL18	NA	Viruses; Duplodnaviria; Heunggongvirae; Uroviricota; Caudoviricetes; Caudovirales; Siphoviridae; Skunavirus; unclassified Skunavirus
GCA_002593465.1	31181	59	Lactococcus phage 936 group phage PhiL6	NA	Viruses; Duplodnaviria; Heunggongvirae; Uroviricota; Caudoviricetes; Caudovirales; Siphoviridae; Skunavirus; unclassified Skunavirus
GCA_002593685.1	28983	49	Lactococcus phage 936 group phage PhiLj	NA	Viruses; Duplodnaviria; Heunggongvirae; Uroviricota; Caudoviricetes; Caudovirales; Siphoviridae; Skunavirus; unclassified Skunavirus
GCA_002593585.1	31869	54	Lactococcus phage 936 group phage PhiM16	NA	Viruses; Duplodnaviria; Heunggongvirae; Uroviricota; Caudoviricetes; Caudovirales; Siphoviridae; Skunavirus; unclassified Skunavirus
GCA_002593545.1	31498	58	Lactococcus phage 936 group phage PhiM5	NA	Viruses; Duplodnaviria; Heunggongvirae; Uroviricota; Caudoviricetes; Caudovirales; Siphoviridae; Skunavirus; unclassified Skunavirus
GCA_002593665.1	32682	59	Lactococcus phage 936 group phage PhiM1127	NA	Viruses; Duplodnaviria; Heunggongvirae; Uroviricota; Caudoviricetes; Caudovirales; Siphoviridae; Skunavirus; unclassified Skunavirus
GCA_002593705.1	30116	53	Lactococcus phage 936 group phage PhiS0139	NA	Viruses; Duplodnaviria; Heunggongvirae; Uroviricota; Caudoviricetes; Caudovirales; Siphoviridae; Skunavirus; unclassified Skunavirus
GCA_002593105.1	28349	49	Lactococcus phage 936 group phage PhiA16	NA	Viruses; Duplodnaviria; Heunggongvirae; Uroviricota; Caudoviricetes; Caudovirales; Siphoviridae; Skunavirus; unclassified Skunavirus
GCA_003033825.1	8621	5	Tomato necrotic streak virus	NA	Viruses; Riboviria; Orthornavirae; Kitrinoviricota; Alsuviricetes; Martellivirales; Bromoviridae; Ilarvirus
GCA_000973395.2	17989	12	Rotavirus I	KE1352012	Viruses; dsRNA viruses; Reoviridae; Sedoreovirinae; Rotavirus; unclassified rotaviruses
GCA_002816815.1	7441	1	Dromedary camel enterovirus 19CC	NA	Viruses; Riboviria; Orthornavirae; Pisuviricota; Pisoniviricetes; Picornavirales; Picornaviridae; Enterovirus; Enterovirus I
GCA_001744375.1	72979	94	Pseudomonas phage vB_PaeP_MAG4	NA	Viruses; dsDNA viruses, no RNA stage; Caudovirales; Podoviridae; unclassified Podoviridae
GCA_001743695.1	94555	174	Pseudomonas phage vB_PaeM_MAG1	NA	Viruses; unclassified phages
GCA_002375125.2	24596	10	Grapevine Cabernet Sauvignon reovirus	NA	Viruses; Riboviria; Orthornavirae; Duplornaviricota; Resentoviricetes; Reovirales; Reoviridae; unclassified Reoviridae
GCA_000973455.1	12800	1	Fusarium graminearum hypovirus 2	NA	Viruses; dsRNA viruses; Hypoviridae; Hypovirus; unclassified Hypovirus
GCA_002756575.1	69110	106	Mycobacterium phage PDRPv	NA	Viruses; Duplodnaviria; Heunggongvirae; Uroviricota; Caudoviricetes; Caudovirales; Siphoviridae; Bclasvirinae; Pegunavirus; Mycobacterium virus Oline
GCA_002756595.1	69171	106	Mycobacterium phage PDRPxv	NA	Viruses; Duplodnaviria; Heunggongvirae; Uroviricota; Caudoviricetes; Caudovirales; Siphoviridae; Bclasvirinae; Pegunavirus; Mycobacterium virus Oline
GCA_001502015.1	130952	198	Enterococcus phage EFLK1	NA	Viruses; dsDNA viruses, no RNA stage; Caudovirales; Myoviridae; unclassified Myoviridae
GCA_001500955.1	45673	71	Pseudomonas phage DL54	NA	Viruses; dsDNA viruses, no RNA stage; Caudovirales; Podoviridae; unclassified Podoviridae
GCA_001500995.1	66103	89	Pseudomonas phage DL60	NA	Viruses; dsDNA viruses, no RNA stage; Caudovirales; Myoviridae; unclassified Myoviridae
GCA_001502995.1	42508	55	Pseudomonas phage DL62	NA	Viruses; dsDNA viruses, no RNA stage; Caudovirales; Podoviridae; unclassified Podoviridae
GCA_001502375.1	72378	90	Pseudomonas phage DL64	NA	Viruses; dsDNA viruses, no RNA stage; Caudovirales; Podoviridae; unclassified Podoviridae

gca	genome.size	proteome.size	phage.name	strain	Taxonomy
GCA_001501595.1	66111	92	Pseudomonas phage DL68	NA	Viruses; dsDNA viruses, no RNA stage; Caudovirales; Myoviridae; unclassified Myoviridae
GCA_002597225.1	65867	90	Pseudomonas phage DL52	NA	Viruses; Duplodnaviria; Heunggongvirae; Uroviricota; Caudoviricetes; Caudovirales; Myoviridae; Pbunavirus; Pseudomonas virus PB1
GCA_002605705.1	39921	65	Fusobacterium phage Funu1	NA	Viruses; Duplodnaviria; Heunggongvirae; Uroviricota; Caudoviricetes; Caudovirales; Myoviridae; unclassified Myoviridae
GCA_001271155.1	31848	30	Skunk adenovirus PB1	NA	Viruses; dsDNA viruses, no RNA stage; Adenoviridae; Mastadenovirus; unclassified Mastadenovirus
GCA_000981755.1	8003	1	Falcovirus A1	kestrelVOVE06222013HUN	Viruses; ssRNA viruses; ssRNA positive-strand viruses, no DNA stage; Picornavirales; Picornaviridae; unclassified Picornaviridae
GCA_000989115.1	6334	4	Cherax quadricarinatus densovirus	NA	Viruses; ssDNA viruses; Parvoviridae; Densovirinae; unclassified Densovirinae
GCA_001020075.1	10200	4	Mariental mammarenavirus	N27 MRMin9	Viruses; ssRNA viruses; ssRNA negative-strand viruses; Arenaviridae; Mammarenavirus
GCA_001019795.1	10549	4	Okahandja mammarenavirus	N73 OkhMin4	Viruses; ssRNA viruses; ssRNA negative-strand viruses; Arenaviridae; Mammarenavirus
GCA_004058275.1	1983	5	Zebra finch circovirus	32469	Viruses; ssDNA viruses; Circoviridae; Circovirus
GCA_002606745.1	42887	57	Stenotrophomonas phage vB_SmaSDLP_1	NA	Viruses; Duplodnaviria; Heunggongvirae; Uroviricota; Caudoviricetes; Caudovirales; Siphoviridae; Septimatrevirus; unclassified Septimatrevirus
GCA_001501575.1	42593	58	Stenotrophomonas phage vB_SmaSDLP_2	NA	Viruses; Duplodnaviria; Heunggongvirae; Uroviricota; Caudoviricetes; Caudovirales; Siphoviridae; Septimatrevirus; unclassified Septimatrevirus
GCA_004789935.1	12227	4	Anadyr virus	LEIV13395Mg	Viruses; Riboviria; Orthornavirae; Negarnaviricota; Polyploviricotina; Ellioviricetes; Bunyavirales; Peribunyaviridae; Orthobunyavirus; Anadyr orthobunyavirus
GCA_000982285.1	157977	137	Lambdina fiscellaria nucleopolyhedrovirus	NA	Viruses; dsDNA viruses, no RNA stage; Baculoviridae; Alphabaculovirus; unclassified Alphabaculovirus
GCA_002149765.1	38663	56	Bacillus phage phi4B1	NA	Viruses; dsDNA viruses, no RNA stage; Caudovirales; Siphoviridae; unclassified Siphoviridae
GCA_002602345.1	41999	63	Bacillus phage phi4I1	NA	Viruses; Duplodnaviria; Heunggongvirae; Uroviricota; Caudoviricetes; Caudovirales; Siphoviridae; unclassified Siphoviridae
GCA_002149545.1	41486	67	Bacillus phage phi4J1	NA	Viruses; dsDNA viruses, no RNA stage; Caudovirales; Siphoviridae; unclassified Siphoviridae
GCA_002605725.1	46635	79	Bacillus phage phiS58	NA	Viruses; Duplodnaviria; Heunggongvirae; Uroviricota; Caudoviricetes; Caudovirales; Siphoviridae; unclassified Siphoviridae
GCA_006441405.1	35192	46	Human adenovirus 71	humanDEUHEIM_00085198771P9H20F71	Viruses; Varidnaviria; Bamfordvirae; Preplasmiviricota; Tectiliviricetes; Rowavirales; Adenoviridae; Mastadenovirus; Human mastadenovirus D
GCA_006401055.1	35914	39	Human adenovirus 4p	NHRC_90870	Viruses; Varidnaviria; Bamfordvirae; Preplasmiviricota; Tectiliviricetes; Rowavirales; Adenoviridae; Mastadenovirus; Human mastadenovirus E; Human adenovirus E4
GCA_000883915.1	10814	1	Wesselsbron virus	SAH177	Viruses; ssRNA viruses; ssRNA positive-strand viruses, no DNA stage; Flaviviridae; Flavivirus; Yellow fever virus group
GCA_000986455.1	13133	11	Influenza A virus AEngland107405712010H1N1	NA	Viruses; Riboviria; Orthornavirae; Negarnaviricota; Polyploviricotina; Insthoviricetes; Articulavirales; Orthomyxoviridae; Alphainfluenzavirus; Influenza A virus; H1N1 subtype
GCA_000986075.1	13133	11	Influenza A virus AEngland122010H1N1	NA	Viruses; Riboviria; Orthornavirae; Negarnaviricota; Polyploviricotina; Insthoviricetes; Articulavirales; Orthomyxoviridae; Alphainfluenzavirus; Influenza A virus; H1N1 subtype
GCA_001013805.1	13133	11	Influenza A virus AEngland6882010H1N1	NA	Viruses; Riboviria; Orthornavirae; Negarnaviricota; Polyploviricotina; Insthoviricetes; Articulavirales; Orthomyxoviridae; Alphainfluenzavirus; Influenza A virus; H1N1 subtype
GCA_001013885.1	13133	11	Influenza A virus AEngland108207332010H1N1	NA	Viruses; Riboviria; Orthornavirae; Negarnaviricota; Polyploviricotina; Insthoviricetes; Articulavirales; Orthomyxoviridae; Alphainfluenzavirus; Influenza A virus; H1N1 subtype
GCA_001013765.1	13133	11	Influenza A virus AEngland107406642011H1N1	NA	Viruses; Riboviria; Orthornavirae; Negarnaviricota; Polyploviricotina; Insthoviricetes; Articulavirales; Orthomyxoviridae; Alphainfluenzavirus; Influenza A virus; H1N1 subtype
GCA_001013745.1	13133	11	Influenza A virus AEnglandWTSI17692010H1N1	NA	Viruses; Riboviria; Orthornavirae; Negarnaviricota; Polyploviricotina; Insthoviricetes; Articulavirales; Orthomyxoviridae; Alphainfluenzavirus; Influenza A virus; H1N1 subtype
GCA_000986375.1	13133	11	Influenza A virus AEngland3312010H1N1	NA	Viruses; Riboviria; Orthornavirae; Negarnaviricota; Polyploviricotina; Insthoviricetes; Articulavirales; Orthomyxoviridae; Alphainfluenzavirus; Influenza A virus; H1N1 subtype
GCA_000986515.1	13133	11	Influenza A virus AEngland92009H1N1	NA	Viruses; Riboviria; Orthornavirae; Negarnaviricota; Polyploviricotina; Insthoviricetes; Articulavirales; Orthomyxoviridae; Alphainfluenzavirus; Influenza A virus; H1N1 subtype
GCA_001013605.1	13133	11	Influenza A virus AEngland292010H1N1	NA	Viruses; Riboviria; Orthornavirae; Negarnaviricota; Polyploviricotina; Insthoviricetes; Articulavirales; Orthomyxoviridae; Alphainfluenzavirus; Influenza A virus; H1N1 subtype
GCA_001013945.1	13133	11	Influenza A virus AEngland6942010H1N1	NA	Viruses; Riboviria; Orthornavirae; Negarnaviricota; Polyploviricotina; Insthoviricetes; Articulavirales; Orthomyxoviridae; Alphainfluenzavirus; Influenza A virus; H1N1 subtype
GCA_001013625.1	13133	11	Influenza A virus AEngland4682010H1N1	NA	Viruses; Riboviria; Orthornavirae; Negarnaviricota; Polyploviricotina; Insthoviricetes; Articulavirales; Orthomyxoviridae; Alphainfluenzavirus; Influenza A virus; H1N1 subtype
GCA_000986055.1	13133	11	Influenza A virus AEngland82009H1N1	NA	Viruses; Riboviria; Orthornavirae; Negarnaviricota; Polyploviricotina; Insthoviricetes; Articulavirales; Orthomyxoviridae; Alphainfluenzavirus; Influenza A virus; H1N1 subtype
GCA_000986095.1	13133	11	Influenza A virus AEngland2512010H1N1	NA	Viruses; Riboviria; Orthornavirae; Negarnaviricota; Polyploviricotina; Insthoviricetes; Articulavirales; Orthomyxoviridae; Alphainfluenzavirus; Influenza A virus; H1N1 subtype
GCA_001013845.1	13133	11	Influenza A virus AEngland6872010H1N1	NA	Viruses; Riboviria; Orthornavirae; Negarnaviricota; Polyploviricotina; Insthoviricetes; Articulavirales; Orthomyxoviridae; Alphainfluenzavirus; Influenza A virus; H1N1 subtype

gca	genome.size	proteome.size	phage.name	strain	Taxonomy
GCA_000986275.1	13133	11	Influenza A virus AEngland4462010H1N1	NA	Viruses; Riboviria; Orthornavirae; Negarnaviricota; Polyploviricotina; Insthoviricetes; Articulavirales; Orthomyxoviridae; Alphainfluenzavirus; Influenza A virus; H1N1 subtype
GCA_001013865.1	13133	11	Influenza A virus AEngland107406882010H1N1	NA	Viruses; Riboviria; Orthornavirae; Negarnaviricota; Polyploviricotina; Insthoviricetes; Articulavirales; Orthomyxoviridae; Alphainfluenzavirus; Influenza A virus; H1N1 subtype
GCA_000986395.1	13133	11	Influenza A virus AEnglandWTSI17782009H1N1	NA	Viruses; Riboviria; Orthornavirae; Negarnaviricota; Polyploviricotina; Insthoviricetes; Articulavirales; Orthomyxoviridae; Alphainfluenzavirus; Influenza A virus; H1N1 subtype
GCA_001013825.1	13133	11	Influenza A virus AEngland051002972010H1N1	NA	Viruses; Riboviria; Orthornavirae; Negarnaviricota; Polyploviricotina; Insthoviricetes; Articulavirales; Orthomyxoviridae; Alphainfluenzavirus; Influenza A virus; H1N1 subtype
GCA_001013685.1	13133	11	Influenza A virus AEngland003807102010H1N1	NA	Viruses; Riboviria; Orthornavirae; Negarnaviricota; Polyploviricotina; Insthoviricetes; Articulavirales; Orthomyxoviridae; Alphainfluenzavirus; Influenza A virus; H1N1 subtype
GCA_001013645.1	13133	11	Influenza A virus AEngland050206622010H1N1	NA	Viruses; Riboviria; Orthornavirae; Negarnaviricota; Polyploviricotina; Insthoviricetes; Articulavirales; Orthomyxoviridae; Alphainfluenzavirus; Influenza A virus; H1N1 subtype
GCA_000986555.1	13133	11	Influenza A virus AEngland322010H1N1	NA	Viruses; Riboviria; Orthornavirae; Negarnaviricota; Polyploviricotina; Insthoviricetes; Articulavirales; Orthomyxoviridae; Alphainfluenzavirus; Influenza A virus; H1N1 subtype
GCA_001013925.1	13133	11	Influenza A virus AEngland7172010H1N1	NA	Viruses; Riboviria; Orthornavirae; Negarnaviricota; Polyploviricotina; Insthoviricetes; Articulavirales; Orthomyxoviridae; Alphainfluenzavirus; Influenza A virus; H1N1 subtype
GCA_000986475.1	13133	11	Influenza A virus AEngland050206652010H1N1	NA	Viruses; Riboviria; Orthornavirae; Negarnaviricota; Polyploviricotina; Insthoviricetes; Articulavirales; Orthomyxoviridae; Alphainfluenzavirus; Influenza A virus; H1N1 subtype
GCA_000986175.1	13133	11	Influenza A virus AEngland107407072011H1N1	NA	Viruses; Riboviria; Orthornavirae; Negarnaviricota; Polyploviricotina; Insthoviricetes; Articulavirales; Orthomyxoviridae; Alphainfluenzavirus; Influenza A virus; H1N1 subtype
GCA_000986495.1	13133	11	Influenza A virus AEngland6712010H1N1	NA	Viruses; Riboviria; Orthornavirae; Negarnaviricota; Polyploviricotina; Insthoviricetes; Articulavirales; Orthomyxoviridae; Alphainfluenzavirus; Influenza A virus; H1N1 subtype
GCA_000986535.1	13133	11	Influenza A virus AEngland107405792010H1N1	NA	Viruses; Riboviria; Orthornavirae; Negarnaviricota; Polyploviricotina; Insthoviricetes; Articulavirales; Orthomyxoviridae; Alphainfluenzavirus; Influenza A virus; H1N1 subtype
GCA_000986255.1	13133	11	Influenza A virus AEngland6762010H1N1	NA	Viruses; Riboviria; Orthornavirae; Negarnaviricota; Polyploviricotina; Insthoviricetes; Articulavirales; Orthomyxoviridae; Alphainfluenzavirus; Influenza A virus; H1N1 subtype
GCA_000986355.1	13133	11	Influenza A virus AEngland7012010H1N1	NA	Viruses; Riboviria; Orthornavirae; Negarnaviricota; Polyploviricotina; Insthoviricetes; Articulavirales; Orthomyxoviridae; Alphainfluenzavirus; Influenza A virus; H1N1 subtype
GCA_001013705.1	13133	11	Influenza A virus AEnglandWTSI18072010H1N1	NA	Viruses; Riboviria; Orthornavirae; Negarnaviricota; Polyploviricotina; Insthoviricetes; Articulavirales; Orthomyxoviridae; Alphainfluenzavirus; Influenza A virus; H1N1 subtype
GCA_001013725.1	13133	11	Influenza A virus AEngland112010H1N1	NA	Viruses; Riboviria; Orthornavirae; Negarnaviricota; Polyploviricotina; Insthoviricetes; Articulavirales; Orthomyxoviridae; Alphainfluenzavirus; Influenza A virus; H1N1 subtype
GCA_001013785.1	13133	11	Influenza A virus AEngland216806332010H1N1	NA	Viruses; Riboviria; Orthornavirae; Negarnaviricota; Polyploviricotina; Insthoviricetes; Articulavirales; Orthomyxoviridae; Alphainfluenzavirus; Influenza A virus; H1N1 subtype
GCA_000986135.1	13133	11	Influenza A virus AEngland6972010H1N1	NA	Viruses; Riboviria; Orthornavirae; Negarnaviricota; Polyploviricotina; Insthoviricetes; Articulavirales; Orthomyxoviridae; Alphainfluenzavirus; Influenza A virus; H1N1 subtype
GCA_001014165.1	13133	11	Influenza A virus AEnglandWTSI18262010H1N1	NA	Viruses; Riboviria; Orthornavirae; Negarnaviricota; Polyploviricotina; Insthoviricetes; Articulavirales; Orthomyxoviridae; Alphainfluenzavirus; Influenza A virus; H1N1 subtype
GCA_000986615.1	13133	11	Influenza A virus AEngland6732010H1N1	NA	Viruses; Riboviria; Orthornavirae; Negarnaviricota; Polyploviricotina; Insthoviricetes; Articulavirales; Orthomyxoviridae; Alphainfluenzavirus; Influenza A virus; H1N1 subtype
GCA_001014045.1	13133	11	Influenza A virus AEngland1322011H1N1	NA	Viruses; Riboviria; Orthornavirae; Negarnaviricota; Polyploviricotina; Insthoviricetes; Articulavirales; Orthomyxoviridae; Alphainfluenzavirus; Influenza A virus; H1N1 subtype
GCA_001014025.1	13133	11	Influenza A virus AEngland6922010H1N1	NA	Viruses; Riboviria; Orthornavirae; Negarnaviricota; Polyploviricotina; Insthoviricetes; Articulavirales; Orthomyxoviridae; Alphainfluenzavirus; Influenza A virus; H1N1 subtype
GCA_001014265.1	13133	11	Influenza A virus AEngland3772010H1N1	NA	Viruses; Riboviria; Orthornavirae; Negarnaviricota; Polyploviricotina; Insthoviricetes; Articulavirales; Orthomyxoviridae; Alphainfluenzavirus; Influenza A virus; H1N1 subtype
GCA_001014205.1	13133	11	Influenza A virus AEngland050206732010H1N1	NA	Viruses; Riboviria; Orthornavirae; Negarnaviricota; Polyploviricotina; Insthoviricetes; Articulavirales; Orthomyxoviridae; Alphainfluenzavirus; Influenza A virus; H1N1 subtype
GCA_001014065.1	13136	11	Influenza A virus AEngland003807092010H1N1	NA	Viruses; Riboviria; Orthornavirae; Negarnaviricota; Polyploviricotina; Insthoviricetes; Articulavirales; Orthomyxoviridae; Alphainfluenzavirus; Influenza A virus; H1N1 subtype
GCA_001014225.1	13133	11	Influenza A virus AEngland011800182010H1N1	NA	Viruses; Riboviria; Orthornavirae; Negarnaviricota; Polyploviricotina; Insthoviricetes; Articulavirales; Orthomyxoviridae; Alphainfluenzavirus; Influenza A virus; H1N1 subtype
GCA_001014105.1	13133	11	Influenza A virus AEngland049000532010H1N1	NA	Viruses; Riboviria; Orthornavirae; Negarnaviricota; Polyploviricotina; Insthoviricetes; Articulavirales; Orthomyxoviridae; Alphainfluenzavirus; Influenza A virus; H1N1 subtype
GCA_001014005.1	13133	11	Influenza A virus AEngland011800072010H1N1	NA	Viruses; Riboviria; Orthornavirae; Negarnaviricota; Polyploviricotina; Insthoviricetes; Articulavirales; Orthomyxoviridae; Alphainfluenzavirus; Influenza A virus; H1N1 subtype
GCA_001006865.1	13133	11	Influenza A virus AEngland107407042011H1N1	NA	Viruses; Riboviria; Orthornavirae; Negarnaviricota; Polyploviricotina; Insthoviricetes; Articulavirales; Orthomyxoviridae; Alphainfluenzavirus; Influenza A virus; H1N1 subtype
GCA_001014145.1	13133	11	Influenza A virus AEngland108207342010H1N1	NA	Viruses; Riboviria; Orthornavirae; Negarnaviricota; Polyploviricotina; Insthoviricetes; Articulavirales; Orthomyxoviridae; Alphainfluenzavirus; Influenza A virus; H1N1 subtype
GCA_001014125.1	13133	11	Influenza A virus AEngland115207592011H1N1	NA	Viruses; Riboviria; Orthornavirae; Negarnaviricota; Polyploviricotina; Insthoviricetes; Articulavirales; Orthomyxoviridae; Alphainfluenzavirus; Influenza A virus; H1N1 subtype
GCA_001014245.1	13133	11	Influenza A virus AEngland3932010H1N1	NA	Viruses; Riboviria; Orthornavirae; Negarnaviricota; Polyploviricotina; Insthoviricetes; Articulavirales; Orthomyxoviridae; Alphainfluenzavirus; Influenza A virus; H1N1 subtype
GCA_001006845.1	13133	11	Influenza A virus AEngland6772010H1N1	NA	Viruses; Riboviria; Orthornavirae; Negarnaviricota; Polyploviricotina; Insthoviricetes; Articulavirales; Orthomyxoviridae; Alphainfluenzavirus; Influenza A virus; H1N1 subtype
GCA_001014085.1	13133	11	Influenza A virus AEngland049800532010H1N1	NA	Viruses; Riboviria; Orthornavirae; Negarnaviricota; Polyploviricotina; Insthoviricetes; Articulavirales; Orthomyxoviridae; Alphainfluenzavirus; Influenza A virus; H1N1 subtype

gca	genome.size	proteome.size	phage.name	strain	Taxonomy
GCA_001006905.1	13133	11	Influenza A virus AEngland051604202010H1N1	NA	Viruses; Riboviria; Orthornavirae; Negarnaviricota; Polyploviricotina; Insthoviricetes; Articulavirales; Orthomyxoviridae; Alphainfluenzavirus; Influenza A virus; H1N1 subtype
GCA_001006685.1	13133	11	Influenza A virus AEngland7192010H1N1	NA	Viruses; Riboviria; Orthornavirae; Negarnaviricota; Polyploviricotina; Insthoviricetes; Articulavirales; Orthomyxoviridae; Alphainfluenzavirus; Influenza A virus; H1N1 subtype
GCA_001014185.1	13133	11	Influenza A virus AEngland107405612010H1N1	NA	Viruses; Riboviria; Orthornavirae; Negarnaviricota; Polyploviricotina; Insthoviricetes; Articulavirales; Orthomyxoviridae; Alphainfluenzavirus; Influenza A virus; H1N1 subtype
GCA_001039805.1	13133	11	Influenza A virus AEngland122406122010H1N1	NA	Viruses; Riboviria; Orthornavirae; Negarnaviricota; Polyploviricotina; Insthoviricetes; Articulavirales; Orthomyxoviridae; Alphainfluenzavirus; Influenza A virus; H1N1 subtype
GCA_001039825.1	13133	11	Influenza A virus AEngland107406812010H1N1	NA	Viruses; Riboviria; Orthornavirae; Negarnaviricota; Polyploviricotina; Insthoviricetes; Articulavirales; Orthomyxoviridae; Alphainfluenzavirus; Influenza A virus; H1N1 subtype
GCA_001006725.1	13133	11	Influenza A virus AEngland102010H1N1	NA	Viruses; Riboviria; Orthornavirae; Negarnaviricota; Polyploviricotina; Insthoviricetes; Articulavirales; Orthomyxoviridae; Alphainfluenzavirus; Influenza A virus; H1N1 subtype
GCA_001039845.1	13133	11	Influenza A virus AEnglandWTSI20602010H1N1	NA	Viruses; Riboviria; Orthornavirae; Negarnaviricota; Polyploviricotina; Insthoviricetes; Articulavirales; Orthomyxoviridae; Alphainfluenzavirus; Influenza A virus; H1N1 subtype
GCA_900004435.1	13133	11	Influenza A virus AEngland050806372010H1N1	NA	Viruses; Riboviria; Orthornavirae; Negarnaviricota; Polyploviricotina; Insthoviricetes; Articulavirales; Orthomyxoviridae; Alphainfluenzavirus; Influenza A virus; H1N1 subtype
GCA_900004775.1	13136	11	Influenza A virus AEngland015800032010H1N1	NA	Viruses; Riboviria; Orthornavirae; Negarnaviricota; Polyploviricotina; Insthoviricetes; Articulavirales; Orthomyxoviridae; Alphainfluenzavirus; Influenza A virus; H1N1 subtype
GCA_900005915.1	13133	11	Influenza A virus AEngland051402832010H1N1	NA	Viruses; Riboviria; Orthornavirae; Negarnaviricota; Polyploviricotina; Insthoviricetes; Articulavirales; Orthomyxoviridae; Alphainfluenzavirus; Influenza A virus; H1N1 subtype
GCA_900004805.1	13133	11	Influenza A virus AEnglandWTSI20672010H1N1	NA	Viruses; Riboviria; Orthornavirae; Negarnaviricota; Polyploviricotina; Insthoviricetes; Articulavirales; Orthomyxoviridae; Alphainfluenzavirus; Influenza A virus; H1N1 subtype
GCA_900006035.1	13133	11	Influenza A virus AEngland2832010H1N1	NA	Viruses; Riboviria; Orthornavirae; Negarnaviricota; Polyploviricotina; Insthoviricetes; Articulavirales; Orthomyxoviridae; Alphainfluenzavirus; Influenza A virus; H1N1 subtype
GCA_900004525.1	13133	11	Influenza A virus AEngland2132011H1N1	NA	Viruses; Riboviria; Orthornavirae; Negarnaviricota; Polyploviricotina; Insthoviricetes; Articulavirales; Orthomyxoviridae; Alphainfluenzavirus; Influenza A virus; H1N1 subtype
GCA_900005905.1	13133	11	Influenza A virus AEngland051401762010H1N1	NA	Viruses; Riboviria; Orthornavirae; Negarnaviricota; Polyploviricotina; Insthoviricetes; Articulavirales; Orthomyxoviridae; Alphainfluenzavirus; Influenza A virus; H1N1 subtype
GCA_001291425.1	13133	11	Influenza A virus AEngland3742010H1N1	NA	Viruses; Riboviria; Orthornavirae; Negarnaviricota; Polyploviricotina; Insthoviricetes; Articulavirales; Orthomyxoviridae; Alphainfluenzavirus; Influenza A virus; H1N1 subtype
GCA_001006745.1	13133	11	Influenza A virus AEngland107406852010H1N1	NA	Viruses; Riboviria; Orthornavirae; Negarnaviricota; Polyploviricotina; Insthoviricetes; Articulavirales; Orthomyxoviridae; Alphainfluenzavirus; Influenza A virus; H1N1 subtype
GCA_900005115.1	13133	11	Influenza A virus AEnglandWTSI17512010H1N1	NA	Viruses; Riboviria; Orthornavirae; Negarnaviricota; Polyploviricotina; Insthoviricetes; Articulavirales; Orthomyxoviridae; Alphainfluenzavirus; Influenza A virus; H1N1 subtype
GCA_900004425.1	13133	11	Influenza A virus AEngland051402812010H1N1	NA	Viruses; Riboviria; Orthornavirae; Negarnaviricota; Polyploviricotina; Insthoviricetes; Articulavirales; Orthomyxoviridae; Alphainfluenzavirus; Influenza A virus; H1N1 subtype
GCA_000986335.1	13133	11	Influenza A virus AEngland6752010H1N1	NA	Viruses; Riboviria; Orthornavirae; Negarnaviricota; Polyploviricotina; Insthoviricetes; Articulavirales; Orthomyxoviridae; Alphainfluenzavirus; Influenza A virus; H1N1 subtype
GCA_000986215.1	13133	11	Influenza A virus AEngland051002982010H1N1	NA	Viruses; Riboviria; Orthornavirae; Negarnaviricota; Polyploviricotina; Insthoviricetes; Articulavirales; Orthomyxoviridae; Alphainfluenzavirus; Influenza A virus; H1N1 subtype
GCA_001006825.1	13133	11	Influenza A virus AEngland3432010H1N1	NA	Viruses; Riboviria; Orthornavirae; Negarnaviricota; Polyploviricotina; Insthoviricetes; Articulavirales; Orthomyxoviridae; Alphainfluenzavirus; Influenza A virus; H1N1 subtype
GCA_900004675.1	13133	11	Influenza A virus AEngland015800052010H1N1	NA	Viruses; Riboviria; Orthornavirae; Negarnaviricota; Polyploviricotina; Insthoviricetes; Articulavirales; Orthomyxoviridae; Alphainfluenzavirus; Influenza A virus; H1N1 subtype
GCA_001291405.1	13133	11	Influenza A virus AEnglandWTSI17742010H1N1	NA	Viruses; Riboviria; Orthornavirae; Negarnaviricota; Polyploviricotina; Insthoviricetes; Articulavirales; Orthomyxoviridae; Alphainfluenzavirus; Influenza A virus; H1N1 subtype
GCA_900005085.1	13133	11	Influenza A virus AEngland6902010H1N1	NA	Viruses; Riboviria; Orthornavirae; Negarnaviricota; Polyploviricotina; Insthoviricetes; Articulavirales; Orthomyxoviridae; Alphainfluenzavirus; Influenza A virus; H1N1 subtype
GCA_900004505.1	13133	11	Influenza A virus AEngland4142010H1N1	NA	Viruses; Riboviria; Orthornavirae; Negarnaviricota; Polyploviricotina; Insthoviricetes; Articulavirales; Orthomyxoviridae; Alphainfluenzavirus; Influenza A virus; H1N1 subtype
GCA_000986115.1	13133	11	Influenza A virus AEngland051200032010H1N1	NA	Viruses; Riboviria; Orthornavirae; Negarnaviricota; Polyploviricotina; Insthoviricetes; Articulavirales; Orthomyxoviridae; Alphainfluenzavirus; Influenza A virus; H1N1 subtype
GCA_900004375.1	13133	11	Influenza A virus AEngland012207412010H1N1	NA	Viruses; Riboviria; Orthornavirae; Negarnaviricota; Polyploviricotina; Insthoviricetes; Articulavirales; Orthomyxoviridae; Alphainfluenzavirus; Influenza A virus; H1N1 subtype
GCA_001006885.1	13133	11	Influenza A virus AEnglandWTSI17812011H1N1	NA	Viruses; Riboviria; Orthornavirae; Negarnaviricota; Polyploviricotina; Insthoviricetes; Articulavirales; Orthomyxoviridae; Alphainfluenzavirus; Influenza A virus; H1N1 subtype
GCA_000986195.1	13133	11	Influenza A virus AEngland050401382010H1N1	NA	Viruses; Riboviria; Orthornavirae; Negarnaviricota; Polyploviricotina; Insthoviricetes; Articulavirales; Orthomyxoviridae; Alphainfluenzavirus; Influenza A virus; H1N1 subtype
GCA_900004465.1	13136	11	Influenza A virus AEnglandWTSI17832010H1N1	NA	Viruses; Riboviria; Orthornavirae; Negarnaviricota; Polyploviricotina; Insthoviricetes; Articulavirales; Orthomyxoviridae; Alphainfluenzavirus; Influenza A virus; H1N1 subtype
GCA_900004835.1	13133	11	Influenza A virus AEngland6652010H1N1	NA	Viruses; Riboviria; Orthornavirae; Negarnaviricota; Polyploviricotina; Insthoviricetes; Articulavirales; Orthomyxoviridae; Alphainfluenzavirus; Influenza A virus; H1N1 subtype
GCA_900004875.1	13133	11	Influenza A virus AEngland011800152010H1N1	NA	Viruses; Riboviria; Orthornavirae; Negarnaviricota; Polyploviricotina; Insthoviricetes; Articulavirales; Orthomyxoviridae; Alphainfluenzavirus; Influenza A virus; H1N1 subtype
GCA_900004575.1	13133	11	Influenza A virus AEngland107406232011H1N1	NA	Viruses; Riboviria; Orthornavirae; Negarnaviricota; Polyploviricotina; Insthoviricetes; Articulavirales; Orthomyxoviridae; Alphainfluenzavirus; Influenza A virus; H1N1 subtype
GCA_900004745.1	13133	11	Influenza A virus AEngland050808312010H1N1	NA	Viruses; Riboviria; Orthornavirae; Negarnaviricota; Polyploviricotina; Insthoviricetes; Articulavirales; Orthomyxoviridae; Alphainfluenzavirus; Influenza A virus; H1N1 subtype

gca	genome.size	proteome.size	phage.name	strain	Taxonomy
GCA_900004365.1	13133	11	Influenza A virus AEngland122406142011H1N1	NA	Viruses; Riboviria; Orthornavirae; Negarnaviricota; Polyploviricotina; Insthoviricetes; Articulavirales; Orthomyxoviridae; Alphainfluenzavirus; Influenza A virus; H1N1 subtype
GCA_900004725.1	13133	11	Influenza A virus AEngland107406582011H1N1	NA	Viruses; Riboviria; Orthornavirae; Negarnaviricota; Polyploviricotina; Insthoviricetes; Articulavirales; Orthomyxoviridae; Alphainfluenzavirus; Influenza A virus; H1N1 subtype
GCA_900004815.1	13133	11	Influenza A virus AEngland048605602010H1N1	NA	Viruses; Riboviria; Orthornavirae; Negarnaviricota; Polyploviricotina; Insthoviricetes; Articulavirales; Orthomyxoviridae; Alphainfluenzavirus; Influenza A virus; H1N1 subtype
GCA_900007785.1	13133	11	Influenza A virus AEngland011800172010H1N1	NA	Viruses; Riboviria; Orthornavirae; Negarnaviricota; Polyploviricotina; Insthoviricetes; Articulavirales; Orthomyxoviridae; Alphainfluenzavirus; Influenza A virus; H1N1 subtype
GCA_000986235.1	13133	11	Influenza A virus AEngland012207362010H1N1	NA	Viruses; Riboviria; Orthornavirae; Negarnaviricota; Polyploviricotina; Insthoviricetes; Articulavirales; Orthomyxoviridae; Alphainfluenzavirus; Influenza A virus; H1N1 subtype
GCA_000986295.1	13133	11	Influenza A virus AEngland2452010H1N1	NA	Viruses; Riboviria; Orthornavirae; Negarnaviricota; Polyploviricotina; Insthoviricetes; Articulavirales; Orthomyxoviridae; Alphainfluenzavirus; Influenza A virus; H1N1 subtype
GCA_000986155.1	13136	11	Influenza A virus AEngland011800162010H1N1	NA	Viruses; Riboviria; Orthornavirae; Negarnaviricota; Polyploviricotina; Insthoviricetes; Articulavirales; Orthomyxoviridae; Alphainfluenzavirus; Influenza A virus; H1N1 subtype
GCA_900004945.1	13133	11	Influenza A virus AEngland107406932011H1N1	NA	Viruses; Riboviria; Orthornavirae; Negarnaviricota; Polyploviricotina; Insthoviricetes; Articulavirales; Orthomyxoviridae; Alphainfluenzavirus; Influenza A virus; H1N1 subtype
GCA_001291385.1	13133	11	Influenza A virus AEngland107405812010H1N1	NA	Viruses; Riboviria; Orthornavirae; Negarnaviricota; Polyploviricotina; Insthoviricetes; Articulavirales; Orthomyxoviridae; Alphainfluenzavirus; Influenza A virus; H1N1 subtype
GCA_000986435.1	13133	11	Influenza A virus AEngland108207422010H1N1	NA	Viruses; Riboviria; Orthornavirae; Negarnaviricota; Polyploviricotina; Insthoviricetes; Articulavirales; Orthomyxoviridae; Alphainfluenzavirus; Influenza A virus; H1N1 subtype
GCA_900004475.1	13133	11	Influenza A virus AEngland049800552010H1N1	NA	Viruses; Riboviria; Orthornavirae; Negarnaviricota; Polyploviricotina; Insthoviricetes; Articulavirales; Orthomyxoviridae; Alphainfluenzavirus; Influenza A virus; H1N1 subtype
GCA_000986575.1	13133	11	Influenza A virus AEngland003807192010H1N1	NA	Viruses; Riboviria; Orthornavirae; Negarnaviricota; Polyploviricotina; Insthoviricetes; Articulavirales; Orthomyxoviridae; Alphainfluenzavirus; Influenza A virus; H1N1 subtype
GCA_900004595.1	13133	11	Influenza A virus AEngland012207402010H1N1	NA	Viruses; Riboviria; Orthornavirae; Negarnaviricota; Polyploviricotina; Insthoviricetes; Articulavirales; Orthomyxoviridae; Alphainfluenzavirus; Influenza A virus; H1N1 subtype
GCA_900004585.1	13133	11	Influenza A virus AEngland051401772010H1N1	NA	Viruses; Riboviria; Orthornavirae; Negarnaviricota; Polyploviricotina; Insthoviricetes; Articulavirales; Orthomyxoviridae; Alphainfluenzavirus; Influenza A virus; H1N1 subtype
GCA_900019215.1	13133	11	Influenza A virus AEngland051200132010H1N1	NA	Viruses; Riboviria; Orthornavirae; Negarnaviricota; Polyploviricotina; Insthoviricetes; Articulavirales; Orthomyxoviridae; Alphainfluenzavirus; Influenza A virus; H1N1 subtype
GCA_900004385.1	13133	11	Influenza A virus AEngland012206392009H1N1	NA	Viruses; Riboviria; Orthornavirae; Negarnaviricota; Polyploviricotina; Insthoviricetes; Articulavirales; Orthomyxoviridae; Alphainfluenzavirus; Influenza A virus; H1N1 subtype
GCA_900019225.1	13133	11	Influenza A virus AEngland051402162010H1N1	NA	Viruses; Riboviria; Orthornavirae; Negarnaviricota; Polyploviricotina; Insthoviricetes; Articulavirales; Orthomyxoviridae; Alphainfluenzavirus; Influenza A virus; H1N1 subtype
GCA_000986315.1	13133	11	Influenza A virus AEnglandWTSI18322010H1N1	NA	Viruses; Riboviria; Orthornavirae; Negarnaviricota; Polyploviricotina; Insthoviricetes; Articulavirales; Orthomyxoviridae; Alphainfluenzavirus; Influenza A virus; H1N1 subtype
GCA_900006335.1	13133	11	Influenza A virus AEngland6862010H1N1	NA	Viruses; Riboviria; Orthornavirae; Negarnaviricota; Polyploviricotina; Insthoviricetes; Articulavirales; Orthomyxoviridae; Alphainfluenzavirus; Influenza A virus; H1N1 subtype
GCA_900007775.1	13133	11	Influenza A virus AEngland107406912011H1N1	NA	Viruses; Riboviria; Orthornavirae; Negarnaviricota; Polyploviricotina; Insthoviricetes; Articulavirales; Orthomyxoviridae; Alphainfluenzavirus; Influenza A virus; H1N1 subtype
GCA_900004535.1	13133	11	Influenza A virus AEngland051408472010H1N1	NA	Viruses; Riboviria; Orthornavirae; Negarnaviricota; Polyploviricotina; Insthoviricetes; Articulavirales; Orthomyxoviridae; Alphainfluenzavirus; Influenza A virus; H1N1 subtype
GCA_900004335.1	13133	11	Influenza A virus AEngland7182010H1N1	NA	Viruses; Riboviria; Orthornavirae; Negarnaviricota; Polyploviricotina; Insthoviricetes; Articulavirales; Orthomyxoviridae; Alphainfluenzavirus; Influenza A virus; H1N1 subtype
GCA_900004455.1	13133	11	Influenza A virus AEngland051408562010H1N1	NA	Viruses; Riboviria; Orthornavirae; Negarnaviricota; Polyploviricotina; Insthoviricetes; Articulavirales; Orthomyxoviridae; Alphainfluenzavirus; Influenza A virus; H1N1 subtype
GCA_000986595.1	13136	11	Influenza A virus AEngland003807182010H1N1	NA	Viruses; Riboviria; Orthornavirae; Negarnaviricota; Polyploviricotina; Insthoviricetes; Articulavirales; Orthomyxoviridae; Alphainfluenzavirus; Influenza A virus; H1N1 subtype
GCA_900004555.1	13133	11	Influenza A virus AEngland6892010H1N1	NA	Viruses; Riboviria; Orthornavirae; Negarnaviricota; Polyploviricotina; Insthoviricetes; Articulavirales; Orthomyxoviridae; Alphainfluenzavirus; Influenza A virus; H1N1 subtype
GCA_001006925.1	13133	11	Influenza A virus AEnglandWTSI20512011H1N1	NA	Viruses; Riboviria; Orthornavirae; Negarnaviricota; Polyploviricotina; Insthoviricetes; Articulavirales; Orthomyxoviridae; Alphainfluenzavirus; Influenza A virus; H1N1 subtype
GCA_900004515.1	13133	11	Influenza A virus AEngland051003012010H1N1	NA	Viruses; Riboviria; Orthornavirae; Negarnaviricota; Polyploviricotina; Insthoviricetes; Articulavirales; Orthomyxoviridae; Alphainfluenzavirus; Influenza A virus; H1N1 subtype
GCA_900004345.1	13133	11	Influenza A virus AEngland6722010H1N1	NA	Viruses; Riboviria; Orthornavirae; Negarnaviricota; Polyploviricotina; Insthoviricetes; Articulavirales; Orthomyxoviridae; Alphainfluenzavirus; Influenza A virus; H1N1 subtype
GCA_900004495.1	13133	11	Influenza A virus AEngland107406902011H1N1	NA	Viruses; Riboviria; Orthornavirae; Negarnaviricota; Polyploviricotina; Insthoviricetes; Articulavirales; Orthomyxoviridae; Alphainfluenzavirus; Influenza A virus; H1N1 subtype
GCA_900004445.1	13133	11	Influenza A virus AEngland051006602010H1N1	NA	Viruses; Riboviria; Orthornavirae; Negarnaviricota; Polyploviricotina; Insthoviricetes; Articulavirales; Orthomyxoviridae; Alphainfluenzavirus; Influenza A virus; H1N1 subtype
GCA_900004485.1	13133	11	Influenza A virus AEngland049800452010H1N1	NA	Viruses; Riboviria; Orthornavirae; Negarnaviricota; Polyploviricotina; Insthoviricetes; Articulavirales; Orthomyxoviridae; Alphainfluenzavirus; Influenza A virus; H1N1 subtype
GCA_900009845.1	13133	11	Influenza A virus AEnglandWTSI20762010H1N1	NA	Viruses; Riboviria; Orthornavirae; Negarnaviricota; Polyploviricotina; Insthoviricetes; Articulavirales; Orthomyxoviridae; Alphainfluenzavirus; Influenza A virus; H1N1 subtype
GCA_900004765.1	13133	11	Influenza A virus AEngland011800112010H1N1	NA	Viruses; Riboviria; Orthornavirae; Negarnaviricota; Polyploviricotina; Insthoviricetes; Articulavirales; Orthomyxoviridae; Alphainfluenzavirus; Influenza A virus; H1N1 subtype
GCA_900004935.1	13133	11	Influenza A virus AEngland049806532010H1N1	NA	Viruses; Riboviria; Orthornavirae; Negarnaviricota; Polyploviricotina; Insthoviricetes; Articulavirales; Orthomyxoviridae; Alphainfluenzavirus; Influenza A virus; H1N1 subtype

gca	genome.size	proteome.size	phage.name	strain	Taxonomy
GCA_002605745.1	38261	56	Paracoccus phage Shpa	NA	Viruses; Duplodnaviria; Heunggongvirae; Uroviricota; Caudoviricetes; Caudovirales; Siphoviridae; Vhulanivirus; Paracoccus virus Shpa
GCA_002605765.1	103424	126	Gordonia phage GMA2	NA	Viruses; Duplodnaviria; Heunggongvirae; Uroviricota; Caudoviricetes; Caudovirales; Siphoviridae; unclassified Siphoviridae
GCA_001470695.1	77779	104	Gordonia phage GMA3	NA	Viruses; dsDNA viruses, no RNA stage; Caudovirales; Podoviridae; unclassified Podoviridae; Escherichia phage phi191 sensu lato
GCA_001736875.1	83324	115	Gordonia phage GMA6	NA	Viruses; dsDNA viruses, no RNA stage; Caudovirales; Siphoviridae; unclassified Siphoviridae
GCA_001470395.1	73419	101	Gordonia phage GMA7	NA	Viruses; dsDNA viruses, no RNA stage; Caudovirales; Podoviridae; unclassified Podoviridae; Escherichia phage phi191 sensu lato
GCA_001501235.1	53387	96	Mycobacterium phage Luchador	NA	Viruses; dsDNA viruses, no RNA stage; Caudovirales; Siphoviridae; unclassified Siphoviridae
GCA_001500575.1	72139	98	Mycobacterium phage Vincenzo	NA	Viruses; dsDNA viruses, no RNA stage; Caudovirales; Siphoviridae; unclassified Siphoviridae
GCA_001502615.1	49203	77	Mycobacterium phage Phayonce	NA	Viruses; dsDNA viruses, no RNA stage; Caudovirales; Siphoviridae; unclassified Siphoviridae
GCA_002598585.1	154553	232	Mycobacterium phage Momo	NA	Viruses; Duplodnaviria; Heunggongvirae; Uroviricota; Caudoviricetes; Caudovirales; Myoviridae; Bixzunavirus; Mycobacterium virus Bxz1
GCA_001501995.1	44576	65	Mycobacterium phage FlagStaff	NA	Viruses; dsDNA viruses, no RNA stage; Caudovirales; Siphoviridae; unclassified Siphoviridae
GCA_001501215.1	45161	67	Mycobacterium phage Cambiare	NA	Viruses; dsDNA viruses, no RNA stage; Caudovirales; Siphoviridae; unclassified Siphoviridae
GCA_001482955.1	70270	96	Mycobacterium phage Baee	NA	Viruses; dsDNA viruses, no RNA stage; Caudovirales; Siphoviridae; unclassified Siphoviridae
GCA_002605785.1	72109	99	Mycobacterium phage AlanGrant	NA	Viruses; Duplodnaviria; Heunggongvirae; Uroviricota; Caudoviricetes; Caudovirales; Siphoviridae; Bclasvirinae; Coopervirus; Mycobacterium virus Vincenzo
GCA_001500555.1	53455	97	Mycobacterium phage Nerujay	NA	Viruses; dsDNA viruses, no RNA stage; Caudovirales; Siphoviridae; unclassified Siphoviridae
GCA_001502595.1	44492	67	Mycobacterium phage MOOREtheMARYer	NA	Viruses; dsDNA viruses, no RNA stage; Caudovirales; Siphoviridae; unclassified Siphoviridae
GCA_002605805.1	68674	102	Mycobacterium phage OrangeOswald	NA	Viruses; Duplodnaviria; Heunggongvirae; Uroviricota; Caudoviricetes; Caudovirales; Siphoviridae; Bclasvirinae; Pipefishvirus; unclassified Pipefishvirus
GCA_001551045.1	75796	147	Mycobacterium phage Mindy	NA	Viruses; dsDNA viruses, no RNA stage; Caudovirales; Siphoviridae; unclassified Siphoviridae
GCA_002605825.1	68685	103	Mycobacterium phage Corofin	NA	Viruses; Duplodnaviria; Heunggongvirae; Uroviricota; Caudoviricetes; Caudovirales; Siphoviridae; Bclasvirinae; Pipefishvirus; unclassified Pipefishvirus
GCA_001501975.1	61081	100	Mycobacterium phage ShedlockHolmes	NA	Viruses; dsDNA viruses, no RNA stage; Caudovirales; Siphoviridae; unclassified Siphoviridae
GCA_001501195.1	41805	54	Acinetobacter phage Fri1	NA	Viruses; dsDNA viruses, no RNA stage; Caudovirales; Podoviridae; unclassified Podoviridae
GCA_001500935.1	41698	46	Klebsiella phage K5	NA	Viruses; dsDNA viruses, no RNA stage; Caudovirales; Podoviridae; unclassified Podoviridae
GCA_001470995.1	38084	48	Delftia phage IMEDE1	NA	Viruses; dsDNA viruses, no RNA stage; Caudovirales; Podoviridae; unclassified Podoviridae; Escherichia phage phi191 sensu lato
GCA_001500535.1	48689	82	Polaribacter phage P12002L	NA	Viruses; dsDNA viruses, no RNA stage; Caudovirales; Siphoviridae; unclassified Siphoviridae
GCA_001502575.1	49847	86	Polaribacter phage P12002S	NA	Viruses; dsDNA viruses, no RNA stage; Caudovirales; Siphoviridae; unclassified Siphoviridae
GCA_001501955.1	31075	37	Streptococcus phage APCM01	NA	Viruses; dsDNA viruses, no RNA stage; Caudovirales; Siphoviridae; unclassified Siphoviridae
GCA_001754705.1	41170	59	Enterococcus phage EcZZ2	NA	Viruses; dsDNA viruses, no RNA stage; Caudovirales; Siphoviridae; unclassified Siphoviridae
GCA_002622405.1	188427	361	Sinorhizobium phage phiM7	NA	Viruses; Duplodnaviria; Heunggongvirae; Uroviricota; Caudoviricetes; Caudovirales; Myoviridae; Emdodecavirus; Sinorhizobium virus M7
GCA_002605865.1	188047	361	Sinorhizobium phage phiM19	NA	Viruses; dsDNA viruses, no RNA stage; Caudovirales; Myoviridae; Tevenvirinae; T4virus; unclassified T4virus
GCA_001501175.1	206713	402	Sinorhizobium phage phiN3	NA	Viruses; dsDNA viruses, no RNA stage; Caudovirales; Myoviridae; Tevenvirinae; T4virus; unclassified T4virus
GCA_001500515.1	49037	72	Klebsiella phage KLPN1	NA	Viruses; dsDNA viruses, no RNA stage; Caudovirales; Siphoviridae; unclassified Siphoviridae
GCA_001745155.1	111325	148	Salmonella phage NR01	NA	Viruses; dsDNA viruses, no RNA stage; Caudovirales; Myoviridae; unclassified Myoviridae
GCA_002604245.1	167552	261	Escherichia phage PEC04	NA	Viruses; Duplodnaviria; Heunggongvirae; Uroviricota; Caudoviricetes; Caudovirales; Myoviridae; Tevenvirinae; Tequatrovirus; Escherichia virus HY01
GCA_008222785.1	114855	147	Salmonella phage SE24	NA	Viruses; Duplodnaviria; Heunggongvirae; Uroviricota; Caudoviricetes; Caudovirales; Demerecviridae; Markadamsvirinae; Epseptimavirus; unclassified Epseptimavirus
GCA_001502555.1	29733	43	Salmonella phage SEN1	NA	Viruses; dsDNA viruses, no RNA stage; Caudovirales; Myoviridae; Peduovirinae; P2virus; unclassified P2likevirus

gca	genome.size	proteome.size	phage.name	strain	Taxonomy
GCA_001470915.1	41338	55	Salmonella phage SEN22	NA	Viruses; dsDNA viruses, no RNA stage; Caudovirales; Podoviridae; unclassified Podoviridae; Escherichia phage phi191 sensu lato
GCA_001470135.1	40740	63	Salmonella phage SEN34	NA	Viruses; dsDNA viruses, no RNA stage; Caudovirales; Podoviridae; unclassified Podoviridae; Escherichia phage phi191 sensu lato
GCA_001501935.1	33509	47	Salmonella phage SEN4	NA	Viruses; dsDNA viruses, no RNA stage; Caudovirales; Myoviridae; Peduovirinae; P2virus; unclassified P2likevirus
GCA_001470675.1	33509	47	Salmonella phage SEN5	NA	Viruses; dsDNA viruses, no RNA stage; Caudovirales; Myoviridae; unclassified Myoviridae
GCA_002605925.1	35203	49	Salmonella phage SEN8	NA	Viruses; Duplodnaviria; Heunggongvirae; Uroviricota; Caudoviricetes; Caudovirales; Myoviridae; Peduovirinae; Felsduovirus; Salmonella virus SEN8
GCA_001884535.1	49979	82	Gordonia phage GAL1	NA	Viruses; dsDNA viruses, no RNA stage; Caudovirales; Siphoviridae; unclassified Siphoviridae
GCA_001737095.1	41207	68	Gordonia phage GMA1	NA	Viruses; dsDNA viruses, no RNA stage; Caudovirales; Siphoviridae; unclassified Siphoviridae
GCA_001736415.1	45537	68	Gordonia phage GMA4	NA	Viruses; dsDNA viruses, no RNA stage; Caudovirales; Siphoviridae; unclassified Siphoviridae
GCA_001736635.1	17562	28	Gordonia phage GMA5	NA	Viruses; dsDNA viruses, no RNA stage; Caudovirales; Siphoviridae; unclassified Siphoviridae
GCA_001736855.1	17727	26	Gordonia phage GRU3	NA	Viruses; dsDNA viruses, no RNA stage; Caudovirales; Siphoviridae; unclassified Siphoviridae
GCA_001470375.1	56982	86	Gordonia phage GTE6	NA	Viruses; dsDNA viruses, no RNA stage; Caudovirales; Podoviridae; unclassified Podoviridae; Escherichia phage phi191 sensu lato
GCA_001470895.1	67617	94	Gordonia phage GTE8	NA	Viruses; dsDNA viruses, no RNA stage; Caudovirales; Podoviridae; unclassified Podoviridae; Escherichia phage phi191 sensu lato
GCA_001737075.1	56212	84	Tsukamurella phage TPA4	NA	Viruses; dsDNA viruses, no RNA stage; Caudovirales; Siphoviridae; unclassified Siphoviridae
GCA_000855265.1	12481	4	Helminthosporium victoriae 145S virus	NA	Viruses; dsRNA viruses; Chrysoviridae; Chrysovirus
GCA_002605945.1	40551	51	Moraxella phage Mcat1	NA	Viruses; Duplodnaviria; Heunggongvirae; Uroviricota; Caudoviricetes; Caudovirales; Siphoviridae; unclassified Siphoviridae
GCA_002605965.1	24841	33	Moraxella phage Mcat10	NA	Viruses; Duplodnaviria; Heunggongvirae; Uroviricota; Caudoviricetes; Caudovirales; Siphoviridae; unclassified Siphoviridae
GCA_002605985.1	25085	33	Moraxella phage Mcat11	NA	Viruses; Duplodnaviria; Heunggongvirae; Uroviricota; Caudoviricetes; Caudovirales; Siphoviridae; unclassified Siphoviridae
GCA_002606005.1	26845	37	Moraxella phage Mcat12	NA	Viruses; Duplodnaviria; Heunggongvirae; Uroviricota; Caudoviricetes; Caudovirales; Siphoviridae; unclassified Siphoviridae
GCA_002606025.1	34664	44	Moraxella phage Mcat13	NA	Viruses; Duplodnaviria; Heunggongvirae; Uroviricota; Caudoviricetes; Caudovirales; Siphoviridae; unclassified Siphoviridae
GCA_002606045.1	17422	24	Moraxella phage Mcat14	NA	Viruses; Duplodnaviria; Heunggongvirae; Uroviricota; Caudoviricetes; Caudovirales; Siphoviridae; unclassified Siphoviridae
GCA_002606065.1	46083	42	Moraxella phage Mcat15	NA	Viruses; Duplodnaviria; Heunggongvirae; Uroviricota; Caudoviricetes; Caudovirales; Siphoviridae; unclassified Siphoviridae
GCA_002606085.1	46782	37	Moraxella phage Mcat16	NA	Viruses; Duplodnaviria; Heunggongvirae; Uroviricota; Caudoviricetes; Caudovirales; Siphoviridae; unclassified Siphoviridae
GCA_002606105.1	60303	51	Moraxella phage Mcat17	NA	Viruses; Duplodnaviria; Heunggongvirae; Uroviricota; Caudoviricetes; Caudovirales; Siphoviridae; unclassified Siphoviridae
GCA_002606125.1	51889	53	Moraxella phage Mcat18	NA	Viruses; Duplodnaviria; Heunggongvirae; Uroviricota; Caudoviricetes; Caudovirales; Siphoviridae; unclassified Siphoviridae
GCA_002606145.1	36471	41	Moraxella phage Mcat19	NA	Viruses; Duplodnaviria; Heunggongvirae; Uroviricota; Caudoviricetes; Caudovirales; Siphoviridae; unclassified Siphoviridae
GCA_002606165.1	40578	52	Moraxella phage Mcat2	NA	Viruses; Duplodnaviria; Heunggongvirae; Uroviricota; Caudoviricetes; Caudovirales; Siphoviridae; unclassified Siphoviridae
GCA_002606185.1	23629	36	Moraxella phage Mcat20	NA	Viruses; Duplodnaviria; Heunggongvirae; Uroviricota; Caudoviricetes; Caudovirales; Siphoviridae; unclassified Siphoviridae
GCA_002606205.1	23410	33	Moraxella phage Mcat21	NA	Viruses; Duplodnaviria; Heunggongvirae; Uroviricota; Caudoviricetes; Caudovirales; Siphoviridae; unclassified Siphoviridae
GCA_002606225.1	40408	61	Moraxella phage Mcat22	NA	Viruses; Duplodnaviria; Heunggongvirae; Uroviricota; Caudoviricetes; Caudovirales; Siphoviridae; unclassified Siphoviridae
GCA_002606245.1	34758	49	Moraxella phage Mcat23	NA	Viruses; Duplodnaviria; Heunggongvirae; Uroviricota; Caudoviricetes; Caudovirales; Siphoviridae; unclassified Siphoviridae
GCA_002606265.1	39290	58	Moraxella phage Mcat24	NA	Viruses; Duplodnaviria; Heunggongvirae; Uroviricota; Caudoviricetes; Caudovirales; Siphoviridae; unclassified Siphoviridae
GCA_002606285.1	41268	51	Moraxella phage Mcat25	NA	Viruses; Duplodnaviria; Heunggongvirae; Uroviricota; Caudoviricetes; Caudovirales; Siphoviridae; unclassified Siphoviridae
GCA_002606305.1	37967	51	Moraxella phage Mcat26	NA	Viruses; Duplodnaviria; Heunggongvirae; Uroviricota; Caudoviricetes; Caudovirales; Siphoviridae; unclassified Siphoviridae
GCA_002606325.1	41487	55	Moraxella phage Mcat27	NA	Viruses; Duplodnaviria; Heunggongvirae; Uroviricota; Caudoviricetes; Caudovirales; Siphoviridae; unclassified Siphoviridae

gca	genome.size	proteome.size	phage.name	strain	Taxonomy
GCA_002606345.1	54840	86	Moraxella phage Mcat28	NA	Viruses; Duplodnaviria; Heunggongvirae; Uroviricota; Caudoviricetes; Caudovirales; Siphoviridae; unclassified Siphoviridae
GCA_002606365.1	34549	53	Moraxella phage Mcat29	NA	Viruses; Duplodnaviria; Heunggongvirae; Uroviricota; Caudoviricetes; Caudovirales; Siphoviridae; unclassified Siphoviridae
GCA_002606385.1	31332	40	Moraxella phage Mcat3	NA	Viruses; Duplodnaviria; Heunggongvirae; Uroviricota; Caudoviricetes; Caudovirales; Siphoviridae; unclassified Siphoviridae
GCA_002606405.1	30974	47	Moraxella phage Mcat30	NA	Viruses; Duplodnaviria; Heunggongvirae; Uroviricota; Caudoviricetes; Caudovirales; Siphoviridae; unclassified Siphoviridae
GCA_002606425.1	30974	45	Moraxella phage Mcat31	NA	Viruses; Duplodnaviria; Heunggongvirae; Uroviricota; Caudoviricetes; Caudovirales; Siphoviridae; unclassified Siphoviridae
GCA_002606445.1	30958	45	Moraxella phage Mcat32	NA	Viruses; Duplodnaviria; Heunggongvirae; Uroviricota; Caudoviricetes; Caudovirales; Siphoviridae; unclassified Siphoviridae
GCA_002606465.1	42819	57	Moraxella phage Mcat4	NA	Viruses; Duplodnaviria; Heunggongvirae; Uroviricota; Caudoviricetes; Caudovirales; Siphoviridae; unclassified Siphoviridae
GCA_002606485.1	47875	68	Moraxella phage Mcat5	NA	Viruses; Duplodnaviria; Heunggongvirae; Uroviricota; Caudoviricetes; Caudovirales; Siphoviridae; unclassified Siphoviridae
GCA_002606505.1	54619	56	Moraxella phage Mcat6	NA	Viruses; Duplodnaviria; Heunggongvirae; Uroviricota; Caudoviricetes; Caudovirales; Siphoviridae; unclassified Siphoviridae
GCA_002606525.1	42027	47	Moraxella phage Mcat7	NA	Viruses; Duplodnaviria; Heunggongvirae; Uroviricota; Caudoviricetes; Caudovirales; Siphoviridae; unclassified Siphoviridae
GCA_002606545.1	40710	56	Moraxella phage Mcat8	NA	Viruses; Duplodnaviria; Heunggongvirae; Uroviricota; Caudoviricetes; Caudovirales; Siphoviridae; unclassified Siphoviridae
GCA_002606565.1	36406	42	Moraxella phage Mcat9	NA	Viruses; Duplodnaviria; Heunggongvirae; Uroviricota; Caudoviricetes; Caudovirales; Siphoviridae; unclassified Siphoviridae
GCA_000989155.1	7143	7	Human papillomavirus KC5	NA	Viruses; dsDNA viruses, no RNA stage; Papillomaviridae; Gammapapillomavirus; unclassified Gammapapillomavirus
GCA_001654285.1	8531	2	Antarctic picornalike virus 1	NA	Viruses; ssRNA viruses; ssRNA positive-strand viruses, no DNA stage; Picornavirales; environmental samples
GCA_001654385.1	9357	2	Antarctic picornalike virus 2	NA	Viruses; ssRNA viruses; ssRNA positive-strand viruses, no DNA stage; Picornavirales; environmental samples
GCA_001654185.1	8644	1	Antarctic picornalike virus 3	NA	Viruses; ssRNA viruses; ssRNA positive-strand viruses, no DNA stage; Picornavirales; environmental samples
GCA_001654105.1	9168	2	Antarctic picornalike virus 4	NA	Viruses; ssRNA viruses; ssRNA positive-strand viruses, no DNA stage; Picornavirales; environmental samples
GCA_001501155.1	32675	44	Escherichia phage pro147	NA	Viruses; dsDNA viruses, no RNA stage; Caudovirales; Myoviridae; Peduovirinae; P2virus; unclassified P2likevirus
GCA_001500495.1	29237	43	Escherichia phage pro483	NA	Viruses; dsDNA viruses, no RNA stage; Caudovirales; Myoviridae; Peduovirinae; P2virus; unclassified P2likevirus
GCA_013086615.1	12731	4	Cocle virus	GML244915	Viruses; Riboviria; Orthornavirae; Negarnaviricota; Polyploviricotina; Ellioviricetes; Bunyavirales; Phenuiviridae; Phlebovirus; Punta Toro phlebovirus
GCA_013086625.1	12531	4	Campana virus	VP334K	Viruses; Riboviria; Orthornavirae; Negarnaviricota; Polyploviricotina; Ellioviricetes; Bunyavirales; Peribunyaviridae; unclassified Peribunyaviridae
GCA_002827045.1	7227	7	Human papillomavirus 204	NA	Viruses; Monodnaviria; Shotokuvirae; Cossaviricota; Papovaviricetes; Zurhausenvirales; Papillomaviridae; Firstpapillomavirinae; Mupapillomavirus; Mupapillomavirus 3
GCA_006465865.1	207428	174	Ectromelia virus Naval	NA	Viruses; Varidnaviria; Bamfordvirae; Nucleocytoviricota; Pokkesviricetes; Chitovirales; Poxviridae; Chordopoxvirinae; Orthopoxvirus; Ectromelia virus
GCA_006465525.1	187723	185	Vaccinia virus WAU86881	NA	Viruses; Varidnaviria; Bamfordvirae; Nucleocytoviricota; Pokkesviricetes; Chitovirales; Poxviridae; Chordopoxvirinae; Orthopoxvirus; Vaccinia virus
GCA_003606015.1	170020	209	Enterobacteria phage ATK47	NA	Viruses; Duplodnaviria; Heunggongvirae; Uroviricota; Caudoviricetes; Caudovirales; Myoviridae; Tevenvirinae; Mosigvirus; unclassified Mosigvirus
GCA_003606035.1	169729	218	Enterobacteria phage ATK48	NA	Viruses; Duplodnaviria; Heunggongvirae; Uroviricota; Caudoviricetes; Caudovirales; Myoviridae; Tevenvirinae; Mosigvirus; unclassified Mosigvirus
GCA_003606075.1	167318	237	Enterobacteria phage Kha5h	NA	Viruses; Duplodnaviria; Heunggongvirae; Uroviricota; Caudoviricetes; Caudovirales; Myoviridae; Tevenvirinae; Tequatrovirus; unclassified Tequatrovirus
GCA_003606055.1	166514	237	Enterobacteria phage GiZh	NA	Viruses; Duplodnaviria; Heunggongvirae; Uroviricota; Caudoviricetes; Caudovirales; Myoviridae; Tevenvirinae; Tequatrovirus; unclassified Tequatrovirus
GCA_003606135.1	88016	78	Enterobacteria phage KhF3	NA	Viruses; Duplodnaviria; Heunggongvirae; Uroviricota; Caudoviricetes; Caudovirales; Myoviridae; Ounavirinae; Felixounavirus; unclassified Felixounavirus
GCA_003606155.1	89635	83	Enterobacteria phage XTG1	NA	Viruses; Duplodnaviria; Heunggongvirae; Uroviricota; Caudoviricetes; Caudovirales; Myoviridae; Ounavirinae; Felixounavirus; unclassified Felixounavirus
GCA_003605995.1	168496	235	Enterobacteria phage Aplg8	NA	Viruses; Duplodnaviria; Heunggongvirae; Uroviricota; Caudoviricetes; Caudovirales; Myoviridae; Tevenvirinae; Tequatrovirus; unclassified Tequatrovirus
GCA_003606095.1	88356	85	Enterobacteria phage KhF1	NA	Viruses; Duplodnaviria; Heunggongvirae; Uroviricota; Caudoviricetes; Caudovirales; Myoviridae; Ounavirinae; Felixounavirus; unclassified Felixounavirus
GCA_003606115.1	88309	87	Enterobacteria phage KhF2	NA	Viruses; Duplodnaviria; Heunggongvirae; Uroviricota; Caudoviricetes; Caudovirales; Myoviridae; Ounavirinae; Felixounavirus; unclassified Felixounavirus
GCA_002606585.1	168489	241	Stenotrophomonas phage vB_SmaSDLP_6	NA	Viruses; Duplodnaviria; Heunggongvirae; Uroviricota; Caudoviricetes; Caudovirales; Ackermannviridae; unclassified Ackermannviridae

gca	genome.size	proteome.size	phage.name	strain	Taxonomy
GCA_000954395.1	8041	5	Alfalfa latent virus	NA	Viruses; ssRNA viruses; ssRNA positive-strand viruses, no DNA stage; Tymovirales; Betaflexiviridae; Quinvirinae; Carlavirus; unclassified Carlavirus
GCA_017347745.1	456821	392	Carp edema virus	NA	Viruses; Varidnaviria; Bamfordvirae; Nucleocytoviricota; Pokkesviricetes; Chitovirales; Poxviridae; unclassified Poxviridae
GCA_002633235.1	158066	261	Vibrio phage RYC	D101	Viruses; Duplodnaviria; Heunggongvirae; Uroviricota; Caudoviricetes; Caudovirales; Myoviridae; unclassified Myoviridae
GCA_000858525.1	25325	11	Lymantria dispar cypovirus 14	NA	Viruses; Riboviria; Orthornavirae; Duplornaviricota; Resentoviricetes; Reovirales; Reoviridae; Spinareovirinae; Cypovirus; Cypovirus 14
GCA_000868905.1	4431	5	Cucumber leaf spot virus	NA	Viruses; ssRNA viruses; ssRNA positive-strand viruses, no DNA stage; Tombusviridae; Aureusvirus
GCA_001019975.1	12422	2	Sclerotinia sclerotiorum botybirnavirus 1	NA	Viruses; dsRNA viruses; unclassified dsRNA viruses
GCA_001019875.1	8865	1	Kilifi Virus	NA	Viruses; ssRNA viruses; ssRNA positive-strand viruses, no DNA stage; Picornavirales; unclassified Picornavirales
GCA_001020055.1	9108	1	Thika virus	NA	Viruses; unclassified viruses
GCA_001019775.1	9786	1	La Jolla virus	NA	Viruses; ssRNA viruses; ssRNA positive-strand viruses, no DNA stage; Picornavirales; Iflaviridae; unclassified Iflaviridae
GCA_002008635.1	152388	95	Kallithea virus	NA	Viruses; dsDNA viruses, no RNA stage; Nudiviridae; unclassified Nudiviridae
GCA_001551225.1	45633	61	Pectobacterium phage Peat1	NA	Viruses; dsDNA viruses, no RNA stage; Caudovirales; Podoviridae; unclassified Podoviridae
GCA_001019835.1	16080	11	Areca palm velarivirus 1	NA	Viruses; ssRNA viruses; ssRNA positive-strand viruses, no DNA stage; Closteroviridae; Velarivirus; unclassified Velarivirus
GCA_002606605.1	159514	202	Stenotrophomonas phage IMESM1	NA	Viruses; Duplodnaviria; Heunggongvirae; Uroviricota; Caudoviricetes; Caudovirales; Ackermannviridae; unclassified Ackermannviridae
GCA_001745815.1	29249	44	Propionibacterium phage BruceLethal	NA	Viruses; dsDNA viruses, no RNA stage; Caudovirales; Siphoviridae; unclassified Siphoviridae
GCA_001744495.1	29347	45	Propionibacterium phage Enoki	NA	Viruses; dsDNA viruses, no RNA stage; Caudovirales; Siphoviridae; unclassified Siphoviridae
GCA_001743815.1	29254	44	Propionibacterium phage Moyashi	NA	Viruses; dsDNA viruses, no RNA stage; Caudovirales; Siphoviridae; unclassified Siphoviridae
GCA_001745135.2	29338	43	Propionibacterium phage QueenBey	NA	Viruses; dsDNA viruses, no RNA stage; Caudovirales; Siphoviridae; unclassified Siphoviridae
GCA_001550665.1	94766	194	Salmonella phage 19	NA	Viruses; dsDNA viruses, no RNA stage; Caudovirales; Myoviridae; unclassified Myoviridae
GCA_002599425.1	41733	80	Salmonella phage 22	NA	Viruses; Duplodnaviria; Heunggongvirae; Uroviricota; Caudoviricetes; Caudovirales; Podoviridae; Lederbergvirus; Salmonella virus P22
GCA_002599405.1	41803	75	Salmonella phage 25	NA	Viruses; Duplodnaviria; Heunggongvirae; Uroviricota; Caudoviricetes; Caudovirales; Podoviridae; Lederbergvirus; Salmonella virus P22
GCA_002599385.1	41783	78	Salmonella phage 34	NA	Viruses; Duplodnaviria; Heunggongvirae; Uroviricota; Caudoviricetes; Caudovirales; Podoviridae; Lederbergvirus; Salmonella virus P22
GCA_002599365.1	55391	91	Salmonella phage 35	NA	Viruses; Duplodnaviria; Heunggongvirae; Uroviricota; Caudoviricetes; Caudovirales; Siphoviridae; Chivirus; unclassified Chivirus
GCA_001517035.1	60216	105	Salmonella phage 37	NA	Viruses; dsDNA viruses, no RNA stage; Caudovirales; Siphoviridae; unclassified Siphoviridae
GCA_001516975.1	156833	265	Salmonella phage 38	NA	Viruses; dsDNA viruses, no RNA stage; Caudovirales; Myoviridae; unclassified Myoviridae
GCA_001551565.1	131903	228	Pseudoalteromonas phage H101	NA	Viruses; dsDNA viruses, no RNA stage; Caudovirales; Myoviridae; unclassified Myoviridae
GCA_001745795.1	170770	269	Escherichia phage HY03	NA	Viruses; dsDNA viruses, no RNA stage; Caudovirales; Myoviridae; unclassified Myoviridae
GCA_001744475.1	50934	78	Klebsiella phage PKP126	NA	Viruses; dsDNA viruses, no RNA stage; Caudovirales; Siphoviridae; unclassified Siphoviridae
GCA_001743795.1	168758	203	Klebsiella phage PKO111	NA	Viruses; dsDNA viruses, no RNA stage; Caudovirales; Myoviridae; unclassified Myoviridae
GCA_004787495.1	223083	81	Mandrillus leucophaeus cytomegalovirus	NA	Viruses; Duplodnaviria; Heunggongvirae; Peploviricota; Herviviricetes; Herpesvirales; Herpesviridae; Betaherpesvirinae; Cytomegalovirus; Cercopithecine betaherpesvirus 5
GCA_001190715.1	28876	45	Propionibacterium phage Attacne	NA	Viruses; dsDNA viruses, no RNA stage; Caudovirales; Siphoviridae; unclassified Siphoviridae
GCA_002623025.1	29339	45	Propionibacterium phage Keiki	NA	Viruses; Duplodnaviria; Heunggongvirae; Uroviricota; Caudoviricetes; Caudovirales; Siphoviridae; Pahexavirus; Propionibacterium virus Keiki
GCA_001190295.1	29461	44	Propionibacterium phage Kubed	NA	Viruses; dsDNA viruses, no RNA stage; Caudovirales; Siphoviridae; unclassified Siphoviridae
GCA_001190435.1	29517	45	Propionibacterium phage Lauchelly	NA	Viruses; dsDNA viruses, no RNA stage; Caudovirales; Siphoviridae; unclassified Siphoviridae
GCA_001190575.1	29726	46	Propionibacterium phage MrAK	NA	Viruses; dsDNA viruses, no RNA stage; Caudovirales; Siphoviridae; unclassified Siphoviridae

gca	genome.size	proteome.size	phage.name	strain	Taxonomy
GCA_001190695.1	29506	45	Propionibacterium phage Ouroboros	NA	Viruses; dsDNA viruses, no RNA stage; Caudovirales; Siphoviridae; unclassified Siphoviridae
GCA_001190275.2	29324	44	Propionibacterium phage Pirate	NA	Viruses; dsDNA viruses, no RNA stage; Caudovirales; Siphoviridae; unclassified Siphoviridae
GCA_001190415.1	29347	45	Propionibacterium phage Procrass1	NA	Viruses; dsDNA viruses, no RNA stage; Caudovirales; Siphoviridae; unclassified Siphoviridae
GCA_001190735.1	29594	47	Propionibacterium phage SKKY	NA	Viruses; dsDNA viruses, no RNA stage; Caudovirales; Siphoviridae; unclassified Siphoviridae
GCA_001190315.1	29440	45	Propionibacterium phage Solid	NA	Viruses; dsDNA viruses, no RNA stage; Caudovirales; Siphoviridae; unclassified Siphoviridae
GCA_001190455.1	29330	46	Propionibacterium phage Stormborn	NA	Viruses; dsDNA viruses, no RNA stage; Caudovirales; Siphoviridae; unclassified Siphoviridae
GCA_001190085.1	29463	44	Propionibacterium phage Wizzo	NA	Viruses; dsDNA viruses, no RNA stage; Caudovirales; Siphoviridae; unclassified Siphoviridae
GCA_001551685.1	168771	272	Escherichia phage APCEc01	NA	Viruses; dsDNA viruses, no RNA stage; Caudovirales; Myoviridae; unclassified Myoviridae
GCA_002606685.1	103737	151	Escherichia phage phiAPCEc03	NA	Viruses; Duplodnaviria; Heunggongvirae; Uroviricota; Caudoviricetes; Caudovirales; Demerecviridae; Markadamsvirinae; Tequintavirus; Escherichia virus phiAPCEc03
GCA_002606705.1	135400	219	Escherichia phage APECc02	NA	Viruses; Duplodnaviria; Heunggongvirae; Uroviricota; Caudoviricetes; Caudovirales; Myoviridae; Vequintavirinae; Vequintavirus; Escherichia virus APECc02
GCA_001502975.1	4623	6	Parabacteroides phage YZ2015a	NA	Viruses; ssDNA viruses; Microviridae; unclassified Microviridae
GCA_001502355.1	5001	4	Parabacteroides phage YZ2015b	NA	Viruses; ssDNA viruses; Microviridae; unclassified Microviridae
GCA_001190615.1	4609	5	Gokushovirinae Bog1183_53	NA	Viruses; ssDNA viruses; Microviridae; Gokushovirinae; unclassified Gokushovirinae
GCA_001190195.1	4451	6	Microviridae Bog1249_12	NA	Viruses; ssDNA viruses; Microviridae; unclassified Microviridae
GCA_001190335.1	4815	5	Microviridae Bog5275_51	NA	Viruses; ssDNA viruses; Microviridae; unclassified Microviridae
GCA_001190475.1	4501	6	Gokushovirinae Bog5712_52	NA	Viruses; ssDNA viruses; Microviridae; Gokushovirinae; unclassified Gokushovirinae
GCA_001190595.1	4656	6	Gokushovirinae Bog8989_22	NA	Viruses; ssDNA viruses; Microviridae; Gokushovirinae; unclassified Gokushovirinae
GCA_001190555.1	4671	4	Microviridae Bog9017_22	NA	Viruses; ssDNA viruses; Microviridae; unclassified Microviridae
GCA_001190675.1	4366	5	Microviridae Fen2266_11	NA	Viruses; ssDNA viruses; Microviridae; unclassified Microviridae
GCA_001190255.1	4949	5	Microviridae Fen418_41	NA	Viruses; ssDNA viruses; Microviridae; unclassified Microviridae
GCA_001190395.1	4552	6	Microviridae Fen4707_41	NA	Viruses; ssDNA viruses; Microviridae; unclassified Microviridae
GCA_002606725.1	4451	5	Microviridae Fen51_42	NA	Viruses; Monodnaviria; Sangervirae; Phixviricota; Malgrandaviricetes; Petitvirales; Microviridae; unclassified Microviridae; Microviridae Bog1249_12
GCA_001190535.1	4624	7	Gokushovirinae Fen672_31	NA	Viruses; ssDNA viruses; Microviridae; Gokushovirinae; unclassified Gokushovirinae
GCA_001190655.1	4654	5	Microviridae Fen685_11	NA	Viruses; ssDNA viruses; Microviridae; unclassified Microviridae
GCA_001190235.1	4686	5	Microviridae Fen7786_21	NA	Viruses; ssDNA viruses; Microviridae; unclassified Microviridae
GCA_001190375.1	4631	6	Gokushovirinae Fen7875_21	NA	Viruses; ssDNA viruses; Microviridae; Gokushovirinae; unclassified Gokushovirinae
GCA_001190515.1	4424	5	Microviridae Fen7895_21	NA	Viruses; ssDNA viruses; Microviridae; unclassified Microviridae
GCA_001190635.1	4199	5	Microviridae Fen7918_21	NA	Viruses; ssDNA viruses; Microviridae; unclassified Microviridae
GCA_001190215.1	4453	5	Microviridae Fen7940_21	NA	Viruses; ssDNA viruses; Microviridae; unclassified Microviridae
GCA_000850245.1	24732	10	Lymantria dispar cypovirus 1	NA	Viruses; Riboviria; Orthornavirae; Duplornaviricota; Resentoviricetes; Reovirales; Reoviridae; Spinareovirinae; Cypovirus; Cypovirus 1
GCA_000839245.1	5325	6	Melon chlorotic leaf curl virus	NA	Viruses; ssDNA viruses; Geminiviridae; Begomovirus
GCA_001019955.1	15478	15	Pebjah virus	NA	Viruses; ssRNA viruses; ssRNA positive-strand viruses, no DNA stage; Nidovirales; Arteriviridae; Arterivirus
GCA_001308635.1	8496	1	Kobuvirus cattleKagoshima224KoV2015JPN	NA	Viruses; ssRNA viruses; ssRNA positive-strand viruses, no DNA stage; Picornavirales; Picornaviridae; Kobuvirus; unclassified Kobuvirus
GCA_001308755.1	8087	1	Kobuvirus cattleKagoshima122KoV2014JPN	NA	Viruses; ssRNA viruses; ssRNA positive-strand viruses, no DNA stage; Picornavirales; Picornaviridae; Kobuvirus; unclassified Kobuvirus

gca	genome.size	proteome.size	phage.name	strain	Taxonomy
GCA_001447065.1	63569	84	Escherichia phage PA2	NA	Viruses; dsDNA viruses, no RNA stage; Caudovirales; Podoviridae; unclassified Podoviridae
GCA_002622525.1	61292	90	Escherichia phage PA28	NA	Viruses; Duplodnaviria; Heunggongvirae; Uroviricota; Caudoviricetes; Caudovirales; Podoviridae; Sepvirinae; Traversvirus; Escherichia virus PA28
GCA_002606765.1	63584	85	Escherichia phage PA8	NA	Viruses; Duplodnaviria; Heunggongvirae; Uroviricota; Caudoviricetes; Caudovirales; Podoviridae; Sepvirinae; Oslovirus; Escherichia virus PA2
GCA_000901435.1	119082	132	Epinotia aporema granulovirus	NA	Viruses; dsDNA viruses, no RNA stage; Baculoviridae; Betabaculovirus; unclassified Betabaculovirus
GCA_001027375.1	7769	4	Sclerotinia sclerotiorum fusarivirus 1	JMTJ14	Viruses; ssRNA viruses; unclassified ssRNA viruses; Fusariviridae
GCA_000913595.1	9472	1	Human endogenous retrovirus K113	NA	Viruses; Riboviria; Pararnavirae; Artverviricota; Revtraviricetes; Ortervirales; Retroviridae; unclassified Retroviridae; Human endogenous retroviruses; Human endogenous retrovirus K
GCA_001030085.1	16715	4	Sclerotinia sclerotiorum megabirnavirus 1	NA	Viruses; unassigned viruses; Megabirnaviridae; Megabirnavirus; unclassified Megabirnavirus
GCA_001654265.1	5583	3	Victorian trout aquabirnavirus	NA	Viruses; dsRNA viruses; Birnaviridae; Aquabirnavirus; unclassified Aquabirnavirus
GCA_001029005.1	9246	1	Ampivirus A1	NEWT2013HUN	Viruses; ssRNA viruses; ssRNA positive-strand viruses, no DNA stage; Picornavirales; Picornaviridae; unclassified Picornaviridae
GCA_001028965.1	5181	2	Magnaporthe oryzae virus 3	QSP5	Viruses; dsRNA viruses; Totiviridae; Victorivirus; unclassified Victorivirus
GCA_002606785.1	60284	77	Thermobifida phage P1312	NA	Viruses; Duplodnaviria; Heunggongvirae; Uroviricota; Caudoviricetes; Caudovirales; Siphoviridae; unclassified Siphoviridae
GCA_001271295.1	6327	4	Dromedary astrovirus	NA	Viruses; ssRNA viruses; ssRNA positive-strand viruses, no DNA stage; Astroviridae; Mamastrovirus; unclassified Mamastrovirus
GCA_001502535.1	56826	105	Mycobacterium phage Kimberlium	NA	Viruses; dsDNA viruses, no RNA stage; Caudovirales; Siphoviridae; unclassified Siphoviridae
GCA_001645975.1	14334	16	Marbled eel polyomavirus	NA	Viruses; dsDNA viruses, no RNA stage; Polyomaviridae; unclassified Polyomaviridae
GCA_018594975.1	10793	3	Eriocheir sinensis Bunyalike virus	NA	Viruses; unclassified viruses
GCA_001551025.1	44496	61	Rhodobacter phage RcTitan	NA	Viruses; dsDNA viruses, no RNA stage; Caudovirales; unclassified Caudovirales
GCA_002623345.1	44194	61	Rhodobacter phage RcSpartan	NA	Viruses; Duplodnaviria; Heunggongvirae; Uroviricota; Caudoviricetes; Caudovirales; Siphoviridae; Titanvirus; Rhodobacter virus RcSpartan
GCA_001501915.1	36065	45	Rhodobacter phage RcRhea	NA	Viruses; dsDNA viruses, no RNA stage; Caudovirales; unclassified Caudovirales
GCA_002756615.1	35985	44	Rhodobacter phage RcCronus	NA	Viruses; Duplodnaviria; Heunggongvirae; Uroviricota; Caudoviricetes; Caudovirales; Siphoviridae; Cronusvirus; Rhodobacter virus RcCronus
GCA_002606805.1	39210	46	Yersinia phage phiYeF10	NA	Viruses; Duplodnaviria; Heunggongvirae; Uroviricota; Caudoviricetes; Caudovirales; Autographiviridae; Studiervirinae; Teetrevirus; Yersinia virus YeF10
GCA_001431875.1	8887	1	Posavirus 3	9584	Viruses; ssRNA viruses; ssRNA positive-strand viruses, no DNA stage; Picornavirales; unclassified Picornavirales
GCA_001399285.1	196300	269	Ostreococcus lucimarinus virus 2	NA	Viruses; dsDNA viruses, no RNA stage; Phycodnaviridae; Prasinovirus; unclassified Prasinovirus
GCA_001399225.1	182309	243	Ostreococcus lucimarinus virus 7	NA	Viruses; dsDNA viruses, no RNA stage; Phycodnaviridae; Prasinovirus; unclassified Prasinovirus
GCA_001399265.1	193301	252	Ostreococcus mediterraneus virus 1	NA	Viruses; dsDNA viruses, no RNA stage; Phycodnaviridae; Prasinovirus; unclassified Prasinovirus
GCA_001551125.1	62220	86	Achromobacter phage phiAxp2	NA	Viruses; dsDNA viruses, no RNA stage; Caudovirales; Siphoviridae; unclassified Siphoviridae
GCA_002211055.1	72825	80	Achromobacter phage phiAxp3	NA	Viruses; dsDNA viruses, no RNA stage; Caudovirales; Podoviridae; N4virus; unclassified N4likevirus
GCA_002831065.1	14098	4	Jonchet virus	B81CI2004	Viruses; Riboviria; Orthornavirae; Negarnaviricota; Polyploviricotina; Ellioviricetes; Bunyavirales; Phasmaviridae; Jonvirus; Jonchet jonvirus
GCA_002814355.1	12739	5	Ferak virus	C51CI2004	Viruses; Riboviria; Orthornavirae; Negarnaviricota; Polyploviricotina; Ellioviricetes; Bunyavirales; Phasmaviridae; Feravirus; Ferak feravirus
GCA_002817735.1	8255	4	Caucasus prunus virus	NA	Viruses; Riboviria; Orthornavirae; Kitrinoviricota; Alsuviricetes; Tymovirales; Betaflexiviridae; Trivirinae; Prunevirus
GCA_001550505.1	33399	56	Pseudoalteromonas phage Pq0	NA	Viruses; dsDNA viruses, no RNA stage; Caudovirales; Siphoviridae; unclassified Siphoviridae
GCA_002595225.1	41143	58	Brucella phage 02_141	NA	Viruses; Duplodnaviria; Heunggongvirae; Uroviricota; Caudoviricetes; Caudovirales; Podoviridae; Perisivirus; Brucella virus Tb
GCA_002595205.1	41143	58	Brucella phage 02_19	NA	Viruses; Duplodnaviria; Heunggongvirae; Uroviricota; Caudoviricetes; Caudovirales; Podoviridae; Perisivirus; Brucella virus Tb
GCA_002595505.1	41143	58	Brucella phage 1066_141	NA	Viruses; Duplodnaviria; Heunggongvirae; Uroviricota; Caudoviricetes; Caudovirales; Podoviridae; Perisivirus; Brucella virus Tb
GCA_002595485.1	41143	58	Brucella phage 1066_19	NA	Viruses; Duplodnaviria; Heunggongvirae; Uroviricota; Caudoviricetes; Caudovirales; Podoviridae; Perisivirus; Brucella virus Tb

gca	genome.size	proteome.size	phage.name	strain	Taxonomy
GCA_002595305.1	41143	58	Brucella phage 110_141	NA	Viruses; Duplodnaviria; Heunggongvirae; Uroviricota; Caudoviricetes; Caudovirales; Podoviridae; Perisivirus; Brucella virus Tb
GCA_002595285.1	41142	58	Brucella phage 110_19	NA	Viruses; Duplodnaviria; Heunggongvirae; Uroviricota; Caudoviricetes; Caudovirales; Podoviridae; Perisivirus; Brucella virus Tb
GCA_002595265.1	41142	58	Brucella phage 11sa_141	NA	Viruses; Duplodnaviria; Heunggongvirae; Uroviricota; Caudoviricetes; Caudovirales; Podoviridae; Perisivirus; Brucella virus Tb
GCA_002595245.1	41143	58	Brucella phage 11sa_19	NA	Viruses; Duplodnaviria; Heunggongvirae; Uroviricota; Caudoviricetes; Caudovirales; Podoviridae; Perisivirus; Brucella virus Tb
GCA_002595345.1	41143	58	Brucella phage 141_141	NA	Viruses; Duplodnaviria; Heunggongvirae; Uroviricota; Caudoviricetes; Caudovirales; Podoviridae; Perisivirus; Brucella virus Tb
GCA_002595325.1	41143	58	Brucella phage 141_19	NA	Viruses; Duplodnaviria; Heunggongvirae; Uroviricota; Caudoviricetes; Caudovirales; Podoviridae; Perisivirus; Brucella virus Tb
GCA_002595385.1	41142	57	Brucella phage 177_141	NA	Viruses; Duplodnaviria; Heunggongvirae; Uroviricota; Caudoviricetes; Caudovirales; Podoviridae; Perisivirus; Brucella virus Tb
GCA_002595365.1	41142	58	Brucella phage 177_19	NA	Viruses; Duplodnaviria; Heunggongvirae; Uroviricota; Caudoviricetes; Caudovirales; Podoviridae; Perisivirus; Brucella virus Tb
GCA_002595425.1	41143	58	Brucella phage 281_141	NA	Viruses; Duplodnaviria; Heunggongvirae; Uroviricota; Caudoviricetes; Caudovirales; Podoviridae; Perisivirus; Brucella virus Tb
GCA_002595405.1	41143	58	Brucella phage 281_19	NA	Viruses; Duplodnaviria; Heunggongvirae; Uroviricota; Caudoviricetes; Caudovirales; Podoviridae; Perisivirus; Brucella virus Tb
GCA_002595465.1	41142	58	Brucella phage 544_141	NA	Viruses; Duplodnaviria; Heunggongvirae; Uroviricota; Caudoviricetes; Caudovirales; Podoviridae; Perisivirus; Brucella virus Tb
GCA_002595445.1	41142	58	Brucella phage 544_19	NA	Viruses; Duplodnaviria; Heunggongvirae; Uroviricota; Caudoviricetes; Caudovirales; Podoviridae; Perisivirus; Brucella virus Tb
GCA_002595545.1	41142	58	Brucella phage Tb_141	NA	Viruses; Duplodnaviria; Heunggongvirae; Uroviricota; Caudoviricetes; Caudovirales; Podoviridae; Perisivirus; Brucella virus Tb
GCA_002595525.1	41142	58	Brucella phage Tbilisi	NA	Viruses; Duplodnaviria; Heunggongvirae; Uroviricota; Caudoviricetes; Caudovirales; Podoviridae; Perisivirus; Brucella virus Tb
GCA_002595585.1	41142	58	Brucella phage V_141	NA	Viruses; Duplodnaviria; Heunggongvirae; Uroviricota; Caudoviricetes; Caudovirales; Podoviridae; Perisivirus; Brucella virus Tb
GCA_002595565.1	41143	58	Brucella phage V_19	NA	Viruses; Duplodnaviria; Heunggongvirae; Uroviricota; Caudoviricetes; Caudovirales; Podoviridae; Perisivirus; Brucella virus Tb
GCA_001008535.1	226084	84	Papio ursinus cytomegalovirus	NA	Viruses; Duplodnaviria; Heunggongvirae; Peploviricota; Herviviricetes; Herpesvirales; Herpesviridae; Betaherpesvirinae; Cytomegalovirus; Cercopithecine betaherpesvirus 5
GCA_000884695.1	156344	157	Lymantria xylina nucleopolyhedrovirus	NA	Viruses; Baculoviridae; Alphabaculovirus
GCA_000930275.1	3892	5	Elderberry latent virus	NA	Viruses; ssRNA viruses; ssRNA positive-strand viruses, no DNA stage; Tombusviridae; Pelarspovirus
GCA_000863485.1	3883	6	Pelargonium line pattern virus	NA	Viruses; ssRNA viruses; ssRNA positive-strand viruses, no DNA stage; Tombusviridae; Pelarspovirus
GCA_000929215.1	3865	5	Pelargonium ringspot virus	NA	Viruses; ssRNA viruses; ssRNA positive-strand viruses, no DNA stage; Tombusviridae; Pelarspovirus
GCA_000853545.1	3904	6	Pelargonium chlorotic ring pattern virus	NA	Viruses; ssRNA viruses; ssRNA positive-strand viruses, no DNA stage; Tombusviridae; Pelarspovirus
GCA_001045385.1	4590	3	Bearded dragon parvovirus	2014	Viruses; ssDNA viruses; Parvoviridae; Parvovirinae; Dependoparvovirus; unclassified Dependovirus
GCA_001185005.1	22115	9	Lutzomyia reovirus 1	NA	Viruses; dsRNA viruses; Reoviridae; unclassified Reoviridae
GCA_001045285.1	2218	3	Gyrovirus GyV8	NA	Viruses; ssDNA viruses; Circoviridae; Gyrovirus; unclassified Gyrovirus
GCA_001045365.1	2903	2	Ustilaginoidea virens unassigned RNA virus HNND1	NA	Viruses; dsRNA viruses; unclassified dsRNA viruses
GCA_000871305.1	9730	1	Moroccan watermelon mosaic virus	MWMVTn	Viruses; ssRNA viruses; ssRNA positive-strand viruses, no DNA stage; Potyviridae; Potyvirus
GCA_000852485.1	6056	5	Tulip virus X	J	Viruses; ssRNA viruses; ssRNA positive-strand viruses, no DNA stage; Tymovirales; Alphaflexiviridae; Potexvirus
GCA_018580395.1	2952	6	Mulberry crinkleassociated virus	NA	Viruses; Monodnaviria; Shotokuvirae; Cressdnaviricota; Repensiviricetes; Geplafuvirales; Geminiviridae; Mulberry mosaic dwarf associated virus
GCA_001045265.1	7025	3	Astrovirus ErSZAL6HUN2011	NA	Viruses; ssRNA viruses; ssRNA positive-strand viruses, no DNA stage; Astroviridae; unclassified Astroviridae
GCA_001500915.1	41054	51	Klebsiella phage vB_KpnP_KpV289	NA	Viruses; dsDNA viruses, no RNA stage; Caudovirales; Podoviridae; unclassified Podoviridae
GCA_000850445.1	12655	2	Strawberry mottle virus	NA	Viruses; ssRNA viruses; ssRNA positive-strand viruses, no DNA stage; Picornavirales; Secoviridae; unassigned Secoviridae
GCA_000864725.1	7759	7	Human papillomavirus type 54	NA	Viruses; Monodnaviria; Shotokuvirae; Cossaviricota; Papovaviricetes; Zurhausenvirales; Papillomaviridae; Firstpapillomavirinae; Alphapapillomavirus; Alphapapillomavirus 13
GCA_003032605.1	10317	4	Tavallinen suomalainen mies virus	NA	Viruses; Riboviria; Orthornavirae; Negarnaviricota; Polyploviricotina; Ellioviricetes; Bunyavirales; Arenaviridae; Reptarenavirus; Ordinary reptarenavirus

gca	genome.size	proteome.size	phage.name	strain	Taxonomy
GCA_003032595.1	10220	4	University of Giessen virus	UGV1	Viruses; Riboviria; Orthornavirae; Negarnaviricota; Polyploviricotina; Ellioviricetes; Bunyavirales; Arenaviridae; Reptarenavirus; Giessen reptarenavirus
GCA_008796805.1	7129	1	Human rhinovirus A43	ATCC VR1153	Viruses; Riboviria; Orthornavirae; Pisuviricota; Pisoniviricetes; Picornavirales; Picornaviridae; Enterovirus; Rhinovirus A
GCA_008798055.1	7149	1	Human rhinovirus A46	NA	Viruses; Riboviria; Orthornavirae; Pisuviricota; Pisoniviricetes; Picornavirales; Picornaviridae; Enterovirus; Rhinovirus A
GCA_008796205.1	7221	1	Human rhinovirus B91	ATCC VR1292	Viruses; Riboviria; Orthornavirae; Pisuviricota; Pisoniviricetes; Picornavirales; Picornaviridae; Enterovirus; Rhinovirus B
GCA_008796185.1	7233	1	Human rhinovirus B92	ATCC VR1293	Viruses; Riboviria; Orthornavirae; Pisuviricota; Pisoniviricetes; Picornavirales; Picornaviridae; Enterovirus; Rhinovirus B
GCA_008797555.1	7215	1	Human rhinovirus B93	NA	Viruses; Riboviria; Orthornavirae; Pisuviricota; Pisoniviricetes; Picornavirales; Picornaviridae; Enterovirus; Rhinovirus B
GCA_008796285.1	7201	1	Human rhinovirus B84	ATCC VR1194	Viruses; Riboviria; Orthornavirae; Pisuviricota; Pisoniviricetes; Picornavirales; Picornaviridae; Enterovirus; Rhinovirus B
GCA_008798415.1	7224	1	Human rhinovirus B35	NA	Viruses; Riboviria; Orthornavirae; Pisuviricota; Pisoniviricetes; Picornavirales; Picornaviridae; Enterovirus; Rhinovirus B
GCA_008796145.1	7207	1	Human rhinovirus B97	ATCC VR1297	Viruses; Riboviria; Orthornavirae; Pisuviricota; Pisoniviricetes; Picornavirales; Picornaviridae; Enterovirus; Rhinovirus B
GCA_004129255.1	2197	4	Torque teno equus virus 1	NA	Viruses; Anelloviridae; Mutorquevirus
GCA_003726955.1	3800	5	Kirkovirus Equ1	NA	Viruses; unclassified viruses
GCA_002820045.1	1843	2	Cyclovirus Equ1	NA	Viruses; Monodnaviria; Shotokuvirae; Cressdnaviricota; Arfiviricetes; Cirlivirales; Circoviridae; Cyclovirus; Horse associated cyclovirus 1
GCA_013086925.1	4198	3	Picobirnavirus Equ3	NA	Viruses; Riboviria; Orthornavirae; Pisuviricota; Duplopiviricetes; Durnavirales; Picobirnaviridae; Picobirnavirus; unclassified Picobirnavirus
GCA_002826005.1	2109	1	Gemycircularvirus C1c	NA	Viruses; Monodnaviria; Shotokuvirae; Cressdnaviricota; Repensiviricetes; Geplafuvirales; Genomoviridae; Gemykibivirus; Human associated gemykibivirus 3
GCA_002606825.1	41882	40	Burkholderia phage BpAMP3	NA	Viruses; Duplodnaviria; Heunggongvirae; Uroviricota; Caudoviricetes; Caudovirales; Autographiviridae; Okabevirinae; Ampunavirus; unclassified Ampunavirus
GCA_001501135.1	170527	275	Escherichia phage QL01	NA	Viruses; Duplodnaviria; Heunggongvirae; Uroviricota; Caudoviricetes; Caudovirales; Myoviridae; Tevenvirinae; Dhakavirus; Escherichia virus QL01
GCA_002606845.1	157147	218	Bacillus phage PBC6	NA	Viruses; Duplodnaviria; Heunggongvirae; Uroviricota; Caudoviricetes; Caudovirales; Herelleviridae; Bastillevirinae; Tsarbombavirus; unclassified Tsarbombavirus
GCA_001500475.1	52392	100	Mycobacterium phage Smeadley	NA	Viruses; dsDNA viruses, no RNA stage; Caudovirales; Siphoviridae; L5virus; unclassified L5likevirus
GCA_002597745.1	141028	209	Staphylococcus phage IMESA119	NA	Viruses; Duplodnaviria; Heunggongvirae; Uroviricota; Caudoviricetes; Caudovirales; Herelleviridae; Twortvirinae; Kayvirus; Staphylococcus virus G1
GCA_002606885.1	74655	143	Mycobacterium phage NoSleep	NA	Viruses; Duplodnaviria; Heunggongvirae; Uroviricota; Caudoviricetes; Caudovirales; Siphoviridae; Kostyavirus; unclassified Kostyavirus
GCA_001502515.1	49158	86	Mycobacterium phage UnionJack	NA	Viruses; dsDNA viruses, no RNA stage; Caudovirales; Siphoviridae; unclassified Siphoviridae
GCA_001501895.1	49452	75	Streptomyces phage Amela	NA	Viruses; dsDNA viruses, no RNA stage; Caudovirales; Siphoviridae; unclassified Siphoviridae
GCA_002606905.1	49483	75	Streptomyces phage Verse	NA	Viruses; Duplodnaviria; Heunggongvirae; Uroviricota; Caudoviricetes; Caudovirales; Siphoviridae; Arquatrovirinae; Camvirus; Streptomyces virus Amela; Streptomyces phage Amela
GCA_002597725.1	139750	204	Staphylococcus phage IMESA118	NA	Viruses; Duplodnaviria; Heunggongvirae; Uroviricota; Caudoviricetes; Caudovirales; Herelleviridae; Twortvirinae; Kayvirus; Staphylococcus virus G1; Staphylococcus phage Team1
GCA_002756635.1	68515	101	Mycobacterium phage Phamished	NA	Viruses; Duplodnaviria; Heunggongvirae; Uroviricota; Caudoviricetes; Caudovirales; Siphoviridae; Bclasvirinae; Pegunavirus; Mycobacterium virus Soto
GCA_001470115.1	68227	100	Mycobacterium phage Apizium	NA	Viruses; dsDNA viruses, no RNA stage; Caudovirales; Siphoviridae; unclassified Siphoviridae; PG1-like phages
GCA_001502955.1	177688	263	Citrobacter phage IMECF2	NA	Viruses; dsDNA viruses, no RNA stage; Caudovirales; Myoviridae; Tevenvirinae; T4virus; unclassified T4virus
GCA_001501115.1	58338	106	Mycobacterium phage Ovechkin	NA	Viruses; dsDNA viruses, no RNA stage; Caudovirales; Siphoviridae; unclassified Siphoviridae
GCA_002606925.1	50364	76	Vibrio phage H188	NA	Viruses; Duplodnaviria; Heunggongvirae; Uroviricota; Caudoviricetes; Caudovirales; Myoviridae; unclassified Myoviridae
GCA_001500455.1	50113	75	Streptomyces phage Izzy	NA	Viruses; dsDNA viruses, no RNA stage; Caudovirales; Siphoviridae; unclassified Siphoviridae
GCA_001502495.1	50165	73	Streptomyces phage Lannister	NA	Viruses; dsDNA viruses, no RNA stage; Caudovirales; Siphoviridae; unclassified Siphoviridae
GCA_001502335.1	44222	61	Staphylococcus phage vB_SauS_phi2	NA	Viruses; dsDNA viruses, no RNA stage; Caudovirales; Siphoviridae; unclassified Siphoviridae
GCA_002606945.1	66158	92	Pseudomonas phage vB_PaeM_CEB_DP1	NA	Viruses; Duplodnaviria; Heunggongvirae; Uroviricota; Caudoviricetes; Caudovirales; Myoviridae; Pbunavirus; Pseudomonas virus CEBDP1
GCA_001501555.1	41449	46	Yersinia phage vB_YenP_ISAO8	NA	Viruses; dsDNA viruses, no RNA stage; Caudovirales; Podoviridae; unclassified Podoviridae

gca	genome.size	proteome.size	phage.name	strain	Taxonomy
GCA_002606965.1	80647	123	Bacillus phage PBC4	NA	Viruses; Duplodnaviria; Heunggongvirae; Uroviricota; Caudoviricetes; Caudovirales; Siphoviridae; unclassified Siphoviridae
GCA_002606985.1	168689	251	Bacillus phage PBC2	NA	Viruses; Duplodnaviria; Heunggongvirae; Uroviricota; Caudoviricetes; Caudovirales; Siphoviridae; unclassified Siphoviridae
GCA_002607005.1	56332	93	Bacillus phage PBC5	NA	Viruses; Duplodnaviria; Heunggongvirae; Uroviricota; Caudoviricetes; Caudovirales; Siphoviridae; Saundersvirus; unclassified Saundersvirus
GCA_001551205.1	34997	43	Erysipelothrix phage SE1	NA	Viruses; dsDNA viruses, no RNA stage; Caudovirales; Siphoviridae; unclassified Siphoviridae
GCA_002756655.1	41898	63	Staphylococcus phage 552	NA	Viruses; Duplodnaviria; Heunggongvirae; Uroviricota; Caudoviricetes; Caudovirales; Siphoviridae; Phietavirus; Staphylococcus virus 55
GCA_002756675.1	42309	65	Staphylococcus phage 553	NA	Viruses; Duplodnaviria; Heunggongvirae; Uroviricota; Caudoviricetes; Caudovirales; Siphoviridae; Phietavirus; Staphylococcus virus 55
GCA_001470215.1	62851	74	Shigella phage SsVASD	NA	Viruses; dsDNA viruses, no RNA stage; Caudovirales; Podoviridae; unclassified Podoviridae; Escherichia phage phi191 sensu lato
GCA_001500895.1	52643	101	Mycobacterium phage Barriga	NA	Viruses; dsDNA viruses, no RNA stage; Caudovirales; Siphoviridae; unclassified Siphoviridae
GCA_001502935.1	59416	117	Mycobacterium phage Florinda	NA	Viruses; dsDNA viruses, no RNA stage; Caudovirales; Siphoviridae; unclassified Siphoviridae
GCA_002607025.1	58456	112	Mycobacterium phage Girafales	NA	Viruses; Duplodnaviria; Heunggongvirae; Uroviricota; Caudoviricetes; Caudovirales; Siphoviridae; Cheoctovirus; unclassified Cheoctovirus
GCA_002756695.1	67277	91	Mycobacterium phage Godines	NA	Viruses; Duplodnaviria; Heunggongvirae; Uroviricota; Caudoviricetes; Caudovirales; Siphoviridae; Bclasvirinae; Rosebushvirus; Mycobacterium virus Godines
GCA_002601505.1	69377	106	Mycobacterium phage Madruga	NA	Viruses; Duplodnaviria; Heunggongvirae; Uroviricota; Caudoviricetes; Caudovirales; Siphoviridae; Patiencevirus; Mycobacterium virus Patience
GCA_001502315.1	51355	88	Mycobacterium phage Nhonho	NA	Viruses; dsDNA viruses, no RNA stage; Caudovirales; Siphoviridae; unclassified Siphoviridae
GCA_001470755.1	68367	99	Mycobacterium phage Pops	NA	Viruses; dsDNA viruses, no RNA stage; Caudovirales; Siphoviridae; unclassified Siphoviridae; PG1-like phages
GCA_001502395.1	58671	112	Mycobacterium phage Quico	NA	Viruses; dsDNA viruses, no RNA stage; Caudovirales; Siphoviridae; unclassified Siphoviridae
GCA_001501875.1	40024	50	Bacillus phage Pavlov	NA	Viruses; dsDNA viruses, no RNA stage; Caudovirales; Podoviridae; unclassified Podoviridae
GCA_002607045.1	40001	66	Bacillus phage Silence	NA	Viruses; Duplodnaviria; Heunggongvirae; Uroviricota; Caudoviricetes; Caudovirales; Siphoviridae; unclassified Siphoviridae
GCA_002607065.1	80297	122	Caulobacter phage Sansa	NA	Viruses; Duplodnaviria; Heunggongvirae; Uroviricota; Caudoviricetes; Caudovirales; Siphoviridae; Sansavirus; Caulobacter virus Sansa
GCA_002607085.1	85138	120	Caulobacter phage Seuss	NA	Viruses; Duplodnaviria; Heunggongvirae; Uroviricota; Caudoviricetes; Caudovirales; Siphoviridae; Seussvirus; Caulobacter virus Seuss
GCA_002149285.1	172733	303	Citrobacter phage Merlin	NA	Viruses; Duplodnaviria; Heunggongvirae; Uroviricota; Caudoviricetes; Caudovirales; Myoviridae; Tevenvirinae; Moonvirus; Citrobacter virus Merlin
GCA_002149565.1	90000	143	Citrobacter phage Michonne	NA	Viruses; dsDNA viruses, no RNA stage; Caudovirales; Myoviridae; Ounavirinae; Mooglevirus; unclassified Mooglevirus
GCA_002607105.1	135391	212	Escherichia phage Murica	NA	Viruses; Duplodnaviria; Heunggongvirae; Uroviricota; Caudoviricetes; Caudovirales; Myoviridae; Vequintavirinae; Vequintavirus; Escherichia virus Murica
GCA_002149185.1	176081	280	Klebsiella phage Matisse	NA	Viruses; Duplodnaviria; Heunggongvirae; Uroviricota; Caudoviricetes; Caudovirales; Myoviridae; Tevenvirinae; Slopekvirus; Klebsiella virus Matisse
GCA_002624505.1	176055	277	Klebsiella phage Miro	NA	Viruses; Duplodnaviria; Heunggongvirae; Uroviricota; Caudoviricetes; Caudovirales; Myoviridae; Tevenvirinae; Slopekvirus; Klebsiella virus Miro
GCA_001501095.1	48754	76	Klebsiella phage Sushi	NA	Viruses; dsDNA viruses, no RNA stage; Caudovirales; Siphoviridae; unclassified Siphoviridae
GCA_001461605.1	98392	125	Diatraea saccharalis granulovirus	Parana2009	Viruses; dsDNA viruses, no RNA stage; Baculoviridae; Betabaculovirus; unclassified Betabaculovirus
GCA_001308475.1	9855	1	Opsiphanes invirae iflavirus 1	NA	Viruses; ssRNA viruses; ssRNA positive-strand viruses, no DNA stage; Picornavirales; Iflaviridae; unclassified Iflaviridae
GCA_001308335.1	132831	145	Perigonia lusca single nucleopolyhedrovirus	NA	Viruses; dsDNA viruses, no RNA stage; Baculoviridae; unclassified Baculoviridae
GCA_002157455.1	105555	119	Urbanus proteus nucleopolyhedrovirus	NA	Viruses; dsDNA viruses, no RNA stage; Baculoviridae; unclassified Baculoviridae
GCA_001685325.1	4851	4	Chimpanzee faeces associated microphage 1	NA	Viruses; ssDNA viruses; Microviridae; unclassified Microviridae
GCA_001684845.1	5184	5	Chimpanzee faeces associated microphage 2	NA	Viruses; ssDNA viruses; Microviridae; unclassified Microviridae
GCA_001685285.1	5016	4	Chimpanzee faeces associated microphage 3	NA	Viruses; ssDNA viruses; Microviridae; unclassified Microviridae
GCA_001684725.1	3811	3	Chimpanzee faeces associated circular DNA virus 1	NA	Viruses; ssDNA viruses; Circoviridae; unclassified Circoviridae
GCA_001684485.1	2356	1	Chimpanzee faeces associated circular DNA molecule 1	NA	Viruses; unclassified viruses

gca	genome.size	proteome.size	phage.name	strain	Taxonomy
GCA_003159095.1	11431	2	Lychnis mottle virus	NA	Viruses; Riboviria; Orthornavirae; Pisuviricota; Pisoniviricetes; Picornavirales; unclassified Picornavirales
GCA_001551545.1	16944	4	Rosellinia necatrix megabirnavirus 2W8	NA	Viruses; unassigned viruses; Megabirnaviridae; Megabirnavirus; unclassified Megabirnavirus
GCA_000851765.1	16527	9	Grapevine rootstock stem lesion associated virus	NA	Viruses; ssRNA viruses; ssRNA positive-strand viruses, no DNA stage; Closteroviridae; Closterovirus; unclassified Closterovirus
GCA_004061695.1	2414	2	Asterias forbesi associated circular virus	NA	Viruses; Monodnaviria; Shotokuvirae; Cressdnaviricota; Arfiviricetes; Cirlivirales; Circoviridae; Circovirus; unclassified Circovirus
GCA_002605345.1	131326	181	Peptoclostridium phage phiCDIF1296T	DSM 1296	Viruses; Duplodnaviria; Heunggongvirae; Uroviricota; Caudoviricetes; Caudovirales; Siphoviridae; unclassified Siphoviridae
GCA_001316395.1	2744	6	Chilli leaf curl Ahmedabad virusIndia IndiaAhmedabad2014	NA	Viruses; ssDNA viruses; Geminiviridae; Begomovirus; unclassified Begomovirus
GCA_001292995.1	651523	523	Mollivirus sibericum	NA	Viruses; unclassified viruses
GCA_001501535.1	73420	113	Pseudomonas phage KPP21	NA	Viruses; dsDNA viruses, no RNA stage; Caudovirales; Podoviridae; N4virus; unclassified N4likevirus
GCA_000861105.1	7176	10	Orthohepevirus A	NA	Viruses; Riboviria; Orthornavirae; Kitrinoviricota; Alsuviricetes; Hepelivirales; Hepeviridae; Orthohepevirus
GCA_000915995.1	6654	3	Orthohepevirus B	NA	Viruses; Riboviria; Orthornavirae; Kitrinoviricota; Alsuviricetes; Hepelivirales; Hepeviridae; Orthohepevirus
GCA_002826465.1	6959	4	Orthohepevirus C	LAB350	Viruses; Riboviria; Orthornavirae; Kitrinoviricota; Alsuviricetes; Hepelivirales; Hepeviridae; Orthohepevirus
GCA_000898475.1	6796	3	Orthohepevirus D	NA	Viruses; ssRNA viruses; ssRNA positive-strand viruses, no DNA stage; Hepeviridae; Orthohepevirus
GCA_003957765.1	3296	2	Diatom colony associated dsRNA virus 1	NA	Viruses; Riboviria; Orthornavirae; Pisuviricota; Duplopiviricetes; Durnavirales; Picobirnaviridae; unclassified Picobirnaviridae
GCA_003956625.1	4026	2	Diatom colony associated dsRNA virus 2	NA	Viruses; Riboviria; dsRNA viruses; unclassified dsRNA viruses
GCA_004128275.1	4911	2	Diatom colony associated dsRNA virus 3	NA	Viruses; Riboviria; Orthornavirae; Duplornaviricota; Chrymotiviricetes; Ghabrivirales; Totiviridae; unclassified Totiviridae
GCA_004128295.1	4982	2	Diatom colony associated dsRNA virus 4 genome type A	NA	Viruses; Riboviria; Orthornavirae; Duplornaviricota; Chrymotiviricetes; Ghabrivirales; Totiviridae; unclassified Totiviridae
GCA_004128315.1	4979	2	Diatom colony associated dsRNA virus 4 genome type B	NA	Viruses; Riboviria; Orthornavirae; Duplornaviricota; Chrymotiviricetes; Ghabrivirales; Totiviridae; unclassified Totiviridae
GCA_004128335.1	5252	2	Diatom colony associated dsRNA virus 5	NA	Viruses; Riboviria; Orthornavirae; Duplornaviricota; Chrymotiviricetes; Ghabrivirales; Totiviridae; unclassified Totiviridae
GCA_004128355.1	4939	2	Diatom colony associated dsRNA virus 6	NA	Viruses; Riboviria; Orthornavirae; Duplornaviricota; Chrymotiviricetes; Ghabrivirales; Totiviridae; unclassified Totiviridae
GCA_004128375.1	5327	2	Diatom colony associated dsRNA virus 7	NA	Viruses; Riboviria; Orthornavirae; Duplornaviricota; Chrymotiviricetes; Ghabrivirales; Totiviridae; unclassified Totiviridae
GCA_004128395.1	4660	2	Diatom colony associated dsRNA virus 8	NA	Viruses; Riboviria; Orthornavirae; Duplornaviricota; Chrymotiviricetes; Ghabrivirales; Totiviridae; unclassified Totiviridae
GCA_004128415.1	4844	2	Diatom colony associated dsRNA virus 9 genome type A	NA	Viruses; Riboviria; Orthornavirae; Duplornaviricota; Chrymotiviricetes; Ghabrivirales; Totiviridae; unclassified Totiviridae
GCA_004128435.1	5082	2	Diatom colony associated dsRNA virus 10	NA	Viruses; Riboviria; Orthornavirae; Duplornaviricota; Chrymotiviricetes; Ghabrivirales; Totiviridae; unclassified Totiviridae
GCA_004128455.1	5160	2	Diatom colony associated dsRNA virus 11	NA	Viruses; Riboviria; Orthornavirae; Duplornaviricota; Chrymotiviricetes; Ghabrivirales; Totiviridae; unclassified Totiviridae
GCA_004128475.1	5941	2	Diatom colony associated dsRNA virus 12	NA	Viruses; Riboviria; Orthornavirae; Duplornaviricota; Chrymotiviricetes; Ghabrivirales; Totiviridae; unclassified Totiviridae
GCA_004128495.1	4671	2	Diatom colony associated dsRNA virus 13	NA	Viruses; Riboviria; Orthornavirae; Duplornaviricota; Chrymotiviricetes; Ghabrivirales; Totiviridae; unclassified Totiviridae
GCA_004106585.1	3066	2	Diatom colony associated dsRNA virus 14	NA	Viruses; Riboviria; Orthornavirae; Pisuviricota; Duplopiviricetes; Durnavirales; Partitiviridae; unclassified Partitiviridae
GCA_004128515.1	12940	1	Diatom colony associated dsRNA virus 15	NA	Viruses; Riboviria; Orthornavirae; Kitrinoviricota; Alsuviricetes; Martellivirales; Endornaviridae; unclassified Endornaviridae
GCA_003726135.1	6635	2	Diatom colony associated dsRNA virus 16	NA	Viruses; unclassified viruses
GCA_003956605.1	5907	2	Diatom colony associated dsRNA virus 17 genome type A	NA	Viruses; Riboviria; dsRNA viruses; unclassified dsRNA viruses
GCA_003956585.1	5909	2	Diatom colony associated dsRNA virus 17 genome type B	NA	Viruses; Riboviria; dsRNA viruses; unclassified dsRNA viruses
GCA_004128535.1	11413	1	Diatom colony associated ssRNA virus 1	NA	Viruses; Riboviria; Orthornavirae; Kitrinoviricota; Flasuviricetes; Amarillovirales; Flaviviridae; unclassified Flaviviridae
GCA_004128555.1	4586	1	Diatom colony associated ssRNA virus 2	NA	Viruses; Riboviria; Orthornavirae; Lenarviricota; Amabiliviricetes; Wolframvirales; Narnaviridae; unclassified Narnaviridae
GCA_001184805.1	7596	2	Rhizoctonia solani RNA virus HN008	NA	Viruses; unclassified viruses

gca	genome.size	proteome.size	phage.name	strain	Taxonomy
GCA_002831265.1	12102	3	Bimiti virus	TRVL 8362	Viruses; Riboviria; Orthornavirae; Negarnaviricota; Polyploviricotina; Ellioviricetes; Bunyavirales; Peribunyaviridae; Orthobunyavirus; Bimiti orthobunyavirus
GCA_002831305.1	12024	3	Catu virus	BeH 151	Viruses; Riboviria; Orthornavirae; Negarnaviricota; Polyploviricotina; Ellioviricetes; Bunyavirales; Peribunyaviridae; Orthobunyavirus; Catu orthobunyavirus
GCA_002831205.1	11967	3	Lukuni virus	TRVL 10076	Viruses; Riboviria; Orthornavirae; Negarnaviricota; Polyploviricotina; Ellioviricetes; Bunyavirales; Peribunyaviridae; Orthobunyavirus; Anopheles A orthobunyavirus
GCA_009731915.1	12138	3	Moju virus	BeAr 12590	Viruses; Riboviria; Orthornavirae; Negarnaviricota; Polyploviricotina; Ellioviricetes; Bunyavirales; Peribunyaviridae; Orthobunyavirus; Guama orthobunyavirus
GCA_009731935.1	12220	3	Matruh virus	An 104761	Viruses; Riboviria; Orthornavirae; Negarnaviricota; Polyploviricotina; Ellioviricetes; Bunyavirales; Peribunyaviridae; Orthobunyavirus; Tete orthobunyavirus
GCA_004790655.1	12251	4	Witwatersrand virus	SAAr 1062	Viruses; Riboviria; Orthornavirae; Negarnaviricota; Polyploviricotina; Ellioviricetes; Bunyavirales; Peribunyaviridae; Orthobunyavirus; Witwatersrand orthobunyavirus
GCA_002831365.1	12123	3	Guama virus	BeAn 277	Viruses; Riboviria; Orthornavirae; Negarnaviricota; Polyploviricotina; Ellioviricetes; Bunyavirales; Peribunyaviridae; Orthobunyavirus; Guama orthobunyavirus
GCA_004062875.1	2063	3	Fox circovirus	NA	Viruses; Monodnaviria; Shotokuvirae; Cressdnaviricota; Arfiviricetes; Cirlivirales; Circoviridae; Circovirus; Canine circovirus
GCA_001745395.1	16072	5	Polygonum ringspot virus	NA	Viruses; ssRNA viruses; ssRNA negative-strand viruses; Bunyaviridae; Tospovirus; Polygonum ringspot tospovirus
GCA_001184885.1	17899	12	Chobar Gorge virus	NA	Viruses; dsRNA viruses; Reoviridae; Sedoreovirinae; Orbivirus
GCA_001184825.1	11804	4	Fusarium oxysporum f sp dianthi mycovirus 1	NA	Viruses; Riboviria; Orthornavirae; Duplornaviricota; Chrymotiviricetes; Ghabrivirales; Chrysoviridae; Betachrysovirus; Fusarium oxysporum chrysovirus 2
GCA_002829205.1	10308	2	Zucchini shoestring virus	NA	Viruses; Riboviria; Orthornavirae; Pisuviricota; Stelpaviricetes; Patatavirales; Potyviridae; Potyvirus
GCA_000853085.1	8146	5	Sugarcane striate mosaicassociated virus	NA	Viruses; ssRNA viruses; ssRNA positive-strand viruses, no DNA stage; Tymovirales; Betaflexiviridae; unassigned Betaflexiviridae
GCA_002117675.1	12008	3	Bruges virus	BEVieuxGenappeTE20131	Viruses; Riboviria; Orthornavirae; Negarnaviricota; Polyploviricotina; Ellioviricetes; Bunyavirales; Hantaviridae; Mammantavirinae; Orthohantavirus; Bruges orthohantavirus
GCA_002830605.1	4772	6	Lettuce necrotic stunt virus	NA	Viruses; Riboviria; Orthornavirae; Kitrinoviricota; Tolucaviricetes; Tolivirales; Tombusviridae; Procedovirinae; Tombusvirus; Moroccan pepper virus
GCA_001550645.1	75339	142	Mycobacterium phage Dusk	NA	Viruses; dsDNA viruses, no RNA stage; Caudovirales; Siphoviridae; Cjw1virus; unclassified Cjwunalikevirus
GCA_002756715.1	53746	84	Mycobacterium phage Kinbote	NA	Viruses; Duplodnaviria; Heunggongvirae; Uroviricota; Caudoviricetes; Caudovirales; Siphoviridae; Gilesvirus; Mycobacterium virus Giles
GCA_001500435.1	72790	139	Mycobacterium phage NelitzaMV	NA	Viruses; dsDNA viruses, no RNA stage; Caudovirales; Siphoviridae; unclassified Siphoviridae
GCA_001184865.1	5318	8	Rattus norvegicus polyomavirus 1	NA	Viruses; dsDNA viruses, no RNA stage; Polyomaviridae; unclassified Polyomaviridae
GCA_008800195.1	8821	1	Equine rhinitis B virus 2	P31375	Viruses; Riboviria; Orthornavirae; Pisuviricota; Pisoniviricetes; Picornavirales; Picornaviridae; Erbovirus; Erbovirus A
GCA_004129235.1	5068	13	Sus scrofa polyomavirus 1	NA	Viruses; Monodnaviria; Shotokuvirae; Cossaviricota; Papovaviricetes; Sepolyvirales; Polyomaviridae; unclassified Polyomaviridae
GCA_001271235.1	241564	210	Salmon gill poxvirus	NA	Viruses; dsDNA viruses, no RNA stage; Poxviridae; unclassified Poxviridae
GCA_000891455.1	9917	1	Apium virus Y	NA	Viruses; ssRNA viruses; ssRNA positive-strand viruses, no DNA stage; Potyviridae; Potyvirus
GCA_001190355.1	6258	3	Bee Maculalike virus	NA	Viruses; ssRNA viruses; ssRNA positive-strand viruses, no DNA stage; Tymovirales; Tymoviridae; unclassified Tymoviridae
GCA_001188625.1	6169	2	Varroa Tymolike virus	NA	Viruses; ssRNA viruses; ssRNA positive-strand viruses, no DNA stage; Tymovirales; Tymoviridae; unclassified Tymoviridae
GCA_003179695.1	7137	6	human papillomavirus 200	NA	Viruses; Monodnaviria; Shotokuvirae; Cossaviricota; Papovaviricetes; Zurhausenvirales; Papillomaviridae; Firstpapillomavirinae; Gammapapillomavirus; Gammapapillomavirus 2
GCA_001184845.1	7291	7	Human papillomavirus type 201	NA	Viruses; dsDNA viruses, no RNA stage; Papillomaviridae; Gammapapillomavirus; unclassified Gammapapillomavirus
GCA_003179715.1	7344	7	Human papillomavirus 202	NA	Viruses; Monodnaviria; Shotokuvirae; Cossaviricota; Papovaviricetes; Zurhausenvirales; Papillomaviridae; Firstpapillomavirinae; Gammapapillomavirus; Gammapapillomavirus 11
GCA_002623245.1	63513	76	Roseobacter phage RDJL Phi 2	NA	Viruses; Duplodnaviria; Heunggongvirae; Uroviricota; Caudoviricetes; Caudovirales; Siphoviridae; Xiamenvirus; Roseobacter virus RDJL2
GCA_002624485.1	175814	278	Enterobacter phage phiEap3	NA	Viruses; Duplodnaviria; Heunggongvirae; Uroviricota; Caudoviricetes; Caudovirales; Myoviridae; Tevenvirinae; Slopekvirus; Enterobacter virus Eap3
GCA_001470535.1	149732	270	Cronobacter phage PBES 02	NA	Viruses; dsDNA viruses, no RNA stage; Caudovirales; Myoviridae; unclassified Myoviridae
GCA_002988055.1	5228	2	Beauveria bassiana victorivirus 1	NA	Viruses; Riboviria; Orthornavirae; Duplornaviricota; Chrymotiviricetes; Ghabrivirales; Totiviridae; Victorivirus
GCA_002607125.1	44539	55	Pectobacterium phage PPWS1	NA	Viruses; Duplodnaviria; Heunggongvirae; Uroviricota; Caudoviricetes; Caudovirales; Autographiviridae; Corkvirinae; Kotilavirus; Pectobacterium virus PPWS1
GCA_002607145.1	7273	12	Ralstonia phage RSSTH1	NA	Viruses; Monodnaviria; Loebvirae; Hofneiviricota; Faserviricetes; Tubulavirales; Inoviridae; unclassified Inoviridae

gca	genome.size	proteome.size	phage.name	strain	Taxonomy
GCA_001685305.1	3206	2	Pseudogymnoascus destructans partitiviruspa	NA	Viruses; dsRNA viruses; Partitiviridae; unclassified Partitiviridae
GCA_001590055.1	1217	1	Lake Sarahassociated circular molecule 1	NA	Viruses; unclassified viruses
GCA_001589735.1	1693	1	Lake Sarahassociated circular molecule 10	NA	Viruses; unclassified viruses
GCA_001589415.1	1123	1	Lake Sarahassociated circular molecule 11	NA	Viruses; unclassified viruses
GCA_001590335.1	1131	1	Lake Sarahassociated circular molecule 2	NA	Viruses; unclassified viruses
GCA_001590035.1	1082	1	Lake Sarahassociated circular molecule 3	NA	Viruses; unclassified viruses
GCA_001589715.1	838	1	Lake Sarahassociated circular molecule 4	NA	Viruses; unclassified viruses
GCA_001589395.1	1045	1	Lake Sarahassociated circular molecule 5	NA	Viruses; unclassified viruses
GCA_001590315.1	1113	1	Lake Sarahassociated circular molecule 6	NA	Viruses; unclassified viruses
GCA_001590015.1	1096	1	Lake Sarahassociated circular molecule 7	NA	Viruses; unclassified viruses
GCA_001589695.1	1182	1	Lake Sarahassociated circular molecule 8	NA	Viruses; unclassified viruses
GCA_001589375.1	1137	1	Lake Sarahassociated circular molecule 9	NA	Viruses; unclassified viruses
GCA_001590495.1	2862	2	Lake Sarahassociated circular virus1	NA	Viruses; unclassified viruses
GCA_001590195.1	1915	2	Lake Sarahassociated circular virus10	NA	Viruses; unclassified viruses
GCA_001589875.1	2198	2	Lake Sarahassociated circular virus11	NA	Viruses; unclassified viruses
GCA_001589555.1	1898	2	Lake Sarahassociated circular virus12	NA	Viruses; unclassified viruses
GCA_001590475.1	1587	2	Lake Sarahassociated circular virus13	NA	Viruses; unclassified viruses
GCA_001590175.1	4301	2	Lake Sarahassociated circular virus14	NA	Viruses; unclassified viruses
GCA_001589855.1	1963	2	Lake Sarahassociated circular virus15	NA	Viruses; unclassified viruses
GCA_001589535.1	1888	2	Lake Sarahassociated circular virus16	NA	Viruses; unclassified viruses
GCA_001590455.1	2678	2	Lake Sarahassociated circular virus17	NA	Viruses; unclassified viruses
GCA_001590155.1	1821	2	Lake Sarahassociated circular virus18	NA	Viruses; unclassified viruses
GCA_001589835.1	1931	2	Lake Sarahassociated circular virus19	NA	Viruses; unclassified viruses
GCA_001589515.1	2301	2	Lake Sarahassociated circular virus2	NA	Viruses; unclassified viruses
GCA_001590435.1	1632	2	Lake Sarahassociated circular virus20	NA	Viruses; unclassified viruses
GCA_001590135.1	1762	2	Lake Sarahassociated circular virus21	NA	Viruses; unclassified viruses
GCA_001589815.1	1660	2	Lake Sarahassociated circular virus22	NA	Viruses; unclassified viruses
GCA_001590095.1	1775	2	Lake Sarahassociated circular virus23	NA	Viruses; unclassified viruses
GCA_001589775.1	1627	2	Lake Sarahassociated circular virus24	NA	Viruses; unclassified viruses
GCA_001589455.1	1776	2	Lake Sarahassociated circular virus25	NA	Viruses; unclassified viruses
GCA_001590375.1	2259	2	Lake Sarahassociated circular virus26	NA	Viruses; unclassified viruses
GCA_001590075.1	2256	2	Lake Sarahassociated circular virus27	NA	Viruses; unclassified viruses
GCA_001589755.1	6380	3	Lake Sarahassociated circular virus28	NA	Viruses; unclassified viruses
GCA_001589435.1	4282	2	Lake Sarahassociated circular virus29	NA	Viruses; unclassified viruses

gca	genome.size	proteome.size	phage.name	strain	Taxonomy
GCA_001590355.1	4441	3	Lake Sarahassociated circular virus3	NA	Viruses; unclassified viruses
GCA_001586885.1	3003	2	Lake Sarahassociated circular virus30	NA	Viruses; unclassified viruses
GCA_001586905.1	3124	2	Lake Sarahassociated circular virus31	NA	Viruses; unclassified viruses
GCA_001589675.1	2546	2	Lake Sarahassociated circular virus32	NA	Viruses; unclassified viruses
GCA_001590595.1	2191	2	Lake Sarahassociated circular virus33	NA	Viruses; unclassified viruses
GCA_001590295.1	2164	2	Lake Sarahassociated circular virus34	NA	Viruses; unclassified viruses
GCA_001589975.1	3481	2	Lake Sarahassociated circular virus35	NA	Viruses; unclassified viruses
GCA_001589655.1	2023	2	Lake Sarahassociated circular virus36	NA	Viruses; unclassified viruses
GCA_001590575.1	1677	2	Lake Sarahassociated circular virus37	NA	Viruses; unclassified viruses
GCA_001590275.1	3531	2	Lake Sarahassociated circular virus38	NA	Viruses; unclassified viruses
GCA_001589955.1	3208	2	Lake Sarahassociated circular virus39	NA	Viruses; unclassified viruses
GCA_001589635.1	1709	2	Lake Sarahassociated circular virus4	NA	Viruses; unclassified viruses
GCA_001590555.1	2746	2	Lake Sarahassociated circular virus40	NA	Viruses; unclassified viruses
GCA_001590255.1	3091	2	Lake Sarahassociated circular virus41	NA	Viruses; unclassified viruses
GCA_001589935.1	3049	2	Lake Sarahassociated circular virus42	NA	Viruses; unclassified viruses
GCA_001589615.1	2999	2	Lake Sarahassociated circular virus43	NA	Viruses; unclassified viruses
GCA_001590535.1	3910	2	Lake Sarahassociated circular virus44	NA	Viruses; unclassified viruses
GCA_001590235.1	2903	2	Lake Sarahassociated circular virus45	NA	Viruses; unclassified viruses
GCA_001589915.1	1912	2	Lake Sarahassociated circular virus46	NA	Viruses; unclassified viruses
GCA_001589595.1	2732	2	Lake Sarahassociated circular virus47	NA	Viruses; unclassified viruses
GCA_001590515.1	2039	2	Lake Sarahassociated circular virus48	NA	Viruses; unclassified viruses
GCA_001590215.1	2927	2	Lake Sarahassociated circular virus49	NA	Viruses; unclassified viruses
GCA_001589895.1	2987	2	Lake Sarahassociated circular virus5	NA	Viruses; unclassified viruses
GCA_001589575.1	1802	2	Lake Sarahassociated circular virus50	NA	Viruses; unclassified viruses
GCA_001589495.1	2175	2	Lake Sarahassociated circular virus51	NA	Viruses; unclassified viruses
GCA_001590415.1	2939	2	Lake Sarahassociated circular virus6	NA	Viruses; unclassified viruses
GCA_001590115.1	2304	2	Lake Sarahassociated circular virus7	NA	Viruses; unclassified viruses
GCA_001589795.1	2205	2	Lake Sarahassociated circular virus8	NA	Viruses; unclassified viruses
GCA_001589475.1	1932	2	Lake Sarahassociated circular virus9	NA	Viruses; unclassified viruses
GCA_001430495.1	22780	1	Macrosiphum euphorbiae virus 1	NA	Viruses; ssRNA viruses; ssRNA positive-strand viruses, no DNA stage; Flaviviridae; unclassified Flaviviridae
GCA_001271135.1	9829	1	Lettuce italian necrotic virus	NA	Viruses; ssRNA viruses; ssRNA positive-strand viruses, no DNA stage; Potyviridae; unclassified Potyviridae
GCA_002607165.1	80578	93	Vibrio phage vB_VorSPVo5	NA	Viruses; Duplodnaviria; Heunggongvirae; Uroviricota; Caudoviricetes; Caudovirales; Demerecviridae; Ermolyevavirinae; Cetovirus; unclassified Cetovirus
GCA_001500875.1	222888	237	Ralstonia phage RSF1	NA	Viruses; dsDNA viruses, no RNA stage; Caudovirales; Myoviridae; unclassified Myoviridae
GCA_003029075.1	11276	1	Porcine pestivirus 1	000515	Viruses; Riboviria; Orthornavirae; Kitrinoviricota; Flasuviricetes; Amarillovirales; Flaviviridae; Pestivirus; Pestivirus K

gca	genome.size	proteome.size	phage.name	strain	Taxonomy
GCA_001502475.1	49949	72	Streptomyces phage Caliburn	NA	Viruses; dsDNA viruses, no RNA stage; Caudovirales; Siphoviridae; unclassified Siphoviridae
GCA_002756735.1	49562	73	Streptomyces phage Aaronocolus	NA	Viruses; Duplodnaviria; Heunggongvirae; Uroviricota; Caudoviricetes; Caudovirales; Siphoviridae; Arquatrovirinae; Likavirus; Streptomyces virus Aaronocolus
GCA_002603425.1	50773	76	Streptomyces phage Danzina	NA	Viruses; Duplodnaviria; Heunggongvirae; Uroviricota; Caudoviricetes; Caudovirales; Siphoviridae; Arquatrovirinae; Likavirus; Streptomyces virus Danzina
GCA_002756755.1	50727	76	Streptomyces phage Hydra	NA	Viruses; Duplodnaviria; Heunggongvirae; Uroviricota; Caudoviricetes; Caudovirales; Siphoviridae; Arquatrovirinae; Likavirus; Streptomyces virus Hydra
GCA_001501855.1	162252	300	Bacillus phage Eyuki	NA	Viruses; dsDNA viruses, no RNA stage; Caudovirales; Myoviridae; unclassified Myoviridae; Bacillus phage DirtyBetty
GCA_001504235.1	162486	247	Bacillus phage TsarBomba	NA	Viruses; dsDNA viruses, no RNA stage; Caudovirales; Myoviridae; unclassified Myoviridae
GCA_001271195.1	10119	1	Bombyx mori iflavirus	BMI1	Viruses; ssRNA viruses; ssRNA positive-strand viruses, no DNA stage; Picornavirales; Iflaviridae; Iflavirus; unclassified Iflavirus
GCA_001308495.1	3542	3	White sucker hepatitis B virus	NA	Viruses; Riboviria; Pararnavirae; Artverviricota; Revtraviricetes; Blubervirales; Hepadnaviridae; Parahepadnavirus
GCA_001470455.1	50418	81	Mycobacterium phage Phlei	NA	Viruses; dsDNA viruses, no RNA stage; Caudovirales; Podoviridae; unclassified Podoviridae; Escherichia phage phi191 sensu lato
GCA_001505695.1	29605	42	Propionibacterium phage PAC1	NA	Viruses; dsDNA viruses, no RNA stage; Caudovirales; Siphoviridae; unclassified Siphoviridae
GCA_002598785.1	29602	43	Propionibacterium phage PAC2	NA	Viruses; Duplodnaviria; Heunggongvirae; Uroviricota; Caudoviricetes; Caudovirales; Siphoviridae; Pahexavirus; unclassified Pahexavirus
GCA_002598805.1	29526	42	Propionibacterium phage PAC3	NA	Viruses; Duplodnaviria; Heunggongvirae; Uroviricota; Caudoviricetes; Caudovirales; Siphoviridae; Pahexavirus; unclassified Pahexavirus
GCA_002598825.1	29581	43	Propionibacterium phage PAC4	NA	Viruses; Duplodnaviria; Heunggongvirae; Uroviricota; Caudoviricetes; Caudovirales; Siphoviridae; Pahexavirus; unclassified Pahexavirus
GCA_002598845.1	29428	43	Propionibacterium phage PAC5	NA	Viruses; Duplodnaviria; Heunggongvirae; Uroviricota; Caudoviricetes; Caudovirales; Siphoviridae; Pahexavirus; unclassified Pahexavirus
GCA_002598865.1	29609	42	Propionibacterium phage PAC6	NA	Viruses; Duplodnaviria; Heunggongvirae; Uroviricota; Caudoviricetes; Caudovirales; Siphoviridae; Pahexavirus; unclassified Pahexavirus
GCA_002598885.1	29551	42	Propionibacterium phage PAC7	NA	Viruses; Duplodnaviria; Heunggongvirae; Uroviricota; Caudoviricetes; Caudovirales; Siphoviridae; Pahexavirus; unclassified Pahexavirus
GCA_002598905.1	29488	42	Propionibacterium phage PAC8	NA	Viruses; Duplodnaviria; Heunggongvirae; Uroviricota; Caudoviricetes; Caudovirales; Siphoviridae; Pahexavirus; unclassified Pahexavirus
GCA_002598925.1	29786	43	Propionibacterium phage PAC9	NA	Viruses; Duplodnaviria; Heunggongvirae; Uroviricota; Caudoviricetes; Caudovirales; Siphoviridae; Pahexavirus; unclassified Pahexavirus
GCA_002598945.1	29704	43	Propionibacterium phage PAC10	NA	Viruses; Duplodnaviria; Heunggongvirae; Uroviricota; Caudoviricetes; Caudovirales; Siphoviridae; Pahexavirus; unclassified Pahexavirus
GCA_001505035.1	43150	52	Streptomyces phage SF1	NA	Viruses; dsDNA viruses, no RNA stage; Caudovirales; Siphoviridae; unclassified Siphoviridae
GCA_001503415.1	60934	90	Streptomyces phage SF3	NA	Viruses; dsDNA viruses, no RNA stage; Caudovirales; Siphoviridae; unclassified Siphoviridae
GCA_001271035.1	10870	1	Spanish goat encephalitis virus	NA	Viruses; ssRNA viruses; ssRNA positive-strand viruses, no DNA stage; Flaviviridae; Flavivirus; unclassified Flavivirus
GCA_001504215.1	126341	178	Brevibacillus phage Jenst	NA	Viruses; dsDNA viruses, no RNA stage; Caudovirales; Siphoviridae; unclassified Siphoviridae
GCA_001505675.1	52955	103	Brevibacillus phage Osiris	NA	Viruses; dsDNA viruses, no RNA stage; Caudovirales; Myoviridae; unclassified Myoviridae
GCA_002607185.1	130482	183	Brevibacillus phage SecTim467	NA	Viruses; Duplodnaviria; Heunggongvirae; Uroviricota; Caudoviricetes; Caudovirales; Siphoviridae; Jenstvirus; Brevibacillus virus Jenst
GCA_002607205.1	52992	103	Brevibacillus phage Powder	NA	Viruses; Duplodnaviria; Heunggongvirae; Uroviricota; Caudoviricetes; Caudovirales; Myoviridae; Jimmervirus; Brevibacillus virus Osiris; Brevibacillus phage Osiris
GCA_001500375.1	134270	194	Brevibacillus phage Sundance	NA	Viruses; dsDNA viruses, no RNA stage; Caudovirales; Siphoviridae; unclassified Siphoviridae
GCA_001271275.1	15395	9	Tobacco virus 1	NA	Viruses; ssRNA viruses; ssRNA positive-strand viruses, no DNA stage; Closteroviridae; Closterovirus; unclassified Closterovirus
GCA_001274225.2	19250	10	Fengkai orbivirus	NA	Viruses; Riboviria; Orthornavirae; Duplornaviricota; Resentoviricetes; Reovirales; Reoviridae; Sedoreovirinae; Orbivirus; unclassified Orbivirus; Tibet orbivirus
GCA_004790475.1	10543	3	Brassica campestris chrysovirus 1	NA	Viruses; Riboviria; Orthornavirae; Duplornaviricota; Chrymotiviricetes; Ghabrivirales; Chrysoviridae; Alphachrysovirus; Brassica campestris chrysovirus
GCA_001274085.1	1901	2	Aiptasia sp sea anemone associated circular virus	NA	Viruses; Monodnaviria; Shotokuvirae; Cressdnaviricota; Arfiviricetes; Cirlivirales; Circoviridae; unclassified Circoviridae
GCA_001274325.1	1826	1	Artemia melana sponge associated circular genome	NA	Viruses; ssDNA viruses; Circoviridae; unclassified Circoviridae
GCA_001274205.1	2469	2	Calanoida sp copepod associated circular virus	NA	Viruses; Monodnaviria; Shotokuvirae; Cressdnaviricota; Arfiviricetes; Cirlivirales; Circoviridae; unclassified Circoviridae
GCA_001274445.1	1241	2	Callinectes ornatus blue crab associated circular virus	NA	Viruses; ssDNA viruses; Circoviridae; unclassified Circoviridae

gca	genome.size	proteome.size	phage.name	strain	Taxonomy
GCA_001274065.1	1876	2	Callinectes sapidus associated circular virus	NA	Viruses; ssDNA viruses; Circoviridae; unclassified Circoviridae
GCA_001274305.1	2061	2	Didemnum sp Sea Squirt associated virus	NA	Viruses; Monodnaviria; Shotokuvirae; Cressdnaviricota; Arfiviricetes; Cirlivirales; Circoviridae; unclassified Circoviridae
GCA_001274185.1	1799	2	Farfantepenaeus duorarum pink shrimp associated circular virus	NA	Viruses; ssDNA viruses; Circoviridae; unclassified Circoviridae
GCA_001274425.1	1635	2	Fiddler Crab associated circular virus	NA	Viruses; ssDNA viruses; Circoviridae; unclassified Circoviridae
GCA_001274045.1	1999	2	Gammarus sp amphipod associated circular virus	NA	Viruses; Monodnaviria; Shotokuvirae; Cressdnaviricota; Arfiviricetes; Cirlivirales; Circoviridae; unclassified Circoviridae
GCA_001274105.1	1063	1	Hermit crab associated circular genome	NA	Viruses; ssDNA viruses; Circoviridae; unclassified Circoviridae
GCA_001274285.1	2291	2	Hermit crab associated circular virus	NA	Viruses; ssDNA viruses; Circoviridae; unclassified Circoviridae
GCA_001274165.1	2237	2	Littorina sp associated circular virus	NA	Viruses; Monodnaviria; Shotokuvirae; Cressdnaviricota; Arfiviricetes; Cirlivirales; Circoviridae; unclassified Circoviridae
GCA_001274505.1	2167	2	Lytechinus variegatus variable sea urchin associated circular virus	NA	Viruses; ssDNA viruses; Circoviridae; unclassified Circoviridae
GCA_001274145.1	2305	2	Marine snail associated circular virus	NA	Viruses; ssDNA viruses; Circoviridae; unclassified Circoviridae
GCA_001274385.1	1894	2	Mytilus sp clam associated circular virus	NA	Viruses; Monodnaviria; Shotokuvirae; Cressdnaviricota; Arfiviricetes; Cirlivirales; Circoviridae; unclassified Circoviridae
GCA_001274265.1	2293	2	Palaemonetes intermedius brackish grass shrimp associated circular virus	NA	Viruses; ssDNA viruses; Circoviridae; unclassified Circoviridae
GCA_001274485.1	1895	2	Palaemonetes kadiakensis Mississippi grass shrimp associated circular virus	NA	Viruses; ssDNA viruses; Circoviridae; unclassified Circoviridae
GCA_001275275.1	2257	2	Palaemonetes sp common grass shrimp associated circular virus	NA	Viruses; Monodnaviria; Shotokuvirae; Cressdnaviricota; Arfiviricetes; Cirlivirales; Circoviridae; unclassified Circoviridae
GCA_001274125.1	2292	2	Paramuricea placomus associated circular virus	NA	Viruses; ssDNA viruses; Circoviridae; unclassified Circoviridae
GCA_001274365.1	1815	2	Petrochirus diogenes giant hermit crab associated circular virus	NA	Viruses; ssDNA viruses; Circoviridae; unclassified Circoviridae
GCA_001274245.1	1240	2	Primnoa pacifica coral associated circular virus	NA	Viruses; ssDNA viruses; Circoviridae; unclassified Circoviridae
GCA_001274465.1	1600	2	Sicyonia brevirostris associated circular virus	NA	Viruses; ssDNA viruses; Circoviridae; unclassified Circoviridae
GCA_002756775.1	41768	52	Salmonella phage fSE4S	NA	Viruses; Duplodnaviria; Heunggongvirae; Uroviricota; Caudoviricetes; Caudovirales; Siphoviridae; Guernseyvirinae; Jerseyvirus; Salmonella virus f18SE; Salmonella phage f18SE
GCA_000842965.1	280334	369	Pseudomonas virus phiKZ	NA	Viruses; Duplodnaviria; Heunggongvirae; Uroviricota; Caudoviricetes; Caudovirales; Myoviridae; Phikzvirus
GCA_003687545.1	58072	96	Halobacterium phage phiH	NA	Viruses; Duplodnaviria; Heunggongvirae; Uroviricota; Caudoviricetes; Caudovirales; Myoviridae; Myohalovirus; Halobacterium virus phiH
GCA_000846445.1	1351	1	Ageratum yellow leaf curl betasatellite	NA	Viruses; Tolecusatellitidae; Betasatellite
GCA_000845585.1	1356	1	Tobacco leaf curl disease associated sequence	NA	Viruses; Tolecusatellitidae; Betasatellite; Tobacco leaf curl betasatellite
GCA_000846465.1	1307	1	Okra yellow vein disease associated sequence	NA	Viruses; Tolecusatellitidae; Betasatellite; unclassified Betasatellite
GCA_019333625.1	131276	136	Scale drop disease virus	NA	Viruses; dsDNA viruses, no RNA stage; Iridoviridae; unclassified Iridoviridae
GCA_001505015.1	71214	97	Mycobacterium phage BrownCNA	NA	Viruses; dsDNA viruses, no RNA stage; Caudovirales; Siphoviridae; unclassified Siphoviridae
GCA_002601565.1	155292	232	Mycobacterium phage Zeenon	NA	Viruses; Duplodnaviria; Heunggongvirae; Uroviricota; Caudoviricetes; Caudovirales; Myoviridae; Bixzunavirus; Mycobacterium virus Bxz1
GCA_002756795.1	50841	89	Mycobacterium phage DaHudson	NA	Viruses; Duplodnaviria; Heunggongvirae; Uroviricota; Caudoviricetes; Caudovirales; Siphoviridae; Fromanvirus; unclassified Fromanvirus
GCA_001503395.1	57293	101	Mycobacterium phage Phatniss	NA	Viruses; dsDNA viruses, no RNA stage; Caudovirales; Siphoviridae; unclassified Siphoviridae
GCA_001504195.1	59652	105	Mycobacterium phage Dante	NA	Viruses; dsDNA viruses, no RNA stage; Caudovirales; Siphoviridae; unclassified Siphoviridae
GCA_001502415.1	47618	78	Mycobacterium phage Brusacoram	NA	Viruses; dsDNA viruses, no RNA stage; Caudovirales; Siphoviridae; unclassified Siphoviridae
GCA_002756815.2	41901	63	Mycobacterium phage Phreak	NA	Viruses; Duplodnaviria; Heunggongvirae; Uroviricota; Caudoviricetes; Caudovirales; Siphoviridae; Liefievirus; unclassified Liefievirus
GCA_001470655.1	166313	278	Klebsiella phage JD18	NA	Viruses; dsDNA viruses, no RNA stage; Caudovirales; Myoviridae; unclassified Myoviridae
GCA_001470355.1	15524	26	Arthrobacter phage Decurro	NA	Viruses; dsDNA viruses, no RNA stage; Caudovirales; Siphoviridae; unclassified Siphoviridae; Arthrobacter phage Muttli

gca	genome.size	proteome.size	phage.name	strain	Taxonomy
GCA_002598625.1	15556	26	Arthrobacter phage Jessica	NA	Viruses; Duplodnaviria; Heunggongvirae; Uroviricota; Caudoviricetes; Caudovirales; Siphoviridae; Decurrovirus; unclassified Decurrovirus
GCA_002598645.1	15630	26	Arthrobacter phage Sandman	NA	Viruses; Duplodnaviria; Heunggongvirae; Uroviricota; Caudoviricetes; Caudovirales; Siphoviridae; Decurrovirus; unclassified Decurrovirus
GCA_002756835.1	41901	63	Mycobacterium phage Cedasite	NA	Viruses; Duplodnaviria; Heunggongvirae; Uroviricota; Caudoviricetes; Caudovirales; Siphoviridae; Liefievirus; unclassified Liefievirus
GCA_002756855.1	41901	63	Mycobacterium phage Frosty24	NA	Viruses; Duplodnaviria; Heunggongvirae; Uroviricota; Caudoviricetes; Caudovirales; Siphoviridae; Liefievirus; unclassified Liefievirus
GCA_001505655.1	49421	88	Mycobacterium phage Chadwick	NA	Viruses; dsDNA viruses, no RNA stage; Caudovirales; Siphoviridae; unclassified Siphoviridae
GCA_002756875.1	53790	84	Mycobacterium phage OBUpride	NA	Viruses; Duplodnaviria; Heunggongvirae; Uroviricota; Caudoviricetes; Caudovirales; Siphoviridae; Gilesvirus; Mycobacterium virus Giles
GCA_001550865.1	24847	48	Lactococcus phage GE1	NA	Viruses; dsDNA viruses, no RNA stage; Caudovirales; Siphoviridae; unclassified Siphoviridae
GCA_001504995.1	53219	99	Mycobacterium phage Pioneer	NA	Viruses; dsDNA viruses, no RNA stage; Caudovirales; Siphoviridae; unclassified Siphoviridae
GCA_002607245.1	50081	86	Mycobacterium phage Texage	NA	Viruses; Duplodnaviria; Heunggongvirae; Uroviricota; Caudoviricetes; Caudovirales; Siphoviridae; Fromanvirus; unclassified Fromanvirus
GCA_001503375.1	51409	89	Mycobacterium phage Tasp14	NA	Viruses; dsDNA viruses, no RNA stage; Caudovirales; Siphoviridae; unclassified Siphoviridae
GCA_001470875.1	43012	70	Staphylococcus phage phiJB	NA	Viruses; dsDNA viruses, no RNA stage; Caudovirales; Siphoviridae; Phietavirus; unclassified Phietalikevirus
GCA_002756895.1	36081	46	Rhodobacter phage RcSaxon	NA	Viruses; Duplodnaviria; Heunggongvirae; Uroviricota; Caudoviricetes; Caudovirales; Siphoviridae; Cronusvirus; Rhodobacter virus RcCronus
GCA_001745115.1	40570	45	Acinetobacter phage phiAB6	NA	Viruses; dsDNA viruses, no RNA stage; Caudovirales; Podoviridae; unclassified Podoviridae
GCA_002604745.2	57256	92	Mycobacterium phage Slarp	NA	Viruses; Duplodnaviria; Heunggongvirae; Uroviricota; Caudoviricetes; Caudovirales; Siphoviridae; Timquatrovirus; unclassified Timquatrovirus
GCA_001504175.1	52357	93	Mycobacterium phage Rufus	NA	Viruses; dsDNA viruses, no RNA stage; Caudovirales; Siphoviridae; unclassified Siphoviridae
GCA_001505635.1	167431	301	Bacillus phage AvesoBmore	NA	Viruses; dsDNA viruses, no RNA stage; Caudovirales; Myoviridae; unclassified Myoviridae
GCA_002607265.1	39591	52	Thiobacimonas phage vB_ThpSP1	NA	Viruses; Duplodnaviria; Heunggongvirae; Uroviricota; Caudoviricetes; Caudovirales; Siphoviridae; unclassified Siphoviridae
GCA_000848005.1	5223	7	Macroptilium yellow mosaic virus	NA	Viruses; ssDNA viruses; Geminiviridae; Begomovirus
GCA_002607285.1	43872	51	Dickeya phage BF2512	NA	Viruses; Duplodnaviria; Heunggongvirae; Uroviricota; Caudoviricetes; Caudovirales; Autographiviridae; Corkvirinae; Stompvirus; Dickeya virus BF25-12
GCA_001504975.1	59052	79	Mycobacterium phage Enkosi	NA	Viruses; dsDNA viruses, no RNA stage; Caudovirales; Siphoviridae; unclassified Siphoviridae
GCA_001503355.1	153714	224	Mycobacterium phage HyRo	NA	Viruses; dsDNA viruses, no RNA stage; Caudovirales; Myoviridae; unclassified Myoviridae
GCA_001504155.1	75816	130	Mycobacterium phage Lolly9	NA	Viruses; dsDNA viruses, no RNA stage; Caudovirales; Siphoviridae; unclassified Siphoviridae
GCA_001505615.1	55094	95	Mycobacterium phage PopTart	NA	Viruses; dsDNA viruses, no RNA stage; Caudovirales; Siphoviridae; unclassified Siphoviridae
GCA_001504955.1	57766	105	Mycobacterium phage Seagreen	NA	Viruses; dsDNA viruses, no RNA stage; Caudovirales; Siphoviridae; unclassified Siphoviridae
GCA_001503335.1	75626	130	Mycobacterium phage Snenia	NA	Viruses; dsDNA viruses, no RNA stage; Caudovirales; Siphoviridae; unclassified Siphoviridae
GCA_001504135.1	55617	95	Mycobacterium phage XFactor	NA	Viruses; dsDNA viruses, no RNA stage; Caudovirales; Siphoviridae; unclassified Siphoviridae
GCA_002603785.1	69653	123	Mycobacterium phage Zakhe101	NA	Viruses; Duplodnaviria; Heunggongvirae; Uroviricota; Caudoviricetes; Caudovirales; Siphoviridae; Corndogvirus; unclassified Corndogvirus
GCA_001273685.1	2152	2	Poecile atricapillus GI tractassociated gemycircularvirus	NA	Viruses; unclassified viruses; Genomoviridae; Gemycircularvirus; unclassified Gemycircularvirus
GCA_001500975.1	27395	7	Camel alphacoronavirus	NA	Viruses; ssRNA viruses; ssRNA positive-strand viruses, no DNA stage; Nidovirales; Coronaviridae; Coronavirinae; Alphacoronavirus; Human coronavirus 229E
GCA_003967955.1	2532	2	Rat stoolassociated circular ssDNA virus	NA	Viruses; ssDNA viruses; Circoviridae; unclassified Circoviridae
GCA_002756915.1	68405	101	Mycobacterium phage Hetaeria	NA	Viruses; Duplodnaviria; Heunggongvirae; Uroviricota; Caudoviricetes; Caudovirales; Siphoviridae; Bclasvirinae; Pegunavirus; Mycobacterium virus Suffolk
GCA_002756935.1	46596	66	Rhodococcus phage Rhodalysa	NA	Viruses; Duplodnaviria; Heunggongvirae; Uroviricota; Caudoviricetes; Caudovirales; Siphoviridae; Rerduovirus; unclassified Rerduovirus
GCA_001471055.1	40491	62	Enterobacter phage phiEap2	NA	Viruses; dsDNA viruses, no RNA stage; Caudovirales; Siphoviridae; unclassified Siphoviridae
GCA_002607325.1	53802	62	Streptococcus phage phiNJ3	NA	Viruses; Duplodnaviria; Heunggongvirae; Uroviricota; Caudoviricetes; Caudovirales; Siphoviridae; unclassified Siphoviridae

gca	genome.size	proteome.size	phage.name	strain	Taxonomy
GCA_002607345.1	57638	66	Streptococcus phage phiSC070807	NA	Viruses; Duplodnaviria; Heunggongvirae; Uroviricota; Caudoviricetes; Caudovirales; Siphoviridae; unclassified Siphoviridae
GCA_001502915.1	4468	1	Gokushovirinae GAIR4	NA	Viruses; ssDNA viruses; Microviridae; Gokushovirinae; unclassified Gokushovirinae
GCA_001502295.1	4273	1	Gokushovirinae GNX3R	NA	Viruses; ssDNA viruses; Microviridae; Gokushovirinae; unclassified Gokushovirinae
GCA_003029095.2	5234	7	Tomato chlorotic mottle Guyane virus	NA	Viruses; Monodnaviria; Shotokuvirae; Cressdnaviricota; Repensiviricetes; Geplafuvirales; Geminiviridae; Begomovirus
GCA_002622425.1	49879	74	Arthrobacter phage Brent	NA	Viruses; Duplodnaviria; Heunggongvirae; Uroviricota; Caudoviricetes; Caudovirales; Myoviridae; Marthavirus; Arthrobacter virus Brent
GCA_002607365.1	51372	91	Mycobacterium phage Maverick	NA	Viruses; Duplodnaviria; Heunggongvirae; Uroviricota; Caudoviricetes; Caudovirales; Siphoviridae; Fromanvirus; unclassified Fromanvirus
GCA_002603505.1	156754	233	Mycobacterium phage DTDevon	NA	Viruses; Duplodnaviria; Heunggongvirae; Uroviricota; Caudoviricetes; Caudovirales; Myoviridae; Bixzunavirus; Mycobacterium virus Bxz1
GCA_002607385.1	41893	62	Mycobacterium phage Annihilator	NA	Viruses; Duplodnaviria; Heunggongvirae; Uroviricota; Caudoviricetes; Caudovirales; Siphoviridae; Liefievirus; unclassified Liefievirus
GCA_002630465.1	153243	226	Mycobacterium phage ErnieJ	NA	Viruses; Duplodnaviria; Heunggongvirae; Uroviricota; Caudoviricetes; Caudovirales; Myoviridae; Bixzunavirus; Mycobacterium virus Bxz1
GCA_002756955.1	67349	92	Mycobacterium phage Tres	NA	Viruses; Duplodnaviria; Heunggongvirae; Uroviricota; Caudoviricetes; Caudovirales; Siphoviridae; Bclasvirinae; Rosebushvirus; Mycobacterium virus Rosebush
GCA_001470975.1	40114	47	Klebsiella phage vB_Kp1	NA	Viruses; dsDNA viruses, no RNA stage; Caudovirales; Podoviridae; Autographivirinae; unclassified Autographivirinae
GCA_001470195.1	43963	58	Klebsiella phage vB_Kp2	NA	Viruses; dsDNA viruses, no RNA stage; Caudovirales; Podoviridae; Autographivirinae; unclassified Autographivirinae
GCA_002607405.1	48493	59	Klebsiella phage vB_Kp3	NA	Viruses; Duplodnaviria; Heunggongvirae; Uroviricota; Caudoviricetes; Caudovirales; Siphoviridae; unclassified Siphoviridae
GCA_001470095.1	41563	48	Acinetobacter phage vB_AbaP_PD6A3	NA	Viruses; dsDNA viruses, no RNA stage; Caudovirales; Podoviridae; unclassified Podoviridae
GCA_001470635.1	40938	48	Acinetobacter phage vB_AbaP_PDAB9	NA	Viruses; dsDNA viruses, no RNA stage; Caudovirales; Podoviridae; unclassified Podoviridae
GCA_002149385.1	44974	55	Caulobacter phage Percy	NA	Viruses; dsDNA viruses, no RNA stage; Caudovirales; Podoviridae; unclassified Podoviridae
GCA_002149305.1	178182	280	Citrobacter phage Margaery	NA	Viruses; dsDNA viruses, no RNA stage; Caudovirales; Myoviridae; Tevenvirinae; T4virus; unclassified T4virus
GCA_001882175.1	41105	48	Streptococcus phage phiARI0004	NA	Viruses; unclassified phages
GCA_001882335.1	41887	51	Streptococcus phage phiARI0031	NA	Viruses; unclassified phages
GCA_001885385.1	42927	53	Streptococcus phage phiARI01311	NA	Viruses; unclassified phages
GCA_001884675.1	34563	37	Streptococcus phage phiARI01312	NA	Viruses; unclassified phages
GCA_001881755.1	41807	51	Streptococcus phage phiARI04601	NA	Viruses; unclassified phages
GCA_001881835.1	40498	51	Streptococcus phage phiARI0462	NA	Viruses; unclassified phages
GCA_001882055.1	41063	48	Streptococcus phage phiARI04681	NA	Viruses; unclassified phages
GCA_001881695.1	42186	49	Streptococcus phage phiARI04682	NA	Viruses; unclassified phages
GCA_001885405.1	37395	49	Streptococcus phage phiARI04684	NA	Viruses; dsDNA viruses, no RNA stage; Caudovirales; Siphoviridae; unclassified Siphoviridae
GCA_001884655.1	33181	42	Streptococcus phage phiARI0746	NA	Viruses; dsDNA viruses, no RNA stage; Caudovirales; Siphoviridae; unclassified Siphoviridae
GCA_001736395.1	33477	35	Streptococcus phage phiARI0923	NA	Viruses; dsDNA viruses, no RNA stage; Caudovirales; Siphoviridae; unclassified Siphoviridae
GCA_001505595.1	52055	90	Mycobacterium phage TheloniousMonk	NA	Viruses; dsDNA viruses, no RNA stage; Caudovirales; Siphoviridae; unclassified Siphoviridae
GCA_002624425.1	89596	138	Citrobacter phage Mordin	NA	Viruses; Duplodnaviria; Heunggongvirae; Uroviricota; Caudoviricetes; Caudovirales; Myoviridae; Ounavirinae; Mooglevirus; Citrobacter virus Mordin
GCA_002603485.1	75114	141	Mycobacterium phage Ukulele	NA	Viruses; Duplodnaviria; Heunggongvirae; Uroviricota; Caudoviricetes; Caudovirales; Siphoviridae; Kostyavirus; unclassified Kostyavirus
GCA_002607425.1	34979	47	Bacillus phage vB_BtS_BMBtp16	NA	Viruses; Duplodnaviria; Heunggongvirae; Uroviricota; Caudoviricetes; Caudovirales; Siphoviridae; unclassified Siphoviridae
GCA_001504935.1	51161	86	Mycobacterium phage Turj99	NA	Viruses; dsDNA viruses, no RNA stage; Caudovirales; Siphoviridae; unclassified Siphoviridae
GCA_001470335.1	33900	46	Streptococcus phage A25	NA	Viruses; dsDNA viruses, no RNA stage; Caudovirales; Podoviridae; unclassified Podoviridae; Escherichia phage phi191 sensu lato

gca	genome.size	proteome.size	phage.name	strain	Taxonomy
GCA_001470855.1	46596	66	Rhodococcus phage CosmicSans	NA	Viruses; dsDNA viruses, no RNA stage; Caudovirales; Siphoviridae; unclassified Siphoviridae
GCA_002607305.1	75586	128	Mycobacterium phage Lumos	NA	Viruses; Duplodnaviria; Heunggongvirae; Uroviricota; Caudoviricetes; Caudovirales; Siphoviridae; Bronvirus; unclassified Bronvirus
GCA_001503315.1	52088	97	Mycobacterium phage Serenity	NA	Viruses; dsDNA viruses, no RNA stage; Caudovirales; Siphoviridae; unclassified Siphoviridae
GCA_002756975.1	50797	92	Mycobacterium phage Wooldri	NA	Viruses; Duplodnaviria; Heunggongvirae; Uroviricota; Caudoviricetes; Caudovirales; Siphoviridae; Fromanvirus; unclassified Fromanvirus
GCA_002597325.1	65917	88	Pseudomonas phage Gallinipper	NA	Viruses; Duplodnaviria; Heunggongvirae; Uroviricota; Caudoviricetes; Caudovirales; Myoviridae; Pbunavirus; Pseudomonas virus PB1
GCA_002597345.1	65795	89	Pseudomonas phage Jollyroger	NA	Viruses; Duplodnaviria; Heunggongvirae; Uroviricota; Caudoviricetes; Caudovirales; Myoviridae; Pbunavirus; Pseudomonas virus PB1
GCA_002597365.1	65762	88	Pseudomonas phage Kraken	NA	Viruses; Duplodnaviria; Heunggongvirae; Uroviricota; Caudoviricetes; Caudovirales; Myoviridae; Pbunavirus; Pseudomonas virus PB1
GCA_002597385.1	65762	88	Pseudomonas phage Kula	NA	Viruses; Duplodnaviria; Heunggongvirae; Uroviricota; Caudoviricetes; Caudovirales; Myoviridae; Pbunavirus; Pseudomonas virus PB1
GCA_002597405.1	66283	87	Pseudomonas phage Nemo	NA	Viruses; Duplodnaviria; Heunggongvirae; Uroviricota; Caudoviricetes; Caudovirales; Myoviridae; Pbunavirus; Pseudomonas virus PB1
GCA_002597425.1	65762	88	Pseudomonas phage Nessie	NA	Viruses; Duplodnaviria; Heunggongvirae; Uroviricota; Caudoviricetes; Caudovirales; Myoviridae; Pbunavirus; Pseudomonas virus PB1
GCA_002597445.1	65762	87	Pseudomonas phage Poseidon	NA	Viruses; Duplodnaviria; Heunggongvirae; Uroviricota; Caudoviricetes; Caudovirales; Myoviridae; Pbunavirus; Pseudomonas virus PB1
GCA_002597465.1	66278	88	Pseudomonas phage Smee	NA	Viruses; Duplodnaviria; Heunggongvirae; Uroviricota; Caudoviricetes; Caudovirales; Myoviridae; Pbunavirus; Pseudomonas virus PB1
GCA_002597485.1	65762	84	Pseudomonas phage Triton	NA	Viruses; Duplodnaviria; Heunggongvirae; Uroviricota; Caudoviricetes; Caudovirales; Myoviridae; Pbunavirus; Pseudomonas virus PB1
GCA_003029105.1	8881	6	Atractylodes mottle virus	NA	Viruses; Riboviria; Orthornavirae; Kitrinoviricota; Alsuviricetes; Tymovirales; Betaflexiviridae; Quinvirinae; Carlavirus
GCA_002605645.1	44172	84	Paenibacillus phage Paisley	NA	Viruses; Duplodnaviria; Heunggongvirae; Uroviricota; Caudoviricetes; Caudovirales; Siphoviridae; Harrisonvirus; Paenibacillus virus Harrison
GCA_002756995.1	44256	84	Paenibacillus phage Hayley	NA	Viruses; Duplodnaviria; Heunggongvirae; Uroviricota; Caudoviricetes; Caudovirales; Siphoviridae; Vegasvirus; Paenibacillus virus Vegas
GCA_001646155.1	125171	76	Canid alphaherpesvirus 1	0194	Viruses; dsDNA viruses, no RNA stage; Herpesvirales; Herpesviridae; Alphaherpesvirinae; Varicellovirus
GCA_002821385.1	9538	1	Human hepegivirus	NA	Viruses; Riboviria; Orthornavirae; Kitrinoviricota; Flasuviricetes; Amarillovirales; Flaviviridae; Pegivirus; Pegivirus H
GCA_001430615.1	1353	1	Luffa begomovirus betasatellite	NA	Viruses; Tolecusatellitidae; Betasatellite; unclassified Betasatellite
GCA_001430415.1	1353	1	Malachra yellow vein mosaic betasatellite	NA	Viruses; Tolecusatellitidae; Betasatellite; unclassified Betasatellite
GCA_001429995.1	1381	1	Ageratum conyzoides symptomless alphasatellite	NA	Viruses; Satellites; Satellite Nucleic Acids; Single stranded DNA satellites; Alphasatellites; Begomovirus-associated alphasatellites; unclassified Begomovirus-associated alphasatellites
GCA_000899235.1	1358	1	Mesta yellow vein mosaic alphasatellite	NA	Viruses; Alphasatellitidae; Geminialphasatellitinae; Gosmusatellite
GCA_000841485.1	99657	119	Adoxophyes orana granulovirus	NA	Viruses; dsDNA viruses, no RNA stage; Baculoviridae; Betabaculovirus
GCA_000849405.1	9476	1	Perina nuda virus	NA	Viruses; ssRNA viruses; ssRNA positive-strand viruses, no DNA stage; Picornavirales; Iflaviridae; Iflavirus
GCA_018580355.1	1901	1	Rhizoctonia solani ourmialike virus 1	NA	Viruses; Riboviria; Orthornavirae; Lenarviricota; Miaviricetes; Ourlivirales; Botourmiaviridae; Magoulivirus; Rhizoctonia magoulivirus 1
GCA_003673785.1	9707	5	Sclerotinia sclerotiorum negativestranded RNA virus 4	NA	Viruses; Riboviria; Orthornavirae; Negarnaviricota; Haploviricotina; Monjiviricetes; Mononegavirales; Mymonaviridae; Sclerotimonavirus; Drop sclerotimonavirus
GCA_004790515.1	13086	4	Macrophomina phaseolina chrysovirus 1	NA	Viruses; Riboviria; Orthornavirae; Duplornaviricota; Chrymotiviricetes; Ghabrivirales; Chrysoviridae; Alphachrysovirus; Macrophomina phaseolina chrysovirus
GCA_001292955.1	7476	1	Phopivirus	NewEngland_USA2011	Viruses; ssRNA viruses; ssRNA positive-strand viruses, no DNA stage; Picornavirales; Picornaviridae; Hepatovirus; unclassified Hepatovirus
GCA_001293015.1	10938	1	Mercadeo virus	NA	Viruses; ssRNA viruses; ssRNA positive-strand viruses, no DNA stage; Flaviviridae; Flavivirus; unclassified Flavivirus
GCA_001292975.1	2188	3	Gemycircularvirus gemychrat1	NA	Viruses; unclassified viruses; Genomoviridae; Gemycircularvirus; unclassified Gemycircularvirus
GCA_001292895.1	10893	1	Parramatta River virus	NA	Viruses; ssRNA viruses; ssRNA positive-strand viruses, no DNA stage; Flaviviridae; Flavivirus; unclassified Flavivirus
GCA_001736275.1	9639	1	Rosellinia necatrix endornavirus 1	W1141	Viruses; dsRNA viruses; Endornaviridae; Endornavirus
GCA_000851585.1	4844	2	Trichomonas vaginalis virus 3	NA	Viruses; dsRNA viruses; Totiviridae; Trichomonasvirus
GCA_001308595.1	14729	1	Hot pepper endornavirus	NA	Viruses; dsRNA viruses; Endornaviridae; Endornavirus; unclassified Endornavirus

gca	genome.size	proteome.size	phage.name	strain	Taxonomy
GCA_001308615.1	8105	6	Atractylodes mild mottle virus	NA	Viruses; Riboviria; Pararnavirae; Artverviricota; Revtraviricetes; Ortervirales; Caulimoviridae; Caulimovirus
GCA_001308375.1	2794	1	Binucleate Rhizoctonia mitovirus K1	NA	Viruses; ssRNA viruses; ssRNA positive-strand viruses, no DNA stage; Narnaviridae; Mitovirus; unclassified Mitovirus
GCA_002867385.1	16580	13	Tetterwort vein chlorosis virus	NA	Viruses; Riboviria; Orthornavirae; Kitrinoviricota; Alsuviricetes; Martellivirales; Closteroviridae; Crinivirus
GCA_001550485.1	41620	48	Cronobacter phage DevCD23823	NA	Viruses; dsDNA viruses, no RNA stage; Caudovirales; Podoviridae; unclassified Podoviridae
GCA_002288735.2	18380	3	Kasokero virus	NA	Viruses; Riboviria; Orthornavirae; Negarnaviricota; Polyploviricotina; Ellioviricetes; Bunyavirales; Nairoviridae; Orthonairovirus; Kasokero orthonairovirus
GCA_001630045.1	18236	3	Yogue virus	NA	Viruses; ssRNA viruses; ssRNA negative-strand viruses; Bunyaviridae; Nairovirus; unclassified Nairovirus
GCA_003177555.1	7983	7	human papillomavirus 86	NA	Viruses; Monodnaviria; Shotokuvirae; Cossaviricota; Papovaviricetes; Zurhausenvirales; Papillomaviridae; Firstpapillomavirinae; Alphapapillomavirus; Alphapapillomavirus 3
GCA_000884275.1	17036	37	Thermus virus P2377	NA	Viruses; Varidnaviria; Helvetiavirae; Dividoviricota; Laserviricetes; Halopanivirales; Sphaerolipoviridae; Gammasphaerolipovirus
GCA_000842885.1	19604	39	Thermus virus IN93	NA	Viruses; Varidnaviria; Helvetiavirae; Dividoviricota; Laserviricetes; Halopanivirales; Sphaerolipoviridae; Gammasphaerolipovirus
GCA_001461445.1	4781	2	Red clover powdery mildewassociated totivirus 1	NA	Viruses; dsRNA viruses; Totiviridae; unclassified Totiviridae
GCA_001461165.1	4178	2	Red clover powdery mildewassociated totivirus 2	NA	Viruses; dsRNA viruses; Totiviridae; unclassified Totiviridae
GCA_001461285.1	4784	2	Red clover powdery mildewassociated totivirus 3	NA	Viruses; dsRNA viruses; Totiviridae; unclassified Totiviridae
GCA_001461585.1	5056	2	Red clover powdery mildewassociated totivirus 5	NA	Viruses; dsRNA viruses; Totiviridae; unclassified Totiviridae
GCA_001461425.1	5007	2	Red clover powdery mildewassociated totivirus 6	NA	Viruses; dsRNA viruses; Totiviridae; unclassified Totiviridae
GCA_001461105.1	4942	2	Red clover powdery mildewassociated totivirus 7	NA	Viruses; dsRNA viruses; Totiviridae; unclassified Totiviridae
GCA_001461265.1	5997	1	Red clover powdery mildewassociated totivirus 9	NA	Viruses; dsRNA viruses; Totiviridae; unclassified Totiviridae
GCA_014883225.1	20126	3	Gossas virus	NA	Viruses; Riboviria; Orthornavirae; Negarnaviricota; Polyploviricotina; Ellioviricetes; Bunyavirales; Nairoviridae; Orthonairovirus; unclassified Nairovirus
GCA_900098775.1	34080	29	Bottlenose dolphin adenovirus 1	NA	Viruses; Varidnaviria; Bamfordvirae; Preplasmiviricota; Tectiliviricetes; Rowavirales; Adenoviridae; Mastadenovirus; Dolphin mastadenovirus B
GCA_900323385.1	8880	6	Borna disease virus 1	NA	Viruses; Riboviria; Orthornavirae; Negarnaviricota; Haploviricotina; Monjiviricetes; Mononegavirales; Bornaviridae; Orthobornavirus; Mammalian 1 orthobornavirus
GCA_000847565.2	8908	6	Borna disease virus 2	No98	Viruses; Riboviria; Orthornavirae; Negarnaviricota; Haploviricotina; Monjiviricetes; Mononegavirales; Bornaviridae; Orthobornavirus; Mammalian 1 orthobornavirus
GCA_001305565.2	8930	6	Canary bornavirus 1	NA	Viruses; Riboviria; Orthornavirae; Negarnaviricota; Haploviricotina; Monjiviricetes; Mononegavirales; Bornaviridae; Orthobornavirus; Passeriform 1 orthobornavirus
GCA_002366325.1	8957	6	Canary bornavirus 2	NA	Viruses; Riboviria; Orthornavirae; Negarnaviricota; Haploviricotina; Monjiviricetes; Mononegavirales; Bornaviridae; Orthobornavirus; Passeriform 1 orthobornavirus
GCA_000921835.1	8914	6	Canary bornavirus 3	NA	Viruses; Riboviria; Orthornavirae; Negarnaviricota; Haploviricotina; Monjiviricetes; Mononegavirales; Bornaviridae; Orthobornavirus; Passeriform 1 orthobornavirus
GCA_002366045.1	9006	6	Aquatic bird bornavirus 1	NA	Viruses; Riboviria; Orthornavirae; Negarnaviricota; Haploviricotina; Monjiviricetes; Mononegavirales; Bornaviridae; Orthobornavirus; Waterbird 1 orthobornavirus
GCA_001430595.1	35159	36	Simian adenovirus 16	C8	Viruses; dsDNA viruses, no RNA stage; Adenoviridae; Mastadenovirus; unclassified Simian adenoviruses
GCA_006433595.1	35670	36	Simian adenovirus 17	B105	Viruses; Varidnaviria; Bamfordvirae; Preplasmiviricota; Tectiliviricetes; Rowavirales; Adenoviridae; Mastadenovirus; unclassified Simian adenoviruses
GCA_001504115.1	93879	178	Pseudomonas phage K8	NA	Viruses; dsDNA viruses, no RNA stage; Caudovirales; Myoviridae; unclassified Myoviridae
GCA_001551785.1	93464	173	Pseudomonas phage PaoP5	NA	Viruses; dsDNA viruses, no RNA stage; Caudovirales; Myoviridae; unclassified Myoviridae
GCA_004787395.1	154158	74	Lymphocryptovirus MacacapfelclE3	NA	Viruses; Duplodnaviria; Heunggongvirae; Peploviricota; Herviviricetes; Herpesvirales; Herpesviridae; Gammaherpesvirinae; Lymphocryptovirus; Macacine gammaherpesvirus 10
GCA_000839905.1	37618	59	Listeria phage PSA	NA	Viruses; Duplodnaviria; Heunggongvirae; Uroviricota; Caudoviricetes; Caudovirales; Siphoviridae; Psavirus; Listeria virus PSA
GCA_008799335.1	7411	1	Enterovirus B69	Toluca1	Viruses; Riboviria; Orthornavirae; Pisuviricota; Pisoniviricetes; Picornavirales; Picornaviridae; Enterovirus; Enterovirus B
GCA_001505575.1	42265	70	Enterococcus phage IMEEFm5	NA	Viruses; dsDNA viruses, no RNA stage; Caudovirales; Siphoviridae; unclassified Siphoviridae
GCA_001504915.1	54439	92	Paenibacillus phage Tripp	NA	Viruses; dsDNA viruses, no RNA stage; Caudovirales; Siphoviridae; unclassified Siphoviridae
GCA_001482935.1	53141	96	Mycobacterium phage LadyBird	NA	Viruses; dsDNA viruses, no RNA stage; Caudovirales; Siphoviridae; unclassified Siphoviridae

gca	genome.size	proteome.size	phage.name	strain	Taxonomy
GCA_001503295.1	50614	91	Mycobacterium phage Pari	NA	Viruses; dsDNA viruses, no RNA stage; Caudovirales; Siphoviridae; unclassified Siphoviridae
GCA_001504095.2	51543	87	Mycobacterium phage Theia	NA	Viruses; dsDNA viruses, no RNA stage; Caudovirales; Siphoviridae; unclassified Siphoviridae
GCA_002757015.1	53746	85	Mycobacterium phage Evanesce	NA	Viruses; Duplodnaviria; Heunggongvirae; Uroviricota; Caudoviricetes; Caudovirales; Siphoviridae; Gilesvirus; Mycobacterium virus Giles
GCA_002600305.1	68596	101	Mycobacterium phage Squid	NA	Viruses; Duplodnaviria; Heunggongvirae; Uroviricota; Caudoviricetes; Caudovirales; Siphoviridae; Bclasvirinae; Pegunavirus; Mycobacterium virus Soto
GCA_002597985.1	51374	87	Mycobacterium phage Caelakin	NA	Viruses; Duplodnaviria; Heunggongvirae; Uroviricota; Caudoviricetes; Caudovirales; Siphoviridae; Fromanvirus; Mycobacterium virus Peaches
GCA_001505555.1	62181	88	Pseudomonas phage PAE1	NA	Viruses; dsDNA viruses, no RNA stage; Caudovirales; Siphoviridae; unclassified Siphoviridae
GCA_001754265.1	46877	86	Brucella phage BiPBO1	NA	Viruses; unclassified phages
GCA_002607445.1	36733	46	Streptococcus phage 73	NA	Viruses; Duplodnaviria; Heunggongvirae; Uroviricota; Caudoviricetes; Caudovirales; Siphoviridae; Moineauvirus; unclassified Moineauvirus
GCA_002607465.1	34239	41	Streptococcus phage 53	NA	Viruses; Duplodnaviria; Heunggongvirae; Uroviricota; Caudoviricetes; Caudovirales; Siphoviridae; Moineauvirus; unclassified Moineauvirus
GCA_002607485.1	34593	40	Streptococcus phage 128	NA	Viruses; Duplodnaviria; Heunggongvirae; Uroviricota; Caudoviricetes; Caudovirales; Siphoviridae; Moineauvirus; unclassified Moineauvirus
GCA_002607505.1	55349	81	Pseudomonas phage POR1	NA	Viruses; Duplodnaviria; Heunggongvirae; Uroviricota; Caudoviricetes; Caudovirales; Myoviridae; unclassified Myoviridae
GCA_001504895.1	76271	128	Mycobacterium phage Archie	NA	Viruses; dsDNA viruses, no RNA stage; Caudovirales; Siphoviridae; unclassified Siphoviridae
GCA_001503275.1	58065	94	Mycobacterium phage Mufasa	NA	Viruses; dsDNA viruses, no RNA stage; Caudovirales; Siphoviridae; unclassified Siphoviridae
GCA_002603405.1	81128	139	Mycobacterium phage Bricole	NA	Viruses; Duplodnaviria; Heunggongvirae; Uroviricota; Caudoviricetes; Caudovirales; Siphoviridae; Mclasvirinae; Bongovirus; unclassified Bongovirus
GCA_001500855.2	90743	138	Escherichia phage SUSP1	NA	Viruses; dsDNA viruses, no RNA stage; Caudovirales; Myoviridae; unclassified Myoviridae
GCA_001502895.2	88698	132	Escherichia phage SUSP2	NA	Viruses; dsDNA viruses, no RNA stage; Caudovirales; Myoviridae; unclassified Myoviridae
GCA_002607525.1	41496	63	Acinetobacter phage Ab1051phi	NA	Viruses; Duplodnaviria; Heunggongvirae; Uroviricota; Caudoviricetes; Caudovirales; Myoviridae; unclassified Myoviridae
GCA_002607545.1	61304	93	Acinetobacter phage Ab1052phi	NA	Viruses; Duplodnaviria; Heunggongvirae; Uroviricota; Caudoviricetes; Caudovirales; Siphoviridae; Vieuvirus; unclassified Vieuvirus
GCA_003673825.1	9041	3	Soybean leafassociated negativestranded RNA virus 1	NA	Viruses; Riboviria; Orthornavirae; Negarnaviricota; Haploviricotina; Monjiviricetes; Mononegavirales; Mymonaviridae; Sclerotimonavirus; Glycine sclerotimonavirus
GCA_003673845.1	7321	1	Soybean leafassociated negativestranded RNA virus 2	NA	Viruses; Riboviria; Orthornavirae; Negarnaviricota; Haploviricotina; Monjiviricetes; Mononegavirales; Mymonaviridae; Sclerotimonavirus; Illinois sclerotimonavirus
GCA_003673865.1	6218	1	Soybean leafassociated negativestranded RNA virus 3	NA	Viruses; Riboviria; Orthornavirae; Negarnaviricota; Haploviricotina; Monjiviricetes; Mononegavirales; Mymonaviridae; Sclerotimonavirus; Dadou sclerotimonavirus
GCA_003673885.1	5317	1	Soybean leafassociated negativestranded RNA virus 4	NA	Viruses; Riboviria; Orthornavirae; Negarnaviricota; Haploviricotina; Monjiviricetes; Mononegavirales; Mymonaviridae; Sclerotimonavirus; Phyllosphere sclerotimonavirus
GCA_001470815.1	142987	245	Klebsiella phage vB_KpnM_KB57	NA	Viruses; dsDNA viruses, no RNA stage; Caudovirales; Myoviridae; unclassified Myoviridae
GCA_008800295.1	7389	1	Baboon enterovirus	A13	Viruses; Riboviria; Orthornavirae; Pisuviricota; Pisoniviricetes; Picornavirales; Picornaviridae; Enterovirus; Enterovirus A
GCA_008800275.1	7378	1	Simian agent 5	B165	Viruses; Riboviria; Orthornavirae; Pisuviricota; Pisoniviricetes; Picornavirales; Picornaviridae; Enterovirus; Enterovirus B
GCA_001502275.1	61235	90	Escherichia phage slur01	NA	Viruses; dsDNA viruses, no RNA stage; Caudovirales; Siphoviridae; unclassified Siphoviridae
GCA_001501495.1	167298	266	Escherichia phage slur02	NA	Viruses; dsDNA viruses, no RNA stage; Caudovirales; Myoviridae; Tevenvirinae; T4virus; unclassified T4virus
GCA_001433645.1	136896	206	Escherichia phage slur16	NA	Viruses; dsDNA viruses, no RNA stage; Caudovirales; Myoviridae; unclassified Myoviridae
GCA_003147245.1	167467	272	Escherichia phage slur03	NA	Viruses; Duplodnaviria; Heunggongvirae; Uroviricota; Caudoviricetes; Caudovirales; Myoviridae; Tevenvirinae; Tequatrovirus; Escherichia virus slur03
GCA_003147265.1	167298	266	Escherichia phage slur04	NA	Viruses; Duplodnaviria; Heunggongvirae; Uroviricota; Caudoviricetes; Caudovirales; Myoviridae; Tevenvirinae; Tequatrovirus; Escherichia virus slur04
GCA_001500835.1	43900	58	Escherichia phage slur05	NA	Viruses; dsDNA viruses, no RNA stage; Caudovirales; Siphoviridae; unclassified Siphoviridae
GCA_003147285.1	44515	58	Escherichia phage slur06	NA	Viruses; Duplodnaviria; Heunggongvirae; Uroviricota; Caudoviricetes; Caudovirales; Siphoviridae; Dhillonvirus; unclassified Dhillonvirus
GCA_001502875.1	167124	266	Escherichia phage slur07	NA	Viruses; dsDNA viruses, no RNA stage; Caudovirales; Myoviridae; Tevenvirinae; T4virus; unclassified T4virus
GCA_003147305.1	167467	273	Escherichia phage slur08	NA	Viruses; dsDNA viruses, no RNA stage; Caudovirales; Myoviridae; Tevenvirinae; T4virus; unclassified T4virus

gca	genome.size	proteome.size	phage.name	strain	Taxonomy
GCA_003147325.1	167298	266	Escherichia phage slur11	NA	Viruses; Duplodnaviria; Heunggongvirae; Uroviricota; Caudoviricetes; Caudovirales; Myoviridae; Tevenvirinae; Tequatrovirus; unclassified Tequatrovirus
GCA_003147345.1	136898	207	Escherichia phage slur12	NA	Viruses; Duplodnaviria; Heunggongvirae; Uroviricota; Caudoviricetes; Caudovirales; Myoviridae; Vequintavirinae; Vequintavirus; unclassified Vequintavirus
GCA_001447045.1	167467	272	Escherichia phage slur14	NA	Viruses; Duplodnaviria; Heunggongvirae; Uroviricota; Caudoviricetes; Caudovirales; Myoviridae; Tevenvirinae; Tequatrovirus; unclassified Tequatrovirus
GCA_003147365.1	167299	266	Escherichia phage slur13	NA	Viruses; Duplodnaviria; Heunggongvirae; Uroviricota; Caudoviricetes; Caudovirales; Myoviridae; Tevenvirinae; Tequatrovirus; unclassified Tequatrovirus
GCA_003147385.1	41318	51	Clostridium phage slur17	NA	Viruses; Duplodnaviria; Heunggongvirae; Uroviricota; Caudoviricetes; Caudovirales; Siphoviridae; unclassified Siphoviridae
GCA_001806195.1	360610	296	Golden Marseillevirus	NA	Viruses; Varidnaviria; Bamfordvirae; Nucleocytoviricota; Megaviricetes; Pimascovirales; Marseilleviridae; Marseillevirus; unclassified Marseillevirus
GCA_001308735.1	11649	1	Bellflower vein chlorosis virus	NA	Viruses; ssRNA viruses; ssRNA positive-strand viruses, no DNA stage; Picornavirales; Secoviridae; Waikavirus; unclassified Waikavirus
GCA_001433565.1	5547	3	Tasmanian aquabirnavirus	NA	Viruses; dsRNA viruses; Birnaviridae; Aquabirnavirus; unclassified Aquabirnavirus
GCA_000863365.1	10685	1	Alkhumra hemorrhagic fever virus	1176	Viruses; ssRNA viruses; ssRNA positive-strand viruses, no DNA stage; Flaviviridae; Flavivirus; tick-borne encephalitis virus group; Kyasanur forest disease virus
GCA_000837345.1	8303	8	Blueberry red ringspot virus	NA	Viruses; Riboviria; Pararnavirae; Artverviricota; Revtraviricetes; Ortervirales; Caulimoviridae; Soymovirus
GCA_002821885.1	2767	7	Chilli leaf curl virus	NA	Viruses; ssDNA viruses; Geminiviridae; Begomovirus
GCA_000846945.1	5610	2	Bovine parvovirus 2	NA	Viruses; Monodnaviria; Shotokuvirae; Cossaviricota; Quintoviricetes; Piccovirales; Parvoviridae; Parvovirinae; Copiparvovirus; Ungulate copiparvovirus 1
GCA_002827445.1	5276	2	Bovine parvovirus 3	NA	Viruses; Monodnaviria; Shotokuvirae; Cossaviricota; Quintoviricetes; Piccovirales; Parvoviridae; Parvovirinae; Erythroparvovirus; Ungulate erythroparvovirus 1
GCA_008767215.1	7603	1	Ljungan virus	Fuz1	Viruses; ssRNA viruses; ssRNA positive-strand viruses, no DNA stage; Picornavirales; Picornaviridae; Parechovirus; unclassified Parechovirus
GCA_002823925.1	2623	5	Tomato chlorotic mottle virus	NA	Viruses; ssDNA viruses; Geminiviridae; Begomovirus
GCA_002867675.1	5229	7	Tomato mottle leaf curl virus	NA	Viruses; ssDNA viruses; Geminiviridae; Begomovirus
GCA_001629905.1	3674	5	Potato necrosis virus	NA	Viruses; ssRNA viruses; ssRNA positive-strand viruses, no DNA stage; Tombusviridae; unclassified Tombusviridae
GCA_013086635.1	12327	4	Alcube virus	S20	Viruses; Riboviria; Orthornavirae; Negarnaviricota; Polyploviricotina; Ellioviricetes; Bunyavirales; Phenuiviridae; Phlebovirus; Alcube phlebovirus
GCA_000857465.1	46339	67	Yersinia phage PY54	NA	Viruses; dsDNA viruses, no RNA stage; Caudovirales; Siphoviridae; unclassified Siphoviridae
GCA_006437315.1	43347	37	Fowl aviadenovirus 11	380	Viruses; Varidnaviria; Bamfordvirae; Preplasmiviricota; Tectiliviricetes; Rowavirales; Adenoviridae; Aviadenovirus; Fowl aviadenovirus D
GCA_006437415.1	43632	37	Fowl aviadenovirus 2	SR48	Viruses; Varidnaviria; Bamfordvirae; Preplasmiviricota; Tectiliviricetes; Rowavirales; Adenoviridae; Aviadenovirus; Fowl aviadenovirus D
GCA_006437395.1	43337	37	Fowl aviadenovirus 3	SR49	Viruses; Varidnaviria; Bamfordvirae; Preplasmiviricota; Tectiliviricetes; Rowavirales; Adenoviridae; Aviadenovirus; Fowl aviadenovirus D
GCA_000907375.1	45781	39	Fowl aviadenovirus 5	340	Viruses; dsDNA viruses, no RNA stage; Adenoviridae; Aviadenovirus; Fowl aviadenovirus B
GCA_002817995.1	43810	37	Fowl aviadenovirus 6	CR119	Viruses; Varidnaviria; Bamfordvirae; Preplasmiviricota; Tectiliviricetes; Rowavirales; Adenoviridae; Aviadenovirus; Fowl aviadenovirus E
GCA_006437375.1	43525	37	Fowl aviadenovirus 7	YR36	Viruses; Varidnaviria; Bamfordvirae; Preplasmiviricota; Tectiliviricetes; Rowavirales; Adenoviridae; Aviadenovirus; Fowl aviadenovirus E
GCA_000886795.1	45063	34	Fowl aviadenovirus 9	NA	Viruses; Varidnaviria; Bamfordvirae; Preplasmiviricota; Tectiliviricetes; Rowavirales; Adenoviridae; Aviadenovirus; Fowl aviadenovirus D
GCA_001504595.1	111751	160	Escherichia phage slur09	NA	Viruses; dsDNA viruses, no RNA stage; Caudovirales; Siphoviridae; T5virus; unclassified T5likevirus
GCA_004130595.1	5206	2	Alternaria arborescens victorivirus 1	NA	Viruses; Riboviria; Orthornavirae; Duplornaviricota; Chrymotiviricetes; Ghabrivirales; Totiviridae; Victorivirus; unclassified Victorivirus
GCA_001310135.2	9867	1	Human pegivirus 2	NA	Viruses; ssRNA viruses; ssRNA positive-strand viruses, no DNA stage; Flaviviridae; Pegivirus; unclassified Pegivirus
GCA_001310215.1	3956	2	Maizeassociated totivirus 1	NA	Viruses; dsRNA viruses; Totiviridae; unclassified Totiviridae
GCA_900007805.1	40466	50	Pseudomonas phage shl2	NA	Viruses; Duplodnaviria; Heunggongvirae; Uroviricota; Caudoviricetes; Caudovirales; Autographiviridae; Studiervirinae; Ghunavirus; Pseudomonas virus shl2
GCA_001551465.1	98765	172	Pseudomonas phage VCM	NA	Viruses; dsDNA viruses, no RNA stage; Caudovirales; Myoviridae; unclassified Myoviridae
GCA_000866805.1	23015	3	Chum salmon reovirus CS	NA	Viruses; dsRNA viruses; Reoviridae; Spinareovirinae; Aquareovirus; Aquareovirus A
GCA_001551345.1	127726	156	Staphylococcus phage SPbetalike	NA	Viruses; dsDNA viruses, no RNA stage; Caudovirales; Siphoviridae; Spbetavirus; unclassified Spbetalikevirus

gca	genome.size	proteome.size	phage.name	strain	Taxonomy
GCA_001504075.1	40670	59	Staphylococcus phage StB20like	NA	Viruses; dsDNA viruses, no RNA stage; Caudovirales; Siphoviridae; unclassified Siphoviridae
GCA_001502255.1	22613	54	Acidianus bottleshaped virus 2	ABV2	Viruses; dsDNA viruses, no RNA stage; Ampullaviridae; Ampullavirus; unclassified Ampullavirus
GCA_001501475.1	28489	66	Acidianus bottleshaped virus 3	ABV3	Viruses; dsDNA viruses, no RNA stage; Ampullaviridae; Ampullavirus; unclassified Ampullavirus
GCA_001579495.1	29763	43	Acidianus rodshaped virus 2	ARV2	Viruses; dsDNA viruses, no RNA stage; Ligamenvirales; Rudiviridae; Rudivirus; unclassified Rudivirus
GCA_001470575.1	19351	26	Hydrogenobaculum phage 1	HP1	Viruses; unclassified viruses; unclassified DNA viruses; unclassified dsDNA phages
GCA_001551185.1	64323	87	Sulfolobus monocaudavirus SMV3	NA	Viruses; dsDNA viruses, no RNA stage; unclassified dsDNA viruses
GCA_001500815.1	51711	68	Sulfolobus monocaudavirus SMV4	NA	Viruses; dsDNA viruses, no RNA stage; unclassified dsDNA viruses
GCA_001502855.1	40860	71	Sulfolobales virus YNP1	SYV1	Viruses; unclassified viruses; unclassified DNA viruses; unclassified archaeal dsDNA viruses
GCA_001502235.1	34783	60	Sulfolobales Virus YNP2	SYV2	Viruses; unclassified viruses; unclassified DNA viruses; unclassified archaeal dsDNA viruses
GCA_001316415.1	1767	2	Tadarida brasiliensis circovirus 1	NA	Viruses; ssDNA viruses; Circoviridae; Circovirus; unclassified Circovirus
GCA_006472045.1	349275	410	Portmiou virus	NA	Viruses; Varidnaviria; Bamfordvirae; Nucleocytoviricota; Megaviricetes; Pimascovirales; Marseilleviridae; Lausannevirus
GCA_001505535.1	18609	25	Bacillus phage VMY22	NA	Viruses; dsDNA viruses, no RNA stage; Caudovirales; Podoviridae; unclassified Podoviridae
GCA_000841165.1	40679	65	Salmonella virus ST64T	NA	Viruses; Duplodnaviria; Heunggongvirae; Uroviricota; Caudoviricetes; Caudovirales; Podoviridae; Lederbergvirus
GCA_001963835.1	15099	10	Rat arterivirus 1	NA	Viruses; ssRNA viruses; ssRNA positive-strand viruses, no DNA stage; Nidovirales; Arteriviridae; unclassified Arteriviridae
GCA_002598665.1	15556	26	Arthrobacter phage TymAbreu	NA	Viruses; Duplodnaviria; Heunggongvirae; Uroviricota; Caudoviricetes; Caudovirales; Siphoviridae; Decurrovirus; unclassified Decurrovirus
GCA_001470075.1	50515	86	Mycobacterium phage Pepe	NA	Viruses; dsDNA viruses, no RNA stage; Caudovirales; Siphoviridae; unclassified Siphoviridae
GCA_002607565.1	35838	58	Bacillus phage BMBtp1	NA	Viruses; Duplodnaviria; Heunggongvirae; Uroviricota; Caudoviricetes; Caudovirales; Siphoviridae; unclassified Siphoviridae
GCA_002607585.1	146135	229	Roseobacter phage DSS3P8	NA	Viruses; Duplodnaviria; Heunggongvirae; Uroviricota; Caudoviricetes; Caudovirales; Siphoviridae; unclassified Siphoviridae
GCA_002745315.1	47319	75	Escherichia phage C119	NA	Viruses; Duplodnaviria; Heunggongvirae; Uroviricota; Caudoviricetes; Caudovirales; Drexlerviridae; Rogunavirinae; Rogunavirus; Escherichia virus C119
GCA_001500795.2	41243	54	Acinetobacter phage vB_AbaM_IME200	NA	Viruses; dsDNA viruses, no RNA stage; Caudovirales; Myoviridae; unclassified Myoviridae
GCA_001470035.1	94109	172	Pseudomonas phage C11	NA	Viruses; dsDNA viruses, no RNA stage; Caudovirales; Myoviridae; unclassified Myoviridae
GCA_001470735.1	39235	47	Yersinia phage vB_YenP_AP10	NA	Viruses; dsDNA viruses, no RNA stage; Caudovirales; Podoviridae; unclassified Podoviridae
GCA_002607605.1	41960	68	Staphylococcus phage P282	NA	Viruses; Duplodnaviria; Heunggongvirae; Uroviricota; Caudoviricetes; Caudovirales; Siphoviridae; Biseptimavirus; unclassified Biseptimavirus
GCA_002607625.1	40448	64	Staphylococcus phage P630	NA	Viruses; Duplodnaviria; Heunggongvirae; Uroviricota; Caudoviricetes; Caudovirales; Siphoviridae; Biseptimavirus; unclassified Biseptimavirus
GCA_009901735.1	43649	69	Burkholderia phage PE067	NA	Viruses; Duplodnaviria; Heunggongvirae; Uroviricota; Caudoviricetes; Caudovirales; Myoviridae; unclassified Myoviridae
GCA_001717355.1	31314	47	Haloarcula californiae icosahedral virus 1	SS136	Viruses; Virus families not assigned to an order; Sphaerolipoviridae; unclassified Sphaerolipoviridae
GCA_001401365.1	1705	1	Torulaspora delbrueckii dsRNA Mbarr1 killer virus	EX1180	Viruses; unclassified viruses
GCA_001669805.1	1367	1	Chayote yellow mosaic Benin betasatellite	NA	Viruses; Tolecusatellitidae; Betasatellite; Momordica yellow mosaic betasatellite
GCA_000838205.2	43106	56	Temperate phage phiNIH11	NA	Viruses; dsDNA viruses, no RNA stage; Caudovirales; Siphoviridae; unclassified Siphoviridae
GCA_002597305.1	65760	91	Pbunalikevirus phiFenriz	NA	Viruses; Duplodnaviria; Heunggongvirae; Uroviricota; Caudoviricetes; Caudovirales; Myoviridae; Pbunavirus; Pseudomonas virus PB1
GCA_002597285.1	65763	90	Pbunalikevirus phiHabibi	NA	Viruses; Duplodnaviria; Heunggongvirae; Uroviricota; Caudoviricetes; Caudovirales; Myoviridae; Pbunavirus; Pseudomonas virus PB1
GCA_002597265.1	65908	90	Pbunalikevirus phiMoody	NA	Viruses; Duplodnaviria; Heunggongvirae; Uroviricota; Caudoviricetes; Caudovirales; Myoviridae; Pbunavirus; Pseudomonas virus PB1
GCA_002597245.1	65762	90	Pbunalikevirus phiVader	NA	Viruses; Duplodnaviria; Heunggongvirae; Uroviricota; Caudoviricetes; Caudovirales; Myoviridae; Pbunavirus; Pseudomonas virus PB1
GCA_001430175.1	5032	5	Bank vole polyomavirus	NA	Viruses; dsDNA viruses, no RNA stage; Polyomaviridae; unclassified Polyomaviridae

gca	genome.size	proteome.size	phage.name	strain	Taxonomy
GCA_001430575.1	5024	5	Common vole polyomavirus	NA	Viruses; dsDNA viruses, no RNA stage; Polyomaviridae; unclassified Polyomaviridae
GCA_001876915.1	5172	7	Passionfruit leaf distortion virus	NA	Viruses; ssDNA viruses; Geminiviridae; Begomovirus; unclassified Begomovirus
GCA_001634555.1	15247	14	Free State vervet virus	NA	Viruses; Riboviria; Orthornavirae; Pisuviricota; Pisoniviricetes; Nidovirales; Arnidovirineae; Arteriviridae; Simarterivirinae; Epsilonarterivirus; Sheartevirus; Epsilonarterivirus safriver
GCA_001441075.1	2754	5	Andrographis yellow vein leaf curl virus	NA	Viruses; ssDNA viruses; Geminiviridae; Begomovirus; unclassified Begomovirus
GCA_000840645.2	31007	37	Methanothermobacter phage psiM100	NA	Viruses; dsDNA viruses, no RNA stage; Caudovirales; Siphoviridae; unclassified Siphoviridae
GCA_001504875.1	39474	62	Lactobacillus phage CL1	NA	Viruses; dsDNA viruses, no RNA stage; Caudovirales; Siphoviridae; unclassified Siphoviridae
GCA_001503255.1	38751	60	Lactobacillus phage CL2	NA	Viruses; dsDNA viruses, no RNA stage; Caudovirales; Siphoviridae; unclassified Siphoviridae
GCA_001504055.1	34155	50	Lactobacillus phage iA2	NA	Viruses; dsDNA viruses, no RNA stage; Caudovirales; Siphoviridae; unclassified Siphoviridae
GCA_001505515.1	39399	60	Lactobacillus phage iLp84	NA	Viruses; dsDNA viruses, no RNA stage; Caudovirales; Siphoviridae; unclassified Siphoviridae
GCA_001504855.1	34176	50	Lactobacillus phage iLp1308	NA	Viruses; dsDNA viruses, no RNA stage; Caudovirales; Siphoviridae; unclassified Siphoviridae
GCA_000860925.1	12138	2	Cycas necrotic stunt virus	NA	Viruses; ssRNA viruses; ssRNA positive-strand viruses, no DNA stage; Picornavirales; Secoviridae; Comovirinae; Nepovirus; Subgroup B
GCA_001470615.1	56275	111	Mycobacterium phage Sparkdehlily	NA	Viruses; dsDNA viruses, no RNA stage; Caudovirales; Siphoviridae; unclassified Siphoviridae
GCA_001470315.1	56669	101	Mycobacterium phage Cabrinians	NA	Viruses; dsDNA viruses, no RNA stage; Caudovirales; Siphoviridae; unclassified Siphoviridae
GCA_002607645.1	255569	318	Bacillus phage vB_BpuMBpSp	NA	Viruses; Duplodnaviria; Heunggongvirae; Uroviricota; Caudoviricetes; Caudovirales; Myoviridae; unclassified Myoviridae
GCA_002607685.1	28788	28	Dishui lake virophage 1	NA	Viruses; Varidnaviria; Bamfordvirae; Preplasmiviricota; Maveriviricetes; Priklausovirales; Lavidaviridae; unclassified Lavidaviridae
GCA_002607705.1	89748	78	Flavobacterium phage FpV21	NA	Viruses; Duplodnaviria; Heunggongvirae; Uroviricota; Caudoviricetes; Caudovirales; Podoviridae; Fipvunavirus; unclassified Fipvunavirus
GCA_002607785.1	88308	75	Flavobacterium phage FpV4	NA	Viruses; Duplodnaviria; Heunggongvirae; Uroviricota; Caudoviricetes; Caudovirales; Podoviridae; Fipvunavirus; Flavobacterium virus Fpv4
GCA_002607815.1	42696	58	Flavobacterium phage FpV9	NA	Viruses; Duplodnaviria; Heunggongvirae; Uroviricota; Caudoviricetes; Caudovirales; Siphoviridae; unclassified Siphoviridae
GCA_001806215.1	2759	6	Lisianthus enation leaf curl virus	NA	Viruses; ssDNA viruses; Geminiviridae; Begomovirus; unclassified Begomovirus
GCA_002080235.1	7979	4	Beauveria bassiana polymycovirus 1	NA	Viruses; unclassified viruses
GCA_000854485.1	6966	5	Botrytis virus X	NA	Viruses; ssRNA viruses; ssRNA positive-strand viruses, no DNA stage; Tymovirales; Alphaflexiviridae; Botrexvirus
GCA_000838245.1	7559	6	Citrus yellow mosaic virus	NA	Viruses; Riboviria; Pararnavirae; Artverviricota; Revtraviricetes; Ortervirales; Caulimoviridae; Badnavirus
GCA_001430075.1	5213	7	Melochia mosaic virus	NA	Viruses; ssDNA viruses; Geminiviridae; Begomovirus; unclassified Begomovirus
GCA_001430275.1	5288	7	Melochia yellow mosaic virus	NA	Viruses; ssDNA viruses; Geminiviridae; Begomovirus; unclassified Begomovirus
GCA_001551525.2	5378	7	Pavonia yellow mosaic virus	NA	Viruses; ssDNA viruses; Geminiviridae; Begomovirus; unclassified Begomovirus
GCA_001470435.1	39593	56	Pseudomonas phage vB_PaeS_PM105	NA	Viruses; dsDNA viruses, no RNA stage; Caudovirales; Siphoviridae; unclassified Siphoviridae
GCA_001689855.1	2568	2	Gorilla anellovirus	NA	Viruses; ssDNA viruses; Anelloviridae; unclassified Anelloviridae
GCA_013086645.1	11109	3	Brno virus	72012CZE	Viruses; Riboviria; Orthornavirae; Negarnaviricota; Polyploviricotina; Ellioviricetes; Bunyavirales; Hantaviridae; Mammantavirinae; Loanvirus; Brno loanvirus
GCA_001443865.1	18697	1	Beihai barnacle virus 1	BHGZ	Viruses; ssRNA viruses; ssRNA positive-strand viruses, no DNA stage; unclassified ssRNA positive-strand viruses
GCA_001444065.1	16248	1	Bole tick virus 4	BLP1	Viruses; ssRNA viruses; ssRNA positive-strand viruses, no DNA stage; unclassified ssRNA positive-strand viruses
GCA_001443745.1	26315	1	Gamboa mosquito virus	GW	Viruses; ssRNA viruses; ssRNA positive-strand viruses, no DNA stage; unclassified ssRNA positive-strand viruses
GCA_001443965.1	22879	1	Sanxia water strider virus 6	SYSZZ2	Viruses; ssRNA viruses; ssRNA positive-strand viruses, no DNA stage; unclassified ssRNA positive-strand viruses
GCA_001444145.1	16053	1	Shayang fly virus 4	SYCY	Viruses; ssRNA viruses; ssRNA positive-strand viruses, no DNA stage; unclassified ssRNA positive-strand viruses
GCA_001443825.1	21414	1	Shayang spider virus 4	SYZZ1	Viruses; ssRNA viruses; ssRNA positive-strand viruses, no DNA stage; unclassified ssRNA positive-strand viruses

gca	genome.size	proteome.size	phage.name	strain	Taxonomy
GCA_001446205.1	10406	6	Shuangao insect virus 7	SKC	Viruses; ssRNA viruses; ssRNA positive-strand viruses, no DNA stage; unclassified ssRNA positive-strand viruses
GCA_001444045.1	18554	1	Shuangao lacewing virus 2	SCL	Viruses; ssRNA viruses; ssRNA positive-strand viruses, no DNA stage; unclassified ssRNA positive-strand viruses
GCA_001443925.1	19537	1	Tacheng tick virus 8	TCP2	Viruses; ssRNA viruses; ssRNA positive-strand viruses, no DNA stage; unclassified ssRNA positive-strand viruses
GCA_001444125.1	9653	1	Wenling shark virus	MHS2	Viruses; ssRNA viruses; ssRNA positive-strand viruses, no DNA stage; unclassified ssRNA positive-strand viruses
GCA_001444165.1	10992	6	Wuhan aphid virus 1	WHYC1	Viruses; ssRNA viruses; ssRNA positive-strand viruses, no DNA stage; unclassified ssRNA positive-strand viruses
GCA_001443945.1	10890	6	Wuhan aphid virus 2	WHYC2	Viruses; ssRNA viruses; ssRNA positive-strand viruses, no DNA stage; unclassified ssRNA positive-strand viruses
GCA_001654325.1	23676	1	Wuhan centipede virus	WHWG11	Viruses; ssRNA viruses; ssRNA positive-strand viruses, no DNA stage; unclassified ssRNA positive-strand viruses
GCA_001446225.1	10485	6	Wuhan cricket virus	WHXS1	Viruses; ssRNA viruses; ssRNA positive-strand viruses, no DNA stage; unclassified ssRNA positive-strand viruses
GCA_001446245.1	10967	6	Wuhan flea virus	WHZM	Viruses; ssRNA viruses; ssRNA positive-strand viruses, no DNA stage; unclassified ssRNA positive-strand viruses
GCA_001443805.1	21779	1	Xingshan cricket virus	XSXS2	Viruses; ssRNA viruses; ssRNA positive-strand viruses, no DNA stage; unclassified ssRNA positive-strand viruses
GCA_001444025.1	24521	1	Xinzhou spider virus 2	XZZZ7	Viruses; ssRNA viruses; ssRNA positive-strand viruses, no DNA stage; unclassified ssRNA positive-strand viruses
GCA_001654425.1	20433	1	Xinzhou spider virus 3	XZZZ3	Viruses; ssRNA viruses; ssRNA positive-strand viruses, no DNA stage; unclassified ssRNA positive-strand viruses
GCA_001470955.1	29407	45	Propionibacterium phage PA114	NA	Viruses; dsDNA viruses, no RNA stage; Caudovirales; Siphoviridae; unclassified Siphoviridae
GCA_002757035.1	46596	66	Rhodococcus phage TWAMP	NA	Viruses; Duplodnaviria; Heunggongvirae; Uroviricota; Caudoviricetes; Caudovirales; Siphoviridae; Rerduovirus; unclassified Rerduovirus
GCA_001470515.1	44203	55	Klebsiella phage vB_KpnP_KpV41	NA	Viruses; dsDNA viruses, no RNA stage; Caudovirales; Podoviridae; unclassified Podoviridae
GCA_002603565.1	145343	198	Cellulophaga phage phi172_18	NA	Viruses; Duplodnaviria; Heunggongvirae; Uroviricota; Caudoviricetes; Caudovirales; Podoviridae; Lightbulbvirus; unclassified Lightbulbvirus
GCA_002757055.1	145865	197	Cellulophaga phage phi41_13	NA	Viruses; Duplodnaviria; Heunggongvirae; Uroviricota; Caudoviricetes; Caudovirales; Podoviridae; Lightbulbvirus; unclassified Lightbulbvirus
GCA_002757075.1	145865	197	Cellulophaga phage phi41_18	NA	Viruses; Duplodnaviria; Heunggongvirae; Uroviricota; Caudoviricetes; Caudovirales; Podoviridae; Lightbulbvirus; Cellulophaga virus Cba41; Cellulophaga phage phi4:1
GCA_002745815.1	42167	46	Aeromonas phage Ahp1	NA	Viruses; Duplodnaviria; Heunggongvirae; Uroviricota; Caudoviricetes; Caudovirales; Autographiviridae; Melnykvirinae; Ahphunavirus; Aeromonas virus Ahp1
GCA_001502835.1	58000	95	Enterococcus phage vB_EfaS_IME198	NA	Viruses; dsDNA viruses, no RNA stage; Caudovirales; Siphoviridae; unclassified Siphoviridae
GCA_001470835.2	18655	27	Enterococcus phage vB_EfaP_IME195	NA	Viruses; dsDNA viruses, no RNA stage; Caudovirales; Podoviridae; unclassified Podoviridae
GCA_001502215.1	38886	57	Enterococcus phage vB_EfaS_IME196	NA	Viruses; dsDNA viruses, no RNA stage; Caudovirales; Siphoviridae; unclassified Siphoviridae
GCA_001470055.2	41098	67	Enterococcus phage vB_EfaS_IME197	NA	Viruses; dsDNA viruses, no RNA stage; Caudovirales; Siphoviridae; unclassified Siphoviridae
GCA_002607875.1	18838	22	Enterococcus phage vB_EfaP_IME199	NA	Viruses; Duplodnaviria; Heunggongvirae; Uroviricota; Caudoviricetes; Caudovirales; Podoviridae; unclassified Podoviridae
GCA_002757095.1	41720	53	Salmonella phage fSE1C	NA	Viruses; Duplodnaviria; Heunggongvirae; Uroviricota; Caudoviricetes; Caudovirales; Siphoviridae; Guernseyvirinae; Jerseyvirus; Salmonella virus f18SE; Salmonella phage f18SE
GCA_003033185.1	7409	6	Cervus elaphus papillomavirus 2	NA	Viruses; Monodnaviria; Shotokuvirae; Cossaviricota; Papovaviricetes; Zurhausenvirales; Papillomaviridae; Firstpapillomavirinae; Xipapillomavirus; Xipapillomavirus 5
GCA_003033135.1	7138	6	Pudu puda papillomavirus 1	NA	Viruses; Monodnaviria; Shotokuvirae; Cossaviricota; Papovaviricetes; Zurhausenvirales; Papillomaviridae; Firstpapillomavirinae; Dyokappapapillomavirus; Dyokappapapillomavirus 5
GCA_003029265.2	5254	7	Cnidoscolus mosaic leaf deformation virus	NA	Viruses; Monodnaviria; Shotokuvirae; Cressdnaviricota; Repensiviricetes; Geplafuvirales; Geminiviridae; Begomovirus
GCA_002757115.1	248605	380	Vibrio phage phiGrn1	NA	Viruses; Duplodnaviria; Heunggongvirae; Uroviricota; Caudoviricetes; Caudovirales; Myoviridae; Tevenvirinae; Schizotequatrovirus; unclassified Schizotequatrovirus
GCA_002757135.1	250485	383	Vibrio phage phiST2	NA	Viruses; Duplodnaviria; Heunggongvirae; Uroviricota; Caudoviricetes; Caudovirales; Myoviridae; Tevenvirinae; Schizotequatrovirus; unclassified Schizotequatrovirus
GCA_002607905.1	47457	61	Aeromonas phage phiARM81ld	NA	Viruses; Duplodnaviria; Heunggongvirae; Uroviricota; Caudoviricetes; Caudovirales; Myoviridae; unclassified Myoviridae
GCA_001567015.1	111246	118	Cnaphalocrocis medinalis granulovirus	Enping	Viruses; dsDNA viruses, no RNA stage; Baculoviridae; Betabaculovirus; unclassified Betabaculovirus
GCA_001448395.1	9850	1	Blueberry fruit drop associated virus	NA	Viruses; Riboviria; Pararnavirae; Artverviricota; Revtraviricetes; Ortervirales; Caulimoviridae; Vaccinivirus
GCA_002623365.1	63560	86	Pseudomonas phage phiR18	NA	Viruses; Duplodnaviria; Heunggongvirae; Uroviricota; Caudoviricetes; Caudovirales; Podoviridae; Kochitakasuvirus; Pseudomonas virus R18

gca	genome.size	proteome.size	phage.name	strain	Taxonomy
GCA_002607935.1	66257	94	Pseudomonas phage S121	NA	Viruses; Duplodnaviria; Heunggongvirae; Uroviricota; Caudoviricetes; Caudovirales; Myoviridae; Pbunavirus; unclassified Pbunavirus
GCA_002607975.1	60012	70	Aeromonas phage phiARM81mr	NA	Viruses; Duplodnaviria; Heunggongvirae; Uroviricota; Caudoviricetes; Caudovirales; Podoviridae; unclassified Podoviridae
GCA_002608035.1	57218	69	Pseudomonas phage phi1	NA	Viruses; Duplodnaviria; Heunggongvirae; Uroviricota; Caudoviricetes; Caudovirales; Siphoviridae; unclassified Siphoviridae
GCA_001736495.1	32637	36	Pseudomonas phage phi3	NA	Viruses; dsDNA viruses, no RNA stage; Caudovirales; Myoviridae; unclassified Myoviridae
GCA_001654225.1	1386	1	Parthenium leaf curl alphasatellite	NA	Viruses; Satellites; Satellite Nucleic Acids; Single stranded DNA satellites; Alphasatellites; Begomovirus-associated alphasatellites; unclassified Begomovirus-associated alphasatellites
GCA_001939215.2	1723	2	Indian encephalitis associated cyclovirus	NA	Viruses; ssDNA viruses; Circoviridae; unclassified Circoviridae; suggested genus Cyclovirus
GCA_001501435.1	5181	2	Penicillium aurantiogriseum totivirus 1	NA	Viruses; dsRNA viruses; Totiviridae; unclassified Totiviridae
GCA_000919575.1	9276	1	Sicinivirus A	UCC001	Viruses; ssRNA viruses; ssRNA positive-strand viruses, no DNA stage; Picornavirales; Picornaviridae; Sicinivirus
GCA_002817165.1	7272	1	Kunsagivirus A	rollerSZAL6KuV2011HUN	Viruses; Riboviria; Orthornavirae; Pisuviricota; Pisoniviricetes; Picornavirales; Picornaviridae; Kunsagivirus
GCA_000848905.1	36519	38	Simian adenovirus 25	NA	Viruses; dsDNA viruses, no RNA stage; Adenoviridae; Mastadenovirus; Human mastadenovirus E
GCA_002757155.1	46596	66	Rhodococcus phage Lillie	NA	Viruses; Duplodnaviria; Heunggongvirae; Uroviricota; Caudoviricetes; Caudovirales; Siphoviridae; Rerduovirus; unclassified Rerduovirus
GCA_001551665.1	165031	224	Bacillus phage BM5	NA	Viruses; dsDNA viruses, no RNA stage; Caudovirales; Myoviridae; unclassified Myoviridae
GCA_006448275.1	36535	36	Simian adenovirus 24	ATCC VR593	Viruses; Varidnaviria; Bamfordvirae; Preplasmiviricota; Tectiliviricetes; Rowavirales; Adenoviridae; Mastadenovirus; Human mastadenovirus E
GCA_001471035.1	51637	88	Mycobacterium phage Iracema64	NA	Viruses; dsDNA viruses, no RNA stage; Caudovirales; Siphoviridae; unclassified Siphoviridae
GCA_001500775.2	56451	76	Streptomyces phage phiSAJS1	NA	Viruses; dsDNA viruses, no RNA stage; Caudovirales; Siphoviridae; unclassified Siphoviridae
GCA_001470255.1	60919	113	Pseudomonas phage YMC1102R656	NA	Viruses; dsDNA viruses, no RNA stage; Caudovirales; Siphoviridae; unclassified Siphoviridae
GCA_006448315.1	36462	36	Simian adenovirus E22	ATCC VR591	Viruses; Varidnaviria; Bamfordvirae; Preplasmiviricota; Tectiliviricetes; Rowavirales; Adenoviridae; Mastadenovirus; Human mastadenovirus E
GCA_002630845.1	37714	59	Pseudomonas phage YMC1111R1836	NA	Viruses; Duplodnaviria; Heunggongvirae; Uroviricota; Caudoviricetes; Caudovirales; Siphoviridae; Casadabanvirus; Pseudomonas virus PA1KOR
GCA_001470795.1	41149	49	Pseudomonas phage PPPL1	NA	Viruses; dsDNA viruses, no RNA stage; Caudovirales; Podoviridae; unclassified Podoviridae
GCA_002608065.1	41619	53	Clostridium phage CDSH1	NA	Viruses; Duplodnaviria; Heunggongvirae; Uroviricota; Caudoviricetes; Caudovirales; Siphoviridae; unclassified Siphoviridae
GCA_003033325.1	4281	2	Protoparvovirus Zsana2013HUN	NA	Viruses; Monodnaviria; Shotokuvirae; Cossaviricota; Quintoviricetes; Piccovirales; Parvoviridae; Parvovirinae; Protoparvovirus; Ungulate protoparvovirus 2
GCA_001882295.1	40470	56	Escherichia phage vB_EcoP_GA2A	NA	Viruses; dsDNA viruses, no RNA stage; Caudovirales; Podoviridae; Autographivirinae; T7virus; unclassified T7-like viruses
GCA_000870445.1	7261	3	Dioscorea bacilliform SN virus	NA	Viruses; Riboviria; Pararnavirae; Artverviricota; Revtraviricetes; Ortervirales; Caulimoviridae; Badnavirus
GCA_002608095.1	79099	121	Lactobacillus phage SAC12	NA	Viruses; Duplodnaviria; Heunggongvirae; Uroviricota; Caudoviricetes; Caudovirales; Siphoviridae; Tybeckvirinae; Lenusvirus; Lactobacillus virus SAC12
GCA_001461565.1	6139	2	Penicillium aurantiogriseum fusarivirus 1	NA	Viruses; ssRNA viruses; unclassified ssRNA viruses; Fusariviridae
GCA_001461405.1	3747	1	Penicillium aurantiogriseum foetiduslike virus	NA	Viruses; unclassified viruses
GCA_001461085.1	3912	3	Penicillium aurantiogriseum bipartite virus 1	NA	Viruses; unclassified viruses
GCA_001461145.1	1813	1	Penicillium aurantiogriseum partitilike virus	NA	Viruses; unclassified viruses
GCA_001461545.1	6733	2	Pleospora typhicola fusarivirus 1	NA	Viruses; ssRNA viruses; unclassified ssRNA viruses; Fusariviridae
GCA_001461245.1	12045	4	Penicillium janczewskii chrysovirus 1	NA	Viruses; dsRNA viruses; Chrysoviridae; Chrysovirus; unclassified Chrysovirus
GCA_004790555.1	10929	4	Penicillium janczewskii chrysovirus 2	NA	Viruses; Riboviria; Orthornavirae; Duplornaviricota; Chrymotiviricetes; Ghabrivirales; Chrysoviridae; Betachrysovirus
GCA_001461365.1	3338	2	Penicillium aurantiogriseum partitivirus 1	NA	Viruses; dsRNA viruses; Partitiviridae; unclassified Partitiviridae
GCA_013087325.1	11504	5	Lone star tick rhabdovirus	NA	Viruses; Riboviria; Orthornavirae; Negarnaviricota; Haploviricotina; Monjiviricetes; Mononegavirales; Rhabdoviridae; Lostrhavirus; Lonestar zarhavirus
GCA_001611665.1	4765	3	Megabat bufavirus 1	NA	Viruses; ssDNA viruses; Parvoviridae; Parvovirinae; Protoparvovirus; unclassified Protoparvovirus

gca	genome.size	proteome.size	phage.name	strain	Taxonomy
GCA_001461125.1	8364	1	Rabbit picornavirus	Rabbit012013HUN	Viruses; ssRNA viruses; ssRNA positive-strand viruses, no DNA stage; Picornavirales; Picornaviridae; unclassified Picornaviridae
GCA_001461525.1	7707	5	Rattus norvegicus papillomavirus 3	NA	Viruses; dsDNA viruses, no RNA stage; Papillomaviridae; unclassified Papillomaviridae; non-primate mammal papillomaviruses
GCA_001461665.1	5221	2	Ustilaginoidea virens RNA virus 5	F10338	Viruses; dsRNA viruses; Totiviridae; Victorivirus; unclassified Victorivirus
GCA_001502815.2	5851	7	Phasey bean mild yellows virus	NA	Viruses; ssRNA viruses; ssRNA positive-strand viruses, no DNA stage; Luteoviridae; Polerovirus; unclassified Polerovirus
GCA_001502195.1	12362	7	Colombian potato soilborne virus	NA	Viruses; ssRNA viruses; ssRNA positive-strand viruses, no DNA stage; Virgaviridae; Pomovirus; unclassified Pomovirus
GCA_000845725.1	8253	7	Cestrum yellow leaf curling virus	NA	Viruses; Riboviria; Pararnavirae; Artverviricota; Revtraviricetes; Ortervirales; Caulimoviridae; Soymovirus
GCA_002589945.1	5111	6	Gokushovirus WZ2015a	NA	Viruses; Monodnaviria; Sangervirae; Phixviricota; Malgrandaviricetes; Petitvirales; Microviridae; Gokushovirinae; unclassified Gokushovirinae
GCA_001461505.1	11343	5	Tongilchon virus 1	A122676ROK2012	Viruses; unclassified viruses
GCA_001461225.1	9632	3	Daeseongdong virus 1	A122708ROK2012	Viruses; unclassified viruses
GCA_001461345.1	4742	2	Daeseongdong virus 2	A122549ROK2012	Viruses; unclassified viruses
GCA_001461645.1	11877	3	Imjin River virus 1	A122496ROK2012	Viruses; unclassified viruses
GCA_000841985.1	40149	56	Salmonella phage ST64B	NA	Viruses; dsDNA viruses, no RNA stage; Caudovirales; unclassified Caudovirales
GCA_002608155.1	52291	70	Escherichia phage RacSA53	NA	Viruses; Duplodnaviria; Heunggongvirae; Uroviricota; Caudoviricetes; Caudovirales; Myoviridae; unclassified Myoviridae
GCA_000838145.1	40900	73	Sulfolobus islandicus filamentous virus	NA	Viruses; dsDNA viruses, no RNA stage; Ligamenvirales; Lipothrixviridae; Betalipothrixvirus
GCA_001461485.1	6393	4	Tomato brown rugose fruit virus	NA	Viruses; ssRNA viruses; ssRNA positive-strand viruses, no DNA stage; Virgaviridae; Tobamovirus; unclassified Tobamovirus
GCA_001465105.1	5163	6	Pan troglodytes verus polyomavirus 8	NA	Viruses; dsDNA viruses, no RNA stage; Polyomaviridae; unclassified Polyomaviridae
GCA_001465505.1	4634	3	Rat bufavirus SY2015	NA	Viruses; ssDNA viruses; Parvoviridae; Parvovirinae; Protoparvovirus; unclassified Protoparvovirus
GCA_004789915.1	11481	4	Guertu virus	DXM	Viruses; Riboviria; Orthornavirae; Negarnaviricota; Polyploviricotina; Ellioviricetes; Bunyavirales; Phenuiviridae; Bandavirus; Guertu bandavirus
GCA_009731955.1	12164	3	Mahogany hammock virus	FE42s	Viruses; Riboviria; Orthornavirae; Negarnaviricota; Polyploviricotina; Ellioviricetes; Bunyavirales; Peribunyaviridae; Orthobunyavirus; Guama orthobunyavirus
GCA_018594785.1	12118	3	Gan Gan virus	NA	Viruses; Riboviria; Orthornavirae; Negarnaviricota; Polyploviricotina; Ellioviricetes; Bunyavirales; Peribunyaviridae; Orthobunyavirus; Gan Gan orthobunyavirus
GCA_001629845.2	12484	4	Toros virus	213	Viruses; ssRNA viruses; ssRNA negative-strand viruses; Bunyaviridae; Phlebovirus; unclassified Phlebovirus
GCA_001629925.2	12512	4	Zerdali virus	37	Viruses; ssRNA viruses; ssRNA negative-strand viruses; Bunyaviridae; Phlebovirus; unclassified Phlebovirus
GCA_013086655.1	12659	4	Dashli virus	131	Viruses; Riboviria; Orthornavirae; Negarnaviricota; Polyploviricotina; Ellioviricetes; Bunyavirales; Phenuiviridae; Phlebovirus; Dashli phlebovirus
GCA_001654085.1	5100	2	Nigrospora oryzae victorivirus 1	NA	Viruses; dsRNA viruses; Totiviridae; Victorivirus; unclassified Victorivirus
GCA_001579375.1	5713	2	Camponotus nipponicus virus	SS2014a	Viruses; dsRNA viruses; Totiviridae; unclassified Totiviridae
GCA_000838105.1	212482	468	Invertebrate iridescent virus 6	NA	Viruses; dsDNA viruses, no RNA stage; Iridoviridae; Iridovirus
GCA_006449455.1	112565	135	Giant seaperch iridovirus	NA	Viruses; Varidnaviria; Bamfordvirae; Nucleocytoviricota; Megaviricetes; Pimascovirales; Iridoviridae; Alphairidovirinae; Megalocytivirus; Infectious spleen and kidney necrosis virus
GCA_001501415.1	28111	9	Swine enteric coronavirus	Italy2133062009	Viruses; ssRNA viruses; ssRNA positive-strand viruses, no DNA stage; Nidovirales; Coronaviridae; Coronavirinae; Alphacoronavirus; unclassified Alphacoronavirus
GCA_001500755.1	8506	1	Gallivirus PfCHK1GV	NA	Viruses; ssRNA viruses; ssRNA positive-strand viruses, no DNA stage; Picornavirales; Picornaviridae; Gallivirus; unclassified Gallivirus
GCA_001502795.1	7270	1	Avisivirus PfCHK1AsV	NA	Viruses; ssRNA viruses; ssRNA positive-strand viruses, no DNA stage; Picornavirales; Picornaviridae; unclassified Picornaviridae
GCA_013122655.1	99290	87	Largemouth bass virus	Pine 14204	Viruses; Varidnaviria; Bamfordvirae; Nucleocytoviricota; Megaviricetes; Pimascovirales; Iridoviridae; Alphairidovirinae; Ranavirus; Santee-Cooper ranavirus
GCA_013087335.1	8204	1	Phacovirus PfCHK1PhV	NA	Viruses; Riboviria; Orthornavirae; Pisuviricota; Pisoniviricetes; Picornavirales; Picornaviridae; unclassified Picornaviridae
GCA_004790535.1	8896	3	Colletotrichum gloeosporioides chrysovirus 1	NA	Viruses; Riboviria; Orthornavirae; Duplornaviricota; Chrymotiviricetes; Ghabrivirales; Chrysoviridae; Alphachrysovirus; Colletotrichum gloeosporioides chrysovirus
GCA_001550805.1	6656	3	Alternaria brassicicola fusarivirus 1	NA	Viruses; unclassified viruses

gca	genome.size	proteome.size	phage.name	strain	Taxonomy
GCA_001550845.1	2965	6	Turnip leaf roll virus	NA	Viruses; ssDNA viruses; Geminiviridae; Turncurtovirus; unclassified Turncurtovirus
GCA_001502175.1	7417	1	Tupaia hepatovirus A	NA	Viruses; ssRNA viruses; ssRNA positive-strand viruses, no DNA stage; Picornavirales; Picornaviridae; Hepatovirus; unclassified Hepatovirus
GCA_001654145.1	3120	2	Sinapis alba cryptic virus 1	NA	Viruses; dsRNA viruses; Partitiviridae; unclassified Partitiviridae
GCA_002990995.1	14961	21	Bacillus phage pGIL02	AM6552	Viruses; Varidnaviria; Bamfordvirae; Preplasmiviricota; Tectiliviricetes; Kalamavirales; Tectiviridae; Betatectivirus; Bacillus virus Bam35
GCA_009731975.1	6613	1	El Huayo virus	IQT110	Viruses; Riboviria; Orthornavirae; Negarnaviricota; Polyploviricotina; Ellioviricetes; Bunyavirales; Peribunyaviridae; Orthobunyavirus; Caraparu orthobunyavirus
GCA_001646035.1	8919	2	Anopheles C virus	NA	Viruses; ssRNA viruses; ssRNA positive-strand viruses, no DNA stage; Picornavirales; Dicistroviridae; Cripavirus; unclassified Cripavirus
GCA_002608235.1	41310	49	Klebsiella phage vB_KpnP_IME205	NA	Viruses; Duplodnaviria; Heunggongvirae; Uroviricota; Caudoviricetes; Caudovirales; Autographiviridae; Studiervirinae; Przondovirus; Klebsiella virus IME205
GCA_001634515.1	5726	5	Alfalfa enamovirus1	NA	Viruses; ssRNA viruses; ssRNA positive-strand viruses, no DNA stage; Luteoviridae; Enamovirus; unclassified Enamovirus
GCA_001500735.1	10316	2	Thelephora terrestris virus 1	NA	Viruses; dsRNA viruses; Totiviridae; unclassified Totiviridae
GCA_001567035.1	7925	2	Currant virus A	NA	Viruses; ssRNA viruses; ssRNA positive-strand viruses, no DNA stage; Tymovirales; Betaflexiviridae; Trivirinae; Capillovirus; unclassified Capillovirus
GCA_002608255.1	50298	80	Escherichia phage JMPW2	NA	Viruses; Duplodnaviria; Heunggongvirae; Uroviricota; Caudoviricetes; Caudovirales; Drexlerviridae; Tunavirinae; Tunavirus; Escherichia virus JMPW2
GCA_002608295.1	49628	78	Escherichia phage JMPW1	NA	Viruses; Duplodnaviria; Heunggongvirae; Uroviricota; Caudoviricetes; Caudovirales; Drexlerviridae; Tunavirinae; Tunavirus; Escherichia virus JMPW1
GCA_002608315.1	64415	86	Pseudomonas phage KPP22	NA	Viruses; Duplodnaviria; Heunggongvirae; Uroviricota; Caudoviricetes; Caudovirales; Myoviridae; Pbunavirus; unclassified Pbunavirus
GCA_002608365.1	64415	86	Pseudomonas phage KPP22M1	NA	Viruses; Duplodnaviria; Heunggongvirae; Uroviricota; Caudoviricetes; Caudovirales; Myoviridae; Pbunavirus; unclassified Pbunavirus
GCA_002608395.1	64415	86	Pseudomonas phage KPP22M2	NA	Viruses; Duplodnaviria; Heunggongvirae; Uroviricota; Caudoviricetes; Caudovirales; Myoviridae; Pbunavirus; unclassified Pbunavirus
GCA_002608435.1	64415	86	Pseudomonas phage KPP22M3	NA	Viruses; Duplodnaviria; Heunggongvirae; Uroviricota; Caudoviricetes; Caudovirales; Myoviridae; Pbunavirus; unclassified Pbunavirus
GCA_003029255.2	5367	7	Pavonia mosaic virus	NA	Viruses; Monodnaviria; Shotokuvirae; Cressdnaviricota; Repensiviricetes; Geplafuvirales; Geminiviridae; Begomovirus
GCA_002622925.1	59173	86	Arthrobacter phage Amigo	NA	Viruses; Duplodnaviria; Heunggongvirae; Uroviricota; Caudoviricetes; Caudovirales; Siphoviridae; Amigovirus; Arthrobacter virus Amigo
GCA_002608465.1	58848	86	Arthrobacter phage Anansi	NA	Viruses; Duplodnaviria; Heunggongvirae; Uroviricota; Caudoviricetes; Caudovirales; Siphoviridae; Amigovirus; unclassified Amigovirus
GCA_002622705.1	43075	62	Arthrobacter phage Bennie	NA	Viruses; Duplodnaviria; Heunggongvirae; Uroviricota; Caudoviricetes; Caudovirales; Siphoviridae; Korravirus; Arthrobacter virus Bennie
GCA_002622965.1	58159	88	Arthrobacter phage CaptnMurica	NA	Viruses; Duplodnaviria; Heunggongvirae; Uroviricota; Caudoviricetes; Caudovirales; Siphoviridae; Gordonvirus; Arthrobacter virus Captnmurica
GCA_002622905.1	58353	99	Arthrobacter phage Circum	NA	Viruses; Duplodnaviria; Heunggongvirae; Uroviricota; Caudoviricetes; Caudovirales; Siphoviridae; Mudcatvirus; Arthrobacter virus Circum
GCA_002622725.1	42601	59	Arthrobacter phage DrRobert	NA	Viruses; Duplodnaviria; Heunggongvirae; Uroviricota; Caudoviricetes; Caudovirales; Siphoviridae; Korravirus; Arthrobacter virus DrRobert
GCA_002622745.1	44389	64	Arthrobacter phage Glenn	NA	Viruses; Duplodnaviria; Heunggongvirae; Uroviricota; Caudoviricetes; Caudovirales; Siphoviridae; Korravirus; Arthrobacter virus Glenn
GCA_002622985.1	58279	89	Arthrobacter phage Gordon	NA	Viruses; Duplodnaviria; Heunggongvirae; Uroviricota; Caudoviricetes; Caudovirales; Siphoviridae; Gordonvirus; Arthrobacter virus Gordon
GCA_002608505.1	58979	86	Arthrobacter phage Gorgeous	NA	Viruses; Duplodnaviria; Heunggongvirae; Uroviricota; Caudoviricetes; Caudovirales; Siphoviridae; Amigovirus; unclassified Amigovirus
GCA_002622765.1	43336	60	Arthrobacter phage HunterDalle	NA	Viruses; Duplodnaviria; Heunggongvirae; Uroviricota; Caudoviricetes; Caudovirales; Siphoviridae; Korravirus; Arthrobacter virus HunterDalle
GCA_002608535.1	43661	62	Arthrobacter phage Immaculata	NA	Viruses; Duplodnaviria; Heunggongvirae; Uroviricota; Caudoviricetes; Caudovirales; Siphoviridae; Korravirus; unclassified Korravirus
GCA_002608575.1	46723	57	Arthrobacter phage Jasmine	NA	Viruses; Duplodnaviria; Heunggongvirae; Uroviricota; Caudoviricetes; Caudovirales; Podoviridae; Jasminevirus; Arthrobacter virus Jasmine
GCA_002622785.1	44183	63	Arthrobacter phage Joann	NA	Viruses; Duplodnaviria; Heunggongvirae; Uroviricota; Caudoviricetes; Caudovirales; Siphoviridae; Korravirus; Arthrobacter virus Joann
GCA_002622805.1	43707	59	Arthrobacter phage Korra	NA	Viruses; Duplodnaviria; Heunggongvirae; Uroviricota; Caudoviricetes; Caudovirales; Siphoviridae; Korravirus; Arthrobacter virus Korra
GCA_002622885.1	60005	99	Arthrobacter phage Laroye	NA	Viruses; Duplodnaviria; Heunggongvirae; Uroviricota; Caudoviricetes; Caudovirales; Siphoviridae; Laroyevirus; Arthrobacter virus Laroye
GCA_002598685.1	15556	26	Arthrobacter phage Maggie	NA	Viruses; Duplodnaviria; Heunggongvirae; Uroviricota; Caudoviricetes; Caudovirales; Siphoviridae; Decurrovirus; unclassified Decurrovirus
GCA_002622465.1	51027	77	Arthrobacter phage Martha	NA	Viruses; Duplodnaviria; Heunggongvirae; Uroviricota; Caudoviricetes; Caudovirales; Myoviridae; Marthavirus; Arthrobacter virus Martha

gca	genome.size	proteome.size	phage.name	strain	Taxonomy
GCA_002598705.1	15630	26	Arthrobacter phage Moloch	NA	Viruses; Duplodnaviria; Heunggongvirae; Uroviricota; Caudoviricetes; Caudovirales; Siphoviridae; Decurrovirus; unclassified Decurrovirus
GCA_002608605.1	15524	26	Arthrobacter phage Muttlie	NA	Viruses; Duplodnaviria; Heunggongvirae; Uroviricota; Caudoviricetes; Caudovirales; Siphoviridae; Decurrovirus; unclassified Decurrovirus
GCA_002622825.1	43374	64	Arthrobacter phage Preamble	NA	Viruses; Duplodnaviria; Heunggongvirae; Uroviricota; Caudoviricetes; Caudovirales; Siphoviridae; Korravirus; Arthrobacter virus Preamble
GCA_002622845.1	42830	61	Arthrobacter phage Pumancara	NA	Viruses; Duplodnaviria; Heunggongvirae; Uroviricota; Caudoviricetes; Caudovirales; Siphoviridae; Korravirus; Arthrobacter virus Pumancara
GCA_002608625.1	44259	63	Arthrobacter phage RAP15	NA	Viruses; Duplodnaviria; Heunggongvirae; Uroviricota; Caudoviricetes; Caudovirales; Siphoviridae; Korravirus; unclassified Korravirus
GCA_002608645.1	59167	87	Arthrobacter phage Rings	NA	Viruses; Duplodnaviria; Heunggongvirae; Uroviricota; Caudoviricetes; Caudovirales; Siphoviridae; Amigovirus; unclassified Amigovirus
GCA_002608665.1	59807	99	Arthrobacter phage Salgado	NA	Viruses; Duplodnaviria; Heunggongvirae; Uroviricota; Caudoviricetes; Caudovirales; Siphoviridae; Laroyevirus; unclassified Laroyevirus
GCA_002622485.1	50909	77	Arthrobacter phage Sonny	NA	Viruses; Duplodnaviria; Heunggongvirae; Uroviricota; Caudoviricetes; Caudovirales; Myoviridae; Marthavirus; Arthrobacter virus Sonny
GCA_002608685.1	58979	86	Arthrobacter phage SorJuana	NA	Viruses; Duplodnaviria; Heunggongvirae; Uroviricota; Caudoviricetes; Caudovirales; Siphoviridae; Amigovirus; unclassified Amigovirus
GCA_002598725.1	15630	26	Arthrobacter phage Stratus	NA	Viruses; Duplodnaviria; Heunggongvirae; Uroviricota; Caudoviricetes; Caudovirales; Siphoviridae; Decurrovirus; unclassified Decurrovirus
GCA_002608705.1	50999	78	Arthrobacter phage TaeYoung	NA	Viruses; Duplodnaviria; Heunggongvirae; Uroviricota; Caudoviricetes; Caudovirales; Myoviridae; Marthavirus; unclassified Marthavirus
GCA_002623305.1	67592	105	Arthrobacter phage Tank	NA	Viruses; Duplodnaviria; Heunggongvirae; Uroviricota; Caudoviricetes; Caudovirales; Siphoviridae; Tankvirus; Arthrobacter virus Tank
GCA_002598745.1	15319	25	Arthrobacter phage Toulouse	NA	Viruses; Duplodnaviria; Heunggongvirae; Uroviricota; Caudoviricetes; Caudovirales; Siphoviridae; Decurrovirus; unclassified Decurrovirus
GCA_002608725.1	43336	60	Arthrobacter phage Vulture	NA	Viruses; Duplodnaviria; Heunggongvirae; Uroviricota; Caudoviricetes; Caudovirales; Siphoviridae; Korravirus; unclassified Korravirus
GCA_002622865.1	44371	63	Arthrobacter phage Wayne	NA	Viruses; Duplodnaviria; Heunggongvirae; Uroviricota; Caudoviricetes; Caudovirales; Siphoviridae; Korravirus; Arthrobacter virus Wayne
GCA_002608745.1	68203	109	Arthrobacter phage Wilde	NA	Viruses; Duplodnaviria; Heunggongvirae; Uroviricota; Caudoviricetes; Caudovirales; Siphoviridae; Tankvirus; unclassified Tankvirus
GCA_002598765.1	15524	26	Arthrobacter phage Yank	NA	Viruses; Duplodnaviria; Heunggongvirae; Uroviricota; Caudoviricetes; Caudovirales; Siphoviridae; Decurrovirus; unclassified Decurrovirus
GCA_002608765.1	37809	65	Arthrobacter phage Galaxy	NA	Viruses; Duplodnaviria; Heunggongvirae; Uroviricota; Caudoviricetes; Caudovirales; Siphoviridae; Galaxyvirus; Arthrobacter virus Galaxy
GCA_002622445.1	49419	73	Arthrobacter phage Jawnski	NA	Viruses; Duplodnaviria; Heunggongvirae; Uroviricota; Caudoviricetes; Caudovirales; Myoviridae; Marthavirus; Arthrobacter virus Jawnski
GCA_002757175.1	70265	112	Arthrobacter phage PrincessTrina	NA	Viruses; Duplodnaviria; Heunggongvirae; Uroviricota; Caudoviricetes; Caudovirales; Myoviridae; Radnorvirus; Arthrobacter virus Trina
GCA_002608785.1	93191	129	Pseudomonas phage SM1	NA	Viruses; Duplodnaviria; Heunggongvirae; Uroviricota; Caudoviricetes; Caudovirales; Siphoviridae; Samunavirus; Pseudomonas virus SM1
GCA_001502775.2	5474	8	Pepper yellow leaf curl Thailand virus	NA	Viruses; ssDNA viruses; Geminiviridae; Begomovirus; unclassified Begomovirus
GCA_001502155.1	14243	1	Hordeum vulgare endornavirus	NA	Viruses; dsRNA viruses; Endornaviridae; Endornavirus; unclassified Endornavirus
GCA_003147405.1	38594	54	Escherichia phage phi467	EF467_stx2f_phage	Viruses; Duplodnaviria; Heunggongvirae; Uroviricota; Caudoviricetes; Caudovirales; Siphoviridae; unclassified Siphoviridae
GCA_001736615.1	38975	67	Bacillus phage vB_BhaS171	NA	Viruses; dsDNA viruses, no RNA stage; Caudovirales; Siphoviridae; unclassified Siphoviridae
GCA_002608805.1	30924	57	Exiguobacterium phage vB_EauS123	NA	Viruses; Duplodnaviria; Heunggongvirae; Uroviricota; Caudoviricetes; Caudovirales; Siphoviridae; unclassified Siphoviridae
GCA_002608825.1	199912	254	Vibrio phage vB_VmeM32	NA	Viruses; Duplodnaviria; Heunggongvirae; Uroviricota; Caudoviricetes; Caudovirales; Myoviridae; Tevenvirinae; unclassified Tevenvirinae
GCA_002608845.1	57599	76	Pseudomonas phage AAT1	NA	Viruses; Duplodnaviria; Heunggongvirae; Uroviricota; Caudoviricetes; Caudovirales; Siphoviridae; Pamexvirus; unclassified Pamexvirus
GCA_002608865.1	49241	79	Xanthomonas phage XAJ2	NA	Viruses; Duplodnaviria; Heunggongvirae; Uroviricota; Caudoviricetes; Caudovirales; Siphoviridae; unclassified Siphoviridae
GCA_002608885.1	44862	53	Xanthomonas phage XAJ24	NA	Viruses; Duplodnaviria; Heunggongvirae; Uroviricota; Caudoviricetes; Caudovirales; Autographiviridae; Pradovirus; Xanthomonas virus XAJ24
GCA_002608905.1	64960	89	Nostoc phage N1	NA	Viruses; Duplodnaviria; Heunggongvirae; Uroviricota; Caudoviricetes; Caudovirales; Myoviridae; unclassified Myoviridae
GCA_002608925.1	68304	96	Nostoc phage A1	NA	Viruses; Duplodnaviria; Heunggongvirae; Uroviricota; Caudoviricetes; Caudovirales; Myoviridae; unclassified Myoviridae
GCA_002608945.1	57256	70	Streptomyces phage Maih	NA	Viruses; Duplodnaviria; Heunggongvirae; Uroviricota; Caudoviricetes; Caudovirales; Siphoviridae; Woodruffvirus; unclassified Woodruffvirus
GCA_002630505.1	66063	90	Pseudomonas phage NP3	NA	Viruses; Duplodnaviria; Heunggongvirae; Uroviricota; Caudoviricetes; Caudovirales; Myoviridae; Pbunavirus; Pseudomonas virus SN

gca	genome.size	proteome.size	phage.name	strain	Taxonomy
GCA_002146065.1	12193	6	Colocasia bobone diseaseassociated virus	SI	Viruses; Riboviria; Orthornavirae; Negarnaviricota; Haploviricotina; Monjiviricetes; Mononegavirales; Rhabdoviridae; Cytorhabdovirus; Colocasia bobone disease-associated cytorhabdovirus
GCA_013086665.1	12377	4	Medjerda Valley virus	T131	Viruses; Riboviria; Orthornavirae; Negarnaviricota; Polyploviricotina; Ellioviricetes; Bunyavirales; Phenuiviridae; Phlebovirus; Medjerda phlebovirus
GCA_001549545.1	9590	1	Jasmine virus T	NA	Viruses; Riboviria; Orthornavirae; Pisuviricota; Stelpaviricetes; Patatavirales; Potyviridae; Potyvirus
GCA_001549565.1	9130	2	Goose dicistrovirus	NA	Viruses; ssRNA viruses; ssRNA positive-strand viruses, no DNA stage; Picornavirales; Dicistroviridae; unclassified Dicistroviridae
GCA_001661795.1	5071	4	Diaphorina citri densovirus	NA	Viruses; ssDNA viruses; Parvoviridae; Densovirinae; Ambidensovirus; unclassified Densovirus
GCA_001550465.1	15078	1	Cucumis melo endornavirus	NA	Viruses; dsRNA viruses; Endornaviridae; Endornavirus; unclassified Endornavirus
GCA_001736835.1	165868	238	Bacillus phage Eldridge	NA	Viruses; dsDNA viruses, no RNA stage; Caudovirales; Myoviridae; unclassified Myoviridae
GCA_002867245.1	14146	5	Actinidia chlorotic ringspotassociated virus	NA	Viruses; Riboviria; Orthornavirae; Negarnaviricota; Polyploviricotina; Ellioviricetes; Bunyavirales; Fimoviridae; Emaravirus; Actinidia chlorotic ringspot-associated emaravirus
GCA_003033175.1	7773	6	Rusa timorensis papillomavirus type 1	NA	Viruses; Monodnaviria; Shotokuvirae; Cossaviricota; Papovaviricetes; Zurhausenvirales; Papillomaviridae; Firstpapillomavirinae; Treisthetapapillomavirus; Treisthetapapillomavirus 1
GCA_001551165.1	11907	1	Erysiphe cichoracearum endornavirus	NA	Viruses; dsRNA viruses; Endornaviridae; Endornavirus; unclassified Endornavirus
GCA_001550445.1	5003	2	Panax notoginseng virus A	NA	Viruses; dsRNA viruses; Totiviridae; unclassified Totiviridae
GCA_001737055.1	49033	80	Pseudomonas phage JBD44	NA	Viruses; dsDNA viruses, no RNA stage; Caudovirales; Siphoviridae; unclassified Siphoviridae
GCA_001736375.1	36938	57	Pseudomonas phage JBD69	NA	Viruses; dsDNA viruses, no RNA stage; Caudovirales; Siphoviridae; unclassified Siphoviridae
GCA_001736595.1	36629	55	Pseudomonas phage JBD93	NA	Viruses; dsDNA viruses, no RNA stage; Caudovirales; Siphoviridae; unclassified Siphoviridae
GCA_002608985.1	50877	91	Mycobacterium phage JenCasNa	NA	Viruses; Duplodnaviria; Heunggongvirae; Uroviricota; Caudoviricetes; Caudovirales; Siphoviridae; Fromanvirus; unclassified Fromanvirus
GCA_002609025.1	75844	132	Mycobacterium phage MkaliMitinis3	NA	Viruses; Duplodnaviria; Heunggongvirae; Uroviricota; Caudoviricetes; Caudovirales; Siphoviridae; Bronvirus; unclassified Bronvirus
GCA_002609045.1	53182	80	Gordonia phage Howe	NA	Viruses; Duplodnaviria; Heunggongvirae; Uroviricota; Caudoviricetes; Caudovirales; Siphoviridae; unclassified Siphoviridae
GCA_002609065.1	52920	98	Mycobacterium phage NaSiaTalie	NA	Viruses; Duplodnaviria; Heunggongvirae; Uroviricota; Caudoviricetes; Caudovirales; Siphoviridae; Fromanvirus; unclassified Fromanvirus
GCA_001736815.1	45417	49	Pseudomonas phage YMC1106C171_PPU_BP	NA	Viruses; dsDNA viruses, no RNA stage; Caudovirales; Podoviridae; unclassified Podoviridae
GCA_001737035.1	50183	73	Pseudomonas phage YMC1107P54_PAE_BP	NA	Viruses; dsDNA viruses, no RNA stage; Caudovirales; Siphoviridae; unclassified Siphoviridae
GCA_001736355.1	90552	159	Pseudomonas phage vB_PsyM_KIL1	NA	Viruses; dsDNA viruses, no RNA stage; Caudovirales; Myoviridae; unclassified Myoviridae
GCA_002757195.1	92466	161	Pseudomonas phage vB_PsyM_KIL2	NA	Viruses; Duplodnaviria; Heunggongvirae; Uroviricota; Caudoviricetes; Caudovirales; Myoviridae; Flaumdravirus; Pseudomonas virus KIL4; Pseudomonas phage vB_PsyM_KIL1
GCA_002757215.1	92068	161	Pseudomonas phage vB_PsyM_KIL3	NA	Viruses; Duplodnaviria; Heunggongvirae; Uroviricota; Caudoviricetes; Caudovirales; Myoviridae; Flaumdravirus; Pseudomonas virus KIL4; Pseudomonas phage vB_PsyM_KIL1
GCA_002757235.1	92095	161	Pseudomonas phage vB_PsyM_KIL3b	NA	Viruses; Duplodnaviria; Heunggongvirae; Uroviricota; Caudoviricetes; Caudovirales; Myoviridae; Flaumdravirus; Pseudomonas virus KIL4; Pseudomonas phage vB_PsyM_KIL1
GCA_002757255.1	92816	167	Pseudomonas phage vB_PsyM_KIL4	NA	Viruses; Duplodnaviria; Heunggongvirae; Uroviricota; Caudoviricetes; Caudovirales; Myoviridae; Flaumdravirus; Pseudomonas virus KIL2
GCA_002757275.1	93385	170	Pseudomonas phage vB_PsyM_KIL5	NA	Viruses; Duplodnaviria; Heunggongvirae; Uroviricota; Caudoviricetes; Caudovirales; Myoviridae; Flaumdravirus; Pseudomonas virus KIL4; Pseudomonas phage vB_PsyM_KIL1
GCA_002609085.1	60897	74	Escherichia phage phiON2011	NA	Viruses; Duplodnaviria; Heunggongvirae; Uroviricota; Caudoviricetes; Caudovirales; Podoviridae; Sepvirinae; Oslovirus; unclassified Oslovirus
GCA_001736575.1	93754	178	Pseudomonas phage K5	NA	Viruses; dsDNA viruses, no RNA stage; Caudovirales; Myoviridae; unclassified Myoviridae
GCA_002609105.1	139100	182	Bacteriophage vB_NpeS2AV2	NA	Viruses; Duplodnaviria; Heunggongvirae; Uroviricota; Caudoviricetes; Caudovirales; Siphoviridae; unclassified Siphoviridae
GCA_003179755.1	8007	6	Canis familiaris papillomavirus 17	NA	Viruses; Monodnaviria; Shotokuvirae; Cossaviricota; Papovaviricetes; Zurhausenvirales; Papillomaviridae; Firstpapillomavirinae; Taupapillomavirus; Taupapillomavirus 1
GCA_001551145.1	1846	2	Ctenophoreassociated circular virus 1	NA	Viruses; ssDNA viruses; unclassified ssDNA viruses
GCA_001551485.1	1853	2	Ctenophoreassociated circular virus 2	NA	Viruses; ssDNA viruses; unclassified ssDNA viruses
GCA_001551005.1	1722	2	Ctenophoreassociated circular virus 3	NA	Viruses; ssDNA viruses; unclassified ssDNA viruses
GCA_001550625.1	1714	2	Ctenophoreassociated circular virus 4	NA	Viruses; ssDNA viruses; unclassified ssDNA viruses

gca	genome.size	proteome.size	phage.name	strain	Taxonomy
GCA_001551325.1	1210	1	Ctenophoreassociated circular genome 1	NA	Viruses; Satellites; ssDNA satellites; unclassified ssDNA satellites
GCA_001550985.1	1223	1	Ctenophoreassociated circular genome 3	NA	Viruses; Satellites; ssDNA satellites; unclassified ssDNA satellites
GCA_001550605.1	1210	1	Ctenophoreassociated circular genome 4	NA	Viruses; Satellites; ssDNA satellites; unclassified ssDNA satellites
GCA_001550585.1	6421	1	Nectarine virus M	NA	Viruses; ssRNA viruses; ssRNA positive-strand viruses, no DNA stage; Tymovirales; Tymoviridae; unclassified Tymoviridae
GCA_004117155.1	8101	4	Apple necrotic mosaic virus	NA	Viruses; Riboviria; Orthornavirae; Kitrinoviricota; Alsuviricetes; Martellivirales; Bromoviridae; Ilarvirus; unclassified Ilarvirus
GCA_002004575.1	2822	1	Phomopsis longicolla RNA virus 1	NA	Viruses; ssRNA viruses; ssRNA positive-strand viruses, no DNA stage; Narnaviridae; unclassified Narnaviridae
GCA_003029225.2	5456	9	Lycianthes yellow mosaic virus	NA	Viruses; Monodnaviria; Shotokuvirae; Cressdnaviricota; Repensiviricetes; Geplafuvirales; Geminiviridae; Begomovirus
GCA_002609125.1	38039	48	Clostridium phage HM T	NA	Viruses; Duplodnaviria; Heunggongvirae; Uroviricota; Caudoviricetes; Caudovirales; Siphoviridae; unclassified Siphoviridae
GCA_004788275.1	7062	1	Swine picornavirus Japan	TottoriWOL	Viruses; Riboviria; Orthornavirae; Pisuviricota; Pisoniviricetes; Picornavirales; Picornaviridae; Tottorivirus; Tottorivirus A
GCA_001560985.1	9581	2	Delisea pulchra RNA virus	NA	Viruses; ssRNA viruses; ssRNA positive-strand viruses, no DNA stage; Picornavirales; unclassified Picornavirales
GCA_000840385.1	7458	3	Taro bacilliform virus	NA	Viruses; Riboviria; Pararnavirae; Artverviricota; Revtraviricetes; Ortervirales; Caulimoviridae; Badnavirus
GCA_002609145.1	71436	100	Mycobacterium phage Weiss13	NA	Viruses; Duplodnaviria; Heunggongvirae; Uroviricota; Caudoviricetes; Caudovirales; Siphoviridae; Papyrusvirus; unclassified Papyrusvirus
GCA_002609165.1	67509	94	Mycobacterium phage Glass	NA	Viruses; Duplodnaviria; Heunggongvirae; Uroviricota; Caudoviricetes; Caudovirales; Siphoviridae; Bclasvirinae; Rosebushvirus; unclassified Rosebushvirus
GCA_002609185.1	64477	78	Streptomyces phage Xkcd426	NA	Viruses; Duplodnaviria; Heunggongvirae; Uroviricota; Caudoviricetes; Caudovirales; Siphoviridae; Woodruffvirus; unclassified Woodruffvirus
GCA_002609205.1	146647	234	Enterobacteria phage ECGD1	NA	Viruses; Duplodnaviria; Heunggongvirae; Uroviricota; Caudoviricetes; Caudovirales; Myoviridae; unclassified Myoviridae
GCA_001755125.1	52129	87	Mycobacterium phage Bactobuster	NA	Viruses; dsDNA viruses, no RNA stage; Caudovirales; Siphoviridae; unclassified Siphoviridae
GCA_003179735.1	7722	7	Alouatta guariba papillomavirus 1	NA	Viruses; Monodnaviria; Shotokuvirae; Cossaviricota; Papovaviricetes; Zurhausenvirales; Papillomaviridae; Firstpapillomavirinae; Dyoomikronpapillomavirus; Dyoomikronpapillomavirus 1
GCA_002624525.1	43851	53	Xanthomonas phage f20Xaj	NA	Viruses; Duplodnaviria; Heunggongvirae; Uroviricota; Caudoviricetes; Caudovirales; Autographiviridae; Pradovirus; Xanthomonas virus f20
GCA_002609225.1	41865	61	Xanthomonas phage f29Xaj	NA	Viruses; Duplodnaviria; Heunggongvirae; Uroviricota; Caudoviricetes; Caudovirales; Siphoviridae; Guernseyvirinae; Jerseyvirus; unclassified Jerseyvirus
GCA_002624545.1	44262	53	Xanthomonas phage f30Xaj	NA	Viruses; Duplodnaviria; Heunggongvirae; Uroviricota; Caudoviricetes; Caudovirales; Autographiviridae; Pradovirus; Xanthomonas virus f30
GCA_001755545.1	50509	76	Pseudomonas phage O4	NA	Viruses; dsDNA viruses, no RNA stage; unclassified dsDNA phages
GCA_002989995.1	43036	49	Shigella phage SFN6B	NA	Viruses; Duplodnaviria; Heunggongvirae; Uroviricota; Caudoviricetes; Caudovirales; Autographiviridae; Slopekvirinae; Drulisvirus; Shigella virus SFN6B
GCA_000872425.2	186713	247	Ostreococcus tauri virus OtV5	NA	Viruses; dsDNA viruses, no RNA stage; Phycodnaviridae; Prasinovirus
GCA_001736715.1	167619	244	Ralstonia phage RSP15	NA	Viruses; dsDNA viruses, no RNA stage; Caudovirales; Myoviridae; unclassified Myoviridae
GCA_002987495.1	9283	1	Tomato mild mottle virus	NA	Viruses; Riboviria; Orthornavirae; Pisuviricota; Stelpaviricetes; Patatavirales; Potyviridae; Ipomovirus
GCA_001881735.1	38979	49	Escherichia phage LM33_P1	NA	Viruses; dsDNA viruses, no RNA stage; Caudovirales; Podoviridae; Autographivirinae; unclassified Autographivirinae
GCA_003029215.1	8178	4	Sclerotinia sclerotiorum deltaflexivirus 1	NA	Viruses; Riboviria; Orthornavirae; Kitrinoviricota; Alsuviricetes; Tymovirales; Deltaflexiviridae; Deltaflexivirus; Sclerotinia deltaflexivirus 1
GCA_001560965.1	5212	5	Rat bocavirus	HK1S	Viruses; ssDNA viruses; Parvoviridae; Parvovirinae; Bocaparvovirus; unclassified Bocaparvovirus
GCA_002757295.1	41865	62	Salmonella phage f2SE	NA	Viruses; Duplodnaviria; Heunggongvirae; Uroviricota; Caudoviricetes; Caudovirales; Siphoviridae; Guernseyvirinae; Jerseyvirus; Salmonella virus f18SE; Salmonella phage f18SE
GCA_002757315.1	41867	61	Salmonella phage f3SE	NA	Viruses; Duplodnaviria; Heunggongvirae; Uroviricota; Caudoviricetes; Caudovirales; Siphoviridae; Guernseyvirinae; Jerseyvirus; Salmonella virus f18SE; Salmonella phage f18SE
GCA_001630065.1	37556	33	Bat mastadenovirus WIV10	NA	Viruses; dsDNA viruses, no RNA stage; Adenoviridae; Mastadenovirus; unclassified Mastadenovirus
GCA_001630005.1	38073	33	Bat mastadenovirus WIV11	NA	Viruses; dsDNA viruses, no RNA stage; Adenoviridae; Mastadenovirus; unclassified Mastadenovirus
GCA_001722965.1	29581	28	Bat mastadenovirus WIV12	NA	Viruses; dsDNA viruses, no RNA stage; Adenoviridae; Mastadenovirus; unclassified Mastadenovirus
GCA_001629825.1	37545	33	Bat mastadenovirus WIV9	NA	Viruses; dsDNA viruses, no RNA stage; Adenoviridae; Mastadenovirus; unclassified Mastadenovirus

gca	genome.size	proteome.size	phage.name	strain	Taxonomy
GCA_001684945.1	1051	1	Circoviruslike genome DCCV1	NA	Viruses; ssDNA viruses; Circoviridae; unclassified Circoviridae
GCA_001684825.1	2320	3	Circoviruslike genome DCCV10	NA	Viruses; ssDNA viruses; Circoviridae; unclassified Circoviridae
GCA_001684585.1	3124	5	Circoviruslike genome DCCV11	NA	Viruses; ssDNA viruses; Circoviridae; unclassified Circoviridae
GCA_001684705.1	2103	3	Circoviruslike genome DCCV12	NA	Viruses; ssDNA viruses; Circoviridae; unclassified Circoviridae
GCA_001684925.1	2433	3	Circoviruslike genome DCCV13	NA	Viruses; ssDNA viruses; Circoviridae; unclassified Circoviridae
GCA_001684805.1	2187	3	Circoviruslike genome DCCV2	NA	Viruses; ssDNA viruses; Circoviridae; unclassified Circoviridae
GCA_001684565.1	1864	3	Circoviruslike genome DCCV3	NA	Viruses; ssDNA viruses; Circoviridae; unclassified Circoviridae
GCA_001684685.1	2985	3	Circoviruslike genome DCCV4	NA	Viruses; ssDNA viruses; Circoviridae; unclassified Circoviridae
GCA_001684905.1	2533	4	Circoviruslike genome DCCV5	NA	Viruses; ssDNA viruses; Circoviridae; unclassified Circoviridae
GCA_001684785.1	2522	3	Circoviruslike genome DCCV6	NA	Viruses; ssDNA viruses; Circoviridae; unclassified Circoviridae
GCA_001684545.1	3021	5	Circoviruslike genome DCCV7	NA	Viruses; ssDNA viruses; Circoviridae; unclassified Circoviridae
GCA_001684665.1	2651	4	Circoviruslike genome DCCV8	NA	Viruses; ssDNA viruses; Circoviridae; unclassified Circoviridae
GCA_001684885.1	1828	3	Circoviruslike genome DCCV9	NA	Viruses; ssDNA viruses; Circoviridae; unclassified Circoviridae
GCA_001684765.1	1173	1	Circoviruslike genome DHCV1	NA	Viruses; ssDNA viruses; Circoviridae; unclassified Circoviridae
GCA_001684525.1	2854	5	Circoviruslike genome DHCV2	NA	Viruses; ssDNA viruses; Circoviridae; unclassified Circoviridae
GCA_001684645.1	2029	3	Circoviruslike genome DHCV3	NA	Viruses; ssDNA viruses; Circoviridae; unclassified Circoviridae
GCA_001684865.1	1784	2	Circoviruslike genome DHCV5	NA	Viruses; ssDNA viruses; Circoviridae; unclassified Circoviridae
GCA_001684745.1	2369	3	Circoviruslike genome DHCV6	NA	Viruses; ssDNA viruses; Circoviridae; unclassified Circoviridae
GCA_002118945.1	11974	3	Eothenomys eleusis hantavirus FUGV	NA	Viruses; Riboviria; Orthornavirae; Negarnaviricota; Polyploviricotina; Ellioviricetes; Bunyavirales; Hantaviridae; Mammantavirinae; Orthohantavirus; Fugong orthohantavirus
GCA_001754685.1	221908	317	Bacillus phage SP15	NA	Viruses; dsDNA viruses, no RNA stage; Caudovirales; Myoviridae; unclassified Myoviridae
GCA_002609245.1	50628	91	Mycobacterium phage Dynamix	NA	Viruses; Duplodnaviria; Heunggongvirae; Uroviricota; Caudoviricetes; Caudovirales; Siphoviridae; Fromanvirus; unclassified Fromanvirus
GCA_001745775.1	44885	83	Acinetobacter phage LZ35	NA	Viruses; dsDNA viruses, no RNA stage; Caudovirales; Myoviridae; unclassified Myoviridae
GCA_002599845.1	50877	88	Mycobacterium phage Marie	NA	Viruses; Duplodnaviria; Heunggongvirae; Uroviricota; Caudoviricetes; Caudovirales; Siphoviridae; Fromanvirus; unclassified Fromanvirus
GCA_001745535.1	157501	226	Bacillus phage Deep Blue	NA	Viruses; dsDNA viruses, no RNA stage; Caudovirales; Myoviridae; unclassified Myoviridae
GCA_001754865.1	53411	101	Mycobacterium phage Catalina	NA	Viruses; dsDNA viruses, no RNA stage; Caudovirales; Siphoviridae; unclassified Siphoviridae
GCA_001755305.1	43293	69	Staphylococcus phage CNPx	NA	Viruses; dsDNA viruses, no RNA stage; Caudovirales; Siphoviridae; unclassified Siphoviridae
GCA_001881715.1	46575	61	Flavobacterium phage 2A	NA	Viruses; unclassified phages
GCA_001882275.1	88994	80	Flavobacterium phage Fpv1	NA	Viruses; unclassified phages
GCA_002609305.1	62331	87	Stx converting phage vB_EcoS_P22	NA	Viruses; Duplodnaviria; Heunggongvirae; Uroviricota; Caudoviricetes; Caudovirales; Podoviridae; Sepvirinae; Traversvirus; unclassified Traversvirus
GCA_002609325.1	61580	88	Stx converting phage vB_EcoS_P27	NA	Viruses; Duplodnaviria; Heunggongvirae; Uroviricota; Caudoviricetes; Caudovirales; Podoviridae; Sepvirinae; Traversvirus; unclassified Traversvirus
GCA_002609345.1	62535	90	Stx converting phage vB_EcoS_P32	NA	Viruses; Duplodnaviria; Heunggongvirae; Uroviricota; Caudoviricetes; Caudovirales; Podoviridae; Sepvirinae; Traversvirus; unclassified Traversvirus
GCA_002609365.1	62822	87	Stx converting phage vB_EcoS_ST28624	NA	Viruses; Duplodnaviria; Heunggongvirae; Uroviricota; Caudoviricetes; Caudovirales; Podoviridae; Sepvirinae; Traversvirus; unclassified Traversvirus
GCA_000837425.1	57416	83	Sinorhizobium phage PBC5	NA	Viruses; dsDNA viruses, no RNA stage; Caudovirales; unclassified Caudovirales
GCA_000863865.1	5307	6	Murine polyomavirus strain BG	NA	Viruses; dsDNA viruses, no RNA stage; Polyomaviridae; Alphapolyomavirus; Mus musculus polyomavirus 1

gca	genome.size	proteome.size	phage.name	strain	Taxonomy
GCA_001646355.1	9896	1	Tobacco mosqueado virus	NA	Viruses; ssRNA viruses; ssRNA positive-strand viruses, no DNA stage; Potyviridae; unclassified Potyviridae
GCA_002826025.1	2202	3	Human gemycircularvirus GeTz1	NA	Viruses; Monodnaviria; Shotokuvirae; Cressdnaviricota; Repensiviricetes; Geplafuvirales; Genomoviridae; Gemykibivirus; Human associated gemykibivirus 4
GCA_002665415.1	18947	12	Rotavirus A pheasantwtHUN2162015G23P37	NA	Viruses; Riboviria; Orthornavirae; Duplornaviricota; Resentoviricetes; Reovirales; Reoviridae; Sedoreovirinae; Rotavirus; Rotavirus A; Rotavirus A isolates
GCA_003972025.2	20398	4	Kadiweu virus	BrMSMQ10	Viruses; Riboviria; Orthornavirae; Pisuviricota; Pisoniviricetes; Nidovirales; Mesnidovirineae; Mesoniviridae; Hexponivirinae; Alphamesonivirus; Kadilivirus; Alphamesonivirus 7
GCA_003972045.1	19867	2	Ofaie virus	BrMSMQ10	Viruses; Riboviria; Orthornavirae; Pisuviricota; Pisoniviricetes; Nidovirales; Mesnidovirineae; Mesoniviridae; Hexponivirinae; Alphamesonivirus; Ofalivirus; Alphamesonivirus 6
GCA_001706925.1	9868	1	Picornavirales TottoriHG1	NA	Viruses; ssRNA viruses; ssRNA positive-strand viruses, no DNA stage; Picornavirales; unclassified Picornavirales
GCA_002819825.1	1923	2	Pacific flying fox associated cyclovirus1	NA	Viruses; Monodnaviria; Shotokuvirae; Cressdnaviricota; Arfiviricetes; Cirlivirales; Circoviridae; Cyclovirus; Bat associated cyclovirus 14
GCA_002819845.1	1916	2	Pacific flying fox associated cyclovirus2	NA	Viruses; Monodnaviria; Shotokuvirae; Cressdnaviricota; Arfiviricetes; Cirlivirales; Circoviridae; Cyclovirus; Bat associated cyclovirus 15
GCA_002819865.1	1838	2	Pacific flying fox associated cyclovirus3	NA	Viruses; Monodnaviria; Shotokuvirae; Cressdnaviricota; Arfiviricetes; Cirlivirales; Circoviridae; Cyclovirus; Bat associated cyclovirus 16
GCA_002825985.1	2233	3	Pacific flying fox faeces associated gemycircularvirus1	NA	Viruses; Monodnaviria; Shotokuvirae; Cressdnaviricota; Repensiviricetes; Geplafuvirales; Genomoviridae; Gemygorvirus; Pteropus associated gemygorvirus 1
GCA_002825765.1	2191	3	Pacific flying fox faeces associated gemycircularvirus10	NA	Viruses; Monodnaviria; Shotokuvirae; Cressdnaviricota; Repensiviricetes; Geplafuvirales; Genomoviridae; Gemycircularvirus; Pteropus associated gemycircularvirus 1
GCA_002826045.1	2310	3	Pacific flying fox faeces associated gemycircularvirus12	NA	Viruses; Monodnaviria; Shotokuvirae; Cressdnaviricota; Repensiviricetes; Geplafuvirales; Genomoviridae; Gemykibivirus; Pteropus associated gemykibivirus 1
GCA_002825785.1	2250	3	Pacific flying fox faeces associated gemycircularvirus2	NA	Viruses; Monodnaviria; Shotokuvirae; Cressdnaviricota; Repensiviricetes; Geplafuvirales; Genomoviridae; Gemycircularvirus; Pteropus associated gemycircularvirus 2
GCA_002826105.1	2220	3	Pacific flying fox faeces associated gemycircularvirus6	NA	Viruses; Monodnaviria; Shotokuvirae; Cressdnaviricota; Repensiviricetes; Geplafuvirales; Genomoviridae; Gemykolovirus; Pteropus associated gemykolovirus 1
GCA_002826125.1	2217	3	Pacific flying fox faeces associated gemycircularvirus7	NA	Viruses; Monodnaviria; Shotokuvirae; Cressdnaviricota; Repensiviricetes; Geplafuvirales; Genomoviridae; Gemykolovirus; Pteropus associated gemykolovirus 2
GCA_003727055.1	1163	1	Pacific flying fox associated multicomponent virus	NA	Viruses; unclassified viruses
GCA_003727155.1	2254	2	Pacific flying fox faeces associated circular DNA virus1	NA	Viruses; unclassified viruses
GCA_003727275.1	2522	2	Pacific flying fox faeces associated circular DNA virus10	NA	Viruses; unclassified viruses
GCA_003727295.1	1948	2	Pacific flying fox faeces associated circular DNA virus11	NA	Viruses; unclassified viruses
GCA_003727335.1	2655	2	Pacific flying fox faeces associated circular DNA virus12	NA	Viruses; unclassified viruses
GCA_003727375.1	2630	3	Pacific flying fox faeces associated circular DNA virus13	NA	Viruses; unclassified viruses
GCA_003727395.1	2732	2	Pacific flying fox faeces associated circular DNA virus14	NA	Viruses; unclassified viruses
GCA_003727415.1	2571	2	Pacific flying fox faeces associated circular DNA virus15	NA	Viruses; unclassified viruses
GCA_003727315.1	2538	2	Pacific flying fox faeces associated circular DNA virus2	NA	Viruses; unclassified viruses
GCA_003727115.1	2214	2	Pacific flying fox faeces associated circular DNA virus3	NA	Viruses; unclassified viruses
GCA_003727135.1	1757	2	Pacific flying fox faeces associated circular DNA virus4	NA	Viruses; unclassified viruses
GCA_003727195.1	2694	2	Pacific flying fox faeces associated circular DNA virus5	NA	Viruses; unclassified viruses
GCA_003727215.1	1963	2	Pacific flying fox faeces associated circular DNA virus6	NA	Viruses; unclassified viruses
GCA_003727235.1	2722	2	Pacific flying fox faeces associated circular DNA virus7	NA	Viruses; unclassified viruses
GCA_003727255.1	2004	2	Pacific flying fox faeces associated circular DNA virus8	NA	Viruses; unclassified viruses
GCA_003727035.1	2707	3	Pacific flying fox faeces associated circular DNA virus9	NA	Viruses; unclassified viruses
GCA_001736935.1	138081	177	Bacillus phage Shbh1	NA	Viruses; dsDNA viruses, no RNA stage; Caudovirales; Myoviridae; unclassified Myoviridae
GCA_002609385.1	58951	80	Bacillus phage Mgbh1	NA	Viruses; Duplodnaviria; Heunggongvirae; Uroviricota; Caudoviricetes; Caudovirales; Siphoviridae; Magadivirus; Bacillus virus Mgbh1
GCA_001754425.1	51290	79	Arthrobacter phage BarretLemon	NA	Viruses; dsDNA viruses, no RNA stage; Caudovirales; Myoviridae; unclassified Myoviridae

gca	genome.size	proteome.size	phage.name	strain	Taxonomy
GCA_001755285.1	58871	98	Arthrobacter phage KellEzio	NA	Viruses; dsDNA viruses, no RNA stage; Caudovirales; Siphoviridae; unclassified Siphoviridae
GCA_001755705.1	58560	100	Arthrobacter phage Kitkat	NA	Viruses; dsDNA viruses, no RNA stage; Caudovirales; Siphoviridae; unclassified Siphoviridae
GCA_001754585.2	59443	95	Arthrobacter phage Mudcat	NA	Viruses; dsDNA viruses, no RNA stage; Caudovirales; Siphoviridae; unclassified Siphoviridae
GCA_001754845.1	43267	53	Klebsiella phage vB_KpnP_KpV71	NA	Viruses; dsDNA viruses, no RNA stage; Caudovirales; Podoviridae; unclassified Podoviridae
GCA_001579395.1	70812	95	Acidianus tailed spindle virus	NA	Viruses; dsDNA viruses, no RNA stage; Bicaudaviridae; unclassified Bicaudaviridae
GCA_001567055.1	6038	3	Piscine myocarditislike virus	NA	Viruses; dsRNA viruses; Totiviridae; unclassified Totiviridae
GCA_007833035.1	111154	132	Infectious spleen and kidney necrosis virus	RSIVKu	Viruses; dsDNA viruses, no RNA stage; Iridoviridae; Megalocytivirus
GCA_000871465.1	295146	163	Cyprinid herpesvirus 3	KHVU	Viruses; dsDNA viruses, no RNA stage; Herpesvirales; Alloherpesviridae; Cyprinivirus
GCA_018580445.1	13062	6	Pararge aegeria rhabdovirus	NA	Viruses; Riboviria; Orthornavirae; Negarnaviricota; Haploviricotina; Monjiviricetes; Mononegavirales; Rhabdoviridae; Alpharhabdovirinae; Alphapaprhavirus; Pararge alphapaprhavirus
GCA_018580405.1	13885	6	Drosophila sturtevanti sigmavirus	NA	Viruses; Riboviria; Orthornavirae; Negarnaviricota; Haploviricotina; Monjiviricetes; Mononegavirales; Rhabdoviridae; Alpharhabdovirinae; Sigmavirus; Sturtevanti sigmavirus
GCA_018580435.1	12583	6	Ceratitis capitata sigmavirus	NA	Viruses; Riboviria; Orthornavirae; Negarnaviricota; Haploviricotina; Monjiviricetes; Mononegavirales; Rhabdoviridae; Alpharhabdovirinae; Sigmavirus; Capitata sigmavirus
GCA_003673805.1	13559	5	Drosophila unispina virus 1	NA	Viruses; Riboviria; Orthornavirae; Negarnaviricota; Haploviricotina; Monjiviricetes; Mononegavirales; Xinmoviridae; Anphevirus; Drosophilid anphevirus
GCA_004129635.1	11798	5	Merida virus	NA	Viruses; Riboviria; Orthornavirae; Negarnaviricota; Haploviricotina; Monjiviricetes; Mononegavirales; Rhabdoviridae; unclassified Rhabdoviridae
GCA_004129675.1	11713	5	Riverside virus 1	RISVDrava 1	Viruses; Riboviria; Orthornavirae; Negarnaviricota; Haploviricotina; Monjiviricetes; Mononegavirales; Rhabdoviridae; unclassified Rhabdoviridae
GCA_002285025.1	6339	3	Bastrovirus 7	NA	Viruses; Riboviria; Orthornavirae; Pisuviricota; Stelpaviricetes; Stellavirales; Astroviridae; unclassified Astroviridae; Bastrovirus BAS-1
GCA_002105405.1	8696	5	Pepper virus A	NA	Viruses; ssRNA viruses; ssRNA positive-strand viruses, no DNA stage; Tymovirales; Betaflexiviridae; Quinvirinae; Carlavirus; unclassified Carlavirus
GCA_000887515.1	5235	7	Melon chlorotic mosaic virus	NA	Viruses; ssDNA viruses; Geminiviridae; Begomovirus; unclassified Begomovirus
GCA_001576855.1	8660	2	Marine RNA virus PAL128	NA	Viruses; unclassified viruses
GCA_001579455.1	7897	2	Marine RNA virus PAL156	NA	Viruses; unclassified viruses
GCA_001579355.1	7047	2	Marine RNA virus PAL438	NA	Viruses; unclassified viruses
GCA_001579475.1	6360	2	Marine RNA virus PAL473	NA	Viruses; unclassified viruses
GCA_001579415.1	8187	2	Marine RNA virus PAL_E4	NA	Viruses; unclassified viruses
GCA_002609565.1	6834	6	Vibrio phage VSKK	NA	Viruses; Monodnaviria; Loebvirae; Hofneiviricota; Faserviricetes; Tubulavirales; Inoviridae; Fibrovirus; Vibrio virus fs1; Vibrio phage VSK
GCA_000840845.1	53373	72	Burkholderia virus phiE125	NA	Viruses; dsDNA viruses, no RNA stage; Caudovirales; Siphoviridae; E125virus
GCA_002601865.1	239461	267	Enterobacteria phage SEGD1	NA	Viruses; Duplodnaviria; Heunggongvirae; Uroviricota; Caudoviricetes; Caudovirales; Myoviridae; Seoulvirus; Salmonella virus SPN3US
GCA_001754405.1	77832	135	Mycobacterium phage Bipper	NA	Viruses; dsDNA viruses, no RNA stage; Caudovirales; Siphoviridae; unclassified Siphoviridae
GCA_002609285.1	53425	99	Mycobacterium phage Myxus	NA	Viruses; Duplodnaviria; Heunggongvirae; Uroviricota; Caudoviricetes; Caudovirales; Siphoviridae; Fromanvirus; unclassified Fromanvirus
GCA_001755265.1	52428	98	Mycobacterium phage Mulciber	NA	Viruses; dsDNA viruses, no RNA stage; Caudovirales; Siphoviridae; unclassified Siphoviridae
GCA_002757335.1	52502	99	Mycobacterium phage McFly	NA	Viruses; Duplodnaviria; Heunggongvirae; Uroviricota; Caudoviricetes; Caudovirales; Siphoviridae; Fromanvirus; unclassified Fromanvirus
GCA_001744455.1	39434	53	Citrobacter phage SH1	NA	Viruses; dsDNA viruses, no RNA stage; Caudovirales; Podoviridae; Autographivirinae; T7virus; unclassified T7-like viruses
GCA_001743775.1	39158	53	Citrobacter phage SH2	NA	Viruses; dsDNA viruses, no RNA stage; Caudovirales; Podoviridae; Autographivirinae; T7virus; unclassified T7-like viruses
GCA_001745095.1	39444	49	Citrobacter phage SH3	NA	Viruses; dsDNA viruses, no RNA stage; Caudovirales; Podoviridae; Autographivirinae; T7virus; unclassified T7-like viruses
GCA_001745755.1	39274	49	Citrobacter phage SH4	NA	Viruses; dsDNA viruses, no RNA stage; Caudovirales; Podoviridae; Autographivirinae; T7virus; unclassified T7-like viruses
GCA_002609405.1	39832	50	Citrobacter phage SH5	NA	Viruses; Duplodnaviria; Heunggongvirae; Uroviricota; Caudoviricetes; Caudovirales; Autographiviridae; Studiervirinae; Kayfunavirus; unclassified Kayfunavirus

gca	genome.size	proteome.size	phage.name	strain	Taxonomy
GCA_001885445.1	17714	39	Pyrobaculum filamentous virus 1	NA	Viruses; dsDNA viruses, no RNA stage; unclassified dsDNA viruses
GCA_002004915.1	10203	1	Bamaga virus	NA	Viruses; ssRNA viruses; ssRNA positive-strand viruses, no DNA stage; Flaviviridae; unclassified Flaviviridae
GCA_000841125.1	5002	3	Casphalia extranea densovirus	NA	Viruses; ssDNA viruses; Parvoviridae; Densovirinae; Iteradensovirus; Lepidopteran iteradensovirus 2
GCA_000837545.1	2757	6	Honeysuckle yellow vein mosaic virus	NA	Viruses; ssDNA viruses; Geminiviridae; Begomovirus
GCA_001744435.1	161552	291	Bacillus phage DIGNKC	NA	Viruses; dsDNA viruses, no RNA stage; Caudovirales; Myoviridae; unclassified Myoviridae
GCA_002604025.1	164227	293	Bacillus phage Juglone	NA	Viruses; Duplodnaviria; Heunggongvirae; Uroviricota; Caudoviricetes; Caudovirales; Herelleviridae; Bastillevirinae; Bequatrovirus; Bacillus virus Troll; Bacillus phage Troll
GCA_001743755.1	162375	301	Bacillus phage Nemo	NA	Viruses; dsDNA viruses, no RNA stage; Caudovirales; Myoviridae; unclassified Myoviridae
GCA_001745075.1	163041	302	Bacillus phage Nigalana	NA	Viruses; dsDNA viruses, no RNA stage; Caudovirales; Myoviridae; unclassified Myoviridae
GCA_001745735.1	161929	296	Bacillus phage NotTheCreek	NA	Viruses; dsDNA viruses, no RNA stage; Caudovirales; Myoviridae; unclassified Myoviridae
GCA_001744415.1	164443	288	Bacillus phage Phrodo	NA	Viruses; dsDNA viruses, no RNA stage; Caudovirales; Myoviridae; unclassified Myoviridae
GCA_001743735.1	162359	300	Bacillus phage SageFayge	NA	Viruses; dsDNA viruses, no RNA stage; Caudovirales; Myoviridae; unclassified Myoviridae
GCA_002604625.1	162865	297	Bacillus phage Vinny	NA	Viruses; Duplodnaviria; Heunggongvirae; Uroviricota; Caudoviricetes; Caudovirales; Herelleviridae; Bastillevirinae; Bastillevirus; unclassified Bastillevirus
GCA_001745055.1	163345	294	Bacillus phage Zuko	NA	Viruses; dsDNA viruses, no RNA stage; Caudovirales; Myoviridae; unclassified Myoviridae
GCA_002609425.1	55060	95	Pseudomonas phage phiAH14a	NA	Viruses; Duplodnaviria; Heunggongvirae; Uroviricota; Caudoviricetes; Caudovirales; Siphoviridae; unclassified Siphoviridae
GCA_004130335.1	15224	8	Rodent arterivirus	NA	Viruses; ssRNA viruses; ssRNA positive-strand viruses, no DNA stage; Nidovirales; Arteriviridae; unclassified Arteriviridae
GCA_002601545.1	18379	24	Bacillus phage vB_BsuPGoe1	NA	Viruses; Duplodnaviria; Heunggongvirae; Uroviricota; Caudoviricetes; Caudovirales; Podoviridae; Picovirinae; unclassified Picovirinae
GCA_013087345.1	15850	1	Rhizoctonia solani endornavirus 2	NA	Viruses; Riboviria; Orthornavirae; Kitrinoviricota; Alsuviricetes; Martellivirales; Endornaviridae; Alphaendornavirus; Rhizoctonia solani alphaendornavirus 2
GCA_002029555.1	3170	2	Phomopsis longicolla circular virus 1	TWH P74	Viruses; unclassified viruses
GCA_004787675.1	8300	7	Angelica bushy stunt virus	NA	Viruses; Riboviria; Pararnavirae; Artverviricota; Revtraviricetes; Ortervirales; Caulimoviridae; Caulimovirus
GCA_000872265.1	13071	7	Pineapple mealybug wiltassociated virus 1	NA	Viruses; ssRNA viruses; ssRNA positive-strand viruses, no DNA stage; Closteroviridae; Ampelovirus
GCA_001590395.1	2022	2	Lake Sarahassociated circular molecule 12	NA	Viruses; unclassified viruses
GCA_004129275.1	7863	1	Fusarium graminearum mycotymovirus 1	NA	Viruses; Riboviria; Orthornavirae; Kitrinoviricota; Alsuviricetes; Tymovirales; unclassified Tymovirales
GCA_001602065.1	6017	6	Ixeridium yellow mottle virus 1	NA	Viruses; ssRNA viruses; ssRNA positive-strand viruses, no DNA stage; Luteoviridae; unclassified Luteoviridae
GCA_009731995.1	12393	4	Oya virus	SC0806	Viruses; Riboviria; Orthornavirae; Negarnaviricota; Polyploviricotina; Ellioviricetes; Bunyavirales; Peribunyaviridae; Orthobunyavirus; Cat Que orthobunyavirus
GCA_002219665.1	11775	3	Squash chlorotic leaf spot virus	NA	Viruses; ssRNA viruses; ssRNA positive-strand viruses, no DNA stage; Picornavirales; Secoviridae; Torradovirus
GCA_001736255.1	42418	64	Salmonella phage 118970_sal4	NA	Viruses; dsDNA viruses, no RNA stage; Caudovirales; Podoviridae; unclassified Podoviridae
GCA_000865825.1	407339	472	Emiliania huxleyi virus 86	NA	Viruses; dsDNA viruses, no RNA stage; Phycodnaviridae; Coccolithovirus
GCA_004128175.1	18977	3	Tunis virus	BrestArT2756	Viruses; unclassified viruses
GCA_018595025.1	18540	3	Sapphire II virus	75V8196	Viruses; unclassified viruses
GCA_004128035.1	18537	3	Great Saltee virus	RML 599726	Viruses; unclassified viruses
GCA_004128235.1	18491	3	Zirqa virus	Por 7866	Viruses; unclassified viruses
GCA_004128015.1	17905	3	Avalon virus	CanAr173	Viruses; unclassified viruses
GCA_001629885.2	23909	11	Callinectes sapidus reovirus 1	NA	Viruses; dsRNA viruses; Reoviridae; unclassified Reoviridae
GCA_001766565.1	7725	3	Privet leaf blotchassociated virus	NA	Viruses; ssRNA viruses; ssRNA positive-strand viruses, no DNA stage; Idaeovirus; unclassified Idaeovirus

gca	genome.size	proteome.size	phage.name	strain	Taxonomy
GCA_008799955.1	7338	3	Bovine calicivirus strain Jena	NA	Viruses; Riboviria; Orthornavirae; Pisuviricota; Pisoniviricetes; Picornavirales; Caliciviridae; Norovirus; Norwalk virus; Norovirus GIII
GCA_001714355.1	7484	2	Bovine calicivirus strain Kirklareli	NA	Viruses; ssRNA viruses; ssRNA positive-strand viruses, no DNA stage; Caliciviridae; Nebovirus; unclassified Nebovirus
GCA_001619035.1	5461	8	Deinbollia mosaic virus	NA	Viruses; ssDNA viruses; Geminiviridae; Begomovirus; unclassified Begomovirus
GCA_001602085.1	362276	491	Brazilian marseillevirus	BH2014	Viruses; dsDNA viruses, no RNA stage; Marseilleviridae; Marseillevirus; unclassified Marseillevirus
GCA_001698355.1	9808	1	Picornavirales Bu3	NA	Viruses; ssRNA viruses; ssRNA positive-strand viruses, no DNA stage; Picornavirales; unclassified Picornavirales
GCA_001706865.1	9224	1	Picornavirales Bu1	NA	Viruses; Riboviria; Orthornavirae; Pisuviricota; Pisoniviricetes; Picornavirales; unclassified Picornavirales; Husavirus; Husa-like virus KS-2016a
GCA_001881875.1	45342	74	Salmonella phage 64795_sal3	NA	Viruses; unclassified phages
GCA_001882095.1	77375	135	Salmonella phage 118970_sal3	NA	Viruses; unclassified phages
GCA_002757355.1	42126	64	Salmonella phage 101962B_sal5	NA	Viruses; Duplodnaviria; Heunggongvirae; Uroviricota; Caudoviricetes; Caudovirales; Podoviridae; Lederbergvirus; unclassified Lederbergvirus
GCA_002757375.1	42850	64	Salmonella phage 146851_sal5	NA	Viruses; Duplodnaviria; Heunggongvirae; Uroviricota; Caudoviricetes; Caudovirales; Podoviridae; Lederbergvirus; unclassified Lederbergvirus
GCA_002757395.1	42154	64	Salmonella phage 146851_sal4	NA	Viruses; Duplodnaviria; Heunggongvirae; Uroviricota; Caudoviricetes; Caudovirales; Podoviridae; Lederbergvirus; unclassified Lederbergvirus
GCA_002757415.1	42134	64	Salmonella phage 64795_sal4	NA	Viruses; Duplodnaviria; Heunggongvirae; Uroviricota; Caudoviricetes; Caudovirales; Podoviridae; Lederbergvirus; unclassified Lederbergvirus
GCA_001881795.1	40443	60	Salmonella phage 103203_sal5	NA	Viruses; dsDNA viruses, no RNA stage; Caudovirales; Podoviridae; unclassified Podoviridae
GCA_002757435.1	42428	64	Salmonella phage 103203_sal4	NA	Viruses; Duplodnaviria; Heunggongvirae; Uroviricota; Caudoviricetes; Caudovirales; Podoviridae; Lederbergvirus; unclassified Lederbergvirus
GCA_001881935.1	59518	71	Salmonella phage 118970_sal1	NA	Viruses; dsDNA viruses, no RNA stage; Caudovirales; Siphoviridae; Chivirus; unclassified Chilikevirus
GCA_001882075.1	114180	168	Salmonella phage 118970_sal2	NA	Viruses; dsDNA viruses, no RNA stage; Caudovirales; Siphoviridae; T5virus; unclassified T5likevirus
GCA_001884575.1	125114	188	Salmonella phage 100268_sal2	NA	Viruses; dsDNA viruses, no RNA stage; Caudovirales; Siphoviridae; T5virus; unclassified T5likevirus
GCA_900078345.1	85865	129	Enterococcus phage VFW	NA	Viruses; Duplodnaviria; Heunggongvirae; Uroviricota; Caudoviricetes; Caudovirales; Siphoviridae; unclassified Siphoviridae
GCA_007647305.1	34037	48	Streptococcus phage P738	NA	Viruses; Duplodnaviria; Heunggongvirae; Uroviricota; Caudoviricetes; Caudovirales; Siphoviridae; unclassified Siphoviridae
GCA_002603385.1	43226	57	Flavobacterium phage 23T	NA	Viruses; Duplodnaviria; Heunggongvirae; Uroviricota; Caudoviricetes; Caudovirales; Siphoviridae; Unahavirus; Flavobacterium virus 23T
GCA_001885365.1	42702	58	Flavobacterium phage Fpv10	NA	Viruses; unclassified phages
GCA_001884635.1	42696	58	Flavobacterium phage Fpv11	NA	Viruses; unclassified phages
GCA_001884595.1	89010	80	Flavobacterium phage Fpv2	NA	Viruses; unclassified phages
GCA_001882215.1	89937	82	Flavobacterium phage Fpv20	NA	Viruses; unclassified phages
GCA_001881895.1	88421	79	Flavobacterium phage Fpv3	NA	Viruses; unclassified phages
GCA_001882235.1	42696	58	Flavobacterium phage Fpv5	NA	Viruses; unclassified phages
GCA_001881855.1	42820	58	Flavobacterium phage Fpv6	NA	Viruses; unclassified phages
GCA_001881775.1	42894	58	Flavobacterium phage Fpv7	NA	Viruses; unclassified phages
GCA_001881815.1	42696	58	Flavobacterium phage Fpv8	NA	Viruses; unclassified phages
GCA_001745715.1	32729	49	Streptococcus virus 9871	NA	Viruses; dsDNA viruses, no RNA stage; Caudovirales; Siphoviridae; unclassified Siphoviridae
GCA_001744395.1	33105	49	Streptococcus virus 9872	NA	Viruses; dsDNA viruses, no RNA stage; Caudovirales; Siphoviridae; unclassified Siphoviridae
GCA_002609485.1	32813	48	Streptococcus virus 9873	NA	Viruses; Duplodnaviria; Heunggongvirae; Uroviricota; Caudoviricetes; Caudovirales; Siphoviridae; unclassified Siphoviridae
GCA_001743715.1	32649	48	Streptococcus virus 9874	NA	Viruses; dsDNA viruses, no RNA stage; Caudovirales; Siphoviridae; unclassified Siphoviridae
GCA_001743835.1	251042	291	Bacillus phage AR9	NA	Viruses; dsDNA viruses, no RNA stage; Caudovirales; Myoviridae; unclassified Myoviridae

gca	genome.size	proteome.size	phage.name	strain	Taxonomy
GCA_001744895.1	41005	60	Lactobacillus phage PLE3	NA	Viruses; dsDNA viruses, no RNA stage; Caudovirales; Siphoviridae; unclassified Siphoviridae
GCA_001745555.1	35068	51	Lactobacillus phage PLE2	NA	Viruses; dsDNA viruses, no RNA stage; Caudovirales; Siphoviridae; unclassified Siphoviridae
GCA_004114835.1	18642	3	Saphire II virus	RML 5232314	Viruses; Riboviria; Orthornavirae; Negarnaviricota; Polyploviricotina; Ellioviricetes; Bunyavirales; Nairoviridae; Orthonairovirus; unclassified Nairovirus
GCA_001885345.1	185102	221	Synechococcus phage SWAM1	NA	Viruses; dsDNA viruses, no RNA stage; Caudovirales; Myoviridae; unclassified Myoviridae
GCA_001882115.1	186386	229	Synechococcus phage SWAM2	NA	Viruses; dsDNA viruses, no RNA stage; Caudovirales; Myoviridae; unclassified Myoviridae
GCA_001605795.1	17608	12	Grapevine leafrollassociated virus 13	NA	Viruses; ssRNA viruses; ssRNA positive-strand viruses, no DNA stage; Closteroviridae; Ampelovirus; unclassified Ampelovirus
GCA_002609505.1	103218	229	Pseudomonas phage phiPMW	NA	Viruses; Duplodnaviria; Heunggongvirae; Uroviricota; Caudoviricetes; Caudovirales; Myoviridae; Plaisancevirus; Pseudomonas virus PMW
GCA_001744235.1	165772	268	Escherichia phage vB_EcoMUFV13	NA	Viruses; dsDNA viruses, no RNA stage; Caudovirales; Myoviridae; unclassified Myoviridae
GCA_001755685.1	54751	96	Mycobacterium phage Brocalys	NA	Viruses; dsDNA viruses, no RNA stage; Caudovirales; Siphoviridae; unclassified Siphoviridae
GCA_002757455.1	74896	143	Mycobacterium phage TeardropMSU	NA	Viruses; Duplodnaviria; Heunggongvirae; Uroviricota; Caudoviricetes; Caudovirales; Siphoviridae; Kostyavirus; unclassified Kostyavirus
GCA_002609525.1	158136	237	Pseudomonas phage pf16	NA	Viruses; Duplodnaviria; Heunggongvirae; Uroviricota; Caudoviricetes; Caudovirales; Myoviridae; Chakrabartyvirus; Pseudomonas virus pf16
GCA_001745955.1	159878	259	Salmonella phage vB_SnwM_CGG41	NA	Viruses; dsDNA viruses, no RNA stage; Caudovirales; Myoviridae; Tevenvirinae; T4virus; unclassified T4virus
GCA_001744635.1	40273	66	Lactococcus phage PLgT1	NA	Viruses; dsDNA viruses, no RNA stage; Caudovirales; Siphoviridae; unclassified Siphoviridae
GCA_002629845.1	42351	49	Pseudomonas phage phiNFS	NA	Viruses; Duplodnaviria; Heunggongvirae; Uroviricota; Caudoviricetes; Caudovirales; Autographiviridae; Krylovirinae; Phikmvvirus; Pseudomonas virus NFS
GCA_001743955.1	93129	172	Pseudomonas phage phiMK	NA	Viruses; dsDNA viruses, no RNA stage; Caudovirales; Myoviridae; Felixo1virus; unclassified FelixO1likevirus
GCA_001744215.1	103445	122	Salmonella phage SJ46	NA	Viruses; dsDNA viruses, no RNA stage; Caudovirales; Myoviridae; P1virus; unclassified Punalikevirus
GCA_001754825.1	53486	97	Mycobacterium phage Loser	NA	Viruses; dsDNA viruses, no RNA stage; Caudovirales; Siphoviridae; unclassified Siphoviridae
GCA_001754385.1	52561	91	Mycobacterium phage ArcherNM	NA	Viruses; dsDNA viruses, no RNA stage; Caudovirales; Siphoviridae; unclassified Siphoviridae
GCA_002609545.1	48589	74	Bacillus phage Leo2	NA	Viruses; Duplodnaviria; Heunggongvirae; Uroviricota; Caudoviricetes; Caudovirales; Siphoviridae; Andromedavirus; unclassified Andromedavirus
GCA_002757475.1	43265	54	Pseudomonas phage PaMx33	NA	Viruses; Duplodnaviria; Heunggongvirae; Uroviricota; Caudoviricetes; Caudovirales; Podoviridae; unclassified Podoviridae
GCA_002757495.1	43733	56	Pseudomonas phage PaMx35	NA	Viruses; Duplodnaviria; Heunggongvirae; Uroviricota; Caudoviricetes; Caudovirales; Podoviridae; unclassified Podoviridae
GCA_002757515.1	43490	55	Pseudomonas phage PaMx41	NA	Viruses; Duplodnaviria; Heunggongvirae; Uroviricota; Caudoviricetes; Caudovirales; Podoviridae; unclassified Podoviridae
GCA_002757535.1	43223	54	Pseudomonas phage PaMx43	NA	Viruses; Duplodnaviria; Heunggongvirae; Uroviricota; Caudoviricetes; Caudovirales; Podoviridae; unclassified Podoviridae
GCA_002757555.1	43266	54	Pseudomonas phage PaMx46	NA	Viruses; Duplodnaviria; Heunggongvirae; Uroviricota; Caudoviricetes; Caudovirales; Podoviridae; unclassified Podoviridae
GCA_002624325.1	273501	324	Erwinia phage vB_EamM_DeimosMinion	NA	Viruses; Duplodnaviria; Heunggongvirae; Uroviricota; Caudoviricetes; Caudovirales; Myoviridae; Agricanvirus; Erwinia virus Deimos
GCA_002624345.1	271182	317	Erwinia phage vB_EamM_RAY	NA	Viruses; Duplodnaviria; Heunggongvirae; Uroviricota; Caudoviricetes; Caudovirales; Myoviridae; Agricanvirus; Erwinia virus RAY
GCA_002624365.1	271088	322	Erwinia phage vB_EamM_Simmy50	NA	Viruses; Duplodnaviria; Heunggongvirae; Uroviricota; Caudoviricetes; Caudovirales; Myoviridae; Agricanvirus; Erwinia virus Simmy50
GCA_002624385.1	273224	321	Erwinia phage vB_EamM_Special G	NA	Viruses; Duplodnaviria; Heunggongvirae; Uroviricota; Caudoviricetes; Caudovirales; Myoviridae; Agricanvirus; Erwinia virus SpecialG
GCA_000843365.1	6882	14	Vibrio phage VSK	NA	Viruses; ssDNA viruses; Inoviridae; Inovirus
GCA_003179775.1	7810	7	Canis familiaris papillomavirus 18	NA	Viruses; Monodnaviria; Shotokuvirae; Cossaviricota; Papovaviricetes; Zurhausenvirales; Papillomaviridae; Firstpapillomavirinae; Chipapillomavirus; Chipapillomavirus 1
GCA_001678435.1	2715	1	Cronartium ribicola mitovirus 1	NA	Viruses; ssRNA viruses; ssRNA positive-strand viruses, no DNA stage; Narnaviridae; Mitovirus; unclassified Mitovirus
GCA_001678275.1	2471	1	Cronartium ribicola mitovirus 2	NA	Viruses; ssRNA viruses; ssRNA positive-strand viruses, no DNA stage; Narnaviridae; Mitovirus; unclassified Mitovirus
GCA_001678355.1	2522	1	Cronartium ribicola mitovirus 3	NA	Viruses; ssRNA viruses; ssRNA positive-strand viruses, no DNA stage; Narnaviridae; Mitovirus; unclassified Mitovirus
GCA_001678315.1	2479	1	Cronartium ribicola mitovirus 4	NA	Viruses; ssRNA viruses; ssRNA positive-strand viruses, no DNA stage; Narnaviridae; Mitovirus; unclassified Mitovirus

gca	genome.size	proteome.size	phage.name	strain	Taxonomy
GCA_001678395.1	2631	1	Cronartium ribicola mitovirus 5	NA	Viruses; ssRNA viruses; ssRNA positive-strand viruses, no DNA stage; Narnaviridae; Mitovirus; unclassified Mitovirus
GCA_003179895.1	7898	8	Macaca fuscata papillomavirus 1	NA	Viruses; Monodnaviria; Shotokuvirae; Cossaviricota; Papovaviricetes; Zurhausenvirales; Papillomaviridae; Firstpapillomavirinae; Alphapapillomavirus; Alphapapillomavirus 12
GCA_002116155.1	4196	4	Ixeridium yellow mottle virus 2	NA	Viruses; ssRNA viruses; ssRNA positive-strand viruses, no DNA stage; Tombusviridae; Umbravirus; unclassified Umbravirus
GCA_002600205.1	68327	96	Mycobacterium phage Potter	NA	Viruses; Duplodnaviria; Heunggongvirae; Uroviricota; Caudoviricetes; Caudovirales; Siphoviridae; Bclasvirinae; Pegunavirus; Mycobacterium virus Soto
GCA_002597765.1	51370	87	Mycobacterium phage Romney	NA	Viruses; Duplodnaviria; Heunggongvirae; Uroviricota; Caudoviricetes; Caudovirales; Siphoviridae; Fromanvirus; Mycobacterium virus Peaches
GCA_003032725.1	13296	8	Beatrice Hill virus	NA	Viruses; Riboviria; Orthornavirae; Negarnaviricota; Haploviricotina; Monjiviricetes; Mononegavirales; Rhabdoviridae; Tibrovirus; Beatrice Hill tibrovirus
GCA_006297945.1	12418	4	Thimiri orthobunyavirus	VRC 66414	Viruses; Riboviria; Orthornavirae; Negarnaviricota; Polyploviricotina; Ellioviricetes; Bunyavirales; Peribunyaviridae; Orthobunyavirus
GCA_001629965.1	1335	2	Cucurbit yellow mosaic alphasatellite	NA	Viruses; Satellites; Satellite Nucleic Acids; Single stranded DNA satellites; Alphasatellites; Begomovirus-associated alphasatellites; unclassified Begomovirus-associated alphasatellites
GCA_004132625.1	3999	2	Porcine parvovirus 7	NA	Viruses; Monodnaviria; Shotokuvirae; Cossaviricota; Quintoviricetes; Piccovirales; Parvoviridae; Hamaparvovirinae; Chaphamaparvovirus; Ungulate chaphamaparvovirus 1
GCA_003033575.1	2601	2	Human feces smacovirus 2	SmaCV2	Viruses; Monodnaviria; Shotokuvirae; Cressdnaviricota; Arfiviricetes; Cremevirales; Smacoviridae; Porprismacovirus; Human associated porprismacovirus 1
GCA_002271185.1	2560	2	Human feces smacovirus 3	SmaCV321	Viruses; unclassified viruses; Smacovirusgroup
GCA_003726975.1	2937	2	Human feces pecovirus	PeCVPE	Viruses; unclassified viruses
GCA_002601685.1	52946	96	Mycobacterium phage Eidsmoe	NA	Viruses; Duplodnaviria; Heunggongvirae; Uroviricota; Caudoviricetes; Caudovirales; Siphoviridae; Fromanvirus; Mycobacterium virus Alma
GCA_001661835.1	10864	1	Culiseta flavivirus	50213	Viruses; ssRNA viruses; ssRNA positive-strand viruses, no DNA stage; Flaviviridae; unclassified Flaviviridae
GCA_002022215.1	10884	1	Xishuangbanna aedes flavivirus	NA	Viruses; ssRNA viruses; ssRNA positive-strand viruses, no DNA stage; Flaviviridae; unclassified Flaviviridae
GCA_001746055.1	17861	20	Staphylococcus phage SLPW	NA	Viruses; dsDNA viruses, no RNA stage; Caudovirales; Podoviridae; unclassified Podoviridae
GCA_002757575.1	43698	72	Mycobacterium phage Xerxes	NA	Viruses; Duplodnaviria; Heunggongvirae; Uroviricota; Caudoviricetes; Caudovirales; Siphoviridae; Nclasvirinae; Charlievirus; unclassified Charlievirus
GCA_001755245.1	43516	66	Mycobacterium phage Panchino	NA	Viruses; dsDNA viruses, no RNA stage; Caudovirales; Siphoviridae; unclassified Siphoviridae
GCA_001755665.1	42395	69	Mycobacterium phage Xeno	NA	Viruses; dsDNA viruses, no RNA stage; Caudovirales; Siphoviridae; unclassified Siphoviridae
GCA_002609585.1	43478	67	Mycobacterium phage SkinnyPete	NA	Viruses; Duplodnaviria; Heunggongvirae; Uroviricota; Caudoviricetes; Caudovirales; Siphoviridae; Nclasvirinae; Redivirus; Mycobacterium virus Skinnyp
GCA_002757595.1	43679	73	Mycobacterium phage Pipsqueaks	NA	Viruses; Duplodnaviria; Heunggongvirae; Uroviricota; Caudoviricetes; Caudovirales; Siphoviridae; Nclasvirinae; Charlievirus; Mycobacterium virus Pipsqueaks
GCA_001754805.1	44872	67	Mycobacterium phage Phrann	NA	Viruses; dsDNA viruses, no RNA stage; Caudovirales; Siphoviridae; unclassified Siphoviridae
GCA_004789955.1	12726	4	Enseada virus	76V25880	Viruses; Riboviria; Orthornavirae; Negarnaviricota; Polyploviricotina; Ellioviricetes; Bunyavirales; Peribunyaviridae; Orthobunyavirus; Enseada orthobunyavirus
GCA_002609625.1	47881	74	Gordonia phage Attis	NA	Viruses; Duplodnaviria; Heunggongvirae; Uroviricota; Caudoviricetes; Caudovirales; Siphoviridae; Attisvirus; Gordonia virus Attis
GCA_001754145.1	53717	86	Gordonia phage BaxterFox	NA	Viruses; dsDNA viruses, no RNA stage; Caudovirales; Siphoviridae; unclassified Siphoviridae
GCA_002609785.1	75380	99	Gordonia phage Benczkowski14	NA	Viruses; Duplodnaviria; Heunggongvirae; Uroviricota; Caudoviricetes; Caudovirales; Siphoviridae; Demosthenesvirus; Gordonia virus Kvothe
GCA_001754365.1	50636	75	Gordonia phage BetterKatz	NA	Viruses; dsDNA viruses, no RNA stage; Caudovirales; Siphoviridae; unclassified Siphoviridae
GCA_001755225.1	43982	67	Gordonia phage Emalyn	NA	Viruses; dsDNA viruses, no RNA stage; Caudovirales; Siphoviridae; unclassified Siphoviridae
GCA_001755645.1	49894	78	Gordonia phage Guacamole	NA	Viruses; dsDNA viruses, no RNA stage; Caudovirales; Siphoviridae; unclassified Siphoviridae
GCA_001754785.1	76972	108	Gordonia phage Hotorobo	NA	Viruses; dsDNA viruses, no RNA stage; Caudovirales; Siphoviridae; unclassified Siphoviridae
GCA_002622945.1	75380	99	Gordonia phage Katyusha	NA	Viruses; Duplodnaviria; Heunggongvirae; Uroviricota; Caudoviricetes; Caudovirales; Siphoviridae; Demosthenesvirus; Gordonia virus Katyusha
GCA_001754345.1	50346	80	Gordonia phage Kita	NA	Viruses; dsDNA viruses, no RNA stage; Caudovirales; Siphoviridae; unclassified Siphoviridae
GCA_001755205.1	42979	70	Gordonia phage Lucky10	NA	Viruses; dsDNA viruses, no RNA stage; Caudovirales; Siphoviridae; unclassified Siphoviridae
GCA_001745275.1	53431	84	Gordonia phage Nymphadora	NA	Viruses; dsDNA viruses, no RNA stage; Caudovirales; Siphoviridae; unclassified Siphoviridae

gca	genome.size	proteome.size	phage.name	strain	Taxonomy
GCA_001755625.1	49286	80	Gordonia phage Obliviate	NA	Viruses; dsDNA viruses, no RNA stage; Caudovirales; Siphoviridae; unclassified Siphoviridae
GCA_001745935.1	59265	87	Gordonia phage Phinally	NA	Viruses; dsDNA viruses, no RNA stage; Caudovirales; Siphoviridae; unclassified Siphoviridae
GCA_001754765.2	46895	71	Gordonia phage Schwabeltier	NA	Viruses; dsDNA viruses, no RNA stage; Caudovirales; Siphoviridae; unclassified Siphoviridae
GCA_001754325.1	47880	74	Gordonia phage SoilAssassin	NA	Viruses; dsDNA viruses, no RNA stage; Caudovirales; Siphoviridae; unclassified Siphoviridae
GCA_001755185.1	50127	83	Gordonia phage UmaThurman	NA	Viruses; dsDNA viruses, no RNA stage; Caudovirales; Siphoviridae; unclassified Siphoviridae
GCA_001755605.1	59337	89	Gordonia phage Vivi2	NA	Viruses; dsDNA viruses, no RNA stage; Caudovirales; Siphoviridae; unclassified Siphoviridae
GCA_001754745.1	51884	86	Gordonia phage Yeezy	NA	Viruses; dsDNA viruses, no RNA stage; Caudovirales; Siphoviridae; unclassified Siphoviridae
GCA_001754305.1	98136	198	Gordonia phage Yvonnetastic	NA	Viruses; dsDNA viruses, no RNA stage; Caudovirales; Siphoviridae; unclassified Siphoviridae
GCA_002599865.1	50839	93	Mycobacterium phage Malinsilva	NA	Viruses; Duplodnaviria; Heunggongvirae; Uroviricota; Caudoviricetes; Caudovirales; Siphoviridae; Fromanvirus; unclassified Fromanvirus
GCA_002597805.1	51371	86	Mycobacterium phage Burger	NA	Viruses; Duplodnaviria; Heunggongvirae; Uroviricota; Caudoviricetes; Caudovirales; Siphoviridae; Fromanvirus; Mycobacterium virus Peaches
GCA_002609645.1	53932	94	Mycobacterium phage ChipMunk	NA	Viruses; Duplodnaviria; Heunggongvirae; Uroviricota; Caudoviricetes; Caudovirales; Siphoviridae; Fromanvirus; unclassified Fromanvirus
GCA_001755165.1	53935	93	Mycobacterium phage EvilGenius	NA	Viruses; dsDNA viruses, no RNA stage; Caudovirales; Siphoviridae; unclassified Siphoviridae
GCA_002599885.1	50892	90	Mycobacterium phage Hercules11	NA	Viruses; Duplodnaviria; Heunggongvirae; Uroviricota; Caudoviricetes; Caudovirales; Siphoviridae; Fromanvirus; unclassified Fromanvirus
GCA_002597785.1	51162	88	Mycobacterium phage Holli	NA	Viruses; Duplodnaviria; Heunggongvirae; Uroviricota; Caudoviricetes; Caudovirales; Siphoviridae; Fromanvirus; Mycobacterium virus Peaches
GCA_002757615.1	52160	101	Mycobacterium phage Kazan	NA	Viruses; Duplodnaviria; Heunggongvirae; Uroviricota; Caudoviricetes; Caudovirales; Siphoviridae; Fromanvirus; unclassified Fromanvirus
GCA_001755585.1	48670	81	Mycobacterium phage Shipwreck	NA	Viruses; dsDNA viruses, no RNA stage; Caudovirales; Siphoviridae; unclassified Siphoviridae
GCA_002609665.1	34742	55	Streptomyces phage Chymera	NA	Viruses; Duplodnaviria; Heunggongvirae; Uroviricota; Caudoviricetes; Caudovirales; Siphoviridae; unclassified Siphoviridae
GCA_014883415.1	7469	1	Hunnivirus 05VZ	NA	Viruses; Riboviria; Orthornavirae; Pisuviricota; Pisoniviricetes; Picornavirales; Picornaviridae; Hunnivirus; unclassified Hunnivirus
GCA_014883395.1	702	1	Hunnivirus 83GR	NA	Viruses; Riboviria; Orthornavirae; Pisuviricota; Pisoniviricetes; Picornavirales; Picornaviridae; Hunnivirus; unclassified Hunnivirus
GCA_001619055.1	7470	4	Bovine retrovirus CH15	NA	Viruses; Riboviria; Pararnavirae; Artverviricota; Revtraviricetes; Ortervirales; Retroviridae; Orthoretrovirinae; Betaretrovirus; unclassified Betaretrovirus
GCA_001550425.1	14924	14	Kafue kinda x chacma baboon virus	NA	Viruses; ssRNA viruses; ssRNA positive-strand viruses, no DNA stage; Nidovirales; Arteriviridae; unclassified Arteriviridae
GCA_002867125.1	9208	2	Gentian mosaic virus	NA	Viruses; Riboviria; Orthornavirae; Pisuviricota; Pisoniviricetes; Picornavirales; Secoviridae; Comovirinae; Fabavirus
GCA_002158835.1	3701	2	Rhizoctonia solani dsRNA virus 3	NA	Viruses; dsRNA viruses; Partitiviridae; Alphapartitivirus; unclassified Alphapartitivirus
GCA_001630025.1	5620	6	Barley virus G	NA	Viruses; ssRNA viruses; ssRNA positive-strand viruses, no DNA stage; Luteoviridae; Polerovirus; unclassified Polerovirus
GCA_001634615.1	2748	6	Tomato yellow leaf curl Shuangbai virus Y4536	NA	Viruses; Monodnaviria; Shotokuvirae; Cressdnaviricota; Repensiviricetes; Geplafuvirales; Geminiviridae; Begomovirus; Tomato yellow leaf curl Shuangbai virus
GCA_003029285.2	5277	7	Triumfetta yellow mosaic virus	NA	Viruses; Monodnaviria; Shotokuvirae; Cressdnaviricota; Repensiviricetes; Geplafuvirales; Geminiviridae; Begomovirus
GCA_001629865.1	7367	1	Enterovirus SEVgx	NA	Viruses; ssRNA viruses; ssRNA positive-strand viruses, no DNA stage; Picornavirales; Picornaviridae; Enterovirus; unclassified Enterovirus
GCA_001654305.1	372707	470	Tokyovirus A1	NA	Viruses; dsDNA viruses, no RNA stage; Marseilleviridae; unclassified Marseilleviridae
GCA_001706985.1	2506	1	Alternaria arborescens mitovirus 1	NA	Viruses; ssRNA viruses; ssRNA positive-strand viruses, no DNA stage; Narnaviridae; Mitovirus; unclassified Mitovirus
GCA_002609685.1	94870	132	Saccharomonospora phage PIS 136	NA	Viruses; Duplodnaviria; Heunggongvirae; Uroviricota; Caudoviricetes; Caudovirales; Siphoviridae; unclassified Siphoviridae
GCA_004787615.1	2748	5	Pepper yellow leaf curl virus PSSWS14	NA	Viruses; Monodnaviria; Shotokuvirae; Cressdnaviricota; Repensiviricetes; Geplafuvirales; Geminiviridae; Begomovirus; Pepper yellow leaf curl Indonesia virus 2
GCA_002824765.1	2832	6	Plantago lanceolata latent virus	NA	Viruses; Monodnaviria; Shotokuvirae; Cressdnaviricota; Repensiviricetes; Geplafuvirales; Geminiviridae; Capulavirus
GCA_001634415.1	5625	6	Maize yellow dwarf virusRMV2	NA	Viruses; ssRNA viruses; ssRNA positive-strand viruses, no DNA stage; Luteoviridae; Polerovirus; unclassified Polerovirus; Maize yellow mosaic virus
GCA_001634095.1	5211	2	Penicillium digitatum virus 1	HSRH1	Viruses; dsRNA viruses; Totiviridae; unclassified Totiviridae

gca	genome.size	proteome.size	phage.name	strain	Taxonomy
GCA_002966425.1	1182200	898	Mimivirus Bombay	NA	Viruses; Varidnaviria; Bamfordvirae; Nucleocytoviricota; Megaviricetes; Imitervirales; Mimiviridae; Mimivirus; Acanthamoeba polyphaga mimivirus
GCA_002667315.1	18433	11	Rotavirus A RVACowtcTHA61A1989G10P5	NA	Viruses; Riboviria; Orthornavirae; Duplornaviricota; Resentoviricetes; Reovirales; Reoviridae; Sedoreovirinae; Rotavirus; Rotavirus A; Rotavirus G10
GCA_002667435.1	18356	11	Rotavirus A RVACowtcUSAB2231983G10P11	NA	Viruses; Riboviria; Orthornavirae; Duplornaviricota; Resentoviricetes; Reovirales; Reoviridae; Sedoreovirinae; Rotavirus; Rotavirus A; Rotavirus G10; Rotavirus G10P11
GCA_002667275.1	18375	11	Rotavirus A RVACowtcJPNKK31983G10P11	NA	Viruses; Riboviria; Orthornavirae; Duplornaviricota; Resentoviricetes; Reovirales; Reoviridae; Sedoreovirinae; Rotavirus; Rotavirus A; Rotavirus G10; Rotavirus G10P11
GCA_002667375.1	18408	11	Rotavirus A RVACowtcTHAA441989G10P11	NA	Viruses; Riboviria; Orthornavirae; Duplornaviricota; Resentoviricetes; Reovirales; Reoviridae; Sedoreovirinae; Rotavirus; Rotavirus A; Rotavirus G10; Rotavirus G10P11
GCA_002667395.1	18462	11	Rotavirus A RVACowtcTHAA5101988G8P1	NA	Viruses; Riboviria; Orthornavirae; Duplornaviricota; Resentoviricetes; Reovirales; Reoviridae; Sedoreovirinae; Rotavirus; Rotavirus A; Rotavirus G8
GCA_002667415.1	18397	11	Rotavirus A RVACowtcTHAA5131988G8P1	NA	Viruses; Riboviria; Orthornavirae; Duplornaviricota; Resentoviricetes; Reovirales; Reoviridae; Sedoreovirinae; Rotavirus; Rotavirus A; Rotavirus G8
GCA_000883375.1	7988	4	Peach mosaic virus	202201 CA1	Viruses; ssRNA viruses; ssRNA positive-strand viruses, no DNA stage; Tymovirales; Betaflexiviridae; Trivirinae; Trichovirus
GCA_002194365.1	8216	1	Canine kobuvirus	NA	Viruses; ssRNA viruses; ssRNA positive-strand viruses, no DNA stage; Picornavirales; Picornaviridae; Kobuvirus; unclassified Kobuvirus
GCA_001634535.1	3038	1	Rhizoctonia oryzaesativae mitovirus 1	NA	Viruses; ssRNA viruses; ssRNA positive-strand viruses, no DNA stage; Narnaviridae; Mitovirus; unclassified Mitovirus
GCA_002194465.1	28487	6	Goat torovirus	SZ	Viruses; ssRNA viruses; ssRNA positive-strand viruses, no DNA stage; Nidovirales; Coronaviridae; Torovirinae; Torovirus; unclassified Torovirus
GCA_001744735.1	49167	83	Salmonella phage phSE2	NA	Viruses; dsDNA viruses, no RNA stage; Caudovirales; Siphoviridae; unclassified Siphoviridae
GCA_002609705.1	49178	83	Salmonella phage phSE5	NA	Viruses; Duplodnaviria; Heunggongvirae; Uroviricota; Caudoviricetes; Caudovirales; Drexlerviridae; Tempevirinae; Tlsvirus; unclassified Tlsvirus
GCA_001744615.1	161140	292	Bacillus phage SalinJah	NA	Viruses; dsDNA viruses, no RNA stage; Caudovirales; Myoviridae; unclassified Myoviridae
GCA_002709625.1	76492	133	Mycobacterium phage Loadrie	NA	Viruses; Duplodnaviria; Heunggongvirae; Uroviricota; Caudoviricetes; Caudovirales; Siphoviridae; Bronvirus; Mycobacterium virus Faith1
GCA_001744055.1	39235	45	Escherichia phage 64795_ec1	NA	Viruses; dsDNA viruses, no RNA stage; Caudovirales; Podoviridae; unclassified Podoviridae
GCA_001745375.1	72697	93	Pseudomonas phage PEV2	NA	Viruses; dsDNA viruses, no RNA stage; Caudovirales; Podoviridae; Lit1virus; Pseudomonas virus Ab09
GCA_001746035.1	33697	35	Weissella phage WCP30	NA	Viruses; dsDNA viruses, no RNA stage; Caudovirales; Siphoviridae; unclassified Siphoviridae
GCA_001743935.1	170788	274	Escherichia phage UFVAREG1	NA	Viruses; dsDNA viruses, no RNA stage; Caudovirales; Myoviridae; unclassified Myoviridae
GCA_002609745.1	166423	263	Escherichia phage PE37	NA	Viruses; Duplodnaviria; Heunggongvirae; Uroviricota; Caudoviricetes; Caudovirales; Myoviridae; Tevenvirinae; Tequatrovirus; unclassified Tequatrovirus
GCA_002609765.1	234900	326	Acinetobacter phage vB_AbaM_ME3	NA	Viruses; Duplodnaviria; Heunggongvirae; Uroviricota; Caudoviricetes; Caudovirales; Myoviridae; Metrivirus; Acinetobacter virus ME3
GCA_002743475.1	44601	50	Proteus phage PM 116	NA	Viruses; Duplodnaviria; Heunggongvirae; Uroviricota; Caudoviricetes; Caudovirales; Autographiviridae; Molineuxvirinae; Acadevirus; Proteus virus PM116
GCA_001884695.1	168929	266	Escherichia phage MX01	NA	Viruses; dsDNA viruses, no RNA stage; Caudovirales; Myoviridae; Tevenvirinae; T4virus; unclassified T4virus
GCA_001882255.1	169936	273	Escherichia phage WG01	NA	Viruses; dsDNA viruses, no RNA stage; Caudovirales; Myoviridae; Tevenvirinae; T4virus; unclassified T4virus
GCA_001736795.1	93843	182	Gordonia phage Bachita	NA	Viruses; dsDNA viruses, no RNA stage; Caudovirales; Siphoviridae; unclassified Siphoviridae
GCA_001737015.1	54990	86	Gordonia phage Blueberry	NA	Viruses; dsDNA viruses, no RNA stage; Caudovirales; Siphoviridae; unclassified Siphoviridae
GCA_001736335.1	46570	67	Gordonia phage Bowser	NA	Viruses; dsDNA viruses, no RNA stage; Caudovirales; Siphoviridae; unclassified Siphoviridae
GCA_001736555.1	55524	98	Gordonia phage BritBrat	NA	Viruses; dsDNA viruses, no RNA stage; Caudovirales; Siphoviridae; unclassified Siphoviridae
GCA_001736775.1	92618	179	Gordonia phage ClubL	NA	Viruses; dsDNA viruses, no RNA stage; Caudovirales; Siphoviridae; unclassified Siphoviridae
GCA_001736995.1	46600	68	Gordonia phage Cozz	NA	Viruses; dsDNA viruses, no RNA stage; Caudovirales; Siphoviridae; unclassified Siphoviridae
GCA_001736315.1	74073	95	Gordonia phage Demosthenes	NA	Viruses; dsDNA viruses, no RNA stage; Caudovirales; Siphoviridae; unclassified Siphoviridae
GCA_002609805.1	17118	27	Gordonia phage Jeanie	NA	Viruses; Duplodnaviria; Heunggongvirae; Uroviricota; Caudoviricetes; Caudovirales; Siphoviridae; unclassified Siphoviridae
GCA_002757635.1	80649	115	Gordonia phage Kampe	NA	Viruses; Duplodnaviria; Heunggongvirae; Uroviricota; Caudoviricetes; Caudovirales; Siphoviridae; Orchidvirus; Gordonia virus Orchid; Gordonia phage Orchid
GCA_001698415.1	52689	98	Gordonia phage KatherineG	NA	Viruses; dsDNA viruses, no RNA stage; Caudovirales; Siphoviridae; unclassified Siphoviridae

gca	genome.size	proteome.size	phage.name	strain	Taxonomy
GCA_001736535.1	75462	99	Gordonia phage Kvothe	NA	Viruses; dsDNA viruses, no RNA stage; Caudovirales; Siphoviridae; unclassified Siphoviridae
GCA_001736755.1	17119	27	Gordonia phage McGonagall	NA	Viruses; dsDNA viruses, no RNA stage; Caudovirales; Siphoviridae; unclassified Siphoviridae
GCA_001736975.1	75680	105	Gordonia phage Monty	NA	Viruses; dsDNA viruses, no RNA stage; Caudovirales; Siphoviridae; unclassified Siphoviridae
GCA_001736295.1	93577	163	Gordonia phage OneUp	NA	Viruses; dsDNA viruses, no RNA stage; Caudovirales; Siphoviridae; unclassified Siphoviridae
GCA_001736515.1	80650	114	Gordonia phage Orchid	NA	Viruses; dsDNA viruses, no RNA stage; Caudovirales; Siphoviridae; unclassified Siphoviridae
GCA_002757655.1	80729	115	Gordonia phage PatrickStar	NA	Viruses; Duplodnaviria; Heunggongvirae; Uroviricota; Caudoviricetes; Caudovirales; Siphoviridae; Orchidvirus; Gordonia virus Orchid; Gordonia phage Orchid
GCA_001698375.1	52684	99	Gordonia phage Rosalind	NA	Viruses; dsDNA viruses, no RNA stage; Caudovirales; Siphoviridae; unclassified Siphoviridae
GCA_001698335.1	93139	179	Gordonia phage Smoothie	NA	Viruses; dsDNA viruses, no RNA stage; Caudovirales; Siphoviridae; unclassified Siphoviridae
GCA_001698275.1	52924	99	Gordonia phage Soups	NA	Viruses; dsDNA viruses, no RNA stage; Caudovirales; Siphoviridae; unclassified Siphoviridae
GCA_001736735.1	45858	79	Gordonia phage Splinter	NA	Viruses; dsDNA viruses, no RNA stage; Caudovirales; Siphoviridae; unclassified Siphoviridae
GCA_001737135.1	49768	71	Gordonia phage Utz	NA	Viruses; dsDNA viruses, no RNA stage; Caudovirales; Siphoviridae; unclassified Siphoviridae
GCA_001736455.1	45858	79	Gordonia phage Vendetta	NA	Viruses; dsDNA viruses, no RNA stage; Caudovirales; Siphoviridae; unclassified Siphoviridae
GCA_001736675.1	58308	89	Gordonia phage Wizard	NA	Viruses; dsDNA viruses, no RNA stage; Caudovirales; Siphoviridae; unclassified Siphoviridae
GCA_001736895.1	73752	91	Gordonia phage Woes	NA	Viruses; dsDNA viruses, no RNA stage; Caudovirales; Siphoviridae; unclassified Siphoviridae
GCA_001745255.1	38071	56	Propionibacterium phage PFR1	NA	Viruses; unclassified phages
GCA_001745915.1	39640	58	Propionibacterium phage PFR2	NA	Viruses; unclassified phages
GCA_002609825.1	61371	88	Freshwater phage uvFWCGRAMDCOMC203	NA	Viruses; Duplodnaviria; Heunggongvirae; Uroviricota; Caudoviricetes; Caudovirales; Siphoviridae; unclassified Siphoviridae
GCA_002609845.1	42180	59	Freshwater phage uvFWCGRAMDCOMC403	NA	Viruses; Duplodnaviria; Heunggongvirae; Uroviricota; Caudoviricetes; Caudovirales; Podoviridae; unclassified Podoviridae
GCA_002609865.1	40987	40	Freshwater phage uvFWCGRAMDCOMC429	NA	Viruses; Duplodnaviria; Heunggongvirae; Uroviricota; Caudoviricetes; Caudovirales; Podoviridae; unclassified Podoviridae
GCA_002609885.1	40606	57	Freshwater phage uvFWCGRAMDCOMC440	NA	Viruses; Duplodnaviria; Heunggongvirae; Uroviricota; Caudoviricetes; Caudovirales; Podoviridae; unclassified Podoviridae
GCA_002609905.1	40306	59	Freshwater phage uvFWCGRAMDCOMC449	NA	Viruses; Duplodnaviria; Heunggongvirae; Uroviricota; Caudoviricetes; Caudovirales; Podoviridae; unclassified Podoviridae
GCA_002609925.1	40207	58	Freshwater phage uvFWCGRAMDCOMC455	NA	Viruses; Duplodnaviria; Heunggongvirae; Uroviricota; Caudoviricetes; Caudovirales; Podoviridae; unclassified Podoviridae
GCA_002609945.1	38969	45	Freshwater phage uvFWCGRAMDCOMC493	NA	Viruses; Duplodnaviria; Heunggongvirae; Uroviricota; Caudoviricetes; Caudovirales; Podoviridae; unclassified Podoviridae
GCA_002609965.1	14429	22	Freshwater phage uvFWCGRAMDFOSS50C341	NA	Viruses; Duplodnaviria; Heunggongvirae; Uroviricota; Caudoviricetes; Caudovirales; Siphoviridae; unclassified Siphoviridae
GCA_000851725.1	4152	4	Tobacco bushy top virus	NA	Viruses; ssRNA viruses; ssRNA positive-strand viruses, no DNA stage; Tombusviridae; Umbravirus
GCA_002609985.1	42282	69	Staphylococcus phage P1105	NA	Viruses; Duplodnaviria; Heunggongvirae; Uroviricota; Caudoviricetes; Caudovirales; Siphoviridae; Biseptimavirus; unclassified Biseptimavirus
GCA_001646235.1	24899	12	Sclerotinia sclerotiorum mycoreovirus 4	NA	Viruses; dsRNA viruses; Reoviridae; Spinareovirinae; Mycoreovirus; unclassified Mycoreovirus
GCA_001579335.1	1361	1	Bhindi yellow vein alphasatellite	NA	Viruses; Alphasatellitidae; Geminialphasatellitinae; unclassified Begomovirus-associated alphasatellites
GCA_002117655.1	12158	3	Quezon virus	NA	Viruses; Riboviria; Orthornavirae; Negarnaviricota; Polyploviricotina; Ellioviricetes; Bunyavirales; Hantaviridae; Mammantavirinae; Mobatvirus; Quezon mobatvirus
GCA_002757675.1	33108	46	Vibrio phage VcP032	NA	Viruses; Duplodnaviria; Heunggongvirae; Uroviricota; Caudoviricetes; Caudovirales; Myoviridae; Peduovirinae; Longwoodvirus; Vibrio virus K139
GCA_002924545.1	1208707	996	Powai lake megavirus	NA	Viruses; Varidnaviria; Bamfordvirae; Nucleocytoviricota; Megaviricetes; Imitervirales; Mimiviridae; Mimivirus; unclassified Mimivirus; Megavirus chiliensis
GCA_003005725.1	16694	11	Rotavirus G28	RVAShrewCHNLW92013	Viruses; Riboviria; Orthornavirae; Duplornaviricota; Resentoviricetes; Reovirales; Reoviridae; Sedoreovirinae; Rotavirus; Rotavirus A
GCA_001646535.1	2194	3	Faeces associated gemycircularvirus 14	NA	Viruses; unclassified viruses; Genomoviridae; Gemycircularvirus; unclassified Gemycircularvirus
GCA_001645955.1	2226	3	Faeces associated gemycircularvirus 15	NA	Viruses; unclassified viruses; Genomoviridae; Gemycircularvirus; unclassified Gemycircularvirus

gca	genome.size	proteome.size	phage.name	strain	Taxonomy
GCA_001646135.1	2384	3	Faeces associated gemycircularvirus 16	NA	Viruses; unclassified viruses; Genomoviridae; Gemycircularvirus; unclassified Gemycircularvirus
GCA_001646335.1	2230	3	Faeces associated gemycircularvirus 17	NA	Viruses; unclassified viruses; Genomoviridae; Gemycircularvirus; unclassified Gemycircularvirus
GCA_001646515.1	2240	3	Faeces associated gemycircularvirus 18	NA	Viruses; unclassified viruses; Genomoviridae; Gemycircularvirus; unclassified Gemycircularvirus
GCA_001645935.1	2308	3	Faeces associated gemycircularvirus 19	NA	Viruses; unclassified viruses; Genomoviridae; Gemycircularvirus; unclassified Gemycircularvirus
GCA_001646115.1	2247	3	Faeces associated gemycircularvirus 20	NA	Viruses; unclassified viruses; Genomoviridae; Gemycircularvirus; unclassified Gemycircularvirus
GCA_001646315.1	2125	3	Faeces associated gemycircularvirus 21	NA	Viruses; unclassified viruses; Genomoviridae; Gemycircularvirus; unclassified Gemycircularvirus
GCA_001646495.1	2164	3	Faeces associated gemycircularvirus 22	NA	Viruses; unclassified viruses; Genomoviridae; Gemycircularvirus; unclassified Gemycircularvirus
GCA_001645915.1	2723	2	Bovine faeces associated smacovirus 1	NA	Viruses; unclassified viruses; Smacovirusgroup
GCA_001646095.1	2755	2	Bovine faeces associated smacovirus 2	NA	Viruses; unclassified viruses; Smacovirusgroup
GCA_001646295.1	2623	2	Bovine faeces associated smacovirus 3	NA	Viruses; unclassified viruses; Smacovirusgroup
GCA_001646475.1	2645	2	Bovine faeces associated smacovirus 4	NA	Viruses; unclassified viruses; Smacovirusgroup
GCA_001645895.1	2468	2	Bovine faeces associated smacovirus 5	NA	Viruses; unclassified viruses; Smacovirusgroup
GCA_001646075.1	2568	2	Bovine faeces associated smacovirus 6	NA	Viruses; unclassified viruses; Smacovirusgroup
GCA_001646275.1	2861	2	Sheep faeces associated smacovirus 1	NA	Viruses; unclassified viruses; Smacovirusgroup
GCA_001646455.1	2712	2	Sheep faeces associated smacovirus 2	NA	Viruses; unclassified viruses; Smacovirusgroup
GCA_001645875.1	2608	2	Sheep faeces associated smacovirus 3	NA	Viruses; unclassified viruses; Smacovirusgroup
GCA_000928295.1	2130	3	Sewage associated gemycircularvirus 3	NA	Viruses; unclassified viruses; Genomoviridae; Gemycircularvirus; unclassified Gemycircularvirus
GCA_001646435.1	1474	1	Bovine faeces associated circular DNA molecule 1	NA	Viruses; unclassified viruses
GCA_001645855.1	3060	2	Bovine faeces associated circular DNA virus 1	NA	Viruses; unclassified viruses
GCA_001646215.1	2782	2	Bovine faeces associated circular DNA virus 2	NA	Viruses; unclassified viruses
GCA_001646415.1	2684	2	Chamois faeces associated circular DNA virus 1	NA	Viruses; unclassified viruses
GCA_001646595.1	2355	2	Deer faeces associated circular DNA virus 1	NA	Viruses; unclassified viruses
GCA_001646015.1	2750	2	Duck faeces associated circular DNA virus 1	NA	Viruses; unclassified viruses
GCA_001646195.1	1845	2	Duck faeces associated circular DNA virus 2	NA	Viruses; unclassified viruses
GCA_001646395.1	1856	2	Duck faeces associated circular DNA virus 3	NA	Viruses; unclassified viruses
GCA_001646575.1	2002	2	Llama faeces associated circular DNA virus1	NA	Viruses; unclassified viruses
GCA_001645995.1	1280	1	Porcine faeces associated circular DNA molecule1	NA	Viruses; unclassified viruses
GCA_001646175.1	2589	2	Porcine stoolassociated circular virus	NA	Viruses; unclassified viruses
GCA_003179875.1	7839	8	Canis familiaris papillomavirus 20	NA	Viruses; Monodnaviria; Shotokuvirae; Cossaviricota; Papovaviricetes; Zurhausenvirales; Papillomaviridae; Firstpapillomavirinae; Chipapillomavirus; Chipapillomavirus 1
GCA_001744715.1	58566	74	Pseudomonas phage NP1	NA	Viruses; dsDNA viruses, no RNA stage; Caudovirales; Siphoviridae; unclassified Siphoviridae
GCA_013086935.1	8108	2	Blacklegged tick phlebovirus 3	NA	Viruses; Riboviria; Orthornavirae; Negarnaviricota; Polyploviricotina; Ellioviricetes; Bunyavirales; Phenuiviridae; Ixovirus; Scapularis ixovirus
GCA_001651165.1	11163	4	Lonestar tick chuvirus 1	NA	Viruses; unclassified viruses
GCA_001651125.1	1939	1	Avianlike circovirus	NA	Viruses; ssDNA viruses; Circoviridae; unclassified Circoviridae
GCA_002610005.1	73787	86	Lactobacillus phage P1	NA	Viruses; Duplodnaviria; Heunggongvirae; Uroviricota; Caudoviricetes; Caudovirales; Siphoviridae; Tybeckvirinae; Maenadvirus; Lactobacillus virus P1

gca	genome.size	proteome.size	phage.name	strain	Taxonomy
GCA_001887845.1	11919	5	Gannoruwa bat lyssavirus	NA	Viruses; ssRNA viruses; ssRNA negative-strand viruses; Mononegavirales; Rhabdoviridae; Lyssavirus; unclassified Lyssavirus
GCA_001651145.1	7535	9	Water chestnut soymovirus 1	NA	Viruses; Riboviria; Pararnavirae; Artverviricota; Revtraviricetes; Ortervirales; Caulimoviridae; Soymovirus; unclassified Soymovirus
GCA_001651085.1	9518	2	Fusarium poae dsRNA virus 2	NA	Viruses; dsRNA viruses; unclassified dsRNA viruses
GCA_001722985.1	2397	1	Fusarium poae mitovirus 1	NA	Viruses; ssRNA viruses; ssRNA positive-strand viruses, no DNA stage; Narnaviridae; Mitovirus; unclassified Mitovirus
GCA_001722785.1	2414	1	Fusarium poae mitovirus 2	NA	Viruses; ssRNA viruses; ssRNA positive-strand viruses, no DNA stage; Narnaviridae; Mitovirus; unclassified Mitovirus
GCA_001722865.1	2718	1	Fusarium poae mitovirus 3	NA	Viruses; ssRNA viruses; ssRNA positive-strand viruses, no DNA stage; Narnaviridae; Mitovirus; unclassified Mitovirus
GCA_001722685.1	2387	1	Fusarium poae mitovirus 4	NA	Viruses; ssRNA viruses; ssRNA positive-strand viruses, no DNA stage; Narnaviridae; Mitovirus; unclassified Mitovirus
GCA_001651205.1	9419	2	Fusarium poae dsRNA virus 3	NA	Viruses; dsRNA viruses; unclassified dsRNA viruses
GCA_002088385.1	7461	1	Enterovirus goatJL14	NA	Viruses; ssRNA viruses; ssRNA positive-strand viruses, no DNA stage; Picornavirales; Picornaviridae; Enterovirus; unclassified Enterovirus
GCA_001725875.1	3722	2	Arabidopsis halleri partitivirus 1	NA	Viruses; dsRNA viruses; Partitiviridae; unclassified Partitiviridae
GCA_001722945.1	2297	1	Fusarium poae narnavirus 1	NA	Viruses; ssRNA viruses; ssRNA positive-strand viruses, no DNA stage; Narnaviridae; Narnavirus; unclassified Narnavirus
GCA_001722765.1	2054	1	Fusarium poae narnavirus 2	NA	Viruses; ssRNA viruses; ssRNA positive-strand viruses, no DNA stage; Narnaviridae; Narnavirus; unclassified Narnavirus
GCA_001723045.1	4816	2	Fusarium poae partitivirus 2	NA	Viruses; dsRNA viruses; Partitiviridae; unclassified Partitiviridae
GCA_001722845.1	4662	2	Fusarium poae virus 1240374	NA	Viruses; dsRNA viruses; Partitiviridae; Betapartitivirus; unclassified Betapartitivirus
GCA_001722925.1	5124	2	Fusarium poae victorivirus 1	NA	Viruses; dsRNA viruses; Totiviridae; Victorivirus; unclassified Victorivirus
GCA_001722745.1	6379	2	Fusarium poae fusarivirus 1	NA	Viruses; ssRNA viruses; unclassified ssRNA viruses; Fusariviridae
GCA_001723025.1	8537	3	Fusarium poae alternavirus 1	NA	Viruses; unclassified viruses
GCA_001722825.1	9148	2	Fusarium poae mycovirus 1	NA	Viruses; dsRNA viruses; unclassified dsRNA viruses
GCA_001722905.1	4224	1	Fusarium poae mycovirus 2	NA	Viruses; dsRNA viruses; unclassified dsRNA viruses
GCA_001722725.1	7747	1	Fusarium poae negativestranded virus 1	NA	Viruses; ssRNA viruses; ssRNA negative-strand viruses; unclassified ssRNA negative-strand viruses
GCA_001723005.1	6608	1	Fusarium poae negativestranded virus 2	NA	Viruses; ssRNA viruses; ssRNA negative-strand viruses; unclassified ssRNA negative-strand viruses
GCA_003033815.1	8802	2	Grapevine fabavirus	NA	Viruses; Riboviria; Orthornavirae; Pisuviricota; Pisoniviricetes; Picornavirales; Secoviridae; Comovirinae; Fabavirus
GCA_001654205.1	128058	130	Catopsilia pomona nucleopolyhedrovirus	NA	Viruses; dsDNA viruses, no RNA stage; Baculoviridae; Alphabaculovirus; unclassified Alphabaculovirus
GCA_002890375.1	11449	4	Kabuto mountain virus	T32	Viruses; Riboviria; Orthornavirae; Negarnaviricota; Polyploviricotina; Ellioviricetes; Bunyavirales; Phenuiviridae; Uukuvirus; Kabuto mountain uukuvirus
GCA_003055605.1	7268	6	Human papillomavirus 187	NA	Viruses; Monodnaviria; Shotokuvirae; Cossaviricota; Papovaviricetes; Zurhausenvirales; Papillomaviridae; Firstpapillomavirinae; Gammapapillomavirus; Gammapapillomavirus 26
GCA_003147485.1	7275	6	Human papillomavirus type 190	NA	Viruses; Monodnaviria; Shotokuvirae; Cossaviricota; Papovaviricetes; Zurhausenvirales; Papillomaviridae; Firstpapillomavirinae; Gammapapillomavirus; unclassified Gammapapillomavirus
GCA_003147505.1	7260	6	Human papillomavirus type 194	NA	Viruses; Monodnaviria; Shotokuvirae; Cossaviricota; Papovaviricetes; Zurhausenvirales; Papillomaviridae; Firstpapillomavirinae; Gammapapillomavirus; unclassified Gammapapillomavirus
GCA_002610025.1	53160	60	Ruegeria phage 45A6	NA	Viruses; Duplodnaviria; Heunggongvirae; Uroviricota; Caudoviricetes; Caudovirales; Podoviridae; unclassified Podoviridae
GCA_000882155.1	7781	5	African oil palm ringspot virus	NA	Viruses; ssRNA viruses; ssRNA positive-strand viruses, no DNA stage; Tymovirales; Betaflexiviridae; unassigned Betaflexiviridae
GCA_002610045.1	40556	46	Pseudomonas phage VSW3	NA	Viruses; Duplodnaviria; Heunggongvirae; Uroviricota; Caudoviricetes; Caudovirales; Autographiviridae; Napahaivirus; Pseudomonas virus VSW3
GCA_001744595.1	51802	87	Escherichia phage vB_EcoS_NBD2	NA	Viruses; Duplodnaviria; Heunggongvirae; Uroviricota; Caudoviricetes; Caudovirales; Drexlerviridae; Vilniusvirus; Escherichia virus NBD2
GCA_001743915.1	162308	295	Bacillus phage Belinda	NA	Viruses; dsDNA viruses, no RNA stage; Caudovirales; Myoviridae; unclassified Myoviridae
GCA_002610065.1	44677	69	Staphylococcus phage vB_SauS_IMEP5	NA	Viruses; Duplodnaviria; Heunggongvirae; Uroviricota; Caudoviricetes; Caudovirales; Siphoviridae; unclassified Siphoviridae
GCA_002611885.1	162040	292	Bacillus phage Smudge	NA	Viruses; Duplodnaviria; Heunggongvirae; Uroviricota; Caudoviricetes; Caudovirales; Herelleviridae; Bastillevirinae; Wphvirus; unclassified Wphvirus

gca	genome.size	proteome.size	phage.name	strain	Taxonomy
GCA_002604505.1	65237	115	Mycobacterium phage Gattaca	NA	Viruses; Duplodnaviria; Heunggongvirae; Uroviricota; Caudoviricetes; Caudovirales; Siphoviridae; Marvinvirus; Mycobacterium virus Mosmoris
GCA_002610085.1	7190	12	Xanthomonas phage Xf109	NA	Viruses; Monodnaviria; Loebvirae; Hofneiviricota; Faserviricetes; Tubulavirales; Inoviridae; Xylivirus; Xanthomonas virus Xf109
GCA_002610105.1	7929	14	Ralstonia phage Rs551	NA	Viruses; Monodnaviria; Loebvirae; Hofneiviricota; Faserviricetes; Tubulavirales; Inoviridae; Habenivirus; Ralstonia virus RS551
GCA_002610125.1	42279	29	Shewanella phage SFCi1	NA	Viruses; Duplodnaviria; Heunggongvirae; Uroviricota; Caudoviricetes; Caudovirales; Myoviridae; unclassified Myoviridae
GCA_002610145.1	60497	79	Xanthomonas phage Xoosp2	NA	Viruses; Duplodnaviria; Heunggongvirae; Uroviricota; Caudoviricetes; Caudovirales; Siphoviridae; Pamexvirus; unclassified Pamexvirus
GCA_001745235.1	168272	275	Klebsiella phage vB_KpnM_KpV477	NA	Viruses; dsDNA viruses, no RNA stage; Caudovirales; Myoviridae; unclassified Myoviridae
GCA_002610165.2	36753	56	Pseudoalteromonas virus vB_PspPH61	NA	Viruses; Duplodnaviria; Heunggongvirae; Uroviricota; Caudoviricetes; Caudovirales; Podoviridae; unclassified Podoviridae
GCA_001745895.1	39616	55	Morganella phage vB_MmoP_MP2	NA	Viruses; dsDNA viruses, no RNA stage; Caudovirales; Podoviridae; unclassified Podoviridae
GCA_001744575.1	163201	271	Morganella phage vB_MmoM_MP1	NA	Viruses; dsDNA viruses, no RNA stage; Caudovirales; Myoviridae; unclassified Myoviridae
GCA_001743895.1	40749	70	Acinetobacter phage vB_AbaS_TRS1	NA	Viruses; dsDNA viruses, no RNA stage; Caudovirales; Siphoviridae; unclassified Siphoviridae
GCA_002610185.1	38890	47	Escherichia phage ECA2	NA	Viruses; Duplodnaviria; Heunggongvirae; Uroviricota; Caudoviricetes; Caudovirales; Autographiviridae; Studiervirinae; Teetrevirus; Escherichia virus ECA2
GCA_018215305.2	50337	61	Vibrio phage vB_ValP_IME234	NA	Viruses; Duplodnaviria; Heunggongvirae; Uroviricota; Caudoviricetes; Caudovirales; Podoviridae; unclassified Podoviridae
GCA_002745835.1	154938	193	Serratia phage vBSruIME250	NA	Viruses; Duplodnaviria; Heunggongvirae; Uroviricota; Caudoviricetes; Caudovirales; Ackermannviridae; Taipeivirus; Serratia virus IME250
GCA_002211075.1	75147	92	Erwinia phage vB_EamP_Frozen	NA	Viruses; dsDNA viruses, no RNA stage; Caudovirales; Podoviridae; N4virus; unclassified N4likevirus
GCA_002757695.1	75448	92	Erwinia phage vB_EamP_Rexella	NA	Viruses; Duplodnaviria; Heunggongvirae; Uroviricota; Caudoviricetes; Caudovirales; Podoviridae; Johnsonvirus; Erwinia virus Frozen
GCA_002757715.1	71173	84	Erwinia phage vB_EamP_Gutmeister	NA	Viruses; Duplodnaviria; Heunggongvirae; Uroviricota; Caudoviricetes; Caudovirales; Podoviridae; Johnsonvirus; Erwinia virus Frozen
GCA_002925595.1	13514	18	Pseudomonas phage phiYY	NA	Viruses; Riboviria; Orthornavirae; Duplornaviricota; Vidaverviricetes; Mindivirales; Cystoviridae; Cystovirus; Pseudomonas virus phiYY
GCA_001745875.1	42201	51	Klebsiella phage vB_KpnP_KpV475	NA	Viruses; dsDNA viruses, no RNA stage; Caudovirales; Podoviridae; unclassified Podoviridae
GCA_002610205.1	28068	33	Helicobacter phage DeM53M	NA	Viruses; Duplodnaviria; Heunggongvirae; Uroviricota; Caudoviricetes; Caudovirales; Podoviridae; Schmidvirus; unclassified Schmidvirus
GCA_002610225.1	31200	37	Helicobacter phage FrANT170U	NA	Viruses; Duplodnaviria; Heunggongvirae; Uroviricota; Caudoviricetes; Caudovirales; Podoviridae; Schmidvirus; unclassified Schmidvirus
GCA_002610245.1	29388	36	Helicobacter phage FrB41M	NA	Viruses; Duplodnaviria; Heunggongvirae; Uroviricota; Caudoviricetes; Caudovirales; Podoviridae; Schmidvirus; unclassified Schmidvirus
GCA_002610285.1	28565	36	Helicobacter phage FrG12G	NA	Viruses; Duplodnaviria; Heunggongvirae; Uroviricota; Caudoviricetes; Caudovirales; Podoviridae; Schmidvirus; unclassified Schmidvirus
GCA_002610305.1	32975	39	Helicobacter phage FrGC43A	NA	Viruses; Duplodnaviria; Heunggongvirae; Uroviricota; Caudoviricetes; Caudovirales; Podoviridae; Schmidvirus; unclassified Schmidvirus
GCA_002610325.1	31236	37	Helicobacter phage FrMEG235U	NA	Viruses; Duplodnaviria; Heunggongvirae; Uroviricota; Caudoviricetes; Caudovirales; Podoviridae; Schmidvirus; unclassified Schmidvirus
GCA_002610345.1	30071	36	Helicobacter phage Pt1293U	NA	Viruses; Duplodnaviria; Heunggongvirae; Uroviricota; Caudoviricetes; Caudovirales; Podoviridae; Schmidvirus; unclassified Schmidvirus
GCA_002610365.1	27960	32	Helicobacter phage Pt1846U	NA	Viruses; Duplodnaviria; Heunggongvirae; Uroviricota; Caudoviricetes; Caudovirales; Podoviridae; Schmidvirus; unclassified Schmidvirus
GCA_002610385.1	28670	34	Helicobacter phage Pt1918U	NA	Viruses; Duplodnaviria; Heunggongvirae; Uroviricota; Caudoviricetes; Caudovirales; Podoviridae; Schmidvirus; unclassified Schmidvirus
GCA_002610405.1	23008	24	Helicobacter phage Pt21299RU	NA	Viruses; Duplodnaviria; Heunggongvirae; Uroviricota; Caudoviricetes; Caudovirales; Podoviridae; Schmidvirus; unclassified Schmidvirus
GCA_002610425.1	30078	38	Helicobacter phage Pt22899G	NA	Viruses; Duplodnaviria; Heunggongvirae; Uroviricota; Caudoviricetes; Caudovirales; Podoviridae; Schmidvirus; unclassified Schmidvirus
GCA_002610445.1	27572	32	Helicobacter phage Pt4472G	NA	Viruses; Duplodnaviria; Heunggongvirae; Uroviricota; Caudoviricetes; Caudovirales; Podoviridae; Schmidvirus; unclassified Schmidvirus
GCA_002610465.1	25388	33	Helicobacter phage Pt4481G	NA	Viruses; Duplodnaviria; Heunggongvirae; Uroviricota; Caudoviricetes; Caudovirales; Podoviridae; Schmidvirus; unclassified Schmidvirus
GCA_002610485.1	29393	36	Helicobacter phage Pt4497U	NA	Viruses; Duplodnaviria; Heunggongvirae; Uroviricota; Caudoviricetes; Caudovirales; Podoviridae; Schmidvirus; unclassified Schmidvirus
GCA_002610505.1	28341	31	Helicobacter phage Pt5322G	NA	Viruses; Duplodnaviria; Heunggongvirae; Uroviricota; Caudoviricetes; Caudovirales; Podoviridae; Schmidvirus; unclassified Schmidvirus
GCA_002610525.1	29801	34	Helicobacter phage Pt5771G	NA	Viruses; Duplodnaviria; Heunggongvirae; Uroviricota; Caudoviricetes; Caudovirales; Podoviridae; Schmidvirus; unclassified Schmidvirus

gca	genome.size	proteome.size	phage.name	strain	Taxonomy
GCA_002610545.1	27363	32	Helicobacter phage PtB89G	NA	Viruses; Duplodnaviria; Heunggongvirae; Uroviricota; Caudoviricetes; Caudovirales; Podoviridae; Schmidvirus; unclassified Schmidvirus
GCA_002610565.1	30548	39	Helicobacter phage PtB92G	NA	Viruses; Duplodnaviria; Heunggongvirae; Uroviricota; Caudoviricetes; Caudovirales; Podoviridae; Schmidvirus; unclassified Schmidvirus
GCA_002610605.1	30977	37	Helicobacter phage SwA626G	NA	Viruses; Duplodnaviria; Heunggongvirae; Uroviricota; Caudoviricetes; Caudovirales; Podoviridae; Schmidvirus; unclassified Schmidvirus
GCA_002610625.1	30456	35	Helicobacter phage UKEN31U	NA	Viruses; Duplodnaviria; Heunggongvirae; Uroviricota; Caudoviricetes; Caudovirales; Podoviridae; Schmidvirus; unclassified Schmidvirus
GCA_002610645.1	29882	35	Helicobacter phage UKEN32U	NA	Viruses; Duplodnaviria; Heunggongvirae; Uroviricota; Caudoviricetes; Caudovirales; Podoviridae; Schmidvirus; unclassified Schmidvirus
GCA_001736475.1	8506	6	Jasmine virus C	NA	Viruses; ssRNA viruses; ssRNA positive-strand viruses, no DNA stage; Tymovirales; Betaflexiviridae; Quinvirinae; Carlavirus; unclassified Carlavirus
GCA_001654365.1	2683	7	Euphorbia caputmedusae latent virus	NA	Viruses; ssDNA viruses; Geminiviridae; unclassified Geminiviridae; Capulaviruses
GCA_003033845.1	9787	2	Prunus virus F	NA	Viruses; Riboviria; Orthornavirae; Pisuviricota; Pisoniviricetes; Picornavirales; Secoviridae; Comovirinae; Fabavirus
GCA_001661715.1	7788	2	Golden shiner totivirus	NA	Viruses; dsRNA viruses; Totiviridae; unclassified Totiviridae
GCA_001661875.1	9310	1	Pecan mosaicassociated virus	NA	Viruses; ssRNA viruses; ssRNA positive-strand viruses, no DNA stage; Potyviridae; unclassified Potyviridae
GCA_002610665.1	45306	73	Pseudoalteromonas phage vB_PspSH401	NA	Viruses; Duplodnaviria; Heunggongvirae; Uroviricota; Caudoviricetes; Caudovirales; Siphoviridae; unclassified Siphoviridae
GCA_000842545.1	6034	7	Mythimna loreyi densovirus	NA	Viruses; ssDNA viruses; Parvoviridae; Densovirinae; Ambidensovirus; Lepidopteran ambidensovirus 1; Junonia coenia densovirus
GCA_000902575.1	5990	7	Pseudoplusia includens densovirus	NA	Viruses; ssDNA viruses; Parvoviridae; Densovirinae; Ambidensovirus; Lepidopteran ambidensovirus 1; Junonia coenia densovirus
GCA_000883835.1	5759	4	Culex pipiens densovirus	NA	Viruses; ssDNA viruses; Parvoviridae; Densovirinae; Ambidensovirus; Dipteran ambidensovirus 1
GCA_003033195.1	5425	5	Acheta domestica densovirus	NA	Viruses; ssDNA viruses; Parvoviridae; Densovirinae; Ambidensovirus; Orthopteran ambidensovirus 1
GCA_004790015.1	12675	4	Bellavista virus	NA	Viruses; Riboviria; Orthornavirae; Negarnaviricota; Polyploviricotina; Ellioviricetes; Bunyavirales; Peribunyaviridae; Orthobunyavirus; Bellavista orthobunyavirus
GCA_001669845.1	3782	1	Phytomonas serpens narnavirus 1	NA	Viruses; ssRNA viruses; ssRNA positive-strand viruses, no DNA stage; Narnaviridae; Narnavirus; unclassified Narnavirus
GCA_003029295.1	5642	6	Maize yellow mosaic virus	NA	Viruses; Riboviria; Orthornavirae; Kitrinoviricota; Tolucaviricetes; Tolivirales; Luteoviridae; Polerovirus
GCA_004128055.1	4369	3	Leptomonas seymouri Narnalike virus 1	NA	Viruses; Riboviria; Orthornavirae; Lenarviricota; Amabiliviricetes; Wolframvirales; Narnaviridae; unclassified Narnaviridae
GCA_001881975.1	43788	65	Salmonella phage BPS11Q3	NA	Viruses; dsDNA viruses, no RNA stage; Caudovirales; Siphoviridae; unclassified Siphoviridae
GCA_001881995.1	89817	130	Salmonella phage BPS15Q2	NA	Viruses; dsDNA viruses, no RNA stage; Caudovirales; Myoviridae; unclassified Myoviridae
GCA_001675505.1	16671	5	Tospovirus kiwifruitYXW2014	NA	Viruses; ssRNA viruses; ssRNA negative-strand viruses; Bunyaviridae; Tospovirus; unclassified Tospovirus
GCA_000844745.1	1347	1	Ageratum yellow vein betasatellite	NA	Viruses; Tolecusatellitidae; Betasatellite
GCA_900092385.1	257877	301	Phage NCTB	NA	Viruses; Duplodnaviria; Heunggongvirae; Uroviricota; Caudoviricetes; Caudovirales; Myoviridae; unclassified Myoviridae
GCA_000853585.1	23696	12	Golden shiner reovirus	NA	Viruses; Riboviria; Orthornavirae; Duplornaviricota; Resentoviricetes; Reovirales; Reoviridae; Spinareovirinae; Aquareovirus; Aquareovirus C
GCA_018388775.1	141985	201	Staphylococcus virus Remus	NA	Viruses; Duplodnaviria; Heunggongvirae; Uroviricota; Caudoviricetes; Caudovirales; Herelleviridae; Twortvirinae; Silviavirus
GCA_000862185.1	2734	6	Tomato yellow leaf curl China virus	TYLCVCHI	Viruses; ssDNA viruses; Geminiviridae; Begomovirus
GCA_005410505.1	2364	1	Magnaporthe oryzae ourmialike virus	NA	Viruses; Riboviria; Orthornavirae; Lenarviricota; Miaviricetes; Ourlivirales; Botourmiaviridae; Magoulivirus; Magnaporthe magoulivirus 1
GCA_008798355.1	7212	1	Human rhinovirus B4	NA	Viruses; Riboviria; Orthornavirae; Pisuviricota; Pisoniviricetes; Picornavirales; Picornaviridae; Enterovirus; Rhinovirus B
GCA_008797135.1	7108	1	Human rhinovirus A8	ATCC VR1118	Viruses; Riboviria; Orthornavirae; Pisuviricota; Pisoniviricetes; Picornavirales; Picornaviridae; Enterovirus; Rhinovirus A
GCA_008796085.1	7132	1	Human rhinovirus A9	ATCC VR489	Viruses; Riboviria; Orthornavirae; Pisuviricota; Pisoniviricetes; Picornavirales; Picornaviridae; Enterovirus; Rhinovirus A
GCA_008798195.1	7137	1	Human rhinovirus A10	NA	Viruses; Riboviria; Orthornavirae; Pisuviricota; Pisoniviricetes; Picornavirales; Picornaviridae; Enterovirus; Rhinovirus A
GCA_008797115.1	7140	1	Human rhinovirus A13	ATCC VR1123	Viruses; Riboviria; Orthornavirae; Pisuviricota; Pisoniviricetes; Picornavirales; Picornaviridae; Enterovirus; Rhinovirus A
GCA_008798295.1	7134	1	Human rhinovirus A15	NA	Viruses; Riboviria; Orthornavirae; Pisuviricota; Pisoniviricetes; Picornavirales; Picornaviridae; Enterovirus; Rhinovirus A

gca	genome.size	proteome.size	phage.name	strain	Taxonomy
GCA_008797075.1	7135	1	Human rhinovirus A19	ATCC VR1129	Viruses; Riboviria; Orthornavirae; Pisuviricota; Pisoniviricetes; Picornavirales; Picornaviridae; Enterovirus; Rhinovirus A
GCA_008796985.1	7129	1	Human rhinovirus A22	ATCC VR1132	Viruses; Riboviria; Orthornavirae; Pisuviricota; Pisoniviricetes; Picornavirales; Picornaviridae; Enterovirus; Rhinovirus A
GCA_008797675.1	7132	1	Human rhinovirus A24	NA	Viruses; Riboviria; Orthornavirae; Pisuviricota; Pisoniviricetes; Picornavirales; Picornaviridae; Enterovirus; Rhinovirus A
GCA_008796965.1	7126	1	Human rhinovirus A25	ATCC VR1135	Viruses; Riboviria; Orthornavirae; Pisuviricota; Pisoniviricetes; Picornavirales; Picornaviridae; Enterovirus; Rhinovirus A
GCA_008796945.1	7211	1	Human rhinovirus B26	ATCC VR1136	Viruses; Riboviria; Orthornavirae; Pisuviricota; Pisoniviricetes; Picornavirales; Picornaviridae; Enterovirus; Rhinovirus B
GCA_008796065.1	7217	1	Human rhinovirus B27	ATCC VR1137	Viruses; Riboviria; Orthornavirae; Pisuviricota; Pisoniviricetes; Picornavirales; Picornaviridae; Enterovirus; Rhinovirus B
GCA_008797935.1	7099	1	Human rhinovirus A30	NA	Viruses; Riboviria; Orthornavirae; Pisuviricota; Pisoniviricetes; Picornavirales; Picornaviridae; Enterovirus; Rhinovirus A
GCA_008796905.1	7131	1	Human rhinovirus A31	ATCC VR506	Viruses; Riboviria; Orthornavirae; Pisuviricota; Pisoniviricetes; Picornavirales; Picornaviridae; Enterovirus; Rhinovirus A
GCA_008796885.1	7133	1	Human rhinovirus A32	ATCC VR1142	Viruses; Riboviria; Orthornavirae; Pisuviricota; Pisoniviricetes; Picornavirales; Picornaviridae; Enterovirus; Rhinovirus A
GCA_008796865.1	7133	1	Human rhinovirus A33	ATCC VR330	Viruses; Riboviria; Orthornavirae; Pisuviricota; Pisoniviricetes; Picornavirales; Picornaviridae; Enterovirus; Rhinovirus A
GCA_008798155.1	7119	1	Human rhinovirus A34	NA	Viruses; Riboviria; Orthornavirae; Pisuviricota; Pisoniviricetes; Picornavirales; Picornaviridae; Enterovirus; Rhinovirus A
GCA_008798255.1	7136	1	Human rhinovirus A38	NA	Viruses; Riboviria; Orthornavirae; Pisuviricota; Pisoniviricetes; Picornavirales; Picornaviridae; Enterovirus; Rhinovirus A
GCA_008798875.1	7137	1	Human rhinovirus A39	NA	Viruses; Riboviria; Orthornavirae; Pisuviricota; Pisoniviricetes; Picornavirales; Picornaviridae; Enterovirus; Rhinovirus A
GCA_008796845.1	7138	1	Human rhinovirus A40	ATCC VR341	Viruses; Riboviria; Orthornavirae; Pisuviricota; Pisoniviricetes; Picornavirales; Picornaviridae; Enterovirus; Rhinovirus A
GCA_008798335.1	7145	1	Human rhinovirus A41	NA	Viruses; Riboviria; Orthornavirae; Pisuviricota; Pisoniviricetes; Picornavirales; Picornaviridae; Enterovirus; Rhinovirus A
GCA_008796765.1	7132	1	Human rhinovirus A47	ATCC VR1157	Viruses; Riboviria; Orthornavirae; Pisuviricota; Pisoniviricetes; Picornavirales; Picornaviridae; Enterovirus; Rhinovirus A
GCA_008796725.1	7152	1	Human rhinovirus A51	ATCC VR1161	Viruses; Riboviria; Orthornavirae; Pisuviricota; Pisoniviricetes; Picornavirales; Picornaviridae; Enterovirus; Rhinovirus A
GCA_008798035.1	7143	1	Human rhinovirus A53	NA	Viruses; Riboviria; Orthornavirae; Pisuviricota; Pisoniviricetes; Picornavirales; Picornaviridae; Enterovirus; Rhinovirus A
GCA_008796685.1	7134	1	Human rhinovirus A54	ATCC VR1164	Viruses; Riboviria; Orthornavirae; Pisuviricota; Pisoniviricetes; Picornavirales; Picornaviridae; Enterovirus; Rhinovirus A
GCA_004790055.1	7822	3	Leptomonas moramango leishbunyavirus	NA	Viruses; Riboviria; Orthornavirae; Negarnaviricota; Polyploviricotina; Ellioviricetes; Bunyavirales; Leishbuviridae; Shilevirus; Leptomonas shilevirus
GCA_001661815.1	2803	3	Torque teno mini virus 18	NA	Viruses; ssDNA viruses; Anelloviridae; Betatorquevirus; unclassified Betatorquevirus
GCA_008797955.1	7036	1	Human rhinovirus A55	NA	Viruses; Riboviria; Orthornavirae; Pisuviricota; Pisoniviricetes; Picornavirales; Picornaviridae; Enterovirus; Rhinovirus A
GCA_008796665.1	7136	1	Human rhinovirus A56	ATCC VR1166	Viruses; Riboviria; Orthornavirae; Pisuviricota; Pisoniviricetes; Picornavirales; Picornaviridae; Enterovirus; Rhinovirus A
GCA_001678415.1	5162	2	Sclerotinia nivalis victorivirus 1	NA	Viruses; dsRNA viruses; Totiviridae; Victorivirus; unclassified Victorivirus
GCA_008796645.1	7134	1	Human rhinovirus A57	NA	Viruses; Riboviria; Orthornavirae; Pisuviricota; Pisoniviricetes; Picornavirales; Picornaviridae; Enterovirus; Rhinovirus A
GCA_008798175.1	7135	1	Human rhinovirus A59	NA	Viruses; Riboviria; Orthornavirae; Pisuviricota; Pisoniviricetes; Picornavirales; Picornaviridae; Enterovirus; Rhinovirus A
GCA_008796605.1	7139	1	Human rhinovirus A60	ATCC VR1473	Viruses; Riboviria; Orthornavirae; Pisuviricota; Pisoniviricetes; Picornavirales; Picornaviridae; Enterovirus; Rhinovirus A
GCA_008796585.1	7139	1	Human rhinovirus A61	ATCC VR1171	Viruses; Riboviria; Orthornavirae; Pisuviricota; Pisoniviricetes; Picornavirales; Picornaviridae; Enterovirus; Rhinovirus A
GCA_008796545.1	7141	1	Human rhinovirus A63	ATCC VR1173	Viruses; Riboviria; Orthornavirae; Pisuviricota; Pisoniviricetes; Picornavirales; Picornaviridae; Enterovirus; Rhinovirus A
GCA_008797655.1	7129	1	Human rhinovirus A64	NA	Viruses; Riboviria; Orthornavirae; Pisuviricota; Pisoniviricetes; Picornavirales; Picornaviridae; Enterovirus; Rhinovirus A
GCA_008796505.1	7139	1	Human rhinovirus A66	ATCC VR1176	Viruses; Riboviria; Orthornavirae; Pisuviricota; Pisoniviricetes; Picornavirales; Picornaviridae; Enterovirus; Rhinovirus A
GCA_008796485.1	7135	1	Human rhinovirus A67	ATCC VR1177	Viruses; Riboviria; Orthornavirae; Pisuviricota; Pisoniviricetes; Picornavirales; Picornaviridae; Enterovirus; Rhinovirus A
GCA_008796465.1	7164	1	Human rhinovirus A68	ATCC VR1178	Viruses; Riboviria; Orthornavirae; Pisuviricota; Pisoniviricetes; Picornavirales; Picornaviridae; Enterovirus; Rhinovirus A
GCA_008798375.1	7223	1	Human rhinovirus B70	NA	Viruses; Riboviria; Orthornavirae; Pisuviricota; Pisoniviricetes; Picornavirales; Picornaviridae; Enterovirus; Rhinovirus B

gca	genome.size	proteome.size	phage.name	strain	Taxonomy
GCA_008796425.1	7161	1	Human rhinovirus A71	ATCC VR1181	Viruses; Riboviria; Orthornavirae; Pisuviricota; Pisoniviricetes; Picornavirales; Picornaviridae; Enterovirus; Rhinovirus A
GCA_008798315.1	7140	1	Human rhinovirus A73	NA	Viruses; Riboviria; Orthornavirae; Pisuviricota; Pisoniviricetes; Picornavirales; Picornaviridae; Enterovirus; Rhinovirus A
GCA_008798275.1	7120	1	Human rhinovirus A74	NA	Viruses; Riboviria; Orthornavirae; Pisuviricota; Pisoniviricetes; Picornavirales; Picornaviridae; Enterovirus; Rhinovirus A
GCA_008797975.1	7137	1	Human rhinovirus A75	NA	Viruses; Riboviria; Orthornavirae; Pisuviricota; Pisoniviricetes; Picornavirales; Picornaviridae; Enterovirus; Rhinovirus A
GCA_008798135.1	7129	1	Human rhinovirus A76	NA	Viruses; Riboviria; Orthornavirae; Pisuviricota; Pisoniviricetes; Picornavirales; Picornaviridae; Enterovirus; Rhinovirus A
GCA_008796385.1	7136	1	Human rhinovirus A77	ATCC VR1187	Viruses; Riboviria; Orthornavirae; Pisuviricota; Pisoniviricetes; Picornavirales; Picornaviridae; Enterovirus; Rhinovirus A
GCA_008796365.1	7224	1	Human rhinovirus B79	ATCC VR1189	Viruses; Riboviria; Orthornavirae; Pisuviricota; Pisoniviricetes; Picornavirales; Picornaviridae; Enterovirus; Rhinovirus B
GCA_008796345.1	7138	1	Human rhinovirus A80	ATCC VR1190	Viruses; Riboviria; Orthornavirae; Pisuviricota; Pisoniviricetes; Picornavirales; Picornaviridae; Enterovirus; Rhinovirus A
GCA_008796325.1	7116	1	Rhinovirus A81	ATCC VR1191	Viruses; Riboviria; Orthornavirae; Pisuviricota; Pisoniviricetes; Picornavirales; Picornaviridae; Enterovirus; Rhinovirus A
GCA_008796305.1	7230	1	Human rhinovirus B83	ATCC VR1193	Viruses; Riboviria; Orthornavirae; Pisuviricota; Pisoniviricetes; Picornavirales; Picornaviridae; Enterovirus; Rhinovirus B
GCA_008796265.1	7140	1	Human rhinovirus A85	ATCC VR1195	Viruses; Riboviria; Orthornavirae; Pisuviricota; Pisoniviricetes; Picornavirales; Picornaviridae; Enterovirus; Rhinovirus A
GCA_008796245.1	7213	1	Human rhinovirus B86	ATCC VR1196	Viruses; Riboviria; Orthornavirae; Pisuviricota; Pisoniviricetes; Picornavirales; Picornaviridae; Enterovirus; Rhinovirus B
GCA_008798095.1	7143	1	Human rhinovirus A88	NA	Viruses; Riboviria; Orthornavirae; Pisuviricota; Pisoniviricetes; Picornavirales; Picornaviridae; Enterovirus; Rhinovirus A
GCA_008796225.1	7124	1	Human rhinovirus A90	ATCC VR1291	Viruses; Riboviria; Orthornavirae; Pisuviricota; Pisoniviricetes; Picornavirales; Picornaviridae; Enterovirus; Rhinovirus A
GCA_008797615.1	7132	1	Human rhinovirus A94	NA	Viruses; Riboviria; Orthornavirae; Pisuviricota; Pisoniviricetes; Picornavirales; Picornaviridae; Enterovirus; Rhinovirus A
GCA_008796165.1	7134	1	Human rhinovirus A96	ATCC VR1296	Viruses; Riboviria; Orthornavirae; Pisuviricota; Pisoniviricetes; Picornavirales; Picornaviridae; Enterovirus; Rhinovirus A
GCA_008796125.1	7208	1	Human rhinovirus B99	ATCC VR1299	Viruses; Riboviria; Orthornavirae; Pisuviricota; Pisoniviricetes; Picornavirales; Picornaviridae; Enterovirus; Rhinovirus B
GCA_008796105.1	7140	1	Human rhinovirus A100	ATCC VR1300	Viruses; Riboviria; Orthornavirae; Pisuviricota; Pisoniviricetes; Picornavirales; Picornaviridae; Enterovirus; Rhinovirus A
GCA_002116175.1	350731	429	Kaumoebavirus	NA	Viruses; unclassified viruses
GCA_000869705.1	29096	12	Mal de Rio Cuarto virus	NA	Viruses; dsRNA viruses; Reoviridae; Spinareovirinae; Fijivirus; Fijivirus group 2
GCA_000867565.1	6485	4	Hibiscus latent Singapore virus	NA	Viruses; ssRNA viruses; ssRNA positive-strand viruses, no DNA stage; Virgaviridae; Tobamovirus
GCA_000839205.1	7768	13	Spiroplasma phage C74	SpV1C74	Viruses; Monodnaviria; Loebvirae; Hofneiviricota; Faserviricetes; Tubulavirales; Plectroviridae; Vespertiliovirus; Spiroplasma virus C74
GCA_002610685.1	36636	60	Streptococcus phage phiLP081102	NA	Viruses; Duplodnaviria; Heunggongvirae; Uroviricota; Caudoviricetes; Caudovirales; Siphoviridae; unclassified Siphoviridae
GCA_002610705.1	45451	72	Streptococcus phage phiJH13011	NA	Viruses; Duplodnaviria; Heunggongvirae; Uroviricota; Caudoviricetes; Caudovirales; Siphoviridae; unclassified Siphoviridae
GCA_002610725.1	14113	24	Streptococcus phage phiJH13013	NA	Viruses; unclassified bacterial viruses
GCA_002610745.1	11295	19	Streptococcus phage phiZJ200911012	NA	Viruses; unclassified bacterial viruses
GCA_002610765.1	22861	26	Streptococcus phage phiZJ200911013	NA	Viruses; Duplodnaviria; Heunggongvirae; Uroviricota; Caudoviricetes; Caudovirales; Siphoviridae; unclassified Siphoviridae
GCA_002610785.1	41057	64	Streptococcus phage phiZJ200911014	NA	Viruses; Duplodnaviria; Heunggongvirae; Uroviricota; Caudoviricetes; Caudovirales; Siphoviridae; unclassified Siphoviridae
GCA_002610805.1	67195	76	Streptococcus phage phiJH13012	NA	Viruses; Duplodnaviria; Heunggongvirae; Uroviricota; Caudoviricetes; Caudovirales; Siphoviridae; unclassified Siphoviridae
GCA_002610825.1	33253	46	Streptococcus phage phiZJ200911011	NA	Viruses; Duplodnaviria; Heunggongvirae; Uroviricota; Caudoviricetes; Caudovirales; Siphoviridae; unclassified Siphoviridae
GCA_000842945.1	44970	49	Staphylococcus virus phi12	NA	Viruses; Duplodnaviria; Heunggongvirae; Uroviricota; Caudoviricetes; Caudovirales; Siphoviridae; Triavirus
GCA_000842085.1	42722	49	Staphylococcus virus 13	NA	Viruses; Duplodnaviria; Heunggongvirae; Uroviricota; Caudoviricetes; Caudovirales; Siphoviridae; Biseptimavirus
GCA_000849505.1	5698	2	Euprosterna elaeasa virus	NA	Viruses; ssRNA viruses; ssRNA positive-strand viruses, no DNA stage; Permutotetraviridae; Alphapermutotetravirus
GCA_018580635.1	2409	3	Feline anellovirus	NA	Viruses; Anelloviridae; Tettorquevirus; Torque teno felid virus 6

gca	genome.size	proteome.size	phage.name	strain	Taxonomy
GCA_001678235.1	9369	1	Wild onion symptomless virus	NA	Viruses; ssRNA viruses; ssRNA positive-strand viruses, no DNA stage; Potyviridae; unclassified Potyviridae
GCA_002607225.1	279593	368	Pseudomonas phage KTN4	NA	Viruses; Duplodnaviria; Heunggongvirae; Uroviricota; Caudoviricetes; Caudovirales; Myoviridae; Phikzvirus; Pseudomonas virus phiKZ
GCA_001679855.1	2264	4	Gemycircularvirus HVGcV1	NA	Viruses; unclassified viruses; Genomoviridae; Gemycircularvirus; unclassified Gemycircularvirus
GCA_001679875.1	2262	3	Gemycircularvirus HVGcV2	NA	Viruses; unclassified viruses; Genomoviridae; Gemycircularvirus; unclassified Gemycircularvirus
GCA_001744035.1	22825	40	Lactococcus phage D4410	NA	Viruses; dsDNA viruses, no RNA stage; Caudovirales; Siphoviridae; C2virus; unclassified C2likevirus
GCA_001745355.1	22884	40	Lactococcus phage D4412	NA	Viruses; dsDNA viruses, no RNA stage; Caudovirales; Siphoviridae; C2virus; unclassified C2likevirus
GCA_001746015.1	22240	38	Lactococcus phage M5938	NA	Viruses; dsDNA viruses, no RNA stage; Caudovirales; Siphoviridae; C2virus; unclassified C2likevirus
GCA_001744695.1	21697	38	Lactococcus phage M6162	NA	Viruses; dsDNA viruses, no RNA stage; Caudovirales; Siphoviridae; C2virus; unclassified C2likevirus
GCA_001744015.1	22179	37	Lactococcus phage M6165	NA	Viruses; dsDNA viruses, no RNA stage; Caudovirales; Siphoviridae; C2virus; unclassified C2likevirus
GCA_002757735.1	22251	38	Lactococcus phage M6202	NA	Viruses; Duplodnaviria; Heunggongvirae; Uroviricota; Caudoviricetes; Caudovirales; Siphoviridae; Ceduovirus; unclassified Ceduovirus
GCA_002757755.1	22157	38	Lactococcus phage M6653	NA	Viruses; Duplodnaviria; Heunggongvirae; Uroviricota; Caudoviricetes; Caudovirales; Siphoviridae; Ceduovirus; unclassified Ceduovirus
GCA_002757775.1	22350	39	Lactococcus phage M6654	NA	Viruses; Duplodnaviria; Heunggongvirae; Uroviricota; Caudoviricetes; Caudovirales; Siphoviridae; Ceduovirus; unclassified Ceduovirus
GCA_001678455.1	23958	12	Etheostoma fonticola aquareovirus	NA	Viruses; dsRNA viruses; Reoviridae; Spinareovirinae; Aquareovirus; unclassified Aquareovirus
GCA_001745335.1	166634	265	Shigella phage SHBML501	NA	Viruses; dsDNA viruses, no RNA stage; Caudovirales; Myoviridae; Tevenvirinae; T4virus; unclassified T4virus
GCA_001743555.1	170650	269	Shigella phage SHFML11	NA	Viruses; dsDNA viruses, no RNA stage; Caudovirales; Myoviridae; Tevenvirinae; T4virus; unclassified T4virus
GCA_001745995.1	168993	269	Shigella phage SHFML26	NA	Viruses; dsDNA viruses, no RNA stage; Caudovirales; Myoviridae; Tevenvirinae; T4virus; unclassified T4virus
GCA_001744675.1	108050	139	Shigella phage SHSML45	NA	Viruses; dsDNA viruses, no RNA stage; Caudovirales; Myoviridae; Tevenvirinae; T4virus; unclassified T4virus
GCA_001746195.1	169621	269	Shigella phage SHSML521	NA	Viruses; dsDNA viruses, no RNA stage; Caudovirales; Myoviridae; Tevenvirinae; T4virus; unclassified T4virus
GCA_002828445.1	9828	1	Vanilla mosaic virus	NA	Viruses; Riboviria; Orthornavirae; Pisuviricota; Stelpaviricetes; Patatavirales; Potyviridae; Potyvirus; Dasheen mosaic virus
GCA_001678295.1	5224	2	Ungulate bocaparvovirus 6	Ungulate bocaparvovirus 6 strain USII03	Viruses; ssDNA viruses; Parvoviridae; Parvovirinae; Bocaparvovirus; unclassified Bocaparvovirus
GCA_001744875.1	41918	61	Enterobacter phage Arya	NA	Viruses; dsDNA viruses, no RNA stage; Caudovirales; Myoviridae; unclassified Myoviridae
GCA_001744195.1	41760	56	Enterobacter phage Tyrion	NA	Viruses; dsDNA viruses, no RNA stage; Caudovirales; Podoviridae; unclassified Podoviridae
GCA_000854085.1	18891	8	Reston ebolavirus	Pennsylvania	Viruses; ssRNA viruses; ssRNA negative-strand viruses; Mononegavirales; Filoviridae; Ebolavirus
GCA_000855585.1	18875	8	Sudan ebolavirus	Gulu	Viruses; ssRNA viruses; ssRNA negative-strand viruses; Mononegavirales; Filoviridae; Ebolavirus
GCA_000888475.1	18935	9	Tai Forest ebolavirus	NA	Viruses; ssRNA viruses; ssRNA negative-strand viruses; Mononegavirales; Filoviridae; Ebolavirus
GCA_018443575.1	248052	334	uncultured marine virus	NA	Viruses; environmental samples
GCA_003033315.1	4456	5	Cutavirus	BR337	Viruses; Monodnaviria; Shotokuvirae; Cossaviricota; Quintoviricetes; Piccovirales; Parvoviridae; Parvovirinae; Protoparvovirus; Primate protoparvovirus 3
GCA_000852365.1	2728	1	Cryphonectria parasitica mitovirus 1NB631	NA	Viruses; ssRNA viruses; ssRNA positive-strand viruses, no DNA stage; Narnaviridae; Mitovirus; unclassified Mitovirus
GCA_000851605.1	2514	1	Saccharomyces 20S RNA narnavirus	374C	Viruses; ssRNA viruses; ssRNA positive-strand viruses, no DNA stage; Narnaviridae; Narnavirus
GCA_000874705.1	4852	3	Ourmia melon virus	NA	Viruses; ssRNA viruses; ssRNA positive-strand viruses, no DNA stage; Ourmiavirus
GCA_002158555.1	12312	3	Wolkberg virus	NA	Viruses; unclassified viruses
GCA_008297395.1	150381	150	Goatpox virus	NA	Viruses; Varidnaviria; Bamfordvirae; Nucleocytoviricota; Pokkesviricetes; Chitovirales; Poxviridae; Chordopoxvirinae; Capripoxvirus
GCA_000841065.1	1305	1	OkLCV satDNA 10	NA	Viruses; Tolecusatellitidae; Betasatellite; unclassified Betasatellite; Okra leaf curl betasatellite
GCA_001885505.1	157855	202	Salmonella phage GG32	NA	Viruses; dsDNA viruses, no RNA stage; Caudovirales; Myoviridae; unclassified Myoviridae

gca	genome.size	proteome.size	phage.name	strain	Taxonomy
GCA_001885525.1	41224	59	Salmonella phage MA12	NA	Viruses; dsDNA viruses, no RNA stage; Caudovirales; Siphoviridae; unclassified Siphoviridae
GCA_002819465.1	1862	2	Bat associated circovirus 1	NA	Viruses; Monodnaviria; Shotokuvirae; Cressdnaviricota; Arfiviricetes; Cirlivirales; Circoviridae; Circovirus
GCA_000908355.1	1798	2	Bat associated circovirus 2	NA	Viruses; Monodnaviria; Shotokuvirae; Cressdnaviricota; Arfiviricetes; Cirlivirales; Circoviridae; Circovirus
GCA_004029355.1	2000	2	Porcine circovirus 3	NA	Viruses; ssDNA viruses; Circoviridae; Circovirus; unclassified Circovirus
GCA_001693545.1	6400	2	Yacon virus A	NA	Viruses; ssRNA viruses; ssRNA positive-strand viruses, no DNA stage; Tymovirales; Betaflexiviridae; Trivirinae; Capillovirus; unclassified Capillovirus
GCA_002610845.1	49822	75	Clostridium phage CDKM9	NA	Viruses; Duplodnaviria; Heunggongvirae; Uroviricota; Caudoviricetes; Caudovirales; Myoviridae; Lubbockvirus; unclassified Lubbockvirus
GCA_002610875.1	50605	79	Clostridium phage CDKM15	NA	Viruses; Duplodnaviria; Heunggongvirae; Uroviricota; Caudoviricetes; Caudovirales; Myoviridae; Lubbockvirus; unclassified Lubbockvirus
GCA_001745515.1	43277	67	Pseudomonas phage MD8	NA	Viruses; dsDNA viruses, no RNA stage; Caudovirales; Siphoviridae; unclassified Siphoviridae
GCA_002610895.2	60973	86	Pseudomonas phage AN14	NA	Viruses; Duplodnaviria; Heunggongvirae; Uroviricota; Caudoviricetes; Caudovirales; Siphoviridae; Yuavirus; unclassified Yuavirus
GCA_002610955.1	43223	67	Bacillus phage PfEFR4	NA	Viruses; Duplodnaviria; Heunggongvirae; Uroviricota; Caudoviricetes; Caudovirales; Siphoviridae; unclassified Siphoviridae
GCA_001746175.1	43773	68	Bacillus phage PfEFR5	NA	Viruses; dsDNA viruses, no RNA stage; Caudovirales; Siphoviridae; unclassified Siphoviridae
GCA_002610985.1	48709	81	Bacillus phage PfIS075	NA	Viruses; Duplodnaviria; Heunggongvirae; Uroviricota; Caudoviricetes; Caudovirales; Siphoviridae; Cecivirus; unclassified Cecivirus
GCA_002611025.1	48055	80	Bacillus phage PfNC7401	NA	Viruses; Duplodnaviria; Heunggongvirae; Uroviricota; Caudoviricetes; Caudovirales; Siphoviridae; Cecivirus; unclassified Cecivirus
GCA_001685345.1	27709	1	Diaphorina citri flavilike virus	NA	Viruses; unclassified viruses
GCA_001744855.1	158081	209	Salmonella phage vB_SalM_PM10	NA	Viruses; dsDNA viruses, no RNA stage; Caudovirales; Myoviridae; unclassified Myoviridae
GCA_002237255.1	2749	4	Sugarcane striate virus	NA	Viruses; ssDNA viruses; Geminiviridae; Mastrevirus; unclassified Mastrevirus
GCA_003502615.1	37950	66	uncultured Mediterranean phage	NA	Viruses; environmental samples
GCA_004874145.1	35595	53	uncultured Mediterranean phage uvDeepCGR0AD1C123	NA	Viruses; Duplodnaviria; Heunggongvirae; Uroviricota; Caudoviricetes; Caudovirales; Podoviridae; environmental samples
GCA_004874005.1	33969	41	uncultured Mediterranean phage uvDeepCGR0AD1C239	NA	Viruses; Duplodnaviria; Heunggongvirae; Uroviricota; Caudoviricetes; Caudovirales; Siphoviridae; environmental samples
GCA_004873885.1	39367	59	uncultured Mediterranean phage uvDeepCGR0KM14C182	NA	Viruses; Duplodnaviria; Heunggongvirae; Uroviricota; Caudoviricetes; Caudovirales; Siphoviridae; environmental samples
GCA_004873985.1	30127	44	uncultured Mediterranean phage uvDeepCGR0KM15C219	NA	Viruses; Duplodnaviria; Heunggongvirae; Uroviricota; Caudoviricetes; Caudovirales; Siphoviridae; environmental samples
GCA_004874065.1	29831	40	uncultured Mediterranean phage uvDeepCGR0KM22C158	NA	Viruses; Duplodnaviria; Heunggongvirae; Uroviricota; Caudoviricetes; Caudovirales; Siphoviridae; environmental samples
GCA_004874185.1	30611	48	uncultured Mediterranean phage uvDeepCGR1KM17C101	NA	Viruses; Duplodnaviria; Heunggongvirae; Uroviricota; Caudoviricetes; Caudovirales; environmental samples
GCA_004874305.1	36798	71	uncultured Mediterranean phage uvDeepCGR2AD10C281	NA	Viruses; Duplodnaviria; Heunggongvirae; Uroviricota; Caudoviricetes; Caudovirales; Podoviridae; environmental samples
GCA_004874325.1	37105	39	uncultured Mediterranean phage uvDeepCGR2AD12C183	NA	Viruses; Duplodnaviria; Heunggongvirae; Uroviricota; Caudoviricetes; Caudovirales; environmental samples
GCA_004875165.1	38060	46	uncultured Mediterranean phage uvDeepCGR2AD3C191	NA	Viruses; Duplodnaviria; Heunggongvirae; Uroviricota; Caudoviricetes; Caudovirales; Podoviridae; environmental samples
GCA_004875135.1	32219	42	uncultured Mediterranean phage uvDeepCGR2AD3C76	NA	Viruses; Duplodnaviria; Heunggongvirae; Uroviricota; Caudoviricetes; Caudovirales; environmental samples
GCA_004875205.1	38021	43	uncultured Mediterranean phage uvDeepCGR2AD7C12	NA	Viruses; Duplodnaviria; Heunggongvirae; Uroviricota; Caudoviricetes; Caudovirales; environmental samples
GCA_004875305.1	32823	62	uncultured Mediterranean phage uvDeepCGR2AD8C175	NA	Viruses; Duplodnaviria; Heunggongvirae; Uroviricota; Caudoviricetes; Caudovirales; Siphoviridae; environmental samples
GCA_004874465.1	36016	52	uncultured Mediterranean phage uvDeepCGR2KM18C269	NA	Viruses; Duplodnaviria; Heunggongvirae; Uroviricota; Caudoviricetes; Caudovirales; environmental samples
GCA_004874405.1	33382	46	uncultured Mediterranean phage uvDeepCGR2KM18C74	NA	Viruses; Duplodnaviria; Heunggongvirae; Uroviricota; Caudoviricetes; Caudovirales; Siphoviridae; environmental samples
GCA_004874545.1	38902	50	uncultured Mediterranean phage uvDeepCGR2KM19C184	NA	Viruses; Duplodnaviria; Heunggongvirae; Uroviricota; Caudoviricetes; Caudovirales; environmental samples
GCA_004874625.1	36469	54	uncultured Mediterranean phage uvDeepCGR2KM19C269	NA	Viruses; Duplodnaviria; Heunggongvirae; Uroviricota; Caudoviricetes; Caudovirales; environmental samples
GCA_004874505.1	36892	44	uncultured Mediterranean phage uvDeepCGR2KM19C37	NA	Viruses; Duplodnaviria; Heunggongvirae; Uroviricota; Caudoviricetes; Caudovirales; environmental samples

gca	genome.size	proteome.size	phage.name	strain	Taxonomy
GCA_004874665.1	34924	37	uncultured Mediterranean phage uvDeepCGR2KM20C133	NA	Viruses; Duplodnaviria; Heunggongvirae; Uroviricota; Caudoviricetes; Caudovirales; environmental samples
GCA_004874795.1	34149	44	uncultured Mediterranean phage uvDeepCGR2KM21C338	NA	Viruses; Duplodnaviria; Heunggongvirae; Uroviricota; Caudoviricetes; Caudovirales; environmental samples
GCA_004874825.1	31562	56	uncultured Mediterranean phage uvDeepCGR2KM21C345	NA	Viruses; Duplodnaviria; Heunggongvirae; Uroviricota; Caudoviricetes; Caudovirales; environmental samples
GCA_004875325.1	36122	59	uncultured Mediterranean phage uvDeepCGR2KM21C368	NA	Viruses; Duplodnaviria; Heunggongvirae; Uroviricota; Caudoviricetes; Caudovirales; Podoviridae; environmental samples
GCA_004874745.1	35974	33	uncultured Mediterranean phage uvDeepCGR2KM21C88	NA	Viruses; Duplodnaviria; Heunggongvirae; Uroviricota; Caudoviricetes; Caudovirales; environmental samples
GCA_004874925.1	40440	82	uncultured Mediterranean phage uvDeepCGR2KM22C255	NA	Viruses; Duplodnaviria; Heunggongvirae; Uroviricota; Caudoviricetes; Caudovirales; Siphoviridae; environmental samples
GCA_004875415.1	32249	42	uncultured Mediterranean phage uvDeepCGR2KM23C198	NA	Viruses; Duplodnaviria; Heunggongvirae; Uroviricota; Caudoviricetes; Caudovirales; Siphoviridae; environmental samples
GCA_004874945.1	37208	46	uncultured Mediterranean phage uvDeepCGR2KM23C246	NA	Viruses; Duplodnaviria; Heunggongvirae; Uroviricota; Caudoviricetes; Caudovirales; environmental samples
GCA_004875045.1	34310	62	uncultured Mediterranean phage uvDeepCGR2KM24C165	NA	Viruses; Duplodnaviria; Heunggongvirae; Uroviricota; Caudoviricetes; Caudovirales; Siphoviridae; environmental samples
GCA_004875005.1	41270	43	uncultured Mediterranean phage uvDeepCGR2KM24C26	NA	Viruses; Duplodnaviria; Heunggongvirae; Uroviricota; Caudoviricetes; Caudovirales; environmental samples
GCA_002611045.1	37385	59	Bacillus phage BMBtpLA3	NA	Viruses; Duplodnaviria; Heunggongvirae; Uroviricota; Caudoviricetes; Caudovirales; Siphoviridae; Lwoffvirus; unclassified Lwoffvirus
GCA_002611065.1	26471	30	Bacillus phage vB_BtS_BMBtp13	NA	Viruses; Duplodnaviria; Heunggongvirae; Uroviricota; Caudoviricetes; Caudovirales; Siphoviridae; unclassified Siphoviridae
GCA_002611085.1	50740	75	Bacillus phage vB_BtS_BMBtp14	NA	Viruses; Duplodnaviria; Heunggongvirae; Uroviricota; Caudoviricetes; Caudovirales; Siphoviridae; unclassified Siphoviridae
GCA_002611105.1	34986	47	Bacillus phage vB_BtS_BMBtp15	NA	Viruses; Duplodnaviria; Heunggongvirae; Uroviricota; Caudoviricetes; Caudovirales; Siphoviridae; unclassified Siphoviridae
GCA_002611125.1	66922	90	Pseudomonas phage vB_Pae436M8	NA	Viruses; Duplodnaviria; Heunggongvirae; Uroviricota; Caudoviricetes; Caudovirales; Myoviridae; Pbunavirus; unclassified Pbunavirus
GCA_002611145.1	72728	92	Pseudomonas phage vB_Pae575P3	NA	Viruses; Duplodnaviria; Heunggongvirae; Uroviricota; Caudoviricetes; Caudovirales; Podoviridae; Litunavirus; unclassified Litunavirus
GCA_002611165.1	72508	91	Pseudomonas phage vB_Pae1396P5	NA	Viruses; Duplodnaviria; Heunggongvirae; Uroviricota; Caudoviricetes; Caudovirales; Podoviridae; Litunavirus; unclassified Litunavirus
GCA_002611185.1	158986	204	Salmonella phage vB_SenM2	NA	Viruses; Duplodnaviria; Heunggongvirae; Uroviricota; Caudoviricetes; Caudovirales; Ackermannviridae; Cvivirinae; Kuttervirus; unclassified Kuttervirus
GCA_002611205.1	139681	203	Staphylococcus phage vB_SscM1	NA	Viruses; Duplodnaviria; Heunggongvirae; Uroviricota; Caudoviricetes; Caudovirales; Herelleviridae; Twortvirinae; Sciuriunavirus; Staphylococcus virus SscM1
GCA_002611225.1	139682	202	Staphylococcus phage vB_SscM2	NA	Viruses; Duplodnaviria; Heunggongvirae; Uroviricota; Caudoviricetes; Caudovirales; Herelleviridae; Twortvirinae; Sciuriunavirus; unclassified Sciuriunavirus
GCA_002611245.1	34067	60	Lactococcus phage 38502	NA	Viruses; Duplodnaviria; Heunggongvirae; Uroviricota; Caudoviricetes; Caudovirales; Siphoviridae; unclassified Siphoviridae
GCA_002611265.1	35800	60	Lactococcus phage 49801	NA	Viruses; Duplodnaviria; Heunggongvirae; Uroviricota; Caudoviricetes; Caudovirales; Siphoviridae; unclassified Siphoviridae
GCA_002611285.1	30745	47	Lactococcus phage 50902	NA	Viruses; Duplodnaviria; Heunggongvirae; Uroviricota; Caudoviricetes; Caudovirales; Siphoviridae; unclassified Siphoviridae
GCA_002611305.1	36378	53	Lactococcus phage 53801	NA	Viruses; Duplodnaviria; Heunggongvirae; Uroviricota; Caudoviricetes; Caudovirales; Siphoviridae; unclassified Siphoviridae
GCA_002611325.1	36445	58	Lactococcus phage 53802	NA	Viruses; Duplodnaviria; Heunggongvirae; Uroviricota; Caudoviricetes; Caudovirales; Siphoviridae; unclassified Siphoviridae
GCA_002611345.1	30238	49	Lactococcus phage 58502	NA	Viruses; Duplodnaviria; Heunggongvirae; Uroviricota; Caudoviricetes; Caudovirales; Siphoviridae; unclassified Siphoviridae
GCA_002611365.1	30616	52	Lactococcus phage 62502	NA	Viruses; Duplodnaviria; Heunggongvirae; Uroviricota; Caudoviricetes; Caudovirales; Siphoviridae; unclassified Siphoviridae
GCA_002611385.1	30556	50	Lactococcus phage 62503	NA	Viruses; Duplodnaviria; Heunggongvirae; Uroviricota; Caudoviricetes; Caudovirales; Siphoviridae; unclassified Siphoviridae
GCA_002611405.1	34635	55	Lactococcus phage 98101	NA	Viruses; Duplodnaviria; Heunggongvirae; Uroviricota; Caudoviricetes; Caudovirales; Siphoviridae; unclassified Siphoviridae
GCA_002611425.1	34421	53	Lactococcus phage 98102	NA	Viruses; Duplodnaviria; Heunggongvirae; Uroviricota; Caudoviricetes; Caudovirales; Siphoviridae; unclassified Siphoviridae
GCA_002611445.1	35904	58	Lactococcus phage 98103	NA	Viruses; Duplodnaviria; Heunggongvirae; Uroviricota; Caudoviricetes; Caudovirales; Siphoviridae; unclassified Siphoviridae
GCA_002611465.1	34989	55	Lactococcus phage 98104	NA	Viruses; Duplodnaviria; Heunggongvirae; Uroviricota; Caudoviricetes; Caudovirales; Siphoviridae; unclassified Siphoviridae
GCA_002611485.1	35636	53	Lactococcus phage 98202	NA	Viruses; Duplodnaviria; Heunggongvirae; Uroviricota; Caudoviricetes; Caudovirales; Siphoviridae; unclassified Siphoviridae
GCA_002611505.1	35867	52	Lactococcus phage 98203	NA	Viruses; Duplodnaviria; Heunggongvirae; Uroviricota; Caudoviricetes; Caudovirales; Siphoviridae; unclassified Siphoviridae

gca	genome.size	proteome.size	phage.name	strain	Taxonomy
GCA_002611525.1	38334	56	Lactococcus phage 98204	NA	Viruses; Duplodnaviria; Heunggongvirae; Uroviricota; Caudoviricetes; Caudovirales; Siphoviridae; unclassified Siphoviridae
GCA_002611545.1	31446	52	Lactococcus phage C41431	NA	Viruses; Duplodnaviria; Heunggongvirae; Uroviricota; Caudoviricetes; Caudovirales; Siphoviridae; unclassified Siphoviridae
GCA_002611565.1	35188	53	Lactococcus phage Dub35A	NA	Viruses; Duplodnaviria; Heunggongvirae; Uroviricota; Caudoviricetes; Caudovirales; Siphoviridae; unclassified Siphoviridae
GCA_004129775.1	7624	2	Culex tritaeniorhynchus totivirus	NA	Viruses; Riboviria; Orthornavirae; Duplornaviricota; Chrymotiviricetes; Ghabrivirales; Totiviridae; unclassified Totiviridae
GCA_003085835.1	7653	1	Poecivirus BCCH449	NA	Viruses; Riboviria; Orthornavirae; Pisuviricota; Pisoniviricetes; Picornavirales; Picornaviridae; Poecivirus; Poecivirus A
GCA_001689835.1	10913	1	Nylanderia fulva virus 1	NA	Viruses; unclassified viruses
GCA_001744555.1	180219	273	Citrobacter phage vB_CfrM_CfP1	NA	Viruses; dsDNA viruses, no RNA stage; Caudovirales; Myoviridae; Tevenvirinae; T4virus; unclassified T4virus
GCA_002611585.1	45260	53	Phage MedPESWcelC56	NA	Viruses; Duplodnaviria; Heunggongvirae; Uroviricota; Caudoviricetes; Caudovirales; Autographiviridae; Kafavirus; Kawavirus SWcelC56
GCA_001695445.1	10644	1	Rhizoctonia solani flexivirus 1	NA	Viruses; ssRNA viruses; ssRNA positive-strand viruses, no DNA stage; Tymovirales; unclassified Flexiviridae
GCA_001743875.1	34396	51	Lactococcus phage 28201	NA	Viruses; dsDNA viruses, no RNA stage; Caudovirales; Siphoviridae; unclassified Siphoviridae
GCA_001745195.1	31987	51	Lactococcus phage 50101	NA	Viruses; dsDNA viruses, no RNA stage; Caudovirales; Siphoviridae; unclassified Siphoviridae
GCA_002757795.1	33440	52	Lactococcus phage 50901	NA	Viruses; Duplodnaviria; Heunggongvirae; Uroviricota; Caudoviricetes; Caudovirales; Siphoviridae; unclassified Siphoviridae
GCA_002611665.1	34117	51	Lactococcus phage 56701	NA	Viruses; Duplodnaviria; Heunggongvirae; Uroviricota; Caudoviricetes; Caudovirales; Siphoviridae; unclassified Siphoviridae
GCA_002757815.1	34084	51	Lactococcus phage 62501	NA	Viruses; Duplodnaviria; Heunggongvirae; Uroviricota; Caudoviricetes; Caudovirales; Siphoviridae; unclassified Siphoviridae
GCA_001745855.1	32409	48	Lactococcus phage 63301	NA	Viruses; dsDNA viruses, no RNA stage; Caudovirales; Siphoviridae; unclassified Siphoviridae
GCA_002611625.1	33035	52	Lactococcus phage 86501	NA	Viruses; Duplodnaviria; Heunggongvirae; Uroviricota; Caudoviricetes; Caudovirales; Siphoviridae; unclassified Siphoviridae
GCA_001744535.1	36202	52	Lactococcus phage 98201	NA	Viruses; dsDNA viruses, no RNA stage; Caudovirales; Siphoviridae; unclassified Siphoviridae
GCA_002757835.1	41901	63	Mycobacterium phage BruceB	NA	Viruses; Duplodnaviria; Heunggongvirae; Uroviricota; Caudoviricetes; Caudovirales; Siphoviridae; Liefievirus; unclassified Liefievirus
GCA_002219845.1	3387	2	Antonospora locustae virus 1	NA	Viruses; dsRNA viruses; Amalgaviridae; unclassified Amalgaviridae
GCA_001695505.1	8246	5	Fusarium graminearum deltaflexivirus 1	NA	Viruses; ssRNA viruses; ssRNA positive-strand viruses, no DNA stage; Tymovirales; unclassified Tymovirales
GCA_001461305.1	2903	1	Botrytis ourmialike virus	NA	Viruses; Riboviria; Orthornavirae; Lenarviricota; Miaviricetes; Ourlivirales; Botourmiaviridae; Botoulivirus; Botrytis botoulivirus
GCA_004874245.1	33081	56	uncultured Mediterranean phage uvDeep1CGR2KM23C896	NA	Viruses; Duplodnaviria; Heunggongvirae; Uroviricota; Caudoviricetes; Caudovirales; environmental samples
GCA_002611685.1	36278	40	Bacillus phage DeepPurple	NA	Viruses; Duplodnaviria; Heunggongvirae; Uroviricota; Caudoviricetes; Caudovirales; Siphoviridae; unclassified Siphoviridae
GCA_002597825.1	51370	88	Mycobacterium phage Wilbur	NA	Viruses; Duplodnaviria; Heunggongvirae; Uroviricota; Caudoviricetes; Caudovirales; Siphoviridae; Fromanvirus; Mycobacterium virus Peaches
GCA_001743855.1	38773	59	Rhodovulum phage vB_RhkS_P1	NA	Viruses; dsDNA viruses, no RNA stage; Caudovirales; Siphoviridae; unclassified Siphoviridae
GCA_001695485.1	10908	1	Atypical porcine pestivirus 1	NA	Viruses; ssRNA viruses; ssRNA positive-strand viruses, no DNA stage; Flaviviridae; Pestivirus; unclassified Pestivirus; Atypical porcine pestivirus
GCA_001745175.1	76012	126	Mycobacterium phage Gardann	NA	Viruses; dsDNA viruses, no RNA stage; Caudovirales; Siphoviridae; unclassified Siphoviridae
GCA_002599905.1	50866	91	Mycobacterium phage Aglet	NA	Viruses; Duplodnaviria; Heunggongvirae; Uroviricota; Caudoviricetes; Caudovirales; Siphoviridae; Fromanvirus; unclassified Fromanvirus
GCA_001695465.1	133492	143	Pteropox virus	Australia	Viruses; dsDNA viruses, no RNA stage; Poxviridae; Chordopoxvirinae; unclassified Chordopoxvirinae
GCA_001745835.1	50082	75	Streptomyces phage Nanodon	NA	Viruses; dsDNA viruses, no RNA stage; Caudovirales; Siphoviridae; unclassified Siphoviridae
GCA_002611705.1	44559	52	Escherichia virus AAPEc6	NA	Viruses; Duplodnaviria; Heunggongvirae; Uroviricota; Caudoviricetes; Caudovirales; Autographiviridae; Molineuxvirinae; Vectrevirus
GCA_001744515.1	42429	64	Mycobacterium phage Sneeze	NA	Viruses; dsDNA viruses, no RNA stage; Caudovirales; Siphoviridae; unclassified Siphoviridae
GCA_002630065.1	155800	236	Mycobacterium phage Littleton	NA	Viruses; Duplodnaviria; Heunggongvirae; Uroviricota; Caudoviricetes; Caudovirales; Myoviridae; Bixzunavirus; Mycobacterium virus Bxz1
GCA_001743995.1	52496	93	Mycobacterium phage Makemake	NA	Viruses; dsDNA viruses, no RNA stage; Caudovirales; Siphoviridae; unclassified Siphoviridae

gca	genome.size	proteome.size	phage.name	strain	Taxonomy
GCA_002604345.1	69000	101	Mycobacterium phage PhatCats2014	NA	Viruses; Duplodnaviria; Heunggongvirae; Uroviricota; Caudoviricetes; Caudovirales; Siphoviridae; Bclasvirinae; Pegunavirus; Mycobacterium virus Pg1
GCA_001745315.1	52501	94	Mycobacterium phage Papez	NA	Viruses; dsDNA viruses, no RNA stage; Caudovirales; Siphoviridae; unclassified Siphoviridae
GCA_002757855.1	50897	83	Mycobacterium phage BuzzBuzz	NA	Viruses; Duplodnaviria; Heunggongvirae; Uroviricota; Caudoviricetes; Caudovirales; Siphoviridae; Fromanvirus; Mycobacterium virus Bxz2
GCA_002611725.1	158127	235	Bacillus phage PK16	NA	Viruses; Duplodnaviria; Heunggongvirae; Uroviricota; Caudoviricetes; Caudovirales; Herelleviridae; Bastillevirinae; Caeruleovirus; unclassified Caeruleovirus
GCA_001745975.1	53623	91	Mycobacterium phage Gompeii16	NA	Viruses; dsDNA viruses, no RNA stage; Caudovirales; Siphoviridae; unclassified Siphoviridae
GCA_002757875.1	70096	111	Arthrobacter phage Conboy	NA	Viruses; Duplodnaviria; Heunggongvirae; Uroviricota; Caudoviricetes; Caudovirales; Myoviridae; Radnorvirus; Arthrobacter virus Trina
GCA_002611745.1	53622	91	Mycobacterium phage Bircsak	NA	Viruses; Duplodnaviria; Heunggongvirae; Uroviricota; Caudoviricetes; Caudovirales; Siphoviridae; Fromanvirus; unclassified Fromanvirus
GCA_002611765.1	40823	57	Vibrio phage 2E1	NA	Viruses; Duplodnaviria; Heunggongvirae; Uroviricota; Caudoviricetes; Caudovirales; Siphoviridae; unclassified Siphoviridae
GCA_002611785.1	129756	176	Vibrio phage S47	NA	Viruses; Duplodnaviria; Heunggongvirae; Uroviricota; Caudoviricetes; Caudovirales; Myoviridae; unclassified Myoviridae
GCA_001744655.1	40008	46	Pseudomonas phage Andromeda	NA	Viruses; dsDNA viruses, no RNA stage; Caudovirales; Podoviridae; Autographivirinae; Phikmvvirus; unclassified phiKMV-like phages
GCA_002604925.1	50884	90	Mycobacterium phage Penny1	NA	Viruses; Duplodnaviria; Heunggongvirae; Uroviricota; Caudoviricetes; Caudovirales; Siphoviridae; Fromanvirus; unclassified Fromanvirus
GCA_002611805.1	181570	284	Cronobacter phage vB_CsaM_leE	NA	Viruses; Duplodnaviria; Heunggongvirae; Uroviricota; Caudoviricetes; Caudovirales; Myoviridae; Tevenvirinae; unclassified Tevenvirinae
GCA_001743975.2	44268	57	Pectobacterium phage PP16	NA	Viruses; dsDNA viruses, no RNA stage; Caudovirales; Podoviridae; unclassified Podoviridae
GCA_001745295.1	44570	56	Pectobacterium phage PP90	NA	Viruses; dsDNA viruses, no RNA stage; Caudovirales; Podoviridae; unclassified Podoviridae
GCA_001882315.1	47564	94	Salmonella phage IME207	NA	Viruses; dsDNA viruses, no RNA stage; Caudovirales; Siphoviridae; unclassified Siphoviridae
GCA_002604065.1	52044	96	Mycobacterium phage ToneTone	NA	Viruses; Duplodnaviria; Heunggongvirae; Uroviricota; Caudoviricetes; Caudovirales; Siphoviridae; Fromanvirus; unclassified Fromanvirus
GCA_001745015.1	87662	133	Escherichia phage vB_EcoM_Alf5	NA	Viruses; dsDNA viruses, no RNA stage; Caudovirales; Myoviridae; unclassified Myoviridae
GCA_002757895.1	39215	48	Pseudomonas phage UNOSLW1	NA	Viruses; Duplodnaviria; Heunggongvirae; Uroviricota; Caudoviricetes; Caudovirales; Autographiviridae; Studiervirinae; Pifdecavirus; Pseudomonas virus UNOSLW1
GCA_002611825.1	51067	77	Streptomyces phage Brataylor	NA	Viruses; Duplodnaviria; Heunggongvirae; Uroviricota; Caudoviricetes; Caudovirales; Siphoviridae; Arquatrovirinae; Likavirus; unclassified Likavirus
GCA_002611845.1	50701	76	Streptomyces phage Godpower	NA	Viruses; Duplodnaviria; Heunggongvirae; Uroviricota; Caudoviricetes; Caudovirales; Siphoviridae; Arquatrovirinae; Likavirus; unclassified Likavirus
GCA_002611865.1	50558	75	Streptomyces phage Lorelei	NA	Viruses; Duplodnaviria; Heunggongvirae; Uroviricota; Caudoviricetes; Caudovirales; Siphoviridae; Arquatrovirinae; Likavirus; unclassified Likavirus
GCA_002954805.1	47331	86	Aeromonas phage Ahp2	NA	Viruses; Duplodnaviria; Heunggongvirae; Uroviricota; Caudoviricetes; Caudovirales; Myoviridae; unclassified Myoviridae
GCA_001745675.1	162151	304	Bacillus phage Kida	NA	Viruses; dsDNA viruses, no RNA stage; Caudovirales; Myoviridae; unclassified Myoviridae; Bacillus phage NotTheCreek
GCA_001744355.1	162415	302	Bacillus phage DirtyBetty	NA	Viruses; dsDNA viruses, no RNA stage; Caudovirales; Myoviridae; unclassified Myoviridae
GCA_001743675.1	25908	40	Bacillus phage Aurora	NA	Viruses; dsDNA viruses, no RNA stage; Caudovirales; Podoviridae; unclassified Podoviridae
GCA_001744995.1	26504	46	Bacillus phage Claudi	NA	Viruses; dsDNA viruses, no RNA stage; Caudovirales; Podoviridae; unclassified Podoviridae
GCA_001745655.1	24320	36	Bacillus phage Stitch	NA	Viruses; dsDNA viruses, no RNA stage; Caudovirales; Podoviridae; unclassified Podoviridae
GCA_002598605.1	15556	26	Arthrobacter phage Courtney3	NA	Viruses; Duplodnaviria; Heunggongvirae; Uroviricota; Caudoviricetes; Caudovirales; Siphoviridae; Decurrovirus; unclassified Decurrovirus
GCA_002757915.1	66507	90	Mycobacterium phage Laurie	NA	Viruses; Duplodnaviria; Heunggongvirae; Uroviricota; Caudoviricetes; Caudovirales; Siphoviridae; Bclasvirinae; Rosebushvirus; Mycobacterium virus Godines
GCA_001678215.2	4998	3	Maizeassociated totivirus 2	NA	Viruses; Riboviria; Orthornavirae; Duplornaviricota; Chrymotiviricetes; Ghabrivirales; Totiviridae; unclassified Totiviridae
GCA_900093805.1	17470	5	Clostridium phage HM2	NA	Viruses; Duplodnaviria; Heunggongvirae; Uroviricota; Caudoviricetes; Caudovirales; Podoviridae; unclassified Podoviridae
GCA_002757935.1	39167	48	Pseudomonas phage UNOSLW2	NA	Viruses; Duplodnaviria; Heunggongvirae; Uroviricota; Caudoviricetes; Caudovirales; Autographiviridae; Studiervirinae; Pifdecavirus; unclassified Pifdecavirus
GCA_002757975.1	39136	47	Pseudomonas phage UNOSLW4	NA	Viruses; Duplodnaviria; Heunggongvirae; Uroviricota; Caudoviricetes; Caudovirales; Autographiviridae; Studiervirinae; Pifdecavirus; unclassified Pifdecavirus
GCA_001882195.1	39949	65	Pseudoalteromonas phage BS5	NA	Viruses; unclassified phages

gca	genome.size	proteome.size	phage.name	strain	Taxonomy
GCA_001882135.1	42685	54	Pseudoalteromonas phage PH1	NA	Viruses; unclassified phages
GCA_003861935.1	31866	61	Lactococcus phage 10W24	NA	Viruses; Duplodnaviria; Heunggongvirae; Uroviricota; Caudoviricetes; Caudovirales; Siphoviridae; Skunavirus; unclassified Skunavirus
GCA_003861955.1	29964	52	Lactococcus phage 11W16L	NA	Viruses; Duplodnaviria; Heunggongvirae; Uroviricota; Caudoviricetes; Caudovirales; Siphoviridae; Skunavirus; unclassified Skunavirus
GCA_003862015.1	32477	56	Lactococcus phage 16W23	NA	Viruses; Duplodnaviria; Heunggongvirae; Uroviricota; Caudoviricetes; Caudovirales; Siphoviridae; Skunavirus; unclassified Skunavirus
GCA_003861975.1	30984	60	Lactococcus phage 17W11	NA	Viruses; Duplodnaviria; Heunggongvirae; Uroviricota; Caudoviricetes; Caudovirales; Siphoviridae; Skunavirus; unclassified Skunavirus
GCA_003861995.1	33017	59	Lactococcus phage 17W12M	NA	Viruses; Duplodnaviria; Heunggongvirae; Uroviricota; Caudoviricetes; Caudovirales; Siphoviridae; Skunavirus; unclassified Skunavirus
GCA_003861875.1	31627	58	Lactococcus phage 2R06A	NA	Viruses; Duplodnaviria; Heunggongvirae; Uroviricota; Caudoviricetes; Caudovirales; Siphoviridae; Skunavirus; unclassified Skunavirus
GCA_003861795.1	30165	58	Lactococcus phage 2R14S	NA	Viruses; Duplodnaviria; Heunggongvirae; Uroviricota; Caudoviricetes; Caudovirales; Siphoviridae; Skunavirus; unclassified Skunavirus
GCA_003861815.1	31632	60	Lactococcus phage 2R15M	NA	Viruses; Duplodnaviria; Heunggongvirae; Uroviricota; Caudoviricetes; Caudovirales; Siphoviridae; Skunavirus; unclassified Skunavirus
GCA_003861835.1	32505	59	Lactococcus phage 2R15S	NA	Viruses; Duplodnaviria; Heunggongvirae; Uroviricota; Caudoviricetes; Caudovirales; Siphoviridae; Skunavirus; unclassified Skunavirus
GCA_003861855.1	31468	61	Lactococcus phage 2R15S2	NA	Viruses; Duplodnaviria; Heunggongvirae; Uroviricota; Caudoviricetes; Caudovirales; Siphoviridae; Skunavirus; unclassified Skunavirus
GCA_003861775.1	31192	60	Lactococcus phage 3R07S	NA	Viruses; Duplodnaviria; Heunggongvirae; Uroviricota; Caudoviricetes; Caudovirales; Siphoviridae; Skunavirus; unclassified Skunavirus
GCA_003861915.1	32793	59	Lactococcus phage 6W06	NA	Viruses; Duplodnaviria; Heunggongvirae; Uroviricota; Caudoviricetes; Caudovirales; Siphoviridae; Skunavirus; unclassified Skunavirus
GCA_003862075.1	30918	58	Lactococcus phage 6W18L	NA	Viruses; Duplodnaviria; Heunggongvirae; Uroviricota; Caudoviricetes; Caudovirales; Siphoviridae; Skunavirus; unclassified Skunavirus
GCA_003861895.1	31034	55	Lactococcus phage 8R06S	NA	Viruses; Duplodnaviria; Heunggongvirae; Uroviricota; Caudoviricetes; Caudovirales; Siphoviridae; Skunavirus; unclassified Skunavirus
GCA_003862035.1	31877	56	Lactococcus phage MW18S	NA	Viruses; Duplodnaviria; Heunggongvirae; Uroviricota; Caudoviricetes; Caudovirales; Siphoviridae; Skunavirus; unclassified Skunavirus
GCA_002954815.1	32393	59	Lactococcus phage 10W18	NA	Viruses; Duplodnaviria; Heunggongvirae; Uroviricota; Caudoviricetes; Caudovirales; Siphoviridae; Skunavirus; unclassified Skunavirus
GCA_002954825.1	30934	59	Lactococcus phage 10W22S	NA	Viruses; Duplodnaviria; Heunggongvirae; Uroviricota; Caudoviricetes; Caudovirales; Siphoviridae; Skunavirus; unclassified Skunavirus
GCA_002954835.1	29952	53	Lactococcus phage 13W11L	NA	Viruses; Duplodnaviria; Heunggongvirae; Uroviricota; Caudoviricetes; Caudovirales; Siphoviridae; Skunavirus; unclassified Skunavirus
GCA_002954845.1	32782	57	Lactococcus phage 16W12L	NA	Viruses; Duplodnaviria; Heunggongvirae; Uroviricota; Caudoviricetes; Caudovirales; Siphoviridae; Skunavirus; unclassified Skunavirus
GCA_002954855.1	30446	57	Lactococcus phage 3R16S	NA	Viruses; Duplodnaviria; Heunggongvirae; Uroviricota; Caudoviricetes; Caudovirales; Siphoviridae; Skunavirus; unclassified Skunavirus
GCA_002954865.1	30782	57	Lactococcus phage 4R15L	NA	Viruses; Duplodnaviria; Heunggongvirae; Uroviricota; Caudoviricetes; Caudovirales; Siphoviridae; Skunavirus; unclassified Skunavirus
GCA_002954875.1	30913	58	Lactococcus phage 4R16L2	NA	Viruses; Duplodnaviria; Heunggongvirae; Uroviricota; Caudoviricetes; Caudovirales; Siphoviridae; Skunavirus; unclassified Skunavirus
GCA_002954885.1	33013	57	Lactococcus phage MW18L	NA	Viruses; Duplodnaviria; Heunggongvirae; Uroviricota; Caudoviricetes; Caudovirales; Siphoviridae; Skunavirus; unclassified Skunavirus
GCA_002954895.1	29513	52	Lactococcus phage i0139	NA	Viruses; Duplodnaviria; Heunggongvirae; Uroviricota; Caudoviricetes; Caudovirales; Siphoviridae; Skunavirus; unclassified Skunavirus
GCA_002954905.1	30890	52	Lactococcus phage 19W07F	NA	Viruses; Duplodnaviria; Heunggongvirae; Uroviricota; Caudoviricetes; Caudovirales; Siphoviridae; Skunavirus; unclassified Skunavirus
GCA_900094095.1	348043	588	Escherichia phage vB_Eco_slurp01	NA	Viruses; Duplodnaviria; Heunggongvirae; Uroviricota; Caudoviricetes; Caudovirales; Myoviridae; Asteriusvirus; unclassified Asteriusvirus
GCA_018583025.1	7506	3	Dioscorea bacilliform RT virus	NA	Viruses; Riboviria; Pararnavirae; Artverviricota; Revtraviricetes; Ortervirales; Caulimoviridae; Badnavirus; unclassified Badnavirus; Dioscorea bacilliform virus
GCA_001698395.1	2759	6	Ramie mosaic Yunnan virus	NA	Viruses; ssDNA viruses; Geminiviridae; Begomovirus; unclassified Begomovirus
GCA_000842005.1	1360	1	Ageratum yellow vein alphasatellite	NA	Viruses; Satellites; Satellite Nucleic Acids; Single stranded DNA satellites; Alphasatellites; Begomovirus-associated alphasatellites
GCA_002989875.1	11648	17	Haloarcula hispanica pleomorphic virus 3	NA	Viruses; Monodnaviria; Trapavirae; Saleviricota; Huolimaviricetes; Haloruvirales; Pleolipoviridae; Betapleolipovirus; unclassified Betapleolipovirus
GCA_002611905.1	81509	125	Vibrio phage 1	NA	Viruses; Duplodnaviria; Heunggongvirae; Uroviricota; Caudoviricetes; Caudovirales; Siphoviridae; unclassified Siphoviridae
GCA_000863445.1	9878	1	Wild potato mosaic virus	NA	Viruses; ssRNA viruses; ssRNA positive-strand viruses, no DNA stage; Potyviridae; Potyvirus
GCA_000861725.1	9899	1	Peru tomato mosaic virus	NA	Viruses; ssRNA viruses; ssRNA positive-strand viruses, no DNA stage; Potyviridae; Potyvirus

gca	genome.size	proteome.size	phage.name	strain	Taxonomy
GCA_002219465.1	127941	120	Seal parapoxvirus	NA	Viruses; dsDNA viruses, no RNA stage; Poxviridae; Chordopoxvirinae; Parapoxvirus; unclassified Parapoxvirus
GCA_002611925.1	234670	297	Tenacibaculum phage pT24	NA	Viruses; Duplodnaviria; Heunggongvirae; Uroviricota; Caudoviricetes; Caudovirales; Myoviridae; unclassified Myoviridae
GCA_900095325.1	175206	276	Klebsiella phage PMBT1	NA	Viruses; Duplodnaviria; Heunggongvirae; Uroviricota; Caudoviricetes; Caudovirales; Myoviridae; Tevenvirinae; Slopekvirus; Klebsiella virus PMBT1
GCA_001707025.1	6775	5	Senna mosaic virus	NA	Viruses; ssRNA viruses; ssRNA positive-strand viruses, no DNA stage; Tymovirales; Alphaflexiviridae; Potexvirus; unclassified Potexvirus
GCA_002611945.1	149813	281	Escherichia phage ESCO13	NA	Viruses; Duplodnaviria; Heunggongvirae; Uroviricota; Caudoviricetes; Caudovirales; Myoviridae; Phapecoctavirus
GCA_002611965.1	51178	88	Mycobacterium phage LittleGuy	NA	Viruses; Duplodnaviria; Heunggongvirae; Uroviricota; Caudoviricetes; Caudovirales; Siphoviridae; Fromanvirus; unclassified Fromanvirus
GCA_002611985.1	51274	85	Mycobacterium phage Badger	NA	Viruses; Duplodnaviria; Heunggongvirae; Uroviricota; Caudoviricetes; Caudovirales; Siphoviridae; Fromanvirus; unclassified Fromanvirus
GCA_002612005.1	51743	92	Mycobacterium phage Magnito	NA	Viruses; Duplodnaviria; Heunggongvirae; Uroviricota; Caudoviricetes; Caudovirales; Siphoviridae; Fromanvirus; unclassified Fromanvirus
GCA_000854785.1	8363	4	Spring beauty latent virus	KU1	Viruses; ssRNA viruses; ssRNA positive-strand viruses, no DNA stage; Bromoviridae; Bromovirus
GCA_003029325.1	2767	6	Velvet bean golden mosaic virus	NA	Viruses; Monodnaviria; Shotokuvirae; Cressdnaviricota; Repensiviricetes; Geplafuvirales; Geminiviridae; Begomovirus
GCA_002374355.1	8884	6	Loveridges garter snake virus 1	251327	Viruses; Riboviria; Orthornavirae; Negarnaviricota; Haplovirícotina; Monjiviricetes; Mononegavirales; Bornaviridae; Orthobornavirus; Elapid 1 orthobornavirus
GCA_002646355.1	18578	11	Rotavirus A RVAHumanwtTHAKKL172013G8P8	NA	Viruses; Riboviria; Orthornavirae; Duplornaviricota; Resentoviricetes; Reovirales; Reoviridae; Sedoreovirinae; Rotavirus; Rotavirus A; Rotavirus G8
GCA_002646575.1	18594	11	Rotavirus A RVAHumanwtTHAPCB792013G8P8	NA	Viruses; Riboviria; Orthornavirae; Duplornaviricota; Resentoviricetes; Reovirales; Reoviridae; Sedoreovirinae; Rotavirus; Rotavirus A; Rotavirus G8
GCA_002646605.1	18589	11	Rotavirus A RVAHumanwtTHAPCB842013G8P8	NA	Viruses; Riboviria; Orthornavirae; Duplornaviricota; Resentoviricetes; Reovirales; Reoviridae; Sedoreovirinae; Rotavirus; Rotavirus A; Rotavirus G8
GCA_002646655.1	18571	11	Rotavirus A RVAHumanwtTHAPCB852013G8P8	NA	Viruses; Riboviria; Orthornavirae; Duplornaviricota; Resentoviricetes; Reovirales; Reoviridae; Sedoreovirinae; Rotavirus; Rotavirus A; Rotavirus G8
GCA_002646495.1	18573	11	Rotavirus A RVAHumanwtTHAPCB1032013G8P8	NA	Viruses; Riboviria; Orthornavirae; Duplornaviricota; Resentoviricetes; Reovirales; Reoviridae; Sedoreovirinae; Rotavirus; Rotavirus A; Rotavirus G8
GCA_002646675.1	18569	11	Rotavirus A RVAHumanwtTHASKT1072013G8P8	NA	Viruses; Riboviria; Orthornavirae; Duplornaviricota; Resentoviricetes; Reovirales; Reoviridae; Sedoreovirinae; Rotavirus; Rotavirus A; Rotavirus G8
GCA_002646935.1	18583	11	Rotavirus A RVAHumanwtTHASWL122013G8P8	NA	Viruses; Riboviria; Orthornavirae; Duplornaviricota; Resentoviricetes; Reovirales; Reoviridae; Sedoreovirinae; Rotavirus; Rotavirus A; Rotavirus G8
GCA_002646455.1	18508	11	Rotavirus A RVAHumanwtTHANP1302014G8P8	NA	Viruses; Riboviria; Orthornavirae; Duplornaviricota; Resentoviricetes; Reovirales; Reoviridae; Sedoreovirinae; Rotavirus; Rotavirus A; Rotavirus G8
GCA_002646555.1	18548	11	Rotavirus A RVAHumanwtTHAPCB6562014G8P8	NA	Viruses; Riboviria; Orthornavirae; Duplornaviricota; Resentoviricetes; Reovirales; Reoviridae; Sedoreovirinae; Rotavirus; Rotavirus A; Rotavirus G8
GCA_002646795.1	18424	11	Rotavirus A RVAHumanwtTHASKT4572014G8P8	NA	Viruses; Riboviria; Orthornavirae; Duplornaviricota; Resentoviricetes; Reovirales; Reoviridae; Sedoreovirinae; Rotavirus; Rotavirus A; Rotavirus G8
GCA_002646875.1	18498	11	Rotavirus A RVAHumanwtTHASSKT2692014G8P8	NA	Viruses; Riboviria; Orthornavirae; Duplornaviricota; Resentoviricetes; Reovirales; Reoviridae; Sedoreovirinae; Rotavirus; Rotavirus A; Rotavirus G8
GCA_002646915.1	18556	11	Rotavirus A RVAHumanwtTHASSL552014G8P8	NA	Viruses; Riboviria; Orthornavirae; Duplornaviricota; Resentoviricetes; Reovirales; Reoviridae; Sedoreovirinae; Rotavirus; Rotavirus A; Rotavirus G8
GCA_002646415.1	18495	11	Rotavirus A RVAHumanwtTHALS2022014G2P4	NA	Viruses; Riboviria; Orthornavirae; Duplornaviricota; Resentoviricetes; Reovirales; Reoviridae; Sedoreovirinae; Rotavirus; Rotavirus A; Rotavirus G2
GCA_002646435.1	18517	11	Rotavirus A RVAHumanwtTHALSL72014G2P4	NA	Viruses; Riboviria; Orthornavirae; Duplornaviricota; Resentoviricetes; Reovirales; Reoviridae; Sedoreovirinae; Rotavirus; Rotavirus A; Rotavirus G2
GCA_001717395.1	4639	3	Sea otter parvovirus 1	NA	Viruses; ssDNA viruses; Parvoviridae; unclassified Parvoviridae
GCA_002612025.1	40765	49	Klebsiella phage vB_KpnP_KpV763	NA	Viruses; Duplodnaviria; Heunggongvirae; Uroviricota; Caudoviricetes; Caudovirales; Autographiviridae; Studiervirinae; Przondovirus; Klebsiella virus KpV763
GCA_002612045.1	70679	94	Mycobacterium phage Fortunato	NA	Viruses; Duplodnaviria; Heunggongvirae; Uroviricota; Caudoviricetes; Caudovirales; Siphoviridae; Bclasvirinae; Coopervirus; unclassified Coopervirus
GCA_002612065.1	41901	63	Mycobacterium phage Jane	NA	Viruses; Duplodnaviria; Heunggongvirae; Uroviricota; Caudoviricetes; Caudovirales; Siphoviridae; Liefievirus; unclassified Liefievirus
GCA_004130615.1	3991	6	Adonis mosaic virus	NA	Viruses; Riboviria; Orthornavirae; Kitrinoviricota; Tolucaviricetes; Tolivirales; Tombusviridae; unclassified Tombusviridae
GCA_001745495.1	45206	62	Escherichia phage Envy	NA	Viruses; dsDNA viruses, no RNA stage; Caudovirales; Siphoviridae; unclassified Siphoviridae
GCA_001746155.1	44513	61	Escherichia phage Gluttony	NA	Viruses; dsDNA viruses, no RNA stage; Caudovirales; Siphoviridae; unclassified Siphoviridae
GCA_002757995.1	41942	57	Escherichia phage Lust	NA	Viruses; Duplodnaviria; Heunggongvirae; Uroviricota; Caudoviricetes; Caudovirales; Siphoviridae; Dhillonvirus; unclassified Dhillonvirus
GCA_002758015.1	44899	63	Escherichia phage Pride	NA	Viruses; Duplodnaviria; Heunggongvirae; Uroviricota; Caudoviricetes; Caudovirales; Siphoviridae; Dhillonvirus; unclassified Dhillonvirus

gca	genome.size	proteome.size	phage.name	strain	Taxonomy
GCA_000859025.1	2746	6	Ageratum enation virus	NA	Viruses; ssDNA viruses; Geminiviridae; Begomovirus
GCA_001881915.1	172345	214	Synechococcus phage SCAM22	NA	Viruses; unclassified phages
GCA_001882155.1	198190	240	Synechococcus phage SCAM3	NA	Viruses; unclassified phages
GCA_002366065.1	191983	238	Synechococcus phage SCAM4	NA	Viruses; Duplodnaviria; Heunggongvirae; Uroviricota; Caudoviricetes; Caudovirales; Myoviridae; unclassified Myoviridae
GCA_013426285.1	221362	267	Synechococcus phage SCAM7	NA	Viruses; unclassified phages
GCA_001881955.1	174830	229	Synechococcus phage SCAM9	NA	Viruses; unclassified phages
GCA_001744335.2	246290	277	Erwinia phage vB_EamM_Asesino	NA	Viruses; dsDNA viruses, no RNA stage; Caudovirales; Myoviridae; unclassified Myoviridae
GCA_001743655.1	244840	277	Erwinia phage vB_EamM_ChrisDB	NA	Viruses; dsDNA viruses, no RNA stage; Caudovirales; Myoviridae; unclassified Myoviridae
GCA_001744975.1	223935	241	Erwinia phage vB_EamM_EarlPhillipIV	NA	Viruses; dsDNA viruses, no RNA stage; Caudovirales; Myoviridae; unclassified Myoviridae
GCA_001745635.1	240761	271	Erwinia phage vB_EamM_Huxley	NA	Viruses; dsDNA viruses, no RNA stage; Caudovirales; Myoviridae; unclassified Myoviridae
GCA_001744315.1	246390	285	Erwinia phage vB_EamM_Kwan	NA	Viruses; dsDNA viruses, no RNA stage; Caudovirales; Myoviridae; unclassified Myoviridae
GCA_002758035.1	241654	272	Erwinia phage Machina	NA	Viruses; Duplodnaviria; Heunggongvirae; Uroviricota; Caudoviricetes; Caudovirales; Myoviridae; Machinavirus; Erwinia virus Machina
GCA_002758055.1	241050	271	Erwinia phage vB_EamM_Parshik	NA	Viruses; Duplodnaviria; Heunggongvirae; Uroviricota; Caudoviricetes; Caudovirales; Myoviridae; Machinavirus; unclassified Machinavirus
GCA_001743635.1	229501	247	Erwinia phage vB_EamM_Phobos	NA	Viruses; dsDNA viruses, no RNA stage; Caudovirales; Myoviridae; unclassified Myoviridae
GCA_002600675.1	243953	276	Erwinia phage vB_EamM_Stratton	NA	Viruses; Duplodnaviria; Heunggongvirae; Uroviricota; Caudoviricetes; Caudovirales; Myoviridae; Erskinevirus; unclassified Erskinevirus
GCA_001744955.1	241147	271	Erwinia phage vB_EamM_Caitlin	NA	Viruses; unclassified phages
GCA_000840805.1	45503	72	Pseudomonas virus PaP3	NA	Viruses; Duplodnaviria; Heunggongvirae; Uroviricota; Caudoviricetes; Caudovirales; Podoviridae; Bruynoghevirus
GCA_001706965.1	3935	5	Gompholobium virus A	NA	Viruses; ssRNA viruses; ssRNA positive-strand viruses, no DNA stage; Tombusviridae; unclassified Tombusviridae
GCA_013087355.2	5164	6	Croton golden mosaic virus	NA	Viruses; Monodnaviria; Shotokuvirae; Cressdnaviricota; Repensiviricetes; Geplafuvirales; Geminiviridae; Begomovirus
GCA_018580615.1	2592	4	Hybanthus yellow mosaic virus	NA	Viruses; Monodnaviria; Shotokuvirae; Cressdnaviricota; Repensiviricetes; Geplafuvirales; Geminiviridae; Begomovirus
GCA_018580625.1	2626	4	Verbena mottle virus	NA	Viruses; Monodnaviria; Shotokuvirae; Cressdnaviricota; Repensiviricetes; Geplafuvirales; Geminiviridae; Begomovirus
GCA_001725915.1	8970	6	Parrot bornavirus 5	NA	Viruses; Riboviria; Orthornavirae; Negarnaviricota; Haploviricotina; Monjiviricetes; Mononegavirales; Bornaviridae; Orthobornavirus; Psittaciform 2 orthobornavirus
GCA_001589995.1	8904	6	Aquatic bird bornavirus 2	NA	Viruses; Riboviria; Orthornavirae; Negarnaviricota; Haploviricotina; Monjiviricetes; Mononegavirales; Bornaviridae; Orthobornavirus; Waterbird 1 orthobornavirus
GCA_001706945.1	2742	7	Senna leaf curl virus	NA	Viruses; ssDNA viruses; Geminiviridae; Begomovirus; unclassified Begomovirus
GCA_000845485.1	8300	7	Felis domesticus papillomavirus type 1	NA	Viruses; dsDNA viruses, no RNA stage; Papillomaviridae; Lambdapapillomavirus; Lambdapapillomavirus 1
GCA_002612105.1	177907	280	Cronobacter phage vB_CsaM_leB	NA	Viruses; Duplodnaviria; Heunggongvirae; Uroviricota; Caudoviricetes; Caudovirales; Myoviridae; Tevenvirinae; unclassified Tevenvirinae
GCA_002612125.1	179516	286	Cronobacter phage vB_CsaM_leN	NA	Viruses; Duplodnaviria; Heunggongvirae; Uroviricota; Caudoviricetes; Caudovirales; Myoviridae; Tevenvirinae; unclassified Tevenvirinae
GCA_001698295.1	8798	6	Variegated squirrel bornavirus 1	NA	Viruses; Riboviria; Orthornavirae; Negarnaviricota; Haploviricotina; Monjiviricetes; Mononegavirales; Bornaviridae; Orthobornavirus; Mammalian 2 orthobornavirus
GCA_000848705.1	15600	10	Human parainfluenza virus 1 strain Washington1964	Washington 1964	Viruses; Riboviria; Orthornavirae; Negarnaviricota; Haploviricotina; Monjiviricetes; Mononegavirales; Paramyxoviridae; Orthoparamyxovirinae; Respirovirus; Human respirovirus 1
GCA_002406395.1	4676	5	Pomona leafnosed bat associated polyomavirus	NA	Viruses; Monodnaviria; Shotokuvirae; Cossaviricota; Papovaviricetes; Sepolyvirales; Polyomaviridae; unclassified Polyomaviridae
GCA_013087365.1	4222	2	Rat parvovirus 2	NA	Viruses; Monodnaviria; Shotokuvirae; Cossaviricota; Quintoviricetes; Piccovirales; Parvoviridae; Hamaparvovirinae; Chaphamaparvovirus; Rodent chaphamaparvovirus 2
GCA_001717195.1	7299	5	Sparus aurata polyomavirus 1	NA	Viruses; dsDNA viruses, no RNA stage; Polyomaviridae; unclassified Polyomaviridae
GCA_001717215.1	5748	9	Sparus aurata papillomavirus 1	NA	Viruses; dsDNA viruses, no RNA stage; Papillomaviridae; unclassified Papillomaviridae; non-mammal papillomaviruses
GCA_001714495.1	9859	2	Callistephus mottle virus	NA	Viruses; ssRNA viruses; ssRNA positive-strand viruses, no DNA stage; Potyviridae; Potyvirus; unclassified Potyvirus

gca	genome.size	proteome.size	phage.name	strain	Taxonomy
GCA_001714395.1	7961	8	Bos taurus papillomavirus 13	14RO12	Viruses; dsDNA viruses, no RNA stage; Papillomaviridae; unclassified Papillomaviridae; non-primate mammal papillomaviruses
GCA_001714535.1	7527	7	Bos taurus papillomavirus 16	NA	Viruses; dsDNA viruses, no RNA stage; Papillomaviridae; unclassified Papillomaviridae; non-primate mammal papillomaviruses
GCA_001714435.1	7402	6	Bos taurus papillomavirus 17	NA	Viruses; dsDNA viruses, no RNA stage; Papillomaviridae; unclassified Papillomaviridae; non-primate mammal papillomaviruses
GCA_001714475.1	7282	6	Bos taurus papillomavirus 18	NA	Viruses; dsDNA viruses, no RNA stage; Papillomaviridae; unclassified Papillomaviridae; non-primate mammal papillomaviruses
GCA_001714375.1	7253	7	Bos taurus papillomavirus 19	NA	Viruses; dsDNA viruses, no RNA stage; Papillomaviridae; unclassified Papillomaviridae; non-primate mammal papillomaviruses
GCA_001714515.1	7277	7	Bos taurus papillomavirus 20	NA	Viruses; dsDNA viruses, no RNA stage; Papillomaviridae; unclassified Papillomaviridae; non-primate mammal papillomaviruses
GCA_001714415.1	7149	6	Bos taurus papillomavirus 21	NA	Viruses; dsDNA viruses, no RNA stage; Papillomaviridae; unclassified Papillomaviridae; non-primate mammal papillomaviruses
GCA_001717415.1	115510	99	Ranavirus maximus	NA	Viruses; Varidnaviria; Bamfordvirae; Nucleocytoviricota; Megaviricetes; Pimascovirales; Iridoviridae; Alphairidovirinae; Ranavirus; Frog virus 3
GCA_006449475.1	108041	109	Pike perch iridovirus	NA	Viruses; Varidnaviria; Bamfordvirae; Nucleocytoviricota; Megaviricetes; Pimascovirales; Iridoviridae; Alphairidovirinae; Ranavirus; Frog virus 3
GCA_006449495.1	114865	98	Cod iridovirus	NA	Viruses; dsDNA viruses, no RNA stage; Iridoviridae; unclassified Iridoviridae
GCA_000843725.1	241087	169	Panine betaherpesvirus 2	Heberling	Viruses; dsDNA viruses, no RNA stage; Herpesvirales; Herpesviridae; Betaherpesvirinae; Cytomegalovirus
GCA_001745615.1	92580	168	Gordonia phage Bantam	NA	Viruses; dsDNA viruses, no RNA stage; Caudovirales; Siphoviridae; unclassified Siphoviridae
GCA_002609605.1	53432	83	Gordonia phage BatStarr	NA	Viruses; Duplodnaviria; Heunggongvirae; Uroviricota; Caudoviricetes; Caudovirales; Siphoviridae; Nymbaxtervirinae; Nymphadoravirus; unclassified Nymphadoravirus
GCA_001744295.1	47898	79	Gordonia phage CaptainKirk2	NA	Viruses; dsDNA viruses, no RNA stage; Caudovirales; Siphoviridae; unclassified Siphoviridae
GCA_001743615.1	54167	80	Gordonia phage CarolAnn	NA	Viruses; dsDNA viruses, no RNA stage; Caudovirales; Siphoviridae; unclassified Siphoviridae
GCA_001744935.1	93686	178	Gordonia phage Cucurbita	NA	Viruses; dsDNA viruses, no RNA stage; Caudovirales; Siphoviridae; unclassified Siphoviridae
GCA_001745595.1	44929	74	Gordonia phage Eyre	NA	Viruses; dsDNA viruses, no RNA stage; Caudovirales; Siphoviridae; unclassified Siphoviridae
GCA_001744275.1	45285	59	Gordonia phage Ghobes	NA	Viruses; dsDNA viruses, no RNA stage; Caudovirales; Siphoviridae; unclassified Siphoviridae
GCA_001743595.1	44536	70	Gordonia phage Hedwig	NA	Viruses; dsDNA viruses, no RNA stage; Caudovirales; Siphoviridae; unclassified Siphoviridae
GCA_001744915.1	52726	101	Gordonia phage JSwag	NA	Viruses; dsDNA viruses, no RNA stage; Caudovirales; Siphoviridae; unclassified Siphoviridae
GCA_001745575.1	78302	102	Gordonia phage Jumbo	NA	Viruses; dsDNA viruses, no RNA stage; Caudovirales; Siphoviridae; unclassified Siphoviridae
GCA_001744255.1	41857	60	Gordonia phage Nyceirae	NA	Viruses; dsDNA viruses, no RNA stage; Caudovirales; Siphoviridae; unclassified Siphoviridae
GCA_001743575.1	52738	98	Gordonia phage Remus	NA	Viruses; dsDNA viruses, no RNA stage; Caudovirales; Siphoviridae; unclassified Siphoviridae
GCA_002612145.1	52738	98	Gordonia phage Strosahl	NA	Viruses; Duplodnaviria; Heunggongvirae; Uroviricota; Caudoviricetes; Caudovirales; Siphoviridae; Soupsvirus; Gordonia virus Strosahl
GCA_001744815.1	66611	97	Gordonia phage Terapin	NA	Viruses; dsDNA viruses, no RNA stage; Caudovirales; Siphoviridae; unclassified Siphoviridae
GCA_001744135.1	57804	93	Gordonia phage Twister6	NA	Viruses; dsDNA viruses, no RNA stage; Caudovirales; Siphoviridae; unclassified Siphoviridae
GCA_001745455.1	52077	79	Gordonia phage Zirinka	NA	Viruses; dsDNA viruses, no RNA stage; Caudovirales; Siphoviridae; unclassified Siphoviridae
GCA_002612165.1	69165	95	Rhodococcus phage ChewyVIII	NA	Viruses; Duplodnaviria; Heunggongvirae; Uroviricota; Caudoviricetes; Caudovirales; Siphoviridae; unclassified Siphoviridae
GCA_001717255.1	12162	4	Wabat virus	KH1	Viruses; unclassified viruses
GCA_001717315.1	8946	1	Washington bat picornavirus	UW1	Viruses; ssRNA viruses; ssRNA positive-strand viruses, no DNA stage; Picornavirales; Picornaviridae; unclassified Picornaviridae
GCA_001717375.1	6434	2	Bat tymolike virus	UW1	Viruses; ssRNA viruses; ssRNA positive-strand viruses, no DNA stage; Tymovirales; unclassified Tymovirales
GCA_001717435.1	4388	3	Duwamo virus	UW1	Viruses; unclassified viruses
GCA_001717235.1	4268	2	Egaro virus	UW1	Viruses; unclassified viruses
GCA_000919835.1	12273	1	Pronghorn antelope pestivirus	NA	Viruses; ssRNA viruses; ssRNA positive-strand viruses, no DNA stage; Flaviviridae; Pestivirus; unclassified Pestivirus

gca	genome.size	proteome.size	phage.name	strain	Taxonomy
GCA_001717335.1	9778	1	Coccinia mottle virus	NA	Viruses; ssRNA viruses; ssRNA positive-strand viruses, no DNA stage; Potyviridae; Ipomovirus; unclassified Ipomovirus
GCA_001746115.1	160985	258	Mycobacterium phage Tonenili	NA	Viruses; dsDNA viruses, no RNA stage; Caudovirales; Myoviridae; unclassified Myoviridae
GCA_001722805.1	3873	2	Sophora japonica powdery mildewassociated partitivirus	NA	Viruses; dsRNA viruses; Partitiviridae; unclassified Partitiviridae
GCA_000903895.1	5372	5	Cardioderma cor polyomavirus 1	NA	Viruses; Monodnaviria; Shotokuvirae; Cossaviricota; Papovaviricetes; Sepolyvirales; Polyomaviridae; Alphapolyomavirus
GCA_000903695.1	5157	4	Chlorocebus pygerythrus polyomavirus 1	NA	Viruses; Monodnaviria; Shotokuvirae; Cossaviricota; Papovaviricetes; Sepolyvirales; Polyomaviridae; Alphapolyomavirus
GCA_000928075.1	5055	5	Chlorocebus pygerythrus polyomavirus 3	NA	Viruses; Monodnaviria; Shotokuvirae; Cossaviricota; Papovaviricetes; Sepolyvirales; Polyomaviridae; Alphapolyomavirus
GCA_000903035.1	5294	5	Eidolon helvum polyomavirus 1	NA	Viruses; Monodnaviria; Shotokuvirae; Cossaviricota; Papovaviricetes; Sepolyvirales; Polyomaviridae; Alphapolyomavirus
GCA_000844005.1	5372	6	Mesocricetus auratus polyomavirus 1	NA	Viruses; dsDNA viruses, no RNA stage; Polyomaviridae; Alphapolyomavirus
GCA_000925455.1	5309	5	Pan troglodytes polyomavirus 3	NA	Viruses; Monodnaviria; Shotokuvirae; Cossaviricota; Papovaviricetes; Sepolyvirales; Polyomaviridae; Alphapolyomavirus
GCA_000902835.1	5333	6	Pan troglodytes polyomavirus 4	NA	Viruses; Monodnaviria; Shotokuvirae; Cossaviricota; Papovaviricetes; Sepolyvirales; Polyomaviridae; Alphapolyomavirus
GCA_000902215.1	5349	4	Pan troglodytes polyomavirus 5	NA	Viruses; Monodnaviria; Shotokuvirae; Cossaviricota; Papovaviricetes; Sepolyvirales; Polyomaviridae; Alphapolyomavirus
GCA_000903735.1	4994	4	Pan troglodytes polyomavirus 6	NA	Viruses; Monodnaviria; Shotokuvirae; Cossaviricota; Papovaviricetes; Sepolyvirales; Polyomaviridae; Alphapolyomavirus
GCA_000901175.1	4970	4	Pan troglodytes polyomavirus 7	NA	Viruses; Monodnaviria; Shotokuvirae; Cossaviricota; Papovaviricetes; Sepolyvirales; Polyomaviridae; Alphapolyomavirus
GCA_000868965.1	5079	6	Corvus monedula polyomavirus 1	NA	Viruses; Monodnaviria; Shotokuvirae; Cossaviricota; Papovaviricetes; Sepolyvirales; Polyomaviridae; Gammapolyomavirus
GCA_000864605.1	5278	6	Pyrrhula pyrrhula polyomavirus 1	NA	Viruses; Monodnaviria; Shotokuvirae; Cossaviricota; Papovaviricetes; Sepolyvirales; Polyomaviridae; Gammapolyomavirus
GCA_000836825.1	4697	6	Bos taurus polyomavirus 1	NA	Viruses; dsDNA viruses, no RNA stage; Polyomaviridae; unassigned Polyomaviridae
GCA_000928955.1	5159	5	Delphinus delphis polyomavirus 1	NA	Viruses; Monodnaviria; Shotokuvirae; Cossaviricota; Papovaviricetes; Sepolyvirales; Polyomaviridae
GCA_000928095.1	5167	6	Chlorocebus pygerythrus polyomavirus 2	NA	Viruses; Monodnaviria; Shotokuvirae; Cossaviricota; Papovaviricetes; Sepolyvirales; Polyomaviridae; Betapolyomavirus
GCA_000898215.1	4987	5	Equus caballus polyomavirus 1	NA	Viruses; Monodnaviria; Shotokuvirae; Cossaviricota; Papovaviricetes; Sepolyvirales; Polyomaviridae; Betapolyomavirus
GCA_000837865.1	5153	6	Human polyomavirus 1	NA	Viruses; dsDNA viruses, no RNA stage; Polyomaviridae; Betapolyomavirus
GCA_000837645.1	5243	7	Macaca mulatta polyomavirus 1	NA	Viruses; dsDNA viruses, no RNA stage; Polyomaviridae; Betapolyomavirus
GCA_000928995.1	4899	6	Mastomys natalensis polyomavirus 1	NA	Viruses; Monodnaviria; Shotokuvirae; Cossaviricota; Papovaviricetes; Sepolyvirales; Polyomaviridae; Betapolyomavirus
GCA_000901355.1	5213	5	Miniopterus africanus polyomavirus 1	NA	Viruses; Monodnaviria; Shotokuvirae; Cossaviricota; Papovaviricetes; Sepolyvirales; Polyomaviridae; Betapolyomavirus
GCA_000837065.1	4754	5	Mus musculus polyomavirus 2	Kilham	Viruses; dsDNA viruses, no RNA stage; Polyomaviridae; Betapolyomavirus
GCA_000903015.1	5136	5	Pteronotus davyi polyomavirus 1	NA	Viruses; Monodnaviria; Shotokuvirae; Cossaviricota; Papovaviricetes; Sepolyvirales; Polyomaviridae; Betapolyomavirus
GCA_001744795.1	61626	95	Mycobacterium phage Gengar	NA	Viruses; dsDNA viruses, no RNA stage; Caudovirales; Siphoviridae; unclassified Siphoviridae
GCA_004790035.1	9720	3	Seattle Prectang virus	UW1	Viruses; Riboviria; Orthornavirae; Negarnaviricota; Polyploviricotina; Ellioviricetes; Bunyavirales; Phasmaviridae; Orthophasmavirus; Seattle orthophasmavirus
GCA_003673725.1	10463	4	Mothra virus	JG1	Viruses; Riboviria; Orthornavirae; Negarnaviricota; Polyploviricotina; Ellioviricetes; Bunyavirales; Phenuiviridae; Mobuvirus; Mothra mobuvirus
GCA_001725835.1	30161	10	Rousettus bat coronavirus	NA	Viruses; ssRNA viruses; ssRNA positive-strand viruses, no DNA stage; Nidovirales; Coronaviridae; Coronavirinae; unclassified coronaviruses
GCA_001725935.1	4074	3	Sclerotium hydrophilum virus 1	NA	Viruses; dsRNA viruses; unclassified dsRNA viruses
GCA_000897035.1	1436	1	Cotton leaf curl virus betasatellite	NA	Viruses; Tolecusatellitidae; Betasatellite; unclassified Betasatellite
GCA_001725895.1	32995	45	Sulfolobus islandicus rudivirus 3	NA	Viruses; dsDNA viruses, no RNA stage; Ligamenvirales; Rudiviridae; unclassified Rudiviridae
GCA_002612185.1	61145	97	Mycobacterium phage TBond007	NA	Viruses; Duplodnaviria; Heunggongvirae; Uroviricota; Caudoviricetes; Caudovirales; Siphoviridae; Timquatrovirus; unclassified Timquatrovirus
GCA_002612205.1	51646	87	Mycobacterium phage Bigfoot	NA	Viruses; Duplodnaviria; Heunggongvirae; Uroviricota; Caudoviricetes; Caudovirales; Siphoviridae; Fromanvirus; unclassified Fromanvirus

gca	genome.size	proteome.size	phage.name	strain	Taxonomy
GCA_002758075.1	68938	101	Mycobacterium phage Pinkman	NA	Viruses; Duplodnaviria; Heunggongvirae; Uroviricota; Caudoviricetes; Caudovirales; Siphoviridae; Bclasvirinae; Pegunavirus; Mycobacterium virus Oline
GCA_002612225.1	43977	61	Arthrobacter phage Greenhouse	NA	Viruses; Duplodnaviria; Heunggongvirae; Uroviricota; Caudoviricetes; Caudovirales; Siphoviridae; Korravirus; unclassified Korravirus
GCA_002612245.1	44163	62	Arthrobacter phage Oxynfrius	NA	Viruses; Duplodnaviria; Heunggongvirae; Uroviricota; Caudoviricetes; Caudovirales; Siphoviridae; Korravirus; unclassified Korravirus
GCA_002612265.1	40395	52	Klebsiella phage vB_KpnP_KpV767	NA	Viruses; Duplodnaviria; Heunggongvirae; Uroviricota; Caudoviricetes; Caudovirales; Autographiviridae; Studiervirinae; Przondovirus; Klebsiella virus KpV767
GCA_002612285.1	41283	50	Klebsiella phage vB_KpnP_KpV766	NA	Viruses; Duplodnaviria; Heunggongvirae; Uroviricota; Caudoviricetes; Caudovirales; Autographiviridae; Studiervirinae; Przondovirus; Klebsiella virus KpV766
GCA_002612305.1	155565	233	Mycobacterium phage Erdmann	NA	Viruses; Duplodnaviria; Heunggongvirae; Uroviricota; Caudoviricetes; Caudovirales; Myoviridae; Bixzunavirus; unclassified Bixzunavirus
GCA_002612325.1	155582	232	Mycobacterium phage Yucca	NA	Viruses; Duplodnaviria; Heunggongvirae; Uroviricota; Caudoviricetes; Caudovirales; Myoviridae; Bixzunavirus; unclassified Bixzunavirus
GCA_002612345.2	39631	48	Serratia phage SM93Y	NA	Viruses; Duplodnaviria; Heunggongvirae; Uroviricota; Caudoviricetes; Caudovirales; Autographiviridae; Studiervirinae; Teetrevirus; Serratia virus SM9-3Y
GCA_002612365.1	154474	233	Mycobacterium phage Gabriel	NA	Viruses; Duplodnaviria; Heunggongvirae; Uroviricota; Caudoviricetes; Caudovirales; Myoviridae; Bixzunavirus; unclassified Bixzunavirus
GCA_002612385.1	32826	49	Salinivibrio phage SMHB1	NA	Viruses; Duplodnaviria; Heunggongvirae; Uroviricota; Caudoviricetes; Caudovirales; Myoviridae; Peduovirinae; Playavirus; Salinivibrio virus SMHB1
GCA_002612405.1	134973	283	Rhodococcus phage Weasels2	NA	Viruses; Duplodnaviria; Heunggongvirae; Uroviricota; Caudoviricetes; Caudovirales; Siphoviridae; Weaselvirus; Rhodococcus virus Weasel
GCA_002612425.1	42454	69	Mycobacterium phage PhancyPhin	NA	Viruses; Duplodnaviria; Heunggongvirae; Uroviricota; Caudoviricetes; Caudovirales; Siphoviridae; Nclasvirinae; Redivirus; unclassified Redivirus
GCA_002612445.1	52462	90	Mycobacterium phage Jaan	NA	Viruses; Duplodnaviria; Heunggongvirae; Uroviricota; Caudoviricetes; Caudovirales; Siphoviridae; Fromanvirus; unclassified Fromanvirus
GCA_002612465.1	52753	93	Mycobacterium phage Kerberos	NA	Viruses; Duplodnaviria; Heunggongvirae; Uroviricota; Caudoviricetes; Caudovirales; Siphoviridae; Fromanvirus; unclassified Fromanvirus
GCA_002612485.1	46962	66	Rhodococcus phage Partridge	NA	Viruses; Duplodnaviria; Heunggongvirae; Uroviricota; Caudoviricetes; Caudovirales; Siphoviridae; Rerduovirus; unclassified Rerduovirus
GCA_002612505.1	46930	66	Rhodococcus phage Yogi	NA	Viruses; Duplodnaviria; Heunggongvirae; Uroviricota; Caudoviricetes; Caudovirales; Siphoviridae; Rerduovirus; unclassified Rerduovirus
GCA_002612525.1	39024	45	Escherichia phage JSS1	NA	Viruses; Duplodnaviria; Heunggongvirae; Uroviricota; Caudoviricetes; Caudovirales; Autographiviridae; Studiervirinae; Kayfunavirus; unclassified Kayfunavirus
GCA_002612545.1	58129	92	Mycobacterium phage Marcoliusprime	NA	Viruses; Duplodnaviria; Heunggongvirae; Uroviricota; Caudoviricetes; Caudovirales; Siphoviridae; Timquatrovirus; unclassified Timquatrovirus
GCA_002612565.1	53111	97	Mycobacterium phage Baehexic	NA	Viruses; Duplodnaviria; Heunggongvirae; Uroviricota; Caudoviricetes; Caudovirales; Siphoviridae; Fromanvirus; unclassified Fromanvirus
GCA_002612585.1	52659	97	Mycobacterium phage CactusRose	NA	Viruses; Duplodnaviria; Heunggongvirae; Uroviricota; Caudoviricetes; Caudovirales; Siphoviridae; Fromanvirus; unclassified Fromanvirus
GCA_002758095.1	68314	100	Mycobacterium phage Held	NA	Viruses; Duplodnaviria; Heunggongvirae; Uroviricota; Caudoviricetes; Caudovirales; Siphoviridae; Bclasvirinae; Pegunavirus; Mycobacterium virus Soto
GCA_002758115.1	68492	101	Mycobacterium phage Daffy	NA	Viruses; Duplodnaviria; Heunggongvirae; Uroviricota; Caudoviricetes; Caudovirales; Siphoviridae; Bclasvirinae; Pegunavirus; Mycobacterium virus Soto
GCA_002758135.1	68479	99	Mycobacterium phage Mana	NA	Viruses; Duplodnaviria; Heunggongvirae; Uroviricota; Caudoviricetes; Caudovirales; Siphoviridae; Bclasvirinae; Pegunavirus; Mycobacterium virus Soto
GCA_002612605.1	51371	88	Mycobacterium phage Florean	NA	Viruses; Duplodnaviria; Heunggongvirae; Uroviricota; Caudoviricetes; Caudovirales; Siphoviridae; Fromanvirus; unclassified Fromanvirus
GCA_002612625.1	52911	96	Mycobacterium phage Qobbit	NA	Viruses; Duplodnaviria; Heunggongvirae; Uroviricota; Caudoviricetes; Caudovirales; Siphoviridae; Fromanvirus; unclassified Fromanvirus
GCA_002612645.1	50657	92	Mycobacterium phage BabyRay	NA	Viruses; Duplodnaviria; Heunggongvirae; Uroviricota; Caudoviricetes; Caudovirales; Siphoviridae; Fromanvirus; unclassified Fromanvirus
GCA_002612665.1	53300	99	Mycobacterium phage Phaeder	NA	Viruses; Duplodnaviria; Heunggongvirae; Uroviricota; Caudoviricetes; Caudovirales; Siphoviridae; Fromanvirus; unclassified Fromanvirus
GCA_002612685.1	50841	89	Mycobacterium phage Watson	NA	Viruses; Duplodnaviria; Heunggongvirae; Uroviricota; Caudoviricetes; Caudovirales; Siphoviridae; Fromanvirus; unclassified Fromanvirus
GCA_002612705.1	50066	88	Mycobacterium phage Todacoro	NA	Viruses; Duplodnaviria; Heunggongvirae; Uroviricota; Caudoviricetes; Caudovirales; Siphoviridae; Fromanvirus; unclassified Fromanvirus
GCA_002758155.1	68493	102	Mycobacterium phage MitKao	NA	Viruses; Duplodnaviria; Heunggongvirae; Uroviricota; Caudoviricetes; Caudovirales; Siphoviridae; Bclasvirinae; Pegunavirus; Mycobacterium virus Soto
GCA_002612725.1	149312	265	Escherichia phage ESCO5	NA	Viruses; Duplodnaviria; Heunggongvirae; Uroviricota; Caudoviricetes; Caudovirales; Myoviridae; Phapecoctavirus; Escherichia virus ESCO5
GCA_002612745.1	50087	88	Mycobacterium phage Margo	NA	Viruses; Duplodnaviria; Heunggongvirae; Uroviricota; Caudoviricetes; Caudovirales; Siphoviridae; Fromanvirus; unclassified Fromanvirus
GCA_002612775.1	49658	76	Mycobacterium phage Tortellini	NA	Viruses; Duplodnaviria; Heunggongvirae; Uroviricota; Caudoviricetes; Caudovirales; Siphoviridae; Tortellinivirus; Mycobacterium virus Tortellini
GCA_002612805.1	41901	63	Mycobacterium phage Zombie	NA	Viruses; Duplodnaviria; Heunggongvirae; Uroviricota; Caudoviricetes; Caudovirales; Siphoviridae; Liefievirus; unclassified Liefievirus

gca	genome.size	proteome.size	phage.name	strain	Taxonomy
GCA__002612825.1	51376	89	Mycobacterium phage Cocoaberry	NA	Viruses; Duplodnaviria; Heunggongvirae; Uroviricota; Caudoviricetes; Caudovirales; Siphoviridae; Fromanvirus; unclassified Fromanvirus
GCA__002612845.1	58390	94	Mycobacterium phage Taquito	NA	Viruses; Duplodnaviria; Heunggongvirae; Uroviricota; Caudoviricetes; Caudovirales; Siphoviridae; Timquatrovirus; unclassified Timquatrovirus
GCA__002612865.1	60190	107	Mycobacterium phage Kersh	NA	Viruses; Duplodnaviria; Heunggongvirae; Uroviricota; Caudoviricetes; Caudovirales; Siphoviridae; Cheoctovirus; unclassified Cheoctovirus
GCA__002612885.1	15556	26	Arthrobacter phage Prospero	NA	Viruses; Duplodnaviria; Heunggongvirae; Uroviricota; Caudoviricetes; Caudovirales; Siphoviridae; Decurrovirus; unclassified Decurrovirus
GCA__002612905.2	53163	95	Mycobacterium phage Jerm	NA	Viruses; Duplodnaviria; Heunggongvirae; Uroviricota; Caudoviricetes; Caudovirales; Siphoviridae; Fromanvirus; unclassified Fromanvirus
GCA__002612925.2	51502	87	Mycobacterium phage Roosevelt	NA	Viruses; Duplodnaviria; Heunggongvirae; Uroviricota; Caudoviricetes; Caudovirales; Siphoviridae; Fromanvirus; unclassified Fromanvirus
GCA__002612945.1	75779	142	Mycobacterium phage Pharsalus	NA	Viruses; Duplodnaviria; Heunggongvirae; Uroviricota; Caudoviricetes; Caudovirales; Siphoviridae; Kostyavirus; unclassified Kostyavirus
GCA__002612965.1	76363	132	Mycobacterium phage Zakai	NA	Viruses; Duplodnaviria; Heunggongvirae; Uroviricota; Caudoviricetes; Caudovirales; Siphoviridae; Bronvirus; unclassified Bronvirus
GCA__002612985.1	75806	130	Mycobacterium phage Wilder	NA	Viruses; Duplodnaviria; Heunggongvirae; Uroviricota; Caudoviricetes; Caudovirales; Siphoviridae; Bronvirus; unclassified Bronvirus
GCA__002709945.2	44024	53	Klebsiella phage phiKpS2	NA	Viruses; Duplodnaviria; Heunggongvirae; Uroviricota; Caudoviricetes; Caudovirales; Autographiviridae; Slopekvirinae; Drulisvirus; Klebsiella virus KpS2
GCA__002613005.1	59812	95	Mycobacterium phage HedwigODU	NA	Viruses; Duplodnaviria; Heunggongvirae; Uroviricota; Caudoviricetes; Caudovirales; Siphoviridae; Timquatrovirus; unclassified Timquatrovirus
GCA__002613025.1	53426	100	Mycobacterium phage HortumSL17	NA	Viruses; Duplodnaviria; Heunggongvirae; Uroviricota; Caudoviricetes; Caudovirales; Siphoviridae; Fromanvirus; unclassified Fromanvirus
GCA__002613045.1	61248	94	Mycobacterium phage Waterfoul	NA	Viruses; Duplodnaviria; Heunggongvirae; Uroviricota; Caudoviricetes; Caudovirales; Siphoviridae; Timquatrovirus; unclassified Timquatrovirus
GCA__002613065.1	50899	89	Mycobacterium phage Louie6	NA	Viruses; Duplodnaviria; Heunggongvirae; Uroviricota; Caudoviricetes; Caudovirales; Siphoviridae; Fromanvirus; unclassified Fromanvirus
GCA__002613085.1	56189	88	Streptomyces phage OlympicHelado	NA	Viruses; Duplodnaviria; Heunggongvirae; Uroviricota; Caudoviricetes; Caudovirales; Siphoviridae; Rimavirus; unclassified Rimavirus
GCA__002613105.2	56168	87	Streptomyces phage Rima	NA	Viruses; Duplodnaviria; Heunggongvirae; Uroviricota; Caudoviricetes; Caudovirales; Siphoviridae; Rimavirus; Strepomyces virus Rima
GCA__002613125.1	69798	111	Arthrobacter phage Chocolat	NA	Viruses; Duplodnaviria; Heunggongvirae; Uroviricota; Caudoviricetes; Caudovirales; Myoviridae; Radnorvirus; unclassified Radnorvirus
GCA__002613145.1	70258	112	Arthrobacter phage Chubster	NA	Viruses; Duplodnaviria; Heunggongvirae; Uroviricota; Caudoviricetes; Caudovirales; Myoviridae; Radnorvirus; unclassified Radnorvirus
GCA__002758175.1	69037	100	Mycobacterium phage Lego3393	NA	Viruses; Duplodnaviria; Heunggongvirae; Uroviricota; Caudoviricetes; Caudovirales; Siphoviridae; Bclasvirinae; Pegunavirus; Mycobacterium virus Soto
GCA__009828315.1	42147	63	Salmonella phage SEW109	NA	Viruses; Duplodnaviria; Heunggongvirae; Uroviricota; Caudoviricetes; Caudovirales; Siphoviridae; Guernseyvirinae; Jerseyvirus; unclassified Jerseyvirus
GCA__002613165.1	35284	55	Propionibacterium phage Anatole	NA	Viruses; Duplodnaviria; Heunggongvirae; Uroviricota; Caudoviricetes; Caudovirales; Siphoviridae; Anatolevirus; Propionibacterium virus Anatole
GCA__002613185.1	37219	61	Propionibacterium phage B22	NA	Viruses; Duplodnaviria; Heunggongvirae; Uroviricota; Caudoviricetes; Caudovirales; Siphoviridae; Doucettevirus; Propionibacterium virus B22
GCA__002613205.1	35948	57	Propionibacterium phage B3	NA	Viruses; Duplodnaviria; Heunggongvirae; Uroviricota; Caudoviricetes; Caudovirales; Siphoviridae; Anatolevirus; Propionibacterium virus B3
GCA__002613225.1	37429	61	Propionibacterium phage Doucette	NA	Viruses; Duplodnaviria; Heunggongvirae; Uroviricota; Caudoviricetes; Caudovirales; Siphoviridae; Doucettevirus; Propionibacterium virus Doucette
GCA__002613245.1	35209	55	Propionibacterium phage E1	NA	Viruses; Duplodnaviria; Heunggongvirae; Uroviricota; Caudoviricetes; Caudovirales; Siphoviridae; Anatolevirus; unclassified Anatolevirus
GCA__002613265.1	38067	60	Propionibacterium phage E6	NA	Viruses; Duplodnaviria; Heunggongvirae; Uroviricota; Caudoviricetes; Caudovirales; Siphoviridae; Doucettevirus; Propionibacterium virus E6
GCA__002613285.1	38555	69	Propionibacterium phage G4	NA	Viruses; Duplodnaviria; Heunggongvirae; Uroviricota; Caudoviricetes; Caudovirales; Siphoviridae; Doucettevirus; Propionibacterium virus G4
GCA__001725855.1	2220	3	Common beanassociated gemycircularvirus	NA	Viruses; unclassified viruses; Genomoviridae; Gemycircularvirus; unclassified Gemycircularvirus
GCA__002613305.1	26559	40	Leuconostoc phage LDG	NA	Viruses; Duplodnaviria; Heunggongvirae; Uroviricota; Caudoviricetes; Caudovirales; Siphoviridae; Mccleskeyvirinae; Limdunavirus; unlassified Lmd1virus
GCA__002613325.1	51257	87	Mycobacterium phage Mundrea	NA	Viruses; Duplodnaviria; Heunggongvirae; Uroviricota; Caudoviricetes; Caudovirales; Siphoviridae; Fromanvirus; unclassified Fromanvirus
GCA__002613345.1	46527	65	Rhodococcus phage Natosaleda	NA	Viruses; Duplodnaviria; Heunggongvirae; Uroviricota; Caudoviricetes; Caudovirales; Siphoviridae; Rerduovirus; unclassified Rerduovirus
GCA__002613365.1	52732	91	Mycobacterium phage PacerPaul	NA	Viruses; Duplodnaviria; Heunggongvirae; Uroviricota; Caudoviricetes; Caudovirales; Siphoviridae; Fromanvirus; unclassified Fromanvirus
GCA__002613385.1	52833	93	Mycobacterium phage Pomar16	NA	Viruses; Duplodnaviria; Heunggongvirae; Uroviricota; Caudoviricetes; Caudovirales; Siphoviridae; Fromanvirus; unclassified Fromanvirus
GCA__002613405.1	51910	96	Mycobacterium phage Isiphiwo	NA	Viruses; Duplodnaviria; Heunggongvirae; Uroviricota; Caudoviricetes; Caudovirales; Siphoviridae; Fromanvirus; unclassified Fromanvirus

gca	genome.size	proteome.size	phage.name	strain	Taxonomy
GCA_002613425.1	53330	96	Mycobacterium phage Kalpine	NA	Viruses; Duplodnaviria; Heunggongvirae; Uroviricota; Caudoviricetes; Caudovirales; Siphoviridae; Fromanvirus; unclassified Fromanvirus
GCA_002613445.1	60053	93	Mycobacterium phage LindNT	NA	Viruses; Duplodnaviria; Heunggongvirae; Uroviricota; Caudoviricetes; Caudovirales; Siphoviridae; Timquatrovirus; unclassified Timquatrovirus
GCA_002613465.1	48870	83	Mycobacterium phage Nazo	NA	Viruses; Duplodnaviria; Heunggongvirae; Uroviricota; Caudoviricetes; Caudovirales; Siphoviridae; Pclasvirinae; Bignuzvirus; unclassified Bignuzvirus
GCA_002613485.1	51591	85	Mycobacterium phage Stasia	NA	Viruses; Duplodnaviria; Heunggongvirae; Uroviricota; Caudoviricetes; Caudovirales; Siphoviridae; Fromanvirus; unclassified Fromanvirus
GCA_002613505.1	41077	60	Mycobacterium phage Taheera	NA	Viruses; Duplodnaviria; Heunggongvirae; Uroviricota; Caudoviricetes; Caudovirales; Siphoviridae; Liefievirus; unclassified Liefievirus
GCA_002613525.1	41077	60	Mycobacterium phage Terror	NA	Viruses; Duplodnaviria; Heunggongvirae; Uroviricota; Caudoviricetes; Caudovirales; Siphoviridae; Liefievirus; unclassified Liefievirus
GCA_002613545.1	46383	66	Rhodococcus phage Harlequin	NA	Viruses; Duplodnaviria; Heunggongvirae; Uroviricota; Caudoviricetes; Caudovirales; Siphoviridae; Rerduovirus; unclassified Rerduovirus
GCA_002758195.1	68597	101	Mycobacterium phage Iridoclysis	NA	Viruses; Duplodnaviria; Heunggongvirae; Uroviricota; Caudoviricetes; Caudovirales; Siphoviridae; Bclasvirinae; Pegunavirus; Mycobacterium virus Soto
GCA_002613565.1	43914	62	Arthrobacter phage Suppi	NA	Viruses; Duplodnaviria; Heunggongvirae; Uroviricota; Caudoviricetes; Caudovirales; Siphoviridae; Korravirus; unclassified Korravirus
GCA_002613585.1	43607	62	Arthrobacter phage Vallejo	NA	Viruses; Duplodnaviria; Heunggongvirae; Uroviricota; Caudoviricetes; Caudovirales; Siphoviridae; Korravirus; unclassified Korravirus
GCA_002613605.1	37631	53	Streptococcus phage SpGS1	NA	Viruses; Duplodnaviria; Heunggongvirae; Uroviricota; Caudoviricetes; Caudovirales; Siphoviridae; unclassified Siphoviridae
GCA_002613625.1	41655	58	Ochrobactrum phage POA1180	NA	Viruses; Duplodnaviria; Heunggongvirae; Uroviricota; Caudoviricetes; Caudovirales; Myoviridae; unclassified Myoviridae
GCA_002613645.2	40291	44	Escherichia phage ZG49	NA	Viruses; Duplodnaviria; Heunggongvirae; Uroviricota; Caudoviricetes; Caudovirales; Autographiviridae; Studiervirinae; Kayfunavirus; Escherichia virus ZG49
GCA_001444105.1	4850	3	Aphis glycines virus 2	NA	Viruses; unclassified viruses
GCA_002613665.1	28093	47	Leuconostoc phage Ln7	NA	Viruses; Duplodnaviria; Heunggongvirae; Uroviricota; Caudoviricetes; Caudovirales; Siphoviridae; Mccleskeyvirinae; Unaquatrovirus; unclassified Unaquatrovirus
GCA_002613685.1	28265	45	Leuconostoc phage CHB	NA	Viruses; Duplodnaviria; Heunggongvirae; Uroviricota; Caudoviricetes; Caudovirales; Siphoviridae; Mccleskeyvirinae; Unaquatrovirus; unclassified Unaquatrovirus
GCA_002613705.1	28142	43	Leuconostoc phage CHA	NA	Viruses; Duplodnaviria; Heunggongvirae; Uroviricota; Caudoviricetes; Caudovirales; Siphoviridae; Mccleskeyvirinae; Unaquatrovirus; unclassified Unaquatrovirus
GCA_008894185.1	59132	75	Salmonella phage ST101	NA	Viruses; Duplodnaviria; Heunggongvirae; Uroviricota; Caudoviricetes; Caudovirales; Siphoviridae; Chivirus; unclassified Chivirus
GCA_008894245.1	59106	77	Salmonella phage ST118	NA	Viruses; Duplodnaviria; Heunggongvirae; Uroviricota; Caudoviricetes; Caudovirales; Siphoviridae; Chivirus; unclassified Chivirus
GCA_009828335.1	59934	76	Salmonella phage ST374	NA	Viruses; Duplodnaviria; Heunggongvirae; Uroviricota; Caudoviricetes; Caudovirales; Siphoviridae; Chivirus; unclassified Chivirus
GCA_009828355.1	157458	210	Salmonella phage STW77	NA	Viruses; Duplodnaviria; Heunggongvirae; Uroviricota; Caudoviricetes; Caudovirales; Ackermannviridae; Cvivirinae; Kuttervirus; unclassified Kuttervirus
GCA_002613725.1	41841	47	Pectobacterium phage PP2	NA	Viruses; Duplodnaviria; Heunggongvirae; Uroviricota; Caudoviricetes; Caudovirales; Autographiviridae; Melnykvirinae; Wanjuvirus; Pectobacterium virus PP2
GCA_002613745.1	53226	90	Vibrio phage Cla	NA	Viruses; Duplodnaviria; Heunggongvirae; Uroviricota; Caudoviricetes; Caudovirales; Siphoviridae; unclassified Siphoviridae
GCA_002613765.1	53226	90	Vibrio phage Her	NA	Viruses; Duplodnaviria; Heunggongvirae; Uroviricota; Caudoviricetes; Caudovirales; Siphoviridae; unclassified Siphoviridae
GCA_002613785.1	53226	90	Vibrio phage Len	NA	Viruses; Duplodnaviria; Heunggongvirae; Uroviricota; Caudoviricetes; Caudovirales; Siphoviridae; unclassified Siphoviridae
GCA_002613805.1	53227	90	Vibrio phage Pel	NA	Viruses; Duplodnaviria; Heunggongvirae; Uroviricota; Caudoviricetes; Caudovirales; Siphoviridae; unclassified Siphoviridae
GCA_002613825.1	53226	90	Vibrio phage Strym	NA	Viruses; Duplodnaviria; Heunggongvirae; Uroviricota; Caudoviricetes; Caudovirales; Siphoviridae; unclassified Siphoviridae
GCA_002613845.1	53216	91	Vibrio phage VaK	NA	Viruses; Duplodnaviria; Heunggongvirae; Uroviricota; Caudoviricetes; Caudovirales; Siphoviridae; unclassified Siphoviridae
GCA_002613865.1	53227	90	Vibrio phage pVa1	NA	Viruses; Duplodnaviria; Heunggongvirae; Uroviricota; Caudoviricetes; Caudovirales; Siphoviridae; unclassified Siphoviridae
GCA_002613885.1	53286	89	Vibrio phage pVa2	NA	Viruses; Duplodnaviria; Heunggongvirae; Uroviricota; Caudoviricetes; Caudovirales; Siphoviridae; unclassified Siphoviridae
GCA_002613925.1	53227	90	Vibrio phage pVa6	NA	Viruses; Duplodnaviria; Heunggongvirae; Uroviricota; Caudoviricetes; Caudovirales; Siphoviridae; unclassified Siphoviridae
GCA_002613945.1	54268	92	Vibrio phage pVa7	NA	Viruses; Duplodnaviria; Heunggongvirae; Uroviricota; Caudoviricetes; Caudovirales; Siphoviridae; unclassified Siphoviridae
GCA_002613965.1	42944	60	Arthrobacter phage Lucy	NA	Viruses; Duplodnaviria; Heunggongvirae; Uroviricota; Caudoviricetes; Caudovirales; Siphoviridae; Korravirus; unclassified Korravirus
GCA_002613985.1	15556	26	Arthrobacter phage Massimo	NA	Viruses; Duplodnaviria; Heunggongvirae; Uroviricota; Caudoviricetes; Caudovirales; Siphoviridae; Decurrovirus; unclassified Decurrovirus

gca	genome.size	proteome.size	phage.name	strain	Taxonomy
GCA_002614005.1	43891	62	Arthrobacter phage RcigaStruga	NA	Viruses; Duplodnaviria; Heunggongvirae; Uroviricota; Caudoviricetes; Caudovirales; Siphoviridae; Korravirus; unclassified Korravirus
GCA_002758215.1	68604	100	Mycobacterium phage CharlieGBrown	NA	Viruses; Duplodnaviria; Heunggongvirae; Uroviricota; Caudoviricetes; Caudovirales; Siphoviridae; Bclasvirinae; Pegunavirus; Mycobacterium virus Oline
GCA_002758235.1	68637	101	Mycobacterium phage Derpp	NA	Viruses; Duplodnaviria; Heunggongvirae; Uroviricota; Caudoviricetes; Caudovirales; Siphoviridae; Bclasvirinae; Pegunavirus; Mycobacterium virus Oline
GCA_002614025.1	52917	94	Mycobacterium phage DudeLittle	NA	Viruses; Duplodnaviria; Heunggongvirae; Uroviricota; Caudoviricetes; Caudovirales; Siphoviridae; Fromanvirus; unclassified Fromanvirus
GCA_002758255.1	68415	99	Mycobacterium phage FriarPreacher	NA	Viruses; Duplodnaviria; Heunggongvirae; Uroviricota; Caudoviricetes; Caudovirales; Siphoviridae; Bclasvirinae; Pegunavirus; Mycobacterium virus Soto
GCA_002758275.1	68637	102	Mycobacterium phage TyrionL	NA	Viruses; Duplodnaviria; Heunggongvirae; Uroviricota; Caudoviricetes; Caudovirales; Siphoviridae; Bclasvirinae; Pegunavirus; Mycobacterium virus Oline
GCA_002614045.1	58608	104	Mycobacterium phage WillSterrel	NA	Viruses; Duplodnaviria; Heunggongvirae; Uroviricota; Caudoviricetes; Caudovirales; Siphoviridae; Cheoctovirus; unclassified Cheoctovirus
GCA_002614065.1	52195	101	Mycobacterium phage Gruunaga	NA	Viruses; Duplodnaviria; Heunggongvirae; Uroviricota; Caudoviricetes; Caudovirales; Siphoviridae; Fromanvirus; unclassified Fromanvirus
GCA_001974475.1	208501	183	Lymphocystis disease virus Sa	NA	Viruses; dsDNA viruses, no RNA stage; Iridoviridae; Lymphocystivirus; unclassified Lymphocystivirus
GCA_004129715.1	6219	2	Eimeria stiedai RNA virus 1	NA	Viruses; Riboviria; Orthornavirae; Duplornaviricota; Chrymotiviricetes; Ghabrivirales; Totiviridae; unclassified Totiviridae
GCA_000838285.1	5804	10	Propionibacterium phage B5	NA	Viruses; ssDNA viruses; Inoviridae; Inovirus; unclassified Inovirus
GCA_002614085.1	57900	63	Acidianus twotailed virus 2	NA	Viruses; Bicaudaviridae; Bicaudavirus; unclassified Bicaudavirus
GCA_002614105.1	61783	66	Acidianus twotailed virus variant 1	NA	Viruses; Bicaudaviridae; Bicaudavirus; unclassified Bicaudavirus
GCA_001755365.1	2749	7	Synedrella leaf curl virus	NA	Viruses; ssDNA viruses; Geminiviridae; Begomovirus; unclassified Begomovirus
GCA_002966345.1	1182801	988	Acanthamoeba castellanii mimivirus	Mimivirus kasaii	Viruses; Varidnaviria; Bamfordvirae; Nucleocytoviricota; Megaviricetes; Imitervirales; Mimiviridae; Mimivirus
GCA_001744835.1	8525	6	Ligustrum virus A	NA	Viruses; ssRNA viruses; ssRNA positive-strand viruses, no DNA stage; Tymovirales; Betaflexiviridae; Quinvirinae; Carlavirus; unclassified Carlavirus
GCA_009738675.1	43804	38	Fowl aviadenovirus A	CELOCORR	Viruses; dsDNA viruses, no RNA stage; Adenoviridae; Aviadenovirus
GCA_006547225.1	45743	36	Fowl aviadenovirus B	40440M2015 Debrecen	Viruses; Varidnaviria; Bamfordvirae; Preplasmiviricota; Tectiliviricetes; Rowavirales; Adenoviridae; Aviadenovirus
GCA_009738735.1	45554	53	Fowl aviadenovirus C	C2BFAdV10	Viruses; dsDNA viruses, no RNA stage; Adenoviridae; Aviadenovirus
GCA_006434175.1	44054	40	Fowl aviadenovirus D	NA	Viruses; dsDNA viruses, no RNA stage; Adenoviridae; Aviadenovirus
GCA_000890555.1	44055	46	Fowl aviadenovirus E	NA	Viruses; dsDNA viruses, no RNA stage; Adenoviridae; Aviadenovirus
GCA_002608185.1	39399	69	Sulfolobus islandicus filamentous virus 2	NA	Viruses; Ligamenvirales; Lipothrixviridae; Betalipothrixvirus; Sulfolobus islandicus filamentous virus
GCA_000851625.1	7453	2	Calicivirus strain NB	NA	Viruses; ssRNA viruses; ssRNA positive-strand viruses, no DNA stage; Caliciviridae; Nebovirus; Newbury-1 virus
GCA_001744155.1	8938	1	Rosavirus B	RNCW0602091R	Viruses; ssRNA viruses; ssRNA positive-strand viruses, no DNA stage; Picornavirales; Picornaviridae; Rosavirus; unclassified Rosavirus
GCA_001745475.1	9112	1	Rosavirus C	RATLC11A	Viruses; ssRNA viruses; ssRNA positive-strand viruses, no DNA stage; Picornavirales; Picornaviridae; Rosavirus; unclassified Rosavirus
GCA_002614125.1	38989	44	Liberibacter phage HHCA12	NA	Viruses; Duplodnaviria; Heunggongvirae; Uroviricota; Caudoviricetes; Caudovirales; Podoviridae; unclassified Podoviridae
GCA_002633255.1	48157	58	Vibrio phage CKBS1	NA	Viruses; Duplodnaviria; Heunggongvirae; Uroviricota; Caudoviricetes; Caudovirales; Myoviridae; unclassified Myoviridae
GCA_002633285.1	34713	49	Vibrio phage CKBS2	NA	Viruses; Duplodnaviria; Heunggongvirae; Uroviricota; Caudoviricetes; Caudovirales; Podoviridae; unclassified Podoviridae
GCA_001755385.1	7753	3	Primate norovirus	SimianNoVnj	Viruses; ssRNA viruses; ssRNA positive-strand viruses, no DNA stage; Caliciviridae; Norovirus; unclassified Norovirus
GCA_001754965.1	10588	2	Anopheles flavivirus variant 1	NA	Viruses; Riboviria; Orthornavirae; Kitrinoviricota; Flasuviricetes; Amarillovirales; Flaviviridae; unclassified Flaviviridae; Anopheles flavivirus Liberia/2013
GCA_003673905.1	11672	6	Bolahun virus variant 2	NA	Viruses; Riboviria; Orthornavirae; Negarnaviricota; Haplovirictina; Monjiviricetes; Mononegavirales; Xinmoviridae; Anphevirus; Bolahun anphevirus; Bolahun virus
GCA_002037795.1	4752	6	Miniopterus schreibersii polyomavirus 1	12SuB07	Viruses; dsDNA viruses, no RNA stage; Polyomaviridae; unclassified Polyomaviridae
GCA_002037755.1	4877	6	Miniopterus schreibersii polyomavirus 2	12SuB08	Viruses; dsDNA viruses, no RNA stage; Polyomaviridae; unclassified Polyomaviridae
GCA_002037695.1	5060	6	Rhinolophus hildebrandtii polyomavirus 1	12SuB17	Viruses; dsDNA viruses, no RNA stage; Polyomaviridae; unclassified Polyomaviridae

gca	genome.size	proteome.size	phage.name	strain	Taxonomy
GCA_002037715.1	5014	6	Rousettus aegyptiacus polyomavirus 1	12SuB01	Viruses; dsDNA viruses, no RNA stage; Polyomaviridae; unclassified Polyomaviridae
GCA_001754525.1	8126	4	Simian retrovirus 8	SRV8SUZ2012	Viruses; Riboviria; Pararnavirae; Artverviricota; Revtraviricetes; Ortervirales; Retroviridae; Orthoretrovirinae; Betaretrovirus; unclassified Betaretrovirus
GCA_002614145.1	134073	169	Campylobacter phage vB_CjeM_Los1	NA	Viruses; Duplodnaviria; Heunggongvirae; Uroviricota; Caudoviricetes; Caudovirales; Myoviridae; Eucampyvirinae; Fletchervirus; Campylobacter virus Los1
GCA_000839145.1	33507	50	Clostridium phage phi3626	NA	Viruses; dsDNA viruses, no RNA stage; Caudovirales; Siphoviridae; unclassified Siphoviridae
GCA_003727435.1	9585	1	Human DNA virus	NA	Viruses; unclassified viruses
GCA_001777185.1	2636	5	Macroptilium bright mosaic virus	NA	Viruses; ssDNA viruses; Geminiviridae; Begomovirus; unclassified Begomovirus
GCA_001777205.1	5230	8	Macroptilium common mosaic virus	NA	Viruses; ssDNA viruses; Geminiviridae; Begomovirus; unclassified Begomovirus
GCA_001777325.1	5349	8	Sida angular mosaic virus	NA	Viruses; ssDNA viruses; Geminiviridae; Begomovirus; unclassified Begomovirus
GCA_001777265.1	5145	7	Sida chlorotic vein virus	NA	Viruses; ssDNA viruses; Geminiviridae; Begomovirus; unclassified Begomovirus
GCA_001777245.1	2621	5	Wissadula yellow mosaic virus	NA	Viruses; ssDNA viruses; Geminiviridae; Begomovirus; unclassified Begomovirus
GCA_002614165.1	43557	47	Klebsiella phage KPRio2015	NA	Viruses; Duplodnaviria; Heunggongvirae; Uroviricota; Caudoviricetes; Caudovirales; Autographiviridae; Slopekvirinae; Drulisvirus; Klebsiella virus KPRio2015
GCA_004129495.1	7342	5	Rusa timorensis papillomavirus type 2	NA	Viruses; Monodnaviria; Shotokuvirae; Cossaviricota; Papovaviricetes; Zurhausenvirales; Papillomaviridae; unclassified Papillomaviridae; non-primate mammal papillomaviruses
GCA_013087375.1	13320	7	Physostegia chlorotic mottle virus	NA	Viruses; Riboviria; Orthornavirae; Negarnaviricota; Haploviricotina; Monjiviricetes; Mononegavirales; Rhabdoviridae; Alphanucleorhabdovirus; Physostegia chlorotic mottle alphanucleorhabdovirus
GCA_001925335.1	5547	2	Bastroviruslike_virusVietNamBat17819_21	NA	Viruses; ssRNA viruses; ssRNA positive-strand viruses, no DNA stage; Astroviridae; unclassified Astroviridae
GCA_002271045.1	5850	2	BastrovirusVietNamBat16715_78	NA	Viruses; Riboviria; Orthornavirae; Pisuviricota; Stelpaviricetes; Stellavirales; Astroviridae; unclassified Astroviridae; Bastrovirus BAS-2
GCA_001925475.1	6009	2	BastrovirusVietNamPorcine17489_85	NA	Viruses; ssRNA viruses; ssRNA positive-strand viruses, no DNA stage; Astroviridae; unclassified Astroviridae
GCA_001926815.1	5818	2	BastrovirusVietNamRat16715_10	NA	Viruses; ssRNA viruses; ssRNA positive-strand viruses, no DNA stage; Astroviridae; unclassified Astroviridae
GCA_001925975.1	5937	2	BastrovirusVietNamRat16715_26	NA	Viruses; ssRNA viruses; ssRNA positive-strand viruses, no DNA stage; Astroviridae; unclassified Astroviridae
GCA_001925315.1	5778	2	BastrovirusVietNamRat16715_58	NA	Viruses; ssRNA viruses; ssRNA positive-strand viruses, no DNA stage; Astroviridae; unclassified Astroviridae
GCA_013087385.1	142900	147	Cyclophragma undans nucleopolyhedrovirus	NA	Viruses; Baculoviridae; Alphabaculovirus
GCA_001766585.1	10056	1	Moku virus	NA	Viruses; ssRNA viruses; ssRNA positive-strand viruses, no DNA stage; Picornavirales; Iflaviridae; Iflavirus; unclassified Iflavirus
GCA_001766605.1	2905	6	Grapevine geminivirus A	NA	Viruses; ssDNA viruses; Geminiviridae; unclassified Geminiviridae
GCA_008775755.1	7077	1	Human parechovirus 17	M36CI2014	Viruses; Riboviria; Orthornavirae; Pisuviricota; Pisoniviricetes; Picornavirales; Picornaviridae; Parechovirus; Parechovirus A
GCA_001777305.1	5213	8	Malvastrum bright yellow mosaic virus	NA	Viruses; ssDNA viruses; Geminiviridae; Begomovirus; unclassified Begomovirus
GCA_002614205.1	75728	150	Mycobacterium phage Goldilocks	NA	Viruses; Duplodnaviria; Heunggongvirae; Uroviricota; Caudoviricetes; Caudovirales; Siphoviridae; Kostyavirus; unclassified Kostyavirus
GCA_002646335.1	18414	11	Rotavirus A RVAHumanwtTHAKKL1172014G9P23	NA	Viruses; Riboviria; Orthornavirae; Duplornaviricota; Resentoviricetes; Reovirales; Reoviridae; Sedoreovirinae; Rotavirus; Rotavirus A; Rotavirus A isolates
GCA_014840105.1	75764	97	Pectobacterium phage A38	NA	Viruses; Duplodnaviria; Heunggongvirae; Uroviricota; Caudoviricetes; Caudovirales; Podoviridae; unclassified Podoviridae
GCA_009372315.1	35215	46	Human adenovirus sp	NA	Viruses; Varidnaviria; Bamfordvirae; Preplasmiviricota; Tectiliviricetes; Rowavirales; Adenoviridae; Mastadenovirus; unclassified Human adenoviruses
GCA_001831345.1	41981	35	Pigeon adenovirus 2	NA	Viruses; dsDNA viruses, no RNA stage; Adenoviridae; Aviadenovirus; unclassified Aviadenovirus
GCA_001777225.1	5166	5	Parus major densovirus	NA	Viruses; ssDNA viruses; Parvoviridae; Densovirinae; unclassified Densovirinae
GCA_002614225.1	40605	52	Klebsiella phage vB_KpnP_PRA33	NA	Viruses; Duplodnaviria; Heunggongvirae; Uroviricota; Caudoviricetes; Caudovirales; Autographiviridae; Studiervirinae; Przondovirus; Klebsiella virus PRA33
GCA_002614245.1	41697	56	Klebsiella phage vB_KpnP_BIS33	NA	Viruses; Duplodnaviria; Heunggongvirae; Uroviricota; Caudoviricetes; Caudovirales; Autographiviridae; Studiervirinae; Przondovirus; Klebsiella virus BIS33
GCA_002614265.1	41335	54	Klebsiella phage vB_KpnP_IL33	NA	Viruses; Duplodnaviria; Heunggongvirae; Uroviricota; Caudoviricetes; Caudovirales; Autographiviridae; Studiervirinae; Przondovirus; Klebsiella virus IL33
GCA_002614285.1	147443	262	Klebsiella phage vB_KpnM_BIS47	NA	Viruses; Duplodnaviria; Heunggongvirae; Uroviricota; Caudoviricetes; Caudovirales; Myoviridae; Vequintavirinae; unclassified Vequintavirinae

gca	genome.size	proteome.size	phage.name	strain	Taxonomy
GCA_004117395.1	4297	4	Picobirnavirus sp	PBVroe_deerSLOD38142014	Viruses; Riboviria; Orthornavirae; Pisuviricota; Duplopiviricetes; Durnavirales; Picobirnaviridae; Picobirnavirus; unclassified Picobirnavirus
GCA_000850505.1	1104	1	Arabis mosaic virus large satellite RNA	NA	Viruses; Satellites; Satellite Nucleic Acids; Single stranded RNA satellites; Large single stranded RNA satellites
GCA_000850485.1	836	1	Bamboo mosaic virus satellite RNA	NA	Viruses; Satellites; Satellite Nucleic Acids; Single stranded RNA satellites; Large single stranded RNA satellites
GCA_002614305.1	160342	210	Salmonella phage STP07	NA	Viruses; Duplodnaviria; Heunggongvirae; Uroviricota; Caudoviricetes; Caudovirales; Ackermannviridae; Cvivirinae; Kuttervirus; unclassified Kuttervirus
GCA_002374895.1	5050	2	Tetraparvovirus sp	NA	Viruses; Monodnaviria; Shotokuvirae; Cossaviricota; Quintoviricetes; Piccovirales; Parvoviridae; Parvovirinae; Tetraparvovirus; unclassified Tetraparvovirus
GCA_002374935.1	4662	2	Amdoparvovirus sp	NA	Viruses; Monodnaviria; Shotokuvirae; Cossaviricota; Quintoviricetes; Piccovirales; Parvoviridae; Parvovirinae; Amdoparvovirus; unclassified Amdoparvovirus
GCA_001792745.1	15207	1	Ceratobasidium endornavirus A	NA	Viruses; dsRNA viruses; Endornaviridae; Endornavirus; unclassified Endornavirus
GCA_001792725.1	23635	2	Ceratobasidium endornavirus B	NA	Viruses; dsRNA viruses; Endornaviridae; Endornavirus; unclassified Endornavirus
GCA_001777285.1	19406	1	Ceratobasidium endornavirus D	NA	Viruses; dsRNA viruses; Endornaviridae; Endornavirus; unclassified Endornavirus
GCA_001792765.1	19293	2	Ceratobasidium endornavirus G	NA	Viruses; dsRNA viruses; Endornaviridae; Endornavirus; unclassified Endornavirus
GCA_002149885.1	21004	2	Ceratobasidium endornavirus C	NA	Viruses; dsRNA viruses; Endornaviridae; unclassified Endornavirus
GCA_002614325.1	26054	41	Bacillus phage QCM11	NA	Viruses; Duplodnaviria; Heunggongvirae; Uroviricota; Caudoviricetes; Caudovirales; Podoviridae; unclassified Podoviridae
GCA_002614345.1	161643	298	Bacillus phage SBP8a	NA	Viruses; Duplodnaviria; Heunggongvirae; Uroviricota; Caudoviricetes; Caudovirales; Herelleviridae; Bastillevirinae; Wphvirus; unclassified Wphvirus
GCA_002614365.1	160732	298	Bacillus phage BJ4	NA	Viruses; Duplodnaviria; Heunggongvirae; Uroviricota; Caudoviricetes; Caudovirales; Herelleviridae; Bastillevirinae; Wphvirus; unclassified Wphvirus
GCA_002614385.1	164911	288	Bacillus phage QCM8	NA	Viruses; Duplodnaviria; Heunggongvirae; Uroviricota; Caudoviricetes; Caudovirales; Herelleviridae; Bastillevirinae; Tsarbombavirus; unclassified Tsarbombavirus
GCA_002614405.1	44520	77	Rathayibacter phage NCPPB3778	NA	Viruses; Duplodnaviria; Heunggongvirae; Uroviricota; Caudoviricetes; Caudovirales; Siphoviridae; unclassified Siphoviridae
GCA_001866895.1	9850	5	Lodeiro virus	N10	Viruses; unclassified viruses
GCA_001866955.1	10193	1	King virus	UWV2	Viruses; ssRNA viruses; ssRNA positive-strand viruses, no DNA stage; Picornavirales; Iflaviridae; unclassified Iflaviridae
GCA_003728895.1	8825	1	Rainier virus	UW1	Viruses; unclassified viruses
GCA_001866245.1	11308	5	Orgi virus	SP1	Viruses; ssRNA viruses; ssRNA negative-strand viruses; Mononegavirales; Rhabdoviridae; unclassified Rhabdoviridae
GCA_001866265.1	11238	5	Gata virus	M4	Viruses; ssRNA viruses; ssRNA negative-strand viruses; Mononegavirales; Rhabdoviridae; unclassified Rhabdoviridae
GCA_003673745.1	11774	3	Pidgey virus	M6	Viruses; Riboviria; Orthornavirae; Negarnaviricota; Polyploviricotina; Ellioviricetes; Bunyavirales; Phenuiviridae; Pidchovirus; Pidgey pidchovirus
GCA_001866305.1	9875	1	Bradson virus	HN1	Viruses; unclassified viruses
GCA_001866325.1	9220	1	Corey virus	UWV	Viruses; unclassified viruses
GCA_001866285.1	9018	2	Mosquito dicistrovirus	FH1	Viruses; ssRNA viruses; ssRNA positive-strand viruses, no DNA stage; Picornavirales; Dicistroviridae; unclassified Dicistroviridae; Centovirus
GCA_003033275.1	4822	5	Miniopterus schreibersii bat bocavirus	str24	Viruses; Monodnaviria; Shotokuvirae; Cossaviricota; Quintoviricetes; Piccovirales; Parvoviridae; Parvovirinae; Bocaparvovirus; Chiropteran bocaparvovirus 4
GCA_001858075.1	5414	5	Rhinolophus sinicus bocaparvovirus	str15	Viruses; Monodnaviria; Shotokuvirae; Cossaviricota; Quintoviricetes; Piccovirales; Parvoviridae; Parvovirinae; Bocaparvovirus; unclassified Bocaparvovirus
GCA_013087395.1	1130	1	Begomovirusassociated alphasatellite sp	NA	Viruses; Alphasatellitidae; Geminialphasatellitinae; unclassified Begomovirus-associated alphasatellites
GCA_001866915.1	10562	1	Riptortus pedestris virus1	NA	Viruses; ssRNA viruses; ssRNA positive-strand viruses, no DNA stage; Picornavirales; unclassified Picornavirales
GCA_002614485.1	123490	149	Klebsiella phage vB_Kpn_IME260	NA	Viruses; Duplodnaviria; Heunggongvirae; Uroviricota; Caudoviricetes; Caudovirales; Demerecviridae; Sugarlandvirus; Klebsiella virus IME260
GCA_002614505.1	44623	57	Klebsiella phage vB_KpnP_KpV48	NA	Viruses; Duplodnaviria; Heunggongvirae; Uroviricota; Caudoviricetes; Caudovirales; Autographiviridae; Slopekvirinae; Drulisvirus; Klebsiella virus KpV48
GCA_002614525.1	51099	79	Klebsiella phage vB_KpnS_KpV522	NA	Viruses; Duplodnaviria; Heunggongvirae; Uroviricota; Caudoviricetes; Caudovirales; Drexlerviridae; Webervirus; Klebsiella virus KpV522
GCA_002614545.1	47405	75	Klebsiella phage vB_KpnM_KpV52	NA	Viruses; Duplodnaviria; Heunggongvirae; Uroviricota; Caudoviricetes; Caudovirales; Myoviridae; Jedunavirus; Klebsiella virus KpV80
GCA_002633335.1	7079	11	Vibrio phage K04M1_VK04M1	NA	Viruses; Monodnaviria; Loebvirae; Hofneiviricota; Faserviricetes; Tubulavirales; Inoviridae; unclassified Inoviridae

gca	genome.size	proteome.size	phage.name	strain	Taxonomy
GCA_002633375.1	21012	34	Vibrio phage K05K4_VK05K4_1	NA	Viruses; Monodnaviria; Loebvirae; Hofneiviricota; Faserviricetes; Tubulavirales; Inoviridae; unclassified Inoviridae
GCA_002633405.1	13327	23	Vibrio phage K05K4_VK05K4_2	NA	Viruses; Monodnaviria; Loebvirae; Hofneiviricota; Faserviricetes; Tubulavirales; Inoviridae; unclassified Inoviridae
GCA_002954915.1	39902	71	Salmonella phage UPF_BP1	NA	Viruses; Duplodnaviria; Heunggongvirae; Uroviricota; Caudoviricetes; Caudovirales; Podoviridae; Lederbergvirus; unclassified Lederbergvirus
GCA_001879265.1	5857	8	Cherry associated luteovirus	NA	Viruses; ssRNA viruses; ssRNA positive-strand viruses, no DNA stage; Luteoviridae; unclassified Luteoviridae
GCA_001866975.1	8155	6	Equus caballus papillomavirus 8	NCSU	Viruses; dsDNA viruses, no RNA stage; Papillomaviridae; unclassified Papillomaviridae; non-primate mammal papillomaviruses
GCA_000820355.1	5886	3	Sclerophthora macrospora virus A	NA	Viruses; ssRNA viruses; ssRNA positive-strand viruses, no DNA stage; unclassified ssRNA positive-strand viruses
GCA_002614565.1	53432	91	Mycobacterium phage DarthPhader	NA	Viruses; Duplodnaviria; Heunggongvirae; Uroviricota; Caudoviricetes; Caudovirales; Siphoviridae; Fromanvirus; unclassified Fromanvirus
GCA_002614585.1	60526	97	Mycobacterium phage DrHayes	NA	Viruses; Duplodnaviria; Heunggongvirae; Uroviricota; Caudoviricetes; Caudovirales; Siphoviridae; Timquatrovirus; unclassified Timquatrovirus
GCA_002614605.1	60526	97	Mycobacterium phage SamuelLPlaqson	NA	Viruses; Duplodnaviria; Heunggongvirae; Uroviricota; Caudoviricetes; Caudovirales; Siphoviridae; Timquatrovirus; unclassified Timquatrovirus
GCA_002614635.1	60526	97	Mycobacterium phage Urkel	NA	Viruses; Duplodnaviria; Heunggongvirae; Uroviricota; Caudoviricetes; Caudovirales; Siphoviridae; Timquatrovirus; unclassified Timquatrovirus
GCA_001866835.1	6205	6	White clover mottle virus	CD	Viruses; ssRNA viruses; ssRNA positive-strand viruses, no DNA stage; Luteoviridae; Polerovirus; unclassified Polerovirus
GCA_002614665.1	48941	81	Streptomyces phage Joe	NA	Viruses; Duplodnaviria; Heunggongvirae; Uroviricota; Caudoviricetes; Caudovirales; Siphoviridae; Arquatrovirinae; Camvirus; unclassified Camvirus
GCA_002614685.1	56439	77	Mycobacterium phage Amelie	NA	Viruses; Duplodnaviria; Heunggongvirae; Uroviricota; Caudoviricetes; Caudovirales; Siphoviridae; Timquatrovirus; unclassified Timquatrovirus
GCA_002614705.1	51374	88	Mycobacterium phage Broseidon	NA	Viruses; Duplodnaviria; Heunggongvirae; Uroviricota; Caudoviricetes; Caudovirales; Siphoviridae; Fromanvirus; unclassified Fromanvirus
GCA_002614735.1	50883	88	Mycobacterium phage Sabinator	NA	Viruses; Duplodnaviria; Heunggongvirae; Uroviricota; Caudoviricetes; Caudovirales; Siphoviridae; Fromanvirus; unclassified Fromanvirus
GCA_002614765.1	59346	103	Mycobacterium phage SuperGrey	NA	Viruses; Duplodnaviria; Heunggongvirae; Uroviricota; Caudoviricetes; Caudovirales; Siphoviridae; Cheoctovirus; unclassified Cheoctovirus
GCA_002614785.1	158432	208	Salmonella phage S8	NA	Viruses; Duplodnaviria; Heunggongvirae; Uroviricota; Caudoviricetes; Caudovirales; Ackermannviridae; Cvivirinae; Kuttervirus; unclassified Kuttervirus
GCA_002954925.1	38749	58	Methylophilaceae phage P19250A	NA	Viruses; Duplodnaviria; Heunggongvirae; Uroviricota; Caudoviricetes; Caudovirales; Siphoviridae; unclassified Siphoviridae
GCA_002614805.1	51372	88	Mycobacterium phage Albee	NA	Viruses; Duplodnaviria; Heunggongvirae; Uroviricota; Caudoviricetes; Caudovirales; Siphoviridae; Fromanvirus; unclassified Fromanvirus
GCA_002614825.1	51373	88	Mycobacterium phage LittleB	NA	Viruses; Duplodnaviria; Heunggongvirae; Uroviricota; Caudoviricetes; Caudovirales; Siphoviridae; Fromanvirus; unclassified Fromanvirus
GCA_002614845.1	76944	148	Mycobacterium phage Tuco	NA	Viruses; Duplodnaviria; Heunggongvirae; Uroviricota; Caudoviricetes; Caudovirales; Siphoviridae; Kostyavirus; unclassified Kostyavirus
GCA_002614865.1	157034	221	Mycobacterium phage Lukilu	NA	Viruses; Duplodnaviria; Heunggongvirae; Uroviricota; Caudoviricetes; Caudovirales; Myoviridae; Bixzunavirus; Mycobacterium virus Lukilu
GCA_002614885.1	136203	242	Pseudoalteromonas phage PH357	NA	Viruses; Duplodnaviria; Heunggongvirae; Uroviricota; Caudoviricetes; Caudovirales; Myoviridae; unclassified Myoviridae
GCA_002614905.1	54894	70	Salmonella phage UPF_BP2	NA	Viruses; Duplodnaviria; Heunggongvirae; Uroviricota; Caudoviricetes; Caudovirales; Myoviridae; Rosemountvirus; unclassified Rosemountvirus
GCA_002614925.1	50552	82	Shigella phage SH6	NA	Viruses; Duplodnaviria; Heunggongvirae; Uroviricota; Caudoviricetes; Caudovirales; Drexlerviridae; Tunavirinae; Tunavirus; Shigella virus SH6
GCA_002614945.1	164870	265	Shigella phage SH7	NA	Viruses; Duplodnaviria; Heunggongvirae; Uroviricota; Caudoviricetes; Caudovirales; Myoviridae; Tevenvirinae; Tequatrovirus; unclassified Tequatrovirus
GCA_002614965.1	70176	111	Arthrobacter phage EdgarPoe	NA	Viruses; Duplodnaviria; Heunggongvirae; Uroviricota; Caudoviricetes; Caudovirales; Myoviridae; Radnorvirus; unclassified Radnorvirus
GCA_002614985.1	69978	111	Arthrobacter phage HumptyDumpty	NA	Viruses; Duplodnaviria; Heunggongvirae; Uroviricota; Caudoviricetes; Caudovirales; Myoviridae; Radnorvirus; unclassified Radnorvirus
GCA_002615005.1	35129	50	Streptococcus phage CHPC577	NA	Viruses; Duplodnaviria; Heunggongvirae; Uroviricota; Caudoviricetes; Caudovirales; Siphoviridae; unclassified Siphoviridae
GCA_002615025.1	30077	44	Streptococcus phage CHPC926	NA	Viruses; Duplodnaviria; Heunggongvirae; Uroviricota; Caudoviricetes; Caudovirales; Siphoviridae; unclassified Siphoviridae
GCA_002615045.1	33507	46	Streptococcus phage CHPC1151	NA	Viruses; Duplodnaviria; Heunggongvirae; Uroviricota; Caudoviricetes; Caudovirales; Siphoviridae; unclassified Siphoviridae
GCA_002615065.1	37270	69	Halomonas phage QHHSV1	NA	Viruses; Duplodnaviria; Heunggongvirae; Uroviricota; Caudoviricetes; Caudovirales; Siphoviridae; unclassified Siphoviridae
GCA_002615085.1	78145	106	Vibrio phage vB_VspP_pVa5	NA	Viruses; Duplodnaviria; Heunggongvirae; Uroviricota; Caudoviricetes; Caudovirales; Podoviridae; unclassified Podoviridae
GCA_002615105.1	52785	93	Mycobacterium phage StarStuff	NA	Viruses; Duplodnaviria; Heunggongvirae; Uroviricota; Caudoviricetes; Caudovirales; Siphoviridae; Fromanvirus; unclassified Fromanvirus

gca	genome.size	proteome.size	phage.name	strain	Taxonomy
GCA_002615125.1	35626	58	Pseudoalteromonas phage PHS3	NA	Viruses; Duplodnaviria; Heunggongvirae; Uroviricota; Caudoviricetes; Caudovirales; Siphoviridae; unclassified Siphoviridae
GCA_002615145.1	51606	73	Aeribacillus phage AP45	NA	Viruses; Duplodnaviria; Heunggongvirae; Uroviricota; Caudoviricetes; Caudovirales; Myoviridae; unclassified Myoviridae
GCA_002615165.1	37333	61	Alteromonas phage PB15	NA	Viruses; Duplodnaviria; Heunggongvirae; Uroviricota; Caudoviricetes; Caudovirales; Siphoviridae; unclassified Siphoviridae
GCA_002615185.1	53263	81	Salmonella phage vB_SenS_Sasha	NA	Viruses; Duplodnaviria; Heunggongvirae; Uroviricota; Caudoviricetes; Caudovirales; Siphoviridae; Sashavirus; Salmonella virus Sasha
GCA_002615205.1	56051	91	Salmonella phage vB_SenS_Sergei	NA	Viruses; Duplodnaviria; Heunggongvirae; Uroviricota; Caudoviricetes; Caudovirales; Siphoviridae; Sashavirus; unclassified Sashavirus
GCA_002615225.1	57155	104	Mycobacterium phage Empress	NA	Viruses; Duplodnaviria; Heunggongvirae; Uroviricota; Caudoviricetes; Caudovirales; Siphoviridae; Cheoctovirus; unclassified Cheoctovirus
GCA_002615245.1	65675	108	Erythrobacter phage vB_EliS_R6L	NA	Viruses; Duplodnaviria; Heunggongvirae; Uroviricota; Caudoviricetes; Caudovirales; Siphoviridae; unclassified Siphoviridae
GCA_003085815.1	155985	229	Bacillus phage BCP12	NA	Viruses; Duplodnaviria; Heunggongvirae; Uroviricota; Caudoviricetes; Caudovirales; Herelleviridae; Bastillevirinae; Tsarbombavirus; unclassified Tsarbombavirus
GCA_002080275.1	5845	7	Cowpea polerovirus 1	NA	Viruses; ssRNA viruses; ssRNA positive-strand viruses, no DNA stage; Luteoviridae; Polerovirus; unclassified Polerovirus
GCA_002080295.1	5945	7	Cowpea polerovirus 2	NA	Viruses; ssRNA viruses; ssRNA positive-strand viruses, no DNA stage; Luteoviridae; Polerovirus; unclassified Polerovirus
GCA_002615265.1	38122	58	Lactococcus phage PLgTB25	NA	Viruses; Duplodnaviria; Heunggongvirae; Uroviricota; Caudoviricetes; Caudovirales; Siphoviridae; unclassified Siphoviridae
GCA_002615285.1	41087	68	Staphylococcus phage SpT152	NA	Viruses; Duplodnaviria; Heunggongvirae; Uroviricota; Caudoviricetes; Caudovirales; Siphoviridae; unclassified Siphoviridae
GCA_002615305.1	40093	66	Staphylococcus phage SpT252	NA	Viruses; Duplodnaviria; Heunggongvirae; Uroviricota; Caudoviricetes; Caudovirales; Siphoviridae; unclassified Siphoviridae
GCA_002615325.1	39804	67	Staphylococcus phage SpT5	NA	Viruses; Duplodnaviria; Heunggongvirae; Uroviricota; Caudoviricetes; Caudovirales; Siphoviridae; unclassified Siphoviridae
GCA_002615345.1	40751	68	Staphylococcus phage SpT99F3	NA	Viruses; Duplodnaviria; Heunggongvirae; Uroviricota; Caudoviricetes; Caudovirales; Siphoviridae; Biseptimavirus; unclassified Biseptimavirus
GCA_002615365.1	66182	101	Pseudomonas phage PA5	NA	Viruses; Duplodnaviria; Heunggongvirae; Uroviricota; Caudoviricetes; Caudovirales; Myoviridae; Pbunavirus; Pseudomonas virus PA5
GCA_002615385.2	97177	166	Acinetobacter phage AM24	NA	Viruses; Duplodnaviria; Heunggongvirae; Uroviricota; Caudoviricetes; Caudovirales; Myoviridae; unclassified Myoviridae
GCA_002615405.1	167034	299	Klebsiella phage KPV15	NA	Viruses; Duplodnaviria; Heunggongvirae; Uroviricota; Caudoviricetes; Caudovirales; Myoviridae; Tevenvirinae; Jiaodavirus; unclassified Jiaodavirus
GCA_002615425.1	42641	43	Klebsiella phage KPV811	NA	Viruses; Duplodnaviria; Heunggongvirae; Uroviricota; Caudoviricetes; Caudovirales; Autographiviridae; Slopekvirinae; Drulisvirus; Klebsiella virus KPV811
GCA_002615445.1	91212	127	Pseudomonas phage PA10	NA	Viruses; Duplodnaviria; Heunggongvirae; Uroviricota; Caudoviricetes; Caudovirales; Myoviridae; Pakpunavirus; Pseudomonas virus PA10
GCA_002615465.1	18023	21	Staphylococcus phage SCH1	NA	Viruses; Duplodnaviria; Heunggongvirae; Uroviricota; Caudoviricetes; Caudovirales; Podoviridae; Rakietenvirinae; Rosenblumvirus; Staphylococcus virus SCH1
GCA_002615485.1	18018	22	Staphylococcus phage SCH111	NA	Viruses; Duplodnaviria; Heunggongvirae; Uroviricota; Caudoviricetes; Caudovirales; Podoviridae; Rakietenvirinae; Rosenblumvirus; unclassified Rosenblumvirus
GCA_002615505.1	112523	188	Streptomyces phage BRock	NA	Viruses; Duplodnaviria; Heunggongvirae; Uroviricota; Caudoviricetes; Caudovirales; Myoviridae; unclassified Myoviridae
GCA_002366125.1	7018	2	Nigrospora oryzae fusarivirus 1	NA	Viruses; Riboviria; unclassified Riboviria; unclassified ssRNA viruses; Fusariviridae
GCA_002615525.1	135502	192	Staphylococcus phage StAP1	NA	Viruses; Duplodnaviria; Heunggongvirae; Uroviricota; Caudoviricetes; Caudovirales; Herelleviridae; Twortvirinae; Silviavirus; unclassified Silviavirus
GCA_000853165.1	4554	3	Gremmeniella abietina RNA virus MS1	NA	Viruses; dsRNA viruses; Partitiviridae; Gammapartitivirus
GCA_003963775.1	2541	2	Human fecesassociated smacovirus	NA	Viruses; Monodnaviria; Shotokuvirae; Cressdnaviricota; Arfiviricetes; Cremevirales; Smacoviridae; Porprismacovirus; unclassified Porprismacovirus
GCA_002889895.1	6861	3	Rodent astrovirus	NA	Viruses; Riboviria; Orthornavirae; Pisuviricota; Stelpaviricetes; Stellavirales; Astroviridae; Mamastrovirus; unclassified Mamastrovirus
GCA_008704005.1	7419	3	Norovirus HuUSA2016GIV3WI7002	NA	Viruses; Riboviria; Orthornavirae; Pisuviricota; Pisoniviricetes; Picornavirales; Caliciviridae; Norovirus; Norwalk virus; Norovirus GIV; Norovirus GIV.3
GCA_013087405.1	8532	1	Potamipivirus A	NA	Viruses; Riboviria; Orthornavirae; Pisuviricota; Pisoniviricetes; Picornavirales; Picornaviridae; Potamipivirus
GCA_002615545.1	43428	70	Salmonella phage STP03	NA	Viruses; Duplodnaviria; Heunggongvirae; Uroviricota; Caudoviricetes; Caudovirales; Siphoviridae; Guernseyvirinae; Jerseyvirus; unclassified Jerseyvirus
GCA_003029435.1	2751	6	Synedrella yellow vein clearing virus	NA	Viruses; Monodnaviria; Shotokuvirae; Cressdnaviricota; Repensiviricetes; Geplafuvirales; Geminiviridae; Begomovirus
GCA_003029365.2	5235	7	Common bean mottle virus	NA	Viruses; Monodnaviria; Shotokuvirae; Cressdnaviricota; Repensiviricetes; Geplafuvirales; Geminiviridae; Begomovirus
GCA_002366145.2	5190	7	Common bean severe mosaic virus	NA	Viruses; Monodnaviria; Shotokuvirae; Cressdnaviricota; Repensiviricetes; Geplafuvirales; Geminiviridae; Begomovirus

gca	genome.size	proteome.size	phage.name	strain	Taxonomy
GCA_000860405.1	12024	2	Beet ringspot virus	S	Viruses; ssRNA viruses; ssRNA positive-strand viruses, no DNA stage; Picornavirales; Secoviridae; Comovirinae; Nepovirus; Subgroup B
GCA_002615565.1	59024	73	Escherichia phage Utah	NA	Viruses; Duplodnaviria; Heunggongvirae; Uroviricota; Caudoviricetes; Caudovirales; Siphoviridae; Chivirus; unclassified Chivirus
GCA_002615585.1	92338	171	Pseudomonas phage Zigelbrucke	NA	Viruses; Duplodnaviria; Heunggongvirae; Uroviricota; Caudoviricetes; Caudovirales; Myoviridae; Pakpunavirus; Pseudomonas virus Zigelbrucke
GCA_002615605.1	39790	50	Pectobacterium phage PP74	NA	Viruses; Duplodnaviria; Heunggongvirae; Uroviricota; Caudoviricetes; Caudovirales; Autographiviridae; Studiervirinae; Berlinvirus; Pectobacterium virus PP74
GCA_002615645.1	35209	46	Pseudoalteromonas phage C5a	NA	Viruses; Duplodnaviria; Heunggongvirae; Uroviricota; Caudoviricetes; Caudovirales; Myoviridae; Peduovirinae; Catalunyavirus; Pseudoalteromonas virus C5a
GCA_002615665.1	124261	228	Vibrio phage JSF4	NA	Viruses; Duplodnaviria; Heunggongvirae; Uroviricota; Caudoviricetes; Caudovirales; Myoviridae; unclassified Myoviridae
GCA_002615685.1	69396	112	Vibrio phage JSF3	NA	Viruses; Duplodnaviria; Heunggongvirae; Uroviricota; Caudoviricetes; Caudovirales; Podoviridae; unclassified Podoviridae
GCA_002615705.1	51381	88	Mycobacterium phage Abdiel	NA	Viruses; Duplodnaviria; Heunggongvirae; Uroviricota; Caudoviricetes; Caudovirales; Siphoviridae; Fromanvirus; unclassified Fromanvirus
GCA_002615725.1	51433	86	Mycobacterium phage Achebe	NA	Viruses; Duplodnaviria; Heunggongvirae; Uroviricota; Caudoviricetes; Caudovirales; Siphoviridae; Fromanvirus; unclassified Fromanvirus
GCA_002615745.1	51374	88	Mycobacterium phage Bruiser	NA	Viruses; Duplodnaviria; Heunggongvirae; Uroviricota; Caudoviricetes; Caudovirales; Siphoviridae; Fromanvirus; unclassified Fromanvirus
GCA_002615765.1	51386	88	Mycobacterium phage Eris	NA	Viruses; Duplodnaviria; Heunggongvirae; Uroviricota; Caudoviricetes; Caudovirales; Siphoviridae; Fromanvirus; unclassified Fromanvirus
GCA_002615785.1	51374	88	Mycobacterium phage Millski	NA	Viruses; Duplodnaviria; Heunggongvirae; Uroviricota; Caudoviricetes; Caudovirales; Siphoviridae; Fromanvirus; unclassified Fromanvirus
GCA_002615805.1	51369	88	Mycobacterium phage Palestino	NA	Viruses; Duplodnaviria; Heunggongvirae; Uroviricota; Caudoviricetes; Caudovirales; Siphoviridae; Fromanvirus; unclassified Fromanvirus
GCA_002615825.1	47079	75	Mycobacterium phage LeoAvram	NA	Viruses; Duplodnaviria; Heunggongvirae; Uroviricota; Caudoviricetes; Caudovirales; Siphoviridae; Fromanvirus; unclassified Fromanvirus
GCA_002615845.1	68398	99	Mycobacterium phage Prann	NA	Viruses; Duplodnaviria; Heunggongvirae; Uroviricota; Caudoviricetes; Caudovirales; Siphoviridae; Bclasvirinae; Pegunavirus; unclassified Pegunavirus
GCA_002954935.1	59779	79	Bordetella phage CN1	NA	Viruses; Duplodnaviria; Heunggongvirae; Uroviricota; Caudoviricetes; Caudovirales; Siphoviridae; Vojvodinavirus; Bordetella virus CN1
GCA_002954945.1	62030	81	Bordetella phage CN2	NA	Viruses; Duplodnaviria; Heunggongvirae; Uroviricota; Caudoviricetes; Caudovirales; Siphoviridae; Vojvodinavirus; Bordetella virus CN2
GCA_002954955.1	60332	80	Bordetella phage FP1	NA	Viruses; Duplodnaviria; Heunggongvirae; Uroviricota; Caudoviricetes; Caudovirales; Siphoviridae; Vojvodinavirus; Bordetella virus FP1
GCA_002954965.1	60160	80	Bordetella phage MW2	NA	Viruses; Duplodnaviria; Heunggongvirae; Uroviricota; Caudoviricetes; Caudovirales; Siphoviridae; Vojvodinavirus; Bordetella virus MW2
GCA_002615865.1	40493	51	Acinetobacter phage SHAb 15519	NA	Viruses; Duplodnaviria; Heunggongvirae; Uroviricota; Caudoviricetes; Caudovirales; Autographiviridae; Beijerinckvirinae; Friunavirus; Acinetobacter virus SH-Ab 15519
GCA_002615885.1	40648	63	Streptococcus phage IPP10	NA	Viruses; Duplodnaviria; Heunggongvirae; Uroviricota; Caudoviricetes; Caudovirales; Siphoviridae; unclassified Siphoviridae
GCA_002615905.1	37518	56	Streptococcus phage IPP11	NA	Viruses; Duplodnaviria; Heunggongvirae; Uroviricota; Caudoviricetes; Caudovirales; Siphoviridae; unclassified Siphoviridae
GCA_002615925.1	34428	57	Streptococcus phage IPP12	NA	Viruses; Duplodnaviria; Heunggongvirae; Uroviricota; Caudoviricetes; Caudovirales; Siphoviridae; unclassified Siphoviridae
GCA_002615945.1	37252	49	Streptococcus phage IPP14	NA	Viruses; Duplodnaviria; Heunggongvirae; Uroviricota; Caudoviricetes; Caudovirales; Siphoviridae; unclassified Siphoviridae
GCA_002615965.1	38083	55	Streptococcus phage IPP15	NA	Viruses; Duplodnaviria; Heunggongvirae; Uroviricota; Caudoviricetes; Caudovirales; Siphoviridae; unclassified Siphoviridae
GCA_002615985.1	34329	55	Streptococcus phage IPP16	NA	Viruses; Duplodnaviria; Heunggongvirae; Uroviricota; Caudoviricetes; Caudovirales; Siphoviridae; unclassified Siphoviridae
GCA_002616005.1	34864	49	Streptococcus phage IPP17	NA	Viruses; Duplodnaviria; Heunggongvirae; Uroviricota; Caudoviricetes; Caudovirales; Siphoviridae; unclassified Siphoviridae
GCA_002616025.1	34235	55	Streptococcus phage IPP18	NA	Viruses; Duplodnaviria; Heunggongvirae; Uroviricota; Caudoviricetes; Caudovirales; Siphoviridae; unclassified Siphoviridae
GCA_002616045.1	33895	48	Streptococcus phage IPP19	NA	Viruses; Duplodnaviria; Heunggongvirae; Uroviricota; Caudoviricetes; Caudovirales; Siphoviridae; unclassified Siphoviridae
GCA_002616065.1	37441	56	Streptococcus phage IPP20	NA	Viruses; Duplodnaviria; Heunggongvirae; Uroviricota; Caudoviricetes; Caudovirales; Siphoviridae; unclassified Siphoviridae
GCA_002616085.1	37192	56	Streptococcus phage IPP21	NA	Viruses; Duplodnaviria; Heunggongvirae; Uroviricota; Caudoviricetes; Caudovirales; Siphoviridae; unclassified Siphoviridae
GCA_002616105.1	36344	53	Streptococcus phage IPP22	NA	Viruses; Duplodnaviria; Heunggongvirae; Uroviricota; Caudoviricetes; Caudovirales; Siphoviridae; unclassified Siphoviridae
GCA_002616125.1	33903	54	Streptococcus phage IPP23	NA	Viruses; Duplodnaviria; Heunggongvirae; Uroviricota; Caudoviricetes; Caudovirales; Siphoviridae; unclassified Siphoviridae
GCA_002616145.1	34638	56	Streptococcus phage IPP24	NA	Viruses; Duplodnaviria; Heunggongvirae; Uroviricota; Caudoviricetes; Caudovirales; Siphoviridae; unclassified Siphoviridae

gca	genome.size	proteome.size	phage.name	strain	Taxonomy
GCA_002616165.1	33895	52	Streptococcus phage IPP25	NA	Viruses; Duplodnaviria; Heunggongvirae; Uroviricota; Caudoviricetes; Caudovirales; Siphoviridae; unclassified Siphoviridae
GCA_002616185.1	34001	51	Streptococcus phage IPP26	NA	Viruses; Duplodnaviria; Heunggongvirae; Uroviricota; Caudoviricetes; Caudovirales; Siphoviridae; unclassified Siphoviridae
GCA_002616205.1	32649	49	Streptococcus phage IPP27	NA	Viruses; Duplodnaviria; Heunggongvirae; Uroviricota; Caudoviricetes; Caudovirales; Siphoviridae; unclassified Siphoviridae
GCA_002616225.1	33045	52	Streptococcus phage IPP28	NA	Viruses; Duplodnaviria; Heunggongvirae; Uroviricota; Caudoviricetes; Caudovirales; Siphoviridae; unclassified Siphoviridae
GCA_002616245.1	33953	54	Streptococcus phage IPP29	NA	Viruses; Duplodnaviria; Heunggongvirae; Uroviricota; Caudoviricetes; Caudovirales; Siphoviridae; unclassified Siphoviridae
GCA_002616265.1	36844	54	Streptococcus phage IPP30	NA	Viruses; Duplodnaviria; Heunggongvirae; Uroviricota; Caudoviricetes; Caudovirales; Siphoviridae; unclassified Siphoviridae
GCA_002616285.1	39828	63	Streptococcus phage IPP31	NA	Viruses; Duplodnaviria; Heunggongvirae; Uroviricota; Caudoviricetes; Caudovirales; Siphoviridae; unclassified Siphoviridae
GCA_002616305.1	41421	64	Streptococcus phage IPP32	NA	Viruses; Duplodnaviria; Heunggongvirae; Uroviricota; Caudoviricetes; Caudovirales; Siphoviridae; unclassified Siphoviridae
GCA_002616325.1	38867	67	Streptococcus phage IPP34	NA	Viruses; Duplodnaviria; Heunggongvirae; Uroviricota; Caudoviricetes; Caudovirales; Siphoviridae; unclassified Siphoviridae
GCA_002616345.1	40164	68	Streptococcus phage IPP35	NA	Viruses; Duplodnaviria; Heunggongvirae; Uroviricota; Caudoviricetes; Caudovirales; Siphoviridae; unclassified Siphoviridae
GCA_002616365.1	40781	67	Streptococcus phage IPP36	NA	Viruses; Duplodnaviria; Heunggongvirae; Uroviricota; Caudoviricetes; Caudovirales; Siphoviridae; unclassified Siphoviridae
GCA_002616385.1	40403	63	Streptococcus phage IPP38	NA	Viruses; Duplodnaviria; Heunggongvirae; Uroviricota; Caudoviricetes; Caudovirales; Siphoviridae; unclassified Siphoviridae
GCA_002616405.1	37761	50	Streptococcus phage IPP39	NA	Viruses; Duplodnaviria; Heunggongvirae; Uroviricota; Caudoviricetes; Caudovirales; Siphoviridae; unclassified Siphoviridae
GCA_002616425.1	40449	65	Streptococcus phage IPP40	NA	Viruses; Duplodnaviria; Heunggongvirae; Uroviricota; Caudoviricetes; Caudovirales; Siphoviridae; unclassified Siphoviridae
GCA_002616445.1	34915	46	Streptococcus phage IPP41	NA	Viruses; Duplodnaviria; Heunggongvirae; Uroviricota; Caudoviricetes; Caudovirales; Siphoviridae; unclassified Siphoviridae
GCA_002616465.1	35061	43	Streptococcus phage IPP42	NA	Viruses; Duplodnaviria; Heunggongvirae; Uroviricota; Caudoviricetes; Caudovirales; Siphoviridae; unclassified Siphoviridae
GCA_002616485.1	33032	47	Streptococcus phage IPP43	NA	Viruses; Duplodnaviria; Heunggongvirae; Uroviricota; Caudoviricetes; Caudovirales; Siphoviridae; unclassified Siphoviridae
GCA_002616505.1	30572	45	Streptococcus phage IPP44	NA	Viruses; Duplodnaviria; Heunggongvirae; Uroviricota; Caudoviricetes; Caudovirales; Siphoviridae; unclassified Siphoviridae
GCA_002616525.1	37387	57	Streptococcus phage IPP45	NA	Viruses; Duplodnaviria; Heunggongvirae; Uroviricota; Caudoviricetes; Caudovirales; Siphoviridae; unclassified Siphoviridae
GCA_002616545.1	40232	67	Streptococcus phage IPP46	NA	Viruses; Duplodnaviria; Heunggongvirae; Uroviricota; Caudoviricetes; Caudovirales; Siphoviridae; unclassified Siphoviridae
GCA_002616565.1	37848	52	Streptococcus phage IPP48	NA	Viruses; Duplodnaviria; Heunggongvirae; Uroviricota; Caudoviricetes; Caudovirales; Siphoviridae; unclassified Siphoviridae
GCA_002616585.1	33495	47	Streptococcus phage IPP5	NA	Viruses; Duplodnaviria; Heunggongvirae; Uroviricota; Caudoviricetes; Caudovirales; Siphoviridae; unclassified Siphoviridae
GCA_002616605.1	40284	62	Streptococcus phage IPP50	NA	Viruses; Duplodnaviria; Heunggongvirae; Uroviricota; Caudoviricetes; Caudovirales; Siphoviridae; unclassified Siphoviridae
GCA_002616625.1	32793	48	Streptococcus phage IPP51	NA	Viruses; Duplodnaviria; Heunggongvirae; Uroviricota; Caudoviricetes; Caudovirales; Siphoviridae; unclassified Siphoviridae
GCA_002616645.1	36805	52	Streptococcus phage IPP52	NA	Viruses; Duplodnaviria; Heunggongvirae; Uroviricota; Caudoviricetes; Caudovirales; Siphoviridae; unclassified Siphoviridae
GCA_002616665.1	37235	55	Streptococcus phage IPP53	NA	Viruses; Duplodnaviria; Heunggongvirae; Uroviricota; Caudoviricetes; Caudovirales; Siphoviridae; unclassified Siphoviridae
GCA_002616685.1	38900	55	Streptococcus phage IPP54	NA	Viruses; Duplodnaviria; Heunggongvirae; Uroviricota; Caudoviricetes; Caudovirales; Siphoviridae; unclassified Siphoviridae
GCA_002616705.1	37847	48	Streptococcus phage IPP55	NA	Viruses; Duplodnaviria; Heunggongvirae; Uroviricota; Caudoviricetes; Caudovirales; Siphoviridae; unclassified Siphoviridae
GCA_002616725.1	33084	50	Streptococcus phage IPP57	NA	Viruses; Duplodnaviria; Heunggongvirae; Uroviricota; Caudoviricetes; Caudovirales; Siphoviridae; unclassified Siphoviridae
GCA_002616745.1	32027	50	Streptococcus phage IPP58	NA	Viruses; Duplodnaviria; Heunggongvirae; Uroviricota; Caudoviricetes; Caudovirales; Siphoviridae; unclassified Siphoviridae
GCA_002616765.1	41977	63	Streptococcus phage IPP60	NA	Viruses; Duplodnaviria; Heunggongvirae; Uroviricota; Caudoviricetes; Caudovirales; Siphoviridae; unclassified Siphoviridae
GCA_002616785.1	59870	82	Streptococcus phage IPP61	NA	Viruses; Duplodnaviria; Heunggongvirae; Uroviricota; Caudoviricetes; Caudovirales; Siphoviridae; unclassified Siphoviridae
GCA_002616805.1	39369	56	Streptococcus phage IPP62	NA	Viruses; Duplodnaviria; Heunggongvirae; Uroviricota; Caudoviricetes; Caudovirales; Siphoviridae; unclassified Siphoviridae
GCA_002616825.1	34280	52	Streptococcus phage IPP63	NA	Viruses; Duplodnaviria; Heunggongvirae; Uroviricota; Caudoviricetes; Caudovirales; Siphoviridae; unclassified Siphoviridae

gca	genome.size	proteome.size	phage.name	strain	Taxonomy
GCA_002616845.1	39689	67	Streptococcus phage IPP64	NA	Viruses; Duplodnaviria; Heunggongvirae; Uroviricota; Caudoviricetes; Caudovirales; Siphoviridae; unclassified Siphoviridae
GCA_002616865.1	39046	50	Streptococcus phage IPP65	NA	Viruses; Duplodnaviria; Heunggongvirae; Uroviricota; Caudoviricetes; Caudovirales; Siphoviridae; unclassified Siphoviridae
GCA_002616885.1	37969	53	Streptococcus phage IPP66	NA	Viruses; Duplodnaviria; Heunggongvirae; Uroviricota; Caudoviricetes; Caudovirales; Siphoviridae; unclassified Siphoviridae
GCA_002616905.1	33607	54	Streptococcus phage IPP67	NA	Viruses; Duplodnaviria; Heunggongvirae; Uroviricota; Caudoviricetes; Caudovirales; Siphoviridae; unclassified Siphoviridae
GCA_002616925.1	36203	54	Streptococcus phage IPP68	NA	Viruses; Duplodnaviria; Heunggongvirae; Uroviricota; Caudoviricetes; Caudovirales; Siphoviridae; unclassified Siphoviridae
GCA_002616945.1	40787	66	Streptococcus phage IPP69	NA	Viruses; Duplodnaviria; Heunggongvirae; Uroviricota; Caudoviricetes; Caudovirales; Siphoviridae; unclassified Siphoviridae
GCA_002616965.1	40080	60	Streptococcus phage IPP8	NA	Viruses; Duplodnaviria; Heunggongvirae; Uroviricota; Caudoviricetes; Caudovirales; Siphoviridae; unclassified Siphoviridae
GCA_002616985.1	41185	63	Streptococcus phage IPP9	NA	Viruses; Duplodnaviria; Heunggongvirae; Uroviricota; Caudoviricetes; Caudovirales; Siphoviridae; unclassified Siphoviridae
GCA_002617005.2	53333	79	Pectobacterium phage PP101	NA	Viruses; Duplodnaviria; Heunggongvirae; Uroviricota; Caudoviricetes; Caudovirales; Chaseviridae; Suwonvirus; Pectobacterium virus PP101
GCA_001866205.1	10025	1	BurkeGilman virus	RC1	Viruses; unclassified viruses
GCA_001866225.1	9070	2	Centovirus AC	NA	Viruses; Riboviria; Orthornavirae; Pisuviricota; Pisoniviricetes; Picornavirales; Dicistroviridae; unclassified Dicistroviridae; Centovirus
GCA_002819285.1	175360	172	Trichoplusia ni granulovirus LBIV12	NA	Viruses; Baculoviridae; Betabaculovirus; Trichoplusia ni granulovirus
GCA_002366225.2	5371	2	Bosavirus MS2016a	NA	Viruses; Monodnaviria; Shotokuvirae; Cossaviricota; Quintoviricetes; Piccovirales; Parvoviridae; unclassified Parvoviridae
GCA_004129515.1	6252	2	Sodiomyces alkalinus fusarivirus 1	NA	Viruses; Riboviria; unclassified Riboviria; unclassified ssRNA viruses; Fusariviridae
GCA_001887825.1	327147	357	Only Syngen Nebraska virus 5	NA	Viruses; dsDNA viruses, no RNA stage; Phycodnaviridae; Chlorovirus; unclassified Chlorovirus
GCA_014883435.1	8821	1	Picornaviridae sp rodentRLPicoVFJ2015	NA	Viruses; Riboviria; Orthornavirae; Pisuviricota; Pisoniviricetes; Picornavirales; Picornaviridae; Rosavirus; unclassified Rosavirus
GCA_008767635.1	7441	1	Picornaviridae sp rodentRnPicoVSX2015_2	NA	Viruses; Riboviria; Orthornavirae; Pisuviricota; Pisoniviricetes; Picornavirales; Picornaviridae; Hunnivirus; unclassified Hunnivirus
GCA_003029385.1	7805	1	Picornaviridae sp rodentEePicoVNX2015	NA	Viruses; Riboviria; Orthornavirae; Pisuviricota; Pisoniviricetes; Picornavirales; Picornaviridae; Enterovirus; Enterovirus K
GCA_008767615.1	7909	1	Picornaviridae sp rodentMcPicoVTibet2015	NA	Viruses; Riboviria; Orthornavirae; Pisuviricota; Pisoniviricetes; Picornavirales; Picornaviridae; Enterovirus; unclassified Enterovirus
GCA_013087415.1	7780	1	Picornaviridae sp rodentDsPicoVIM2014	NA	Viruses; Riboviria; Orthornavirae; Pisuviricota; Pisoniviricetes; Picornavirales; Picornaviridae; unclassified Picornaviridae
GCA_014883425.1	7823	1	Picornaviridae sp rodentCKPicoVTibet2014	NA	Viruses; Riboviria; Orthornavirae; Pisuviricota; Pisoniviricetes; Picornavirales; Picornaviridae; unclassified Picornaviridae
GCA_002617025.1	57895	94	Mycobacterium phage Mitti	NA	Viruses; Duplodnaviria; Heunggongvirae; Uroviricota; Caudoviricetes; Caudovirales; Siphoviridae; Timquatrovirus; unclassified Timquatrovirus
GCA_000899715.1	12094	2	Botrytis porri botybirnavirus 1	GarlicBc72	Viruses; Virus families not assigned to an order; Botybirnavirus
GCA_001891015.1	2757	5	Sugarcane chlorotic streak virus	NA	Viruses; ssDNA viruses; Geminiviridae; Mastrevirus; unclassified Mastrevirus
GCA_004129935.1	10744	6	Menghai rhabdovirus	NA	Viruses; Riboviria; Orthornavirae; Negarnaviricota; Haploviricotina; Monjiviricetes; Mononegavirales; Rhabdoviridae; Almendravirus; Menghai almendravirus
GCA_001963235.1	224064	173	Cynomolgus cytomegalovirus	NA	Viruses; dsDNA viruses, no RNA stage; Herpesvirales; Herpesviridae; Betaherpesvirinae; Cytomegalovirus; unclassified Cytomegalovirus
GCA_001891055.1	5108	6	Rattus norvegicus polyomavirus 2	NA	Viruses; dsDNA viruses, no RNA stage; Polyomaviridae; unclassified Polyomaviridae
GCA_000852985.1	1165	1	Chicory yellow mottle virus large satellite RNA	NA	Viruses; Satellites; Satellite Nucleic Acids; Single stranded RNA satellites; unclassified Single stranded RNA satellites
GCA_000854685.1	457	1	Chicory yellow mottle virus satellite RNA	NA	Viruses; Satellites; Satellite Nucleic Acids; Single stranded RNA satellites; unclassified Single stranded RNA satellites
GCA_000854005.1	618	1	Cymbidium ringspot virus satellite RNA	NA	Viruses; Satellites; Satellite Nucleic Acids; Single stranded RNA satellites; Small linear single stranded RNA satellites
GCA_002743495.1	117842	156	Salmonella phage SP01	NA	Viruses; Duplodnaviria; Heunggongvirae; Uroviricota; Caudoviricetes; Caudovirales; Demerecviridae; Markadamsvirinae; Tequintavirus; Salmonella virus SP01
GCA_002617045.1	61812	94	Mycobacterium phage Hammy	NA	Viruses; Duplodnaviria; Heunggongvirae; Uroviricota; Caudoviricetes; Caudovirales; Siphoviridae; Timquatrovirus; unclassified Timquatrovirus
GCA_002617065.1	60973	92	Mycobacterium phage Taptic	NA	Viruses; Duplodnaviria; Heunggongvirae; Uroviricota; Caudoviricetes; Caudovirales; Siphoviridae; unclassified Siphoviridae
GCA_002617085.1	37830	54	Streptomyces phage Bioscum	NA	Viruses; Duplodnaviria; Heunggongvirae; Uroviricota; Caudoviricetes; Caudovirales; Siphoviridae; Austintatiousvirus; unclassified Austintatiousvirus

gca	genome.size	proteome.size	phage.name	strain	Taxonomy
GCA_002617105.1	37817	55	Streptomyces phage Ididsumtinwong	NA	Viruses; Duplodnaviria; Heunggongvirae; Uroviricota; Caudoviricetes; Caudovirales; Siphoviridae; Austintatiousvirus; Streptomyces virus Ididsumtinwong
GCA_002617125.1	38496	56	Streptomyces phage Mojorita	NA	Viruses; Duplodnaviria; Heunggongvirae; Uroviricota; Caudoviricetes; Caudovirales; Siphoviridae; Picardvirus; unclassified Picardvirus
GCA_002617145.1	38411	54	Streptomyces phage PapayaSalad	NA	Viruses; Duplodnaviria; Heunggongvirae; Uroviricota; Caudoviricetes; Caudovirales; Siphoviridae; Austintatiousvirus; Streptomyces virus PapayaSalad
GCA_002617165.1	39522	56	Streptomyces phage Picard	NA	Viruses; Duplodnaviria; Heunggongvirae; Uroviricota; Caudoviricetes; Caudovirales; Siphoviridae; Picardvirus; Streptomyces virus Picard
GCA_002617185.1	40785	53	Streptomyces phage Raleigh	NA	Viruses; Duplodnaviria; Heunggongvirae; Uroviricota; Caudoviricetes; Caudovirales; Siphoviridae; Raleighvirus; Streptomyces virus Raleigh
GCA_002617205.1	41446	64	Brucella phage Iz	NA	Viruses; Duplodnaviria; Heunggongvirae; Uroviricota; Caudoviricetes; Caudovirales; Podoviridae; Perisivirus; unclassified Perisivirus
GCA_002617225.1	45985	67	Staphylococcus phage P240	NA	Viruses; Duplodnaviria; Heunggongvirae; Uroviricota; Caudoviricetes; Caudovirales; Siphoviridae; Triavirus; unclassified Triavirus
GCA_002633435.1	99517	52	Azobacteroides phage ProJPtBp1	NA	Viruses; Duplodnaviria; Heunggongvirae; Uroviricota; Caudoviricetes; Caudovirales; Podoviridae; unclassified Podoviridae; crAss-like viruses
GCA_001904925.1	10623	1	Euscelidius variegatus virus 1	NA	Viruses; ssRNA viruses; ssRNA positive-strand viruses, no DNA stage; Picornavirales; Iflaviridae; unclassified Iflaviridae
GCA_001904885.1	28363	8	NL63related bat coronavirus	BtKYNL639a	Viruses; ssRNA viruses; ssRNA positive-strand viruses, no DNA stage; Nidovirales; Coronaviridae; Coronavirinae; Alphacoronavirus; unclassified Alphacoronavirus
GCA_000921435.1	9388	7	Gentian ovary ringspot virus	NA	Viruses; ssRNA viruses; ssRNA positive-strand viruses, no DNA stage; Virgaviridae; Goravirus
GCA_002116275.1	10493	1	Diabrotica virgifera virgifera virus 2	NA	Viruses; unclassified viruses
GCA_002080155.1	13202	2	Diabrotica virgifera virgifera virus 3	NA	Viruses; unclassified viruses
GCA_003729055.1	9823	1	Diabrotica virgifera virgifera virus 1	NA	Viruses; unclassified viruses
GCA_000865785.1	46072	82	Escherichia virus Jk06	NA	Viruses; Duplodnaviria; Heunggongvirae; Uroviricota; Caudoviricetes; Caudovirales; Drexlerviridae; Rogunavirinae; Rogunavirus
GCA_002743515.1	47285	78	Escherichia virus KP26	NA	Viruses; dsDNA viruses, no RNA stage; Caudovirales; Siphoviridae; Tunavirinae; Rogue1virus
GCA_000845005.1	48836	78	Escherichia virus T1	NA	Viruses; dsDNA viruses, no RNA stage; Caudovirales; Siphoviridae; Tunavirinae; T1virus
GCA_001904945.1	7599	5	Arachis pintoi virus	NA	Viruses; ssRNA viruses; ssRNA positive-strand viruses, no DNA stage; Tymovirales; Alphaflexiviridae; unclassified Alphaflexiviridae
GCA_004787835.1	11599	6	Caligus rogercresseyi rhabdovirus	CrRVCh01	Viruses; Riboviria; Orthornavirae; Negarnaviricota; Haploviricotina; Monjiviricetes; Mononegavirales; Rhabdoviridae; Caligrhavirus; Caligus caligrhavirus
GCA_001904905.1	4580	2	Saccharomyces kudriavzevii virus LA1	NA	Viruses; dsRNA viruses; Totiviridae; unclassified Totiviridae
GCA_002118585.1	12259	4	Caraparu virus	BeAn3994	Viruses; Riboviria; Orthornavirae; Negarnaviricota; Polyploviricotina; Ellioviricetes; Bunyavirales; Peribunyaviridae; Orthobunyavirus; Caraparu orthobunyavirus
GCA_002118565.1	12259	4	Oriboca virus	BeAn17	Viruses; Riboviria; Orthornavirae; Negarnaviricota; Polyploviricotina; Ellioviricetes; Bunyavirales; Peribunyaviridae; Orthobunyavirus; Oriboca orthobunyavirus
GCA_000879975.1	5920	6	Tobacco vein distorting virus	NA	Viruses; ssRNA viruses; ssRNA positive-strand viruses, no DNA stage; Luteoviridae; Polerovirus
GCA_003033125.1	7688	8	Giraffa camelopardalis papillomavirus 1	NA	Viruses; Monodnaviria; Shotokuvirae; Cossaviricota; Papovaviricetes; Zurhausenvirales; Papillomaviridae; Firstpapillomavirinae; Deltapapillomavirus; Deltapapillomavirus 7
GCA_001926795.1	24648	9	Beihai Nidolike virus 2	BHXun32263	Viruses; unclassified RNA viruses; unclassified RNA viruses ShiM-2016
GCA_001925395.1	11540	6	Beihai anemone virus 1	BHHK129576	Viruses; unclassified RNA viruses; unclassified RNA viruses ShiM-2016
GCA_001925295.1	6856	3	Beihai astrolike virus	BHWZXX13371	Viruses; unclassified RNA viruses; unclassified RNA viruses ShiM-2016
GCA_001925435.1	2953	1	Beihai barnacle virus 10	BHTH18714	Viruses; unclassified RNA viruses; unclassified RNA viruses ShiM-2016
GCA_001926775.1	4490	2	Beihai barnacle virus 11	BHTH10280	Viruses; unclassified RNA viruses; unclassified RNA viruses ShiM-2016
GCA_001925375.1	3277	2	Beihai barnacle virus 12	BHTH16091	Viruses; unclassified RNA viruses; unclassified RNA viruses ShiM-2016
GCA_001921375.1	2960	2	Beihai barnacle virus 13	BHTH16173	Viruses; unclassified RNA viruses; unclassified RNA viruses ShiM-2016
GCA_001925275.1	7382	2	Beihai barnacle virus 15	BHTH16139	Viruses; unclassified RNA viruses; unclassified RNA viruses ShiM-2016
GCA_001925415.1	9154	4	Beihai barnacle virus 2	BHTH16123	Viruses; unclassified RNA viruses; unclassified RNA viruses ShiM-2016
GCA_001926755.1	11035	5	Beihai barnacle virus 3	BHTH16145	Viruses; unclassified RNA viruses; unclassified RNA viruses ShiM-2016

gca	genome.size	proteome.size	phage.name	strain	Taxonomy
GCA_001925715.1	7612	1	Beihai barnacle virus 4	BHTH16136	Viruses; unclassified RNA viruses; unclassified RNA viruses ShiM-2016
GCA_001926475.1	11112	6	Beihai barnacle virus 7	BHTH16013	Viruses; unclassified RNA viruses; unclassified RNA viruses ShiM-2016
GCA_001926975.1	12395	4	Beihai barnacle virus 8	BHTH14652	Viruses; unclassified RNA viruses; unclassified RNA viruses ShiM-2016
GCA_001926135.1	11058	3	Beihai barnacle virus 9	BHTH10927	Viruses; unclassified RNA viruses; unclassified RNA viruses ShiM-2016
GCA_001925695.1	12469	1	Beihai blue swimmer crab virus 1	YYSZX13554	Viruses; unclassified RNA viruses; unclassified RNA viruses ShiM-2016
GCA_001926455.1	4998	2	Beihai blue swimmer crab virus 3	YYSZX17757	Viruses; unclassified RNA viruses; unclassified RNA viruses ShiM-2016
GCA_001926955.1	6969	2	Beihai charybdis crab virus 1	BHXun33339	Viruses; unclassified RNA viruses; unclassified RNA viruses ShiM-2016
GCA_001926115.1	6589	1	Wenling chuviruslike virus 1	WLJQ101487	Viruses; unclassified RNA viruses; unclassified RNA viruses ShiM-2016
GCA_001925675.1	6877	1	Wenling chuviruslike virus 2	WLJQ104274	Viruses; unclassified RNA viruses; unclassified RNA viruses ShiM-2016
GCA_001926435.1	8175	1	Beihai echinoderm virus 1	BHJP42036	Viruses; unclassified RNA viruses; unclassified RNA viruses ShiM-2016
GCA_001926935.1	10817	5	Beihai hepelike virus 10	BHZY60406	Viruses; unclassified RNA viruses; unclassified RNA viruses ShiM-2016
GCA_001926095.1	10084	3	Beihai hepelike virus 8	BHZY60925	Viruses; unclassified RNA viruses; unclassified RNA viruses ShiM-2016
GCA_001925655.1	10128	5	Beihai hepelike virus 9	HOU157038	Viruses; unclassified RNA viruses; unclassified RNA viruses ShiM-2016
GCA_001921475.1	12648	4	Beihai hepelike virus 4	BHZY60842	Viruses; unclassified RNA viruses; unclassified RNA viruses ShiM-2016
GCA_001926415.1	9888	2	Beihai hermit crab virus 1	BHWZXX15637	Viruses; unclassified RNA viruses; unclassified RNA viruses ShiM-2016
GCA_001926915.1	12996	4	Beihai hermit crab virus 3	BHJJX21702	Viruses; unclassified RNA viruses; unclassified RNA viruses ShiM-2016
GCA_001925635.1	5163	3	Beihai horseshoe crab virus 1	HOU157708	Viruses; unclassified RNA viruses; unclassified RNA viruses ShiM-2016
GCA_001925555.1	9925	1	Beihai hypolike virus 1	BHZC36965	Viruses; dsRNA viruses; Hypoviridae; unclassified Hypoviridae
GCA_001926895.1	10973	6	Beihai mantis shrimp virus 1	BHXG26050	Viruses; unclassified RNA viruses; unclassified RNA viruses ShiM-2016
GCA_001926055.1	11959	5	Beihai mantis shrimp virus 2	BHXG46195	Viruses; unclassified RNA viruses; unclassified RNA viruses ShiM-2016
GCA_001925615.1	9599	1	Beihai mantis shrimp virus 3	BHXG26678	Viruses; unclassified RNA viruses; unclassified RNA viruses ShiM-2016
GCA_001925535.1	10177	2	Beihai mantis shrimp virus 4	BHXG23944	Viruses; unclassified RNA viruses; unclassified RNA viruses ShiM-2016
GCA_001926875.1	10256	2	Beihai mantis shrimp virus 5	BHXG25299	Viruses; unclassified RNA viruses; unclassified RNA viruses ShiM-2016
GCA_001926035.1	4653	4	Beihai mantis shrimp virus 6	BHXG24422	Viruses; unclassified RNA viruses; unclassified RNA viruses ShiM-2016
GCA_001921455.1	8805	2	Beihai mollusks virus 1	WZSLuoI86140	Viruses; unclassified RNA viruses; unclassified RNA viruses ShiM-2016
GCA_001925595.1	9054	2	Beihai mollusks virus 2	WZSLuoI86113	Viruses; unclassified RNA viruses; unclassified RNA viruses ShiM-2016
GCA_001925515.1	2584	1	Beihai narnalike virus 1	BWBFG61141	Viruses; unclassified RNA viruses; unclassified RNA viruses ShiM-2016
GCA_001926855.1	4282	2	Beihai narnalike virus 10	BHZY60512	Viruses; unclassified RNA viruses; unclassified RNA viruses ShiM-2016
GCA_001926015.1	3950	2	Beihai narnalike virus 11	BWBFG39775	Viruses; unclassified RNA viruses; unclassified RNA viruses ShiM-2016
GCA_001925575.1	2544	1	Beihai narnalike virus 12	BHZC35702	Viruses; unclassified RNA viruses; unclassified RNA viruses ShiM-2016
GCA_001925495.1	2785	1	Beihai narnalike virus 13	BHWZXX14912	Viruses; unclassified RNA viruses; unclassified RNA viruses ShiM-2016
GCA_001926295.1	3003	1	Beihai narnalike virus 14	BHJP52694	Viruses; unclassified RNA viruses; unclassified RNA viruses ShiM-2016
GCA_001925855.1	2962	1	Beihai narnalike virus 15	BHHZL10358	Viruses; unclassified RNA viruses; unclassified RNA viruses ShiM-2016
GCA_001926615.1	2417	1	Beihai narnalike virus 16	BHJP26864	Viruses; unclassified RNA viruses; unclassified RNA viruses ShiM-2016

gca	genome.size	proteome.size	phage.name	strain	Taxonomy
GCA_001927115.1	4527	1	Beihai narnalike virus 17	SCJXSX39297	Viruses; unclassified RNA viruses; unclassified RNA viruses ShiM-2016
GCA_001926275.1	3761	1	Beihai narnalike virus 18	HOU157597	Viruses; unclassified RNA viruses; unclassified RNA viruses ShiM-2016
GCA_001925835.1	4198	1	Beihai narnalike virus 19	BHNXC41285	Viruses; unclassified RNA viruses; unclassified RNA viruses ShiM-2016
GCA_001926595.1	2624	1	Beihai narnalike virus 2	BHZY58217	Viruses; unclassified RNA viruses; unclassified RNA viruses ShiM-2016
GCA_001927095.1	3790	1	Beihai narnalike virus 20	BWBFG40664	Viruses; unclassified RNA viruses; unclassified RNA viruses ShiM-2016
GCA_001926255.1	3537	1	Beihai narnalike virus 21	SCJXSX34167	Viruses; unclassified RNA viruses; unclassified RNA viruses ShiM-2016
GCA_001925355.1	3244	1	Beihai narnalike virus 22	BHBJDX18651	Viruses; unclassified RNA viruses; unclassified RNA viruses ShiM-2016
GCA_001925815.1	3610	2	Beihai narnalike virus 23	BHZY57139	Viruses; unclassified RNA viruses; unclassified RNA viruses ShiM-2016
GCA_001926575.1	2677	2	Beihai narnalike virus 24	BHXG26712	Viruses; unclassified RNA viruses; unclassified RNA viruses ShiM-2016
GCA_001927075.1	2317	1	Beihai narnalike virus 26	BHHQ66335	Viruses; unclassified RNA viruses; unclassified RNA viruses ShiM-2016
GCA_001926235.1	2816	1	Beihai narnalike virus 3	BHBJDX18174	Viruses; unclassified RNA viruses; unclassified RNA viruses ShiM-2016
GCA_001925795.1	2687	1	Beihai narnalike virus 4	BHZY53599	Viruses; unclassified RNA viruses; unclassified RNA viruses ShiM-2016
GCA_001926555.1	2881	1	Beihai narnalike virus 6	BHZC37402	Viruses; unclassified RNA viruses; unclassified RNA viruses ShiM-2016
GCA_001927055.1	3906	2	Beihai narnalike virus 8	BHZY59840	Viruses; unclassified RNA viruses; unclassified RNA viruses ShiM-2016
GCA_001926215.1	4035	2	Beihai narnalike virus 9	HOU146315	Viruses; unclassified RNA viruses; unclassified RNA viruses ShiM-2016
GCA_001925775.1	4369	2	Beihai nodalike virus 11	BHJJX49550	Viruses; unclassified RNA viruses; unclassified RNA viruses ShiM-2016
GCA_001923575.1	3220	1	Beihai nodalike virus 18	YYSZX13070	Viruses; unclassified RNA viruses; unclassified RNA viruses ShiM-2016
GCA_001924455.1	3168	1	Beihai nodalike virus 29	BHWZXX15622	Viruses; unclassified RNA viruses; unclassified RNA viruses ShiM-2016
GCA_001924915.1	4491	2	Beihai nodalike virus 5	HOU157766	Viruses; unclassified RNA viruses; unclassified RNA viruses ShiM-2016
GCA_001926535.1	9211	2	Beihai octopus virus 1	BHZY180716	Viruses; unclassified RNA viruses; unclassified RNA viruses ShiM-2016
GCA_001927035.1	9973	1	Beihai octopus virus 2	BHZY60909	Viruses; unclassified RNA viruses; unclassified RNA viruses ShiM-2016
GCA_001926195.1	9974	2	Beihai paphia shell virus 1	BHZY60696	Viruses; unclassified RNA viruses; unclassified RNA viruses ShiM-2016
GCA_001925755.1	9528	2	Beihai paphia shell virus 2	YYSZX17625	Viruses; unclassified RNA viruses; unclassified RNA viruses ShiM-2016
GCA_001926515.1	9521	1	Beihai paphia shell virus 3	BWBFG40853	Viruses; unclassified RNA viruses; unclassified RNA viruses ShiM-2016
GCA_001927015.1	9840	1	Beihai paphia shell virus 4	BWBFG40859	Viruses; unclassified RNA viruses; unclassified RNA viruses ShiM-2016
GCA_001926175.1	3266	2	Beihai partitilike virus 2	BHZY60797	Viruses; unclassified RNA viruses; unclassified RNA viruses ShiM-2016
GCA_001921275.1	4208	2	Beihai permutotetralike virus 2	HOU234589	Viruses; unclassified RNA viruses; unclassified RNA viruses ShiM-2016
GCA_001925735.1	3597	2	Beihai picobirnalike virus 7	BHNXC57916	Viruses; unclassified RNA viruses; unclassified RNA viruses ShiM-2016
GCA_001926495.1	3458	2	Beihai picobirnalike virus 8	BHNXC71065	Viruses; unclassified RNA viruses; unclassified RNA viruses ShiM-2016
GCA_001935065.1	9149	2	Beihai picornalike virus 1	BHZC36988	Viruses; unclassified RNA viruses; unclassified RNA viruses ShiM-2016
GCA_001935705.1	9895	2	Beihai picornalike virus 100	BHNXC41148	Viruses; unclassified RNA viruses; unclassified RNA viruses ShiM-2016
GCA_001935285.1	7349	2	Beihai picornalike virus 101	BHBJDX18559	Viruses; unclassified RNA viruses; unclassified RNA viruses ShiM-2016
GCA_001935645.1	7273	1	Beihai picornalike virus 102	BHZC35144	Viruses; unclassified RNA viruses; unclassified RNA viruses ShiM-2016
GCA_001923535.1	6313	1	Beihai picornalike virus 103	BHBei77089	Viruses; unclassified RNA viruses; unclassified RNA viruses ShiM-2016

gca	genome.size	proteome.size	phage.name	strain	Taxonomy
GCA_001934125.1	6729	1	Beihai picornalike virus 104	BHZC37407	Viruses; unclassified RNA viruses; unclassified RNA viruses ShiM-2016
GCA_001934485.1	8854	2	Beihai picornalike virus 105	BHHK79817	Viruses; unclassified RNA viruses; unclassified RNA viruses ShiM-2016
GCA_001934985.1	9396	1	Beihai picornalike virus 106	BHZY60875	Viruses; unclassified RNA viruses; unclassified RNA viruses ShiM-2016
GCA_001935625.1	8626	1	Beihai picornalike virus 107	BHZY60580	Viruses; unclassified RNA viruses; unclassified RNA viruses ShiM-2016
GCA_001934105.1	13842	1	Beihai picornalike virus 108	BHTH16067	Viruses; unclassified RNA viruses; unclassified RNA viruses ShiM-2016
GCA_001934465.1	9245	2	Beihai picornalike virus 11	BHZY60905	Viruses; unclassified RNA viruses; unclassified RNA viruses ShiM-2016
GCA_001934965.1	9780	1	Beihai picornalike virus 110	BHXG26677	Viruses; unclassified RNA viruses; unclassified RNA viruses ShiM-2016
GCA_001934905.1	9484	1	Beihai picornalike virus 111	BHBei77071	Viruses; unclassified RNA viruses; unclassified RNA viruses ShiM-2016
GCA_001934085.1	10676	2	Beihai picornalike virus 112	BHXG26692	Viruses; unclassified RNA viruses; unclassified RNA viruses ShiM-2016
GCA_001934445.1	10929	2	Beihai picornalike virus 113	BHXG26415	Viruses; unclassified RNA viruses; unclassified RNA viruses ShiM-2016
GCA_001934945.1	10342	2	Beihai picornalike virus 114	BHZY60932	Viruses; unclassified RNA viruses; unclassified RNA viruses ShiM-2016
GCA_001934885.1	10456	2	Beihai picornalike virus 115	BHHK131520	Viruses; unclassified RNA viruses; unclassified RNA viruses ShiM-2016
GCA_001934065.1	10897	2	Beihai picornalike virus 116	YYSZX16386	Viruses; unclassified RNA viruses; unclassified RNA viruses ShiM-2016
GCA_001934425.1	9450	3	Beihai picornalike virus 117	BHJP63029	Viruses; unclassified RNA viruses; unclassified RNA viruses ShiM-2016
GCA_001934925.1	9029	3	Beihai picornalike virus 118	BHHK118641	Viruses; unclassified RNA viruses; unclassified RNA viruses ShiM-2016
GCA_001934865.1	10859	2	Beihai picornalike virus 119	BHJP60437	Viruses; unclassified RNA viruses; unclassified RNA viruses ShiM-2016
GCA_001934045.1	9928	4	Beihai picornalike virus 120	BHSC167783	Viruses; unclassified RNA viruses; unclassified RNA viruses ShiM-2016
GCA_001934405.1	8621	2	Beihai picornalike virus 121	BHHZL10364	Viruses; unclassified RNA viruses; unclassified RNA viruses ShiM-2016
GCA_001933765.1	7981	1	Beihai picornalike virus 122	BHZY60922	Viruses; unclassified RNA viruses; unclassified RNA viruses ShiM-2016
GCA_001934845.1	10608	1	Beihai picornalike virus 123	BHCL81476	Viruses; unclassified RNA viruses; unclassified RNA viruses ShiM-2016
GCA_001934025.1	8221	2	Beihai picornalike virus 124	BHBJDX18598	Viruses; unclassified RNA viruses; unclassified RNA viruses ShiM-2016
GCA_001934385.1	8546	1	Beihai picornalike virus 125	BHZY54174	Viruses; unclassified RNA viruses; unclassified RNA viruses ShiM-2016
GCA_001933745.1	9234	2	Beihai picornalike virus 14	BHHK130444	Viruses; unclassified RNA viruses; unclassified RNA viruses ShiM-2016
GCA_001923995.1	9110	2	Beihai picornalike virus 15	BHZY59344	Viruses; unclassified RNA viruses; unclassified RNA viruses ShiM-2016
GCA_001934825.1	8466	1	Beihai picornalike virus 16	BHHK123579	Viruses; unclassified RNA viruses; unclassified RNA viruses ShiM-2016
GCA_001934005.1	8733	2	Beihai picornalike virus 17	BHZY55665	Viruses; unclassified RNA viruses; unclassified RNA viruses ShiM-2016
GCA_001934365.1	9186	2	Beihai picornalike virus 18	BHSC157282	Viruses; unclassified RNA viruses; unclassified RNA viruses ShiM-2016
GCA_001933725.1	8839	2	Beihai picornalike virus 19	BHJJX24523	Viruses; unclassified RNA viruses; unclassified RNA viruses ShiM-2016
GCA_001934805.1	9983	2	Beihai picornalike virus 2	BHNXC40556	Viruses; unclassified RNA viruses; unclassified RNA viruses ShiM-2016
GCA_001933985.1	10091	2	Beihai picornalike virus 20	BHJJX25607	Viruses; unclassified RNA viruses; unclassified RNA viruses ShiM-2016
GCA_001935825.1	8817	2	Beihai picornalike virus 21	BHZY60822	Viruses; unclassified RNA viruses; unclassified RNA viruses ShiM-2016
GCA_001935405.1	9176	2	Beihai picornalike virus 23	YYSZX17154	Viruses; unclassified RNA viruses; unclassified RNA viruses ShiM-2016
GCA_001935545.1	9665	2	Beihai picornalike virus 24	BHZY60459	Viruses; unclassified RNA viruses; unclassified RNA viruses ShiM-2016
GCA_002149785.1	9864	2	Beihai picornalike virus 26	BZL93356	Viruses; unclassified RNA viruses; unclassified RNA viruses ShiM-2016

gca	genome.size	proteome.size	phage.name	strain	Taxonomy
GCA_001935165.1	9410	2	Beihai picornalike virus 27	BHNXC41439	Viruses; unclassified RNA viruses; unclassified RNA viruses ShiM-2016
GCA_001935805.1	9559	2	Beihai picornalike virus 28	BHZY60695	Viruses; unclassified RNA viruses; unclassified RNA viruses ShiM-2016
GCA_001935385.1	9460	2	Beihai picornalike virus 30	BHNXC41477	Viruses; unclassified RNA viruses; unclassified RNA viruses ShiM-2016
GCA_001935525.1	6040	1	Beihai picornalike virus 32	BHNXC41402	Viruses; unclassified RNA viruses; unclassified RNA viruses ShiM-2016
GCA_001935145.1	8887	2	Beihai picornalike virus 33	BWBFG40845	Viruses; unclassified RNA viruses; unclassified RNA viruses ShiM-2016
GCA_001924435.1	8955	2	Beihai picornalike virus 35	BHZC37213	Viruses; unclassified RNA viruses; unclassified RNA viruses ShiM-2016
GCA_001935785.1	9206	2	Beihai picornalike virus 39	BHSC164089	Viruses; unclassified RNA viruses; unclassified RNA viruses ShiM-2016
GCA_001935365.1	9376	2	Beihai picornalike virus 4	BHZY60671	Viruses; unclassified RNA viruses; unclassified RNA viruses ShiM-2016
GCA_001935505.1	6214	1	Beihai picornalike virus 40	BHNXC39087	Viruses; unclassified RNA viruses; unclassified RNA viruses ShiM-2016
GCA_001935125.1	9261	2	Beihai picornalike virus 41	BHJJX25957	Viruses; unclassified RNA viruses; unclassified RNA viruses ShiM-2016
GCA_001935765.1	9166	2	Beihai picornalike virus 42	SCJXSX39263	Viruses; unclassified RNA viruses; unclassified RNA viruses ShiM-2016
GCA_001924895.1	8943	2	Beihai picornalike virus 43	BHNXC41212	Viruses; unclassified RNA viruses; unclassified RNA viruses ShiM-2016
GCA_001935345.1	8711	2	Beihai picornalike virus 44	BHXG26143	Viruses; unclassified RNA viruses; unclassified RNA viruses ShiM-2016
GCA_001935485.1	6098	1	Beihai picornalike virus 45	BHZC37433	Viruses; unclassified RNA viruses; unclassified RNA viruses ShiM-2016
GCA_001935105.1	9153	2	Beihai picornalike virus 46	BHZC37388	Viruses; unclassified RNA viruses; unclassified RNA viruses ShiM-2016
GCA_001935745.1	8920	2	Beihai picornalike virus 47	SCJXSX39359	Viruses; unclassified RNA viruses; unclassified RNA viruses ShiM-2016
GCA_001935325.1	9382	1	Beihai picornalike virus 48	BHZC37443	Viruses; unclassified RNA viruses; unclassified RNA viruses ShiM-2016
GCA_001934585.1	8407	2	Beihai picornalike virus 49	BHZC36855	Viruses; unclassified RNA viruses; unclassified RNA viruses ShiM-2016
GCA_001923555.1	9888	2	Beihai picornalike virus 5	BHNXC41415	Viruses; unclassified RNA viruses; unclassified RNA viruses ShiM-2016
GCA_001934545.1	7599	1	Beihai picornalike virus 50	BHZC37234	Viruses; unclassified RNA viruses; unclassified RNA viruses ShiM-2016
GCA_001935045.1	10149	2	Beihai picornalike virus 51	SCJXSX38939	Viruses; unclassified RNA viruses; unclassified RNA viruses ShiM-2016
GCA_001935685.1	10278	1	Beihai picornalike virus 52	BHNXC41485	Viruses; unclassified RNA viruses; unclassified RNA viruses ShiM-2016
GCA_001923515.1	9949	2	Beihai picornalike virus 53	BHNXC41443	Viruses; unclassified RNA viruses; unclassified RNA viruses ShiM-2016
GCA_001935265.1	10040	2	Beihai picornalike virus 54	BHNXC41489	Viruses; unclassified RNA viruses; unclassified RNA viruses ShiM-2016
GCA_001934525.1	8697	1	Beihai picornalike virus 55	BHBei68636	Viruses; unclassified RNA viruses; unclassified RNA viruses ShiM-2016
GCA_001935025.1	10546	1	Beihai picornalike virus 56	BWBFG40791	Viruses; unclassified RNA viruses; unclassified RNA viruses ShiM-2016
GCA_002008835.1	8518	1	Beihai picornalike virus 57	BHHQ57630	Viruses; unclassified RNA viruses; unclassified RNA viruses ShiM-2016
GCA_001935665.1	8411	2	Beihai picornalike virus 58	BHBei77093	Viruses; unclassified RNA viruses; unclassified RNA viruses ShiM-2016
GCA_002008475.1	9096	1	Beihai picornalike virus 59	BHBei77099	Viruses; unclassified RNA viruses; unclassified RNA viruses ShiM-2016
GCA_001934145.1	9416	2	Beihai picornalike virus 6	BHNXC41284	Viruses; unclassified RNA viruses; unclassified RNA viruses ShiM-2016
GCA_001934505.1	8818	1	Beihai picornalike virus 60	BHBei55726	Viruses; unclassified RNA viruses; unclassified RNA viruses ShiM-2016
GCA_001935005.1	9113	1	Beihai picornalike virus 61	BHBei76813	Viruses; unclassified RNA viruses; unclassified RNA viruses ShiM-2016
GCA_001923975.1	8507	1	Beihai picornalike virus 62	BHBei74566	Viruses; unclassified RNA viruses; unclassified RNA viruses ShiM-2016
GCA_001934345.1	8334	2	Beihai picornalike virus 63	BHTSS17878	Viruses; unclassified RNA viruses; unclassified RNA viruses ShiM-2016

gca	genome.size	proteome.size	phage.name	strain	Taxonomy
GCA_001933705.1	8647	2	Beihai picornalike virus 64	BHJP54755	Viruses; unclassified RNA viruses; unclassified RNA viruses ShiM-2016
GCA_001934785.1	8918	1	Beihai picornalike virus 65	BHZY60937	Viruses; unclassified RNA viruses; unclassified RNA viruses ShiM-2016
GCA_001933965.1	9233	1	Beihai picornalike virus 66	BHHQ76843	Viruses; unclassified RNA viruses; unclassified RNA viruses ShiM-2016
GCA_001934325.1	10121	1	Beihai picornalike virus 67	BHZY60873	Viruses; unclassified RNA viruses; unclassified RNA viruses ShiM-2016
GCA_001933685.1	7826	1	Beihai picornalike virus 68	HOU158652	Viruses; unclassified RNA viruses; unclassified RNA viruses ShiM-2016
GCA_001934765.1	8407	1	Beihai picornalike virus 69	HOU158647	Viruses; unclassified RNA viruses; unclassified RNA viruses ShiM-2016
GCA_001933945.1	9338	3	Beihai picornalike virus 7	BHNXC41478	Viruses; unclassified RNA viruses; unclassified RNA viruses ShiM-2016
GCA_001924415.1	9214	2	Beihai picornalike virus 70	BHJJX25952	Viruses; unclassified RNA viruses; unclassified RNA viruses ShiM-2016
GCA_001934305.1	9348	2	Beihai picornalike virus 71	BHXG26494	Viruses; unclassified RNA viruses; unclassified RNA viruses ShiM-2016
GCA_001933665.1	9502	2	Beihai picornalike virus 72	BHHK126587	Viruses; unclassified RNA viruses; unclassified RNA viruses ShiM-2016
GCA_001924875.1	9107	1	Beihai picornalike virus 73	BHTH16077	Viruses; unclassified RNA viruses; unclassified RNA viruses ShiM-2016
GCA_001934745.1	8658	2	Beihai picornalike virus 74	BHHK130969	Viruses; unclassified RNA viruses; unclassified RNA viruses ShiM-2016
GCA_001933925.1	10108	2	Beihai picornalike virus 75	BHJP52955	Viruses; unclassified RNA viruses; unclassified RNA viruses ShiM-2016
GCA_001934285.1	8729	2	Beihai picornalike virus 77	BHBei75652	Viruses; unclassified RNA viruses; unclassified RNA viruses ShiM-2016
GCA_001933645.1	9352	2	Beihai picornalike virus 79	BHBJDX18844	Viruses; unclassified RNA viruses; unclassified RNA viruses ShiM-2016
GCA_001934725.1	9273	2	Beihai picornalike virus 8	BHNXC41454	Viruses; unclassified RNA viruses; unclassified RNA viruses ShiM-2016
GCA_001933905.1	7927	2	Beihai picornalike virus 80	BHZC36213	Viruses; unclassified RNA viruses; unclassified RNA viruses ShiM-2016
GCA_001934265.1	7822	2	Beihai picornalike virus 81	HOU149705	Viruses; unclassified RNA viruses; unclassified RNA viruses ShiM-2016
GCA_001933625.1	9386	2	Beihai picornalike virus 82	YYSZX17728	Viruses; unclassified RNA viruses; unclassified RNA viruses ShiM-2016
GCA_001934705.1	9953	3	Beihai picornalike virus 83	BHWZXX15514	Viruses; unclassified RNA viruses; unclassified RNA viruses ShiM-2016
GCA_001933885.1	8143	2	Beihai picornalike virus 84	BHTH16053	Viruses; unclassified RNA viruses; unclassified RNA viruses ShiM-2016
GCA_001934245.1	9188	2	Beihai picornalike virus 85	BHHK129719	Viruses; unclassified RNA viruses; unclassified RNA viruses ShiM-2016
GCA_001933605.1	11435	1	Beihai picornalike virus 87	BHJJX25970	Viruses; unclassified RNA viruses; unclassified RNA viruses ShiM-2016
GCA_001934685.1	8677	2	Beihai picornalike virus 9	BHBJDX18814	Viruses; unclassified RNA viruses; unclassified RNA viruses ShiM-2016
GCA_001933865.1	9632	2	Beihai picornalike virus 90	BHWZXX14572	Viruses; unclassified RNA viruses; unclassified RNA viruses ShiM-2016
GCA_001934225.1	9364	2	Beihai picornalike virus 91	BHJJX25930	Viruses; unclassified RNA viruses; unclassified RNA viruses ShiM-2016
GCA_001933585.1	10613	2	Beihai picornalike virus 93	BHBJDX18546	Viruses; unclassified RNA viruses; unclassified RNA viruses ShiM-2016
GCA_001935085.1	9323	1	Beihai picornalike virus 96	HOU158314	Viruses; unclassified RNA viruses; unclassified RNA viruses ShiM-2016
GCA_001935725.1	6503	1	Beihai picornalike virus 99	BHJJX22326	Viruses; unclassified RNA viruses; unclassified RNA viruses ShiM-2016
GCA_001935305.1	8628	2	Beihai razor shell virus 1	BHZC37272	Viruses; unclassified RNA viruses; unclassified RNA viruses ShiM-2016
GCA_001934565.1	8702	2	Beihai razor shell virus 2	BHBei76898	Viruses; unclassified RNA viruses; unclassified RNA viruses ShiM-2016
GCA_001934665.1	4376	2	Beihai razor shell virus 3	BHZC45609	Viruses; unclassified RNA viruses; unclassified RNA viruses ShiM-2016
GCA_001933845.1	7927	1	Beihai razor shell virus 4	BHZC37363	Viruses; unclassified RNA viruses; unclassified RNA viruses ShiM-2016
GCA_001934205.1	10551	3	Beihai rhabdolike virus 1	BHTSS15727	Viruses; unclassified RNA viruses; unclassified RNA viruses ShiM-2016

gca	genome.size	proteome.size	phage.name	strain	Taxonomy
GCA_001933565.1	12016	4	Beihai rhabdolike virus 2	BHTSS7258	Viruses; unclassified RNA viruses; unclassified RNA viruses ShiM-2016
GCA_001934645.1	10714	3	Beihai rhabdolike virus 4	BHJP58499	Viruses; unclassified RNA viruses; unclassified RNA viruses ShiM-2016
GCA_001933825.1	9404	4	Beihai rhabdolike virus 5	BHJP63888	Viruses; unclassified RNA viruses; unclassified RNA viruses ShiM-2016
GCA_001934185.1	9663	4	Beihai rhabdolike virus 6	BHJJX49420	Viruses; unclassified RNA viruses; unclassified RNA viruses ShiM-2016
GCA_001933545.1	9751	1	Beihai sea slater virus 1	BHHZL10310	Viruses; unclassified RNA viruses; unclassified RNA viruses ShiM-2016
GCA_001934625.1	12772	2	Beihai sea slater virus 2	BHHZL10411	Viruses; unclassified RNA viruses; unclassified RNA viruses ShiM-2016
GCA_001933805.1	6969	3	Beihai sea slater virus 3	BHHZL10233	Viruses; unclassified RNA viruses; unclassified RNA viruses ShiM-2016
GCA_001934165.1	12483	8	Beihai sea slater virus 4	BHHZL10410	Viruses; unclassified RNA viruses; unclassified RNA viruses ShiM-2016
GCA_001933525.1	8870	2	Beihai sesarmid crab virus 1	SCJXSX39422	Viruses; unclassified RNA viruses; unclassified RNA viruses ShiM-2016
GCA_001934605.1	8351	1	Beihai sesarmid crab virus 2	SCJXSX39002	Viruses; unclassified RNA viruses; unclassified RNA viruses ShiM-2016
GCA_002288695.1	8178	2	Beihai sesarmid crab virus 3	SCJXSX39048	Viruses; unclassified RNA viruses; unclassified RNA viruses ShiM-2016
GCA_002288775.1	7380	2	Beihai sesarmid crab virus 4	SCJXSX38901	Viruses; unclassified RNA viruses; unclassified RNA viruses ShiM-2016
GCA_001933785.1	8045	1	Beihai sesarmid crab virus 7	SCJXSX14567	Viruses; unclassified RNA viruses; unclassified RNA viruses ShiM-2016
GCA_001935245.1	11457	2	Beihai shrimp virus 1	BHBJDX17672	Viruses; unclassified RNA viruses; unclassified RNA viruses ShiM-2016
GCA_001935885.1	10355	2	Beihai shrimp virus 2	BHWZXX12501	Viruses; unclassified RNA viruses; unclassified RNA viruses ShiM-2016
GCA_002004255.1	18814	4	Beihai shrimp virus 3	BHBJDX18821	Viruses; unclassified RNA viruses; unclassified RNA viruses ShiM-2016
GCA_001935465.1	3675	2	Beihai shrimp virus 4	BHWZXX19227	Viruses; unclassified RNA viruses; unclassified RNA viruses ShiM-2016
GCA_001935605.1	5431	3	Beihai shrimp virus 5	BHWZXX15615	Viruses; unclassified RNA viruses; unclassified RNA viruses ShiM-2016
GCA_001935225.1	4532	3	Beihai shrimp virus 6	BHBJDX18793	Viruses; unclassified RNA viruses; unclassified RNA viruses ShiM-2016
GCA_001935865.1	8879	2	Beihai sipunculid worm virus 1	BHNXC40689	Viruses; unclassified RNA viruses; unclassified RNA viruses ShiM-2016
GCA_001935445.1	9395	2	Beihai sipunculid worm virus 2	BHNXC41483	Viruses; unclassified RNA viruses; unclassified RNA viruses ShiM-2016
GCA_001935585.1	9110	2	Beihai sipunculid worm virus 3	BHZC37455	Viruses; unclassified RNA viruses; unclassified RNA viruses ShiM-2016
GCA_001935205.1	8487	2	Beihai sipunculid worm virus 4	BHZC37368	Viruses; unclassified RNA viruses; unclassified RNA viruses ShiM-2016
GCA_001935845.1	12524	4	Beihai sipunculid worm virus 5	BHNXC41492	Viruses; unclassified RNA viruses; unclassified RNA viruses ShiM-2016
GCA_001935425.1	8923	1	Beihai sipunculid worm virus 6	BHNXC41400	Viruses; dsRNA viruses; Hypoviridae; unclassified Hypoviridae
GCA_001935565.1	4797	3	Beihai sobemolike virus 1	BHZY60709	Viruses; unclassified RNA viruses; unclassified RNA viruses ShiM-2016
GCA_001924395.1	4902	2	Beihai sobemolike virus 10	BHZY60634	Viruses; unclassified RNA viruses; unclassified RNA viruses ShiM-2016
GCA_001935185.1	5074	2	Beihai sobemolike virus 11	BHZY59277	Viruses; unclassified RNA viruses; unclassified RNA viruses ShiM-2016
GCA_001961555.1	3799	2	Beihai sobemolike virus 12	HOU156693	Viruses; unclassified RNA viruses; unclassified RNA viruses ShiM-2016
GCA_001962295.1	3679	2	Beihai sobemolike virus 13	YYSZX17641	Viruses; unclassified RNA viruses; unclassified RNA viruses ShiM-2016
GCA_001924855.1	3570	2	Beihai sobemolike virus 14	HOU157842	Viruses; unclassified RNA viruses; unclassified RNA viruses ShiM-2016
GCA_001963815.1	3507	2	Beihai sobemolike virus 15	BHBJDX18383	Viruses; unclassified RNA viruses; unclassified RNA viruses ShiM-2016
GCA_001963215.1	3474	2	Beihai sobemolike virus 16	BHCL80925	Viruses; unclassified RNA viruses; unclassified RNA viruses ShiM-2016
GCA_001923495.1	3458	2	Beihai sobemolike virus 17	HOU157151	Viruses; unclassified RNA viruses; unclassified RNA viruses ShiM-2016

gca	genome.size	proteome.size	phage.name	strain	Taxonomy
GCA_001961535.1	4963	2	Beihai sobemolike virus 18	BWBFG40814	Viruses; unclassified RNA viruses; unclassified RNA viruses ShiM-2016
GCA_001962275.1	3559	2	Beihai sobemolike virus 19	BHBJDX18617	Viruses; unclassified RNA viruses; unclassified RNA viruses ShiM-2016
GCA_001963795.1	2930	2	Beihai sobemolike virus 2	BHZC35241	Viruses; unclassified RNA viruses; unclassified RNA viruses ShiM-2016
GCA_001963195.1	2971	2	Beihai sobemolike virus 20	BHNXC40978	Viruses; unclassified RNA viruses; unclassified RNA viruses ShiM-2016
GCA_001961515.1	4177	2	Beihai sobemolike virus 21	BHZY58647	Viruses; unclassified RNA viruses; unclassified RNA viruses ShiM-2016
GCA_001962255.1	3092	2	Beihai sobemolike virus 22	BHWZXX14614	Viruses; unclassified RNA viruses; unclassified RNA viruses ShiM-2016
GCA_001963775.1	4180	2	Beihai sobemolike virus 23	BHZY181484	Viruses; unclassified RNA viruses; unclassified RNA viruses ShiM-2016
GCA_001923955.1	3023	2	Beihai sobemolike virus 24	BHBJDX18033	Viruses; unclassified RNA viruses; unclassified RNA viruses ShiM-2016
GCA_001963175.1	3995	3	Beihai sobemolike virus 25	BHZC34084	Viruses; unclassified RNA viruses; unclassified RNA viruses ShiM-2016
GCA_001924375.1	2729	2	Beihai sobemolike virus 26	BHTH8711	Viruses; unclassified RNA viruses; unclassified RNA viruses ShiM-2016
GCA_001961495.1	3164	2	Beihai sobemolike virus 27	BHWZXX15541	Viruses; unclassified RNA viruses; unclassified RNA viruses ShiM-2016
GCA_001962235.1	4536	2	Beihai sobemolike virus 3	BHZY60830	Viruses; unclassified RNA viruses; unclassified RNA viruses ShiM-2016
GCA_001963755.1	4288	2	Beihai sobemolike virus 4	BHZY59438	Viruses; unclassified RNA viruses; unclassified RNA viruses ShiM-2016
GCA_001963155.1	4484	2	Beihai sobemolike virus 5	BHZY60175	Viruses; unclassified RNA viruses; unclassified RNA viruses ShiM-2016
GCA_001961475.1	5222	2	Beihai sobemolike virus 6	BWBFG40829	Viruses; unclassified RNA viruses; unclassified RNA viruses ShiM-2016
GCA_001962215.1	4711	2	Beihai sobemolike virus 7	BHZY60654	Viruses; unclassified RNA viruses; unclassified RNA viruses ShiM-2016
GCA_001963735.1	4496	2	Beihai sobemolike virus 8	BWBFG36635	Viruses; unclassified RNA viruses; unclassified RNA viruses ShiM-2016
GCA_001963135.1	4560	2	Beihai sobemolike virus 9	BHZY60769	Viruses; unclassified RNA viruses; unclassified RNA viruses ShiM-2016
GCA_001961455.1	9538	1	Beihai sphaeromadae virus 1	BHTSS18012	Viruses; unclassified RNA viruses; unclassified RNA viruses ShiM-2016
GCA_001962195.1	3518	1	Beihai sphaeromadae virus 2	BHTSS26432	Viruses; unclassified RNA viruses; unclassified RNA viruses ShiM-2016
GCA_001921295.1	4478	2	Beihai sphaeromadae virus 3	BHTSS17005	Viruses; unclassified RNA viruses; unclassified RNA viruses ShiM-2016
GCA_001963715.1	6096	2	Beihai sphaeromadae virus 4	BHTSS17969	Viruses; unclassified RNA viruses; unclassified RNA viruses ShiM-2016
GCA_001963115.1	9621	5	Beihai tiger crab virus 1	HWRTX14333	Viruses; unclassified RNA viruses; unclassified RNA viruses ShiM-2016
GCA_001961435.1	4145	3	Beihai tombuslike virus 1	SCJXSX39078	Viruses; unclassified RNA viruses; unclassified RNA viruses ShiM-2016
GCA_001962175.1	4836	4	Beihai tombuslike virus 10	BHZY59908	Viruses; unclassified RNA viruses; unclassified RNA viruses ShiM-2016
GCA_001963695.1	5037	3	Beihai tombuslike virus 11	BHHK128439	Viruses; unclassified RNA viruses; unclassified RNA viruses ShiM-2016
GCA_001961715.1	6097	5	Beihai tombuslike virus 12	BHTSS17936	Viruses; unclassified RNA viruses; unclassified RNA viruses ShiM-2016
GCA_001962455.1	4473	3	Beihai tombuslike virus 13	BHZC37436	Viruses; unclassified RNA viruses; unclassified RNA viruses ShiM-2016
GCA_001963975.1	5160	1	Beihai tombuslike virus 14	BHZC37000	Viruses; unclassified RNA viruses; unclassified RNA viruses ShiM-2016
GCA_001963375.1	3325	2	Beihai tombuslike virus 15	BHBJDX18752	Viruses; unclassified RNA viruses; unclassified RNA viruses ShiM-2016
GCA_001961695.1	4822	3	Beihai tombuslike virus 16	BHWZXX15284	Viruses; unclassified RNA viruses; unclassified RNA viruses ShiM-2016
GCA_001962435.1	5363	2	Beihai tombuslike virus 17	BHZC33014	Viruses; unclassified RNA viruses; unclassified RNA viruses ShiM-2016
GCA_001963955.1	5440	2	Beihai tombuslike virus 18	BHZY60611	Viruses; unclassified RNA viruses; unclassified RNA viruses ShiM-2016
GCA_001967275.1	5695	3	Beihai tombuslike virus 19	BHTH16144	Viruses; unclassified RNA viruses; unclassified RNA viruses ShiM-2016

gca	genome.size	proteome.size	phage.name	strain	Taxonomy
GCA_001963355.1	3737	2	Beihai tombuslike virus 2	BHNXC41455	Viruses; unclassified RNA viruses; unclassified RNA viruses ShiM-2016
GCA_001961675.1	5029	3	Beihai tombuslike virus 3	BHZY54477	Viruses; unclassified RNA viruses; unclassified RNA viruses ShiM-2016
GCA_001924835.1	5201	3	Beihai tombuslike virus 4	BHZY58951	Viruses; unclassified RNA viruses; unclassified RNA viruses ShiM-2016
GCA_001962415.1	3611	2	Beihai tombuslike virus 5	HOU131178	Viruses; unclassified RNA viruses; unclassified RNA viruses ShiM-2016
GCA_001963935.1	3891	4	Beihai tombuslike virus 7	BHBJDX18773	Viruses; unclassified RNA viruses; unclassified RNA viruses ShiM-2016
GCA_001963335.1	3343	2	Beihai tombuslike virus 8	BHZY60516	Viruses; unclassified RNA viruses; unclassified RNA viruses ShiM-2016
GCA_001961655.1	4382	3	Beihai tombuslike virus 9	BHZY58393	Viruses; unclassified RNA viruses; unclassified RNA viruses ShiM-2016
GCA_001962395.1	6618	2	Beihai totilike virus 4	HOU154008	Viruses; unclassified RNA viruses; unclassified RNA viruses ShiM-2016
GCA_001963915.1	6381	1	Beihai uca arcuata virus 1	BFZCX5633	Viruses; unclassified RNA viruses; unclassified RNA viruses ShiM-2016
GCA_001963315.1	4755	2	Beihai victorilike virus 1	BHZC37363	Viruses; unclassified RNA viruses; unclassified RNA viruses ShiM-2016
GCA_001961635.1	5040	2	Beihai weiviruslike virus 1	BWBFG40821	Viruses; unclassified RNA viruses; unclassified RNA viruses ShiM-2016
GCA_001962375.1	4971	2	Beihai weiviruslike virus 11	BWBFG40530	Viruses; unclassified RNA viruses; unclassified RNA viruses ShiM-2016
GCA_001963895.1	4632	2	Beihai weiviruslike virus 12	BWBFG40815	Viruses; unclassified RNA viruses; unclassified RNA viruses ShiM-2016
GCA_001963295.1	4629	3	Beihai weiviruslike virus 13	BHZY60103	Viruses; unclassified RNA viruses; unclassified RNA viruses ShiM-2016
GCA_001961615.1	4740	3	Beihai weiviruslike virus 15	BWBFG27289	Viruses; unclassified RNA viruses; unclassified RNA viruses ShiM-2016
GCA_001962355.1	4423	2	Beihai weiviruslike virus 16	BHZY60618	Viruses; unclassified RNA viruses; unclassified RNA viruses ShiM-2016
GCA_001963875.1	4581	3	Beihai weiviruslike virus 17	BHZC37426	Viruses; unclassified RNA viruses; unclassified RNA viruses ShiM-2016
GCA_001963275.1	3714	1	Beihai weiviruslike virus 18	BHZY59250	Viruses; unclassified RNA viruses; unclassified RNA viruses ShiM-2016
GCA_001961595.1	5094	3	Beihai weiviruslike virus 2	BHZC36779	Viruses; unclassified RNA viruses; unclassified RNA viruses ShiM-2016
GCA_001962335.1	4130	2	Beihai weiviruslike virus 20	BWBFG40173	Viruses; unclassified RNA viruses; unclassified RNA viruses ShiM-2016
GCA_001963855.1	4570	2	Beihai weiviruslike virus 21	BHZY60867	Viruses; unclassified RNA viruses; unclassified RNA viruses ShiM-2016
GCA_001963255.1	4716	2	Beihai weiviruslike virus 3	BHZY60776	Viruses; unclassified RNA viruses; unclassified RNA viruses ShiM-2016
GCA_001961575.1	4692	2	Beihai weiviruslike virus 4	BWBFG40762	Viruses; unclassified RNA viruses; unclassified RNA viruses ShiM-2016
GCA_001964135.1	4409	2	Beihai weiviruslike virus 5	BHZY60693	Viruses; unclassified RNA viruses; unclassified RNA viruses ShiM-2016
GCA_001963535.1	4221	2	Beihai weiviruslike virus 7	BHZY60887	Viruses; unclassified RNA viruses; unclassified RNA viruses ShiM-2016
GCA_001961855.1	5197	2	Beihai weiviruslike virus 8	BHZC36478	Viruses; unclassified RNA viruses; unclassified RNA viruses ShiM-2016
GCA_001962595.1	5218	2	Beihai weiviruslike virus 9	BWBFG39428	Viruses; unclassified RNA viruses; unclassified RNA viruses ShiM-2016
GCA_001964115.1	5420	1	Beihai zhaoviruslike virus 1	BHZY59866	Viruses; unclassified RNA viruses; unclassified RNA viruses ShiM-2016
GCA_001963515.1	9086	1	Beihai zhaoviruslike virus 2	BHTSS12281	Viruses; unclassified RNA viruses; unclassified RNA viruses ShiM-2016
GCA_001961835.1	5448	1	Beihai zhaoviruslike virus 3	BHZY60564	Viruses; unclassified RNA viruses; unclassified RNA viruses ShiM-2016
GCA_001962575.1	8223	1	Beihai zhaoviruslike virus 4	BHZY60633	Viruses; unclassified RNA viruses; unclassified RNA viruses ShiM-2016
GCA_001964095.1	4925	1	Beihai zhaoviruslike virus 5	BHNXC41311	Viruses; unclassified RNA viruses; unclassified RNA viruses ShiM-2016
GCA_001963495.1	5795	4	Changjiang astrolike virus	CJLX30757	Viruses; unclassified RNA viruses; unclassified RNA viruses ShiM-2016
GCA_001961815.1	9103	2	Changjiang crawfish virus 1	CJLX30787	Viruses; unclassified RNA viruses; unclassified RNA viruses ShiM-2016

gca	genome.size	proteome.size	phage.name	strain	Taxonomy
GCA_001962555.1	8712	2	Changjiang crawfish virus 2	CJLX30764	Viruses; unclassified RNA viruses; unclassified RNA viruses ShiM-2016
GCA_001964075.1	8375	2	Changjiang crawfish virus 3	CJLX30786	Viruses; unclassified RNA viruses; unclassified RNA viruses ShiM-2016
GCA_001923475.1	9512	1	Changjiang crawfish virus 4	CLX8819	Viruses; unclassified RNA viruses; unclassified RNA viruses ShiM-2016
GCA_001963475.1	9481	2	Changjiang crawfish virus 5	CJLX22437	Viruses; unclassified RNA viruses; unclassified RNA viruses ShiM-2016
GCA_001961795.1	9743	2	Changjiang crawfish virus 6	CJLX30496	Viruses; unclassified RNA viruses; unclassified RNA viruses ShiM-2016
GCA_001962535.1	3919	3	Changjiang crawfish virus 7	CJLX29643	Viruses; unclassified RNA viruses; unclassified RNA viruses ShiM-2016
GCA_001964055.1	10473	5	Changjiang hepelike virus 1	CJLX30636	Viruses; unclassified RNA viruses; unclassified RNA viruses ShiM-2016
GCA_001963455.1	3229	1	Changjiang narnalike virus 1	CJLX30050	Viruses; unclassified RNA viruses; unclassified RNA viruses ShiM-2016
GCA_001961775.1	4823	2	Changjiang narnalike virus 2	CJLX29055	Viruses; unclassified RNA viruses; unclassified RNA viruses ShiM-2016
GCA_001962515.1	3685	2	Changjiang narnalike virus 3	CJLX30350	Viruses; unclassified RNA viruses; unclassified RNA viruses ShiM-2016
GCA_001964035.1	3964	1	Changjiang narnalike virus 4	CJLX30771	Viruses; unclassified RNA viruses; unclassified RNA viruses ShiM-2016
GCA_001924615.1	8729	1	Changjiang picornalike virus 1	CJLX30149	Viruses; unclassified RNA viruses; unclassified RNA viruses ShiM-2016
GCA_001963435.1	8158	2	Changjiang picornalike virus 10	CJLX30646	Viruses; unclassified RNA viruses; unclassified RNA viruses ShiM-2016
GCA_001961755.1	8031	2	Changjiang picornalike virus 11	CJLX30672	Viruses; unclassified RNA viruses; unclassified RNA viruses ShiM-2016
GCA_001962495.1	8774	2	Changjiang picornalike virus 12	CJLX29956	Viruses; unclassified RNA viruses; unclassified RNA viruses ShiM-2016
GCA_001964015.1	8410	2	Changjiang picornalike virus 13	CJLX40994	Viruses; unclassified RNA viruses; unclassified RNA viruses ShiM-2016
GCA_001963415.1	9296	2	Changjiang picornalike virus 14	CJLX29953	Viruses; unclassified RNA viruses; unclassified RNA viruses ShiM-2016
GCA_001961735.1	9551	1	Changjiang picornalike virus 15	CJLX30633	Viruses; unclassified RNA viruses; unclassified RNA viruses ShiM-2016
GCA_001962475.1	10610	1	Changjiang picornalike virus 16	CJLX20820	Viruses; unclassified RNA viruses; unclassified RNA viruses ShiM-2016
GCA_001963995.1	7171	1	Changjiang picornalike virus 2	CJLX30436	Viruses; unclassified RNA viruses; unclassified RNA viruses ShiM-2016
GCA_001963395.1	9316	2	Changjiang picornalike virus 3	CJLX28786	Viruses; unclassified RNA viruses; unclassified RNA viruses ShiM-2016
GCA_001964415.1	8923	2	Changjiang picornalike virus 4	CJLX29654	Viruses; unclassified RNA viruses; unclassified RNA viruses ShiM-2016
GCA_001965115.1	9182	1	Changjiang picornalike virus 5	CJLX30750	Viruses; unclassified RNA viruses; unclassified RNA viruses ShiM-2016
GCA_001964275.1	8489	2	Changjiang picornalike virus 6	CJLX30470	Viruses; unclassified RNA viruses; unclassified RNA viruses ShiM-2016
GCA_001965935.1	8266	2	Changjiang picornalike virus 7	CJLX30780	Viruses; unclassified RNA viruses; unclassified RNA viruses ShiM-2016
GCA_001964395.1	8685	2	Changjiang picornalike virus 8	CJLX30776	Viruses; unclassified RNA viruses; unclassified RNA viruses ShiM-2016
GCA_001962735.1	8652	2	Changjiang picornalike virus 9	CJLX26226	Viruses; unclassified RNA viruses; unclassified RNA viruses ShiM-2016
GCA_001964255.1	5278	3	Changjiang polerolike virus 1	CJLX30310	Viruses; unclassified RNA viruses; unclassified RNA viruses ShiM-2016
GCA_001965915.1	4863	3	Changjiang sobemolike virus 1	CJLX29826	Viruses; unclassified RNA viruses; unclassified RNA viruses ShiM-2016
GCA_001964375.1	6056	3	Changjiang sobemolike virus 2	CJLX29674	Viruses; unclassified RNA viruses; unclassified RNA viruses ShiM-2016
GCA_001962715.1	3008	2	Changjiang tombuslike virus 1	CJLX26263	Viruses; unclassified RNA viruses; unclassified RNA viruses ShiM-2016
GCA_001964235.1	3776	3	Changjiang tombuslike virus 10	CJLX21891	Viruses; unclassified RNA viruses; unclassified RNA viruses ShiM-2016
GCA_001965895.1	3631	3	Changjiang tombuslike virus 11	CJLX30677	Viruses; unclassified RNA viruses; unclassified RNA viruses ShiM-2016
GCA_001964355.1	3675	2	Changjiang tombuslike virus 12	CJLX27860	Viruses; unclassified RNA viruses; unclassified RNA viruses ShiM-2016

gca	genome.size	proteome.size	phage.name	strain	Taxonomy
GCA_001962695.1	3456	2	Changjiang tombuslike virus 14	CJLX30318	Viruses; unclassified RNA viruses; unclassified RNA viruses ShiM-2016
GCA_001923255.1	3639	2	Changjiang tombuslike virus 15	CJLX30583	Viruses; unclassified RNA viruses; unclassified RNA viruses ShiM-2016
GCA_001964215.1	3570	3	Changjiang tombuslike virus 16	CJLX25258	Viruses; unclassified RNA viruses; unclassified RNA viruses ShiM-2016
GCA_001965875.1	3791	3	Changjiang tombuslike virus 17	CJLX30686	Viruses; unclassified RNA viruses; unclassified RNA viruses ShiM-2016
GCA_001961935.1	4084	3	Changjiang tombuslike virus 18	CJLX21588	Viruses; unclassified RNA viruses; unclassified RNA viruses ShiM-2016
GCA_001962675.1	3051	2	Changjiang tombuslike virus 19	CJLX57318	Viruses; unclassified RNA viruses; unclassified RNA viruses ShiM-2016
GCA_001964195.1	3015	2	Changjiang tombuslike virus 2	CJLX30692	Viruses; unclassified RNA viruses; unclassified RNA viruses ShiM-2016
GCA_001965855.1	4964	2	Changjiang tombuslike virus 20	CJLX30731	Viruses; unclassified RNA viruses; unclassified RNA viruses ShiM-2016
GCA_001961915.1	3932	2	Changjiang tombuslike virus 21	CJLX8746	Viruses; unclassified RNA viruses; unclassified RNA viruses ShiM-2016
GCA_001962655.1	3608	2	Changjiang tombuslike virus 22	CJLX30074	Viruses; unclassified RNA viruses; unclassified RNA viruses ShiM-2016
GCA_001964175.1	4067	4	Changjiang tombuslike virus 3	CJLX30495	Viruses; unclassified RNA viruses; unclassified RNA viruses ShiM-2016
GCA_001965835.1	3694	3	Changjiang tombuslike virus 4	CJLX30236	Viruses; unclassified RNA viruses; unclassified RNA viruses ShiM-2016
GCA_001961895.1	3929	3	Changjiang tombuslike virus 5	CJLX42586	Viruses; unclassified RNA viruses; unclassified RNA viruses ShiM-2016
GCA_001962635.1	3540	3	Changjiang tombuslike virus 6	CJLX30707	Viruses; unclassified RNA viruses; unclassified RNA viruses ShiM-2016
GCA_001964155.1	3875	3	Changjiang tombuslike virus 7	CJLX49320	Viruses; unclassified RNA viruses; unclassified RNA viruses ShiM-2016
GCA_001965815.1	4427	3	Changjiang tombuslike virus 8	CJLX29920	Viruses; unclassified RNA viruses; unclassified RNA viruses ShiM-2016
GCA_001961875.1	4230	2	Changjiang tombuslike virus 9	CJLX30737	Viruses; unclassified RNA viruses; unclassified RNA viruses ShiM-2016
GCA_001962615.1	6844	1	Changjiang zhaoviruslike virus 1	CJLX30599	Viruses; unclassified RNA viruses; unclassified RNA viruses ShiM-2016
GCA_001966675.1	9339	1	Changping earthworm virus 1	CPQY105740	Viruses; unclassified RNA viruses; unclassified RNA viruses ShiM-2016
GCA_002288755.1	13141	7	Changping earthworm virus 2	CPQY18140	Viruses; unclassified RNA viruses; unclassified RNA viruses ShiM-2016
GCA_001964555.1	9356	1	Hubei Potylike virus 1	SCM51506	Viruses; ssRNA viruses; ssRNA positive-strand viruses, no DNA stage; Potyviridae; unclassified Potyviridae
GCA_001965255.1	12562	6	Hubei Wuhan insect virus 9	QTM27230	Viruses; unclassified RNA viruses; unclassified RNA viruses ShiM-2016
GCA_001966655.1	9539	1	Hubei arthropod virus 1	arthropodmix4509	Viruses; unclassified RNA viruses; unclassified RNA viruses ShiM-2016
GCA_001966075.1	9828	1	Hubei arthropod virus 3	GCM7473	Viruses; unclassified RNA viruses; unclassified RNA viruses ShiM-2016
GCA_001923715.1	6784	4	Hubei astrolike virus	WGML136555	Viruses; unclassified RNA viruses; unclassified RNA viruses ShiM-2016
GCA_001964535.1	10831	3	Hubei chuviruslike virus 1	QTM26249	Viruses; unclassified RNA viruses; unclassified RNA viruses ShiM-2016
GCA_001965235.1	11280	3	Hubei chuviruslike virus 3	QTM26698	Viruses; unclassified RNA viruses; unclassified RNA viruses ShiM-2016
GCA_001966635.1	10107	1	Hubei coleoptera virus 1	QCM131202	Viruses; unclassified RNA viruses; unclassified RNA viruses ShiM-2016
GCA_001966055.1	11668	1	Hubei coleoptera virus 2	QCM76287	Viruses; unclassified RNA viruses; unclassified RNA viruses ShiM-2016
GCA_001964515.1	13741	3	Hubei coleoptera virus 3	QCM109726	Viruses; unclassified RNA viruses; unclassified RNA viruses ShiM-2016
GCA_001965215.1	11145	5	Hubei dimarhabdovirus virus 1	SCM51525	Viruses; unclassified RNA viruses; unclassified RNA viruses ShiM-2016
GCA_001966615.1	11332	5	Hubei dimarhabdovirus virus 2	QTM26629	Viruses; unclassified RNA viruses; unclassified RNA viruses ShiM-2016
GCA_001966035.1	11670	5	Hubei dimarhabdovirus virus 3	QTM26149	Viruses; unclassified RNA viruses; unclassified RNA viruses ShiM-2016
GCA_001964495.1	8860	2	Hubei diptera virus 1	SCM51289	Viruses; unclassified RNA viruses; unclassified RNA viruses ShiM-2016

gca	genome.size	proteome.size	phage.name	strain	Taxonomy
GCA_001965195.1	13161	6	Hubei diptera virus 10	SCM43656	Viruses; unclassified RNA viruses; unclassified RNA viruses ShiM-2016
GCA_001966595.1	12513	4	Hubei diptera virus 11	SCM51501	Viruses; unclassified RNA viruses; unclassified RNA viruses ShiM-2016
GCA_001966015.1	4805	3	Hubei diptera virus 12	NA	Viruses; unclassified RNA viruses; unclassified RNA viruses ShiM-2016
GCA_001964475.1	4712	3	Hubei diptera virus 13	SCM94998	Viruses; unclassified RNA viruses; unclassified RNA viruses ShiM-2016
GCA_001965175.1	4257	4	Hubei diptera virus 14	QTM46268	Viruses; unclassified RNA viruses; unclassified RNA viruses ShiM-2016
GCA_001964335.1	2073	2	Hubei diptera virus 15	SCM95219	Viruses; unclassified RNA viruses; unclassified RNA viruses ShiM-2016
GCA_001965995.1	2730	1	Hubei diptera virus 16	SCM32007	Viruses; unclassified RNA viruses; unclassified RNA viruses ShiM-2016
GCA_001964455.1	3333	2	Hubei diptera virus 17	SCM172187	Viruses; unclassified RNA viruses; unclassified RNA viruses ShiM-2016
GCA_001921555.1	3436	2	Hubei diptera virus 18	SCM37169	Viruses; unclassified RNA viruses; unclassified RNA viruses ShiM-2016
GCA_001965155.1	7691	2	Hubei diptera virus 22	SCM45279	Viruses; unclassified RNA viruses; unclassified RNA viruses ShiM-2016
GCA_001921655.1	12096	3	Hubei diptera virus 3	SCM17647	Viruses; unclassified RNA viruses; unclassified RNA viruses ShiM-2016
GCA_001924135.1	12369	3	Hubei diptera virus 4	SCM94992	Viruses; unclassified RNA viruses; unclassified RNA viruses ShiM-2016
GCA_001924595.1	12871	3	Hubei diptera virus 5	SCM245062	Viruses; unclassified RNA viruses; unclassified RNA viruses ShiM-2016
GCA_001964315.1	13000	6	Hubei diptera virus 9	SCM172232	Viruses; unclassified RNA viruses; unclassified RNA viruses ShiM-2016
GCA_001923235.1	13072	2	Hubei earwig virus 3	arthropodmix12795	Viruses; unclassified RNA viruses; unclassified RNA viruses ShiM-2016
GCA_001965975.1	13693	1	Hubei endornalike virus 1	QCM148882	Viruses; unclassified RNA viruses; unclassified RNA viruses ShiM-2016
GCA_001964435.1	7594	3	Hubei hepelike virus 1	WHCC116187	Viruses; unclassified RNA viruses; unclassified RNA viruses ShiM-2016
GCA_001965135.1	5933	3	Hubei hepelike virus 2	WHCC101555	Viruses; unclassified RNA viruses; unclassified RNA viruses ShiM-2016
GCA_001964295.1	7736	3	Hubei hepelike virus 3	WHYY20710	Viruses; unclassified RNA viruses; unclassified RNA viruses ShiM-2016
GCA_001921355.1	30268	11	Hubei insect virus 2	arthropodmix13736	Viruses; unclassified RNA viruses; unclassified RNA viruses ShiM-2016
GCA_001965955.1	4779	3	Hubei leech virus 1	SZmix32467	Viruses; unclassified RNA viruses; unclassified RNA viruses ShiM-2016
GCA_001923695.1	9073	2	Hubei leech virus 2	SZmix21584	Viruses; unclassified RNA viruses; unclassified RNA viruses ShiM-2016
GCA_001965415.1	8848	2	Hubei leech virus 3	SZmix63518	Viruses; unclassified RNA viruses; unclassified RNA viruses ShiM-2016
GCA_001966815.1	8267	2	Hubei leech virus 4	SZmix20870	Viruses; unclassified RNA viruses; unclassified RNA viruses ShiM-2016
GCA_001924115.1	13913	3	Hubei lepidoptera virus 1	LCM141331	Viruses; unclassified RNA viruses; unclassified RNA viruses ShiM-2016
GCA_001966215.1	13109	6	Hubei lepidoptera virus 2	LCM101902	Viruses; unclassified RNA viruses; unclassified RNA viruses ShiM-2016
GCA_001924575.1	24514	10	Hubei lepidoptera virus 3	LCM141729	Viruses; unclassified RNA viruses; unclassified RNA viruses ShiM-2016
GCA_001964695.1	5917	3	Hubei maculalike virus 1	QTM27280	Viruses; unclassified RNA viruses; unclassified RNA viruses ShiM-2016
GCA_001965395.1	6288	2	Hubei maculalike virus 2	QTM27299	Viruses; unclassified RNA viruses; unclassified RNA viruses ShiM-2016
GCA_001966795.1	9074	1	Hubei mosquito virus 1	mosHB236488	Viruses; unclassified RNA viruses; unclassified RNA viruses ShiM-2016
GCA_001966195.1	4811	3	Hubei mosquito virus 2	mosZJ35453	Viruses; unclassified RNA viruses; unclassified RNA viruses ShiM-2016
GCA_001964675.1	2012	2	Hubei mosquito virus 3	3mos6141	Viruses; unclassified RNA viruses; unclassified RNA viruses ShiM-2016
GCA_001923215.1	4971	3	Hubei mosquito virus 4	3mos6213	Viruses; unclassified RNA viruses; unclassified RNA viruses ShiM-2016
GCA_001923675.1	10283	1	Hubei myriapoda virus 1	WGML505798	Viruses; unclassified RNA viruses; unclassified RNA viruses ShiM-2016

gca	genome.size	proteome.size	phage.name	strain	Taxonomy
GCA_001924095.1	11177	3	Hubei myriapoda virus 2	WGML147749	Viruses; unclassified RNA viruses; unclassified RNA viruses ShiM-2016
GCA_001924555.1	10892	4	Hubei myriapoda virus 3	WGML139652	Viruses; unclassified RNA viruses; unclassified RNA viruses ShiM-2016
GCA_001923195.1	12877	2	Hubei myriapoda virus 4	WGML147816	Viruses; unclassified RNA viruses; unclassified RNA viruses ShiM-2016
GCA_002003935.1	15977	3	Hubei myriapoda virus 5	GCM10499	Viruses; unclassified RNA viruses; unclassified RNA viruses ShiM-2016
GCA_001923655.1	16196	6	Hubei myriapoda virus 7	WGML146453	Viruses; unclassified RNA viruses; unclassified RNA viruses ShiM-2016
GCA_001924075.1	13517	4	Hubei myriapoda virus 8	WGML66308	Viruses; unclassified RNA viruses; unclassified RNA viruses ShiM-2016
GCA_002004555.1	4429	4	Hubei myriapoda virus 9	arthropodmix12082	Viruses; unclassified RNA viruses; unclassified RNA viruses ShiM-2016
GCA_001965375.1	5313	2	Hubei narnalike virus 11	WHBL72829	Viruses; unclassified RNA viruses; unclassified RNA viruses ShiM-2016
GCA_001966775.1	3992	1	Hubei narnalike virus 12	SZmix21586	Viruses; unclassified RNA viruses; unclassified RNA viruses ShiM-2016
GCA_001966175.1	2469	1	Hubei narnalike virus 13	QTM27075	Viruses; unclassified RNA viruses; unclassified RNA viruses ShiM-2016
GCA_001964655.1	2872	1	Hubei narnalike virus 15	QCM133922	Viruses; unclassified RNA viruses; unclassified RNA viruses ShiM-2016
GCA_001965355.1	2835	1	Hubei narnalike virus 17	mosHB204971	Viruses; unclassified RNA viruses; unclassified RNA viruses ShiM-2016
GCA_001924535.1	2252	2	Hubei narnalike virus 18	CC64130	Viruses; unclassified RNA viruses; unclassified RNA viruses ShiM-2016
GCA_001966755.1	2275	2	Hubei narnalike virus 19	QTM26970	Viruses; unclassified RNA viruses; unclassified RNA viruses ShiM-2016
GCA_001966155.1	2677	1	Hubei narnalike virus 2	WHBL67117	Viruses; unclassified RNA viruses; unclassified RNA viruses ShiM-2016
GCA_001964635.1	2892	2	Hubei narnalike virus 21	QCM13322	Viruses; unclassified RNA viruses; unclassified RNA viruses ShiM-2016
GCA_001965335.1	2592	1	Hubei narnalike virus 22	SZmix20730	Viruses; unclassified RNA viruses; unclassified RNA viruses ShiM-2016
GCA_001966735.1	2247	1	Hubei narnalike virus 23	SZmix21602	Viruses; unclassified RNA viruses; unclassified RNA viruses ShiM-2016
GCA_001910755.1	2226	1	Hubei narnalike virus 24	SCM50734	Viruses; unclassified RNA viruses; unclassified RNA viruses ShiM-2016
GCA_001968355.1	2375	1	Hubei narnalike virus 25	SCM51430	Viruses; unclassified RNA viruses; unclassified RNA viruses ShiM-2016
GCA_001966135.1	2816	1	Hubei narnalike virus 3	QTM25395	Viruses; unclassified RNA viruses; unclassified RNA viruses ShiM-2016
GCA_001964615.1	2981	1	Hubei narnalike virus 4	WHYY20523	Viruses; unclassified RNA viruses; unclassified RNA viruses ShiM-2016
GCA_001965315.1	2664	1	Hubei narnalike virus 5	SSZZ3007	Viruses; unclassified RNA viruses; unclassified RNA viruses ShiM-2016
GCA_001966715.1	4007	1	Hubei narnalike virus 7	WHBL72300	Viruses; unclassified RNA viruses; unclassified RNA viruses ShiM-2016
GCA_001966115.1	4380	2	Hubei narnalike virus 9	WHBL72555	Viruses; unclassified RNA viruses; unclassified RNA viruses ShiM-2016
GCA_001923175.1	3034	1	Hubei nodalike virus 17	HC30049	Viruses; unclassified RNA viruses; unclassified RNA viruses ShiM-2016
GCA_001964595.1	5619	2	Hubei nodalike virus 8	WHLC5312	Viruses; unclassified RNA viruses; unclassified RNA viruses ShiM-2016
GCA_001965295.1	4959	2	Hubei nodalike virus 9	WHLC5367	Viruses; unclassified RNA viruses; unclassified RNA viruses ShiM-2016
GCA_001966695.1	8313	1	Hubei odonate virus 1	QTM27271	Viruses; unclassified RNA viruses; unclassified RNA viruses ShiM-2016
GCA_001966095.1	14440	6	Hubei odonate virus 10	QTM133243	Viruses; unclassified RNA viruses; unclassified RNA viruses ShiM-2016
GCA_001964575.1	11349	3	Hubei odonate virus 11	QTM161788	Viruses; unclassified RNA viruses; unclassified RNA viruses ShiM-2016
GCA_001965275.1	4035	2	Hubei odonate virus 12	QTM25968	Viruses; unclassified RNA viruses; unclassified RNA viruses ShiM-2016
GCA_001966375.1	10126	1	Hubei odonate virus 2	QTM74832	Viruses; unclassified RNA viruses; unclassified RNA viruses ShiM-2016
GCA_001964855.1	10046	1	Hubei odonate virus 3	QTM25153	Viruses; unclassified RNA viruses; unclassified RNA viruses ShiM-2016

gca	genome.size	proteome.size	phage.name	strain	Taxonomy
GCA_001965555.1	9596	1	Hubei odonate virus 4	QTM161803	Viruses; unclassified RNA viruses; unclassified RNA viruses ShiM-2016
GCA_001966955.1	9820	1	Hubei odonate virus 5	QTM27277	Viruses; unclassified RNA viruses; unclassified RNA viruses ShiM-2016
GCA_001966355.1	12535	4	Hubei odonate virus 6	QTM11719	Viruses; unclassified RNA viruses; unclassified RNA viruses ShiM-2016
GCA_001964835.1	12044	3	Hubei odonate virus 7	QTM27298	Viruses; unclassified RNA viruses; unclassified RNA viruses ShiM-2016
GCA_001921435.1	10837	3	Hubei odonate virus 8	QTM19232	Viruses; unclassified RNA viruses; unclassified RNA viruses ShiM-2016
GCA_001921535.1	10542	3	Hubei odonate virus 9	QTM74767	Viruses; unclassified RNA viruses; unclassified RNA viruses ShiM-2016
GCA_001965535.1	9555	2	Hubei orthoptera virus 1	ZCM21319	Viruses; unclassified RNA viruses; unclassified RNA viruses ShiM-2016
GCA_001966935.1	10327	2	Hubei orthoptera virus 3	ZCM18544	Viruses; unclassified RNA viruses; unclassified RNA viruses ShiM-2016
GCA_001966335.1	4700	2	Hubei orthoptera virus 4	ZCM21011	Viruses; unclassified RNA viruses; unclassified RNA viruses ShiM-2016
GCA_001964815.1	10903	4	Hubei orthoptera virus 5	ZCM94262	Viruses; unclassified RNA viruses; unclassified RNA viruses ShiM-2016
GCA_001921635.1	3358	2	Hubei partitilike virus 11	QTM25188	Viruses; unclassified RNA viruses; unclassified RNA viruses ShiM-2016
GCA_001965515.1	4560	3	Hubei permutotetralike virus 1	mosZJ32939	Viruses; unclassified RNA viruses; unclassified RNA viruses ShiM-2016
GCA_001966915.1	4250	2	Hubei permutotetralike virus 10	ZCM16232	Viruses; unclassified RNA viruses; unclassified RNA viruses ShiM-2016
GCA_001966315.1	4775	2	Hubei permutotetralike virus 11	mosHB234479	Viruses; unclassified RNA viruses; unclassified RNA viruses ShiM-2016
GCA_001964795.1	4551	3	Hubei permutotetralike virus 2	QTM26624	Viruses; unclassified RNA viruses; unclassified RNA viruses ShiM-2016
GCA_001965495.1	4792	3	Hubei permutotetralike virus 3	spider127212	Viruses; unclassified RNA viruses; unclassified RNA viruses ShiM-2016
GCA_001966895.1	4772	3	Hubei permutotetralike virus 4	QTM26287	Viruses; unclassified RNA viruses; unclassified RNA viruses ShiM-2016
GCA_001966295.1	4886	2	Hubei permutotetralike virus 5	WHYY24053	Viruses; unclassified RNA viruses; unclassified RNA viruses ShiM-2016
GCA_001964775.1	5394	2	Hubei permutotetralike virus 6	QCM123521	Viruses; unclassified RNA viruses; unclassified RNA viruses ShiM-2016
GCA_001965475.1	4928	2	Hubei permutotetralike virus 7	QTM27196	Viruses; unclassified RNA viruses; unclassified RNA viruses ShiM-2016
GCA_001966875.1	5117	3	Hubei permutotetralike virus 8	WHSWHC54546	Viruses; unclassified RNA viruses; unclassified RNA viruses ShiM-2016
GCA_001966275.1	5032	3	Hubei permutotetralike virus 9	ZCM21318	Viruses; unclassified RNA viruses; unclassified RNA viruses ShiM-2016
GCA_001964755.1	9635	2	Hubei picornalike virus 1	WHSF7464	Viruses; unclassified RNA viruses; unclassified RNA viruses ShiM-2016
GCA_001965455.1	8307	1	Hubei picornalike virus 10	QTM27287	Viruses; unclassified RNA viruses; unclassified RNA viruses ShiM-2016
GCA_001966855.1	8974	1	Hubei picornalike virus 11	WHZHC53090	Viruses; unclassified RNA viruses; unclassified RNA viruses ShiM-2016
GCA_001966255.1	9598	1	Hubei picornalike virus 12	WHZHC35617	Viruses; unclassified RNA viruses; unclassified RNA viruses ShiM-2016
GCA_001964735.1	8848	1	Hubei picornalike virus 13	QTM26736	Viruses; unclassified RNA viruses; unclassified RNA viruses ShiM-2016
GCA_001965435.1	9435	2	Hubei picornalike virus 14	QTM26191	Viruses; unclassified RNA viruses; unclassified RNA viruses ShiM-2016
GCA_001966835.1	9910	2	Hubei picornalike virus 15	arthropodmix13755	Viruses; unclassified RNA viruses; unclassified RNA viruses ShiM-2016
GCA_001966235.1	9100	2	Hubei picornalike virus 16	SCM51353	Viruses; unclassified RNA viruses; unclassified RNA viruses ShiM-2016
GCA_001964715.1	10234	2	Hubei picornalike virus 17	horsefly124179	Viruses; unclassified RNA viruses; unclassified RNA viruses ShiM-2016
GCA_001967355.1	9478	2	Hubei picornalike virus 18	QTM26408	Viruses; unclassified RNA viruses; unclassified RNA viruses ShiM-2016
GCA_001967855.1	9147	2	Hubei picornalike virus 2	WHBL73152	Viruses; unclassified RNA viruses; unclassified RNA viruses ShiM-2016
GCA_001957395.1	8382	2	Hubei picornalike virus 20	WGML146806	Viruses; unclassified RNA viruses; unclassified RNA viruses ShiM-2016

gca	genome.size	proteome.size	phage.name	strain	Taxonomy
GCA_001968415.1	9311	2	Hubei picornalike virus 21	WHBL73090	Viruses; unclassified RNA viruses; unclassified RNA viruses ShiM-2016
GCA_001965695.1	8253	2	Hubei picornalike virus 22	WHSF7472	Viruses; unclassified RNA viruses; unclassified RNA viruses ShiM-2016
GCA_001923635.1	8358	2	Hubei picornalike virus 24	WGML142000	Viruses; unclassified RNA viruses; unclassified RNA viruses ShiM-2016
GCA_001967095.1	9235	2	Hubei picornalike virus 25	QTM27237	Viruses; unclassified RNA viruses; unclassified RNA viruses ShiM-2016
GCA_001966495.1	8938	1	Hubei picornalike virus 26	spider128568	Viruses; unclassified RNA viruses; unclassified RNA viruses ShiM-2016
GCA_001964975.1	10184	1	Hubei picornalike virus 27	QCM155345	Viruses; unclassified RNA viruses; unclassified RNA viruses ShiM-2016
GCA_001965675.1	10061	1	Hubei picornalike virus 28	QTM24820	Viruses; unclassified RNA viruses; unclassified RNA viruses ShiM-2016
GCA_001967075.1	10308	1	Hubei picornalike virus 29	arthropodmix14031	Viruses; unclassified RNA viruses; unclassified RNA viruses ShiM-2016
GCA_001966475.1	6111	1	Hubei picornalike virus 30	QTM27289	Viruses; unclassified RNA viruses; unclassified RNA viruses ShiM-2016
GCA_001924055.1	8584	1	Hubei picornalike virus 31	QTM26628	Viruses; unclassified RNA viruses; unclassified RNA viruses ShiM-2016
GCA_001964955.1	7696	1	Hubei picornalike virus 32	QTM25882	Viruses; unclassified RNA viruses; unclassified RNA viruses ShiM-2016
GCA_001965655.1	9658	1	Hubei picornalike virus 33	QTM27281	Viruses; unclassified RNA viruses; unclassified RNA viruses ShiM-2016
GCA_001967055.1	9185	1	Hubei picornalike virus 34	QCM148994	Viruses; unclassified RNA viruses; unclassified RNA viruses ShiM-2016
GCA_001966455.1	9266	1	Hubei picornalike virus 35	QTM27260	Viruses; unclassified RNA viruses; unclassified RNA viruses ShiM-2016
GCA_001964935.1	9856	1	Hubei picornalike virus 36	SCM50248	Viruses; unclassified RNA viruses; unclassified RNA viruses ShiM-2016
GCA_001965635.1	9789	1	Hubei picornalike virus 37	WHLC4784	Viruses; unclassified RNA viruses; unclassified RNA viruses ShiM-2016
GCA_001967035.1	9836	1	Hubei picornalike virus 38	spider133826	Viruses; unclassified RNA viruses; unclassified RNA viruses ShiM-2016
GCA_001966435.1	8821	1	Hubei picornalike virus 39	SCM51450	Viruses; unclassified RNA viruses; unclassified RNA viruses ShiM-2016
GCA_001964915.1	8604	2	Hubei picornalike virus 4	WHBL90007	Viruses; unclassified RNA viruses; unclassified RNA viruses ShiM-2016
GCA_001965615.1	8842	1	Hubei picornalike virus 40	WHYY23452	Viruses; unclassified RNA viruses; unclassified RNA viruses ShiM-2016
GCA_001967015.1	8964	1	Hubei picornalike virus 41	QTM133099	Viruses; unclassified RNA viruses; unclassified RNA viruses ShiM-2016
GCA_001966415.1	9129	2	Hubei picornalike virus 42	arthropodmix13971	Viruses; unclassified RNA viruses; unclassified RNA viruses ShiM-2016
GCA_001964895.1	9758	1	Hubei picornalike virus 43	QTM27027	Viruses; unclassified RNA viruses; unclassified RNA viruses ShiM-2016
GCA_001965595.1	9565	1	Hubei picornalike virus 44	WHYY20621	Viruses; unclassified RNA viruses; unclassified RNA viruses ShiM-2016
GCA_001966995.1	10908	1	Hubei picornalike virus 45	spider133521	Viruses; unclassified RNA viruses; unclassified RNA viruses ShiM-2016
GCA_001924515.1	10188	1	Hubei picornalike virus 46	spider133332	Viruses; unclassified RNA viruses; unclassified RNA viruses ShiM-2016
GCA_001966395.1	10106	1	Hubei picornalike virus 47	WHYY21982	Viruses; unclassified RNA viruses; unclassified RNA viruses ShiM-2016
GCA_001964875.1	9702	1	Hubei picornalike virus 48	spider129651	Viruses; unclassified RNA viruses; unclassified RNA viruses ShiM-2016
GCA_001965575.1	9773	1	Hubei picornalike virus 49	WGML112833	Viruses; unclassified RNA viruses; unclassified RNA viruses ShiM-2016
GCA_001966975.1	8763	2	Hubei picornalike virus 5	WHBL70549	Viruses; unclassified RNA viruses; unclassified RNA viruses ShiM-2016
GCA_001968575.1	11297	1	Hubei picornalike virus 50	spider113255	Viruses; unclassified RNA viruses; unclassified RNA viruses ShiM-2016
GCA_001967495.1	8905	2	Hubei picornalike virus 51	QTM27291	Viruses; unclassified RNA viruses; unclassified RNA viruses ShiM-2016
GCA_001967995.1	12566	1	Hubei picornalike virus 52	QCM127295	Viruses; unclassified RNA viruses; unclassified RNA viruses ShiM-2016
GCA_001957535.1	13389	1	Hubei picornalike virus 53	GCM3912	Viruses; unclassified RNA viruses; unclassified RNA viruses ShiM-2016

gca	genome.size	proteome.size	phage.name	strain	Taxonomy
GCA_001968555.1	11022	1	Hubei picornalike virus 54	ZCM19837	Viruses; unclassified RNA viruses; unclassified RNA viruses ShiM-2016
GCA_001967475.1	9795	2	Hubei picornalike virus 55	spider134013	Viruses; unclassified RNA viruses; unclassified RNA viruses ShiM-2016
GCA_001967975.1	10129	2	Hubei picornalike virus 56	SCM49537	Viruses; unclassified RNA viruses; unclassified RNA viruses ShiM-2016
GCA_001957515.1	9145	1	Hubei picornalike virus 58	SSZZ391	Viruses; unclassified RNA viruses; unclassified RNA viruses ShiM-2016
GCA_001968535.1	9035	1	Hubei picornalike virus 59	spider133744	Viruses; unclassified RNA viruses; unclassified RNA viruses ShiM-2016
GCA_001967455.1	8747	2	Hubei picornalike virus 6	WHBL73048	Viruses; unclassified RNA viruses; unclassified RNA viruses ShiM-2016
GCA_001967955.1	9487	1	Hubei picornalike virus 60	QTM27104	Viruses; unclassified RNA viruses; unclassified RNA viruses ShiM-2016
GCA_001957495.1	8681	1	Hubei picornalike virus 61	mosHB235903	Viruses; unclassified RNA viruses; unclassified RNA viruses ShiM-2016
GCA_001968515.1	8840	1	Hubei picornalike virus 62	spider133936	Viruses; unclassified RNA viruses; unclassified RNA viruses ShiM-2016
GCA_001967435.1	8882	1	Hubei picornalike virus 63	insectZJ97544	Viruses; unclassified RNA viruses; unclassified RNA viruses ShiM-2016
GCA_001967935.1	9158	2	Hubei picornalike virus 64	SSZZ3507	Viruses; unclassified RNA viruses; unclassified RNA viruses ShiM-2016
GCA_001957475.1	8941	1	Hubei picornalike virus 65	WHCC118065	Viruses; unclassified RNA viruses; unclassified RNA viruses ShiM-2016
GCA_002366185.1	11931	3	Hubei picornalike virus 66	SSZZ2530	Viruses; Riboviria; unclassified Riboviria; unclassified RNA viruses ShiM-2016
GCA_001968495.1	12379	3	Hubei picornalike virus 67	WGML147056	Viruses; unclassified RNA viruses; unclassified RNA viruses ShiM-2016
GCA_001967415.1	10857	1	Hubei picornalike virus 68	WHWG56209	Viruses; unclassified RNA viruses; unclassified RNA viruses ShiM-2016
GCA_001967915.1	11079	3	Hubei picornalike virus 69	WGML147773	Viruses; unclassified RNA viruses; unclassified RNA viruses ShiM-2016
GCA_001957455.1	9614	1	Hubei picornalike virus 7	WHSF7327	Viruses; unclassified RNA viruses; unclassified RNA viruses ShiM-2016
GCA_001968475.1	10429	4	Hubei picornalike virus 70	WGML134035	Viruses; unclassified RNA viruses; unclassified RNA viruses ShiM-2016
GCA_001967395.1	11260	4	Hubei picornalike virus 71	spider133937	Viruses; unclassified RNA viruses; unclassified RNA viruses ShiM-2016
GCA_001967895.1	11507	3	Hubei picornalike virus 72	WHSFII16052	Viruses; unclassified RNA viruses; unclassified RNA viruses ShiM-2016
GCA_001957435.1	10912	3	Hubei picornalike virus 73	WGML140238	Viruses; unclassified RNA viruses; unclassified RNA viruses ShiM-2016
GCA_001968455.1	10959	3	Hubei picornalike virus 74	WGML145648	Viruses; unclassified RNA viruses; unclassified RNA viruses ShiM-2016
GCA_001967375.1	10123	5	Hubei picornalike virus 75	WGML145097	Viruses; unclassified RNA viruses; unclassified RNA viruses ShiM-2016
GCA_001967875.1	11218	3	Hubei picornalike virus 76	WGML143182	Viruses; unclassified RNA viruses; unclassified RNA viruses ShiM-2016
GCA_001957415.1	8961	4	Hubei picornalike virus 77	WHCC115343	Viruses; unclassified RNA viruses; unclassified RNA viruses ShiM-2016
GCA_001968435.1	9440	2	Hubei picornalike virus 78	WHSF6879	Viruses; unclassified RNA viruses; unclassified RNA viruses ShiM-2016
GCA_001966515.1	9186	2	Hubei picornalike virus 79	WHCCII12350	Viruses; unclassified RNA viruses; unclassified RNA viruses ShiM-2016
GCA_001964995.1	9668	2	Hubei picornalike virus 8	QTM27288	Viruses; unclassified RNA viruses; unclassified RNA viruses ShiM-2016
GCA_001968715.1	6021	1	Hubei picornalike virus 80	WHCCII10252	Viruses; unclassified RNA viruses; unclassified RNA viruses ShiM-2016
GCA_001967635.1	10315	3	Hubei picornalike virus 81	QTM27117	Viruses; unclassified RNA viruses; unclassified RNA viruses ShiM-2016
GCA_001923155.1	11081	3	Hubei picornalike virus 82	spider133992	Viruses; unclassified RNA viruses; unclassified RNA viruses ShiM-2016
GCA_001968135.1	8799	1	Hubei picornalike virus 9	QTM26755	Viruses; unclassified RNA viruses; unclassified RNA viruses ShiM-2016
GCA_001923615.1	4213	3	Hubei polerolike virus 1	WHCC118254	Viruses; unclassified RNA viruses; unclassified RNA viruses ShiM-2016
GCA_001957675.1	6083	5	Hubei polerolike virus 2	QTM26674	Viruses; unclassified RNA viruses; unclassified RNA viruses ShiM-2016

gca	genome.size	proteome.size	phage.name	strain	Taxonomy
GCA_001968695.1	11834	5	Hubei rhabdolike virus 1	QTM25107	Viruses; unclassified RNA viruses; unclassified RNA viruses ShiM-2016
GCA_001967615.1	10841	4	Hubei rhabdolike virus 2	WHSWHC60518	Viruses; unclassified RNA viruses; unclassified RNA viruses ShiM-2016
GCA_001968115.1	12303	6	Hubei rhabdolike virus 3	QCM135517	Viruses; unclassified RNA viruses; unclassified RNA viruses ShiM-2016
GCA_001957655.1	10003	3	Hubei rhabdolike virus 4	arthropodmix13990	Viruses; unclassified RNA viruses; unclassified RNA viruses ShiM-2016
GCA_001968675.1	9614	3	Hubei rhabdolike virus 7	QTM26925	Viruses; unclassified RNA viruses; unclassified RNA viruses ShiM-2016
GCA_001967595.1	13776	7	Hubei rhabdolike virus 9	WHZHC73015	Viruses; unclassified RNA viruses; unclassified RNA viruses ShiM-2016
GCA_001924035.1	3936	3	Hubei sobemolike virus 1	SZmix20791	Viruses; unclassified RNA viruses; unclassified RNA viruses ShiM-2016
GCA_001924495.1	2850	2	Hubei sobemolike virus 10	spider133871	Viruses; unclassified RNA viruses; unclassified RNA viruses ShiM-2016
GCA_001923135.1	2803	2	Hubei sobemolike virus 11	spider125434	Viruses; unclassified RNA viruses; unclassified RNA viruses ShiM-2016
GCA_001923595.1	2543	2	Hubei sobemolike virus 12	spider127544	Viruses; unclassified RNA viruses; unclassified RNA viruses ShiM-2016
GCA_001924015.1	2750	3	Hubei sobemolike virus 13	WHYY18840	Viruses; unclassified RNA viruses; unclassified RNA viruses ShiM-2016
GCA_001924475.1	2566	2	Hubei sobemolike virus 14	QTM6420	Viruses; unclassified RNA viruses; unclassified RNA viruses ShiM-2016
GCA_001923115.1	3049	2	Hubei sobemolike virus 15	tick96090	Viruses; unclassified RNA viruses; unclassified RNA viruses ShiM-2016
GCA_001923935.1	3113	2	Hubei sobemolike virus 16	WGML146458	Viruses; unclassified RNA viruses; unclassified RNA viruses ShiM-2016
GCA_001924355.1	3226	2	Hubei sobemolike virus 18	arthropodmix13949	Viruses; unclassified RNA viruses; unclassified RNA viruses ShiM-2016
GCA_001924815.1	3252	2	Hubei sobemolike virus 19	SCM51527	Viruses; unclassified RNA viruses; unclassified RNA viruses ShiM-2016
GCA_001923455.1	4288	3	Hubei sobemolike virus 2	WGML145690	Viruses; unclassified RNA viruses; unclassified RNA viruses ShiM-2016
GCA_001923915.1	3152	2	Hubei sobemolike virus 20	CC64562	Viruses; unclassified RNA viruses; unclassified RNA viruses ShiM-2016
GCA_001924335.1	3076	2	Hubei sobemolike virus 22	ZCM21279	Viruses; unclassified RNA viruses; unclassified RNA viruses ShiM-2016
GCA_001924795.1	3119	2	Hubei sobemolike virus 23	QTM27250	Viruses; unclassified RNA viruses; unclassified RNA viruses ShiM-2016
GCA_001923435.1	2223	2	Hubei sobemolike virus 24	WHLC5390	Viruses; unclassified RNA viruses; unclassified RNA viruses ShiM-2016
GCA_001923895.1	3284	2	Hubei sobemolike virus 25	QTM27214	Viruses; unclassified RNA viruses; unclassified RNA viruses ShiM-2016
GCA_001924315.1	3211	2	Hubei sobemolike virus 26	arthropodmix14077	Viruses; unclassified RNA viruses; unclassified RNA viruses ShiM-2016
GCA_001924775.1	3344	2	Hubei sobemolike virus 27	WHCCII13324	Viruses; unclassified RNA viruses; unclassified RNA viruses ShiM-2016
GCA_001923415.1	3164	2	Hubei sobemolike virus 28	QTM26976	Viruses; unclassified RNA viruses; unclassified RNA viruses ShiM-2016
GCA_001923875.1	3237	2	Hubei sobemolike virus 29	QTM12808	Viruses; unclassified RNA viruses; unclassified RNA viruses ShiM-2016
GCA_001924295.1	5221	3	Hubei sobemolike virus 3	WHBL72900	Viruses; unclassified RNA viruses; unclassified RNA viruses ShiM-2016
GCA_001924755.1	3215	2	Hubei sobemolike virus 30	QTM19610	Viruses; unclassified RNA viruses; unclassified RNA viruses ShiM-2016
GCA_001923395.1	3179	2	Hubei sobemolike virus 31	WHCC117732	Viruses; unclassified RNA viruses; unclassified RNA viruses ShiM-2016
GCA_001923855.1	3325	2	Hubei sobemolike virus 32	spider112797	Viruses; unclassified RNA viruses; unclassified RNA viruses ShiM-2016
GCA_001924275.1	3057	2	Hubei sobemolike virus 33	LCM101922	Viruses; unclassified RNA viruses; unclassified RNA viruses ShiM-2016
GCA_001924735.1	3342	2	Hubei sobemolike virus 34	SSZZ41	Viruses; unclassified RNA viruses; unclassified RNA viruses ShiM-2016
GCA_001923375.1	3447	2	Hubei sobemolike virus 35	QTM24286	Viruses; unclassified RNA viruses; unclassified RNA viruses ShiM-2016
GCA_001923835.1	2841	2	Hubei sobemolike virus 36	spider124913	Viruses; unclassified RNA viruses; unclassified RNA viruses ShiM-2016

gca	genome.size	proteome.size	phage.name	strain	Taxonomy
GCA_001924255.1	2585	3	Hubei sobemolike virus 37	QTM25849	Viruses; unclassified RNA viruses; unclassified RNA viruses ShiM-2016
GCA_001924715.1	2768	3	Hubei sobemolike virus 38	QTM27202	Viruses; unclassified RNA viruses; unclassified RNA viruses ShiM-2016
GCA_001923355.1	2802	2	Hubei sobemolike virus 40	QTM104806	Viruses; unclassified RNA viruses; unclassified RNA viruses ShiM-2016
GCA_001923815.1	2787	2	Hubei sobemolike virus 41	3mos6151	Viruses; unclassified RNA viruses; unclassified RNA viruses ShiM-2016
GCA_001924235.1	2791	2	Hubei sobemolike virus 42	QTM26192	Viruses; unclassified RNA viruses; unclassified RNA viruses ShiM-2016
GCA_001924695.1	2645	2	Hubei sobemolike virus 43	arthropodmix11560	Viruses; unclassified RNA viruses; unclassified RNA viruses ShiM-2016
GCA_001923335.1	2718	2	Hubei sobemolike virus 44	WGML133391	Viruses; unclassified RNA viruses; unclassified RNA viruses ShiM-2016
GCA_001923795.1	2748	2	Hubei sobemolike virus 45	QTM25680	Viruses; unclassified RNA viruses; unclassified RNA viruses ShiM-2016
GCA_001924215.1	2642	2	Hubei sobemolike virus 46	spider133700	Viruses; unclassified RNA viruses; unclassified RNA viruses ShiM-2016
GCA_001924675.1	2721	2	Hubei sobemolike virus 47	spider133229	Viruses; unclassified RNA viruses; unclassified RNA viruses ShiM-2016
GCA_001923315.1	2654	2	Hubei sobemolike virus 48	QCM18154	Viruses; unclassified RNA viruses; unclassified RNA viruses ShiM-2016
GCA_001923775.1	2767	2	Hubei sobemolike virus 49	QTM12655	Viruses; unclassified RNA viruses; unclassified RNA viruses ShiM-2016
GCA_001924195.1	3250	2	Hubei sobemolike virus 5	spider59976	Viruses; unclassified RNA viruses; unclassified RNA viruses ShiM-2016
GCA_001924655.1	2892	2	Hubei sobemolike virus 6	spider128669	Viruses; unclassified RNA viruses; unclassified RNA viruses ShiM-2016
GCA_001923295.1	2662	2	Hubei sobemolike virus 7	spider134446	Viruses; unclassified RNA viruses; unclassified RNA viruses ShiM-2016
GCA_001923755.1	2721	2	Hubei sobemolike virus 8	QTM19779	Viruses; unclassified RNA viruses; unclassified RNA viruses ShiM-2016
GCA_001924175.1	2782	2	Hubei sobemolike virus 9	spider112041	Viruses; unclassified RNA viruses; unclassified RNA viruses ShiM-2016
GCA_001968095.1	8601	1	Hubei tetragnatha maxillosa virus 1	arthropodmix23158	Viruses; unclassified RNA viruses; unclassified RNA viruses ShiM-2016
GCA_001957635.1	9763	1	Hubei tetragnatha maxillosa virus 2	QTM20713	Viruses; unclassified RNA viruses; unclassified RNA viruses ShiM-2016
GCA_001968655.1	9528	1	Hubei tetragnatha maxillosa virus 3	SSZZ2994	Viruses; unclassified RNA viruses; unclassified RNA viruses ShiM-2016
GCA_001967575.1	9037	1	Hubei tetragnatha maxillosa virus 4	arthropodmix13651	Viruses; unclassified RNA viruses; unclassified RNA viruses ShiM-2016
GCA_001968075.1	9650	1	Hubei tetragnatha maxillosa virus 5	SSZZ3471	Viruses; unclassified RNA viruses; unclassified RNA viruses ShiM-2016
GCA_001924635.1	4640	3	Hubei tetragnatha maxillosa virus 6	SSZZ3520	Viruses; unclassified RNA viruses; unclassified RNA viruses ShiM-2016
GCA_001957615.1	19864	3	Hubei tetragnatha maxillosa virus 7	SSZZ3468	Viruses; unclassified RNA viruses; unclassified RNA viruses ShiM-2016
GCA_001968635.1	6641	4	Hubei tetragnatha maxillosa virus 8	arthropodmix13417	Viruses; unclassified RNA viruses; unclassified RNA viruses ShiM-2016
GCA_001967555.1	9487	1	Hubei tick virus 1	tick109202	Viruses; unclassified RNA viruses; unclassified RNA viruses ShiM-2016
GCA_001968055.1	9660	1	Hubei tick virus 2	tick109131	Viruses; unclassified RNA viruses; unclassified RNA viruses ShiM-2016
GCA_001957595.1	9177	1	Hubei tick virus 3	tick108426	Viruses; unclassified RNA viruses; unclassified RNA viruses ShiM-2016
GCA_001968615.1	5430	4	Hubei tombuslike virus 1	WHWN54407	Viruses; unclassified RNA viruses; unclassified RNA viruses ShiM-2016
GCA_001967535.1	3851	2	Hubei tombuslike virus 10	WHCC112361	Viruses; unclassified RNA viruses; unclassified RNA viruses ShiM-2016
GCA_001968035.1	2724	2	Hubei tombuslike virus 11	WHSFII11317	Viruses; unclassified RNA viruses; unclassified RNA viruses ShiM-2016
GCA_001957575.1	2495	2	Hubei tombuslike virus 12	WHBL71803	Viruses; unclassified RNA viruses; unclassified RNA viruses ShiM-2016
GCA_001968595.1	5904	2	Hubei tombuslike virus 13	WHYY18977	Viruses; unclassified RNA viruses; unclassified RNA viruses ShiM-2016
GCA_001967515.1	4303	3	Hubei tombuslike virus 14	spider118341	Viruses; unclassified RNA viruses; unclassified RNA viruses ShiM-2016

gca	genome.size	proteome.size	phage.name	strain	Taxonomy
GCA_001968015.1	4000	3	Hubei tombuslike virus 15	WHYY21098	Viruses; unclassified RNA viruses; unclassified RNA viruses ShiM-2016
GCA_001926995.1	4401	3	Hubei tombuslike virus 16	spider58816	Viruses; unclassified RNA viruses; unclassified RNA viruses ShiM-2016
GCA_001957555.1	3717	3	Hubei tombuslike virus 17	QTM26798	Viruses; unclassified RNA viruses; unclassified RNA viruses ShiM-2016
GCA_001967335.1	4059	4	Hubei tombuslike virus 18	QTM27278	Viruses; unclassified RNA viruses; unclassified RNA viruses ShiM-2016
GCA_001967835.1	4917	4	Hubei tombuslike virus 19	WHCCII13323	Viruses; unclassified RNA viruses; unclassified RNA viruses ShiM-2016
GCA_001957375.1	5510	4	Hubei tombuslike virus 2	WHSFII19265	Viruses; unclassified RNA viruses; unclassified RNA viruses ShiM-2016
GCA_001968395.1	4854	4	Hubei tombuslike virus 20	spider131362	Viruses; unclassified RNA viruses; unclassified RNA viruses ShiM-2016
GCA_001967315.1	4633	3	Hubei tombuslike virus 21	spider121926	Viruses; unclassified RNA viruses; unclassified RNA viruses ShiM-2016
GCA_002366245.1	4268	3	Hubei tombuslike virus 22	QTM26784	Viruses; Riboviria; unclassified Riboviria; unclassified RNA viruses ShiM-2016
GCA_001967815.1	3196	2	Hubei tombuslike virus 23	WHCCII13249	Viruses; unclassified RNA viruses; unclassified RNA viruses ShiM-2016
GCA_001957355.1	2613	2	Hubei tombuslike virus 24	GCM76561	Viruses; unclassified RNA viruses; unclassified RNA viruses ShiM-2016
GCA_001968375.1	4209	3	Hubei tombuslike virus 25	WHBL69613	Viruses; unclassified RNA viruses; unclassified RNA viruses ShiM-2016
GCA_001967295.1	2286	2	Hubei tombuslike virus 26	QTM27120	Viruses; unclassified RNA viruses; unclassified RNA viruses ShiM-2016
GCA_001967795.1	2209	2	Hubei tombuslike virus 27	SSZZ3453	Viruses; unclassified RNA viruses; unclassified RNA viruses ShiM-2016
GCA_001965095.1	2539	2	Hubei tombuslike virus 28	SSZZ1762	Viruses; unclassified RNA viruses; unclassified RNA viruses ShiM-2016
GCA_001965795.1	2550	2	Hubei tombuslike virus 29	spider124630	Viruses; unclassified RNA viruses; unclassified RNA viruses ShiM-2016
GCA_001967195.1	3663	3	Hubei tombuslike virus 3	WGML143241	Viruses; unclassified RNA viruses; unclassified RNA viruses ShiM-2016
GCA_001926155.1	2328	2	Hubei tombuslike virus 30	spider132818	Viruses; unclassified RNA viruses; unclassified RNA viruses ShiM-2016
GCA_001967235.1	4264	3	Hubei tombuslike virus 31	WHBL72828	Viruses; unclassified RNA viruses; unclassified RNA viruses ShiM-2016
GCA_001926735.1	3477	2	Hubei tombuslike virus 32	WHBL71139	Viruses; unclassified RNA viruses; unclassified RNA viruses ShiM-2016
GCA_001965075.1	4193	2	Hubei tombuslike virus 33	WHBL67076	Viruses; unclassified RNA viruses; unclassified RNA viruses ShiM-2016
GCA_001927235.1	5703	3	Hubei tombuslike virus 34	WHBL73047	Viruses; unclassified RNA viruses; unclassified RNA viruses ShiM-2016
GCA_001965775.1	4168	2	Hubei tombuslike virus 36	WGML125257	Viruses; unclassified RNA viruses; unclassified RNA viruses ShiM-2016
GCA_001926395.1	5516	1	Hubei tombuslike virus 37	WHBL72297	Viruses; unclassified RNA viruses; unclassified RNA viruses ShiM-2016
GCA_001967175.1	3628	3	Hubei tombuslike virus 38	WHWG56034	Viruses; unclassified RNA viruses; unclassified RNA viruses ShiM-2016
GCA_001966575.1	6453	3	Hubei tombuslike virus 39	WGML9144	Viruses; unclassified RNA viruses; unclassified RNA viruses ShiM-2016
GCA_001965055.1	3833	3	Hubei tombuslike virus 4	WGML147612	Viruses; unclassified RNA viruses; unclassified RNA viruses ShiM-2016
GCA_001965755.1	3498	3	Hubei tombuslike virus 40	QCM118690	Viruses; unclassified RNA viruses; unclassified RNA viruses ShiM-2016
GCA_001967155.1	3668	2	Hubei tombuslike virus 42	fly55787	Viruses; unclassified RNA viruses; unclassified RNA viruses ShiM-2016
GCA_001966555.1	5102	3	Hubei tombuslike virus 43	WGML146568	Viruses; unclassified RNA viruses; unclassified RNA viruses ShiM-2016
GCA_001965035.1	3085	2	Hubei tombuslike virus 5	WGML136364	Viruses; unclassified RNA viruses; unclassified RNA viruses ShiM-2016
GCA_001965735.1	3770	3	Hubei tombuslike virus 6	WGML12775	Viruses; unclassified RNA viruses; unclassified RNA viruses ShiM-2016
GCA_001967135.1	5082	3	Hubei tombuslike virus 7	WHBL69243	Viruses; unclassified RNA viruses; unclassified RNA viruses ShiM-2016
GCA_001966535.1	4326	3	Hubei tombuslike virus 8	WHCC116238	Viruses; unclassified RNA viruses; unclassified RNA viruses ShiM-2016

gca	genome.size	proteome.size	phage.name	strain	Taxonomy
GCA_001965015.1	3501	2	Hubei tombuslike virus 9	WHBL63242	Viruses; unclassified RNA viruses; unclassified RNA viruses ShiM-2016
GCA_001965715.1	7081	2	Hubei totilike virus 10	3mos6210	Viruses; unclassified RNA viruses; unclassified RNA viruses ShiM-2016
GCA_001967115.1	5336	2	Hubei totilike virus 12	HC21305	Viruses; unclassified RNA viruses; unclassified RNA viruses ShiM-2016
GCA_001961395.1	6293	2	Hubei totilike virus 13	QTM25941	Viruses; unclassified RNA viruses; unclassified RNA viruses ShiM-2016
GCA_001962135.1	5859	2	Hubei totilike virus 15	SCM51462	Viruses; unclassified RNA viruses; unclassified RNA viruses ShiM-2016
GCA_001963655.1	6754	3	Hubei totilike virus 16	spider123775	Viruses; unclassified RNA viruses; unclassified RNA viruses ShiM-2016
GCA_001963055.1	6761	3	Hubei totilike virus 17	tick107859	Viruses; unclassified RNA viruses; unclassified RNA viruses ShiM-2016
GCA_001961375.1	7552	2	Hubei totilike virus 18	WHCC116098	Viruses; unclassified RNA viruses; unclassified RNA viruses ShiM-2016
GCA_001925955.1	7198	2	Hubei totilike virus 19	horsefly123848	Viruses; unclassified RNA viruses; unclassified RNA viruses ShiM-2016
GCA_001962115.1	4958	2	Hubei totilike virus 2	arthropodmix13450	Viruses; unclassified RNA viruses; unclassified RNA viruses ShiM-2016
GCA_001963635.1	7804	2	Hubei totilike virus 20	CC62244	Viruses; unclassified RNA viruses; unclassified RNA viruses ShiM-2016
GCA_001963035.1	6659	2	Hubei totilike virus 21	CC64629	Viruses; unclassified RNA viruses; unclassified RNA viruses ShiM-2016
GCA_001961355.1	5595	2	Hubei totilike virus 24	tick106628	Viruses; unclassified RNA viruses; unclassified RNA viruses ShiM-2016
GCA_001962095.1	7505	1	Hubei totilike virus 5	WHSWHC37547	Viruses; unclassified RNA viruses; unclassified RNA viruses ShiM-2016
GCA_001963615.1	3615	1	Hubei totilike virus 9	QTM27092	Viruses; unclassified RNA viruses; unclassified RNA viruses ShiM-2016
GCA_001963015.1	4524	3	Hubei unio douglasiae virus 1	WHBL72336	Viruses; unclassified RNA viruses; unclassified RNA viruses ShiM-2016
GCA_001961335.1	4431	3	Hubei unio douglasiae virus 2	WHBL72658	Viruses; unclassified RNA viruses; unclassified RNA viruses ShiM-2016
GCA_001962075.1	4130	2	Hubei unio douglasiae virus 3	WHBL73106	Viruses; unclassified RNA viruses; unclassified RNA viruses ShiM-2016
GCA_001963595.1	9141	2	Hubei virgalike virus 1	mosHB236471	Viruses; unclassified RNA viruses; unclassified RNA viruses ShiM-2016
GCA_001962995.1	6206	4	Hubei virgalike virus 11	fly114676	Viruses; unclassified RNA viruses; unclassified RNA viruses ShiM-2016
GCA_001961315.1	8318	6	Hubei virgalike virus 12	SCM49209	Viruses; unclassified RNA viruses; unclassified RNA viruses ShiM-2016
GCA_001962055.1	10423	3	Hubei virgalike virus 15	QCM619087	Viruses; unclassified RNA viruses; unclassified RNA viruses ShiM-2016
GCA_001963575.1	13549	6	Hubei virgalike virus 16	arthropodmix13816	Viruses; unclassified RNA viruses; unclassified RNA viruses ShiM-2016
GCA_001962975.1	9481	3	Hubei virgalike virus 17	QTM23703	Viruses; unclassified RNA viruses; unclassified RNA viruses ShiM-2016
GCA_001926715.1	11189	5	Hubei virgalike virus 18	fly97447	Viruses; unclassified RNA viruses; unclassified RNA viruses ShiM-2016
GCA_001961295.1	10650	2	Hubei virgalike virus 2	mosHB235832	Viruses; unclassified RNA viruses; unclassified RNA viruses ShiM-2016
GCA_001962035.1	10072	4	Hubei virgalike virus 21	mosHB236537	Viruses; unclassified RNA viruses; unclassified RNA viruses ShiM-2016
GCA_001963555.1	14639	6	Hubei virgalike virus 23	mosHB236486	Viruses; unclassified RNA viruses; unclassified RNA viruses ShiM-2016
GCA_001962955.1	9480	3	Hubei virgalike virus 7	QTM27262	Viruses; unclassified RNA viruses; unclassified RNA viruses ShiM-2016
GCA_001961275.1	10976	4	Hubei virgalike virus 9	SCM51642	Viruses; unclassified RNA viruses; unclassified RNA viruses ShiM-2016
GCA_001962015.1	5804	3	Hubei yanviruslike virus 1	WHCC105213	Viruses; unclassified RNA viruses; unclassified RNA viruses ShiM-2016
GCA_001961135.1	6041	2	Hubei zhaoviruslike virus 1	WHBL52766	Viruses; unclassified RNA viruses; unclassified RNA viruses ShiM-2016
GCA_001962935.1	5682	2	Hubei zhaoviruslike virus 2	WHBL71389	Viruses; unclassified RNA viruses; unclassified RNA viruses ShiM-2016
GCA_001961255.1	10069	1	Jingmen picornalike virus	JMYJCC71227	Viruses; unclassified RNA viruses; unclassified RNA viruses ShiM-2016

gca	genome.size	proteome.size	phage.name	strain	Taxonomy
GCA_002008755.1	7949	2	Jingmen tombuslike virus 1	JMYJCC68055	Viruses; unclassified RNA viruses; unclassified RNA viruses ShiM-2016
GCA_001961115.1	8238	2	Sanxia Qinviruslike virus 1	SXXX11646	Viruses; unclassified RNA viruses; unclassified RNA viruses ShiM-2016
GCA_001962915.1	11157	5	Sanxia atyid shrimp virus 1	SXXX35475	Viruses; unclassified RNA viruses; unclassified RNA viruses ShiM-2016
GCA_001961235.1	10445	1	Sanxia atyid shrimp virus 2	WHCCII13331	Viruses; unclassified RNA viruses; unclassified RNA viruses ShiM-2016
GCA_001961975.1	8772	2	Sanxia atyid shrimp virus 3	SXXX37589	Viruses; unclassified RNA viruses; unclassified RNA viruses ShiM-2016
GCA_001961095.1	11270	3	Sanxia atyid shrimp virus 4	SXXX37205	Viruses; unclassified RNA viruses; unclassified RNA viruses ShiM-2016
GCA_001962895.1	3643	2	Sanxia narnalike virus 1	SXXX35820	Viruses; unclassified RNA viruses; unclassified RNA viruses ShiM-2016
GCA_001961215.1	4650	3	Sanxia permutotetralike virus 1	SXSSP2461	Viruses; unclassified RNA viruses; unclassified RNA viruses ShiM-2016
GCA_001961955.1	9301	2	Sanxia picornalike virus 1	SXXX37713	Viruses; unclassified RNA viruses; unclassified RNA viruses ShiM-2016
GCA_001961075.1	9596	1	Sanxia picornalike virus 10	SXXX37528	Viruses; unclassified RNA viruses; unclassified RNA viruses ShiM-2016
GCA_001962875.1	8342	2	Sanxia picornalike virus 11	SXXX37695	Viruses; unclassified RNA viruses; unclassified RNA viruses ShiM-2016
GCA_001925255.1	8518	2	Sanxia picornalike virus 12	SXXX24153	Viruses; unclassified RNA viruses; unclassified RNA viruses ShiM-2016
GCA_001961195.1	8978	2	Sanxia picornalike virus 13	SXXX34014	Viruses; unclassified RNA viruses; unclassified RNA viruses ShiM-2016
GCA_001959555.1	9058	2	Sanxia picornalike virus 2	SXXX37883	Viruses; unclassified RNA viruses; unclassified RNA viruses ShiM-2016
GCA_001961055.1	8903	2	Sanxia picornalike virus 3	SXXX36208	Viruses; unclassified RNA viruses; unclassified RNA viruses ShiM-2016
GCA_001962855.1	7388	1	Sanxia picornalike virus 4	SXXX37816	Viruses; unclassified RNA viruses; unclassified RNA viruses ShiM-2016
GCA_001961175.1	8673	2	Sanxia picornalike virus 5	SXXX34146	Viruses; unclassified RNA viruses; unclassified RNA viruses ShiM-2016
GCA_001959535.1	7418	1	Sanxia picornalike virus 8	SXXX35540	Viruses; unclassified RNA viruses; unclassified RNA viruses ShiM-2016
GCA_001961035.1	8114	2	Sanxia picornalike virus 9	SXXX36560	Viruses; unclassified RNA viruses; unclassified RNA viruses ShiM-2016
GCA_001962835.1	2863	3	Sanxia sobemolike virus 1	SXSSP20269	Viruses; unclassified RNA viruses; unclassified RNA viruses ShiM-2016
GCA_001961155.1	3332	2	Sanxia sobemolike virus 2	SXSSP2316	Viruses; unclassified RNA viruses; unclassified RNA viruses ShiM-2016
GCA_001959515.1	3216	2	Sanxia sobemolike virus 3	SXSSP2531	Viruses; unclassified RNA viruses; unclassified RNA viruses ShiM-2016
GCA_001961015.1	2934	2	Sanxia sobemolike virus 4	SXSSP2021	Viruses; unclassified RNA viruses; unclassified RNA viruses ShiM-2016
GCA_001962815.1	3181	2	Sanxia sobemolike virus 5	SXSSP2561	Viruses; unclassified RNA viruses; unclassified RNA viruses ShiM-2016
GCA_001958755.1	3446	3	Sanxia tombuslike virus 1	SXXX36383	Viruses; unclassified RNA viruses; unclassified RNA viruses ShiM-2016
GCA_001959495.1	3557	3	Sanxia tombuslike virus 2	SXXX29202	Viruses; unclassified RNA viruses; unclassified RNA viruses ShiM-2016
GCA_001960995.1	2981	2	Sanxia tombuslike virus 3	SXXX37381	Viruses; unclassified RNA viruses; unclassified RNA viruses ShiM-2016
GCA_001962795.1	3815	3	Sanxia tombuslike virus 4	SXXX7002	Viruses; unclassified RNA viruses; unclassified RNA viruses ShiM-2016
GCA_001958735.1	2585	2	Sanxia tombuslike virus 5	SXSSP12445	Viruses; unclassified RNA viruses; unclassified RNA viruses ShiM-2016
GCA_001959475.1	3284	2	Sanxia tombuslike virus 6	SXSSP2574	Viruses; unclassified RNA viruses; unclassified RNA viruses ShiM-2016
GCA_001927215.1	4557	4	Sanxia tombuslike virus 7	SXSSP2567	Viruses; unclassified RNA viruses; unclassified RNA viruses ShiM-2016
GCA_001960975.1	2566	2	Sanxia tombuslike virus 8	SXSSP2544	Viruses; unclassified RNA viruses; unclassified RNA viruses ShiM-2016
GCA_001962775.1	5094	3	Sanxia tombuslike virus 9	SXXX37849	Viruses; unclassified RNA viruses; unclassified RNA viruses ShiM-2016
GCA_001958715.1	4626	3	Sanxia water strider virus 10	SXSSP2571	Viruses; unclassified RNA viruses; unclassified RNA viruses ShiM-2016

gca	genome.size	proteome.size	phage.name	strain	Taxonomy
GCA_001959455.1	2308	1	Sanxia water strider virus 13	SXSSP2578	Viruses; unclassified RNA viruses; unclassified RNA viruses ShiM-2016
GCA_001960955.1	4175	4	Sanxia water strider virus 14	SXSSP2565	Viruses; unclassified RNA viruses; unclassified RNA viruses ShiM-2016
GCA_001926375.1	3705	2	Sanxia water strider virus 15	SXSSP1894	Viruses; unclassified RNA viruses; unclassified RNA viruses ShiM-2016
GCA_001962755.1	3108	1	Sanxia water strider virus 16	SXXX38069	Viruses; unclassified RNA viruses; unclassified RNA viruses ShiM-2016
GCA_001958695.1	4369	2	Sanxia water strider virus 17	SXSSP1704	Viruses; unclassified RNA viruses; unclassified RNA viruses ShiM-2016
GCA_001959435.1	4981	3	Sanxia water strider virus 19	SXSSP2568	Viruses; unclassified RNA viruses; unclassified RNA viruses ShiM-2016
GCA_001960935.1	7192	2	Sanxia water strider virus 20	SXSSP2584	Viruses; unclassified RNA viruses; unclassified RNA viruses ShiM-2016
GCA_001960335.1	10153	1	Sanxia water strider virus 21	SXSSP2576	Viruses; unclassified RNA viruses; unclassified RNA viruses ShiM-2016
GCA_001958675.1	10194	3	Sanxia water strider virus 7	SXSSP2499	Viruses; unclassified RNA viruses; unclassified RNA viruses ShiM-2016
GCA_001959415.1	9166	1	Sanxia water strider virus 8	SXSSP2580	Viruses; unclassified RNA viruses; unclassified RNA viruses ShiM-2016
GCA_001960915.1	8687	1	Sanxia water strider virus 9	SXSSP2367	Viruses; unclassified RNA viruses; unclassified RNA viruses ShiM-2016
GCA_001925935.1	9324	2	Shahe arthropod virus 1	SHWC0209c12762	Viruses; unclassified RNA viruses; unclassified RNA viruses ShiM-2016
GCA_001960315.1	15783	1	Shahe endornalike virus 1	SHWC0209c12790	Viruses; unclassified RNA viruses; unclassified RNA viruses ShiM-2016
GCA_001921575.1	9839	5	Shahe hepelike virus 1	SHWC13572	Viruses; unclassified RNA viruses; unclassified RNA viruses ShiM-2016
GCA_001921335.1	10233	6	Shahe hepelike virus 2	SHWC13612	Viruses; unclassified RNA viruses; unclassified RNA viruses ShiM-2016
GCA_001958655.1	10019	1	Shahe heteroptera virus 1	SHWC13624	Viruses; unclassified RNA viruses; unclassified RNA viruses ShiM-2016
GCA_001926695.1	9893	1	Shahe heteroptera virus 2	SHWC01c3694	Viruses; unclassified RNA viruses; unclassified RNA viruses ShiM-2016
GCA_001921415.1	11076	3	Shahe heteroptera virus 3	SHWC01c3680	Viruses; unclassified RNA viruses; unclassified RNA viruses ShiM-2016
GCA_001959395.1	8739	2	Shahe heteroptera virus 4	SHWC01c2975	Viruses; unclassified RNA viruses; unclassified RNA viruses ShiM-2016
GCA_001960895.1	10120	1	Shahe isopoda virus 1	SHWC0209c12784	Viruses; unclassified RNA viruses; unclassified RNA viruses ShiM-2016
GCA_001960295.1	10297	1	Shahe isopoda virus 2	SHWC0209c12758	Viruses; unclassified RNA viruses; unclassified RNA viruses ShiM-2016
GCA_001958635.1	6235	2	Shahe isopoda virus 3	SHWC0209c10670	Viruses; unclassified RNA viruses; unclassified RNA viruses ShiM-2016
GCA_001927195.1	4371	4	Shahe isopoda virus 5	SHWC0209c16708	Viruses; unclassified RNA viruses; unclassified RNA viruses ShiM-2016
GCA_001959375.1	3209	1	Shahe narnalike virus 1	SHWC0209c12582	Viruses; unclassified RNA viruses; unclassified RNA viruses ShiM-2016
GCA_001960875.1	3375	1	Shahe narnalike virus 2	SHWC0209c12594	Viruses; unclassified RNA viruses; unclassified RNA viruses ShiM-2016
GCA_001960275.1	2982	1	Shahe narnalike virus 3	SHWC01c3372	Viruses; unclassified RNA viruses; unclassified RNA viruses ShiM-2016
GCA_001958615.1	1684	1	Shahe narnalike virus 5	SHWC0209c20055	Viruses; unclassified RNA viruses; unclassified RNA viruses ShiM-2016
GCA_001959355.1	2281	1	Shahe narnalike virus 7	SHWC0209c12713	Viruses; unclassified RNA viruses; unclassified RNA viruses ShiM-2016
GCA_001960855.1	8922	2	Shahe picornalike virus 1	SHWC13622	Viruses; unclassified RNA viruses; unclassified RNA viruses ShiM-2016
GCA_001960255.1	8281	1	Shahe picornalike virus 10	SHWC13586	Viruses; unclassified RNA viruses; unclassified RNA viruses ShiM-2016
GCA_001958595.1	9081	2	Shahe picornalike virus 11	SHWC13613	Viruses; unclassified RNA viruses; unclassified RNA viruses ShiM-2016
GCA_001959335.1	7849	2	Shahe picornalike virus 12	SHWC0209c11951	Viruses; unclassified RNA viruses; unclassified RNA viruses ShiM-2016
GCA_001960835.1	9077	1	Shahe picornalike virus 13	SHWC13556	Viruses; unclassified RNA viruses; unclassified RNA viruses ShiM-2016
GCA_001960235.1	10360	1	Shahe picornalike virus 14	SHWC13617	Viruses; unclassified RNA viruses; unclassified RNA viruses ShiM-2016

gca	genome.size	proteome.size	phage.name	strain	Taxonomy
GCA_001958575.1	9272	2	Shahe picornalike virus 2	SHWC0209c12507	Viruses; unclassified RNA viruses; unclassified RNA viruses ShiM-2016
GCA_001959315.1	8352	2	Shahe picornalike virus 3	SHWC13623	Viruses; unclassified RNA viruses; unclassified RNA viruses ShiM-2016
GCA_001960815.1	8860	1	Shahe picornalike virus 4	SHWC13603	Viruses; unclassified RNA viruses; unclassified RNA viruses ShiM-2016
GCA_001960215.1	8658	2	Shahe picornalike virus 5	SHWC13542	Viruses; unclassified RNA viruses; unclassified RNA viruses ShiM-2016
GCA_001958555.1	8831	2	Shahe picornalike virus 6	SHWC0209c11084	Viruses; unclassified RNA viruses; unclassified RNA viruses ShiM-2016
GCA_001959295.1	10076	1	Shahe picornalike virus 7	SHWC0209c12710	Viruses; unclassified RNA viruses; unclassified RNA viruses ShiM-2016
GCA_001926355.1	8675	2	Shahe picornalike virus 8	SHWC12398	Viruses; unclassified RNA viruses; unclassified RNA viruses ShiM-2016
GCA_001960795.1	9126	2	Shahe picornalike virus 9	SHWC0209c12638	Viruses; unclassified RNA viruses; unclassified RNA viruses ShiM-2016
GCA_001960195.1	7807	2	Shahe qinviruslike virus 1	SHWC0209c11359	Viruses; unclassified RNA viruses; unclassified RNA viruses ShiM-2016
GCA_001958535.1	3593	2	Shahe sobemolike virus 1	SHWCII5101	Viruses; unclassified RNA viruses; unclassified RNA viruses ShiM-2016
GCA_001959275.1	2824	2	Shahe tombuslike virus 1	SHWC0209c12685	Viruses; unclassified RNA viruses; unclassified RNA viruses ShiM-2016
GCA_001960775.1	3090	2	Shahe tombuslike virus 2	SHWC01c3797	Viruses; unclassified RNA viruses; unclassified RNA viruses ShiM-2016
GCA_001960175.1	7768	2	Shahe yueviruslike virus 1	SHWC0209c11789	Viruses; unclassified RNA viruses; unclassified RNA viruses ShiM-2016
GCA_001958515.1	13283	6	Shayang ascaridia galli virus 2	SYJFC48550	Viruses; unclassified RNA viruses; unclassified RNA viruses ShiM-2016
GCA_001925915.1	12384	3	Shayang virgalike virus 1	SYJFC48301	Viruses; unclassified RNA viruses; unclassified RNA viruses ShiM-2016
GCA_001959255.1	7548	4	Shuangao alphatetralike virus 1	insectZJ93971	Viruses; unclassified RNA viruses; unclassified RNA viruses ShiM-2016
GCA_004790575.1	12740	4	Shuangao chrysolike virus 1	mosWSB68555	Viruses; Riboviria; Orthornavirae; Duplornaviricota; Chrymotiviricetes; Ghabrivirales; Chrysoviridae; Alphachrysovirus; Shuangao insect-associated chrysovirus
GCA_001960755.1	2758	1	Shuangao insect virus 10	insectZJ94627	Viruses; unclassified RNA viruses; unclassified RNA viruses ShiM-2016
GCA_001960155.1	2704	1	Shuangao insect virus 11	insectZJ65889	Viruses; unclassified RNA viruses; unclassified RNA viruses ShiM-2016
GCA_001958495.1	9854	1	Shuangao insect virus 12	insectZJ98124	Viruses; unclassified RNA viruses; unclassified RNA viruses ShiM-2016
GCA_001959235.1	12155	4	Shuangao insect virus 8	insectZJ96360	Viruses; unclassified RNA viruses; unclassified RNA viruses ShiM-2016
GCA_001960735.1	2799	2	Shuangao insect virus 9	insectZJ96499	Viruses; unclassified RNA viruses; unclassified RNA viruses ShiM-2016
GCA_001960135.1	3718	2	Shuangao sobemolike virus 1	insectZJ66941	Viruses; unclassified RNA viruses; unclassified RNA viruses ShiM-2016
GCA_001958475.1	2791	2	Shuangao sobemolike virus 2	insectZJ89114	Viruses; unclassified RNA viruses; unclassified RNA viruses ShiM-2016
GCA_001959215.1	2781	2	Shuangao sobemolike virus 3	insectZJ66198	Viruses; unclassified RNA viruses; unclassified RNA viruses ShiM-2016
GCA_001960715.1	6891	2	Shuangao totilike virus	insectZJ97483	Viruses; unclassified RNA viruses; unclassified RNA viruses ShiM-2016
GCA_001960115.1	10183	3	Wenling crustacean virus 1	WLJQ101409	Viruses; unclassified RNA viruses; unclassified RNA viruses ShiM-2016
GCA_001958455.1	11938	5	Wenling crustacean virus 10	WLJQ101844	Viruses; unclassified RNA viruses; unclassified RNA viruses ShiM-2016
GCA_001959195.1	11560	5	Wenling crustacean virus 11	WLJQ201798	Viruses; unclassified RNA viruses; unclassified RNA viruses ShiM-2016
GCA_001960695.1	7447	3	Wenling crustacean virus 12	WLJQ47777	Viruses; unclassified RNA viruses; unclassified RNA viruses ShiM-2016
GCA_001960095.1	10829	3	Wenling crustacean virus 13	WLJQ104251	Viruses; unclassified RNA viruses; unclassified RNA viruses ShiM-2016
GCA_001958435.1	11314	4	Wenling crustacean virus 14	WLJQ104130	Viruses; unclassified RNA viruses; unclassified RNA viruses ShiM-2016
GCA_001959175.1	11119	3	Wenling crustacean virus 15	WLJQ91782	Viruses; unclassified RNA viruses; unclassified RNA viruses ShiM-2016
GCA_001960675.1	8664	2	Wenling crustacean virus 2	WLJQ102135	Viruses; unclassified RNA viruses; unclassified RNA viruses ShiM-2016

gca	genome.size	proteome.size	phage.name	strain	Taxonomy
GCA_001960075.1	9887	2	Wenling crustacean virus 3	WLJQ142924	Viruses; unclassified RNA viruses; unclassified RNA viruses ShiM-2016
GCA_001958415.1	9291	1	Wenling crustacean virus 4	WLJQ79834	Viruses; unclassified RNA viruses; unclassified RNA viruses ShiM-2016
GCA_001959155.1	10528	2	Wenling crustacean virus 5	WLJQ103138	Viruses; unclassified RNA viruses; unclassified RNA viruses ShiM-2016
GCA_001960655.1	9716	2	Wenling crustacean virus 6	WLJQ101491	Viruses; unclassified RNA viruses; unclassified RNA viruses ShiM-2016
GCA_001921515.1	10757	3	Wenling crustacean virus 9	WLJQ100911	Viruses; unclassified RNA viruses; unclassified RNA viruses ShiM-2016
GCA_001960055.1	10962	5	Wenling hepelike virus 1	WLJQ103981	Viruses; unclassified RNA viruses; unclassified RNA viruses ShiM-2016
GCA_001958395.1	12441	4	Wenling hepelike virus 2	WLJQ99021	Viruses; unclassified RNA viruses; unclassified RNA viruses ShiM-2016
GCA_001959135.1	11515	4	Wenling hepelike virus 3	WLJQ102665	Viruses; unclassified RNA viruses; unclassified RNA viruses ShiM-2016
GCA_001960635.1	10025	6	Wenling hepelike virus 4	WLJQ102701	Viruses; unclassified RNA viruses; unclassified RNA viruses ShiM-2016
GCA_001960035.1	2583	1	Wenling narnalike virus 1	WLJQ102793	Viruses; unclassified RNA viruses; unclassified RNA viruses ShiM-2016
GCA_001958375.1	2781	1	Wenling narnalike virus 2	WLJQ103952	Viruses; unclassified RNA viruses; unclassified RNA viruses ShiM-2016
GCA_001959115.1	3319	1	Wenling narnalike virus 3	WLJQ100768	Viruses; unclassified RNA viruses; unclassified RNA viruses ShiM-2016
GCA_001960615.1	1793	1	Wenling narnalike virus 4	WLJQ103797	Viruses; unclassified RNA viruses; unclassified RNA viruses ShiM-2016
GCA_001960015.1	1790	1	Wenling narnalike virus 5	WLJQ94194	Viruses; unclassified RNA viruses; unclassified RNA viruses ShiM-2016
GCA_001958355.1	2723	2	Wenling narnalike virus 7	WLJQ103612	Viruses; unclassified RNA viruses; unclassified RNA viruses ShiM-2016
GCA_001959095.1	2702	1	Wenling narnalike virus 9	WLJQ205243	Viruses; unclassified RNA viruses; unclassified RNA viruses ShiM-2016
GCA_001959995.1	9155	2	Wenling picornalike virus 1	WLJQ101464	Viruses; unclassified RNA viruses; unclassified RNA viruses ShiM-2016
GCA_001926675.1	10000	2	Wenling picornalike virus 2	WLJQ104117	Viruses; unclassified RNA viruses; unclassified RNA viruses ShiM-2016
GCA_001958335.1	10236	2	Wenling picornalike virus 3	WLJQ100015	Viruses; unclassified RNA viruses; unclassified RNA viruses ShiM-2016
GCA_001959075.1	8837	2	Wenling picornalike virus 4	WLJQ100990	Viruses; unclassified RNA viruses; unclassified RNA viruses ShiM-2016
GCA_001960575.1	8467	2	Wenling picornalike virus 5	WLJQ102796	Viruses; unclassified RNA viruses; unclassified RNA viruses ShiM-2016
GCA_001959975.1	10033	1	Wenling picornalike virus 6	WLJQ101512	Viruses; unclassified RNA viruses; unclassified RNA viruses ShiM-2016
GCA_001958315.1	7484	2	Wenling picornalike virus 7	WLJQ102051	Viruses; unclassified RNA viruses; unclassified RNA viruses ShiM-2016
GCA_001959055.1	9299	1	Wenling picornalike virus 8	WLJQ103995	Viruses; unclassified RNA viruses; unclassified RNA viruses ShiM-2016
GCA_001960555.1	9041	1	Wenling picornalike virus 9	WLJQ104209	Viruses; unclassified RNA viruses; unclassified RNA viruses ShiM-2016
GCA_001959955.1	3259	2	Wenling sobemolike virus 1	WLJQ103508	Viruses; unclassified RNA viruses; unclassified RNA viruses ShiM-2016
GCA_001958295.1	2571	2	Wenling sobemolike virus 2	WLJQ95081	Viruses; unclassified RNA viruses; unclassified RNA viruses ShiM-2016
GCA_001959035.1	9247	2	Wenling togalike virus	WLJQ101932	Viruses; unclassified RNA viruses; unclassified RNA viruses ShiM-2016
GCA_001960535.1	3348	2	Wenling tombuslike virus 1	WLJQ104192	Viruses; unclassified RNA viruses; unclassified RNA viruses ShiM-2016
GCA_001959935.1	4118	4	Wenling tombuslike virus 2	WLJQ104302	Viruses; unclassified RNA viruses; unclassified RNA viruses ShiM-2016
GCA_001958275.1	3908	3	Wenling tombuslike virus 3	WLJQ76752	Viruses; unclassified RNA viruses; unclassified RNA viruses ShiM-2016
GCA_001959015.1	5321	3	Wenling tombuslike virus 4	WLJQ100551	Viruses; unclassified RNA viruses; unclassified RNA viruses ShiM-2016
GCA_001960515.1	4752	3	Wenling tombuslike virus 5	WLJQ104103	Viruses; unclassified RNA viruses; unclassified RNA viruses ShiM-2016
GCA_001959915.1	8033	2	Wenling totilike virus 2	WLJQ104148	Viruses; unclassified RNA viruses; unclassified RNA viruses ShiM-2016

gca	genome.size	proteome.size	phage.name	strain	Taxonomy
GCA_001958255.1	5635	2	Wenling zhaoviruslike virus 1	WLJQ103300	Viruses; unclassified RNA viruses; unclassified RNA viruses ShiM-2016
GCA_001958995.1	11290	1	Wenzhou bivalvia virus 1	beimix75829	Viruses; unclassified RNA viruses; unclassified RNA viruses ShiM-2016
GCA_001960495.1	6570	2	Wenzhou bivalvia virus 2	beimix75763	Viruses; unclassified RNA viruses; unclassified RNA viruses ShiM-2016
GCA_001959895.1	5077	2	Wenzhou bivalvia virus 3	beimix38484	Viruses; unclassified RNA viruses; unclassified RNA viruses ShiM-2016
GCA_001958235.1	8943	2	Wenzhou channeled applesnail virus 1	WZFSL85493	Viruses; unclassified RNA viruses; unclassified RNA viruses ShiM-2016
GCA_001958975.1	10481	2	Wenzhou channeled applesnail virus 2	WZFSL85846	Viruses; unclassified RNA viruses; unclassified RNA viruses ShiM-2016
GCA_001960475.1	8377	1	Wenzhou channeled applesnail virus 3	BHBei77067	Viruses; unclassified RNA viruses; unclassified RNA viruses ShiM-2016
GCA_001959875.1	4778	3	Wenzhou crab virus 4	WZRBX43276	Viruses; unclassified RNA viruses; unclassified RNA viruses ShiM-2016
GCA_001958215.1	8691	5	Wenzhou crab virus 5	WZRBX37749	Viruses; unclassified RNA viruses; unclassified RNA viruses ShiM-2016
GCA_001958955.1	9309	2	Wenzhou gastropodes virus 1	WZSLuoI86017	Viruses; unclassified RNA viruses; unclassified RNA viruses ShiM-2016
GCA_001960455.1	8926	1	Wenzhou gastropodes virus 2	WZSLuoI86086	Viruses; unclassified RNA viruses; unclassified RNA viruses ShiM-2016
GCA_001959855.1	7652	2	Wenzhou hepelike virus 2	WZFSL85851	Viruses; unclassified RNA viruses; unclassified RNA viruses ShiM-2016
GCA_001958195.1	10162	5	Wenzhou hepelike virus 1	WZSLuoII97618	Viruses; unclassified RNA viruses; unclassified RNA viruses ShiM-2016
GCA_001958935.1	2642	1	Wenzhou narnalike virus 2	shrimp13495	Viruses; unclassified RNA viruses; unclassified RNA viruses ShiM-2016
GCA_001960435.1	2716	1	Wenzhou narnalike virus 3	shrimp14503	Viruses; unclassified RNA viruses; unclassified RNA viruses ShiM-2016
GCA_001927175.1	4234	2	Wenzhou narnalike virus 4	WZSLuoI82501	Viruses; unclassified RNA viruses; unclassified RNA viruses ShiM-2016
GCA_001959835.1	3959	2	Wenzhou narnalike virus 5	WZFSL79329	Viruses; unclassified RNA viruses; unclassified RNA viruses ShiM-2016
GCA_001958175.1	3206	1	Wenzhou narnalike virus 7	WZFSL82100	Viruses; unclassified RNA viruses; unclassified RNA viruses ShiM-2016
GCA_001958915.1	2768	1	Wenzhou narnalike virus 8	WZRBX43201	Viruses; unclassified RNA viruses; unclassified RNA viruses ShiM-2016
GCA_001960415.1	4228	1	Wenzhou narnalike virus 9	WZSLuoI85295	Viruses; unclassified RNA viruses; unclassified RNA viruses ShiM-2016
GCA_001959815.1	8804	2	Wenzhou picornalike virus 1	WZSLuoI85940	Viruses; unclassified RNA viruses; unclassified RNA viruses ShiM-2016
GCA_001958155.1	9142	2	Wenzhou picornalike virus 10	WZRBX43164	Viruses; unclassified RNA viruses; unclassified RNA viruses ShiM-2016
GCA_001958895.1	7094	1	Wenzhou picornalike virus 11	WZFSL83389	Viruses; unclassified RNA viruses; unclassified RNA viruses ShiM-2016
GCA_001960395.1	9025	2	Wenzhou picornalike virus 12	WZFSL74240	Viruses; unclassified RNA viruses; unclassified RNA viruses ShiM-2016
GCA_001959795.1	9458	1	Wenzhou picornalike virus 13	WZSBei69459	Viruses; unclassified RNA viruses; unclassified RNA viruses ShiM-2016
GCA_001958135.1	8951	2	Wenzhou picornalike virus 14	WZSLuoII97619	Viruses; unclassified RNA viruses; unclassified RNA viruses ShiM-2016
GCA_001958875.1	8610	2	Wenzhou picornalike virus 15	WZFSL85504	Viruses; unclassified RNA viruses; unclassified RNA viruses ShiM-2016
GCA_001960375.1	6372	1	Wenzhou picornalike virus 16	WZFSL67369	Viruses; unclassified RNA viruses; unclassified RNA viruses ShiM-2016
GCA_001959775.1	8127	2	Wenzhou picornalike virus 17	WZSBei69283	Viruses; unclassified RNA viruses; unclassified RNA viruses ShiM-2016
GCA_001958115.1	6682	1	Wenzhou picornalike virus 18	shrimp14534	Viruses; unclassified RNA viruses; unclassified RNA viruses ShiM-2016
GCA_001958855.1	10153	2	Wenzhou picornalike virus 19	WZSLuoII97616	Viruses; unclassified RNA viruses; unclassified RNA viruses ShiM-2016
GCA_001960355.1	8895	2	Wenzhou picornalike virus 2	beimix73672	Viruses; unclassified RNA viruses; unclassified RNA viruses ShiM-2016
GCA_001959755.1	8476	2	Wenzhou picornalike virus 20	WZSBei68985	Viruses; unclassified RNA viruses; unclassified RNA viruses ShiM-2016
GCA_001958095.1	7620	2	Wenzhou picornalike virus 21	WZSBei23778	Viruses; unclassified RNA viruses; unclassified RNA viruses ShiM-2016

gca	genome.size	proteome.size	phage.name	strain	Taxonomy
GCA_001958835.1	8890	1	Wenzhou picornalike virus 22	WZFSL83961	Viruses; unclassified RNA viruses; unclassified RNA viruses ShiM-2016
GCA_001957955.1	9223	1	Wenzhou picornalike virus 23	WZFSL85852	Viruses; unclassified RNA viruses; unclassified RNA viruses ShiM-2016
GCA_001959735.1	8778	1	Wenzhou picornalike virus 24	WZSBei69493	Viruses; unclassified RNA viruses; unclassified RNA viruses ShiM-2016
GCA_001958075.1	8464	1	Wenzhou picornalike virus 25	WZFSL75477	Viruses; unclassified RNA viruses; unclassified RNA viruses ShiM-2016
GCA_001958815.1	9287	2	Wenzhou picornalike virus 26	WZSLuoII93478	Viruses; unclassified RNA viruses; unclassified RNA viruses ShiM-2016
GCA_001957935.1	8091	1	Wenzhou picornalike virus 27	shrimp14502	Viruses; unclassified RNA viruses; unclassified RNA viruses ShiM-2016
GCA_001959715.1	10012	2	Wenzhou picornalike virus 28	WZRBX42853	Viruses; unclassified RNA viruses; unclassified RNA viruses ShiM-2016
GCA_001958055.1	9463	2	Wenzhou picornalike virus 29	BHBei77092	Viruses; unclassified RNA viruses; unclassified RNA viruses ShiM-2016
GCA_001926335.1	8452	1	Wenzhou picornalike virus 3	WZSBei69560	Viruses; unclassified RNA viruses; unclassified RNA viruses ShiM-2016
GCA_001958795.1	8678	2	Wenzhou picornalike virus 31	WZFSL72092	Viruses; unclassified RNA viruses; unclassified RNA viruses ShiM-2016
GCA_001957915.1	9319	2	Wenzhou picornalike virus 32	WZSBei69487	Viruses; unclassified RNA viruses; unclassified RNA viruses ShiM-2016
GCA_001959695.1	8465	2	Wenzhou picornalike virus 33	WZFSL76563	Viruses; unclassified RNA viruses; unclassified RNA viruses ShiM-2016
GCA_001958035.1	9865	2	Wenzhou picornalike virus 35	WZRBX39547	Viruses; unclassified RNA viruses; unclassified RNA viruses ShiM-2016
GCA_001958775.1	9667	1	Wenzhou picornalike virus 36	WZRBX14225	Viruses; unclassified RNA viruses; unclassified RNA viruses ShiM-2016
GCA_001957895.1	9763	2	Wenzhou picornalike virus 37	WZSBei69579	Viruses; unclassified RNA viruses; unclassified RNA viruses ShiM-2016
GCA_001959675.1	8215	1	Wenzhou picornalike virus 39	WZFSL83776	Viruses; unclassified RNA viruses; unclassified RNA viruses ShiM-2016
GCA_001958015.1	9345	2	Wenzhou picornalike virus 4	WZSLuoI86003	Viruses; unclassified RNA viruses; unclassified RNA viruses ShiM-2016
GCA_001968335.1	8752	2	Wenzhou picornalike virus 40	WZFSL83107	Viruses; unclassified RNA viruses; unclassified RNA viruses ShiM-2016
GCA_001957875.1	7966	2	Wenzhou picornalike virus 41	WZFSL80064	Viruses; unclassified RNA viruses; unclassified RNA viruses ShiM-2016
GCA_001959655.1	7810	2	Wenzhou picornalike virus 42	WZFSL74289	Viruses; unclassified RNA viruses; unclassified RNA viruses ShiM-2016
GCA_001957995.1	7396	1	Wenzhou picornalike virus 43	WZSBei69573	Viruses; unclassified RNA viruses; unclassified RNA viruses ShiM-2016
GCA_001968315.1	10856	1	Wenzhou picornalike virus 44	WZSLuoII91200	Viruses; unclassified RNA viruses; unclassified RNA viruses ShiM-2016
GCA_001957855.1	9581	1	Wenzhou picornalike virus 45	WZSBei69068	Viruses; unclassified RNA viruses; unclassified RNA viruses ShiM-2016
GCA_001959635.1	10473	1	Wenzhou picornalike virus 46	WZFSL85813	Viruses; unclassified RNA viruses; unclassified RNA viruses ShiM-2016
GCA_001957975.1	8235	1	Wenzhou picornalike virus 47	WHCCII11151	Viruses; unclassified RNA viruses; unclassified RNA viruses ShiM-2016
GCA_001968295.1	9550	1	Wenzhou picornalike virus 48	beimix75770	Viruses; unclassified RNA viruses; unclassified RNA viruses ShiM-2016
GCA_001957835.1	8916	1	Wenzhou picornalike virus 49	WZFSL84024	Viruses; unclassified RNA viruses; unclassified RNA viruses ShiM-2016
GCA_001959615.1	9166	2	Wenzhou picornalike virus 5	WZSLuoI84329	Viruses; unclassified RNA viruses; unclassified RNA viruses ShiM-2016
GCA_001967775.1	12660	2	Wenzhou picornalike virus 51	WZSBei69571	Viruses; unclassified RNA viruses; unclassified RNA viruses ShiM-2016
GCA_001968275.1	8991	2	Wenzhou picornalike virus 52	beimix75583	Viruses; unclassified RNA viruses; unclassified RNA viruses ShiM-2016
GCA_001957815.1	9388	2	Wenzhou picornalike virus 53	WZSLuoI86067	Viruses; unclassified RNA viruses; unclassified RNA viruses ShiM-2016
GCA_001959595.1	7500	1	Wenzhou picornalike virus 54	WZSBei68749	Viruses; unclassified RNA viruses; unclassified RNA viruses ShiM-2016
GCA_001967755.1	9549	2	Wenzhou picornalike virus 6	WZSBei69574	Viruses; unclassified RNA viruses; unclassified RNA viruses ShiM-2016
GCA_001968255.1	8783	2	Wenzhou picornalike virus 7	shrimp14504	Viruses; unclassified RNA viruses; unclassified RNA viruses ShiM-2016

gca	genome.size	proteome.size	phage.name	strain	Taxonomy
GCA_001957795.1	9171	2	Wenzhou picornalike virus 9	WZSBei69430	Viruses; unclassified RNA viruses; unclassified RNA viruses ShiM-2016
GCA_001959575.1	7812	2	Wenzhou qinviruslike virus 1	WZSLuoI86141	Viruses; unclassified RNA viruses; unclassified RNA viruses ShiM-2016
GCA_001967735.1	8052	2	Wenzhou qinviruslike virus 2	WZRBX42684	Viruses; unclassified RNA viruses; unclassified RNA viruses ShiM-2016
GCA_001968235.1	3195	1	Wenzhou shrimp virus 10	WZRBX43419	Viruses; unclassified RNA viruses; unclassified RNA viruses ShiM-2016
GCA_001957775.1	10472	5	Wenzhou shrimp virus 3	shrimp12824	Viruses; unclassified RNA viruses; unclassified RNA viruses ShiM-2016
GCA_001957315.1	8636	2	Wenzhou shrimp virus 4	WZRBX43306	Viruses; unclassified RNA viruses; unclassified RNA viruses ShiM-2016
GCA_001967715.1	8524	2	Wenzhou shrimp virus 5	shrimp14323	Viruses; unclassified RNA viruses; unclassified RNA viruses ShiM-2016
GCA_001968215.1	10378	2	Wenzhou shrimp virus 6	WZRBX43423	Viruses; unclassified RNA viruses; unclassified RNA viruses ShiM-2016
GCA_001925895.1	9997	2	Wenzhou shrimp virus 7	beimix73547	Viruses; unclassified RNA viruses; unclassified RNA viruses ShiM-2016
GCA_001957755.1	10445	1	Wenzhou shrimp virus 8	shrimp14543	Viruses; unclassified RNA viruses; unclassified RNA viruses ShiM-2016
GCA_001957295.1	4609	3	Wenzhou shrimp virus 9	shrimp6189	Viruses; unclassified RNA viruses; unclassified RNA viruses ShiM-2016
GCA_001967695.1	4225	1	Wenzhou sobemolike virus 1	WZFSL61418	Viruses; unclassified RNA viruses; unclassified RNA viruses ShiM-2016
GCA_001968195.1	2748	2	Wenzhou sobemolike virus 2	mosZJ15137	Viruses; unclassified RNA viruses; unclassified RNA viruses ShiM-2016
GCA_001957735.1	2841	3	Wenzhou sobemolike virus 3	mosZJ35256	Viruses; unclassified RNA viruses; unclassified RNA viruses ShiM-2016
GCA_001957275.1	2961	2	Wenzhou sobemolike virus 4	mosZJ35391	Viruses; unclassified RNA viruses; unclassified RNA viruses ShiM-2016
GCA_001970265.1	10698	5	Wenzhou tapeworm virus 1	SGJSC14943	Viruses; unclassified RNA viruses; unclassified RNA viruses ShiM-2016
GCA_001970565.1	2841	2	Wenzhou tombuslike virus 1	WZFSL78713	Viruses; unclassified RNA viruses; unclassified RNA viruses ShiM-2016
GCA_001970725.1	4443	2	Wenzhou tombuslike virus 10	WZFSL57346	Viruses; unclassified RNA viruses; unclassified RNA viruses ShiM-2016
GCA_001970405.1	4931	3	Wenzhou tombuslike virus 11	mosZJ33874	Viruses; unclassified RNA viruses; unclassified RNA viruses ShiM-2016
GCA_001970705.1	4884	4	Wenzhou tombuslike virus 12	WZFSL84432	Viruses; unclassified RNA viruses; unclassified RNA viruses ShiM-2016
GCA_001970545.1	5144	3	Wenzhou tombuslike virus 14	WZFSL15005	Viruses; unclassified RNA viruses; unclassified RNA viruses ShiM-2016
GCA_001970245.1	4739	2	Wenzhou tombuslike virus 15	WZFSL70232	Viruses; unclassified RNA viruses; unclassified RNA viruses ShiM-2016
GCA_001970225.1	4467	1	Wenzhou tombuslike virus 16	WZFSL78689	Viruses; unclassified RNA viruses; unclassified RNA viruses ShiM-2016
GCA_001970685.1	2696	2	Wenzhou tombuslike virus 17	WZFSL82820	Viruses; unclassified RNA viruses; unclassified RNA viruses ShiM-2016
GCA_001970385.1	3883	3	Wenzhou tombuslike virus 18	beimix29339	Viruses; unclassified RNA viruses; unclassified RNA viruses ShiM-2016
GCA_001970525.1	2945	2	Wenzhou tombuslike virus 2	WZFSL84050	Viruses; unclassified RNA viruses; unclassified RNA viruses ShiM-2016
GCA_001970825.1	2847	3	Wenzhou tombuslike virus 3	WZFSL80503	Viruses; unclassified RNA viruses; unclassified RNA viruses ShiM-2016
GCA_001970665.1	5558	2	Wenzhou tombuslike virus 4	shrimp14425	Viruses; unclassified RNA viruses; unclassified RNA viruses ShiM-2016
GCA_001970365.1	4112	3	Wenzhou tombuslike virus 5	WZFSL67420	Viruses; unclassified RNA viruses; unclassified RNA viruses ShiM-2016
GCA_001970505.1	5659	3	Wenzhou tombuslike virus 6	WZFSL85189	Viruses; unclassified RNA viruses; unclassified RNA viruses ShiM-2016
GCA_001970805.1	2790	2	Wenzhou tombuslike virus 7	WZFSL44625	Viruses; unclassified RNA viruses; unclassified RNA viruses ShiM-2016
GCA_001970645.1	4683	2	Wenzhou tombuslike virus 8	beimix73523	Viruses; unclassified RNA viruses; unclassified RNA viruses ShiM-2016
GCA_001970345.1	4947	3	Wenzhou tombuslike virus 9	WZSLuoI85212	Viruses; unclassified RNA viruses; unclassified RNA viruses ShiM-2016
GCA_001926655.1	8000	2	Wenzhou totilike virus 1	WZRBX43319	Viruses; unclassified RNA viruses; unclassified RNA viruses ShiM-2016

gca	genome.size	proteome.size	phage.name	strain	Taxonomy
GCA_001970485.1	4635	2	Wenzhou weiviruslike virus 1	beimix75799	Viruses; unclassified RNA viruses; unclassified RNA viruses ShiM-2016
GCA_001970785.1	6003	2	Wenzhou yanviruslike virus 1	WZFSL78003	Viruses; unclassified RNA viruses; unclassified RNA viruses ShiM-2016
GCA_001970625.1	6271	2	Wenzhou yanviruslike virus 2	beimix74779	Viruses; unclassified RNA viruses; unclassified RNA viruses ShiM-2016
GCA_001970325.1	14984	1	Wuchan romanomermis nematode virus 1	WCLSXC58279	Viruses; unclassified RNA viruses; unclassified RNA viruses ShiM-2016
GCA_001970765.1	4267	4	Wuchan romanomermis nematode virus 3	WCLSXC83893	Viruses; unclassified RNA viruses; unclassified RNA viruses ShiM-2016
GCA_001970605.1	2972	1	Wuchang cockroach Virus 4	ZL17511	Viruses; unclassified RNA viruses; unclassified RNA viruses ShiM-2016
GCA_001970305.1	6505	4	Wuhan Millipede virus 4	GCM8225	Viruses; unclassified RNA viruses; unclassified RNA viruses ShiM-2016
GCA_001970445.1	12209	8	Wuhan arthropod virus 1	WHCC81862	Viruses; unclassified RNA viruses; unclassified RNA viruses ShiM-2016
GCA_001970745.1	9876	2	Wuhan arthropod virus 2	WHSF0	Viruses; unclassified RNA viruses; unclassified RNA viruses ShiM-2016
GCA_001970585.1	9956	3	Wuhan arthropod virus 3	WHCC110739	Viruses; unclassified RNA viruses; unclassified RNA viruses ShiM-2016
GCA_001970285.1	4504	3	Wuhan arthropod virus 4	WHCC117028	Viruses; unclassified RNA viruses; unclassified RNA viruses ShiM-2016
GCA_001970425.1	10182	1	Wuhan coneheads virus 1	ZCM4592	Viruses; unclassified RNA viruses; unclassified RNA viruses ShiM-2016
GCA_001973835.1	11748	3	Wuhan coneheads virus 2	ZCM13613	Viruses; unclassified RNA viruses; unclassified RNA viruses ShiM-2016
GCA_002004895.1	9105	6	Wuhan cricket virus 2	WHXS3745	Viruses; unclassified RNA viruses; unclassified RNA viruses ShiM-2016
GCA_001974315.1	9096	1	Wuhan fly virus 4	fly116586	Viruses; unclassified RNA viruses; unclassified RNA viruses ShiM-2016
GCA_001973995.1	3370	2	Wuhan fly virus 5	fly34516	Viruses; unclassified RNA viruses; unclassified RNA viruses ShiM-2016
GCA_001974135.1	16121	8	Wuhan heteroptera virus 1	arthropodmix8462	Viruses; unclassified RNA viruses; unclassified RNA viruses ShiM-2016
GCA_001974455.1	4982	3	Wuhan heteroptera virus 2	arthropodmix10101	Viruses; unclassified RNA viruses; unclassified RNA viruses ShiM-2016
GCA_001927155.1	29566	11	Wuhan heteroptera virus 3	WHYCM468	Viruses; unclassified RNA viruses; unclassified RNA viruses ShiM-2016
GCA_001974295.1	3042	1	Wuhan horsefly Virus 3	horsefly123863	Viruses; unclassified RNA viruses; unclassified RNA viruses ShiM-2016
GCA_001973975.1	10310	4	Wuhan house centipede virus 1	WHYY23932	Viruses; unclassified RNA viruses; unclassified RNA viruses ShiM-2016
GCA_001974115.1	12965	1	Wuhan house centipede virus 2	arthropodmix22554	Viruses; unclassified RNA viruses; unclassified RNA viruses ShiM-2016
GCA_001974435.1	11226	1	Wuhan house centipede virus 3	WHYY18014	Viruses; unclassified RNA viruses; unclassified RNA viruses ShiM-2016
GCA_001974275.1	4420	3	Wuhan house centipede virus 4	WHYY19909	Viruses; unclassified RNA viruses; unclassified RNA viruses ShiM-2016
GCA_001973955.1	4474	3	Wuhan house centipede virus 5	WHYY23902	Viruses; unclassified RNA viruses; unclassified RNA viruses ShiM-2016
GCA_001974095.1	4746	3	Wuhan house centipede virus 6	WHWG55397	Viruses; unclassified RNA viruses; unclassified RNA viruses ShiM-2016
GCA_001974415.1	3491	1	Wuhan house centipede virus 9	arthropodmix13921	Viruses; unclassified RNA viruses; unclassified RNA viruses ShiM-2016
GCA_001974255.1	9939	1	Wuhan insect virus 10	WHCCII13330	Viruses; unclassified RNA viruses; unclassified RNA viruses ShiM-2016
GCA_001973935.1	9952	2	Wuhan insect virus 11	CJLX30623	Viruses; unclassified RNA viruses; unclassified RNA viruses ShiM-2016
GCA_002008615.1	11094	1	Wuhan insect virus 12	arthropodmix13526	Viruses; unclassified RNA viruses; unclassified RNA viruses ShiM-2016
GCA_001974155.1	9733	1	Wuhan insect virus 13	CC64511	Viruses; unclassified RNA viruses; unclassified RNA viruses ShiM-2016
GCA_001974075.1	12473	1	Wuhan insect virus 14	WHZM10168	Viruses; dsRNA viruses; Hypoviridae; unclassified Hypoviridae
GCA_001974395.1	7490	2	Wuhan insect virus 15	WHCCII13252	Viruses; unclassified RNA viruses; unclassified RNA viruses ShiM-2016
GCA_001974235.1	2976	2	Wuhan insect virus 17	WHCCII1277	Viruses; unclassified RNA viruses; unclassified RNA viruses ShiM-2016

gca	genome.size	proteome.size	phage.name	strain	Taxonomy
GCA_001973915.1	2714	1	Wuhan insect virus 18	CC63583	Viruses; unclassified RNA viruses; unclassified RNA viruses ShiM-2016
GCA_001974055.1	15441	3	Wuhan insect virus 19	WHCCII13334	Viruses; unclassified RNA viruses; unclassified RNA viruses ShiM-2016
GCA_001974375.1	4975	3	Wuhan insect virus 20	WHCCII13322	Viruses; unclassified RNA viruses; unclassified RNA viruses ShiM-2016
GCA_001974215.1	5981	4	Wuhan insect virus 21	WHCCII13077	Viruses; unclassified RNA viruses; unclassified RNA viruses ShiM-2016
GCA_002008815.1	3635	2	Wuhan insect virus 22	arthropodmix13806	Viruses; unclassified RNA viruses; unclassified RNA viruses ShiM-2016
GCA_001921615.1	2858	2	Wuhan insect virus 23	WHCCII13263	Viruses; unclassified RNA viruses; unclassified RNA viruses ShiM-2016
GCA_001973895.1	4571	2	Wuhan insect virus 26	WHZM10161	Viruses; unclassified RNA viruses; unclassified RNA viruses ShiM-2016
GCA_001974035.1	4605	2	Wuhan insect virus 27	WHZM10130	Viruses; unclassified RNA viruses; unclassified RNA viruses ShiM-2016
GCA_001974355.1	6718	2	Wuhan insect virus 28	WHZM10169	Viruses; unclassified RNA viruses; unclassified RNA viruses ShiM-2016
GCA_001974195.1	7404	2	Wuhan insect virus 31	WHZM10182	Viruses; unclassified RNA viruses; unclassified RNA viruses ShiM-2016
GCA_002004235.1	10232	2	Wuhan insect virus 33	WHCCII11871	Viruses; unclassified RNA viruses; unclassified RNA viruses ShiM-2016
GCA_002003915.1	2836	2	Wuhan insect virus 34	CC64594	Viruses; unclassified RNA viruses; unclassified RNA viruses ShiM-2016
GCA_002004535.1	4388	3	Wuhan insect virus 35	NA	Viruses; unclassified RNA viruses; unclassified RNA viruses ShiM-2016
GCA_002004875.1	10644	3	Wuhan insect virus 8	WHCCII10272	Viruses; unclassified RNA viruses; unclassified RNA viruses ShiM-2016
GCA_002004215.1	9305	3	Wuhan insect virus 9	WHCCII13328	Viruses; unclassified RNA viruses; unclassified RNA viruses ShiM-2016
GCA_001921315.1	2838	2	Wuhan large pig roundworm virus 1	WHZHC73278	Viruses; unclassified RNA viruses; unclassified RNA viruses ShiM-2016
GCA_002003895.1	10655	2	Wuhan millipede virus 3	WHWG56524	Viruses; unclassified RNA viruses; unclassified RNA viruses ShiM-2016
GCA_002004515.1	17562	3	Wuhan nidolike virus 1	SCM49454	Viruses; unclassified RNA viruses; unclassified RNA viruses ShiM-2016
GCA_001926315.1	20843	4	Wuhan pillworm virus 1	WHSFII20185	Viruses; unclassified RNA viruses; unclassified RNA viruses ShiM-2016
GCA_002004855.1	12548	6	Wuhan pillworm virus 2	WHSFII14871	Viruses; unclassified RNA viruses; unclassified RNA viruses ShiM-2016
GCA_002004195.1	4317	3	Wuhan pillworm virus 3	WHSFII20254	Viruses; unclassified RNA viruses; unclassified RNA viruses ShiM-2016
GCA_002003875.1	8197	1	Wuhan potylike virus 1	WHWN51517	Viruses; unclassified RNA viruses; unclassified RNA viruses ShiM-2016
GCA_001921395.1	10305	1	Wuhan spider virus 2	spider133995	Viruses; unclassified RNA viruses; unclassified RNA viruses ShiM-2016
GCA_002004495.1	9421	1	Wuhan spider virus 3	spider133889	Viruses; unclassified RNA viruses; unclassified RNA viruses ShiM-2016
GCA_002004835.1	11652	4	Wuhan spider virus 4	spider133854	Viruses; unclassified RNA viruses; unclassified RNA viruses ShiM-2016
GCA_002004175.1	11448	4	Wuhan spider virus 5	spider133684	Viruses; unclassified RNA viruses; unclassified RNA viruses ShiM-2016
GCA_002003855.1	10647	4	Wuhan spider virus 6	spider122561	Viruses; unclassified RNA viruses; unclassified RNA viruses ShiM-2016
GCA_002004475.1	2696	1	Wuhan spider virus 7	spider133569	Viruses; unclassified RNA viruses; unclassified RNA viruses ShiM-2016
GCA_002004815.1	6216	4	Wuhan spider virus 8	spider134060	Viruses; unclassified RNA viruses; unclassified RNA viruses ShiM-2016
GCA_002004155.1	4059	4	Wuhan spider virus 9	spider112003	Viruses; unclassified RNA viruses; unclassified RNA viruses ShiM-2016
GCA_002003835.1	9961	1	Wuhan spirurian nematodes virus 1	WHSWHC143244	Viruses; unclassified RNA viruses; unclassified RNA viruses ShiM-2016
GCA_001925875.1	11374	3	Xingshan nematode virus 1	XSNXC21749	Viruses; unclassified RNA viruses; unclassified RNA viruses ShiM-2016
GCA_002004455.1	11330	3	Xingshan nematode virus 2	XSNXC13152	Viruses; unclassified RNA viruses; unclassified RNA viruses ShiM-2016
GCA_002004795.1	11743	5	Xingshan nematode virus 4	XSNXC32924	Viruses; unclassified RNA viruses; unclassified RNA viruses ShiM-2016

gca	genome.size	proteome.size	phage.name	strain	Taxonomy
GCA_002005095.1	4284	3	Xingshan nematode virus 5	XSNXC27943	Viruses; unclassified RNA viruses; unclassified RNA viruses ShiM-2016
GCA_002004775.1	6527	3	Xingshan nematode virus 6	XSNXC32793	Viruses; unclassified RNA viruses; unclassified RNA viruses ShiM-2016
GCA_002004115.1	9314	3	Xinzhou dimarhabdovirus virus 1	XZSJSC65538	Viruses; unclassified RNA viruses; unclassified RNA viruses ShiM-2016
GCA_002004735.1	11525	3	Xinzhou nematode virus 1	XZSJSC65770	Viruses; unclassified RNA viruses; unclassified RNA viruses ShiM-2016
GCA_002005075.1	9547	2	Xinzhou nematode virus 2	XZSJSC33555	Viruses; unclassified RNA viruses; unclassified RNA viruses ShiM-2016
GCA_002004755.1	7661	2	Xinzhou nematode virus 3	XZSJSC65579	Viruses; unclassified RNA viruses; unclassified RNA viruses ShiM-2016
GCA_002004095.1	11731	5	Xinzhou nematode virus 4	XZSJSC65771	Viruses; unclassified RNA viruses; unclassified RNA viruses ShiM-2016
GCA_002004715.1	7977	2	Xinzhou nematode virus 5	XZSJSC65765	Viruses; unclassified RNA viruses; unclassified RNA viruses ShiM-2016
GCA_002005055.1	3243	2	Xinzhou nematode virus 7	XZSJSC65582	Viruses; unclassified RNA viruses; unclassified RNA viruses ShiM-2016
GCA_001921495.1	2745	2	Xinzhou partitilike virus 1	XZSJSC65291	Viruses; unclassified RNA viruses; unclassified RNA viruses ShiM-2016
GCA_002004395.1	30353	4	Xinzhou torolike virus	XZSJSC65757	Viruses; unclassified RNA viruses; unclassified RNA viruses ShiM-2016
GCA_002004075.1	9558	1	Zhejiang mosquito virus 1	mosZJ35497	Viruses; unclassified RNA viruses; unclassified RNA viruses ShiM-2016
GCA_001926635.1	3226	2	Zhejiang mosquito virus 3	mosZJ35354	Viruses; unclassified RNA viruses; unclassified RNA viruses ShiM-2016
GCA_001927135.1	2629	1	narnalike virus 6	WGML136220	Viruses; unclassified RNA viruses; unclassified RNA viruses ShiM-2016
GCA_002004695.1	8824	2	ubei picornalike virus 3	WHCC101453	Viruses; unclassified RNA viruses; unclassified RNA viruses ShiM-2016
GCA_002617345.1	279845	289	Ralstonia phage RP12	NA	Viruses; Duplodnaviria; Heunggongvirae; Uroviricota; Caudoviricetes; Caudovirales; Myoviridae; Ripduovirus; Ralstonia virus RP12
GCA_002617365.1	276958	287	Ralstonia phage RP31	NA	Viruses; Duplodnaviria; Heunggongvirae; Uroviricota; Caudoviricetes; Caudovirales; Myoviridae; Ripduovirus; unclassified Ripduovirus
GCA_001904845.1	33562	34	Simian mastadenovirus WIV19	NA	Viruses; dsDNA viruses, no RNA stage; Adenoviridae; Mastadenovirus; unclassified Simian adenoviruses
GCA_006436595.1	31314	25	Pigeon adenovirus 2a	M144	Viruses; Varidnaviria; Bamfordvirae; Preplasmiviricota; Tectiliviricetes; Rowavirales; Adenoviridae; Aviadenovirus; unclassified Aviadenovirus
GCA_000862565.1	9663	1	Cocksfoot streak virus	NA	Viruses; ssRNA viruses; ssRNA positive-strand viruses, no DNA stage; Potyviridae; Potyvirus
GCA_001907925.1	5186	7	Leptonychotes weddellii polyomavirus 1	NA	Viruses; dsDNA viruses, no RNA stage; Polyomaviridae; unclassified Polyomaviridae
GCA_001907845.1	1365	1	Vernonia crinkle betasatellite	NA	Viruses; Tolecusatellitidae; Betasatellite; unclassified Betasatellite
GCA_001907745.1	2791	6	Vernonia crinkle virus	NA	Viruses; ssDNA viruses; Geminiviridae; Begomovirus; unclassified Begomovirus
GCA_018580755.1	6844	1	Chuvirus Mos8Chu0	NA	Viruses; unclassified viruses
GCA_000842725.1	119217	130	Phthorimaea operculella granulovirus	NA	Viruses; dsDNA viruses, no RNA stage; Baculoviridae; Betabaculovirus
GCA_001904865.1	4284	2	Desmodus rotundus parvovirus	DRA25	Viruses; ssDNA viruses; Parvoviridae; unclassified Parvoviridae
GCA_002008455.1	12639	4	Ambe virus	BeAr407981	Viruses; ssRNA viruses; ssRNA negative-strand viruses; Bunyavirales; Phenuiviridae; Phlebovirus; unclassified Phlebovirus
GCA_002008735.1	12450	4	Tapara virus	BeAr413570	Viruses; ssRNA viruses; ssRNA negative-strand viruses; Bunyavirales; Phenuiviridae; Phlebovirus; unclassified Phlebovirus
GCA_002008595.1	12896	4	Urucuri virus	BeAn100049	Viruses; ssRNA viruses; ssRNA negative-strand viruses; Bunyavirales; Phenuiviridae; Phlebovirus; unclassified Phlebovirus
GCA_900095755.1	37033	52	Pseudomonas phage vB_PaeS_PcyII40_PfII40a	NA	Viruses; Duplodnaviria; Heunggongvirae; Uroviricota; Caudoviricetes; Caudovirales; Siphoviridae; Casadabanvirus; unclassified Casadabanvirus
GCA_900199765.1	5752	7	Poophage MBI2016a	NA	Viruses; Monodnaviria; Sangervirae; Phixviricota; Malgrandaviricetes; Petitvirales; Microviridae; unclassified Microviridae
GCA_002617385.1	155053	193	Enterococcus phage EFP01	NA	Viruses; Duplodnaviria; Heunggongvirae; Uroviricota; Caudoviricetes; Caudovirales; Herelleviridae; Brockvirinae; Schiekvirus; Enterococcus virus EFP01
GCA_002815135.1	12230	5	Pteromalus puparum negativestrand RNA virus 1	NA	Viruses; Riboviria; Orthornavirae; Negarnaviricota; Haploviricotina; Monjiviricetes; Mononegavirales; Artoviridae; Peropuvirus; Pteromalus puparum peropuvirus
GCA_001923275.1	12839	1	Fusarium langsethiae hypovirus 1	NA	Viruses; dsRNA viruses; Hypoviridae; unclassified Hypoviridae

gca	genome.size	proteome.size	phage.name	strain	Taxonomy
GCA_002954975.2	59831	79	Bordetella virus LK3	NA	Viruses; Duplodnaviria; Heunggongvirae; Uroviricota; Caudoviricetes; Caudovirales; Siphoviridae; Vojvodinavirus; unclassified Vojvodinavirus
GCA_001907765.1	9756	2	Bivalve RNA virus G1	NA	Viruses; ssRNA viruses; ssRNA positive-strand viruses, no DNA stage; Picornavirales; Dicistroviridae; unclassified Dicistroviridae
GCA_001907785.1	7835	2	Bivalve RNA virus G5	NA	Viruses; ssRNA viruses; ssRNA positive-strand viruses, no DNA stage; Picornavirales; Dicistroviridae; unclassified Dicistroviridae
GCA_001907905.1	7915	1	Bivalve RNA virus G3	NA	Viruses; ssRNA viruses; ssRNA positive-strand viruses, no DNA stage; Picornavirales; unclassified Picornavirales
GCA_001907885.1	8051	2	Bivalve RNA virus G2	NA	Viruses; ssRNA viruses; ssRNA positive-strand viruses, no DNA stage; Picornavirales; unclassified Picornavirales
GCA_001907805.1	7567	1	Bivalve RNA virus G4	NA	Viruses; unclassified RNA viruses
GCA_001907865.1	7401	3	Bivalve hepelivirus G	NA	Viruses; unclassified RNA viruses
GCA_002617405.1	40551	48	Aquamicrobium phage P14	NA	Viruses; Duplodnaviria; Heunggongvirae; Uroviricota; Caudoviricetes; Caudovirales; Autographiviridae; Aqualcavirus; Aquamicrobium virus P14
GCA_002617425.3	40751	53	Pectobacterium phage PP81	NA	Viruses; Duplodnaviria; Heunggongvirae; Uroviricota; Caudoviricetes; Caudovirales; Autographiviridae; Studiervirinae; Pektosvirus; unclassified Pektosvirus; Pectobacterium virus PP81
GCA_002617445.1	46335	65	Pasteurella phage PMPGADVASUIND	NA	Viruses; Duplodnaviria; Heunggongvirae; Uroviricota; Caudoviricetes; Caudovirales; Siphoviridae; unclassified Siphoviridae
GCA_002617465.1	50401	69	Clostridium phage CloPEP1	NA	Viruses; Duplodnaviria; Heunggongvirae; Uroviricota; Caudoviricetes; Caudovirales; Myoviridae; unclassified Myoviridae
GCA_003861635.1	46077	66	Xanthomonas phage KPhi1	NA	Viruses; Duplodnaviria; Heunggongvirae; Uroviricota; Caudoviricetes; Caudovirales; Myoviridae; unclassified Myoviridae
GCA_002617485.1	49531	90	Mycobacterium phage Petruchio	NA	Viruses; Duplodnaviria; Heunggongvirae; Uroviricota; Caudoviricetes; Caudovirales; Siphoviridae; Fromanvirus; unclassified Fromanvirus
GCA_002617505.1	50864	84	Mycobacterium phage Blue	NA	Viruses; Duplodnaviria; Heunggongvirae; Uroviricota; Caudoviricetes; Caudovirales; Siphoviridae; Fromanvirus; unclassified Fromanvirus
GCA_002617525.1	84303	155	Mycobacterium phage MrMagoo	NA	Viruses; Duplodnaviria; Heunggongvirae; Uroviricota; Caudoviricetes; Caudovirales; Siphoviridae; Mclasvirinae; Reyvirus; unclassified Reyvirus
GCA_002617545.1	69819	95	Mycobacterium phage Rich	NA	Viruses; Duplodnaviria; Heunggongvirae; Uroviricota; Caudoviricetes; Caudovirales; Siphoviridae; Bclasvirinae; Acadianvirus; unclassified Acadianvirus
GCA_002617565.1	58200	101	Corynebacterium phage phi16	NA	Viruses; Duplodnaviria; Heunggongvirae; Uroviricota; Caudoviricetes; Caudovirales; Siphoviridae; unclassified Siphoviridae
GCA_002617585.1	51397	88	Mycobacterium phage BubbleTrouble	NA	Viruses; Duplodnaviria; Heunggongvirae; Uroviricota; Caudoviricetes; Caudovirales; Siphoviridae; Fromanvirus; unclassified Fromanvirus
GCA_002617605.1	51131	87	Mycobacterium phage Camperdownii	NA	Viruses; Duplodnaviria; Heunggongvirae; Uroviricota; Caudoviricetes; Caudovirales; Siphoviridae; Fromanvirus; unclassified Fromanvirus
GCA_002617625.1	51372	88	Mycobacterium phage Clarenza	NA	Viruses; Duplodnaviria; Heunggongvirae; Uroviricota; Caudoviricetes; Caudovirales; Siphoviridae; Fromanvirus; unclassified Fromanvirus
GCA_002617645.1	51372	88	Mycobacterium phage Funston	NA	Viruses; Duplodnaviria; Heunggongvirae; Uroviricota; Caudoviricetes; Caudovirales; Siphoviridae; Fromanvirus; unclassified Fromanvirus
GCA_002617665.1	51366	88	Mycobacterium phage JoongJeon	NA	Viruses; Duplodnaviria; Heunggongvirae; Uroviricota; Caudoviricetes; Caudovirales; Siphoviridae; Fromanvirus; unclassified Fromanvirus
GCA_002617685.1	51365	88	Mycobacterium phage KFPoly	NA	Viruses; Duplodnaviria; Heunggongvirae; Uroviricota; Caudoviricetes; Caudovirales; Siphoviridae; Fromanvirus; unclassified Fromanvirus
GCA_002617705.1	51407	88	Mycobacterium phage Kratark	NA	Viruses; Duplodnaviria; Heunggongvirae; Uroviricota; Caudoviricetes; Caudovirales; Siphoviridae; Fromanvirus; unclassified Fromanvirus
GCA_002617725.1	51370	88	Mycobacterium phage Thanksgivukkah	NA	Viruses; Duplodnaviria; Heunggongvirae; Uroviricota; Caudoviricetes; Caudovirales; Siphoviridae; Fromanvirus; unclassified Fromanvirus
GCA_001923735.1	13946	2	Soybean latent spherical virus	NA	Viruses; ssRNA viruses; ssRNA positive-strand viruses, no DNA stage; Picornavirales; Secoviridae; Comovirinae; Nepovirus; unclassified Nepovirus
GCA_009732055.1	12753	3	Ananindeua virus	NA	Viruses; Riboviria; Orthornavirae; Negarnaviricota; Polyploviricotina; Ellioviricetes; Bunyavirales; Peribunyaviridae; Orthobunyavirus; Guama orthobunyavirus
GCA_009732075.1	12607	3	Mirim virus	BEAN7722	Viruses; Riboviria; Orthornavirae; Negarnaviricota; Polyploviricotina; Ellioviricetes; Bunyavirales; Peribunyaviridae; Orthobunyavirus; Bertioga orthobunyavirus
GCA_900149655.1	3669	3	LeviOr01 phage	NA	Viruses; Riboviria; Orthornavirae; Lenarviricota; Allassoviricetes; Levivirales; Leviviridae; unclassified Leviviridae
GCA_003032665.1	15444	8	Avian paramyxovirus 14	NA	Viruses; Riboviria; Orthornavirae; Negarnaviricota; Haploviricotina; Monjiviricetes; Mononegavirales; Paramyxoviridae; Avulavirinae; Metaavulavirus; Avian metaavulavirus 14
GCA_003290305.1	77937	96	Lactobacillus phage P2	NA	Viruses; Duplodnaviria; Heunggongvirae; Uroviricota; Caudoviricetes; Caudovirales; Siphoviridae; Tybeckvirinae; Maenadvirus; unclassified Maenadvirus
GCA_000874845.1	10441	4	Bear Canyon mammarenavirus	AV A0070039	Viruses; ssRNA viruses; ssRNA negative-strand viruses; Arenaviridae; Mammarenavirus
GCA_002149865.1	3256	1	Swagivirus HG	B7Hg_S7_0	Viruses; unclassified viruses
GCA_002005035.1	7414	1	Enterovirus AN12	NA	Viruses; ssRNA viruses; ssRNA positive-strand viruses, no DNA stage; Picornavirales; Picornaviridae; Enterovirus; unclassified Enterovirus

gca	genome.size	proteome.size	phage.name	strain	Taxonomy
GCA_004287915.1	7203	7	Human papillomavirus sp	NA	Viruses; Monodnaviria; Shotokuvirae; Cossaviricota; Papovaviricetes; Zurhausenvirales; Papillomaviridae; unclassified Papillomaviridae; Human papillomavirus types
GCA_002617745.1	41854	59	Salmonella phage LPSE1	NA	Viruses; Duplodnaviria; Heunggongvirae; Uroviricota; Caudoviricetes; Caudovirales; Siphoviridae; Guernseyvirinae; Jerseyvirus; unclassified Jerseyvirus
GCA_013086085.1	18135	13	Rotavirus J	BO4351Ms2014	Viruses; Riboviria; Orthornavirae; Duplornaviricota; Resentoviricetes; Reovirales; Reoviridae; Sedoreovirinae; Rotavirus
GCA_002617765.1	52320	92	Mycobacterium phage Zephyr	NA	Viruses; Duplodnaviria; Heunggongvirae; Uroviricota; Caudoviricetes; Caudovirales; Siphoviridae; Fromanvirus; unclassified Fromanvirus
GCA_001926835.1	4044	6	Bermuda grass latent virus	NA	Viruses; ssRNA viruses; ssRNA positive-strand viruses, no DNA stage; Tombusviridae; Panicovirus; unclassified Panicovirus
GCA_001967675.1	2143	2	Chicken stoolassociated gemycircularvirus	RSBR2015	Viruses; unclassified viruses; Genomoviridae; Gemycircularvirus; unclassified Gemycircularvirus
GCA_001968175.1	4668	3	Chicken stoolassociated circular virus	RSBR2015	Viruses; unclassified viruses
GCA_004117635.1	4232	4	Chicken picobirnavirus	PBVCHKM3841HUN2011	Viruses; Riboviria; Orthornavirae; Pisuviricota; Duplopiviricetes; Durnavirales; Picobirnaviridae; Picobirnavirus; unclassified Picobirnavirus
GCA_018583525.1	9075	1	Sicinivirus sp	RSBR20154R	Viruses; Riboviria; Orthornavirae; Pisuviricota; Pisoniviricetes; Picornavirales; Picornaviridae; Sicinivirus; unclassified Sicinivirus
GCA_002375015.1	9591	1	Basavirus sp	NA	Viruses; unclassified viruses
GCA_002374995.1	8497	1	Rasavirus sp	NA	Viruses; unclassified viruses
GCA_002374975.1	8856	1	Husavirus sp	NA	Viruses; Riboviria; Orthornavirae; Pisuviricota; Pisoniviricetes; Picornavirales; unclassified Picornavirales; Husavirus; unclassified Husavirus
GCA_002374955.1	9570	1	Posavirus sp	NA	Viruses; unclassified viruses
GCA_001939235.1	11379	2	Tai Forest alphavirus	C21CI2004	Viruses; ssRNA viruses; ssRNA positive-strand viruses, no DNA stage; Togaviridae; Alphavirus; unclassified Alphavirus
GCA_001957335.1	10845	5	Adelphocoris suturalis virus	NA	Viruses; unclassified RNA viruses
GCA_001957715.1	3970	2	Nesidiocoris tenuis virus	NA	Viruses; unclassified RNA viruses
GCA_001968735.1	12807	4	Isaria javanica chrysovirus 1	NA	Viruses; dsRNA viruses; Chrysoviridae; Chrysovirus; unclassified Chrysovirus
GCA_004790195.1	10460	3	Ganda bee virus	NA	Viruses; Riboviria; Orthornavirae; Negarnaviricota; Polyploviricotina; Ellioviricetes; Bunyavirales; Phasmaviridae; Orthophasmavirus; Ganda orthophasmavirus
GCA_018580785.1	14130	3	Scaldis River bee virus	NA	Viruses; Riboviria; Orthornavirae; Negarnaviricota; Haploviricotina; Monjiviricetes; Jingchuvirales; Aliusviridae; Ollusvirus; Ollusvirus scaldisense
GCA_001974515.1	2738	6	Pea leaf distortion virus	NA	Viruses; ssDNA viruses; Geminiviridae; Begomovirus; unclassified Begomovirus
GCA_001995595.1	1347	1	Pea leaf distortion betasatellite	NA	Viruses; Tolecusatellitidae; Betasatellite; unclassified Betasatellite
GCA_000850825.1	4253	4	Pea enation mosaic virus2	NA	Viruses; ssRNA viruses; ssRNA positive-strand viruses, no DNA stage; Tombusviridae; Umbravirus
GCA_000852845.1	5706	5	Pea enation mosaic virus1	WSG	Viruses; ssRNA viruses; ssRNA positive-strand viruses, no DNA stage; Luteoviridae; Enamovirus
GCA_004129795.1	12774	7	Rice stripe mosaic virus	NA	Viruses; Riboviria; Orthornavirae; Negarnaviricota; Haploviricotina; Monjiviricetes; Mononegavirales; Rhabdoviridae; Cytorhabdovirus; Rice stripe mosaic cytorhabdovirus
GCA_001967655.1	10140	2	Biomphalaria virus 1	NA	Viruses; ssRNA viruses; ssRNA positive-strand viruses, no DNA stage; Picornavirales; unclassified Picornavirales
GCA_001974175.1	8999	1	Biomphalaria virus 2	NA	Viruses; ssRNA viruses; ssRNA positive-strand viruses, no DNA stage; Picornavirales; unclassified Picornavirales
GCA_001968155.1	9997	1	Biomphalaria virus 3	NA	Viruses; ssRNA viruses; ssRNA positive-strand viruses, no DNA stage; Picornavirales; unclassified Picornavirales
GCA_002617785.1	118537	157	Providencia phage vB_PreS_PR1	NA	Viruses; Duplodnaviria; Heunggongvirae; Uroviricota; Caudoviricetes; Caudovirales; Siphoviridae; Priunavirus; Providencia virus PR1
GCA_001957695.1	2464	2	Chicken associated smacovirus	RSBR20154	Viruses; unclassified viruses; Smacovirusgroup
GCA_003723375.1	40721	57	Lactobacillus phage BH1	NA	Viruses; Duplodnaviria; Heunggongvirae; Uroviricota; Caudoviricetes; Caudovirales; Siphoviridae; unclassified Siphoviridae
GCA_002617805.1	59782	78	Ochrobactrum phage POI1126	NA	Viruses; Duplodnaviria; Heunggongvirae; Uroviricota; Caudoviricetes; Caudovirales; Podoviridae; unclassified Podoviridae
GCA_002617845.1	41116	42	Klebsiella phage K52	NA	Viruses; Duplodnaviria; Heunggongvirae; Uroviricota; Caudoviricetes; Caudovirales; Autographiviridae; Studiervirinae; Przondovirus; Klebsiella virus K5-2
GCA_002617865.1	40163	42	Klebsiella phage K54	NA	Viruses; Duplodnaviria; Heunggongvirae; Uroviricota; Caudoviricetes; Caudovirales; Autographiviridae; Studiervirinae; Przondovirus; Klebsiella virus K5-4
GCA_002617885.1	59707	94	Mycobacterium phage CREW	NA	Viruses; Duplodnaviria; Heunggongvirae; Uroviricota; Caudoviricetes; Caudovirales; Siphoviridae; Timquatrovirus; unclassified Timquatrovirus

gca	genome.size	proteome.size	phage.name	strain	Taxonomy
GCA_002890195.1	18222	33	Aeropyrum globular virus 1	NA	Viruses; unclassified archaeal viruses
GCA_002114025.1	395405	465	Pacmanvirus A23	NA	Viruses; unclassified viruses
GCA_002617925.2	40995	54	Pectobacterium phage PP47	NA	Viruses; Duplodnaviria; Heunggongvirae; Uroviricota; Caudoviricetes; Caudovirales; Autographiviridae; Studiervirinae; Pektosvirus; unclassified Pektosvirus; Pectobacterium virus PP47
GCA_002617945.1	44609	56	Pectobacterium phage PP99	NA	Viruses; Duplodnaviria; Heunggongvirae; Uroviricota; Caudoviricetes; Caudovirales; Autographiviridae; Gajwadongvirus; Pectobacterium virus PP99
GCA_002617965.1	41402	49	Acinetobacter phage vB_AbaP_AS12	NA	Viruses; Duplodnaviria; Heunggongvirae; Uroviricota; Caudoviricetes; Caudovirales; Autographiviridae; Beijerinckvirinae; Friunavirus; Acinetobacter virus AS12
GCA_002617985.1	41642	51	Acinetobacter phage vB_AbaP_AS11	NA	Viruses; Duplodnaviria; Heunggongvirae; Uroviricota; Caudoviricetes; Caudovirales; Autographiviridae; Beijerinckvirinae; Friunavirus; Acinetobacter virus AS11
GCA_002618005.2	168266	266	Escherichia phage YUEEL01	NA	Viruses; Duplodnaviria; Heunggongvirae; Uroviricota; Caudoviricetes; Caudovirales; Myoviridae; Tevenvirinae; Tequatrovirus; unclassified Tequatrovirus
GCA_002618025.1	43121	49	Vibrio phage Vp670	NA	Viruses; Duplodnaviria; Heunggongvirae; Uroviricota; Caudoviricetes; Caudovirales; Autographiviridae; Colwellvirinae; Kaohsiungvirus; Vibrio virus Vp670
GCA_002618045.1	58984	101	Enterococcus phage EFP29	NA	Viruses; Duplodnaviria; Heunggongvirae; Uroviricota; Caudoviricetes; Caudovirales; Siphoviridae; Saphexavirus; unclassified Saphexavirus
GCA_002618065.1	42042	46	Ralstonia phage RSPII1	NA	Viruses; Duplodnaviria; Heunggongvirae; Uroviricota; Caudoviricetes; Caudovirales; Autographiviridae; Okabevirinae; Sukuvirus; Ralstonia virus RSPII1
GCA_002618085.1	42771	56	Yersinia phage fHeYen301	NA	Viruses; Duplodnaviria; Heunggongvirae; Uroviricota; Caudoviricetes; Caudovirales; Autographiviridae; Melnykvirinae; Pokrovskaiavirus; Yersinia virus fHeYen301
GCA_002618105.1	76483	143	Mycobacterium phage CrystalP	NA	Viruses; Duplodnaviria; Heunggongvirae; Uroviricota; Caudoviricetes; Caudovirales; Siphoviridae; Kostyavirus; unclassified Kostyavirus
GCA_002618125.1	57647	100	Mycobacterium phage Bubbles123	NA	Viruses; Duplodnaviria; Heunggongvirae; Uroviricota; Caudoviricetes; Caudovirales; Siphoviridae; Cheoctovirus; unclassified Cheoctovirus
GCA_002618145.1	236567	410	Aeromonas phage 652	NA	Viruses; Duplodnaviria; Heunggongvirae; Uroviricota; Caudoviricetes; Caudovirales; Myoviridae; Tevenvirinae; unclassified Tevenvirinae
GCA_002618165.1	46349	83	Aeromonas phage 3	NA	Viruses; Duplodnaviria; Heunggongvirae; Uroviricota; Caudoviricetes; Caudovirales; Myoviridae; unclassified Myoviridae
GCA_002618185.1	172957	245	Aeromonas phage 312	NA	Viruses; Duplodnaviria; Heunggongvirae; Uroviricota; Caudoviricetes; Caudovirales; Myoviridae; Biquartavirus; unclassified Biquartavirus
GCA_002618205.1	48252	83	Aeromonas phage 32	NA	Viruses; Duplodnaviria; Heunggongvirae; Uroviricota; Caudoviricetes; Caudovirales; Myoviridae; unclassified Myoviridae
GCA_002618225.1	173590	253	Aeromonas phage 44RR28t2	NA	Viruses; Duplodnaviria; Heunggongvirae; Uroviricota; Caudoviricetes; Caudovirales; Myoviridae; Biquartavirus; unclassified Biquartavirus
GCA_002618245.1	43551	84	Aeromonas phage 51	NA	Viruses; Duplodnaviria; Heunggongvirae; Uroviricota; Caudoviricetes; Caudovirales; Myoviridae; Popoffvirus; unclassified Popoffvirus
GCA_002618265.1	43551	84	Aeromonas phage 56	NA	Viruses; Duplodnaviria; Heunggongvirae; Uroviricota; Caudoviricetes; Caudovirales; Myoviridae; Popoffvirus; Aeromonas virus 56
GCA_002618285.1	46057	87	Aeromonas phage 591	NA	Viruses; Duplodnaviria; Heunggongvirae; Uroviricota; Caudoviricetes; Caudovirales; Myoviridae; unclassified Myoviridae
GCA_002618305.1	47977	83	Aeromonas phage Asp37	NA	Viruses; Duplodnaviria; Heunggongvirae; Uroviricota; Caudoviricetes; Caudovirales; Myoviridae; unclassified Myoviridae
GCA_002618325.1	173578	251	Aeromonas phage L96	NA	Viruses; Duplodnaviria; Heunggongvirae; Uroviricota; Caudoviricetes; Caudovirales; Myoviridae; Biquartavirus; unclassified Biquartavirus
GCA_002618345.1	174311	249	Aeromonas phage Riv10	NA	Viruses; Duplodnaviria; Heunggongvirae; Uroviricota; Caudoviricetes; Caudovirales; Myoviridae; Biquartavirus; unclassified Biquartavirus
GCA_002618365.1	173097	249	Aeromonas phage SW699	NA	Viruses; Duplodnaviria; Heunggongvirae; Uroviricota; Caudoviricetes; Caudovirales; Myoviridae; Biquartavirus; unclassified Biquartavirus
GCA_002954985.1	40196	22	Cafeteriavirusdependent mavirus	NA	Viruses; Varidnaviria; Bamfordvirae; Preplasmiviricota; Maveriviricetes; Priklausovirales; Lavidaviridae; Mavirus
GCA_000890715.1	19063	20	Maverickrelated virus strain Spezl	NA	Viruses; Varidnaviria; Bamfordvirae; Preplasmiviricota; Maveriviricetes; Priklausovirales; Lavidaviridae; Mavirus; Cafeteriavirus-dependent mavirus
GCA_002618385.1	146141	226	Bacillus phage vB_BsuMGoe2	NA	Viruses; Duplodnaviria; Heunggongvirae; Uroviricota; Caudoviricetes; Caudovirales; Herelleviridae; Spounavirinae; Okubovirus; unclassified Okubovirus; Bacillus virus vB_BsuM-Goe2
GCA_002618405.1	156430	246	Bacillus phage vB_BsuMGoe3	NA	Viruses; Duplodnaviria; Heunggongvirae; Uroviricota; Caudoviricetes; Caudovirales; Herelleviridae; Bastillevirinae; unclassified Bastillevirinae; Bacillus virus vB_BsuM-Goe3
GCA_002618425.1	39558	70	Proteus phage vB_PvuS_Pm34	NA	Viruses; Duplodnaviria; Heunggongvirae; Uroviricota; Caudoviricetes; Caudovirales; Siphoviridae; unclassified Siphoviridae
GCA_002618445.1	35736	33	Xenohaliotis phage pCXcHC2016	NA	Viruses; Duplodnaviria; Heunggongvirae; Uroviricota; Caudoviricetes; Caudovirales; Siphoviridae; unclassified Siphoviridae
GCA_002618465.1	35728	33	Xenohaliotis phage pCXcHR2015	NA	Viruses; Duplodnaviria; Heunggongvirae; Uroviricota; Caudoviricetes; Caudovirales; Siphoviridae; unclassified Siphoviridae
GCA_002618485.1	44018	55	Escherichia phage vB_EcoP_B	NA	Viruses; Duplodnaviria; Heunggongvirae; Uroviricota; Caudoviricetes; Caudovirales; Autographiviridae; Molineuxvirinae; Vectrevirus; Escherichia virus B
GCA_002618505.1	44970	59	Escherichia phage vB_EcoP_C	NA	Viruses; Duplodnaviria; Heunggongvirae; Uroviricota; Caudoviricetes; Caudovirales; Autographiviridae; Molineuxvirinae; Vectrevirus; Escherichia virus C

gca	genome.size	proteome.size	phage.name	strain	Taxonomy
GCA_002618525.1	44931	59	Escherichia phage vB_EcoP_D	NA	Viruses; Duplodnaviria; Heunggongvirae; Uroviricota; Caudoviricetes; Caudovirales; Autographiviridae; Molineuxvirinae; Vectrevirus; unclassified Vectrevirus
GCA_002618545.1	39300	48	Escherichia phage vB_EcoP_F	NA	Viruses; Duplodnaviria; Heunggongvirae; Uroviricota; Caudoviricetes; Caudovirales; Autographiviridae; Studiervirinae; Kayfunavirus; Escherichia virus F
GCA_002618585.1	44941	60	Escherichia phage vB_EcoP_R	NA	Viruses; Duplodnaviria; Heunggongvirae; Uroviricota; Caudoviricetes; Caudovirales; Autographiviridae; Molineuxvirinae; Vectrevirus; unclassified Vectrevirus
GCA_002618605.1	41519	68	Escherichia phage G AB2017	NA	Viruses; Duplodnaviria; Heunggongvirae; Uroviricota; Caudoviricetes; Caudovirales; Siphoviridae; Guernseyvirinae; Kagunavirus; unclassified Kagunavirus
GCA_002618625.1	41039	67	Escherichia phage L AB2017	NA	Viruses; Duplodnaviria; Heunggongvirae; Uroviricota; Caudoviricetes; Caudovirales; Siphoviridae; Guernseyvirinae; Kagunavirus; unclassified Kagunavirus
GCA_002618645.1	41184	62	Escherichia phage P AB2017	NA	Viruses; Duplodnaviria; Heunggongvirae; Uroviricota; Caudoviricetes; Caudovirales; Siphoviridae; Guernseyvirinae; Kagunavirus; unclassified Kagunavirus
GCA_002831345.1	12095	3	Guajara orthobunyavirus	BeAn 10615	Viruses; Riboviria; Orthornavirae; Negarnaviricota; Polyploviricotina; Ellioviricetes; Bunyavirales; Peribunyaviridae; Orthobunyavirus
GCA_000854725.1	16634	4	Tomato spotted wilt tospovirus	BR01 CNPH1	Viruses; Riboviria; Orthornavirae; Negarnaviricota; Polyploviricotina; Ellioviricetes; Bunyavirales; Tospoviridae; Orthotospovirus
GCA_000858945.1	17331	5	Watermelon silver mottle tospovirus	NA	Viruses; Riboviria; Orthornavirae; Negarnaviricota; Polyploviricotina; Ellioviricetes; Bunyavirales; Tospoviridae; Orthotospovirus
GCA_002618665.1	46717	74	Escherichia phage vB_EcoSIME253	NA	Viruses; Duplodnaviria; Heunggongvirae; Uroviricota; Caudoviricetes; Caudovirales; Drexlerviridae; Braunvirinae; Rtpvirus; Escherichia virus IME253
GCA_002758295.1	69082	103	Mycobacterium phage Ashraf	NA	Viruses; Duplodnaviria; Heunggongvirae; Uroviricota; Caudoviricetes; Caudovirales; Siphoviridae; Bclasvirinae; Pegunavirus; Mycobacterium virus Oline
GCA_002758315.1	69082	103	Mycobacterium phage Maskar	NA	Viruses; Duplodnaviria; Heunggongvirae; Uroviricota; Caudoviricetes; Caudovirales; Siphoviridae; Bclasvirinae; Pegunavirus; Mycobacterium virus Oline
GCA_002758335.1	69082	103	Mycobacterium phage ImtiyazSitla	NA	Viruses; Duplodnaviria; Heunggongvirae; Uroviricota; Caudoviricetes; Caudovirales; Siphoviridae; Bclasvirinae; Pegunavirus; Mycobacterium virus Oline
GCA_002618685.1	57165	72	Streptomyces phage BabyGotBac	NA	Viruses; Duplodnaviria; Heunggongvirae; Uroviricota; Caudoviricetes; Caudovirales; Siphoviridae; Woodruffvirus; unclassified Woodruffvirus
GCA_002618705.1	32296	48	Streptococcus phage Str03	NA	Viruses; Duplodnaviria; Heunggongvirae; Uroviricota; Caudoviricetes; Caudovirales; Siphoviridae; unclassified Siphoviridae
GCA_002618725.1	37030	54	Streptococcus phage Str01	NA	Viruses; Duplodnaviria; Heunggongvirae; Uroviricota; Caudoviricetes; Caudovirales; Siphoviridae; unclassified Siphoviridae
GCA_002955005.1	78536	148	Mycobacterium phage EniyanLRS	NA	Viruses; Duplodnaviria; Heunggongvirae; Uroviricota; Caudoviricetes; Caudovirales; Siphoviridae; Wildcatvirus; unclassified Wildcatvirus
GCA_002955015.1	56335	103	Mycobacterium phage SimranZ1	NA	Viruses; Duplodnaviria; Heunggongvirae; Uroviricota; Caudoviricetes; Caudovirales; Siphoviridae; Cheoctovirus; unclassified Cheoctovirus
GCA_002618745.1	15556	26	Arthrobacter phage Chestnut	NA	Viruses; Duplodnaviria; Heunggongvirae; Uroviricota; Caudoviricetes; Caudovirales; Siphoviridae; Decurrovirus; unclassified Decurrovirus
GCA_002618765.1	44094	56	Klebsiella phage vB_KpnP_KpV74	NA	Viruses; Duplodnaviria; Heunggongvirae; Uroviricota; Caudoviricetes; Caudovirales; Autographiviridae; Slopekvirinae; Drulisvirus; Klebsiella virus KpV74
GCA_002618785.1	44234	70	Escherichia phage vB_EcoS_CEB_EC3a	NA	Viruses; Duplodnaviria; Heunggongvirae; Uroviricota; Caudoviricetes; Caudovirales; Drexlerviridae; Braunvirinae; Guelphvirus; Escherichia virus EC3a
GCA_002643815.1	17634	11	Rotavirus A RVAHumanwtNPL07N17602007G26P19	NA	Viruses; Riboviria; Orthornavirae; Duplornaviricota; Resentoviricetes; Reovirales; Reoviridae; Sedoreovirinae; Rotavirus; Rotavirus A; Rotavirus A isolates
GCA_900156925.2	40525	61	Salmonella virus BTP1	NA	Viruses; Duplodnaviria; Heunggongvirae; Uroviricota; Caudoviricetes; Caudovirales; Podoviridae; Lederbergvirus
GCA_001973855.1	8566	2	Trichoderma atroviride mycovirus	NA	Viruses; dsRNA viruses; unclassified dsRNA viruses
GCA_002618805.1	43922	66	Staphylococcus phage 3 AJ2017	NA	Viruses; Duplodnaviria; Heunggongvirae; Uroviricota; Caudoviricetes; Caudovirales; Siphoviridae; Biseptimavirus; unclassified Biseptimavirus
GCA_003729215.1	6647	3	Alternaria alternata fusarivirus 1	NA	Viruses; unclassified viruses
GCA_001973875.1	11404	2	Petunia chlorotic mottle virus	NA	Viruses; ssRNA viruses; ssRNA positive-strand viruses, no DNA stage; Picornavirales; Secoviridae; Comovirinae; Nepovirus; unclassified Nepovirus
GCA_002004415.1	10017	1	Helicoverpa armigera iflavirus	HBLF20131	Viruses; ssRNA viruses; ssRNA positive-strand viruses, no DNA stage; Picornavirales; Iflaviridae; Iflavirus; unclassified Iflavirus
GCA_002288795.1	7627	3	Black currant leaf chlorosis associated virus	NA	Viruses; ssRNA viruses; ssRNA positive-strand viruses, no DNA stage; Idaeovirus; unclassified Idaeovirus
GCA_002004375.1	3728	2	Spinach cryptic virus 1	NA	Viruses; dsRNA viruses; Partitiviridae; unclassified Partitiviridae
GCA_002618825.1	239276	209	Vibrio phage pTD1	NA	Viruses; Duplodnaviria; Heunggongvirae; Uroviricota; Caudoviricetes; Caudovirales; Myoviridae; Tidunavirus; Vibrio virus pTD1
GCA_002004055.1	4100	3	Jingmen tombuslike virus 2	JMYJCC11049	Viruses; unclassified RNA viruses; unclassified RNA viruses ShiM-2016
GCA_002368945.1	7366	2	Hubei qinviruslike virus 1	SCM49583	Viruses; Riboviria; Orthornavirae; Negarnaviricota; Haploviricotina; Chunqiuviricetes; Muvirales; Qinviridae; Yingvirus; Hubei yingvirus
GCA_018583985.1	2760	7	Papaya yellow leaf curl virus	NA	Viruses; Monodnaviria; Shotokuvirae; Cressdnaviricota; Repensiviricetes; Geplafuvirales; Geminiviridae; Begomovirus

gca	genome.size	proteome.size	phage.name	strain	Taxonomy
GCA_002004675.1	7533	4	Cacao mild mosaic virus	NA	Viruses; Riboviria; Pararnavirae; Artverviricota; Revtraviricetes; Ortervirales; Caulimoviridae; Badnavirus
GCA_002003795.1	173861	132	Murine roseolovirus	NA	Viruses; dsDNA viruses, no RNA stage; Herpesvirales; Herpesviridae; Betaherpesvirinae; Roseolovirus; unclassified Roseolovirus
GCA_003389895.1	4305	2	Parvoviridae sp	NA	Viruses; Monodnaviria; Shotokuvirae; Cossaviricota; Quintoviricetes; Piccovirales; Parvoviridae; unclassified Parvoviridae
GCA_002004355.1	2208	3	Thripsassociated gemycircularvirus 1	NA	Viruses; unclassified viruses; Genomoviridae; Gemycircularvirus; unclassified Gemycircularvirus
GCA_002004035.1	2182	3	Thripsassociated gemycircularvirus 2	NA	Viruses; unclassified viruses; Genomoviridae; Gemycircularvirus; unclassified Gemycircularvirus
GCA_002004655.1	2196	3	Thripsassociated gemykibivirus 1	NA	Viruses; unclassified viruses; Genomoviridae; unclassified Genomoviridae; Gemykibivirus
GCA_002004995.1	2243	3	Thripsassociated gemykolovirus 1	NA	Viruses; unclassified viruses; Genomoviridae; unclassified Genomoviridae; Gemykolovirus
GCA_002004335.1	4729	4	Amphibola crenata associated bacilladnavirus 1	NA	Viruses; unassigned viruses; Bacilladnavirus; unclassified Bacilladnavirus
GCA_002004015.1	4576	4	Amphibola crenata associated bacilladnavirus 2	NA	Viruses; unassigned viruses; Bacilladnavirus; unclassified Bacilladnavirus
GCA_002004635.1	4742	5	AvonHeathcote estuary associated bacilladnavirus	NA	Viruses; unassigned viruses; Bacilladnavirus; unclassified Bacilladnavirus
GCA_000857105.1	6621	4	Fusarium graminearum dsRNA mycovirus1	DK21	Viruses; dsRNA viruses; unclassified dsRNA viruses
GCA_000845365.1	7857	7	Mirabilis mosaic virus	NA	Viruses; Riboviria; Pararnavirae; Artverviricota; Revtraviricetes; Ortervirales; Caulimoviridae; Caulimovirus
GCA_002117695.1	18748	3	Nairobi sheep disease virus	NA	Viruses; Riboviria; Orthornavirae; Negarnaviricota; Polyploviricotina; Ellioviricetes; Bunyavirales; Nairoviridae; Orthonairovirus; Nairobi sheep disease orthonairovirus
GCA_000906255.1	20070	7	Alphamesonivirus 2	A4CI2004	Viruses; Riboviria; Orthornavirae; Pisuviricota; Pisoniviricetes; Nidovirales; Mesnidovirineae; Mesoniviridae; Hexponivirinae; Alphamesonivirus; Karsalivirus
GCA_000908195.1	19979	6	Alphamesonivirus 3	E9CI2004	Viruses; Riboviria; Orthornavirae; Pisuviricota; Pisoniviricetes; Nidovirales; Mesnidovirineae; Mesoniviridae; Hexponivirinae; Alphamesonivirus; Karsalivirus
GCA_000907135.1	20074	7	Alphamesonivirus 4	F24CI2004	Viruses; Riboviria; Orthornavirae; Pisuviricota; Pisoniviricetes; Nidovirales; Mesnidovirineae; Mesoniviridae; Hexponivirinae; Alphamesonivirus; Casualivirus
GCA_013087425.1	12730	1	Agaricus bisporus endornavirus 1	NA	Viruses; Riboviria; Orthornavirae; Kitrinoviricota; Alsuviricetes; Martellivirales; Endornaviridae; Alphaendornavirus; Agaricus bisporus alphaendornavirus 1
GCA_000841725.1	42493	49	Bordetella virus BPP1	NA	Viruses; dsDNA viruses, no RNA stage; Caudovirales; Podoviridae; Bpp1virus
GCA_000843265.1	46012	67	Vibrio virus VpV262	NA	Viruses; Duplodnaviria; Heunggongvirae; Uroviricota; Caudoviricetes; Caudovirales; Podoviridae
GCA_003029405.2	5348	7	Sida bright yellow mosaic virus	NA	Viruses; Monodnaviria; Shotokuvirae; Cressdnaviricota; Repensiviricetes; Geplafuvirales; Geminiviridae; Begomovirus
GCA_003029395.1	2601	5	Sida chlorotic mottle virus	NA	Viruses; Monodnaviria; Shotokuvirae; Cressdnaviricota; Repensiviricetes; Geplafuvirales; Geminiviridae; Begomovirus
GCA_003029415.1	2813	6	Sida golden yellow spot virus	NA	Viruses; Monodnaviria; Shotokuvirae; Cressdnaviricota; Repensiviricetes; Geplafuvirales; Geminiviridae; Begomovirus
GCA_002285005.1	6564	2	Fathead minnow calicivirus	NA	Viruses; ssRNA viruses; ssRNA positive-strand viruses, no DNA stage; Caliciviridae; unclassified Caliciviridae
GCA_000853805.1	8374	1	Aichivirus B	U1	Viruses; Riboviria; Orthornavirae; Pisuviricota; Pisoniviricetes; Picornavirales; Picornaviridae; Kobuvirus
GCA_000843145.1	43769	52	Salmonella virus SP6	NA	Viruses; dsDNA viruses, no RNA stage; Caudovirales; Podoviridae; Autographivirinae; Sp6virus
GCA_002005705.1	3880	5	Clematis chlorotic mottle virus	NA	Viruses; ssRNA viruses; ssRNA positive-strand viruses, no DNA stage; Tombusviridae; unclassified Tombusviridae
GCA_008800975.1	7321	1	Human parechovirus 3	A30899	Viruses; Riboviria; Orthornavirae; Pisuviricota; Pisoniviricetes; Picornavirales; Picornaviridae; Parechovirus; Parechovirus A
GCA_001432095.1	13188	6	Datura yellow vein virus	NA	Viruses; ssRNA viruses; ssRNA negative-strand viruses; Mononegavirales; Rhabdoviridae; Nucleorhabdovirus
GCA_000895555.1	12875	7	Potato yellow dwarf virus	SYDV	Viruses; ssRNA viruses; ssRNA negative-strand viruses; Mononegavirales; Rhabdoviridae; Nucleorhabdovirus
GCA_000858845.1	1118	1	Strawberry latent ringspot virus satellite RNA	T 39	Viruses; Satellites; Satellite Nucleic Acids; Single stranded RNA satellites; Large single stranded RNA satellites
GCA_002655315.1	18584	11	Rotavirus A RVAHumanwtVNMSP1272013G1P4	NA	Viruses; Riboviria; Orthornavirae; Duplornaviricota; Resentoviricetes; Reovirales; Reoviridae; Sedoreovirinae; Rotavirus; Rotavirus A; Rotavirus G1
GCA_002655335.1	18592	11	Rotavirus A RVAHumanwtVNMSP1932013G1P4	NA	Viruses; Riboviria; Orthornavirae; Duplornaviricota; Resentoviricetes; Reovirales; Reoviridae; Sedoreovirinae; Rotavirus; Rotavirus A; Rotavirus G1
GCA_004099425.1	4423	8	Circoviridae sp	NA	Viruses; Monodnaviria; Shotokuvirae; Cressdnaviricota; Arfiviricetes; Cirlivirales; Circoviridae; unclassified Circoviridae
GCA_002667195.1	17261	11	Bovine rotavirus G6	RVACowtcINDCR2313919941997G6P1	Viruses; Riboviria; Orthornavirae; Duplornaviricota; Resentoviricetes; Reovirales; Reoviridae; Sedoreovirinae; Rotavirus; Rotavirus A; Bovine rotavirus A

gca	genome.size	proteome.size	phage.name	strain	Taxonomy
GCA_002005645.1	2784	6	Tomato leaf curl Burkina Faso virus	NA	Viruses; ssDNA viruses; Geminiviridae; Begomovirus; unclassified Begomovirus
GCA_018582835.1	10866	5	Rhabdoviridae sp	IH17	Viruses; Riboviria; Orthornavirae; Negarnaviricota; Haplovirícotina; Monjiviricetes; Mononegavirales; Rhabdoviridae; unclassified Rhabdoviridae
GCA_013086155.1	8422	1	Picornavirales sp	NA	Viruses; Riboviria; Orthornavirae; Pisuviricota; Pisoniviricetes; Picornavirales; unclassified Picornavirales
GCA_002024695.1	7615	1	Cordoba virus	GMC30	Viruses; unclassified viruses
GCA_002619005.1	42160	58	Staphylococcus phage phi575	NA	Viruses; Duplodnaviria; Heunggongvirae; Uroviricota; Caudoviricetes; Caudovirales; Siphoviridae; Biseptimavirus; unclassified Biseptimavirus
GCA_002619025.1	41448	55	Staphylococcus phage phi879	NA	Viruses; Duplodnaviria; Heunggongvirae; Uroviricota; Caudoviricetes; Caudovirales; Siphoviridae; Biseptimavirus; unclassified Biseptimavirus
GCA_002024675.1	9506	3	Big Cypress virus	BCNP224 A	Viruses; unclassified viruses
GCA_002024795.1	9111	3	Biratnagar virus	Nepal121	Viruses; unclassified viruses
GCA_013377425.1	9294	3	Fort Crockett virus	JCC1212	Viruses; unclassified viruses
GCA_002024715.1	9748	3	San Bernardo virus	CoB37dA	Viruses; unclassified viruses
GCA_002619045.1	35802	50	Pseudoalteromonas phage PHS21	NA	Viruses; Duplodnaviria; Heunggongvirae; Uroviricota; Caudoviricetes; Caudovirales; Siphoviridae; unclassified Siphoviridae
GCA_002619065.1	52338	24	Klebsiella phage 48ST307	NA	Viruses; Duplodnaviria; Heunggongvirae; Uroviricota; Caudoviricetes; Caudovirales; Siphoviridae; unclassified Siphoviridae
GCA_002619085.1	75215	146	Mycobacterium phage Maxxinista	NA	Viruses; Duplodnaviria; Heunggongvirae; Uroviricota; Caudoviricetes; Caudovirales; Siphoviridae; Kostyavirus; unclassified Kostyavirus
GCA_002619105.1	51402	89	Mycobacterium phage Tinybot	NA	Viruses; Duplodnaviria; Heunggongvirae; Uroviricota; Caudoviricetes; Caudovirales; Siphoviridae; Fromanvirus; unclassified Fromanvirus
GCA_002619125.1	46597	65	Rhodococcus phage AngryOrchard	NA	Viruses; Duplodnaviria; Heunggongvirae; Uroviricota; Caudoviricetes; Caudovirales; Siphoviridae; Rerduovirus; unclassified Rerduovirus
GCA_002619145.1	46641	65	Rhodococcus phage BobbyDazzler	NA	Viruses; Duplodnaviria; Heunggongvirae; Uroviricota; Caudoviricetes; Caudovirales; Siphoviridae; Rerduovirus; unclassified Rerduovirus
GCA_002005745.1	19272	10	Orbivirus SX2017a	NA	Viruses; dsRNA viruses; Reoviridae; Sedoreovirinae; Orbivirus; unclassified Orbivirus
GCA_002005685.1	376207	452	Noumeavirus	NA	Viruses; dsDNA viruses, no RNA stage; Marseilleviridae; unclassified Marseilleviridae
GCA_002008435.1	6612	1	Peach virus D	NA	Viruses; ssRNA viruses; ssRNA positive-strand viruses, no DNA stage; Tymovirales; Tymoviridae; Marafivirus; unclassified Marafivirus
GCA_004790235.1	12138	6	Citrus leprosis virus N	NA	Viruses; Riboviria; Orthornavirae; Negarnaviricota; Haplovirícotina; Monjiviricetes; Mononegavirales; Rhabdoviridae; Dichorhavirus; Citrus leprosis N dichorhavirus
GCA_002008535.1	7746	1	Bat sapelovirus	NA	Viruses; ssRNA viruses; ssRNA positive-strand viruses, no DNA stage; Picornavirales; Picornaviridae; Sapelovirus; unclassified Sapelovirus
GCA_002008415.1	10519	1	Bat iflavirus	NA	Viruses; ssRNA viruses; ssRNA positive-strand viruses, no DNA stage; Picornavirales; Iflaviridae; Iflavirus; unclassified Iflavirus
GCA_002008695.1	8882	2	Bat dicibavirus	NA	Viruses; unclassified viruses
GCA_002008875.1	8335	2	Bat badicivirus 1	NA	Viruses; unclassified viruses
GCA_002008675.1	8630	2	Bat badicivirus 2	NA	Viruses; unclassified viruses
GCA_006450515.1	361368	386	Kurlavirus BKC1	NA	Viruses; Varidnaviria; Bamfordvirae; Nucleocytoviricota; Megaviricetes; Pimascovirales; Marseilleviridae; Marseillevirus; unclassified Marseillevirus
GCA_002619165.1	49156	84	Mycobacterium phage Idleandcovert	NA	Viruses; Duplodnaviria; Heunggongvirae; Uroviricota; Caudoviricetes; Caudovirales; Siphoviridae; Fromanvirus; unclassified Fromanvirus
GCA_002619185.1	18546	20	Staphylococcus phage Andhra	NA	Viruses; Duplodnaviria; Heunggongvirae; Uroviricota; Caudoviricetes; Caudovirales; Podoviridae; Rakietenvirinae; Andhravirus; Staphylococcus virus Andhra
GCA_002619205.1	44936	82	Gordonia phage DinoDaryn	NA	Viruses; Duplodnaviria; Heunggongvirae; Uroviricota; Caudoviricetes; Caudovirales; Siphoviridae; Vendettavirus; unclassified Vendettavirus
GCA_002619225.1	44936	82	Gordonia phage Huffy	NA	Viruses; Duplodnaviria; Heunggongvirae; Uroviricota; Caudoviricetes; Caudovirales; Siphoviridae; Vendettavirus; unclassified Vendettavirus
GCA_002619245.1	55094	95	Mycobacterium phage SassyB	NA	Viruses; Duplodnaviria; Heunggongvirae; Uroviricota; Caudoviricetes; Caudovirales; Siphoviridae; Cheoctovirus; unclassified Cheoctovirus
GCA_002619265.1	51367	86	Mycobacterium phage TinaFeyge	NA	Viruses; Duplodnaviria; Heunggongvirae; Uroviricota; Caudoviricetes; Caudovirales; Siphoviridae; Fromanvirus; unclassified Fromanvirus
GCA_002619285.1	57408	127	Enterococcus phage EFP10	NA	Viruses; Duplodnaviria; Heunggongvirae; Uroviricota; Caudoviricetes; Caudovirales; Siphoviridae; Saphexavirus; unclassified Saphexavirus
GCA_002989985.1	59097	78	Salmonella phage BSPM4	NA	Viruses; Duplodnaviria; Heunggongvirae; Uroviricota; Caudoviricetes; Caudovirales; Siphoviridae; Chivirus; unclassified Chivirus

gca	genome.size	proteome.size	phage.name	strain	Taxonomy
GCA_002990035.1	157665	219	Salmonella phage BSP101	NA	Viruses; Duplodnaviria; Heunggongvirae; Uroviricota; Caudoviricetes; Caudovirales; Ackermannviridae; Cvivirinae; Kuttervirus; unclassified Kuttervirus
GCA_002989885.1	142778	253	Pectobacterium phage vB_PatM_CB7	NA	Viruses; Duplodnaviria; Heunggongvirae; Uroviricota; Caudoviricetes; Caudovirales; Myoviridae; Vequintavirinae; Certrevirus; unclassified Certrevirus
GCA_002989895.1	76041	97	Pectobacterium phage vB_PatP_CB1	NA	Viruses; Duplodnaviria; Heunggongvirae; Uroviricota; Caudoviricetes; Caudovirales; Podoviridae; unclassified Podoviridae
GCA_002989935.1	76169	102	Pectobacterium phage vB_PatP_CB3	NA	Viruses; Duplodnaviria; Heunggongvirae; Uroviricota; Caudoviricetes; Caudovirales; Podoviridae; unclassified Podoviridae
GCA_002989955.1	76621	100	Pectobacterium phage vB_PatP_CB4	NA	Viruses; Duplodnaviria; Heunggongvirae; Uroviricota; Caudoviricetes; Caudovirales; Podoviridae; unclassified Podoviridae
GCA_002619305.1	231998	241	Vibrio phage pVa21	NA	Viruses; Duplodnaviria; Heunggongvirae; Uroviricota; Caudoviricetes; Caudovirales; Myoviridae; unclassified Myoviridae
GCA_002619325.1	18275	21	Staphylococcus phage St 134	NA	Viruses; Duplodnaviria; Heunggongvirae; Uroviricota; Caudoviricetes; Caudovirales; Podoviridae; Rakietenvirinae; Andhravirus; Staphylococcus virus St134
GCA_002619345.1	259700	333	Erwinia phage vB_EamM_Yoloswag	NA	Viruses; Duplodnaviria; Heunggongvirae; Uroviricota; Caudoviricetes; Caudovirales; Myoviridae; Yoloswagvirus; Erwinia virus Yoloswag
GCA_002008795.1	1228	2	Cassava satellite virus	NA	Viruses; Satellites; Satellite Viruses; unclassified Satellite Viruses
GCA_002619365.1	43211	48	Ralstonia phage RSPI1	NA	Viruses; Duplodnaviria; Heunggongvirae; Uroviricota; Caudoviricetes; Caudovirales; Autographiviridae; Okabevirinae; Ampunavirus; Ralstonia virus RSPI1
GCA_010455235.1	45618	65	Paenibacillus phage phiERICV	NA	Viruses; Duplodnaviria; Heunggongvirae; Uroviricota; Caudoviricetes; Caudovirales; Myoviridae; unclassified Myoviridae
GCA_002619385.1	16271	38	Sulfolobus spindleshaped virus Lassen	NA	Viruses; Fuselloviridae; Alphafusellovirus; unclassified Alphafusellovirus
GCA_002619405.1	15230	31	Sulfolobus spindleshaped virus 3	REY 154	Viruses; Fuselloviridae; Alphafusellovirus; unclassified Alphafusellovirus
GCA_002271005.1	9426	1	Reed chlorotic stripe virus	Tianshui	Viruses; ssRNA viruses; ssRNA positive-strand viruses, no DNA stage; Potyviridae; unclassified Potyviridae
GCA_002619425.1	52715	99	Mycobacterium phage Jabith	NA	Viruses; Duplodnaviria; Heunggongvirae; Uroviricota; Caudoviricetes; Caudovirales; Siphoviridae; Fromanvirus; unclassified Fromanvirus
GCA_002619445.1	219348	347	Caulobacter phage Ccr10	NA	Viruses; Duplodnaviria; Heunggongvirae; Uroviricota; Caudoviricetes; Caudovirales; Siphoviridae; Dolichocephalovirinae; Shapirovirus; unclassified Shapirovirus
GCA_002619465.1	220299	344	Caulobacter phage Ccr2	NA	Viruses; Duplodnaviria; Heunggongvirae; Uroviricota; Caudoviricetes; Caudovirales; Siphoviridae; Dolichocephalovirinae; Shapirovirus; unclassified Shapirovirus
GCA_002619485.1	229319	361	Caulobacter phage Ccr29	NA	Viruses; Duplodnaviria; Heunggongvirae; Uroviricota; Caudoviricetes; Caudovirales; Siphoviridae; Dolichocephalovirinae; Shapirovirus; unclassified Shapirovirus
GCA_002619505.1	215799	331	Caulobacter phage Ccr32	NA	Viruses; Duplodnaviria; Heunggongvirae; Uroviricota; Caudoviricetes; Caudovirales; Siphoviridae; Dolichocephalovirinae; Shapirovirus; unclassified Shapirovirus
GCA_002619525.1	216240	339	Caulobacter phage Ccr34	NA	Viruses; Duplodnaviria; Heunggongvirae; Uroviricota; Caudoviricetes; Caudovirales; Siphoviridae; Dolichocephalovirinae; Shapirovirus; unclassified Shapirovirus
GCA_002619545.1	218729	340	Caulobacter phage Ccr5	NA	Viruses; Duplodnaviria; Heunggongvirae; Uroviricota; Caudoviricetes; Caudovirales; Siphoviridae; Dolichocephalovirinae; Shapirovirus; unclassified Shapirovirus
GCA_004045975.1	1587	1	Molossus molossus circovirus 1	NA	Viruses; Monodnaviria; Shotokuvirae; Cressdnaviricota; Arfiviricetes; Cirlivirales; Circoviridae; Circovirus; unclassified Circovirus
GCA_004045955.1	3156	2	Molossus molossus circovirus 2	NA	Viruses; Monodnaviria; Shotokuvirae; Cressdnaviricota; Arfiviricetes; Cirlivirales; Circoviridae; Circovirus; unclassified Circovirus
GCA_004045935.1	2055	2	Molossus molossus circovirus 3	NA	Viruses; Monodnaviria; Shotokuvirae; Cressdnaviricota; Arfiviricetes; Cirlivirales; Circoviridae; Circovirus; unclassified Circovirus
GCA_004045915.1	1805	2	Molossus molossus circovirus 4	NA	Viruses; Monodnaviria; Shotokuvirae; Cressdnaviricota; Arfiviricetes; Cirlivirales; Circoviridae; Circovirus; unclassified Circovirus
GCA_004130195.1	7869	6	Molossus molossus papillomavirus 1	NA	Viruses; Monodnaviria; Shotokuvirae; Cossaviricota; Papovaviricetes; Zurhausenvirales; Papillomaviridae; unclassified Papillomaviridae; non-primate mammal papillomaviruses
GCA_002005625.1	7475	2	Bat sapovirus	NA	Viruses; ssRNA viruses; ssRNA positive-strand viruses, no DNA stage; Caliciviridae; Sapovirus; unclassified Sapovirus
GCA_002118765.1	8399	3	Hom1 vesivirus	NA	Viruses; ssRNA viruses; ssRNA positive-strand viruses, no DNA stage; Caliciviridae; Vesivirus; unclassified Vesivirus
GCA_002008395.1	10101	1	Goose megrivirus	NA	Viruses; ssRNA viruses; ssRNA positive-strand viruses, no DNA stage; Picornaviridae; Picornavirales; Megrivirus; unclassified Megrivirus
GCA_009901835.1	55906	81	uncultured archaeal virus	NA	Viruses; environmental samples
GCA_002819425.1	7444	3	Sugarcane bacilliform Guadeloupe A virus	NA	Viruses; Riboviria; Pararnavirae; Artverviricota; Revtraviricetes; Ortervirales; Caulimoviridae; Badnavirus
GCA_000886875.1	7317	3	Sugarcane bacilliform Guadeloupe D virus	NA	Viruses; Riboviria; Pararnavirae; Artverviricota; Revtraviricetes; Ortervirales; Caulimoviridae; Badnavirus
GCA_002819345.1	13221	9	Cacao swollen shoot Togo A virus	NA	Viruses; Riboviria; Pararnavirae; Artverviricota; Revtraviricetes; Ortervirales; Caulimoviridae; Badnavirus
GCA_002819325.1	7203	4	Cacao swollen shoot CD virus	NA	Viruses; Riboviria; Pararnavirae; Artverviricota; Revtraviricetes; Ortervirales; Caulimoviridae; Badnavirus

gca	genome.size	proteome.size	phage.name	strain	Taxonomy
GCA_002990025.1	110741	174	Salmonella phage BSP22A	NA	Viruses; Duplodnaviria; Heunggongvirae; Uroviricota; Caudoviricetes; Caudovirales; Demerecviridae; Markadamsvirinae; Epseptimavirus; unclassified Epseptimavirus
GCA_002619565.1	29880	24	Klebsiella phage 1 LV2017	NA	Viruses; Duplodnaviria; Heunggongvirae; Uroviricota; Caudoviricetes; Caudovirales; Siphoviridae; unclassified Siphoviridae
GCA_002619585.1	44400	50	Klebsiella phage 2 LV2017	NA	Viruses; Duplodnaviria; Heunggongvirae; Uroviricota; Caudoviricetes; Caudovirales; Siphoviridae; unclassified Siphoviridae
GCA_002619605.1	44279	46	Klebsiella phage 2b LV2017	NA	Viruses; Duplodnaviria; Heunggongvirae; Uroviricota; Caudoviricetes; Caudovirales; Siphoviridae; unclassified Siphoviridae
GCA_002619625.1	35100	36	Klebsiella phage 3LV2017	NA	Viruses; Duplodnaviria; Heunggongvirae; Uroviricota; Caudoviricetes; Caudovirales; Myoviridae; Peduovirinae; Reginaelenavirus; Klebsiella virus 3LV2017
GCA_002619645.1	33540	38	Klebsiella phage 4LV2017	NA	Viruses; Duplodnaviria; Heunggongvirae; Uroviricota; Caudoviricetes; Caudovirales; Myoviridae; Peduovirinae; Felsduovirus; Klebsiella virus 4LV2017
GCA_002619665.1	47014	41	Klebsiella phage 5 LV2017	NA	Viruses; Duplodnaviria; Heunggongvirae; Uroviricota; Caudoviricetes; Caudovirales; Siphoviridae; unclassified Siphoviridae
GCA_003029545.2	5480	8	Desmodium mottle virus	NA	Viruses; Monodnaviria; Shotokuvirae; Cressdnaviricota; Repensiviricetes; Geplafuvirales; Geminiviridae; Begomovirus
GCA_018580745.1	3867	5	Jasmine virus H	NA	Viruses; Riboviria; Orthornavirae; Kitrinoviricota; Tolucaviricetes; Tolivirales; Tombusviridae; Procedovirinae; Pelarspovirus
GCA_000855605.1	1374	1	Tomato black ring virus satellite RNA	C serotype	Viruses; Satellites; Satellite Nucleic Acids; Single stranded RNA satellites; Large single stranded RNA satellites
GCA_000840145.1	2739	6	Tomato leaf curl Taiwan virus	NA	Viruses; ssDNA viruses; Geminiviridae; Begomovirus
GCA_002118725.1	4242	5	Skunk amdoparvovirus	SK23	Viruses; ssDNA viruses; Parvoviridae; Parvovirinae; Amdoparvovirus; unclassified Amdoparvovirus
GCA_002118545.1	1707	1	Picobirnavirus green monkeyKNA2015	PBVSimianKNA089842015	Viruses; dsRNA viruses; Picobirnaviridae; Picobirnavirus; unclassified Picobirnavirus
GCA_002219985.1	1689	1	Picobirnavirus dogKNA2015	PBVDogKNARVC72015	Viruses; dsRNA viruses; Picobirnaviridae; Picobirnavirus; unclassified Picobirnavirus
GCA_002008515.1	6712	3	Qinghai Himalayan marmot astrovirus 1	NA	Viruses; ssRNA viruses; ssRNA positive-strand viruses, no DNA stage; Astroviridae; Mamastrovirus; unclassified Mamastrovirus
GCA_002008655.1	6640	3	Qinghai Himalayan marmot astrovirus 2	NA	Viruses; ssRNA viruses; ssRNA positive-strand viruses, no DNA stage; Astroviridae; Mamastrovirus; unclassified Mamastrovirus
GCA_004288135.1	7217	7	Gammapapillomavirus 22	NA	Viruses; Monodnaviria; Shotokuvirae; Cossaviricota; Papovaviricetes; Zurhausenvirales; Papillomaviridae; Firstpapillomavirinae; Gammapapillomavirus
GCA_004288095.1	7193	7	Gammapapillomavirus 23	NA	Viruses; Monodnaviria; Shotokuvirae; Cossaviricota; Papovaviricetes; Zurhausenvirales; Papillomaviridae; Firstpapillomavirinae; Gammapapillomavirus
GCA_004287975.1	7324	7	Gammapapillomavirus 24	NA	Viruses; Monodnaviria; Shotokuvirae; Cossaviricota; Papovaviricetes; Zurhausenvirales; Papillomaviridae; Firstpapillomavirinae; Gammapapillomavirus
GCA_004287955.1	7291	7	Gammapapillomavirus 25	NA	Viruses; Monodnaviria; Shotokuvirae; Cossaviricota; Papovaviricetes; Zurhausenvirales; Papillomaviridae; Firstpapillomavirinae; Gammapapillomavirus
GCA_002619705.1	137836	209	Staphylococcus phage pSa3	NA	Viruses; Duplodnaviria; Heunggongvirae; Uroviricota; Caudoviricetes; Caudovirales; Herelleviridae; Twortvirinae; Kayvirus; unclassified Kayvirus
GCA_002619725.1	39906	49	Pectobacterium phage PPWS4	NA	Viruses; Duplodnaviria; Heunggongvirae; Uroviricota; Caudoviricetes; Caudovirales; Autographiviridae; Studiervirinae; Pektosvirus; Pectobacterium virus PPWS4
GCA_000906395.1	34391	36	Simian mastadenovirus C	NA	Viruses; Varidnaviria; Bamfordvirae; Preplasmiviricota; Tectiliviricetes; Rowavirales; Adenoviridae; Mastadenovirus
GCA_000843125.1	57930	85	Enterobacteria phage BP4795	NA	Viruses; dsDNA viruses, no RNA stage; Caudovirales; Siphoviridae; Lambdavirus; unclassified Lambda-like viruses
GCA_000868985.1	147544	153	Agrotis segetum nucleopolyhedrovirus A	NA	Viruses; dsDNA viruses, no RNA stage; Baculoviridae; Alphabaculovirus
GCA_002867145.1	11559	2	Aeonium ringspot virus	NA	Viruses; Riboviria; Orthornavirae; Pisuviricota; Pisoniviricetes; Picornavirales; Secoviridae; Comovirinae; Nepovirus
GCA_002619745.1	357154	549	Serratia phage BF	NA	Viruses; Duplodnaviria; Heunggongvirae; Uroviricota; Caudoviricetes; Caudovirales; Myoviridae; Eneladusvirus; Serratia virus BF
GCA_002619765.1	56075	77	Streptococcus virus MS1	NA	Viruses; Duplodnaviria; Heunggongvirae; Uroviricota; Caudoviricetes; Caudovirales; Siphoviridae; unclassified Siphoviridae
GCA_003059655.1	43337	60	Pseudomonas phage PAXYB1	NA	Viruses; Duplodnaviria; Heunggongvirae; Uroviricota; Caudoviricetes; Caudovirales; Autographiviridae; Krylovirinae; Phikmvvirus; Pseudomonas virus PAXYB1
GCA_002029595.1	10897	1	Menghai flavivirus	NA	Viruses; ssRNA viruses; ssRNA positive-strand viruses, no DNA stage; Flaviviridae; unclassified Flaviviridae
GCA_002288715.1	23307	12	Fall chinook aquareovirus	NA	Viruses; dsRNA viruses; Reoviridae; Spinareovirinae; Aquareovirus; unclassified Aquareovirus
GCA_002029515.1	5953	6	African eggplant yellowing virus	NA	Viruses; ssRNA viruses; ssRNA positive-strand viruses, no DNA stage; Luteoviridae; Polerovirus; unclassified Polerovirus
GCA_002029655.1	9555	1	Sunflower ring blotch virus	NA	Viruses; ssRNA viruses; ssRNA positive-strand viruses, no DNA stage; Potyviridae; Potyvirus; unclassified Potyvirus
GCA_000855285.1	3644	6	Beet black scorch virus	NA	Viruses; ssRNA viruses; ssRNA positive-strand viruses, no DNA stage; Tombusviridae; Betanecrovirus

gca	genome.size	proteome.size	phage.name	strain	Taxonomy
GCA_000863105.1	4194	4	Southern cowpea mosaic virus	NA	Viruses; ssRNA viruses; ssRNA positive-strand viruses, no DNA stage; Sobemovirus
GCA_002355005.1	7964	1	Falcon picornavirus	falconHA180802014HUN	Viruses; Riboviria; Orthornavirae; Pisuviricota; Pisoniviricetes; Picornavirales; Picornaviridae; unclassified Picornaviridae
GCA_002375075.1	1873	2	Circovirus sp	NA	Viruses; Monodnaviria; Shotokuvirae; Cressdnaviricota; Arfiviricetes; Cirlivirales; Circoviridae; Circovirus; unclassified Circovirus
GCA_002619805.1	60362	52	Synechococcus virus SESS1	NA	Viruses; Duplodnaviria; Heunggongvirae; Uroviricota; Caudoviricetes; Caudovirales; Siphoviridae; unclassified Siphoviridae
GCA_002194405.1	27682	9	Coronavirus AcCoVJC34	NA	Viruses; ssRNA viruses; ssRNA positive-strand viruses, no DNA stage; Nidovirales; Coronaviridae; Coronavirinae; Alphacoronavirus; unclassified Alphacoronavirus
GCA_013087445.1	3018	2	Human PoSCV5like circular virus	NA	Viruses; Monodnaviria; Shotokuvirae; Cressdnaviricota; Arfiviricetes; Cirlivirales; Circoviridae; unclassified Circoviridae
GCA_000943665.1	15684	13	DeBrazzas monkey arterivirus	NA	Viruses; Riboviria; Orthornavirae; Pisuviricota; Pisoniviricetes; Nidovirales; Arnidovirineae; Arteriviridae; Simarterivirinae; Iotaarterivirus; Debiartevirus; Iotaarterivirus debrazmo
GCA_000931135.1	14953	10	African pouched rat arterivirus	NA	Viruses; Riboviria; Orthornavirae; Pisuviricota; Pisoniviricetes; Nidovirales; Arnidovirineae; Arteriviridae; Heroarterivirinae; Lambdaarterivirus; Lambdaarterivirus afriporav
GCA_000862745.1	15428	8	Porcine reproductive and respiratory syndrome virus 1	NA	Viruses; Riboviria; Orthornavirae; Pisuviricota; Pisoniviricetes; Nidovirales; Arnidovirineae; Arteriviridae; Variarterivirinae; Betaarterivirus; Eurpobartevirus; Betaarterivirus suid 1
GCA_002816115.1	15411	9	Porcine reproductive and respiratory syndrome virus 2	ATCC VR2332	Viruses; Riboviria; Orthornavirae; Pisuviricota; Pisoniviricetes; Nidovirales; Arnidovirineae; Arteriviridae; Variarterivirinae; Betaarterivirus; Ampobartevirus; Betaarterivirus suid 2
GCA_002816155.1	15370	13	Simian hemorrhagic encephalitis virus	NA	Viruses; Riboviria; Orthornavirae; Pisuviricota; Pisoniviricetes; Nidovirales; Arnidovirineae; Arteriviridae; Simarterivirinae; Epsilonarterivirus; Sheartevirus; Epsilonarterivirus hemcep
GCA_004130415.1	9503	1	Sugar beet cyst nematode virus 1	NA	Viruses; Riboviria; Orthornavirae; Pisuviricota; Pisoniviricetes; Picornavirales; unclassified Picornavirales
GCA_002037775.1	9834	1	Pityohyphantes rubrofasciatus iflavirus	NA	Viruses; ssRNA viruses; ssRNA positive-strand viruses, no DNA stage; Picornavirales; Iflaviridae; unclassified Iflaviridae
GCA_003029575.1	44760	55	Pectobacterium phage POP72	NA	Viruses; Duplodnaviria; Heunggongvirae; Uroviricota; Caudoviricetes; Caudovirales; Autographiviridae; Molineuxvirinae; Axomammavirus; unclassified Axomammavirus
GCA_002619825.1	36671	48	Corynebacterium phage LGCMVI	NA	Viruses; Duplodnaviria; Heunggongvirae; Uroviricota; Caudoviricetes; Caudovirales; Siphoviridae; unclassified Siphoviridae
GCA_002116255.1	15098	1	Lagenaria siceraria endornavirusHubei	NA	Viruses; dsRNA viruses; Endornaviridae; unclassified Endornavirus
GCA_002080215.1	5269	5	LI polyomavirus	NA	Viruses; dsDNA viruses, no RNA stage; Polyomaviridae; unclassified Polyomaviridae
GCA_009828375.1	75764	97	Pectobacterium phage phiA41	NA	Viruses; Duplodnaviria; Heunggongvirae; Uroviricota; Caudoviricetes; Caudovirales; Podoviridae; unclassified Podoviridae
GCA_002922445.1	6125	7	Pepper vein yellows virus 5	NA	Viruses; Riboviria; Orthornavirae; Kitrinoviricota; Tolucaviricetes; Tolivirales; Luteoviridae; Polerovirus
GCA_002619845.2	47703	79	Geobacillus phage TP84	NA	Viruses; Duplodnaviria; Heunggongvirae; Uroviricota; Caudoviricetes; Caudovirales; Siphoviridae; Saundersvirus; Geobacillus virus Tp84
GCA_002619865.1	167773	280	Yersinia phage fHeYen901	NA	Viruses; Duplodnaviria; Heunggongvirae; Uroviricota; Caudoviricetes; Caudovirales; Myoviridae; Tegunavirus; unclassified Tegunavirus
GCA_002619945.1	77874	123	Dinoroseobacter phage vB_DshSR5C	NA	Viruses; Duplodnaviria; Heunggongvirae; Uroviricota; Caudoviricetes; Caudovirales; Siphoviridae; Nanhaivirus; Dinoroseobacter virus D5C
GCA_002619965.1	44244	50	Marinomonas phage CPG1g	NA	Viruses; Duplodnaviria; Heunggongvirae; Uroviricota; Caudoviricetes; Caudovirales; Autographiviridae; Colwellvirinae; Murciavirus; unclassified Murciavirus
GCA_002619985.1	44245	50	Marinomonas phage CPP1m	NA	Viruses; Duplodnaviria; Heunggongvirae; Uroviricota; Caudoviricetes; Caudovirales; Autographiviridae; Colwellvirinae; Murciavirus; Marinomonas virus CPP1m
GCA_002620005.1	43567	49	Shewanella phage SppYZU01	NA	Viruses; Duplodnaviria; Heunggongvirae; Uroviricota; Caudoviricetes; Caudovirales; Myoviridae; unclassified Myoviridae
GCA_002620025.1	84419	119	Salmonella phage Si3	NA	Viruses; Duplodnaviria; Heunggongvirae; Uroviricota; Caudoviricetes; Caudovirales; Myoviridae; Ounavirinae; Felixounavirus; Salmonella virus Si3
GCA_002620085.1	5387	11	Salmonella phage alphaalpha	NA	Viruses; Monodnaviria; Sangervirae; Phixviricota; Malgrandaviricetes; Petitvirales; Microviridae; Bullavirinae; Sinsheimervirus; unclassified Sinsheimervirus
GCA_002758355.1	68506	103	Mycobacterium phage Chorkpop	NA	Viruses; Duplodnaviria; Heunggongvirae; Uroviricota; Caudoviricetes; Caudovirales; Siphoviridae; Bclasvirinae; Pegunavirus; Mycobacterium virus Soto
GCA_002620105.1	39693	52	Streptomyces phage ToastyFinz	NA	Viruses; Duplodnaviria; Heunggongvirae; Uroviricota; Caudoviricetes; Caudovirales; Siphoviridae; Raleighvirus; unclassified Raleighvirus
GCA_002620125.1	45415	88	Acinetobacter phage WCHABP12	NA	Viruses; Duplodnaviria; Heunggongvirae; Uroviricota; Caudoviricetes; Caudovirales; Myoviridae; Obolenskvirus; Acinetobacter virus WCHABP12
GCA_002620165.1	87998	131	Citrobacter phage Mijalis	NA	Viruses; Duplodnaviria; Heunggongvirae; Uroviricota; Caudoviricetes; Caudovirales; Myoviridae; Ounavirinae; Mooglevirus; unclassified Mooglevirus
GCA_002620185.1	53224	90	Vibrio phage H20	NA	Viruses; Duplodnaviria; Heunggongvirae; Uroviricota; Caudoviricetes; Caudovirales; Siphoviridae; unclassified Siphoviridae
GCA_002620205.1	46157	75	Vibrio phage H8	NA	Viruses; Duplodnaviria; Heunggongvirae; Uroviricota; Caudoviricetes; Caudovirales; Siphoviridae; unclassified Siphoviridae
GCA_002620225.1	46149	75	Vibrio phage P2	NA	Viruses; Duplodnaviria; Heunggongvirae; Uroviricota; Caudoviricetes; Caudovirales; Siphoviridae; unclassified Siphoviridae

gca	genome.size	proteome.size	phage.name	strain	Taxonomy
GCA_002620245.1	53242	90	Vibrio phage P3	NA	Viruses; Duplodnaviria; Heunggongvirae; Uroviricota; Caudoviricetes; Caudovirales; Siphoviridae; unclassified Siphoviridae
GCA_002620265.1	54344	93	Vibrio phage pVa3	NA	Viruses; Duplodnaviria; Heunggongvirae; Uroviricota; Caudoviricetes; Caudovirales; Siphoviridae; unclassified Siphoviridae
GCA_002620285.1	54295	92	Vibrio phage pVa4	NA	Viruses; Duplodnaviria; Heunggongvirae; Uroviricota; Caudoviricetes; Caudovirales; Siphoviridae; unclassified Siphoviridae
GCA_002620305.1	53227	90	Vibrio phage pVa8	NA	Viruses; Duplodnaviria; Heunggongvirae; Uroviricota; Caudoviricetes; Caudovirales; Siphoviridae; unclassified Siphoviridae
GCA_002620325.1	50407	80	Salmonella phage GJL01	NA	Viruses; Duplodnaviria; Heunggongvirae; Uroviricota; Caudoviricetes; Caudovirales; Drexlerviridae; Tempevirinae; Tlsvirus; unclassified Tlsvirus
GCA_002620345.1	107263	160	Escherichia phage phiLLS	NA	Viruses; Duplodnaviria; Heunggongvirae; Uroviricota; Caudoviricetes; Caudovirales; Demerecviridae; Markadamsvirinae; Tequintavirus; Escherichia virus phiLLS
GCA_002620365.1	125658	178	Lactococcus phage AM1	NA	Viruses; Duplodnaviria; Heunggongvirae; Uroviricota; Caudoviricetes; Caudovirales; Siphoviridae; unclassified Siphoviridae
GCA_002620385.1	126161	178	Lactococcus phage AM11	NA	Viruses; Duplodnaviria; Heunggongvirae; Uroviricota; Caudoviricetes; Caudovirales; Siphoviridae; unclassified Siphoviridae
GCA_002620405.1	125842	179	Lactococcus phage AM12	NA	Viruses; Duplodnaviria; Heunggongvirae; Uroviricota; Caudoviricetes; Caudovirales; Siphoviridae; unclassified Siphoviridae
GCA_002620425.1	125656	177	Lactococcus phage AM2	NA	Viruses; Duplodnaviria; Heunggongvirae; Uroviricota; Caudoviricetes; Caudovirales; Siphoviridae; unclassified Siphoviridae
GCA_002620445.1	126032	177	Lactococcus phage AM3	NA	Viruses; Duplodnaviria; Heunggongvirae; Uroviricota; Caudoviricetes; Caudovirales; Siphoviridae; unclassified Siphoviridae
GCA_002620465.1	132949	194	Lactococcus phage AM4	NA	Viruses; Duplodnaviria; Heunggongvirae; Uroviricota; Caudoviricetes; Caudovirales; Siphoviridae; unclassified Siphoviridae
GCA_002620485.1	128178	182	Lactococcus phage AM5	NA	Viruses; Duplodnaviria; Heunggongvirae; Uroviricota; Caudoviricetes; Caudovirales; Siphoviridae; unclassified Siphoviridae
GCA_002620505.1	62054	90	Lactococcus phage AM6	NA	Viruses; Duplodnaviria; Heunggongvirae; Uroviricota; Caudoviricetes; Caudovirales; Siphoviridae; unclassified Siphoviridae
GCA_002620525.1	62252	90	Lactococcus phage AM7	NA	Viruses; Duplodnaviria; Heunggongvirae; Uroviricota; Caudoviricetes; Caudovirales; Siphoviridae; unclassified Siphoviridae
GCA_002620545.1	126177	178	Lactococcus phage AM8	NA	Viruses; Duplodnaviria; Heunggongvirae; Uroviricota; Caudoviricetes; Caudovirales; Siphoviridae; unclassified Siphoviridae
GCA_002620565.1	125294	179	Lactococcus phage AM9	NA	Viruses; Duplodnaviria; Heunggongvirae; Uroviricota; Caudoviricetes; Caudovirales; Siphoviridae; unclassified Siphoviridae
GCA_002620585.1	60551	85	Lactococcus phage LW31	NA	Viruses; Duplodnaviria; Heunggongvirae; Uroviricota; Caudoviricetes; Caudovirales; Siphoviridae; unclassified Siphoviridae
GCA_002620605.1	60161	87	Lactococcus phage LW32	NA	Viruses; Duplodnaviria; Heunggongvirae; Uroviricota; Caudoviricetes; Caudovirales; Siphoviridae; unclassified Siphoviridae
GCA_002620625.1	59899	86	Lactococcus phage LW33	NA	Viruses; Duplodnaviria; Heunggongvirae; Uroviricota; Caudoviricetes; Caudovirales; Siphoviridae; unclassified Siphoviridae
GCA_002620645.1	60217	87	Lactococcus phage LW4	NA	Viruses; Duplodnaviria; Heunggongvirae; Uroviricota; Caudoviricetes; Caudovirales; Siphoviridae; unclassified Siphoviridae
GCA_002620665.1	128179	178	Lactococcus phage LW81	NA	Viruses; Duplodnaviria; Heunggongvirae; Uroviricota; Caudoviricetes; Caudovirales; Siphoviridae; unclassified Siphoviridae
GCA_002620685.1	27704	49	Lactococcus phage R34	NA	Viruses; Duplodnaviria; Heunggongvirae; Uroviricota; Caudoviricetes; Caudovirales; Siphoviridae; Skunavirus; unclassified Skunavirus
GCA_002620705.1	27203	51	Lactococcus phage R31	NA	Viruses; Duplodnaviria; Heunggongvirae; Uroviricota; Caudoviricetes; Caudovirales; Siphoviridae; Skunavirus; unclassified Skunavirus
GCA_002620725.1	74325	126	Vibrio phage pVco5	NA	Viruses; Duplodnaviria; Heunggongvirae; Uroviricota; Caudoviricetes; Caudovirales; Podoviridae; unclassified Podoviridae
GCA_002620745.1	45236	72	Staphylococcus phage IME1318_01	NA	Viruses; Duplodnaviria; Heunggongvirae; Uroviricota; Caudoviricetes; Caudovirales; Siphoviridae; unclassified Siphoviridae
GCA_002620765.1	44282	67	Staphylococcus phage IME1323_01	NA	Viruses; Duplodnaviria; Heunggongvirae; Uroviricota; Caudoviricetes; Caudovirales; Siphoviridae; unclassified Siphoviridae
GCA_002620785.1	39387	60	Morganella phage IME1369_02	NA	Viruses; Duplodnaviria; Heunggongvirae; Uroviricota; Caudoviricetes; Caudovirales; Siphoviridae; unclassified Siphoviridae
GCA_002620805.1	42371	63	Staphylococcus phage IME1348_01	NA	Viruses; Duplodnaviria; Heunggongvirae; Uroviricota; Caudoviricetes; Caudovirales; Siphoviridae; Phietavirus; unclassified Phietavirus
GCA_002620825.1	43516	67	Staphylococcus phage IME1361_01	NA	Viruses; Duplodnaviria; Heunggongvirae; Uroviricota; Caudoviricetes; Caudovirales; Siphoviridae; Biseptimavirus; unclassified Biseptimavirus
GCA_002620845.1	43135	68	Staphylococcus phage IME1367_01	NA	Viruses; Duplodnaviria; Heunggongvirae; Uroviricota; Caudoviricetes; Caudovirales; Siphoviridae; unclassified Siphoviridae
GCA_002620865.1	42689	72	Staphylococcus phage IME1346_01	NA	Viruses; Duplodnaviria; Heunggongvirae; Uroviricota; Caudoviricetes; Caudovirales; Siphoviridae; Biseptimavirus; unclassified Biseptimavirus
GCA_002620885.1	42706	64	Staphylococcus phage IME1354_01	NA	Viruses; Duplodnaviria; Heunggongvirae; Uroviricota; Caudoviricetes; Caudovirales; Siphoviridae; unclassified Siphoviridae
GCA_002620905.1	44875	63	Staphylococcus phage IME1365_01	NA	Viruses; Duplodnaviria; Heunggongvirae; Uroviricota; Caudoviricetes; Caudovirales; Siphoviridae; Phietavirus; unclassified Phietavirus

gca	genome.size	proteome.size	phage.name	strain	Taxonomy
GCA_002620925.1	47739	67	Morganella phage IME1369_01	NA	Viruses; Duplodnaviria; Heunggongvirae; Uroviricota; Caudoviricetes; Caudovirales; Siphoviridae; unclassified Siphoviridae
GCA_002620985.1	40068	53	Corynebacterium phage IME1320_01	NA	Viruses; Duplodnaviria; Heunggongvirae; Uroviricota; Caudoviricetes; Caudovirales; Siphoviridae; unclassified Siphoviridae
GCA_002029575.1	7429	1	Bakunsa virus	NA	Viruses; ssRNA viruses; ssRNA positive-strand viruses, no DNA stage; Picornavirales; Picornaviridae; Kunsagivirus; unclassified Kunsagivirus
GCA_013086145.1	13559	5	Blackberry leaf mottle associated virus	NA	Viruses; Riboviria; Orthornavirae; Negarnaviricota; Polyploviricotina; Ellioviricetes; Bunyavirales; Fimoviridae; Emaravirus; unclassified Emaravirus
GCA_000853525.3	5207	2	Helicobasidium mompa totivirus 117	NA	Viruses; dsRNA viruses; Totiviridae; Victorivirus
GCA_002986915.1	2622	5	Tobacco leaf rugose virus	NA	Viruses; Monodnaviria; Shotokuvirae; Cressdnaviricota; Repensiviricetes; Geplafuvirales; Geminiviridae; Begomovirus
GCA_002116055.1	13389	7	Tomato yellow mottleassociated virus	NA	Viruses; ssRNA viruses; ssRNA negative-strand viruses; Mononegavirales; Rhabdoviridae; unclassified Rhabdoviridae
GCA_002621025.1	5688	8	Thermus phage phiOH3	NA	Viruses; Monodnaviria; Loebvirae; Hofneiviricota; Faserviricetes; Tubulavirales; Inoviridae; Thomixvirus; Thermus virus OH3
GCA_002621045.1	6533	9	Thermus phage phiOH16	NA	Viruses; Monodnaviria; Loebvirae; Hofneiviricota; Faserviricetes; Tubulavirales; Inoviridae; unclassified Inoviridae
GCA_002149845.1	9403	3	Castlerea virus	NA	Viruses; ssRNA viruses; ssRNA positive-strand viruses, no DNA stage; unclassified ssRNA positive-strand viruses; Negevirus
GCA_002210735.1	10646	1	Karumba virus	NA	Viruses; ssRNA viruses; ssRNA positive-strand viruses, no DNA stage; Flaviviridae; unclassified Flaviviridae
GCA_002219825.1	10026	1	Mac Peak virus	NA	Viruses; ssRNA viruses; ssRNA positive-strand viruses, no DNA stage; Flaviviridae; unclassified Flaviviridae
GCA_003179655.1	7189	7	Bos taurus papillomavirus 15	Aks02	Viruses; Monodnaviria; Shotokuvirae; Cossaviricota; Papovaviricetes; Zurhausenvirales; Papillomaviridae; Firstpapillomavirinae; Xipapillomavirus; Xipapillomavirus 1
GCA_002621065.1	34730	53	Staphylococcus phage SA7	NA	Viruses; Duplodnaviria; Heunggongvirae; Uroviricota; Caudoviricetes; Caudovirales; Siphoviridae; Biseptimavirus; unclassified Biseptimavirus
GCA_004320245.1	42473	53	Microcystis phage MACPNOA1	NA	Viruses; Duplodnaviria; Heunggongvirae; Uroviricota; Caudoviricetes; Caudovirales; Siphoviridae; unclassified Siphoviridae
GCA_004130215.1	11798	5	Meridalike virus KE2017a	NA	Viruses; Riboviria; Orthornavirae; Negarnaviricota; Haploviricotina; Monjiviricetes; Mononegavirales; Rhabdoviridae; unclassified Rhabdoviridae
GCA_002621085.1	54836	91	Proteus phage VB_PmiSIsfahan	NA	Viruses; Duplodnaviria; Heunggongvirae; Uroviricota; Caudoviricetes; Caudovirales; Siphoviridae; Gorganvirus; Proteus virus Isfahan
GCA_900010585.1	41308	51	Acinetobacter phage Loki	NA	Viruses; Duplodnaviria; Heunggongvirae; Uroviricota; Caudoviricetes; Caudovirales; Siphoviridae; Lokivirus; Acinetobacter virus Loki
GCA_002621105.1	135563	199	Staphylococcus phage qdsa001	NA	Viruses; Duplodnaviria; Heunggongvirae; Uroviricota; Caudoviricetes; Caudovirales; Herelleviridae; Twortvirinae; Silviavirus; unclassified Silviavirus
GCA_002621125.1	142499	231	Staphylococcus phage qdsa002	NA	Viruses; Duplodnaviria; Heunggongvirae; Uroviricota; Caudoviricetes; Caudovirales; Herelleviridae; Twortvirinae; Kayvirus; unclassified Kayvirus
GCA_002621145.1	51738	87	Salmonella phage 29485	NA	Viruses; Duplodnaviria; Heunggongvirae; Uroviricota; Caudoviricetes; Caudovirales; Siphoviridae; unclassified Siphoviridae
GCA_002621185.1	186570	281	Escherichia phage phiC120	NA	Viruses; Duplodnaviria; Heunggongvirae; Uroviricota; Caudoviricetes; Caudovirales; Myoviridae; Tevenvirinae; Mosigvirus; unclassified Mosigvirus
GCA_003693725.1	38783	54	Weissella phage PWc	NA	Viruses; Duplodnaviria; Heunggongvirae; Uroviricota; Caudoviricetes; Caudovirales; Siphoviridae; unclassified Siphoviridae
GCA_002621205.1	80309	142	Mycobacterium phage GardenSalsa	NA	Viruses; Duplodnaviria; Heunggongvirae; Uroviricota; Caudoviricetes; Caudovirales; Siphoviridae; Mclasvirinae; Reyvirus; unclassified Reyvirus
GCA_002621225.1	60300	112	Mycobacterium phage Kingsley	NA	Viruses; Duplodnaviria; Heunggongvirae; Uroviricota; Caudoviricetes; Caudovirales; Siphoviridae; Cheoctovirus; unclassified Cheoctovirus
GCA_002621245.1	54319	69	Shewanella phage SppYZU05	NA	Viruses; Duplodnaviria; Heunggongvirae; Uroviricota; Caudoviricetes; Caudovirales; Chaseviridae; Yushanvirus; Shewanella virus SppYZU05
GCA_002621265.1	41289	56	Escherichia phage vB_EcoM_ECOO78	NA	Viruses; Duplodnaviria; Heunggongvirae; Uroviricota; Caudoviricetes; Caudovirales; Myoviridae; Jilinvirus; Escherichia virus ECOO78
GCA_002621285.1	50526	81	Klebsiella phage KOX1	NA	Viruses; Duplodnaviria; Heunggongvirae; Uroviricota; Caudoviricetes; Caudovirales; Drexlerviridae; Webervirus; Klebsiella virus KOX1
GCA_002955025.1	58359	77	Pseudomonas phage JG012	NA	Viruses; Duplodnaviria; Heunggongvirae; Uroviricota; Caudoviricetes; Caudovirales; Siphoviridae; Nipunavirus; unclassified Nipunavirus
GCA_002955035.1	57839	74	Pseudomonas phage JG054	NA	Viruses; Duplodnaviria; Heunggongvirae; Uroviricota; Caudoviricetes; Caudovirales; Siphoviridae; Nipunavirus; unclassified Nipunavirus
GCA_002830525.1	4766	5	Pear latent virus	NA	Viruses; Riboviria; Orthornavirae; Kitrinoviricota; Tolucaviricetes; Tolivirales; Tombusviridae; Procedovirinae; Tombusvirus; Eggplant mottled crinkle virus
GCA_002621305.1	56606	74	Clostridioides phage phiSemix9P1	NA	Viruses; Duplodnaviria; Heunggongvirae; Uroviricota; Caudoviricetes; Caudovirales; Myoviridae; Lubbockvirus; unclassified Lubbockvirus
GCA_018594915.1	18582	2	Grotenhout virus	NA	Viruses; Riboviria; Orthornavirae; Negarnaviricota; Polyploviricotina; Ellioviricetes; Bunyavirales; Nairoviridae; Norwavirus; Grotenhout norwavirus
GCA_002621325.1	35653	44	Streptococcus phage P0091	NA	Viruses; Duplodnaviria; Heunggongvirae; Uroviricota; Caudoviricetes; Caudovirales; Siphoviridae; Moineauvirus; unclassified Moineauvirus

gca	genome.size	proteome.size	phage.name	strain	Taxonomy
GCA_002621345.1	34580	50	Streptococcus phage P0092	NA	Viruses; Duplodnaviria; Heunggongvirae; Uroviricota; Caudoviricetes; Caudovirales; Siphoviridae; unclassified Siphoviridae
GCA_002621365.1	34932	51	Streptococcus phage P0093	NA	Viruses; Duplodnaviria; Heunggongvirae; Uroviricota; Caudoviricetes; Caudovirales; Siphoviridae; unclassified Siphoviridae
GCA_002621385.1	33335	47	Streptococcus phage P0094	NA	Viruses; Duplodnaviria; Heunggongvirae; Uroviricota; Caudoviricetes; Caudovirales; Siphoviridae; unclassified Siphoviridae
GCA_002621405.1	35966	54	Streptococcus phage P0095	NA	Viruses; Duplodnaviria; Heunggongvirae; Uroviricota; Caudoviricetes; Caudovirales; Siphoviridae; unclassified Siphoviridae
GCA_002621425.1	36168	45	Streptococcus phage P3681	NA	Viruses; Duplodnaviria; Heunggongvirae; Uroviricota; Caudoviricetes; Caudovirales; Siphoviridae; Moineauvirus; unclassified Moineauvirus
GCA_002621445.1	35444	44	Streptococcus phage P3684	NA	Viruses; Duplodnaviria; Heunggongvirae; Uroviricota; Caudoviricetes; Caudovirales; Siphoviridae; Moineauvirus; unclassified Moineauvirus
GCA_002621465.1	38139	55	Streptococcus phage P4761	NA	Viruses; Duplodnaviria; Heunggongvirae; Uroviricota; Caudoviricetes; Caudovirales; Siphoviridae; unclassified Siphoviridae
GCA_002621485.1	37235	52	Streptococcus phage P5641	NA	Viruses; Duplodnaviria; Heunggongvirae; Uroviricota; Caudoviricetes; Caudovirales; Siphoviridae; Moineauvirus; unclassified Moineauvirus
GCA_002621505.1	34292	43	Streptococcus phage P5651	NA	Viruses; Duplodnaviria; Heunggongvirae; Uroviricota; Caudoviricetes; Caudovirales; Siphoviridae; Moineauvirus; unclassified Moineauvirus
GCA_002621525.1	33448	43	Streptococcus phage P5652	NA	Viruses; Duplodnaviria; Heunggongvirae; Uroviricota; Caudoviricetes; Caudovirales; Siphoviridae; Moineauvirus; unclassified Moineauvirus
GCA_002621545.1	35843	44	Streptococcus phage P7132	NA	Viruses; Duplodnaviria; Heunggongvirae; Uroviricota; Caudoviricetes; Caudovirales; Siphoviridae; Moineauvirus; unclassified Moineauvirus
GCA_002621565.1	33284	42	Streptococcus phage P7133	NA	Viruses; Duplodnaviria; Heunggongvirae; Uroviricota; Caudoviricetes; Caudovirales; Siphoviridae; Moineauvirus; unclassified Moineauvirus
GCA_002621585.1	34019	45	Streptococcus phage P7134	NA	Viruses; Duplodnaviria; Heunggongvirae; Uroviricota; Caudoviricetes; Caudovirales; Siphoviridae; Moineauvirus; unclassified Moineauvirus
GCA_002621605.1	35025	47	Streptococcus phage P7151	NA	Viruses; Duplodnaviria; Heunggongvirae; Uroviricota; Caudoviricetes; Caudovirales; Siphoviridae; Moineauvirus; unclassified Moineauvirus
GCA_002621625.1	35663	46	Streptococcus phage P7152	NA	Viruses; Duplodnaviria; Heunggongvirae; Uroviricota; Caudoviricetes; Caudovirales; Siphoviridae; Moineauvirus; unclassified Moineauvirus
GCA_002621645.1	34507	42	Streptococcus phage P7154	NA	Viruses; Duplodnaviria; Heunggongvirae; Uroviricota; Caudoviricetes; Caudovirales; Siphoviridae; Moineauvirus; unclassified Moineauvirus
GCA_002621665.1	34907	49	Streptococcus phage P7571	NA	Viruses; Duplodnaviria; Heunggongvirae; Uroviricota; Caudoviricetes; Caudovirales; Siphoviridae; Brussowvirus; unclassified Brussowvirus
GCA_002621685.1	33410	43	Streptococcus phage P7572	NA	Viruses; Duplodnaviria; Heunggongvirae; Uroviricota; Caudoviricetes; Caudovirales; Siphoviridae; Brussowvirus; unclassified Brussowvirus
GCA_002621705.1	37715	48	Streptococcus phage P7573	NA	Viruses; Duplodnaviria; Heunggongvirae; Uroviricota; Caudoviricetes; Caudovirales; Siphoviridae; Moineauvirus; unclassified Moineauvirus
GCA_002621725.1	36298	49	Streptococcus phage P7574	NA	Viruses; Duplodnaviria; Heunggongvirae; Uroviricota; Caudoviricetes; Caudovirales; Siphoviridae; Moineauvirus; unclassified Moineauvirus
GCA_002621745.1	35395	51	Streptococcus phage P7601	NA	Viruses; Duplodnaviria; Heunggongvirae; Uroviricota; Caudoviricetes; Caudovirales; Siphoviridae; Moineauvirus; unclassified Moineauvirus
GCA_002621765.1	37202	51	Streptococcus phage P7602	NA	Viruses; Duplodnaviria; Heunggongvirae; Uroviricota; Caudoviricetes; Caudovirales; Siphoviridae; Moineauvirus; unclassified Moineauvirus
GCA_002621785.1	36164	48	Streptococcus phage P7631	NA	Viruses; Duplodnaviria; Heunggongvirae; Uroviricota; Caudoviricetes; Caudovirales; Siphoviridae; Moineauvirus; unclassified Moineauvirus
GCA_002621805.1	35988	47	Streptococcus phage P7632	NA	Viruses; Duplodnaviria; Heunggongvirae; Uroviricota; Caudoviricetes; Caudovirales; Siphoviridae; Moineauvirus; unclassified Moineauvirus
GCA_002621825.1	35921	47	Streptococcus phage P7633	NA	Viruses; Duplodnaviria; Heunggongvirae; Uroviricota; Caudoviricetes; Caudovirales; Siphoviridae; Moineauvirus; unclassified Moineauvirus
GCA_002621845.1	34123	47	Streptococcus phage P7951	NA	Viruses; Duplodnaviria; Heunggongvirae; Uroviricota; Caudoviricetes; Caudovirales; Siphoviridae; unclassified Siphoviridae
GCA_002621865.1	37594	48	Streptococcus phage P7952	NA	Viruses; Duplodnaviria; Heunggongvirae; Uroviricota; Caudoviricetes; Caudovirales; Siphoviridae; Brussowvirus; unclassified Brussowvirus
GCA_002621885.1	35786	43	Streptococcus phage P7953	NA	Viruses; Duplodnaviria; Heunggongvirae; Uroviricota; Caudoviricetes; Caudovirales; Siphoviridae; Brussowvirus; unclassified Brussowvirus
GCA_002621905.1	35688	46	Streptococcus phage P7954	NA	Viruses; Duplodnaviria; Heunggongvirae; Uroviricota; Caudoviricetes; Caudovirales; Siphoviridae; Brussowvirus; unclassified Brussowvirus
GCA_002621925.1	36931	50	Streptococcus phage P7955	NA	Viruses; Duplodnaviria; Heunggongvirae; Uroviricota; Caudoviricetes; Caudovirales; Siphoviridae; unclassified Siphoviridae
GCA_002621945.1	34516	46	Streptococcus phage P8921	NA	Viruses; Duplodnaviria; Heunggongvirae; Uroviricota; Caudoviricetes; Caudovirales; Siphoviridae; Moineauvirus; unclassified Moineauvirus
GCA_002621965.1	34509	43	Streptococcus phage P8922	NA	Viruses; Duplodnaviria; Heunggongvirae; Uroviricota; Caudoviricetes; Caudovirales; Siphoviridae; Moineauvirus; unclassified Moineauvirus
GCA_002621985.1	35835	48	Streptococcus phage P9851	NA	Viruses; Duplodnaviria; Heunggongvirae; Uroviricota; Caudoviricetes; Caudovirales; Siphoviridae; Moineauvirus; unclassified Moineauvirus
GCA_002622005.1	34638	44	Streptococcus phage P9852	NA	Viruses; Duplodnaviria; Heunggongvirae; Uroviricota; Caudoviricetes; Caudovirales; Siphoviridae; Brussowvirus; unclassified Brussowvirus

gca	genome.size	proteome.size	phage.name	strain	Taxonomy
GCA_002622025.1	34469	45	Streptococcus phage P9853	NA	Viruses; Duplodnaviria; Heunggongvirae; Uroviricota; Caudoviricetes; Caudovirales; Siphoviridae; Brussowvirus; unclassified Brussowvirus
GCA_002622045.1	36979	48	Streptococcus phage P9854	NA	Viruses; Duplodnaviria; Heunggongvirae; Uroviricota; Caudoviricetes; Caudovirales; Siphoviridae; Moineauvirus; unclassified Moineauvirus
GCA_002622065.1	35211	47	Streptococcus phage P9901	NA	Viruses; Duplodnaviria; Heunggongvirae; Uroviricota; Caudoviricetes; Caudovirales; Siphoviridae; Moineauvirus; unclassified Moineauvirus
GCA_002622085.1	35535	50	Streptococcus phage P9902	NA	Viruses; Duplodnaviria; Heunggongvirae; Uroviricota; Caudoviricetes; Caudovirales; Siphoviridae; Moineauvirus; unclassified Moineauvirus
GCA_002622105.1	35256	50	Streptococcus phage P9903	NA	Viruses; Duplodnaviria; Heunggongvirae; Uroviricota; Caudoviricetes; Caudovirales; Siphoviridae; Moineauvirus; unclassified Moineauvirus
GCA_002622125.1	36671	48	Corynebacterium phage LGCMV2	NA	Viruses; Duplodnaviria; Heunggongvirae; Uroviricota; Caudoviricetes; Caudovirales; Siphoviridae; unclassified Siphoviridae
GCA_002622145.1	36671	48	Corynebacterium phage LGCMV3	NA	Viruses; Duplodnaviria; Heunggongvirae; Uroviricota; Caudoviricetes; Caudovirales; Siphoviridae; unclassified Siphoviridae
GCA_002622165.1	36674	49	Corynebacterium phage LGCMV4	NA	Viruses; Duplodnaviria; Heunggongvirae; Uroviricota; Caudoviricetes; Caudovirales; Siphoviridae; unclassified Siphoviridae
GCA_002622185.1	36671	48	Corynebacterium phage LGCMV5	NA	Viruses; Duplodnaviria; Heunggongvirae; Uroviricota; Caudoviricetes; Caudovirales; Siphoviridae; unclassified Siphoviridae
GCA_002622205.1	36671	48	Corynebacterium phage LGCMV6	NA	Viruses; Duplodnaviria; Heunggongvirae; Uroviricota; Caudoviricetes; Caudovirales; Siphoviridae; unclassified Siphoviridae
GCA_002622225.1	36671	48	Corynebacterium phage LGCMV7	NA	Viruses; Duplodnaviria; Heunggongvirae; Uroviricota; Caudoviricetes; Caudovirales; Siphoviridae; unclassified Siphoviridae
GCA_002622245.1	36671	48	Corynebacterium phage LGCMV8	NA	Viruses; Duplodnaviria; Heunggongvirae; Uroviricota; Caudoviricetes; Caudovirales; Siphoviridae; unclassified Siphoviridae
GCA_002622265.1	36671	48	Corynebacterium phage LGCMV9	NA	Viruses; Duplodnaviria; Heunggongvirae; Uroviricota; Caudoviricetes; Caudovirales; Siphoviridae; unclassified Siphoviridae
GCA_002145705.1	11181	5	Morreton vesiculovirus	CoAr191048	Viruses; ssRNA viruses; ssRNA negative-strand viruses; Mononegavirales; Rhabdoviridae; Vesiculovirus
GCA_002816075.1	11068	5	Radi vesiculovirus	ISS Phl166	Viruses; Riboviria; Orthornavirae; Negarnaviricota; Haploviricotina; Monjiviricetes; Mononegavirales; Rhabdoviridae; Vesiculovirus
GCA_000925435.1	11202	5	Yug Bogdanovac vesiculovirus	NA	Viruses; ssRNA viruses; ssRNA negative-strand viruses; Mononegavirales; Rhabdoviridae; Vesiculovirus
GCA_000926635.1	11121	5	Jurona vesiculovirus	NA	Viruses; ssRNA viruses; ssRNA negative-strand viruses; Mononegavirales; Rhabdoviridae; Vesiculovirus
GCA_000926675.1	11103	5	Perinet vesiculovirus	NA	Viruses; ssRNA viruses; ssRNA negative-strand viruses; Mononegavirales; Rhabdoviridae; Vesiculovirus
GCA_000927135.1	11019	5	Malpais Spring vesiculovirus	85488NM	Viruses; ssRNA viruses; ssRNA negative-strand viruses; Mononegavirales; Rhabdoviridae; Vesiculovirus
GCA_002815595.1	13038	9	Flanders hapavirus	617484	Viruses; ssRNA viruses; ssRNA negative-strand viruses; Mononegavirales; Rhabdoviridae; Hapavirus
GCA_000886315.1	15764	15	Ngaingan hapavirus	MRM14556	Viruses; ssRNA viruses; ssRNA negative-strand viruses; Mononegavirales; Rhabdoviridae; Hapavirus
GCA_000882535.1	13196	10	Wongabel hapavirus	CS264	Viruses; ssRNA viruses; ssRNA negative-strand viruses; Mononegavirales; Rhabdoviridae; Hapavirus
GCA_000927335.1	11482	6	Arboretum almendravirus	NA	Viruses; Riboviria; Orthornavirae; Negarnaviricota; Haploviricotina; Monjiviricetes; Mononegavirales; Rhabdoviridae; Almendravirus
GCA_002117775.1	9580	1	Diamondback moth iflavirus	NA	Viruses; ssRNA viruses; ssRNA positive-strand viruses, no DNA stage; Picornavirales; Iflaviridae; Iflavirus; unclassified Iflavirus
GCA_002116215.1	7398	4	Arracacha virus V	NA	Viruses; ssRNA viruses; ssRNA positive-strand viruses, no DNA stage; Tymovirales; Betaflexiviridae; Trivirinae; Vitivirus; unclassified Vitivirus
GCA_006435595.1	35198	37	Human adenovirus 81	Chiba_E0862012	Viruses; Varidnaviria; Bamfordvirae; Preplasmiviricota; Tectiliviricetes; Rowavirales; Adenoviridae; Mastadenovirus; Human mastadenovirus D
GCA_013087455.1	9998	2	Gomphocarpus mosaic virus	NA	Viruses; Riboviria; Orthornavirae; Pisuviricota; Stelpaviricetes; Patatavirales; Potyviridae; Potyvirus
GCA_002087205.1	5979	6	Tobacco virus 2	NA	Viruses; ssRNA viruses; ssRNA positive-strand viruses, no DNA stage; Luteoviridae; Polerovirus; unclassified Polerovirus
GCA_002641435.1	18586	11	Rotavirus A RVAHumanwtJPNIS10782015G3P8	NA	Viruses; Riboviria; Orthornavirae; Duplornaviricota; Resentoviricetes; Reovirales; Reoviridae; Sedoreovirinae; Rotavirus; Rotavirus A; Rotavirus G3
GCA_002641475.1	18552	11	Rotavirus A RVAHumanwtJPNIS10902016G3P8	NA	Viruses; Riboviria; Orthornavirae; Duplornaviricota; Resentoviricetes; Reovirales; Reoviridae; Sedoreovirinae; Rotavirus; Rotavirus A; Rotavirus G3
GCA_002641555.1	18432	11	Rotavirus A RVAHumanwtJPNMI11252016G3P8	NA	Viruses; Riboviria; Orthornavirae; Duplornaviricota; Resentoviricetes; Reovirales; Reoviridae; Sedoreovirinae; Rotavirus; Rotavirus A; Rotavirus G3
GCA_002641195.1	18575	11	Rotavirus A RVAHumanwtJPNCH10202016G2P4	NA	Viruses; Riboviria; Orthornavirae; Duplornaviricota; Resentoviricetes; Reovirales; Reoviridae; Sedoreovirinae; Rotavirus; Rotavirus A; Rotavirus G2
GCA_002641535.1	18572	11	Rotavirus A RVAHumanwtJPNK3162016G2P4	NA	Viruses; Riboviria; Orthornavirae; Duplornaviricota; Resentoviricetes; Reovirales; Reoviridae; Sedoreovirinae; Rotavirus; Rotavirus A; Rotavirus G2
GCA_002641495.1	18567	11	Rotavirus A RVAHumanwtJPNK21162016G2P4	NA	Viruses; Riboviria; Orthornavirae; Duplornaviricota; Resentoviricetes; Reovirales; Reoviridae; Sedoreovirinae; Rotavirus; Rotavirus A; Rotavirus G2

gca	genome.size	proteome.size	phage.name	strain	Taxonomy
GCA_002641595.1	18511	11	Rotavirus A RVAHumanwtJPNMI11322016G2P4	NA	Viruses; Riboviria; Orthornavirae; Duplornaviricota; Resentoviricetes; Reovirales; Reoviridae; Sedoreovirinae; Rotavirus; Rotavirus A; Rotavirus G2
GCA_002641215.1	18455	11	Rotavirus A RVAHumanwtJPNCH10232016G9P8	NA	Viruses; Riboviria; Orthornavirae; Duplornaviricota; Resentoviricetes; Reovirales; Reoviridae; Sedoreovirinae; Rotavirus; Rotavirus A; Rotavirus G9
GCA_002641455.1	18346	11	Rotavirus A RVAHumanwtJPNIS10802016G9P8	NA	Viruses; Riboviria; Orthornavirae; Duplornaviricota; Resentoviricetes; Reovirales; Reoviridae; Sedoreovirinae; Rotavirus; Rotavirus A; Rotavirus G9
GCA_002641575.1	18461	11	Rotavirus A RVAHumanwtJPNMI11282016G9P8	NA	Viruses; Riboviria; Orthornavirae; Duplornaviricota; Resentoviricetes; Reovirales; Reoviridae; Sedoreovirinae; Rotavirus; Rotavirus A; Rotavirus G9
GCA_002080255.1	7326	4	Wisteria badnavirus 1	NA	Viruses; Riboviria; Pararnavirae; Artverviricota; Revtraviricetes; Ortervirales; Caulimoviridae; Badnavirus
GCA_900176665.1	470659	478	Faustovirus ST1	NA	Viruses; unclassified viruses
GCA_002622285.1	47657	87	Salmonella phage LPST10	NA	Viruses; Duplodnaviria; Heunggongvirae; Uroviricota; Caudoviricetes; Caudovirales; Siphoviridae; unclassified Siphoviridae
GCA_003729015.1	2660	2	Diporeiaassociated CRESSDNA virus LH481	NA	Viruses; unclassified viruses
GCA_003729035.1	1955	2	Diporeiaassociated CRESSDNA virus LM122	NA	Viruses; unclassified viruses
GCA_006450595.1	351108	312	Shearwaterpox virus	SWPV2	Viruses; Varidnaviria; Bamfordvirae; Nucleocytoviricota; Pokkesviricetes; Chitovirales; Poxviridae; Chordopoxvirinae; Avipoxvirus; Canarypox virus
GCA_002622305.1	17253	35	Rhodococcus phage Toil	NA	Viruses; Varidnaviria; Bamfordvirae; Preplasmiviricota; Tectiliviricetes; Kalamavirales; Tectiviridae; unclassified Tectiviridae
GCA_018594895.1	18653	3	Pacific coast tick nairovirus	NA	Viruses; Riboviria; Orthornavirae; Negarnaviricota; Polyploviricotina; Ellioviricetes; Bunyavirales; Nairoviridae; Orthonairovirus; unclassified Nairovirus
GCA_013086945.1	7794	2	Pacific coast tick phlebovirus	NA	Viruses; Riboviria; Orthornavirae; Negarnaviricota; Polyploviricotina; Ellioviricetes; Bunyavirales; Phenuiviridae; Phlebovirus; unclassified Phlebovirus
GCA_002116295.1	5879	4	Cassava virus X	NA	Viruses; ssRNA viruses; ssRNA positive-strand viruses, no DNA stage; Tymovirales; Alphaflexiviridae; Potexvirus; unclassified Potexvirus
GCA_000845205.1	6407	10	Escherichia virus M13	NA	Viruses; ssDNA viruses; Inoviridae; Inovirus
GCA_000847085.1	4491	4	Acholeplasma phage MVL51	JA1	Viruses; Monodnaviria; Loebvirae; Hofneiviricota; Faserviricetes; Tubulavirales; Plectroviridae; Plectrovirus; Acholeplasma virus L51
GCA_000841045.1	7729	6	Etapapillomavirus 1	NA	Viruses; dsDNA viruses, no RNA stage; Papillomaviridae; Etapapillomavirus
GCA_003080455.1	19201	13	Bluetongue virus 8	NA	Viruses; Riboviria; Orthornavirae; Duplornaviricota; Resentoviricetes; Reovirales; Reoviridae; Sedoreovirinae; Orbivirus; Bluetongue virus
GCA_003080995.1	19186	10	Bluetongue virus 18	NA	Viruses; Riboviria; Orthornavirae; Duplornaviricota; Resentoviricetes; Reovirales; Reoviridae; Sedoreovirinae; Orbivirus; Bluetongue virus
GCA_000842165.1	37359	42	Pseudomonad phage gh1	NA	Viruses; dsDNA viruses, no RNA stage; Caudovirales; Podoviridae; Autographivirinae; T7virus
GCA_002105425.1	8372	4	Watermelon virus A	NA	Viruses; ssRNA viruses; ssRNA positive-strand viruses, no DNA stage; Tymovirales; Betaflexiviridae; unclassified Betaflexiviridae
GCA_002815635.1	10881	4	Nishimuro ledantevirus	NA	Viruses; Riboviria; Orthornavirae; Negarnaviricota; Haploviricotina; Monjiviricetes; Mononegavirales; Rhabdoviridae; Ledantevirus
GCA_002114005.1	5225	7	Raccoonassociated polyomavirus 2	NA	Viruses; dsDNA viruses, no RNA stage; Polyomaviridae; unclassified Polyomaviridae
GCA_002622325.1	144974	185	Aeromonas phage phiA829	NA	Viruses; Duplodnaviria; Heunggongvirae; Uroviricota; Caudoviricetes; Caudovirales; Ackermannviridae; unclassified Ackermannviridae
GCA_002622345.1	8280	14	Xanthomonas phage Xf409	NA	Viruses; Monodnaviria; Loebvirae; Hofneiviricota; Faserviricetes; Tubulavirales; Inoviridae; unclassified Inoviridae
GCA_002145505.1	6386	4	Hoya chlorotic spot virus	NA	Viruses; ssRNA viruses; ssRNA positive-strand viruses, no DNA stage; Virgaviridae; Tobamovirus; unclassified Tobamovirus
GCA_002827165.1	5050	2	Sea starassociated densovirus	NA	Viruses; Monodnaviria; Shotokuvirae; Cossaviricota; Quintoviricetes; Piccovirales; Parvoviridae; Densovirinae; Aquambidensovirus; Asteroid aquambidensovirus 1
GCA_018580835.1	5860	4	Ambidensovirus CaaDV1	CHOY1	Viruses; Monodnaviria; Shotokuvirae; Cossaviricota; Quintoviricetes; Piccovirales; Parvoviridae; Densovirinae; Ambidensovirus; unclassified Ambidensovirus
GCA_002622365.1	130138	196	Sphingobium phage Lacusarx	NA	Viruses; Duplodnaviria; Heunggongvirae; Uroviricota; Caudoviricetes; Caudovirales; Siphoviridae; Lacusarxvirus; Sphingobium virus Lacusarx
GCA_003012505.1	15010	24	Haloarcula hispanica pleomorphic virus 4	1A_s5a1hispanica	Viruses; Monodnaviria; Trapavirae; Saleviricota; Huolimaviricetes; Haloruvirales; Pleolipoviridae; Betapleolipovirus; unclassified Betapleolipovirus
GCA_018591435.1	7893	8	Sophora yellow stunt virus	NA	Viruses; Monodnaviria; Shotokuvirae; Cressdnaviricota; Arfiviricetes; Mulpavirales; Nanoviridae; Nanovirus
GCA_003029465.1	996	1	Sophora alopecuroides yellow stunt alphasatellite 1a	NA	Viruses; Alphasatellitidae; Nanoalphasatellitinae; unclassified Nanoalphasatellitinae
GCA_003029485.1	1009	1	Sophora alopecuroides yellow stunt alphasatellite 4	NA	Viruses; Alphasatellitidae; Nanoalphasatellitinae; unclassified Nanoalphasatellitinae
GCA_003029475.1	1022	1	Sophora alopecuroides yellow stunt alphasatellite 7a	NA	Viruses; Alphasatellitidae; Nanoalphasatellitinae; unclassified Nanoalphasatellitinae

gca	genome.size	proteome.size	phage.name	strain	Taxonomy
GCA_003029455.1	1010	1	Sophora alopecuroides yellow stunt alphasatellite 8	NA	Viruses; Alphasatellitidae; Nanoalphasatellitinae; Clostunsatellite; Sophora yellow stunt alphasatellite 5
GCA_003029505.1	1003	1	Sophora alopecuroides yellow stunt alphasatellite 9	NA	Viruses; Alphasatellitidae; Nanoalphasatellitinae; Clostunsatellite; Sophora yellow stunt alphasatellite 4
GCA_000884235.1	12282	4	European mountain ash ringspotassociated emaravirus	NA	Viruses; ssRNA viruses; ssRNA negative-strand viruses; Bunyavirales; Fimoviridae; Emaravirus
GCA_001580335.1	15217	6	Fig mosaic emaravirus	NA	Viruses; ssRNA viruses; ssRNA negative-strand viruses; Bunyavirales; Fimoviridae; Emaravirus
GCA_002375145.1	18555	8	High Plains wheat mosaic emaravirus	NA	Viruses; Riboviria; Orthornavirae; Negarnaviricota; Polyploviricotina; Ellioviricetes; Bunyavirales; Fimoviridae; Emaravirus
GCA_001580355.1	14051	5	Pigeonpea sterility mosaic emaravirus 1	NA	Viruses; Riboviria; Orthornavirae; Negarnaviricota; Polyploviricotina; Ellioviricetes; Bunyavirales; Fimoviridae; Emaravirus
GCA_001695425.1	15091	6	Pigeonpea sterility mosaic emaravirus 2	NA	Viruses; ssRNA viruses; ssRNA negative-strand viruses; Bunyavirales; Fimoviridae; Emaravirus
GCA_001580315.1	17412	8	Raspberry leaf blotch emaravirus	NA	Viruses; ssRNA viruses; ssRNA negative-strand viruses; Bunyavirales; Fimoviridae; Emaravirus
GCA_000891875.2	17072	8	Rose rosette emaravirus	SEQISO	Viruses; ssRNA viruses; ssRNA negative-strand viruses; Bunyavirales; Fimoviridae; Emaravirus
GCA_000850405.1	12104	3	Andes orthohantavirus	Chile9717869	Viruses; ssRNA viruses; ssRNA negative-strand viruses; Bunyavirales; Hantaviridae; Orthohantavirus
GCA_002815935.1	6612	3	Asikkala orthohantavirus	CZBeskydy4122010Sm	Viruses; Riboviria; Orthornavirae; Negarnaviricota; Polyploviricotina; Ellioviricetes; Bunyavirales; Hantaviridae; Mammantavirinae; Orthohantavirus
GCA_002816435.1	12189	3	Bayou orthohantavirus	NA	Viruses; Riboviria; Orthornavirae; Negarnaviricota; Polyploviricotina; Ellioviricetes; Bunyavirales; Hantaviridae; Mammantavirinae; Orthohantavirus
GCA_002145545.1	12239	3	Cano Delgadito orthohantavirus	VHV574	Viruses; ssRNA viruses; ssRNA negative-strand viruses; Bunyavirales; Hantaviridae; Orthohantavirus
GCA_002119025.1	12003	3	Cao Bang orthohantavirus	3	Viruses; ssRNA viruses; ssRNA negative-strand viruses; Bunyavirales; Hantaviridae; Orthohantavirus
GCA_000863045.1	11840	3	DobravaBelgrade orthohantavirus	DOBVAnoPoroiaAfl91999	Viruses; ssRNA viruses; ssRNA negative-strand viruses; Bunyavirales; Hantaviridae; Orthohantavirus
GCA_000856085.1	11845	3	Hantaan orthohantavirus	76118	Viruses; ssRNA viruses; ssRNA negative-strand viruses; Bunyavirales; Hantaviridae; Orthohantavirus
GCA_002146125.1	12132	3	Khabarovsk orthohantavirus	NA	Viruses; ssRNA viruses; ssRNA negative-strand viruses; Bunyavirales; Hantaviridae; Orthohantavirus
GCA_002117715.1	12171	3	Montano orthohantavirus	NA	Viruses; ssRNA viruses; ssRNA negative-strand viruses; Bunyavirales; Hantaviridae; Orthohantavirus
GCA_002994685.1	11941	2	Prospect Hill orthohantavirus	PH1	Viruses; Riboviria; Orthornavirae; Negarnaviricota; Polyploviricotina; Ellioviricetes; Bunyavirales; Hantaviridae; Mammantavirinae; Orthohantavirus
GCA_000854405.1	12062	2	Puumala orthohantavirus	Sotkamo	Viruses; ssRNA viruses; ssRNA negative-strand viruses; Bunyavirales; Hantaviridae; Orthohantavirus
GCA_002145925.1	11928	3	Sangassou orthohantavirus	SA14	Viruses; ssRNA viruses; ssRNA negative-strand viruses; Bunyavirales; Hantaviridae; Orthohantavirus
GCA_000854765.1	12317	3	Sin Nombre orthohantavirus	NM H10	Viruses; ssRNA viruses; ssRNA negative-strand viruses; Bunyavirales; Hantaviridae; Orthohantavirus
GCA_000879955.1	11732	3	Thottopalayam virus	VRC 66412	Viruses; Riboviria; Orthornavirae; Negarnaviricota; Polyploviricotina; Ellioviricetes; Bunyavirales; Hantaviridae; Mammantavirinae; Thottimvirus; Thottopalayam thottimvirus
GCA_000855225.1	12066	1	Tula orthohantavirus	NA	Viruses; ssRNA viruses; ssRNA negative-strand viruses; Bunyavirales; Hantaviridae; Orthohantavirus
GCA_000854165.1	19146	3	CrimeanCongo hemorrhagic fever orthonairovirus	NA	Viruses; ssRNA viruses; ssRNA negative-strand viruses; Bunyavirales; Nairoviridae; Orthonairovirus
GCA_014883265.1	18334	3	Hughes orthonairovirus	G2126	Viruses; ssRNA viruses; ssRNA negative-strand viruses; Bunyavirales; Nairoviridae; Orthonairovirus
GCA_014883295.1	17906	3	Sakhalin orthonairovirus	LEIV71C	Viruses; Riboviria; Orthornavirae; Negarnaviricota; Polyploviricotina; Ellioviricetes; Bunyavirales; Nairoviridae; Orthonairovirus
GCA_002831145.1	18044	3	Thiafora orthonairovirus	NA	Viruses; ssRNA viruses; ssRNA negative-strand viruses; Bunyavirales; Nairoviridae; Orthonairovirus
GCA_002118825.1	11701	3	Kigluaik phantom orthophasmavirus	G10N	Viruses; ssRNA viruses; ssRNA negative-strand viruses; Bunyavirales; Phasmaviridae; Orthophasmavirus
GCA_002814795.1	12247	3	Badu phasivirus	NA	Viruses; Riboviria; Orthornavirae; Negarnaviricota; Polyploviricotina; Ellioviricetes; Bunyavirales; Phenuiviridae; Phasivirus
GCA_002814835.1	12099	3	Phasi Charoenlike phasivirus	NA	Viruses; Riboviria; Orthornavirae; Negarnaviricota; Polyploviricotina; Ellioviricetes; Bunyavirales; Phenuiviridae; Phasivirus
GCA_001755805.1	12072	3	Wutai mosquito phasivirus	QN35	Viruses; Riboviria; Orthornavirae; Negarnaviricota; Polyploviricotina; Ellioviricetes; Bunyavirales; Phenuiviridae; Phasivirus
GCA_004790215.1	12505	6	Citrus chlorotic spot virus	Trs1	Viruses; Riboviria; Orthornavirae; Negarnaviricota; Haploviricotina; Monjiviricetes; Mononegavirales; Rhabdoviridae; Dichorhavirus; Citrus chlorotic spot dichorhavirus
GCA_002146105.1	3135	2	Carnation cryptic virus 3	NA	Viruses; dsRNA viruses; Partitiviridae; unclassified Partitiviridae

gca	genome.size	proteome.size	phage.name	strain	Taxonomy
GCA_002145905.1	3720	2	Bipolaris maydis partitivirus 1	JZ04	Viruses; dsRNA viruses; Partitiviridae; unclassified Partitiviridae
GCA_002118645.1	5070	6	Canis familiaris polyomavirus 1	NA	Viruses; dsDNA viruses, no RNA stage; Polyomaviridae; Betapolyomavirus; unclassified Betapolyomavirus
GCA_000857965.1	11034	6	Hirame novirhabdovirus	CA 9703	Viruses; ssRNA viruses; ssRNA negative-strand viruses; Mononegavirales; Rhabdoviridae; Novirhabdovirus
GCA_000879455.1	231255	343	Ralstonia phage phiRSL1	NA	Viruses; dsDNA viruses, no RNA stage; Caudovirales; Myoviridae; Rslunavirus; Ralstonia virus RSL1
GCA_000875305.1	316674	461	Pseudomonas phage 201phi21	NA	Viruses; dsDNA viruses, no RNA stage; Caudovirales; Myoviridae; Phikzvirus; unclassified phiKZ-like viruses
GCA_000852585.1	10140	1	Deformed wing virus	NA	Viruses; ssRNA viruses; ssRNA positive-strand viruses, no DNA stage; Picornavirales; Iflaviridae; Iflavirus
GCA_002622545.1	164706	262	Escherichia phage ECD7	NA	Viruses; Duplodnaviria; Heunggongvirae; Uroviricota; Caudoviricetes; Caudovirales; Myoviridae; Tevenvirinae; Krischvirus; Escherichia virus ECD7
GCA_002622565.1	129454	207	Escherichia phage V18	NA	Viruses; Duplodnaviria; Heunggongvirae; Uroviricota; Caudoviricetes; Caudovirales; Myoviridae; Vequintavirinae; Vequintavirus; Escherichia virus V18
GCA_002622585.1	142934	224	Staphylococcus phage vB_Sau_CG	NA	Viruses; Duplodnaviria; Heunggongvirae; Uroviricota; Caudoviricetes; Caudovirales; Herelleviridae; Twortvirinae; Kayvirus; unclassified Kayvirus
GCA_002622605.1	143734	213	Staphylococcus phage vB_Sau_Clo6	NA	Viruses; Duplodnaviria; Heunggongvirae; Uroviricota; Caudoviricetes; Caudovirales; Herelleviridae; Twortvirinae; Kayvirus; unclassified Kayvirus
GCA_002622625.1	139997	209	Staphylococcus phage vB_Sau_S24	NA	Viruses; Duplodnaviria; Heunggongvirae; Uroviricota; Caudoviricetes; Caudovirales; Herelleviridae; Twortvirinae; Kayvirus; unclassified Kayvirus
GCA_002118465.1	4562	2	Salmovirus WFRC1	NA	Viruses; unclassified viruses
GCA_002622645.1	21684	33	Bacillus phage Harambe	NA	Viruses; Duplodnaviria; Heunggongvirae; Uroviricota; Caudoviricetes; Caudovirales; Podoviridae; unclassified Podoviridae
GCA_002623385.1	20222	45	Sulfolobus polyhedral virus 1	NA	Viruses; Portogloboviridae; Alphaportoglobovirus; Sulfolobus alphaportoglobovirus 1
GCA_003029555.1	8259	1	Bellflower veinal mottle virus	NA	Viruses; Riboviria; Orthornavirae; Pisuviricota; Stelpaviricetes; Patatavirales; Potyviridae; Bevemovirus
GCA_004788095.2	5128	7	Jacquemontia yellow vein virus	NA	Viruses; Monodnaviria; Shotokuvirae; Cressdnaviricota; Repensiviricetes; Geplafuvirales; Geminiviridae; Begomovirus
GCA_008792705.1	7984	1	Saffold virus 3	NA	Viruses; Riboviria; Orthornavirae; Pisuviricota; Pisoniviricetes; Picornavirales; Picornaviridae; Cardiovirus; Cardiovirus B
GCA_008765705.1	12477	4	Bipolaris maydis chrysovirus 1	SS01	Viruses; Riboviria; Orthornavirae; Duplornaviricota; Chrymotiviricetes; Ghabrivirales; Chrysoviridae; Alphachrysovirus; Helminthosporium victoriae virus 145S
GCA_000920315.1	69864	97	Mycobacterium virus Acadian	NA	Viruses; Duplodnaviria; Heunggongvirae; Uroviricota; Caudoviricetes; Caudovirales; Siphoviridae; Bclasvirinae; Acadianvirus
GCA_000928335.1	7836	5	Rubus yellow net virus	Baumforths Seedling A	Viruses; Riboviria; Pararnavirae; Artverviricota; Revtraviricetes; Ortervirales; Caulimoviridae; Badnavirus
GCA_002623445.1	48502	81	Mycobacterium phage Journey13	NA	Viruses; Duplodnaviria; Heunggongvirae; Uroviricota; Caudoviricetes; Caudovirales; Siphoviridae; Fromanvirus; unclassified Fromanvirus
GCA_002623465.1	45888	89	Acinetobacter phage WCHABP1	NA	Viruses; Duplodnaviria; Heunggongvirae; Uroviricota; Caudoviricetes; Caudovirales; Myoviridae; Obolenskvirus; Acinetobacter virus WCHABP1
GCA_002623485.1	27206	44	Bacillus phage SerPounce	NA	Viruses; Duplodnaviria; Heunggongvirae; Uroviricota; Caudoviricetes; Caudovirales; Podoviridae; unclassified Podoviridae
GCA_002623505.1	60618	96	Mycobacterium phage Shandong1	NA	Viruses; Duplodnaviria; Heunggongvirae; Uroviricota; Caudoviricetes; Caudovirales; Siphoviridae; Timquatrovirus; unclassified Timquatrovirus
GCA_002623525.1	121342	207	Bradyrhizobium phage BDUMI1	NA	Viruses; Duplodnaviria; Heunggongvirae; Uroviricota; Caudoviricetes; Caudovirales; Myoviridae; unclassified Myoviridae
GCA_002623545.1	21054	30	Bacillus phage BeachBum	NA	Viruses; Duplodnaviria; Heunggongvirae; Uroviricota; Caudoviricetes; Caudovirales; Podoviridae; unclassified Podoviridae
GCA_003051265.1	62020	80	Escherichia phage ArgO145	NA	Viruses; Duplodnaviria; Heunggongvirae; Uroviricota; Caudoviricetes; Caudovirales; Podoviridae; Sepvirinae; Oslovirus; unclassified Oslovirus
GCA_002623565.1	166137	288	Bacillus phage Flapjack	NA	Viruses; Duplodnaviria; Heunggongvirae; Uroviricota; Caudoviricetes; Caudovirales; Herelleviridae; Bastillevirinae; Bequatrovirus; unclassified Bequatrovirus
GCA_002623585.1	40409	47	Acinetobacter phage WCHABP5	NA	Viruses; Duplodnaviria; Heunggongvirae; Uroviricota; Caudoviricetes; Caudovirales; Autographiviridae; Beijerinckvirinae; Friunavirus; Acinetobacter virus WCHABP5
GCA_002145885.1	8019	2	Picornalike virus AWando15	NA	Viruses; ssRNA viruses; ssRNA positive-strand viruses, no DNA stage; Picornavirales; unclassified Picornavirales
GCA_003307335.2	30189	56	Lactococcus phage PLgW1	NA	Viruses; Duplodnaviria; Heunggongvirae; Uroviricota; Caudoviricetes; Caudovirales; Siphoviridae; Skunavirus; unclassified Skunavirus
GCA_002623605.1	87646	156	Pseudomonas phage vB_PaeM_G1	NA	Viruses; Duplodnaviria; Heunggongvirae; Uroviricota; Caudoviricetes; Caudovirales; Myoviridae; Nankokuvirus; Pseudomonas virus G1
GCA_002743535.1	19089	25	Clostridium phage CPS1	NA	Viruses; Duplodnaviria; Heunggongvirae; Uroviricota; Caudoviricetes; Caudovirales; Podoviridae; unclassified Podoviridae
GCA_002623625.1	49016	78	Flavobacterium phage V175	NA	Viruses; Duplodnaviria; Heunggongvirae; Uroviricota; Caudoviricetes; Caudovirales; Myoviridae; Ficleduovirus; unclassified Ficleduovirus

gca	genome.size	proteome.size	phage.name	strain	Taxonomy
GCA_002623645.1	49014	77	Flavobacterium phage V181	NA	Viruses; Duplodnaviria; Heunggongvirae; Uroviricota; Caudoviricetes; Caudovirales; Myoviridae; Ficleduovirus; unclassified Ficleduovirus
GCA_002955045.1	261365	333	Erwinia phage vB_EamM_Y3	NA	Viruses; Duplodnaviria; Heunggongvirae; Uroviricota; Caudoviricetes; Caudovirales; Myoviridae; Sasquatchvirus; Erwinia virus Y3
GCA_002623665.1	49088	80	Escherichia phage vB_EcoS_SH2	NA	Viruses; Duplodnaviria; Heunggongvirae; Uroviricota; Caudoviricetes; Caudovirales; Drexlerviridae; Tunavirinae; Tunavirus; Escherichia virus SH2
GCA_003226615.1	58279	67	Pseudomonas phage PspYZU01	NA	Viruses; Duplodnaviria; Heunggongvirae; Uroviricota; Caudoviricetes; Caudovirales; Siphoviridae; unclassified Siphoviridae
GCA_003226635.1	166442	224	Pseudomonas phage PspYZU05	NA	Viruses; Duplodnaviria; Heunggongvirae; Uroviricota; Caudoviricetes; Caudovirales; Myoviridae; Tevenvirinae; unclassified Tevenvirinae
GCA_003226655.1	40184	48	Pseudomonas phage PspYZU08	NA	Viruses; Duplodnaviria; Heunggongvirae; Uroviricota; Caudoviricetes; Caudovirales; Autographiviridae; Studiervirinae; Pijolavirus; Pseudomonas virus PspYZU08
GCA_002210515.1	5921	4	Lake Sinai Virus NE	NA	Viruses; Virus families not assigned to an order; Sinaivirus; unclassified Sinaivirus
GCA_002237195.1	5889	3	Lake Sinai Virus SA1	NA	Viruses; Virus families not assigned to an order; Sinaivirus; unclassified Sinaivirus
GCA_002210875.1	5898	4	Lake Sinai Virus SA2	NA	Viruses; Virus families not assigned to an order; Sinaivirus; unclassified Sinaivirus
GCA_002210715.1	5877	4	Lake Sinai Virus TO	NA	Viruses; Virus families not assigned to an order; Sinaivirus; unclassified Sinaivirus
GCA_002210615.1	9126	2	Apis dicistrovirus	NA	Viruses; ssRNA viruses; ssRNA positive-strand viruses, no DNA stage; Picornavirales; Dicistroviridae; unclassified Dicistroviridae
GCA_002204045.1	20414	1	Apis flavivirus	NA	Viruses; ssRNA viruses; ssRNA positive-strand viruses, no DNA stage; Flaviviridae; unclassified Flaviviridae
GCA_002623685.1	174231	204	Synechococcus phage SH35	NA	Viruses; Duplodnaviria; Heunggongvirae; Uroviricota; Caudoviricetes; Caudovirales; Myoviridae; unclassified Myoviridae
GCA_002623705.1	71726	102	Mycobacterium phage Zenon	NA	Viruses; Duplodnaviria; Heunggongvirae; Uroviricota; Caudoviricetes; Caudovirales; Siphoviridae; Papyrusvirus; unclassified Papyrusvirus
GCA_002623725.1	68714	101	Mycobacterium phage BlackStallion	NA	Viruses; Duplodnaviria; Heunggongvirae; Uroviricota; Caudoviricetes; Caudovirales; Siphoviridae; Bclasvirinae; Pegunavirus; unclassified Pegunavirus
GCA_002623745.1	53085	92	Mycobacterium phage Heffalump	NA	Viruses; Duplodnaviria; Heunggongvirae; Uroviricota; Caudoviricetes; Caudovirales; Siphoviridae; Fromanvirus; unclassified Fromanvirus
GCA_002623765.1	56663	103	Mycobacterium phage Pippy	NA	Viruses; Duplodnaviria; Heunggongvirae; Uroviricota; Caudoviricetes; Caudovirales; Siphoviridae; Cheoctovirus; unclassified Cheoctovirus
GCA_002623785.1	48596	83	Mycobacterium phage PurpleHaze	NA	Viruses; Duplodnaviria; Heunggongvirae; Uroviricota; Caudoviricetes; Caudovirales; Siphoviridae; Fromanvirus; unclassified Fromanvirus
GCA_002623805.1	40165	56	Bacillus phage Carmel_SA	NA	Viruses; Duplodnaviria; Heunggongvirae; Uroviricota; Caudoviricetes; Caudovirales; Siphoviridae; Wbetavirus; unclassified Wbetavirus
GCA_002623825.1	40375	57	Bacillus phage Negev_SA	NA	Viruses; Duplodnaviria; Heunggongvirae; Uroviricota; Caudoviricetes; Caudovirales; Siphoviridae; Wbetavirus; unclassified Wbetavirus
GCA_002623865.1	40397	61	Bacillus phage Tavor_SA	NA	Viruses; Duplodnaviria; Heunggongvirae; Uroviricota; Caudoviricetes; Caudovirales; Siphoviridae; Wbetavirus; unclassified Wbetavirus
GCA_003181195.1	44549	59	Pectobacterium phage vB_PatP_CB5	NA	Viruses; Duplodnaviria; Heunggongvirae; Uroviricota; Caudoviricetes; Caudovirales; Autographiviridae; Corkvirinae; Phimunavirus; Pectobacterium virus CB5
GCA_002623885.1	46481	74	Flavobacterium phage FCV11	NA	Viruses; Duplodnaviria; Heunggongvirae; Uroviricota; Caudoviricetes; Caudovirales; Myoviridae; Ficleduovirus; unclassified Ficleduovirus
GCA_002623905.1	46496	74	Flavobacterium phage FCV3	NA	Viruses; Duplodnaviria; Heunggongvirae; Uroviricota; Caudoviricetes; Caudovirales; Myoviridae; Ficleduovirus; unclassified Ficleduovirus
GCA_002955055.1	113299	176	Shigella phage SSP1	NA	Viruses; Duplodnaviria; Heunggongvirae; Uroviricota; Caudoviricetes; Caudovirales; Demerecviridae; Markadamsvirinae; Tequintavirus; Shigella virus SSP1
GCA_003307355.2	29342	55	Lactococcus phage PLgY16	NA	Viruses; Duplodnaviria; Heunggongvirae; Uroviricota; Caudoviricetes; Caudovirales; Siphoviridae; Skunavirus; unclassified Skunavirus
GCA_003307375.2	29284	55	Lactococcus phage PLgY30	NA	Viruses; Duplodnaviria; Heunggongvirae; Uroviricota; Caudoviricetes; Caudovirales; Siphoviridae; Skunavirus; unclassified Skunavirus
GCA_002955065.1	126082	227	Vibrio phage JSF1	NA	Viruses; Duplodnaviria; Heunggongvirae; Uroviricota; Caudoviricetes; Caudovirales; Myoviridae; unclassified Myoviridae
GCA_002955075.1	111671	149	Vibrio phage JSF10	NA	Viruses; Duplodnaviria; Heunggongvirae; Uroviricota; Caudoviricetes; Caudovirales; Demerecviridae; Ermolyevavirinae; Jesfedecavirus; Vibrio virus JSF10
GCA_002955085.1	39341	50	Vibrio phage JSF11	NA	Viruses; Duplodnaviria; Heunggongvirae; Uroviricota; Caudoviricetes; Caudovirales; Autographiviridae; Studiervirinae; Chatterjeevirus; unclassified Chatterjeevirus
GCA_002955095.1	111671	152	Vibrio phage JSF12	NA	Viruses; Duplodnaviria; Heunggongvirae; Uroviricota; Caudoviricetes; Caudovirales; Demerecviridae; Ermolyevavirinae; Jesfedecavirus; Vibrio virus JSF12
GCA_002955105.1	128814	225	Vibrio phage JSF13	NA	Viruses; Duplodnaviria; Heunggongvirae; Uroviricota; Caudoviricetes; Caudovirales; Myoviridae; unclassified Myoviridae
GCA_002955115.1	125096	217	Vibrio phage JSF14	NA	Viruses; Duplodnaviria; Heunggongvirae; Uroviricota; Caudoviricetes; Caudovirales; Myoviridae; unclassified Myoviridae
GCA_002955125.1	39643	46	Vibrio phage JSF15	NA	Viruses; Duplodnaviria; Heunggongvirae; Uroviricota; Caudoviricetes; Caudovirales; Podoviridae; Enhodamvirus; unclassified Enhodamvirus

gca	genome.size	proteome.size	phage.name	strain	Taxonomy
GCA_002955135.1	125174	217	Vibrio phage JSF17	NA	Viruses; Duplodnaviria; Heunggongvirae; Uroviricota; Caudoviricetes; Caudovirales; Myoviridae; unclassified Myoviridae
GCA_002955145.1	38570	47	Vibrio phage JSF18	NA	Viruses; Duplodnaviria; Heunggongvirae; Uroviricota; Caudoviricetes; Caudovirales; Autographiviridae; Studiervirinae; Chatterjeevirus; unclassified Chatterjeevirus
GCA_002955155.1	126082	229	Vibrio phage JSF2	NA	Viruses; Duplodnaviria; Heunggongvirae; Uroviricota; Caudoviricetes; Caudovirales; Myoviridae; unclassified Myoviridae
GCA_002955165.1	39378	48	Vibrio phage JSF20	NA	Viruses; Duplodnaviria; Heunggongvirae; Uroviricota; Caudoviricetes; Caudovirales; Autographiviridae; Studiervirinae; Chatterjeevirus; unclassified Chatterjeevirus
GCA_002955175.1	50325	69	Vibrio phage JSF23	NA	Viruses; Duplodnaviria; Heunggongvirae; Uroviricota; Caudoviricetes; Caudovirales; Podoviridae; unclassified Podoviridae
GCA_002955185.1	39378	48	Vibrio phage JSF24	NA	Viruses; Duplodnaviria; Heunggongvirae; Uroviricota; Caudoviricetes; Caudovirales; Autographiviridae; Studiervirinae; Chatterjeevirus; unclassified Chatterjeevirus
GCA_002955195.1	48690	69	Vibrio phage JSF27	NA	Viruses; Duplodnaviria; Heunggongvirae; Uroviricota; Caudoviricetes; Caudovirales; Podoviridae; unclassified Podoviridae
GCA_002955205.1	38489	48	Vibrio phage JSF28	NA	Viruses; Duplodnaviria; Heunggongvirae; Uroviricota; Caudoviricetes; Caudovirales; Autographiviridae; Studiervirinae; Chatterjeevirus; unclassified Chatterjeevirus
GCA_002955215.1	38056	48	Vibrio phage JSF30	NA	Viruses; Duplodnaviria; Heunggongvirae; Uroviricota; Caudoviricetes; Caudovirales; Autographiviridae; Studiervirinae; Chatterjeevirus; unclassified Chatterjeevirus
GCA_002955225.1	38581	47	Vibrio phage JSF31	NA	Viruses; Duplodnaviria; Heunggongvirae; Uroviricota; Caudoviricetes; Caudovirales; Autographiviridae; Studiervirinae; Chatterjeevirus; unclassified Chatterjeevirus
GCA_002955235.1	38581	48	Vibrio phage JSF32	NA	Viruses; Duplodnaviria; Heunggongvirae; Uroviricota; Caudoviricetes; Caudovirales; Autographiviridae; Studiervirinae; Chatterjeevirus; unclassified Chatterjeevirus
GCA_002955245.1	39661	46	Vibrio phage JSF33	NA	Viruses; Duplodnaviria; Heunggongvirae; Uroviricota; Caudoviricetes; Caudovirales; Podoviridae; Enhodamvirus; unclassified Enhodamvirus
GCA_002955255.1	39378	48	Vibrio phage JSF34	NA	Viruses; Duplodnaviria; Heunggongvirae; Uroviricota; Caudoviricetes; Caudovirales; Autographiviridae; Studiervirinae; Chatterjeevirus; unclassified Chatterjeevirus
GCA_002955265.1	38688	48	Vibrio phage JSF35	NA	Viruses; Duplodnaviria; Heunggongvirae; Uroviricota; Caudoviricetes; Caudovirales; Autographiviridae; Studiervirinae; Chatterjeevirus; unclassified Chatterjeevirus
GCA_002955275.1	38581	47	Vibrio phage JSF36	NA	Viruses; Duplodnaviria; Heunggongvirae; Uroviricota; Caudoviricetes; Caudovirales; Autographiviridae; Studiervirinae; Chatterjeevirus; unclassified Chatterjeevirus
GCA_002955285.1	132142	232	Vibrio phage JSF5	NA	Viruses; Duplodnaviria; Heunggongvirae; Uroviricota; Caudoviricetes; Caudovirales; Myoviridae; unclassified Myoviridae
GCA_002955295.1	133685	232	Vibrio phage JSF6	NA	Viruses; Duplodnaviria; Heunggongvirae; Uroviricota; Caudoviricetes; Caudovirales; Myoviridae; unclassified Myoviridae
GCA_002955305.1	40007	51	Vibrio phage JSF9	NA	Viruses; Duplodnaviria; Heunggongvirae; Uroviricota; Caudoviricetes; Caudovirales; Podoviridae; Enhodamvirus; unclassified Enhodamvirus
GCA_002623925.1	29533	44	Propionibacterium phage PacnesP1	NA	Viruses; Duplodnaviria; Heunggongvirae; Uroviricota; Caudoviricetes; Caudovirales; Siphoviridae; Pahexavirus; unclassified Pahexavirus
GCA_002623945.1	30016	44	Propionibacterium phage PacnesP2	NA	Viruses; Duplodnaviria; Heunggongvirae; Uroviricota; Caudoviricetes; Caudovirales; Siphoviridae; Pahexavirus; unclassified Pahexavirus
GCA_002623965.1	153757	199	Dickeya phage JA15	NA	Viruses; Duplodnaviria; Heunggongvirae; Uroviricota; Caudoviricetes; Caudovirales; Ackermannviridae; Aglimvirinae; Limestonevirus; unclassified Limestonevirus
GCA_002623985.1	151519	196	Dickeya phage XF4	NA	Viruses; Duplodnaviria; Heunggongvirae; Uroviricota; Caudoviricetes; Caudovirales; Ackermannviridae; Aglimvirinae; Limestonevirus; unclassified Limestonevirus
GCA_003848445.1	2155	3	Gemycircularvirus sp	M64gHun2013	Viruses; Monodnaviria; Shotokuvirae; Cressdnaviricota; Repensiviricetes; Geplafuvirales; Genomoviridae; Gemycircularvirus; unclassified Gemycircularvirus
GCA_002197575.1	14964	6	Avian paramyxovirus 15	NA	Viruses; ssRNA viruses; ssRNA negative-strand viruses; Mononegavirales; Paramyxoviridae; Avulavirus; unclassified Avulavirus
GCA_004050735.1	1936	2	Tick circovirus	NA	Viruses; Monodnaviria; Shotokuvirae; Cressdnaviricota; Arfiviricetes; Cirlivirales; Circoviridae; Circovirus; Tick associated circovirus 2
GCA_003571845.1	84008	124	Curvibacter phage P26059A	NA	Viruses; Duplodnaviria; Heunggongvirae; Uroviricota; Caudoviricetes; Caudovirales; Siphoviridae; unclassified Siphoviridae
GCA_003571865.1	41344	46	Curvibacter phage P26059B	NA	Viruses; Duplodnaviria; Heunggongvirae; Uroviricota; Caudoviricetes; Caudovirales; Autographiviridae; Kalppathivirus; Curvibacter virus P26059B
GCA_002624065.1	39693	45	Escherichia phage ST31	NA	Viruses; Duplodnaviria; Heunggongvirae; Uroviricota; Caudoviricetes; Caudovirales; Autographiviridae; Studiervirinae; Kayfunavirus; Escherichia virus ST31
GCA_002624085.1	46482	74	Flavobacterium phage FCV10	NA	Viruses; Duplodnaviria; Heunggongvirae; Uroviricota; Caudoviricetes; Caudovirales; Myoviridae; Ficleduovirus; unclassified Ficleduovirus
GCA_002624105.1	46481	74	Flavobacterium phage FCV16	NA	Viruses; Duplodnaviria; Heunggongvirae; Uroviricota; Caudoviricetes; Caudovirales; Myoviridae; Ficleduovirus; unclassified Ficleduovirus
GCA_002624125.1	48564	78	Flavobacterium phage V156	NA	Viruses; Duplodnaviria; Heunggongvirae; Uroviricota; Caudoviricetes; Caudovirales; Myoviridae; Ficleduovirus; unclassified Ficleduovirus
GCA_002624145.1	48565	78	Flavobacterium phage V157	NA	Viruses; Duplodnaviria; Heunggongvirae; Uroviricota; Caudoviricetes; Caudovirales; Myoviridae; Ficleduovirus; unclassified Ficleduovirus
GCA_002624165.1	49013	78	Flavobacterium phage V165	NA	Viruses; Duplodnaviria; Heunggongvirae; Uroviricota; Caudoviricetes; Caudovirales; Myoviridae; Ficleduovirus; unclassified Ficleduovirus
GCA_002624185.1	49099	79	Flavobacterium phage V182	NA	Viruses; Duplodnaviria; Heunggongvirae; Uroviricota; Caudoviricetes; Caudovirales; Myoviridae; Ficleduovirus; unclassified Ficleduovirus

gca	genome.size	proteome.size	phage.name	strain	Taxonomy
GCA_002624205.1	46496	74	Flavobacterium phage VK20	NA	Viruses; Duplodnaviria; Heunggongvirae; Uroviricota; Caudoviricetes; Caudovirales; Myoviridae; Ficleduovirus; unclassified Ficleduovirus
GCA_002624225.1	46455	74	Flavobacterium phage VK42	NA	Viruses; Duplodnaviria; Heunggongvirae; Uroviricota; Caudoviricetes; Caudovirales; Myoviridae; Ficleduovirus; unclassified Ficleduovirus
GCA_002624245.1	46570	76	Flavobacterium phage VK48	NA	Viruses; Duplodnaviria; Heunggongvirae; Uroviricota; Caudoviricetes; Caudovirales; Myoviridae; Ficleduovirus; unclassified Ficleduovirus
GCA_002624265.1	46455	74	Flavobacterium phage VK52	NA	Viruses; Duplodnaviria; Heunggongvirae; Uroviricota; Caudoviricetes; Caudovirales; Myoviridae; Ficleduovirus; unclassified Ficleduovirus
GCA_002624285.1	46448	75	Flavobacterium phage VK58	NA	Viruses; Duplodnaviria; Heunggongvirae; Uroviricota; Caudoviricetes; Caudovirales; Myoviridae; Ficleduovirus; unclassified Ficleduovirus
GCA_002624305.1	46496	74	Flavobacterium phage FCV20	NA	Viruses; Duplodnaviria; Heunggongvirae; Uroviricota; Caudoviricetes; Caudovirales; Myoviridae; Ficleduovirus; unclassified Ficleduovirus
GCA_000847245.1	35524	38	Simian adenovirus 21	NA	Viruses; dsDNA viruses, no RNA stage; Adenoviridae; Mastadenovirus; Human mastadenovirus B; Human adenovirus B2
GCA_002210495.1	2389	1	Neofusicoccum luteum mitovirus 1	NA	Viruses; ssRNA viruses; ssRNA positive-strand viruses, no DNA stage; Narnaviridae; unclassified Narnaviridae
GCA_002204065.1	3420	2	Spinach amalgavirus 1	NA	Viruses; dsRNA viruses; Amalgaviridae; unclassified Amalgaviridae
GCA_002617825.1	165213	266	Bacillus virus BM15	NA	Viruses; Duplodnaviria; Heunggongvirae; Uroviricota; Caudoviricetes; Caudovirales; Herelleviridae; Bastillevirinae; Caeruleovirus
GCA_004130495.1	6244	2	Neofusicoccum luteum fusarivirus 1	NA	Viruses; Riboviria; unclassified Riboviria; unclassified ssRNA viruses; Fusariviridae
GCA_002184155.1	3383	2	Zostera marina amalgavirus 1	NA	Viruses; dsRNA viruses; Amalgaviridae; unclassified Amalgaviridae
GCA_002153845.1	3316	2	Zostera marina amalgavirus 2	NA	Viruses; dsRNA viruses; Amalgaviridae; unclassified Amalgaviridae
GCA_000907255.1	11279	1	Pegivirus J	NA	Viruses; Riboviria; Orthornavirae; Kitrinoviricota; Flasuviricetes; Amarillovirales; Flaviviridae; Pegivirus
GCA_002118605.1	9760	1	Pegivirus K	NA	Viruses; Riboviria; Orthornavirae; Kitrinoviricota; Flasuviricetes; Amarillovirales; Flaviviridae; Pegivirus
GCA_000927895.1	2197	2	Blackbird associated gemycircularvirus 1	NA	Viruses; Monodnaviria; Shotokuvirae; Cressdnaviricota; Repensiviricetes; Geplafuvirales; Genomoviridae; Gemycircularvirus
GCA_000919515.1	2220	2	Cassava associated gemycircularvirus 1	NA	Viruses; Monodnaviria; Shotokuvirae; Cressdnaviricota; Repensiviricetes; Geplafuvirales; Genomoviridae; Gemycircularvirus
GCA_002825685.1	2193	2	Miniopterus associated gemycircularvirus 1	NA	Viruses; Monodnaviria; Shotokuvirae; Cressdnaviricota; Repensiviricetes; Geplafuvirales; Genomoviridae; Gemycircularvirus
GCA_002825725.1	2145	2	Odonata associated gemycircularvirus 1	NA	Viruses; Monodnaviria; Shotokuvirae; Cressdnaviricota; Repensiviricetes; Geplafuvirales; Genomoviridae; Gemycircularvirus
GCA_002825745.1	2190	2	Odonata associated gemycircularvirus 2	NA	Viruses; Monodnaviria; Shotokuvirae; Cressdnaviricota; Repensiviricetes; Geplafuvirales; Genomoviridae; Gemycircularvirus
GCA_001292915.1	2253	3	Poaceae associated gemycircularvirus 1	NA	Viruses; Monodnaviria; Shotokuvirae; Cressdnaviricota; Repensiviricetes; Geplafuvirales; Genomoviridae; Gemycircularvirus
GCA_002825805.1	2172	3	Pteropus associated gemycircularvirus 3	NA	Viruses; Monodnaviria; Shotokuvirae; Cressdnaviricota; Repensiviricetes; Geplafuvirales; Genomoviridae; Gemycircularvirus
GCA_002825825.1	2250	3	Pteropus associated gemycircularvirus 4	NA	Viruses; Monodnaviria; Shotokuvirae; Cressdnaviricota; Repensiviricetes; Geplafuvirales; Genomoviridae; Gemycircularvirus
GCA_002825845.1	2205	3	Pteropus associated gemycircularvirus 5	NA	Viruses; Monodnaviria; Shotokuvirae; Cressdnaviricota; Repensiviricetes; Geplafuvirales; Genomoviridae; Gemycircularvirus
GCA_002825865.1	2230	3	Pteropus associated gemycircularvirus 6	NA	Viruses; Monodnaviria; Shotokuvirae; Cressdnaviricota; Repensiviricetes; Geplafuvirales; Genomoviridae; Gemycircularvirus
GCA_002825885.1	2155	3	Pteropus associated gemycircularvirus 7	NA	Viruses; Monodnaviria; Shotokuvirae; Cressdnaviricota; Repensiviricetes; Geplafuvirales; Genomoviridae; Gemycircularvirus
GCA_002825905.1	2185	3	Pteropus associated gemycircularvirus 8	NA	Viruses; Monodnaviria; Shotokuvirae; Cressdnaviricota; Repensiviricetes; Geplafuvirales; Genomoviridae; Gemycircularvirus
GCA_002825925.1	2140	3	Pteropus associated gemycircularvirus 9	NA	Viruses; Monodnaviria; Shotokuvirae; Cressdnaviricota; Repensiviricetes; Geplafuvirales; Genomoviridae; Gemycircularvirus
GCA_002825945.1	2209	3	Pteropus associated gemycircularvirus 10	NA	Viruses; Monodnaviria; Shotokuvirae; Cressdnaviricota; Repensiviricetes; Geplafuvirales; Genomoviridae; Gemycircularvirus
GCA_000928275.1	2234	3	Sewage derived gemycircularvirus 1	NA	Viruses; Monodnaviria; Shotokuvirae; Cressdnaviricota; Repensiviricetes; Geplafuvirales; Genomoviridae; Gemycircularvirus
GCA_000930215.1	2196	3	Sewage derived gemycircularvirus 2	NA	Viruses; Monodnaviria; Shotokuvirae; Cressdnaviricota; Repensiviricetes; Geplafuvirales; Genomoviridae; Gemycircularvirus
GCA_001448415.2	2231	2	Soybean associated gemycircularvirus 1	SlaGemV11	Viruses; Monodnaviria; Shotokuvirae; Cressdnaviricota; Repensiviricetes; Geplafuvirales; Genomoviridae; Gemycircularvirus
GCA_001448435.1	2272	3	Human associated gemyvongvirus 1	NA	Viruses; Monodnaviria; Shotokuvirae; Cressdnaviricota; Repensiviricetes; Geplafuvirales; Genomoviridae; Gemyvongvirus
GCA_000881815.1	12878	6	Lettuce bigvein associated varicosavirus	NA	Viruses; ssRNA viruses; ssRNA negative-strand viruses; Mononegavirales; Rhabdoviridae; Varicosavirus

gca	genome.size	proteome.size	phage.name	strain	Taxonomy
GCA_001432155.1	12706	10	Barley yellow striate mosaic cytorhabdovirus	Hebei	Viruses; ssRNA viruses; ssRNA negative-strand viruses; Mononegavirales; Rhabdoviridae; Cytorhabdovirus
GCA_000854665.1	13222	8	Northern cereal mosaic cytorhabdovirus	NA	Viruses; ssRNA viruses; ssRNA negative-strand viruses; Mononegavirales; Rhabdoviridae; Cytorhabdovirus
GCA_018583675.1	14545	8	Strawberry crinkle cytorhabdovirus	NA	Viruses; Riboviria; Orthornavirae; Negarnaviricota; Haploviricotina; Monjiviricetes; Mononegavirales; Rhabdoviridae; Cytorhabdovirus
GCA_000851905.2	2844	2	Beet western yellows ST9 associated virus	NA	Viruses; ssRNA viruses; ssRNA positive-strand viruses, no DNA stage; unclassified ssRNA positive-strand viruses
GCA_002158655.1	8349	6	Alfalfa virus S	NA	Viruses; ssRNA viruses; ssRNA positive-strand viruses, no DNA stage; Tymovirales; unclassified Tymovirales
GCA_000850925.1	2617	1	Ophiostoma mitovirus 3a	NA	Viruses; ssRNA viruses; ssRNA positive-strand viruses, no DNA stage; Narnaviridae; Mitovirus
GCA_000853185.1	2891	1	Saccharomyces 23S RNA narnavirus	374C	Viruses; ssRNA viruses; ssRNA positive-strand viruses, no DNA stage; Narnaviridae; Narnavirus
GCA_001275455.1	4532	6	Chlamydia virus CPAR39	NA	Viruses; ssDNA viruses; Microviridae; Gokushovirinae; Chlamydiamicrovirus
GCA_002624565.1	5508	4	Human gokushovirus	NA	Viruses; Monodnaviria; Sangervirae; Phixviricota; Malgrandaviricetes; Petitvirales; Microviridae; Gokushovirinae; unclassified Gokushovirinae
GCA_002922305.1	5135	6	Human gut gokushovirus	NA	Viruses; Monodnaviria; Sangervirae; Phixviricota; Malgrandaviricetes; Petitvirales; Microviridae; Gokushovirinae; unclassified Gokushovirinae
GCA_002624585.1	6363	4	Human gut microviridae SHCHD12	NA	Viruses; Monodnaviria; Sangervirae; Phixviricota; Malgrandaviricetes; Petitvirales; Microviridae; unclassified Microviridae
GCA_002624605.1	6312	4	Human gut microviridae SHCHD8	NA	Viruses; Monodnaviria; Sangervirae; Phixviricota; Malgrandaviricetes; Petitvirales; Microviridae; unclassified Microviridae
GCA_000924075.1	33452	8	Ball python nidovirus 1	0753	Viruses; ssRNA viruses; ssRNA positive-strand viruses, no DNA stage; Nidovirales; Coronaviridae; Torovirinae
GCA_002819225.1	107439	123	Clostera anastomosis granulovirus B	ClasGVB	Viruses; Baculoviridae; Betabaculovirus
GCA_000911215.1	101818	122	Clostera anastomosis granulovirus Henan	NA	Viruses; Baculoviridae; Betabaculovirus; Clostera anastomosis granulovirus A
GCA_002210855.1	2199	2	Bemisiaassociated genomovirus AdDF	NA	Viruses; Genomoviridae; unclassified Genomoviridae
GCA_002211035.1	2211	2	Bemisiaassociated genomovirus AdO	NA	Viruses; Genomoviridae; unclassified Genomoviridae
GCA_002210835.1	2231	2	Bemisiaassociated genomovirus NfO	NA	Viruses; Genomoviridae; unclassified Genomoviridae
GCA_002158575.1	29923	29	Bat mastadenovirus WIV17	NA	Viruses; dsDNA viruses, no RNA stage; Adenoviridae; Mastadenovirus; Bat mastadenovirus G
GCA_002366205.1	29812	30	Bat mastadenovirus WIV18	NA	Viruses; Varidnaviria; Bamfordvirae; Preplasmiviricota; Tectiliviricetes; Rowavirales; Adenoviridae; Mastadenovirus; Bat mastadenovirus G
GCA_004117655.1	18088	8	Daphnis nerii cypovirus	NA	Viruses; Riboviria; Orthornavirae; Duplornaviricota; Resentoviricetes; Reovirales; Reoviridae; Spinareovirinae; Cypovirus; unclassified Cypovirus
GCA_002815875.1	12674	8	Ekpoma virus 2	NA	Viruses; Riboviria; Orthornavirae; Negarnaviricota; Haploviricotina; Monjiviricetes; Mononegavirales; Rhabdoviridae; Tibrovirus; Ekpoma 2 tibrovirus
GCA_002816915.1	7570	1	Bat hepatovirus SMG18520Minmav2014	NA	Viruses; Riboviria; Orthornavirae; Pisuviricota; Pisoniviricetes; Picornavirales; Picornaviridae; Hepatovirus; Hepatovirus C
GCA_001444005.1	7656	1	Rodent hepatovirus RMU101637Micarv2010	NA	Viruses; Riboviria; Orthornavirae; Pisuviricota; Pisoniviricetes; Picornavirales; Picornaviridae; Hepatovirus; Hepatovirus D
GCA_002816965.1	7406	1	Rodent hepatovirus CIV459Lopsik2004	NA	Viruses; Riboviria; Orthornavirae; Pisuviricota; Pisoniviricetes; Picornavirales; Picornaviridae; Hepatovirus; Hepatovirus E
GCA_002817005.1	7563	1	Rodent hepatovirus KEF121Sigmas2012	NA	Viruses; Riboviria; Orthornavirae; Pisuviricota; Pisoniviricetes; Picornavirales; Picornaviridae; Hepatovirus; Hepatovirus F
GCA_002817035.1	7399	1	Bat hepatovirus BUO2BF86Colafr2010	NA	Viruses; Riboviria; Orthornavirae; Pisuviricota; Pisoniviricetes; Picornavirales; Picornaviridae; Hepatovirus; Hepatovirus G
GCA_001444085.1	6929	1	Hedgehog hepatovirus Igel8Erieur2014	NA	Viruses; Riboviria; Orthornavirae; Pisuviricota; Pisoniviricetes; Picornavirales; Picornaviridae; Hepatovirus; Hepatovirus H
GCA_001443885.1	7810	1	Shrew hepatovirus KS121232Sorara2012	NA	Viruses; Riboviria; Orthornavirae; Pisuviricota; Pisoniviricetes; Picornavirales; Picornaviridae; Hepatovirus; Hepatovirus I
GCA_000895155.1	157304	204	Escherichia virus CBA120	vB_EcoM_CBA120	Viruses; dsDNA viruses, no RNA stage; Caudovirales; Myoviridae; Vi1virus
GCA_002158635.1	2971	7	Pepper yellow dwarf virus Mexico	PepYDVCurto	Viruses; Monodnaviria; Shotokuvirae; Cressdnaviricota; Repensiviricetes; Geplafuvirales; Geminiviridae; Curtovirus; unclassified Curtovirus
GCA_002828025.1	8174	1	Broadleafed dock virus A	NA	Viruses; Riboviria; Orthornavirae; Pisuviricota; Stelpaviricetes; Patatavirales; Potyviridae; Macluravirus
GCA_000915935.1	124688	147	Choristoneura murinana nucleopolyhedrovirus	Darmstadt	Viruses; dsDNA viruses, no RNA stage; Baculoviridae; Alphabaculovirus
GCA_002158775.1	207914	186	Ranid herpesvirus 3	NA	Viruses; dsDNA viruses, no RNA stage; Herpesvirales; Alloherpesviridae; Batrachovirus; unclassified Batrachovirus

gca	genome.size	proteome.size	phage.name	strain	Taxonomy
GCA_002184175.1	8541	1	Harrier picornavirus 1	harrierMR01HUN2014	Viruses; ssRNA viruses; ssRNA positive-strand viruses, no DNA stage; Picornavirales; Picornaviridae; Megrivirus; unclassified Megrivirus
GCA_002819705.1	1688	1	Bat associated cyclovirus 6	NA	Viruses; Monodnaviria; Shotokuvirae; Cressdnaviricota; Arfiviricetes; Cirlivirales; Circoviridae; Cyclovirus
GCA_002819725.1	2379	1	Bat associated cyclovirus 7	NA	Viruses; Monodnaviria; Shotokuvirae; Cressdnaviricota; Arfiviricetes; Cirlivirales; Circoviridae; Cyclovirus
GCA_002819745.1	1746	1	Bat associated cyclovirus 8	NA	Viruses; Monodnaviria; Shotokuvirae; Cressdnaviricota; Arfiviricetes; Cirlivirales; Circoviridae; Cyclovirus
GCA_002819765.1	1760	1	Bat associated cyclovirus 9	NA	Viruses; Monodnaviria; Shotokuvirae; Cressdnaviricota; Arfiviricetes; Cirlivirales; Circoviridae; Cyclovirus
GCA_002819785.1	1844	1	Bat associated cyclovirus 11	NA	Viruses; Monodnaviria; Shotokuvirae; Cressdnaviricota; Arfiviricetes; Cirlivirales; Circoviridae; Cyclovirus
GCA_002819805.1	1809	1	Bat associated cyclovirus 13	NA	Viruses; Monodnaviria; Shotokuvirae; Cressdnaviricota; Arfiviricetes; Cirlivirales; Circoviridae; Cyclovirus
GCA_000890895.1	157061	208	Salmonella virus ViI	NA	Viruses; Duplodnaviria; Heunggongvirae; Uroviricota; Caudoviricetes; Caudovirales; Myoviridae; Viunavirus
GCA_000924515.1	11070	5	Vesicular stomatitis Alagoas virus	NA	Viruses; ssRNA viruses; ssRNA negative-strand viruses; Mononegavirales; Rhabdoviridae; Vesiculovirus
GCA_000851825.1	4048	5	Pea stem necrosis virus	NA	Viruses; ssRNA viruses; ssRNA positive-strand viruses, no DNA stage; Tombusviridae; Gammacarmovirus
GCA_002163385.1	9428	1	Longan witches broomassociated virus	NA	Viruses; ssRNA viruses; ssRNA positive-strand viruses, no DNA stage; Potyviridae; unclassified Potyviridae
GCA_000851125.1	12499	7	Mirafiori lettuce bigvein virus	NA	Viruses; Riboviria; Orthornavirae; Negarnaviricota; Haploviricotina; Milneviricetes; Serpentovirales; Aspiviridae; Ophiovirus; Mirafiori lettuce big-vein ophiovirus
GCA_013087465.1	11877	6	Maize associated rhabdovirus	Peru	Viruses; Riboviria; Orthornavirae; Negarnaviricota; Haploviricotina; Monjiviricetes; Mononegavirales; Rhabdoviridae; unclassified Rhabdoviridae
GCA_002819505.1	2094	1	Bat associated circovirus 5	NA	Viruses; Monodnaviria; Shotokuvirae; Cressdnaviricota; Arfiviricetes; Cirlivirales; Circoviridae; Circovirus
GCA_002819525.1	1880	1	Bat associated circovirus 6	NA	Viruses; Monodnaviria; Shotokuvirae; Cressdnaviricota; Arfiviricetes; Cirlivirales; Circoviridae; Circovirus
GCA_002819545.1	1944	1	Bat associated circovirus 7	NA	Viruses; Monodnaviria; Shotokuvirae; Cressdnaviricota; Arfiviricetes; Cirlivirales; Circoviridae; Circovirus
GCA_002819565.1	2069	1	Bat associated circovirus 8	NA	Viruses; Monodnaviria; Shotokuvirae; Cressdnaviricota; Arfiviricetes; Cirlivirales; Circoviridae; Circovirus
GCA_000839525.1	4877	12	Chlamydia virus Chp1	NA	Viruses; ssDNA viruses; Microviridae; Gokushovirinae; Chlamydiamicrovirus
GCA_000920615.1	10513	1	Sclerotinia sclerotiorum betaendornavirus 1	NA	Viruses; Riboviria; Orthornavirae; Kitrinoviricota; Alsuviricetes; Martellivirales; Endornaviridae; Betaendornavirus
GCA_002817245.1	6855	1	Bat picornavirus BatPVV113 Hun	BatPVV113Hun	Viruses; Riboviria; Orthornavirae; Pisuviricota; Pisoniviricetes; Picornavirales; Picornaviridae; Mischivirus; Mischivirus B
GCA_000931355.1	8096	1	African bat icavirus PREDICT06105	NA	Viruses; Riboviria; Orthornavirae; Pisuviricota; Pisoniviricetes; Picornavirales; Picornaviridae; Mischivirus; Mischivirus C
GCA_002117635.1	6656	1	Cosavirus F	PK5006	Viruses; ssRNA viruses; ssRNA positive-strand viruses, no DNA stage; Picornavirales; Picornaviridae; Cosavirus
GCA_002118985.1	13104	9	Hart Park virus	AR7C	Viruses; Riboviria; Orthornavirae; Negarnaviricota; Haploviricotina; Monjiviricetes; Mononegavirales; Rhabdoviridae; Hapavirus; Hart Park hapavirus
GCA_002118705.1	13209	10	Kamese virus	MP6186	Viruses; Riboviria; Orthornavirae; Negarnaviricota; Haploviricotina; Monjiviricetes; Mononegavirales; Rhabdoviridae; Hapavirus; Kamese hapavirus
GCA_002118525.1	12957	11	Mosqueiro virus	BeAr185559	Viruses; Riboviria; Orthornavirae; Negarnaviricota; Haploviricotina; Monjiviricetes; Mononegavirales; Rhabdoviridae; Hapavirus; Mosqueiro hapavirus
GCA_002815615.1	13106	10	Mossuril virus	SAAr1995	Viruses; Riboviria; Orthornavirae; Negarnaviricota; Haploviricotina; Monjiviricetes; Mononegavirales; Rhabdoviridae; Hapavirus; Mossuril hapavirus
GCA_004787795.2	5136	7	Bean leaf crumple virus	NA	Viruses; Monodnaviria; Shotokuvirae; Cressdnaviricota; Repensiviricetes; Geplafuvirales; Geminiviridae; Begomovirus
GCA_003849345.1	2265	3	Human associated gemykibivirus 1	NA	Viruses; Monodnaviria; Shotokuvirae; Cressdnaviricota; Repensiviricetes; Geplafuvirales; Genomoviridae; Gemykibivirus
GCA_003846505.1	2210	2	Human associated gemykibivirus 2	LVdogParaibaBrazil	Viruses; Monodnaviria; Shotokuvirae; Cressdnaviricota; Repensiviricetes; Geplafuvirales; Genomoviridae; Gemykibivirus
GCA_002826065.1	2153	2	Rhinolophus associated gemykibivirus 1	NA	Viruses; Monodnaviria; Shotokuvirae; Cressdnaviricota; Repensiviricetes; Geplafuvirales; Genomoviridae; Gemykibivirus
GCA_002826085.1	1998	1	Rhinolophus associated gemykibivirus 2	NA	Viruses; Monodnaviria; Shotokuvirae; Cressdnaviricota; Repensiviricetes; Geplafuvirales; Genomoviridae; Gemykibivirus
GCA_002210975.1	9969	2	Cucurbit vein banding virus	NA	Viruses; ssRNA viruses; ssRNA positive-strand viruses, no DNA stage; Potyviridae; Potyvirus; unclassified Potyvirus
GCA_002624805.1	170496	266	Escherichia phage ST0	NA	Viruses; Duplodnaviria; Heunggongvirae; Uroviricota; Caudoviricetes; Caudovirales; Myoviridae; Tevenvirinae; Mosigvirus; Escherichia virus ST0
GCA_002624825.2	53092	79	Escherichia phage ST32	NA	Viruses; Duplodnaviria; Heunggongvirae; Uroviricota; Caudoviricetes; Caudovirales; Chaseviridae; Carltongylesvirus; Escherichia virus ST32

gca	genome.size	proteome.size	phage.name	strain	Taxonomy
GCA_002624845.1	38670	47	Pasteurella phage vB_PmuP_PHB02	NA	Viruses; Duplodnaviria; Heunggongvirae; Uroviricota; Caudoviricetes; Caudovirales; Autographiviridae; Wuhanvirus; Pasteurella virus PHB02
GCA_003729255.1	10475	1	Milolii virus	NA	Viruses; unclassified viruses
GCA_002194445.1	9445	2	Aphis glycines virus 3	NA	Viruses; ssRNA viruses; ssRNA positive-strand viruses, no DNA stage; Picornavirales; Dicistroviridae; Cripavirus; unclassified Cripavirus
GCA_002624905.1	44005	59	Sinorhizobium phage phiM5	NA	Viruses; Duplodnaviria; Heunggongvirae; Uroviricota; Caudoviricetes; Caudovirales; Podoviridae; unclassified Podoviridae
GCA_003032685.1	14926	6	Antarctic penguin virus A	NA	Viruses; Riboviria; Orthornavirae; Negarnaviricota; Haploviricotina; Monjiviricetes; Mononegavirales; Paramyxoviridae; Avulavirinae; Orthoavulavirus; Avian orthoavulavirus 17
GCA_003032695.1	14931	6	Antarctic penguin virus B	NA	Viruses; Riboviria; Orthornavirae; Negarnaviricota; Haploviricotina; Monjiviricetes; Mononegavirales; Paramyxoviridae; Avulavirinae; Orthoavulavirus; Avian orthoavulavirus 18
GCA_003032705.1	15017	6	Antarctic penguin virus C	NA	Viruses; Riboviria; Orthornavirae; Negarnaviricota; Haploviricotina; Monjiviricetes; Mononegavirales; Paramyxoviridae; Avulavirinae; Orthoavulavirus; Avian orthoavulavirus 19
GCA_018987125.1	156127	241	Mycobacterium virus I3	NA	Viruses; Duplodnaviria; Heunggongvirae; Uroviricota; Caudoviricetes; Caudovirales; Myoviridae; Bixzunavirus
GCA_004130715.1	4962	3	Shinobi tetravirus	shinobi	Viruses; Riboviria; unclassified Riboviria; unclassified ssRNA viruses
GCA_002194525.1	5819	8	Peach associated luteovirus	NA	Viruses; ssRNA viruses; ssRNA positive-strand viruses, no DNA stage; Luteoviridae; unclassified Luteoviridae
GCA_002194385.1	1902	2	Duck associated cyclovirus 1	DuACyV11	Viruses; ssDNA viruses; Circoviridae; Cyclovirus; unclassified Cyclovirus
GCA_002219445.1	1971	2	Garrulus glandarius associated circular virus 1	GgaCV11	Viruses; ssDNA viruses; Circoviridae; unclassified Circoviridae
GCA_002624965.1	309157	468	Pseudomonas phage Phabio	NA	Viruses; Duplodnaviria; Heunggongvirae; Uroviricota; Caudoviricetes; Caudovirales; Myoviridae; Phikzvirus; unclassified Phikzvirus
GCA_002624985.1	62978	72	Pseudomonas phage Skulduggery	NA	Viruses; Duplodnaviria; Heunggongvirae; Uroviricota; Caudoviricetes; Caudovirales; Podoviridae; unclassified Podoviridae
GCA_002625005.1	278136	334	Pseudomonas phage Noxifer	NA	Viruses; Duplodnaviria; Heunggongvirae; Uroviricota; Caudoviricetes; Caudovirales; Myoviridae; Noxifervirus; Pseudomonas virus Noxifer
GCA_003032675.1	15180	6	Avian paramyxovirus UPO216	NA	Viruses; Riboviria; Orthornavirae; Negarnaviricota; Haploviricotina; Monjiviricetes; Mononegavirales; Paramyxoviridae; Avulavirinae; Orthoavulavirus; Avian orthoavulavirus 16
GCA_005075085.1	39130	58	Lactobacillus phage phiEF11	NA	Viruses; Duplodnaviria; Heunggongvirae; Uroviricota; Caudoviricetes; Caudovirales; Siphoviridae; unclassified Siphoviridae
GCA_002625025.1	50564	76	Shigella phage vB_SsoSISF002	NA	Viruses; Duplodnaviria; Heunggongvirae; Uroviricota; Caudoviricetes; Caudovirales; Drexlerviridae; Tunavirinae; Tunavirus; Shigella virus ISF002
GCA_002625045.1	109575	241	Mycobacterium phage Porcelain	NA	Viruses; Duplodnaviria; Heunggongvirae; Uroviricota; Caudoviricetes; Caudovirales; Siphoviridae; Omegavirus; unclassified Omegavirus
GCA_003186085.1	4248	4	Ruegeria phage DSS3P22	NA	Viruses; Monodnaviria; Sangervirae; Phixviricota; Malgrandaviricetes; Petitvirales; Microviridae; unclassified Microviridae
GCA_002625085.1	43344	67	Mycobacterium phage ZenTime222	NA	Viruses; Duplodnaviria; Heunggongvirae; Uroviricota; Caudoviricetes; Caudovirales; Siphoviridae; Bernalvirus; unclassified Bernalvirus
GCA_002625105.1	39707	54	Streptococcus phage D4276	NA	Viruses; Duplodnaviria; Heunggongvirae; Uroviricota; Caudoviricetes; Caudovirales; Siphoviridae; Moineauvirus; unclassified Moineauvirus
GCA_002625125.1	37287	43	Pasteurella phage PHB01	NA	Viruses; Duplodnaviria; Heunggongvirae; Uroviricota; Caudoviricetes; Caudovirales; Autographiviridae; Studiervirinae; unclassified Studiervirinae
GCA_002955325.1	90000	84	Erysipelothrix phage phi1605	NA	Viruses; Duplodnaviria; Heunggongvirae; Uroviricota; Caudoviricetes; Caudovirales; Siphoviridae; unclassified Siphoviridae
GCA_002625145.1	171175	275	Serratia phage CBH8	NA	Viruses; Duplodnaviria; Heunggongvirae; Uroviricota; Caudoviricetes; Caudovirales; Myoviridae; Winklervirus; unclassified Winklervirus
GCA_002625165.1	171175	275	Serratia phage CHI14	NA	Viruses; Duplodnaviria; Heunggongvirae; Uroviricota; Caudoviricetes; Caudovirales; Myoviridae; Winklervirus; Serratia virus CHI14
GCA_002625185.1	172450	277	Serratia phage X20	NA	Viruses; Duplodnaviria; Heunggongvirae; Uroviricota; Caudoviricetes; Caudovirales; Myoviridae; Winklervirus; unclassified Winklervirus
GCA_002955335.1	34641	52	Synechococcus phage SLBS1	NA	Viruses; Duplodnaviria; Heunggongvirae; Uroviricota; Caudoviricetes; Caudovirales; Siphoviridae; unclassified Siphoviridae
GCA_002820785.1	9143	1	Hepacivirus B	NA	Viruses; Riboviria; Orthornavirae; Kitrinoviricota; Flasuviricetes; Amarillovirales; Flaviviridae; Hepacivirus
GCA_000904775.1	8879	1	Hepacivirus E	NA	Viruses; Riboviria; Orthornavirae; Kitrinoviricota; Flasuviricetes; Amarillovirales; Flaviviridae; Hepacivirus
GCA_002820985.1	9188	1	Hepacivirus F	NA	Viruses; Riboviria; Orthornavirae; Kitrinoviricota; Flasuviricetes; Amarillovirales; Flaviviridae; Hepacivirus
GCA_000929615.1	8976	1	Hepacivirus G	NA	Viruses; Riboviria; Orthornavirae; Kitrinoviricota; Flasuviricetes; Amarillovirales; Flaviviridae; Hepacivirus
GCA_000925175.1	8568	1	Hepacivirus H	NA	Viruses; Riboviria; Orthornavirae; Kitrinoviricota; Flasuviricetes; Amarillovirales; Flaviviridae; Hepacivirus
GCA_002821005.1	8883	1	Hepacivirus I	NA	Viruses; Riboviria; Orthornavirae; Kitrinoviricota; Flasuviricetes; Amarillovirales; Flaviviridae; Hepacivirus

gca	genome.size	proteome.size	phage.name	strain	Taxonomy
GCA_002821025.1	9687	1	Hepacivirus J	NA	Viruses; Riboviria; Orthornavirae; Kitrinoviricota; Flasuviricetes; Amarillovirales; Flaviviridae; Hepacivirus
GCA_002821045.1	9609	1	Hepacivirus K	NA	Viruses; Riboviria; Orthornavirae; Kitrinoviricota; Flasuviricetes; Amarillovirales; Flaviviridae; Hepacivirus
GCA_002375095.1	8916	1	Hepacivirus L	NA	Viruses; Riboviria; Orthornavirae; Kitrinoviricota; Flasuviricetes; Amarillovirales; Flaviviridae; Hepacivirus
GCA_002821065.1	9188	1	Hepacivirus M	NA	Viruses; Riboviria; Orthornavirae; Kitrinoviricota; Flasuviricetes; Amarillovirales; Flaviviridae; Hepacivirus
GCA_002821085.1	8880	1	Hepacivirus N	NA	Viruses; Riboviria; Orthornavirae; Kitrinoviricota; Flasuviricetes; Amarillovirales; Flaviviridae; Hepacivirus
GCA_002599345.1	41724	72	Salmonella phage P22pbi	NA	Viruses; Duplodnaviria; Heunggongvirae; Uroviricota; Caudoviricetes; Caudovirales; Podoviridae; Lederbergvirus; Salmonella virus P22
GCA_003032635.1	10646	4	Souris virus	NA	Viruses; Riboviria; Orthornavirae; Negarnaviricota; Polyploviricotina; Ellioviricetes; Bunyavirales; Arenaviridae; Mammarenavirus; Souris mammarenavirus
GCA_002194485.1	2572	3	Torque teno indri virus 1	NA	Viruses; ssDNA viruses; Anelloviridae; unclassified Anelloviridae
GCA_002210815.1	6363	2	Aedes alboannulatus totilike virus 1	mosWSCP114121	Viruses; unclassified viruses
GCA_002211015.1	6363	2	Aedes camptorhynchus totilike virus 1	mos172CP87493	Viruses; unclassified viruses
GCA_002210595.1	11470	5	Aedes camptorhynchus negevlike virus	mos191CP47896	Viruses; unclassified viruses
GCA_002288815.1	14009	4	Aedes camptorhynchus reolike virus	mos182CP78499	Viruses; unclassified viruses
GCA_002210955.1	13297	6	Culex mononegalike virus 2	mos191gb23464	Viruses; unclassified viruses
GCA_002210795.1	10867	3	Culex negevlike virus 1	mosWSX37710	Viruses; unclassified viruses
GCA_002210995.1	9324	3	Culex negevlike virus 2	mos172X30622	Viruses; unclassified viruses
GCA_002210575.1	9176	3	Culex negevlike virus 3	mos172X44875	Viruses; unclassified viruses
GCA_004790295.1	10814	4	Culex phasmalike virus	mos172gb37606	Viruses; Riboviria; Orthornavirae; Negarnaviricota; Polyploviricotina; Ellioviricetes; Bunyavirales; Phasmaviridae; Orthophasmavirus; Culex orthophasmavirus
GCA_002210935.1	11503	5	Culex rhabdolike virus	mosWSB71420	Viruses; unclassified viruses
GCA_002210775.1	2709	1	Leshenault partitilike virus	mos182CP80460	Viruses; unclassified viruses
GCA_002210675.1	2857	1	PointDouro narnalike virus	mos172gb29666	Viruses; unclassified viruses
GCA_002210555.1	2968	1	Wilkie narnalike virus 1	mosWSCP70929	Viruses; unclassified viruses
GCA_002210915.1	3367	1	Wilkie narnalike virus 2	mosWSCP85442	Viruses; unclassified viruses
GCA_002210755.1	2100	1	Wilkie partitilike virus 1	mosWSCP36002	Viruses; unclassified viruses
GCA_002210655.1	1819	1	Wilkie partitilike virus 2	mosWSCP53020	Viruses; unclassified viruses
GCA_000843905.1	1387	1	Chili leaf curl betasatellite	NA	Viruses; Tolecusatellitidae; Betasatellite
GCA_002830025.1	1371	1	Chili leaf curl Sri Lanka betasatellite	NA	Viruses; Tolecusatellitidae; Betasatellite
GCA_002987825.1	1353	1	Ageratum yellow vein India betasatellite	NA	Viruses; Tolecusatellitidae; Betasatellite
GCA_002987855.1	1351	1	Ageratum yellow vein Sri Lanka betasatellite	NA	Viruses; Tolecusatellitidae; Betasatellite
GCA_002830065.1	1359	1	Eupatorium yellow vein mosaic betasatellite	NA	Viruses; Tolecusatellitidae; Betasatellite
GCA_002830125.1	1351	1	Okra leaf curl Oman betasatellite	NA	Viruses; Tolecusatellitidae; Betasatellite
GCA_002830185.1	1359	1	Rhynchosia yellow mosaic betasatellite	NA	Viruses; Tolecusatellitidae; Betasatellite
GCA_000871565.1	1350	1	Tobacco leaf curl Japan betasatellite	NA	Viruses; Tolecusatellitidae; Betasatellite
GCA_002830245.1	1338	1	Tobacco leaf curl Patna betasatellite	IndiaPUSA 22010	Viruses; Tolecusatellitidae; Betasatellite
GCA_002625205.1	156281	253	Acidovorax phage ACP17	NA	Viruses; Duplodnaviria; Heunggongvirae; Uroviricota; Caudoviricetes; Caudovirales; Myoviridae; Busanvirus; Acidovorax virus ACP17

gca	genome.size	proteome.size	phage.name	strain	Taxonomy
GCA_002830265.1	1377	1	Tomato leaf curl Bangalore betasatellite	NA	Viruses; Tolecusatellitidae; Betasatellite
GCA_002830305.1	1346	1	Tomato leaf curl Laguna betasatellite	NA	Viruses; Tolecusatellitidae; Betasatellite
GCA_002987955.1	1348	1	Tomato leaf curl Laos betasatellite	NA	Viruses; Tolecusatellitidae; Betasatellite
GCA_003505815.1	19043	9	Bombali ebolavirus	NA	Viruses; Riboviria; Orthornavirae; Negarnaviricota; Haploviricotina; Monjiviricetes; Mononegavirales; Filoviridae; Ebolavirus
GCA_000847025.1	7349	14	Pseudomonas virus Pf1	ATCC 25102B1	Viruses; Monodnaviria; Loebvirae; Hofneiviricota; Faserviricetes; Tubulavirales; Inoviridae; Primolicivirus
GCA_002826545.1	6867	7	Stenotrophomonas phage PSH1	NA	Viruses; Monodnaviria; Loebvirae; Hofneiviricota; Faserviricetes; Tubulavirales; Inoviridae; Psecadovirus; Stenotrophomonas virus PSH1
GCA_003029355.1	7438	3	Dioscorea bacilliform RT virus 2	NA	Viruses; Riboviria; Pararnavirae; Artverviricota; Revtraviricetes; Ortervirales; Caulimoviridae; Badnavirus
GCA_002210535.1	13766	10	Oliviers shrew virus 1	NA	Viruses; ssRNA viruses; ssRNA positive-strand viruses, no DNA stage; Nidovirales; Arteriviridae; unclassified Arteriviridae
GCA_004129915.1	2603	3	Lesser panda anellovirus	NA	Viruses; Anelloviridae; unclassified Anelloviridae
GCA_002210695.1	2119	4	Torque teno Leptonychotes weddellii virus1	NA	Viruses; ssDNA viruses; Anelloviridae; unclassified Anelloviridae
GCA_002210895.1	2105	4	Torque teno Leptonychotes weddellii virus2	NA	Viruses; ssDNA viruses; Anelloviridae; unclassified Anelloviridae
GCA_004130515.1	2757	4	Rice latent virus 1	NA	Viruses; Monodnaviria; Shotokuvirae; Cressdnaviricota; Repensiviricetes; Geplafuvirales; Geminiviridae; Mastrevirus
GCA_004130535.1	2843	4	Rice latent virus 2	NA	Viruses; Monodnaviria; Shotokuvirae; Cressdnaviricota; Repensiviricetes; Geplafuvirales; Geminiviridae; Mastrevirus
GCA_002237235.1	8136	2	Fur seal picornalike virus	NA	Viruses; ssRNA viruses; ssRNA positive-strand viruses, no DNA stage; Picornavirales; Picornaviridae; unclassified Picornaviridae
GCA_002219625.1	7279	5	Euonymus yellow vein virus	NA	Viruses; ssRNA viruses; ssRNA positive-strand viruses, no DNA stage; Tymovirales; Alphaflexiviridae; unclassified Alphaflexiviridae
GCA_013086075.1	28762	13	Tarumizu tick virus	13T269	Viruses; Riboviria; Orthornavirae; Duplornaviricota; Resentoviricetes; Reovirales; Reoviridae; Spinareovirinae; Coltivirus; Tarumizu coltivirus
GCA_002625245.1	58405	93	Arthrobacter phage Niktson	NA	Viruses; Duplodnaviria; Heunggongvirae; Uroviricota; Caudoviricetes; Caudovirales; Siphoviridae; Gordonvirus; unclassified Gordonvirus
GCA_002625265.1	58405	93	Arthrobacter phage ElephantMan	NA	Viruses; Duplodnaviria; Heunggongvirae; Uroviricota; Caudoviricetes; Caudovirales; Siphoviridae; Gordonvirus; unclassified Gordonvirus
GCA_002625285.1	108627	237	Mycobacterium phage Lucky2013	NA	Viruses; Duplodnaviria; Heunggongvirae; Uroviricota; Caudoviricetes; Caudovirales; Siphoviridae; Omegavirus; unclassified Omegavirus
GCA_002625305.1	48639	81	Mycobacterium phage Bogie	NA	Viruses; Duplodnaviria; Heunggongvirae; Uroviricota; Caudoviricetes; Caudovirales; Siphoviridae; Pclasvirinae; Fishburnevirus; unclassified Fishburnevirus
GCA_002625325.1	91159	118	Escherichia phage D6	NA	Viruses; Duplodnaviria; Heunggongvirae; Uroviricota; Caudoviricetes; Caudovirales; Myoviridae; unclassified Myoviridae
GCA_002625345.2	30413	45	Erwinia phage EtG	NA	Viruses; Duplodnaviria; Heunggongvirae; Uroviricota; Caudoviricetes; Caudovirales; Myoviridae; Peduovirinae; Eganvirus; Erwinia virus EtG
GCA_002219805.1	5183	4	Dromedary camel bocaparvovirus 1	NA	Viruses; ssDNA viruses; Parvoviridae; Parvovirinae; Bocaparvovirus; unclassified Bocaparvovirus
GCA_002237215.1	5189	3	Dromedary camel bocaparvovirus 2	NA	Viruses; ssDNA viruses; Parvoviridae; Parvovirinae; Bocaparvovirus; unclassified Bocaparvovirus
GCA_002829385.1	19453	10	Equine encephalosis virus	NA	Viruses; Riboviria; Orthornavirae; Duplornaviricota; Resentoviricetes; Reovirales; Reoviridae; Sedoreovirinae; Orbivirus
GCA_003032715.1	10843	5	Kanyawara virus	NA	Viruses; Riboviria; Orthornavirae; Negarnaviricota; Haploviricotina; Monjiviricetes; Mononegavirales; Rhabdoviridae; Ledantevirus; Kanyawara ledantevirus
GCA_002223795.1	12614	1	Linda virus	Austria1	Viruses; ssRNA viruses; ssRNA positive-strand viruses, no DNA stage; Flaviviridae; Pestivirus; unclassified Pestivirus
GCA_001885425.1	31481	32	Bat mastadenovirus G	NA	Viruses; Varidnaviria; Bamfordvirae; Preplasmiviricota; Tectiliviricetes; Rowavirales; Adenoviridae; Mastadenovirus
GCA_004788055.2	5221	7	Tomato mosaic Trujillo virus	NA	Viruses; Monodnaviria; Shotokuvirae; Cressdnaviricota; Repensiviricetes; Geplafuvirales; Geminiviridae; Begomovirus; Tomato wrinkled mosaic virus
GCA_013087485.2	5147	7	Tomato chlorotic leaf curl virus	NA	Viruses; Monodnaviria; Shotokuvirae; Cressdnaviricota; Repensiviricetes; Geplafuvirales; Geminiviridae; Begomovirus
GCA_008797835.1	7364	1	Enterovirus D94	NA	Viruses; Riboviria; Orthornavirae; Pisuviricota; Pisoniviricetes; Picornavirales; Picornaviridae; Enterovirus; Enterovirus D
GCA_002625365.1	45373	88	Acinetobacter phage AbP2	NA	Viruses; Duplodnaviria; Heunggongvirae; Uroviricota; Caudoviricetes; Caudovirales; Myoviridae; Obolenskvirus; Acinetobacter virus AbP2
GCA_002625385.1	132996	233	Streptomyces phage Warpy	NA	Viruses; Duplodnaviria; Heunggongvirae; Uroviricota; Caudoviricetes; Caudovirales; Siphoviridae; Samistivirus; unclassified Samistivirus
GCA_002625405.1	133917	229	Streptomyces phage Sushi23	NA	Viruses; Duplodnaviria; Heunggongvirae; Uroviricota; Caudoviricetes; Caudovirales; Siphoviridae; Samistivirus; unclassified Samistivirus

gca	genome.size	proteome.size	phage.name	strain	Taxonomy
GCA_002625425.1	40469	63	Escherichia phage ECP1	NA	Viruses; Duplodnaviria; Heunggongvirae; Uroviricota; Caudoviricetes; Caudovirales; Siphoviridae; Hendrixvirus; unclassified Hendrixvirus
GCA_002625465.1	50053	85	Mycobacterium phage Fred313	NA	Viruses; Duplodnaviria; Heunggongvirae; Uroviricota; Caudoviricetes; Caudovirales; Siphoviridae; Fromanvirus; unclassified Fromanvirus
GCA_002625485.1	61318	98	Mycobacterium phage Hurricane	NA	Viruses; Duplodnaviria; Heunggongvirae; Uroviricota; Caudoviricetes; Caudovirales; Siphoviridae; Timquatrovirus; unclassified Timquatrovirus
GCA_002625505.1	48815	81	Mycobacterium phage StevieRay	NA	Viruses; Duplodnaviria; Heunggongvirae; Uroviricota; Caudoviricetes; Caudovirales; Siphoviridae; Pclasvirinae; Fishburnevirus; unclassified Fishburnevirus
GCA_002625525.1	46314	65	Rhodococcus phage Jester	NA	Viruses; Duplodnaviria; Heunggongvirae; Uroviricota; Caudoviricetes; Caudovirales; Siphoviridae; Rerduovirus; unclassified Rerduovirus
GCA_002625545.1	440001	541	Salicola phage SCTP2	NA	Viruses; Duplodnaviria; Heunggongvirae; Uroviricota; Caudoviricetes; Caudovirales; Myoviridae; unclassified Myoviridae
GCA_002625565.1	49797	73	Arthrobacter phage Beans	NA	Viruses; Duplodnaviria; Heunggongvirae; Uroviricota; Caudoviricetes; Caudovirales; Myoviridae; Marthavirus; Arthrobacter virus Beans
GCA_002625585.1	58739	101	Arthrobacter phage Cheesy	NA	Viruses; Duplodnaviria; Heunggongvirae; Uroviricota; Caudoviricetes; Caudovirales; Siphoviridae; Mudcatvirus; unclassified Mudcatvirus
GCA_002625605.1	62236	97	Mycobacterium phage Amgine	NA	Viruses; Duplodnaviria; Heunggongvirae; Uroviricota; Caudoviricetes; Caudovirales; Siphoviridae; Timquatrovirus; unclassified Timquatrovirus
GCA_002625625.1	60813	100	Mycobacterium phage Cain	NA	Viruses; Duplodnaviria; Heunggongvirae; Uroviricota; Caudoviricetes; Caudovirales; Siphoviridae; Timquatrovirus; unclassified Timquatrovirus
GCA_002625645.1	76315	129	Mycobacterium phage GuuelaD	NA	Viruses; Duplodnaviria; Heunggongvirae; Uroviricota; Caudoviricetes; Caudovirales; Siphoviridae; Bronvirus; unclassified Bronvirus
GCA_002625665.1	60321	100	Mycobacterium phage Krueger	NA	Viruses; Duplodnaviria; Heunggongvirae; Uroviricota; Caudoviricetes; Caudovirales; Siphoviridae; Timquatrovirus; unclassified Timquatrovirus
GCA_002625685.1	51008	89	Mycobacterium phage Lokk	NA	Viruses; Duplodnaviria; Heunggongvirae; Uroviricota; Caudoviricetes; Caudovirales; Siphoviridae; Fromanvirus; unclassified Fromanvirus
GCA_002625705.1	56580	90	Mycobacterium phage PhelpsODU	NA	Viruses; Duplodnaviria; Heunggongvirae; Uroviricota; Caudoviricetes; Caudovirales; Siphoviridae; Timquatrovirus; unclassified Timquatrovirus
GCA_002625725.1	61109	101	Mycobacterium phage Phrank	NA	Viruses; Duplodnaviria; Heunggongvirae; Uroviricota; Caudoviricetes; Caudovirales; Siphoviridae; Timquatrovirus; unclassified Timquatrovirus
GCA_002625745.1	61882	97	Mycobacterium phage SirPhilip	NA	Viruses; Duplodnaviria; Heunggongvirae; Uroviricota; Caudoviricetes; Caudovirales; Siphoviridae; Timquatrovirus; unclassified Timquatrovirus
GCA_002625765.1	61208	100	Mycobacterium phage Unicorn	NA	Viruses; Duplodnaviria; Heunggongvirae; Uroviricota; Caudoviricetes; Caudovirales; Siphoviridae; Timquatrovirus; unclassified Timquatrovirus
GCA_002625785.1	46673	65	Rhodococcus phage Alatin	NA	Viruses; Duplodnaviria; Heunggongvirae; Uroviricota; Caudoviricetes; Caudovirales; Siphoviridae; Rerduovirus; unclassified Rerduovirus
GCA_002625805.1	46389	64	Rhodococcus phage AppleCloud	NA	Viruses; Duplodnaviria; Heunggongvirae; Uroviricota; Caudoviricetes; Caudovirales; Siphoviridae; Rerduovirus; unclassified Rerduovirus
GCA_002625825.1	46854	65	Rhodococcus phage Hiro	NA	Viruses; Duplodnaviria; Heunggongvirae; Uroviricota; Caudoviricetes; Caudovirales; Siphoviridae; Rerduovirus; unclassified Rerduovirus
GCA_002625845.1	46985	67	Rhodococcus phage Krishelle	NA	Viruses; Duplodnaviria; Heunggongvirae; Uroviricota; Caudoviricetes; Caudovirales; Siphoviridae; Rerduovirus; unclassified Rerduovirus
GCA_002625865.1	46619	65	Rhodococcus phage Naiad	NA	Viruses; Duplodnaviria; Heunggongvirae; Uroviricota; Caudoviricetes; Caudovirales; Siphoviridae; Rerduovirus; unclassified Rerduovirus
GCA_002625885.1	46627	65	Rhodococcus phage RexFury	NA	Viruses; Duplodnaviria; Heunggongvirae; Uroviricota; Caudoviricetes; Caudovirales; Siphoviridae; Rerduovirus; unclassified Rerduovirus
GCA_002625905.1	46619	65	Rhodococcus phage StCroix	NA	Viruses; Duplodnaviria; Heunggongvirae; Uroviricota; Caudoviricetes; Caudovirales; Siphoviridae; Rerduovirus; unclassified Rerduovirus
GCA_002625925.1	48699	80	Mycobacterium phage Ksquared	NA	Viruses; Duplodnaviria; Heunggongvirae; Uroviricota; Caudoviricetes; Caudovirales; Siphoviridae; Pclasvirinae; Fishburnevirus; unclassified Fishburnevirus
GCA_002625945.1	70707	114	Arthrobacter phage Colucci	NA	Viruses; Duplodnaviria; Heunggongvirae; Uroviricota; Caudoviricetes; Caudovirales; Myoviridae; Radnorvirus; Arthrobacter virus Colucci
GCA_002625965.1	70035	111	Arthrobacter phage Kabreeze	NA	Viruses; Duplodnaviria; Heunggongvirae; Uroviricota; Caudoviricetes; Caudovirales; Myoviridae; Radnorvirus; unclassified Radnorvirus
GCA_002625985.1	70396	112	Arthrobacter phage RosiePosie	NA	Viruses; Duplodnaviria; Heunggongvirae; Uroviricota; Caudoviricetes; Caudovirales; Myoviridae; Radnorvirus; unclassified Radnorvirus
GCA_002626005.1	70123	112	Arthrobacter phage Scavito	NA	Viruses; Duplodnaviria; Heunggongvirae; Uroviricota; Caudoviricetes; Caudovirales; Myoviridae; Radnorvirus; unclassified Radnorvirus
GCA_002626025.1	70091	111	Arthrobacter phage Tophat	NA	Viruses; Duplodnaviria; Heunggongvirae; Uroviricota; Caudoviricetes; Caudovirales; Myoviridae; Radnorvirus; unclassified Radnorvirus
GCA_002626045.1	58247	92	Arthrobacter phage Molivia	NA	Viruses; Duplodnaviria; Heunggongvirae; Uroviricota; Caudoviricetes; Caudovirales; Siphoviridae; Amigovirus; Arthrobacteria virus Molivia
GCA_002626065.1	62112	99	Mycobacterium phage AlleyCat	NA	Viruses; Duplodnaviria; Heunggongvirae; Uroviricota; Caudoviricetes; Caudovirales; Siphoviridae; Timquatrovirus; unclassified Timquatrovirus
GCA_002626085.1	73808	125	Mycobacterium phage Appletree2	NA	Viruses; Duplodnaviria; Heunggongvirae; Uroviricota; Caudoviricetes; Caudovirales; Siphoviridae; Bronvirus; unclassified Bronvirus
GCA_002626105.1	50400	88	Mycobacterium phage BobSwaget	NA	Viruses; Duplodnaviria; Heunggongvirae; Uroviricota; Caudoviricetes; Caudovirales; Siphoviridae; Fromanvirus; unclassified Fromanvirus

gca	genome.size	proteome.size	phage.name	strain	Taxonomy
GCA_002626125.1	63344	92	Mycobacterium phage Edugator	NA	Viruses; Duplodnaviria; Heunggongvirae; Uroviricota; Caudoviricetes; Caudovirales; Siphoviridae; Timquatrovirus; unclassified Timquatrovirus
GCA_002743555.1	77031	131	Mycobacterium phage Finemlucis	NA	Viruses; Duplodnaviria; Heunggongvirae; Uroviricota; Caudoviricetes; Caudovirales; Siphoviridae; Bronvirus; unclassified Bronvirus
GCA_002626145.1	63153	95	Mycobacterium phage Guillsminger	NA	Viruses; Duplodnaviria; Heunggongvirae; Uroviricota; Caudoviricetes; Caudovirales; Siphoviridae; Timquatrovirus; unclassified Timquatrovirus
GCA_002626165.1	50898	87	Mycobacterium phage KADY	NA	Viruses; Duplodnaviria; Heunggongvirae; Uroviricota; Caudoviricetes; Caudovirales; Siphoviridae; Fromanvirus; unclassified Fromanvirus
GCA_002626185.1	76653	132	Mycobacterium phage Miley16	NA	Viruses; Duplodnaviria; Heunggongvirae; Uroviricota; Caudoviricetes; Caudovirales; Siphoviridae; Bronvirus; unclassified Bronvirus
GCA_002626205.1	61041	98	Mycobacterium phage Peanam	NA	Viruses; Duplodnaviria; Heunggongvirae; Uroviricota; Caudoviricetes; Caudovirales; Siphoviridae; Timquatrovirus; unclassified Timquatrovirus
GCA_002626225.1	50856	86	Mycobacterium phage Stagni	NA	Viruses; Duplodnaviria; Heunggongvirae; Uroviricota; Caudoviricetes; Caudovirales; Siphoviridae; Fromanvirus; unclassified Fromanvirus
GCA_002626245.1	50841	90	Mycobacterium phage StepMih	NA	Viruses; Duplodnaviria; Heunggongvirae; Uroviricota; Caudoviricetes; Caudovirales; Siphoviridae; Fromanvirus; unclassified Fromanvirus
GCA_002626265.2	40353	54	Corynebacterium phage Poushou	NA	Viruses; Duplodnaviria; Heunggongvirae; Uroviricota; Caudoviricetes; Caudovirales; Siphoviridae; Poushouvirus; Corynebacterium virus Poushou
GCA_002626285.1	39122	66	Arthrobacter phage Abidatro	NA	Viruses; Duplodnaviria; Heunggongvirae; Uroviricota; Caudoviricetes; Caudovirales; Siphoviridae; Galaxyvirus; Arthrobacter virus Abidatro
GCA_002626305.1	43914	62	Arthrobacter phage Canowicakte	NA	Viruses; Duplodnaviria; Heunggongvirae; Uroviricota; Caudoviricetes; Caudovirales; Siphoviridae; Korravirus; unclassified Korravirus
GCA_002626325.1	56622	86	Arthrobacter phage Caterpillar	NA	Viruses; Duplodnaviria; Heunggongvirae; Uroviricota; Caudoviricetes; Caudovirales; Siphoviridae; Gordonvirus; unclassified Gordonvirus
GCA_002626345.1	43081	61	Arthrobacter phage Christian	NA	Viruses; Duplodnaviria; Heunggongvirae; Uroviricota; Caudoviricetes; Caudovirales; Siphoviridae; Korravirus; unclassified Korravirus
GCA_002626365.1	43562	62	Arthrobacter phage Dino	NA	Viruses; Duplodnaviria; Heunggongvirae; Uroviricota; Caudoviricetes; Caudovirales; Siphoviridae; Korravirus; unclassified Korravirus
GCA_002626385.1	15556	26	Arthrobacter phage Elkhorn	NA	Viruses; Duplodnaviria; Heunggongvirae; Uroviricota; Caudoviricetes; Caudovirales; Siphoviridae; Decurrovirus; unclassified Decurrovirus
GCA_002626405.1	15540	27	Arthrobacter phage KylieMac	NA	Viruses; Duplodnaviria; Heunggongvirae; Uroviricota; Caudoviricetes; Caudovirales; Siphoviridae; Decurrovirus; unclassified Decurrovirus
GCA_002626425.1	60137	96	Arthrobacter phage LiSara	NA	Viruses; Duplodnaviria; Heunggongvirae; Uroviricota; Caudoviricetes; Caudovirales; Siphoviridae; Laroyevirus; unclassified Laroyevirus
GCA_002626445.1	15521	26	Arthrobacter phage Link	NA	Viruses; Duplodnaviria; Heunggongvirae; Uroviricota; Caudoviricetes; Caudovirales; Siphoviridae; Decurrovirus; unclassified Decurrovirus
GCA_002626465.1	15556	26	Arthrobacter phage Lore	NA	Viruses; Duplodnaviria; Heunggongvirae; Uroviricota; Caudoviricetes; Caudovirales; Siphoviridae; Decurrovirus; unclassified Decurrovirus
GCA_002626485.1	15556	26	Arthrobacter phage Mariposa	NA	Viruses; Duplodnaviria; Heunggongvirae; Uroviricota; Caudoviricetes; Caudovirales; Siphoviridae; Decurrovirus; unclassified Decurrovirus
GCA_002626505.1	58839	92	Arthrobacter phage Nightmare	NA	Viruses; Duplodnaviria; Heunggongvirae; Uroviricota; Caudoviricetes; Caudovirales; Siphoviridae; Gordonvirus; unclassified Gordonvirus
GCA_002626525.1	44045	61	Arthrobacter phage Nubia	NA	Viruses; Duplodnaviria; Heunggongvirae; Uroviricota; Caudoviricetes; Caudovirales; Siphoviridae; Korravirus; unclassified Korravirus
GCA_002626545.1	42963	61	Arthrobacter phage PitaDog	NA	Viruses; Duplodnaviria; Heunggongvirae; Uroviricota; Caudoviricetes; Caudovirales; Siphoviridae; Korravirus; unclassified Korravirus
GCA_002626565.1	15319	26	Arthrobacter phage Seume	NA	Viruses; Duplodnaviria; Heunggongvirae; Uroviricota; Caudoviricetes; Caudovirales; Siphoviridae; Decurrovirus; unclassified Decurrovirus
GCA_002626585.1	59707	98	Arthrobacter phage Shrooms	NA	Viruses; Duplodnaviria; Heunggongvirae; Uroviricota; Caudoviricetes; Caudovirales; Siphoviridae; Laroyevirus; unclassified Laroyevirus
GCA_002626605.1	15680	26	Arthrobacter phage Swenson	NA	Viruses; Duplodnaviria; Heunggongvirae; Uroviricota; Caudoviricetes; Caudovirales; Siphoviridae; Decurrovirus; unclassified Decurrovirus
GCA_002626625.1	15546	26	Arthrobacter phage Taj14	NA	Viruses; Duplodnaviria; Heunggongvirae; Uroviricota; Caudoviricetes; Caudovirales; Siphoviridae; Decurrovirus; unclassified Decurrovirus
GCA_002626645.1	58238	88	Arthrobacter phage Teacup	NA	Viruses; Duplodnaviria; Heunggongvirae; Uroviricota; Caudoviricetes; Caudovirales; Siphoviridae; Gordonvirus; unclassified Gordonvirus
GCA_002626665.1	15556	26	Arthrobacter phage TinoCrisci	NA	Viruses; Duplodnaviria; Heunggongvirae; Uroviricota; Caudoviricetes; Caudovirales; Siphoviridae; Decurrovirus; unclassified Decurrovirus
GCA_002626685.1	59879	94	Arthrobacter phage Wheelbite	NA	Viruses; Duplodnaviria; Heunggongvirae; Uroviricota; Caudoviricetes; Caudovirales; Siphoviridae; Laroyevirus; unclassified Laroyevirus
GCA_002626705.1	61761	94	Mycobacterium phage Amohnition	NA	Viruses; Duplodnaviria; Heunggongvirae; Uroviricota; Caudoviricetes; Caudovirales; Siphoviridae; Timquatrovirus; unclassified Timquatrovirus
GCA_002626725.1	46484	77	Mycobacterium phage Bartholomew	NA	Viruses; Duplodnaviria; Heunggongvirae; Uroviricota; Caudoviricetes; Caudovirales; Siphoviridae; Pclasvirinae; Fishburnevirus; unclassified Fishburnevirus
GCA_002626745.1	57697	94	Mycobacterium phage Chancellor	NA	Viruses; Duplodnaviria; Heunggongvirae; Uroviricota; Caudoviricetes; Caudovirales; Siphoviridae; Timquatrovirus; unclassified Timquatrovirus
GCA_002626765.1	52991	92	Mycobacterium phage Changeling	NA	Viruses; Duplodnaviria; Heunggongvirae; Uroviricota; Caudoviricetes; Caudovirales; Siphoviridae; Fromanvirus; unclassified Fromanvirus

gca	genome.size	proteome.size	phage.name	strain	Taxonomy
GCA_002626785.1	72055	122	Mycobacterium phage Clautastrophe	NA	Viruses; Duplodnaviria; Heunggongvirae; Uroviricota; Caudoviricetes; Caudovirales; Siphoviridae; Bronvirus; unclassified Bronvirus
GCA_002626805.1	61594	94	Mycobacterium phage DarthP	NA	Viruses; Duplodnaviria; Heunggongvirae; Uroviricota; Caudoviricetes; Caudovirales; Siphoviridae; Timquatrovirus; unclassified Timquatrovirus
GCA_002626825.1	58129	94	Mycobacterium phage DismalFunk	NA	Viruses; Duplodnaviria; Heunggongvirae; Uroviricota; Caudoviricetes; Caudovirales; Siphoviridae; Timquatrovirus; unclassified Timquatrovirus
GCA_002626845.3	52497	101	Mycobacterium phage Et2Brutus	NA	Viruses; Duplodnaviria; Heunggongvirae; Uroviricota; Caudoviricetes; Caudovirales; Siphoviridae; Fromanvirus; unclassified Fromanvirus
GCA_002626865.1	58150	94	Mycobacterium phage Findley	NA	Viruses; Duplodnaviria; Heunggongvirae; Uroviricota; Caudoviricetes; Caudovirales; Siphoviridae; Timquatrovirus; unclassified Timquatrovirus
GCA_002626885.1	59708	96	Mycobacterium phage Jeckyll	NA	Viruses; Duplodnaviria; Heunggongvirae; Uroviricota; Caudoviricetes; Caudovirales; Siphoviridae; Timquatrovirus; unclassified Timquatrovirus
GCA_002626905.1	69537	110	Mycobacterium phage Kingsolomon	NA	Viruses; Duplodnaviria; Heunggongvirae; Uroviricota; Caudoviricetes; Caudovirales; Siphoviridae; Bronvirus; unclassified Bronvirus
GCA_002626925.1	76075	130	Mycobacterium phage Krypton555	NA	Viruses; Duplodnaviria; Heunggongvirae; Uroviricota; Caudoviricetes; Caudovirales; Siphoviridae; Bronvirus; unclassified Bronvirus
GCA_002626945.1	60934	102	Mycobacterium phage LastHope	NA	Viruses; Duplodnaviria; Heunggongvirae; Uroviricota; Caudoviricetes; Caudovirales; Siphoviridae; Timquatrovirus; unclassified Timquatrovirus
GCA_002626965.1	70413	112	Mycobacterium phage Nicholas	NA	Viruses; Duplodnaviria; Heunggongvirae; Uroviricota; Caudoviricetes; Caudovirales; Siphoviridae; Bronvirus; unclassified Bronvirus
GCA_002626985.1	75822	130	Mycobacterium phage Nicholasp3	NA	Viruses; Duplodnaviria; Heunggongvirae; Uroviricota; Caudoviricetes; Caudovirales; Siphoviridae; Bronvirus; unclassified Bronvirus
GCA_002627005.1	60143	98	Mycobacterium phage Slimphazie	NA	Viruses; Duplodnaviria; Heunggongvirae; Uroviricota; Caudoviricetes; Caudovirales; Siphoviridae; Timquatrovirus; unclassified Timquatrovirus
GCA_002627025.1	59556	96	Mycobacterium phage Tachez	NA	Viruses; Duplodnaviria; Heunggongvirae; Uroviricota; Caudoviricetes; Caudovirales; Siphoviridae; Timquatrovirus; unclassified Timquatrovirus
GCA_002627045.1	58046	94	Mycobacterium phage Wintermute	NA	Viruses; Duplodnaviria; Heunggongvirae; Uroviricota; Caudoviricetes; Caudovirales; Siphoviridae; Timquatrovirus; unclassified Timquatrovirus
GCA_002627065.1	47631	49	Alteromonas virus vB_AspPH44	NA	Viruses; Duplodnaviria; Heunggongvirae; Uroviricota; Caudoviricetes; Caudovirales; Autographiviridae; Foturvirus; Alteromonas virus H4-4
GCA_002219425.1	7476	5	Grapevine virus K	NA	Viruses; ssRNA viruses; ssRNA positive-strand viruses, no DNA stage; Tymovirales; Betaflexiviridae; Trivirinae; Vitivirus; unclassified Vitivirus
GCA_002627085.1	40879	51	Acinetobacter phage vB_AbaP_B1	NA	Viruses; Duplodnaviria; Heunggongvirae; Uroviricota; Caudoviricetes; Caudovirales; Autographiviridae; Beijerinckvirinae; Friunavirus; Acinetobacter virus B1
GCA_002627105.1	40598	49	Acinetobacter phage vB_AbaP_B3	NA	Viruses; Duplodnaviria; Heunggongvirae; Uroviricota; Caudoviricetes; Caudovirales; Autographiviridae; Beijerinckvirinae; Friunavirus; Acinetobacter virus B3
GCA_002627145.1	41208	49	Acinetobacter phage vB_ApiP_P1	NA	Viruses; Duplodnaviria; Heunggongvirae; Uroviricota; Caudoviricetes; Caudovirales; Autographiviridae; Beijerinckvirinae; Friunavirus; Acinetobacter virus P1
GCA_002627165.1	41514	54	Acinetobacter phage vB_ApiP_P2	NA	Viruses; Duplodnaviria; Heunggongvirae; Uroviricota; Caudoviricetes; Caudovirales; Autographiviridae; Beijerinckvirinae; Friunavirus; Acinetobacter virus P2
GCA_002270705.1	32399	9	Morelia viridis nidovirus	S141323_MVNV	Viruses; ssRNA viruses; ssRNA positive-strand viruses, no DNA stage; Nidovirales; unclassified Nidovirales
GCA_003032645.1	8804	6	Jungle carpet python virus	NA	Viruses; Riboviria; Orthornavirae; Negarnaviricota; Haploviricotina; Monjiviricetes; Mononegavirales; Bornaviridae; Carbovirus; Queensland carbovirus
GCA_003032655.1	8810	6	Southwest carpet python virus	NA	Viruses; Riboviria; Orthornavirae; Negarnaviricota; Haploviricotina; Monjiviricetes; Mononegavirales; Bornaviridae; Carbovirus; Southwest carbovirus
GCA_002219605.1	7462	6	Vanilla latent virus	NA	Viruses; ssRNA viruses; ssRNA positive-strand viruses, no DNA stage; Tymovirales; Alphaflexiviridae; unclassified Alphaflexiviridae
GCA_002219785.1	6295	5	Vanilla virus X	NA	Viruses; ssRNA viruses; ssRNA positive-strand viruses, no DNA stage; Tymovirales; Alphaflexiviridae; unclassified Alphaflexiviridae
GCA_004788395.1	8029	1	Aimelvirus 1	gpai001	Viruses; Riboviria; Orthornavirae; Pisuviricota; Pisoniviricetes; Picornavirales; Picornaviridae; Ailurivirus; Ailurivirus A
GCA_002219405.1	8080	1	Aimelvirus 2	gpai002	Viruses; ssRNA viruses; ssRNA positive-strand viruses, no DNA stage; Picornavirales; Picornaviridae; unclassified Picornaviridae
GCA_002219945.1	7996	7	Ailuropoda melanoleuca papillomavirus 4	gpam004	Viruses; dsDNA viruses, no RNA stage; Papillomaviridae; unclassified Papillomaviridae; non-primate mammal papillomaviruses
GCA_002219585.1	7676	5	Ailuropoda melanoleuca papillomavirus 1	gpam001	Viruses; dsDNA viruses, no RNA stage; Papillomaviridae; unclassified Papillomaviridae; non-primate mammal papillomaviruses
GCA_002219765.1	7582	5	Ailuropoda melanoleuca papillomavirus 2	gpam002	Viruses; dsDNA viruses, no RNA stage; Papillomaviridae; unclassified Papillomaviridae; non-primate mammal papillomaviruses
GCA_002219385.1	2745	2	Giant panda circovirus 1	gpci001	Viruses; ssDNA viruses; Circoviridae; Circovirus; unclassified Circovirus
GCA_002219925.1	1908	3	Giant panda circovirus 2	gpci002	Viruses; ssDNA viruses; Circoviridae; Circovirus; unclassified Circovirus
GCA_002219565.1	2653	3	Giant panda circovirus 3	gpci003	Viruses; ssDNA viruses; Circoviridae; Circovirus; unclassified Circovirus
GCA_002219745.1	1928	2	Giant panda circovirus 4	gpci004	Viruses; ssDNA viruses; Circoviridae; Circovirus; unclassified Circovirus

gca	genome.size	proteome.size	phage.name	strain	Taxonomy
GCA_018582875.1	2078	4	Giant panda anellovirus	gpan20859	Viruses; ssDNA viruses; Anelloviridae; unclassified Anelloviridae
GCA_002219905.1	2185	3	Giant panda associated gemycircularvirus	gpge004	Viruses; Monodnaviria; Shotokuvirae; Cressdnaviricota; Repensiviricetes; Geplafuvirales; Genomoviridae; Gemycircularvirus; unclassified Gemycircularvirus
GCA_003729495.1	1509	1	Giant panda associated partitilike virus	gppc1509	Viruses; unclassified viruses
GCA_002219545.1	5144	5	Giant panda polyomavirus	NA	Viruses; dsDNA viruses, no RNA stage; Polyomaviridae; unclassified Polyomaviridae
GCA_002219725.1	8703	1	Pansavirus 1	gppn001	Viruses; ssRNA viruses; unclassified ssRNA viruses
GCA_002219345.1	8612	1	Pansavirus 2	gppn002	Viruses; ssRNA viruses; unclassified ssRNA viruses
GCA_002219885.1	3925	5	Human fecal virus Jorvi2	NA	Viruses; ssDNA viruses; Circoviridae; unclassified Circoviridae
GCA_002219525.1	1942	2	Human fecal virus Jorvi3	NA	Viruses; ssDNA viruses; Circoviridae; unclassified Circoviridae
GCA_002219705.1	2343	2	Human fecal virus Jorvi4	NA	Viruses; ssDNA viruses; Circoviridae; unclassified Circoviridae
GCA_900092395.1	18168	25	Enterococcus phage Idefix	NA	Viruses; Duplodnaviria; Heunggongvirae; Uroviricota; Caudoviricetes; Caudovirales; Podoviridae; unclassified Podoviridae
GCA_003849025.1	2240	2	Canine fecesassociated gemycircularvirus	NA	Viruses; Monodnaviria; Shotokuvirae; Cressdnaviricota; Repensiviricetes; Geplafuvirales; Genomoviridae; Gemycircularvirus; unclassified Gemycircularvirus
GCA_003849005.1	2238	2	Lupine fecesassociated gemycircularvirus 2	NA	Viruses; Monodnaviria; Shotokuvirae; Cressdnaviricota; Repensiviricetes; Geplafuvirales; Genomoviridae; Gemycircularvirus; unclassified Gemycircularvirus
GCA_003848985.1	2198	2	Lupine fecesassociated gemycircularvirus 1	NA	Viruses; Monodnaviria; Shotokuvirae; Cressdnaviricota; Repensiviricetes; Geplafuvirales; Genomoviridae; Gemycircularvirus; unclassified Gemycircularvirus
GCA_002271165.1	2204	2	Porcine fecesassociated gemycircularvirus	NA	Viruses; Genomoviridae; Gemycircularvirus; unclassified Gemycircularvirus
GCA_004130235.1	5228	3	Lupine bocavirus	NA	Viruses; Monodnaviria; Shotokuvirae; Cossaviricota; Quintoviricetes; Piccovirales; Parvoviridae; Parvovirinae; Bocaparvovirus; unclassified Bocaparvovirus
GCA_002270825.1	3081	2	Porcine stoolassociated circular virusBEL15V010	NA	Viruses; ssDNA viruses; Circoviridae; unclassified Circoviridae
GCA_002627185.1	40574	68	Salmonella phage vB_SalP_PM43	NA	Viruses; Duplodnaviria; Heunggongvirae; Uroviricota; Caudoviricetes; Caudovirales; Podoviridae; Lederbergvirus; unclassified Lederbergvirus
GCA_002270985.1	10232	1	Sudan watermelon mosaic virus	NA	Viruses; ssRNA viruses; ssRNA positive-strand viruses, no DNA stage; Potyviridae; Potyvirus; unclassified Potyvirus
GCA_002270685.1	10152	1	Wild melon vein banding virus	NA	Viruses; ssRNA viruses; ssRNA positive-strand viruses, no DNA stage; Potyviridae; Potyvirus; unclassified Potyvirus
GCA_002758375.1	33613	50	Lactococcus phage P335	NA	Viruses; Duplodnaviria; Heunggongvirae; Uroviricota; Caudoviricetes; Caudovirales; Siphoviridae; unclassified Siphoviridae
GCA_000843105.1	36596	49	Lactococcus phage 4268	NA	Viruses; Duplodnaviria; Heunggongvirae; Uroviricota; Caudoviricetes; Caudovirales; Siphoviridae; unclassified Siphoviridae
GCA_002889975.1	9283	1	Armigeres iflavirus	10P38310	Viruses; Riboviria; Orthornavirae; Pisuviricota; Pisoniviricetes; Picornavirales; Iflaviridae; Iflavirus; unclassified Iflavirus
GCA_018583785.1	9851	5	Formica fusca virus 1	NA	Viruses; Riboviria; Orthornavirae; Negarnaviricota; Haploviricotina; Monjiviricetes; Mononegavirales; Nyamiviridae; Formivirus; Fusca formivirus
GCA_002271145.1	12102	6	Solenopsis invicta virus 4	NA	Viruses; ssRNA viruses; ssRNA positive-strand viruses, no DNA stage; unclassified ssRNA positive-strand viruses
GCA_002355045.1	11851	6	Lasius neglectus virus 1	NA	Viruses; Riboviria; Orthornavirae; Pisuviricota; Pisoniviricetes; Picornavirales; Polycipiviridae; Sopolycivirus
GCA_002270805.1	11900	6	Myrmica scabrinodis virus 1	NA	Viruses; ssRNA viruses; ssRNA positive-strand viruses, no DNA stage; unclassified ssRNA positive-strand viruses
GCA_000866545.1	9695	1	Wisteria vein mosaic virus	NA	Viruses; ssRNA viruses; ssRNA positive-strand viruses, no DNA stage; Potyviridae; Potyvirus
GCA_002219325.1	3024	5	Tinamou hepatitis B virus	NA	Viruses; Riboviria; Pararnavirae; Artverviricota; Revtraviricetes; Blubervirales; Hepadnaviridae; Avihepadnavirus; unclassified Avihepadnavirus
GCA_002270665.1	5772	7	Wheat leaf yellowingassociated virus	NA	Viruses; ssRNA viruses; ssRNA positive-strand viruses, no DNA stage; Luteoviridae; Polerovirus; unclassified Polerovirus
GCA_002271125.1	6930	2	Ocimum basilicum divavirus 1	NA	Viruses; ssRNA viruses; ssRNA positive-strand viruses, no DNA stage; Tymovirales; Betaflexiviridae; Trivirinae; Divavirus; unclassified Divavirus
GCA_002270785.1	2784	1	Ocimum basilicum mitovirus 1	NA	Viruses; ssRNA viruses; ssRNA positive-strand viruses, no DNA stage; Narnaviridae; Mitovirus; unclassified Mitovirus
GCA_002627205.1	110901	166	Escherichia phage OSYSP	NA	Viruses; Duplodnaviria; Heunggongvirae; Uroviricota; Caudoviricetes; Caudovirales; Demerecviridae; Markadamsvirinae; Tequintavirus; Escherichia virus OSYSP
GCA_002270945.1	2746	6	Emilia sonchifolia yellow vein Thailand virus	NA	Viruses; ssDNA viruses; Geminiviridae; Begomovirus; unclassified Begomovirus
GCA_002270645.1	7194	5	Jujube mosaicassociated virus	NA	Viruses; Retro-transcribing viruses; Caulimoviridae; unclassified Caulimoviridae

gca	genome.size	proteome.size	phage.name	strain	Taxonomy
GCA_002271105.1	4832	5	Rice virus A	NA	Viruses; ssRNA viruses; ssRNA positive-strand viruses, no DNA stage; Tombusviridae; unclassified Tombusviridae
GCA_002378485.1	5224	8	Macroptilium golden yellow mosaic virus	NA	Viruses; Monodnaviria; Shotokuvirae; Cressdnaviricota; Repensiviricetes; Geplafuvirales; Geminiviridae; Begomovirus; unclassified Begomovirus
GCA_002270925.1	2629	6	Tomato leaf curl purple vein virus	NA	Viruses; ssDNA viruses; Geminiviridae; Begomovirus; unclassified Begomovirus
GCA_013087495.1	13168	10	Holmes Jungle virus	NA	Viruses; Riboviria; Orthornavirae; Negarnaviricota; Haploviricotina; Monjiviricetes; Mononegavirales; Rhabdoviridae; Hapavirus; Holmes hapavirus
GCA_002375055.1	2585	2	Hudisavirus sp	NA	Viruses; unclassified viruses
GCA_004788535.1	3174	6	Prunus geminivirus A	NA	Viruses; Monodnaviria; Shotokuvirae; Cressdnaviricota; Repensiviricetes; Geplafuvirales; Geminiviridae; Grablovirus; Prunus latent virus
GCA_002627225.1	42091	38	Ralstonia phage DU_RP_II	NA	Viruses; Duplodnaviria; Heunggongvirae; Uroviricota; Caudoviricetes; Caudovirales; Podoviridae; unclassified Podoviridae
GCA_018595035.1	10653	4	Mammarenavirus sp	AreV2170KEN	Viruses; Riboviria; Orthornavirae; Negarnaviricota; Polyploviricotina; Ellioviricetes; Bunyavirales; Arenaviridae; Mammarenavirus; unclassified Mammarenavirus
GCA_002627245.1	19105	26	Bacillus phage vB_BvePGoe6	NA	Viruses; Duplodnaviria; Heunggongvirae; Uroviricota; Caudoviricetes; Caudovirales; Podoviridae; Picovirinae; Salasvirus; unclassified Salasvirus
GCA_004131225.1	7496	5	Grapevine virus G	NA	Viruses; Riboviria; Orthornavirae; Kitrinoviricota; Alsuviricetes; Tymovirales; Betaflexiviridae; Trivirinae; Vitivirus
GCA_003177595.1	8078	7	human papillomavirus 89	NA	Viruses; Monodnaviria; Shotokuvirae; Cossaviricota; Papovaviricetes; Zurhausenvirales; Papillomaviridae; Firstpapillomavirinae; Alphapapillomavirus; Alphapapillomavirus 3
GCA_003177575.1	7966	7	human papillomavirus 91	NA	Viruses; Monodnaviria; Shotokuvirae; Cossaviricota; Papovaviricetes; Zurhausenvirales; Papillomaviridae; Firstpapillomavirinae; Alphapapillomavirus; Alphapapillomavirus 8
GCA_002210635.1	167212	72	Common bottlenose dolphin gammaherpesvirus 1 strain Sarasota	NA	Viruses; Duplodnaviria; Heunggongvirae; Peploviricota; Herviviricetes; Herpesvirales; Herpesviridae; Gammaherpesvirinae; Rhadinovirus; unclassified Rhadinovirus
GCA_002627265.1	45385	54	Marinomonas phage CB5A	NA	Viruses; Duplodnaviria; Heunggongvirae; Uroviricota; Caudoviricetes; Caudovirales; Autographiviridae; Colwellvirinae; Murciavirus; Marinomonas virus CB5A
GCA_002271085.1	9780	21	Metallosphaera turreted icosahedral virus	MTIV1	Viruses; unclassified archaeal viruses
GCA_900205285.1	575161	643	Cedratvirus lausannensis	NA	Viruses; unclassified viruses; unclassified DNA viruses; unclassified dsDNA viruses; Pithoviridae
GCA_002627285.1	9174	16	Flavobacterium phage FLiP	NA	Viruses; Finnlakeviridae; Finnlakevirus; Flavobacterium virus FLiP
GCA_006450685.1	107469	99	Pelophylax esculentus virus	NA	Viruses; Varidnaviria; Bamfordvirae; Nucleocytoviricota; Megaviricetes; Pimascovirales; Iridoviridae; Alphairidovirinae; Ranavirus; Common midwife toad virus
GCA_002627305.1	25032	34	Bacillus phage Juan	NA	Viruses; Duplodnaviria; Heunggongvirae; Uroviricota; Caudoviricetes; Caudovirales; Podoviridae; unclassified Podoviridae
GCA_002627325.1	26061	40	Bacillus phage KonjoTrouble	NA	Viruses; Duplodnaviria; Heunggongvirae; Uroviricota; Caudoviricetes; Caudovirales; Podoviridae; unclassified Podoviridae
GCA_002627345.1	23946	34	Bacillus phage RadRaab	NA	Viruses; Duplodnaviria; Heunggongvirae; Uroviricota; Caudoviricetes; Caudovirales; Podoviridae; unclassified Podoviridae
GCA_006869605.1	26061	46	Bacillus phage VioletteMad	NA	Viruses; Duplodnaviria; Heunggongvirae; Uroviricota; Caudoviricetes; Caudovirales; Podoviridae; unclassified Podoviridae
GCA_002627365.1	55735	103	Mycobacterium phage Spoonbill	NA	Viruses; Duplodnaviria; Heunggongvirae; Uroviricota; Caudoviricetes; Caudovirales; Siphoviridae; Cheoctovirus; unclassified Cheoctovirus
GCA_002627385.1	68324	102	Mycobacterium phage LemonSlice	NA	Viruses; Duplodnaviria; Heunggongvirae; Uroviricota; Caudoviricetes; Caudovirales; Siphoviridae; Bclasvirinae; Pegunavirus; unclassified Pegunavirus
GCA_002627405.1	131976	234	Streptomyces phage Mildred21	NA	Viruses; Duplodnaviria; Heunggongvirae; Uroviricota; Caudoviricetes; Caudovirales; Siphoviridae; Samistivirus; Streptomyces virus Mildred21
GCA_002627425.1	82101	123	Salmonella phage ST11	NA	Viruses; Duplodnaviria; Heunggongvirae; Uroviricota; Caudoviricetes; Caudovirales; Myoviridae; Ounavirinae; Felixounavirus; unclassified Felixounavirus
GCA_002627445.1	131086	221	Streptomyces phage NootNoot	NA	Viruses; Duplodnaviria; Heunggongvirae; Uroviricota; Caudoviricetes; Caudovirales; Siphoviridae; Samistivirus; Streptomyces virus NootNoot
GCA_002627465.1	133486	216	Streptomyces phage Paradiddles	NA	Viruses; Duplodnaviria; Heunggongvirae; Uroviricota; Caudoviricetes; Caudovirales; Siphoviridae; Samistivirus; Streptomyces virus Paradiddles
GCA_002627485.1	133047	226	Streptomyces phage Peebs	NA	Viruses; Duplodnaviria; Heunggongvirae; Uroviricota; Caudoviricetes; Caudovirales; Siphoviridae; Samistivirus; Streptomyces virus Peebs
GCA_002627505.1	133710	226	Streptomyces phage Samisti12	NA	Viruses; Duplodnaviria; Heunggongvirae; Uroviricota; Caudoviricetes; Caudovirales; Siphoviridae; Samistivirus; Streptomyces virus Samisti12
GCA_002627525.1	204930	269	Synechococcus phage Bellamy	NA	Viruses; Duplodnaviria; Heunggongvirae; Uroviricota; Caudoviricetes; Caudovirales; Myoviridae; Bellamyvirus; Synechococcus virus Bellamy
GCA_002743895.1	142204	180	Pseudoalteromonas phage SL20	NA	Viruses; Duplodnaviria; Heunggongvirae; Uroviricota; Caudoviricetes; Caudovirales; Myoviridae; unclassified Myoviridae
GCA_002627565.1	33906	44	Pseudoalteromonas phage SL25	NA	Viruses; Duplodnaviria; Heunggongvirae; Uroviricota; Caudoviricetes; Caudovirales; Siphoviridae; unclassified Siphoviridae
GCA_014488515.1	41559	48	Escherichia phage DN1	NA	Viruses; Duplodnaviria; Heunggongvirae; Uroviricota; Caudoviricetes; Caudovirales; Siphoviridae; Lambdavirus; unclassified Lambdavirus

gca	genome.size	proteome.size	phage.name	strain	Taxonomy
GCA_002627585.1	60746	97	Mycobacterium phage Bella96	NA	Viruses; Duplodnaviria; Heunggongvirae; Uroviricota; Caudoviricetes; Caudovirales; Siphoviridae; Timquatrovirus; unclassified Timquatrovirus
GCA_002627605.1	51138	79	Arthrobacter phage Timinator	NA	Viruses; Duplodnaviria; Heunggongvirae; Uroviricota; Caudoviricetes; Caudovirales; Myoviridae; Marthavirus; unclassified Marthavirus
GCA_002627625.1	49976	72	Arthrobacter phage Franzy	NA	Viruses; Duplodnaviria; Heunggongvirae; Uroviricota; Caudoviricetes; Caudovirales; Myoviridae; Marthavirus; unclassified Marthavirus
GCA_002627645.1	80123	144	Mycobacterium phage GenevaB15	NA	Viruses; Duplodnaviria; Heunggongvirae; Uroviricota; Caudoviricetes; Caudovirales; Siphoviridae; Mclasvirinae; Reyvirus; unclassified Reyvirus
GCA_003288495.1	29837	56	Lactococcus phage vB_Llc_bIBBF14	NA	Viruses; Duplodnaviria; Heunggongvirae; Uroviricota; Caudoviricetes; Caudovirales; Siphoviridae; Skunavirus; unclassified Skunavirus
GCA_002627665.1	229929	402	Aeromonas phage ASzj	NA	Viruses; Duplodnaviria; Heunggongvirae; Uroviricota; Caudoviricetes; Caudovirales; Myoviridae; Tevenvirinae; unclassified Tevenvirinae
GCA_002627685.1	39779	68	Staphylococcus phage SN10	NA	Viruses; Duplodnaviria; Heunggongvirae; Uroviricota; Caudoviricetes; Caudovirales; Siphoviridae; unclassified Siphoviridae
GCA_002627705.1	40108	70	Staphylococcus phage SN11	NA	Viruses; Duplodnaviria; Heunggongvirae; Uroviricota; Caudoviricetes; Caudovirales; Siphoviridae; unclassified Siphoviridae
GCA_002627725.1	40462	71	Staphylococcus phage SN13	NA	Viruses; Duplodnaviria; Heunggongvirae; Uroviricota; Caudoviricetes; Caudovirales; Siphoviridae; unclassified Siphoviridae
GCA_002627745.1	39803	69	Staphylococcus phage SN8	NA	Viruses; Duplodnaviria; Heunggongvirae; Uroviricota; Caudoviricetes; Caudovirales; Siphoviridae; unclassified Siphoviridae
GCA_002627765.1	42425	60	Salmonella phage vB_SenS_PVPSE2	NA	Viruses; Duplodnaviria; Heunggongvirae; Uroviricota; Caudoviricetes; Caudovirales; Siphoviridae; Guernseyvirinae; Jerseyvirus; unclassified Jerseyvirus
GCA_002627785.1	50345	67	Vibrio phage vB_ValP_IME271	NA	Viruses; Duplodnaviria; Heunggongvirae; Uroviricota; Caudoviricetes; Caudovirales; Podoviridae; unclassified Podoviridae
GCA_002627805.1	161772	301	Bacillus phage AaronPhadgers	NA	Viruses; Duplodnaviria; Heunggongvirae; Uroviricota; Caudoviricetes; Caudovirales; Herelleviridae; Bastillevirinae; Wphvirus; unclassified Wphvirus
GCA_002627825.1	162449	302	Bacillus phage Bubs	NA	Viruses; Duplodnaviria; Heunggongvirae; Uroviricota; Caudoviricetes; Caudovirales; Herelleviridae; Bastillevirinae; Wphvirus; unclassified Wphvirus
GCA_002627845.1	160705	285	Bacillus phage Janet	NA	Viruses; Duplodnaviria; Heunggongvirae; Uroviricota; Caudoviricetes; Caudovirales; Herelleviridae; Bastillevirinae; Bastillevirus; unclassified Bastillevirus
GCA_002627865.1	161807	291	Bacillus phage OTooleKemple52	NA	Viruses; Duplodnaviria; Heunggongvirae; Uroviricota; Caudoviricetes; Caudovirales; Herelleviridae; Bastillevirinae; Bastillevirus; unclassified Bastillevirus
GCA_002627885.1	162281	301	Bacillus phage PPIsBest	NA	Viruses; Duplodnaviria; Heunggongvirae; Uroviricota; Caudoviricetes; Caudovirales; Herelleviridae; Bastillevirinae; Wphvirus; unclassified Wphvirus
GCA_002627905.1	162692	303	Bacillus phage Zainny	NA	Viruses; Duplodnaviria; Heunggongvirae; Uroviricota; Caudoviricetes; Caudovirales; Herelleviridae; Bastillevirinae; Wphvirus; unclassified Wphvirus
GCA_003085795.1	147135	233	Staphylococcus phage JA1	Staphylococcus aureus CIT281189	Viruses; Duplodnaviria; Heunggongvirae; Uroviricota; Caudoviricetes; Caudovirales; Herelleviridae; Twortvirinae; Kayvirus; unclassified Kayvirus
GCA_002627925.1	44597	54	Xanthomonas phage phi Xc10	NA	Viruses; Duplodnaviria; Heunggongvirae; Uroviricota; Caudoviricetes; Caudovirales; Autographiviridae; Pradovirus; Xanthomonas virus Xc10
GCA_002627945.1	59100	79	Klebsiella phage KPN N137	NA	Viruses; Duplodnaviria; Heunggongvirae; Uroviricota; Caudoviricetes; Caudovirales; Siphoviridae; unclassified Siphoviridae
GCA_002627965.1	49090	76	Klebsiella phage KPN N141	NA	Viruses; Duplodnaviria; Heunggongvirae; Uroviricota; Caudoviricetes; Caudovirales; Drexlerviridae; Webervirus; Klebsiella virus KPN N141
GCA_002627985.1	59100	78	Klebsiella phage KPN N54	NA	Viruses; Duplodnaviria; Heunggongvirae; Uroviricota; Caudoviricetes; Caudovirales; Siphoviridae; unclassified Siphoviridae
GCA_002628005.1	59087	80	Klebsiella phage KPN U2874	NA	Viruses; Duplodnaviria; Heunggongvirae; Uroviricota; Caudoviricetes; Caudovirales; Siphoviridae; unclassified Siphoviridae
GCA_002628025.1	40048	44	Klebsiella phage 2044307w	NA	Viruses; Duplodnaviria; Heunggongvirae; Uroviricota; Caudoviricetes; Caudovirales; Autographiviridae; Studiervirinae; Przondovirus; Klebsiella virus 2044-307w
GCA_002628045.1	39064	46	Leclercia phage 10164302	NA	Viruses; Duplodnaviria; Heunggongvirae; Uroviricota; Caudoviricetes; Caudovirales; Autographiviridae; Studiervirinae; Teetrevirus; Leclercia virus 10164-302
GCA_002628065.1	39300	46	Leclercia phage 10164RH	NA	Viruses; Duplodnaviria; Heunggongvirae; Uroviricota; Caudoviricetes; Caudovirales; Autographiviridae; Studiervirinae; Teetrevirus; unclassified Teetrevirus
GCA_002628085.1	159631	213	Serratia phage 2050H1	NA	Viruses; Duplodnaviria; Heunggongvirae; Uroviricota; Caudoviricetes; Caudovirales; Ackermannviridae; unclassified Ackermannviridae
GCA_002628105.1	39216	43	Serratia phage 2050H2	NA	Viruses; Duplodnaviria; Heunggongvirae; Uroviricota; Caudoviricetes; Caudovirales; Autographiviridae; Studiervirinae; Teetrevirus; Serratia virus 2050H2
GCA_002628125.1	276025	363	Serratia phage 2050HW	NA	Viruses; Duplodnaviria; Heunggongvirae; Uroviricota; Caudoviricetes; Caudovirales; Myoviridae; unclassified Myoviridae
GCA_002628145.1	159983	279	Bacillus phage Anthony	NA	Viruses; Duplodnaviria; Heunggongvirae; Uroviricota; Caudoviricetes; Caudovirales; Herelleviridae; Bastillevirinae; Bastillevirus; unclassified Bastillevirus
GCA_002628165.1	46932	66	Rhodococcus phage Bonanza	NA	Viruses; Duplodnaviria; Heunggongvirae; Uroviricota; Caudoviricetes; Caudovirales; Siphoviridae; Rerduovirus; unclassified Rerduovirus
GCA_002628185.1	45423	55	Agrobacterium phage Atu_ph02	NA	Viruses; Duplodnaviria; Heunggongvirae; Uroviricota; Caudoviricetes; Caudovirales; Autographiviridae; Atuphduovirus; Agrobacterium virus Atuph02
GCA_002628205.1	45175	58	Agrobacterium phage Atu_ph03	NA	Viruses; Duplodnaviria; Heunggongvirae; Uroviricota; Caudoviricetes; Caudovirales; Autographiviridae; Atuphduovirus; Agrobacterium virus Atuph03

gca	genome.size	proteome.size	phage.name	strain	Taxonomy
GCA_002628225.1	143349	48	Agrobacterium phage Atu_ph04	NA	Viruses; Duplodnaviria; Heunggongvirae; Uroviricota; Caudoviricetes; Caudovirales; Ackermannviridae; unclassified Ackermannviridae
GCA_002628245.1	490380	714	Agrobacterium phage Atu_ph07	NA	Viruses; Duplodnaviria; Heunggongvirae; Uroviricota; Caudoviricetes; Caudovirales; Myoviridae; Polybotosvirus; Agrobacterium virus Atuph07
GCA_002628265.1	58966	75	Agrobacterium phage Atu_ph08	NA	Viruses; Duplodnaviria; Heunggongvirae; Uroviricota; Caudoviricetes; Caudovirales; Podoviridae; unclassified Podoviridae
GCA_002628285.1	58059	97	Arthrobacter phage Arcadia	NA	Viruses; Duplodnaviria; Heunggongvirae; Uroviricota; Caudoviricetes; Caudovirales; Siphoviridae; Mudcatvirus; unclassified Mudcatvirus
GCA_002628305.1	57401	96	Arthrobacter phage Correa	NA	Viruses; Duplodnaviria; Heunggongvirae; Uroviricota; Caudoviricetes; Caudovirales; Siphoviridae; Mudcatvirus; unclassified Mudcatvirus
GCA_002628325.1	58059	97	Arthrobacter phage Elsa	NA	Viruses; Duplodnaviria; Heunggongvirae; Uroviricota; Caudoviricetes; Caudovirales; Siphoviridae; Mudcatvirus; unclassified Mudcatvirus
GCA_002628345.1	58208	100	Arthrobacter phage Heisenberger	NA	Viruses; Duplodnaviria; Heunggongvirae; Uroviricota; Caudoviricetes; Caudovirales; Siphoviridae; Mudcatvirus; unclassified Mudcatvirus
GCA_002628365.1	58059	97	Arthrobacter phage Nason	NA	Viruses; Duplodnaviria; Heunggongvirae; Uroviricota; Caudoviricetes; Caudovirales; Siphoviridae; Mudcatvirus; unclassified Mudcatvirus
GCA_002628385.1	59084	102	Arthrobacter phage Tribby	NA	Viruses; Duplodnaviria; Heunggongvirae; Uroviricota; Caudoviricetes; Caudovirales; Siphoviridae; Mudcatvirus; unclassified Mudcatvirus
GCA_002628405.1	46353	65	Rhodococcus phage Yoncess	NA	Viruses; Duplodnaviria; Heunggongvirae; Uroviricota; Caudoviricetes; Caudovirales; Siphoviridae; Rerduovirus; unclassified Rerduovirus
GCA_002628425.1	51347	78	Arthrobacter phage Jordan	NA	Viruses; Duplodnaviria; Heunggongvirae; Uroviricota; Caudoviricetes; Caudovirales; Myoviridae; Marthavirus; unclassified Marthavirus
GCA_002628445.1	49808	74	Arthrobacter phage Piccoletto	NA	Viruses; Duplodnaviria; Heunggongvirae; Uroviricota; Caudoviricetes; Caudovirales; Myoviridae; Marthavirus; Arthrobacter virus Piccoletto
GCA_002628465.1	50930	77	Arthrobacter phage Shade	NA	Viruses; Duplodnaviria; Heunggongvirae; Uroviricota; Caudoviricetes; Caudovirales; Myoviridae; Marthavirus; Arthrobacter virus Shade
GCA_002628485.1	56076	82	Streptomyces phage DrGrey	NA	Viruses; Duplodnaviria; Heunggongvirae; Uroviricota; Caudoviricetes; Caudovirales; Siphoviridae; Rimavirus; Strepomyces virus Drgrey
GCA_002628505.1	55707	85	Streptomyces phage Spectropatronm	NA	Viruses; Duplodnaviria; Heunggongvirae; Uroviricota; Caudoviricetes; Caudovirales; Siphoviridae; Rimavirus; unclassified Rimavirus
GCA_002628525.1	49941	74	Streptomyces phage BeardedLady	NA	Viruses; Duplodnaviria; Heunggongvirae; Uroviricota; Caudoviricetes; Caudovirales; Siphoviridae; Arquatrovirinae; Likavirus; unclassified Likavirus
GCA_002628545.1	50066	75	Streptomyces phage BryanRecycles	NA	Viruses; Duplodnaviria; Heunggongvirae; Uroviricota; Caudoviricetes; Caudovirales; Siphoviridae; Arquatrovirinae; Likavirus; unclassified Likavirus
GCA_002628565.1	50536	76	Streptomyces phage Celeste	NA	Viruses; Duplodnaviria; Heunggongvirae; Uroviricota; Caudoviricetes; Caudovirales; Siphoviridae; Arquatrovirinae; Likavirus; unclassified Likavirus
GCA_002628585.1	50976	77	Streptomyces phage Dattran	NA	Viruses; Duplodnaviria; Heunggongvirae; Uroviricota; Caudoviricetes; Caudovirales; Siphoviridae; Arquatrovirinae; Likavirus; unclassified Likavirus
GCA_002628605.1	49908	74	Streptomyces phage Esperer	NA	Viruses; Duplodnaviria; Heunggongvirae; Uroviricota; Caudoviricetes; Caudovirales; Siphoviridae; Arquatrovirinae; Likavirus; unclassified Likavirus
GCA_002628625.1	50066	75	Streptomyces phage Jash	NA	Viruses; Duplodnaviria; Heunggongvirae; Uroviricota; Caudoviricetes; Caudovirales; Siphoviridae; Arquatrovirinae; Likavirus; unclassified Likavirus
GCA_002628645.1	49976	75	Streptomyces phage Oliynyk	NA	Viruses; Duplodnaviria; Heunggongvirae; Uroviricota; Caudoviricetes; Caudovirales; Siphoviridae; Arquatrovirinae; Likavirus; unclassified Likavirus
GCA_002628665.1	49961	73	Streptomyces phage Ozzie	NA	Viruses; Duplodnaviria; Heunggongvirae; Uroviricota; Caudoviricetes; Caudovirales; Siphoviridae; Arquatrovirinae; Likavirus; unclassified Likavirus
GCA_002628685.1	53366	97	Mycobacterium phage JoshKayV	NA	Viruses; Duplodnaviria; Heunggongvirae; Uroviricota; Caudoviricetes; Caudovirales; Siphoviridae; Fromanvirus; unclassified Fromanvirus
GCA_002628705.1	50283	87	Mycobacterium phage Kimona	NA	Viruses; Duplodnaviria; Heunggongvirae; Uroviricota; Caudoviricetes; Caudovirales; Siphoviridae; Fromanvirus; unclassified Fromanvirus
GCA_002628725.1	47612	81	Mycobacterium phage Majeke	NA	Viruses; Duplodnaviria; Heunggongvirae; Uroviricota; Caudoviricetes; Caudovirales; Siphoviridae; Pclasvirinae; Fishburnevirus; unclassified Fishburnevirus
GCA_002628745.1	50263	92	Mycobacterium phage MissWhite	NA	Viruses; Duplodnaviria; Heunggongvirae; Uroviricota; Caudoviricetes; Caudovirales; Siphoviridae; Fromanvirus; unclassified Fromanvirus
GCA_002628765.1	60311	97	Mycobacterium phage TreyKay	NA	Viruses; Duplodnaviria; Heunggongvirae; Uroviricota; Caudoviricetes; Caudovirales; Siphoviridae; Timquatrovirus; unclassified Timquatrovirus
GCA_002628785.1	45389	68	Mycobacterium phage Avocado	NA	Viruses; Duplodnaviria; Heunggongvirae; Uroviricota; Caudoviricetes; Caudovirales; Siphoviridae; unclassified Siphoviridae
GCA_002628805.1	50514	94	Mycobacterium phage MyraDee	NA	Viruses; Duplodnaviria; Heunggongvirae; Uroviricota; Caudoviricetes; Caudovirales; Siphoviridae; Fromanvirus; unclassified Fromanvirus
GCA_002955345.1	87575	131	Shigella phage Sf14	NA	Viruses; Duplodnaviria; Heunggongvirae; Uroviricota; Caudoviricetes; Caudovirales; Myoviridae; Ounavirinae; Mooglevirus; Shigella virus Sf14
GCA_002955355.1	90092	138	Shigella phage Sf17	NA	Viruses; Duplodnaviria; Heunggongvirae; Uroviricota; Caudoviricetes; Caudovirales; Myoviridae; Ounavirinae; Mooglevirus; Shigella virus Sf17
GCA_002955365.1	90375	135	Shigella phage Sf19	NA	Viruses; Duplodnaviria; Heunggongvirae; Uroviricota; Caudoviricetes; Caudovirales; Myoviridae; Ounavirinae; Mooglevirus; unclassified Mooglevirus
GCA_002955375.1	163982	270	Shigella phage Sf20	NA	Viruses; Duplodnaviria; Heunggongvirae; Uroviricota; Caudoviricetes; Caudovirales; Myoviridae; Tevenvirinae; Krischvirus; unclassified Krischvirus

gca	genome.size	proteome.size	phage.name	strain	Taxonomy
GCA_002955385.1	166002	266	Shigella phage Sf21	NA	Viruses; Duplodnaviria; Heunggongvirae; Uroviricota; Caudoviricetes; Caudovirales; Myoviridae; Tevenvirinae; Tequatrovirus; Shigella virus Sf21
GCA_002955395.1	168112	271	Shigella phage Sf24	NA	Viruses; Duplodnaviria; Heunggongvirae; Uroviricota; Caudoviricetes; Caudovirales; Myoviridae; Tevenvirinae; Tequatrovirus; Shigella virus Sf24
GCA_002955405.1	168573	272	Shigella phage Sf25	NA	Viruses; Duplodnaviria; Heunggongvirae; Uroviricota; Caudoviricetes; Caudovirales; Myoviridae; Tevenvirinae; Tequatrovirus; unclassified Tequatrovirus
GCA_002628845.1	42701	62	Salmonella phage St162	NA	Viruses; Duplodnaviria; Heunggongvirae; Uroviricota; Caudoviricetes; Caudovirales; Siphoviridae; Guernseyvirinae; Cornellvirus; unclassified Cornellvirus
GCA_002628865.1	48262	73	Shigella phage Sd1	NA	Viruses; Duplodnaviria; Heunggongvirae; Uroviricota; Caudoviricetes; Caudovirales; Drexlerviridae; Rogunavirinae; Wilsonroadvirus; Shigella virus Sd1
GCA_003323775.1	50403	81	Shigella phage Sfin1	NA	Viruses; Duplodnaviria; Heunggongvirae; Uroviricota; Caudoviricetes; Caudovirales; Drexlerviridae; Tunavirinae; Tunavirus; Shigella virus Sfin1
GCA_002628905.1	47647	74	Shigella phage Sf12	NA	Viruses; Duplodnaviria; Heunggongvirae; Uroviricota; Caudoviricetes; Caudovirales; Drexlerviridae; Rogunavirinae; Eastlansingvirus; Shigella virus Sf12
GCA_002628925.1	87570	132	Shigella phage Sf13	NA	Viruses; Duplodnaviria; Heunggongvirae; Uroviricota; Caudoviricetes; Caudovirales; Myoviridae; Ounavirinae; Mooglevirus; Shigella virus Sf13
GCA_002628945.1	88474	134	Shigella phage Sf15	NA	Viruses; Duplodnaviria; Heunggongvirae; Uroviricota; Caudoviricetes; Caudovirales; Myoviridae; Ounavirinae; Mooglevirus; unclassified Mooglevirus
GCA_002628965.1	88580	134	Shigella phage Sf16	NA	Viruses; Duplodnaviria; Heunggongvirae; Uroviricota; Caudoviricetes; Caudovirales; Myoviridae; Ounavirinae; Mooglevirus; unclassified Mooglevirus
GCA_002628985.1	90270	136	Shigella phage Sf18	NA	Viruses; Duplodnaviria; Heunggongvirae; Uroviricota; Caudoviricetes; Caudovirales; Myoviridae; Ounavirinae; Mooglevirus; unclassified Mooglevirus
GCA_002743575.1	166283	267	Shigella phage Sf22	NA	Viruses; Duplodnaviria; Heunggongvirae; Uroviricota; Caudoviricetes; Caudovirales; Myoviridae; Tevenvirinae; Tequatrovirus; Shigella virus Sf22
GCA_002629005.1	167678	271	Shigella phage Sf23	NA	Viruses; Duplodnaviria; Heunggongvirae; Uroviricota; Caudoviricetes; Caudovirales; Myoviridae; Tevenvirinae; Tequatrovirus; unclassified Tequatrovirus
GCA_002955415.1	120620	165	Bacteriophage T5like saus111K	NA	Viruses; Duplodnaviria; Heunggongvirae; Uroviricota; Caudoviricetes; Caudovirales; Demerecviridae; Markadamsvirinae; Tequintavirus; unclassified Tequintavirus
GCA_002955425.1	120629	165	Bacteriophage T5like poul124	NA	Viruses; Duplodnaviria; Heunggongvirae; Uroviricota; Caudoviricetes; Caudovirales; Demerecviridae; Markadamsvirinae; Tequintavirus; unclassified Tequintavirus
GCA_002955435.1	121986	167	Bacteriophage T5like chee130_1	NA	Viruses; Duplodnaviria; Heunggongvirae; Uroviricota; Caudoviricetes; Caudovirales; Demerecviridae; Markadamsvirinae; Epseptimavirus; unclassified Epseptimavirus
GCA_002955445.1	121986	164	Escherichia phage saus132	NA	Viruses; Duplodnaviria; Heunggongvirae; Uroviricota; Caudoviricetes; Caudovirales; Demerecviridae; Markadamsvirinae; Epseptimavirus; Escherichia virus saus132
GCA_002955455.1	121986	164	Bacteriophage T5like poul149	NA	Viruses; Duplodnaviria; Heunggongvirae; Uroviricota; Caudoviricetes; Caudovirales; Demerecviridae; Markadamsvirinae; Epseptimavirus; unclassified Epseptimavirus
GCA_002955465.1	121986	167	Bacteriophage T5like chee158	NA	Viruses; Duplodnaviria; Heunggongvirae; Uroviricota; Caudoviricetes; Caudovirales; Demerecviridae; Markadamsvirinae; Epseptimavirus; unclassified Epseptimavirus
GCA_002955475.1	121986	165	Bacteriophage T5like cott162	NA	Viruses; Duplodnaviria; Heunggongvirae; Uroviricota; Caudoviricetes; Caudovirales; Demerecviridae; Markadamsvirinae; Epseptimavirus; unclassified Epseptimavirus
GCA_002955485.1	121986	167	Bacteriophage T5like saus176N	NA	Viruses; Duplodnaviria; Heunggongvirae; Uroviricota; Caudoviricetes; Caudovirales; Demerecviridae; Markadamsvirinae; Epseptimavirus; unclassified Epseptimavirus
GCA_002955495.1	120622	166	Escherichia phage chee24	NA	Viruses; Duplodnaviria; Heunggongvirae; Uroviricota; Caudoviricetes; Caudovirales; Demerecviridae; Markadamsvirinae; Tequintavirus; Escherichia virus chee24
GCA_002955505.1	120618	168	Bacteriophage T5like pork27	NA	Viruses; Duplodnaviria; Heunggongvirae; Uroviricota; Caudoviricetes; Caudovirales; Demerecviridae; Markadamsvirinae; Tequintavirus; unclassified Tequintavirus
GCA_002955515.1	120622	166	Bacteriophage T5like pork29	NA	Viruses; Duplodnaviria; Heunggongvirae; Uroviricota; Caudoviricetes; Caudovirales; Demerecviridae; Markadamsvirinae; Tequintavirus; unclassified Tequintavirus
GCA_002955525.1	120622	165	Bacteriophage T5like saus47N	NA	Viruses; Duplodnaviria; Heunggongvirae; Uroviricota; Caudoviricetes; Caudovirales; Demerecviridae; Markadamsvirinae; Tequintavirus; unclassified Tequintavirus
GCA_003314955.1	22434	38	Lactococcus phage 05802	NA	Viruses; Duplodnaviria; Heunggongvirae; Uroviricota; Caudoviricetes; Caudovirales; Siphoviridae; Ceduovirus; unclassified Ceduovirus
GCA_003314875.1	22029	38	Lactococcus phage 37203	NA	Viruses; Duplodnaviria; Heunggongvirae; Uroviricota; Caudoviricetes; Caudovirales; Siphoviridae; Ceduovirus; unclassified Ceduovirus
GCA_003314935.1	22242	38	Lactococcus phage 50102	NA	Viruses; Duplodnaviria; Heunggongvirae; Uroviricota; Caudoviricetes; Caudovirales; Siphoviridae; Ceduovirus; unclassified Ceduovirus
GCA_003314915.1	21562	37	Lactococcus phage 50504	NA	Viruses; Duplodnaviria; Heunggongvirae; Uroviricota; Caudoviricetes; Caudovirales; Siphoviridae; Ceduovirus; unclassified Ceduovirus
GCA_003314835.1	21980	37	Lactococcus phage 62402	NA	Viruses; Duplodnaviria; Heunggongvirae; Uroviricota; Caudoviricetes; Caudovirales; Siphoviridae; Ceduovirus; unclassified Ceduovirus
GCA_003314855.1	21704	37	Lactococcus phage 62403	NA	Viruses; Duplodnaviria; Heunggongvirae; Uroviricota; Caudoviricetes; Caudovirales; Siphoviridae; Ceduovirus; unclassified Ceduovirus
GCA_003314815.1	22185	37	Lactococcus phage 62606	NA	Viruses; Duplodnaviria; Heunggongvirae; Uroviricota; Caudoviricetes; Caudovirales; Siphoviridae; Ceduovirus; unclassified Ceduovirus
GCA_003314895.1	21879	38	Lactococcus phage 74001	NA	Viruses; Duplodnaviria; Heunggongvirae; Uroviricota; Caudoviricetes; Caudovirales; Siphoviridae; Ceduovirus; unclassified Ceduovirus
GCA_002629025.1	148464	236	Staphylococcus phage vB_SauMfRuSau02	NA	Viruses; Duplodnaviria; Heunggongvirae; Uroviricota; Caudoviricetes; Caudovirales; Herelleviridae; Twortvirinae; Kayvirus; unclassified Kayvirus

gca	genome.size	proteome.size	phage.name	strain	Taxonomy
GCA_002270765.2	9384	3	Citrus concave gumassociated virus	NA	Viruses; ssRNA viruses; ssRNA negative-strand viruses; Bunyavirales; Phenuiviridae; unclassified Phenuiviridae
GCA_002743595.1	44638	52	Caulobacter phage Lullwater	NA	Viruses; Duplodnaviria; Heunggongvirae; Uroviricota; Caudoviricetes; Caudovirales; Autographiviridae; Lullwatervirus; Caulobacter virus Lullwater
GCA_002966405.1	1385869	1207	Bodo saltans virus	NG1	Viruses; Varidnaviria; Bamfordvirae; Nucleocytoviricota; Megaviricetes; Imitervirales; Mimiviridae; unclassified Mimiviridae; Klosneuvirinae; Klosneuvirus
GCA_002270905.1	18848	12	Actinidia virus 1	NA	Viruses; ssRNA viruses; ssRNA positive-strand viruses, no DNA stage; Closteroviridae; unclassified Closteroviridae
GCA_003147465.1	9626	1	Narcissus virus 1	NA	Viruses; Riboviria; Orthornavirae; Pisuviricota; Stelpaviricetes; Patatavirales; Potyviridae; Potyvirus; unclassified Potyvirus
GCA_002988085.1	1221	1	Satellite tobacco necrosis virus C	NA	Viruses; Riboviria; Albetovirus; Tobacco albetovirus 3
GCA_002890595.1	4819	2	Rosellinia necatrix partitivirus 8	RnBb	Viruses; Riboviria; Orthornavirae; Pisuviricota; Duplopiviricetes; Durnavirales; Partitiviridae; unclassified Partitiviridae
GCA_002270625.1	8218	6	Sus scrofa papillomavirus 2	NA	Viruses; dsDNA viruses, no RNA stage; Papillomaviridae; unclassified Papillomaviridae; non-primate mammal papillomaviruses
GCA_002271065.1	7600	7	Felis catus papillomavirus type 5	NA	Viruses; dsDNA viruses, no RNA stage; Papillomaviridae; unclassified Papillomaviridae; non-primate mammal papillomaviruses
GCA_002270745.1	3278	6	Wild vitis virus 1	NA	Viruses; ssDNA viruses; Geminiviridae; unclassified Geminiviridae
GCA_002270885.1	204055	206	Murmansk poxvirus	LEIV11411	Viruses; dsDNA viruses, no RNA stage; Poxviridae; Chordopoxvirinae; Centapoxvirus; unclassified Centapoxvirus
GCA_002270605.1	200223	197	NY_014 poxvirus	2013	Viruses; dsDNA viruses, no RNA stage; Poxviridae; Chordopoxvirinae; Centapoxvirus; unclassified Centapoxvirus
GCA_004130985.1	2746	4	Maize striate mosaic virus	NA	Viruses; Monodnaviria; Shotokuvirae; Cressdnaviricota; Repensiviricetes; Geplafuvirales; Geminiviridae; Mastrevirus
GCA_003848685.1	2373	3	Tickassociated genomovirus 1	NA	Viruses; Monodnaviria; Shotokuvirae; Cressdnaviricota; Repensiviricetes; Geplafuvirales; Genomoviridae; unclassified Genomoviridae
GCA_003848665.1	2196	3	Tickassociated genomovirus 2	NA	Viruses; Monodnaviria; Shotokuvirae; Cressdnaviricota; Repensiviricetes; Geplafuvirales; Genomoviridae; unclassified Genomoviridae
GCA_003848645.1	2238	3	Tickassociated genomovirus 3	NA	Viruses; Monodnaviria; Shotokuvirae; Cressdnaviricota; Repensiviricetes; Geplafuvirales; Genomoviridae; unclassified Genomoviridae
GCA_018582805.1	2782	3	Tick associated torque teno virus	NA	Viruses; Anelloviridae; Thetatorquevirus; Torque teno arthrovec virus 3
GCA_002270725.1	10187	3	Abisko virus	NA	Viruses; unclassified RNA viruses
GCA_000859565.1	10349	2	Cherry rasp leaf virus	NA	Viruses; ssRNA viruses; ssRNA positive-strand viruses, no DNA stage; Picornavirales; Secoviridae; Cheravirus
GCA_002629045.1	235108	243	Erwinia phage vB_EamM_RisingSun	NA	Viruses; Duplodnaviria; Heunggongvirae; Uroviricota; Caudoviricetes; Caudovirales; Myoviridae; Risingsunvirus; Erwinia virus Risingsun
GCA_002629065.1	235374	245	Erwinia phage vB_EamM_Joad	NA	Viruses; Duplodnaviria; Heunggongvirae; Uroviricota; Caudoviricetes; Caudovirales; Myoviridae; Risingsunvirus; unclassified Risingsunvirus
GCA_002629085.1	162422	237	Aeromonas phage ASgz	NA	Viruses; Duplodnaviria; Heunggongvirae; Uroviricota; Caudoviricetes; Caudovirales; Myoviridae; Tulanevirus; Aeromonas virus Asgz
GCA_009674025.1	65275	97	Nocardia phage NTR1	NA	Viruses; Duplodnaviria; Heunggongvirae; Uroviricota; Caudoviricetes; Caudovirales; Siphoviridae; unclassified Siphoviridae
GCA_002743615.1	43573	51	Vibrio phage vB_VspS_VSABTNL3	NA	Viruses; Duplodnaviria; Heunggongvirae; Uroviricota; Caudoviricetes; Caudovirales; Podoviridae; unclassified Podoviridae
GCA_002955545.1	38593	49	Vibrio phage JSF25	NA	Viruses; Duplodnaviria; Heunggongvirae; Uroviricota; Caudoviricetes; Caudovirales; Autographiviridae; Studiervirinae; Chatterjeevirus; unclassified Chatterjeevirus
GCA_002629105.1	59100	78	Klebsiella phage YMC1511N53_KPN_BP	NA	Viruses; Duplodnaviria; Heunggongvirae; Uroviricota; Caudoviricetes; Caudovirales; Siphoviridae; unclassified Siphoviridae
GCA_002629125.1	58387	70	Klebsiella phage YMC1601N133_KPN_BP	NA	Viruses; Duplodnaviria; Heunggongvirae; Uroviricota; Caudoviricetes; Caudovirales; Siphoviridae; unclassified Siphoviridae
GCA_002743635.1	229957	407	Aeromonas phage ASszw	NA	Viruses; Duplodnaviria; Heunggongvirae; Uroviricota; Caudoviricetes; Caudovirales; Myoviridae; Tevenvirinae; unclassified Tevenvirinae
GCA_013087665.1	11988	5	Taiwan bat lyssavirus	NA	Viruses; Riboviria; Orthornavirae; Negarnaviricota; Haploviricotina; Monjiviricetes; Mononegavirales; Rhabdoviridae; Lyssavirus
GCA_003991625.1	50910	89	Escherichia phage vB_EcoS95	NA	Viruses; Duplodnaviria; Heunggongvirae; Uroviricota; Caudoviricetes; Caudovirales; Drexlerviridae; Tempevirinae; Warwickvirus; Escherichia virus 95
GCA_000912175.1	2786	6	Sweet potato leaf curl Sichuan virus 2	NA	Viruses; Monodnaviria; Shotokuvirae; Cressdnaviricota; Repensiviricetes; Geplafuvirales; Geminiviridae; Begomovirus
GCA_002823585.1	2764	6	Sweet potato leaf curl Sichuan virus 1	NA	Viruses; Monodnaviria; Shotokuvirae; Cressdnaviricota; Repensiviricetes; Geplafuvirales; Geminiviridae; Begomovirus
GCA_002354925.1	6203	2	Australian Anopheles totivirus	NA	Viruses; Riboviria; Orthornavirae; Duplornaviricota; Chrymotiviricetes; Ghabrivirales; Totiviridae; unclassified Totiviridae
GCA_002955555.1	29953	52	Lactococcus phage LP8511	NA	Viruses; Duplodnaviria; Heunggongvirae; Uroviricota; Caudoviricetes; Caudovirales; Siphoviridae; Skunavirus; unclassified Skunavirus

gca	genome.size	proteome.size	phage.name	strain	Taxonomy
GCA_004049255.1	2117	2	Bat bug circovirus	NA	Viruses; Monodnaviria; Shotokuvirae; Cressdnaviricota; Arfiviricetes; Cirlivirales; Circoviridae; Circovirus; unclassified Circovirus
GCA_006450775.1	104968	101	Rana catesbeiana virus 2	NA	Viruses; Varidnaviria; Bamfordvirae; Nucleocytoviricota; Megaviricetes; Pimascovirales; Iridoviridae; Alphairidovirinae; Ranavirus; unclassified Ranavirus
GCA_002629145.1	43012	53	Klebsiella phage AltoGao	NA	Viruses; Duplodnaviria; Heunggongvirae; Uroviricota; Caudoviricetes; Caudovirales; Autographiviridae; Slopekvirinae; Drulisvirus; Klebsiella virus AltoGao
GCA_002629165.1	61644	77	Klebsiella phage SopranoGao	NA	Viruses; Duplodnaviria; Heunggongvirae; Uroviricota; Caudoviricetes; Caudovirales; Podoviridae; unclassified Podoviridae
GCA_002629185.1	49807	76	Klebsiella phage MezzoGao	NA	Viruses; Duplodnaviria; Heunggongvirae; Uroviricota; Caudoviricetes; Caudovirales; Drexlerviridae; Webervirus; Klebsiella virus MezzoGao
GCA_014131355.1	3776	3	Leviviridae sp	NA	Viruses; Riboviria; Orthornavirae; Lenarviricota; Allassoviricetes; Levivirales; Leviviridae; unclassified Leviviridae
GCA_002955565.1	29157	52	Lactococcus phage LP9104	NA	Viruses; Duplodnaviria; Heunggongvirae; Uroviricota; Caudoviricetes; Caudovirales; Siphoviridae; Skunavirus; unclassified Skunavirus
GCA_002955575.1	29098	51	Lactococcus phage LP9205a	NA	Viruses; Duplodnaviria; Heunggongvirae; Uroviricota; Caudoviricetes; Caudovirales; Siphoviridae; Skunavirus; unclassified Skunavirus
GCA_002955585.1	30296	53	Lactococcus phage LP9205b	NA	Viruses; Duplodnaviria; Heunggongvirae; Uroviricota; Caudoviricetes; Caudovirales; Siphoviridae; Skunavirus; unclassified Skunavirus
GCA_002955595.1	29097	52	Lactococcus phage LP9206a	NA	Viruses; Duplodnaviria; Heunggongvirae; Uroviricota; Caudoviricetes; Caudovirales; Siphoviridae; Skunavirus; unclassified Skunavirus
GCA_002955605.1	30309	53	Lactococcus phage LP9206b	NA	Viruses; Duplodnaviria; Heunggongvirae; Uroviricota; Caudoviricetes; Caudovirales; Siphoviridae; Skunavirus; unclassified Skunavirus
GCA_002955615.1	29291	53	Lactococcus phage LP9206c	NA	Viruses; Duplodnaviria; Heunggongvirae; Uroviricota; Caudoviricetes; Caudovirales; Siphoviridae; Skunavirus; unclassified Skunavirus
GCA_002955625.1	29203	54	Lactococcus phage LP9207	NA	Viruses; Duplodnaviria; Heunggongvirae; Uroviricota; Caudoviricetes; Caudovirales; Siphoviridae; Skunavirus; unclassified Skunavirus
GCA_002955635.1	29525	52	Lactococcus phage LP9210	NA	Viruses; Duplodnaviria; Heunggongvirae; Uroviricota; Caudoviricetes; Caudovirales; Siphoviridae; Skunavirus; unclassified Skunavirus
GCA_002955645.1	30072	54	Lactococcus phage LP9404	NA	Viruses; Duplodnaviria; Heunggongvirae; Uroviricota; Caudoviricetes; Caudovirales; Siphoviridae; Skunavirus; unclassified Skunavirus
GCA_002955655.1	30083	53	Lactococcus phage LP9405a	NA	Viruses; Duplodnaviria; Heunggongvirae; Uroviricota; Caudoviricetes; Caudovirales; Siphoviridae; Skunavirus; unclassified Skunavirus
GCA_002955665.1	29294	52	Lactococcus phage LP9405b	NA	Viruses; Duplodnaviria; Heunggongvirae; Uroviricota; Caudoviricetes; Caudovirales; Siphoviridae; Skunavirus; unclassified Skunavirus
GCA_002955675.1	30237	54	Lactococcus phage LP9406	NA	Viruses; Duplodnaviria; Heunggongvirae; Uroviricota; Caudoviricetes; Caudovirales; Siphoviridae; Skunavirus; unclassified Skunavirus
GCA_002955685.1	30945	55	Lactococcus phage LP9609	NA	Viruses; Duplodnaviria; Heunggongvirae; Uroviricota; Caudoviricetes; Caudovirales; Siphoviridae; Skunavirus; unclassified Skunavirus
GCA_002955695.1	30545	54	Lactococcus phage LP9701	NA	Viruses; Duplodnaviria; Heunggongvirae; Uroviricota; Caudoviricetes; Caudovirales; Siphoviridae; Skunavirus; unclassified Skunavirus
GCA_002955705.1	30204	55	Lactococcus phage LP9801	NA	Viruses; Duplodnaviria; Heunggongvirae; Uroviricota; Caudoviricetes; Caudovirales; Siphoviridae; Skunavirus; unclassified Skunavirus
GCA_002955715.1	29557	54	Lactococcus phage LP9903	NA	Viruses; Duplodnaviria; Heunggongvirae; Uroviricota; Caudoviricetes; Caudovirales; Siphoviridae; Skunavirus; unclassified Skunavirus
GCA_002955725.1	29668	53	Lactococcus phage LP9908	NA	Viruses; Duplodnaviria; Heunggongvirae; Uroviricota; Caudoviricetes; Caudovirales; Siphoviridae; Skunavirus; unclassified Skunavirus
GCA_002955735.1	30093	55	Lactococcus phage LP0004a	NA	Viruses; Duplodnaviria; Heunggongvirae; Uroviricota; Caudoviricetes; Caudovirales; Siphoviridae; Skunavirus; unclassified Skunavirus
GCA_002955745.1	30953	56	Lactococcus phage LP0004b	NA	Viruses; Duplodnaviria; Heunggongvirae; Uroviricota; Caudoviricetes; Caudovirales; Siphoviridae; Skunavirus; unclassified Skunavirus
GCA_002955755.1	30044	55	Lactococcus phage LP0004c	NA	Viruses; Duplodnaviria; Heunggongvirae; Uroviricota; Caudoviricetes; Caudovirales; Siphoviridae; Skunavirus; unclassified Skunavirus
GCA_002955765.1	30104	55	Lactococcus phage LP0004d	NA	Viruses; Duplodnaviria; Heunggongvirae; Uroviricota; Caudoviricetes; Caudovirales; Siphoviridae; Skunavirus; unclassified Skunavirus
GCA_002955775.1	30537	54	Lactococcus phage LP0109	NA	Viruses; Duplodnaviria; Heunggongvirae; Uroviricota; Caudoviricetes; Caudovirales; Siphoviridae; Skunavirus; unclassified Skunavirus
GCA_002955785.1	30236	54	Lactococcus phage LP0202	NA	Viruses; Duplodnaviria; Heunggongvirae; Uroviricota; Caudoviricetes; Caudovirales; Siphoviridae; Skunavirus; unclassified Skunavirus
GCA_002955795.1	30339	53	Lactococcus phage LP0209	NA	Viruses; Duplodnaviria; Heunggongvirae; Uroviricota; Caudoviricetes; Caudovirales; Siphoviridae; Skunavirus; unclassified Skunavirus
GCA_002955805.1	29916	52	Lactococcus phage LP0212	NA	Viruses; Duplodnaviria; Heunggongvirae; Uroviricota; Caudoviricetes; Caudovirales; Siphoviridae; Skunavirus; unclassified Skunavirus
GCA_002955815.1	30344	54	Lactococcus phage LP0304	NA	Viruses; Duplodnaviria; Heunggongvirae; Uroviricota; Caudoviricetes; Caudovirales; Siphoviridae; Skunavirus; unclassified Skunavirus
GCA_002955825.1	30547	54	Lactococcus phage LP0509	NA	Viruses; Duplodnaviria; Heunggongvirae; Uroviricota; Caudoviricetes; Caudovirales; Siphoviridae; Skunavirus; unclassified Skunavirus
GCA_002955835.1	30547	54	Lactococcus phage LP0604	NA	Viruses; Duplodnaviria; Heunggongvirae; Uroviricota; Caudoviricetes; Caudovirales; Siphoviridae; Skunavirus; unclassified Skunavirus

gca	genome.size	proteome.size	phage.name	strain	Taxonomy
GCA_002955845.1	31049	54	Lactococcus phage LP0903	NA	Viruses; Duplodnaviria; Heunggongvirae; Uroviricota; Caudoviricetes; Caudovirales; Siphoviridae; Skunavirus; unclassified Skunavirus
GCA_002955855.1	30541	56	Lactococcus phage LP1005	NA	Viruses; Duplodnaviria; Heunggongvirae; Uroviricota; Caudoviricetes; Caudovirales; Siphoviridae; Skunavirus; unclassified Skunavirus
GCA_002955865.1	30542	56	Lactococcus phage LP1011	NA	Viruses; Duplodnaviria; Heunggongvirae; Uroviricota; Caudoviricetes; Caudovirales; Siphoviridae; Skunavirus; unclassified Skunavirus
GCA_002955875.1	30528	55	Lactococcus phage LP1110	NA	Viruses; Duplodnaviria; Heunggongvirae; Uroviricota; Caudoviricetes; Caudovirales; Siphoviridae; Skunavirus; unclassified Skunavirus
GCA_002955885.1	30508	55	Lactococcus phage LP1407	NA	Viruses; Duplodnaviria; Heunggongvirae; Uroviricota; Caudoviricetes; Caudovirales; Siphoviridae; Skunavirus; unclassified Skunavirus
GCA_002955895.1	30247	52	Lactococcus phage LP1502a	NA	Viruses; Duplodnaviria; Heunggongvirae; Uroviricota; Caudoviricetes; Caudovirales; Siphoviridae; Skunavirus; unclassified Skunavirus
GCA_002955905.1	30227	52	Lactococcus phage LP1502b	NA	Viruses; Duplodnaviria; Heunggongvirae; Uroviricota; Caudoviricetes; Caudovirales; Siphoviridae; Skunavirus; unclassified Skunavirus
GCA_002955915.1	30247	52	Lactococcus phage LP1502c	NA	Viruses; Duplodnaviria; Heunggongvirae; Uroviricota; Caudoviricetes; Caudovirales; Siphoviridae; Skunavirus; unclassified Skunavirus
GCA_004794635.1	10901	3	Laurel Lake virus	NA	Viruses; Riboviria; Orthornavirae; Negarnaviricota; Polyploviricotina; Ellioviricetes; Bunyavirales; Phenuiviridae; Laulavirus; Laurel Lake laulavirus
GCA_002285045.1	2611	5	Tomato associated geminivirus 1	NA	Viruses; ssDNA viruses; Geminiviridae; unclassified Geminiviridae
GCA_002629205.1	45428	56	Arthrobacter phage Adat	NA	Viruses; Duplodnaviria; Heunggongvirae; Uroviricota; Caudoviricetes; Caudovirales; Podoviridae; Jasminevirus; Arthrobacter virus Adat
GCA_002629225.1	45426	56	Arthrobacter phage GurgleFerb	NA	Viruses; Duplodnaviria; Heunggongvirae; Uroviricota; Caudoviricetes; Caudovirales; Podoviridae; Jasminevirus; unclassified Jasminevirus
GCA_002629245.1	70362	112	Arthrobacter phage JayCookie	NA	Viruses; Duplodnaviria; Heunggongvirae; Uroviricota; Caudoviricetes; Caudovirales; Myoviridae; Radnorvirus; unclassified Radnorvirus
GCA_002629265.1	45428	57	Arthrobacter phage Nellie	NA	Viruses; Duplodnaviria; Heunggongvirae; Uroviricota; Caudoviricetes; Caudovirales; Podoviridae; Jasminevirus; unclassified Jasminevirus
GCA_002629285.1	43950	60	Arthrobacter phage Temper16	NA	Viruses; Duplodnaviria; Heunggongvirae; Uroviricota; Caudoviricetes; Caudovirales; Siphoviridae; Korravirus; unclassified Korravirus
GCA_002629305.1	50774	67	Brevibacterium phage LuckyBarnes	NA	Viruses; Duplodnaviria; Heunggongvirae; Uroviricota; Caudoviricetes; Caudovirales; Siphoviridae; Luckybarnesvirus; Brevibacterium virus LuckyBarnes
GCA_002629325.1	51238	97	Gordonia phage ShayRa	NA	Viruses; Duplodnaviria; Heunggongvirae; Uroviricota; Caudoviricetes; Caudovirales; Siphoviridae; Soupsvirus; unclassified Soupsvirus
GCA_002629345.1	59947	98	Mycobacterium phage Apocalypse	NA	Viruses; Duplodnaviria; Heunggongvirae; Uroviricota; Caudoviricetes; Caudovirales; Siphoviridae; Timquatrovirus; unclassified Timquatrovirus
GCA_002629365.1	41903	63	Mycobacterium phage Aroostook	NA	Viruses; Duplodnaviria; Heunggongvirae; Uroviricota; Caudoviricetes; Caudovirales; Siphoviridae; Liefievirus; unclassified Liefievirus
GCA_002629385.1	52719	96	Mycobacterium phage Drake55	NA	Viruses; Duplodnaviria; Heunggongvirae; Uroviricota; Caudoviricetes; Caudovirales; Siphoviridae; Fromanvirus; unclassified Fromanvirus
GCA_002629405.1	56418	109	Mycobacterium phage Emma	NA	Viruses; Duplodnaviria; Heunggongvirae; Uroviricota; Caudoviricetes; Caudovirales; Siphoviridae; Cheoctovirus; unclassified Cheoctovirus
GCA_002629425.1	55765	97	Mycobacterium phage Geralt	NA	Viruses; Duplodnaviria; Heunggongvirae; Uroviricota; Caudoviricetes; Caudovirales; Siphoviridae; Cheoctovirus; unclassified Cheoctovirus
GCA_002629445.1	41903	63	Mycobacterium phage Gideon	NA	Viruses; Duplodnaviria; Heunggongvirae; Uroviricota; Caudoviricetes; Caudovirales; Siphoviridae; Liefievirus; unclassified Liefievirus
GCA_002629465.1	68056	97	Mycobacterium phage Lulumae	NA	Viruses; Duplodnaviria; Heunggongvirae; Uroviricota; Caudoviricetes; Caudovirales; Siphoviridae; Bclasvirinae; Pegunavirus; unclassified Pegunavirus
GCA_002629485.1	75554	146	Mycobacterium phage MadamMonkfish	NA	Viruses; Duplodnaviria; Heunggongvirae; Uroviricota; Caudoviricetes; Caudovirales; Siphoviridae; Kostyavirus; unclassified Kostyavirus
GCA_002629505.1	51294	86	Mycobacterium phage Morpher26	NA	Viruses; Duplodnaviria; Heunggongvirae; Uroviricota; Caudoviricetes; Caudovirales; Siphoviridae; Fromanvirus; unclassified Fromanvirus
GCA_002629525.1	164254	244	Mycobacterium phage Phabba	NA	Viruses; Duplodnaviria; Heunggongvirae; Uroviricota; Caudoviricetes; Caudovirales; Myoviridae; unclassified Myoviridae
GCA_002629545.1	57789	103	Mycobacterium phage RitaG	NA	Viruses; Duplodnaviria; Heunggongvirae; Uroviricota; Caudoviricetes; Caudovirales; Siphoviridae; Cheoctovirus; unclassified Cheoctovirus
GCA_002629565.1	54655	100	Mycobacterium phage Smairt	NA	Viruses; Duplodnaviria; Heunggongvirae; Uroviricota; Caudoviricetes; Caudovirales; Siphoviridae; Fromanvirus; unclassified Fromanvirus
GCA_002629585.1	110240	238	Mycobacterium phage Squint	NA	Viruses; Duplodnaviria; Heunggongvirae; Uroviricota; Caudoviricetes; Caudovirales; Siphoviridae; Omegavirus; unclassified Omegavirus
GCA_002629605.1	54513	101	Mycobacterium phage Wachhund	NA	Viruses; Duplodnaviria; Heunggongvirae; Uroviricota; Caudoviricetes; Caudovirales; Siphoviridae; Cheoctovirus; unclassified Cheoctovirus
GCA_002629625.1	52122	100	Mycobacterium phage Wiks	NA	Viruses; Duplodnaviria; Heunggongvirae; Uroviricota; Caudoviricetes; Caudovirales; Siphoviridae; Fromanvirus; unclassified Fromanvirus
GCA_002743655.1	139262	252	Rhodococcus phage Trina	NA	Viruses; Duplodnaviria; Heunggongvirae; Uroviricota; Caudoviricetes; Caudovirales; Siphoviridae; Trinavirus; Rhodococcus virus Trina
GCA_000845645.1	4721	2	Adenoassociated virus 7	NA	Viruses; ssDNA viruses; Parvoviridae; Parvovirinae; Dependoparvovirus; Adeno-associated dependoparvovirus A; Adeno-associated virus - 4

gca	genome.size	proteome.size	phage.name	strain	Taxonomy
GCA_000846525.1	4393	2	Adenoassociated virus 8	NA	Viruses; ssDNA viruses; Parvoviridae; Parvovirinae; Dependoparvovirus; Adeno-associated dependoparvovirus A; Adeno-associated virus - 4
GCA_002989755.1	141038	217	Staphylococcus phage phiSA039	NA	Viruses; Duplodnaviria; Heunggongvirae; Uroviricota; Caudoviricetes; Caudovirales; Herelleviridae; Twortvirinae; Kayvirus; unclassified Kayvirus
GCA_002743675.1	45585	65	Staphylococcus phage phiSa2wa_st1	NA	Viruses; Duplodnaviria; Heunggongvirae; Uroviricota; Caudoviricetes; Caudovirales; Siphoviridae; Triavirus; unclassified Triavirus
GCA_002814715.1	137452	70	Bubaline alphaherpesvirus 1	b6	Viruses; Duplodnaviria; Heunggongvirae; Peploviricota; Herviviricetes; Herpesvirales; Herpesviridae; Alphaherpesvirinae; Varicellovirus
GCA_002990855.1	58998	89	Escherichia phage vB_Eco_SLUR25	NA	Viruses; Duplodnaviria; Heunggongvirae; Uroviricota; Caudoviricetes; Caudovirales; Siphoviridae; Seuratvirus; unclassified Seuratvirus
GCA_900303335.1	58998	89	Escherichia phage vB_Eco_SLUR63	NA	Viruses; Duplodnaviria; Heunggongvirae; Uroviricota; Caudoviricetes; Caudovirales; Siphoviridae; Seuratvirus; unclassified Seuratvirus
GCA_900303325.1	59880	87	Escherichia phage vB_Eco_SLUR75	NA	Viruses; Duplodnaviria; Heunggongvirae; Uroviricota; Caudoviricetes; Caudovirales; Siphoviridae; Seuratvirus; unclassified Seuratvirus
GCA_900303315.1	60165	92	Escherichia phage vB_Eco_SLUR76	NA	Viruses; Duplodnaviria; Heunggongvirae; Uroviricota; Caudoviricetes; Caudovirales; Siphoviridae; Seuratvirus; unclassified Seuratvirus
GCA_900303305.1	60165	92	Escherichia phage vB_Eco_SLUR26	NA	Viruses; Duplodnaviria; Heunggongvirae; Uroviricota; Caudoviricetes; Caudovirales; Siphoviridae; Seuratvirus; unclassified Seuratvirus
GCA_004130635.1	5215	6	Rhinolophus blasii polyomavirus 2	SUB1314	Viruses; Monodnaviria; Shotokuvirae; Cossaviricota; Papovaviricetes; Sepolyvirales; Polyomaviridae; unclassified Polyomaviridae
GCA_004130655.1	5249	6	Rhinolophus simulator polyomavirus 1	SUB1317	Viruses; Monodnaviria; Shotokuvirae; Cossaviricota; Papovaviricetes; Sepolyvirales; Polyomaviridae; unclassified Polyomaviridae
GCA_004130675.1	5016	6	Rhinolophus simulator polyomavirus 2	SUB1323	Viruses; Monodnaviria; Shotokuvirae; Cossaviricota; Papovaviricetes; Sepolyvirales; Polyomaviridae; unclassified Polyomaviridae
GCA_004130695.1	5082	6	Rhinolophus simulator polyomavirus 3	SUB1325	Viruses; Monodnaviria; Shotokuvirae; Cossaviricota; Papovaviricetes; Sepolyvirales; Polyomaviridae; unclassified Polyomaviridae
GCA_013086135.1	12258	9	Colletotrichum camelliae filamentous virus 1	LT31	Viruses; unclassified viruses
GCA_002955925.1	40955	62	Staphylococcus phage UPMK_2	NA	Viruses; Duplodnaviria; Heunggongvirae; Uroviricota; Caudoviricetes; Caudovirales; Siphoviridae; Phietavirus; unclassified Phietavirus
GCA_002629645.1	46600	69	Sinorhizobium phage phi2LM21	NA	Viruses; Duplodnaviria; Heunggongvirae; Uroviricota; Caudoviricetes; Caudovirales; Siphoviridae; unclassified Siphoviridae
GCA_002629665.1	41448	59	Sinorhizobium phage phi3LM21	NA	Viruses; Duplodnaviria; Heunggongvirae; Uroviricota; Caudoviricetes; Caudovirales; Siphoviridae; unclassified Siphoviridae
GCA_003522505.1	66189	94	Pseudomonas phage BrSP1	NA	Viruses; Duplodnaviria; Heunggongvirae; Uroviricota; Caudoviricetes; Caudovirales; Myoviridae; Pbunavirus; unclassified Pbunavirus
GCA_003260735.1	52474	68	Salmonella phage vB_SenM_PA13076	NA	Viruses; Duplodnaviria; Heunggongvirae; Uroviricota; Caudoviricetes; Caudovirales; Myoviridae; Rosemountvirus; unclassified Rosemountvirus
GCA_002743695.1	94005	124	Bordetella phage vB_BbrM_PHB04	NA	Viruses; Duplodnaviria; Heunggongvirae; Uroviricota; Caudoviricetes; Caudovirales; Myoviridae; Phabquatrovirus; Bordetella virus PHB04
GCA_003389515.1	32895	59	Lactococcus phage 05601	NA	Viruses; Duplodnaviria; Heunggongvirae; Uroviricota; Caudoviricetes; Caudovirales; Siphoviridae; Skunavirus; unclassified Skunavirus
GCA_003389535.1	28721	47	Lactococcus phage 16802	NA	Viruses; Duplodnaviria; Heunggongvirae; Uroviricota; Caudoviricetes; Caudovirales; Siphoviridae; Skunavirus; unclassified Skunavirus
GCA_003389555.1	30401	56	Lactococcus phage 30804	NA	Viruses; Duplodnaviria; Heunggongvirae; Uroviricota; Caudoviricetes; Caudovirales; Siphoviridae; Skunavirus; unclassified Skunavirus
GCA_003389575.1	30989	52	Lactococcus phage 37201	NA	Viruses; Duplodnaviria; Heunggongvirae; Uroviricota; Caudoviricetes; Caudovirales; Siphoviridae; Skunavirus; unclassified Skunavirus
GCA_003389595.1	27948	54	Lactococcus phage 38503	NA	Viruses; Duplodnaviria; Heunggongvirae; Uroviricota; Caudoviricetes; Caudovirales; Siphoviridae; Skunavirus; unclassified Skunavirus
GCA_003389615.1	32458	60	Lactococcus phage 38507	NA	Viruses; Duplodnaviria; Heunggongvirae; Uroviricota; Caudoviricetes; Caudovirales; Siphoviridae; Skunavirus; unclassified Skunavirus
GCA_003389475.1	32529	55	Lactococcus phage 51701	NA	Viruses; Duplodnaviria; Heunggongvirae; Uroviricota; Caudoviricetes; Caudovirales; Siphoviridae; Skunavirus; unclassified Skunavirus
GCA_003389635.1	30926	61	Lactococcus phage 56003	NA	Viruses; Duplodnaviria; Heunggongvirae; Uroviricota; Caudoviricetes; Caudovirales; Siphoviridae; Skunavirus; unclassified Skunavirus
GCA_003389655.1	32555	57	Lactococcus phage 56301	NA	Viruses; Duplodnaviria; Heunggongvirae; Uroviricota; Caudoviricetes; Caudovirales; Siphoviridae; Skunavirus; unclassified Skunavirus
GCA_003389675.1	31819	48	Lactococcus phage 57001	NA	Viruses; Duplodnaviria; Heunggongvirae; Uroviricota; Caudoviricetes; Caudovirales; Siphoviridae; Skunavirus; unclassified Skunavirus
GCA_003389695.1	30852	53	Lactococcus phage 62601	NA	Viruses; Duplodnaviria; Heunggongvirae; Uroviricota; Caudoviricetes; Caudovirales; Siphoviridae; Skunavirus; unclassified Skunavirus
GCA_003389715.1	31014	53	Lactococcus phage 62605	NA	Viruses; Duplodnaviria; Heunggongvirae; Uroviricota; Caudoviricetes; Caudovirales; Siphoviridae; Skunavirus; unclassified Skunavirus
GCA_003389735.1	31883	50	Lactococcus phage 63302	NA	Viruses; Duplodnaviria; Heunggongvirae; Uroviricota; Caudoviricetes; Caudovirales; Siphoviridae; Skunavirus; unclassified Skunavirus
GCA_003389755.1	28917	46	Lactococcus phage 66901	NA	Viruses; Duplodnaviria; Heunggongvirae; Uroviricota; Caudoviricetes; Caudovirales; Siphoviridae; Skunavirus; unclassified Skunavirus

gca	genome.size	proteome.size	phage.name	strain	Taxonomy
GCA_003389775.1	31123	53	Lactococcus phage 79201	NA	Viruses; Duplodnaviria; Heunggongvirae; Uroviricota; Caudoviricetes; Caudovirales; Siphoviridae; Skunavirus; unclassified Skunavirus
GCA_003389795.1	31508	54	Lactococcus phage 88605	NA	Viruses; Duplodnaviria; Heunggongvirae; Uroviricota; Caudoviricetes; Caudovirales; Siphoviridae; Skunavirus; unclassified Skunavirus
GCA_003389815.1	29306	54	Lactococcus phage 96401	NA	Viruses; Duplodnaviria; Heunggongvirae; Uroviricota; Caudoviricetes; Caudovirales; Siphoviridae; Skunavirus; unclassified Skunavirus
GCA_003389835.1	26874	47	Lactococcus phage 96403	NA	Viruses; Duplodnaviria; Heunggongvirae; Uroviricota; Caudoviricetes; Caudovirales; Siphoviridae; Skunavirus; unclassified Skunavirus
GCA_003389495.1	30690	50	Lactococcus phage 96603	NA	Viruses; Duplodnaviria; Heunggongvirae; Uroviricota; Caudoviricetes; Caudovirales; Siphoviridae; Skunavirus; unclassified Skunavirus
GCA_002355025.1	8681	2	Tioga picornalike virus 1	NA	Viruses; Riboviria; Orthornavirae; Pisuviricota; Pisoniviricetes; Picornavirales; unclassified Picornavirales
GCA_002629685.1	164241	284	Bacillus phage Taffo16	NA	Viruses; Duplodnaviria; Heunggongvirae; Uroviricota; Caudoviricetes; Caudovirales; Herelleviridae; Bastillevirinae; Bequatrovirus; unclassified Bequatrovirus
GCA_013087675.1	1342	1	Eclipta yellow vein alphasatellite	AlYVAS3	Viruses; Alphasatellitidae; Geminialphasatellitinae; Gosmusatellite
GCA_013087685.1	2752	6	Malvastrum yellow vein Lahore virus	NA	Viruses; Monodnaviria; Shotokuvirae; Cressdnaviricota; Repensiviricetes; Geplafuvirales; Geminiviridae; Begomovirus
GCA_002629705.1	41712	68	Enterococcus phage phiSHEF2	NA	Viruses; Duplodnaviria; Heunggongvirae; Uroviricota; Caudoviricetes; Caudovirales; Siphoviridae; Efquatrovirus; Enterococcus virus phiSHEF2
GCA_002629725.1	41081	63	Enterococcus phage phiSHEF4	NA	Viruses; Duplodnaviria; Heunggongvirae; Uroviricota; Caudoviricetes; Caudovirales; Siphoviridae; Efquatrovirus; Enterococcus virus phiSHEF4
GCA_002629745.1	41598	69	Enterococcus phage phiSHEF5	NA	Viruses; Duplodnaviria; Heunggongvirae; Uroviricota; Caudoviricetes; Caudovirales; Siphoviridae; Efquatrovirus; Enterococcus virus phiSHEF5
GCA_002868635.1	10800	6	Japanese soilborne wheat mosaic virus	NA	Viruses; Riboviria; Orthornavirae; Kitrinoviricota; Alsuviricetes; Martellivirales; Virgaviridae; Furovirus
GCA_000865985.1	4375	2	Heterocapsa circularisquama RNA virus 01	NA	Viruses; ssRNA viruses; unassigned ssRNA viruses; Alvernaviridae; Dinornavirus
GCA_002987965.1	1350	1	Tomato leaf curl Nepal betasatellite	NA	Viruses; Tolecusatellitidae; Betasatellite
GCA_000895655.1	13807	6	Grapevine leafrollassociated virus 6	NA	Viruses; ssRNA viruses; ssRNA positive-strand viruses, no DNA stage; Closteroviridae; Ampelovirus; unclassified Ampelovirus
GCA_006458145.1	202205	265	Camelpox virus CMS	NA	Viruses; Varidnaviria; Bamfordvirae; Nucleocytoviricota; Pokkesviricetes; Chitovirales; Poxviridae; Chordopoxvirinae; Orthopoxvirus; Camelpox virus
GCA_000839105.1	205719	211	Camelpox virus M96	NA	Viruses; Varidnaviria; Bamfordvirae; Nucleocytoviricota; Pokkesviricetes; Chitovirales; Poxviridae; Chordopoxvirinae; Orthopoxvirus; Camelpox virus
GCA_002890295.1	13156	1	Rosellinia necatrix hypovirus 1	RnCa	Viruses; Riboviria; Orthornavirae; Pisuviricota; Duplopiviricetes; Durnavirales; Hypoviridae; Hypovirus; unclassified Hypovirus
GCA_000889415.1	7543	3	Pineapple bacilliform CO virus	NA	Viruses; Riboviria; Pararnavirae; Artverviricota; Revtraviricetes; Ortervirales; Caulimoviridae; Badnavirus
GCA_002830385.1	1337	1	Tomato yellow leaf curl Yunnan betasatellite	NA	Viruses; Tolecusatellitidae; Betasatellite
GCA_002629765.1	39241	57	Klebsiella phage KPP56652	NA	Viruses; Duplodnaviria; Heunggongvirae; Uroviricota; Caudoviricetes; Caudovirales; Siphoviridae; unclassified Siphoviridae
GCA_013086955.1	9210	2	Norway phlebovirus 1	NA	Viruses; Riboviria; Orthornavirae; Negarnaviricota; Polyploviricotina; Ellioviricetes; Bunyavirales; Phenuiviridae; Ixovirus; Norway ixovirus
GCA_002955935.1	72697	91	Pseudomonas phage vB_PaeP_PYO2	NA	Viruses; Duplodnaviria; Heunggongvirae; Uroviricota; Caudoviricetes; Caudovirales; Podoviridae; Litunavirus; unclassified Litunavirus
GCA_002955945.1	62874	67	Pseudomonas phage vB_PaeP_E220	NA	Viruses; Duplodnaviria; Heunggongvirae; Uroviricota; Caudoviricetes; Caudovirales; Podoviridae; Hollowayvirus; unclassified Hollowayvirus
GCA_002955955.1	72697	91	Pseudomonas phage vB_PaeP_DEV	NA	Viruses; Duplodnaviria; Heunggongvirae; Uroviricota; Caudoviricetes; Caudovirales; Podoviridae; Litunavirus; unclassified Litunavirus
GCA_002955965.1	61680	86	Pseudomonas phage vB_PaeS_S218	NA	Viruses; Duplodnaviria; Heunggongvirae; Uroviricota; Caudoviricetes; Caudovirales; Siphoviridae; Yuavirus; unclassified Yuavirus
GCA_002955975.1	66291	94	Pseudomonas phage vB_PaeM_E217	NA	Viruses; Duplodnaviria; Heunggongvirae; Uroviricota; Caudoviricetes; Caudovirales; Myoviridae; Pbunavirus; Pseudomonas virus E217
GCA_002955985.1	66789	95	Pseudomonas phage vB_PaeM_E215	NA	Viruses; Duplodnaviria; Heunggongvirae; Uroviricota; Caudoviricetes; Caudovirales; Myoviridae; Pbunavirus; Pseudomonas virus E215
GCA_000893715.1	8082	5	Sweet potato pakakuy virus	NA	Viruses; Riboviria; Pararnavirae; Artverviricota; Revtraviricetes; Ortervirales; Caulimoviridae; Badnavirus
GCA_009732115.1	545	1	Pineapple bacilliform ER virus	NA	Viruses; Riboviria; Pararnavirae; Artverviricota; Revtraviricetes; Ortervirales; Caulimoviridae; Badnavirus
GCA_002816635.1	8530	1	Boone cardiovirus 1	NA	Viruses; Riboviria; Orthornavirae; Pisuviricota; Pisoniviricetes; Picornavirales; Picornaviridae; Cardiovirus; Cardiovirus C
GCA_004131205.1	166748	75	Eptesicus fuscus gammaherpesvirus	NA	Viruses; Duplodnaviria; Heunggongvirae; Peploviricota; Herviviricetes; Herpesvirales; Herpesviridae; Gammaherpesvirinae; unclassified Gammaherpesvirinae
GCA_002990055.1	10567	22	Methanosarcina spherical virus	NA	Viruses; Varidnaviria; Bamfordvirae; Preplasmiviricota; Tectiliviricetes; Kalamavirales; Tectiviridae; unclassified Tectiviridae

gca	genome.size	proteome.size	phage.name	strain	Taxonomy
GCA_004131025.1	165695	178	Cherax quadricarinatus iridovirus	CQIVCN01	Viruses; Varidnaviria; Bamfordvirae; Nucleocytoviricota; Megaviricetes; Pimascovirales; Iridoviridae; unclassified Iridoviridae
GCA_000890335.1	5086	5	Chimpanzee polyomavirus Bob	NA	Viruses; Monodnaviria; Shotokuvirae; Cossaviricota; Papovaviricetes; Sepolyvirales; Polyomaviridae; Alphapolyomavirus; Pan troglodytes polyomavirus 1
GCA_000903955.1	4914	5	Otomops polyomavirus KY156	NA	Viruses; Monodnaviria; Shotokuvirae; Cossaviricota; Papovaviricetes; Sepolyvirales; Polyomaviridae; Alphapolyomavirus; Otomops martiensseni polyomavirus 2
GCA_000903915.1	5176	5	Otomops polyomavirus KY157	NA	Viruses; Monodnaviria; Shotokuvirae; Cossaviricota; Papovaviricetes; Sepolyvirales; Polyomaviridae; Alphapolyomavirus; Otomops martiensseni polyomavirus 1
GCA_002743715.1	93296	172	Pseudomonas phage YS35	NA	Viruses; Duplodnaviria; Heunggongvirae; Uroviricota; Caudoviricetes; Caudovirales; Myoviridae; Pakpunavirus; unclassified Pakpunavirus
GCA_003014395.1	38421	50	Lactobacillus phage T25	NA	Viruses; Duplodnaviria; Heunggongvirae; Uroviricota; Caudoviricetes; Caudovirales; Siphoviridae; unclassified Siphoviridae
GCA_013693975.1	36172	51	Oenococcus phage vB_OeS_unk162	NA	Viruses; unclassified bacterial viruses
GCA_009732135.1	11858	3	Achiote virus	NA	Viruses; Riboviria; Orthornavirae; Negarnaviricota; Polyploviricotina; Ellioviricetes; Bunyavirales; Peribunyaviridae; Orthobunyavirus; Trivittatus orthobunyavirus
GCA_009732155.1	12430	3	Infirmatus virus	NA	Viruses; Riboviria; Orthornavirae; Negarnaviricota; Polyploviricotina; Ellioviricetes; Bunyavirales; Peribunyaviridae; Orthobunyavirus; Trivittatus orthobunyavirus
GCA_002629785.1	37316	66	Caldibacillus phage CBP1	NA	Viruses; Duplodnaviria; Heunggongvirae; Uroviricota; Caudoviricetes; Caudovirales; Siphoviridae; unclassified Siphoviridae
GCA_009732175.1	13258	4	Pueblo Viejo virus	NA	Viruses; Riboviria; Orthornavirae; Negarnaviricota; Polyploviricotina; Ellioviricetes; Bunyavirales; Peribunyaviridae; Orthobunyavirus; Gamboa orthobunyavirus
GCA_004114855.1	10218	2	Peach leaf pittingassociated virus	NA	Viruses; Riboviria; Orthornavirae; Pisuviricota; Pisoniviricetes; Picornavirales; Secoviridae; Comovirinae; Fabavirus; unclassified Fabavirus
GCA_000918035.1	1790	2	Human associated cyclovirus 10	7078A	Viruses; Monodnaviria; Shotokuvirae; Cressdnaviricota; Arfiviricetes; Cirlivirales; Circoviridae; Cyclovirus
GCA_000914275.1	8091	8	Black medic leaf roll virus	NA	Viruses; ssDNA viruses; Nanoviridae; Nanovirus
GCA_013086105.1	24938	10	Tai Forest reovirus	NA	Viruses; Riboviria; Orthornavirae; Duplornaviricota; Resentoviricetes; Reovirales; Reoviridae; unclassified Reoviridae
GCA_006425395.1	30134	30	Eidolon helvum adenovirus	NA	Viruses; Varidnaviria; Bamfordvirae; Preplasmiviricota; Tectiliviricetes; Rowavirales; Adenoviridae; Mastadenovirus; unclassified Mastadenovirus
GCA_004788555.1	165809	170	Shrimp hemocyte iridescent virus	NA	Viruses; Varidnaviria; Bamfordvirae; Nucleocytoviricota; Megaviricetes; Pimascovirales; Iridoviridae; Betairidovirinae; Decapodiridovirus; Decapod iridescent virus 1
GCA_003555485.1	158385	239	Bacillus phage BC01	NA	Viruses; Duplodnaviria; Heunggongvirae; Uroviricota; Caudoviricetes; Caudovirales; Herelleviridae; Bastillevirinae; Tsarbombavirus; unclassified Tsarbombavirus
GCA_002629805.1	44708	58	Escherichia phage Ayreon	NA	Viruses; Duplodnaviria; Heunggongvirae; Uroviricota; Caudoviricetes; Caudovirales; Siphoviridae; unclassified Siphoviridae
GCA_002629825.1	50072	83	Mycobacterium phage OKCentral2016	NA	Viruses; Duplodnaviria; Heunggongvirae; Uroviricota; Caudoviricetes; Caudovirales; Siphoviridae; Fromanvirus; unclassified Fromanvirus
GCA_013087695.1	11624	5	Ohlsdorf virus	Germany2012Occantans	Viruses; Riboviria; Orthornavirae; Negarnaviricota; Haploviricotina; Monjiviricetes; Mononegavirales; Rhabdoviridae; Ohlsrhavirus; Ohlsdorf ohlsrhavirus
GCA_002830365.1	1334	1	Tomato yellow leaf curl Shandong betasatellite	NA	Viruses; Tolecusatellitidae; Betasatellite
GCA_000843045.1	16784	21	Staphylococcus virus 44AHJD	NA	Viruses; Duplodnaviria; Heunggongvirae; Uroviricota; Caudoviricetes; Caudovirales; Podoviridae; Rakietenvirinae; Rosenblumvirus
GCA_000842185.1	18227	22	Staphylococcus phage P68	NA	Viruses; dsDNA viruses, no RNA stage; Caudovirales; Podoviridae; Picovirinae; P68virus; unclassified P68virus
GCA_004131005.1	8838	2	Hedgehog dicipivirus	hedgehogH142015HUN	Viruses; Riboviria; Orthornavirae; Pisuviricota; Pisoniviricetes; Picornavirales; Picornaviridae; Dicipivirus; Cadicivirus B
GCA_002743735.1	49372	79	Streptomyces phage Amethyst	NA	Viruses; Duplodnaviria; Heunggongvirae; Uroviricota; Caudoviricetes; Caudovirales; Siphoviridae; Arquatrovirinae; Arequatrovirus; unclassified Arequatrovirus
GCA_002743755.1	50602	83	Streptomyces phage Daudau	NA	Viruses; Duplodnaviria; Heunggongvirae; Uroviricota; Caudoviricetes; Caudovirales; Siphoviridae; Arquatrovirinae; Arequatrovirus; unclassified Arequatrovirus
GCA_002743775.1	50483	79	Streptomyces phage Diane	NA	Viruses; Duplodnaviria; Heunggongvirae; Uroviricota; Caudoviricetes; Caudovirales; Siphoviridae; Arquatrovirinae; Arequatrovirus; unclassified Arequatrovirus
GCA_002743795.1	50837	78	Streptomyces phage SqueakyClean	NA	Viruses; Duplodnaviria; Heunggongvirae; Uroviricota; Caudoviricetes; Caudovirales; Siphoviridae; Arquatrovirinae; Arequatrovirus; unclassified Arequatrovirus
GCA_002743815.1	50574	81	Streptomyces phage Tefunt	NA	Viruses; Duplodnaviria; Heunggongvirae; Uroviricota; Caudoviricetes; Caudovirales; Siphoviridae; Arquatrovirinae; Arequatrovirus; unclassified Arequatrovirus
GCA_002743835.1	145162	100	Lactobacillus phage LpeD	NA	Viruses; Duplodnaviria; Heunggongvirae; Uroviricota; Caudoviricetes; Caudovirales; Herelleviridae; unclassified Herelleviridae
GCA_002743855.1	42518	59	Klebsiella phage vB_KpnS_IME279	NA	Viruses; Duplodnaviria; Heunggongvirae; Uroviricota; Caudoviricetes; Caudovirales; Podoviridae; Sortsnevirus; Klebsiella virus IME279
GCA_002743875.1	47760	75	Klebsiella phage vB_KpnM_KpV79	NA	Viruses; Duplodnaviria; Heunggongvirae; Uroviricota; Caudoviricetes; Caudovirales; Myoviridae; Jedunavirus; Klebsiella virus KpV52
GCA_002955995.1	44194	63	Pseudomonas phage SL4	NA	Viruses; Duplodnaviria; Heunggongvirae; Uroviricota; Caudoviricetes; Caudovirales; Podoviridae; Bruynoghevirus; unclassified Bruynoghevirus

gca	genome.size	proteome.size	phage.name	strain	Taxonomy
GCA_002956005.1	65847	90	Pseudomonas phage SL1	NA	Viruses; Duplodnaviria; Heunggongvirae; Uroviricota; Caudoviricetes; Caudovirales; Myoviridae; Pbunavirus; unclassified Pbunavirus
GCA_003601495.1	155958	228	Bacillus phage BSP9	NA	Viruses; Duplodnaviria; Heunggongvirae; Uroviricota; Caudoviricetes; Caudovirales; Herelleviridae; Bastillevirinae; Nitunavirus; unclassified Nitunavirus
GCA_002743915.1	42168	54	Marinobacter phage PS3	NA	Viruses; Duplodnaviria; Heunggongvirae; Uroviricota; Caudoviricetes; Caudovirales; Siphoviridae; unclassified Siphoviridae
GCA_002743935.1	58226	80	Marinobacter phage PS6	NA	Viruses; Duplodnaviria; Heunggongvirae; Uroviricota; Caudoviricetes; Caudovirales; Siphoviridae; unclassified Siphoviridae
GCA_002743955.1	70373	115	Mycobacterium phage Kumao	NA	Viruses; Duplodnaviria; Heunggongvirae; Uroviricota; Caudoviricetes; Caudovirales; Siphoviridae; unclassified Siphoviridae
GCA_002956015.1	279696	355	Pseudomonas phage SL2	NA	Viruses; Duplodnaviria; Heunggongvirae; Uroviricota; Caudoviricetes; Caudovirales; Myoviridae; Phikzvirus; Pseudomonas virus SL2
GCA_002743975.1	147659	231	Escherichia phage vB_EcoM_PHB05	NA	Viruses; Duplodnaviria; Heunggongvirae; Uroviricota; Caudoviricetes; Caudovirales; Myoviridae; unclassified Myoviridae
GCA_002743995.1	49494	74	Pseudomonas phage IME180	NA	Viruses; Duplodnaviria; Heunggongvirae; Uroviricota; Caudoviricetes; Caudovirales; Podoviridae; unclassified Podoviridae
GCA_003029625.1	97330	168	Pseudomonas phage PPSC2	NA	Viruses; Duplodnaviria; Heunggongvirae; Uroviricota; Caudoviricetes; Caudovirales; Myoviridae; Otagovirus; unclassified Otagovirus
GCA_003288515.1	39628	43	Ralstonia phage RPSC1	NA	Viruses; Duplodnaviria; Heunggongvirae; Uroviricota; Caudoviricetes; Caudovirales; Autographiviridae; Stompelvirus; Ralstonia virus RPSC1
GCA_002744015.1	57385	71	Streptomyces phage Abt2graduatex2	NA	Viruses; Duplodnaviria; Heunggongvirae; Uroviricota; Caudoviricetes; Caudovirales; Siphoviridae; Woodruffvirus; unclassified Woodruffvirus
GCA_002744035.1	43060	56	Streptomyces phage Scap1	NA	Viruses; Duplodnaviria; Heunggongvirae; Uroviricota; Caudoviricetes; Caudovirales; Siphoviridae; Scapunavirus; Streptomyces virus Scap1
GCA_003423065.1	48288	83	Leptospira phage LE3	NA	Viruses; Duplodnaviria; Heunggongvirae; Uroviricota; Caudoviricetes; Caudovirales; Myoviridae; unclassified Myoviridae
GCA_003423085.1	47866	81	Leptospira phage LE4	NA	Viruses; Duplodnaviria; Heunggongvirae; Uroviricota; Caudoviricetes; Caudovirales; Myoviridae; unclassified Myoviridae
GCA_002956025.1	50753	84	Lysinibacillus phage vB_LspM01	NA	Viruses; Duplodnaviria; Heunggongvirae; Uroviricota; Caudoviricetes; Caudovirales; Myoviridae; unclassified Myoviridae
GCA_002744075.1	159323	264	Salmonella phage Melville	NA	Viruses; Duplodnaviria; Heunggongvirae; Uroviricota; Caudoviricetes; Caudovirales; Myoviridae; Tevenvirinae; Gelderlandvirus; Salmonella virus Melville
GCA_003846545.1	2213	3	Gopherus associated genomovirus 1	NA	Viruses; Monodnaviria; Shotokuvirae; Cressdnaviricota; Repensiviricetes; Geplafuvirales; Genomoviridae; unclassified Genomoviridae
GCA_003846525.1	2270	2	Gopherus associated circular DNA virus 1	NA	Viruses; unclassified viruses; unclassified DNA viruses; unclassified ssDNA viruses
GCA_002744095.1	44260	65	Lactobacillus phage LJ	NA	Viruses; Duplodnaviria; Heunggongvirae; Uroviricota; Caudoviricetes; Caudovirales; Siphoviridae; unclassified Siphoviridae
GCA_003441355.1	43237	46	Vibrio phage vB_VpaP_KF1	NA	Viruses; Duplodnaviria; Heunggongvirae; Uroviricota; Caudoviricetes; Caudovirales; Autographiviridae; Maculvirus; Vibrio virus KF1
GCA_003441375.1	43571	48	Vibrio phage vB_VpaP_KF2	NA	Viruses; Duplodnaviria; Heunggongvirae; Uroviricota; Caudoviricetes; Caudovirales; Autographiviridae; Maculvirus; Vibrio virus KF2
GCA_003441395.1	75955	97	Vibrio phage vB_VpaS_KF3	NA	Viruses; Duplodnaviria; Heunggongvirae; Uroviricota; Caudoviricetes; Caudovirales; Siphoviridae; Mardecavirus; unclassified Mardecavirus
GCA_003441415.1	75501	97	Vibrio phage vB_VpaS_KF4	NA	Viruses; Duplodnaviria; Heunggongvirae; Uroviricota; Caudoviricetes; Caudovirales; Siphoviridae; Mardecavirus; unclassified Mardecavirus
GCA_003441435.1	76402	98	Vibrio phage vB_VpaS_KF5	NA	Viruses; Duplodnaviria; Heunggongvirae; Uroviricota; Caudoviricetes; Caudovirales; Siphoviridae; Mardecavirus; unclassified Mardecavirus
GCA_003441455.1	75904	99	Vibrio phage vB_VpaS_KF6	NA	Viruses; Duplodnaviria; Heunggongvirae; Uroviricota; Caudoviricetes; Caudovirales; Siphoviridae; Mardecavirus; unclassified Mardecavirus
GCA_002744115.1	35306	46	Pseudomonas phage VW6B	NA	Viruses; Duplodnaviria; Heunggongvirae; Uroviricota; Caudoviricetes; Caudovirales; Siphoviridae; unclassified Siphoviridae
GCA_002744135.1	37917	52	Pseudomonas phage VW6S	NA	Viruses; Duplodnaviria; Heunggongvirae; Uroviricota; Caudoviricetes; Caudovirales; Siphoviridae; unclassified Siphoviridae
GCA_002956035.1	144959	267	Pectobacterium phage DU_PP_I	NA	Viruses; Duplodnaviria; Heunggongvirae; Uroviricota; Caudoviricetes; Caudovirales; Myoviridae; Vequintavirinae; Certrevirus; unclassified Certrevirus
GCA_002956045.1	40142	42	Pectobacterium phage DU_PP_II	NA	Viruses; Duplodnaviria; Heunggongvirae; Uroviricota; Caudoviricetes; Caudovirales; Autographiviridae; Studiervirinae; Unyawovirus; Pectobacterium virus DUPPII
GCA_002956055.1	11504	7	Pectobacterium phage DU_PP_III	NA	Viruses; Duplodnaviria; Heunggongvirae; Uroviricota; Caudoviricetes; Caudovirales; Podoviridae; unclassified Podoviridae
GCA_002956065.1	145233	268	Pectobacterium phage DU_PP_IV	NA	Viruses; Duplodnaviria; Heunggongvirae; Uroviricota; Caudoviricetes; Caudovirales; Myoviridae; Vequintavirinae; Certrevirus; unclassified Certrevirus
GCA_002956075.1	106185	127	Pectobacterium phage DU_PP_V	NA	Viruses; Duplodnaviria; Heunggongvirae; Uroviricota; Caudoviricetes; Caudovirales; Demerecviridae; Mccorquodalevirinae; Hongcheonvirus; Pectobacterium virus DUPPV
GCA_002956085.1	40657	39	Ralstonia phage DU_RP_I	NA	Viruses; Duplodnaviria; Heunggongvirae; Uroviricota; Caudoviricetes; Caudovirales; Autographiviridae; Gyeongsanvirus; Ralstonia virus DURPI
GCA_003012575.1	34379	38	Streptococcus phage 16B8	NA	Viruses; Duplodnaviria; Heunggongvirae; Uroviricota; Caudoviricetes; Caudovirales; Siphoviridae; Moineauvirus; unclassified Moineauvirus

gca	genome.size	proteome.size	phage.name	strain	Taxonomy
GCA_003012585.1	36438	40	Streptococcus phage 31B4	NA	Viruses; Duplodnaviria; Heunggongvirae; Uroviricota; Caudoviricetes; Caudovirales; Siphoviridae; Moineauvirus; unclassified Moineauvirus
GCA_003012545.1	35936	44	Streptococcus phage 7A5	NA	Viruses; Duplodnaviria; Heunggongvirae; Uroviricota; Caudoviricetes; Caudovirales; Siphoviridae; Moineauvirus; unclassified Moineauvirus
GCA_003012525.1	35141	41	Streptococcus phage 7T	NA	Viruses; Duplodnaviria; Heunggongvirae; Uroviricota; Caudoviricetes; Caudovirales; Siphoviridae; Moineauvirus; unclassified Moineauvirus
GCA_003012555.1	35604	41	Streptococcus phage 9A	NA	Viruses; Duplodnaviria; Heunggongvirae; Uroviricota; Caudoviricetes; Caudovirales; Siphoviridae; Moineauvirus; unclassified Moineauvirus
GCA_003012565.1	34636	41	Streptococcus phage 9B4	NA	Viruses; Duplodnaviria; Heunggongvirae; Uroviricota; Caudoviricetes; Caudovirales; Siphoviridae; Moineauvirus; unclassified Moineauvirus
GCA_003012535.1	35481	44	Streptococcus phage A0	NA	Viruses; Duplodnaviria; Heunggongvirae; Uroviricota; Caudoviricetes; Caudovirales; Siphoviridae; Moineauvirus; unclassified Moineauvirus
GCA_003012595.1	36773	47	Streptococcus phage B0	NA	Viruses; Duplodnaviria; Heunggongvirae; Uroviricota; Caudoviricetes; Caudovirales; Siphoviridae; Moineauvirus; unclassified Moineauvirus
GCA_003012605.1	35636	42	Streptococcus phage B5	NA	Viruses; Duplodnaviria; Heunggongvirae; Uroviricota; Caudoviricetes; Caudovirales; Siphoviridae; Moineauvirus; unclassified Moineauvirus
GCA_003012615.1	34718	42	Streptococcus phage C0	NA	Viruses; Duplodnaviria; Heunggongvirae; Uroviricota; Caudoviricetes; Caudovirales; Siphoviridae; Moineauvirus; unclassified Moineauvirus
GCA_003012625.1	36884	44	Streptococcus phage L5A1	NA	Viruses; Duplodnaviria; Heunggongvirae; Uroviricota; Caudoviricetes; Caudovirales; Siphoviridae; Moineauvirus; unclassified Moineauvirus
GCA_003012635.1	34328	44	Streptococcus phage M19	NA	Viruses; Duplodnaviria; Heunggongvirae; Uroviricota; Caudoviricetes; Caudovirales; Siphoviridae; Moineauvirus; unclassified Moineauvirus
GCA_003012645.1	34059	41	Streptococcus phage MM25	NA	Viruses; Duplodnaviria; Heunggongvirae; Uroviricota; Caudoviricetes; Caudovirales; Siphoviridae; Moineauvirus; unclassified Moineauvirus
GCA_003012655.1	34652	39	Streptococcus phage R1	NA	Viruses; Duplodnaviria; Heunggongvirae; Uroviricota; Caudoviricetes; Caudovirales; Siphoviridae; Moineauvirus; unclassified Moineauvirus
GCA_003012665.1	35283	41	Streptococcus phage STP1	NA	Viruses; Duplodnaviria; Heunggongvirae; Uroviricota; Caudoviricetes; Caudovirales; Siphoviridae; Moineauvirus; unclassified Moineauvirus
GCA_003012675.1	35286	44	Streptococcus phage STP2	NA	Viruses; Duplodnaviria; Heunggongvirae; Uroviricota; Caudoviricetes; Caudovirales; Siphoviridae; Moineauvirus; unclassified Moineauvirus
GCA_003012685.1	34698	40	Streptococcus phage V2	NA	Viruses; Duplodnaviria; Heunggongvirae; Uroviricota; Caudoviricetes; Caudovirales; Siphoviridae; Moineauvirus; unclassified Moineauvirus
GCA_002744155.1	65869	95	Gordonia phage Kabluna	NA	Viruses; Duplodnaviria; Heunggongvirae; Uroviricota; Caudoviricetes; Caudovirales; Siphoviridae; unclassified Siphoviridae
GCA_002744185.1	60237	85	Gordonia phage Lennon	NA	Viruses; Duplodnaviria; Heunggongvirae; Uroviricota; Caudoviricetes; Caudovirales; Siphoviridae; Vividuovirus; Gordonia virus Lennon
GCA_002744215.1	93201	179	Gordonia phage Lozinak	NA	Viruses; Duplodnaviria; Heunggongvirae; Uroviricota; Caudoviricetes; Caudovirales; Siphoviridae; Smoothievirus; unclassified Smoothievirus
GCA_002744255.1	50948	83	Gordonia phage Lysidious	NA	Viruses; Duplodnaviria; Heunggongvirae; Uroviricota; Caudoviricetes; Caudovirales; Siphoviridae; unclassified Siphoviridae
GCA_002956095.1	66251	90	Gordonia phage Patio	NA	Viruses; Duplodnaviria; Heunggongvirae; Uroviricota; Caudoviricetes; Caudovirales; Siphoviridae; unclassified Siphoviridae
GCA_002956105.1	92546	179	Gordonia phage Toniann	NA	Viruses; Duplodnaviria; Heunggongvirae; Uroviricota; Caudoviricetes; Caudovirales; Siphoviridae; Smoothievirus; unclassified Smoothievirus
GCA_002744295.1	53545	98	Mycobacterium phage Anselm	NA	Viruses; Duplodnaviria; Heunggongvirae; Uroviricota; Caudoviricetes; Caudovirales; Siphoviridae; Fromanvirus; unclassified Fromanvirus
GCA_002744315.1	46732	78	Mycobacterium phage Arib1	NA	Viruses; Duplodnaviria; Heunggongvirae; Uroviricota; Caudoviricetes; Caudovirales; Siphoviridae; Pclasvirinae; Fishburnevirus; unclassified Fishburnevirus
GCA_002744335.1	155205	235	Mycobacterium phage Audrick	NA	Viruses; Duplodnaviria; Heunggongvirae; Uroviricota; Caudoviricetes; Caudovirales; Myoviridae; Bixzunavirus; unclassified Bixzunavirus
GCA_002744355.1	154061	225	Mycobacterium phage BeanWater	NA	Viruses; Duplodnaviria; Heunggongvirae; Uroviricota; Caudoviricetes; Caudovirales; Myoviridae; Bixzunavirus; unclassified Bixzunavirus
GCA_002744375.1	156514	237	Mycobacterium phage Bigswole	NA	Viruses; Duplodnaviria; Heunggongvirae; Uroviricota; Caudoviricetes; Caudovirales; Myoviridae; Bixzunavirus; unclassified Bixzunavirus
GCA_002744395.1	51809	92	Mycobacterium phage Cerulean	NA	Viruses; Duplodnaviria; Heunggongvirae; Uroviricota; Caudoviricetes; Caudovirales; Siphoviridae; Fromanvirus; unclassified Fromanvirus
GCA_002744415.1	41903	63	Mycobacterium phage Chance64	NA	Viruses; Duplodnaviria; Heunggongvirae; Uroviricota; Caudoviricetes; Caudovirales; Siphoviridae; Liefievirus; unclassified Liefievirus
GCA_002744435.1	51575	84	Mycobacterium phage Cindaradix	NA	Viruses; Duplodnaviria; Heunggongvirae; Uroviricota; Caudoviricetes; Caudovirales; Siphoviridae; Fromanvirus; unclassified Fromanvirus
GCA_002744455.1	56827	103	Mycobacterium phage DaWorst	NA	Viruses; Duplodnaviria; Heunggongvirae; Uroviricota; Caudoviricetes; Caudovirales; Siphoviridae; Cheoctovirus; unclassified Cheoctovirus
GCA_002744475.1	155034	238	Mycobacterium phage Daffodil	NA	Viruses; Duplodnaviria; Heunggongvirae; Uroviricota; Caudoviricetes; Caudovirales; Myoviridae; Bixzunavirus; unclassified Bixzunavirus
GCA_002744495.1	52850	95	Mycobacterium phage Dalmatian	NA	Viruses; Duplodnaviria; Heunggongvirae; Uroviricota; Caudoviricetes; Caudovirales; Siphoviridae; Fromanvirus; unclassified Fromanvirus
GCA_002744525.1	57437	117	Mycobacterium phage Demsculpinboyz	NA	Viruses; Duplodnaviria; Heunggongvirae; Uroviricota; Caudoviricetes; Caudovirales; Siphoviridae; Cheoctovirus; unclassified Cheoctovirus

gca	genome.size	proteome.size	phage.name	strain	Taxonomy
GCA_002744565.1	67776	98	Mycobacterium phage Dingo	NA	Viruses; Duplodnaviria; Heunggongvirae; Uroviricota; Caudoviricetes; Caudovirales; Siphoviridae; Bclasvirinae; Pegunavirus; unclassified Pegunavirus
GCA_002744615.1	112285	241	Mycobacterium phage DmpstrDiver	NA	Viruses; Duplodnaviria; Heunggongvirae; Uroviricota; Caudoviricetes; Caudovirales; Siphoviridae; Omegavirus; unclassified Omegavirus
GCA_002744635.1	76217	148	Mycobacterium phage FireRed	NA	Viruses; Duplodnaviria; Heunggongvirae; Uroviricota; Caudoviricetes; Caudovirales; Siphoviridae; Kostyavirus; unclassified Kostyavirus
GCA_002744655.1	68171	96	Mycobacterium phage Horchata	NA	Viruses; Duplodnaviria; Heunggongvirae; Uroviricota; Caudoviricetes; Caudovirales; Siphoviridae; Bclasvirinae; Pegunavirus; unclassified Pegunavirus
GCA_002744675.1	51017	91	Mycobacterium phage ILeeKay	NA	Viruses; Duplodnaviria; Heunggongvirae; Uroviricota; Caudoviricetes; Caudovirales; Siphoviridae; Fromanvirus; unclassified Fromanvirus
GCA_002744695.1	51374	90	Mycobacterium phage JetBlade	NA	Viruses; Duplodnaviria; Heunggongvirae; Uroviricota; Caudoviricetes; Caudovirales; Siphoviridae; Fromanvirus; unclassified Fromanvirus
GCA_002744725.1	68953	100	Mycobacterium phage Kailash	NA	Viruses; Duplodnaviria; Heunggongvirae; Uroviricota; Caudoviricetes; Caudovirales; Siphoviridae; Bclasvirinae; Pegunavirus; unclassified Pegunavirus
GCA_002744775.1	112329	248	Mycobacterium phage Klein	NA	Viruses; Duplodnaviria; Heunggongvirae; Uroviricota; Caudoviricetes; Caudovirales; Siphoviridae; Omegavirus; unclassified Omegavirus
GCA_002744815.1	155759	230	Mycobacterium phage Koguma	NA	Viruses; Duplodnaviria; Heunggongvirae; Uroviricota; Caudoviricetes; Caudovirales; Myoviridae; Bixzunavirus; unclassified Bixzunavirus
GCA_002744835.1	53746	85	Mycobacterium phage LilHazelnut	NA	Viruses; Duplodnaviria; Heunggongvirae; Uroviricota; Caudoviricetes; Caudovirales; Siphoviridae; Gilesvirus; unclassified Gilesvirus
GCA_002744855.1	56167	78	Mycobacterium phage LilPharaoh	NA	Viruses; Duplodnaviria; Heunggongvirae; Uroviricota; Caudoviricetes; Caudovirales; Siphoviridae; Timquatrovirus; unclassified Timquatrovirus
GCA_002744875.1	68804	102	Mycobacterium phage Longacauda	NA	Viruses; Duplodnaviria; Heunggongvirae; Uroviricota; Caudoviricetes; Caudovirales; Siphoviridae; Bclasvirinae; Pegunavirus; unclassified Pegunavirus
GCA_002956115.1	75808	145	Mycobacterium phage MISSy	NA	Viruses; Duplodnaviria; Heunggongvirae; Uroviricota; Caudoviricetes; Caudovirales; Siphoviridae; Kostyavirus; unclassified Kostyavirus
GCA_002744895.1	67768	96	Mycobacterium phage Mikota	NA	Viruses; Duplodnaviria; Heunggongvirae; Uroviricota; Caudoviricetes; Caudovirales; Siphoviridae; Bclasvirinae; Pegunavirus; unclassified Pegunavirus
GCA_002744915.1	77053	147	Mycobacterium phage Murica	NA	Viruses; Duplodnaviria; Heunggongvirae; Uroviricota; Caudoviricetes; Caudovirales; Siphoviridae; Kostyavirus; unclassified Kostyavirus
GCA_002745355.1	56125	100	Mycobacterium phage Phasih	NA	Viruses; Duplodnaviria; Heunggongvirae; Uroviricota; Caudoviricetes; Caudovirales; Siphoviridae; Cheoctovirus; unclassified Cheoctovirus
GCA_002744935.1	154874	229	Mycobacterium phage Phox	NA	Viruses; Duplodnaviria; Heunggongvirae; Uroviricota; Caudoviricetes; Caudovirales; Myoviridae; Bixzunavirus; unclassified Bixzunavirus
GCA_002744955.1	68375	99	Mycobacterium phage Phunky	NA	Viruses; Duplodnaviria; Heunggongvirae; Uroviricota; Caudoviricetes; Caudovirales; Siphoviridae; Bclasvirinae; Pegunavirus; unclassified Pegunavirus
GCA_002744975.1	73495	139	Mycobacterium phage Sassay	NA	Viruses; Duplodnaviria; Heunggongvirae; Uroviricota; Caudoviricetes; Caudovirales; Siphoviridae; Kostyavirus; unclassified Kostyavirus
GCA_002744995.1	67880	96	Mycobacterium phage Sheila	NA	Viruses; Duplodnaviria; Heunggongvirae; Uroviricota; Caudoviricetes; Caudovirales; Siphoviridae; Bclasvirinae; Pegunavirus; unclassified Pegunavirus
GCA_002745015.1	53051	95	Mycobacterium phage SnapTap	NA	Viruses; Duplodnaviria; Heunggongvirae; Uroviricota; Caudoviricetes; Caudovirales; Siphoviridae; Fromanvirus; unclassified Fromanvirus
GCA_002956125.1	59873	94	Mycobacterium phage Sulley	NA	Viruses; Duplodnaviria; Heunggongvirae; Uroviricota; Caudoviricetes; Caudovirales; Siphoviridae; Timquatrovirus; unclassified Timquatrovirus
GCA_002956135.1	109799	239	Mycobacterium phage Superphikiman	NA	Viruses; Duplodnaviria; Heunggongvirae; Uroviricota; Caudoviricetes; Caudovirales; Siphoviridae; Omegavirus; unclassified Omegavirus
GCA_002745035.1	76169	147	Mycobacterium phage Terminus	NA	Viruses; Duplodnaviria; Heunggongvirae; Uroviricota; Caudoviricetes; Caudovirales; Siphoviridae; Kostyavirus; unclassified Kostyavirus
GCA_002745055.1	52350	93	Mycobacterium phage TipsytheTRex	NA	Viruses; Duplodnaviria; Heunggongvirae; Uroviricota; Caudoviricetes; Caudovirales; Siphoviridae; Fromanvirus; unclassified Fromanvirus
GCA_002745075.1	53043	97	Mycobacterium phage Updawg	NA	Viruses; Duplodnaviria; Heunggongvirae; Uroviricota; Caudoviricetes; Caudovirales; Siphoviridae; Fromanvirus; unclassified Fromanvirus
GCA_002745095.1	68682	103	Mycobacterium phage Virapocalypse	NA	Viruses; Duplodnaviria; Heunggongvirae; Uroviricota; Caudoviricetes; Caudovirales; Siphoviridae; Bclasvirinae; Pegunavirus; unclassified Pegunavirus
GCA_002745115.1	74576	142	Mycobacterium phage Willez	NA	Viruses; Duplodnaviria; Heunggongvirae; Uroviricota; Caudoviricetes; Caudovirales; Siphoviridae; Kostyavirus; unclassified Kostyavirus
GCA_002745135.1	73697	139	Mycobacterium phage YassJohnny	NA	Viruses; Duplodnaviria; Heunggongvirae; Uroviricota; Caudoviricetes; Caudovirales; Siphoviridae; Kostyavirus; unclassified Kostyavirus
GCA_002745155.1	30112	48	Propionibacterium phage Aquarius	NA	Viruses; Duplodnaviria; Heunggongvirae; Uroviricota; Caudoviricetes; Caudovirales; Siphoviridae; Pahexavirus; unclassified Pahexavirus
GCA_002745175.1	29742	45	Propionibacterium phage DrParker	NA	Viruses; Duplodnaviria; Heunggongvirae; Uroviricota; Caudoviricetes; Caudovirales; Siphoviridae; Pahexavirus; unclassified Pahexavirus
GCA_002745195.1	29451	44	Propionibacterium phage Leviosa	NA	Viruses; Duplodnaviria; Heunggongvirae; Uroviricota; Caudoviricetes; Caudovirales; Siphoviridae; Pahexavirus; unclassified Pahexavirus
GCA_002745215.1	29041	45	Propionibacterium phage LilBandit	NA	Viruses; Duplodnaviria; Heunggongvirae; Uroviricota; Caudoviricetes; Caudovirales; Siphoviridae; Pahexavirus; unclassified Pahexavirus
GCA_002745235.1	29223	44	Propionibacterium phage MEAK	NA	Viruses; Duplodnaviria; Heunggongvirae; Uroviricota; Caudoviricetes; Caudovirales; Siphoviridae; Pahexavirus; unclassified Pahexavirus

gca	genome.size	proteome.size	phage.name	strain	Taxonomy
GCA_002745255.1	29217	45	Propionibacterium phage Supernova	NA	Viruses; Duplodnaviria; Heunggongvirae; Uroviricota; Caudoviricetes; Caudovirales; Siphoviridae; Pahexavirus; unclassified Pahexavirus
GCA_003601515.1	40060	83	Escherichia phage IMM002	NA	Viruses; Duplodnaviria; Heunggongvirae; Uroviricota; Caudoviricetes; Caudovirales; Autographiviridae; Studiervirinae; Kayfunavirus; Escherichia virus IMM002
GCA_003173815.1	384670	592	Xanthomonas phage XacN1	NA	Viruses; Duplodnaviria; Heunggongvirae; Uroviricota; Caudoviricetes; Caudovirales; Myoviridae; unclassified Myoviridae
GCA_006452015.1	170681	185	Eastern grey kangaroopox virus	NSW	Viruses; Varidnaviria; Bamfordvirae; Nucleocytoviricota; Pokkesviricetes; Chitovirales; Poxviridae; Chordopoxvirinae; Macropopoxvirus; Eastern kangaroopox virus
GCA_000887635.1	17858	11	Great Island virus	NA	Viruses; dsRNA viruses; Reoviridae; Sedoreovirinae; Orbivirus
GCA_000858425.1	42519	48	Pseudomonas phage phiKMV	NA	Viruses; dsDNA viruses, no RNA stage; Caudovirales; Podoviridae; Autographivirinae; Phikmvvirus
GCA_004117755.1	3694	2	Medicago sativa alphapartitivirus 1	NA	Viruses; Riboviria; Orthornavirae; Pisuviricota; Duplopiviricetes; Durnavirales; Partitiviridae; Alphapartitivirus; unclassified Alphapartitivirus
GCA_000857945.1	158482	168	Mamestra configurata nucleopolyhedrovirus B	NA	Viruses; dsDNA viruses, no RNA stage; Baculoviridae; Alphabaculovirus
GCA_002956145.1	96542	149	Stenotrophomonas phage vB_SmaS_DLP_5	NA	Viruses; Duplodnaviria; Heunggongvirae; Uroviricota; Caudoviricetes; Caudovirales; Siphoviridae; Delepquintavirus; Stenotrophomonas virus DLP5
GCA_000897875.1	9544	1	Iranian johnsongrass mosaic virus	NA	Viruses; ssRNA viruses; ssRNA positive-strand viruses, no DNA stage; Potyviridae; Potyvirus; Zea mosaic virus
GCA_006451505.1	104286	104	Rana nigromaculata ranavirus	MWH421017	Viruses; Varidnaviria; Bamfordvirae; Nucleocytoviricota; Megaviricetes; Pimascovirales; Iridoviridae; Alphairidovirinae; Ranavirus; Frog virus 3
GCA_000867485.1	84264	93	Neodiprion abietis NPV	NA	Viruses; dsDNA viruses, no RNA stage; Baculoviridae; Gammabaculovirus; unclassified Gammabaculovirus
GCA_003180115.1	7654	7	Pygoscelis adeliae papillomavirus 2	NA	Viruses; Monodnaviria; Shotokuvirae; Cossaviricota; Papovaviricetes; Zurhausenvirales; Papillomaviridae; Firstpapillomavirinae; Treisepsilonpapillomavirus; Treisepsilonpapillomavirus 1
GCA_004130295.1	10311	1	Kampung Karu virus	NA	Viruses; Riboviria; Orthornavirae; Kitrinoviricota; Flasuviricetes; Amarillovirales; Flaviviridae; Flavivirus; unclassified Flavivirus
GCA_004131305.1	7446	5	Grapevine virus H	NA	Viruses; Riboviria; Orthornavirae; Kitrinoviricota; Alsuviricetes; Tymovirales; Betaflexiviridae; Trivirinae; Vitivirus
GCA_002745275.1	39761	57	Escherichia phage APC_JM32	NA	Viruses; Duplodnaviria; Heunggongvirae; Uroviricota; Caudoviricetes; Caudovirales; Podoviridae; Lederbergvirus; unclassified Lederbergvirus
GCA_004117335.1	3721	2	Rhizoctonia solani dsRNA virus 4	RsRV4D168	Viruses; Riboviria; Orthornavirae; Pisuviricota; Duplopiviricetes; Durnavirales; Partitiviridae; unclassified Partitiviridae
GCA_008800955.1	8021	2	Theilerslike virus of rats	NGS910	Viruses; Riboviria; Orthornavirae; Pisuviricota; Pisoniviricetes; Picornavirales; Picornaviridae; Cardiovirus; Cardiovirus B; Theilovirus
GCA_002956155.1	63945	82	Stenotrophomonas phage DLP4	NA	Viruses; Duplodnaviria; Heunggongvirae; Uroviricota; Caudoviricetes; Caudovirales; Siphoviridae; Pamexvirus; unclassified Pamexvirus
GCA_002956165.1	43891	61	Arthrobacter phage Huntingdon	NA	Viruses; Duplodnaviria; Heunggongvirae; Uroviricota; Caudoviricetes; Caudovirales; Siphoviridae; Korravirus; unclassified Korravirus
GCA_002956185.1	72444	89	Escherichia phage St11Ph5	NA	Viruses; Duplodnaviria; Heunggongvirae; Uroviricota; Caudoviricetes; Caudovirales; Podoviridae; Gamaleyavirus; unclassified Gamaleyavirus
GCA_002956195.1	112793	149	Aphanizomenon phage vB_AphaSCL131	NA	Viruses; Duplodnaviria; Heunggongvirae; Uroviricota; Caudoviricetes; Caudovirales; Siphoviridae; unclassified Siphoviridae
GCA_002956205.1	42686	46	Escherichia phage PGT2	NA	Viruses; Duplodnaviria; Heunggongvirae; Uroviricota; Caudoviricetes; Caudovirales; Autographiviridae; Ermolevavirus; Escherichia virus PGT2
GCA_003991645.1	47797	17	Klebsiella phage 1611EK21	NA	Viruses; Duplodnaviria; Heunggongvirae; Uroviricota; Caudoviricetes; Caudovirales; Myoviridae; Jedunavirus; unclassified Jedunavirus
GCA_002956215.1	16523	36	Gluconobacter phage GC1	NA	Viruses; Varidnaviria; Bamfordvirae; Preplasmiviricota; Tectiliviricetes; Kalamavirales; Tectiviridae; Gammatectivirus; Gluconobacter virus GC1
GCA_002956225.1	44829	78	Escherichia phage VB_EcoSGolestan	NA	Viruses; Duplodnaviria; Heunggongvirae; Uroviricota; Caudoviricetes; Caudovirales; Siphoviridae; Guernseyvirinae; Kagunavirus; Escherichia virus Golestan
GCA_002956235.1	58499	96	Arthrobacter phage Waltz	NA	Viruses; Duplodnaviria; Heunggongvirae; Uroviricota; Caudoviricetes; Caudovirales; Siphoviridae; Laroyevirus; unclassified Laroyevirus
GCA_002956245.1	73851	92	Gordonia phage Anamika	NA	Viruses; Duplodnaviria; Heunggongvirae; Uroviricota; Caudoviricetes; Caudovirales; Siphoviridae; Woesvirus; unclassified Woesvirus
GCA_002956255.1	54867	102	Gordonia phage BENtherdunthat	NA	Viruses; Duplodnaviria; Heunggongvirae; Uroviricota; Caudoviricetes; Caudovirales; Siphoviridae; Langleyhallvirinae; Getalongvirus; Gordonia virus BENtherdunthat
GCA_002956265.1	77354	110	Gordonia phage BirksAndSocks	NA	Viruses; Duplodnaviria; Heunggongvirae; Uroviricota; Caudoviricetes; Caudovirales; Siphoviridae; Montyvirus; Gordonia virus Birksandsocks
GCA_002956275.1	46042	72	Gordonia phage Bjanes7	NA	Viruses; Duplodnaviria; Heunggongvirae; Uroviricota; Caudoviricetes; Caudovirales; Siphoviridae; Attisvirus; unclassified Attisvirus
GCA_002956285.1	50503	85	Mycobacterium phage AgentM	NA	Viruses; Duplodnaviria; Heunggongvirae; Uroviricota; Caudoviricetes; Caudovirales; Siphoviridae; Fromanvirus; unclassified Fromanvirus
GCA_002956295.1	43779	65	Mycobacterium phage Andies	NA	Viruses; Duplodnaviria; Heunggongvirae; Uroviricota; Caudoviricetes; Caudovirales; Siphoviridae; Nclasvirinae; Charlievirus; unclassified Charlievirus
GCA_002956305.1	50812	86	Mycobacterium phage Aragog	NA	Viruses; Duplodnaviria; Heunggongvirae; Uroviricota; Caudoviricetes; Caudovirales; Siphoviridae; Fromanvirus; unclassified Fromanvirus

gca	genome.size	proteome.size	phage.name	strain	Taxonomy
GCA_002956315.1	47409	75	Mycobacterium phage Archetta	NA	Viruses; Duplodnaviria; Heunggongvirae; Uroviricota; Caudoviricetes; Caudovirales; Siphoviridae; Fromanvirus; unclassified Fromanvirus
GCA_002956325.1	51374	87	Mycobacterium phage Blackmoor	NA	Viruses; Duplodnaviria; Heunggongvirae; Uroviricota; Caudoviricetes; Caudovirales; Siphoviridae; Fromanvirus; unclassified Fromanvirus
GCA_002956335.1	71807	129	Mycobacterium phage Familton	NA	Viruses; Duplodnaviria; Heunggongvirae; Uroviricota; Caudoviricetes; Caudovirales; Siphoviridae; Corndogvirus; unclassified Corndogvirus
GCA_002956345.1	51050	87	Mycobacterium phage ForGetIt	NA	Viruses; Duplodnaviria; Heunggongvirae; Uroviricota; Caudoviricetes; Caudovirales; Siphoviridae; Fromanvirus; unclassified Fromanvirus
GCA_002956355.1	52879	102	Mycobacterium phage Koko	NA	Viruses; Duplodnaviria; Heunggongvirae; Uroviricota; Caudoviricetes; Caudovirales; Siphoviridae; Fromanvirus; unclassified Fromanvirus
GCA_002956365.1	44145	65	Mycobacterium phage LouisV14	NA	Viruses; Duplodnaviria; Heunggongvirae; Uroviricota; Caudoviricetes; Caudovirales; Siphoviridae; Liefievirus; unclassified Liefievirus
GCA_002956375.1	52874	97	Mycobacterium phage Ph8s	NA	Viruses; Duplodnaviria; Heunggongvirae; Uroviricota; Caudoviricetes; Caudovirales; Siphoviridae; Fromanvirus; unclassified Fromanvirus
GCA_002956385.1	43886	72	Mycobacterium phage Philonius	NA	Viruses; Duplodnaviria; Heunggongvirae; Uroviricota; Caudoviricetes; Caudovirales; Siphoviridae; Nclasvirinae; Charlievirus; unclassified Charlievirus
GCA_002956395.1	50403	84	Mycobacterium phage Phlorence	NA	Viruses; Duplodnaviria; Heunggongvirae; Uroviricota; Caudoviricetes; Caudovirales; Siphoviridae; Fromanvirus; unclassified Fromanvirus
GCA_002956405.1	50907	94	Mycobacterium phage Wilkins	NA	Viruses; Duplodnaviria; Heunggongvirae; Uroviricota; Caudoviricetes; Caudovirales; Siphoviridae; Fromanvirus; unclassified Fromanvirus
GCA_002956415.1	48724	89	Mycobacterium phage WunderPhul	NA	Viruses; Duplodnaviria; Heunggongvirae; Uroviricota; Caudoviricetes; Caudovirales; Siphoviridae; Fromanvirus; unclassified Fromanvirus
GCA_002956425.1	75896	148	Mycobacterium phage Youngblood	NA	Viruses; Duplodnaviria; Heunggongvirae; Uroviricota; Caudoviricetes; Caudovirales; Siphoviridae; Kostyavirus; unclassified Kostyavirus
GCA_002956435.1	43812	62	Arthrobacter phage Fluke	NA	Viruses; Duplodnaviria; Heunggongvirae; Uroviricota; Caudoviricetes; Caudovirales; Siphoviridae; Korravirus; unclassified Korravirus
GCA_002956445.1	44258	63	Arthrobacter phage MeganNoll	NA	Viruses; Duplodnaviria; Heunggongvirae; Uroviricota; Caudoviricetes; Caudovirales; Siphoviridae; Korravirus; unclassified Korravirus
GCA_002956455.1	43940	62	Arthrobacter phage Urla	NA	Viruses; Duplodnaviria; Heunggongvirae; Uroviricota; Caudoviricetes; Caudovirales; Siphoviridae; Korravirus; unclassified Korravirus
GCA_002956465.1	67383	93	Corynebacterium phage C3PO	NA	Viruses; Duplodnaviria; Heunggongvirae; Uroviricota; Caudoviricetes; Caudovirales; Siphoviridae; Ceetrepovirus; Corynebacterium virus C3PO
GCA_002956475.1	67743	94	Corynebacterium phage Darwin	NA	Viruses; Duplodnaviria; Heunggongvirae; Uroviricota; Caudoviricetes; Caudovirales; Siphoviridae; Ceetrepovirus; Corynebacterium virus Darwin
GCA_002956485.1	66359	92	Corynebacterium phage PotatoChip	NA	Viruses; Duplodnaviria; Heunggongvirae; Uroviricota; Caudoviricetes; Caudovirales; Siphoviridae; Ceetrepovirus; unclassified Ceetrepovirus
GCA_002956495.1	66392	92	Corynebacterium phage Zion	NA	Viruses; Duplodnaviria; Heunggongvirae; Uroviricota; Caudoviricetes; Caudovirales; Siphoviridae; Ceetrepovirus; Corynebacterium virus Zion
GCA_002956505.1	44408	69	Gordonia phage Gustav	NA	Viruses; Duplodnaviria; Heunggongvirae; Uroviricota; Caudoviricetes; Caudovirales; Siphoviridae; Gustavvirus; Gordonia virus Gustav
GCA_002956515.1	42475	61	Gordonia phage Mahdia	NA	Viruses; Duplodnaviria; Heunggongvirae; Uroviricota; Caudoviricetes; Caudovirales; Siphoviridae; Gustavvirus; Gordonia virus Mahdia
GCA_002956525.1	47618	78	Mycobacterium phage Thespis	NA	Viruses; Duplodnaviria; Heunggongvirae; Uroviricota; Caudoviricetes; Caudovirales; Siphoviridae; Pclasvirinae; Fishburnevirus; unclassified Fishburnevirus
GCA_002956535.1	166596	234	Lake Baikal phage Baikal205mC28	NA	Viruses; Duplodnaviria; Heunggongvirae; Uroviricota; Caudoviricetes; Caudovirales; Myoviridae; unclassified Myoviridae
GCA_002745295.1	50552	78	Shigella phage vB_SflSISF001	NA	Viruses; Duplodnaviria; Heunggongvirae; Uroviricota; Caudoviricetes; Caudovirales; Drexlerviridae; Tunavirinae; Tunavirus; Shigella virus ISF001
GCA_002957125.1	59128	57	Proteus phage PM87	NA	Viruses; Duplodnaviria; Heunggongvirae; Uroviricota; Caudoviricetes; Caudovirales; Siphoviridae; unclassified Siphoviridae
GCA_002957135.1	104329	102	Proteus phage PM135	NA	Viruses; Duplodnaviria; Heunggongvirae; Uroviricota; Caudoviricetes; Caudovirales; Demerecviridae; Novosibvirus; Proteus virus PM135
GCA_002957145.1	45814	60	Escherichia phage DTL	NA	Viruses; Duplodnaviria; Heunggongvirae; Uroviricota; Caudoviricetes; Caudovirales; Drexlerviridae; Braunvirinae; Loudonvirus; Escherichia virus DTL
GCA_003308915.1	138073	213	Phage 203	NA	Viruses; Duplodnaviria; Heunggongvirae; Uroviricota; Caudoviricetes; Caudovirales; Myoviridae; Vequintavirinae; Vequintavirus; unclassified Vequintavirus
GCA_003308935.1	138073	213	Phage 206	NA	Viruses; Duplodnaviria; Heunggongvirae; Uroviricota; Caudoviricetes; Caudovirales; Myoviridae; Vequintavirinae; Vequintavirus; unclassified Vequintavirus
GCA_003288535.1	43416	61	Lactobacillus phage Lb	NA	Viruses; Duplodnaviria; Heunggongvirae; Uroviricota; Caudoviricetes; Caudovirales; Myoviridae; Heilongjiangvirus; Lactobacillus virus Lb
GCA_000875345.1	10207	2	Xenopus laevis endogenous retrovirus Xen1	NA	Viruses; Riboviria; Pararnavirae; Artverviricota; Revtraviricetes; Ortervirales; Retroviridae; unclassified Retroviridae; amphibian retroviruses
GCA_013087705.1	1361	2	Tomato leaf curl Virudhunagar alphasatellite	severe	Viruses; Alphasatellitidae; Geminialphasatellitinae; Clecrusatellite
GCA_013087715.1	2756	7	Coccinia mosaic Virudhunagar virus	severe	Viruses; Monodnaviria; Shotokuvirae; Cressdnaviricota; Repensiviricetes; Geplafuvirales; Geminiviridae; Begomovirus; unclassified Begomovirus
GCA_003850205.1	59715	73	Salmonella phage KFSSE1	NA	Viruses; Duplodnaviria; Heunggongvirae; Uroviricota; Caudoviricetes; Caudovirales; Siphoviridae; Chivirus; unclassified Chivirus

gca	genome.size	proteome.size	phage.name	strain	Taxonomy
GCA_002957155.1	70211	76	Pseudomonas phage inbricus	NA	Viruses; Duplodnaviria; Heunggongvirae; Uroviricota; Caudoviricetes; Caudovirales; Podoviridae; Jwalphavirus; unclassified Jwalphavirus
GCA_002957165.1	112225	161	Pseudomonas phage nickie	NA	Viruses; Duplodnaviria; Heunggongvirae; Uroviricota; Caudoviricetes; Caudovirales; Siphoviridae; Nickievirus; Pseudomonas virus nickie
GCA_002957175.1	67498	89	Pseudomonas phage tabernarius	NA	Viruses; Duplodnaviria; Heunggongvirae; Uroviricota; Caudoviricetes; Caudovirales; Myoviridae; Tabernariusvirus; Pseudomonas virus tabernarius
GCA_002957185.2	47868	46	Pseudomonas phage uligo	NA	Viruses; Duplodnaviria; Heunggongvirae; Uroviricota; Caudoviricetes; Caudovirales; Autographiviridae; Colwellvirinae; Uliginvirus; Pseudomonas virus uligo
GCA_002957195.1	97425	172	Pseudomonas phage ventosus	NA	Viruses; Duplodnaviria; Heunggongvirae; Uroviricota; Caudoviricetes; Caudovirales; Myoviridae; unclassified Myoviridae
GCA_000908175.1	3919	5	Calibrachoa mottle virus	NA	Viruses; ssRNA viruses; ssRNA positive-strand viruses, no DNA stage; Tombusviridae; Alphacarmovirus
GCA_002957205.2	51963	96	Sinorhizobium phage HMSP1Susan	NA	Viruses; Duplodnaviria; Heunggongvirae; Uroviricota; Caudoviricetes; Caudovirales; Siphoviridae; unclassified Siphoviridae
GCA_002645735.1	18503	11	Caprine rotavirus A	GO34	Viruses; Riboviria; Orthornavirae; Duplornaviricota; Resentoviricetes; Reovirales; Reoviridae; Sedoreovirinae; Rotavirus; Rotavirus A
GCA_002029535.1	6730	1	Grapevine rupestris vein feathering virus	NA	Viruses; ssRNA viruses; ssRNA positive-strand viruses, no DNA stage; Tymovirales; Tymoviridae; Marafivirus; unclassified Marafivirus
GCA_004131525.1	7257	7	Gammapapillomavirus sp	NA	Viruses; Monodnaviria; Shotokuvirae; Cossaviricota; Papovaviricetes; Zurhausenvirales; Papillomaviridae; Firstpapillomavirinae; Gammapapillomavirus; unclassified Gammapapillomavirus
GCA_000852605.1	15192	6	Goose paramyxovirus SF02	NA	Viruses; ssRNA viruses; ssRNA negative-strand viruses; Mononegavirales; Paramyxoviridae; Avulavirus; unclassified Avulavirus
GCA_002987875.1	1367	1	Mirabilis leaf curl betasatellite	NA	Viruses; Tolecusatellitidae; Betasatellite
GCA_002830325.1	1342	1	Tomato leaf curl Malaysia betasatellite	NA	Viruses; Tolecusatellitidae; Betasatellite
GCA_002826165.1	3278	4	Pomona bat hepatitis B virus	NA	Viruses; Riboviria; Pararnavirae; Artverviricota; Revtraviricetes; Blubervirales; Hepadnaviridae; Orthohepadnavirus
GCA_002824185.1	2739	7	Tomato leaf curl New Delhi virus 4	NA	Viruses; Monodnaviria; Shotokuvirae; Cressdnaviricota; Repensiviricetes; Geplafuvirales; Geminiviridae; Begomovirus
GCA_012271615.1	31393	6	Rodent coronavirus	NA	Viruses; Riboviria; Orthornavirae; Pisuviricota; Pisoniviricetes; Nidovirales; Cornidovirineae; Coronaviridae; Coronavirinae; unclassified Coronavirinae
GCA_012271625.1	27102	5	Shrew coronavirus	NA	Viruses; Riboviria; Orthornavirae; Pisuviricota; Pisoniviricetes; Nidovirales; Cornidovirineae; Coronaviridae; Orthocoronavirinae; unclassified Orthocoronavirinae
GCA_004130355.1	7373	6	Rodent papillomavirus	NA	Viruses; Monodnaviria; Shotokuvirae; Cossaviricota; Papovaviricetes; Zurhausenvirales; Papillomaviridae; unclassified Papillomaviridae; non-primate mammal papillomaviruses
GCA_000905615.1	3230	4	Longfingered bat hepatitis B virus	NA	Viruses; Riboviria; Pararnavirae; Artverviricota; Revtraviricetes; Blubervirales; Hepadnaviridae; Orthohepadnavirus
GCA_000905135.1	1719	4	Cockroach associated cyclovirus 1	NA	Viruses; Monodnaviria; Shotokuvirae; Cressdnaviricota; Arfiviricetes; Cirlivirales; Circoviridae; Cyclovirus
GCA_009789795.1	37617	27	Mastadenovirus sp	NA	Viruses; Varidnaviria; Bamfordvirae; Preplasmiviricota; Tectiliviricetes; Rowavirales; Adenoviridae; Mastadenovirus; unclassified Mastadenovirus
GCA_002824165.1	2735	6	Tomato leaf curl New Delhi virus 2	NA	Viruses; Monodnaviria; Shotokuvirae; Cressdnaviricota; Repensiviricetes; Geplafuvirales; Geminiviridae; Begomovirus
GCA_000899775.1	2741	7	French bean leaf curl virus	NA	Viruses; Monodnaviria; Shotokuvirae; Cressdnaviricota; Repensiviricetes; Geplafuvirales; Geminiviridae; Begomovirus
GCA_002819645.1	1771	2	Bat associated cyclovirus 2	NA	Viruses; Monodnaviria; Shotokuvirae; Cressdnaviricota; Arfiviricetes; Cirlivirales; Circoviridae; Cyclovirus
GCA_002819665.1	1741	2	Bat associated cyclovirus 3	NA	Viruses; Monodnaviria; Shotokuvirae; Cressdnaviricota; Arfiviricetes; Cirlivirales; Circoviridae; Cyclovirus
GCA_002819685.1	1818	2	Bat associated cyclovirus 4	NA	Viruses; Monodnaviria; Shotokuvirae; Cressdnaviricota; Arfiviricetes; Cirlivirales; Circoviridae; Cyclovirus
GCA_000892915.1	1377	1	Vernonia yellow vein Fujian betasatellite	NA	Viruses; Tolecusatellitidae; Betasatellite
GCA_002957215.2	40139	62	Enterococcus phage phiNASRA1	NA	Viruses; Duplodnaviria; Heunggongvirae; Uroviricota; Caudoviricetes; Caudovirales; Siphoviridae; Efquatrovirus; unclassified Efquatrovirus
GCA_004132105.1	11336	1	Lumpfish flavivirus	NA	Viruses; Riboviria; Orthornavirae; Kitrinoviricota; Flasuviricetes; Amarillovirales; Flaviviridae; unclassified Flaviviridae
GCA_002957225.1	29339	52	Lactococcus phage CB14a	NA	Viruses; Duplodnaviria; Heunggongvirae; Uroviricota; Caudoviricetes; Caudovirales; Siphoviridae; Skunavirus; unclassified Skunavirus
GCA_002957235.1	29339	52	Lactococcus phage CB14b	NA	Viruses; Duplodnaviria; Heunggongvirae; Uroviricota; Caudoviricetes; Caudovirales; Siphoviridae; Skunavirus; unclassified Skunavirus
GCA_013087725.1	12949	6	Cabbage cytorhabdovirus 1	FERA_050726	Viruses; Riboviria; Orthornavirae; Negarnaviricota; Haploviricotina; Monjiviricetes; Mononegavirales; Rhabdoviridae; Cytorhabdovirus; Cabbage cytorhabdovirus
GCA_002937185.1	7507	5	Grapevine virus I	NA	Viruses; Riboviria; Orthornavirae; Kitrinoviricota; Alsuviricetes; Tymovirales; Betaflexiviridae; Trivirinae; Vitivirus
GCA_002937205.1	2571	1	Erysiphe necator mitovirus 1	NA	Viruses; Riboviria; Orthornavirae; Lenarviricota; Howeltoviricetes; Cryppavirales; Mitoviridae; Mitovirus; unclassified Mitovirus

gca	genome.size	proteome.size	phage.name	strain	Taxonomy
GCA_002937215.1	2581	1	Erysiphe necator mitovirus 2	NA	Viruses; Riboviria; Orthornavirae; Lenarviricota; Howeltoviricetes; Cryppavirales; Mitoviridae; Mitovirus; unclassified Mitovirus
GCA_002937225.1	2892	1	Erysiphe necator mitovirus 3	NA	Viruses; Riboviria; Orthornavirae; Lenarviricota; Howeltoviricetes; Cryppavirales; Mitoviridae; Mitovirus; unclassified Mitovirus
GCA_013087735.1	1005	1	Faba bean necrotic yellows virus associated alphasatellite 1	NA	Viruses; Alphasatellitidae; Nanoalphasatellitinae; Subclovsatellite; Faba bean necrotic yellows alphasatellite 3
GCA_018583145.1	109717	71	Vombatid gammaherpesvirus 1	V318711	Viruses; Duplodnaviria; Heunggongvirae; Peploviricota; Herviviricetes; Herpesvirales; Herpesviridae; Gammaherpesvirinae; Manticavirus
GCA_002868615.1	13706	7	Citrus leprosis virus C2	NA	Viruses; Riboviria; Orthornavirae; Kitrinoviricota; Alsuviricetes; Martellivirales; Kitaviridae; Cilevirus
GCA_002990485.1	354378	531	Yersinia phage fHeYen904	NA	Viruses; Duplodnaviria; Heunggongvirae; Uroviricota; Caudoviricetes; Caudovirales; Myoviridae; Eneladusvirus; Yersinia virus Yen9-04
GCA_002990515.1	352596	538	Yersinia phage fHeYen903	NA	Viruses; Duplodnaviria; Heunggongvirae; Uroviricota; Caudoviricetes; Caudovirales; Myoviridae; Eneladusvirus; unclassified Eneladusvirus
GCA_002990645.1	40405	50	Yersinia phage fPS89	NA	Viruses; Duplodnaviria; Heunggongvirae; Uroviricota; Caudoviricetes; Caudovirales; Autographiviridae; Studiervirinae; Helsettvirus; unclassified Helsettvirus
GCA_002990635.1	38967	51	Yersinia phage fPS7	NA	Viruses; Duplodnaviria; Heunggongvirae; Uroviricota; Caudoviricetes; Caudovirales; Autographiviridae; Studiervirinae; Helsettvirus; unclassified Helsettvirus
GCA_002990525.1	39034	52	Yersinia phage fPS9	NA	Viruses; Duplodnaviria; Heunggongvirae; Uroviricota; Caudoviricetes; Caudovirales; Autographiviridae; Studiervirinae; Helsettvirus; Yersinia virus fPS9
GCA_002990595.1	38938	51	Yersinia phage fPS19	NA	Viruses; Duplodnaviria; Heunggongvirae; Uroviricota; Caudoviricetes; Caudovirales; Autographiviridae; Studiervirinae; Helsettvirus; unclassified Helsettvirus
GCA_002990715.1	39180	51	Yersinia phage fPS21	NA	Viruses; Duplodnaviria; Heunggongvirae; Uroviricota; Caudoviricetes; Caudovirales; Autographiviridae; Studiervirinae; Helsettvirus; unclassified Helsettvirus
GCA_002990555.1	38792	51	Yersinia phage fPS26	NA	Viruses; Duplodnaviria; Heunggongvirae; Uroviricota; Caudoviricetes; Caudovirales; Autographiviridae; Studiervirinae; Helsettvirus; unclassified Helsettvirus
GCA_002990695.1	39764	50	Yersinia phage fPS50	NA	Viruses; Duplodnaviria; Heunggongvirae; Uroviricota; Caudoviricetes; Caudovirales; Autographiviridae; Studiervirinae; Helsettvirus; unclassified Helsettvirus
GCA_002990585.1	39888	50	Yersinia phage fPS52	NA	Viruses; Duplodnaviria; Heunggongvirae; Uroviricota; Caudoviricetes; Caudovirales; Autographiviridae; Studiervirinae; Helsettvirus; unclassified Helsettvirus
GCA_002990765.1	40694	52	Yersinia phage fPS53	NA	Viruses; Duplodnaviria; Heunggongvirae; Uroviricota; Caudoviricetes; Caudovirales; Autographiviridae; Studiervirinae; Helsettvirus; Yersinia virus fPS53
GCA_002990805.1	40075	48	Yersinia phage fPS54ocr	NA	Viruses; Duplodnaviria; Heunggongvirae; Uroviricota; Caudoviricetes; Caudovirales; Autographiviridae; Studiervirinae; Helsettvirus; Yersinia virus fPS54ocr
GCA_002990725.1	38391	47	Yersinia phage fPS59	NA	Viruses; Duplodnaviria; Heunggongvirae; Uroviricota; Caudoviricetes; Caudovirales; Autographiviridae; Studiervirinae; Helsettvirus; Yersinia virus fPS59
GCA_002990735.1	39326	50	Yersinia phage fPS64	NA	Viruses; Duplodnaviria; Heunggongvirae; Uroviricota; Caudoviricetes; Caudovirales; Autographiviridae; Studiervirinae; Helsettvirus; unclassified Helsettvirus
GCA_002990775.1	40429	49	Yersinia phage fPS85	NA	Viruses; Duplodnaviria; Heunggongvirae; Uroviricota; Caudoviricetes; Caudovirales; Autographiviridae; Studiervirinae; Helsettvirus; unclassified Helsettvirus
GCA_002990675.1	39024	51	Yersinia phage fPS86	NA	Viruses; Duplodnaviria; Heunggongvirae; Uroviricota; Caudoviricetes; Caudovirales; Autographiviridae; Studiervirinae; Helsettvirus; unclassified Helsettvirus
GCA_003004815.1	44530	56	Corynebacterium phage phi673	NA	Viruses; Duplodnaviria; Heunggongvirae; Uroviricota; Caudoviricetes; Caudovirales; Siphoviridae; Ikedavirus; Corynebacterium virus phi673
GCA_003004825.1	43193	54	Corynebacterium phage phi674	NA	Viruses; Duplodnaviria; Heunggongvirae; Uroviricota; Caudoviricetes; Caudovirales; Siphoviridae; Ikedavirus; Corynebacterium virus phi674
GCA_013087745.1	7145	3	Grapevine badnavirus 1	NA	Viruses; Riboviria; Pararnavirae; Artverviricota; Revtraviricetes; Ortervirales; Caulimoviridae; Badnavirus
GCA_004117315.1	8295	4	Sambucus virus S	NA	Viruses; Riboviria; Orthornavirae; Kitrinoviricota; Alsuviricetes; Martellivirales; Bromoviridae; unclassified Bromoviridae
GCA_003972105.1	7396	3	Cladosporium fulvum T1 virus	Race 4	Viruses; Riboviria; Pararnavirae; Artverviricota; Revtraviricetes; Ortervirales; Metaviridae; Metavirus
GCA_002990835.1	39179	51	Yersinia phage fPS10	NA	Viruses; Duplodnaviria; Heunggongvirae; Uroviricota; Caudoviricetes; Caudovirales; Autographiviridae; Studiervirinae; Helsettvirus; unclassified Helsettvirus
GCA_002990815.1	39227	51	Yersinia phage fPS16	NA	Viruses; Duplodnaviria; Heunggongvirae; Uroviricota; Caudoviricetes; Caudovirales; Autographiviridae; Studiervirinae; Helsettvirus; unclassified Helsettvirus
GCA_003861675.1	39688	49	Salmonella phage BSP161	NA	Viruses; Duplodnaviria; Heunggongvirae; Uroviricota; Caudoviricetes; Caudovirales; Autographiviridae; Studiervirinae; Berlinvirus; Salmonella virus BSP161
GCA_004130315.1	20949	7	Yichang virus	NA	Viruses; Riboviria; Orthornavirae; Pisuviricota; Pisoniviricetes; Nidovirales; Mesnidovirineae; Mesoniviridae; unclassified Mesoniviridae
GCA_002957245.1	48659	55	Pectinobacterium phage PEAT2	NA	Viruses; Duplodnaviria; Heunggongvirae; Uroviricota; Caudoviricetes; Caudovirales; Myoviridae; Peatvirus; Pectinobacterium virus PEAT2
GCA_004131925.1	4880	3	Rhinolophus pusillus bocaparvovirus 1	RpBtBoV1_48C_MJ_YN_2012	Viruses; Monodnaviria; Shotokuvirae; Cossaviricota; Quintoviricetes; Piccovirales; Parvoviridae; Parvovirinae; Bocaparvovirus; unclassified Bocaparvovirus
GCA_004131945.1	4726	3	Rhinolophus pusillus bocaparvovirus 2	RpBtBoV2_83C_MJ_YN_2012	Viruses; Monodnaviria; Shotokuvirae; Cossaviricota; Quintoviricetes; Piccovirales; Parvoviridae; Parvovirinae; Bocaparvovirus; unclassified Bocaparvovirus
GCA_004131965.1	5038	3	Rousettus leschenaultii bocaparvovirus 1	RolBtBoV1_56C_ML_YN_2012	Viruses; Monodnaviria; Shotokuvirae; Cossaviricota; Quintoviricetes; Piccovirales; Parvoviridae; Parvovirinae; Bocaparvovirus; unclassified Bocaparvovirus

gca	genome.size	proteome.size	phage.name	strain	Taxonomy
GCA_002957255.1	39919	51	Escherichia phage EG1	NA	Viruses; Duplodnaviria; Heunggongvirae; Uroviricota; Caudoviricetes; Caudovirales; Autographiviridae; Studiervirinae; Teseptimavirus; Escherichia virus EG1
GCA_002957265.1	38840	50	TM7 phage DolZOral124_53_65	NA	Viruses; Duplodnaviria; Heunggongvirae; Uroviricota; Caudoviricetes; Caudovirales; Podoviridae; unclassified Podoviridae
GCA_002957275.2	111103	169	Klebsiella phage Sugarland	NA	Viruses; Duplodnaviria; Heunggongvirae; Uroviricota; Caudoviricetes; Caudovirales; Demerecviridae; Sugarlandvirus; Klebsiella virus Sugarland
GCA_003861695.1	139069	178	Synechococcus phage SCBWM1	NA	Viruses; Duplodnaviria; Heunggongvirae; Uroviricota; Caudoviricetes; Caudovirales; Myoviridae; Aokuangvirus; Synechococcus virus SCBWM1
GCA_002957295.2	158639	209	Escherichia phage FEC14	NA	Viruses; Duplodnaviria; Heunggongvirae; Uroviricota; Caudoviricetes; Caudovirales; Ackermannviridae; Cvivirinae; Kuttervirus; unclassified Kuttervirus
GCA_002957305.1	48803	76	Streptomyces phage Alsaber	NA	Viruses; Duplodnaviria; Heunggongvirae; Uroviricota; Caudoviricetes; Caudovirales; Siphoviridae; Arquatrovirinae; Camvirus; unclassified Camvirus
GCA_002957315.1	152048	198	Dickeya phage PP35	NA	Viruses; Duplodnaviria; Heunggongvirae; Uroviricota; Caudoviricetes; Caudovirales; Ackermannviridae; Aglimvirinae; Limestonevirus; unclassified Limestonevirus
GCA_003861655.1	45970	69	Pseudomonas phage Delta	NA	Viruses; Duplodnaviria; Heunggongvirae; Uroviricota; Caudoviricetes; Caudovirales; Podoviridae; Bruynoghevirus; unclassified Bruynoghevirus
GCA_002957325.1	78891	131	Lactobacillus phage Lenus	NA	Viruses; Duplodnaviria; Heunggongvirae; Uroviricota; Caudoviricetes; Caudovirales; Siphoviridae; Tybeckvirinae; Lenusvirus; Lactobacillus virus Lenus
GCA_002957335.1	4360	6	Citromicrobium phage vB_Cib_ssDNA_P1	NA	Viruses; Monodnaviria; Sangervirae; Phixviricota; Malgrandaviricetes; Petitvirales; Microviridae; unclassified Microviridae
GCA_002957345.1	50316	87	Salmonella phage YSP2	NA	Viruses; Duplodnaviria; Heunggongvirae; Uroviricota; Caudoviricetes; Caudovirales; Drexlerviridae; Tempevirinae; Tlsvirus; Salmonella virus YSP2
GCA_004610855.1	41317	55	Escherichia phage vB_EcoS_HSE2	NA	Viruses; Duplodnaviria; Heunggongvirae; Uroviricota; Caudoviricetes; Caudovirales; Siphoviridae; Guernseyvirinae; Kagunavirus; unclassified Kagunavirus
GCA_002957375.1	42789	62	Pseudomonas phage Psp6	NA	Viruses; Duplodnaviria; Heunggongvirae; Uroviricota; Caudoviricetes; Caudovirales; Siphoviridae; unclassified Siphoviridae
GCA_002957395.1	86360	129	Salmonella virus VSe11	NA	Viruses; Duplodnaviria; Heunggongvirae; Uroviricota; Caudoviricetes; Caudovirales; Myoviridae; Ounavirinae; Felixounavirus; unclassified Felixounavirus
GCA_002957405.1	86365	129	Salmonella virus VSe102	NA	Viruses; Duplodnaviria; Heunggongvirae; Uroviricota; Caudoviricetes; Caudovirales; Myoviridae; Ounavirinae; Felixounavirus; unclassified Felixounavirus
GCA_002957415.1	221116	332	Aeromonas phage Ah1	NA	Viruses; Duplodnaviria; Heunggongvirae; Uroviricota; Caudoviricetes; Caudovirales; Myoviridae; Tevenvirinae; unclassified Tevenvirinae
GCA_002957425.1	145759	245	Raoultella phage Ro1	NA	Viruses; Duplodnaviria; Heunggongvirae; Uroviricota; Caudoviricetes; Caudovirales; Myoviridae; Vequintavirinae; unclassified Vequintavirinae
GCA_003288575.1	30249	57	Lactococcus phage vB_Llc_bIBB14s	NA	Viruses; Duplodnaviria; Heunggongvirae; Uroviricota; Caudoviricetes; Caudovirales; Siphoviridae; Skunavirus; unclassified Skunavirus
GCA_003288595.1	29736	55	Lactococcus phage vB_Llc_bIBB24tp1	NA	Viruses; Duplodnaviria; Heunggongvirae; Uroviricota; Caudoviricetes; Caudovirales; Siphoviridae; Skunavirus; unclassified Skunavirus
GCA_003288555.1	30132	55	Lactococcus phage vB_Llc_bIBB5g1	NA	Viruses; Duplodnaviria; Heunggongvirae; Uroviricota; Caudoviricetes; Caudovirales; Siphoviridae; Skunavirus; unclassified Skunavirus
GCA_003288615.1	29423	54	Lactococcus phage vB_Llc_bIBB77s	NA	Viruses; Duplodnaviria; Heunggongvirae; Uroviricota; Caudoviricetes; Caudovirales; Siphoviridae; Skunavirus; unclassified Skunavirus
GCA_003288635.1	29659	54	Lactococcus phage vB_Llc_bIBBEg1	NA	Viruses; Duplodnaviria; Heunggongvirae; Uroviricota; Caudoviricetes; Caudovirales; Siphoviridae; Skunavirus; unclassified Skunavirus
GCA_003288655.1	29454	55	Lactococcus phage vB_Llc_bIBBF12	NA	Viruses; Duplodnaviria; Heunggongvirae; Uroviricota; Caudoviricetes; Caudovirales; Siphoviridae; Skunavirus; unclassified Skunavirus
GCA_003288675.1	29374	53	Lactococcus phage vB_Llc_bIBBF13	NA	Viruses; Duplodnaviria; Heunggongvirae; Uroviricota; Caudoviricetes; Caudovirales; Siphoviridae; Skunavirus; unclassified Skunavirus
GCA_002957435.1	45221	64	Streptomyces phage WRightOn	NA	Viruses; Duplodnaviria; Heunggongvirae; Uroviricota; Caudoviricetes; Caudovirales; Podoviridae; unclassified Podoviridae
GCA_002957445.1	45177	63	Streptomyces phage Manuel	NA	Viruses; Duplodnaviria; Heunggongvirae; Uroviricota; Caudoviricetes; Caudovirales; Podoviridae; unclassified Podoviridae
GCA_002957455.1	46094	62	Streptomyces phage Immanuel3	NA	Viruses; Duplodnaviria; Heunggongvirae; Uroviricota; Caudoviricetes; Caudovirales; Podoviridae; unclassified Podoviridae
GCA_002957465.1	6945	12	Ralstonia virus RSIBR1	NA	Viruses; Monodnaviria; Loebvirae; Hofneiviricota; Faserviricetes; Tubulavirales; Inoviridae; Habenivirus; unclassified Habenivirus
GCA_004130395.1	112307	93	Tomelloso virus	NA	Viruses; Nudiviridae; unclassified Nudiviridae
GCA_004130735.1	147790	84	Rhinolophus gammaherpesvirus 1	BV1	Viruses; Duplodnaviria; Heunggongvirae; Peploviricota; Herviviricetes; Herpesvirales; Herpesviridae; Gammaherpesvirinae; unclassified Gammaherpesvirinae
GCA_002957475.1	159631	214	Klebsiella phage May	NA	Viruses; Duplodnaviria; Heunggongvirae; Uroviricota; Caudoviricetes; Caudovirales; Ackermannviridae; Taipeivirus; Klebsiella virus May
GCA_002957485.1	157281	215	Klebsiella phage Menlow	NA	Viruses; Duplodnaviria; Heunggongvirae; Uroviricota; Caudoviricetes; Caudovirales; Ackermannviridae; Taipeivirus; Klebsiella virus Menlow
GCA_002957495.1	161502	218	Salmonella phage Mutine	NA	Viruses; Duplodnaviria; Heunggongvirae; Uroviricota; Caudoviricetes; Caudovirales; Ackermannviridae; Cvivirinae; Kuttervirus; unclassified Kuttervirus
GCA_004131505.1	5890	8	Appleassociated luteovirus	NA	Viruses; Riboviria; Orthornavirae; Kitrinoviricota; Tolucaviricetes; Tolivirales; Luteoviridae; Luteovirus

gca	genome.size	proteome.size	phage.name	strain	Taxonomy
GCA_004131385.1	2538	3	Rodent Torque teno virus 3	NA	Viruses; Anelloviridae; unclassified Anelloviridae
GCA_004131365.1	2561	1	Rodent Torque teno virus 4	NA	Viruses; Anelloviridae; unclassified Anelloviridae
GCA_004131425.1	2505	3	Rodent Torque teno virus 6	NA	Viruses; Anelloviridae; unclassified Anelloviridae
GCA_004131445.1	2379	1	Rodent Torque teno virus 7	NA	Viruses; Anelloviridae; unclassified Anelloviridae
GCA_018583045.1	2325	2	Rodent Torque teno virus 8	NA	Viruses; Anelloviridae; Wawtorquevirus; Torque teno rodent virus 6
GCA_018583035.1	2605	3	Torque teno didelphis albiventris virus	NA	Viruses; Anelloviridae; Xitorquevirus; Torque teno didelphi virus 1
GCA_003861715.1	140358	224	Staphylococcus phage HSA30	NA	Viruses; Duplodnaviria; Heunggongvirae; Uroviricota; Caudoviricetes; Caudovirales; Herelleviridae; Twortvirinae; Kayvirus; unclassified Kayvirus
GCA_002831445.1	9932	1	Chequa iflavirus	NA	Viruses; Riboviria; Orthornavirae; Pisuviricota; Pisoniviricetes; Picornavirales; Iflaviridae; unclassified Iflaviridae
GCA_900291915.2	50547	76	Pseudomonas phage GP100	NA	Viruses; Duplodnaviria; Heunggongvirae; Uroviricota; Caudoviricetes; Caudovirales; Podoviridae; unclassified Podoviridae
GCA_004285615.1	3624	5	Anelloviridae sp	NA	Viruses; Anelloviridae; unclassified Anelloviridae
GCA_005280455.2	42551	69	Pseudomonas phage HU1	NA	Viruses; Duplodnaviria; Heunggongvirae; Uroviricota; Caudoviricetes; Caudovirales; Podoviridae; unclassified Podoviridae
GCA_004788695.1	9646	1	African eggplant mosaic virus	NA	Viruses; Riboviria; Orthornavirae; Pisuviricota; Stelpaviricetes; Patatavirales; Potyviridae; Potyvirus
GCA_002957505.1	23219	38	Sulfolobus ellipsoid virus 1	NA	Viruses; Ovaliviridae; Alphaovalivirus
GCA_004129835.1	9552	1	Varroa destructor virus 2	IL	Viruses; Riboviria; Orthornavirae; Pisuviricota; Pisoniviricetes; Picornavirales; Iflaviridae; unclassified Iflaviridae
GCA_003727455.1	4146	3	Varroa destructor virus 3	IL	Viruses; unclassified viruses
GCA_004117295.1	7896	8	Cow vetch latent virus	NA	Viruses; Monodnaviria; Shotokuvirae; Cressdnaviricota; Arfiviricetes; Mulpavirales; Nanoviridae; unclassified Nanoviridae
GCA_013087755.1	1035	1	Cow vetch latent virus alphasatellite	NA	Viruses; Alphasatellitidae; Nanoalphasatellitinae; unclassified Nanoalphasatellitinae
GCA_004131865.1	7215	4	Cacao Bacilliform SriLanka Virus	NA	Viruses; Riboviria; Pararnavirae; Artverviricota; Revtraviricetes; Ortervirales; Caulimoviridae; Badnavirus; unclassified Badnavirus
GCA_004131745.1	7131	4	Cacao swollen shoot CE virus	NA	Viruses; Riboviria; Pararnavirae; Artverviricota; Revtraviricetes; Ortervirales; Caulimoviridae; Badnavirus
GCA_004131765.1	7167	5	Cacao swollen shoot Ghana J virus	NA	Viruses; Riboviria; Pararnavirae; Artverviricota; Revtraviricetes; Ortervirales; Caulimoviridae; Badnavirus; unclassified Badnavirus
GCA_004131785.1	7119	5	Cacao swollen shoot Ghana K virus	NA	Viruses; Riboviria; Pararnavirae; Artverviricota; Revtraviricetes; Ortervirales; Caulimoviridae; Badnavirus; unclassified Badnavirus
GCA_004131805.1	6994	5	Cacao swollen shoot Ghana L virus	NA	Viruses; Riboviria; Pararnavirae; Artverviricota; Revtraviricetes; Ortervirales; Caulimoviridae; Badnavirus; unclassified Badnavirus
GCA_004788595.1	7009	4	Cacao swollen shoot Ghana M virus	NA	Viruses; Riboviria; Pararnavirae; Artverviricota; Revtraviricetes; Ortervirales; Caulimoviridae; Badnavirus
GCA_004131825.1	7173	4	Cacao swollen shoot Ghana N virus	NA	Viruses; Riboviria; Pararnavirae; Artverviricota; Revtraviricetes; Ortervirales; Caulimoviridae; Badnavirus
GCA_004788615.1	7102	4	Cacao swollen shoot Ghana Q virus	NA	Viruses; Riboviria; Pararnavirae; Artverviricota; Revtraviricetes; Ortervirales; Caulimoviridae; Badnavirus
GCA_004131845.1	6985	4	Cacao swollen shoot Ghana R virus	NA	Viruses; Riboviria; Pararnavirae; Artverviricota; Revtraviricetes; Ortervirales; Caulimoviridae; Badnavirus; unclassified Badnavirus
GCA_004132165.1	155555	106	Drosophila innubila nudivirus	NA	Viruses; Nudiviridae; unclassified Nudiviridae
GCA_002890095.1	4990	3	Maizeassociated totivirus 3	NA	Viruses; Riboviria; Orthornavirae; Duplornaviricota; Chrymotiviricetes; Ghabrivirales; Totiviridae; unclassified Totiviridae
GCA_014883455.1	3054	3	Human respiratory circular DNA virus	NA	Viruses; Monodnaviria; Shotokuvirae; Cressdnaviricota; Arfiviricetes; Cirlivirales; Circoviridae; unclassified Circoviridae
GCA_018580825.1	2880	3	Torque teno mini virus SHA	NA	Viruses; Anelloviridae; Betatorquevirus; Torque teno mini virus 26
GCA_002890135.1	2255	3	Pleurochrysis carterae circular virus	NA	Viruses; Monodnaviria; Shotokuvirae; Cressdnaviricota; Arfiviricetes; Cirlivirales; Circoviridae; unclassified Circoviridae
GCA_004132845.1	7864	4	Birch leaf rollassociated virus	NA	Viruses; Riboviria; Pararnavirae; Artverviricota; Revtraviricetes; Ortervirales; Caulimoviridae; Badnavirus
GCA_018595565.1	10346	4	Red clover varicosavirus	NA	Viruses; Riboviria; Orthornavirae; Negarnaviricota; Haploviricotina; Monjiviricetes; Mononegavirales; Rhabdoviridae; unclassified Rhabdoviridae
GCA_004117435.1	12008	2	Red clover nepovirus A	NA	Viruses; Riboviria; Orthornavirae; Pisuviricota; Pisoniviricetes; Picornavirales; Secoviridae; Comovirinae; Nepovirus; unclassified Nepovirus

gca	genome.size	proteome.size	phage.name	strain	Taxonomy
GCA_004130375.1	8145	1	Pelodiscus sinensis picornavirus 1	CNSR2011	Viruses; Riboviria; Orthornavirae; Pisuviricota; Pisoniviricetes; Picornavirales; Picornaviridae; unclassified Picornaviridae
GCA_004788515.1	9660	1	Paris mosaic necrosis virus	NA	Viruses; Riboviria; Orthornavirae; Pisuviricota; Stelpaviricetes; Patatavirales; Potyviridae; Potyvirus
GCA_004788675.1	9560	1	Mediterranean ruda virus	NA	Viruses; Riboviria; Orthornavirae; Pisuviricota; Stelpaviricetes; Patatavirales; Potyviridae; Potyvirus
GCA_000846225.1	59471	112	Mycobacterium virus Che8	NA	Viruses; Duplodnaviria; Heunggongvirae; Uroviricota; Caudoviricetes; Caudovirales; Siphoviridae; Cheoctovirus
GCA_000840565.1	75931	141	Mycobacterium virus CJW1	NA	Viruses; Duplodnaviria; Heunggongvirae; Uroviricota; Caudoviricetes; Caudovirales; Siphoviridae; Kostyavirus
GCA_000841385.2	50913	86	Mycobacterium virus Bxz2	NA	Viruses; Duplodnaviria; Heunggongvirae; Uroviricota; Caudoviricetes; Caudovirales; Siphoviridae; Fromanvirus
GCA_000841365.1	57050	84	Mycobacterium virus Che9c	NA	Viruses; Duplodnaviria; Heunggongvirae; Uroviricota; Caudoviricetes; Caudovirales; Siphoviridae; Chenonavirus
GCA_000842345.1	67480	90	Mycobacterium phage Rosebush	NA	Viruses; dsDNA viruses, no RNA stage; Caudovirales; Siphoviridae; Pg1virus
GCA_000843065.1	69777	122	Mycobacterium virus Corndog	NA	Viruses; Duplodnaviria; Heunggongvirae; Uroviricota; Caudoviricetes; Caudovirales; Siphoviridae; Corndogvirus
GCA_003729695.1	3478	2	Idotea virus IWaV278	NA	Viruses; unclassified viruses; unclassified DNA viruses; unclassified ssDNA viruses; Cruciviridae
GCA_000840885.2	56275	111	Mycobacterium virus Che9d	NA	Viruses; Duplodnaviria; Heunggongvirae; Uroviricota; Caudoviricetes; Caudovirales; Siphoviridae; Cheoctovirus
GCA_000842365.1	156102	225	Mycobacterium phage Bxz1	NA	Viruses; dsDNA viruses, no RNA stage; Caudovirales; Myoviridae; Bxz1virus; unclassified Bxz1virus
GCA_002890315.1	3453	2	Allium cepa amalgavirus 1	AcAV1OH1	Viruses; Riboviria; Orthornavirae; Pisuviricota; Duplopiviricetes; Durnavirales; Amalgaviridae; Amalgavirus
GCA_002889815.1	3453	2	Allium cepa amalgavirus 2	AcAV2DH5225	Viruses; Riboviria; Orthornavirae; Pisuviricota; Duplopiviricetes; Durnavirales; Amalgaviridae; Amalgavirus
GCA_000842205.1	110865	237	Mycobacterium virus Omega	NA	Viruses; Duplodnaviria; Heunggongvirae; Uroviricota; Caudoviricetes; Caudovirales; Siphoviridae; Omegavirus
GCA_000840585.1	70797	109	Mycobacterium phage Barnyard	NA	Viruses; dsDNA viruses, no RNA stage; Caudovirales; Siphoviridae; Barnyardvirus
GCA_018594925.1	18335	3	Vinegar Hill virus	NA	Viruses; Riboviria; Orthornavirae; Negarnaviricota; Polyploviricotina; Ellioviricetes; Bunyavirales; Nairoviridae; Orthonairovirus; Vinegar Hill orthonairovirus
GCA_004127975.1	10617	4	Apore mammarenavirus	LBCE 12071	Viruses; Riboviria; Orthornavirae; Negarnaviricota; Polyploviricotina; Ellioviricetes; Bunyavirales; Arenaviridae; Mammarenavirus; unclassified Mammarenavirus
GCA_002889855.1	293123	256	Flamingopox virus FGPVKD09	NA	Viruses; Varidnaviria; Bamfordvirae; Nucleocytoviricota; Pokkesviricetes; Chitovirales; Poxviridae; Chordopoxvirinae; Avipoxvirus; unclassified Avipoxvirus
GCA_014840115.1	29632	43	Siphovirus 29632	NA	Viruses; Duplodnaviria; Heunggongvirae; Uroviricota; Caudoviricetes; Caudovirales; Siphoviridae; unclassified Siphoviridae
GCA_003991685.1	142567	105	Lactobacillus phage Lpa804	NA	Viruses; Duplodnaviria; Heunggongvirae; Uroviricota; Caudoviricetes; Caudovirales; Herelleviridae; Twortvirinae; Harbinvirus; Lactobacillus virus Lpa804
GCA_003085775.1	148884	239	Staphylococcus phage B1	Staphylococcus aureus DPC5246	Viruses; Duplodnaviria; Heunggongvirae; Uroviricota; Caudoviricetes; Caudovirales; Herelleviridae; Twortvirinae; Kayvirus; unclassified Kayvirus
GCA_002957515.2	47820	76	Pseudomonas phage PMBT14	NA	Viruses; Duplodnaviria; Heunggongvirae; Uroviricota; Caudoviricetes; Caudovirales; Siphoviridae; unclassified Siphoviridae
GCA_002957525.1	87196	116	Pseudomonas phage PMBT3	NA	Viruses; Duplodnaviria; Heunggongvirae; Uroviricota; Caudoviricetes; Caudovirales; Siphoviridae; Maxrubnervirus; Pseudomonas virus PMBT3
GCA_003861735.1	42650	60	Staphylococcus phage HSA84	NA	Viruses; Duplodnaviria; Heunggongvirae; Uroviricota; Caudoviricetes; Caudovirales; Siphoviridae; Phietavirus; unclassified Phietavirus
GCA_002957535.1	112127	147	Vibrio phage Vp_R1	NA	Viruses; Duplodnaviria; Heunggongvirae; Uroviricota; Caudoviricetes; Caudovirales; Podoviridae; unclassified Podoviridae
GCA_003979555.1	76423	102	Vibrio phage VVP001	NA	Viruses; Duplodnaviria; Heunggongvirae; Uroviricota; Caudoviricetes; Caudovirales; Siphoviridae; Mardecavirus; unclassified Mardecavirus
GCA_003979575.1	157958	224	Bacillus phage BCP01	NA	Viruses; Duplodnaviria; Heunggongvirae; Uroviricota; Caudoviricetes; Caudovirales; Herelleviridae; Bastillevirinae; Caeruleovirus; unclassified Caeruleovirus
GCA_002957545.1	44603	55	Vibrio phage VEN	NA	Viruses; Duplodnaviria; Heunggongvirae; Uroviricota; Caudoviricetes; Caudovirales; Autographiviridae; Colwellvirinae; Trungvirus; Vibrio virus VEN
GCA_002957555.1	42551	59	Streptomyces phage AbbeyMikolon	NA	Viruses; Duplodnaviria; Heunggongvirae; Uroviricota; Caudoviricetes; Caudovirales; Siphoviridae; Abbeymikolonvirus; Streptomyces virus AbbeyMikolon
GCA_002957565.1	41872	53	Streptomyces phage Attoomi	NA	Viruses; Duplodnaviria; Heunggongvirae; Uroviricota; Caudoviricetes; Caudovirales; Siphoviridae; Attoomivirus; Streptomyces virus Attoomi
GCA_002957575.1	49299	80	Streptomyces phage Omar	NA	Viruses; Duplodnaviria; Heunggongvirae; Uroviricota; Caudoviricetes; Caudovirales; Siphoviridae; Arquatrovirinae; Arequatrovirus; unclassified Arequatrovirus
GCA_002957585.1	42890	62	Streptomyces phage Rowa	NA	Viruses; Duplodnaviria; Heunggongvirae; Uroviricota; Caudoviricetes; Caudovirales; Siphoviridae; Rowavirus; Streptomyces virus Rowa
GCA_002957595.1	46135	64	Streptomyces phage HaugeAnator	NA	Viruses; Duplodnaviria; Heunggongvirae; Uroviricota; Caudoviricetes; Caudovirales; Podoviridae; unclassified Podoviridae

gca	genome.size	proteome.size	phage.name	strain	Taxonomy
GCA_002957605.1	45999	64	Streptomyces phage Percastrophe	NA	Viruses; Duplodnaviria; Heunggongvirae; Uroviricota; Caudoviricetes; Caudovirales; Podoviridae; unclassified Podoviridae
GCA_002957615.1	46079	64	Streptomyces phage Romero	NA	Viruses; Duplodnaviria; Heunggongvirae; Uroviricota; Caudoviricetes; Caudovirales; Podoviridae; unclassified Podoviridae
GCA_002957625.1	46077	64	Streptomyces phage ToriToki	NA	Viruses; Duplodnaviria; Heunggongvirae; Uroviricota; Caudoviricetes; Caudovirales; Podoviridae; unclassified Podoviridae
GCA_002957635.1	46135	64	Streptomyces phage ZooBear	NA	Viruses; Duplodnaviria; Heunggongvirae; Uroviricota; Caudoviricetes; Caudovirales; Podoviridae; unclassified Podoviridae
GCA_002990175.1	41135	41	Ralstonia phage RsoP1IDN	NA	Viruses; Duplodnaviria; Heunggongvirae; Uroviricota; Caudoviricetes; Caudovirales; Autographiviridae; Okabevirinae; Higashivirus; Ralstonia virus RsoP1IDN
GCA_002957665.1	39826	57	Vibrio phage Athena1	NA	Viruses; Duplodnaviria; Heunggongvirae; Uroviricota; Caudoviricetes; Caudovirales; Myoviridae; unclassified Myoviridae
GCA_003364355.1	44283	65	Escherichia phage B2	NA	Viruses; Duplodnaviria; Heunggongvirae; Uroviricota; Caudoviricetes; Caudovirales; Siphoviridae; Dhillonvirus; unclassified Dhillonvirus
GCA_002957675.1	5489	11	Escherichia phage EMCL318	NA	Viruses; Monodnaviria; Sangervirae; Phixviricota; Malgrandaviricetes; Petitvirales; Microviridae; Bullavirinae; Gequatrovirus; unclassified Gequatrovirus
GCA_002957685.1	43797	62	Salmonella phage BPS11T2	NA	Viruses; Duplodnaviria; Heunggongvirae; Uroviricota; Caudoviricetes; Caudovirales; Siphoviridae; Guernseyvirinae; Jerseyvirus; unclassified Jerseyvirus
GCA_002957695.1	87609	130	Salmonella phage BPS15S6	NA	Viruses; Duplodnaviria; Heunggongvirae; Uroviricota; Caudoviricetes; Caudovirales; Myoviridae; Ounavirinae; Felixounavirus; unclassified Felixounavirus
GCA_002957705.1	84916	126	Salmonella phage BPS17L1	NA	Viruses; Duplodnaviria; Heunggongvirae; Uroviricota; Caudoviricetes; Caudovirales; Myoviridae; Ounavirinae; Felixounavirus; Salmonella virus BPS17L1
GCA_002957715.1	87628	129	Salmonella phage BPS17S6	NA	Viruses; Duplodnaviria; Heunggongvirae; Uroviricota; Caudoviricetes; Caudovirales; Myoviridae; Ounavirinae; Felixounavirus; unclassified Felixounavirus
GCA_002957725.1	87609	129	Salmonella phage BPS17W1	NA	Viruses; Duplodnaviria; Heunggongvirae; Uroviricota; Caudoviricetes; Caudovirales; Myoviridae; Ounavirinae; Felixounavirus; Salmonella virus BPS17W1
GCA_002957735.1	272228	320	Erwinia phage vB_EamM_Bosolaphorus	NA	Viruses; Duplodnaviria; Heunggongvirae; Uroviricota; Caudoviricetes; Caudovirales; Myoviridae; Agricanvirus; unclassified Agricanvirus
GCA_002957745.1	272458	320	Erwinia phage vB_EamM_Desertfox	NA	Viruses; Duplodnaviria; Heunggongvirae; Uroviricota; Caudoviricetes; Caudovirales; Myoviridae; Agricanvirus; Erwinia virus Desertfox
GCA_002957755.1	275000	321	Erwinia phage vB_EamM_MadMel	NA	Viruses; Duplodnaviria; Heunggongvirae; Uroviricota; Caudoviricetes; Caudovirales; Myoviridae; Agricanvirus; unclassified Agricanvirus
GCA_002957765.1	273914	324	Erwinia phage vB_EamM_Mortimer	NA	Viruses; Duplodnaviria; Heunggongvirae; Uroviricota; Caudoviricetes; Caudovirales; Myoviridae; Agricanvirus; unclassified Agricanvirus
GCA_002890115.1	2873	6	Tomato apical leaf curl virus	NA	Viruses; Monodnaviria; Shotokuvirae; Cressdnaviricota; Repensiviricetes; Geplafuvirales; Geminiviridae; unclassified Geminiviridae
GCA_002890255.1	20106	3	Dianke virus	SEN235030	Viruses; Riboviria; Orthornavirae; Pisuviricota; Pisoniviricetes; Nidovirales; Mesnidovirineae; Mesoniviridae; Hexponivirinae; Alphamesonivirus; Namcalivirus; Alphamesonivirus 10
GCA_002890015.1	6692	5	Babaco mosaic virus	NA	Viruses; Riboviria; Orthornavirae; Kitrinoviricota; Alsuviricetes; Tymovirales; Alphaflexiviridae; Potexvirus; unclassified Potexvirus
GCA_003057795.1	668031	653	Tetraselmis virus 1	NA	Viruses; Varidnaviria; Bamfordvirae; Nucleocytoviricota; Megaviricetes; Algavirales; Phycodnaviridae; unclassified Phycodnaviridae
GCA_003094415.1	167812	280	Escherichia phage PP01	NA	Viruses; Duplodnaviria; Heunggongvirae; Uroviricota; Caudoviricetes; Caudovirales; Myoviridae; Tevenvirinae; Tequatrovirus; unclassified Tequatrovirus
GCA_003094435.1	163825	276	Escherichia phage T2	NA	Viruses; Duplodnaviria; Heunggongvirae; Uroviricota; Caudoviricetes; Caudovirales; Myoviridae; Tevenvirinae; Tequatrovirus; unclassified Tequatrovirus
GCA_002890645.1	5994	2	Fusarium graminearum alternavirus 1	NA	Viruses; unclassified viruses
GCA_002957775.1	50249	78	Klebsiella phage GMLKpCol1	NA	Viruses; Duplodnaviria; Heunggongvirae; Uroviricota; Caudoviricetes; Caudovirales; Drexlerviridae; Webervirus; Klebsiella virus KpCol1
GCA_002957785.1	43420	58	Acinetobacter phage SHAb 15497	NA	Viruses; Duplodnaviria; Heunggongvirae; Uroviricota; Caudoviricetes; Caudovirales; Siphoviridae; unclassified Siphoviridae
GCA_002957795.1	49796	71	Pseudomonas phage TC6	NA	Viruses; Duplodnaviria; Heunggongvirae; Uroviricota; Caudoviricetes; Caudovirales; Podoviridae; unclassified Podoviridae
GCA_002957805.1	45621	65	Staphylococcus phage phiSa2wa_st121mssa	NA	Viruses; Duplodnaviria; Heunggongvirae; Uroviricota; Caudoviricetes; Caudovirales; Siphoviridae; Triavirus; unclassified Triavirus
GCA_002957815.1	38576	53	Staphylococcus phage phiSa2wa_st22	NA	Viruses; Duplodnaviria; Heunggongvirae; Uroviricota; Caudoviricetes; Caudovirales; Siphoviridae; Biseptimavirus; unclassified Biseptimavirus
GCA_002957825.1	45780	65	Staphylococcus phage phiSa2wa_st30	NA	Viruses; Duplodnaviria; Heunggongvirae; Uroviricota; Caudoviricetes; Caudovirales; Siphoviridae; Triavirus; unclassified Triavirus
GCA_002957835.1	44823	77	Staphylococcus phage phiSa2wa_st5	NA	Viruses; Duplodnaviria; Heunggongvirae; Uroviricota; Caudoviricetes; Caudovirales; Siphoviridae; Triavirus; unclassified Triavirus
GCA_002957845.1	42133	72	Staphylococcus phage phiSa2wa_st59	NA	Viruses; Duplodnaviria; Heunggongvirae; Uroviricota; Caudoviricetes; Caudovirales; Siphoviridae; Biseptimavirus; unclassified Biseptimavirus
GCA_002957855.1	47213	76	Staphylococcus phage phiSa2wa_st72	NA	Viruses; Duplodnaviria; Heunggongvirae; Uroviricota; Caudoviricetes; Caudovirales; Siphoviridae; Triavirus; unclassified Triavirus
GCA_002957865.1	45878	67	Staphylococcus phage phiSa2wa_st78	NA	Viruses; Duplodnaviria; Heunggongvirae; Uroviricota; Caudoviricetes; Caudovirales; Siphoviridae; Triavirus; unclassified Triavirus

gca	genome.size	proteome.size	phage.name	strain	Taxonomy
GCA_002957875.1	45164	67	Staphylococcus phage phiSa2wa_st80	NA	Viruses; Duplodnaviria; Heunggongvirae; Uroviricota; Caudoviricetes; Caudovirales; Siphoviridae; Triavirus; unclassified Triavirus
GCA_002957885.1	45913	67	Staphylococcus phage phiSa2wa_st93	NA	Viruses; Duplodnaviria; Heunggongvirae; Uroviricota; Caudoviricetes; Caudovirales; Siphoviridae; Triavirus; unclassified Triavirus
GCA_002957895.1	45913	71	Staphylococcus phage phiSa2wa_st93mssa	NA	Viruses; Duplodnaviria; Heunggongvirae; Uroviricota; Caudoviricetes; Caudovirales; Siphoviridae; Triavirus; unclassified Triavirus
GCA_013086675.1	12462	4	Sandfly fever Naples virus	NA	Viruses; ssRNA viruses; ssRNA negative-strand viruses; Bunyaviridae; Phlebovirus; Sandfly fever Naples phlebovirus
GCA_013086685.1	11966	4	Tehran virus	I47	Viruses; Riboviria; Orthornavirae; Negarnaviricota; Polyploviricotina; Ellioviricetes; Bunyavirales; Phenuiviridae; Phlebovirus; Tehran phlebovirus
GCA_018594795.1	12486	4	Corfou virus	Pa Ar 814	Viruses; Riboviria; Orthornavirae; Negarnaviricota; Polyploviricotina; Ellioviricetes; Bunyavirales; Phenuiviridae; Phlebovirus; Corfou phlebovirus
GCA_013086695.1	12674	4	Buenaventura virus	CoAr3319	Viruses; Riboviria; Orthornavirae; Negarnaviricota; Polyploviricotina; Ellioviricetes; Bunyavirales; Phenuiviridae; Phlebovirus; Buenaventura phlebovirus
GCA_006433575.1	34433	24	Rhesus adenovirus 54	NA	Viruses; Varidnaviria; Bamfordvirae; Preplasmiviricota; Tectiliviricetes; Rowavirales; Adenoviridae; Mastadenovirus; unclassified Simian adenoviruses
GCA_006433555.1	35178	24	Rhesus adenovirus 55	NA	Viruses; Varidnaviria; Bamfordvirae; Preplasmiviricota; Tectiliviricetes; Rowavirales; Adenoviridae; Mastadenovirus; unclassified Simian adenoviruses
GCA_006433535.1	34449	24	Rhesus adenovirus 56	NA	Viruses; Varidnaviria; Bamfordvirae; Preplasmiviricota; Tectiliviricetes; Rowavirales; Adenoviridae; Mastadenovirus; unclassified Simian adenoviruses
GCA_006433515.1	35094	24	Rhesus adenovirus 57	NA	Viruses; Varidnaviria; Bamfordvirae; Preplasmiviricota; Tectiliviricetes; Rowavirales; Adenoviridae; Mastadenovirus; unclassified Simian adenoviruses
GCA_006433495.1	34364	23	Rhesus adenovirus 58	NA	Viruses; Varidnaviria; Bamfordvirae; Preplasmiviricota; Tectiliviricetes; Rowavirales; Adenoviridae; Mastadenovirus; unclassified Simian adenoviruses
GCA_006433475.1	35126	24	Rhesus adenovirus 59	NA	Viruses; Varidnaviria; Bamfordvirae; Preplasmiviricota; Tectiliviricetes; Rowavirales; Adenoviridae; Mastadenovirus; unclassified Simian adenoviruses
GCA_006433455.1	35182	24	Rhesus adenovirus 60	NA	Viruses; Varidnaviria; Bamfordvirae; Preplasmiviricota; Tectiliviricetes; Rowavirales; Adenoviridae; Mastadenovirus; unclassified Simian adenoviruses
GCA_006433435.1	35192	23	Rhesus adenovirus 61	NA	Viruses; Varidnaviria; Bamfordvirae; Preplasmiviricota; Tectiliviricetes; Rowavirales; Adenoviridae; Mastadenovirus; unclassified Simian adenoviruses
GCA_006433415.1	34466	20	Rhesus adenovirus 62	NA	Viruses; Varidnaviria; Bamfordvirae; Preplasmiviricota; Tectiliviricetes; Rowavirales; Adenoviridae; Mastadenovirus; unclassified Simian adenoviruses
GCA_006426875.1	35052	24	Rhesus adenovirus 63	NA	Viruses; Varidnaviria; Bamfordvirae; Preplasmiviricota; Tectiliviricetes; Rowavirales; Adenoviridae; Mastadenovirus; unclassified Simian adenoviruses
GCA_006426815.1	35213	24	Rhesus adenovirus 64	NA	Viruses; Varidnaviria; Bamfordvirae; Preplasmiviricota; Tectiliviricetes; Rowavirales; Adenoviridae; Mastadenovirus; unclassified Simian adenoviruses
GCA_006426755.1	35252	23	Rhesus adenovirus 65	NA	Viruses; Varidnaviria; Bamfordvirae; Preplasmiviricota; Tectiliviricetes; Rowavirales; Adenoviridae; Mastadenovirus; unclassified Simian adenoviruses
GCA_006426715.1	35158	24	Rhesus adenovirus 66	NA	Viruses; Varidnaviria; Bamfordvirae; Preplasmiviricota; Tectiliviricetes; Rowavirales; Adenoviridae; Mastadenovirus; unclassified Simian adenoviruses
GCA_006426675.1	35125	24	Rhesus adenovirus 67	NA	Viruses; Varidnaviria; Bamfordvirae; Preplasmiviricota; Tectiliviricetes; Rowavirales; Adenoviridae; Mastadenovirus; unclassified Simian adenoviruses
GCA_002817955.1	6532	2	Bombyx mori latent virus	NA	Viruses; Riboviria; Orthornavirae; Kitrinoviricota; Alsuviricetes; Tymovirales; Tymoviridae
GCA_002818445.1	2887	4	Torque teno mini virus 10	NA	Viruses; Anelloviridae; Betatorquevirus
GCA_002818465.1	2871	3	Torque teno mini virus 11	NA	Viruses; Anelloviridae; Betatorquevirus
GCA_002818485.1	2912	4	Torque teno mini virus 12	NA	Viruses; Anelloviridae; Betatorquevirus
GCA_002818525.1	2019	3	Torque teno felis virus 2	NA	Viruses; Anelloviridae; Etatorquevirus
GCA_002818545.1	2002	2	Torque teno midi virus 3	NA	Viruses; Anelloviridae; Gammatorquevirus
GCA_002818565.1	2454	3	Torque teno midi virus 4	NA	Viruses; Anelloviridae; Gammatorquevirus
GCA_002818585.1	3208	4	Torque teno midi virus 5	NA	Viruses; Anelloviridae; Gammatorquevirus
GCA_002818605.1	3198	4	Torque teno midi virus 6	NA	Viruses; Anelloviridae; Gammatorquevirus
GCA_002818625.1	3197	4	Torque teno midi virus 7	NA	Viruses; Anelloviridae; Gammatorquevirus
GCA_002818645.1	3223	4	Torque teno midi virus 8	NA	Viruses; Anelloviridae; Gammatorquevirus
GCA_002818665.1	3187	4	Torque teno midi virus 9	NA	Viruses; Anelloviridae; Gammatorquevirus
GCA_002818685.1	3175	4	Torque teno midi virus 10	NA	Viruses; Anelloviridae; Gammatorquevirus

gca	genome.size	proteome.size	phage.name	strain	Taxonomy
GCA_002818705.1	3198	4	Torque teno midi virus 11	NA	Viruses; Anelloviridae; Gammatorquevirus
GCA_002818725.1	3187	4	Torque teno midi virus 12	NA	Viruses; Anelloviridae; Gammatorquevirus
GCA_002818745.1	3230	4	Torque teno midi virus 13	NA	Viruses; Anelloviridae; Gammatorquevirus
GCA_002818765.1	3223	4	Torque teno midi virus 14	NA	Viruses; Anelloviridae; Gammatorquevirus
GCA_002818785.1	3257	4	Torque teno midi virus 15	NA	Viruses; Anelloviridae; Gammatorquevirus
GCA_002957905.1	68521	95	Escherichia phage LAMP	NA	Viruses; Duplodnaviria; Heunggongvirae; Uroviricota; Caudoviricetes; Caudovirales; Podoviridae; Kuravirus; unclassified Kuravirus
GCA_002957915.1	41593	66	Microbacterium phage Dismas	NA	Viruses; Duplodnaviria; Heunggongvirae; Uroviricota; Caudoviricetes; Caudovirales; Siphoviridae; Dismasvirus; Microbacterium virus Dismas
GCA_003059675.1	45263	67	Pseudoalteromonas phage Maelstrom	NA	Viruses; Duplodnaviria; Heunggongvirae; Uroviricota; Caudoviricetes; Caudovirales; Myoviridae; unclassified Myoviridae
GCA_002957925.1	167849	270	Proteus phage phiP43	NA	Viruses; Duplodnaviria; Heunggongvirae; Uroviricota; Caudoviricetes; Caudovirales; Myoviridae; Tevenvirinae; unclassified Tevenvirinae
GCA_002957935.1	39825	72	Plesiomonas phage phiP47	NA	Viruses; Duplodnaviria; Heunggongvirae; Uroviricota; Caudoviricetes; Caudovirales; Siphoviridae; unclassified Siphoviridae
GCA_002890055.1	9130	1	Passerivirus sp	waxbillDB01HUN2014	Viruses; Riboviria; Orthornavirae; Pisuviricota; Pisoniviricetes; Picornavirales; Picornaviridae; Passerivirus; unclassified Passerivirus
GCA_005931115.1	43748	70	Pseudomonas phage TC7	NA	Viruses; Duplodnaviria; Heunggongvirae; Uroviricota; Caudoviricetes; Caudovirales; Siphoviridae; unclassified Siphoviridae
GCA_004128595.1	3356	2	Anthoxanthum odoratum amalgavirus 1	AoAV1UN29855	Viruses; Riboviria; Orthornavirae; Pisuviricota; Duplopiviricetes; Durnavirales; Amalgaviridae; unclassified Amalgaviridae
GCA_002957945.1	45111	64	Staphylococcus phage SHSt 15644	NA	Viruses; Duplodnaviria; Heunggongvirae; Uroviricota; Caudoviricetes; Caudovirales; Siphoviridae; Triavirus; unclassified Triavirus
GCA_002957955.1	40945	46	Klebsiella phage SHKp 152410	NA	Viruses; Duplodnaviria; Heunggongvirae; Uroviricota; Caudoviricetes; Caudovirales; Autographiviridae; Studiervirinae; Przondovirus; Klebsiella virus SHKp152410
GCA_004117195.1	12897	5	Alternaria alternata chrysovirus 1	AaCV1	Viruses; Riboviria; Orthornavirae; Duplornaviricota; Chrymotiviricetes; Ghabrivirales; Chrysoviridae; Betachrysovirus; Alternaria alternata chrysovirus
GCA_006451045.1	163340	171	Whitetailed deer poxvirus	NA	Viruses; Varidnaviria; Bamfordvirae; Nucleocytoviricota; Pokkesviricetes; Chitovirales; Poxviridae; Chordopoxvirinae; unclassified Chordopoxvirinae
GCA_002957965.1	153767	236	Bacillus phage BSP10	NA	Viruses; Duplodnaviria; Heunggongvirae; Uroviricota; Caudoviricetes; Caudovirales; Herelleviridae; Bastillevirinae; Nitunavirus; unclassified Nitunavirus
GCA_002957975.1	42076	63	Bacillus phage BtiUFT651F	NA	Viruses; Duplodnaviria; Heunggongvirae; Uroviricota; Caudoviricetes; Caudovirales; Siphoviridae; unclassified Siphoviridae
GCA_004128695.1	3333	2	Camellia oleifera amalgavirus 1	CoAV1Xianglin4	Viruses; Riboviria; Orthornavirae; Pisuviricota; Duplopiviricetes; Durnavirales; Amalgaviridae; unclassified Amalgaviridae
GCA_004128655.1	3478	2	Capsicum annuum amalgavirus 1	CaAV1CM334	Viruses; Riboviria; Orthornavirae; Pisuviricota; Duplopiviricetes; Durnavirales; Amalgaviridae; unclassified Amalgaviridae
GCA_004128675.1	3443	2	Cleome droserifolia amalgavirus 1	CdAV1WUR	Viruses; Riboviria; Orthornavirae; Pisuviricota; Duplopiviricetes; Durnavirales; Amalgaviridae; unclassified Amalgaviridae
GCA_004128715.1	3433	2	Erigeron breviscapus amalgavirus 1	EbAV1SMMU	Viruses; Riboviria; Orthornavirae; Pisuviricota; Duplopiviricetes; Durnavirales; Amalgaviridae; unclassified Amalgaviridae
GCA_004128735.1	3408	2	Erigeron breviscapus amalgavirus 2	EbAV2SMMU	Viruses; Riboviria; Orthornavirae; Pisuviricota; Duplopiviricetes; Durnavirales; Amalgaviridae; unclassified Amalgaviridae
GCA_004128755.1	3412	2	Festuca pratensis amalgavirus 1	FpAV1Laura	Viruses; Riboviria; Orthornavirae; Pisuviricota; Duplopiviricetes; Durnavirales; Amalgaviridae; unclassified Amalgaviridae
GCA_004128775.1	3411	2	Festuca pratensis amalgavirus 2	FpAV2Laura	Viruses; Riboviria; Orthornavirae; Pisuviricota; Duplopiviricetes; Durnavirales; Amalgaviridae; unclassified Amalgaviridae
GCA_004128635.1	3423	2	Medicago sativa amalgavirus 1	MsAV1Maverick	Viruses; Riboviria; Orthornavirae; Pisuviricota; Duplopiviricetes; Durnavirales; Amalgaviridae; unclassified Amalgaviridae
GCA_004128615.1	3394	2	Phalaenopsis equestris amalgavirus 1	PeAV1JZL4566	Viruses; Riboviria; Orthornavirae; Pisuviricota; Duplopiviricetes; Durnavirales; Amalgaviridae; unclassified Amalgaviridae
GCA_002957985.1	38253	53	Streptococcus phage vB_SthS_VA214	NA	Viruses; Duplodnaviria; Heunggongvirae; Uroviricota; Caudoviricetes; Caudovirales; Siphoviridae; Moineauvirus; unclassified Moineauvirus
GCA_002957995.1	41169	56	Streptococcus phage vB_SthS_VA460	NA	Viruses; Duplodnaviria; Heunggongvirae; Uroviricota; Caudoviricetes; Caudovirales; Siphoviridae; Moineauvirus; unclassified Moineauvirus
GCA_003051285.1	134243	172	Clostridioides phage LIBA6276	NA	Viruses; Duplodnaviria; Heunggongvirae; Uroviricota; Caudoviricetes; Caudovirales; Siphoviridae; unclassified Siphoviridae
GCA_003051305.1	132508	172	Clostridioides phage LIBA2945	NA	Viruses; Duplodnaviria; Heunggongvirae; Uroviricota; Caudoviricetes; Caudovirales; Siphoviridae; unclassified Siphoviridae
GCA_003031025.1	41297	50	Ralstonia phage RsoP1EGY	NA	Viruses; Duplodnaviria; Heunggongvirae; Uroviricota; Caudoviricetes; Caudovirales; Autographiviridae; Gyeongsanvirus; Ralstonia virus RsoP1EGY
GCA_018603535.1	343806	486	Ralstonia phage RsoM2USA	NA	Viruses; Duplodnaviria; Heunggongvirae; Uroviricota; Caudoviricetes; Caudovirales; Myoviridae; unclassified Myoviridae

gca	genome.size	proteome.size	phage.name	strain	Taxonomy
GCA_002958005.1	170764	283	Klebsiella phage vB_Kpn_F48	NA	Viruses; Duplodnaviria; Heunggongvirae; Uroviricota; Caudoviricetes; Caudovirales; Myoviridae; Tevenvirinae; unclassified Tevenvirinae
GCA_002958015.1	237722	198	Vibrio phage Aphrodite1	NA	Viruses; Duplodnaviria; Heunggongvirae; Uroviricota; Caudoviricetes; Caudovirales; Myoviridae; Aphroditevirus; Vibrio virus Aphrodite1
GCA_002958025.1	80500	119	Vibrio phage Ares1	NA	Viruses; Duplodnaviria; Heunggongvirae; Uroviricota; Caudoviricetes; Caudovirales; Siphoviridae; unclassified Siphoviridae
GCA_002922455.1	9693	2	Saffron latent virus	NA	Viruses; Riboviria; Orthornavirae; Pisuviricota; Stelpaviricetes; Patatavirales; Potyviridae; Potyvirus
GCA_002958035.1	88114	88	Escherichia phage GER2	NA	Viruses; Duplodnaviria; Heunggongvirae; Uroviricota; Caudoviricetes; Caudovirales; Podoviridae; Sepvirinae; Oslovirus; unclassified Oslovirus
GCA_002958045.1	143652	227	Staphylococcus phage vB_SauM_LM12	NA	Viruses; Duplodnaviria; Heunggongvirae; Uroviricota; Caudoviricetes; Caudovirales; Herelleviridae; Twortvirinae; Kayvirus; unclassified Kayvirus
GCA_003013875.1	51894	85	Enterobacter phage Ec_L1	NA	Viruses; Duplodnaviria; Heunggongvirae; Uroviricota; Caudoviricetes; Caudovirales; Drexlerviridae; Eclunavirus; Enterobacter virus EcL1
GCA_002958055.1	61716	94	Faecalibacterium phage FP_Brigit	NA	Viruses; Duplodnaviria; Heunggongvirae; Uroviricota; Caudoviricetes; Caudovirales; Myoviridae; Brigitvirus; Faecalibacterium virus Brigit
GCA_002958065.1	49573	76	Faecalibacterium phage FP_Epona	NA	Viruses; Duplodnaviria; Heunggongvirae; Uroviricota; Caudoviricetes; Caudovirales; Myoviridae; Eponavirus; Faecalibacterium virus Epona
GCA_002958075.1	48211	65	Faecalibacterium phage FP_Lagaffe	NA	Viruses; Duplodnaviria; Heunggongvirae; Uroviricota; Caudoviricetes; Caudovirales; Myoviridae; Lagaffevirus; Faecalibacterium virus Lagaffe
GCA_002958085.1	34075	42	Faecalibacterium phage FP_Lugh	NA	Viruses; Duplodnaviria; Heunggongvirae; Uroviricota; Caudoviricetes; Caudovirales; Siphoviridae; Lughvirus; Faecalibacterium virus Lugh
GCA_002958095.1	36636	54	Faecalibacterium phage FP_Mushu	NA	Viruses; Duplodnaviria; Heunggongvirae; Uroviricota; Caudoviricetes; Caudovirales; Myoviridae; Mushuvirus; Faecalibacterium virus Mushu
GCA_002958105.1	54286	85	Faecalibacterium phage FP_Taranis	NA	Viruses; Duplodnaviria; Heunggongvirae; Uroviricota; Caudoviricetes; Caudovirales; Myoviridae; Taranisvirus; Faecalibacterium virus Taranis
GCA_002958115.1	54725	89	Faecalibacterium phage FP_Toutatis	NA	Viruses; Duplodnaviria; Heunggongvirae; Uroviricota; Caudoviricetes; Caudovirales; Myoviridae; Toutatisvirus; Faecalibacterium virus Toutatis
GCA_002958125.1	58391	84	Faecalibacterium phage FP_oengus	NA	Viruses; Duplodnaviria; Heunggongvirae; Uroviricota; Caudoviricetes; Caudovirales; Siphoviridae; Oengusvirus; Faecalibacterium virus Oengus
GCA_002958135.1	39485	73	Paenibacillus phage BN12	NA	Viruses; Duplodnaviria; Heunggongvirae; Uroviricota; Caudoviricetes; Caudovirales; Siphoviridae; Sitaravirus; unclassified Sitaravirus
GCA_002958145.1	41131	68	Paenibacillus phage Dragolir	NA	Viruses; Duplodnaviria; Heunggongvirae; Uroviricota; Caudoviricetes; Caudovirales; Siphoviridae; Vegasvirus; unclassified Vegasvirus
GCA_002958155.1	37985	63	Paenibacillus phage Kiel007	NA	Viruses; Duplodnaviria; Heunggongvirae; Uroviricota; Caudoviricetes; Caudovirales; Siphoviridae; Sitaravirus; unclassified Sitaravirus
GCA_002958165.1	42276	75	Paenibacillus phage Leyra	NA	Viruses; Duplodnaviria; Heunggongvirae; Uroviricota; Caudoviricetes; Caudovirales; Siphoviridae; Sitaravirus; unclassified Sitaravirus
GCA_002958175.1	39778	65	Paenibacillus phage Likha	NA	Viruses; Duplodnaviria; Heunggongvirae; Uroviricota; Caudoviricetes; Caudovirales; Siphoviridae; Sitaravirus; unclassified Sitaravirus
GCA_002958185.1	40611	79	Paenibacillus phage PBL1c	NA	Viruses; Duplodnaviria; Heunggongvirae; Uroviricota; Caudoviricetes; Caudovirales; Siphoviridae; Sitaravirus; unclassified Sitaravirus
GCA_002958195.1	40035	70	Paenibacillus phage Pagassa	NA	Viruses; Duplodnaviria; Heunggongvirae; Uroviricota; Caudoviricetes; Caudovirales; Siphoviridae; Sitaravirus; unclassified Sitaravirus
GCA_002958205.1	37880	65	Paenibacillus phage Tadhana	NA	Viruses; Duplodnaviria; Heunggongvirae; Uroviricota; Caudoviricetes; Caudovirales; Siphoviridae; Sitaravirus; unclassified Sitaravirus
GCA_002958215.1	41489	67	Enterococcus phage PMBT2	NA	Viruses; Duplodnaviria; Heunggongvirae; Uroviricota; Caudoviricetes; Caudovirales; Siphoviridae; Efquatrovirus; Enterococcus virus PMBT2
GCA_002958225.1	45495	49	Erwinia phage vB_EamPS2	NA	Viruses; Duplodnaviria; Heunggongvirae; Uroviricota; Caudoviricetes; Caudovirales; Autographiviridae; Molineuxvirinae; Eracentumvirus; Erwinia virus S2
GCA_003288715.1	76622	125	Escherichia phage EP335	NA	Viruses; Duplodnaviria; Heunggongvirae; Uroviricota; Caudoviricetes; Caudovirales; Podoviridae; Kuravirus; unclassified Kuravirus
GCA_003288695.1	158143	209	Escherichia phage EP75	NA	Viruses; Duplodnaviria; Heunggongvirae; Uroviricota; Caudoviricetes; Caudovirales; Ackermannviridae; Cvivirinae; Kuttervirus; unclassified Kuttervirus
GCA_002958235.1	81076	111	Lactobacillus phage Satyr	NA	Viruses; Duplodnaviria; Heunggongvirae; Uroviricota; Caudoviricetes; Caudovirales; Siphoviridae; Tybeckvirinae; Maenadvirus; Lactobacillus virus Satyr
GCA_002958245.1	167989	281	Klebsiella phage KP1	NA	Viruses; Duplodnaviria; Heunggongvirae; Uroviricota; Caudoviricetes; Caudovirales; Myoviridae; Tevenvirinae; Jiaodavirus; unclassified Jiaodavirus
GCA_002958255.1	61290	89	Mycobacterium phage D12	NA	Viruses; Duplodnaviria; Heunggongvirae; Uroviricota; Caudoviricetes; Caudovirales; Siphoviridae; unclassified Siphoviridae
GCA_002958265.1	61267	90	Mycobacterium phage PR	NA	Viruses; Duplodnaviria; Heunggongvirae; Uroviricota; Caudoviricetes; Caudovirales; Siphoviridae; unclassified Siphoviridae
GCA_003004845.1	49728	85	Mycobacterium phage A6	NA	Viruses; Duplodnaviria; Heunggongvirae; Uroviricota; Caudoviricetes; Caudovirales; Siphoviridae; Fromanvirus; unclassified Fromanvirus
GCA_003004855.1	49823	88	Mycobacterium phage GS4E	NA	Viruses; Duplodnaviria; Heunggongvirae; Uroviricota; Caudoviricetes; Caudovirales; Siphoviridae; Fromanvirus; unclassified Fromanvirus
GCA_003005755.1	51141	88	Mycobacterium phage C3	NA	Viruses; Duplodnaviria; Heunggongvirae; Uroviricota; Caudoviricetes; Caudovirales; Siphoviridae; Fromanvirus; unclassified Fromanvirus

gca	genome.size	proteome.size	phage.name	strain	Taxonomy
GCA_003004835.1	49728	85	Mycobacterium phage BK1	NA	Viruses; Duplodnaviria; Heunggongvirae; Uroviricota; Caudoviricetes; Caudovirales; Siphoviridae; Fromanvirus; unclassified Fromanvirus
GCA_002989785.1	57990	91	Mycobacterium phage Y10	NA	Viruses; Duplodnaviria; Heunggongvirae; Uroviricota; Caudoviricetes; Caudovirales; Siphoviridae; Timquatrovirus; Mycobacterium virus Fionnbarth
GCA_002958275.1	57990	91	Mycobacterium phage Y2	NA	Viruses; Duplodnaviria; Heunggongvirae; Uroviricota; Caudoviricetes; Caudovirales; Siphoviridae; Timquatrovirus; unclassified Timquatrovirus
GCA_001634575.1	134272	145	Mocis latipes granulovirus	NA	Viruses; Baculoviridae; Betabaculovirus
GCA_004130435.1	183261	87	Esparto virus	NA	Viruses; Nudiviridae; unclassified Nudiviridae
GCA_000841445.1	5455	8	Luffa yellow mosaic virus	NA	Viruses; ssDNA viruses; Geminiviridae; Begomovirus
GCA_002958285.1	41820	51	Bacillus phage Carmen17	NA	Viruses; Duplodnaviria; Heunggongvirae; Uroviricota; Caudoviricetes; Caudovirales; Siphoviridae; unclassified Siphoviridae
GCA_002990155.1	4907	5	Gokushovirus MK2017	NA	Viruses; Monodnaviria; Sangervirae; Phixviricota; Malgrandaviricetes; Petitvirales; Microviridae; Gokushovirinae; unclassified Gokushovirinae
GCA_002937245.1	5656	4	Pepper enamovirus	NA	Viruses; Riboviria; Orthornavirae; Kitrinoviricota; Tolucaviricetes; Tolivirales; Luteoviridae; Enamovirus; unclassified Enamovirus
GCA_000848225.1	12002	5	Bovine foamy virus	NA	Viruses; Riboviria; Pararnavirae; Artverviricota; Revtraviricetes; Ortervirales; Retroviridae; Spumaretrovirinae; Bovispumavirus
GCA_004132605.1	8620	2	Cherry virus Trakiya	NA	Viruses; Riboviria; Orthornavirae; Pisuviricota; Pisoniviricetes; Picornavirales; unclassified Picornavirales
GCA_002958295.1	17603	20	Staphylococcus phage vB__SauP__phiAGO13	NA	Viruses; Duplodnaviria; Heunggongvirae; Uroviricota; Caudoviricetes; Caudovirales; Podoviridae; Rakietenvirinae; Rosenblumvirus; Staphylococcus virus phiAGO13
GCA_002958305.1	17637	20	Staphylococcus phage vB__SauP__phiAGO19	NA	Viruses; Duplodnaviria; Heunggongvirae; Uroviricota; Caudoviricetes; Caudovirales; Podoviridae; Rakietenvirinae; Rosenblumvirus; unclassified Rosenblumvirus
GCA_000841185.1	43198	57	Vibrio phage VHML	NA	Viruses; dsDNA viruses, no RNA stage; Caudovirales; Myoviridae; Vhmlvirus; Vibrio virus VHML
GCA_000908075.1	155631	219	Bacillus phage phiNIT1	NA	Viruses; dsDNA viruses, no RNA stage; Caudovirales; Myoviridae; Nit1virus; Bacillus virus NIT1
GCA_000857305.1	2757	7	Croton yellow vein mosaic virus	NA	Viruses; ssDNA viruses; Geminiviridae; Begomovirus
GCA_002958315.1	45961	73	Mycobacterium phage B1	NA	Viruses; Duplodnaviria; Heunggongvirae; Uroviricota; Caudoviricetes; Caudovirales; Siphoviridae; Fromanvirus; unclassified Fromanvirus
GCA_002958325.1	51509	80	Mycobacterium phage PP	NA	Viruses; Duplodnaviria; Heunggongvirae; Uroviricota; Caudoviricetes; Caudovirales; Siphoviridae; Fromanvirus; unclassified Fromanvirus
GCA_002958335.1	46305	78	Mycobacterium phage HC	NA	Viruses; Duplodnaviria; Heunggongvirae; Uroviricota; Caudoviricetes; Caudovirales; Siphoviridae; Chebruvirinae; Brujitavirus; unclassified Brujitavirus
GCA_002958345.2	50013	76	Mycobacterium phage SWU2	NA	Viruses; Duplodnaviria; Heunggongvirae; Uroviricota; Caudoviricetes; Caudovirales; Siphoviridae; Fromanvirus; unclassified Fromanvirus
GCA_006451095.1	185664	178	Trichoplusia ni ascovirus 6b	NA	Viruses; Varidnaviria; Bamfordvirae; Nucleocytoviricota; Megaviricetes; Pimascovirales; Ascoviridae; Ascovirus; unclassified Ascovirus
GCA_900323715.1	38622	57	Yersinia phage fEV1	NA	Viruses; Duplodnaviria; Heunggongvirae; Uroviricota; Caudoviricetes; Caudovirales; Myoviridae; unclassified Myoviridae
GCA_003848565.1	2192	3	Cattle bloodassociated gemycircularvirus	BGmv001	Viruses; Monodnaviria; Shotokuvirae; Cressdnaviricota; Repensiviricetes; Geplafuvirales; Genomoviridae; Gemycircularvirus; unclassified Gemycircularvirus
GCA_004131905.1	2717	3	Cattle bloodassociated circoviruslike virus	Bvch001	Viruses; Monodnaviria; Shotokuvirae; Cressdnaviricota; Arfiviricetes; Cremevirales; Smacoviridae; Porprismacovirus; unclassified Porprismacovirus
GCA_002937255.1	2194	3	Varroa mite associated genomovirus 1	NA	Viruses; Monodnaviria; Shotokuvirae; Cressdnaviricota; Repensiviricetes; Geplafuvirales; Genomoviridae; unclassified Genomoviridae
GCA_002937265.1	1811	2	Varroa mite associated virus 1	NA	Viruses; unclassified viruses
GCA_002937275.1	7502	6	Leptonychotes weddellii papillomavirus 1	NA	Viruses; Monodnaviria; Shotokuvirae; Cossaviricota; Papovaviricetes; Zurhausenvirales; Papillomaviridae; unclassified Papillomaviridae; non-primate mammal papillomaviruses
GCA_002937285.1	7392	6	Leptonychotes weddellii papillomavirus 2	NA	Viruses; Monodnaviria; Shotokuvirae; Cossaviricota; Papovaviricetes; Zurhausenvirales; Papillomaviridae; unclassified Papillomaviridae; non-primate mammal papillomaviruses
GCA_002937295.1	7677	6	Leptonychotes weddellii papillomavirus 3	NA	Viruses; Monodnaviria; Shotokuvirae; Cossaviricota; Papovaviricetes; Zurhausenvirales; Papillomaviridae; unclassified Papillomaviridae; non-primate mammal papillomaviruses
GCA_002937305.1	7836	6	Leptonychotes weddellii papillomavirus 4	NA	Viruses; Monodnaviria; Shotokuvirae; Cossaviricota; Papovaviricetes; Zurhausenvirales; Papillomaviridae; unclassified Papillomaviridae; non-primate mammal papillomaviruses
GCA_002937315.1	7682	6	Leptonychotes weddellii papillomavirus 5	NA	Viruses; Monodnaviria; Shotokuvirae; Cossaviricota; Papovaviricetes; Zurhausenvirales; Papillomaviridae; unclassified Papillomaviridae; non-primate mammal papillomaviruses
GCA_004132685.1	7658	5	Leptonychotes weddellii papillomavirus 6	NA	Viruses; Monodnaviria; Shotokuvirae; Cossaviricota; Papovaviricetes; Zurhausenvirales; Papillomaviridae; unclassified Papillomaviridae; non-primate mammal papillomaviruses
GCA_000837525.1	155060	169	Mamestra configurata nucleopolyhedrovirus A	902	Viruses; dsDNA viruses, no RNA stage; Baculoviridae; Alphabaculovirus
GCA_002937195.1	181035	227	Dishui lake phycodnavirus 1	NA	Viruses; Varidnaviria; Bamfordvirae; Nucleocytoviricota; Megaviricetes; Algavirales; Phycodnaviridae; Prasinovirus; unclassified Prasinovirus

gca	genome.size	proteome.size	phage.name	strain	Taxonomy
GCA_004134385.1	10910	4	Culex pipiens associated Tunisia virus	NA	Viruses; Riboviria; unclassified Riboviria; unclassified ssRNA viruses; unclassified ssRNA positive-strand viruses
GCA_006083715.1	39309	55	Ralstonia phage RsoM1USA	NA	Viruses; Duplodnaviria; Heunggongvirae; Uroviricota; Caudoviricetes; Caudovirales; Myoviridae; Peduovirinae; Aresaunavirus; unclassified Aresaunavirus
GCA_002958355.1	161129	286	Bacillus phage HonestAbe	NA	Viruses; Duplodnaviria; Heunggongvirae; Uroviricota; Caudoviricetes; Caudovirales; Herelleviridae; Bastillevirinae; Wphvirus; unclassified Wphvirus
GCA_002958365.1	70857	74	Escherichia phage PMBT57	NA	Viruses; Duplodnaviria; Heunggongvirae; Uroviricota; Caudoviricetes; Caudovirales; Podoviridae; Enquatrovirus; unclassified Enquatrovirus
GCA_002990235.1	156801	201	Klebsiella phage vB_KpnM_KpS110	NA	Viruses; Duplodnaviria; Heunggongvirae; Uroviricota; Caudoviricetes; Caudovirales; Ackermannviridae; Taipeivirus; Klebsiella virus KpS110
GCA_002958375.2	44349	53	Citrobacter phage vB_CroP_CrRp3	NA	Viruses; Duplodnaviria; Heunggongvirae; Uroviricota; Caudoviricetes; Caudovirales; Autographiviridae; Molineuxvirinae; Vectrevirus; Citrobacter CrRp3
GCA_002958385.2	168403	264	Citrobacter phage vB_CroM_CrRp10	NA	Viruses; Duplodnaviria; Heunggongvirae; Uroviricota; Caudoviricetes; Caudovirales; Myoviridae; Tevenvirinae; Tequatrovirus; unclassified Tequatrovirus
GCA_003862055.1	32575	55	Lactococcus phage MP1	NA	Viruses; Duplodnaviria; Heunggongvirae; Uroviricota; Caudoviricetes; Caudovirales; Siphoviridae; Skunavirus; unclassified Skunavirus
GCA_002958395.1	76889	107	Gordonia phage Flakey	NA	Viruses; Duplodnaviria; Heunggongvirae; Uroviricota; Caudoviricetes; Caudovirales; Siphoviridae; Montyvirus; Gordonia virus Flakey
GCA_002958405.1	75687	103	Gordonia phage SteveFrench	NA	Viruses; Duplodnaviria; Heunggongvirae; Uroviricota; Caudoviricetes; Caudovirales; Siphoviridae; Montyvirus; Gordonia virus Stevefrench
GCA_002958415.1	45909	71	Gordonia phage Troje	NA	Viruses; Duplodnaviria; Heunggongvirae; Uroviricota; Caudoviricetes; Caudovirales; Siphoviridae; Emalynvirus; Gordonia virus Troje
GCA_002958425.1	68296	99	Mycobacterium phage Haimas	NA	Viruses; Duplodnaviria; Heunggongvirae; Uroviricota; Caudoviricetes; Caudovirales; Siphoviridae; Bclasvirinae; Pegunavirus; unclassified Pegunavirus
GCA_002958435.1	57159	101	Mycobacterium phage OldBen	NA	Viruses; Duplodnaviria; Heunggongvirae; Uroviricota; Caudoviricetes; Caudovirales; Siphoviridae; Cheoctovirus; unclassified Cheoctovirus
GCA_002958445.1	50832	85	Mycobacterium phage Rem711	NA	Viruses; Duplodnaviria; Heunggongvirae; Uroviricota; Caudoviricetes; Caudovirales; Siphoviridae; Trigintaduovirus; unclassified Trigintaduovirus
GCA_002958455.1	40923	52	Pseudomonas phage Henninger	NA	Viruses; Duplodnaviria; Heunggongvirae; Uroviricota; Caudoviricetes; Caudovirales; Autographiviridae; Studiervirinae; Ghunavirus; Pseudomonas virus Henninger
GCA_002958465.1	45936	69	Pseudomonas phage Bjorn	NA	Viruses; Duplodnaviria; Heunggongvirae; Uroviricota; Caudoviricetes; Caudovirales; Podoviridae; Bjornvirus; Pseudomonas virus Bjorn
GCA_002958475.1	74895	95	Pseudomonas phage Littlefix	NA	Viruses; Duplodnaviria; Heunggongvirae; Uroviricota; Caudoviricetes; Caudovirales; Podoviridae; unclassified Podoviridae
GCA_002958485.1	41651	49	Pseudomonas phage PollyC	NA	Viruses; Duplodnaviria; Heunggongvirae; Uroviricota; Caudoviricetes; Caudovirales; Autographiviridae; Pollyceevirus; Pseudomonas virus PollyC
GCA_003011835.1	69649	91	Lactobacillus phage PM411	NA	Viruses; Duplodnaviria; Heunggongvirae; Uroviricota; Caudoviricetes; Caudovirales; Siphoviridae; unclassified Siphoviridae
GCA_002958495.1	167076	274	Escherichia phage vB_EcoMfFiEco06	NA	Viruses; Duplodnaviria; Heunggongvirae; Uroviricota; Caudoviricetes; Caudovirales; Myoviridae; Tevenvirinae; Tequatrovirus; unclassified Tequatrovirus
GCA_002958505.1	167064	272	Escherichia phage vB_EcoMfHoEco02	NA	Viruses; Duplodnaviria; Heunggongvirae; Uroviricota; Caudoviricetes; Caudovirales; Myoviridae; Tevenvirinae; Tequatrovirus; unclassified Tequatrovirus
GCA_002958515.1	39482	38	Escherichia phage HZ2R8	NA	Viruses; Duplodnaviria; Heunggongvirae; Uroviricota; Caudoviricetes; Caudovirales; Autographiviridae; Studiervirinae; Teseptimavirus; Escherichia virus HZ2R8
GCA_002958525.1	59214	79	Klebsiella phage KPN N98	NA	Viruses; Duplodnaviria; Heunggongvirae; Uroviricota; Caudoviricetes; Caudovirales; Siphoviridae; unclassified Siphoviridae
GCA_002958535.1	31069	56	Pontimonas phage phiPsal1	NA	Viruses; Duplodnaviria; Heunggongvirae; Uroviricota; Caudoviricetes; Caudovirales; Siphoviridae; unclassified Siphoviridae
GCA_002958545.1	47953	73	Gordonia phage Beenie	NA	Viruses; Duplodnaviria; Heunggongvirae; Uroviricota; Caudoviricetes; Caudovirales; Siphoviridae; unclassified Siphoviridae
GCA_002958555.1	29309	42	Propionibacterium phage pa15	NA	Viruses; Duplodnaviria; Heunggongvirae; Uroviricota; Caudoviricetes; Caudovirales; Siphoviridae; Pahexavirus; unclassified Pahexavirus
GCA_002958565.1	29593	43	Propionibacterium phage pa27	NA	Viruses; Duplodnaviria; Heunggongvirae; Uroviricota; Caudoviricetes; Caudovirales; Siphoviridae; Pahexavirus; unclassified Pahexavirus
GCA_002958575.1	29733	44	Propionibacterium phage pa28	NA	Viruses; Duplodnaviria; Heunggongvirae; Uroviricota; Caudoviricetes; Caudovirales; Siphoviridae; Pahexavirus; unclassified Pahexavirus
GCA_002958585.1	29370	45	Propionibacterium phage pa293991D_1	NA	Viruses; Duplodnaviria; Heunggongvirae; Uroviricota; Caudoviricetes; Caudovirales; Siphoviridae; Pahexavirus; unclassified Pahexavirus
GCA_002958595.1	28892	44	Propionibacterium phage pa293991D_2	NA	Viruses; Duplodnaviria; Heunggongvirae; Uroviricota; Caudoviricetes; Caudovirales; Siphoviridae; Pahexavirus; unclassified Pahexavirus
GCA_002958605.1	29137	43	Propionibacterium phage pa3SS3	NA	Viruses; Duplodnaviria; Heunggongvirae; Uroviricota; Caudoviricetes; Caudovirales; Siphoviridae; Pahexavirus; unclassified Pahexavirus
GCA_002958615.1	29508	43	Propionibacterium phage pa310	NA	Viruses; Duplodnaviria; Heunggongvirae; Uroviricota; Caudoviricetes; Caudovirales; Siphoviridae; Pahexavirus; unclassified Pahexavirus
GCA_002958625.1	29135	43	Propionibacterium phage pa33	NA	Viruses; Duplodnaviria; Heunggongvirae; Uroviricota; Caudoviricetes; Caudovirales; Siphoviridae; Pahexavirus; unclassified Pahexavirus
GCA_002958635.1	29491	45	Propionibacterium phage pa35	NA	Viruses; Duplodnaviria; Heunggongvirae; Uroviricota; Caudoviricetes; Caudovirales; Siphoviridae; Pahexavirus; unclassified Pahexavirus

gca	genome.size	proteome.size	phage.name	strain	Taxonomy
GCA_002958645.1	29507	43	Propionibacterium phage pa59	NA	Viruses; Duplodnaviria; Heunggongvirae; Uroviricota; Caudoviricetes; Caudovirales; Siphoviridae; Pahexavirus; unclassified Pahexavirus
GCA_002958655.1	29307	43	Propionibacterium phage pa615	NA	Viruses; Duplodnaviria; Heunggongvirae; Uroviricota; Caudoviricetes; Caudovirales; Siphoviridae; Pahexavirus; unclassified Pahexavirus
GCA_002958665.1	29446	44	Propionibacterium phage pa63	NA	Viruses; Duplodnaviria; Heunggongvirae; Uroviricota; Caudoviricetes; Caudovirales; Siphoviridae; Pahexavirus; unclassified Pahexavirus
GCA_002958675.1	29784	43	Propionibacterium phage pa69194	NA	Viruses; Duplodnaviria; Heunggongvirae; Uroviricota; Caudoviricetes; Caudovirales; Siphoviridae; Pahexavirus; unclassified Pahexavirus
GCA_002958685.1	29784	43	Propionibacterium phage pa969194	NA	Viruses; Duplodnaviria; Heunggongvirae; Uroviricota; Caudoviricetes; Caudovirales; Siphoviridae; Pahexavirus; unclassified Pahexavirus
GCA_002958695.1	76008	108	Gordonia phage Adgers	NA	Viruses; Duplodnaviria; Heunggongvirae; Uroviricota; Caudoviricetes; Caudovirales; Siphoviridae; Montyvirus; unclassified Montyvirus
GCA_002958705.1	77497	109	Gordonia phage Boneham	NA	Viruses; Duplodnaviria; Heunggongvirae; Uroviricota; Caudoviricetes; Caudovirales; Siphoviridae; Montyvirus; unclassified Montyvirus
GCA_002958715.1	67527	96	Gordonia phage Flapper	NA	Viruses; Duplodnaviria; Heunggongvirae; Uroviricota; Caudoviricetes; Caudovirales; Siphoviridae; unclassified Siphoviridae
GCA_002958725.1	64124	86	Gordonia phage SuperSulley	NA	Viruses; Duplodnaviria; Heunggongvirae; Uroviricota; Caudoviricetes; Caudovirales; Siphoviridae; unclassified Siphoviridae
GCA_002958745.1	68869	127	Mycobacterium phage Cuke	NA	Viruses; Duplodnaviria; Heunggongvirae; Uroviricota; Caudoviricetes; Caudovirales; Siphoviridae; unclassified Siphoviridae
GCA_002958755.1	68821	104	Mycobacterium phage HighStump	NA	Viruses; Duplodnaviria; Heunggongvirae; Uroviricota; Caudoviricetes; Caudovirales; Siphoviridae; Bclasvirinae; Pegunavirus; unclassified Pegunavirus
GCA_002958765.1	68673	102	Mycobacterium phage JangoPhett	NA	Viruses; Duplodnaviria; Heunggongvirae; Uroviricota; Caudoviricetes; Caudovirales; Siphoviridae; Bclasvirinae; Pegunavirus; unclassified Pegunavirus
GCA_002958775.1	75829	149	Mycobacterium phage Kimchi	NA	Viruses; Duplodnaviria; Heunggongvirae; Uroviricota; Caudoviricetes; Caudovirales; Siphoviridae; Kostyavirus; unclassified Kostyavirus
GCA_002958785.1	50878	93	Mycobacterium phage LugYA	NA	Viruses; Duplodnaviria; Heunggongvirae; Uroviricota; Caudoviricetes; Caudovirales; Siphoviridae; Fromanvirus; unclassified Fromanvirus
GCA_002958805.1	67401	93	Mycobacterium phage Opia	NA	Viruses; Duplodnaviria; Heunggongvirae; Uroviricota; Caudoviricetes; Caudovirales; Siphoviridae; Bclasvirinae; Rosebushvirus; unclassified Rosebushvirus
GCA_002958815.1	68727	102	Mycobacterium phage PhenghisKhan	NA	Viruses; Duplodnaviria; Heunggongvirae; Uroviricota; Caudoviricetes; Caudovirales; Siphoviridae; Bclasvirinae; Pegunavirus; unclassified Pegunavirus
GCA_002958825.1	68716	103	Mycobacterium phage Phergie	NA	Viruses; Duplodnaviria; Heunggongvirae; Uroviricota; Caudoviricetes; Caudovirales; Siphoviridae; Bclasvirinae; Pegunavirus; unclassified Pegunavirus
GCA_002958835.1	64625	111	Mycobacterium phage Tesla	NA	Viruses; Duplodnaviria; Heunggongvirae; Uroviricota; Caudoviricetes; Caudovirales; Siphoviridae; Marvinvirus; unclassified Marvinvirus
GCA_002958845.1	127084	217	Streptomyces phage BillNye	NA	Viruses; Duplodnaviria; Heunggongvirae; Uroviricota; Caudoviricetes; Caudovirales; Siphoviridae; Wilnyevirus; Streptomyces virus BillNye
GCA_002958855.1	56341	87	Streptomyces phage Bing	NA	Viruses; Duplodnaviria; Heunggongvirae; Uroviricota; Caudoviricetes; Caudovirales; Siphoviridae; Bingvirus; Streptomyces virus Bing
GCA_002958865.1	51464	75	Streptomyces phage OzzyJ	NA	Viruses; Duplodnaviria; Heunggongvirae; Uroviricota; Caudoviricetes; Caudovirales; Siphoviridae; Arquatrovirinae; Likavirus; unclassified Likavirus
GCA_002958875.2	141124	180	Lactobacillus phage Bacchae	NA	Viruses; Duplodnaviria; Heunggongvirae; Uroviricota; Caudoviricetes; Caudovirales; Herelleviridae; Twortvirinae; Harbinvirus; Lactobacillus virus Bacchae
GCA_002958885.2	37921	58	Lactobacillus phage Bassarid	NA	Viruses; Duplodnaviria; Heunggongvirae; Uroviricota; Caudoviricetes; Caudovirales; Siphoviridae; Coetzeevirus; unclassified Coetzeevirus
GCA_002990195.1	78646	109	Lactobacillus phage Maenad	NA	Viruses; Duplodnaviria; Heunggongvirae; Uroviricota; Caudoviricetes; Caudovirales; Siphoviridae; Tybeckvirinae; Maenadvirus; Lactobacillus virus Maenad
GCA_002958895.1	79221	125	Lactobacillus phage Nyseid	NA	Viruses; Duplodnaviria; Heunggongvirae; Uroviricota; Caudoviricetes; Caudovirales; Siphoviridae; Tybeckvirinae; Lenusvirus; Lactobacillus virus Nyseid
GCA_002958905.2	139450	177	Lactobacillus phage Semele	NA	Viruses; Duplodnaviria; Heunggongvirae; Uroviricota; Caudoviricetes; Caudovirales; Herelleviridae; Twortvirinae; Harbinvirus; Lactobacillus virus Semele
GCA_002990225.2	38716	59	Lactobacillus phage Silenus	NA	Viruses; Duplodnaviria; Heunggongvirae; Uroviricota; Caudoviricetes; Caudovirales; Siphoviridae; Coetzeevirus; unclassified Coetzeevirus
GCA_004128075.1	20346	2	Osedax japonicus RNA virus 1	NA	Viruses; Riboviria; unclassified Riboviria
GCA_004131145.1	8051	1	Goose picornavirus 1	gooseNLSZK2HUN2013	Viruses; Riboviria; Orthornavirae; Pisuviricota; Pisoniviricetes; Picornavirales; Picornaviridae; unclassified Picornaviridae
GCA_013087765.1	14432	6	Black currant nucleorhabdovirus 1	NA	Viruses; Riboviria; Orthornavirae; Negarnaviricota; Haploviricotina; Monjiviricetes; Mononegavirales; Rhabdoviridae; Nucleorhabdovirus; unclassified Nucleorhabdovirus
GCA_002958915.1	38823	41	Escherichia phage YZ1	NA	Viruses; Duplodnaviria; Heunggongvirae; Uroviricota; Caudoviricetes; Caudovirales; Autographiviridae; Studiervirinae; Kayfunavirus; Escherichia virus YZ1
GCA_004146625.2	42031	67	Escherichia phage vB_EcoSRo145clw	NA	Viruses; Duplodnaviria; Heunggongvirae; Uroviricota; Caudoviricetes; Caudovirales; Siphoviridae; Guernseyvirinae; Kagunavirus; unclassified Kagunavirus
GCA_002958925.1	45058	64	Pseudomonas phage NV1	NA	Viruses; Duplodnaviria; Heunggongvirae; Uroviricota; Caudoviricetes; Caudovirales; Podoviridae; Vicosavirus; Pseudomonas virus NV1
GCA_002990265.1	43184	48	Pseudomonas phage phiNV3	NA	Viruses; Duplodnaviria; Heunggongvirae; Uroviricota; Caudoviricetes; Caudovirales; Autographiviridae; Krylovirinae; Kirikabuvirus; Pseudomonas virus NV3

gca	genome.size	proteome.size	phage.name	strain	Taxonomy
GCA_004134265.1	4743	2	Equine parvovirus H	D14	Viruses; Monodnaviria; Shotokuvirae; Cossaviricota; Quintoviricetes; Piccovirales; Parvoviridae; Parvovirinae; unclassified Parvovirinae
GCA_002958935.1	51793	91	Mycobacterium phage Acme	NA	Viruses; Duplodnaviria; Heunggongvirae; Uroviricota; Caudoviricetes; Caudovirales; Siphoviridae; Fromanvirus; unclassified Fromanvirus
GCA_002958945.1	58842	89	Gordonia phage Brandonk123	NA	Viruses; Duplodnaviria; Heunggongvirae; Uroviricota; Caudoviricetes; Caudovirales; Siphoviridae; Vividuovirus; Gordonia virus Brandonk123
GCA_002958955.1	57036	103	Mycobacterium phage Frankie	NA	Viruses; Duplodnaviria; Heunggongvirae; Uroviricota; Caudoviricetes; Caudovirales; Siphoviridae; Cheoctovirus; unclassified Cheoctovirus
GCA_002958965.1	51083	90	Mycobacterium phage Greg	NA	Viruses; Duplodnaviria; Heunggongvirae; Uroviricota; Caudoviricetes; Caudovirales; Siphoviridae; Fromanvirus; unclassified Fromanvirus
GCA_002958975.1	67044	91	Mycobacterium phage Holeinone	NA	Viruses; Duplodnaviria; Heunggongvirae; Uroviricota; Caudoviricetes; Caudovirales; Siphoviridae; Bclasvirinae; Rosebushvirus; unclassified Rosebushvirus
GCA_002958985.1	67570	89	Mycobacterium phage ItsyBitsy1	NA	Viruses; Duplodnaviria; Heunggongvirae; Uroviricota; Caudoviricetes; Caudovirales; Siphoviridae; Bclasvirinae; Rosebushvirus; unclassified Rosebushvirus
GCA_002958995.1	50042	88	Mycobacterium phage Lambert1	NA	Viruses; Duplodnaviria; Heunggongvirae; Uroviricota; Caudoviricetes; Caudovirales; Siphoviridae; Fromanvirus; unclassified Fromanvirus
GCA_002997425.1	15896	21	Gordonia phage SallySpecial	NA	Viruses; Duplodnaviria; Heunggongvirae; Uroviricota; Caudoviricetes; Caudovirales; Siphoviridae; unclassified Siphoviridae
GCA_002959005.1	52034	97	Mycobacterium phage Pippin	NA	Viruses; Duplodnaviria; Heunggongvirae; Uroviricota; Caudoviricetes; Caudovirales; Siphoviridae; Fromanvirus; unclassified Fromanvirus
GCA_002997415.1	156471	234	Mycobacterium phage Pier	NA	Viruses; Duplodnaviria; Heunggongvirae; Uroviricota; Caudoviricetes; Caudovirales; Myoviridae; Bixzunavirus; unclassified Bixzunavirus
GCA_002997405.1	155943	236	Mycobacterium phage NuevoMundo	NA	Viruses; Duplodnaviria; Heunggongvirae; Uroviricota; Caudoviricetes; Caudovirales; Myoviridae; Bixzunavirus; unclassified Bixzunavirus
GCA_002959015.1	41555	62	Microbacterium phage Aubergine	NA	Viruses; Duplodnaviria; Heunggongvirae; Uroviricota; Caudoviricetes; Caudovirales; Siphoviridae; Ilzatvirus; unclassified Ilzatvirus
GCA_002959025.1	41770	63	Microbacterium phage AxiPup	NA	Viruses; Duplodnaviria; Heunggongvirae; Uroviricota; Caudoviricetes; Caudovirales; Siphoviridae; Ilzatvirus; unclassified Ilzatvirus
GCA_002959035.1	41555	62	Microbacterium phage Baines	NA	Viruses; Duplodnaviria; Heunggongvirae; Uroviricota; Caudoviricetes; Caudovirales; Siphoviridae; Ilzatvirus; unclassified Ilzatvirus
GCA_002959045.1	41862	62	Microbacterium phage Balsa	NA	Viruses; Duplodnaviria; Heunggongvirae; Uroviricota; Caudoviricetes; Caudovirales; Siphoviridae; Ilzatvirus; unclassified Ilzatvirus
GCA_002959055.1	40366	63	Microbacterium phage Eleri	NA	Viruses; Duplodnaviria; Heunggongvirae; Uroviricota; Caudoviricetes; Caudovirales; Siphoviridae; Elerivirus; Microbacterium virus Eleri
GCA_002959065.1	41553	62	Microbacterium phage Espinosa	NA	Viruses; Duplodnaviria; Heunggongvirae; Uroviricota; Caudoviricetes; Caudovirales; Siphoviridae; Ilzatvirus; unclassified Ilzatvirus
GCA_002959075.1	41934	63	Microbacterium phage Hamlet	NA	Viruses; Duplodnaviria; Heunggongvirae; Uroviricota; Caudoviricetes; Caudovirales; Siphoviridae; Ilzatvirus; Microbacterium virus Hamlet
GCA_002959085.1	41525	62	Microbacterium phage Ilzat	NA	Viruses; Duplodnaviria; Heunggongvirae; Uroviricota; Caudoviricetes; Caudovirales; Siphoviridae; Ilzatvirus; Microbacterium virus Ilzat
GCA_002959095.1	41558	62	Microbacterium phage Kale	NA	Viruses; Duplodnaviria; Heunggongvirae; Uroviricota; Caudoviricetes; Caudovirales; Siphoviridae; Ilzatvirus; unclassified Ilzatvirus
GCA_002959105.1	41797	63	Microbacterium phage Knox	NA	Viruses; Duplodnaviria; Heunggongvirae; Uroviricota; Caudoviricetes; Caudovirales; Siphoviridae; Ilzatvirus; unclassified Ilzatvirus
GCA_002959115.1	41799	63	Microbacterium phage Ludgate	NA	Viruses; Duplodnaviria; Heunggongvirae; Uroviricota; Caudoviricetes; Caudovirales; Siphoviridae; Ilzatvirus; unclassified Ilzatvirus
GCA_002959125.1	41856	63	Microbacterium phage Peep	NA	Viruses; Duplodnaviria; Heunggongvirae; Uroviricota; Caudoviricetes; Caudovirales; Siphoviridae; Ilzatvirus; unclassified Ilzatvirus
GCA_002959135.1	41932	63	Microbacterium phage Peppino	NA	Viruses; Duplodnaviria; Heunggongvirae; Uroviricota; Caudoviricetes; Caudovirales; Siphoviridae; Ilzatvirus; unclassified Ilzatvirus
GCA_002959145.1	41894	63	Microbacterium phage Raccoon	NA	Viruses; Duplodnaviria; Heunggongvirae; Uroviricota; Caudoviricetes; Caudovirales; Siphoviridae; Ilzatvirus; unclassified Ilzatvirus
GCA_002959155.1	41860	63	Microbacterium phage Superfresh	NA	Viruses; Duplodnaviria; Heunggongvirae; Uroviricota; Caudoviricetes; Caudovirales; Siphoviridae; Ilzatvirus; unclassified Ilzatvirus
GCA_002959175.1	41553	62	Microbacterium phage Tenda	NA	Viruses; Duplodnaviria; Heunggongvirae; Uroviricota; Caudoviricetes; Caudovirales; Siphoviridae; Ilzatvirus; unclassified Ilzatvirus
GCA_002959185.1	68670	103	Mycobacterium phage RagingRooster	NA	Viruses; Duplodnaviria; Heunggongvirae; Uroviricota; Caudoviricetes; Caudovirales; Siphoviridae; Bclasvirinae; Pipefishvirus; unclassified Pipefishvirus
GCA_003029615.1	9702	1	Penguin megrivirus	NA	Viruses; Riboviria; Orthornavirae; Pisuviricota; Pisoniviricetes; Picornavirales; Picornaviridae; Megrivirus; Megrivirus E
GCA_004130255.1	5515	4	Redcrowned crane parvovirus	NA	Viruses; Monodnaviria; Shotokuvirae; Cossaviricota; Quintoviricetes; Piccovirales; Parvoviridae; Parvovirinae; Aveparvovirus; Gruiform aveparvovirus 1
GCA_018583055.1	14814	11	New Kent County virus	NA	Viruses; Riboviria; Orthornavirae; Negarnaviricota; Haploviricotina; Monjiviricetes; Mononegavirales; Rhabdoviridae; Alpharhabdovirinae; Ephemerovirus; Kent ephemerovirus
GCA_018582975.1	11518	4	Blacklegged tick chuvirus2	NA	Viruses; Riboviria; Orthornavirae; Negarnaviricota; Haploviricotina; Monjiviricetes; Jingchuvirales; Chuviridae; Nigecruvirus; Nigecruvirus ixodes
GCA_018582985.1	13841	5	Blacklegged tick rhabdovirus1	NA	Viruses; Riboviria; Orthornavirae; Negarnaviricota; Haploviricotina; Monjiviricetes; Mononegavirales; Rhabdoviridae; unclassified Rhabdoviridae

gca	genome.size	proteome.size	phage.name	strain	Taxonomy
GCA_000838325.1	2766	6	Eupatorium yellow vein virus	NA	Viruses; Monodnaviria; Shotokuvirae; Cressdnaviricota; Repensiviricetes; Geplafuvirales; Geminiviridae; Begomovirus
GCA_000883155.1	155122	163	Agrotis ipsilon multiple nucleopolyhedrovirus	Illinois	Viruses; dsDNA viruses, no RNA stage; Baculoviridae; Alphabaculovirus
GCA_002986845.1	2659	5	Sida golden mosaic Brazil virus	NA	Viruses; Monodnaviria; Shotokuvirae; Cressdnaviricota; Repensiviricetes; Geplafuvirales; Geminiviridae; Begomovirus
GCA_013087785.1	12434	5	Lepeophtheirus virus LS24	NA	Viruses; Riboviria; Orthornavirae; Negarnaviricota; Haploviricotina; Monjiviricetes; Mononegavirales; unclassified Mononegavirales
GCA_000883275.1	4731	5	Grapevine Algerian latent virus	nipplefruit	Viruses; ssRNA viruses; ssRNA positive-strand viruses, no DNA stage; Tombusviridae; Tombusvirus
GCA_000903935.1	4772	6	Moroccan pepper virus	NA	Viruses; ssRNA viruses; ssRNA positive-strand viruses, no DNA stage; Tombusviridae; Tombusvirus
GCA_002997375.2	41832	53	Salmonella phage vB_SpuP_Spp16	NA	Viruses; Duplodnaviria; Heunggongvirae; Uroviricota; Caudoviricetes; Caudovirales; Autographiviridae; Melnykvirinae; Panjvirus; Salmonella virus Spp16
GCA_004139175.1	170121	267	Escherichia phage vB_EcoMG28	NA	Viruses; Duplodnaviria; Heunggongvirae; Uroviricota; Caudoviricetes; Caudovirales; Myoviridae; Tevenvirinae; Tequatrovirus; unclassified Tequatrovirus
GCA_002990275.1	74594	142	Mycobacterium phage Asriel	NA	Viruses; Duplodnaviria; Heunggongvirae; Uroviricota; Caudoviricetes; Caudovirales; Siphoviridae; Kostyavirus; unclassified Kostyavirus
GCA_002990285.1	74594	144	Mycobacterium phage Barbarian	NA	Viruses; Duplodnaviria; Heunggongvirae; Uroviricota; Caudoviricetes; Caudovirales; Siphoviridae; Kostyavirus; unclassified Kostyavirus
GCA_002990295.1	51702	95	Mycobacterium phage BeesKnees	NA	Viruses; Duplodnaviria; Heunggongvirae; Uroviricota; Caudoviricetes; Caudovirales; Siphoviridae; Fromanvirus; unclassified Fromanvirus
GCA_002990335.1	50646	79	Gordonia phage Confidence	NA	Viruses; Duplodnaviria; Heunggongvirae; Uroviricota; Caudoviricetes; Caudovirales; Siphoviridae; unclassified Siphoviridae
GCA_002990355.1	52953	96	Mycobacterium phage Conquerage	NA	Viruses; Duplodnaviria; Heunggongvirae; Uroviricota; Caudoviricetes; Caudovirales; Siphoviridae; Fromanvirus; unclassified Fromanvirus
GCA_002990365.1	51083	90	Mycobacterium phage Fajezeel	NA	Viruses; Duplodnaviria; Heunggongvirae; Uroviricota; Caudoviricetes; Caudovirales; Siphoviridae; Fromanvirus; unclassified Fromanvirus
GCA_002990395.1	71650	135	Mycobacterium phage Gage	NA	Viruses; Duplodnaviria; Heunggongvirae; Uroviricota; Caudoviricetes; Caudovirales; Siphoviridae; Kostyavirus; unclassified Kostyavirus
GCA_002990415.1	70883	124	Mycobacterium phage JangDynasty	NA	Viruses; Duplodnaviria; Heunggongvirae; Uroviricota; Caudoviricetes; Caudovirales; Siphoviridae; Corndogvirus; unclassified Corndogvirus
GCA_002990465.1	53219	99	Mycobacterium phage Phonnegut	NA	Viruses; Duplodnaviria; Heunggongvirae; Uroviricota; Caudoviricetes; Caudovirales; Siphoviridae; Fromanvirus; unclassified Fromanvirus
GCA_002997885.1	58230	105	Mycobacterium phage Priscilla	NA	Viruses; Duplodnaviria; Heunggongvirae; Uroviricota; Caudoviricetes; Caudovirales; Siphoviridae; Cheoctovirus; unclassified Cheoctovirus
GCA_002997895.1	53355	97	Mycobacterium phage Scherzo	NA	Viruses; Duplodnaviria; Heunggongvirae; Uroviricota; Caudoviricetes; Caudovirales; Siphoviridae; Fromanvirus; unclassified Fromanvirus
GCA_002997905.1	76299	149	Mycobacterium phage Sotrice96	NA	Viruses; Duplodnaviria; Heunggongvirae; Uroviricota; Caudoviricetes; Caudovirales; Siphoviridae; Kostyavirus; unclassified Kostyavirus
GCA_002990435.1	156804	233	Mycobacterium phage LifeSavor	NA	Viruses; Duplodnaviria; Heunggongvirae; Uroviricota; Caudoviricetes; Caudovirales; Myoviridae; Bixzunavirus; unclassified Bixzunavirus
GCA_002997395.1	89910	147	Salmonella phage SE131	NA	Viruses; Duplodnaviria; Heunggongvirae; Uroviricota; Caudoviricetes; Caudovirales; Podoviridae; unclassified Podoviridae
GCA_000842785.1	2745	6	Tomato leaf curl Vietnam virus	NA	Viruses; ssDNA viruses; Geminiviridae; Begomovirus
GCA_002959195.1	48113	68	Salmonella phage PMBT28	NA	Viruses; Duplodnaviria; Heunggongvirae; Uroviricota; Caudoviricetes; Caudovirales; Siphoviridae; unclassified Siphoviridae
GCA_003368605.1	75893	100	Vibrio phage VP06	NA	Viruses; Duplodnaviria; Heunggongvirae; Uroviricota; Caudoviricetes; Caudovirales; Siphoviridae; Mardecavirus; unclassified Mardecavirus
GCA_004132645.1	6310	2	Sogatella furcifera totivirus 1	NA	Viruses; Riboviria; Orthornavirae; Duplornaviricota; Chrymotiviricetes; Ghabrivirales; Totiviridae; unclassified Totiviridae
GCA_004132665.1	6303	2	Sogatella furcifera totivirus 2	NA	Viruses; Riboviria; Orthornavirae; Duplornaviricota; Chrymotiviricetes; Ghabrivirales; Totiviridae; unclassified Totiviridae
GCA_004132545.1	4947	2	Gigaspora margarita giardialike virus 1	NA	Viruses; Riboviria; Orthornavirae; Duplornaviricota; Chrymotiviricetes; Ghabrivirales; Totiviridae; Giardiavirus; unclassified Giardiavirus
GCA_004132465.1	3243	1	Gigaspora margarita mitovirus 1	NA	Viruses; Riboviria; Orthornavirae; Lenarviricota; Howeltoviricetes; Cryppavirales; Mitoviridae; Mitovirus; unclassified Mitovirus
GCA_004132485.1	3394	1	Gigaspora margarita mitovirus 2	NA	Viruses; Riboviria; Orthornavirae; Lenarviricota; Howeltoviricetes; Cryppavirales; Mitoviridae; Mitovirus; unclassified Mitovirus
GCA_004132505.1	3387	1	Gigaspora margarita mitovirus 3	NA	Viruses; Riboviria; Orthornavirae; Lenarviricota; Howeltoviricetes; Cryppavirales; Mitoviridae; Mitovirus; unclassified Mitovirus
GCA_004132525.1	3389	1	Gigaspora margarita mitovirus 4	NA	Viruses; Riboviria; Orthornavirae; Lenarviricota; Howeltoviricetes; Cryppavirales; Mitoviridae; Mitovirus; unclassified Mitovirus
GCA_006451235.1	187976	218	Saltwater crocodilepox virus	NA	Viruses; Varidnaviria; Bamfordvirae; Nucleocytoviricota; Pokkesviricetes; Chitovirales; Poxviridae; Chordopoxvirinae; Crocodylidpoxvirus; unclassified Crocodylidpoxvirus
GCA_004788315.1	113971	124	Oxyplax ochracea nucleopolyhedrovirus	NA	Viruses; Baculoviridae; Alphabaculovirus

gca	genome.size	proteome.size	phage.name	strain	Taxonomy
GCA_002826745.1	7510	3	Trichoplusia ni TED virus	mutant FPD	Viruses; Riboviria; Pararnavirae; Artverviricota; Revtraviricetes; Ortervirales; Metaviridae; Errantivirus
GCA_000838565.1	236120	267	Melanoplus sanguinipes entomopoxvirus O	NA	Viruses; Varidnaviria; Bamfordvirae; Nucleocytoviricota; Pokkesviricetes; Chitovirales; Poxviridae; Entomopoxvirinae; Deltaentomopoxvirus; Melanoplus sanguinipes entomopoxvirus
GCA_002829865.1	11395	2	Everglades virus	Everglades Fe37c	Viruses; Riboviria; Orthornavirae; Kitrinoviricota; Alsuviricetes; Martellivirales; Togaviridae; Alphavirus
GCA_002888775.1	11465	2	Mosso das Pedras virus	78V3531	Viruses; Riboviria; Orthornavirae; Kitrinoviricota; Alsuviricetes; Martellivirales; Togaviridae; Alphavirus
GCA_002830345.1	1371	1	Tomato yellow leaf curl Rajasthan betasatellite	NA	Viruses; Tolecusatellitidae; Betasatellite
GCA_004117215.1	3182	2	Pythium nunn virus 1	UZ415	Viruses; Riboviria; Orthornavirae; Pisuviricota; Duplopiviricetes; Durnavirales; Partitiviridae; Gammapartitivirus; unclassified Gammapartitivirus
GCA_003004875.1	175437	295	Escherichia phage EcS1	NA	Viruses; Duplodnaviria; Heunggongvirae; Uroviricota; Caudoviricetes; Caudovirales; Myoviridae; Winklervirus; unclassified Winklervirus
GCA_000841425.1	5256	6	Goose hemorrhagic polyomavirus	NA	Viruses; dsDNA viruses, no RNA stage; Polyomaviridae; Avipolyomavirus group
GCA_002990475.1	37247	39	Vibrio phage Rostov1	NA	Viruses; Duplodnaviria; Heunggongvirae; Uroviricota; Caudoviricetes; Caudovirales; Autographiviridae; Studiervirinae; Chatterjeevirus; unclassified Chatterjeevirus
GCA_013086965.1	10072	3	Entoleuca phenuilike virus 1	NA	Viruses; Riboviria; Orthornavirae; Negarnaviricota; Polyploviricotina; Ellioviricetes; Bunyavirales; Phenuiviridae; Entovirus; Entoleuca entovirus
GCA_018582995.1	3412	1	Entoleuca ourmialike virus 1	NA	Viruses; Riboviria; Orthornavirae; Lenarviricota; Miaviricetes; Ourlivirales; Botourmiaviridae; Botoulivirus; Entoleuca botoulivirus
GCA_003266525.1	15074	11	Human respiratory syncytial virus B	16B7	Viruses; Riboviria; Orthornavirae; Negarnaviricota; Haploviricotina; Monjiviricetes; Mononegavirales; Pneumoviridae; Orthopneumovirus; Human orthopneumovirus
GCA_000872485.1	10438	4	Cupixi mammarenavirus	BeAn 119303	Viruses; ssRNA viruses; ssRNA negative-strand viruses; Arenaviridae; Mammarenavirus
GCA_000857005.1	129593	146	Choristoneura fumiferana multiple nucleopolyhedrovirus	NA	Viruses; dsDNA viruses, no RNA stage; Baculoviridae; Alphabaculovirus
GCA_000845425.1	48247	66	Burkholderia virus Bcep781	NA	Viruses; dsDNA viruses, no RNA stage; Caudovirales; Myoviridae; Bcep78virus
GCA_013087795.1	17357	12	Pistachio ampelovirus A	NA	Viruses; Riboviria; Orthornavirae; Kitrinoviricota; Alsuviricetes; Martellivirales; Closteroviridae; Ampelovirus
GCA_002937335.1	2740	5	Juncus maritimus associated virus	NA	Viruses; Monodnaviria; Shotokuvirae; Cressdnaviricota; Repensiviricetes; Geplafuvirales; Geminiviridae; unclassified Geminiviridae
GCA_002937355.1	2798	5	Polygala garcinii associated virus	NA	Viruses; Monodnaviria; Shotokuvirae; Cressdnaviricota; Repensiviricetes; Geplafuvirales; Geminiviridae; unclassified Geminiviridae
GCA_002937325.1	2974	5	Exomis microphylla associated virus	NA	Viruses; Monodnaviria; Shotokuvirae; Cressdnaviricota; Repensiviricetes; Geplafuvirales; Geminiviridae; Becurtovirus; Exomis microphylla latent virus
GCA_002937345.1	2963	5	Limeum africanum associated virus	NA	Viruses; Monodnaviria; Shotokuvirae; Cressdnaviricota; Repensiviricetes; Geplafuvirales; Geminiviridae; unclassified Geminiviridae
GCA_004132725.1	7390	5	Grapevine virus J	NA	Viruses; Riboviria; Orthornavirae; Kitrinoviricota; Alsuviricetes; Tymovirales; Betaflexiviridae; Trivirinae; Vitivirus
GCA_004133345.1	7372	7	Human papillomavirus type 203	NA	Viruses; Monodnaviria; Shotokuvirae; Cossaviricota; Papovaviricetes; Zurhausenvirales; Papillomaviridae; unclassified Papillomaviridae; Human papillomavirus types
GCA_003014245.1	68056	99	Mycobacterium phage AltPhacts	NA	Viruses; Duplodnaviria; Heunggongvirae; Uroviricota; Caudoviricetes; Caudovirales; Siphoviridae; Bclasvirinae; Pegunavirus; unclassified Pegunavirus
GCA_003014255.1	68509	100	Mycobacterium phage BatteryCK	NA	Viruses; Duplodnaviria; Heunggongvirae; Uroviricota; Caudoviricetes; Caudovirales; Siphoviridae; Bclasvirinae; Pegunavirus; unclassified Pegunavirus
GCA_003014265.1	60463	95	Mycobacterium phage Deby	NA	Viruses; Duplodnaviria; Heunggongvirae; Uroviricota; Caudoviricetes; Caudovirales; Siphoviridae; Timquatrovirus; unclassified Timquatrovirus
GCA_003014275.1	68331	99	Mycobacterium phage DocsntMatter	NA	Viruses; Duplodnaviria; Heunggongvirae; Uroviricota; Caudoviricetes; Caudovirales; Siphoviridae; Bclasvirinae; Pegunavirus; unclassified Pegunavirus
GCA_003014295.1	41562	62	Microbacterium phage Gelo	NA	Viruses; Duplodnaviria; Heunggongvirae; Uroviricota; Caudoviricetes; Caudovirales; Siphoviridae; Ilzatvirus; unclassified Ilzatvirus
GCA_003014305.1	59545	93	Gordonia phage Gravy	NA	Viruses; Duplodnaviria; Heunggongvirae; Uroviricota; Caudoviricetes; Caudovirales; Siphoviridae; unclassified Siphoviridae
GCA_003014315.1	59608	93	Gordonia phage Kerry	NA	Viruses; Duplodnaviria; Heunggongvirae; Uroviricota; Caudoviricetes; Caudovirales; Siphoviridae; unclassified Siphoviridae
GCA_003014325.1	50294	87	Mycobacterium phage Kykar	NA	Viruses; Duplodnaviria; Heunggongvirae; Uroviricota; Caudoviricetes; Caudovirales; Siphoviridae; Fromanvirus; unclassified Fromanvirus
GCA_003014335.1	60143	95	Mycobacterium phage LaterM	NA	Viruses; Duplodnaviria; Heunggongvirae; Uroviricota; Caudoviricetes; Caudovirales; Siphoviridae; Timquatrovirus; unclassified Timquatrovirus
GCA_003014345.1	51521	92	Mycobacterium phage McGuire	NA	Viruses; Duplodnaviria; Heunggongvirae; Uroviricota; Caudoviricetes; Caudovirales; Siphoviridae; Fromanvirus; unclassified Fromanvirus
GCA_003014355.1	51411	91	Mycobacterium phage Morrow	NA	Viruses; Duplodnaviria; Heunggongvirae; Uroviricota; Caudoviricetes; Caudovirales; Siphoviridae; Fromanvirus; unclassified Fromanvirus
GCA_003014365.1	61535	92	Mycobacterium phage Paola	NA	Viruses; Duplodnaviria; Heunggongvirae; Uroviricota; Caudoviricetes; Caudovirales; Siphoviridae; Timquatrovirus; unclassified Timquatrovirus

gca	genome.size	proteome.size	phage.name	strain	Taxonomy
GCA_003014375.1	68086	99	Mycobacterium phage ProfessorX	NA	Viruses; Duplodnaviria; Heunggongvirae; Uroviricota; Caudoviricetes; Caudovirales; Siphoviridae; Bclasvirinae; Pegunavirus; unclassified Pegunavirus
GCA_003014385.1	68408	94	Mycobacterium phage Thumb	NA	Viruses; Duplodnaviria; Heunggongvirae; Uroviricota; Caudoviricetes; Caudovirales; Siphoviridae; unclassified Siphoviridae
GCA_003014225.1	41834	63	Microbacterium phage AlexAdler	NA	Viruses; Duplodnaviria; Heunggongvirae; Uroviricota; Caudoviricetes; Caudovirales; Siphoviridae; Ilzatvirus; unclassified Ilzatvirus
GCA_003014235.1	57734	104	Mycobacterium phage Alexphander	NA	Viruses; Duplodnaviria; Heunggongvirae; Uroviricota; Caudoviricetes; Caudovirales; Siphoviridae; Cheoctovirus; unclassified Cheoctovirus
GCA_003014285.1	138896	228	Rhodococcus phage Finch	NA	Viruses; Duplodnaviria; Heunggongvirae; Uroviricota; Caudoviricetes; Caudovirales; Myoviridae; unclassified Myoviridae
GCA_003956545.1	8971	2	Yadonushi virus 1A	1W1032S5	Viruses; Riboviria; dsRNA viruses; unclassified dsRNA viruses
GCA_004132185.1	8806	3	Andrena haemorrhoa negelike virus	NA	Viruses; Riboviria; unclassified Riboviria; unclassified ssRNA viruses; unclassified ssRNA positive-strand viruses; Negevirus
GCA_004132205.1	6373	3	Bee MaculaLike virus 2	NA	Viruses; Riboviria; Orthornavirae; Kitrinoviricota; Alsuviricetes; Tymovirales; Tymoviridae; Maculavirus; unclassified Maculavirus
GCA_013087815.1	15408	6	Avian paramyxovirus 17	NA	Viruses; Riboviria; Orthornavirae; Negarnaviricota; Haploviricotina; Monjiviricetes; Mononegavirales; Paramyxoviridae; Avulavirinae; unclassified Avulavirinae
GCA_002990045.1	35132	50	Alteromonadaceae phage B23	NA	Viruses; Duplodnaviria; Heunggongvirae; Uroviricota; Caudoviricetes; Caudovirales; Myoviridae; unclassified Myoviridae
GCA_004131485.1	8071	8	Serinus canaria papillomavirus 1	York1	Viruses; Monodnaviria; Shotokuvirae; Cossaviricota; Papovaviricetes; Zurhausenvirales; Papillomaviridae; Firstpapillomavirinae; Etapapillomavirus; unclassified Etapapillomavirus
GCA_014883625.1	6340	1	Kobuvirus sp	16715x14	Viruses; Riboviria; Orthornavirae; Pisuviricota; Pisoniviricetes; Picornavirales; Picornaviridae; Kobuvirus; unclassified Kobuvirus
GCA_003389855.1	3398	2	Circoviridae TaCV1	NA	Viruses; Monodnaviria; Shotokuvirae; Cressdnaviricota; Arfiviricetes; Cirlivirales; Circoviridae; unclassified Circoviridae
GCA_003389875.1	2967	3	Circoviridae TaCV2	NA	Viruses; Monodnaviria; Shotokuvirae; Cressdnaviricota; Arfiviricetes; Cirlivirales; Circoviridae; unclassified Circoviridae
GCA_003570785.1	80764	120	Bacillus phage v_BBak1	NA	Viruses; Duplodnaviria; Heunggongvirae; Uroviricota; Caudoviricetes; Caudovirales; Siphoviridae; unclassified Siphoviridae
GCA_003570825.1	82931	115	Bacillus phage v_BBak10	NA	Viruses; Duplodnaviria; Heunggongvirae; Uroviricota; Caudoviricetes; Caudovirales; Siphoviridae; unclassified Siphoviridae
GCA_003570805.1	80764	120	Bacillus phage v_BBak6	NA	Viruses; Duplodnaviria; Heunggongvirae; Uroviricota; Caudoviricetes; Caudovirales; Siphoviridae; unclassified Siphoviridae
GCA_000853305.1	9812	2	Aphid lethal paralysis virus	NA	Viruses; ssRNA viruses; ssRNA positive-strand viruses, no DNA stage; Picornavirales; Dicistroviridae; Cripavirus
GCA_000859145.1	13782	7	Maize fine streak virus	NA	Viruses; ssRNA viruses; ssRNA negative-strand viruses; Mononegavirales; Rhabdoviridae; Nucleorhabdovirus
GCA_003014175.1	164037	175	Erwinia phage vB_EamMBue1	NA	Viruses; Duplodnaviria; Heunggongvirae; Uroviricota; Caudoviricetes; Caudovirales; Ackermannviridae; unclassified Ackermannviridae
GCA_004132805.1	9228	2	Blackbird arilivirus	NA	Viruses; Riboviria; Orthornavirae; Pisuviricota; Pisoniviricetes; Picornavirales; unclassified Picornavirales
GCA_002997865.1	39752	53	Escherichia phage Ebrios	NA	Viruses; Duplodnaviria; Heunggongvirae; Uroviricota; Caudoviricetes; Caudovirales; Autographiviridae; Studiervirinae; Teseptimavirus; Escherichia virus Ebrios
GCA_003023915.1	45135	49	Klebsiella phage myPSH1235	NA	Viruses; Duplodnaviria; Heunggongvirae; Uroviricota; Caudoviricetes; Caudovirales; Autographiviridae; Slopekvirinae; Drulisvirus; Klebsiella virus myPSH1235
GCA_002997805.1	39307	59	Microbacterium phage Casey	NA	Viruses; Duplodnaviria; Heunggongvirae; Uroviricota; Caudoviricetes; Caudovirales; Siphoviridae; Pikminvirus; unclassified Pikminvirus
GCA_002997695.1	39307	59	Microbacterium phage Pajaza	NA	Viruses; Duplodnaviria; Heunggongvirae; Uroviricota; Caudoviricetes; Caudovirales; Siphoviridae; Pikminvirus; unclassified Pikminvirus
GCA_002997715.1	39307	59	Microbacterium phage Pikmin	NA	Viruses; Duplodnaviria; Heunggongvirae; Uroviricota; Caudoviricetes; Caudovirales; Siphoviridae; Pikminvirus; Microbacterium virus Pikmin
GCA_002997725.1	41803	63	Microbacterium phage PuppyEggo	NA	Viruses; Duplodnaviria; Heunggongvirae; Uroviricota; Caudoviricetes; Caudovirales; Siphoviridae; Ilzatvirus; unclassified Ilzatvirus
GCA_002997775.1	41597	62	Microbacterium phage StingRay	NA	Viruses; Duplodnaviria; Heunggongvirae; Uroviricota; Caudoviricetes; Caudovirales; Siphoviridae; Ilzatvirus; unclassified Ilzatvirus
GCA_002997705.1	51373	88	Mycobacterium phage Phacado	NA	Viruses; Duplodnaviria; Heunggongvirae; Uroviricota; Caudoviricetes; Caudovirales; Siphoviridae; Fromanvirus; unclassified Fromanvirus
GCA_002997755.1	52601	94	Mycobacterium phage Ruotula	NA	Viruses; Duplodnaviria; Heunggongvirae; Uroviricota; Caudoviricetes; Caudovirales; Siphoviridae; Fromanvirus; unclassified Fromanvirus
GCA_002997765.1	51690	91	Mycobacterium phage Scowl	NA	Viruses; Duplodnaviria; Heunggongvirae; Uroviricota; Caudoviricetes; Caudovirales; Siphoviridae; Fromanvirus; unclassified Fromanvirus
GCA_003013885.1	68798	101	Mycobacterium phage Trypo	NA	Viruses; Duplodnaviria; Heunggongvirae; Uroviricota; Caudoviricetes; Caudovirales; Siphoviridae; Bclasvirinae; Pegunavirus; unclassified Pegunavirus
GCA_002997785.1	51366	88	Mycobacterium phage Wander	NA	Viruses; Duplodnaviria; Heunggongvirae; Uroviricota; Caudoviricetes; Caudovirales; Siphoviridae; Fromanvirus; unclassified Fromanvirus
GCA_002997795.1	68493	101	Mycobacterium phage Xavier	NA	Viruses; Duplodnaviria; Heunggongvirae; Uroviricota; Caudoviricetes; Caudovirales; Siphoviridae; Bclasvirinae; Pegunavirus; unclassified Pegunavirus

gca	genome.size	proteome.size	phage.name	strain	Taxonomy
GCA_004130575.1	10663	2	Nesidiocoris tenuis iflavirus 1	SD	Viruses; Riboviria; Orthornavirae; Pisuviricota; Pisoniviricetes; Picornavirales; Iflaviridae; unclassified Iflaviridae
GCA_018595315.1	13387	5	Ti ringspotassociated emaravirus	NA	Viruses; Riboviria; Orthornavirae; Negarnaviricota; Polyploviricotina; Ellioviricetes; Bunyavirales; Fimoviridae; Emaravirus
GCA_002997555.1	39640	58	Microbacterium phage Golden	NA	Viruses; Duplodnaviria; Heunggongvirae; Uroviricota; Caudoviricetes; Caudovirales; Siphoviridae; Kojivirus; Microbacterium virus Golden
GCA_002997575.1	39403	56	Microbacterium phage Koji	NA	Viruses; Duplodnaviria; Heunggongvirae; Uroviricota; Caudoviricetes; Caudovirales; Siphoviridae; Kojivirus; Microbacterium virus Koji
GCA_002997595.1	39640	58	Microbacterium phage Lucky3	NA	Viruses; Duplodnaviria; Heunggongvirae; Uroviricota; Caudoviricetes; Caudovirales; Siphoviridae; Kojivirus; unclassified Kojivirus
GCA_002997645.1	41544	62	Microbacterium phage Nattles	NA	Viruses; Duplodnaviria; Heunggongvirae; Uroviricota; Caudoviricetes; Caudovirales; Siphoviridae; Ilzatvirus; unclassified Ilzatvirus
GCA_002997525.1	50884	91	Mycobacterium phage EpicPhail	NA	Viruses; Duplodnaviria; Heunggongvirae; Uroviricota; Caudoviricetes; Caudovirales; Siphoviridae; Fromanvirus; unclassified Fromanvirus
GCA_002997495.1	68668	103	Mycobacterium phage ChaChing	NA	Viruses; Duplodnaviria; Heunggongvirae; Uroviricota; Caudoviricetes; Caudovirales; Siphoviridae; Bclasvirinae; Pipefishvirus; unclassified Pipefishvirus
GCA_002997505.1	68249	100	Mycobacterium phage Chunky	NA	Viruses; Duplodnaviria; Heunggongvirae; Uroviricota; Caudoviricetes; Caudovirales; Siphoviridae; Bclasvirinae; Pegunavirus; unclassified Pegunavirus
GCA_002997515.1	53325	94	Mycobacterium phage Corvo	NA	Viruses; Duplodnaviria; Heunggongvirae; Uroviricota; Caudoviricetes; Caudovirales; Siphoviridae; Fromanvirus; unclassified Fromanvirus
GCA_002997545.1	52695	96	Mycobacterium phage Forsytheast	NA	Viruses; Duplodnaviria; Heunggongvirae; Uroviricota; Caudoviricetes; Caudovirales; Siphoviridae; Fromanvirus; unclassified Fromanvirus
GCA_002997565.1	53317	95	Mycobacterium phage Ichabod	NA	Viruses; Duplodnaviria; Heunggongvirae; Uroviricota; Caudoviricetes; Caudovirales; Siphoviridae; Fromanvirus; unclassified Fromanvirus
GCA_002997585.1	68921	100	Mycobacterium phage LeeLot	NA	Viruses; Duplodnaviria; Heunggongvirae; Uroviricota; Caudoviricetes; Caudovirales; Siphoviridae; Bclasvirinae; Pegunavirus; unclassified Pegunavirus
GCA_002997635.1	51372	92	Mycobacterium phage MPlant7149	NA	Viruses; Duplodnaviria; Heunggongvirae; Uroviricota; Caudoviricetes; Caudovirales; Siphoviridae; Fromanvirus; unclassified Fromanvirus
GCA_002997605.1	69009	103	Mycobacterium phage Megatron	NA	Viruses; Duplodnaviria; Heunggongvirae; Uroviricota; Caudoviricetes; Caudovirales; Siphoviridae; Bclasvirinae; Pegunavirus; unclassified Pegunavirus
GCA_002997615.1	43511	65	Mycobacterium phage Mendokysei	NA	Viruses; Duplodnaviria; Heunggongvirae; Uroviricota; Caudoviricetes; Caudovirales; Siphoviridae; Bernalvirus; unclassified Bernalvirus
GCA_002997625.1	68488	99	Mycobacterium phage Mosaic	NA	Viruses; Duplodnaviria; Heunggongvirae; Uroviricota; Caudoviricetes; Caudovirales; Siphoviridae; Bclasvirinae; Pegunavirus; unclassified Pegunavirus
GCA_002997655.1	69439	103	Mycobacterium phage Nozo	NA	Viruses; Duplodnaviria; Heunggongvirae; Uroviricota; Caudoviricetes; Caudovirales; Siphoviridae; Bclasvirinae; Pipefishvirus; unclassified Pipefishvirus
GCA_002997535.1	56867	110	Mycobacterium phage Ogopogo	NA	Viruses; Duplodnaviria; Heunggongvirae; Uroviricota; Caudoviricetes; Caudovirales; Siphoviridae; Cheoctovirus; unclassified Cheoctovirus
GCA_002997665.1	50755	87	Mycobacterium phage OlanP	NA	Viruses; Duplodnaviria; Heunggongvirae; Uroviricota; Caudoviricetes; Caudovirales; Siphoviridae; Fromanvirus; unclassified Fromanvirus
GCA_002997675.1	68792	101	Mycobacterium phage OliverWalter	NA	Viruses; Duplodnaviria; Heunggongvirae; Uroviricota; Caudoviricetes; Caudovirales; Siphoviridae; Bclasvirinae; Pegunavirus; unclassified Pegunavirus
GCA_002997485.1	41534	62	Microbacterium phage Bonino	NA	Viruses; Duplodnaviria; Heunggongvirae; Uroviricota; Caudoviricetes; Caudovirales; Siphoviridae; Ilzatvirus; unclassified Ilzatvirus
GCA_002997465.1	68514	102	Mycobacterium phage Baloo	NA	Viruses; Duplodnaviria; Heunggongvirae; Uroviricota; Caudoviricetes; Caudovirales; Siphoviridae; Bclasvirinae; Pipefishvirus; unclassified Pipefishvirus
GCA_002997475.1	52233	94	Mycobacterium phage Bob3	NA	Viruses; Duplodnaviria; Heunggongvirae; Uroviricota; Caudoviricetes; Caudovirales; Siphoviridae; Fromanvirus; unclassified Fromanvirus
GCA_002997435.1	66095	93	Pseudomonas phage vB_PaeM_LS1	NA	Viruses; Duplodnaviria; Heunggongvirae; Uroviricota; Caudoviricetes; Caudovirales; Myoviridae; Pbunavirus; unclassified Pbunavirus
GCA_002997385.1	43133	59	Pseudomonas phage vB_PaeS_C1	NA	Viruses; Duplodnaviria; Heunggongvirae; Uroviricota; Caudoviricetes; Caudovirales; Siphoviridae; Septimatrevirus; unclassified Septimatrevirus
GCA_002997445.1	31399	50	Lactobacillus phage P1174	NA	Viruses; Duplodnaviria; Heunggongvirae; Uroviricota; Caudoviricetes; Caudovirales; Siphoviridae; Cequinquevirus; unclassified Cequinquevirus
GCA_002997835.1	61437	99	Pantoea phage vB_PagS_Vid5	NA	Viruses; Duplodnaviria; Heunggongvirae; Uroviricota; Caudoviricetes; Caudovirales; Siphoviridae; Vidquintavirus; Pantoea virus Vid5
GCA_002997685.1	35031	54	Actinomyces phage xhp1	NA	Viruses; Duplodnaviria; Heunggongvirae; Uroviricota; Caudoviricetes; Caudovirales; Siphoviridae; unclassified Siphoviridae
GCA_002997845.1	138427	10	Escherichia phage PDX	NA	Viruses; Duplodnaviria; Heunggongvirae; Uroviricota; Caudoviricetes; Caudovirales; Myoviridae; Vequintavirinae; Vequintavirus; unclassified Vequintavirus
GCA_002997455.1	73679	97	Klebsiella phage KP8	NA	Viruses; Duplodnaviria; Heunggongvirae; Uroviricota; Caudoviricetes; Caudovirales; Podoviridae; Gamaleyavirus; unclassified Gamaleyavirus
GCA_002997855.1	10584	20	Pseudoalteromonas phage Cr39582	NA	Viruses; Varidnaviria; Bamfordvirae; Preplasmiviricota; Tectiliviricetes; Vinavirales; Corticoviridae; Corticovirus; Pseudoalteromonas virus Cr39582
GCA_004193995.1	46195	73	Xanthomonas phage XPP1	NA	Viruses; Duplodnaviria; Heunggongvirae; Uroviricota; Caudoviricetes; Caudovirales; Myoviridae; Vidavervirus; unclassified Vidavervirus
GCA_004194015.1	46480	72	Xanthomonas phage XPP2	NA	Viruses; Duplodnaviria; Heunggongvirae; Uroviricota; Caudoviricetes; Caudovirales; Myoviridae; Vidavervirus; unclassified Vidavervirus

gca	genome.size	proteome.size	phage.name	strain	Taxonomy
GCA_004194035.1	49612	78	Xanthomonas phage XPP3	NA	Viruses; Duplodnaviria; Heunggongvirae; Uroviricota; Caudoviricetes; Caudovirales; Myoviridae; Vidavervirus; unclassified Vidavervirus
GCA_004194055.1	47397	73	Xanthomonas phage XPP4	NA	Viruses; Duplodnaviria; Heunggongvirae; Uroviricota; Caudoviricetes; Caudovirales; Myoviridae; Vidavervirus; unclassified Vidavervirus
GCA_004194075.1	46281	72	Xanthomonas phage XPP6	NA	Viruses; Duplodnaviria; Heunggongvirae; Uroviricota; Caudoviricetes; Caudovirales; Myoviridae; Vidavervirus; unclassified Vidavervirus
GCA_004194095.1	46278	74	Xanthomonas phage XPP8	NA	Viruses; Duplodnaviria; Heunggongvirae; Uroviricota; Caudoviricetes; Caudovirales; Myoviridae; Vidavervirus; unclassified Vidavervirus
GCA_004194115.1	48669	80	Xanthomonas phage XPP9	NA	Viruses; Duplodnaviria; Heunggongvirae; Uroviricota; Caudoviricetes; Caudovirales; Myoviridae; Vidavervirus; unclassified Vidavervirus
GCA_004194135.1	46503	77	Xanthomonas phage XPV1	NA	Viruses; Duplodnaviria; Heunggongvirae; Uroviricota; Caudoviricetes; Caudovirales; Myoviridae; Vidavervirus; unclassified Vidavervirus
GCA_004194155.1	45969	74	Xanthomonas phage XPV2	NA	Viruses; Duplodnaviria; Heunggongvirae; Uroviricota; Caudoviricetes; Caudovirales; Myoviridae; Vidavervirus; unclassified Vidavervirus
GCA_004194175.1	47046	76	Xanthomonas phage XPV3	NA	Viruses; Duplodnaviria; Heunggongvirae; Uroviricota; Caudoviricetes; Caudovirales; Myoviridae; Vidavervirus; unclassified Vidavervirus
GCA_003428315.1	144311	190	Synechococcus virus SPRM1	NA	Viruses; Duplodnaviria; Heunggongvirae; Uroviricota; Caudoviricetes; Caudovirales; Myoviridae; Kanaloavirus; unclassified Kanaloavirus
GCA_000843185.1	5499	5	Myzus persicae densovirus	NA	Viruses; ssDNA viruses; Parvoviridae; Densovirinae; unclassified Densovirinae
GCA_003972125.1	41178	1	Planarian secretory cell nidovirus	NA	Viruses; Riboviria; Orthornavirae; Pisuviricota; Pisoniviricetes; Nidovirales; Monidovirineae; Mononiviridae; Mononivirinae; Alphamononivirus; Dumedivirus; Planidovirus 1
GCA_902993965.1	446859	646	uncultured Caudovirales phage	NA	Viruses; Duplodnaviria; Heunggongvirae; Uroviricota; Caudoviricetes; Caudovirales; environmental samples
GCA_004788715.1	11774	6	Vaprio virus	NA	Viruses; Riboviria; Orthornavirae; Negarnaviricota; Haploviricotina; Monjiviricetes; Mononegavirales; Rhabdoviridae; Ledantevirus; Vaprio ledantevirus
GCA_004133985.1	3187	4	Domestic cat hepadnavirus	NA	Viruses; Riboviria; Pararnavirae; Artverviricota; Revtraviricetes; Blubervirales; Hepadnaviridae; Orthohepadnavirus; Domestic cat hepatitis B virus
GCA_003233935.1	1838258	926	Pandoravirus macleodensis	NA	Viruses; unclassified viruses; unclassified DNA viruses; unclassified dsDNA viruses; Pandoravirus
GCA_003233915.1	2003191	1081	Pandoravirus neocaledonia	NA	Viruses; unclassified viruses; unclassified DNA viruses; unclassified dsDNA viruses; Pandoravirus
GCA_003233895.1	2077288	1185	Pandoravirus quercus	NA	Viruses; unclassified viruses; unclassified DNA viruses; unclassified dsDNA viruses; Pandoravirus
GCA_003182725.1	37757	49	Streptococcus phage D1024	NA	Viruses; Duplodnaviria; Heunggongvirae; Uroviricota; Caudoviricetes; Caudovirales; Siphoviridae; Moineauvirus; unclassified Moineauvirus
GCA_003182745.1	34083	40	Streptococcus phage D1811	NA	Viruses; Duplodnaviria; Heunggongvirae; Uroviricota; Caudoviricetes; Caudovirales; Siphoviridae; Moineauvirus; unclassified Moineauvirus
GCA_003182705.1	36501	43	Streptococcus phage D5842	NA	Viruses; Duplodnaviria; Heunggongvirae; Uroviricota; Caudoviricetes; Caudovirales; Siphoviridae; Moineauvirus; unclassified Moineauvirus
GCA_003024145.1	68712	89	Escherichia phage myPSH2311	NA	Viruses; Duplodnaviria; Heunggongvirae; Uroviricota; Caudoviricetes; Caudovirales; Podoviridae; Kuravirus; unclassified Kuravirus
GCA_003014185.1	52369	76	Bacillus phage Anath	NA	Viruses; Duplodnaviria; Heunggongvirae; Uroviricota; Caudoviricetes; Caudovirales; Siphoviridae; unclassified Siphoviridae
GCA_003085755.1	61564	79	Escherichia phage SH2026Stx1	NA	Viruses; Duplodnaviria; Heunggongvirae; Uroviricota; Caudoviricetes; Caudovirales; Podoviridae; Sepvirinae; Traversvirus; unclassified Traversvirus
GCA_003014195.2	76335	90	Xanthomonas phage RiverRider	NA	Viruses; Duplodnaviria; Heunggongvirae; Uroviricota; Caudoviricetes; Caudovirales; Podoviridae; unclassified Podoviridae
GCA_003031055.1	76163	97	Escherichia phage myPSH1131	NA	Viruses; Duplodnaviria; Heunggongvirae; Uroviricota; Caudoviricetes; Caudovirales; Podoviridae; Kuravirus; unclassified Kuravirus
GCA_003014205.1	52157	86	Mycobacterium phage Fascinus	NA	Viruses; Duplodnaviria; Heunggongvirae; Uroviricota; Caudoviricetes; Caudovirales; Siphoviridae; Fromanvirus; unclassified Fromanvirus
GCA_003014215.1	75565	144	Mycobacterium phage RiverMonster	NA	Viruses; Duplodnaviria; Heunggongvirae; Uroviricota; Caudoviricetes; Caudovirales; Siphoviridae; Kostyavirus; unclassified Kostyavirus
GCA_003013975.1	52856	98	Gordonia phage Waits	NA	Viruses; Duplodnaviria; Heunggongvirae; Uroviricota; Caudoviricetes; Caudovirales; Siphoviridae; Soupsvirus; Gordonia virus Waits
GCA_003013965.1	60031	95	Mycobacterium phage Adonis	NA	Viruses; Duplodnaviria; Heunggongvirae; Uroviricota; Caudoviricetes; Caudovirales; Siphoviridae; Timquatrovirus; unclassified Timquatrovirus
GCA_003013995.1	41456	62	Mycobacterium phage CLED96	NA	Viruses; Duplodnaviria; Heunggongvirae; Uroviricota; Caudoviricetes; Caudovirales; Siphoviridae; Liefievirus; unclassified Liefievirus
GCA_003013935.1	60908	85	Mycobacterium phage Jeon	NA	Viruses; Duplodnaviria; Heunggongvirae; Uroviricota; Caudoviricetes; Caudovirales; Siphoviridae; unclassified Siphoviridae
GCA_003013955.1	67406	92	Mycobacterium phage Kheth	NA	Viruses; Duplodnaviria; Heunggongvirae; Uroviricota; Caudoviricetes; Caudovirales; Siphoviridae; Bclasvirinae; Rosebushvirus; unclassified Rosebushvirus
GCA_003014025.1	49894	78	Mycobacterium phage KittenMittens	NA	Viruses; Duplodnaviria; Heunggongvirae; Uroviricota; Caudoviricetes; Caudovirales; Siphoviridae; Fromanvirus; unclassified Fromanvirus
GCA_003013895.1	60579	89	Mycobacterium phage Megabear	NA	Viruses; Duplodnaviria; Heunggongvirae; Uroviricota; Caudoviricetes; Caudovirales; Siphoviridae; unclassified Siphoviridae

gca	genome.size	proteome.size	phage.name	strain	Taxonomy
GCA_003013925.1	55178	97	Mycobacterium phage MooMoo	NA	Viruses; Duplodnaviria; Heunggongvirae; Uroviricota; Caudoviricetes; Caudovirales; Siphoviridae; Cheoctovirus; unclassified Cheoctovirus
GCA_003013945.1	42393	59	Mycobacterium phage Nairb	NA	Viruses; Duplodnaviria; Heunggongvirae; Uroviricota; Caudoviricetes; Caudovirales; Siphoviridae; Bernalvirus; unclassified Bernalvirus
GCA_003013905.1	71752	102	Mycobacterium phage Nilo	NA	Viruses; Duplodnaviria; Heunggongvirae; Uroviricota; Caudoviricetes; Caudovirales; Siphoviridae; Papyrusvirus; unclassified Papyrusvirus
GCA_003013985.1	41901	63	Mycobacterium phage Remy19	NA	Viruses; Duplodnaviria; Heunggongvirae; Uroviricota; Caudoviricetes; Caudovirales; Siphoviridae; Liefievirus; unclassified Liefievirus
GCA_003014015.1	53134	90	Mycobacterium phage Smeagol	NA	Viruses; Duplodnaviria; Heunggongvirae; Uroviricota; Caudoviricetes; Caudovirales; Siphoviridae; Fromanvirus; unclassified Fromanvirus
GCA_003013915.1	57256	105	Mycobacterium phage UncleRicky	NA	Viruses; Duplodnaviria; Heunggongvirae; Uroviricota; Caudoviricetes; Caudovirales; Siphoviridae; Cheoctovirus; unclassified Cheoctovirus
GCA_003014005.1	42446	58	Streptomyces phage Nesbitt	NA	Viruses; Duplodnaviria; Heunggongvirae; Uroviricota; Caudoviricetes; Caudovirales; Siphoviridae; Abbeymikolonvirus; unclassified Abbeymikolonvirus
GCA_003014035.1	52743	84	Streptomyces phage Paedore	NA	Viruses; Duplodnaviria; Heunggongvirae; Uroviricota; Caudoviricetes; Caudovirales; Siphoviridae; Arquatrovirinae; Arequatrovirus; unclassified Arequatrovirus
GCA_003034585.1	172614	240	Enterobacter phage myPSH1140	NA	Viruses; Duplodnaviria; Heunggongvirae; Uroviricota; Caudoviricetes; Caudovirales; Myoviridae; Tevenvirinae; Karamvirus; unclassified Karamvirus
GCA_004131105.1	8138	2	Nephila clavipes virus 3	NA	Viruses; Riboviria; Orthornavirae; Kitrinoviricota; Alsuviricetes; Martellivirales; Virgaviridae; unclassified Virgaviridae
GCA_004131125.1	11919	3	Nephila clavipes virus 4	NA	Viruses; Riboviria; Orthornavirae; Kitrinoviricota; Alsuviricetes; Martellivirales; Virgaviridae; unclassified Virgaviridae
GCA_004117255.1	6677	2	Nephila clavipes virus 6	NA	Viruses; Riboviria; Orthornavirae; Duplornaviricota; Resentoviricetes; Reovirales; Reoviridae; unclassified Reoviridae
GCA_004131065.1	10198	1	Nephila clavipes virus 1	NA	Viruses; Riboviria; Orthornavirae; Pisuviricota; Pisoniviricetes; Picornavirales; unclassified Picornavirales
GCA_004131085.1	11699	4	Nephila clavipes virus 2	NA	Viruses; Riboviria; Orthornavirae; Pisuviricota; Pisoniviricetes; Picornavirales; unclassified Picornavirales
GCA_000866765.1	4975	2	Coniothyrium minitans RNA virus	NA	Viruses; dsRNA viruses; Totiviridae; Victorivirus
GCA_004284875.1	17034	18	Staphylococcus phage CSA13	NA	Viruses; Duplodnaviria; Heunggongvirae; Uroviricota; Caudoviricetes; Caudovirales; Podoviridae; Rakietenvirinae; Rosenblumvirus; Staphylococcus virus CSA13
GCA_003848425.1	2156	2	Termite associated circular virus 1	NA	Viruses; unclassified viruses; unclassified DNA viruses; unclassified ssDNA viruses
GCA_003847405.1	2226	2	Termite associated circular virus 2	NA	Viruses; Monodnaviria; Shotokuvirae; Cressdnaviricota; Repensiviricetes; Geplafuvirales; Genomoviridae; unclassified Genomoviridae
GCA_003848405.1	2220	2	Termite associated circular virus 3	NA	Viruses; unclassified viruses; unclassified DNA viruses; unclassified ssDNA viruses
GCA_003848525.1	2152	2	Termite associated circular virus 4	NA	Viruses; unclassified viruses; unclassified DNA viruses; unclassified ssDNA viruses
GCA_004133245.1	1778	2	Chicken associated cyclovirus 2	RSBR20154R	Viruses; Monodnaviria; Shotokuvirae; Cressdnaviricota; Arfiviricetes; Cirlivirales; Circoviridae; Cyclovirus
GCA_013087825.1	4432	2	Chicken chapparvovirus 2	RSBR152S	Viruses; Monodnaviria; Shotokuvirae; Cossaviricota; Quintoviricetes; Piccovirales; Parvoviridae; unclassified Parvoviridae
GCA_003729875.1	3173	2	Chicken stool associated circular virus 1	RSBR20151R	Viruses; unclassified viruses
GCA_003729855.1	3161	2	Chicken stool associated circular virus 2	RSBR20151R	Viruses; unclassified viruses
GCA_004130835.1	5329	3	Sarawak virus	SWKM26	Viruses; Riboviria; Orthornavirae; Kitrinoviricota; Alsuviricetes; Hepelivirales; Alphatetraviridae; unclassified Alphatetraviridae
GCA_004156275.1	1242360	1027	Megavirus vitis	NA	Viruses; Varidnaviria; Bamfordvirae; Nucleocytoviricota; Megaviricetes; Imitervirales; Mimiviridae; Mimivirus; unclassified Mimivirus
GCA_004156295.1	1098002	900	Moumouvirus australiensis	NA	Viruses; Varidnaviria; Bamfordvirae; Nucleocytoviricota; Megaviricetes; Imitervirales; Mimiviridae; unclassified Mimiviridae; Moumouvirus
GCA_000865725.1	13588	10	Influenza A virus APuerto Rico81934H1N1	NA	Viruses; ssRNA viruses; ssRNA negative-strand viruses; Orthomyxoviridae; Influenzavirus A; Influenza A virus; H1N1 subtype
GCA_002629925.1	60030	93	Salmonella phage SE1 inNonagvirus	NA	Viruses; Duplodnaviria; Heunggongvirae; Uroviricota; Caudoviricetes; Caudovirales; Siphoviridae; Nonagvirus; Salmonella virus SE1Kor
GCA_003094055.1	42250	65	Burkholderia phage vB_BmuP_KL4	NA	Viruses; Duplodnaviria; Heunggongvirae; Uroviricota; Caudoviricetes; Caudovirales; Podoviridae; Kelquatrovirus; Burkholderia virus KL4
GCA_013087835.1	9218	1	Chinese broadheaded pond turtle picornavirus 1	WHWGC151314	Viruses; Riboviria; Orthornavirae; Pisuviricota; Pisoniviricetes; Picornavirales; unclassified Picornavirales
GCA_014883645.1	7999	1	Chinese broadheaded pond turtle picornavirus 2	WHWGGF75534	Viruses; Riboviria; Orthornavirae; Pisuviricota; Pisoniviricetes; Picornavirales; unclassified Picornavirales
GCA_013087845.1	8049	1	Guangdong chinese water skink picornavirus	ZGLXR119682	Viruses; Riboviria; Orthornavirae; Pisuviricota; Pisoniviricetes; Picornavirales; unclassified Picornavirales
GCA_013087855.1	7994	1	Guangdong chinese water snake picornavirus	LPSF20501	Viruses; Riboviria; Orthornavirae; Pisuviricota; Pisoniviricetes; Picornavirales; unclassified Picornavirales

gca	genome.size	proteome.size	phage.name	strain	Taxonomy
GCA_018583435.1	8318	1	Guangdong fish caecilians picornavirus	GDYYC83005	Viruses; Riboviria; Orthornavirae; Pisuviricota; Pisoniviricetes; Picornavirales; unclassified Picornavirales
GCA_018583425.1	7504	1	Guangdong spotted longbarbel catfish picornavirus	GDDSYC43605	Viruses; Riboviria; Orthornavirae; Pisuviricota; Pisoniviricetes; Picornavirales; unclassified Picornavirales
GCA_004789095.1	7400	1	Hainan gekko similignum picornavirus	LPXYC222841	Viruses; Riboviria; Orthornavirae; Pisuviricota; Pisoniviricetes; Picornavirales; Picornaviridae; Rafivirus; Rafivirus B
GCA_013087865.1	7969	1	Hainan oriental leaftoed gecko picornavirus	LPXYC213122	Viruses; Riboviria; Orthornavirae; Pisuviricota; Pisoniviricetes; Picornavirales; unclassified Picornavirales
GCA_013087875.1	8173	1	Wenling banjofish picornavirus 1	LXMC34076	Viruses; Riboviria; Orthornavirae; Pisuviricota; Pisoniviricetes; Picornavirales; unclassified Picornavirales
GCA_018583405.1	8343	1	tropivirus B1	LPWC175499	Viruses; Riboviria; Orthornavirae; Pisuviricota; Pisoniviricetes; Picornavirales; Picornaviridae; Tropivirus; Tropivirus B
GCA_013087885.1	7471	1	Yili teratoscincus roborowskii picornavirus 2	LPWC210215	Viruses; Riboviria; Orthornavirae; Pisuviricota; Pisoniviricetes; Picornavirales; unclassified Picornavirales
GCA_018583395.1	8332	1	Wenling chelidoperca picornavirus	LXMC188591	Viruses; Riboviria; Orthornavirae; Pisuviricota; Pisoniviricetes; Picornavirales; unclassified Picornavirales
GCA_013087895.1	7790	1	Wenling crossorhombus picornavirus	XDXMC21480	Viruses; Riboviria; Orthornavirae; Pisuviricota; Pisoniviricetes; Picornavirales; unclassified Picornavirales
GCA_013087905.1	7965	1	Wuhan sharpbelly picornavirus 2	DSYC36136	Viruses; Riboviria; Orthornavirae; Pisuviricota; Pisoniviricetes; Picornavirales; unclassified Picornavirales
GCA_013087915.1	7871	1	Wuhan sharpbelly picornavirus 3	DSYC47507	Viruses; Riboviria; Orthornavirae; Pisuviricota; Pisoniviricetes; Picornavirales; unclassified Picornavirales
GCA_018583415.1	7947	1	rajidapivirus A1	DHBYCGS18742	Viruses; Riboviria; Orthornavirae; Pisuviricota; Pisoniviricetes; Picornavirales; Picornaviridae; Rajidapivirus; Rajidapivirus A
GCA_013087925.1	8591	1	Wenling triplecross lizardfish picornavirus	XYHYC185246	Viruses; Riboviria; Orthornavirae; Pisuviricota; Pisoniviricetes; Picornavirales; unclassified Picornavirales
GCA_013087935.1	8084	1	Wenling jack mackerels picornavirus	LXMC375591	Viruses; Riboviria; Orthornavirae; Pisuviricota; Pisoniviricetes; Picornavirales; unclassified Picornavirales
GCA_012271695.1	14939	5	Chinese broadheaded pond turtle arterivirus	WHWGC150683	Viruses; Riboviria; Orthornavirae; Duplornaviricota; Resentoviricetes; Reovirales; Reoviridae; unclassified Reoviridae
GCA_013087945.1	11110	5	Hainan blackspectacled toad dimarhabdovirus	HKCCG762	Viruses; Riboviria; Orthornavirae; Negarnaviricota; Haploviricotina; Monjiviricetes; Mononegavirales; Rhabdoviridae; unclassified Rhabdoviridae
GCA_012271715.1	30859	6	Guangdong redbanded snake torovirus	LPSF30546	Viruses; Riboviria; Orthornavirae; Pisuviricota; Pisoniviricetes; Nidovirales; Tornidovirineae; Tobaniviridae; Torovirinae; Torovirus; unclassified Torovirus
GCA_012271705.1	29409	6	Hainan hebius popei torovirus	LPSC33749	Viruses; Riboviria; Orthornavirae; Pisuviricota; Pisoniviricetes; Nidovirales; Tornidovirineae; Tobaniviridae; Torovirinae; Torovirus; unclassified Torovirus
GCA_004788895.1	5301	1	Wenling minipizza batfish hantavirus	XQTMS16810	Viruses; Riboviria; Orthornavirae; Negarnaviricota; Polyploviricotina; Ellioviricetes; Bunyavirales; Hantaviridae; Actantavirinae; Actinovirus; Batfish actinovirus
GCA_004788935.1	6305	1	Wenling red spikefish hantavirus	XTXMS70955	Viruses; Riboviria; Orthornavirae; Negarnaviricota; Polyploviricotina; Ellioviricetes; Bunyavirales; Hantaviridae; Actantavirinae; Actinovirus; Spikefish actinovirus
GCA_004788915.1	6403	1	Wenling yellow goosefish hantavirus	XQTMS34106	Viruses; Riboviria; Orthornavirae; Negarnaviricota; Polyploviricotina; Ellioviricetes; Bunyavirales; Hantaviridae; Actantavirinae; Actinovirus; Goosefish actinovirus
GCA_004788875.1	6627	1	Hainan oriental leaftoed gecko hantavirus	LPXYF84819	Viruses; Riboviria; Orthornavirae; Negarnaviricota; Polyploviricotina; Ellioviricetes; Bunyavirales; Hantaviridae; Repantavirinae; Reptillovirus; Gecko reptillovirus
GCA_004788955.1	6761	1	Wenling hagfish virus	DHMMS25300	Viruses; Riboviria; Orthornavirae; Negarnaviricota; Polyploviricotina; Ellioviricetes; Bunyavirales; Hantaviridae; Agantavirinae; Agnathovirus; Hagfish agnathovirus
GCA_012271665.1	15301	6	Hainan oligodon formosanus arterivirus	LPSF32245	Viruses; Riboviria; Orthornavirae; Pisuviricota; Pisoniviricetes; Nidovirales; Arnidovirineae; Olifoviridae; Gofosavirinae; Kukrinivirus; Oligodon snake nidovirus 1
GCA_012271685.1	13162	5	Nanhai ghost shark arterivirus	NHYJS6157	Viruses; Riboviria; Orthornavirae; Pisuviricota; Pisoniviricetes; Nidovirales; Nanidovirineae; Nanghoshaviridae; Chimanivirinae; Chimshavirus; Nangarvirus 1
GCA_012271675.1	18410	5	Guangdong greater green snake arterivirus	LPSG2430	Viruses; Riboviria; Orthornavirae; Pisuviricota; Pisoniviricetes; Nidovirales; Arnidovirineae; Gresnaviridae; Reternivirinae; Cyclophivirus; Ptyasnivirus 1
GCA_012271655.1	18239	5	Wuhan japanese halfbeak arterivirus	DSYS15584	Viruses; Riboviria; Orthornavirae; Pisuviricota; Pisoniviricetes; Nidovirales; Nanidovirineae; Nanhypoviridae; Hyporhamsavirinae; Sajorinivirus; Halfbeak nidovirus 1
GCA_004789035.1	16560	7	Wenling triplecross lizardfish paramyxovirus	XYHYC179963	Viruses; Riboviria; Orthornavirae; Negarnaviricota; Haploviricotina; Monjiviricetes; Mononegavirales; Paramyxoviridae; Metaparamyxovirinae; Synodonvirus; Synodus synodonvirus
GCA_004789015.1	21523	10	Wenzhou pacific spadenose shark paramyxovirus	DWXCSG11347	Viruses; Riboviria; Orthornavirae; Negarnaviricota; Haploviricotina; Monjiviricetes; Mononegavirales; Paramyxoviridae; Scoliodonvirus; Scoliodon scoliodonvirus
GCA_004789075.1	15326	9	Wenling hoplichthys paramyxovirus	XYXMC57250	Viruses; Riboviria; Orthornavirae; Negarnaviricota; Haploviricotina; Monjiviricetes; Mononegavirales; Paramyxoviridae; Hoplichthysvirus; Hoplichthys hoplichthysvirus
GCA_004789055.1	15043	9	Wenling tonguesole paramyxovirus	XYHYC190750	Viruses; Riboviria; Orthornavirae; Negarnaviricota; Haploviricotina; Monjiviricetes; Mononegavirales; Paramyxoviridae; Cynoglossusvirus; Cynoglossus cynoglossusvirus
GCA_004128195.1	11885	4	Wenling frogfish arenavirus 1	XYHYG11303	Viruses; Riboviria; Orthornavirae; Negarnaviricota; Polyploviricotina; Ellioviricetes; Bunyavirales; Arenaviridae; Antennavirus; Striated antennavirus
GCA_004128215.1	10992	4	Wenling frogfish arenavirus 2	XYHYG24857	Viruses; Riboviria; Orthornavirae; Negarnaviricota; Polyploviricotina; Ellioviricetes; Bunyavirales; Arenaviridae; unclassified Arenaviridae
GCA_004788975.1	17221	10	Wenling frogfish filovirus	XYHYS28627	Viruses; Riboviria; Orthornavirae; Negarnaviricota; Haploviricotina; Monjiviricetes; Mononegavirales; Filoviridae; Striavirus; Xilang striavirus

gca	genome.size	proteome.size	phage.name	strain	Taxonomy
GCA_004788995.1	14280	6	Wenling thamnaconus septentrionalis filovirus	LQMMTII17328	Viruses; Riboviria; Orthornavirae; Negarnaviricota; Haploviricotina; Monjiviricetes; Mononegavirales; Filoviridae; Thamnovirus; Huangjiao thamnovirus
GCA_004788855.1	8989	6	Wuhan sharpbelly bornavirus	DSYS4497	Viruses; Riboviria; Orthornavirae; Negarnaviricota; Haploviricotina; Monjiviricetes; Mononegavirales; Bornaviridae; Cultervirus; Sharpbelly cultervirus
GCA_018583375.1	10625	3	Guangdong redbanded snake chuviruslike virus	LPSC27055	Viruses; Riboviria; Orthornavirae; Negarnaviricota; Haploviricotina; Monjiviricetes; Jingchuvirales; Chuviridae; Piscichuvirus; Piscichuvirus lycodontis
GCA_018583385.1	10385	3	Wenling fish chulike virus	XQTMS36511	Viruses; Riboviria; Orthornavirae; Negarnaviricota; Haploviricotina; Monjiviricetes; Jingchuvirales; Chuviridae; Piscichuvirus; Piscichuvirus wenlingense
GCA_018583125.1	13706	5	Chimay rhabdovirus	NA	Viruses; Riboviria; Orthornavirae; Negarnaviricota; Haploviricotina; Monjiviricetes; Mononegavirales; Rhabdoviridae; Betaricinrhavirus; Chimay betaricinrhavirus
GCA_004788755.1	17940	8	Mount Mabu Lophuromys virus 1	NA	Viruses; Riboviria; Orthornavirae; Negarnaviricota; Haploviricotina; Monjiviricetes; Mononegavirales; Paramyxoviridae; Orthoparamyxovirinae; Jeilongvirus; Lophuromys jeilongvirus 1
GCA_004788775.1	17220	8	Mount Mabu Lophuromys virus 2	NA	Viruses; Riboviria; Orthornavirae; Negarnaviricota; Haploviricotina; Monjiviricetes; Mononegavirales; Paramyxoviridae; Orthoparamyxovirinae; Jeilongvirus; Lophuromys jeilongvirus 2
GCA_004117415.1	8589	5	Cape gooseberry ilarvirus 1	NA	Viruses; Riboviria; Orthornavirae; Kitrinoviricota; Alsuviricetes; Martellivirales; Bromoviridae; Ilarvirus; unclassified Ilarvirus
GCA_004788735.1	20148	9	Pohorje myodes paramyxovirus 1	NA	Viruses; Riboviria; Orthornavirae; Negarnaviricota; Haploviricotina; Monjiviricetes; Mononegavirales; Paramyxoviridae; Orthoparamyxovirinae; Jeilongvirus; Myodes jeilongvirus
GCA_003094035.1	39934	15	Vibrio phage Rostov 6	NA	Viruses; Duplodnaviria; Heunggongvirae; Uroviricota; Caudoviricetes; Caudovirales; Podoviridae; Enhodamvirus; unclassified Enhodamvirus
GCA_004132565.1	8890	6	Chrysanthemum virus R	BJ	Viruses; Riboviria; Orthornavirae; Kitrinoviricota; Alsuviricetes; Tymovirales; Betaflexiviridae; Quinvirinae; Carlavirus; unclassified Carlavirus
GCA_000846285.1	7461	7	Human papillomavirus type 92	NA	Viruses; dsDNA viruses, no RNA stage; Papillomaviridae; Betapapillomavirus; Betapapillomavirus 4
GCA_000905955.1	11918	5	Aravan lyssavirus	NA	Viruses; ssRNA viruses; ssRNA negative-strand viruses; Mononegavirales; Rhabdoviridae; Lyssavirus
GCA_000888735.1	1181549	979	Acanthamoeba polyphaga mimivirus	NA	Viruses; dsDNA viruses, no RNA stage; Mimiviridae; Mimivirus
GCA_004132585.1	3687	6	Camellia chlorotic dwarfassociated virus	NA	Viruses; Monodnaviria; Shotokuvirae; Cressdnaviricota; Repensiviricetes; Geplafuvirales; Geminiviridae; unclassified Geminiviridae
GCA_004133485.1	4978	4	Porcine polyomavirus	180230	Viruses; Monodnaviria; Shotokuvirae; Cossaviricota; Papovaviricetes; Sepolyvirales; Polyomaviridae; unclassified Polyomaviridae
GCA_004291235.1	16159	28	Halorubrum pleomorphic virus 9	B22SS54	Viruses; Monodnaviria; Trapavirae; Saleviricota; Huolimaviricetes; Haloruvirales; Pleolipoviridae; Betapleolipovirus; Betapleolipovirus HRPV9
GCA_003034785.1	67539	112	Microbacterium phage Triscuit	NA	Viruses; Duplodnaviria; Heunggongvirae; Uroviricota; Caudoviricetes; Caudovirales; Siphoviridae; unclassified Siphoviridae
GCA_003034805.1	50006	88	Mycobacterium phage Pistachio	NA	Viruses; Duplodnaviria; Heunggongvirae; Uroviricota; Caudoviricetes; Caudovirales; Siphoviridae; Fromanvirus; unclassified Fromanvirus
GCA_003034795.1	50084	89	Mycobacterium phage Puppy	NA	Viruses; Duplodnaviria; Heunggongvirae; Uroviricota; Caudoviricetes; Caudovirales; Siphoviridae; Fromanvirus; unclassified Fromanvirus
GCA_003034595.1	149229	209	Staphylococcus phage phiSA_BS2	NA	Viruses; Duplodnaviria; Heunggongvirae; Uroviricota; Caudoviricetes; Caudovirales; Herelleviridae; Twortvirinae; Baoshanvirus; Staphylococcus virus BS2
GCA_003023925.1	142978	200	Staphylococcus phage phiSA_BS1	NA	Viruses; Duplodnaviria; Heunggongvirae; Uroviricota; Caudoviricetes; Caudovirales; Herelleviridae; Twortvirinae; Baoshanvirus; Staphylococcus virus BS1
GCA_003023955.1	68260	103	Streptomyces phage Wentworth	NA	Viruses; Duplodnaviria; Heunggongvirae; Uroviricota; Caudoviricetes; Caudovirales; Siphoviridae; unclassified Siphoviridae
GCA_003023945.1	68671	105	Streptomyces phage Yara	NA	Viruses; Duplodnaviria; Heunggongvirae; Uroviricota; Caudoviricetes; Caudovirales; Siphoviridae; unclassified Siphoviridae
GCA_003024135.1	66609	96	Gordonia phage Djokovic	NA	Viruses; Duplodnaviria; Heunggongvirae; Uroviricota; Caudoviricetes; Caudovirales; Siphoviridae; Terapinvirus; unclassified Terapinvirus
GCA_003024125.1	73659	91	Gordonia phage Hail2Pitt	NA	Viruses; Duplodnaviria; Heunggongvirae; Uroviricota; Caudoviricetes; Caudovirales; Siphoviridae; Woesvirus; unclassified Woesvirus
GCA_003024115.1	64592	87	Mycobacterium phage BigMama	NA	Viruses; Duplodnaviria; Heunggongvirae; Uroviricota; Caudoviricetes; Caudovirales; Siphoviridae; Dclasvirinae; Plotvirus; Mycobacterium virus Plot
GCA_003024085.1	75605	146	Mycobacterium phage MPhalcon	NA	Viruses; Duplodnaviria; Heunggongvirae; Uroviricota; Caudoviricetes; Caudovirales; Siphoviridae; Kostyavirus; unclassified Kostyavirus
GCA_003024065.1	56439	78	Mycobacterium phage SgtBeansprout	NA	Viruses; Duplodnaviria; Heunggongvirae; Uroviricota; Caudoviricetes; Caudovirales; Siphoviridae; Timquatrovirus; unclassified Timquatrovirus
GCA_003024095.1	50080	83	Mycobacterium phage Panamaxus	NA	Viruses; Duplodnaviria; Heunggongvirae; Uroviricota; Caudoviricetes; Caudovirales; Siphoviridae; Fromanvirus; unclassified Fromanvirus
GCA_003024075.1	71651	128	Mycobacterium phage SchoolBus	NA	Viruses; Duplodnaviria; Heunggongvirae; Uroviricota; Caudoviricetes; Caudovirales; Siphoviridae; Corndogvirus; unclassified Corndogvirus
GCA_003024105.1	51365	87	Mycobacterium phage Skipitt	NA	Viruses; Duplodnaviria; Heunggongvirae; Uroviricota; Caudoviricetes; Caudovirales; Siphoviridae; Fromanvirus; unclassified Fromanvirus
GCA_003023935.1	39964	47	Acinetobacter phage vB_AbaP_D2	NA	Viruses; Duplodnaviria; Heunggongvirae; Uroviricota; Caudoviricetes; Caudovirales; Autographiviridae; Beijerinckvirinae; Friunavirus; Acinetobacter virus D2
GCA_003034615.1	68875	102	Mycobacterium phage Cobra	NA	Viruses; Duplodnaviria; Heunggongvirae; Uroviricota; Caudoviricetes; Caudovirales; Siphoviridae; Bclasvirinae; Pegunavirus; unclassified Pegunavirus

gca	genome.size	proteome.size	phage.name	strain	Taxonomy
GCA_003034625.1	68482	101	Mycobacterium phage Yahalom	NA	Viruses; Duplodnaviria; Heunggongvirae; Uroviricota; Caudoviricetes; Caudovirales; Siphoviridae; Bclasvirinae; Pipefishvirus; unclassified Pipefishvirus
GCA_003034825.1	155916	234	Mycobacterium phage MikeLiesIn	NA	Viruses; Duplodnaviria; Heunggongvirae; Uroviricota; Caudoviricetes; Caudovirales; Myoviridae; Bixzunavirus; unclassified Bixzunavirus
GCA_003024025.1	49965	78	Gordonia phage Fenry	NA	Viruses; Duplodnaviria; Heunggongvirae; Uroviricota; Caudoviricetes; Caudovirales; Siphoviridae; unclassified Siphoviridae
GCA_003024055.1	52394	87	Mycobacterium phage Lopton	NA	Viruses; Duplodnaviria; Heunggongvirae; Uroviricota; Caudoviricetes; Caudovirales; Siphoviridae; Fromanvirus; unclassified Fromanvirus
GCA_003024035.1	57455	93	Mycobacterium phage Misha28	NA	Viruses; Duplodnaviria; Heunggongvirae; Uroviricota; Caudoviricetes; Caudovirales; Siphoviridae; Cheoctovirus; unclassified Cheoctovirus
GCA_003024045.1	57455	93	Mycobacterium phage TootsiePop	NA	Viruses; Duplodnaviria; Heunggongvirae; Uroviricota; Caudoviricetes; Caudovirales; Siphoviridae; Cheoctovirus; unclassified Cheoctovirus
GCA_003023965.1	57737	105	Mycobacterium phage Batiatus	NA	Viruses; Duplodnaviria; Heunggongvirae; Uroviricota; Caudoviricetes; Caudovirales; Siphoviridae; Cheoctovirus; unclassified Cheoctovirus
GCA_003023975.1	51904	91	Mycobacterium phage GageAP	NA	Viruses; Duplodnaviria; Heunggongvirae; Uroviricota; Caudoviricetes; Caudovirales; Siphoviridae; Fromanvirus; unclassified Fromanvirus
GCA_003023985.1	52967	101	Mycobacterium phage Jsquared	NA	Viruses; Duplodnaviria; Heunggongvirae; Uroviricota; Caudoviricetes; Caudovirales; Siphoviridae; Fromanvirus; unclassified Fromanvirus
GCA_003023995.1	50841	87	Mycobacterium phage Lilith	NA	Viruses; Duplodnaviria; Heunggongvirae; Uroviricota; Caudoviricetes; Caudovirales; Siphoviridae; Fromanvirus; unclassified Fromanvirus
GCA_003024005.1	51594	87	Mycobacterium phage Naca	NA	Viruses; Duplodnaviria; Heunggongvirae; Uroviricota; Caudoviricetes; Caudovirales; Siphoviridae; Fromanvirus; unclassified Fromanvirus
GCA_003024015.1	52686	99	Mycobacterium phage SuperAwesome	NA	Viruses; Duplodnaviria; Heunggongvirae; Uroviricota; Caudoviricetes; Caudovirales; Siphoviridae; Fromanvirus; unclassified Fromanvirus
GCA_003014935.1	46833	86	Escherichia phage vB_EcoS Sa179lw	NA	Viruses; Duplodnaviria; Heunggongvirae; Uroviricota; Caudoviricetes; Caudovirales; Siphoviridae; unclassified Siphoviridae
GCA_003034745.1	41858	63	Microbacterium phage Antoinette	NA	Viruses; Duplodnaviria; Heunggongvirae; Uroviricota; Caudoviricetes; Caudovirales; Siphoviridae; Ilzatvirus; unclassified Ilzatvirus
GCA_003034635.1	41804	63	Microbacterium phage Bandik	NA	Viruses; Duplodnaviria; Heunggongvirae; Uroviricota; Caudoviricetes; Caudovirales; Siphoviridae; Ilzatvirus; unclassified Ilzatvirus
GCA_003034645.1	42027	64	Microbacterium phage BeeBee8	NA	Viruses; Duplodnaviria; Heunggongvirae; Uroviricota; Caudoviricetes; Caudovirales; Siphoviridae; Ilzatvirus; unclassified Ilzatvirus
GCA_003034655.1	17451	26	Microbacterium phage BonaeVitae	NA	Viruses; Duplodnaviria; Heunggongvirae; Uroviricota; Caudoviricetes; Caudovirales; Siphoviridae; unclassified Siphoviridae
GCA_003034665.1	17445	25	Microbacterium phage BurtonThePup	NA	Viruses; Duplodnaviria; Heunggongvirae; Uroviricota; Caudoviricetes; Caudovirales; Siphoviridae; unclassified Siphoviridae
GCA_003034675.1	41858	63	Microbacterium phage Dave	NA	Viruses; Duplodnaviria; Heunggongvirae; Uroviricota; Caudoviricetes; Caudovirales; Siphoviridae; Ilzatvirus; unclassified Ilzatvirus
GCA_003034755.1	42655	74	Microbacterium phage Didgeridoo	NA	Viruses; Duplodnaviria; Heunggongvirae; Uroviricota; Caudoviricetes; Caudovirales; Siphoviridae; Dismasvirus; unclassified Dismasvirus
GCA_003034765.1	42139	71	Microbacterium phage Elva	NA	Viruses; Duplodnaviria; Heunggongvirae; Uroviricota; Caudoviricetes; Caudovirales; Siphoviridae; Dismasvirus; unclassified Dismasvirus
GCA_003034685.1	41908	63	Microbacterium phage Etna	NA	Viruses; Duplodnaviria; Heunggongvirae; Uroviricota; Caudoviricetes; Caudovirales; Siphoviridae; Ilzatvirus; unclassified Ilzatvirus
GCA_003034815.1	41417	64	Microbacterium phage Kieran	NA	Viruses; Duplodnaviria; Heunggongvirae; Uroviricota; Caudoviricetes; Caudovirales; Siphoviridae; Dismasvirus; unclassified Dismasvirus
GCA_003034695.1	41846	63	Microbacterium phage Nagem	NA	Viruses; Duplodnaviria; Heunggongvirae; Uroviricota; Caudoviricetes; Caudovirales; Siphoviridae; Ilzatvirus; unclassified Ilzatvirus
GCA_003034705.1	17362	25	Microbacterium phage PaoPu	NA	Viruses; Duplodnaviria; Heunggongvirae; Uroviricota; Caudoviricetes; Caudovirales; Siphoviridae; unclassified Siphoviridae
GCA_003034715.1	41801	63	Microbacterium phage Raptor	NA	Viruses; Duplodnaviria; Heunggongvirae; Uroviricota; Caudoviricetes; Caudovirales; Siphoviridae; Ilzatvirus; unclassified Ilzatvirus
GCA_003034725.1	41874	63	Microbacterium phage Robinson	NA	Viruses; Duplodnaviria; Heunggongvirae; Uroviricota; Caudoviricetes; Caudovirales; Siphoviridae; Ilzatvirus; unclassified Ilzatvirus
GCA_003034735.1	41555	62	Microbacterium phage TeddyBear	NA	Viruses; Duplodnaviria; Heunggongvirae; Uroviricota; Caudoviricetes; Caudovirales; Siphoviridae; Ilzatvirus; unclassified Ilzatvirus
GCA_003034775.1	41441	61	Mycobacterium phage Schiebel	NA	Viruses; Duplodnaviria; Heunggongvirae; Uroviricota; Caudoviricetes; Caudovirales; Siphoviridae; Liefievirus; unclassified Liefievirus
GCA_003051445.1	52618	94	Mycobacterium phage Fameo	NA	Viruses; Duplodnaviria; Heunggongvirae; Uroviricota; Caudoviricetes; Caudovirales; Siphoviridae; Fromanvirus; unclassified Fromanvirus
GCA_003051325.1	59812	96	Mycobacterium phage ActinUp	NA	Viruses; Duplodnaviria; Heunggongvirae; Uroviricota; Caudoviricetes; Caudovirales; Siphoviridae; Timquatrovirus; unclassified Timquatrovirus
GCA_003051345.1	51418	87	Mycobacterium phage Annyong	NA	Viruses; Duplodnaviria; Heunggongvirae; Uroviricota; Caudoviricetes; Caudovirales; Siphoviridae; Fromanvirus; unclassified Fromanvirus
GCA_003051365.1	53618	100	Mycobacterium phage BigPhil	NA	Viruses; Duplodnaviria; Heunggongvirae; Uroviricota; Caudoviricetes; Caudovirales; Siphoviridae; Cheoctovirus; unclassified Cheoctovirus
GCA_003051385.1	67491	92	Mycobacterium phage Boyle	NA	Viruses; Duplodnaviria; Heunggongvirae; Uroviricota; Caudoviricetes; Caudovirales; Siphoviridae; Bclasvirinae; Rosebushvirus; unclassified Rosebushvirus

gca	genome.size	proteome.size	phage.name	strain	Taxonomy
GCA_003051405.1	51215	84	Mycobacterium phage Coog	NA	Viruses; Duplodnaviria; Heunggongvirae; Uroviricota; Caudoviricetes; Caudovirales; Siphoviridae; Fromanvirus; unclassified Fromanvirus
GCA_003051425.1	68235	99	Mycobacterium phage DuchessDung	NA	Viruses; Duplodnaviria; Heunggongvirae; Uroviricota; Caudoviricetes; Caudovirales; Siphoviridae; Bclasvirinae; Pegunavirus; unclassified Pegunavirus
GCA_003051465.1	52091	88	Mycobacterium phage GreedyLawyer	NA	Viruses; Duplodnaviria; Heunggongvirae; Uroviricota; Caudoviricetes; Caudovirales; Siphoviridae; Fromanvirus; unclassified Fromanvirus
GCA_003051485.1	50530	86	Mycobacterium phage Jabiru	NA	Viruses; Duplodnaviria; Heunggongvirae; Uroviricota; Caudoviricetes; Caudovirales; Siphoviridae; Fromanvirus; unclassified Fromanvirus
GCA_003051505.1	61808	94	Mycobacterium phage Leston	NA	Viruses; Duplodnaviria; Heunggongvirae; Uroviricota; Caudoviricetes; Caudovirales; Siphoviridae; Timquatrovirus; unclassified Timquatrovirus
GCA_003051525.1	51215	84	Mycobacterium phage Midas2	NA	Viruses; Duplodnaviria; Heunggongvirae; Uroviricota; Caudoviricetes; Caudovirales; Siphoviridae; Fromanvirus; unclassified Fromanvirus
GCA_003051545.1	56515	103	Mycobacterium phage OwlsT2W	NA	Viruses; Duplodnaviria; Heunggongvirae; Uroviricota; Caudoviricetes; Caudovirales; Siphoviridae; Cheoctovirus; unclassified Cheoctovirus
GCA_003051565.1	58351	94	Mycobacterium phage SamScheppers	NA	Viruses; Duplodnaviria; Heunggongvirae; Uroviricota; Caudoviricetes; Caudovirales; Siphoviridae; Timquatrovirus; unclassified Timquatrovirus
GCA_003051585.1	50413	91	Mycobacterium phage TNguyen7	NA	Viruses; Duplodnaviria; Heunggongvirae; Uroviricota; Caudoviricetes; Caudovirales; Siphoviridae; Fromanvirus; unclassified Fromanvirus
GCA_003051625.1	156679	231	Mycobacterium phage Tyke	NA	Viruses; Duplodnaviria; Heunggongvirae; Uroviricota; Caudoviricetes; Caudovirales; Myoviridae; Bixzunavirus; unclassified Bixzunavirus
GCA_003051605.1	156221	237	Mycobacterium phage InterFolia	NA	Viruses; Duplodnaviria; Heunggongvirae; Uroviricota; Caudoviricetes; Caudovirales; Myoviridae; Bixzunavirus; unclassified Bixzunavirus
GCA_000865105.1	5470	1	Sclerotinia sclerotiorum debilitationassociated RNA virus	NA	Viruses; ssRNA viruses; ssRNA positive-strand viruses, no DNA stage; Tymovirales; Alphaflexiviridae; Sclerodarnavirus
GCA_002758395.1	68999	100	Mycobacterium phage PG1	NA	Viruses; Duplodnaviria; Heunggongvirae; Uroviricota; Caudoviricetes; Caudovirales; Siphoviridae; Bclasvirinae; Pegunavirus; Mycobacterium virus Pg1
GCA_003031035.1	51818	87	Salmonella phage vB_SenS_PHB07	NA	Viruses; Duplodnaviria; Heunggongvirae; Uroviricota; Caudoviricetes; Caudovirales; Drexlerviridae; Tempevirinae; Tlsvirus; Salmonella virus PHB07
GCA_004131465.1	9811	1	Lily yellow mosaic virus	NA	Viruses; Riboviria; Orthornavirae; Pisuviricota; Stelpaviricetes; Patatavirales; Potyviridae; Potyvirus; Lily virus Y
GCA_000887375.1	6155	4	Providence virus	NA	Viruses; ssRNA viruses; ssRNA positive-strand viruses, no DNA stage; Carmotetraviridae; Alphacarmotetravirus
GCA_003183805.1	250739	287	Salmonella phage SPAsTU	NA	Viruses; Duplodnaviria; Heunggongvirae; Uroviricota; Caudoviricetes; Caudovirales; Myoviridae; Seoulvirus; unclassified Seoulvirus
GCA_004117175.1	10645	6	Oz virus	EH8	Viruses; Riboviria; Orthornavirae; Negarnaviricota; Polyploviricotina; Insthoviricetes; Articulavirales; Orthomyxoviridae; Thogotovirus; unclassified Thogotovirus
GCA_006451625.1	111347	116	South American cichlid iridovirus	NA	Viruses; Varidnaviria; Bamfordvirae; Nucleocytoviricota; Megaviricetes; Pimascovirales; Iridoviridae; Alphairidovirinae; Megalocytivirus; unclassified Megalocytivirus
GCA_006451655.1	111591	116	Three spot gourami iridovirus	NA	Viruses; Varidnaviria; Bamfordvirae; Nucleocytoviricota; Megaviricetes; Pimascovirales; Iridoviridae; Alphairidovirinae; Megalocytivirus; unclassified Megalocytivirus
GCA_006451765.1	368078	472	Marseillevirus Shanghai 1	NA	Viruses; Varidnaviria; Bamfordvirae; Nucleocytoviricota; Megaviricetes; Pimascovirales; Marseilleviridae; Marseillevirus; unclassified Marseillevirus
GCA_003729235.1	7567	2	Pythium polare bunyalike RNA virus 1	PpBRV1OPU1176	Viruses; unclassified viruses
GCA_004130755.1	5397	2	Pythium polare RNA virus 1	PpRV1OPU1176	Viruses; Riboviria; Orthornavirae; Duplornaviricota; Chrymotiviricetes; Ghabrivirales; Totiviridae; unclassified Totiviridae
GCA_004130775.1	5491	1	Pythium polare RNA virus 2	PpRV2OPU1176	Viruses; Riboviria; Orthornavirae; Duplornaviricota; Chrymotiviricetes; Ghabrivirales; Totiviridae; unclassified Totiviridae
GCA_000868485.1	30510	55	Lactococcus virus 712	NA	Viruses; Duplodnaviria; Heunggongvirae; Uroviricota; Caudoviricetes; Caudovirales; Siphoviridae; Skunavirus
GCA_002592945.1	30796	58	Lactococcus lactis phage P113G	NA	Viruses; Duplodnaviria; Heunggongvirae; Uroviricota; Caudoviricetes; Caudovirales; Siphoviridae; Skunavirus; unclassified Skunavirus
GCA_002592985.1	30961	57	Lactococcus lactis phage P475	NA	Viruses; Duplodnaviria; Heunggongvirae; Uroviricota; Caudoviricetes; Caudovirales; Siphoviridae; Skunavirus; unclassified Skunavirus
GCA_003034835.2	160766	219	Enterobacter phage EspM4VN	NA	Viruses; Duplodnaviria; Heunggongvirae; Uroviricota; Caudoviricetes; Caudovirales; Ackermannviridae; Aglimvirinae; Agtrevirus; unclassified Agtrevirus
GCA_000870105.1	27453	49	Lactococcus virus jj50	NA	Viruses; Duplodnaviria; Heunggongvirae; Uroviricota; Caudoviricetes; Caudovirales; Siphoviridae; Skunavirus
GCA_002592965.1	30778	61	Lactococcus lactis phage p272	NA	Viruses; Duplodnaviria; Heunggongvirae; Uroviricota; Caudoviricetes; Caudovirales; Siphoviridae; Skunavirus; unclassified Skunavirus
GCA_004030215.1	839	2	Mini PCVlike virus	NA	Viruses; Monodnaviria; Shotokuvirae; Cressdnaviricota; Arfiviricetes; Cirlivirales; Circoviridae; Circovirus; unclassified Circovirus
GCA_002592925.1	30674	53	Lactococcus phage fd13	NA	Viruses; Duplodnaviria; Heunggongvirae; Uroviricota; Caudoviricetes; Caudovirales; Siphoviridae; Skunavirus; unclassified Skunavirus
GCA_004133125.1	7644	2	Mume virus A	NA	Viruses; Riboviria; Orthornavirae; Kitrinoviricota; Alsuviricetes; Tymovirales; Betaflexiviridae; Trivirinae; Capillovirus
GCA_003044105.1	115739	141	Aeromonas phage AhSzw1	NA	Viruses; Duplodnaviria; Heunggongvirae; Uroviricota; Caudoviricetes; Caudovirales; Demerecviridae; Shenzhenvirus; Aeromonas virus AhSzw1

gca	genome.size	proteome.size	phage.name	strain	Taxonomy
GCA_004117235.1	12422	2	Bipolaris maydis botybirnavirus 1	JZ11	Viruses; Riboviria; Orthornavirae; Botybirnavirus; unclassified Botybirnavirus
GCA_000900115.1	7916	8	Dahlia mosaic virus	NA	Viruses; Riboviria; Pararnavirae; Artverviricota; Revtraviricetes; Ortervirales; Caulimoviridae; Caulimovirus
GCA_004794915.1	151671	266	Escherichia phage vB_EcoMRo121c4YLVW	NA	Viruses; Duplodnaviria; Heunggongvirae; Uroviricota; Caudoviricetes; Caudovirales; Myoviridae; Phapecoctavirus; unclassified Phapecoctavirus
GCA_004768885.1	72179	109	Escherichia phage vB_EcoMRo157lw	NA	Viruses; Duplodnaviria; Heunggongvirae; Uroviricota; Caudoviricetes; Caudovirales; Myoviridae; Wifcevirus; unclassified Wifcevirus
GCA_004768855.1	42145	71	Escherichia phage vB_EcoSRo145c2YLVW	NA	Viruses; Duplodnaviria; Heunggongvirae; Uroviricota; Caudoviricetes; Caudovirales; Siphoviridae; Guernseyvirinae; Kagunavirus; unclassified Kagunavirus
GCA_000880815.1	6528	5	Hosta virus X	type strain HVXKr	Viruses; ssRNA viruses; ssRNA positive-strand viruses, no DNA stage; Tymovirales; Alphaflexiviridae; Potexvirus
GCA_000840625.2	39672	51	Salmonella phage epsilon15	NA	Viruses; dsDNA viruses, no RNA stage; Caudovirales; Podoviridae; Epsilon15virus
GCA_000859385.1	43095	67	Staphylococcus virus 3a	NA	Viruses; Duplodnaviria; Heunggongvirae; Uroviricota; Caudoviricetes; Caudovirales; Siphoviridae; Triavirus
GCA_004133105.1	1899	2	Dragonfly associated cyclovirus	NA	Viruses; Monodnaviria; Shotokuvirae; Cressdnaviricota; Arfiviricetes; Cirlivirales; Circoviridae; Cyclovirus; unclassified Cyclovirus
GCA_013087955.1	14486	7	Wheat yellow striate virus	NA	Viruses; Riboviria; Orthornavirae; Negarnaviricota; Haploviricotina; Monjiviricetes; Mononegavirales; Rhabdoviridae; Alphanucleorhabdovirus; Wheat yellow striate alphanucleorhabdovirus
GCA_004133265.1	7312	3	Sogatella furcifera hepelike virus	NA	Viruses; Riboviria; Orthornavirae; Kitrinoviricota; Alsuviricetes; Hepelivirales; Hepeviridae; unclassified Hepeviridae
GCA_003764565.1	44716	60	Pectobacterium phage PPWS2	NA	Viruses; Duplodnaviria; Heunggongvirae; Uroviricota; Caudoviricetes; Caudovirales; Autographiviridae; Corkvirinae; Kotilavirus; Pectobacterium virus PPWS2
GCA_003059785.1	44205	62	Pseudomonas phage vB_PaeP_130_113	NA	Viruses; Duplodnaviria; Heunggongvirae; Uroviricota; Caudoviricetes; Caudovirales; Autographiviridae; Krylovirinae; Phikmvvirus; Pseudomonas virus 130-113
GCA_003059745.1	151627	249	Staphylococcus phage vB_SauM_0414_108	NA	Viruses; Duplodnaviria; Heunggongvirae; Uroviricota; Caudoviricetes; Caudovirales; Herelleviridae; Twortvirinae; Kayvirus; unclassified Kayvirus
GCA_004130475.1	6453	1	Marmot norovirus	HT16	Viruses; Riboviria; Orthornavirae; Pisuviricota; Pisoniviricetes; Picornavirales; Caliciviridae; Norovirus; unclassified Norovirus
GCA_013087965.1	8170	1	Marmot mosavirus	HT8	Viruses; Riboviria; Orthornavirae; Pisuviricota; Pisoniviricetes; Picornavirales; Picornaviridae; Mosavirus; unclassified Mosavirus
GCA_018583095.1	1277	4	Coconut foliar decay alphasatellite 2	NA	Viruses; Alphasatellitidae; Petromoalphasatellitinae; Cocosatellite
GCA_004132025.1	1252	2	Coconut foliar decay alphasatellite 3	NA	Viruses; Alphasatellitidae; Geminialphasatellitinae; unclassified Begomovirus-associated alphasatellites
GCA_018583105.1	1276	4	Coconut foliar decay alphasatellite 4	NA	Viruses; Alphasatellitidae; Petromoalphasatellitinae; Cocosatellite
GCA_018583115.1	1295	4	Coconut foliar decay alphasatellite 5	NA	Viruses; Alphasatellitidae; Petromoalphasatellitinae; Cocosatellite
GCA_004132065.1	1264	1	Coconut foliar decay alphasatellite 6	NA	Viruses; Alphasatellitidae; Geminialphasatellitinae; unclassified Begomovirus-associated alphasatellites
GCA_004132085.1	1259	2	Coconut foliar decay alphasatellite 7	NA	Viruses; Alphasatellitidae; Geminialphasatellitinae; unclassified Begomovirus-associated alphasatellites
GCA_004132865.1	3743	6	Passion fruit chlorotic mottle virus	NA	Viruses; Monodnaviria; Shotokuvirae; Cressdnaviricota; Repensiviricetes; Geplafuvirales; Geminiviridae; unclassified Geminiviridae
GCA_004130855.1	19936	2	Rhizoctonia solani endornavirus 1	NA	Viruses; Riboviria; Orthornavirae; Kitrinoviricota; Alsuviricetes; Martellivirales; Endornaviridae; unclassified Endornaviridae
GCA_013087975.1	5224	5	Red clover associated virus	NA	Viruses; Riboviria; Orthornavirae; Kitrinoviricota; Tolucaviricetes; Tolivirales; Luteoviridae; Luteovirus; unclassified Luteovirus
GCA_003059805.1	139675	208	Staphylococcus phage HYZ21	NA	Viruses; Duplodnaviria; Heunggongvirae; Uroviricota; Caudoviricetes; Caudovirales; Herelleviridae; Twortvirinae; Kayvirus; unclassified Kayvirus
GCA_004133585.1	6052	5	Akodon montensis polyomavirus	NA	Viruses; Monodnaviria; Shotokuvirae; Cossaviricota; Papovaviricetes; Sepolyvirales; Polyomaviridae; unclassified Polyomaviridae
GCA_003143015.1	53225	96	Mycobacterium phage AbbyPaige	NA	Viruses; Duplodnaviria; Heunggongvirae; Uroviricota; Caudoviricetes; Caudovirales; Siphoviridae; Fromanvirus; unclassified Fromanvirus
GCA_003143055.1	57797	111	Mycobacterium phage DillTech15	NA	Viruses; Duplodnaviria; Heunggongvirae; Uroviricota; Caudoviricetes; Caudovirales; Siphoviridae; Cheoctovirus; unclassified Cheoctovirus
GCA_003143075.1	112351	245	Mycobacterium phage Halley	NA	Viruses; Duplodnaviria; Heunggongvirae; Uroviricota; Caudoviricetes; Caudovirales; Siphoviridae; Omegavirus; unclassified Omegavirus
GCA_003143095.1	54356	95	Mycobacterium phage Melissauren88	NA	Viruses; Duplodnaviria; Heunggongvirae; Uroviricota; Caudoviricetes; Caudovirales; Siphoviridae; Cheoctovirus; unclassified Cheoctovirus
GCA_003143115.1	51372	88	Mycobacterium phage Nemo27	NA	Viruses; Duplodnaviria; Heunggongvirae; Uroviricota; Caudoviricetes; Caudovirales; Siphoviridae; Fromanvirus; unclassified Fromanvirus
GCA_003143135.1	68740	98	Mycobacterium phage Olive	NA	Viruses; Duplodnaviria; Heunggongvirae; Uroviricota; Caudoviricetes; Caudovirales; Siphoviridae; Bclasvirinae; Pegunavirus; unclassified Pegunavirus
GCA_003094395.1	50840	92	Mycobacterium phage PGHhamlin	NA	Viruses; Duplodnaviria; Heunggongvirae; Uroviricota; Caudoviricetes; Caudovirales; Siphoviridae; Fromanvirus; unclassified Fromanvirus

gca	genome.size	proteome.size	phage.name	strain	Taxonomy
GCA_003143155.1	41901	62	Mycobacterium phage Phish	NA	Viruses; Duplodnaviria; Heunggongvirae; Uroviricota; Caudoviricetes; Caudovirales; Siphoviridae; Liefievirus; unclassified Liefievirus
GCA_003143175.1	57742	101	Mycobacterium phage TChen	NA	Viruses; Duplodnaviria; Heunggongvirae; Uroviricota; Caudoviricetes; Caudovirales; Siphoviridae; Cheoctovirus; unclassified Cheoctovirus
GCA_006516655.1	286783	445	Pseudomonas phage vB_PaeM_PA5oct	NA	Viruses; Duplodnaviria; Heunggongvirae; Uroviricota; Caudoviricetes; Caudovirales; Myoviridae; unclassified Myoviridae
GCA_003093915.1	41436	49	Dickeya phage Amaethon	NA	Viruses; Duplodnaviria; Heunggongvirae; Uroviricota; Caudoviricetes; Caudovirales; Podoviridae; Kafunavirus; unclassified Kafunavirus
GCA_003094255.2	40484	53	Dickeya phage Dagda	NA	Viruses; Duplodnaviria; Heunggongvirae; Uroviricota; Caudoviricetes; Caudovirales; Autographiviridae; Studiervirinae; Aarhusvirus; Dickeya virus Dagda
GCA_003094335.1	39699	46	Dickeya phage Ninurta	NA	Viruses; Duplodnaviria; Heunggongvirae; Uroviricota; Caudoviricetes; Caudovirales; Autographiviridae; Studiervirinae; Ningirsuvirus; Dickeya virus Ninurta
GCA_003093895.1	39826	55	Dickeya phage Sucellus	NA	Viruses; Duplodnaviria; Heunggongvirae; Uroviricota; Caudoviricetes; Caudovirales; Siphoviridae; unclassified Siphoviridae
GCA_003093935.2	175774	291	Erwinia phage Cronus	NA	Viruses; Duplodnaviria; Heunggongvirae; Uroviricota; Caudoviricetes; Caudovirales; Myoviridae; Tevenvirinae; unclassified Tevenvirinae
GCA_003094295.1	40552	46	Pectobacterium phage Jarilo	NA	Viruses; Duplodnaviria; Heunggongvirae; Uroviricota; Caudoviricetes; Caudovirales; Autographiviridae; Studiervirinae; Jarilovirus; Pectobacterium virus Jarilo
GCA_003094315.1	74509	92	Pectobacterium phage Nepra	NA	Viruses; Duplodnaviria; Heunggongvirae; Uroviricota; Caudoviricetes; Caudovirales; Podoviridae; unclassified Podoviridae
GCA_003094275.1	61939	86	Xanthomonas phage Carpasina	NA	Viruses; Duplodnaviria; Heunggongvirae; Uroviricota; Caudoviricetes; Caudovirales; Myoviridae; Carpasinavirus; Xanthomonas virus Carpasina
GCA_000856185.1	3336	2	Penicillium stoloniferum virus S	NA	Viruses; dsRNA viruses; Partitiviridae; Gammapartitivirus
GCA_003094095.1	49794	77	Enterobacteria phage vB_EcoS_IME167	NA	Viruses; Duplodnaviria; Heunggongvirae; Uroviricota; Caudoviricetes; Caudovirales; Drexlerviridae; Tunavirinae; Tunavirus; unclassified Tunavirus
GCA_003094075.1	50354	82	Enterobacteria phage vB_EcoS_IME18	NA	Viruses; Duplodnaviria; Heunggongvirae; Uroviricota; Caudoviricetes; Caudovirales; Drexlerviridae; Tunavirinae; Tunavirus; Escherichia virus IME18
GCA_003094115.1	170531	276	Enterobacteria phage vB_EcoM_IME281	NA	Viruses; Duplodnaviria; Heunggongvirae; Uroviricota; Caudoviricetes; Caudovirales; Myoviridae; Tevenvirinae; Dhakavirus; unclassified Dhakavirus
GCA_003094135.1	85675	126	Enterobacteria phage vB_EcoM_IME338	NA	Viruses; Duplodnaviria; Heunggongvirae; Uroviricota; Caudoviricetes; Caudovirales; Myoviridae; Ounavirinae; Felixounavirus; unclassified Felixounavirus
GCA_003094155.1	164366	255	Enterobacteria phage vB_EcoM_IME339	NA	Viruses; Duplodnaviria; Heunggongvirae; Uroviricota; Caudoviricetes; Caudovirales; Myoviridae; Tevenvirinae; Tequatrovirus; unclassified Tequatrovirus
GCA_003094175.1	165549	260	Enterobacteria phage vB_EcoM_IME340	NA	Viruses; Duplodnaviria; Heunggongvirae; Uroviricota; Caudoviricetes; Caudovirales; Myoviridae; Tevenvirinae; Tequatrovirus; unclassified Tequatrovirus
GCA_003094195.1	172379	273	Enterobacteria phage vB_EcoM_IME341	NA	Viruses; Duplodnaviria; Heunggongvirae; Uroviricota; Caudoviricetes; Caudovirales; Myoviridae; Tevenvirinae; Dhakavirus; unclassified Dhakavirus
GCA_003094375.1	93641	167	Acinetobacter phage vB_AbaM_B9	NA	Viruses; Duplodnaviria; Heunggongvirae; Uroviricota; Caudoviricetes; Caudovirales; Myoviridae; unclassified Myoviridae
GCA_004132785.1	2568	1	Leptosphaeria biglobosa mitovirus 1	NA	Viruses; Riboviria; Orthornavirae; Lenarviricota; Howeltoviricetes; Cryppavirales; Mitoviridae; Mitovirus; unclassified Mitovirus
GCA_003143195.1	47828	80	Aeromonas phage 13AhydR10PP	NA	Viruses; Duplodnaviria; Heunggongvirae; Uroviricota; Caudoviricetes; Caudovirales; Myoviridae; unclassified Myoviridae
GCA_003143235.1	43755	64	Aeromonas phage 62AhydR11PP	NA	Viruses; Duplodnaviria; Heunggongvirae; Uroviricota; Caudoviricetes; Caudovirales; Myoviridae; unclassified Myoviridae
GCA_003143295.1	47194	80	Aeromonas phage 85AhydR10PP	NA	Viruses; Duplodnaviria; Heunggongvirae; Uroviricota; Caudoviricetes; Caudovirales; Myoviridae; unclassified Myoviridae
GCA_003143395.1	74361	92	Pseudomonas phage 98PfluR60PP	NA	Viruses; Duplodnaviria; Heunggongvirae; Uroviricota; Caudoviricetes; Caudovirales; Podoviridae; unclassified Podoviridae
GCA_003143215.2	40822	53	Pseudomonas phage 22PfluR64PP	NA	Viruses; Duplodnaviria; Heunggongvirae; Uroviricota; Caudoviricetes; Caudovirales; Autographiviridae; Studiervirinae; Pifdecavirus; Pseudomonas virus 22PfluR64PP
GCA_003143375.2	42696	51	Aeromonas phage 25AhydR2PP	NA	Viruses; Duplodnaviria; Heunggongvirae; Uroviricota; Caudoviricetes; Caudovirales; Autographiviridae; Melnykvirinae; Aerosvirus; Aeromonas virus 25AhydR2PP
GCA_003143255.2	40582	52	Pseudomonas phage 71PfluR64PP	NA	Viruses; Duplodnaviria; Heunggongvirae; Uroviricota; Caudoviricetes; Caudovirales; Autographiviridae; Studiervirinae; Pifdecavirus; unclassified Pifdecavirus
GCA_003143275.1	164983	245	Aeromonas phage 50AhydR13PP	NA	Viruses; Duplodnaviria; Heunggongvirae; Uroviricota; Caudoviricetes; Caudovirales; Myoviridae; Tulanevirus; unclassified Tulanevirus
GCA_003143415.1	165795	247	Aeromonas phage 60AhydR15PP	NA	Viruses; Duplodnaviria; Heunggongvirae; Uroviricota; Caudoviricetes; Caudovirales; Myoviridae; Tulanevirus; unclassified Tulanevirus
GCA_003143435.2	40748	51	Pseudomonas phage 67PfluR64PP	NA	Viruses; Duplodnaviria; Heunggongvirae; Uroviricota; Caudoviricetes; Caudovirales; Autographiviridae; Studiervirinae; Pifdecavirus; unclassified Pifdecavirus
GCA_003093995.1	46585	47	Pseudomonas phage Achelous	NA	Viruses; Duplodnaviria; Heunggongvirae; Uroviricota; Caudoviricetes; Caudovirales; Autographiviridae; Colwellvirinae; Uliginvirus; Pseudomonas virus Achelous
GCA_003094015.1	45756	47	Pseudomonas phage Alpheus	NA	Viruses; Duplodnaviria; Heunggongvirae; Uroviricota; Caudoviricetes; Caudovirales; Autographiviridae; Colwellvirinae; Uliginvirus; Pseudomonas virus Alpheus
GCA_003093975.1	45779	45	Pseudomonas phage Nerthus	NA	Viruses; Duplodnaviria; Heunggongvirae; Uroviricota; Caudoviricetes; Caudovirales; Autographiviridae; Colwellvirinae; Uliginvirus; Pseudomonas virus Nerthus

gca	genome.size	proteome.size	phage.name	strain	Taxonomy
GCA_003093955.1	46644	44	Pseudomonas phage Njord	NA	Viruses; Duplodnaviria; Heunggongvirae; Uroviricota; Caudoviricetes; Caudovirales; Autographiviridae; Colwellvirinae; Uliginvirus; Pseudomonas virus Njord
GCA_004989725.1	52031	70	Acinetobacter phage AM106	NA	Viruses; Duplodnaviria; Heunggongvirae; Uroviricota; Caudoviricetes; Caudovirales; Siphoviridae; Vieuvirus; unclassified Vieuvirus
GCA_003143355.1	48335	82	Aeromonas phage 14AhydR10PP	NA	Viruses; Duplodnaviria; Heunggongvirae; Uroviricota; Caudoviricetes; Caudovirales; Myoviridae; unclassified Myoviridae
GCA_004788135.1	3182	6	Capuchin monkey hepatitis B virus	NA	Viruses; Riboviria; Pararnavirae; Artverviricota; Revtraviricetes; Blubervirales; Hepadnaviridae; Orthohepadnavirus
GCA_018591365.1	11551	4	Aspergillus thermomutatus chrysovirus 1	NA	Viruses; Riboviria; Orthornavirae; Duplornaviricota; Chrymotiviricetes; Ghabrivirales; Chrysoviridae; Betachrysovirus
GCA_004131045.1	1702	1	Penicillium digitatum narnalike virus 1	NA	Viruses; Riboviria; Orthornavirae; Lenarviricota; Amabiliviricetes; Wolframvirales; Narnaviridae; unclassified Narnaviridae
GCA_004128095.1	8026	4	Penicillium digitatum polymycoviruses 1	NA	Viruses; Riboviria; Polymycoviridae; Polymycovirus; Penicillium digitatum polymycovirus 1
GCA_013086705.1	10135	3	Apple rubbery wood virus 1	NA	Viruses; Riboviria; Orthornavirae; Negarnaviricota; Polyploviricotina; Ellioviricetes; Bunyavirales; Phenuiviridae; Rubodvirus; Apple rubodvirus 1
GCA_013088665.1	13320	5	Apple rubbery wood virus 2	NA	Viruses; Riboviria; Orthornavirae; Negarnaviricota; Polyploviricotina; Ellioviricetes; Bunyavirales; Phenuiviridae; Rubodvirus; Apple rubodvirus 2
GCA_018582775.1	2740	6	Hibiscus vein enation virus	NA	Viruses; Monodnaviria; Shotokuvirae; Cressdnaviricota; Repensiviricetes; Geplafuvirales; Geminiviridae; Begomovirus; unclassified Begomovirus
GCA_018582785.1	1355	1	Hibiscus vein enation betasatellite	NA	Viruses; Tolecusatellitidae; Betasatellite
GCA_000867245.1	8739	6	Daphne virus S	type strain K	Viruses; ssRNA viruses; ssRNA positive-strand viruses, no DNA stage; Tymovirales; Betaflexiviridae; Quinvirinae; Carlavirus
GCA_016455495.1	112043	119	Large yellow croaker iridovirus	NA	Viruses; Varidnaviria; Bamfordvirae; Nucleocytoviricota; Megaviricetes; Pimascovirales; Iridoviridae; Alphairidovirinae; Megalocytivirus; Infectious spleen and kidney necrosis virus
GCA_000912655.1	159991	154	Klebsiella virus 0507KN21	NA	Viruses; Duplodnaviria; Heunggongvirae; Uroviricota; Caudoviricetes; Caudovirales; Ackermannviridae; Taipeivirus
GCA_001755825.1	14623	1	Winged bean alphaendornavirus 1	NA	Viruses; Riboviria; Orthornavirae; Kitrinoviricota; Alsuviricetes; Martellivirales; Endornaviridae; Alphaendornavirus
GCA_000863005.1	11835	2	Onyongnyong virus	NA	Viruses; Riboviria; Orthornavirae; Kitrinoviricota; Alsuviricetes; Martellivirales; Togaviridae; Alphavirus
GCA_007310695.1	30687	27	Deer atadenovirus A	NA	Viruses; Varidnaviria; Bamfordvirae; Preplasmiviricota; Tectiliviricetes; Rowavirales; Adenoviridae; Atadenovirus
GCA_003029585.1	1096	4	Banana bunchy top alphasatellite 1	NA	Viruses; Alphasatellitidae; Nanoalphasatellitinae; Babusatellite
GCA_003028905.1	1100	1	Banana bunchy top alphasatellite 2	NA	Viruses; Alphasatellitidae; Nanoalphasatellitinae; Babusatellite
GCA_003028945.1	1093	1	Banana bunchy top alphasatellite 3	NA	Viruses; Alphasatellitidae; Nanoalphasatellitinae; Babusatellite
GCA_000913955.1	1127	1	Cardamom bushy dwarf alphasatellite	NA	Viruses; Alphasatellitidae; Nanoalphasatellitinae; Babusatellite
GCA_000882095.1	9467	2	Chaetoceros socialis forma radians RNA virus 1	NA	Viruses; Riboviria; Orthornavirae; Pisuviricota; Pisoniviricetes; Picornavirales; Marnaviridae; Bacillarnavirus
GCA_002005015.1	7454	4	Cacao yellow vein banding virus	NA	Viruses; Riboviria; Pararnavirae; Artverviricota; Revtraviricetes; Ortervirales; Caulimoviridae; Badnavirus
GCA_003029345.1	7702	3	Dioscorea bacilliform RT virus 1	NA	Viruses; Riboviria; Pararnavirae; Artverviricota; Revtraviricetes; Ortervirales; Caulimoviridae; Badnavirus
GCA_003029445.1	7333	3	Dioscorea bacilliform TR virus	DBV9	Viruses; Riboviria; Pararnavirae; Artverviricota; Revtraviricetes; Ortervirales; Caulimoviridae; Badnavirus
GCA_009732195.1	555	1	Schefflera ringspot virus	NA	Viruses; Riboviria; Pararnavirae; Artverviricota; Revtraviricetes; Ortervirales; Caulimoviridae; Badnavirus
GCA_000910695.1	5163	7	Bean white chlorosis mosaic virus	NA	Viruses; Monodnaviria; Shotokuvirae; Cressdnaviricota; Repensiviricetes; Geplafuvirales; Geminiviridae; Begomovirus
GCA_003034025.1	2600	5	Euphorbia mosaic Peru virus	NA	Viruses; Monodnaviria; Shotokuvirae; Cressdnaviricota; Repensiviricetes; Geplafuvirales; Geminiviridae; Begomovirus
GCA_003029055.1	2758	6	Jatropha leaf curl Gujarat virus	NA	Viruses; Monodnaviria; Shotokuvirae; Cressdnaviricota; Repensiviricetes; Geplafuvirales; Geminiviridae; Begomovirus
GCA_003029275.1	2807	6	Sweet potato golden vein Korea virus	NA	Viruses; Monodnaviria; Shotokuvirae; Cressdnaviricota; Repensiviricetes; Geplafuvirales; Geminiviridae; Begomovirus
GCA_001678335.2	2742	6	Telfairia golden mosaic virus	NA	Viruses; Monodnaviria; Shotokuvirae; Cressdnaviricota; Repensiviricetes; Geplafuvirales; Geminiviridae; Begomovirus
GCA_003029115.1	2609	5	Whiteflyassociated begomovirus 1	NA	Viruses; Monodnaviria; Shotokuvirae; Cressdnaviricota; Repensiviricetes; Geplafuvirales; Geminiviridae; Begomovirus
GCA_003029125.1	2590	5	Whiteflyassociated begomovirus 2	NA	Viruses; Monodnaviria; Shotokuvirae; Cressdnaviricota; Repensiviricetes; Geplafuvirales; Geminiviridae; Begomovirus
GCA_003029135.1	2629	5	Whiteflyassociated begomovirus 3	NA	Viruses; Monodnaviria; Shotokuvirae; Cressdnaviricota; Repensiviricetes; Geplafuvirales; Geminiviridae; Begomovirus

gca	genome.size	proteome.size	phage.name	strain	Taxonomy
GCA_003029145.1	2608	5	Whiteflyassociated begomovirus 4	NA	Viruses; Monodnaviria; Shotokuvirae; Cressdnaviricota; Repensiviricetes; Geplafuvirales; Geminiviridae; Begomovirus
GCA_003029155.1	2638	5	Whiteflyassociated begomovirus 6	NA	Viruses; Monodnaviria; Shotokuvirae; Cressdnaviricota; Repensiviricetes; Geplafuvirales; Geminiviridae; Begomovirus
GCA_003029165.1	2767	6	Whiteflyassociated begomovirus 7	NA	Viruses; Monodnaviria; Shotokuvirae; Cressdnaviricota; Repensiviricetes; Geplafuvirales; Geminiviridae; Begomovirus
GCA_002005665.2	144673	153	Mythimna unipuncta granulovirus B	NA	Viruses; Baculoviridae; Betabaculovirus
GCA_001866855.1	11557	1	Botrytis cinerea betaendornavirus 1	HBtom372	Viruses; Riboviria; Orthornavirae; Kitrinoviricota; Alsuviricetes; Martellivirales; Endornaviridae; Betaendornavirus
GCA_002080195.1	5052	5	Vicugna pacos polyomavirus 1	NA	Viruses; Monodnaviria; Shotokuvirae; Cossaviricota; Papovaviricetes; Sepolyvirales; Polyomaviridae; Betapolyomavirus
GCA_001501395.1	5027	3	Lagomorph bocaparvovirus 1	LBoV 16001ITA	Viruses; Monodnaviria; Shotokuvirae; Cossaviricota; Quintoviricetes; Piccovirales; Parvoviridae; Parvovirinae; Bocaparvovirus
GCA_000930935.1	7018	1	Bopivirus A	NA	Viruses; Riboviria; Orthornavirae; Pisuviricota; Pisoniviricetes; Picornavirales; Picornaviridae; Bopivirus
GCA_003033015.1	1900	1	Bat associated circovirus 9	NA	Viruses; Monodnaviria; Shotokuvirae; Cressdnaviricota; Arfiviricetes; Cirlivirales; Circoviridae; Circovirus
GCA_000890255.1	1351	1	Cleome leaf crumple alphasatellite	NA	Viruses; Alphasatellitidae; Geminialphasatellitinae; Clecrusatellite
GCA_000890035.1	1334	2	Melon chlorotic mosaic alphasatellite	NA	Viruses; Alphasatellitidae; Geminialphasatellitinae; Clecrusatellite
GCA_003029425.1	1367	1	Tomato yellow spot alphasatellite	NA	Viruses; Alphasatellitidae; Geminialphasatellitinae; Clecrusatellite
GCA_003029175.1	1370	1	Whitefly associated Guatemala alphasatellite 2	NA	Viruses; Alphasatellitidae; Geminialphasatellitinae; Clecrusatellite
GCA_003029195.1	1300	1	Whitefly associated Puerto Rico alphasatellite 1	NA	Viruses; Alphasatellitidae; Geminialphasatellitinae; Clecrusatellite
GCA_000890935.1	1396	1	Cotton leaf curl Lucknow alphasatellite	NA	Viruses; Alphasatellitidae; Geminialphasatellitinae; Colecusatellite
GCA_001967255.1	1311	1	Pedilanthus leaf curl alphasatellite	NA	Viruses; Alphasatellitidae; Geminialphasatellitinae; Colecusatellite
GCA_000864325.1	1384	1	Sida leaf curl alphasatellite	NA	Viruses; Alphasatellitidae; Geminialphasatellitinae; Colecusatellite
GCA_000842325.1	1363	1	Tomato yellow leaf curl Thailand alphasatellite	NA	Viruses; Alphasatellitidae; Geminialphasatellitinae; Colecusatellite
GCA_003029525.1	1354	1	Tomato yellow leaf curl Yunnan alphasatellite	NA	Viruses; Alphasatellitidae; Geminialphasatellitinae; Colecusatellite
GCA_000925975.1	7321	1	Crohivirus A	ZM54	Viruses; Riboviria; Orthornavirae; Pisuviricota; Pisoniviricetes; Picornavirales; Picornaviridae; Crohivirus
GCA_002008575.1	7085	1	Crohivirus B	NA	Viruses; Riboviria; Orthornavirae; Pisuviricota; Pisoniviricetes; Picornavirales; Picornaviridae; Crohivirus
GCA_001866815.1	2229	2	Mouse associated cyclovirus 1	NA	Viruses; Monodnaviria; Shotokuvirae; Cressdnaviricota; Arfiviricetes; Cirlivirales; Circoviridae; Cyclovirus
GCA_003033415.1	2575	3	Camel associated drosmacovirus 1	NA	Viruses; Monodnaviria; Shotokuvirae; Cressdnaviricota; Arfiviricetes; Cremevirales; Smacoviridae; Drosmacovirus
GCA_003033425.1	2580	3	Camel associated drosmacovirus 2	NA	Viruses; Monodnaviria; Shotokuvirae; Cressdnaviricota; Arfiviricetes; Cremevirales; Smacoviridae; Drosmacovirus
GCA_003032735.1	11649	5	Puma feline foamy virus	NA	Viruses; Riboviria; Pararnavirae; Artverviricota; Revtraviricetes; Ortervirales; Retroviridae; Spumaretrovirinae; Felispumavirus
GCA_003033365.1	5172	6	Erythrura gouldiae polyomavirus 1	1209	Viruses; Monodnaviria; Shotokuvirae; Cossaviricota; Papovaviricetes; Sepolyvirales; Polyomaviridae; Gammapolyomavirus
GCA_003033375.1	5284	6	Lonchura maja polyomavirus 1	145342011	Viruses; Monodnaviria; Shotokuvirae; Cossaviricota; Papovaviricetes; Sepolyvirales; Polyomaviridae; Gammapolyomavirus
GCA_000900895.1	1479	1	Dragonfly associated alphasatellite	NA	Viruses; Alphasatellitidae; Geminialphasatellitinae
GCA_003028925.1	1306	1	Gossypium mustelinum symptomless alphasatellite	NA	Viruses; Alphasatellitidae; Geminialphasatellitinae; Gosmusatellite
GCA_000894775.1	1343	1	Vernonia yellow vein Fujian alphasatellite	NA	Viruses; Alphasatellitidae; Geminialphasatellitinae; Gosmusatellite
GCA_001678835.1	3260	6	Bluegill hepatitis B virus	NA	Viruses; Riboviria; Pararnavirae; Artverviricota; Revtraviricetes; Blubervirales; Hepadnaviridae; Metahepadnavirus; Blue gill hepatitis B virus
GCA_001679895.1	3138	4	Tibetan frog hepatitis B virus	NA	Viruses; Riboviria; Pararnavirae; Artverviricota; Revtraviricetes; Blubervirales; Hepadnaviridae; Herpetohepadnavirus
GCA_003033445.1	2408	2	Chicken associated huchismacovirus 1	RSBR20152	Viruses; Monodnaviria; Shotokuvirae; Cressdnaviricota; Arfiviricetes; Cremevirales; Smacoviridae; Huchismacovirus
GCA_003033465.1	2459	2	Chicken associated huchismacovirus 2	RSBR20153	Viruses; Monodnaviria; Shotokuvirae; Cressdnaviricota; Arfiviricetes; Cremevirales; Smacoviridae; Huchismacovirus

gca	genome.size	proteome.size	phage.name	strain	Taxonomy
GCA_003033475.1	2460	2	Human associated huchismacovirus 1	NA	Viruses; Monodnaviria; Shotokuvirae; Cressdnaviricota; Arfiviricetes; Cremevirales; Smacoviridae; Huchismacovirus
GCA_003033485.1	2471	2	Human associated huchismacovirus 2	NA	Viruses; Monodnaviria; Shotokuvirae; Cressdnaviricota; Arfiviricetes; Cremevirales; Smacoviridae; Huchismacovirus
GCA_003033495.1	2459	2	Human associated huchismacovirus 3	NA	Viruses; Monodnaviria; Shotokuvirae; Cressdnaviricota; Arfiviricetes; Cremevirales; Smacoviridae; Huchismacovirus
GCA_001308655.1	8418	5	Privet ringspot virus	NA	Viruses; Riboviria; Orthornavirae; Kitrinoviricota; Alsuviricetes; Martellivirales; Bromoviridae; Ilarvirus
GCA_002008715.1	7092	1	Kunsagivirus B	NA	Viruses; Riboviria; Orthornavirae; Pisuviricota; Pisoniviricetes; Picornavirales; Picornaviridae; Kunsagivirus
GCA_000849025.1	9202	6	Visnamaedi virus	kv1772	Viruses; Riboviria; Pararnavirae; Artverviricota; Revtraviricetes; Ortervirales; Retroviridae; Orthoretrovirinae; Lentivirus
GCA_000850165.1	5695	6	Barley yellow dwarf virus PAS	NA	Viruses; Riboviria; Orthornavirae; Kitrinoviricota; Tolucaviricetes; Tolivirales; Luteoviridae; Luteovirus
GCA_000859045.1	5677	6	Barley yellow dwarf virus PAV	NA	Viruses; Riboviria; Orthornavirae; Kitrinoviricota; Tolucaviricetes; Tolivirales; Luteoviridae; Luteovirus
GCA_000907695.1	5736	6	Barley yellow dwarf virus kerII	NA	Viruses; Riboviria; Orthornavirae; Kitrinoviricota; Tolucaviricetes; Tolivirales; Luteoviridae; Luteovirus
GCA_000856545.1	10525	4	Argentinian mammarenavirus	XJ13	Viruses; Riboviria; Orthornavirae; Negarnaviricota; Polyploviricotina; Ellioviricetes; Bunyavirales; Arenaviridae; Mammarenavirus
GCA_000855825.1	10499	4	Brazilian mammarenavirus	SPH114202	Viruses; Riboviria; Orthornavirae; Negarnaviricota; Polyploviricotina; Ellioviricetes; Bunyavirales; Arenaviridae; Mammarenavirus
GCA_000856465.1	10416	4	Cali mammarenavirus	AN3739	Viruses; Riboviria; Orthornavirae; Negarnaviricota; Polyploviricotina; Ellioviricetes; Bunyavirales; Arenaviridae; Mammarenavirus
GCA_000880015.1	10494	4	Paraguayan mammarenavirus	12056	Viruses; Riboviria; Orthornavirae; Negarnaviricota; Polyploviricotina; Ellioviricetes; Bunyavirales; Arenaviridae; Mammarenavirus
GCA_003032625.1	10507	4	Ryukyu mammarenavirus	NA	Viruses; Riboviria; Orthornavirae; Negarnaviricota; Polyploviricotina; Ellioviricetes; Bunyavirales; Arenaviridae; Mammarenavirus
GCA_000879015.1	10387	4	Serra do Navio mammarenavirus	BeAn 70563	Viruses; Riboviria; Orthornavirae; Negarnaviricota; Polyploviricotina; Ellioviricetes; Bunyavirales; Arenaviridae; Mammarenavirus
GCA_001856635.1	6719	3	Grapevine asteroid mosaic associated virus	NA	Viruses; Riboviria; Orthornavirae; Kitrinoviricota; Alsuviricetes; Tymovirales; Tymoviridae; Marafivirus
GCA_002163405.1	33945	28	Deer mastadenovirus B	NA	Viruses; Varidnaviria; Bamfordvirae; Preplasmiviricota; Tectiliviricetes; Rowavirales; Adenoviridae; Mastadenovirus
GCA_000920755.1	9700	1	Megrivirus A	LY	Viruses; Riboviria; Orthornavirae; Pisuviricota; Pisoniviricetes; Picornavirales; Picornaviridae; Megrivirus
GCA_004117715.1	10762	2	Potato virus U	NA	Viruses; Riboviria; Orthornavirae; Pisuviricota; Pisoniviricetes; Picornavirales; Secoviridae; Comovirinae; Nepovirus
GCA_000926835.1	7037	1	Orivirus A	chickenPfCHK12013HUN	Viruses; Riboviria; Orthornavirae; Pisuviricota; Pisoniviricetes; Picornavirales; Picornaviridae; Orivirus
GCA_004790355.1	17934	3	Estero Real virus	NA	Viruses; Riboviria; Orthornavirae; Negarnaviricota; Polyploviricotina; Ellioviricetes; Bunyavirales; Nairoviridae; Orthonairovirus; Estero Real orthonairovirus
GCA_003032565.1	17454	3	Artashat orthonairovirus	LEIV10898Az	Viruses; Riboviria; Orthornavirae; Negarnaviricota; Polyploviricotina; Ellioviricetes; Bunyavirales; Nairoviridae; Orthonairovirus
GCA_003032545.1	18510	3	Chim orthonairovirus	LEIV858Uz	Viruses; Riboviria; Orthornavirae; Negarnaviricota; Polyploviricotina; Ellioviricetes; Bunyavirales; Nairoviridae; Orthonairovirus
GCA_018595155.1	17472	3	Tamdy orthonairovirus	NA	Viruses; Riboviria; Orthornavirae; Negarnaviricota; Polyploviricotina; Ellioviricetes; Bunyavirales; Nairoviridae; Orthonairovirus
GCA_001630105.1	23195	11	Mahlapitsi orthoreovirus	NA	Viruses; Riboviria; Orthornavirae; Duplornaviricota; Resentoviricetes; Reovirales; Reoviridae; Spinareovirinae; Orthoreovirus
GCA_000926015.1	12983	1	Pestivirus J	NA	Viruses; Riboviria; Orthornavirae; Kitrinoviricota; Flasuviricetes; Amarillovirales; Flaviviridae; Pestivirus
GCA_000853365.1	5723	6	Cereal yellow dwarf virus RPV	NA	Viruses; Riboviria; Orthornavirae; Kitrinoviricota; Tolucaviricetes; Tolivirales; Luteoviridae; Polerovirus
GCA_000909295.1	5612	6	Maize yellow dwarf virus RMV	RMV MTFE87	Viruses; Riboviria; Orthornavirae; Kitrinoviricota; Tolucaviricetes; Tolivirales; Luteoviridae; Polerovirus
GCA_000928315.1	3962	4	Rhynchobatus djiddensis polyomavirus 1	NA	Viruses; Monodnaviria; Shotokuvirae; Cossaviricota; Papovaviricetes; Sepolyvirales; Polyomaviridae
GCA_000989095.1	6219	4	Trematomus pennellii polyomavirus 1	NA	Viruses; Monodnaviria; Shotokuvirae; Cossaviricota; Papovaviricetes; Sepolyvirales; Polyomaviridae
GCA_003033505.1	2565	3	Camel associated porprismacovirus 1	NA	Viruses; Monodnaviria; Shotokuvirae; Cressdnaviricota; Arfiviricetes; Cremevirales; Smacoviridae; Porprismacovirus
GCA_003033515.1	2516	2	Camel associated porprismacovirus 2	NA	Viruses; Monodnaviria; Shotokuvirae; Cressdnaviricota; Arfiviricetes; Cremevirales; Smacoviridae; Porprismacovirus
GCA_003033525.1	2582	2	Camel associated porprismacovirus 3	NA	Viruses; Monodnaviria; Shotokuvirae; Cressdnaviricota; Arfiviricetes; Cremevirales; Smacoviridae; Porprismacovirus
GCA_003033535.1	2575	2	Camel associated porprismacovirus 4	NA	Viruses; Monodnaviria; Shotokuvirae; Cressdnaviricota; Arfiviricetes; Cremevirales; Smacoviridae; Porprismacovirus

gca	genome.size	proteome.size	phage.name	strain	Taxonomy
GCA_003033555.1	2609	2	Chimpanzee associated porprismacovirus 1	NA	Viruses; Monodnaviria; Shotokuvirae; Cressdnaviricota; Arfiviricetes; Cremevirales; Smacoviridae; Porprismacovirus
GCA_003033565.1	2640	6	Chimpanzee associated porprismacovirus 2	NA	Viruses; Monodnaviria; Shotokuvirae; Cressdnaviricota; Arfiviricetes; Cremevirales; Smacoviridae; Porprismacovirus
GCA_000929415.1	2532	2	Gorilla associated porprismacovirus 1	NA	Viruses; Monodnaviria; Shotokuvirae; Cressdnaviricota; Arfiviricetes; Cremevirales; Smacoviridae; Porprismacovirus
GCA_000931115.1	2485	2	Howler monkey associated porprismacovirus 1	NA	Viruses; Monodnaviria; Shotokuvirae; Cressdnaviricota; Arfiviricetes; Cremevirales; Smacoviridae; Porprismacovirus
GCA_000931315.1	2529	2	Human associated porprismacovirus 2	NA	Viruses; Monodnaviria; Shotokuvirae; Cressdnaviricota; Arfiviricetes; Cremevirales; Smacoviridae; Porprismacovirus
GCA_000928535.1	2651	2	Lemur associated porprismacovirus 1	NA	Viruses; Monodnaviria; Shotokuvirae; Cressdnaviricota; Arfiviricetes; Cremevirales; Smacoviridae; Porprismacovirus
GCA_000897655.1	2589	2	Porcine associated porprismacovirus 1	NA	Viruses; Monodnaviria; Shotokuvirae; Cressdnaviricota; Arfiviricetes; Cremevirales; Smacoviridae; Porprismacovirus
GCA_001646255.1	2684	2	Porcine associated porprismacovirus 10	NA	Viruses; Monodnaviria; Shotokuvirae; Cressdnaviricota; Arfiviricetes; Cremevirales; Smacoviridae; Porprismacovirus
GCA_000906435.1	2495	2	Porcine associated porprismacovirus 3	NA	Viruses; Monodnaviria; Shotokuvirae; Cressdnaviricota; Arfiviricetes; Cremevirales; Smacoviridae; Porprismacovirus
GCA_003033615.1	2403	2	Porcine associated porprismacovirus 4	NA	Viruses; Monodnaviria; Shotokuvirae; Cressdnaviricota; Arfiviricetes; Cremevirales; Smacoviridae; Porprismacovirus
GCA_000926055.1	2590	2	Porcine associated porprismacovirus 5	NA	Viruses; Monodnaviria; Shotokuvirae; Cressdnaviricota; Arfiviricetes; Cremevirales; Smacoviridae; Porprismacovirus
GCA_000922455.1	2603	2	Porcine associated porprismacovirus 6	NA	Viruses; Monodnaviria; Shotokuvirae; Cressdnaviricota; Arfiviricetes; Cremevirales; Smacoviridae; Porprismacovirus
GCA_000921615.1	2601	2	Porcine associated porprismacovirus 7	NA	Viruses; Monodnaviria; Shotokuvirae; Cressdnaviricota; Arfiviricetes; Cremevirales; Smacoviridae; Porprismacovirus
GCA_000924135.1	2477	2	Porcine associated porprismacovirus 8	NA	Viruses; Monodnaviria; Shotokuvirae; Cressdnaviricota; Arfiviricetes; Cremevirales; Smacoviridae; Porprismacovirus
GCA_000926035.1	2564	2	Porcine associated porprismacovirus 9	NA	Viruses; Monodnaviria; Shotokuvirae; Cressdnaviricota; Arfiviricetes; Cremevirales; Smacoviridae; Porprismacovirus
GCA_001273705.1	2530	2	Rat associated porprismacovirus 1	NA	Viruses; Monodnaviria; Shotokuvirae; Cressdnaviricota; Arfiviricetes; Cremevirales; Smacoviridae; Porprismacovirus
GCA_000918075.1	2479	2	Turkey associated porprismacovirus 1	TuSCV	Viruses; Monodnaviria; Shotokuvirae; Cressdnaviricota; Arfiviricetes; Cremevirales; Smacoviridae; Porprismacovirus
GCA_001717275.1	9837	1	Barbacena virus Y	NA	Viruses; Riboviria; Orthornavirae; Pisuviricota; Stelpaviricetes; Patatavirales; Potyviridae; Potyvirus
GCA_001654345.1	9622	1	Impatiens flower break virus	NA	Viruses; Riboviria; Orthornavirae; Pisuviricota; Stelpaviricetes; Patatavirales; Potyviridae; Potyvirus
GCA_003032745.1	12118	4	Brown greater galago prosimian foamy virus	NA	Viruses; Riboviria; Pararnavirae; Artverviricota; Revtraviricetes; Ortervirales; Retroviridae; Spumaretrovirinae; Prosimiispumavirus
GCA_000863905.1	6000	6	Chaetoceros protobacilladnavirus 1	NA	Viruses; Monodnaviria; Shotokuvirae; Cressdnaviricota; Arfiviricetes; Baphyvirales; Bacilladnaviridae; Protobacilladnavirus
GCA_000892315.1	5813	3	Chaetoceros protobacilladnavirus 3	ClorDNAV01	Viruses; Monodnaviria; Shotokuvirae; Cressdnaviricota; Arfiviricetes; Baphyvirales; Bacilladnaviridae; Protobacilladnavirus
GCA_000887835.1	5639	3	Chaetoceros protobacilladnavirus 4	NA	Viruses; Monodnaviria; Shotokuvirae; Cressdnaviricota; Arfiviricetes; Baphyvirales; Bacilladnaviridae; Protobacilladnavirus
GCA_000973435.1	4613	3	Eulipotyphla protoparvovirus 1	ZM38	Viruses; Monodnaviria; Shotokuvirae; Cossaviricota; Quintoviricetes; Piccovirales; Parvoviridae; Parvovirinae; Protoparvovirus
GCA_001876835.1	9789	2	Dioscorea mosaic associated virus	NA	Viruses; Riboviria; Orthornavirae; Pisuviricota; Pisoniviricetes; Picornavirales; Secoviridae; Sadwavirus; Cholivirus
GCA_003029045.1	7264	1	Shanbavirus A	NA	Viruses; Riboviria; Orthornavirae; Pisuviricota; Pisoniviricetes; Picornavirales; Picornaviridae; Shanbavirus
GCA_003032755.1	13220	5	Central cimpanzee simian foamy virus	Pan troglodytes troglodytes	Viruses; Riboviria; Pararnavirae; Artverviricota; Revtraviricetes; Ortervirales; Retroviridae; Spumaretrovirinae; Simiispumavirus
GCA_003032765.1	11954	5	Eastern chimpanzee simian foamy virus	NA	Viruses; Riboviria; Pararnavirae; Artverviricota; Revtraviricetes; Ortervirales; Retroviridae; Spumaretrovirinae; Simiispumavirus
GCA_000874485.1	13111	6	Grivet simian foamy virus	NA	Viruses; Riboviria; Pararnavirae; Artverviricota; Revtraviricetes; Ortervirales; Retroviridae; Spumaretrovirinae; Simiispumavirus
GCA_003032775.1	13072	5	Guenon simian foamy virus	Cercopithecus nictitans	Viruses; Riboviria; Pararnavirae; Artverviricota; Revtraviricetes; Ortervirales; Retroviridae; Spumaretrovirinae; Simiispumavirus
GCA_003032785.1	12957	5	Japanese macaque simian foamy virus	NA	Viruses; Riboviria; Pararnavirae; Artverviricota; Revtraviricetes; Ortervirales; Retroviridae; Spumaretrovirinae; Simiispumavirus
GCA_003032795.1	12983	5	Rhesus macaque simian foamy virus	NA	Viruses; Riboviria; Pararnavirae; Artverviricota; Revtraviricetes; Ortervirales; Retroviridae; Spumaretrovirinae; Simiispumavirus
GCA_003032805.1	12212	5	Spider monkey simian foamy virus	ATCC VR940	Viruses; Riboviria; Pararnavirae; Artverviricota; Revtraviricetes; Ortervirales; Retroviridae; Spumaretrovirinae; Simiispumavirus
GCA_003032815.1	11684	5	Squirrel monkey simian foamy virus	ATCC VR643	Viruses; Riboviria; Pararnavirae; Artverviricota; Revtraviricetes; Ortervirales; Retroviridae; Spumaretrovirinae; Simiispumavirus

gca	genome.size	proteome.size	phage.name	strain	Taxonomy
GCA_000848485.1	13246	5	Western chimpanzee simian foamy virus	SFVcpz	Viruses; Riboviria; Pararnavirae; Artverviricota; Revtraviricetes; Ortervirales; Retroviridae; Spumaretrovirinae; Simiispumavirus
GCA_003032825.1	12258	6	Western lowland gorilla simian foamy virus	NA	Viruses; Riboviria; Pararnavirae; Artverviricota; Revtraviricetes; Ortervirales; Retroviridae; Spumaretrovirinae; Simiispumavirus
GCA_003032835.1	11744	5	Whitetuftedear marmoset simian foamy virus	NA	Viruses; Riboviria; Pararnavirae; Artverviricota; Revtraviricetes; Ortervirales; Retroviridae; Spumaretrovirinae; Simiispumavirus
GCA_003032845.1	11764	5	Yellowbreasted capuchin simian foamy virus	NA	Viruses; Riboviria; Pararnavirae; Artverviricota; Revtraviricetes; Ortervirales; Retroviridae; Spumaretrovirinae; Simiispumavirus
GCA_018583795.1	12041	5	Lasius neglectus virus 2	NA	Viruses; Riboviria; Orthornavirae; Pisuviricota; Pisoniviricetes; Picornavirales; Polycipiviridae; Sopolycivirus
GCA_000869745.1	9345	2	Homalodisca coagulata virus 1	NA	Viruses; Riboviria; Orthornavirae; Pisuviricota; Pisoniviricetes; Picornavirales; Dicistroviridae; Triatovirus
GCA_000922155.1	9292	1	Yellow oat grass mosaic virus	NA	Viruses; Riboviria; Orthornavirae; Pisuviricota; Stelpaviricetes; Patatavirales; Potyviridae; Tritimovirus
GCA_004132925.1	5234	9	Tupaia glis polyomavirus 1	NA	Viruses; Monodnaviria; Shotokuvirae; Cossaviricota; Papovaviricetes; Sepolyvirales; Polyomaviridae; unclassified Polyomaviridae
GCA_018583475.1	5215	6	Canis lupus polyomavirus 1	NA	Viruses; Monodnaviria; Shotokuvirae; Cossaviricota; Papovaviricetes; Sepolyvirales; Polyomaviridae; Deltapolyomavirus
GCA_004132885.1	5338	6	Glis glis polyomavirus 1	NA	Viruses; Monodnaviria; Shotokuvirae; Cossaviricota; Papovaviricetes; Sepolyvirales; Polyomaviridae; unclassified Polyomaviridae
GCA_004132905.1	5018	10	Panthera leo polyomavirus 1	NA	Viruses; Monodnaviria; Shotokuvirae; Cossaviricota; Papovaviricetes; Sepolyvirales; Polyomaviridae; unclassified Polyomaviridae
GCA_018583445.1	5327	9	Apodemus flavicollis polyomavirus 1	NA	Viruses; Monodnaviria; Shotokuvirae; Cossaviricota; Papovaviricetes; Sepolyvirales; Polyomaviridae; Alphapolyomavirus
GCA_018583455.1	4700	6	Capra aegagrus polyomavirus 1	NA	Viruses; Monodnaviria; Shotokuvirae; Cossaviricota; Papovaviricetes; Sepolyvirales; Polyomaviridae; Epsilonpolyomavirus
GCA_004132745.1	4825	7	Potamochoerus porcus polyomavirus 1	NA	Viruses; Monodnaviria; Shotokuvirae; Cossaviricota; Papovaviricetes; Sepolyvirales; Polyomaviridae; unclassified Polyomaviridae
GCA_004132765.1	5034	8	Philantomba monticola polyomavirus 1	NA	Viruses; Monodnaviria; Shotokuvirae; Cossaviricota; Papovaviricetes; Sepolyvirales; Polyomaviridae; unclassified Polyomaviridae
GCA_003652105.1	103355	148	Siphoviridae sp	NA	Viruses; Duplodnaviria; Heunggongvirae; Uroviricota; Caudoviricetes; Caudovirales; Siphoviridae; unclassified Siphoviridae
GCA_004132825.1	6818	1	Alfalfa virus F	NA	Viruses; Riboviria; Orthornavirae; Kitrinoviricota; Alsuviricetes; Tymovirales; Tymoviridae; Marafivirus; unclassified Marafivirus
GCA_004130875.1	6001	10	Apple luteovirus 1	NA	Viruses; Riboviria; Orthornavirae; Kitrinoviricota; Tolucaviricetes; Tolivirales; Luteoviridae; Luteovirus
GCA_018583085.1	3744	1	Kiln Barn virus	NA	Viruses; Riboviria; Orthornavirae; Negarnaviricota; Haploviricotina; Monjiviricetes; Mononegavirales; Mymonaviridae; Penicillimonavirus; Penicillimonavirus kilnbarnense
GCA_003288735.1	124002	144	Bacillus phage vB_BceMHSE3	NA	Viruses; Duplodnaviria; Heunggongvirae; Uroviricota; Caudoviricetes; Caudovirales; Myoviridae; unclassified Myoviridae
GCA_013087985.1	4339	2	Murine adenoassociated virus 1	NA	Viruses; Monodnaviria; Shotokuvirae; Cossaviricota; Quintoviricetes; Piccovirales; Parvoviridae; Parvovirinae; Dependoparvovirus; Rodent dependoparvovirus 1
GCA_013087995.1	4449	2	Murine adenoassociated virus 2	NA	Viruses; Monodnaviria; Shotokuvirae; Cossaviricota; Quintoviricetes; Piccovirales; Parvoviridae; Parvovirinae; Dependoparvovirus; Rodent dependoparvovirus 2
GCA_013088005.1	5069	4	Murine bocavirus	NA	Viruses; Monodnaviria; Shotokuvirae; Cossaviricota; Quintoviricetes; Piccovirales; Parvoviridae; Parvovirinae; Bocaparvovirus; Rodent bocaparvovirus 2
GCA_004042915.1	909	1	Murine circovirus	NA	Viruses; Monodnaviria; Shotokuvirae; Cressdnaviricota; Arfiviricetes; Cirlivirales; Circoviridae; Circovirus; unclassified Circovirus
GCA_003848505.1	2129	2	Murine fecesassociated gemycircularvirus 1	NA	Viruses; Monodnaviria; Shotokuvirae; Cressdnaviricota; Repensiviricetes; Geplafuvirales; Genomoviridae; Gemycircularvirus; unclassified Gemycircularvirus
GCA_003848485.1	2418	2	Murine fecesassociated gemycircularvirus 2	NA	Viruses; Monodnaviria; Shotokuvirae; Cressdnaviricota; Repensiviricetes; Geplafuvirales; Genomoviridae; Gemycircularvirus; unclassified Gemycircularvirus
GCA_013088015.1	5091	5	Mus musculus polyomavirus 3	NA	Viruses; Monodnaviria; Shotokuvirae; Cossaviricota; Papovaviricetes; Sepolyvirales; Polyomaviridae; Betapolyomavirus
GCA_003094235.1	40934	64	Enterococcus phage LY0322	NA	Viruses; Duplodnaviria; Heunggongvirae; Uroviricota; Caudoviricetes; Caudovirales; Siphoviridae; Efquatrovirus; Enterococcus virus LY0322
GCA_018582825.1	1369	1	Codiaeum leaf curl betasatellite	NA	Viruses; Tolecusatellitidae; Betasatellite
GCA_018582815.1	4219	3	Canine protoparvovirus	ITA2015297	Viruses; Monodnaviria; Shotokuvirae; Cossaviricota; Quintoviricetes; Piccovirales; Parvoviridae; Parvovirinae; Protoparvovirus; unclassified Protoparvovirus
GCA_000870925.1	1349	1	Tomato leaf curl Philippine betasatellite	NA	Viruses; Tolecusatellitidae; Betasatellite
GCA_018582795.1	2661	6	Sida yellow golden mosaic virus	NA	Viruses; Monodnaviria; Shotokuvirae; Cressdnaviricota; Repensiviricetes; Geplafuvirales; Geminiviridae; Begomovirus
GCA_013088025.2	5225	8	Cowpea bright yellow mosaic virus	NA	Viruses; Monodnaviria; Shotokuvirae; Cressdnaviricota; Repensiviricetes; Geplafuvirales; Geminiviridae; Begomovirus
GCA_004132125.1	21527	8	Rhynchobatus djiddensis adomavirus 1	NA	Viruses; unclassified viruses; unclassified DNA viruses; unclassified dsDNA viruses; Adomaviridae

gca	genome.size	proteome.size	phage.name	strain	Taxonomy
GCA_004132145.1	19275	10	Symphysodon discus adomavirus 1	NA	Viruses; unclassified viruses; unclassified DNA viruses; unclassified dsDNA viruses; Adomaviridae
GCA_003308575.1	49372	76	Escherichia phage Eco_BIFF	NA	Viruses; Duplodnaviria; Heunggongvirae; Uroviricota; Caudoviricetes; Caudovirales; Drexlerviridae; Tunavirinae; Tunavirus; Escherichia virus BIFF
GCA_003147445.1	3405	2	Neurachne minor latent virus	NA	Viruses; Riboviria; Orthornavirae; Pisuviricota; Duplopiviricetes; Durnavirales; Amalgaviridae; unclassified Amalgaviridae
GCA_013088035.1	8261	1	Yam chlorotic necrosis virus	NA	Viruses; Riboviria; Orthornavirae; Pisuviricota; Stelpaviricetes; Patatavirales; Potyviridae; Macluravirus
GCA_003364375.1	17961	25	Clostridium phage CPS2	NA	Viruses; Duplodnaviria; Heunggongvirae; Uroviricota; Caudoviricetes; Caudovirales; Podoviridae; unclassified Podoviridae
GCA_003143315.1	40789	61	Enterococcus phage vB_EfaS_AL3	NA	Viruses; Duplodnaviria; Heunggongvirae; Uroviricota; Caudoviricetes; Caudovirales; Siphoviridae; Efquatrovirus; Enterococcus virus AL3
GCA_003143335.1	40836	62	Enterococcus phage vB_EfaS_AL2	NA	Viruses; Duplodnaviria; Heunggongvirae; Uroviricota; Caudoviricetes; Caudovirales; Siphoviridae; Efquatrovirus; Enterococcus virus AL2
GCA_018595295.1	11973	4	Palo verde broom virus	NA	Viruses; Riboviria; Orthornavirae; Negarnaviricota; Polyploviricotina; Ellioviricetes; Bunyavirales; Fimoviridae; Emaravirus; unclassified Emaravirus
GCA_000895675.1	16404	10	Grapevine leafrollassociated virus 7	NA	Viruses; ssRNA viruses; ssRNA positive-strand viruses, no DNA stage; Closteroviridae; Velarivirus
GCA_000862325.1	16934	9	Little cherry virus 1	UW2	Viruses; ssRNA viruses; ssRNA positive-strand viruses, no DNA stage; Closteroviridae; Velarivirus
GCA_000841005.1	106332	95	Ambystoma tigrinum stebbensi virus	NA	Viruses; Varidnaviria; Bamfordvirae; Nucleocytoviricota; Megaviricetes; Pimascovirales; Iridoviridae; Alphairidovirinae; Ranavirus; Ambystoma tigrinum virus
GCA_008221705.1	65128	111	Listeria phage LPKV022	NA	Viruses; Duplodnaviria; Heunggongvirae; Uroviricota; Caudoviricetes; Caudovirales; Siphoviridae; Homburgvirus; unclassified Homburgvirus
GCA_003182765.1	68296	98	Mycobacterium phage Banjo	NA	Viruses; Duplodnaviria; Heunggongvirae; Uroviricota; Caudoviricetes; Caudovirales; Siphoviridae; Bclasvirinae; Pegunavirus; unclassified Pegunavirus
GCA_003182785.1	68503	98	Mycobacterium phage CheetO	NA	Viruses; Duplodnaviria; Heunggongvirae; Uroviricota; Caudoviricetes; Caudovirales; Siphoviridae; Bclasvirinae; Pegunavirus; unclassified Pegunavirus
GCA_003182805.1	53287	97	Mycobacterium phage ConceptII	NA	Viruses; Duplodnaviria; Heunggongvirae; Uroviricota; Caudoviricetes; Caudovirales; Siphoviridae; Fromanvirus; unclassified Fromanvirus
GCA_003182825.1	51374	86	Mycobacterium phage NorthStar	NA	Viruses; Duplodnaviria; Heunggongvirae; Uroviricota; Caudoviricetes; Caudovirales; Siphoviridae; Fromanvirus; unclassified Fromanvirus
GCA_003182845.1	51745	87	Mycobacterium phage Oogway	NA	Viruses; Duplodnaviria; Heunggongvirae; Uroviricota; Caudoviricetes; Caudovirales; Siphoviridae; Fromanvirus; unclassified Fromanvirus
GCA_003182865.1	49808	71	Mycobacterium phage Xavia	NA	Viruses; Duplodnaviria; Heunggongvirae; Uroviricota; Caudoviricetes; Caudovirales; Siphoviridae; unclassified Siphoviridae
GCA_006869785.2	166487	250	Acinetobacter phage AM101	NA	Viruses; Duplodnaviria; Heunggongvirae; Uroviricota; Caudoviricetes; Caudovirales; Myoviridae; Tevenvirinae; unclassified Tevenvirinae
GCA_003719115.1	75929	78	Erwinia phage phiEaP8	NA	Viruses; Duplodnaviria; Heunggongvirae; Uroviricota; Caudoviricetes; Caudovirales; Podoviridae; Johnsonvirus; unclassified Johnsonvirus
GCA_004768905.1	149803	281	Escherichia phage vB_EcoMRo121lw	NA	Viruses; Duplodnaviria; Heunggongvirae; Uroviricota; Caudoviricetes; Caudovirales; Myoviridae; Phapecoctavirus; unclassified Phapecoctavirus
GCA_006448195.1	35215	16	Human adenovirus 49	NA	Viruses; Varidnaviria; Bamfordvirae; Preplasmiviricota; Tectiliviricetes; Rowavirales; Adenoviridae; Mastadenovirus; Human mastadenovirus D
GCA_003183565.1	50605	76	Streptomyces phage Eddasa	NA	Viruses; Duplodnaviria; Heunggongvirae; Uroviricota; Caudoviricetes; Caudovirales; Siphoviridae; Arquatrovirinae; Likavirus; unclassified Likavirus
GCA_003183585.1	51393	76	Streptomyces phage Goby	NA	Viruses; Duplodnaviria; Heunggongvirae; Uroviricota; Caudoviricetes; Caudovirales; Siphoviridae; Arquatrovirinae; Likavirus; unclassified Likavirus
GCA_003183545.1	51612	73	Streptomyces phage Maneekul	NA	Viruses; Duplodnaviria; Heunggongvirae; Uroviricota; Caudoviricetes; Caudovirales; Siphoviridae; Arquatrovirinae; Likavirus; unclassified Likavirus
GCA_003183525.1	51127	76	Streptomyces phage Nabi	NA	Viruses; Duplodnaviria; Heunggongvirae; Uroviricota; Caudoviricetes; Caudovirales; Siphoviridae; Arquatrovirinae; Likavirus; unclassified Likavirus
GCA_003183505.1	50980	76	Streptomyces phage Rana	NA	Viruses; Duplodnaviria; Heunggongvirae; Uroviricota; Caudoviricetes; Caudovirales; Siphoviridae; Arquatrovirinae; Likavirus; unclassified Likavirus
GCA_003183605.1	51396	76	Streptomyces phage Toma	NA	Viruses; Duplodnaviria; Heunggongvirae; Uroviricota; Caudoviricetes; Caudovirales; Siphoviridae; Arquatrovirinae; Likavirus; unclassified Likavirus
GCA_003342435.1	39014	52	Aeromonas phage AsXd1	NA	Viruses; Duplodnaviria; Heunggongvirae; Uroviricota; Caudoviricetes; Caudovirales; Siphoviridae; unclassified Siphoviridae
GCA_013387615.1	53387	73	Salmonella phage vB_SalMLPSEYT	NA	Viruses; Duplodnaviria; Heunggongvirae; Uroviricota; Caudoviricetes; Caudovirales; Myoviridae; Rosemountvirus; unclassified Rosemountvirus
GCA_003183625.1	97757	155	Microbacterium phage Hendrix	NA	Viruses; Duplodnaviria; Heunggongvirae; Uroviricota; Caudoviricetes; Caudovirales; Siphoviridae; Rogerhendrixvirus; Microbacterium virus Hendrix
GCA_003183705.1	57243	71	Streptomyces phage BayC	NA	Viruses; Duplodnaviria; Heunggongvirae; Uroviricota; Caudoviricetes; Caudovirales; Siphoviridae; Woodruffvirus; unclassified Woodruffvirus
GCA_003183725.1	57243	71	Streptomyces phage Salete	NA	Viruses; Duplodnaviria; Heunggongvirae; Uroviricota; Caudoviricetes; Caudovirales; Siphoviridae; Woodruffvirus; unclassified Woodruffvirus
GCA_003183765.1	101665	134	Escherichia phage Gostya9	NA	Viruses; Duplodnaviria; Heunggongvirae; Uroviricota; Caudoviricetes; Caudovirales; Demerecviridae; Markadamsvirinae; Tequintavirus; Escherichia virus Gostya9

gca	genome.size	proteome.size	phage.name	strain	Taxonomy
GCA_003183745.1	54065	78	Erwinia phage Faunus	NA	Viruses; Duplodnaviria; Heunggongvirae; Uroviricota; Caudoviricetes; Caudovirales; Chaseviridae; Faunusvirus; Erwinia virus Faunus
GCA_003183065.1	52304	89	Gordonia phage Petra	NA	Viruses; Duplodnaviria; Heunggongvirae; Uroviricota; Caudoviricetes; Caudovirales; Siphoviridae; unclassified Siphoviridae
GCA_003258215.1	59641	84	Gordonia phage Sitar	NA	Viruses; Duplodnaviria; Heunggongvirae; Uroviricota; Caudoviricetes; Caudovirales; Siphoviridae; Vividuovirus; unclassified Vividuovirus
GCA_003183085.1	61670	79	Gordonia phage Sour	NA	Viruses; Duplodnaviria; Heunggongvirae; Uroviricota; Caudoviricetes; Caudovirales; Siphoviridae; Sourvirus; Gordonia virus Sour
GCA_003182885.1	38684	65	Microbacterium phage Appa	NA	Viruses; Duplodnaviria; Heunggongvirae; Uroviricota; Caudoviricetes; Caudovirales; Siphoviridae; Appavirus; Microbacterium virus Appa
GCA_003182905.1	53097	81	Microbacterium phage Camille	NA	Viruses; Duplodnaviria; Heunggongvirae; Uroviricota; Caudoviricetes; Caudovirales; Siphoviridae; unclassified Siphoviridae
GCA_003182925.1	78922	131	Microbacterium phage Count	NA	Viruses; Duplodnaviria; Heunggongvirae; Uroviricota; Caudoviricetes; Caudovirales; Siphoviridae; unclassified Siphoviridae
GCA_003182945.1	41803	63	Microbacterium phage Gargoyle	NA	Viruses; Duplodnaviria; Heunggongvirae; Uroviricota; Caudoviricetes; Caudovirales; Siphoviridae; Ilzatvirus; unclassified Ilzatvirus
GCA_003182965.1	61769	104	Microbacterium phage Hyperion	NA	Viruses; Duplodnaviria; Heunggongvirae; Uroviricota; Caudoviricetes; Caudovirales; Siphoviridae; Squashvirus; Microbacterium virus Hyperion
GCA_003183005.1	41812	63	Microbacterium phage Martin	NA	Viruses; Duplodnaviria; Heunggongvirae; Uroviricota; Caudoviricetes; Caudovirales; Siphoviridae; Ilzatvirus; unclassified Ilzatvirus
GCA_003183025.1	41555	62	Microbacterium phage Oats	NA	Viruses; Duplodnaviria; Heunggongvirae; Uroviricota; Caudoviricetes; Caudovirales; Siphoviridae; Ilzatvirus; unclassified Ilzatvirus
GCA_003183145.1	62312	108	Microbacterium phage Squash	NA	Viruses; Duplodnaviria; Heunggongvirae; Uroviricota; Caudoviricetes; Caudovirales; Siphoviridae; Squashvirus; Microbacterium virus Squash
GCA_003183105.1	41793	63	Microbacterium phage Teagan	NA	Viruses; Duplodnaviria; Heunggongvirae; Uroviricota; Caudoviricetes; Caudovirales; Siphoviridae; Ilzatvirus; unclassified Ilzatvirus
GCA_003183125.1	131801	275	Rhodococcus phage Grayson	NA	Viruses; Duplodnaviria; Heunggongvirae; Uroviricota; Caudoviricetes; Caudovirales; Siphoviridae; Weaselvirus; unclassified Weaselvirus
GCA_003182985.1	53912	93	Rhodococcus phage Jace	NA	Viruses; Duplodnaviria; Heunggongvirae; Uroviricota; Caudoviricetes; Caudovirales; Siphoviridae; unclassified Siphoviridae
GCA_003183045.1	133006	273	Rhodococcus phage Peregrin	NA	Viruses; Duplodnaviria; Heunggongvirae; Uroviricota; Caudoviricetes; Caudovirales; Siphoviridae; Weaselvirus; unclassified Weaselvirus
GCA_003183165.1	50884	89	Mycobacterium phage Beauxregard13	NA	Viruses; Duplodnaviria; Heunggongvirae; Uroviricota; Caudoviricetes; Caudovirales; Siphoviridae; Fromanvirus; unclassified Fromanvirus
GCA_003183185.1	59815	108	Mycobacterium phage BobaPhett	NA	Viruses; Duplodnaviria; Heunggongvirae; Uroviricota; Caudoviricetes; Caudovirales; Siphoviridae; Cheoctovirus; unclassified Cheoctovirus
GCA_003183205.1	54685	101	Mycobacterium phage Byougenkin	NA	Viruses; Duplodnaviria; Heunggongvirae; Uroviricota; Caudoviricetes; Caudovirales; Siphoviridae; Cheoctovirus; unclassified Cheoctovirus
GCA_003183225.1	46717	77	Gordonia phage Easley	NA	Viruses; Duplodnaviria; Heunggongvirae; Uroviricota; Caudoviricetes; Caudovirales; Siphoviridae; unclassified Siphoviridae
GCA_003183265.1	43699	57	Streptomyces phage HotFries	NA	Viruses; Duplodnaviria; Heunggongvirae; Uroviricota; Caudoviricetes; Caudovirales; Siphoviridae; Scapunavirus; unclassified Scapunavirus
GCA_003183285.1	56362	105	Streptomyces phage Ibantik	NA	Viruses; Duplodnaviria; Heunggongvirae; Uroviricota; Caudoviricetes; Caudovirales; Siphoviridae; unclassified Siphoviridae
GCA_003183305.1	58074	105	Mycobacterium phage Mattes	NA	Viruses; Duplodnaviria; Heunggongvirae; Uroviricota; Caudoviricetes; Caudovirales; Siphoviridae; Cheoctovirus; unclassified Cheoctovirus
GCA_003183325.1	43545	58	Streptomyces phage Moozy	NA	Viruses; Duplodnaviria; Heunggongvirae; Uroviricota; Caudoviricetes; Caudovirales; Siphoviridae; Scapunavirus; unclassified Scapunavirus
GCA_003183345.1	52935	91	Microbacterium phage Paschalis	NA	Viruses; Duplodnaviria; Heunggongvirae; Uroviricota; Caudoviricetes; Caudovirales; Siphoviridae; Quhwahvirus
GCA_003183365.1	68721	102	Mycobacterium phage PhrankReynolds	NA	Viruses; Duplodnaviria; Heunggongvirae; Uroviricota; Caudoviricetes; Caudovirales; Siphoviridae; Bclasvirinae; Pegunavirus; unclassified Pegunavirus
GCA_003183385.1	51376	85	Mycobacterium phage Pipcraft	NA	Viruses; Duplodnaviria; Heunggongvirae; Uroviricota; Caudoviricetes; Caudovirales; Siphoviridae; Fromanvirus; unclassified Fromanvirus
GCA_003183425.1	51633	98	Mycobacterium phage Priamo	NA	Viruses; Duplodnaviria; Heunggongvirae; Uroviricota; Caudoviricetes; Caudovirales; Siphoviridae; Fromanvirus; unclassified Fromanvirus
GCA_003183445.1	57623	91	Streptomyces phage Rainydai	NA	Viruses; Duplodnaviria; Heunggongvirae; Uroviricota; Caudoviricetes; Caudovirales; Siphoviridae; Bingvirus; unclassified Bingvirus
GCA_003183405.1	43709	59	Streptomyces phage RavenPuff	NA	Viruses; Duplodnaviria; Heunggongvirae; Uroviricota; Caudoviricetes; Caudovirales; Siphoviridae; Scapunavirus; unclassified Scapunavirus
GCA_003183465.1	51368	87	Mycobacterium phage Relief	NA	Viruses; Duplodnaviria; Heunggongvirae; Uroviricota; Caudoviricetes; Caudovirales; Siphoviridae; Fromanvirus; unclassified Fromanvirus
GCA_003183485.1	55993	86	Streptomyces phage SendItCS	NA	Viruses; Duplodnaviria; Heunggongvirae; Uroviricota; Caudoviricetes; Caudovirales; Siphoviridae; Bingvirus; unclassified Bingvirus
GCA_003183245.1	46133	66	Streptomyces phage FlowerPower	NA	Viruses; Duplodnaviria; Heunggongvirae; Uroviricota; Caudoviricetes; Caudovirales; Podoviridae; unclassified Podoviridae
GCA_018583465.1	5322	8	Mastomys natalensis polyomavirus 2	NA	Viruses; Monodnaviria; Shotokuvirae; Cossaviricota; Papovaviricetes; Sepolyvirales; Polyomaviridae; Alphapolyomavirus

gca	genome.size	proteome.size	phage.name	strain	Taxonomy
GCA_013088045.1	2749	6	Ageratum leaf curl Sichuan virus	NA	Viruses; Monodnaviria; Shotokuvirae; Cressdnaviricota; Repensiviricetes; Geplafuvirales; Geminiviridae; Begomovirus
GCA_013086975.1	10864	2	Viola virus	BRMT__PanAr2015	Viruses; Riboviria; Orthornavirae; Negarnaviricota; Polyploviricotina; Ellioviricetes; Bunyavirales; Phenuiviridae; Phlebovirus; Viola phlebovirus
GCA_000842025.1	7591	3	Kalanchoe topspotting virus	NA	Viruses; Retro-transcribing viruses; Caulimoviridae; Badnavirus
GCA_003814495.1	39878	54	Streptococcus phage VS2018a	NA	Viruses; Duplodnaviria; Heunggongvirae; Uroviricota; Caudoviricetes; Caudovirales; Siphoviridae; unclassified Siphoviridae
GCA_004768945.1	110964	163	Escherichia phage SP15	NA	Viruses; Duplodnaviria; Heunggongvirae; Uroviricota; Caudoviricetes; Caudovirales; Demerecviridae; Markadamsvirinae; Tequintavirus; unclassified Tequintavirus
GCA_013088055.1	7510	1	Apodemus agrarius picornavirus	LongwanRn37	Viruses; Riboviria; Orthornavirae; Pisuviricota; Pisoniviricetes; Picornavirales; unclassified Picornavirales
GCA_018582965.1	7407	1	Suncus murinus picornavirus	WenchengSm294	Viruses; Riboviria; Orthornavirae; Pisuviricota; Pisoniviricetes; Picornavirales; unclassified Picornavirales
GCA_004131165.1	14663	1	Helianthus annuus alphaendornavirus	NA	Viruses; Riboviria; Orthornavirae; Kitrinoviricota; Alsuviricetes; Martellivirales; Endornaviridae; Alphaendornavirus
GCA_003177195.1	168869	269	Escherichia phage vB__EcoM__NBG1	NA	Viruses; Duplodnaviria; Heunggongvirae; Uroviricota; Caudoviricetes; Caudovirales; Myoviridae; Tevenvirinae; Mosigvirus; unclassified Mosigvirus
GCA_003177215.1	166083	261	Escherichia phage vB__EcoM__NBG2	NA	Viruses; Duplodnaviria; Heunggongvirae; Uroviricota; Caudoviricetes; Caudovirales; Myoviridae; Tevenvirinae; Tequatrovirus; unclassified Tequatrovirus
GCA_004132425.1	7285	5	Blackberry virus A	NA	Viruses; Riboviria; Orthornavirae; Kitrinoviricota; Alsuviricetes; Tymovirales; Betaflexiviridae; Trivirinae; Vitivirus
GCA_004131185.1	2730	1	Chenopodium quinoa mitovirus 1	NA	Viruses; Riboviria; Orthornavirae; Lenarviricota; Howeltoviricetes; Cryppavirales; Mitoviridae; Mitovirus; unclassified Mitovirus
GCA_004134005.1	7188	3	Rana hepevirus	agile forgRD62015HUN	Viruses; Riboviria; Orthornavirae; Kitrinoviricota; Alsuviricetes; Hepelivirales; Hepeviridae; unclassified Hepeviridae
GCA_000854505.1	5784	7	Oyster mushroom spherical virus	NA	Viruses; ssRNA viruses; ssRNA positive-strand viruses, no DNA stage; unclassified ssRNA positive-strand viruses
GCA_003052185.1	1336	1	Tobacco leaf curl Yunnan virus satellite DNA beta	NA	Viruses; Satellites; Satellite Nucleic Acids; Single stranded DNA satellites; Betasatellites; unclassified Begomovirus-associated DNA beta-like sequences
GCA_000851165.1	11375	1	Kamiti River virus	NA	Viruses; ssRNA viruses; ssRNA positive-strand viruses, no DNA stage; Flaviviridae; Flavivirus; unclassified Flavivirus
GCA_000916675.1	4086	4	Turnip rosette virus	NA	Viruses; ssRNA viruses; ssRNA positive-strand viruses, no DNA stage; Sobemovirus
GCA_000926415.1	11097	5	Pike fry sprivivirus	NA	Viruses; Riboviria; Orthornavirae; Negarnaviricota; Haploviricotina; Monjiviricetes; Mononegavirales; Rhabdoviridae; Sprivivirus
GCA_000845605.1	1354	1	Tobacco curly shoot betasatellite	NA	Viruses; Tolecusatellitidae; Betasatellite
GCA_000844805.1	4694	2	Avian adenoassociated virus ATCC VR865	NA	Viruses; ssDNA viruses; Parvoviridae; Parvovirinae; Dependoparvovirus; unclassified Dependovirus
GCA_000842905.1	2782	6	Tomato yellow leaf curl Malaga virus	NA	Viruses; ssDNA viruses; Geminiviridae; Begomovirus
GCA_004131245.2	6453	4	Opuntia virus 2	NA	Viruses; Riboviria; Orthornavirae; Kitrinoviricota; Alsuviricetes; Martellivirales; Virgaviridae; Tobamovirus; unclassified Tobamovirus
GCA_004319625.1	39695	49	Cronobacter phage GW1	NA	Viruses; Duplodnaviria; Heunggongvirae; Uroviricota; Caudoviricetes; Caudovirales; Autographiviridae; Studiervirinae; Kayfunavirus; Cronobacter virus GW1
GCA_006452035.1	221923	220	Akhmeta virus	NA	Viruses; Varidnaviria; Bamfordvirae; Nucleocytoviricota; Pokkesviricetes; Chitovirales; Poxviridae; Chordopoxvirinae; Orthopoxvirus
GCA_004132245.1	8536	4	Lettuce chordovirus 1	NA	Viruses; Riboviria; Orthornavirae; Kitrinoviricota; Alsuviricetes; Tymovirales; Betaflexiviridae; unclassified Betaflexiviridae
GCA_004133325.1	15763	9	Arracacha virus 1	NA	Viruses; Riboviria; Orthornavirae; Kitrinoviricota; Alsuviricetes; Martellivirales; Closteroviridae; Closterovirus
GCA_003308795.1	6372	11	Xanthomonas phage phiLf2	Xanthomonas campestris pv campestris str 8004	Viruses; Monodnaviria; Loebvirae; Hofneiviricota; Faserviricetes; Tubulavirales; Inoviridae; unclassified Inoviridae
GCA_003308415.1	55137	82	Streptomyces phage Henoccus	NA	Viruses; Duplodnaviria; Heunggongvirae; Uroviricota; Caudoviricetes; Caudovirales; Siphoviridae; unclassified Siphoviridae
GCA_003308435.1	54912	82	Streptomyces phage JackieB	NA	Viruses; Duplodnaviria; Heunggongvirae; Uroviricota; Caudoviricetes; Caudovirales; Siphoviridae; unclassified Siphoviridae
GCA_003308475.1	54798	81	Streptomyces phage LazerLemon	NA	Viruses; Duplodnaviria; Heunggongvirae; Uroviricota; Caudoviricetes; Caudovirales; Siphoviridae; unclassified Siphoviridae
GCA_003308455.1	54495	81	Streptomyces phage UNTPL	NA	Viruses; Duplodnaviria; Heunggongvirae; Uroviricota; Caudoviricetes; Caudovirales; Siphoviridae; unclassified Siphoviridae
GCA_003991605.1	59646	67	Aeromonas phage SD04	NA	Viruses; Duplodnaviria; Heunggongvirae; Uroviricota; Caudoviricetes; Caudovirales; Siphoviridae; Seuratvirus; unclassified Seuratvirus
GCA_003258195.1	39866	57	Oenococcus phage phiOE33PA	NA	Viruses; Duplodnaviria; Heunggongvirae; Uroviricota; Caudoviricetes; Caudovirales; Siphoviridae; unclassified Siphoviridae
GCA_013086985.1	10529	3	Lijiang virus	NA	Viruses; Riboviria; Orthornavirae; Negarnaviricota; Polyploviricotina; Ellioviricetes; Bunyavirales; Arenaviridae; Mammarenavirus; unclassified Mammarenavirus

gca	genome.size	proteome.size	phage.name	strain	Taxonomy
GCA_003308535.1	150925	267	Klebsiella phage ZCKP1	NA	Viruses; Duplodnaviria; Heunggongvirae; Uroviricota; Caudoviricetes; Caudovirales; Myoviridae; Phapecoctavirus; Klebsiella virus ZCKP1
GCA_003368625.1	44526	24	Ralstonia phage phiRSP	NA	Viruses; Duplodnaviria; Heunggongvirae; Uroviricota; Caudoviricetes; Caudovirales; Myoviridae; unclassified Myoviridae
GCA_003308495.1	50129	78	Streptomyces phage Yosif	NA	Viruses; Duplodnaviria; Heunggongvirae; Uroviricota; Caudoviricetes; Caudovirales; Siphoviridae; Arquatrovirinae; Camvirus; unclassified Camvirus
GCA_003308555.1	266532	345	Erwinia phage vB_EamM_Alexandra	NA	Viruses; Duplodnaviria; Heunggongvirae; Uroviricota; Caudoviricetes; Caudovirales; Myoviridae; Alexandravirus; Erwinia virus Alexandra
GCA_003308135.2	40526	54	Corynebacterium phage TouchMeNot	NA	Viruses; Duplodnaviria; Heunggongvirae; Uroviricota; Caudoviricetes; Caudovirales; Siphoviridae; Poushouvirus; unclassified Poushouvirus
GCA_003307695.1	46653	78	Gordonia phage Ebert	NA	Viruses; Duplodnaviria; Heunggongvirae; Uroviricota; Caudoviricetes; Caudovirales; Siphoviridae; Attisvirus; unclassified Attisvirus
GCA_003307715.1	16604	24	Gordonia phage Emperor	NA	Viruses; Duplodnaviria; Heunggongvirae; Uroviricota; Caudoviricetes; Caudovirales; Siphoviridae; unclassified Siphoviridae
GCA_003307835.1	46950	75	Gordonia phage Margaret	NA	Viruses; Duplodnaviria; Heunggongvirae; Uroviricota; Caudoviricetes; Caudovirales; Siphoviridae; unclassified Siphoviridae
GCA_003307895.1	44924	66	Gordonia phage Morrissey	NA	Viruses; Duplodnaviria; Heunggongvirae; Uroviricota; Caudoviricetes; Caudovirales; Siphoviridae; Gustavvirus; unclassified Gustavvirus
GCA_003308055.1	67118	96	Gordonia phage Suzy	NA	Viruses; Duplodnaviria; Heunggongvirae; Uroviricota; Caudoviricetes; Caudovirales; Siphoviridae; Terapinvirus; Gordonia virus Suzy
GCA_003308155.1	43758	66	Gordonia phage Trine	NA	Viruses; Duplodnaviria; Heunggongvirae; Uroviricota; Caudoviricetes; Caudovirales; Siphoviridae; Trinevirus; Gordonia virus Trine
GCA_003307635.1	56707	83	Microbacterium phage AnnaSerena	NA	Viruses; Duplodnaviria; Heunggongvirae; Uroviricota; Caudoviricetes; Caudovirales; Siphoviridae; Krampusvirus; unclassified Krampusvirus
GCA_003307755.1	48500	80	Microbacterium phage Floof	NA	Viruses; Duplodnaviria; Heunggongvirae; Uroviricota; Caudoviricetes; Caudovirales; Siphoviridae; unclassified Siphoviridae
GCA_003307795.1	63119	114	Microbacterium phage Hortus1	NA	Viruses; Duplodnaviria; Heunggongvirae; Uroviricota; Caudoviricetes; Caudovirales; Siphoviridae; unclassified Siphoviridae
GCA_003307815.1	56708	83	Microbacterium phage Krampus	NA	Viruses; Duplodnaviria; Heunggongvirae; Uroviricota; Caudoviricetes; Caudovirales; Siphoviridae; Krampusvirus; Microbacterium virus Krampus
GCA_003307855.1	55572	96	Microbacterium phage MementoMori	NA	Viruses; Duplodnaviria; Heunggongvirae; Uroviricota; Caudoviricetes; Caudovirales; Siphoviridae; Mementomorivirus; Microbacterium virus MementoMori
GCA_003307875.1	54148	93	Microbacterium phage Metamorphoo	NA	Viruses; Duplodnaviria; Heunggongvirae; Uroviricota; Caudoviricetes; Caudovirales; Siphoviridae; Metamorphoovirus; Microbacterium virus Metamorphoo
GCA_003307935.1	63123	114	Microbacterium phage OlinDD	NA	Viruses; Duplodnaviria; Heunggongvirae; Uroviricota; Caudoviricetes; Caudovirales; Siphoviridae; unclassified Siphoviridae
GCA_003307955.1	47364	74	Microbacterium phage Percival	NA	Viruses; Duplodnaviria; Heunggongvirae; Uroviricota; Caudoviricetes; Caudovirales; Siphoviridae; unclassified Siphoviridae
GCA_003307995.1	62954	114	Microbacterium phage Pioneer3	NA	Viruses; Duplodnaviria; Heunggongvirae; Uroviricota; Caudoviricetes; Caudovirales; Siphoviridae; unclassified Siphoviridae
GCA_003308215.1	53549	95	Microbacterium phage Quhwah	NA	Viruses; Duplodnaviria; Heunggongvirae; Uroviricota; Caudoviricetes; Caudovirales; Siphoviridae; Quhwahvirus
GCA_003308035.1	54189	99	Microbacterium phage RobsFeet	NA	Viruses; Duplodnaviria; Heunggongvirae; Uroviricota; Caudoviricetes; Caudovirales; Siphoviridae; Metamorphoovirus; Microbacterium virus RobsFeet
GCA_003308115.1	63128	114	Microbacterium phage Tandem	NA	Viruses; Duplodnaviria; Heunggongvirae; Uroviricota; Caudoviricetes; Caudovirales; Siphoviridae; unclassified Siphoviridae
GCA_003308195.1	47921	75	Microbacterium phage Zeta1847	NA	Viruses; Duplodnaviria; Heunggongvirae; Uroviricota; Caudoviricetes; Caudovirales; Siphoviridae; Zetavirus; Microbacterium virus Zeta1847
GCA_003308175.1	46539	66	Rhodococcus phage UhSalsa	NA	Viruses; Duplodnaviria; Heunggongvirae; Uroviricota; Caudoviricetes; Caudovirales; Siphoviridae; Rerduovirus; unclassified Rerduovirus
GCA_003307615.1	46493	66	Rhodococcus phage Alpacados	NA	Viruses; Duplodnaviria; Heunggongvirae; Uroviricota; Caudoviricetes; Caudovirales; Siphoviridae; Rerduovirus; unclassified Rerduovirus
GCA_003307655.1	46606	65	Rhodococcus phage Bradshaw	NA	Viruses; Duplodnaviria; Heunggongvirae; Uroviricota; Caudoviricetes; Caudovirales; Siphoviridae; Rerduovirus; unclassified Rerduovirus
GCA_003307675.1	46347	65	Rhodococcus phage Bryce	NA	Viruses; Duplodnaviria; Heunggongvirae; Uroviricota; Caudoviricetes; Caudovirales; Siphoviridae; Rerduovirus; unclassified Rerduovirus
GCA_003307735.1	46429	66	Rhodococcus phage Erik	NA	Viruses; Duplodnaviria; Heunggongvirae; Uroviricota; Caudoviricetes; Caudovirales; Siphoviridae; Rerduovirus; unclassified Rerduovirus
GCA_003307775.1	46535	66	Rhodococcus phage Gollum	NA	Viruses; Duplodnaviria; Heunggongvirae; Uroviricota; Caudoviricetes; Caudovirales; Siphoviridae; Rerduovirus; unclassified Rerduovirus
GCA_003307915.1	45936	65	Rhodococcus phage Nancinator	NA	Viruses; Duplodnaviria; Heunggongvirae; Uroviricota; Caudoviricetes; Caudovirales; Siphoviridae; Rerduovirus; unclassified Rerduovirus
GCA_003307975.1	46540	66	Rhodococcus phage Phrankenstein	NA	Viruses; Duplodnaviria; Heunggongvirae; Uroviricota; Caudoviricetes; Caudovirales; Siphoviridae; Rerduovirus; unclassified Rerduovirus
GCA_003308015.1	46568	66	Rhodococcus phage Rasputin	NA	Viruses; Duplodnaviria; Heunggongvirae; Uroviricota; Caudoviricetes; Caudovirales; Siphoviridae; Rerduovirus; unclassified Rerduovirus
GCA_003308075.1	46596	66	Rhodococcus phage Swann	NA	Viruses; Duplodnaviria; Heunggongvirae; Uroviricota; Caudoviricetes; Caudovirales; Siphoviridae; Rerduovirus; unclassified Rerduovirus

gca	genome.size	proteome.size	phage.name	strain	Taxonomy
GCA_003308095.1	46807	67	Rhodococcus phage Takoda	NA	Viruses; Duplodnaviria; Heunggongvirae; Uroviricota; Caudoviricetes; Caudovirales; Siphoviridae; Rerduovirus; unclassified Rerduovirus
GCA_003308855.1	40914	45	Pseudomonas phage PFP1	NA	Viruses; Duplodnaviria; Heunggongvirae; Uroviricota; Caudoviricetes; Caudovirales; Autographiviridae; Studiervirinae; Pifdecavirus; Pseudomonas virus PFP1
GCA_003308815.1	6564	12	Xanthomonas phage phiXv2	NA	Viruses; Monodnaviria; Loebvirae; Hofneiviricota; Faserviricetes; Tubulavirales; Inoviridae; unclassified Inoviridae
GCA_003307535.1	56048	81	Pasteurella phage Pm86	NA	Viruses; Duplodnaviria; Heunggongvirae; Uroviricota; Caudoviricetes; Caudovirales; Siphoviridae; unclassified Siphoviridae
GCA_003307515.1	33296	44	Pasteurella phage AFS2018a	NA	Viruses; Duplodnaviria; Heunggongvirae; Uroviricota; Caudoviricetes; Caudovirales; Myoviridae; Bcepmuvirus; unclassified Bcepmuvirus
GCA_003369245.1	143204	177	Acinetobacter phage SHAb 15599	NA	Viruses; Duplodnaviria; Heunggongvirae; Uroviricota; Caudoviricetes; Caudovirales; Ackermannviridae; unclassified Ackermannviridae
GCA_003029595.1	2739	6	Malachra yellow mosaic virus	NA	Viruses; Monodnaviria; Shotokuvirae; Cressdnaviricota; Repensiviricetes; Geplafuvirales; Geminiviridae; Begomovirus; unclassified Begomovirus
GCA_004132385.1	8684	1	Hepacivirus P	NA	Viruses; Riboviria; Orthornavirae; Kitrinoviricota; Flasuviricetes; Amarillovirales; Flaviviridae; Hepacivirus; unclassified Hepacivirus
GCA_003183645.1	38601	66	Listeria phage PSUVKHLP019	NA	Viruses; Duplodnaviria; Heunggongvirae; Uroviricota; Caudoviricetes; Caudovirales; Siphoviridae; unclassified Siphoviridae
GCA_003183665.1	39585	67	Listeria phage PSUVKHLP040	NA	Viruses; Duplodnaviria; Heunggongvirae; Uroviricota; Caudoviricetes; Caudovirales; Siphoviridae; unclassified Siphoviridae
GCA_003183685.1	48286	81	Listeria phage PSUVKHLP041	NA	Viruses; Duplodnaviria; Heunggongvirae; Uroviricota; Caudoviricetes; Caudovirales; Myoviridae; unclassified Myoviridae
GCA_003651565.2	6444	6	Microvirus sp	NA	Viruses; Monodnaviria; Sangervirae; Phixviricota; Malgrandaviricetes; Petitvirales; Microviridae; unclassified Microviridae
GCA_003623715.1	6249	9	CRESS virus sp	NA	Viruses; Monodnaviria; Shotokuvirae; Cressdnaviricota; unclassified Cressdnaviricota; CRESS viruses
GCA_003657725.1	419387	545	Myoviridae sp	NA	Viruses; Duplodnaviria; Heunggongvirae; Uroviricota; Caudoviricetes; Caudovirales; Myoviridae; unclassified Myoviridae
GCA_003846465.1	2177	4	Genomoviridae sp	NA	Viruses; Monodnaviria; Shotokuvirae; Cressdnaviricota; Repensiviricetes; Geplafuvirales; Genomoviridae; unclassified Genomoviridae
GCA_003659065.1	1747	4	Geminiviridae sp	NA	Viruses; Monodnaviria; Shotokuvirae; Cressdnaviricota; Repensiviricetes; Geplafuvirales; Geminiviridae; unclassified Geminiviridae
GCA_018448345.1	38434	47	Podoviridae sp	NA	Viruses; Duplodnaviria; Heunggongvirae; Uroviricota; Caudoviricetes; Caudovirales; Podoviridae; unclassified Podoviridae
GCA_018448575.1	189560	321	Caudovirales sp	NA	Viruses; Duplodnaviria; Heunggongvirae; Uroviricota; Caudoviricetes; Caudovirales; unclassified Caudovirales
GCA_003381765.1	5349	9	Microviridae sp	NA	Viruses; Monodnaviria; Sangervirae; Phixviricota; Malgrandaviricetes; Petitvirales; Microviridae; unclassified Microviridae
GCA_003657365.1	5058	6	Bacilladnaviridae sp	NA	Viruses; Monodnaviria; Shotokuvirae; Cressdnaviricota; Arfiviricetes; Baphyvirales; Bacilladnaviridae; unclassified Bacilladnaviridae
GCA_003659325.1	4388	8	Virus sp	NA	Viruses; unclassified viruses
GCA_004197235.1	4177	9	Circular genetic element sp	NA	Viruses; unclassified viruses
GCA_013150885.1	65554	90	Pseudomonas phage PaGU11	NA	Viruses; Duplodnaviria; Heunggongvirae; Uroviricota; Caudoviricetes; Caudovirales; Myoviridae; Pbunavirus; unclassified Pbunavirus
GCA_004131285.1	1361	1	Tomato leaf curl Yunnan betasatellite	ChinaYunnan 2Tomato2015	Viruses; Tolecusatellitidae; Betasatellite; unclassified Betasatellite
GCA_000839265.1	2749	6	Tomato leaf curl Bangalore virus	Indian tomato leaf curl virus ITmLCV	Viruses; ssDNA viruses; Geminiviridae; Begomovirus
GCA_000843285.1	5335	6	Blattella germanica densovirus 1	NA	Viruses; ssDNA viruses; Parvoviridae; Densovirinae; Ambidensovirus; Blattodean ambidensovirus 2
GCA_000848965.1	1424	1	Tomato leaf curl betasatellite	NA	Viruses; Tolecusatellitidae; Betasatellite
GCA_000844325.1	1354	1	Zinnia leaf curl virusassociated DNA beta	NA	Viruses; Satellites; Satellite Nucleic Acids; Single stranded DNA satellites; Betasatellites; unclassified Begomovirus-associated DNA beta-like sequences
GCA_000840185.1	2759	6	Tomato leaf curl Karnataka virus	Bangalore II India	Viruses; ssDNA viruses; Geminiviridae; Begomovirus
GCA_002824025.1	2759	6	Tomato leaf curl Bangalore virusBan5	NA	Viruses; Monodnaviria; Shotokuvirae; Cressdnaviricota; Repensiviricetes; Geplafuvirales; Geminiviridae; Begomovirus; Tomato leaf curl Bangalore virus
GCA_002824045.1	2759	6	Tomato leaf curl Bangalore virusBan4	LCVBan4	Viruses; Monodnaviria; Shotokuvirae; Cressdnaviricota; Repensiviricetes; Geplafuvirales; Geminiviridae; Begomovirus; Tomato leaf curl Bangalore virus
GCA_000858205.1	2781	6	Tomato yellow leaf curl virusAlmeria	NA	Viruses; Monodnaviria; Shotokuvirae; Cressdnaviricota; Repensiviricetes; Geplafuvirales; Geminiviridae; Begomovirus; Tomato yellow leaf curl virus
GCA_002987155.1	2771	6	Tomato yellow leaf curl virusIran	NA	Viruses; Monodnaviria; Shotokuvirae; Cressdnaviricota; Repensiviricetes; Geplafuvirales; Geminiviridae; Begomovirus; Tomato yellow leaf curl virus
GCA_002987165.1	2790	6	Tomato yellow leaf curl virus Mild	NA	Viruses; Monodnaviria; Shotokuvirae; Cressdnaviricota; Repensiviricetes; Geplafuvirales; Geminiviridae; Begomovirus; Tomato yellow leaf curl virus

gca	genome.size	proteome.size	phage.name	strain	Taxonomy
GCA_000846745.1	41391	63	Enterobacteria phage ST104	NA	Viruses; dsDNA viruses, no RNA stage; Caudovirales; Podoviridae; P22virus; unclassified P22likevirus
GCA_000861305.1	1348	1	Malvastrum yellow vein betasatellite	NA	Viruses; Tolecusatellitidae; Betasatellite
GCA_000848105.1	1344	1	Honeysuckle yellow vein betasatellite	NA	Viruses; Tolecusatellitidae; Betasatellite
GCA_000846485.1	1356	1	Eupatorium yellow vein betasatellite	NA	Viruses; Tolecusatellitidae; Betasatellite
GCA_013088065.1	8213	1	Alpinia oxyphylla mosaic virus	NA	Viruses; Riboviria; Orthornavirae; Pisuviricota; Stelpaviricetes; Patatavirales; Potyviridae; Macluravirus
GCA_003456955.1	94878	81	CrAssphage sp	NA	Viruses; Duplodnaviria; Heunggongvirae; Uroviricota; Caudoviricetes; Caudovirales; Podoviridae; unclassified Podoviridae; crAss-like viruses
GCA_000863645.1	9611	1	Pennisetum mosaic virus	NA	Viruses; ssRNA viruses; ssRNA positive-strand viruses, no DNA stage; Potyviridae; Potyvirus
GCA_004117275.1	16396	5	Alstroemeria yellow spot virus	NA	Viruses; Riboviria; Orthornavirae; Negarnaviricota; Polyploviricotina; Ellioviricetes; Bunyavirales; Tospoviridae; Orthotospovirus; Alstroemeria yellow spot orthotospovirus
GCA_018580905.1	2691	2	Chlorocebus cynosuros associated smacovirus	ZM0986	Viruses; unclassified viruses; unclassified DNA viruses; unclassified ssDNA viruses
GCA_018580915.1	2488	2	Papio cynocephalus associated smacovirus	ZM0974	Viruses; Monodnaviria; Shotokuvirae; Cressdnaviricota; Arfiviricetes; Cremevirales; Smacoviridae; unclassified Smacoviridae
GCA_001046705.1	2741	7	Bhendi yellow vein mosaic virusMadurai	NA	Viruses; Monodnaviria; Shotokuvirae; Cressdnaviricota; Repensiviricetes; Geplafuvirales; Geminiviridae; Begomovirus; Bhendi yellow vein mosaic virus
GCA_002987135.1	2735	6	Tomato yellow leaf curl China virusTbY5	NA	Viruses; Monodnaviria; Shotokuvirae; Cressdnaviricota; Repensiviricetes; Geplafuvirales; Geminiviridae; Begomovirus; Tomato yellow leaf curl China virus
GCA_002987095.1	2737	6	Tomato yellow leaf curl China virusTbY10	NA	Viruses; Monodnaviria; Shotokuvirae; Cressdnaviricota; Repensiviricetes; Geplafuvirales; Geminiviridae; Begomovirus; Tomato yellow leaf curl China virus
GCA_002987105.1	2738	6	Tomato yellow leaf curl China virusToY25	NA	Viruses; Monodnaviria; Shotokuvirae; Cressdnaviricota; Repensiviricetes; Geplafuvirales; Geminiviridae; Begomovirus; Tomato yellow leaf curl China virus
GCA_000857225.1	5488	10	Tomato yellow leaf curl Thailand virus2	NA	Viruses; Monodnaviria; Shotokuvirae; Cressdnaviricota; Repensiviricetes; Geplafuvirales; Geminiviridae; Begomovirus; Tomato yellow leaf curl Thailand virus
GCA_003047575.1	12823	5	Simian foamy virus Pongo pygmaeus pygmaeus	SFVora	Viruses; Riboviria; Pararnavirae; Artverviricota; Revtraviricetes; Ortervirales; Retroviridae; Spumaretrovirinae; Simiispumavirus; Bornean orangutan simian foamy virus
GCA_003231575.1	143638	204	Enterococcus phage phiEF17H	NA	Viruses; Duplodnaviria; Heunggongvirae; Uroviricota; Caudoviricetes; Caudovirales; Herelleviridae; Brockvirinae; Kochikohdavirus; unclassified Kochikohdavirus
GCA_003231595.1	143046	204	Enterococcus phage phiM1EF22	NA	Viruses; Duplodnaviria; Heunggongvirae; Uroviricota; Caudoviricetes; Caudovirales; Herelleviridae; Brockvirinae; Kochikohdavirus; unclassified Kochikohdavirus
GCA_003764585.1	166848	268	Escherichia phage KIT03	NA	Viruses; Duplodnaviria; Heunggongvirae; Uroviricota; Caudoviricetes; Caudovirales; Myoviridae; Tevenvirinae; Tequatrovirus; unclassified Tequatrovirus
GCA_004133145.1	5541	3	Trematomus bernacchii polyomavirus 1	NA	Viruses; Monodnaviria; Shotokuvirae; Cossaviricota; Papovaviricetes; Sepolyvirales; Polyomaviridae
GCA_004133465.1	7456	1	Rhimavirus A	AU1	Viruses; Riboviria; Orthornavirae; Pisuviricota; Pisoniviricetes; Picornavirales; Picornaviridae; unclassified Picornaviridae
GCA_003308635.1	4248	4	Ruegeria phage vB_RpoMiV15	NA	Viruses; Monodnaviria; Sangervirae; Phixviricota; Malgrandaviricetes; Petitvirales; Microviridae; unclassified Microviridae
GCA_003308615.1	74675	85	Ruegeria phage vB_RpoPV12	NA	Viruses; Duplodnaviria; Heunggongvirae; Uroviricota; Caudoviricetes; Caudovirales; Podoviridae; Baltimorevirus; unclassified Baltimorevirus
GCA_003308735.1	74830	79	Ruegeria phage vB_RpoPV13	NA	Viruses; Duplodnaviria; Heunggongvirae; Uroviricota; Caudoviricetes; Caudovirales; Podoviridae; Baltimorevirus; unclassified Baltimorevirus
GCA_003369325.1	74792	87	Ruegeria phage vB_RpoPV14	NA	Viruses; Duplodnaviria; Heunggongvirae; Uroviricota; Caudoviricetes; Caudovirales; Podoviridae; Baltimorevirus; unclassified Baltimorevirus
GCA_003308775.1	74665	82	Ruegeria phage vB_RpoPV17	NA	Viruses; Duplodnaviria; Heunggongvirae; Uroviricota; Caudoviricetes; Caudovirales; Podoviridae; Baltimorevirus; unclassified Baltimorevirus
GCA_003308675.1	74665	82	Ruegeria phage vB_RpoPV21	NA	Viruses; Duplodnaviria; Heunggongvirae; Uroviricota; Caudoviricetes; Caudovirales; Podoviridae; Baltimorevirus; unclassified Baltimorevirus
GCA_003308715.1	147480	235	Ruegeria phage vB_RpoSV10	NA	Viruses; Duplodnaviria; Heunggongvirae; Uroviricota; Caudoviricetes; Caudovirales; Siphoviridae; unclassified Siphoviridae
GCA_003308695.1	59549	82	Ruegeria phage vB_RpoSV11	NA	Viruses; Duplodnaviria; Heunggongvirae; Uroviricota; Caudoviricetes; Caudovirales; Siphoviridae; unclassified Siphoviridae
GCA_003308755.1	61382	83	Ruegeria phage vB_RpoSV16	NA	Viruses; Duplodnaviria; Heunggongvirae; Uroviricota; Caudoviricetes; Caudovirales; Siphoviridae; unclassified Siphoviridae
GCA_003308655.1	59111	82	Ruegeria phage vB_RpoSV18	NA	Viruses; Duplodnaviria; Heunggongvirae; Uroviricota; Caudoviricetes; Caudovirales; Siphoviridae; unclassified Siphoviridae
GCA_003308595.1	59573	84	Ruegeria phage vB_RpoSV7	NA	Viruses; Duplodnaviria; Heunggongvirae; Uroviricota; Caudoviricetes; Caudovirales; Siphoviridae; unclassified Siphoviridae
GCA_003260835.1	2195	3	Gyrovirus 10	NA	Viruses; Anelloviridae; Gyrovirus; unclassified Gyrovirus
GCA_018582655.1	2483	3	Paguma larvata torque teno virus	NA	Viruses; Anelloviridae; unclassified Anelloviridae; Torque teno virus

gca	genome.size	proteome.size	phage.name	strain	Taxonomy
GCA_004133965.1	6735	1	Sclerotinia sclerotiorum deltaflexivirus 2	NA	Viruses; Riboviria; Orthornavirae; Kitrinoviricota; Alsuviricetes; Tymovirales; Deltaflexiviridae; Deltaflexivirus; unclassified Deltaflexivirus
GCA_003651865.1	8707	18	Inoviridae sp	NA	Viruses; Monodnaviria; Loebvirae; Hofneiviricota; Faserviricetes; Tubulavirales; Inoviridae; unclassified Inoviridae
GCA_003260855.1	10252	1	Botrytis cinerea hypovirus 1	NA	Viruses; Riboviria; Orthornavirae; Pisuviricota; Duplopiviricetes; Durnavirales; Hypoviridae; unclassified Hypoviridae
GCA_003260875.1	8448	2	Botrytis cinerea fusarivirus 1	NA	Viruses; Riboviria; unclassified Riboviria; unclassified ssRNA viruses; Fusariviridae
GCA_003260895.1	3482	2	Botrytis cinerea fusarivirus 1S1	NA	Viruses; Riboviria; unclassified Riboviria; unclassified ssRNA viruses; Fusariviridae; Botrytis cinerea fusarivirus 1
GCA_003260915.1	3253	2	Botrytis cinerea fusarivirus 1S2	NA	Viruses; Riboviria; unclassified Riboviria; unclassified ssRNA viruses; Fusariviridae; Botrytis cinerea fusarivirus 1
GCA_003260935.1	3960	1	Botrytis cinerea hypovirus 1 satellitelike RNA	NA	Viruses; Satellites; RNA satellites
GCA_003847845.1	2159	2	Alces alces faeces associated genomovirus MP111	NA	Viruses; Monodnaviria; Shotokuvirae; Cressdnaviricota; Repensiviricetes; Geplafuvirales; Genomoviridae; unclassified Genomoviridae
GCA_003847825.1	2169	2	Alces alces faeces associated genomovirus MP146	NA	Viruses; Monodnaviria; Shotokuvirae; Cressdnaviricota; Repensiviricetes; Geplafuvirales; Genomoviridae; unclassified Genomoviridae
GCA_003847805.1	2162	2	Alces alces faeces associated genomovirus MP157	NA	Viruses; Monodnaviria; Shotokuvirae; Cressdnaviricota; Repensiviricetes; Geplafuvirales; Genomoviridae; unclassified Genomoviridae
GCA_003847905.1	2141	2	Alces alces faeces associated genomovirus MP43	NA	Viruses; Monodnaviria; Shotokuvirae; Cressdnaviricota; Repensiviricetes; Geplafuvirales; Genomoviridae; unclassified Genomoviridae
GCA_003847885.1	2249	2	Alces alces faeces associated genomovirus MP68	NA	Viruses; Monodnaviria; Shotokuvirae; Cressdnaviricota; Repensiviricetes; Geplafuvirales; Genomoviridae; unclassified Genomoviridae
GCA_003847865.1	2175	2	Alces alces faeces associated genomovirus MP84	NA	Viruses; Monodnaviria; Shotokuvirae; Cressdnaviricota; Repensiviricetes; Geplafuvirales; Genomoviridae; unclassified Genomoviridae
GCA_003847705.1	2254	2	Lepus americanus faeces associated genomovirus SHP11	NA	Viruses; Monodnaviria; Shotokuvirae; Cressdnaviricota; Repensiviricetes; Geplafuvirales; Genomoviridae; unclassified Genomoviridae
GCA_003847785.1	2134	2	Lepus americanus faeces associated genomovirus SHP111	NA	Viruses; Monodnaviria; Shotokuvirae; Cressdnaviricota; Repensiviricetes; Geplafuvirales; Genomoviridae; unclassified Genomoviridae
GCA_003847765.1	2134	2	Lepus americanus faeces associated genomovirus SHP216	NA	Viruses; Monodnaviria; Shotokuvirae; Cressdnaviricota; Repensiviricetes; Geplafuvirales; Genomoviridae; unclassified Genomoviridae
GCA_003847745.1	2142	2	Lepus americanus faeces associated genomovirus SHP7	NA	Viruses; Monodnaviria; Shotokuvirae; Cressdnaviricota; Repensiviricetes; Geplafuvirales; Genomoviridae; unclassified Genomoviridae
GCA_003847725.1	2104	2	Lepus americanus faeces associated genomovirus SHP9	NA	Viruses; Monodnaviria; Shotokuvirae; Cressdnaviricota; Repensiviricetes; Geplafuvirales; Genomoviridae; unclassified Genomoviridae
GCA_003848025.1	2211	2	Lynx canadensis faeces associated genomovirus CL1 128	NA	Viruses; Monodnaviria; Shotokuvirae; Cressdnaviricota; Repensiviricetes; Geplafuvirales; Genomoviridae; unclassified Genomoviridae
GCA_003848005.1	2188	2	Lynx canadensis faeces associated genomovirus CL1 148	NA	Viruses; Monodnaviria; Shotokuvirae; Cressdnaviricota; Repensiviricetes; Geplafuvirales; Genomoviridae; unclassified Genomoviridae
GCA_003848125.1	2121	2	Lynx canadensis faeces associated genomovirus CL1 46	NA	Viruses; Monodnaviria; Shotokuvirae; Cressdnaviricota; Repensiviricetes; Geplafuvirales; Genomoviridae; unclassified Genomoviridae
GCA_003848105.1	2309	2	Lynx canadensis faeces associated genomovirus CL1 48	NA	Viruses; Monodnaviria; Shotokuvirae; Cressdnaviricota; Repensiviricetes; Geplafuvirales; Genomoviridae; unclassified Genomoviridae
GCA_003848085.1	2302	2	Lynx canadensis faeces associated genomovirus CL1 58	NA	Viruses; Monodnaviria; Shotokuvirae; Cressdnaviricota; Repensiviricetes; Geplafuvirales; Genomoviridae; unclassified Genomoviridae
GCA_003848065.1	2211	2	Lynx canadensis faeces associated genomovirus CL1 71	NA	Viruses; Monodnaviria; Shotokuvirae; Cressdnaviricota; Repensiviricetes; Geplafuvirales; Genomoviridae; unclassified Genomoviridae
GCA_003848045.1	2278	2	Lynx canadensis faeces associated genomovirus CL1 74	NA	Viruses; Monodnaviria; Shotokuvirae; Cressdnaviricota; Repensiviricetes; Geplafuvirales; Genomoviridae; unclassified Genomoviridae
GCA_003847985.1	2188	2	Lynx canadensis faeces associated genomovirus CL3 128	NA	Viruses; Monodnaviria; Shotokuvirae; Cressdnaviricota; Repensiviricetes; Geplafuvirales; Genomoviridae; unclassified Genomoviridae
GCA_003847945.1	2208	2	Lynx canadensis faeces associated genomovirus CL4 128	NA	Viruses; Monodnaviria; Shotokuvirae; Cressdnaviricota; Repensiviricetes; Geplafuvirales; Genomoviridae; unclassified Genomoviridae
GCA_003847965.1	2211	2	Lynx canadensis faeces associated genomovirus CL4 71	NA	Viruses; Monodnaviria; Shotokuvirae; Cressdnaviricota; Repensiviricetes; Geplafuvirales; Genomoviridae; unclassified Genomoviridae
GCA_003847925.1	2201	2	Lynx canadensis faeces associated genomovirus CL5 48	NA	Viruses; Monodnaviria; Shotokuvirae; Cressdnaviricota; Repensiviricetes; Geplafuvirales; Genomoviridae; unclassified Genomoviridae
GCA_003848285.1	5497	6	Alces alces faeces associated microvirus MP10 5560	NA	Viruses; Monodnaviria; Sangervirae; Phixviricota; Malgrandaviricetes; Petitvirales; Microviridae; unclassified Microviridae
GCA_003848265.1	5398	6	Alces alces faeces associated microvirus MP11 5517	NA	Viruses; Monodnaviria; Sangervirae; Phixviricota; Malgrandaviricetes; Petitvirales; Microviridae; unclassified Microviridae
GCA_003848245.1	5360	6	Alces alces faeces associated microvirus MP12 5423	NA	Viruses; Monodnaviria; Sangervirae; Phixviricota; Malgrandaviricetes; Petitvirales; Microviridae; unclassified Microviridae
GCA_003848225.1	5004	4	Alces alces faeces associated microvirus MP15 5067	NA	Viruses; Monodnaviria; Sangervirae; Phixviricota; Malgrandaviricetes; Petitvirales; Microviridae; unclassified Microviridae
GCA_003848205.1	4892	4	Alces alces faeces associated microvirus MP18 4940	NA	Viruses; Monodnaviria; Sangervirae; Phixviricota; Malgrandaviricetes; Petitvirales; Microviridae; unclassified Microviridae

gca	genome.size	proteome.size	phage.name	strain	Taxonomy
GCA_003848185.1	4662	3	Alces alces faeces associated microvirus MP21 4718	NA	Viruses; Monodnaviria; Sangervirae; Phixviricota; Malgrandaviricetes; Petitvirales; Microviridae; unclassified Microviridae
GCA_003848305.1	6434	3	Alces alces faeces associated microvirus MP3 6497	NA	Viruses; Monodnaviria; Sangervirae; Phixviricota; Malgrandaviricetes; Petitvirales; Microviridae; unclassified Microviridae
GCA_003848325.1	5265	4	Lynx canadensis associated microvirus CLP 9366	NA	Viruses; Monodnaviria; Sangervirae; Phixviricota; Malgrandaviricetes; Petitvirales; Microviridae; unclassified Microviridae
GCA_003848345.1	5312	4	Lynx canadensis associated microvirus CLP 9413	NA	Viruses; Monodnaviria; Sangervirae; Phixviricota; Malgrandaviricetes; Petitvirales; Microviridae; unclassified Microviridae
GCA_003963675.1	3028	2	Alces alces faeces associated smacovirus MP78	NA	Viruses; Monodnaviria; Shotokuvirae; Cressdnaviricota; Arfiviricetes; Cremevirales; Smacoviridae; Porprismacovirus; unclassified Porprismacovirus
GCA_003848145.1	2826	2	Alces alces faeces associated circular virus MP65	NA	Viruses; Monodnaviria; Shotokuvirae; Cressdnaviricota; Repensiviricetes; Geplafuvirales; Genomoviridae; unclassified Genomoviridae
GCA_000846645.1	148317	233	Staphylococcus virus K	NA	Viruses; dsDNA viruses, no RNA stage; Caudovirales; Myoviridae; Spounavirinae; Kayvirus
GCA_014883515.1	7154	1	Porcine teschovirus 15	JPNIshiTa22016G new	Viruses; Riboviria; Orthornavirae; Pisuviricota; Pisoniviricetes; Picornavirales; Picornaviridae; Teschovirus; unclassified Teschovirus
GCA_014883525.1	3342	1	Porcine teschovirus 14	JPNMoI2222015G new	Viruses; Riboviria; Orthornavirae; Pisuviricota; Pisoniviricetes; Picornavirales; Picornaviridae; Teschovirus; unclassified Teschovirus
GCA_003848165.1	6357	3	Lepus americanus faeces associated microvirus SHP1 6472	NA	Viruses; Monodnaviria; Sangervirae; Phixviricota; Malgrandaviricetes; Petitvirales; Microviridae; unclassified Microviridae
GCA_006451855.1	104894	95	Terrapene carolina carolina ranavirus	NA	Viruses; Varidnaviria; Bamfordvirae; Nucleocytoviricota; Megaviricetes; Pimascovirales; Iridoviridae; Alphairidovirinae; Ranavirus; Frog virus 3
GCA_006451895.1	104803	95	Trioceros melleri ranavirus	NA	Viruses; Varidnaviria; Bamfordvirae; Nucleocytoviricota; Megaviricetes; Pimascovirales; Iridoviridae; Alphairidovirinae; Ranavirus; Frog virus 3
GCA_004133065.1	2707	5	Yerba mateassociated circular DNA virus 1	NA	Viruses; unclassified viruses; unclassified DNA viruses; unclassified ssDNA viruses
GCA_003260755.1	6519	1	Sichuan takin enterovirus	NA	Viruses; Riboviria; Orthornavirae; Pisuviricota; Pisoniviricetes; Picornavirales; Picornaviridae; Enterovirus; unclassified Enterovirus
GCA_003260775.1	6233	3	Sichuan takin astrovirus	NA	Viruses; Riboviria; Orthornavirae; Pisuviricota; Stelpaviricetes; Stellavirales; Astroviridae; unclassified Astroviridae
GCA_000845825.1	2768	6	Ageratum yellow vein China virus Hn2	NA	Viruses; Monodnaviria; Shotokuvirae; Cressdnaviricota; Repensiviricetes; Geplafuvirales; Geminiviridae; Begomovirus; Ageratum yellow vein virus
GCA_000838085.1	2748	6	Ageratum yellow vein Sri Lanka virus	NA	Viruses; ssDNA viruses; Geminiviridae; Begomovirus
GCA_000841845.1	5197	6	Bean golden mosaic virusBrazil	Type I	Viruses; Monodnaviria; Shotokuvirae; Cressdnaviricota; Repensiviricetes; Geplafuvirales; Geminiviridae; Begomovirus; Bean golden mosaic virus
GCA_002867405.1	5255	5	Bean golden yellow mosaic virusDominican Republic	type II	Viruses; Monodnaviria; Shotokuvirae; Cressdnaviricota; Repensiviricetes; Geplafuvirales; Geminiviridae; Begomovirus; Bean golden yellow mosaic virus
GCA_000840225.1	5232	8	Bean golden yellow mosaic virusPuerto RicoJapan	NA	Viruses; Monodnaviria; Shotokuvirae; Cressdnaviricota; Repensiviricetes; Geplafuvirales; Geminiviridae; Begomovirus; Bean golden yellow mosaic virus
GCA_000859865.1	2787	5	Chayote yellow mosaic virus	NA	Viruses; ssDNA viruses; Geminiviridae; Begomovirus
GCA_000841265.1	2754	6	Chilli leaf curl virusMultan	NA	Viruses; Monodnaviria; Shotokuvirae; Cressdnaviricota; Repensiviricetes; Geplafuvirales; Geminiviridae; Begomovirus; Chilli leaf curl virus
GCA_000838385.1	5213	7	Chino del tomate virusIC	NA	Viruses; Monodnaviria; Shotokuvirae; Cressdnaviricota; Repensiviricetes; Geplafuvirales; Geminiviridae; Begomovirus; Chino del tomate virus
GCA_002822045.1	2730	6	Cotton leaf curl Alabad virus	NA	Viruses; ssDNA viruses; Geminiviridae; Begomovirus
GCA_000842045.1	2744	6	Cotton leaf curl Alabad virus802a	Pakistan	Viruses; Monodnaviria; Shotokuvirae; Cressdnaviricota; Repensiviricetes; Geplafuvirales; Geminiviridae; Begomovirus; Cotton leaf curl Alabad virus
GCA_000838805.1	2764	6	Cotton leaf curl Gezira virus	NA	Viruses; ssDNA viruses; Geminiviridae; Begomovirus
GCA_002822085.1	2761	5	Cotton leaf curl Gezira virusCotton	NA	Viruses; Monodnaviria; Shotokuvirae; Cressdnaviricota; Repensiviricetes; Geplafuvirales; Geminiviridae; Begomovirus; Cotton leaf curl Gezira virus
GCA_002822185.1	2736	6	Cotton leaf curl Kokhran virus	NA	Viruses; ssDNA viruses; Geminiviridae; Begomovirus
GCA_000840425.1	2748	6	Cotton leaf curl Kokhran virus806b	Pakistan	Viruses; Monodnaviria; Shotokuvirae; Cressdnaviricota; Repensiviricetes; Geplafuvirales; Geminiviridae; Begomovirus; Cotton leaf curl Kokhran virus
GCA_000858265.1	2714	6	Squash leaf curl Yunnan virus	NA	Viruses; ssDNA viruses; Geminiviridae; Begomovirus
GCA_000842105.1	2731	6	Malvastrum yellow vein virusY47	NA	Viruses; Monodnaviria; Shotokuvirae; Cressdnaviricota; Repensiviricetes; Geplafuvirales; Geminiviridae; Begomovirus; Malvastrum yellow vein virus
GCA_000856985.1	4377	3	Macrobrachium rosenbergii nodavirus	NA	Viruses; ssRNA viruses; ssRNA positive-strand viruses, no DNA stage; Nodaviridae; unclassified Nodaviridae
GCA_000841465.2	75898	125	Halovirus HF1	NA	Viruses; dsDNA viruses, no RNA stage; unclassified dsDNA viruses; unclassified archaeal dsDNA viruses; Haloviruses
GCA_008799915.1	7415	1	Enterovirus B77	NA	Viruses; Riboviria; Orthornavirae; Pisuviricota; Pisoniviricetes; Picornavirales; Picornaviridae; Enterovirus; Enterovirus B

gca	genome.size	proteome.size	phage.name	strain	Taxonomy
GCA__008799115.1	7410	1	Enterovirus B74	USACA7510213	Viruses; Riboviria; Orthornavirae; Pisuviricota; Pisoniviricetes; Picornavirales; Picornaviridae; Enterovirus; Enterovirus B
GCA__003847685.1	2123	2	Bark beetleassociated genomovirus 1	NA	Viruses; Monodnaviria; Shotokuvirae; Cressdnaviricota; Repensiviricetes; Geplafuvirales; Genomoviridae; unclassified Genomoviridae
GCA__003847665.1	2106	2	Bark beetleassociated genomovirus 2	NA	Viruses; Monodnaviria; Shotokuvirae; Cressdnaviricota; Repensiviricetes; Geplafuvirales; Genomoviridae; unclassified Genomoviridae
GCA__003847645.1	2125	2	Bark beetleassociated genomovirus 3	NA	Viruses; Monodnaviria; Shotokuvirae; Cressdnaviricota; Repensiviricetes; Geplafuvirales; Genomoviridae; unclassified Genomoviridae
GCA__003847625.1	2122	2	Bark beetleassociated genomovirus 4	NA	Viruses; Monodnaviria; Shotokuvirae; Cressdnaviricota; Repensiviricetes; Geplafuvirales; Genomoviridae; unclassified Genomoviridae
GCA__003847605.1	2205	2	Bark beetleassociated genomovirus 5	NA	Viruses; Monodnaviria; Shotokuvirae; Cressdnaviricota; Repensiviricetes; Geplafuvirales; Genomoviridae; unclassified Genomoviridae
GCA__003847585.1	2264	2	Bark beetleassociated genomovirus 6	NA	Viruses; Monodnaviria; Shotokuvirae; Cressdnaviricota; Repensiviricetes; Geplafuvirales; Genomoviridae; unclassified Genomoviridae
GCA__003260715.1	12842	2	Aedes anphevirus	RML12	Viruses; Riboviria; Orthornavirae; Negarnaviricota; Haploviricotina; Monjiviricetes; Mononegavirales; Xinmoviridae; Anphevirus; unclassified Anphevirus
GCA__003341835.1	43468	66	Salmonella phage S100	NA	Viruses; Duplodnaviria; Heunggongvirae; Uroviricota; Caudoviricetes; Caudovirales; Siphoviridae; Guernseyvirinae; Jerseyvirus; unclassified Jerseyvirus
GCA__003342515.1	42621	65	Salmonella phage S101	NA	Viruses; Duplodnaviria; Heunggongvirae; Uroviricota; Caudoviricetes; Caudovirales; Siphoviridae; Guernseyvirinae; Jerseyvirus; unclassified Jerseyvirus
GCA__003341855.1	42439	64	Salmonella phage S102	NA	Viruses; Duplodnaviria; Heunggongvirae; Uroviricota; Caudoviricetes; Caudovirales; Siphoviridae; Guernseyvirinae; Jerseyvirus; unclassified Jerseyvirus
GCA__003341875.1	42441	63	Salmonella phage S103	NA	Viruses; Duplodnaviria; Heunggongvirae; Uroviricota; Caudoviricetes; Caudovirales; Siphoviridae; Guernseyvirinae; Jerseyvirus; unclassified Jerseyvirus
GCA__003341895.1	43118	67	Salmonella phage S104	NA	Viruses; Duplodnaviria; Heunggongvirae; Uroviricota; Caudoviricetes; Caudovirales; Siphoviridae; Guernseyvirinae; Jerseyvirus; unclassified Jerseyvirus
GCA__003341915.1	42976	66	Salmonella phage S106	NA	Viruses; Duplodnaviria; Heunggongvirae; Uroviricota; Caudoviricetes; Caudovirales; Siphoviridae; Guernseyvirinae; Jerseyvirus; unclassified Jerseyvirus
GCA__003341935.1	49818	82	Salmonella phage S107	NA	Viruses; Duplodnaviria; Heunggongvirae; Uroviricota; Caudoviricetes; Caudovirales; Siphoviridae; unclassified Siphoviridae
GCA__003341955.1	43421	67	Salmonella phage S111	NA	Viruses; Duplodnaviria; Heunggongvirae; Uroviricota; Caudoviricetes; Caudovirales; Siphoviridae; Guernseyvirinae; Jerseyvirus; unclassified Jerseyvirus
GCA__003341975.1	112582	168	Salmonella phage S113	NA	Viruses; Duplodnaviria; Heunggongvirae; Uroviricota; Caudoviricetes; Caudovirales; Demerecviridae; Markadamsvirinae; Epseptimavirus; Salmonella virus S113
GCA__003341995.1	110926	163	Salmonella phage S114	NA	Viruses; Duplodnaviria; Heunggongvirae; Uroviricota; Caudoviricetes; Caudovirales; Demerecviridae; Markadamsvirinae; Epseptimavirus; Salmonella virus S114
GCA__003342015.1	157946	204	Salmonella phage S115	NA	Viruses; Duplodnaviria; Heunggongvirae; Uroviricota; Caudoviricetes; Caudovirales; Ackermannviridae; Cvivirinae; Kuttervirus; unclassified Kuttervirus
GCA__003342035.1	110664	159	Salmonella phage S116	NA	Viruses; Duplodnaviria; Heunggongvirae; Uroviricota; Caudoviricetes; Caudovirales; Demerecviridae; Markadamsvirinae; Epseptimavirus; Salmonella virus S116
GCA__003342055.1	158110	204	Salmonella phage S117	NA	Viruses; Duplodnaviria; Heunggongvirae; Uroviricota; Caudoviricetes; Caudovirales; Ackermannviridae; Cvivirinae; Kuttervirus; unclassified Kuttervirus
GCA__003342535.1	157013	203	Salmonella phage S118	NA	Viruses; Duplodnaviria; Heunggongvirae; Uroviricota; Caudoviricetes; Caudovirales; Ackermannviridae; Cvivirinae; Kuttervirus; unclassified Kuttervirus
GCA__003342075.1	43876	67	Salmonella phage S119	NA	Viruses; Duplodnaviria; Heunggongvirae; Uroviricota; Caudoviricetes; Caudovirales; Siphoviridae; Guernseyvirinae; Jerseyvirus; unclassified Jerseyvirus
GCA__003342095.1	43467	66	Salmonella phage S120	NA	Viruses; Duplodnaviria; Heunggongvirae; Uroviricota; Caudoviricetes; Caudovirales; Siphoviridae; Guernseyvirinae; Jerseyvirus; unclassified Jerseyvirus
GCA__003342115.1	43467	66	Salmonella phage S123	NA	Viruses; Duplodnaviria; Heunggongvirae; Uroviricota; Caudoviricetes; Caudovirales; Siphoviridae; Guernseyvirinae; Jerseyvirus; unclassified Jerseyvirus
GCA__003342415.1	112564	156	Salmonella phage S124	NA	Viruses; Duplodnaviria; Heunggongvirae; Uroviricota; Caudoviricetes; Caudovirales; Demerecviridae; Markadamsvirinae; Epseptimavirus; Salmonella virus S124
GCA__003342135.1	111999	153	Salmonella phage S126	NA	Viruses; Duplodnaviria; Heunggongvirae; Uroviricota; Caudoviricetes; Caudovirales; Demerecviridae; Markadamsvirinae; Epseptimavirus; Salmonella virus S126
GCA__003342175.1	110091	150	Salmonella phage S131	NA	Viruses; Duplodnaviria; Heunggongvirae; Uroviricota; Caudoviricetes; Caudovirales; Demerecviridae; Markadamsvirinae; Tequintavirus; Salmonella virus S131
GCA__003342195.1	116832	147	Salmonella phage S132	NA	Viruses; Duplodnaviria; Heunggongvirae; Uroviricota; Caudoviricetes; Caudovirales; Demerecviridae; Markadamsvirinae; Epseptimavirus; Salmonella virus S132
GCA__003342215.1	110926	163	Salmonella phage S133	NA	Viruses; Duplodnaviria; Heunggongvirae; Uroviricota; Caudoviricetes; Caudovirales; Demerecviridae; Markadamsvirinae; Epseptimavirus; Salmonella virus S133
GCA__003342555.1	43118	67	Salmonella phage S134	NA	Viruses; Duplodnaviria; Heunggongvirae; Uroviricota; Caudoviricetes; Caudovirales; Siphoviridae; Guernseyvirinae; Jerseyvirus; unclassified Jerseyvirus
GCA__003342235.1	50591	84	Salmonella phage S135	NA	Viruses; Duplodnaviria; Heunggongvirae; Uroviricota; Caudoviricetes; Caudovirales; Siphoviridae; unclassified Siphoviridae
GCA__003342255.1	50593	83	Salmonella phage S137	NA	Viruses; Duplodnaviria; Heunggongvirae; Uroviricota; Caudoviricetes; Caudovirales; Siphoviridae; unclassified Siphoviridae
GCA__003342275.1	43119	67	Salmonella phage S138	NA	Viruses; Duplodnaviria; Heunggongvirae; Uroviricota; Caudoviricetes; Caudovirales; Siphoviridae; Guernseyvirinae; Jerseyvirus; unclassified Jerseyvirus

gca	genome.size	proteome.size	phage.name	strain	Taxonomy
GCA_003342295.1	43119	67	Salmonella phage S142	NA	Viruses; Duplodnaviria; Heunggongvirae; Uroviricota; Caudoviricetes; Caudovirales; Siphoviridae; Guernseyvirinae; Jerseyvirus; unclassified Jerseyvirus
GCA_003342315.1	111447	161	Salmonella phage S147	NA	Viruses; Duplodnaviria; Heunggongvirae; Uroviricota; Caudoviricetes; Caudovirales; Demerecviridae; Markadamsvirinae; Epseptimavirus; Salmonella virus S147
GCA_013088075.1	2964	6	Sesame yellow mosaic virus	NA	Viruses; Monodnaviria; Shotokuvirae; Cressdnaviricota; Repensiviricetes; Geplafuvirales; Geminiviridae; Turncurtovirus; unclassified Turncurtovirus
GCA_018583015.1	9450	5	Sclerotinia sclerotiorum negativestranded RNA virus 2A	NA	Viruses; Riboviria; unclassified Riboviria; unclassified ssRNA viruses; unclassified ssRNA negative-strand viruses
GCA_018583005.1	2442	1	Sclerotinia sclerotiorum ourmialike virus 3	NA	Viruses; Riboviria; Orthornavirae; Lenarviricota; Miaviricetes; Ourlivirales; Botourmiaviridae; Botoulivirus; Sclerotinia botoulivirus 3
GCA_003848365.1	2166	2	Sclerotinia sclerotiorum hypovirulence associated DNA virus 1A	NA	Viruses; Monodnaviria; Shotokuvirae; Cressdnaviricota; Repensiviricetes; Geplafuvirales; Genomoviridae; Gemycircularvirus; Sclerotinia gemycircularvirus 1; Sclerotinia sclerotiorum hypovirulence associated DNA virus 1
GCA_000859325.1	2743	6	Tobacco curly shoot virus Y41	NA	Viruses; Monodnaviria; Shotokuvirae; Cressdnaviricota; Repensiviricetes; Geplafuvirales; Geminiviridae; Begomovirus; Tobacco curly shoot virus
GCA_000839845.1	2754	6	Cotton leaf curl Multan virus	NA	Viruses; ssDNA viruses; Geminiviridae; Begomovirus
GCA_000841225.1	2751	6	Cotton leaf curl Multan virus62	Pakistan	Viruses; Monodnaviria; Shotokuvirae; Cressdnaviricota; Repensiviricetes; Geplafuvirales; Geminiviridae; Begomovirus; Cotton leaf curl Multan virus
GCA_002986465.1	2722	6	Cotton leaf curl Multan virusFaisalabad3	NA	Viruses; Monodnaviria; Shotokuvirae; Cressdnaviricota; Repensiviricetes; Geplafuvirales; Geminiviridae; Begomovirus; Cotton leaf curl Multan virus
GCA_002822245.1	2728	6	Cowpea golden mosaic virusNigeria	NA	Viruses; Monodnaviria; Shotokuvirae; Cressdnaviricota; Repensiviricetes; Geplafuvirales; Geminiviridae; Begomovirus; unclassified Begomovirus
GCA_000858965.1	5543	8	East African cassava mosaic Cameroon virus	WACMVCM	Viruses; ssDNA viruses; Geminiviridae; Begomovirus
GCA_000912855.1	5558	7	East African cassava mosaic Malawi virus	NA	Viruses; ssDNA viruses; Geminiviridae; Begomovirus
GCA_002986505.1	2804	3	East African cassava mosaic Malawi virusMalawiK	NA	Viruses; Monodnaviria; Shotokuvirae; Cressdnaviricota; Repensiviricetes; Geplafuvirales; Geminiviridae; Begomovirus; East African cassava mosaic Malawi virus
GCA_000859785.1	5576	4	East African cassava mosaic virusUganda2 Severe	NA	Viruses; Monodnaviria; Shotokuvirae; Cressdnaviricota; Repensiviricetes; Geplafuvirales; Geminiviridae; Begomovirus; East African cassava mosaic virus
GCA_000845125.1	5548	8	East African cassava mosaic Zanzibar virus	NA	Viruses; ssDNA viruses; Geminiviridae; Begomovirus
GCA_002986575.1	2765	6	Eupatorium yellow vein virusMNS2	NA	Viruses; Monodnaviria; Shotokuvirae; Cressdnaviricota; Repensiviricetes; Geplafuvirales; Geminiviridae; Begomovirus; Eupatorium yellow vein virus
GCA_000842745.1	2755	6	Hollyhock leaf crumple virusCairo	NA	Viruses; Monodnaviria; Shotokuvirae; Cressdnaviricota; Repensiviricetes; Geplafuvirales; Geminiviridae; Begomovirus; Hollyhock leaf crumple virus
GCA_000846245.1	5186	7	Macroptilium mosaic Puerto Rico virus	NA	Viruses; ssDNA viruses; Geminiviridae; Begomovirus
GCA_000844525.1	5247	7	Macroptilium yellow mosaic Florida virus	NA	Viruses; ssDNA viruses; Geminiviridae; Begomovirus
GCA_002986635.1	2630	4	Macroptilium yellow mosaic virusCuba	NA	Viruses; Monodnaviria; Shotokuvirae; Cressdnaviricota; Repensiviricetes; Geplafuvirales; Geminiviridae; Begomovirus; Macroptilium yellow mosaic virus
GCA_001430695.1	5300	6	Melon chlorotic leaf curl virusGuatemala	NA	Viruses; Monodnaviria; Shotokuvirae; Cressdnaviricota; Repensiviricetes; Geplafuvirales; Geminiviridae; Begomovirus; Melon chlorotic leaf curl virus
GCA_000858565.1	5361	9	Mungbean yellow mosaic India virus	bg3	Viruses; ssDNA viruses; Geminiviridae; Begomovirus
GCA_000840545.1	2739	6	Okra yellow vein mosaic virus201	Pakistan	Viruses; Monodnaviria; Shotokuvirae; Cressdnaviricota; Repensiviricetes; Geplafuvirales; Geminiviridae; Begomovirus; Bhendi yellow vein mosaic virus
GCA_000842765.1	5208	6	Pepper golden mosaic virus	Tamaulipas	Viruses; ssDNA viruses; Geminiviridae; Begomovirus
GCA_002823045.1	2619	4	Pepper golden mosaic virusCR	Costa Rica	Viruses; Monodnaviria; Shotokuvirae; Cressdnaviricota; Repensiviricetes; Geplafuvirales; Geminiviridae; Begomovirus; Pepper golden mosaic virus
GCA_000839505.1	5220	6	Pepper huasteco yellow vein virus	NA	Viruses; ssDNA viruses; Geminiviridae; Begomovirus
GCA_002823105.1	2754	7	Pepper leaf curl Bangladesh virus	NA	Viruses; ssDNA viruses; Geminiviridae; Begomovirus
GCA_002823145.1	2739	6	Pepper leaf curl virusMalaysia	NA	Viruses; Monodnaviria; Shotokuvirae; Cressdnaviricota; Repensiviricetes; Geplafuvirales; Geminiviridae; Begomovirus; Pepper leaf curl virus
GCA_000839305.1	5126	7	Potato yellow mosaic Panama virus	NA	Viruses; ssDNA viruses; Geminiviridae; Begomovirus
GCA_000838665.1	5140	6	Potato yellow mosaic virus Venezuela	NA	Viruses; Monodnaviria; Shotokuvirae; Cressdnaviricota; Repensiviricetes; Geplafuvirales; Geminiviridae; Begomovirus; Potato yellow mosaic virus
GCA_000840525.1	5192	2	Sida golden mosaic Costa Rica virus	NA	Viruses; ssDNA viruses; Geminiviridae; Begomovirus
GCA_002823365.1	2581	7	Sida golden mosaic Florida virusUSA	NA	Viruses; Monodnaviria; Shotokuvirae; Cressdnaviricota; Repensiviricetes; Geplafuvirales; Geminiviridae; Begomovirus; Sida golden mosaic Florida virus
GCA_000841325.1	5192	7	Sida golden mosaic Honduras virus	NA	Viruses; ssDNA viruses; Geminiviridae; Begomovirus

gca	genome.size	proteome.size	phage.name	strain	Taxonomy
GCA_002823405.1	2603	6	Sida golden yellow vein virusA11	NA	Viruses; Monodnaviria; Shotokuvirae; Cressdnaviricota; Repensiviricetes; Geplafuvirales; Geminiviridae; Begomovirus; Sida golden yellow vein virus
GCA_000841285.1	2668	6	Sida mottle virusBrazil	NA	Viruses; Monodnaviria; Shotokuvirae; Cressdnaviricota; Repensiviricetes; Geplafuvirales; Geminiviridae; Begomovirus; Sida mottle virus
GCA_000864865.1	2661	6	Sida yellow mosaic virusBrazil	NA	Viruses; Monodnaviria; Shotokuvirae; Cressdnaviricota; Repensiviricetes; Geplafuvirales; Geminiviridae; Begomovirus; Sida yellow mosaic virus
GCA_000843025.1	5205	7	Sida yellow vein virus	Honduras substr yellow vein	Viruses; ssDNA viruses; Geminiviridae; Begomovirus
GCA_000844565.1	5190	6	Squash mild leaf curl virusImperial Valley	NA	Viruses; Monodnaviria; Shotokuvirae; Cressdnaviricota; Repensiviricetes; Geplafuvirales; Geminiviridae; Begomovirus; Squash mild leaf curl virus
GCA_000858725.1	5466	8	Sri Lankan cassava mosaic virusColombo	NA	Viruses; Monodnaviria; Shotokuvirae; Cressdnaviricota; Repensiviricetes; Geplafuvirales; Geminiviridae; Begomovirus; Sri Lankan cassava mosaic virus
GCA_000843765.1	2749	6	Stachytarpheta leaf curl virus Hn5	NA	Viruses; Monodnaviria; Shotokuvirae; Cressdnaviricota; Repensiviricetes; Geplafuvirales; Geminiviridae; Begomovirus; Stachytarpheta leaf curl virus
GCA_000842125.1	2773	6	Sweet potato leaf curl Georgia virus16	NA	Viruses; Monodnaviria; Shotokuvirae; Cressdnaviricota; Repensiviricetes; Geplafuvirales; Geminiviridae; Begomovirus; Sweet potato leaf curl Georgia virus
GCA_000842145.1	2761	6	Tobacco leaf curl Japan virus	NA	Viruses; ssDNA viruses; Geminiviridae; Begomovirus
GCA_000838925.1	2767	6	Tobacco leaf curl Zimbabwe virus	NA	Viruses; ssDNA viruses; Geminiviridae; Begomovirus
GCA_000838345.1	5195	7	Tomato chlorotic mottle virusBrazil	NA	Viruses; Monodnaviria; Shotokuvirae; Cressdnaviricota; Repensiviricetes; Geplafuvirales; Geminiviridae; Begomovirus; Tomato chlorotic mottle virus
GCA_002823945.1	2625	6	Tomato chlorotic mottle virusCrumple	NA	Viruses; Monodnaviria; Shotokuvirae; Cressdnaviricota; Repensiviricetes; Geplafuvirales; Geminiviridae; Begomovirus; Tomato chlorotic mottle virus
GCA_000839565.1	5096	1	Tomato golden mosaic virusYellow vein	NA	Viruses; Monodnaviria; Shotokuvirae; Cressdnaviricota; Repensiviricetes; Geplafuvirales; Geminiviridae; Begomovirus; Tomato golden mosaic virus
GCA_000839225.1	5172	7	Tomato golden mottle virus	NA	Viruses; ssDNA viruses; Geminiviridae; Begomovirus
GCA_000840445.1	2761	6	Tomato leaf curl Bangladesh virus	TLCVBD2	Viruses; ssDNA viruses; Geminiviridae; Begomovirus
GCA_000842065.1	2748	6	Tomato leaf curl Laos virus	TLCVLA	Viruses; ssDNA viruses; Geminiviridae; Begomovirus
GCA_000843005.1	2754	6	Tomato leaf curl Malaysia virus	NA	Viruses; ssDNA viruses; Geminiviridae; Begomovirus
GCA_000842925.1	5435	10	Tomato leaf curl New Delhi virusSevere	NA	Viruses; Monodnaviria; Shotokuvirae; Cressdnaviricota; Repensiviricetes; Geplafuvirales; Geminiviridae; Begomovirus; Tomato leaf curl New Delhi virus
GCA_000841305.1	2756	6	Tomato leaf curl Sri Lanka virus	NA	Viruses; ssDNA viruses; Geminiviridae; Begomovirus
GCA_000838425.1	2766	6	Tomato leaf curl virusAU	NA	Viruses; Monodnaviria; Shotokuvirae; Cressdnaviricota; Repensiviricetes; Geplafuvirales; Geminiviridae; Begomovirus; Tomato leaf curl virus
GCA_006416475.1	43842	32	Duck adenovirus 3	NA	Viruses; Varidnaviria; Bamfordvirae; Preplasmiviricota; Tectiliviricetes; Rowavirales; Adenoviridae; Aviadenovirus; unclassified Aviadenovirus
GCA_003307495.1	40876	60	Enterococcus phage LY0323	NA	Viruses; Duplodnaviria; Heunggongvirae; Uroviricota; Caudoviricetes; Caudovirales; Siphoviridae; Efquatrovirus; unclassified Efquatrovirus
GCA_000837605.1	5206	7	Tomato mosaic Havana virusQuivican	NA	Viruses; Monodnaviria; Shotokuvirae; Cressdnaviricota; Repensiviricetes; Geplafuvirales; Geminiviridae; Begomovirus; Tomato mosaic Havana virus
GCA_000838745.1	5159	6	Tomato mottle Taino virus	NA	Viruses; ssDNA viruses; Geminiviridae; Begomovirus
GCA_000837105.1	5145	6	Tomato mottle virusFlorida	NA	Viruses; Monodnaviria; Shotokuvirae; Cressdnaviricota; Repensiviricetes; Geplafuvirales; Geminiviridae; Begomovirus; Tomato mottle virus
GCA_000837205.1	5194	7	Tomato rugose mosaic virusUbe	NA	Viruses; Monodnaviria; Shotokuvirae; Cressdnaviricota; Repensiviricetes; Geplafuvirales; Geminiviridae; Begomovirus; Tomato rugose mosaic virus
GCA_000845785.1	2593	4	Tomato severe leaf curl virusGuatemala 961	NA	Viruses; Monodnaviria; Shotokuvirae; Cressdnaviricota; Repensiviricetes; Geplafuvirales; Geminiviridae; Begomovirus; Tomato severe leaf curl virus
GCA_003307575.1	39825	45	Escherichia phage vB_EcoP_S523	NA	Viruses; Duplodnaviria; Heunggongvirae; Uroviricota; Caudoviricetes; Caudovirales; Autographiviridae; Studiervirinae; Berlinvirus; Escherichia virus S523
GCA_003307595.1	49788	88	Escherichia phage LL5	Escherichia coli str MG1655	Viruses; Duplodnaviria; Heunggongvirae; Uroviricota; Caudoviricetes; Caudovirales; Drexlerviridae; Tempevirinae; Tlsvirus; Escherichia virus LL5
GCA_003342355.1	136026	213	Escherichia phage LL12	Escherichia coli str MG1655	Viruses; Duplodnaviria; Heunggongvirae; Uroviricota; Caudoviricetes; Caudovirales; Myoviridae; Vequintavirinae; Vequintavirus; unclassified Vequintavirus
GCA_003308315.1	53140	97	Gordonia phage Nedarya	NA	Viruses; Duplodnaviria; Heunggongvirae; Uroviricota; Caudoviricetes; Caudovirales; Siphoviridae; Soupsvirus; unclassified Soupsvirus
GCA_003308875.1	156199	229	Mycobacterium phage Derek	NA	Viruses; Duplodnaviria; Heunggongvirae; Uroviricota; Caudoviricetes; Caudovirales; Myoviridae; Bixzunavirus; unclassified Bixzunavirus
GCA_003308235.1	64877	89	Mycobacterium phage Erk16	NA	Viruses; Duplodnaviria; Heunggongvirae; Uroviricota; Caudoviricetes; Caudovirales; Siphoviridae; Dclasvirinae; Plotvirus; Mycobacterium virus Plot
GCA_003308255.1	50061	88	Mycobacterium phage Hookmount	NA	Viruses; Duplodnaviria; Heunggongvirae; Uroviricota; Caudoviricetes; Caudovirales; Siphoviridae; Fromanvirus; unclassified Fromanvirus

gca	genome.size	proteome.size	phage.name	strain	Taxonomy
GCA_003308275.1	54118	95	Mycobacterium phage Koella	NA	Viruses; Duplodnaviria; Heunggongvirae; Uroviricota; Caudoviricetes; Caudovirales; Siphoviridae; Cheoctovirus; unclassified Cheoctovirus
GCA_003308295.1	69800	103	Mycobacterium phage Mortcellus	NA	Viruses; Duplodnaviria; Heunggongvirae; Uroviricota; Caudoviricetes; Caudovirales; Siphoviridae; Bclasvirinae; Pipefishvirus; unclassified Pipefishvirus
GCA_003308895.1	155006	234	Mycobacterium phage ParkTD	NA	Viruses; Duplodnaviria; Heunggongvirae; Uroviricota; Caudoviricetes; Caudovirales; Myoviridae; Bixzunavirus; unclassified Bixzunavirus
GCA_003308335.1	68527	100	Mycobacterium phage Phleuron	NA	Viruses; Duplodnaviria; Heunggongvirae; Uroviricota; Caudoviricetes; Caudovirales; Siphoviridae; Bclasvirinae; Pegunavirus; unclassified Pegunavirus
GCA_003308375.1	43243	74	Mycobacterium phage Silvafighter	NA	Viruses; Duplodnaviria; Heunggongvirae; Uroviricota; Caudoviricetes; Caudovirales; Siphoviridae; Nclasvirinae; Charlievirus; unclassified Charlievirus
GCA_003308355.1	46544	67	Rhodococcus phage Shuman	NA	Viruses; Duplodnaviria; Heunggongvirae; Uroviricota; Caudoviricetes; Caudovirales; Siphoviridae; Rerduovirus; unclassified Rerduovirus
GCA_003342395.1	47393	90	Salmonella phage Skate	NA	Viruses; Duplodnaviria; Heunggongvirae; Uroviricota; Caudoviricetes; Caudovirales; Siphoviridae; unclassified Siphoviridae
GCA_003368645.1	141446	203	Staphylococcus phage Quidividi	NA	Viruses; Duplodnaviria; Heunggongvirae; Uroviricota; Caudoviricetes; Caudovirales; Herelleviridae; Twortvirinae; Sepunavirus; unclassified Sepunavirus
GCA_003368665.1	142592	206	Staphylococcus phage Twillingate	NA	Viruses; Duplodnaviria; Heunggongvirae; Uroviricota; Caudoviricetes; Caudovirales; Herelleviridae; Twortvirinae; Sepunavirus; unclassified Sepunavirus
GCA_003342375.1	43147	67	Staphylococcus phage Lorac	NA	Viruses; Duplodnaviria; Heunggongvirae; Uroviricota; Caudoviricetes; Caudovirales; Siphoviridae; Phietavirus; unclassified Phietavirus
GCA_003308395.1	166130	276	Klebsiella phage Mineola	NA	Viruses; Duplodnaviria; Heunggongvirae; Uroviricota; Caudoviricetes; Caudovirales; Myoviridae; Tevenvirinae; Jiaodavirus; unclassified Jiaodavirus
GCA_003340835.1	53256	95	Mycobacterium phage Crucio	NA	Viruses; Duplodnaviria; Heunggongvirae; Uroviricota; Caudoviricetes; Caudovirales; Siphoviridae; Fromanvirus; unclassified Fromanvirus
GCA_003340855.1	50280	87	Mycobacterium phage Dublin	NA	Viruses; Duplodnaviria; Heunggongvirae; Uroviricota; Caudoviricetes; Caudovirales; Siphoviridae; Fromanvirus; unclassified Fromanvirus
GCA_003340875.1	52152	98	Mycobacterium phage Ebony	NA	Viruses; Duplodnaviria; Heunggongvirae; Uroviricota; Caudoviricetes; Caudovirales; Siphoviridae; Fromanvirus; unclassified Fromanvirus
GCA_003340895.1	52347	97	Mycobacterium phage Joselito	NA	Viruses; Duplodnaviria; Heunggongvirae; Uroviricota; Caudoviricetes; Caudovirales; Siphoviridae; Fromanvirus; unclassified Fromanvirus
GCA_003340915.1	51527	92	Mycobacterium phage Michley	NA	Viruses; Duplodnaviria; Heunggongvirae; Uroviricota; Caudoviricetes; Caudovirales; Siphoviridae; Fromanvirus; unclassified Fromanvirus
GCA_003340935.1	50505	87	Mycobacterium phage Mryolo	NA	Viruses; Duplodnaviria; Heunggongvirae; Uroviricota; Caudoviricetes; Caudovirales; Siphoviridae; Fromanvirus; unclassified Fromanvirus
GCA_003340955.1	50855	87	Mycobacterium phage Sabia	NA	Viruses; Duplodnaviria; Heunggongvirae; Uroviricota; Caudoviricetes; Caudovirales; Siphoviridae; Fromanvirus; unclassified Fromanvirus
GCA_003340975.1	51143	89	Mycobacterium phage Tarynearal	NA	Viruses; Duplodnaviria; Heunggongvirae; Uroviricota; Caudoviricetes; Caudovirales; Siphoviridae; Fromanvirus; unclassified Fromanvirus
GCA_003308515.1	168695	262	Escherichia phage SF	NA	Viruses; Duplodnaviria; Heunggongvirae; Uroviricota; Caudoviricetes; Caudovirales; Myoviridae; Tevenvirinae; Mosigvirus; unclassified Mosigvirus
GCA_003307395.1	73080	93	Pseudomonas phage LP14	NA	Viruses; Duplodnaviria; Heunggongvirae; Uroviricota; Caudoviricetes; Caudovirales; Podoviridae; Litunavirus; unclassified Litunavirus
GCA_003307435.1	89055	130	Staphylococcus phage VBSauSSA2	NA	Viruses; Duplodnaviria; Heunggongvirae; Uroviricota; Caudoviricetes; Caudovirales; Siphoviridae; Sextaecvirus; unclassified Sextaecvirus
GCA_003307455.1	43614	67	Salmonella phage vB_SpuS_Sp4	NA	Viruses; Duplodnaviria; Heunggongvirae; Uroviricota; Caudoviricetes; Caudovirales; Siphoviridae; Guernseyvirinae; Jerseyvirus; unclassified Jerseyvirus
GCA_003342455.1	40203	64	Enterococcus phage vB_EfaS_LM99	NA	Viruses; Duplodnaviria; Heunggongvirae; Uroviricota; Caudoviricetes; Caudovirales; Siphoviridae; Efquatrovirus; unclassified Efquatrovirus
GCA_003340995.1	167208	277	Escherichia phage vB_EcoM_JB75	NA	Viruses; Duplodnaviria; Heunggongvirae; Uroviricota; Caudoviricetes; Caudovirales; Myoviridae; Tevenvirinae; Tequatrovirus; unclassified Tequatrovirus
GCA_003307555.1	68270	78	Escherichia phage phi G17	NA	Viruses; Duplodnaviria; Heunggongvirae; Uroviricota; Caudoviricetes; Caudovirales; Podoviridae; Gamaleyavirus; unclassified Gamaleyavirus
GCA_005924165.1	150764	104	Vibrio phage VP1	NA	Viruses; Duplodnaviria; Heunggongvirae; Uroviricota; Caudoviricetes; Caudovirales; Ackermannviridae; unclassified Ackermannviridae
GCA_003341015.1	42858	56	Escherichia phage Halfdan	NA	Viruses; Duplodnaviria; Heunggongvirae; Uroviricota; Caudoviricetes; Caudovirales; Siphoviridae; Lokivirus; unclassified Lokivirus
GCA_003600665.1	41775	59	Escherichia phage C130_2	NA	Viruses; Duplodnaviria; Heunggongvirae; Uroviricota; Caudoviricetes; Caudovirales; Podoviridae; Giessenvirus; Escherichia virus C1302
GCA_000840605.1	7542	13	Vibrio phage VGJphi	NA	Viruses; ssDNA viruses; Inoviridae; Inovirus; unclassified Inovirus
GCA_000897495.1	11895	2	Grapevine Anatolian ringspot virus	NA	Viruses; ssRNA viruses; ssRNA positive-strand viruses, no DNA stage; Picornavirales; Secoviridae; Comovirinae; Nepovirus; Subgroup B
GCA_000868425.1	7382	3	Murine norovirus 1	NA	Viruses; ssRNA viruses; ssRNA positive-strand viruses, no DNA stage; Caliciviridae; Norovirus; Norwalk virus; Norovirus GV; Murine norovirus
GCA_000843745.1	113220	125	Adoxophyes honmai nucleopolyhedrovirus	ADN001	Viruses; dsDNA viruses, no RNA stage; Baculoviridae; Alphabaculovirus
GCA_000854205.1	5685	6	Barley yellow dwarf virusGAV	NA	Viruses; ssRNA viruses; ssRNA positive-strand viruses, no DNA stage; Luteoviridae; Luteovirus; unclassified Luteovirus

gca	genome.size	proteome.size	phage.name	strain	Taxonomy
GCA_004131705.1	4993	2	Panax notoginseng virus B	NA	Viruses; Riboviria; Orthornavirae; Duplornaviricota; Chrymotiviricetes; Ghabrivirales; Totiviridae; unclassified Totiviridae; Panax notoginseng virus A
GCA_018583155.1	116729	76	Phascolarctid gammaherpesvirus 1	36M11	Viruses; Duplodnaviria; Heunggongvirae; Peploviricota; Herviviricetes; Herpesvirales; Herpesviridae; Gammaherpesvirinae; Manticavirus
GCA_003307415.1	46500	58	Alteromonas phage JH01	NA	Viruses; Duplodnaviria; Heunggongvirae; Uroviricota; Caudoviricetes; Caudovirales; Siphoviridae; unclassified Siphoviridae
GCA_003307475.3	47475	62	Pseudomonas phage SCYZ1	NA	Viruses; Duplodnaviria; Heunggongvirae; Uroviricota; Caudoviricetes; Caudovirales; Podoviridae; Krylovvirus; unclassified Krylovvirus
GCA_003368965.1	56468	88	Paenibacillus phage Ash	NA	Viruses; Duplodnaviria; Heunggongvirae; Uroviricota; Caudoviricetes; Caudovirales; Siphoviridae; Trippvirus; unclassified Trippvirus
GCA_003368985.1	38519	66	Paenibacillus phage Bloom	NA	Viruses; Duplodnaviria; Heunggongvirae; Uroviricota; Caudoviricetes; Caudovirales; Siphoviridae; unclassified Siphoviridae
GCA_003369005.1	38675	67	Paenibacillus phage Eltigre	NA	Viruses; Duplodnaviria; Heunggongvirae; Uroviricota; Caudoviricetes; Caudovirales; Siphoviridae; Sitaravirus; unclassified Sitaravirus
GCA_003369085.1	38540	66	Paenibacillus phage Genki	NA	Viruses; Duplodnaviria; Heunggongvirae; Uroviricota; Caudoviricetes; Caudovirales; Siphoviridae; unclassified Siphoviridae
GCA_003369025.1	38526	66	Paenibacillus phage Jacopo	NA	Viruses; Duplodnaviria; Heunggongvirae; Uroviricota; Caudoviricetes; Caudovirales; Siphoviridae; unclassified Siphoviridae
GCA_003369045.1	56465	88	Paenibacillus phage Ley	NA	Viruses; Duplodnaviria; Heunggongvirae; Uroviricota; Caudoviricetes; Caudovirales; Siphoviridae; Trippvirus; unclassified Trippvirus
GCA_003369065.1	40437	72	Paenibacillus phage LincolnB	NA	Viruses; Duplodnaviria; Heunggongvirae; Uroviricota; Caudoviricetes; Caudovirales; Siphoviridae; Vegasvirus; unclassified Vegasvirus
GCA_003369105.1	37962	65	Paenibacillus phage Saudage	NA	Viruses; Duplodnaviria; Heunggongvirae; Uroviricota; Caudoviricetes; Caudovirales; Siphoviridae; Sitaravirus; unclassified Sitaravirus
GCA_003369125.1	38832	64	Paenibacillus phage Toothless	NA	Viruses; Duplodnaviria; Heunggongvirae; Uroviricota; Caudoviricetes; Caudovirales; Siphoviridae; Sitaravirus; unclassified Sitaravirus
GCA_003364395.1	38518	66	Paenibacillus phage Arcticfreeze	NA	Viruses; Duplodnaviria; Heunggongvirae; Uroviricota; Caudoviricetes; Caudovirales; Siphoviridae; unclassified Siphoviridae
GCA_003368945.1	55774	87	Paenibacillus phage C7Cdelta	NA	Viruses; Duplodnaviria; Heunggongvirae; Uroviricota; Caudoviricetes; Caudovirales; Siphoviridae; Trippvirus; unclassified Trippvirus
GCA_003364415.1	38520	66	Paenibacillus phage DevRi	NA	Viruses; Duplodnaviria; Heunggongvirae; Uroviricota; Caudoviricetes; Caudovirales; Siphoviridae; unclassified Siphoviridae
GCA_003369365.1	38541	66	Paenibacillus phage Gryphonian	NA	Viruses; Duplodnaviria; Heunggongvirae; Uroviricota; Caudoviricetes; Caudovirales; Siphoviridae; unclassified Siphoviridae
GCA_003368885.1	40054	66	Paenibacillus phage Honeybear	NA	Viruses; Duplodnaviria; Heunggongvirae; Uroviricota; Caudoviricetes; Caudovirales; Siphoviridae; Sitaravirus; unclassified Sitaravirus
GCA_003368905.1	40769	71	Paenibacillus phage Kawika	NA	Viruses; Duplodnaviria; Heunggongvirae; Uroviricota; Caudoviricetes; Caudovirales; Siphoviridae; Sitaravirus; unclassified Sitaravirus
GCA_003368925.1	37947	65	Paenibacillus phage Lucielle	NA	Viruses; Duplodnaviria; Heunggongvirae; Uroviricota; Caudoviricetes; Caudovirales; Siphoviridae; Sitaravirus; unclassified Sitaravirus
GCA_003368845.1	40448	72	Paenibacillus phage Wanderer	NA	Viruses; Duplodnaviria; Heunggongvirae; Uroviricota; Caudoviricetes; Caudovirales; Siphoviridae; Vegasvirus; unclassified Vegasvirus
GCA_003368865.1	43126	69	Paenibacillus phage Yerffej	NA	Viruses; Duplodnaviria; Heunggongvirae; Uroviricota; Caudoviricetes; Caudovirales; Siphoviridae; unclassified Siphoviridae
GCA_004134085.1	2394	4	Ashy storm petrel gyrovirus	NA	Viruses; Anelloviridae; Gyrovirus; unclassified Gyrovirus
GCA_003848385.1	2191	3	Gila monsterassociated gemycircularvirus	NA	Viruses; Monodnaviria; Shotokuvirae; Cressdnaviricota; Repensiviricetes; Geplafuvirales; Genomoviridae; Gemycircularvirus; unclassified Gemycircularvirus
GCA_018583505.1	2028	3	Torque teno Arctocephalus gazella virus 1	NA	Viruses; Anelloviridae; Sigmatorquevirus; Torque teno pinniped virus 6
GCA_018583515.1	2127	3	Torque teno Arctocephalus gazella virus 2	NA	Viruses; Anelloviridae; Sigmatorquevirus; Torque teno pinniped virus 7
GCA_004041515.1	2046	2	Feline stoolassociated circular virus KU14	NA	Viruses; Monodnaviria; Shotokuvirae; Cressdnaviricota; Arfiviricetes; Cirlivirales; Circoviridae; Circovirus; unclassified Circovirus
GCA_003345025.1	164715	260	Escherichia virus KFSEC	NA	Viruses; Duplodnaviria; Heunggongvirae; Uroviricota; Caudoviricetes; Caudovirales; Myoviridae; Tevenvirinae; Krischvirus; unclassified Krischvirus
GCA_004117575.1	25028	9	Chiqui virus	CoB 38d	Viruses; Riboviria; Orthornavirae; Duplornaviricota; Resentoviricetes; Reovirales; Reoviridae; Spinareovirinae; unclassified Spinareovirinae
GCA_003365255.1	50961	75	Gordonia phage DelRio	NA	Viruses; Duplodnaviria; Heunggongvirae; Uroviricota; Caudoviricetes; Caudovirales; Siphoviridae; Betterkatzvirus; unclassified Betterkatzvirus
GCA_003365275.1	40833	69	Microbacterium phage Eden	NA	Viruses; Duplodnaviria; Heunggongvirae; Uroviricota; Caudoviricetes; Caudovirales; Siphoviridae; Edenvirus; Microbacterium virus Eden
GCA_003365155.1	52668	92	Mycobacterium phage Acolyte	NA	Viruses; Duplodnaviria; Heunggongvirae; Uroviricota; Caudoviricetes; Caudovirales; Siphoviridae; Fromanvirus; unclassified Fromanvirus
GCA_003365175.1	60430	105	Mycobacterium phage Aminay	NA	Viruses; Duplodnaviria; Heunggongvirae; Uroviricota; Caudoviricetes; Caudovirales; Siphoviridae; Timquatrovirus; unclassified Timquatrovirus
GCA_003365195.1	51366	88	Mycobacterium phage Avle17	NA	Viruses; Duplodnaviria; Heunggongvirae; Uroviricota; Caudoviricetes; Caudovirales; Siphoviridae; Fromanvirus; unclassified Fromanvirus

gca	genome.size	proteome.size	phage.name	strain	Taxonomy
GCA_003365215.1	59906	95	Mycobacterium phage BEEST	NA	Viruses; Duplodnaviria; Heunggongvirae; Uroviricota; Caudoviricetes; Caudovirales; Siphoviridae; Timquatrovirus; unclassified Timquatrovirus
GCA_003365235.1	68508	94	Mycobacterium phage Cborch11	NA	Viruses; Duplodnaviria; Heunggongvirae; Uroviricota; Caudoviricetes; Caudovirales; Siphoviridae; unclassified Siphoviridae
GCA_003365375.1	52619	97	Gordonia phage LastResort	NA	Viruses; Duplodnaviria; Heunggongvirae; Uroviricota; Caudoviricetes; Caudovirales; Siphoviridae; Soupsvirus; unclassified Soupsvirus
GCA_003365475.1	52488	82	Gordonia phage Pollux	NA	Viruses; Duplodnaviria; Heunggongvirae; Uroviricota; Caudoviricetes; Caudovirales; Siphoviridae; unclassified Siphoviridae
GCA_003365515.1	41573	62	Microbacterium phage Papafritta	NA	Viruses; Duplodnaviria; Heunggongvirae; Uroviricota; Caudoviricetes; Caudovirales; Siphoviridae; Ilzatvirus; unclassified Ilzatvirus
GCA_003365535.1	40306	62	Microbacterium phage Sansa	NA	Viruses; Duplodnaviria; Heunggongvirae; Uroviricota; Caudoviricetes; Caudovirales; Siphoviridae; Elerivirus; unclassified Elerivirus
GCA_003365295.1	71376	97	Mycobacterium phage Hangman	NA	Viruses; Duplodnaviria; Heunggongvirae; Uroviricota; Caudoviricetes; Caudovirales; Siphoviridae; Bclasvirinae; Coopervirus; unclassified Coopervirus
GCA_003365315.1	50455	88	Mycobacterium phage Hope4ever	NA	Viruses; Duplodnaviria; Heunggongvirae; Uroviricota; Caudoviricetes; Caudovirales; Siphoviridae; Fromanvirus; unclassified Fromanvirus
GCA_003365335.1	75668	149	Mycobacterium phage IHOP	NA	Viruses; Duplodnaviria; Heunggongvirae; Uroviricota; Caudoviricetes; Caudovirales; Siphoviridae; Kostyavirus; unclassified Kostyavirus
GCA_003365355.1	68822	103	Mycobacterium phage Kwksand96	NA	Viruses; Duplodnaviria; Heunggongvirae; Uroviricota; Caudoviricetes; Caudovirales; Siphoviridae; Bclasvirinae; Pegunavirus; unclassified Pegunavirus
GCA_003365395.1	48504	86	Mycobacterium phage Misomonster	NA	Viruses; Duplodnaviria; Heunggongvirae; Uroviricota; Caudoviricetes; Caudovirales; Siphoviridae; Fromanvirus; unclassified Fromanvirus
GCA_003365415.1	69431	101	Mycobacterium phage Morty007	NA	Viruses; Duplodnaviria; Heunggongvirae; Uroviricota; Caudoviricetes; Caudovirales; Siphoviridae; Bclasvirinae; Pipefishvirus; unclassified Pipefishvirus
GCA_003365435.1	60055	96	Mycobacterium phage Mynx	NA	Viruses; Duplodnaviria; Heunggongvirae; Uroviricota; Caudoviricetes; Caudovirales; Siphoviridae; Timquatrovirus; unclassified Timquatrovirus
GCA_003365455.1	75685	149	Mycobacterium phage Phaja	NA	Viruses; Duplodnaviria; Heunggongvirae; Uroviricota; Caudoviricetes; Caudovirales; Siphoviridae; Kostyavirus; unclassified Kostyavirus
GCA_003365495.1	68056	99	Mycobacterium phage Roy17	NA	Viruses; Duplodnaviria; Heunggongvirae; Uroviricota; Caudoviricetes; Caudovirales; Siphoviridae; Bclasvirinae; Pegunavirus; unclassified Pegunavirus
GCA_003365555.1	76133	143	Mycobacterium phage ShereKhan	NA	Viruses; Duplodnaviria; Heunggongvirae; Uroviricota; Caudoviricetes; Caudovirales; Siphoviridae; Kostyavirus; unclassified Kostyavirus
GCA_003365575.1	53420	90	Mycobacterium phage Steamy	NA	Viruses; Duplodnaviria; Heunggongvirae; Uroviricota; Caudoviricetes; Caudovirales; Siphoviridae; Fromanvirus; unclassified Fromanvirus
GCA_003365595.1	52876	93	Mycobacterium phage Tomathan	NA	Viruses; Duplodnaviria; Heunggongvirae; Uroviricota; Caudoviricetes; Caudovirales; Siphoviridae; Fromanvirus; unclassified Fromanvirus
GCA_007859095.1	53343	87	Proteus phage vB_PmiP_RS10pmA	NA	Viruses; Duplodnaviria; Heunggongvirae; Uroviricota; Caudoviricetes; Caudovirales; Siphoviridae; Gorganvirus; unclassified Gorganvirus
GCA_006964805.1	42112	53	Proteus phage vB_PmiP_RS1pmA	NA	Viruses; Duplodnaviria; Heunggongvirae; Uroviricota; Caudoviricetes; Caudovirales; Autographiviridae; Slopekvirinae; Novosibovirus; unclassified Novosibovirus
GCA_006964845.1	42396	83	Proteus phage vB_PmiP_RS51pmB	NA	Viruses; Duplodnaviria; Heunggongvirae; Uroviricota; Caudoviricetes; Caudovirales; Myoviridae; unclassified Myoviridae
GCA_006964825.1	42696	54	Proteus phage vB_PmiP_RS8pmA	NA	Viruses; Duplodnaviria; Heunggongvirae; Uroviricota; Caudoviricetes; Caudovirales; Autographiviridae; Slopekvirinae; Novosibovirus; unclassified Novosibovirus
GCA_003365615.1	52195	101	Gordonia phage Apricot	NA	Viruses; Duplodnaviria; Heunggongvirae; Uroviricota; Caudoviricetes; Caudovirales; Siphoviridae; Apricotvirus; Gordonia virus Apricot
GCA_003365735.1	52531	84	Gordonia phage Frokostdame	NA	Viruses; Duplodnaviria; Heunggongvirae; Uroviricota; Caudoviricetes; Caudovirales; Siphoviridae; unclassified Siphoviridae
GCA_003365755.1	64104	96	Gordonia phage Fury	NA	Viruses; Duplodnaviria; Heunggongvirae; Uroviricota; Caudoviricetes; Caudovirales; Siphoviridae; unclassified Siphoviridae
GCA_003365855.1	67081	93	Gordonia phage NatB6	NA	Viruses; Duplodnaviria; Heunggongvirae; Uroviricota; Caudoviricetes; Caudovirales; Siphoviridae; unclassified Siphoviridae
GCA_003365895.1	51265	73	Gordonia phage Ruthy	NA	Viruses; Duplodnaviria; Heunggongvirae; Uroviricota; Caudoviricetes; Caudovirales; Siphoviridae; Ruthyvirus; Gordonia virus Ruthy
GCA_003365835.1	63604	119	Microbacterium phage Musetta	NA	Viruses; Duplodnaviria; Heunggongvirae; Uroviricota; Caudoviricetes; Caudovirales; Siphoviridae; unclassified Siphoviridae
GCA_003365635.1	48545	87	Mycobacterium phage AugsMagnumOpus	NA	Viruses; Duplodnaviria; Heunggongvirae; Uroviricota; Caudoviricetes; Caudovirales; Siphoviridae; Fromanvirus; unclassified Fromanvirus
GCA_003365695.1	50155	85	Mycobacterium phage DrFeelGood	NA	Viruses; Duplodnaviria; Heunggongvirae; Uroviricota; Caudoviricetes; Caudovirales; Siphoviridae; Fromanvirus; unclassified Fromanvirus
GCA_003365715.1	51343	87	Mycobacterium phage Druantia	NA	Viruses; Duplodnaviria; Heunggongvirae; Uroviricota; Caudoviricetes; Caudovirales; Siphoviridae; Fromanvirus; unclassified Fromanvirus
GCA_003365775.1	76498	148	Mycobacterium phage Glexan	NA	Viruses; Duplodnaviria; Heunggongvirae; Uroviricota; Caudoviricetes; Caudovirales; Siphoviridae; Kostyavirus; unclassified Kostyavirus
GCA_003365795.1	59708	94	Mycobacterium phage Homura	NA	Viruses; Duplodnaviria; Heunggongvirae; Uroviricota; Caudoviricetes; Caudovirales; Siphoviridae; Timquatrovirus; unclassified Timquatrovirus
GCA_003365815.1	59837	97	Mycobacterium phage Joy99	NA	Viruses; Duplodnaviria; Heunggongvirae; Uroviricota; Caudoviricetes; Caudovirales; Siphoviridae; Timquatrovirus; unclassified Timquatrovirus

gca	genome.size	proteome.size	phage.name	strain	Taxonomy
GCA_003365875.1	50721	90	Mycobacterium phage Ollie	NA	Viruses; Duplodnaviria; Heunggongvirae; Uroviricota; Caudoviricetes; Caudovirales; Siphoviridae; Fromanvirus; unclassified Fromanvirus
GCA_003365915.1	75366	145	Mycobacterium phage Simpliphy	NA	Viruses; Duplodnaviria; Heunggongvirae; Uroviricota; Caudoviricetes; Caudovirales; Siphoviridae; Kostyavirus; unclassified Kostyavirus
GCA_003365935.1	52138	99	Mycobacterium phage Snape	NA	Viruses; Duplodnaviria; Heunggongvirae; Uroviricota; Caudoviricetes; Caudovirales; Siphoviridae; Fromanvirus; unclassified Fromanvirus
GCA_003365955.1	75359	149	Mycobacterium phage TBrady12	NA	Viruses; Duplodnaviria; Heunggongvirae; Uroviricota; Caudoviricetes; Caudovirales; Siphoviridae; Kostyavirus; unclassified Kostyavirus
GCA_003354185.1	125726	229	Streptomyces phage Annadreamy	NA	Viruses; Duplodnaviria; Heunggongvirae; Uroviricota; Caudoviricetes; Caudovirales; Siphoviridae; unclassified Siphoviridae
GCA_003365655.1	130473	240	Streptomyces phage Blueeyedbeauty	NA	Viruses; Duplodnaviria; Heunggongvirae; Uroviricota; Caudoviricetes; Caudovirales; Siphoviridae; unclassified Siphoviridae
GCA_003365675.1	54036	82	Streptomyces phage Crosby	NA	Viruses; Duplodnaviria; Heunggongvirae; Uroviricota; Caudoviricetes; Caudovirales; Siphoviridae; unclassified Siphoviridae
GCA_003341395.1	62090	117	Microbacterium phage Fork	NA	Viruses; Duplodnaviria; Heunggongvirae; Uroviricota; Caudoviricetes; Caudovirales; Siphoviridae; unclassified Siphoviridae
GCA_003341215.1	17455	25	Microbacterium phage KayPaulus	NA	Viruses; Duplodnaviria; Heunggongvirae; Uroviricota; Caudoviricetes; Caudovirales; Siphoviridae; unclassified Siphoviridae
GCA_003341375.1	62716	122	Microbacterium phage Lyell	NA	Viruses; Duplodnaviria; Heunggongvirae; Uroviricota; Caudoviricetes; Caudovirales; Siphoviridae; unclassified Siphoviridae
GCA_003341335.1	17450	25	Microbacterium phage Quaker	NA	Viruses; Duplodnaviria; Heunggongvirae; Uroviricota; Caudoviricetes; Caudovirales; Siphoviridae; unclassified Siphoviridae
GCA_003341095.1	17534	25	Microbacterium phage VitulaEligans	NA	Viruses; Duplodnaviria; Heunggongvirae; Uroviricota; Caudoviricetes; Caudovirales; Siphoviridae; unclassified Siphoviridae
GCA_003341315.1	75208	148	Mycobacterium phage Adnama	NA	Viruses; Duplodnaviria; Heunggongvirae; Uroviricota; Caudoviricetes; Caudovirales; Siphoviridae; Kostyavirus; unclassified Kostyavirus
GCA_003342475.1	60494	99	Mycobacterium phage Beezoo	NA	Viruses; Duplodnaviria; Heunggongvirae; Uroviricota; Caudoviricetes; Caudovirales; Siphoviridae; Timquatrovirus; unclassified Timquatrovirus
GCA_003341295.1	69044	102	Mycobacterium phage Childish	NA	Viruses; Duplodnaviria; Heunggongvirae; Uroviricota; Caudoviricetes; Caudovirales; Siphoviridae; Bclasvirinae; Pegunavirus; unclassified Pegunavirus
GCA_003341275.1	55727	96	Mycobacterium phage Clifton	NA	Viruses; Duplodnaviria; Heunggongvirae; Uroviricota; Caudoviricetes; Caudovirales; Siphoviridae; Cheoctovirus; unclassified Cheoctovirus
GCA_003341255.1	41880	61	Mycobacterium phage DMoney	NA	Viruses; Duplodnaviria; Heunggongvirae; Uroviricota; Caudoviricetes; Caudovirales; Siphoviridae; Liefievirus; unclassified Liefievirus
GCA_003341415.1	68243	98	Mycobacterium phage Doddsville	NA	Viruses; Duplodnaviria; Heunggongvirae; Uroviricota; Caudoviricetes; Caudovirales; Siphoviridae; Bclasvirinae; Pegunavirus; unclassified Pegunavirus
GCA_003341235.1	69031	102	Mycobacterium phage Kahve	NA	Viruses; Duplodnaviria; Heunggongvirae; Uroviricota; Caudoviricetes; Caudovirales; Siphoviridae; Bclasvirinae; Pegunavirus; unclassified Pegunavirus
GCA_003341355.1	51428	91	Mycobacterium phage Magnar	NA	Viruses; Duplodnaviria; Heunggongvirae; Uroviricota; Caudoviricetes; Caudovirales; Siphoviridae; Fromanvirus; unclassified Fromanvirus
GCA_003341075.1	68878	102	Mycobacterium phage Morty	NA	Viruses; Duplodnaviria; Heunggongvirae; Uroviricota; Caudoviricetes; Caudovirales; Siphoviridae; Bclasvirinae; Pegunavirus; unclassified Pegunavirus
GCA_003341195.1	41880	62	Mycobacterium phage OctaviousRex	NA	Viruses; Duplodnaviria; Heunggongvirae; Uroviricota; Caudoviricetes; Caudovirales; Siphoviridae; Liefievirus; unclassified Liefievirus
GCA_003341175.1	58297	107	Mycobacterium phage OlympiaSaint	NA	Viruses; Duplodnaviria; Heunggongvirae; Uroviricota; Caudoviricetes; Caudovirales; Siphoviridae; Cheoctovirus; unclassified Cheoctovirus
GCA_003341155.1	48024	81	Mycobacterium phage Phranny	NA	Viruses; Duplodnaviria; Heunggongvirae; Uroviricota; Caudoviricetes; Caudovirales; Siphoviridae; Fromanvirus; unclassified Fromanvirus
GCA_003341135.1	52949	96	Mycobacterium phage Priya	NA	Viruses; Duplodnaviria; Heunggongvirae; Uroviricota; Caudoviricetes; Caudovirales; Siphoviridae; Fromanvirus; unclassified Fromanvirus
GCA_003341115.1	59540	104	Mycobacterium phage Sandalphon	NA	Viruses; Duplodnaviria; Heunggongvirae; Uroviricota; Caudoviricetes; Caudovirales; Siphoviridae; Cheoctovirus; unclassified Cheoctovirus
GCA_003341795.1	58120	85	Arthrobacter phage Tatanka	NA	Viruses; Duplodnaviria; Heunggongvirae; Uroviricota; Caudoviricetes; Caudovirales; Siphoviridae; Gordonvirus; unclassified Gordonvirus
GCA_003341655.1	49897	74	Gordonia phage Nadeem	NA	Viruses; Duplodnaviria; Heunggongvirae; Uroviricota; Caudoviricetes; Caudovirales; Siphoviridae; Betterkatzvirus; unclassified Betterkatzvirus
GCA_003341615.1	61421	114	Microbacterium phage Jacko	NA	Viruses; Duplodnaviria; Heunggongvirae; Uroviricota; Caudoviricetes; Caudovirales; Siphoviridae; unclassified Siphoviridae
GCA_003341695.1	17349	25	Microbacterium phage Noelani	NA	Viruses; Duplodnaviria; Heunggongvirae; Uroviricota; Caudoviricetes; Caudovirales; Siphoviridae; unclassified Siphoviridae
GCA_003341495.1	56454	77	Mycobacterium phage Biglebops	NA	Viruses; Duplodnaviria; Heunggongvirae; Uroviricota; Caudoviricetes; Caudovirales; Siphoviridae; Timquatrovirus; unclassified Timquatrovirus
GCA_003341515.1	53123	95	Mycobacterium phage ByChance	NA	Viruses; Duplodnaviria; Heunggongvirae; Uroviricota; Caudoviricetes; Caudovirales; Siphoviridae; Cheoctovirus; unclassified Cheoctovirus
GCA_003345045.1	110169	228	Mycobacterium phage Constella	NA	Viruses; Duplodnaviria; Heunggongvirae; Uroviricota; Caudoviricetes; Caudovirales; Siphoviridae; Omegavirus; unclassified Omegavirus
GCA_003341535.1	69263	103	Mycobacterium phage Craff	NA	Viruses; Duplodnaviria; Heunggongvirae; Uroviricota; Caudoviricetes; Caudovirales; Siphoviridae; Bclasvirinae; Pegunavirus; unclassified Pegunavirus

gca	genome.size	proteome.size	phage.name	strain	Taxonomy
GCA_003341555.1	75070	143	Mycobacterium phage DoctorDiddles	NA	Viruses; Duplodnaviria; Heunggongvirae; Uroviricota; Caudoviricetes; Caudovirales; Siphoviridae; Kostyavirus; unclassified Kostyavirus
GCA_003342495.1	68866	103	Mycobacterium phage Gareth	NA	Viruses; Duplodnaviria; Heunggongvirae; Uroviricota; Caudoviricetes; Caudovirales; Siphoviridae; Bclasvirinae; Pegunavirus; unclassified Pegunavirus
GCA_003341575.1	50883	90	Mycobacterium phage Grum1	NA	Viruses; Duplodnaviria; Heunggongvirae; Uroviricota; Caudoviricetes; Caudovirales; Siphoviridae; Fromanvirus; unclassified Fromanvirus
GCA_003345065.1	51376	89	Mycobacterium phage Houdini22	NA	Viruses; Duplodnaviria; Heunggongvirae; Uroviricota; Caudoviricetes; Caudovirales; Siphoviridae; Fromanvirus; unclassified Fromanvirus
GCA_003341595.1	74169	144	Mycobacterium phage Icee	NA	Viruses; Duplodnaviria; Heunggongvirae; Uroviricota; Caudoviricetes; Caudovirales; Siphoviridae; Kostyavirus; unclassified Kostyavirus
GCA_003341635.1	68494	103	Mycobacterium phage Mutante	NA	Viruses; Duplodnaviria; Heunggongvirae; Uroviricota; Caudoviricetes; Caudovirales; Siphoviridae; Bclasvirinae; Pegunavirus; unclassified Pegunavirus
GCA_003341675.1	51672	89	Mycobacterium phage Niza	NA	Viruses; Duplodnaviria; Heunggongvirae; Uroviricota; Caudoviricetes; Caudovirales; Siphoviridae; Fromanvirus; unclassified Fromanvirus
GCA_003341715.1	58102	102	Mycobacterium phage NormanBulbieJr	NA	Viruses; Duplodnaviria; Heunggongvirae; Uroviricota; Caudoviricetes; Caudovirales; Siphoviridae; Cheoctovirus; unclassified Cheoctovirus
GCA_003345085.1	51376	89	Mycobacterium phage NotAPhaseMom	NA	Viruses; Duplodnaviria; Heunggongvirae; Uroviricota; Caudoviricetes; Caudovirales; Siphoviridae; Fromanvirus; unclassified Fromanvirus
GCA_003341735.1	68873	105	Mycobacterium phage PhrodoBaggins	NA	Viruses; Duplodnaviria; Heunggongvirae; Uroviricota; Caudoviricetes; Caudovirales; Siphoviridae; Bclasvirinae; Pegunavirus; unclassified Pegunavirus
GCA_003341755.1	41491	66	Mycobacterium phage Rubeelu	NA	Viruses; Duplodnaviria; Heunggongvirae; Uroviricota; Caudoviricetes; Caudovirales; Siphoviridae; Nclasvirinae; Buttersvirus; unclassified Buttersvirus
GCA_003341775.1	64570	89	Mycobacterium phage Visconti	NA	Viruses; Duplodnaviria; Heunggongvirae; Uroviricota; Caudoviricetes; Caudovirales; Siphoviridae; Dclasvirinae; Plotvirus; Mycobacterium virus Plot
GCA_003365135.1	57542	84	Arthrobacter phage Synepsis	NA	Viruses; Duplodnaviria; Heunggongvirae; Uroviricota; Caudoviricetes; Caudovirales; Siphoviridae; Gordonvirus; unclassified Gordonvirus
GCA_003364875.1	58695	95	Gordonia phage Danyall	NA	Viruses; Duplodnaviria; Heunggongvirae; Uroviricota; Caudoviricetes; Caudovirales; Siphoviridae; Wizardvirus; unclassified Wizardvirus
GCA_007647155.1	53407	82	Gordonia phage Eviarto	NA	Viruses; Duplodnaviria; Heunggongvirae; Uroviricota; Caudoviricetes; Caudovirales; Siphoviridae; Nymbaxtervirinae; Nymphadoravirus; unclassified Nymphadoravirus
GCA_003423245.1	67234	144	Gordonia phage Fryberger	NA	Viruses; Duplodnaviria; Heunggongvirae; Uroviricota; Caudoviricetes; Caudovirales; Siphoviridae; Ronaldovirus; Gordonia virus Fryberger
GCA_003364935.1	46728	73	Gordonia phage GEazy	NA	Viruses; Duplodnaviria; Heunggongvirae; Uroviricota; Caudoviricetes; Caudovirales; Siphoviridae; Bowservirus; unclassified Bowservirus
GCA_003364975.1	58755	92	Gordonia phage KimmyK	NA	Viruses; Duplodnaviria; Heunggongvirae; Uroviricota; Caudoviricetes; Caudovirales; Siphoviridae; Wizardvirus; unclassified Wizardvirus
GCA_003365095.1	81834	124	Gordonia phage RobinSparkles	NA	Viruses; Duplodnaviria; Heunggongvirae; Uroviricota; Caudoviricetes; Caudovirales; Siphoviridae; Orchidvirus; unclassified Orchidvirus
GCA_003365115.1	58767	85	Gordonia phage Rofo	NA	Viruses; Duplodnaviria; Heunggongvirae; Uroviricota; Caudoviricetes; Caudovirales; Siphoviridae; Vividuovirus; unclassified Vividuovirus
GCA_003423265.1	68389	147	Gordonia phage Ronaldo	NA	Viruses; Duplodnaviria; Heunggongvirae; Uroviricota; Caudoviricetes; Caudovirales; Siphoviridae; Ronaldovirus; Gordonia virus Ronaldo
GCA_007647185.1	53431	82	Gordonia phage TimTam	NA	Viruses; Duplodnaviria; Heunggongvirae; Uroviricota; Caudoviricetes; Caudovirales; Siphoviridae; Nymbaxtervirinae; Nymphadoravirus; unclassified Nymphadoravirus
GCA_003365075.1	41930	63	Microbacterium phage Redfield	NA	Viruses; Duplodnaviria; Heunggongvirae; Uroviricota; Caudoviricetes; Caudovirales; Siphoviridae; Ilzatvirus; unclassified Ilzatvirus
GCA_003364855.1	51369	86	Mycobacterium phage Commander	NA	Viruses; Duplodnaviria; Heunggongvirae; Uroviricota; Caudoviricetes; Caudovirales; Siphoviridae; Fromanvirus; unclassified Fromanvirus
GCA_003364895.1	51366	89	Mycobacterium phage Eapen	NA	Viruses; Duplodnaviria; Heunggongvirae; Uroviricota; Caudoviricetes; Caudovirales; Siphoviridae; Fromanvirus; unclassified Fromanvirus
GCA_003364915.1	69476	102	Mycobacterium phage FugateOSU	NA	Viruses; Duplodnaviria; Heunggongvirae; Uroviricota; Caudoviricetes; Caudovirales; Siphoviridae; Bclasvirinae; Pegunavirus; unclassified Pegunavirus
GCA_003364955.1	68547	100	Mycobacterium phage GeneCoco	NA	Viruses; Duplodnaviria; Heunggongvirae; Uroviricota; Caudoviricetes; Caudovirales; Siphoviridae; Bclasvirinae; Pegunavirus; unclassified Pegunavirus
GCA_003364995.1	68637	102	Mycobacterium phage Labeouficaum	NA	Viruses; Duplodnaviria; Heunggongvirae; Uroviricota; Caudoviricetes; Caudovirales; Siphoviridae; Bclasvirinae; Pegunavirus; unclassified Pegunavirus
GCA_003365015.1	52695	95	Mycobacterium phage Moose	NA	Viruses; Duplodnaviria; Heunggongvirae; Uroviricota; Caudoviricetes; Caudovirales; Siphoviridae; Fromanvirus; unclassified Fromanvirus
GCA_003365035.1	41902	63	Mycobacterium phage Mowgli	NA	Viruses; Duplodnaviria; Heunggongvirae; Uroviricota; Caudoviricetes; Caudovirales; Siphoviridae; Liefievirus; unclassified Liefievirus
GCA_003365055.1	69085	99	Mycobacterium phage Placalicious	NA	Viruses; Duplodnaviria; Heunggongvirae; Uroviricota; Caudoviricetes; Caudovirales; Siphoviridae; Bclasvirinae; Pegunavirus; unclassified Pegunavirus
GCA_003364455.1	45418	56	Arthrobacter phage Brad	NA	Viruses; Duplodnaviria; Heunggongvirae; Uroviricota; Caudoviricetes; Caudovirales; Podoviridae; Jasminevirus; unclassified Jasminevirus
GCA_003364475.1	57822	84	Arthrobacter phage Breylor17	NA	Viruses; Duplodnaviria; Heunggongvirae; Uroviricota; Caudoviricetes; Caudovirales; Siphoviridae; Gordonvirus; unclassified Gordonvirus
GCA_003364515.1	43950	60	Arthrobacter phage Daiboju	NA	Viruses; Duplodnaviria; Heunggongvirae; Uroviricota; Caudoviricetes; Caudovirales; Siphoviridae; Korravirus; unclassified Korravirus

gca	genome.size	proteome.size	phage.name	strain	Taxonomy
GCA_003364535.1	43949	60	Arthrobacter phage Herb	NA	Viruses; Duplodnaviria; Heunggongvirae; Uroviricota; Caudoviricetes; Caudovirales; Siphoviridae; Korravirus; unclassified Korravirus
GCA_003364575.1	57847	95	Arthrobacter phage KeaneyLin	NA	Viruses; Duplodnaviria; Heunggongvirae; Uroviricota; Caudoviricetes; Caudovirales; Siphoviridae; Mudcatvirus; unclassified Mudcatvirus
GCA_003364595.1	43950	60	Arthrobacter phage KingBob	NA	Viruses; Duplodnaviria; Heunggongvirae; Uroviricota; Caudoviricetes; Caudovirales; Siphoviridae; Korravirus; unclassified Korravirus
GCA_003364635.1	39448	72	Arthrobacter phage MargaretKali	NA	Viruses; Duplodnaviria; Heunggongvirae; Uroviricota; Caudoviricetes; Caudovirales; Siphoviridae; unclassified Siphoviridae
GCA_003364675.1	56978	85	Arthrobacter phage MediumFry	NA	Viruses; Duplodnaviria; Heunggongvirae; Uroviricota; Caudoviricetes; Caudovirales; Siphoviridae; Gordonvirus; unclassified Gordonvirus
GCA_003364795.1	43950	60	Arthrobacter phage Sergei	NA	Viruses; Duplodnaviria; Heunggongvirae; Uroviricota; Caudoviricetes; Caudovirales; Siphoviridae; Korravirus; unclassified Korravirus
GCA_003364755.1	57448	85	Gordonia phage Ribeye	NA	Viruses; Duplodnaviria; Heunggongvirae; Uroviricota; Caudoviricetes; Caudovirales; Siphoviridae; Vividuovirus; unclassified Vividuovirus
GCA_003364815.1	45847	67	Gordonia phage SketchMex	NA	Viruses; Duplodnaviria; Heunggongvirae; Uroviricota; Caudoviricetes; Caudovirales; Siphoviridae; Emalynvirus; unclassified Emalynvirus
GCA_003364435.1	52632	91	Mycobacterium phage BigMau	NA	Viruses; Duplodnaviria; Heunggongvirae; Uroviricota; Caudoviricetes; Caudovirales; Siphoviridae; Fromanvirus; unclassified Fromanvirus
GCA_003364495.1	69174	100	Mycobacterium phage Buckeye	NA	Viruses; Duplodnaviria; Heunggongvirae; Uroviricota; Caudoviricetes; Caudovirales; Siphoviridae; Bclasvirinae; Pegunavirus; unclassified Pegunavirus
GCA_003364555.1	50571	89	Mycobacterium phage Kalnoky	NA	Viruses; Duplodnaviria; Heunggongvirae; Uroviricota; Caudoviricetes; Caudovirales; Siphoviridae; Fromanvirus; unclassified Fromanvirus
GCA_003364615.1	64827	92	Mycobacterium phage KingTut	NA	Viruses; Duplodnaviria; Heunggongvirae; Uroviricota; Caudoviricetes; Caudovirales; Siphoviridae; Bclasvirinae; Pegunavirus; unclassified Pegunavirus
GCA_003364655.1	50570	89	Mycobacterium phage Marius	NA	Viruses; Duplodnaviria; Heunggongvirae; Uroviricota; Caudoviricetes; Caudovirales; Siphoviridae; Fromanvirus; unclassified Fromanvirus
GCA_003364695.1	49756	85	Mycobacterium phage PhishRPhriends	NA	Viruses; Duplodnaviria; Heunggongvirae; Uroviricota; Caudoviricetes; Caudovirales; Siphoviridae; Fromanvirus; unclassified Fromanvirus
GCA_003364715.1	41722	63	Mycobacterium phage Plagueis	NA	Viruses; Duplodnaviria; Heunggongvirae; Uroviricota; Caudoviricetes; Caudovirales; Siphoviridae; Liefievirus; unclassified Liefievirus
GCA_003364735.1	49412	84	Mycobacterium phage Reba	NA	Viruses; Duplodnaviria; Heunggongvirae; Uroviricota; Caudoviricetes; Caudovirales; Siphoviridae; Fromanvirus; unclassified Fromanvirus
GCA_003364775.1	53483	92	Mycobacterium phage Rohr	NA	Viruses; Duplodnaviria; Heunggongvirae; Uroviricota; Caudoviricetes; Caudovirales; Siphoviridae; Fromanvirus; unclassified Fromanvirus
GCA_003364835.1	51822	95	Mycobacterium phage SwissCheese	NA	Viruses; Duplodnaviria; Heunggongvirae; Uroviricota; Caudoviricetes; Caudovirales; Siphoviridae; Fromanvirus; unclassified Fromanvirus
GCA_004117595.1	3460	2	Aspergillus fumigatus partitivirus 2	NA	Viruses; Riboviria; Orthornavirae; Pisuviricota; Duplopiviricetes; Durnavirales; Partitiviridae; unclassified Partitiviridae
GCA_004127995.1	7497	4	Aspergillus fumigatus polymycovirus 1	NA	Viruses; Riboviria; Polymycoviridae; Polymycovirus
GCA_002601265.1	4554	8	Chlamydia phage 3	NA	Viruses; Monodnaviria; Sangervirae; Phixviricota; Malgrandaviricetes; Petitvirales; Microviridae; Gokushovirinae; Chlamydiamicrovirus; Chlamydia virus Chp2
GCA_000843085.1	41831	55	Streptomyces virus phiBT1	NA	Viruses; Duplodnaviria; Heunggongvirae; Uroviricota; Caudoviricetes; Caudovirales; Siphoviridae; Lomovskayavirus
GCA_003179815.1	7154	6	Human papillomavirus 157	NA	Viruses; Monodnaviria; Shotokuvirae; Cossaviricota; Papovaviricetes; Zurhausenvirales; Papillomaviridae; Firstpapillomavirinae; Gammapapillomavirus; Gammapapillomavirus 12
GCA_003177475.1	7779	7	Human papillomavirus 160	TM	Viruses; Monodnaviria; Shotokuvirae; Cossaviricota; Papovaviricetes; Zurhausenvirales; Papillomaviridae; Firstpapillomavirinae; Alphapapillomavirus; Alphapapillomavirus 2
GCA_003179835.1	7298	6	Human papillomavirus 205	NA	Viruses; Monodnaviria; Shotokuvirae; Cossaviricota; Papovaviricetes; Zurhausenvirales; Papillomaviridae; Firstpapillomavirinae; Gammapapillomavirus; Gammapapillomavirus 1
GCA_003179855.1	7192	6	Human papillomavirus 158	NA	Viruses; Monodnaviria; Shotokuvirae; Cossaviricota; Papovaviricetes; Zurhausenvirales; Papillomaviridae; Firstpapillomavirinae; Gammapapillomavirus; Gammapapillomavirus 12
GCA_000912015.1	7985	8	Mustela putorius papillomavirus 1	NA	Viruses; Monodnaviria; Shotokuvirae; Cossaviricota; Papovaviricetes; Zurhausenvirales; Papillomaviridae; Firstpapillomavirinae; Taupapillomavirus; Taupapillomavirus 4
GCA_006516695.1	37251	49	Pelagibacter phage HTVC025P	NA	Viruses; Duplodnaviria; Heunggongvirae; Uroviricota; Caudoviricetes; Caudovirales; Autographiviridae; unclassified Autographiviridae
GCA_003033235.1	5407	4	Feline bocaparvovirus 3	NA	Viruses; Monodnaviria; Shotokuvirae; Cossaviricota; Quintoviricetes; Piccovirales; Parvoviridae; Parvovirinae; Bocaparvovirus; Carnivore bocaparvovirus 5
GCA_001722885.1	5224	4	Mink bocavirus 1	NA	Viruses; Monodnaviria; Shotokuvirae; Cossaviricota; Quintoviricetes; Piccovirales; Parvoviridae; Parvovirinae; Bocaparvovirus; Carnivore bocaparvovirus 6
GCA_003033255.1	4995	4	Bat bocavirus WM40	NA	Viruses; Monodnaviria; Shotokuvirae; Cossaviricota; Quintoviricetes; Piccovirales; Parvoviridae; Parvovirinae; Bocaparvovirus; Chiropteran bocaparvovirus 2
GCA_003033265.1	4832	4	Bat bocavirus XM30	NA	Viruses; Monodnaviria; Shotokuvirae; Cossaviricota; Quintoviricetes; Piccovirales; Parvoviridae; Parvovirinae; Bocaparvovirus; Chiropteran bocaparvovirus 3
GCA_000919495.1	24043	11	Reptilian orthoreovirus	4702	Viruses; Riboviria; Orthornavirae; Duplornaviricota; Resentoviricetes; Reovirales; Reoviridae; Spinareovirinae; Orthoreovirus
GCA_002922425.1	171911	306	Citrobacter phage CF1 ERZ2017	NA	Viruses; Duplodnaviria; Heunggongvirae; Uroviricota; Caudoviricetes; Caudovirales; Myoviridae; Tevenvirinae; Moonvirus; Citrobacter virus CF1

gca	genome.size	proteome.size	phage.name	strain	Taxonomy
GCA_002621165.1	50339	87	Citrobacter phage CF1 DK2017	NA	Viruses; Duplodnaviria; Heunggongvirae; Uroviricota; Caudoviricetes; Caudovirales; Drexlerviridae; Tempevirinae; Tlsvirus; Citrobacter virus DK2017
GCA_003342335.1	41391	67	Salmonella phage S149	NA	Viruses; Duplodnaviria; Heunggongvirae; Uroviricota; Caudoviricetes; Caudovirales; Podoviridae; Lederbergvirus; unclassified Lederbergvirus
GCA_003441855.1	147890	195	Vibrio phage YC	NA	Viruses; Duplodnaviria; Heunggongvirae; Uroviricota; Caudoviricetes; Caudovirales; Ackermannviridae; unclassified Ackermannviridae
GCA_013086995.1	10454	4	Xapuri virus	LBCE 19881	Viruses; Riboviria; Orthornavirae; Negarnaviricota; Polyploviricotina; Ellioviricetes; Bunyavirales; Arenaviridae; Mammarenavirus; Xapuri mammarenavirus
GCA_003344985.1	244950	287	Erwinia phage Wellington	NA	Viruses; Duplodnaviria; Heunggongvirae; Uroviricota; Caudoviricetes; Caudovirales; Myoviridae; Wellingtonvirus; Erwinia virus Wellington
GCA_003423405.1	84559	119	Erwinia phage SunLIRen	NA	Viruses; Duplodnaviria; Heunggongvirae; Uroviricota; Caudoviricetes; Caudovirales; Myoviridae; Ounavirinae; Kolesnikvirus; unclassified Kolesnikvirus
GCA_003345005.1	61401	62	Erwinia phage Pavtok	NA	Viruses; Duplodnaviria; Heunggongvirae; Uroviricota; Caudoviricetes; Caudovirales; Podoviridae; unclassified Podoviridae
GCA_004134305.1	7107	1	Pinus nigra virus 1	NA	Viruses; Riboviria; Pararnavirae; Artverviricota; Revtraviricetes; Ortervirales; Caulimoviridae; unclassified Caulimoviridae
GCA_003656665.1	5269	9	Bacilladnavirus sp	NA	Viruses; Monodnaviria; Shotokuvirae; Cressdnaviricota; Arfiviricetes; Baphyvirales; Bacilladnaviridae; Bacilladnavirus; unclassified Bacilladnavirus
GCA_004134045.1	8809	7	Erethizon dorsatum papillomavirus 2	NA	Viruses; Monodnaviria; Shotokuvirae; Cossaviricota; Papovaviricetes; Zurhausenvirales; Papillomaviridae; unclassified Papillomaviridae; non-primate mammal papillomaviruses
GCA_004132405.1	15205	1	Phaseolus vulgaris endornavirus 3	NA	Viruses; Riboviria; Orthornavirae; Kitrinoviricota; Alsuviricetes; Martellivirales; Endornaviridae; Alphaendornavirus; Phaseolus vulgaris alphaendornavirus 3
GCA_003342575.1	39906	49	Klebsiella phage vB_KpnP_IME321	NA	Viruses; Duplodnaviria; Heunggongvirae; Uroviricota; Caudoviricetes; Caudovirales; Autographiviridae; Studiervirinae; Przondovirus; Klebsiella virus IME321
GCA_003443335.1	181082	226	Synechococcus phage ST4	NA	Viruses; Duplodnaviria; Heunggongvirae; Uroviricota; Caudoviricetes; Caudovirales; Myoviridae; Tamkungvirus; Synechococcus virus ST4
GCA_003341435.1	72821	98	Pseudomonas phage phCDa	NA	Viruses; Duplodnaviria; Heunggongvirae; Uroviricota; Caudoviricetes; Caudovirales; Podoviridae; Shizishanvirus; Pseudomonas virus phCDa
GCA_003341455.1	353081	515	Escherichia phage UB	NA	Viruses; Duplodnaviria; Heunggongvirae; Uroviricota; Caudoviricetes; Caudovirales; Myoviridae; Asteriusvirus; unclassified Asteriusvirus
GCA_003341035.2	39883	47	Escherichia phage SRT7	NA	Viruses; Duplodnaviria; Heunggongvirae; Uroviricota; Caudoviricetes; Caudovirales; Autographiviridae; Studiervirinae; Foetvirus; Escherichia virus SRT7
GCA_003341055.1	91364	170	Pseudomonas phage SRT6	NA	Viruses; Duplodnaviria; Heunggongvirae; Uroviricota; Caudoviricetes; Caudovirales; Myoviridae; Pakpunavirus; unclassified Pakpunavirus
GCA_003341475.1	38821	48	Salmonella phage 3A_8767	NA	Viruses; Duplodnaviria; Heunggongvirae; Uroviricota; Caudoviricetes; Caudovirales; Autographiviridae; Studiervirinae; Teseptimavirus; Salmonella virus 3A8767
GCA_003368685.1	43504	64	Staphylococcus phage SA1014ruMSSAST7	NA	Viruses; Duplodnaviria; Heunggongvirae; Uroviricota; Caudoviricetes; Caudovirales; Siphoviridae; Biseptimavirus; unclassified Biseptimavirus
GCA_003575805.1	43184	65	Staphylococcus phage SA780ruMSSAST101	NA	Viruses; Duplodnaviria; Heunggongvirae; Uroviricota; Caudoviricetes; Caudovirales; Siphoviridae; Biseptimavirus; unclassified Biseptimavirus
GCA_003368825.1	49292	71	Klebsiella phage NJS1	NA	Viruses; Duplodnaviria; Heunggongvirae; Uroviricota; Caudoviricetes; Caudovirales; Drexlerviridae; Webervirus; Klebsiella virus NJS1
GCA_003368785.1	42735	64	Staphylococcus phage SA345ruMSSAST8	NA	Viruses; Duplodnaviria; Heunggongvirae; Uroviricota; Caudoviricetes; Caudovirales; Siphoviridae; Biseptimavirus; unclassified Biseptimavirus
GCA_003368745.1	43454	68	Staphylococcus phage SA45ruMSSAST97	NA	Viruses; Duplodnaviria; Heunggongvirae; Uroviricota; Caudoviricetes; Caudovirales; Siphoviridae; Biseptimavirus; unclassified Biseptimavirus
GCA_003368765.1	43458	67	Staphylococcus phage SA537ruMSSAST97	NA	Viruses; Duplodnaviria; Heunggongvirae; Uroviricota; Caudoviricetes; Caudovirales; Siphoviridae; Biseptimavirus; unclassified Biseptimavirus
GCA_004134365.1	5281	2	Fusarium asiaticum victorivirus 1	NA	Viruses; Riboviria; Orthornavirae; Duplornaviricota; Chrymotiviricetes; Ghabrivirales; Totiviridae; Victorivirus; unclassified Victorivirus
GCA_002599725.1	46149	75	Vibrio phage H2 PGK2017	NA	Viruses; Duplodnaviria; Heunggongvirae; Uroviricota; Caudoviricetes; Caudovirales; Siphoviridae; unclassified Siphoviridae
GCA_002599785.1	39530	48	Vibrio phage H2 SGB2014	NA	Viruses; Duplodnaviria; Heunggongvirae; Uroviricota; Caudoviricetes; Caudovirales; Podoviridae; Enhodamvirus; unclassified Enhodamvirus
GCA_003369145.1	165975	247	Acinetobacter phage vB_ApiM_fHyAci03	NA	Viruses; Duplodnaviria; Heunggongvirae; Uroviricota; Caudoviricetes; Caudovirales; Myoviridae; Tevenvirinae; unclassified Tevenvirinae
GCA_003369165.1	40387	49	Shigella phage SFPH2	NA	Viruses; Duplodnaviria; Heunggongvirae; Uroviricota; Caudoviricetes; Caudovirales; Autographiviridae; Studiervirinae; Kayfunavirus; Shigella virus SFPH2
GCA_000843245.1	233234	352	Aeromonas virus Aeh1	NA	Viruses; dsDNA viruses, no RNA stage; Caudovirales; Myoviridae; Tevenvirinae; T4virus; unclassified T4virus
GCA_000855845.1	16044	10	Strawberry pallidosisassociated virus	NA	Viruses; ssRNA viruses; ssRNA positive-strand viruses, no DNA stage; Closteroviridae; Crinivirus
GCA_000843345.1	37555	50	Yersinia phage phiA1122	NA	Viruses; dsDNA viruses, no RNA stage; Caudovirales; Podoviridae; Autographivirinae; T7virus; unclassified T7-like viruses
GCA_000841405.1	30728	40	Yersinia virus L413C	NA	Viruses; Duplodnaviria; Heunggongvirae; Uroviricota; Caudoviricetes; Caudovirales; Myoviridae; Peduovirinae; Peduovirus
GCA_000864885.1	29751	14	SARS coronavirus Tor2	NA	Viruses; Riboviria; Orthornavirae; Pisuviricota; Pisoniviricetes; Nidovirales; Cornidovirineae; Coronaviridae; Orthocoronavirinae; Betacoronavirus; Sarbecovirus; Severe acute respiratory syndrome-related coronavirus

gca	genome.size	proteome.size	phage.name	strain	Taxonomy
GCA_002628885.1	46454	80	Shigella phage Sf11 SMD2017	NA	Viruses; Duplodnaviria; Heunggongvirae; Uroviricota; Caudoviricetes; Caudovirales; Siphoviridae; unclassified Siphoviridae
GCA_004130925.1	158250	152	Bufonid herpesvirus 1	FO1_2015	Viruses; Duplodnaviria; Heunggongvirae; Peploviricota; Herviviricetes; Herpesvirales; unclassified Herpesvirales
GCA_003369405.1	50316	78	Paenibacillus phage Unity	NA	Viruses; Duplodnaviria; Heunggongvirae; Uroviricota; Caudoviricetes; Caudovirales; Siphoviridae; Trippvirus; unclassified Trippvirus
GCA_013387625.1	156548	197	Salmonella phage vB_SalMLPST94	NA	Viruses; Duplodnaviria; Heunggongvirae; Uroviricota; Caudoviricetes; Caudovirales; Ackermannviridae; Cvivirinae; Kuttervirus; unclassified Kuttervirus
GCA_003423345.1	149960	243	Acinetobacter phage ABPH49	NA	Viruses; Duplodnaviria; Heunggongvirae; Uroviricota; Caudoviricetes; Caudovirales; Myoviridae; Vequintavirinae; unclassified Vequintavirinae
GCA_003369265.1	66061	95	Pseudomonas phage E79	NA	Viruses; Duplodnaviria; Heunggongvirae; Uroviricota; Caudoviricetes; Caudovirales; Myoviridae; Pbunavirus; unclassified Pbunavirus
GCA_003369285.1	142353	208	Bacillus phage Saddex	NA	Viruses; Duplodnaviria; Heunggongvirae; Uroviricota; Caudoviricetes; Caudovirales; Herelleviridae; Bastillevirinae; Wphvirus; unclassified Wphvirus
GCA_003575625.1	141288	205	Staphylococcus phage Terranova	NA	Viruses; Duplodnaviria; Heunggongvirae; Uroviricota; Caudoviricetes; Caudovirales; Herelleviridae; Twortvirinae; Sepunavirus; unclassified Sepunavirus
GCA_003365975.1	42969	59	Serratia phage Scapp	NA	Viruses; Duplodnaviria; Heunggongvirae; Uroviricota; Caudoviricetes; Caudovirales; Siphoviridae; unclassified Siphoviridae
GCA_003423365.1	41969	67	Enterobacteria phage O276	NA	Viruses; Duplodnaviria; Heunggongvirae; Uroviricota; Caudoviricetes; Caudovirales; Siphoviridae; Lambdavirus; unclassified Lambdavirus
GCA_013088085.1	2774	6	Sweet potato leaf curl Hubei virus	NA	Viruses; Monodnaviria; Shotokuvirae; Cressdnaviricota; Repensiviricetes; Geplafuvirales; Geminiviridae; Begomovirus
GCA_003958785.1	40236	22	Klebsiella phage KN11	NA	Viruses; Duplodnaviria; Heunggongvirae; Uroviricota; Caudoviricetes; Caudovirales; Autographiviridae; Studiervirinae; Przondovirus; Klebsiella virus KN1-1
GCA_003958825.1	41059	24	Klebsiella phage KN31	NA	Viruses; Duplodnaviria; Heunggongvirae; Uroviricota; Caudoviricetes; Caudovirales; Autographiviridae; Studiervirinae; Przondovirus; Klebsiella virus KN3-1
GCA_003958805.1	41219	20	Klebsiella phage KN41	NA	Viruses; Duplodnaviria; Heunggongvirae; Uroviricota; Caudoviricetes; Caudovirales; Autographiviridae; Studiervirinae; Przondovirus; Klebsiella virus KN4-1
GCA_004134125.1	2458	2	Arctopus echinatusassociated virus	NA	Viruses; unclassified viruses; unclassified DNA viruses
GCA_004134205.1	2197	2	Holcus lanatusassociated virus	NA	Viruses; unclassified viruses; unclassified DNA viruses
GCA_004134185.1	2293	2	Lolium perenneassociated virus	NA	Viruses; unclassified viruses; unclassified DNA viruses
GCA_004134165.1	2070	2	Panicum ecklоniiassociated virus	NA	Viruses; unclassified viruses; unclassified DNA viruses
GCA_004134145.1	2296	2	Stipagrostis associaed virus	NA	Viruses; unclassified viruses; unclassified DNA viruses
GCA_900500615.1	15378	11	Giant squirrel respirovirus	NA	Viruses; Riboviria; Orthornavirae; Negarnaviricota; Haploviricotina; Monjiviricetes; Mononegavirales; Paramyxoviridae; Orthoparamyxovirinae; Respirovirus; unclassified Respirovirus
GCA_003354205.1	61129	83	Ralstonia phage GP4	NA	Viruses; Duplodnaviria; Heunggongvirae; Uroviricota; Caudoviricetes; Caudovirales; Podoviridae; unclassified Podoviridae
GCA_003423165.1	48502	87	Mycobacterium phage Homines	NA	Viruses; Duplodnaviria; Heunggongvirae; Uroviricota; Caudoviricetes; Caudovirales; Siphoviridae; Fromanvirus; unclassified Fromanvirus
GCA_003423205.1	58731	107	Mycobacterium phage Harley	NA	Viruses; Duplodnaviria; Heunggongvirae; Uroviricota; Caudoviricetes; Caudovirales; Siphoviridae; Cheoctovirus; unclassified Cheoctovirus
GCA_003423225.1	69471	108	Mycobacterium phage Thonko	NA	Viruses; Duplodnaviria; Heunggongvirae; Uroviricota; Caudoviricetes; Caudovirales; Siphoviridae; Bclasvirinae; unclassified Bclasvirinae
GCA_003423185.1	53828	91	Mycobacterium phage Zeeculate	NA	Viruses; Duplodnaviria; Heunggongvirae; Uroviricota; Caudoviricetes; Caudovirales; Siphoviridae; Fromanvirus; unclassified Fromanvirus
GCA_003423285.1	43077	51	Sphingomonas phage Scott	NA	Viruses; Duplodnaviria; Heunggongvirae; Uroviricota; Caudoviricetes; Caudovirales; Autographiviridae; Scottvirus; Sphingomonas virus Scott
GCA_004768925.1	86950	131	Escherichia phage vB_EcoMRo111lw	NA	Viruses; Duplodnaviria; Heunggongvirae; Uroviricota; Caudoviricetes; Caudovirales; Myoviridae; Ounavirinae; Felixounavirus; unclassified Felixounavirus
GCA_006516895.1	42809	60	Pelagibacter phage HTVC021P	NA	Viruses; Duplodnaviria; Heunggongvirae; Uroviricota; Caudoviricetes; Caudovirales; Autographiviridae; Pelagivirus; unclassified Pelagivirus
GCA_003441475.1	288967	301	Vibrio phage BONAISHI	NA	Viruses; Duplodnaviria; Heunggongvirae; Uroviricota; Caudoviricetes; Caudovirales; Myoviridae; unclassified Myoviridae
GCA_003423425.1	42248	54	Bacillus phage Wes44	NA	Viruses; Duplodnaviria; Heunggongvirae; Uroviricota; Caudoviricetes; Caudovirales; Siphoviridae; unclassified Siphoviridae
GCA_003367035.1	153268	254	Bacillus phage BSP38	NA	Viruses; Duplodnaviria; Heunggongvirae; Uroviricota; Caudoviricetes; Caudovirales; Herelleviridae; Bastillevirinae; unclassified Bastillevirinae
GCA_006964085.1	57811	95	Enterococcus phage vB_EfaS_HEf13	NA	Viruses; Duplodnaviria; Heunggongvirae; Uroviricota; Caudoviricetes; Caudovirales; Siphoviridae; Saphexavirus; unclassified Saphexavirus
GCA_003367055.1	30930	44	Eggerthella phage PMBT5	NA	Viruses; Duplodnaviria; Heunggongvirae; Uroviricota; Caudoviricetes; Caudovirales; Siphoviridae; Lentavirus; Eggerthella virus PMBT5
GCA_006516675.1	42010	54	Pelagibacter phage HTVC022P	NA	Viruses; Duplodnaviria; Heunggongvirae; Uroviricota; Caudoviricetes; Caudovirales; Autographiviridae; Pelagivirus; unclassified Pelagivirus

gca	genome.size	proteome.size	phage.name	strain	Taxonomy
GCA_006516715.1	41046	53	Pelagibacter phage HTVC031P	NA	Viruses; Duplodnaviria; Heunggongvirae; Uroviricota; Caudoviricetes; Caudovirales; Autographiviridae; Pelagivirus; unclassified Pelagivirus
GCA_006516915.1	42385	56	Pelagibacter phage HTVC105P	NA	Viruses; Duplodnaviria; Heunggongvirae; Uroviricota; Caudoviricetes; Caudovirales; Autographiviridae; Pelagivirus; unclassified Pelagivirus
GCA_006516795.1	41323	54	Pelagibacter phage HTVC109P	NA	Viruses; Duplodnaviria; Heunggongvirae; Uroviricota; Caudoviricetes; Caudovirales; Autographiviridae; Pelagivirus; unclassified Pelagivirus
GCA_006516815.1	38357	54	Pelagibacter phage HTVC119P	NA	Viruses; Duplodnaviria; Heunggongvirae; Uroviricota; Caudoviricetes; Caudovirales; Autographiviridae; Votkovvirus; unclassified Votkovvirus
GCA_006516835.1	42622	53	Pelagibacter phage HTVC120P	NA	Viruses; Duplodnaviria; Heunggongvirae; Uroviricota; Caudoviricetes; Caudovirales; Autographiviridae; unclassified Autographiviridae
GCA_006516775.1	42600	57	Pelagibacter phage HTVC121P	NA	Viruses; Duplodnaviria; Heunggongvirae; Uroviricota; Caudoviricetes; Caudovirales; Autographiviridae; Pelagivirus; unclassified Pelagivirus
GCA_006516735.1	42221	53	Pelagibacter phage HTVC200P	NA	Viruses; Duplodnaviria; Heunggongvirae; Uroviricota; Caudoviricetes; Caudovirales; Autographiviridae; Votkovvirus; unclassified Votkovvirus
GCA_006516755.1	41415	52	Pelagibacter phage HTVC201P	NA	Viruses; Duplodnaviria; Heunggongvirae; Uroviricota; Caudoviricetes; Caudovirales; Autographiviridae; Pelagivirus; unclassified Pelagivirus
GCA_006304615.1	47116	80	Erwinia phage vB_EhrS_59	NA	Viruses; Duplodnaviria; Heunggongvirae; Uroviricota; Caudoviricetes; Caudovirales; Siphoviridae; Feofaniavirus; Erwinia virus Eho59
GCA_006304545.1	46835	80	Erwinia phage vB_EhrS_49	NA	Viruses; Duplodnaviria; Heunggongvirae; Uroviricota; Caudoviricetes; Caudovirales; Siphoviridae; Feofaniavirus; Erwinia virus Eho49
GCA_003423105.1	37011	56	Pseudomonas phage YMC1201R960	NA	Viruses; Duplodnaviria; Heunggongvirae; Uroviricota; Caudoviricetes; Caudovirales; Siphoviridae; Casadabanvirus; unclassified Casadabanvirus
GCA_003423145.1	46148	70	Pseudomonas phage YMC1201R24	NA	Viruses; Duplodnaviria; Heunggongvirae; Uroviricota; Caudoviricetes; Caudovirales; Siphoviridae; Casadabanvirus; unclassified Casadabanvirus
GCA_003568515.1	261658	330	Dickeya phage vB_DsoM_AD1	NA	Viruses; Duplodnaviria; Heunggongvirae; Uroviricota; Caudoviricetes; Caudovirales; Myoviridae; Alexandravirus; Dickeya virus AD1
GCA_003568455.1	254061	323	Dickeya phage vB_DsoM_JA13	NA	Viruses; Duplodnaviria; Heunggongvirae; Uroviricota; Caudoviricetes; Caudovirales; Myoviridae; Salmondvirus; unclassified Salmondvirus
GCA_003568475.1	253323	318	Dickeya phage vB_DsoM_JA29	NA	Viruses; Duplodnaviria; Heunggongvirae; Uroviricota; Caudoviricetes; Caudovirales; Myoviridae; Salmondvirus; Dickeya virus JA29
GCA_003568495.1	255356	321	Dickeya phage vB_DsoM_JA33	NA	Viruses; Duplodnaviria; Heunggongvirae; Uroviricota; Caudoviricetes; Caudovirales; Myoviridae; Salmondvirus; unclassified Salmondvirus
GCA_003568435.1	40131	50	Dickeya phage vB_DsoP_JA10	NA	Viruses; Duplodnaviria; Heunggongvirae; Uroviricota; Caudoviricetes; Caudovirales; Autographiviridae; Studiervirinae; Ningirsuvirus; Dickeya virus JA10
GCA_003367015.1	75514	101	Vibrio phage R01	NA	Viruses; Duplodnaviria; Heunggongvirae; Uroviricota; Caudoviricetes; Caudovirales; Siphoviridae; Mardecavirus; unclassified Mardecavirus
GCA_004133285.1	35906	3	Aplysia californica nidolike virus	NA	Viruses; Riboviria; Orthornavirae; Pisuviricota; Pisoniviricetes; Nidovirales; unclassified Nidovirales
GCA_003365995.1	15556	26	Arthrobacter phage Azathoth	NA	Viruses; Duplodnaviria; Heunggongvirae; Uroviricota; Caudoviricetes; Caudovirales; Siphoviridae; Decurrovirus; unclassified Decurrovirus
GCA_003366035.1	15556	26	Arthrobacter phage CGermain	NA	Viruses; Duplodnaviria; Heunggongvirae; Uroviricota; Caudoviricetes; Caudovirales; Siphoviridae; Decurrovirus; unclassified Decurrovirus
GCA_003366095.1	15556	26	Arthrobacter phage Copper	NA	Viruses; Duplodnaviria; Heunggongvirae; Uroviricota; Caudoviricetes; Caudovirales; Siphoviridae; Decurrovirus; unclassified Decurrovirus
GCA_003366115.1	15556	26	Arthrobacter phage Dewayne	NA	Viruses; Duplodnaviria; Heunggongvirae; Uroviricota; Caudoviricetes; Caudovirales; Siphoviridae; Decurrovirus; unclassified Decurrovirus
GCA_003366175.1	15556	26	Arthrobacter phage Hunnie	NA	Viruses; Duplodnaviria; Heunggongvirae; Uroviricota; Caudoviricetes; Caudovirales; Siphoviridae; Decurrovirus; unclassified Decurrovirus
GCA_003366215.1	15556	26	Arthrobacter phage Inspire2	NA	Viruses; Duplodnaviria; Heunggongvirae; Uroviricota; Caudoviricetes; Caudovirales; Siphoviridae; Decurrovirus; unclassified Decurrovirus
GCA_003366295.1	15556	26	Arthrobacter phage Ronnie	NA	Viruses; Duplodnaviria; Heunggongvirae; Uroviricota; Caudoviricetes; Caudovirales; Siphoviridae; Decurrovirus; unclassified Decurrovirus
GCA_003366515.1	66610	96	Gordonia phage Beyoncage	NA	Viruses; Duplodnaviria; Heunggongvirae; Uroviricota; Caudoviricetes; Caudovirales; Siphoviridae; Terapinvirus; unclassified Terapinvirus
GCA_003366275.1	64105	96	Gordonia phage Pleakley	NA	Viruses; Duplodnaviria; Heunggongvirae; Uroviricota; Caudoviricetes; Caudovirales; Siphoviridae; unclassified Siphoviridae
GCA_003366595.1	75345	100	Gordonia phage Teatealatte	NA	Viruses; Duplodnaviria; Heunggongvirae; Uroviricota; Caudoviricetes; Caudovirales; Siphoviridae; Demosthenesvirus; unclassified Demosthenesvirus
GCA_003366455.1	49729	84	Gordonia phage Zarbodnamra	NA	Viruses; Duplodnaviria; Heunggongvirae; Uroviricota; Caudoviricetes; Caudovirales; Siphoviridae; unclassified Siphoviridae
GCA_003366335.1	17452	25	Microbacterium phage Scamander	NA	Viruses; Duplodnaviria; Heunggongvirae; Uroviricota; Caudoviricetes; Caudovirales; Siphoviridae; unclassified Siphoviridae
GCA_003366495.1	52960	96	Mycobacterium phage Arlo	NA	Viruses; Duplodnaviria; Heunggongvirae; Uroviricota; Caudoviricetes; Caudovirales; Siphoviridae; Fromanvirus; unclassified Fromanvirus
GCA_003366015.1	50843	90	Mycobacterium phage BreSam8	NA	Viruses; Duplodnaviria; Heunggongvirae; Uroviricota; Caudoviricetes; Caudovirales; Siphoviridae; Fromanvirus; unclassified Fromanvirus
GCA_003366535.1	70183	118	Mycobacterium phage Bromden	NA	Viruses; Duplodnaviria; Heunggongvirae; Uroviricota; Caudoviricetes; Caudovirales; Siphoviridae; Bronvirus; unclassified Bronvirus

gca	genome.size	proteome.size	phage.name	strain	Taxonomy
GCA_003366475.1	68015	97	Mycobacterium phage DonSanchon	NA	Viruses; Duplodnaviria; Heunggongvirae; Uroviricota; Caudoviricetes; Caudovirales; Siphoviridae; Bclasvirinae; Pegunavirus; unclassified Pegunavirus
GCA_003366155.1	67993	99	Mycobacterium phage HSavage	NA	Viruses; Duplodnaviria; Heunggongvirae; Uroviricota; Caudoviricetes; Caudovirales; Siphoviridae; Bclasvirinae; Pegunavirus; unclassified Pegunavirus
GCA_003366135.1	75586	142	Mycobacterium phage Hopey	NA	Viruses; Duplodnaviria; Heunggongvirae; Uroviricota; Caudoviricetes; Caudovirales; Siphoviridae; Kostyavirus; unclassified Kostyavirus
GCA_003366195.1	74070	145	Mycobacterium phage Inca	NA	Viruses; Duplodnaviria; Heunggongvirae; Uroviricota; Caudoviricetes; Caudovirales; Siphoviridae; Kostyavirus; unclassified Kostyavirus
GCA_003366235.1	68494	100	Mycobacterium phage MichaelPhcott	NA	Viruses; Duplodnaviria; Heunggongvirae; Uroviricota; Caudoviricetes; Caudovirales; Siphoviridae; Bclasvirinae; Pegunavirus; unclassified Pegunavirus
GCA_003366255.1	51486	91	Mycobacterium phage Pita2	NA	Viruses; Duplodnaviria; Heunggongvirae; Uroviricota; Caudoviricetes; Caudovirales; Siphoviridae; Fromanvirus; unclassified Fromanvirus
GCA_003366555.1	50210	95	Mycobacterium phage Sibs6	NA	Viruses; Duplodnaviria; Heunggongvirae; Uroviricota; Caudoviricetes; Caudovirales; Siphoviridae; Fromanvirus; unclassified Fromanvirus
GCA_003366575.1	49097	90	Mycobacterium phage Target	NA	Viruses; Duplodnaviria; Heunggongvirae; Uroviricota; Caudoviricetes; Caudovirales; Siphoviridae; Fromanvirus; unclassified Fromanvirus
GCA_003366395.1	63874	96	Mycobacterium phage Thyatira	NA	Viruses; Duplodnaviria; Heunggongvirae; Uroviricota; Caudoviricetes; Caudovirales; Siphoviridae; Timquatrovirus; unclassified Timquatrovirus
GCA_003366615.1	68657	99	Mycobacterium phage Tydolla	NA	Viruses; Duplodnaviria; Heunggongvirae; Uroviricota; Caudoviricetes; Caudovirales; Siphoviridae; Bclasvirinae; Pipefishvirus; unclassified Pipefishvirus
GCA_003366415.1	68405	102	Mycobacterium phage UAch1	NA	Viruses; Duplodnaviria; Heunggongvirae; Uroviricota; Caudoviricetes; Caudovirales; Siphoviridae; Bclasvirinae; Pegunavirus; unclassified Pegunavirus
GCA_003366315.1	186702	325	Streptomyces phage Satis	NA	Viruses; Duplodnaviria; Heunggongvirae; Uroviricota; Caudoviricetes; Caudovirales; Siphoviridae; unclassified Siphoviridae
GCA_003366375.1	133886	251	Streptomyces phage StarPlatinum	NA	Viruses; Duplodnaviria; Heunggongvirae; Uroviricota; Caudoviricetes; Caudovirales; Siphoviridae; unclassified Siphoviridae
GCA_003366355.1	131427	238	Streptomyces phage Starbow	NA	Viruses; Duplodnaviria; Heunggongvirae; Uroviricota; Caudoviricetes; Caudovirales; Siphoviridae; unclassified Siphoviridae
GCA_003366435.1	133007	236	Streptomyces phage Wofford	NA	Viruses; Duplodnaviria; Heunggongvirae; Uroviricota; Caudoviricetes; Caudovirales; Siphoviridae; unclassified Siphoviridae
GCA_003443255.1	220934	335	Caulobacter phage CcrBL10	NA	Viruses; Duplodnaviria; Heunggongvirae; Uroviricota; Caudoviricetes; Caudovirales; Siphoviridae; Dolichocephalovirinae; Poindextervirus; Caulobacter virus CcrBL10
GCA_003443295.1	322272	558	Caulobacter phage CcrBL9	NA	Viruses; Duplodnaviria; Heunggongvirae; Uroviricota; Caudoviricetes; Caudovirales; Siphoviridae; Dolichocephalovirinae; Bertelyvirus; Caulobacter virus CcrBL9
GCA_003443275.1	308141	485	Caulobacter phage CcrPW	NA	Viruses; Duplodnaviria; Heunggongvirae; Uroviricota; Caudoviricetes; Caudovirales; Siphoviridae; Dolichocephalovirinae; Colossusvirus; Caulobacter virus CcrPW
GCA_003443315.2	317489	545	Caulobacter phage CcrSC	NA	Viruses; Duplodnaviria; Heunggongvirae; Uroviricota; Caudoviricetes; Caudovirales; Siphoviridae; Dolichocephalovirinae; Bertelyvirus; Caulobacter virus CcrSC
GCA_003575865.1	55978	92	Salmonella phage Solent	NA	Viruses; Duplodnaviria; Heunggongvirae; Uroviricota; Caudoviricetes; Caudovirales; Siphoviridae; Sashavirus; unclassified Sashavirus
GCA_003575885.1	84668	131	Salmonella phage Meda	NA	Viruses; Duplodnaviria; Heunggongvirae; Uroviricota; Caudoviricetes; Caudovirales; Myoviridae; Ounavirinae; Felixounavirus; unclassified Felixounavirus
GCA_003366935.1	90490	165	Gordonia phage Daredevil	NA	Viruses; Duplodnaviria; Heunggongvirae; Uroviricota; Caudoviricetes; Caudovirales; Siphoviridae; Daredevilvirus; Gordonia virus DareDevil
GCA_003366995.1	40494	63	Microbacterium phage Andromedas	NA	Viruses; Duplodnaviria; Heunggongvirae; Uroviricota; Caudoviricetes; Caudovirales; Siphoviridae; Elerivirus; unclassified Elerivirus
GCA_003366955.1	40494	63	Microbacterium phage ColaCorta	NA	Viruses; Duplodnaviria; Heunggongvirae; Uroviricota; Caudoviricetes; Caudovirales; Siphoviridae; Elerivirus; unclassified Elerivirus
GCA_003366875.1	52992	92	Microbacterium phage KaiHaiDragon	NA	Viruses; Duplodnaviria; Heunggongvirae; Uroviricota; Caudoviricetes; Caudovirales; Siphoviridae; Quhwahvirus
GCA_003366695.1	41872	63	Microbacterium phage Schnapsidee	NA	Viruses; Duplodnaviria; Heunggongvirae; Uroviricota; Caudoviricetes; Caudovirales; Siphoviridae; Ilzatvirus; unclassified Ilzatvirus
GCA_003423305.1	154861	229	Mycobacterium phage Rabinovish	NA	Viruses; Duplodnaviria; Heunggongvirae; Uroviricota; Caudoviricetes; Caudovirales; Myoviridae; Bixzunavirus; unclassified Bixzunavirus
GCA_003366735.1	72658	132	Mycobacterium phage Ryadel	NA	Viruses; Duplodnaviria; Heunggongvirae; Uroviricota; Caudoviricetes; Caudovirales; Siphoviridae; Corndogvirus; unclassified Corndogvirus
GCA_003366715.1	67304	92	Mycobacterium phage Sabella	NA	Viruses; Duplodnaviria; Heunggongvirae; Uroviricota; Caudoviricetes; Caudovirales; Siphoviridae; Bclasvirinae; Rosebushvirus; unclassified Rosebushvirus
GCA_003366975.1	41456	62	Mycobacterium phage Cherrybomb426	NA	Viruses; Duplodnaviria; Heunggongvirae; Uroviricota; Caudoviricetes; Caudovirales; Siphoviridae; Liefievirus; unclassified Liefievirus
GCA_003366915.1	57955	103	Mycobacterium phage Gorge	NA	Viruses; Duplodnaviria; Heunggongvirae; Uroviricota; Caudoviricetes; Caudovirales; Siphoviridae; Cheoctovirus; unclassified Cheoctovirus
GCA_003366835.1	53058	97	Mycobacterium phage Krakatau	NA	Viruses; Duplodnaviria; Heunggongvirae; Uroviricota; Caudoviricetes; Caudovirales; Siphoviridae; Cheoctovirus; unclassified Cheoctovirus
GCA_003366795.1	57001	102	Mycobacterium phage Mantra	NA	Viruses; Duplodnaviria; Heunggongvirae; Uroviricota; Caudoviricetes; Caudovirales; Siphoviridae; Cheoctovirus; unclassified Cheoctovirus
GCA_003366775.1	50535	88	Mycobacterium phage NEHalo	NA	Viruses; Duplodnaviria; Heunggongvirae; Uroviricota; Caudoviricetes; Caudovirales; Siphoviridae; Fromanvirus; unclassified Fromanvirus

gca	genome.size	proteome.size	phage.name	strain	Taxonomy
GCA_003366755.1	68390	100	Mycobacterium phage PinheadLarry	NA	Viruses; Duplodnaviria; Heunggongvirae; Uroviricota; Caudoviricetes; Caudovirales; Siphoviridae; Bclasvirinae; Pegunavirus; unclassified Pegunavirus
GCA_003366655.1	66887	99	Mycobacterium phage Vaticameos	NA	Viruses; Duplodnaviria; Heunggongvirae; Uroviricota; Caudoviricetes; Caudovirales; Siphoviridae; Bclasvirinae; Pegunavirus; unclassified Pegunavirus
GCA_003366635.1	76915	149	Mycobacterium phage xkcd	NA	Viruses; Duplodnaviria; Heunggongvirae; Uroviricota; Caudoviricetes; Caudovirales; Siphoviridae; Kostyavirus; unclassified Kostyavirus
GCA_003366895.1	50660	81	Streptomyces phage Haizum	NA	Viruses; Duplodnaviria; Heunggongvirae; Uroviricota; Caudoviricetes; Caudovirales; Siphoviridae; Arquatrovirinae; Arequatrovirus; unclassified Arequatrovirus
GCA_003366855.1	131909	241	Streptomyces phage Karimac	NA	Viruses; Duplodnaviria; Heunggongvirae; Uroviricota; Caudoviricetes; Caudovirales; Siphoviridae; unclassified Siphoviridae
GCA_003366815.1	133195	248	Streptomyces phage LukeCage	NA	Viruses; Duplodnaviria; Heunggongvirae; Uroviricota; Caudoviricetes; Caudovirales; Siphoviridae; unclassified Siphoviridae
GCA_003366675.1	129742	232	Streptomyces phage SparkleGoddess	NA	Viruses; Duplodnaviria; Heunggongvirae; Uroviricota; Caudoviricetes; Caudovirales; Siphoviridae; unclassified Siphoviridae
GCA_013088115.1	5631	6	Faba bean polerovirus 1	5253	Viruses; Riboviria; Orthornavirae; Kitrinoviricota; Tolucaviricetes; Tolivirales; Luteoviridae; Polerovirus
GCA_004134325.1	5839	4	Potexvirus sp	NA	Viruses; Riboviria; Orthornavirae; Kitrinoviricota; Alsuviricetes; Tymovirales; Alphaflexiviridae; Potexvirus; unclassified Potexvirus
GCA_000852505.1	6303	3	Youcai mosaic virus	NA	Viruses; ssRNA viruses; ssRNA positive-strand viruses, no DNA stage; Virgaviridae; Tobamovirus
GCA_000848445.1	5662	6	Cereal yellow dwarf virusRPS	NA	Viruses; ssRNA viruses; ssRNA positive-strand viruses, no DNA stage; Luteoviridae; Polerovirus
GCA_000840905.1	5331	7	Sida micrantha mosaic virus	NA	Viruses; ssDNA viruses; Geminiviridae; Begomovirus
GCA_000883515.1	6449	4	Cactus mild mottle virus	NA	Viruses; ssRNA viruses; ssRNA positive-strand viruses, no DNA stage; Virgaviridae; Tobamovirus
GCA_008632495.1	37136	54	Curvibacter phage TJ1	NA	Viruses; Duplodnaviria; Heunggongvirae; Uroviricota; Caudoviricetes; Caudovirales; Myoviridae; unclassified Myoviridae
GCA_013086065.1	28778	13	Kundal virus	MCL13T316	Viruses; Riboviria; Orthornavirae; Duplornaviricota; Resentoviricetes; Reovirales; Reoviridae; Spinareovirinae; Coltivirus; Kundal coltivirus
GCA_000853605.1	4421	5	Johnsongrass chlorotic stripe mosaic virus	NA	Viruses; ssRNA viruses; ssRNA positive-strand viruses, no DNA stage; Tombusviridae; Aureusvirus
GCA_003847565.1	2195	2	Momordica charantia associated gemycircularvirus	NA	Viruses; Monodnaviria; Shotokuvirae; Cressdnaviricota; Repensiviricetes; Geplafuvirales; Genomoviridae; Gemycircularvirus; unclassified Gemycircularvirus
GCA_003847545.1	2125	2	Euphorbia heterophylla associated gemycircularvirus	NA	Viruses; Monodnaviria; Shotokuvirae; Cressdnaviricota; Repensiviricetes; Geplafuvirales; Genomoviridae; Gemycircularvirus; unclassified Gemycircularvirus
GCA_018582725.1	2528	1	Pyricularia oryzae ourmialike virus 1	NA	Viruses; Riboviria; Orthornavirae; Lenarviricota; Miaviricetes; Ourlivirales; Botourmiaviridae; Penoulivirus; Pyricularia penoulivirus
GCA_018582735.1	1671	1	Pyricularia oryzae ourmialike virus 2	NA	Viruses; Riboviria; Orthornavirae; Lenarviricota; Miaviricetes; Ourlivirales; Botourmiaviridae; Scleroulivirus; Pyricularia scleroulivirus 2
GCA_018582745.1	2557	1	Pyricularia oryzae ourmialike virus 3	NA	Viruses; Riboviria; Orthornavirae; Lenarviricota; Miaviricetes; Ourlivirales; Botourmiaviridae; Scleroulivirus; Pyricularia scleroulivirus 3
GCA_004134245.1	16996	10	Blackcurrant leafrollassociated virus 1	NA	Viruses; Riboviria; Orthornavirae; Kitrinoviricota; Alsuviricetes; Martellivirales; Closteroviridae; Closterovirus; unclassified Closterovirus
GCA_004133565.1	6278	5	Turtle grass virus X	NA	Viruses; Riboviria; Orthornavirae; Kitrinoviricota; Alsuviricetes; Tymovirales; Alphaflexiviridae; Potexvirus; unclassified Potexvirus
GCA_008801055.1	95343	127	Achromobacter phage vB_Ade_ART	NA	Viruses; Duplodnaviria; Heunggongvirae; Uroviricota; Caudoviricetes; Caudovirales; Siphoviridae; unclassified Siphoviridae
GCA_003847185.1	1769	2	Arboreal ant associated circular virus 1	NA	Viruses; Monodnaviria; Shotokuvirae; Cressdnaviricota; Arfiviricetes; Cirlivirales; Circoviridae; Cyclovirus; Ant associated cyclovirus 1
GCA_003847505.1	2237	2	Bark beetle associated circular virus 1	NA	Viruses; Monodnaviria; Shotokuvirae; Cressdnaviricota; Repensiviricetes; Geplafuvirales; Genomoviridae; unclassified Genomoviridae
GCA_003846925.1	2516	2	Cricket associated circular virus 1	NA	Viruses; unclassified viruses; unclassified DNA viruses; unclassified ssDNA viruses; Volvovirus
GCA_003847005.1	1991	2	Cybaeus spider associated circular virus 1	NA	Viruses; unclassified viruses; unclassified DNA viruses; unclassified ssDNA viruses; Circularisvirus
GCA_003847305.1	2344	2	Cybaeus spider associated circular virus 2	NA	Viruses; Monodnaviria; Shotokuvirae; Cressdnaviricota; Repensiviricetes; Geplafuvirales; Genomoviridae; unclassified Genomoviridae
GCA_003846625.1	2199	2	False black widow spider associated circular virus 1	NA	Viruses; unclassified viruses; unclassified DNA viruses; unclassified ssDNA viruses
GCA_003846985.1	3226	2	Fire ant associated circular virus 1	NA	Viruses; unclassified viruses; unclassified DNA viruses; unclassified ssDNA viruses; Cruciviridae; Crucivirus
GCA_003847165.1	1722	2	Fly associated circular virus 1	NA	Viruses; Monodnaviria; Shotokuvirae; Cressdnaviricota; Arfiviricetes; Cirlivirales; Circoviridae; Cyclovirus; unclassified Cyclovirus
GCA_003847485.1	2207	2	Fly associated circular virus 2	NA	Viruses; Monodnaviria; Shotokuvirae; Cressdnaviricota; Repensiviricetes; Geplafuvirales; Genomoviridae; unclassified Genomoviridae
GCA_003846965.1	2537	2	Fly associated circular virus 3	NA	Viruses; Monodnaviria; Shotokuvirae; Cressdnaviricota; Arfiviricetes; Cremevirales; Smacoviridae; Porprismacovirus; unclassified Porprismacovirus

gca	genome.size	proteome.size	phage.name	strain	Taxonomy
GCA_003846945.1	2546	2	Fly associated circular virus 4	NA	Viruses; Monodnaviria; Shotokuvirae; Cressdnaviricota; Arfiviricetes; Cremevirales; Smacoviridae; Porprismacovirus; unclassified Porprismacovirus
GCA_003847105.1	1997	2	Fly associated circular virus 5	NA	Viruses; unclassified viruses; unclassified DNA viruses; unclassified ssDNA viruses
GCA_003846845.1	2103	2	Fly associated circular virus 6	NA	Viruses; unclassified viruses; unclassified DNA viruses; unclassified ssDNA viruses
GCA_003846825.1	2010	2	Fly associated circular virus 7	NA	Viruses; unclassified viruses; unclassified DNA viruses; unclassified ssDNA viruses
GCA_003847265.1	2093	2	Giant house spider associated circular virus 1	NA	Viruses; Monodnaviria; Shotokuvirae; Cressdnaviricota; Repensiviricetes; Geplafuvirales; Genomoviridae; unclassified Genomoviridae
GCA_003846725.1	2040	2	Giant house spider associated circular virus 2	NA	Viruses; unclassified viruses; unclassified DNA viruses; unclassified ssDNA viruses
GCA_003846705.1	2290	2	Giant house spider associated circular virus 3	NA	Viruses; unclassified viruses; unclassified DNA viruses; unclassified ssDNA viruses
GCA_003846645.1	2494	2	Giant house spider associated circular virus 4	NA	Viruses; unclassified viruses; unclassified DNA viruses; unclassified ssDNA viruses
GCA_003847045.1	2054	2	Golden silk orbweaver associated circular virus 1	NA	Viruses; unclassified viruses; unclassified DNA viruses; unclassified ssDNA viruses; Circularisvirus
GCA_003847455.1	2309	2	Grasshopper associated circular virus 1	NA	Viruses; Monodnaviria; Shotokuvirae; Cressdnaviricota; Repensiviricetes; Geplafuvirales; Genomoviridae; unclassified Genomoviridae
GCA_003847025.1	1905	2	Longjawed orbweaver circular virus 1	NA	Viruses; unclassified viruses; unclassified DNA viruses; unclassified ssDNA viruses; Circularisvirus
GCA_003846865.1	2321	2	Longjawed orbweaver circular virus 2	NA	Viruses; unclassified viruses; unclassified DNA viruses; unclassified ssDNA viruses
GCA_003846805.1	1987	2	Millipede associated circular virus 1	NA	Viruses; unclassified viruses; unclassified DNA viruses; unclassified ssDNA viruses
GCA_003847285.1	2240	2	Pimoid spider associated circular virus 1	NA	Viruses; Monodnaviria; Shotokuvirae; Cressdnaviricota; Repensiviricetes; Geplafuvirales; Genomoviridae; unclassified Genomoviridae
GCA_003846765.1	2125	2	Pimoid spider associated circular virus 2	NA	Viruses; unclassified viruses; unclassified DNA viruses; unclassified ssDNA viruses
GCA_003847245.1	2232	2	Sierra dome spider associated circular virus 1	NA	Viruses; Monodnaviria; Shotokuvirae; Cressdnaviricota; Repensiviricetes; Geplafuvirales; Genomoviridae; unclassified Genomoviridae
GCA_003846745.1	1860	2	Sierra dome spider associated circular virus 2	NA	Viruses; unclassified viruses; unclassified DNA viruses; unclassified ssDNA viruses
GCA_003847125.1	1937	2	Soft spider associated circular virus 1	NA	Viruses; Monodnaviria; Shotokuvirae; Cressdnaviricota; Arfiviricetes; Cirlivirales; Circoviridae; Cyclovirus; Spider associated cyclovirus 1
GCA_003847345.1	2214	2	Spider associated circular virus 1	NA	Viruses; Monodnaviria; Shotokuvirae; Cressdnaviricota; Repensiviricetes; Geplafuvirales; Genomoviridae; unclassified Genomoviridae
GCA_003847325.1	2204	2	Spider associated circular virus 2	NA	Viruses; Monodnaviria; Shotokuvirae; Cressdnaviricota; Repensiviricetes; Geplafuvirales; Genomoviridae; unclassified Genomoviridae
GCA_003846685.1	1889	2	Spider associated circular virus 3	NA	Viruses; unclassified viruses; unclassified DNA viruses; unclassified ssDNA viruses
GCA_003847065.1	1995	2	Spinybacked orbweaver circular virus 1	NA	Viruses; unclassified viruses; unclassified DNA viruses; unclassified ssDNA viruses; Circularisvirus
GCA_003847145.1	1707	2	Spinybacked orbweaver circular virus 2	NA	Viruses; Monodnaviria; Shotokuvirae; Cressdnaviricota; Arfiviricetes; Cirlivirales; Circoviridae; Cyclovirus; unclassified Cyclovirus
GCA_003846785.1	2127	2	Tentweb spider associated circular virus 1	NA	Viruses; unclassified viruses; unclassified DNA viruses; unclassified ssDNA viruses
GCA_003847425.1	2174	2	Tubeweb spider associated circular virus 1	NA	Viruses; Monodnaviria; Shotokuvirae; Cressdnaviricota; Repensiviricetes; Geplafuvirales; Genomoviridae; unclassified Genomoviridae
GCA_003846885.1	2244	2	Water beetle associated circular virus 1	NA	Viruses; unclassified viruses; unclassified DNA viruses; unclassified ssDNA viruses
GCA_003846665.1	2176	2	Woodlouse hunter spider associated circular virus 1	NA	Viruses; unclassified viruses; unclassified DNA viruses; unclassified ssDNA viruses
GCA_006452115.1	185650	181	Heliothis virescens ascovirus 3i	NA	Viruses; Varidnaviria; Bamfordvirae; Nucleocytoviricota; Megaviricetes; Pimascovirales; Ascoviridae; Ascovirus; unclassified Ascovirus
GCA_013088125.1	9769	1	Mashua virus Y	NA	Viruses; Riboviria; Orthornavirae; Pisuviricota; Stelpaviricetes; Patatavirales; Potyviridae; Potyvirus
GCA_003403335.1	104363	123	Cylindrospermopsis raciborskii virus RM2018a	NA	Viruses; Duplodnaviria; Heunggongvirae; Uroviricota; Caudoviricetes; Caudovirales; Siphoviridae; unclassified Siphoviridae
GCA_018583565.1	3885	5	Jasmine mosaicassociated virus 2	NA	Viruses; Riboviria; Orthornavirae; Kitrinoviricota; Tolucaviricetes; Tolivirales; Tombusviridae; Procedovirinae; Pelarspovirus; unclassified Pelarspovirus
GCA_004130795.1	2077	2	Paguma larvata circovirus	NA	Viruses; Monodnaviria; Shotokuvirae; Cressdnaviricota; Arfiviricetes; Cirlivirales; Circoviridae; Circovirus; Civet circovirus
GCA_013088135.1	9952	2	Passiflora edulis symptomless virus	NA	Viruses; Riboviria; Orthornavirae; Pisuviricota; Stelpaviricetes; Patatavirales; Potyviridae; Roymovirus
GCA_004133185.1	7876	7	Macaca mulatta papillomavirus 2	NA	Viruses; Monodnaviria; Shotokuvirae; Cossaviricota; Papovaviricetes; Zurhausenvirales; Papillomaviridae; unclassified Papillomaviridae; primate papillomaviruses

gca	genome.size	proteome.size	phage.name	strain	Taxonomy
GCA_004133205.1	8051	7	Macaca mulatta papillomavirus 3	NA	Viruses; Monodnaviria; Shotokuvirae; Cossaviricota; Papovaviricetes; Zurhausenvirales; Papillomaviridae; unclassified Papillomaviridae; primate papillomaviruses
GCA_004133225.1	7424	7	Macaca mulatta papillomavirus 4	NA	Viruses; Monodnaviria; Shotokuvirae; Cossaviricota; Papovaviricetes; Zurhausenvirales; Papillomaviridae; unclassified Papillomaviridae; primate papillomaviruses
GCA_004134345.1	7126	7	Boa constrictor papillomavirus 1	NA	Viruses; Monodnaviria; Shotokuvirae; Cossaviricota; Papovaviricetes; Zurhausenvirales; Papillomaviridae; Firstpapillomavirinae; Dyomupapillomavirus; unclassified Dyomupapillomavirus
GCA_002822845.1	2742	6	Papaya leaf curl China virus	NA	Viruses; ssDNA viruses; Geminiviridae; Begomovirus
GCA_002824085.1	2738	6	Tomato leaf curl China virus	NA	Viruses; ssDNA viruses; Geminiviridae; Begomovirus
GCA_003429165.1	68176	121	Sinorhizobium phage phiM6	NA	Viruses; Duplodnaviria; Heunggongvirae; Uroviricota; Caudoviricetes; Caudovirales; Podoviridae; unclassified Podoviridae
GCA_003843585.1	40907	55	Lentibacter virus vB_LenP_ICBM2	NA	Viruses; Duplodnaviria; Heunggongvirae; Uroviricota; Caudoviricetes; Caudovirales; Podoviridae; unclassified Podoviridae
GCA_003843565.1	40497	56	Lentibacter virus vB_LenP_ICBM3	NA	Viruses; Duplodnaviria; Heunggongvirae; Uroviricota; Caudoviricetes; Caudovirales; Podoviridae; unclassified Podoviridae
GCA_003575685.1	156882	211	Salmonella phage Mooltan	NA	Viruses; Duplodnaviria; Heunggongvirae; Uroviricota; Caudoviricetes; Caudovirales; Myoviridae; Viunavirus; unclassified Viunavirus
GCA_003934415.1	49861	74	Klebsiella phage TSK1	NA	Viruses; Duplodnaviria; Heunggongvirae; Uroviricota; Caudoviricetes; Caudovirales; Drexlerviridae; Webervirus; Klebsiella virus TSK1
GCA_003991725.2	11129	26	Thermus phage phiKo	NA	Viruses; Varidnaviria; Bamfordvirae; Preplasmiviricota; Tectiliviricetes; Kalamavirales; Tectiviridae; unclassified Tectiviridae
GCA_003991705.1	178531	166	Thermus phage phiLo	NA	Viruses; Duplodnaviria; Heunggongvirae; Uroviricota; Caudoviricetes; Caudovirales; Myoviridae; unclassified Myoviridae
GCA_003441955.1	46888	78	Gordonia phage Catfish	NA	Viruses; Duplodnaviria; Heunggongvirae; Uroviricota; Caudoviricetes; Caudovirales; Siphoviridae; Vendettavirus; unclassified Vendettavirus
GCA_003442115.1	41706	62	Microbacterium phage Neferthena	NA	Viruses; Duplodnaviria; Heunggongvirae; Uroviricota; Caudoviricetes; Caudovirales; Siphoviridae; Neferthenavirus; Microbacterium virus Neferthena
GCA_003441915.1	54145	86	Mycobacterium phage Amochick	NA	Viruses; Duplodnaviria; Heunggongvirae; Uroviricota; Caudoviricetes; Caudovirales; Siphoviridae; Gilesvirus; unclassified Gilesvirus
GCA_003441935.1	59708	96	Mycobacterium phage Belladonna	NA	Viruses; Duplodnaviria; Heunggongvirae; Uroviricota; Caudoviricetes; Caudovirales; Siphoviridae; Timquatrovirus; unclassified Timquatrovirus
GCA_003442015.1	113536	239	Mycobacterium phage EricMillard	NA	Viruses; Duplodnaviria; Heunggongvirae; Uroviricota; Caudoviricetes; Caudovirales; Siphoviridae; Omegavirus; unclassified Omegavirus
GCA_003442035.1	67494	93	Mycobacterium phage FrenchFry	NA	Viruses; Duplodnaviria; Heunggongvirae; Uroviricota; Caudoviricetes; Caudovirales; Siphoviridae; Bclasvirinae; Rosebushvirus; unclassified Rosebushvirus
GCA_003442135.1	53348	95	Mycobacterium phage QueenBeesly	NA	Viruses; Duplodnaviria; Heunggongvirae; Uroviricota; Caudoviricetes; Caudovirales; Siphoviridae; Fromanvirus; unclassified Fromanvirus
GCA_003442175.1	44205	70	Mycobacterium phage Tapioca	NA	Viruses; Duplodnaviria; Heunggongvirae; Uroviricota; Caudoviricetes; Caudovirales; Siphoviridae; Nclasvirinae; Charlievirus; unclassified Charlievirus
GCA_003441875.1	51703	84	Mycobacterium phage Adahisdi	NA	Viruses; Duplodnaviria; Heunggongvirae; Uroviricota; Caudoviricetes; Caudovirales; Siphoviridae; Fromanvirus; unclassified Fromanvirus
GCA_003441895.1	44333	67	Mycobacterium phage Aggie	NA	Viruses; Duplodnaviria; Heunggongvirae; Uroviricota; Caudoviricetes; Caudovirales; Siphoviridae; Nclasvirinae; Charlievirus; unclassified Charlievirus
GCA_003435005.1	160330	223	Mycobacterium phage Cane17	NA	Viruses; Duplodnaviria; Heunggongvirae; Uroviricota; Caudoviricetes; Caudovirales; Myoviridae; Bixzunavirus; unclassified Bixzunavirus
GCA_003441975.1	50827	89	Mycobacterium phage Crispicous1	NA	Viruses; Duplodnaviria; Heunggongvirae; Uroviricota; Caudoviricetes; Caudovirales; Siphoviridae; Fromanvirus; unclassified Fromanvirus
GCA_003441995.1	111924	234	Mycobacterium phage Ejimix	NA	Viruses; Duplodnaviria; Heunggongvirae; Uroviricota; Caudoviricetes; Caudovirales; Siphoviridae; Omegavirus; unclassified Omegavirus
GCA_003442055.1	43697	71	Mycobacterium phage Gex	NA	Viruses; Duplodnaviria; Heunggongvirae; Uroviricota; Caudoviricetes; Caudovirales; Siphoviridae; Nclasvirinae; Charlievirus; unclassified Charlievirus
GCA_003442075.1	81036	137	Mycobacterium phage IPhane7	NA	Viruses; Duplodnaviria; Heunggongvirae; Uroviricota; Caudoviricetes; Caudovirales; Siphoviridae; Mclasvirinae; Bongovirus; unclassified Bongovirus
GCA_003575765.1	155217	236	Mycobacterium phage InigoMontoya	NA	Viruses; Duplodnaviria; Heunggongvirae; Uroviricota; Caudoviricetes; Caudovirales; Myoviridae; Bixzunavirus; unclassified Bixzunavirus
GCA_003442095.1	51367	86	Mycobacterium phage Jaykayelowell	NA	Viruses; Duplodnaviria; Heunggongvirae; Uroviricota; Caudoviricetes; Caudovirales; Siphoviridae; Fromanvirus; unclassified Fromanvirus
GCA_003575785.1	155959	237	Mycobacterium phage Phlegm	NA	Viruses; Duplodnaviria; Heunggongvirae; Uroviricota; Caudoviricetes; Caudovirales; Myoviridae; Bixzunavirus; unclassified Bixzunavirus
GCA_003442155.1	59925	91	Mycobacterium phage Rando14	NA	Viruses; Duplodnaviria; Heunggongvirae; Uroviricota; Caudoviricetes; Caudovirales; Siphoviridae; Timquatrovirus; unclassified Timquatrovirus
GCA_003442335.1	64370	91	Gordonia phage Marietta	NA	Viruses; Duplodnaviria; Heunggongvirae; Uroviricota; Caudoviricetes; Caudovirales; Siphoviridae; unclassified Siphoviridae
GCA_003442455.1	67359	95	Gordonia phage Skysand	NA	Viruses; Duplodnaviria; Heunggongvirae; Uroviricota; Caudoviricetes; Caudovirales; Siphoviridae; unclassified Siphoviridae
GCA_003442495.1	58677	93	Gordonia phage TillyBobJoe	NA	Viruses; Duplodnaviria; Heunggongvirae; Uroviricota; Caudoviricetes; Caudovirales; Siphoviridae; Wizardvirus; unclassified Wizardvirus

gca	genome.size	proteome.size	phage.name	strain	Taxonomy
GCA_003442195.1	58301	84	Gordonia phage Ali17	NA	Viruses; Duplodnaviria; Heunggongvirae; Uroviricota; Caudoviricetes; Caudovirales; Siphoviridae; unclassified Siphoviridae
GCA_003442355.1	154780	230	Mycobacterium phage Megamind	NA	Viruses; Duplodnaviria; Heunggongvirae; Uroviricota; Caudoviricetes; Caudovirales; Myoviridae; Bixzunavirus; unclassified Bixzunavirus
GCA_003442215.1	59482	108	Mycobacterium phage EleanorGeorge	NA	Viruses; Duplodnaviria; Heunggongvirae; Uroviricota; Caudoviricetes; Caudovirales; Siphoviridae; Cheoctovirus; unclassified Cheoctovirus
GCA_003442235.1	75295	146	Mycobacterium phage Emmina	NA	Viruses; Duplodnaviria; Heunggongvirae; Uroviricota; Caudoviricetes; Caudovirales; Siphoviridae; Kostyavirus; unclassified Kostyavirus
GCA_003442255.1	57754	102	Mycobacterium phage Girr	NA	Viruses; Duplodnaviria; Heunggongvirae; Uroviricota; Caudoviricetes; Caudovirales; Siphoviridae; Cheoctovirus; unclassified Cheoctovirus
GCA_003442295.1	58962	107	Mycobacterium phage JoeyJr	NA	Viruses; Duplodnaviria; Heunggongvirae; Uroviricota; Caudoviricetes; Caudovirales; Siphoviridae; Cheoctovirus; unclassified Cheoctovirus
GCA_003442375.1	55767	96	Mycobacterium phage Nimbo	NA	Viruses; Duplodnaviria; Heunggongvirae; Uroviricota; Caudoviricetes; Caudovirales; Siphoviridae; Cheoctovirus; unclassified Cheoctovirus
GCA_003442395.1	57989	104	Mycobacterium phage PHappiness	NA	Viruses; Duplodnaviria; Heunggongvirae; Uroviricota; Caudoviricetes; Caudovirales; Siphoviridae; Cheoctovirus; unclassified Cheoctovirus
GCA_003442415.1	49209	88	Mycobacterium phage PherrisBueller	NA	Viruses; Duplodnaviria; Heunggongvirae; Uroviricota; Caudoviricetes; Caudovirales; Siphoviridae; Fromanvirus; unclassified Fromanvirus
GCA_003442435.1	57526	105	Mycobacterium phage QuickMath	NA	Viruses; Duplodnaviria; Heunggongvirae; Uroviricota; Caudoviricetes; Caudovirales; Siphoviridae; Cheoctovirus; unclassified Cheoctovirus
GCA_003442475.1	58604	104	Mycobacterium phage Spikelee	NA	Viruses; Duplodnaviria; Heunggongvirae; Uroviricota; Caudoviricetes; Caudovirales; Siphoviridae; Cheoctovirus; unclassified Cheoctovirus
GCA_003442535.1	111740	236	Mycobacterium phage Zelink	NA	Viruses; Duplodnaviria; Heunggongvirae; Uroviricota; Caudoviricetes; Caudovirales; Siphoviridae; Omegavirus; unclassified Omegavirus
GCA_003442275.1	50547	78	Streptomyces phage Hank144	NA	Viruses; Duplodnaviria; Heunggongvirae; Uroviricota; Caudoviricetes; Caudovirales; Siphoviridae; Arquatrovirinae; Arequatrovirus; unclassified Arequatrovirus
GCA_003442315.1	52733	79	Streptomyces phage LibertyBell	NA	Viruses; Duplodnaviria; Heunggongvirae; Uroviricota; Caudoviricetes; Caudovirales; Siphoviridae; unclassified Siphoviridae
GCA_003442515.1	52600	81	Streptomyces phage TrvxScott	NA	Viruses; Duplodnaviria; Heunggongvirae; Uroviricota; Caudoviricetes; Caudovirales; Siphoviridae; Arquatrovirinae; Arequatrovirus; unclassified Arequatrovirus
GCA_000859205.1	43033	66	Haemophilus phage Aaphi23	NA	Viruses; dsDNA viruses, no RNA stage; Caudovirales; Myoviridae; unclassified Myoviridae
GCA_003601415.1	41394	59	Corynebacterium phage Juicebox	NA	Viruses; Duplodnaviria; Heunggongvirae; Uroviricota; Caudoviricetes; Caudovirales; Siphoviridae; Juiceboxvirus; Corynebacterium virus Juicebox
GCA_003601335.1	44609	60	Corynebacterium phage SamW	NA	Viruses; Duplodnaviria; Heunggongvirae; Uroviricota; Caudoviricetes; Caudovirales; Siphoviridae; Samwavirus; Corynebacterium virus SamW
GCA_003601095.1	67773	92	Gordonia phage Foxboro	NA	Viruses; Duplodnaviria; Heunggongvirae; Uroviricota; Caudoviricetes; Caudovirales; Siphoviridae; unclassified Siphoviridae
GCA_003601175.1	67802	92	Gordonia phage KidneyBean	NA	Viruses; Duplodnaviria; Heunggongvirae; Uroviricota; Caudoviricetes; Caudovirales; Siphoviridae; unclassified Siphoviridae
GCA_003601255.1	61703	103	Microbacterium phage OneinaGillian	NA	Viruses; Duplodnaviria; Heunggongvirae; Uroviricota; Caudoviricetes; Caudovirales; Siphoviridae; Gillianvirus; Microbacterium virus OneinaGillian
GCA_003601455.1	59708	96	Mycobacterium phage Dalmuri	NA	Viruses; Duplodnaviria; Heunggongvirae; Uroviricota; Caudoviricetes; Caudovirales; Siphoviridae; Timquatrovirus; unclassified Timquatrovirus
GCA_003601215.1	48545	87	Mycobacterium phage MuchMore	NA	Viruses; Duplodnaviria; Heunggongvirae; Uroviricota; Caudoviricetes; Caudovirales; Siphoviridae; Fromanvirus; unclassified Fromanvirus
GCA_003601235.1	68521	101	Mycobacterium phage Mulan	NA	Viruses; Duplodnaviria; Heunggongvirae; Uroviricota; Caudoviricetes; Caudovirales; Siphoviridae; Bclasvirinae; Pegunavirus; unclassified Pegunavirus
GCA_003601275.1	51029	86	Mycobacterium phage Wizard007	NA	Viruses; Duplodnaviria; Heunggongvirae; Uroviricota; Caudoviricetes; Caudovirales; Siphoviridae; Fromanvirus; unclassified Fromanvirus
GCA_003719055.1	155886	232	Mycobacterium phage CharlieB	NA	Viruses; Duplodnaviria; Heunggongvirae; Uroviricota; Caudoviricetes; Caudovirales; Myoviridae; Bixzunavirus; unclassified Bixzunavirus
GCA_003601075.1	58129	94	Mycobacterium phage DismalStressor	NA	Viruses; Duplodnaviria; Heunggongvirae; Uroviricota; Caudoviricetes; Caudovirales; Siphoviridae; Timquatrovirus; unclassified Timquatrovirus
GCA_003601395.1	67391	93	Mycobacterium phage Eaglehorse	NA	Viruses; Duplodnaviria; Heunggongvirae; Uroviricota; Caudoviricetes; Caudovirales; Siphoviridae; Bclasvirinae; Rosebushvirus; unclassified Rosebushvirus
GCA_003601115.1	57861	109	Mycobacterium phage Galactic	NA	Viruses; Duplodnaviria; Heunggongvirae; Uroviricota; Caudoviricetes; Caudovirales; Siphoviridae; Cheoctovirus; unclassified Cheoctovirus
GCA_003601135.1	54125	86	Mycobacterium phage Gancho	NA	Viruses; Duplodnaviria; Heunggongvirae; Uroviricota; Caudoviricetes; Caudovirales; Siphoviridae; Gilesvirus; unclassified Gilesvirus
GCA_003601155.1	110713	230	Mycobacterium phage Kalah2	NA	Viruses; Duplodnaviria; Heunggongvirae; Uroviricota; Caudoviricetes; Caudovirales; Siphoviridae; Omegavirus; unclassified Omegavirus
GCA_003601195.1	64834	112	Mycobacterium phage LittleLaf	NA	Viruses; Duplodnaviria; Heunggongvirae; Uroviricota; Caudoviricetes; Caudovirales; Siphoviridae; Marvinvirus; unclassified Marvinvirus
GCA_003601295.1	75640	142	Mycobacterium phage Paperbeatsrock	NA	Viruses; Duplodnaviria; Heunggongvirae; Uroviricota; Caudoviricetes; Caudovirales; Siphoviridae; Kostyavirus; unclassified Kostyavirus
GCA_003614615.1	156610	236	Mycobacterium phage Salacia	NA	Viruses; Duplodnaviria; Heunggongvirae; Uroviricota; Caudoviricetes; Caudovirales; Myoviridae; Bixzunavirus; unclassified Bixzunavirus

gca	genome.size	proteome.size	phage.name	strain	Taxonomy
GCA_003601315.1	75307	127	Mycobacterium phage Samty	NA	Viruses; Duplodnaviria; Heunggongvirae; Uroviricota; Caudoviricetes; Caudovirales; Siphoviridae; Bronvirus; unclassified Bronvirus
GCA_003601435.1	69867	98	Mycobacterium phage Serendipitous	NA	Viruses; Duplodnaviria; Heunggongvirae; Uroviricota; Caudoviricetes; Caudovirales; Siphoviridae; Bclasvirinae; Acadianvirus; unclassified Acadianvirus
GCA_003601355.1	64911	113	Mycobacterium phage VasuNzinga	NA	Viruses; Duplodnaviria; Heunggongvirae; Uroviricota; Caudoviricetes; Caudovirales; Siphoviridae; Marvinvirus; unclassified Marvinvirus
GCA_003601375.1	131250	242	Streptomyces phage Yaboi	NA	Viruses; Duplodnaviria; Heunggongvirae; Uroviricota; Caudoviricetes; Caudovirales; Siphoviridae; unclassified Siphoviridae
GCA_007923645.1	38122	54	Pseudomonas phage Fc02	NA	Viruses; Duplodnaviria; Heunggongvirae; Uroviricota; Caudoviricetes; Caudovirales; Siphoviridae; unclassified Siphoviridae
GCA_007923705.1	38255	53	Pseudomonas phage Fc22	NA	Viruses; Duplodnaviria; Heunggongvirae; Uroviricota; Caudoviricetes; Caudovirales; Siphoviridae; unclassified Siphoviridae
GCA_007923675.1	38223	54	Pseudomonas phage H71	NA	Viruses; Duplodnaviria; Heunggongvirae; Uroviricota; Caudoviricetes; Caudovirales; Siphoviridae; unclassified Siphoviridae
GCA_007923725.1	38579	55	Pseudomonas phage H72	NA	Viruses; Duplodnaviria; Heunggongvirae; Uroviricota; Caudoviricetes; Caudovirales; Siphoviridae; unclassified Siphoviridae
GCA_007923795.1	39816	57	Pseudomonas phage Ps56	NA	Viruses; Duplodnaviria; Heunggongvirae; Uroviricota; Caudoviricetes; Caudovirales; Siphoviridae; unclassified Siphoviridae
GCA_007923765.1	39020	54	Pseudomonas phage Ps59	NA	Viruses; Duplodnaviria; Heunggongvirae; Uroviricota; Caudoviricetes; Caudovirales; Siphoviridae; unclassified Siphoviridae
GCA_007923745.1	39676	56	Pseudomonas phage Ps60	NA	Viruses; Duplodnaviria; Heunggongvirae; Uroviricota; Caudoviricetes; Caudovirales; Siphoviridae; Beetrevirus; unclassified Beetrevirus
GCA_003575945.1	92348	168	Pseudomonas phage PaYy2	NA	Viruses; Duplodnaviria; Heunggongvirae; Uroviricota; Caudoviricetes; Caudovirales; Myoviridae; Pakpunavirus; unclassified Pakpunavirus
GCA_003575925.1	154811	199	Salmonella phage SenALZ1	NA	Viruses; Duplodnaviria; Heunggongvirae; Uroviricota; Caudoviricetes; Caudovirales; Ackermannviridae; Cvivirinae; Kuttervirus; unclassified Kuttervirus
GCA_003575905.1	157630	204	Salmonella phage SenASZ3	NA	Viruses; Duplodnaviria; Heunggongvirae; Uroviricota; Caudoviricetes; Caudovirales; Ackermannviridae; Cvivirinae; Kuttervirus; unclassified Kuttervirus
GCA_003575825.1	116460	85	Escherichia phage vB_EcoM_IME392	NA	Viruses; Duplodnaviria; Heunggongvirae; Uroviricota; Caudoviricetes; Caudovirales; Myoviridae; unclassified Myoviridae
GCA_003575705.1	39382	51	Escherichia phage LL2	NA	Viruses; Duplodnaviria; Heunggongvirae; Uroviricota; Caudoviricetes; Caudovirales; Autographiviridae; Studiervirinae; Teetrevirus; Escherichia virus LL2
GCA_008802975.1	55455	70	Bacillus phage BSP7	NA	Viruses; unclassified bacterial viruses
GCA_003606175.1	166560	253	Acinetobacter phage KARL1	NA	Viruses; Duplodnaviria; Heunggongvirae; Uroviricota; Caudoviricetes; Caudovirales; Myoviridae; Tevenvirinae; unclassified Tevenvirinae
GCA_003443075.1	112106	157	Vibrio phage vB_VpS_PG07	NA	Viruses; Duplodnaviria; Heunggongvirae; Uroviricota; Caudoviricetes; Caudovirales; Demerecviridae; Pogseptimavirus; Vibrio virus PG07
GCA_003443095.1	165213	263	Bacillus phage Hobo	NA	Viruses; Duplodnaviria; Heunggongvirae; Uroviricota; Caudoviricetes; Caudovirales; Herelleviridae; Bastillevirinae; Caeruleovirus; unclassified Caeruleovirus
GCA_003443115.1	161830	293	Bacillus phage Kamfam	NA	Viruses; Duplodnaviria; Heunggongvirae; Uroviricota; Caudoviricetes; Caudovirales; Herelleviridae; Bastillevirinae; Bastillevirus; unclassified Bastillevirus
GCA_003443155.1	165676	261	Bacillus phage Kioshi	NA	Viruses; Duplodnaviria; Heunggongvirae; Uroviricota; Caudoviricetes; Caudovirales; Herelleviridae; Bastillevirinae; Caeruleovirus; unclassified Caeruleovirus
GCA_003443135.1	161833	299	Bacillus phage OmnioDeoPrimus	NA	Viruses; Duplodnaviria; Heunggongvirae; Uroviricota; Caudoviricetes; Caudovirales; Herelleviridae; Bastillevirinae; Wphvirus; unclassified Wphvirus
GCA_003443235.1	49468	75	Klebsiella phage NJR15	NA	Viruses; Duplodnaviria; Heunggongvirae; Uroviricota; Caudoviricetes; Caudovirales; Drexlerviridae; Webervirus; Klebsiella virus NJR15
GCA_003443195.1	50132	79	Klebsiella phage NJS2	NA	Viruses; Duplodnaviria; Heunggongvirae; Uroviricota; Caudoviricetes; Caudovirales; Drexlerviridae; Webervirus; Klebsiella virus NJS2
GCA_003443215.1	49387	76	Klebsiella phage NJS3	NA	Viruses; Duplodnaviria; Heunggongvirae; Uroviricota; Caudoviricetes; Caudovirales; Drexlerviridae; Webervirus; unclassified Webervirus
GCA_003443175.1	49344	76	Klebsiella phage TAH8	NA	Viruses; Duplodnaviria; Heunggongvirae; Uroviricota; Caudoviricetes; Caudovirales; Drexlerviridae; Webervirus; Klebsiella virus TAH8
GCA_003442595.1	50970	76	Gordonia phage Angelique	NA	Viruses; Duplodnaviria; Heunggongvirae; Uroviricota; Caudoviricetes; Caudovirales; Siphoviridae; unclassified Siphoviridae
GCA_003442615.1	57555	85	Gordonia phage Ashertheman	NA	Viruses; Duplodnaviria; Heunggongvirae; Uroviricota; Caudoviricetes; Caudovirales; Siphoviridae; Vividuovirus; unclassified Vividuovirus
GCA_003442895.1	75813	129	Gordonia phage Neville	NA	Viruses; Duplodnaviria; Heunggongvirae; Uroviricota; Caudoviricetes; Caudovirales; Siphoviridae; unclassified Siphoviridae
GCA_003442955.1	53085	106	Gordonia phage Phistory	NA	Viruses; Duplodnaviria; Heunggongvirae; Uroviricota; Caudoviricetes; Caudovirales; Siphoviridae; Langleyhallvirinae; Phistoryvirus; Gordonia virus Phistory
GCA_003442575.1	59215	84	Gordonia phage Angelicage	NA	Viruses; Duplodnaviria; Heunggongvirae; Uroviricota; Caudoviricetes; Caudovirales; Siphoviridae; Vividuovirus; unclassified Vividuovirus
GCA_003442775.1	55668	103	Gordonia phage Horus	NA	Viruses; Duplodnaviria; Heunggongvirae; Uroviricota; Caudoviricetes; Caudovirales; Siphoviridae; Langleyhallvirinae; Horusvirus; Gordonia virus Horus
GCA_003443035.1	43099	76	Gordonia phage Schmidt	NA	Viruses; Duplodnaviria; Heunggongvirae; Uroviricota; Caudoviricetes; Caudovirales; Siphoviridae; Vendettavirus; unclassified Vendettavirus

gca	genome.size	proteome.size	phage.name	strain	Taxonomy
[truncated: 967,356 more chars]
